# Macrophage-infectivity potentiator of *Trypanosoma cruzi* (TcMIP) is a new pro-type 1 immuno-stimulating protein for neonatal human cells and vaccines in mice

**DOI:** 10.3389/fimmu.2023.1138526

**Published:** 2023-03-23

**Authors:** Magdalena Radwanska, Frédéric de Lemos Esteves, Loes Linsen, Nicolas Coltel, Sabrina Cencig, Joelle Widart, Anne-Cécile Massart, Séverine Colson, Alexandre Di Paolo, Pauline Percier, Sarra Ait Djebbara, François Guillonneau, Véronique Flamand, Edwin De Pauw, Jean-Marie Frère, Yves Carlier, Carine Truyens

**Affiliations:** ^1^ Laboratory of Parasitology, Faculty of Medicine, and ULB Center for Research in Immunology (UCRI), Université Libre de Bruxelles (ULB), Brussels, Belgium; ^2^ Center for Protein Engineering (CIP), Université de Liège (ULg), Liège, Belgium; ^3^ Laboratory of Mass Spectrometry (LSM), Department of Chemistry, Université de Liège, Liège, Belgium; ^4^ Institute for Medical Immunology (IMI), and ULB Center for Research in Immunology (U-CRI), Gosselies, Belgium; ^5^ Department of Tropical Medicine, School of Public Health and Tropical Medicine, Tulane University, New Orleans, MA, United States

**Keywords:** neonatal immunity, adjuvant, cord blood, type 1 immune response, gamma-interferon, macrophage infectivity potentiator, *Trypanosoma cruzi*

## Abstract

This work identifies the protein “macrophage infectivity potentiator” of *Trypanosoma cruzi* trypomastigotes, as supporting a new property, namely a pro-type 1 immunostimulatory activity on neonatal cells. In its recombinant form (rTcMIP), this protein triggers the secretion of the chemokines CCL2 and CCL3 by human umbilical cord blood cells from healthy newborns, after 24h *in vitro* culture. Further stimulation for 72h results in secretion of IFN-γ, provided cultures are supplemented with IL-2 and IL-18. rTcMIP activity is totally abolished by protease treatment and is not associated with its peptidyl-prolyl cis-trans isomerase enzymatic activity. The ability of rTcMIP to act as adjuvant was studied *in vivo* in neonatal mouse immunization models, using acellular diphtheria-tetanus-pertussis-vaccine (DTPa) or ovalbumin, and compared to the classical alum adjuvant. As compared to the latter, rTcMIP increases the IgG antibody response towards several antigens meanwhile skewing antibody production towards the Th-1 dependent IgG2a isotype. The amplitude of the rTcMIP adjuvant effect varied depending on the antigen and the co-presence of alum. rTcMIP did by contrast not increase the IgE response to OVA combined with alum. The discovery of the rTcMIP immunostimulatory effect on neonatal cells opens new possibilities for potential use as pro-type 1 adjuvant for neonatal vaccines. This, in turn, may facilitate the development of more efficient vaccines that can be given at birth, reducing infection associated morbidity and mortality which are the highest in the first weeks after birth.

## 1 Introduction

Vaccines constitute one of the best ways to prevent infectious diseases. Conventional, “antigen-based” vaccines comprise live attenuated and inactivated pathogens or recombinant antigens. The lower immunogenicity of recombinant antigen vaccines requires the addition of adjuvants to evoke a strong immune response. Adjuvants aim to increase the amplitude of the immune response as well as to modulate their orientation towards the appropriated protective response. Only a limited number of adjuvants are currently approved for human applications, with aluminum salts (alum), monophosphoryl lipid A (MPLA) and saponin being the most commonly used (1, 2). Several reasons support the need to diversify adjuvants. Firstly, this would broaden the choice to allow the best possible association with a given antigen. Secondly, adjuvants should be adapted to distinct features of the immune system of defined vulnerable populations such as infants (3). Thirdly, the combination of adjuvants with different modes of action appears to better elicit optimal protective responses while limiting the risk of adverse effects by reducing the concentration of each of them and possibly by avoiding the repeated use of the same adjuvant (2).

Due to their potent immunostimulatory capacity, pathogen-derived substances constitute major potential sources of adjuvants (4). We showed that the protozoan parasite *Trypanosoma cruzi*, responsible for Chagas disease, displays adjuvant properties in neonates/infants. Indeed, both congenitally infected and uninfected infants born to *T. cruzi* chronically infected mothers display a pro-inflammatory environment associated with activated monocytes, probably by receiving circulating parasite molecules from their mothers (5). In addition, uninfected infants from these mothers develop boosted type 1 immune responses to vaccines routinely administered in early life like BCG or those against hepatitis B, tetanus and diphtheria (6). These data suggest the transplacental transmission of parasite compound(s) from infected pregnant women to their fetus, susceptible to stimulate *T. cruzi*-unrelated type 1 immune responses in early life by epigenetic reprogramming (7–9). This led us to identify the *T. cruzi*-derived macrophage infectivity-potentiator (TcMIP), a protein that displays interesting immunostimulatory properties on neonatal human cells and vaccines in mice. As such, TcMIP constitutes a potential adjuvant candidate for vaccines, particularly for those administered in early life. The current study describes the identification, production and immunostimulatory properties of this protein.

## 2 Material and methods

### 2.1 Obtention of live and lysed *T. cruzi* and soluble extracts of parasite

Live *T. cruzi* trypomastigotes (the extracellular parasite stage found in vertebrate hosts), of TcVI genotype [Tulahuen strain (10),] were obtained from supernatants of infected fibroblasts as previously described (11). *T. cruzi* epimastigotes (TcVI genotype, CL Brener strain) were cultured at 28°C in RPMI-1640 liquid medium supplemented with 0.5% (w/v) tryptone (Thermo Fisher Scientific, Merelbeke, Belgium), 20 mM HEPES buffer pH 7.2 (Thermo Fisher Scientific), 30 µM hemin (Sigma-Aldrich, Overijse, Belgium), 10% (v/v) heat-inactivated fetal calf serum, 2 mM sodium glutamate, 2 mM sodium pyruvate and 25 µg/ml gentamycin (Sigma-Aldrich) (12). Lysates of *T. cruzi* trypomastigotes or epimastigotes were prepared by resuspending the parasites in RPMI at a concentration of 3 x 10^5^ parasites/mL and lysing them by 10 successive cycles of freezing-thawing in liquid nitrogen and 37°C. Lysates were aliquoted and conserved at -20°C until use.

For fractionation experiments, live parasites (trypomastigotes or epimastigotes) were resuspended at a concentration of 3 x 10^9^ parasites/mL in PBS containing protease inhibitors (complete mini protease inhibitor cocktail, Cat. No. 04 693 124 001, Roche Diagnostics GmbH, Mannheim, Germany) and lysed by 3 freezing-thawing cycles in liquid nitrogen and 37°C. After centrifugation at 100,000 g during 30 min at 4°C, the membrane proteins present in the sediment were extracted by n-octyl-β-D-glucopyranoside (OG, Sigma-Aldrich O9882) 2% in ice-cold PBS during 30 min. The preparation was vortexed for 30s every 10 min. Insoluble material was discarded by centrifugation at 100,000g during 1h at 4°C. OG was eliminated from the supernatant by dialysis overnight at 4°C against PBS using Slide-A-Lyzer™ dialysis cassettes with a cut-off of 10 kDa (Thermo Fisher Scientific). The soluble extract was then concentrated 10-fold by freeze-drying and resuspended in apyrogen water. Based on preliminary results showing that the bioactivity of the trypomastigote extract was thermoresistant up to 70°C and with a molecular mass (MM) lower than 50 kDa, thermosensitive proteins were eliminated by heating the extract at 70°C during 30 min, and those of MM above 50kDa by centrifugation on Vivaspin 500 (Sartorius). The preparation was then again concentrated by evaporation on SpeedVac. This preparation, named OGE (for OG extract), was aliquoted and conserved at -20°C before fractionation.

### 2.2 Fractionation of *T. cruzi* OGE

OGE fractionation was performed by Fast Protein Liquid Chromatography on FPLC AKTA EXPLORER 2DLC using endotoxin-free reagents and material. The chromatography equipment was cleaned before use with 1 M NaOH overnight (0.5 mL/min). After installing the column, the system was cleaned again with NaOH 1 M during 1h, followed by 30% acetonitrile + 0.5% trifluoroacetic acid during 1h, again NaOH 1 M for 1h, and finally PBS to obtain a neutral pH. Different approaches were tried, and the following protocol adopted. The OGE was fractionated by high-resolution size exclusion chromatography on Superdex 75 GL (GE HealthCare Life Sciences, France), separating proteins of MM between 3 and 70 kDa. Column calibration was done with chicken lysozyme (14.4 kDa), trypsin (21 kDa), carbon anhydrase (31 kDa), ovalbumin (45 kDa) and bovine serum albumin (66.2 kDa). A 0.5 mL solution of OGE containing around 250 µg of proteins was loaded onto a 24mL Superdex, the column was washed with 1 vol PBS (0.5 mL/min) and 52 fractions of 1 mL collected. Each fraction was aliquoted in 2 parts for screening the bioactivity and analyzing the protein content by mass spectrometry.

### 2.3 Mass spectrometry of protein fractions

Samples were dialyzed against 500 mM ammonium acetate to replace salts and digested with trypsin (Promega, sequencing grade) in order to perform nanoflow liquid chromatographic (nHPLC) separation of subsequent peptides. The nLC (Dionex Famos-Switchos) was hyphenated to an electrospray ion-source (ESI) to produce cationized peptides and measure their mass to charge ratio using a Bruker Esquire ion-Trap Mass Spectrometer (MS). The analysis was performed either in one dimension directly using a 15 cm pepmap C18 reverse phase column or by intercalation of a second dimension of separation using a strong cationic exchange phase (all columns were from Dionex, Thermo Fisher Scientific). In some cases, the desalted samples were separated on reducing and denaturing SDS PAGE beforehand and extracted gel bands were in-gel digested with trypsin before injection to the nano-HPLC-ESI-MS and 1D analysis (C18 column). Whenever needed, 2D HPLC was used to improve the separation.

The MS data were extracted using the Bruker data analysis suite of software and compared to the Swissprot, MSDB and NCBI databases using the mascot algorithm (https://eur01.safelinks.protection.outlook.com/?url=http%3A%2F%2Fwww.matrixscience.com%2F&data=05%7C01%7Ccarine.truyens%40ulb.be%7Cf8a785c087104e3dd2db08dae76da34b%7C30a5145e75bd4212bb028ff9c0ea4ae9%7C0%7C0%7C638076755676235063%7CUnknown%7CTWFpbGZsb3d8eyJWIjoiMC4wLjAwMDAiLCJQIjoiV2luMzIiLCJBTiI6Ik1haWwiLCJXVCI6Mn0%3D%7C2000%7C%7C%7C&sdata=MOS9kplRHGw5FERK9Xld%2BNzeuZxthdxyt6EC6hIUiBk%3D&reserved=0). Proteins yielding at least 2 distinct peptides with a mascot score of minimum 20 were considered present under 5% risk of false positive hits. Besides, specific proteins were also selectively sought for by multiple reaction monitoring (MRM).

### 2.4 Preparation of recombinant TcMIP

The sequence of the gene of the macrophage infectivity potentiator of *T. cruzi* (*Tcmip*) has been published (13). The coding sequence of *Tcmip* from purified parasite DNA was amplified by PCR, using primers recognizing its signal peptide and designed from the published sequence of *Tcmip* (13) (forward: ATGCACAGAGAGAATTATTTTTCCAA, reverse: CATAATTACATGTCTTCTCTGTCTTCTTC). The obtained blunt-end DNA was cloned in pGEM-T Easy vector (Promega Benelux B.V., Leiden, the Netherlands) and the integrity of the construction verified by sequencing. This construct was used as template to amplify *Tcmip* without the signal peptide, i.e., the sequence corresponding to residues 30 to 196, using forward and reverse oligonucleotides flanked by the restriction sites *BamHI* et *Not1* respectively (oligo BamHI-MIP forward: GGATCCCCAGTGGGGATGCGGCGTCG and Not1-MIP reverse: GCGGCCGCTTACATGTCTTCTCT). The PCR product of 518 bp was purified from an agarose gel using the GFX PCR DNA and Gel Band Purification Kit (Amersham, now Cytiva Europe GmbH, Freiburg im Breisgau, Germany) and this blunt-end DNA cloned in the pJET plasmid to verify its integrity. *E. coli* DH5α cells were transformed with this construct and several transformed clones cultivated. Their plasmids were isolated and digested with BamHI et Not1 to verify the presence of *Tcmip.* After control of the sequence, the BamHI-*mip*-Not1 insert was introduced in the expression vector pGEX-5x-1 (Amersham) previously digested by the same restriction enzymes. This cloning results in the fusion of the N-terminal part of *Tcmip* with the gene coding for glutathione-S-transferase (GST, 26 kDa). After transformation of *E. coli* DH5α with pGEX-5X-1+*Tcmip*, a clone containing the *Tcmip* gene was identified by PCR and cultivated again to obtain and purify the correct plasmid containing the *Tcmip* gene fused with GST (pGEX-5X-1+*Tcmip*). DNA and protein sequences are shown in **Supplementary Figure 1**).

**Figure 1:**
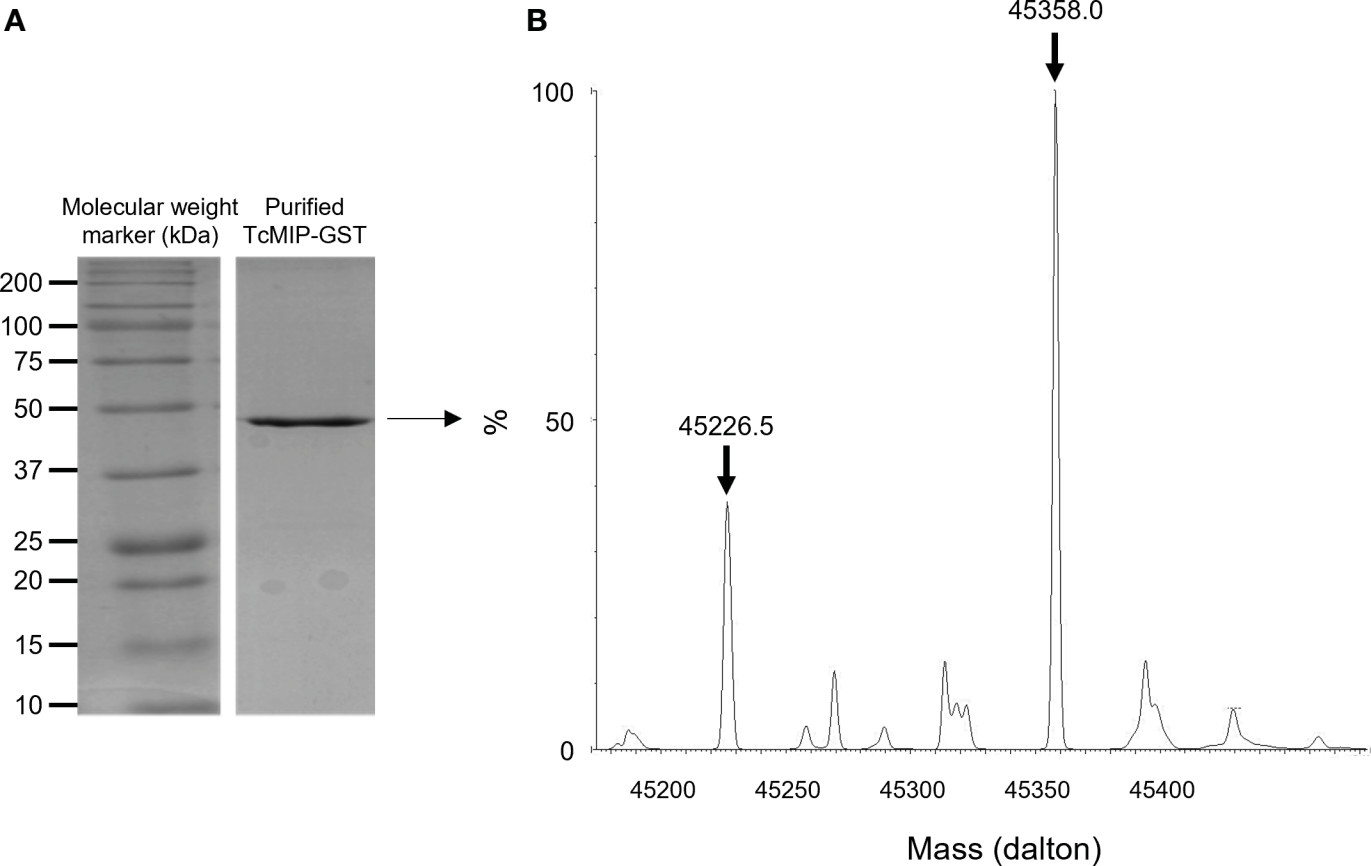
Analysis of the purified recombinant TcMIP fused to GST. **(A)** SDS-Page analysis showing the purified recombinant protein TcMIP fused to GST (rTcMIP). **(B)** Deconvoluted mass spectrum of the purified rTcMIP analyzed by Q-Tof mass spectrometry. The spectrum shows two major peaks: one at 45358 Da corresponding to the mass of the fused recombinant protein supplemented with one H+ cation, and one at 45226.5 Da corresponding to the loss of a methionine.

The expression of the fusion protein was obtained in *E. coli* BL21 cells transformed with pGEX-5X-1+*Tcmip.* The fusion protein was purified from the supernatant of lysed cells on a column of Superdex 75 Prep Grade followed by affinity chromatography on GSTrap HP (GE Healthcare). The fractions were then analyzed by SDS-PAGE, those containing the protein pooled and the purity and integrity of the TcMIP-GST verified by SDS-Page (**Figure 1A**) and Q-tof mass spectrometry (**Figure 1B**). These analyses indicate the absence of contaminants and of degradation products. The fact that the MM of the fused protein found by Q-Tof spectrometry (45358 Da) corresponds to the expected mass based on the amino-acid sequence (45352 Da), implies the absence of any post-translational modifications, indicating that the fused protein is of proteinic nature only.

It was attempted to cleave the GST tag with the Factor Xa Protease (Promega) without success although various conditions were tested. So, all results presented in this publication have been obtained with the TcMIP (167 aminoacids, i.e., without the signal peptide) fused to the GST, which is from now on referred to as rTcMIP in our experiments.

Endotoxins present in the purified rTcMIP were eliminated by chromatography on a polymixin column (EndoTrap^®^ HD Endotoxin Removal Kit, Hyglos GmbH, Bernried, Germany) down to levels < 0.5 EU/mg TcMIP. The TcMIP concentrations mainly used in this work are between 2.5 and 5 µg/mL, containing thus less than 0.005 EU/mL ((i.e. 1 pg entotoxin/mL), which is well below the threshold of 0.5 EU/mL accepted by the FDA in medical devices (14) and endotoxin levels found in most commercialized vaccines (15).

### 2.5 Quality control of parasite-derived preparations and recombinant TcMIP

All procedures were performed under laminar flow with sterile endotoxin-free reagents and material. Before their use in bioassays and blood cell stimulation, each parasite preparation (lysate, OGE…) and batches of the rTcMIP were verified to be free of mycoplasma by ELISA detecting the four more common *Mycoplasma* species (Mycoplasma Detection Kit Enzyme immunoassay Cat. No. 11 296 744 001, Roche) as well as by PCR detecting 26 *Mycoplasma* species (Venor^®^GeM # 11-1025, Minerva Biolabs, Berlin, Deutschland), and we verified the endotoxin levels to be undetectable or very low, with the Limulus Amoebocyte Lysate test (Cambrex, Lonza Biosciences, Verviers, Belgium) or the LAL Chromogenic Endpoint Assay » (HyCult Biotechnology, Uden, The Netherlands). Protein concentrations were measured with the BCA test (Pierce #23235, Thermo Fisher Scientific), and the profile of components was verified by SDS-PAGE to be similar between batches. Each OGE batch was confirmed to induce the production of IFN-γ, CCL2 and CCL3 by cord blood cells (cf. bioassay) before being further used for fractionation.

### 2.6 Human blood samples

Umbilical cord blood samples were obtained from full-term healthy newborns at the maternity ward of Erasmus Hospital (U.L.B., Brussels). Blood samples were harvested in endotoxin-free heparinized tubes (Becton Dickinson, Erembodegem, Belgium]. They were kept at room temperature and processed within 8 hours of harvest. The study was approved by the Ethical Committee Erasme-U.L.B. of the Faculty of Medicine, U.L.B. (protocols P2011-254 and P2014-339). Informed written consent was obtained from the parents of newborns.

### 2.7 Set-up of a bioassay to screen the immuno-stimulatory properties of *T. cruzi* compounds

**Figure 2:**
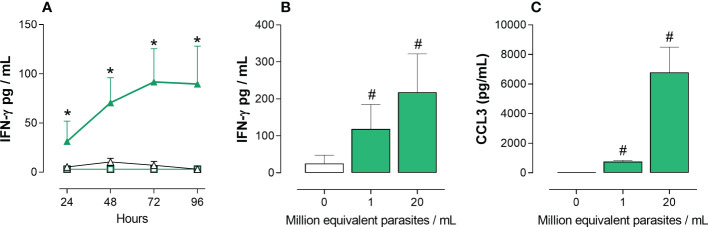
IFN-γ and CCL3 release by whole cord blood cells in response to a *T. cruzi* trypomastigotes lysate. Ten-fold diluted whole cord blood from healthy newborns were cultured with (green symbols) or without (white symbols) *T. cruzi* trypomastigote lysate. IFN-γ and CCL3 were measured in the supernatants by ELISA. Results are expressed as mean ± SEM. **(A)** Cultures (n=4) were performed for 24 to 96h in the absence (square symbols) or the presence (triangle symbols) of IL-2 (5U/mL) and IL-18 (10ng/mL). Parasite lysate was at 10^6^ equivalent parasites/mL. *P < 0.05 vs. unstimulated cells cultures in the presence of IL-2 and IL-18 (Two-way Anova). **(B)** Cultures (n=5) were performed for 72h in the presence of IL-2 (5U/mL) and IL-18 (10ng/mL), with two parasite lysate concentrations. ^#^P<0.05 vs. the absence of parasite lysate (Wilcoxon test). **(C)** Cultures (n=5) were performed for 24h with two parasite lysate concentrations. ^#^P<0.05 vs. the absence of parasite lysate (Wilcoxon test).

To identify parasite compound(s) able to induce the production of IFN-γ by cord blood cells from healthy newborns (not infected with *T. cruzi*), a quick and simple bioassay was developed. The release of IFN-γ was firstly chosen to select parasite compounds with pro-type 1 adjuvant-like activity. Preliminary assays, in which whole cord blood cells were stimulated with lysed *T. cruzi* trypomastigotes during 24 to 96h, in the presence or not of potential co-stimulatory factors, indicated that best results were obtained after 72h in the presence of 5U/mL IL-2 and 10 ng/mL IL-18. At these concentrations, these cytokines used alone barely induced an IFN-γ response but synergized with the parasite lysate (**Figure 2A**). This indicates that *T. cruzi* may exert an immuno-stimulatory effect independently of cell infection. We also searched, by ELISA and dot-blot arrays, for other markers of inflammatory/pro-type 1 factors produced in supernatants of whole cord blood cultured with the *T. cruzi* lysate in the absence of co-stimulatory cytokines. The inflammatory chemokines CCL2 (monocyte chemoattractant protein 1, -1) and CCL3 (macrophage inflammatory protein-alpha, MIP1-α) were selected as additional markers released after 24h of culture. **Figures 2B, C** show that the trypomastigote lysate reliably triggered IFN-γ and CCL3 release by several cord blood samples.

As a result, the final used bioassay protocol was the following: whole cord blood was diluted 10-fold in 96-well culture plates with RPMI supplemented with 1x non-essential amino acids, 1 mM sodium pyruvate, 100 U/mL penicillin and 100 µg/mL streptomycin. The 10-fold diluted *T. cruzi* fraction or the rTcMIP were added to cells at the indicated concentrations as stimulant, associated or not to the co-stimulatory cytokines IL-2 (5 U/mL) and IL-18 (10 ng/mL). A culture supernatant from non-stimulated cells was used as a negative control. GST (#PK-RP577-1243-1, Bio-Connect B.V., Huissen, The Netherlands) alone was used as control for rTcMIP (which, as said above, comprises the GST tag). Cells were then incubated at 37°C in 5% CO_2_ atmosphere. The amounts of CCL2 and CCL3 produced after 24h of culture (performed without the co-stimulatory cytokines IL-2 and IL-18), as well as the amount of IFN-γ after 72h of culture (performed in the presence of IL-2 and IL-18) were measured by ELISA. In some experiments, rTcMIP was heated at different temperatures immediately before use. In other experiments, rTcMIP was incubated for 30 min with either a protease (pronase E from *Streptomyces griseus* at 1 µg/mL - E = 3.4.24.31 – Sigma-Aldrich # P8811), or FK506 (or Tacrolimus, a macrolide that inhibits the PPIase enzymatic activity; used at 25 ng/mL, Sigma-Aldrich # F4679), before being added on cells. As a control of FK506 activity, its immunosuppressive effect was tested on cells stimulated with 1 µg/mL of phorbol myristate acetate (PMA, InvivoGen, Toulouse, France) combined to 10 µg/mL ionomycin (InvivoGen) (16).

### 2.8 ELISA detection of produced cytokines and chemokines

Levels of IFN-γ, CCL2 (MCP-1) and CCL3 (MIP-1α) in culture supernatants were determined by ELISA using antibody (Ab) pairs from InVitrogen (#CHC1233, 88-7399-88 and 88-7035-22 respectively, Thermo Fisher Scientific). Assays were performed in duplicate following the manufacturer’s instructions. Detection limits were 2 pg/mL for all three assays.

### 2.9 Immunization of mice with acellular tetanus-diphteria–pertussis vaccine or ovalbumin

BALBc mice were obtained from Janvier (Le Genest-St-Isle, France, or Harlan, now Envigo, Horst, The Netherlands). They were bred and maintained in our animal facility in compliance with the guidelines of the ULB Ethic Committee for the use of laboratory animals (protocols 529A and BUC-2013-03).

In one experiment, mice received two forms of acellular diphtheria-tetanus- pertussis vaccine (DTPa, kindly given by GlaxoSmithKline Biologicals SA-GSK-, Rixensart, Belgium), containing 4 IU/mL of tetanus toxoid, 40 IU/mL of diphtheria toxoid and three antigens of *Pertussis* (16 µg/mL of pertussis toxoid -PT, 16 µg/mL of filamentous hemagglutinin -FHA, and 5 µg/mL of pertactin -PRN). The first vaccine form was adsorbed on a mix of 0.6 mg/mL of hydrated aluminium hydroxide (Al(OH)_3_) and 0.4 mg/mL of aluminium phosphate (AlPO_4_), further called DTPa-alum (BOOSTRIX^®^). The second form was the same formulation non adsorbed on alum (crude DTPa). Six groups of 20 to 28 neonatal mice per group received at days 7 and 28 of life, sub-cutaneous (sc) injections of either 125 µL of crude DTPa, or crude DTPa combined with 5 µg rTcMIP as adjuvant, or crude DTPa combined with 2.5 µg GST (as control of GST-tagged rTcMIP), or DTPa-alum, or TcMIP alone (5 µg) or PBS. Blood plasma samples were collected 12 days after the second immunization.

In another experiment, three groups of 6 neonatal mice received, in a final volume of 50 µL, intraperitoneal (ip) injections of 2.5 µg of endotoxin-free ovalbumin (OVA, EndoGrade^®^ Ovalbumin, Hyglos GmBH) associated with either rTcMIP (10µg) or GST (5 µg), or adsorbed on alum (Imject Alum Adjuvant, Thermo Fisher Scientific) according to the procedure described by the manufacturer. Another group of 6 mice received OVA combined with rTcMIP and alum together (same concentrations as above). Three control groups of 3 mice each received OVA, rTcMIP or GST alone. The same experiment where mice received sub-cutaneous (sc) in place of ip injections was performed in parallel. Immunizations were performed at days 7 and 21 of life. Blood plasma samples were collected just before the second immunization and 1, 2 and 4 weeks after the second immunization.

### 2.10 ELISA determination of antibody levels in immunized mice

IgG1 and IgG2a antibody (Ab) levels against each antigen of DTPa were determined by ELISA. GSK provided filamentous hemagglutinin (FHA), pertactin (PRN), pertussis toxin (PT), diphtheria toxin (DT) and tetanus toxin (TT). Nunc Maxisorp plates (VWR, Leuven, Belgium) were coated overnight at 4°C in carbonate buffer pH 9.6 with FHA (8µg/mL), PRN (6 µg/mL), PT, DT or TT (each at 2 µg/mL). After each step, plates were washed with 0.9% NaCl 0.05% Tween20. After blocking with 1% bovine serum albumin (BSA, Sigma-Aldrich #A7030) in PBS for 2h at 37°C, plasma samples were diluted in PBS containing 0.2% BSA and 0.05% Tween20 and incubated for 2h at 37°C. Sample dilutions were variable according to the antigens. Rat monoclonal antibodies (MoAb), specific for the Fc fragment of mouse IgG1 or IgG2a (5 µg/mL; clones LO-MG1-2 and LO-MG2a-9 respectively, SynAbs S.A., Brussels, Belgium), were incubated for 2h at 37°C, followed by horseradish peroxidase (HRP)-conjugated goat antibodies specific for rat IgG (H+L) (Jackson ImmunoResearch Europe Ltd, Ely, Cambridgeshire, UK). Finally, substrate and chromogen were added (hydrogen peroxide and 3,3′,5,5′-tetramethylbenzidine - BD Biosciences, Erembodegem, Belgium). After 30 min color development and stopping the reaction with 2N sulfuric acid, absorbances at 450 nm were measured. Results are expressed in absorbances.

OVA-specific total IgG and IgG1 were titrated by standard ELISA as described above. Coating was performed with OVA (Grade V, Sigma-Aldrich) at 10 µg/mL in PBS. Serum samples were tested as serial dilutions. Abs were detected with rat MoAb against mouse IgG kappa chain (for total IgG detection – clone LO-MK1, SynAbs) or mouse IgG1 (clone LO-MG1-2) used at 5 µg/mL followed by HRP-conjugated anti-rat antibodies (R&D System) and substrate. For IgG2a and IgE quantification, plates were coated with capture mAb to IgG2a (LO-MG2a-9) or IgE (LO-ME-3) and then incubated successively with serial dilutions of sera, biotinylated OVA at 5 µg/mL (Immunosource), HRP-labelled streptavidin diluted at 1/200 (R&D) and substrate. Results are expressed in titers, corresponding to the last dilution giving an absorbance above the cut-off, calculated as the mean + 3 SDs of absorbances given by negative controls.

## 3 Results

### 3.1 Identification of OGE fractions of *T. cruzi* trypomastigotes supporting immune-stimulatory activities

**Figure 3:**
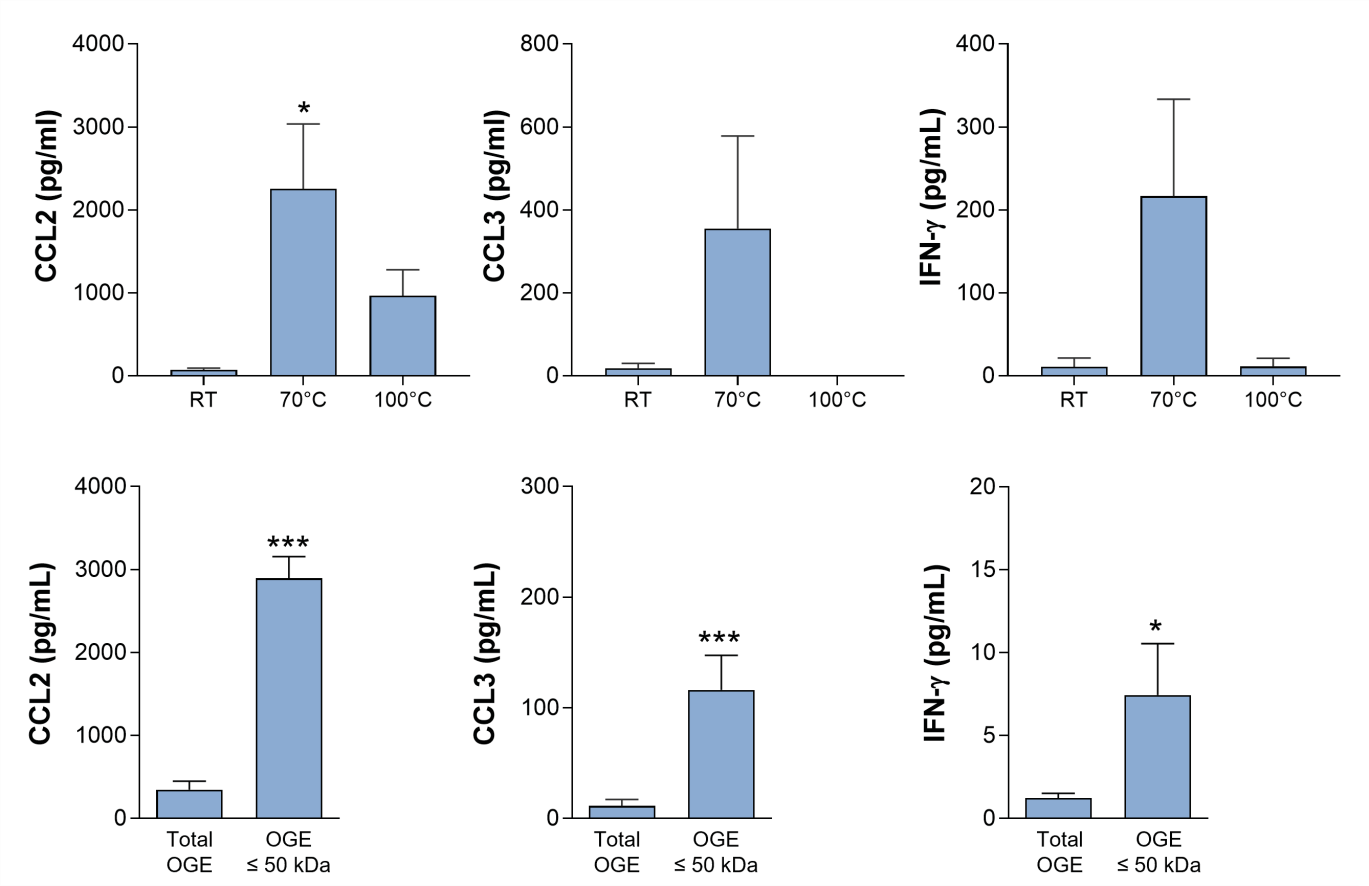
Bioactivity of the octyl-glucoside extract (OGE) of *T. cruzi* trypomastigote lysate. Ten-fold diluted whole cord blood from healthy newborns were cultured with the OG extract of trypomastigote lysate, either pretreated at room temperature (RT), 70°C or 100°C for 30 min **(A)** or filtered in order to conserve molecules of molecular mass ≤ 50 kDa **(B)**. Cultures were performed during 24h for CCL2 and CCL3 production or 72h, in the presence of IL-2 (5U/mL) and IL-18 (10ng/mL), for IFN-γ production). Results are expressed as mean ± SEM (A: n=4; B: n=10). *P<0.05, ***P< 0.005 vs. RT or unfiltered extract (“total extract”) (Wilcoxon test).

**Figure 4:**
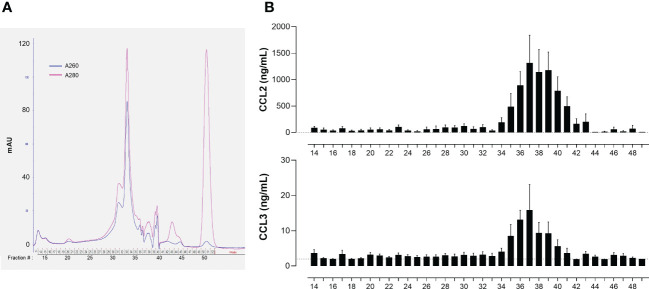
Bioactivity of *T. cruzi* trypomastigote OGE fractions obtained by size exclusion chromatography. **(A)** Typical chromatogram of a *T. cruzi* trypomastigote OGE separated on Superdex 75 GL. **(B)** CCL2 and CCL3 produced by ten-fold diluted whole cord blood cells cultured during 24h in response to each OGE fraction collected by size exclusion chromatography. CCL2 and CCL3 levels were determined in supernatants by ELISA (detection threshold 2 pg/mL, dotted line). Results are expressed as mean ± SEM of 23 tests corresponding to 7 fractionated OGE batches, each batch of fractions being tested on 3 to 4 different cord blood samples.

Preliminary experiments indicated that the immune-stimulating bioactivity of the parasite trypomastigote OGE increased by heating, showing thermoresistance up to 70°C and displayed a molecular mass lower than 50 kDa (**Figure 3**). The fractions obtained from trypomastigote OGEs using the size exclusion chromatography were tested in the bioassay for their ability to trigger the release of CCL2 and CCL3 by cord blood cells from healthy newborns. **Figure 4A** shows a typical chromatogram of OGE fractionation on Superdex 75GL. Several protein peaks were reproducibly present in fractions # 31 to 40-41. The ability of each fraction to induce CCL2 and CCL3 production by cord blood cells is shown in **Figure 4B**. A major bioactivity was found in fractions 35 to 41. In contrast, the major protein peak in fraction 33 does not possess immune-stimulatory activity. Conversely, despite the low protein concentrations in the fractions 35 to 41, their bioactivity was markedly higher than that detected in unfractionated OGE.

### 3.2 Identification of TcMIP as an immuno-stimulatory protein of *T. cruzi*

A pool of the 5 to 6 bioactive adjacent fractions of each fractionated OGE batches from trypomastigotes was prepared and analyzed by mass spectrometry (2D HPLC ESI Trap). It allowed the recurrent identification of 19 to 29 proteins depending on the OGE batches (**Table 1**, OGEs 1 to 4). Eight proteins were common to the 4 batches, while the other proteins were found in 3 of them. Five of these proteins could be considered as non-bioactive since they were also detected in an OGE prepared from *T. cruzi* epimastigotes, that was found unable to induce CCL2, CCL3 and IFN-γ production by cord blood cells. These proteins are indicated in italics in **Table 1**. More information on protein identification is available in **Supplementary Material**. This yielded 24 candidate bioactive proteins.

**Table 1:** Proteins identified in bioactive fractions of trypomastigote OG.

	UniProtKB	NCBI	SWISSPROT	Mass (kDA)	Name	1 (a)	2 (a)	3 (a)	4 (a)	5 (b)
1	P07749	gi|119859	FCA1_TRYCR	24	***Flagellar calcium-binding protein** (FCaBP)* (c) (d)	x	x	x	x	
2	P22513	gi|120679	G3PG_TRYCR	40	*Glyceraldehyde-3-phosphate dehydrogenase, glycosomal (GAPDH)* (c)	*x*	*x*	*x*	*x*	
3	P14834	gi|123592	HSP70_LEIMA	56	Heat shock 70 kDa protein	x	x	x	x	x
4	XP_807701	gi|71410853	Q4DFB0_TRYCC	11	Heat shock protein 10 kDa	x	x	x	x	
5	XP_806144	gi|71407337	Q4CVJ1_TRYCC	16	Hypothetical / uncharacterized protein	x	x	x	x	
6	XP_808914	gi|71413559	Q4D3F7_TRYCC	44	Hypothetical / uncharacterized protein	x	x	x	x	
7	XP_820921	gi|71667953	Q4E2Q5_TRYCC	46	Hypothetical / uncharacterized protein	x	x	x	x	
8	AAG08956	gi|9954108	Q9GZC7_TRYCR	35	RNA binding protein RGGm	x	x	x	x	
9	P26643	gi|133055	RLA1_TRYCR	11	*60S acidic ribosomal protein P1* (c)		x	x	x	
10	XP_805182	gi|71405064	Q4CSS8_TRYCC	11	60S acidic ribosomal protein P2		x	x	x	
11	XP_807299	gi|71409962	Q4CYU3_TRYCC	13	Calpain-like cysteine peptidase	x	x	x		
12	BAA06214	gi|704459	Q26913_TRYCR	44	Elongation factor 1 alpha	x	x	x		
13	P05456	gi|123603	HSP70_TRYCR	74	*Heat shock 70 kDa protein* (c)		x	x	x	x
14	CAA71400	gi|1781355	P90596_TRYCR	13	Histone H2A		x	x	x	
15	XP_802643	gi|71398774	Q4CLP6_TRYCC	30	Hypothetical / uncharacterized protein		x	x	x	
16	XP_808345	gi|71412308	Q4D1U0_TRYCC	153	Hypothetical/ uncharacterized protein		x	x	x	
17	XP_808929	gi|71413591	Q4D3H5_TRYCC	17	Hypothetical / uncharacterized protein	x		x	x	
18	XP_814262	gi|71651158	Q4DIP8_TRYCC	17	Hypothetical / uncharacterized protein		x	x	x	
19	XP_818370	gi|71662736	Q4DVF8_TRYCC	43	Hypothetical / uncharacterized protein		x	x	x	
20	XP_820432	gi|71666956	Q4E1C6_TRYCC	41	Hypothetical / uncharacterized protein	x		x	x	
21	XP_820557	gi|71667211	Q4E1M8_TRYCC	30	Hypothetical / uncharacterized protein	x		x	x	
22	XP_808505	gi|71412664	Q4D2A8_TRYCC	61	LsmAD domain-containing protein		x	x	x	
23	Q09734	gi|1170958	MIP_TRYCR	22	**Macrophage infectivity potentiator** (d)	x		x	x	x
24	AAG12985	gi|10119899	Q9GN79_TRYCR	101	Pyruvate phosphate dikinase 1	x		x	x	
25	XP_818583	gi|71663174	Q4DW49_TRYCC	45	Succinyl-CoA ligase [GDP-forming] beta-chain		x	x	x	
26	XP_802786	gi|71399455	Q4CM39_TRYCC	25	Surface protein TolT	x	x		x	
27	XP_805084	gi|71404821	Q4CSI1_TRYCC	89	Trans-sialidase	x		x	x	
28	AAA99441	gi|1314208	Q26973_TRYCR	47	*Tubulin alpha* (c)	x	x	x		
29	P14795	gi|10673	RL40_TRYCR	15	Ubiquitin-60S ribosomal protein L40	x	x	x	x	

(a) Proteins identified by bottom-up proteomics (cf. Material and Methods) in pools of bioactive fractions from trypomastigote OGE.

(b) Proteins identified by bottom-up proteomics in the fraction from a trypomastigote OGE presenting the highest bioactivity.

(c) in italic: proteins identified by bottom-up proteomics, common to unfractionnated epimastigote OGE and bioactive fractions of trypomastigote OGEs.

(d) in bold: major proteins < 50 kDa detected in an unfractionnated trypomastigote OGE (see text).

To narrow the list of proteins of interest, the fraction presenting the highest bioactivity (rather than a pool of fractions), prepared from a fifth fractionated OGE, was subsequently analyzed by mass spectrometry. Three proteins were identified in this fraction: 2 heat shock proteins and the macrophage infectivity potentiator of *T. cruzi* (TcMIP) (**Table 1**). We confirmed the presence of TcMIP in the bioactive fraction of a sixth trypomastigote OGE by multiple reaction monitoring. Of note, TcMIP could also be identified by mass spectrometry among major bands of MM < 50 kDa in an unfractionated OGE separated by SDS-PAGE (highlighted in bold in **Table 1**). For all these reasons, and since the sequence of TcMIP was published (13), this protein was produced in a recombinant form.

### 3.3 The recombinant TcMIP displays immuno-stimulating bioactivity

**Figure 5:**
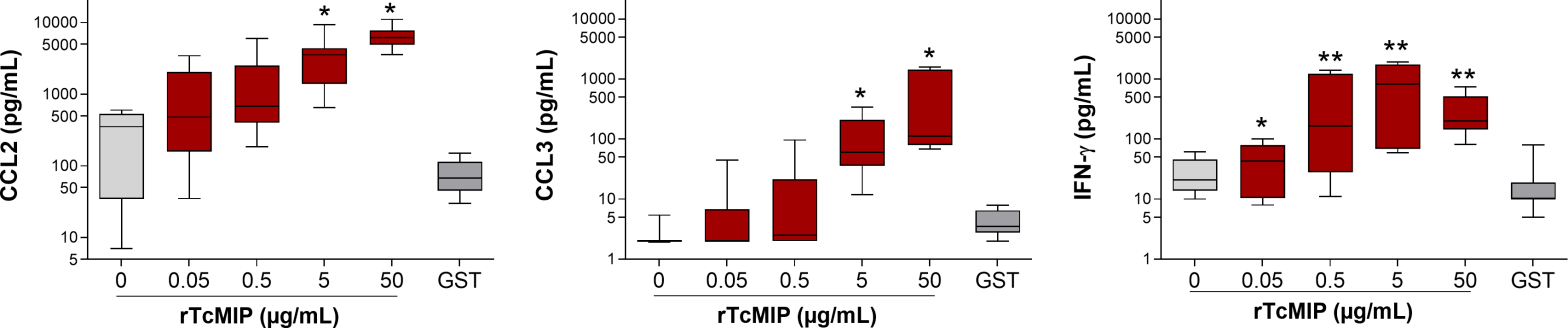
Production of CCL2, CCL3 and IFN-γ by cord blood cells in response to rTcMIP. Whole cord blood (ten-fold diluted) was incubated with increasing concentrations of rTcMIP during 24h (for CCL2 and CCL3 responses) or 72h in the presence of IL-2 (5 U/mL) and IL-18 (10 ng/mL) (for IFN-γ response). CCL2, CCL3 and IFN-γ were measured by ELISA in the supernatants. GST: control of the rTcMIP, used at 25 µg/mL. Results are expressed as mean ± SEM (n = 12). *P<0.05, **P<0.005, rTcMIP vs. GST (Wilcoxon test).

**Figure 6:**
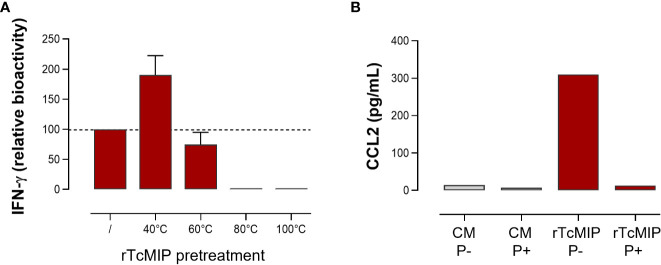
Effect of temperature and protease treatment on the bioactivity of rTcMIP. Whole cord blood samples (ten-fold diluted) were incubated with rTcMIP during 24h for CCL2 response or 72h in the presence IL-2 (5 U/mL) and IL-18 (10 ng/mL) for IFN-γ response. CCL2 and IFN-γ were measured by ELISA in the supernatants. **(A)** IFN-γ response to rTcMIP (5 µg/mL) pretreated by heating during 30 min at the indicated temperatures. Results are expressed as % of the bioactivity of unheated rTcMIP (n=3). **(B)** CCL2 response to rTcMIP in the presence (P+) or not (P-) of proteases from *Streptomyces griseus* at 1 µg/mL in the culture medium.

As shown in **Figure 5**, rTcMIP induced a dose-dependent production of IFN-γ, CCL2 and CCL3 by whole cord blood cells whereas GST alone had no effect. The IFN-γ response was maximal at rTcMIP concentrations between 0.5 and 50 µg/mL. Of note, at 5 µg rTcMIP/mL, all tested newborns (n=9) responded by the production of CCL2, CCL3 and IFN-γ. These results show that the rTcMIP reproduced the immune-stimulatory activities of the bioactive fractions of *T. cruzi* trypomastigotes OGEs on neonatal cells. We also observed that the bioactivity of rTcMIP increased after heating at 40°C and was thermoresistant up to at least 60°C, as expected from the earlier experiments of parasite fractionation (**Figure 6A**). **Figure 6B** further shows that the rTcMIP bioactivity was totally abolished after protease treatment, indicating that no contaminant LPS activity could account for the immuno-stimulatory activity of the recombinant protein.

### 3.4 The immunostimulatory activity of TcMIP is not associated with its PPIase activity

**Figure 7:**
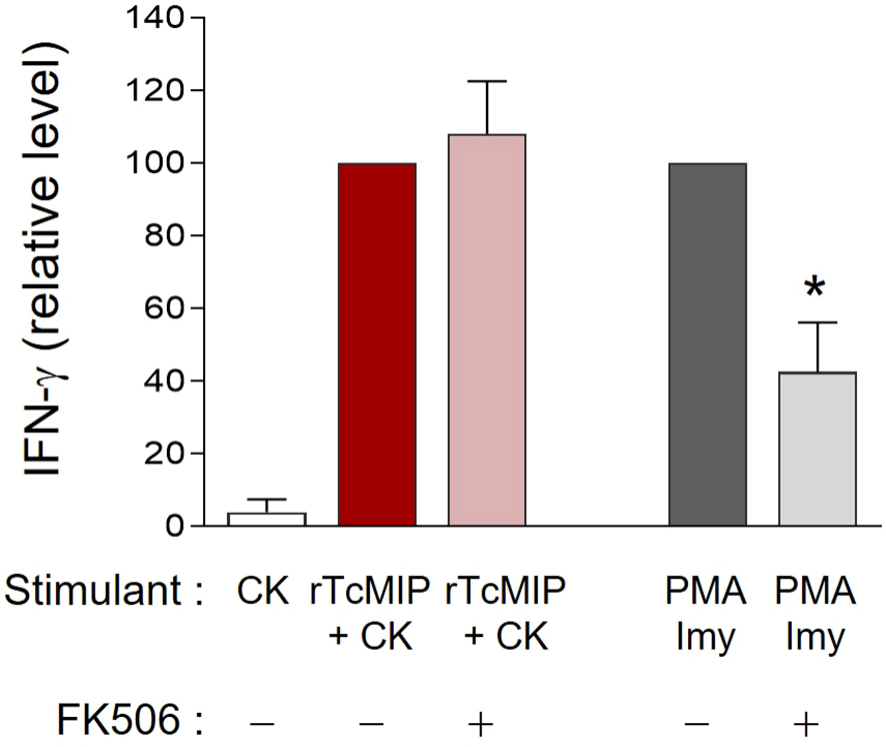
The rTcMIP bioactivity is independent of its PPIase enzymatic activity. Ten-fold diluted whole cord blood samples were cultured with rTcMIP (2.5 µg/mL), IL-2 (5U/mL) and IL-18 (10ng/mL) in the presence or not of FK506 (25 ng/mL). IFN-γ were measured in the supernatants by ELISA after 72h. Results are expressed as mean ± SEM (n=6). *P< 0.05 as compared to the absence FK506 (Wilcoxon test).

TcMIP belongs to the family of FK506 binding proteins (FKBPs) (13). Such proteins are peptidyl-prolyl cis-trans isomerases (PPIase), and this enzymatic activity can be inhibited by the macrolide antibiotic FK506 (17). To investigate if the immunostimulatory activity of rTcMIP depended on its PPIase activity, FK506 was added to cell cultures stimulated by rTcMIP. Cells stimulated by PMA and ionomycin were used as controls of the inhibitory effect of FK506. **Figure 7** shows that FK506 did not affect the rTcMIP ability to induce IFN-γ release, while the response to PMA and ionomycin was significantly inhibited by 58 ± 14%. These results indicate that the bio-stimulating activity of rTcMIP does not depend on its isomerase activity.

### 3.5 rTcMIP adjuvant property increases the IgG2a to IgG1 Ab ratio in a neonatal DTPa mouse vaccination model

The ability of rTcMIP to act as immunological adjuvant was investigated *in vivo* by studying its effect on the neonatal humoral immune response in mice that received the acellular diphtheria-tetanus-pertussis (DTPa) vaccine. Its effect was compared to that of alum, the adjuvant of the commercial DTPa vaccine. The IgG1 and IgG2a antibody (Ab) responses were specifically studied, since the switch to IgG1 or to IgG2a is strongly associated with Th2-type (IL-4) and Th1-type (IFN-γ responses respectively (18). Mice were immunized with either crude DTPa (i.e., without any adjuvant, n=20), or crude DTPa combined with rTcMIP (n=25), GST (n=15) or alum (n=25). Mice received intraperitoneal (ip) injections at days 7 and 28 of life. One week after the second immunization, the circulating levels of IgG1 and IgG2a Abs against the antigens present in this vaccine were measured: the filamentous hemagglutinin (FHA), pertactin (PRN), inactivated pertussis (PT)-, diphtheria (DT)- and tetanus (TT)-toxoids and the ratios between IgG2a and IgG1 Ab levels were determined.

**Figure 8:**
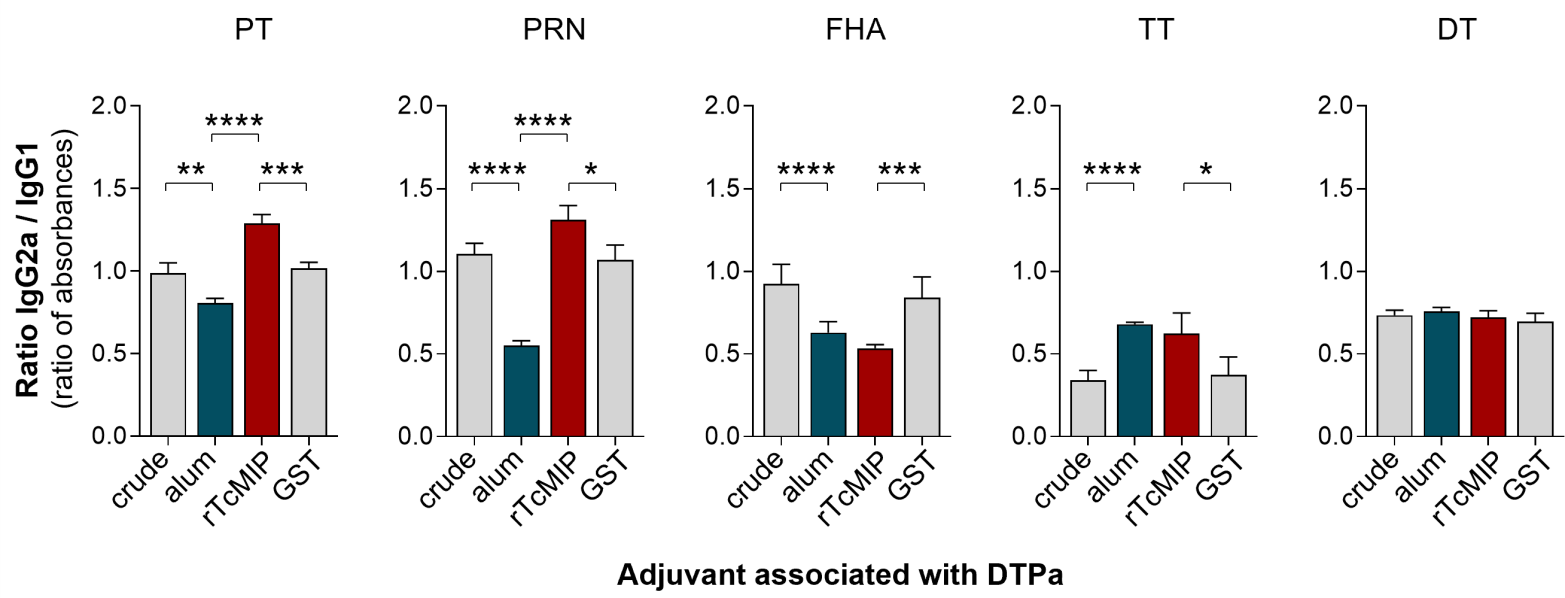
Ratio of IgG2a to IgG1 antibody levels in neonatal mice immunized with DTPa adjuvanted by rTcMIP. Neonatal Balb/c mice received at days 7 and 28 of life, injections of 125 µL DTPa either unadjuvanted (“crude”, n=20) or adjuvanted with alum (n=25), with rTcMIP (n=25) or with GST (n=15) (cf. M&M). Ab levels directed against the indicated vaccinal antigens were measured by ELISA 7 days after the second immunization. Results are expressed as mean ± SEM or the individual ratios between absorbances of IgG2a and IgG1. *P< 0.05, **P< 0.005, ***P< 0.0005, ****P< 0.0001 (Mann Withney test).

Ab levels found in immunized mice are presented in **Supplementary Figure 2**. We observed that the adjuvant effect of alum considerably varied according to the vaccinal antigen, either increasing or reducing the Ab response, or having no effect, as compared to the Ab response triggered by the unadjuvanted crude vaccine. Interestingly, rTcMIP used in place of alum exerted an adjuvant effect, increasing the Ab levels (though slightly in most cases at the concentration used) against four of the five antigens present in the vaccine, as compared to the Ab levels observed in response to the crude vaccine associated with GST alone. Most interestingly, the analysis of the ratios between IgG2a and IgG1 Ab levels (absorbances) showed that rTcMIP significantly increased the IgG2a/IgG1 antibody ratio against some antigens (PRN and PT) as compared to DTPa adjuvanted by alum (**Figure 8**) whereas alum reduced the IgG2a/IgG1 ratio in response to these two antigens. Yet, IgG2a/IgG1 Ab ratios observed in response to FHA, TT or DT did not differ with the use of alum or rTcMIP. In no case this ratio was lower when rTcMIP was used in place of alum.

Since switches to IgG2a and IgG1 Ab production are known to be associated with Th1-type and Th2-type responses respectively, these results suggest that rTcMIP exerts a preferential pro-type 1 immunostimulatory property in response to some antigens in neonatal mice immunized with DTPa.

### 3.6 Co-administration of rTcMIP with alum increases the IgG1 and IgG2a but not IgE antibody responses to OVA in neonatal mice

**Figure 9:**
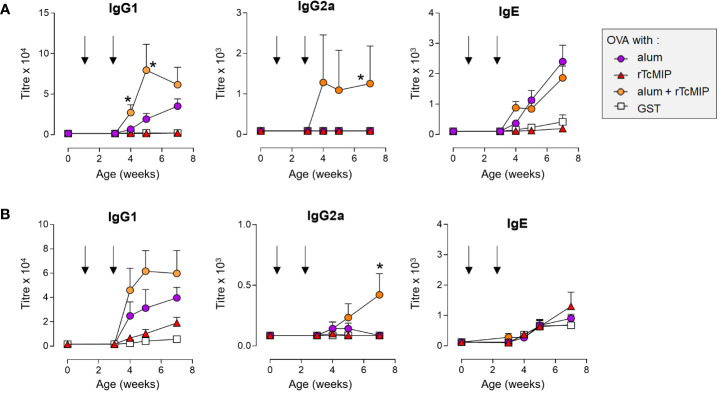
Antibody levels in neonatal mice immunized against OVA adjuvanted by rTcMIP. Neonatal Balb/c mice (n = 6/group) were immunized with OVA (2.5 µg/injection) adjuvanted with alum, rTcMIP (10 µg), alum + rTcMIP or alum + GST (5 µg). Mice received intraperitoneal **(A)** or subcutaneous **(B)** injections at 7 and 21 days of age (arrows). OVA-specific circulating antibodies were titrated just before the second immunization and 1, 2 and 4 weeks after the second immunization. No Abs were detected in non-immunized mice neither in those receiving OVA, alum or rTcMIP alone (not shown). Antibody titers were determined by ELISA. *P< 0.05 alum + rTcMIP vs. alum (Mann Whitney test). Statistically significant results between other mouse groups are not shown.

Neonatal mice were immunized with ovalbumin (OVA), administered alone, or associated with rTcMIP (or GST as negative control of the GST-tagged TcMIP), with alum, or with rTcMIP and alum together (6 mice/group). Control groups of 3 mice received rTcMIP or alum alone. Mice were immunized at days 7 and 21 of life by ip route and the circulating levels of IgG1, IgG2a and IgE OVA-specific Abs measured at different time points. No Abs were detected in mice receiving OVA, rTcMIP or alum alone. The OVA-alum combination induced IgG1 Abs whereas IgG2a Abs were not detectable (**Figure 9A**), while co-administration of OVA with rTcMIP did not induce any Ab response. However, the combination rTcMIP and alum with OVA markedly increased the IgG1 response as compared to the use of alum alone and, still more interestingly, IgG2a Abs were now produced at clearly detectable levels. This again highlights the ability of rTcMIP to trigger a switch of the Ab response to IgG2a and its pro-type 1 immunostimulatory property. Besides, rTcMIP alone did not modify the IgE Ab response triggered in the presence of alum. A similar response profile was obtained in another experiment where injections were performed by sub-cutaneous route (**Figure 9B**).

No macroscopic local reaction (no swelling) was observed at the injection sites and all young mice survived and exhibited similar weight increases during their growth, whatever the group (data not shown).

These results strongly support the ability of rTcMIP to exhibit an adjuvant effect *in vivo* on neonatal Ab immune responses to various Ags. In addition, rTcMIP was able to shift some responses towards the production of IgG2a Abs.

## 4 Discussion

We have identified a protein derived from *T. cruzi* trypomastigotes, i.e., the macrophage infectivity potentiator (TcMIP), as a potent immunostimulatory molecule capable of triggering the release of IFN-γ, CCL2 and CCL3, by umbilical cord blood cells from healthy newborns *in vitro*. In addition, rTcMIP exerts adjuvant properties in neonatal vaccination models. Here, rTcMIP was shown to skew antibody secretion towards IgG2a, a hallmark of Th1 mediated immunity, to some antigens comprised in the DTPa vaccine or to ovalbumin. In addition, the ovalbumin vaccination study demonstrates that rTcMIP can act synergistically with alum in generating strong antibody response against albumin by neonatal mice. The quality control of rTcMIP utilized in this study allowed to attribute the immunostimulatory properties to rTcMIP itself, since despite the presence of a GST tag, the tag itself did not exhibit any of the reported activities *in vitro* nor *in vivo*. Furthermore, the total inhibition of the rTcMIP immunostimulatory properties was achieved by pre-treatment with a protease, eliminating a possibility of an artefact related to contamination with endotoxin. The latter was found well below the threshold accepted by the FDA (14) and most commercial vaccines (15). Additionally, our *in vitro* results are as close as possible to physiological conditions since whole cord blood cells were used to avoid the step of mononuclear cell purification and the associated risk of their artefactual stimulation. Also, whole cord blood cells include neutrophils, numerous immune-modulating cells such as regulatory T cells and CD71+ erythroid cells, and autologous plasma, that can also exhibit modulating effects on observed responses (19–21). Together, these observations strongly support the immunostimulatory property of TcMIP itself.

TcMIP belongs to the family of FK506 binding protein (FKBP)-type peptidyl-prolyl cis-trans isomerases and was initially described as playing a major role in *T. cruzi* cell infection (13). These authors showed that TcMIP is secreted by trypomastigotes and enhances parasite invasion of mammalian cells by an unknown mechanism that depends on its peptidyl-prolyl cis-trans isomerase (PPIase) enzymatic activity. Based on the ability of FK506, which is a strong and specific inhibitor of the PPIase activity, to reduce parasite entry into host cells, Moro et al. hypothesized that TcMIP’s enzymatic activity might interact with some proteins of the host cell membrane, contributing to render the host cell more susceptible to invasion through conformational changes of prolyl peptide bonds, but the molecular target(s) were not identified. The three-dimensional crystal structure of TcMIP and, more recently, its atomic-resolution structure, were described (22, 23). In this context, we have tested whether the immunostimulatory properties of rTcMIP depend on its PPIase activity, using FK506 inhibitor. We show that the ability of rTcMIP to trigger IFN-γ was not blocked by FK506, indicating that its immunostimulatory property was independent of its PPIase activity, confirming that FKBPs are multifunctional molecules that may display diverse cellular functions independently of their PPIase domain (24). Besides, rTcMIP showed improved biological activity after treatment at higher temperature, as did the native protein present in OGE extracts. This suggests that a native folding/conformation might not be crucial for its biological activity. As such, our work characterizes new properties of TcMIP, showing that this molecule is a pro-type 1 immune modulator of neonatal cells since it induces i) the production of IFN-γ, a key cytokine that primes differentiation of Th1 T cells by activating the transcription factor (TF) STAT1 in naive T CD4+ T cells (25–27), ii) the production of the chemokines CCL2 and CCL3 that favor the development of type 1 immune responses (28–30) and iii) a switch towards IgG2a humoral immune response in neonatal mice, known to be strongly dependent on IFN-γ production (18).

rTcMIP is a potent immune stimulator as low concentrations were sufficient to trigger the release of IFN-γ, CCL2 and CCL3 by cord blood cells. Interestingly, at a concentration of 5 µg/mL, rTcMIP could trigger a response in all tested cord blood samples. CCL2 and CCL3 release was observed after 24h while the IFN-γ response was measured after 72h and required co-stimulation with IL-2 and IL-18. Occasionally, IFN-γ release by cord blood cells could be detected after 24h and 48h (data not shown). *In vivo*, neonatal mouse immunization with DTPa or OVA showed that rTcMIP increased the IgG2a Ab response to some Ags, indicating that the stimulating effect was working without exogenous addition of co-stimulatory cytokines. Although we may not strictly compare an *in vitro* response of human cells to an *in vivo* response in mouse, this suggests that *in vivo*, either co-stimulatory factors might have been produced in response to the injected material, or that rTcMIP could be self-sufficient to exert a pro-type 1 response effect.

In mouse immunization models, the ability of rTcMIP to increase the Ab response and/or to skew the Ab isotype towards IgG2a, varied according to the used antigen and the co-presence or not of the commonly used adjuvant, alum. A variable effect was also observed for alum, when used alone. This is in line with observations that not every adjuvant can efficiently be coupled to every vaccine (31–33). In addition, as in our experiments of mouse immunization with OVA, the adjuvant effect of rTcMIP combined with alum was superior to that of alum alone, it might be interesting to combine both. This might possibly allow to reduce the concentration of each adjuvant as well as their potential side effects, a strategy already suggested otherwise (1, 2).

Our study also shows that trypomastigote lysate, OGE fractions and rTcMIP were all able to trigger IFN-γ, CCL2 and CCL3 responses by human umbilical cord cells, indicating that the immune-stimulating effect did not depend on cell infection. It can be hypothesized that in *T. cruzi*-infected pregnant women, TcMIP, secreted by circulating trypomastigotes, crosses the placenta as others parasitic bio-relevant molecules (9), thereby explaining our previous observations that chagasic mothers induce profound perturbations in the cytokine response of their neonates and boost type 1 immune responses to vaccines routinely administered in early life (5, 6), as well as why such effects can be observed in both congenitally infected and uninfected neonates. The latter are generally more vulnerable to infectious diseases than adults, mostly to infections requiring type 1 immune responses to be controlled, due to particular features of their immune system (34). Accordingly, neonates are also less prone to mount protective type 1 immune responses to vaccines. This highlights the need for pro-type 1 adjuvants for protein and peptide-based vaccines (3, 35, 36) to complement the limited number of adjuvants that are currently approved for human use (1, 2). In this context, our present work resulted in the discovery of a candidate pro-type 1 vaccinal adjuvant for pediatric vaccines. The mode of action of rTcMIP is under investigation. We may however underline some advantages of rTcMIP that make it an attractive adjuvant when compared to some currently licensed adjuvants. Firstly, it is a pure protein without post-translational modifications, easy to produce under recombinant form in *E. coli*, which opens the possibility of constructing a fusion protein with a vaccinal antigenic peptide, thereby improving the immune response (37, 38). Secondly, the immunostimulatory property of rTcMIP was preserved up to temperatures of 50-60°C, an interesting point eliminating the need for a cold chain. Thirdly, in OVA immunized mice, rTcMIP does not trigger IgE Ab production at higher level than alum, suggesting that the risk for inducing allergic adverse effects is minimal. Finally, when administered to neonatal mice, we did not observe any macroscopically signs of local inflammation, and young mice grew normally, gaining weight as did the mock-treated mice.

Some aspects deserve attention for the potential use of rTcMIP as adjuvant in vaccines. Firstly, we may not presume with certainty that the immunostimulatory property of rTcMIP observed *in vitro* on cord blood cells and *in vivo* in mice will be the same *in vivo* in humans. Although we have attractive preliminary results in mice, neither rodent models nor adult human leukocytes accurately model human newborn and infant responses (39). Secondly, molecules similar to TcMIP are expressed by various other pathogens such as *Legionella pneumophila*, *Neisseria meningitidis*, *N. gonorrhoeae* and *Chlamydia trachomatis* (24, 40–43). Like TcMIP, these MIPs act as virulence factors through their PPIase activity. Whereas some of them are proposed for use as diagnosis biomarkers or antigens for vaccinations (44), none have, as far as we know, been investigated for a potential adjuvant effect. On the other hand, in relation to its role in cell host invasion, TcMIP could also be considered for use as a vaccine candidate for Chagas disease, knowing that TcMIP-specific Abs inhibit the entry of parasites into host cells (13). This has not been investigated so far but could offer the additional advantage of displaying an auto-adjuvant effect. Thirdly, the family of FKBP–type PPIases is also widely distributed and highly conserved among various other organisms including humans (13, 45). For instance, the TcMIP amino acid sequence shares 26% similarity with human FKBP12. Homology concerns the PPIase catalytic domain of the protein (13). Thus, there is a possible risk that rTcMIP induces antibodies that could cross-react with host FKBPs, which might induce side effects (24). Further work could investigate the possibility to truncate the PPIase part of TcMIP, since its enzymatic domain is not involved in its immune-stimulatory action. This strategy has previously been used with the MIP from *Neisseria meningitidis*, which is a candidate vaccinal antigen (46). Finally, Abs against TcMIP, that will likely be induced in response to its administration, might thwart its repetitive use in vaccines requiring boosts, except if different adjuvants or strategies are used between priming and boost.

In conclusion, this study discloses the pro-type 1 immuno-stimulating property of the *T. cruzi* secreted TcMIP protein, that possibly contributes to the induction of the maternal immune inflammatory and pro-type 1 immune imprinting of neonates born to *T. cruzi* chronically infected mothers (5, 6). Further studies should better identify the immuno-stimulating part of the molecule and explore its mode of action. Its adjuvant activity and potentially immunogenic properties should be tested using specific pre-clinical models of vaccination against pathogens followed by challenge experiments. Vaccination against infectious disease should be given as early as possible in early life, preferably at birth, to gain protection as rapidly as possible and limit the infectious morbi-mortality which are the highest in the first weeks after birth (47). Our work might open such a possibility. As rTcMIP also stimulated the neonatal murine immune system, its use in veterinary vaccines may also be considered (48). As such, our current study opens new possibilities in neonatal vaccine design.

## Data availability statement

The original contributions presented in the study are included in the article/**Supplementary Material**, further inquiries can be directed to the corresponding author/s.

## Ethics statement

The studies involving human participants were reviewed and approved by Ethical Committee Erasme-ULB of the Faculty of Medicine, ULB (protocols P2011-254 and P2014-339). Written informed consent to participate in this study was provided by the participants’ legal guardian/next of kin. The animal study was reviewed and approved by ULB Ethic Committee for the use of laboratory animals (CEBEA) (protocols 529A and BUC-2013-03).

## Author contributions

Conceptualization: CT, YC, ED, J-MF. Funding acquisition: CT, YC, ED, J-MF. Investigation: all authors. Data analysis: MR, FdLE, LL, NC, SaC, JW, SeC, VF, ED, J-MF, YC, CT. Writing–original draft: CT. Writing–review and editing: MR, FdLE, PP, VF, J-MF, ED, YC, CT. All authors contributed to the article and approved the submitted version.
