# Supplementary figures and images for: Macrophage-infectivity potentiator of Trypanosoma cruzi (TcMIP) is a new pro-type 1 immuno-stimulating protein for neonatal human cells and vaccines in mice
Source: Front Immunol. 2023 Mar 23;14:1138526. doi: 10.3389/fimmu.2023.1138526 (PMC10077492; doi:10.3389/fimmu.2023.1138526)

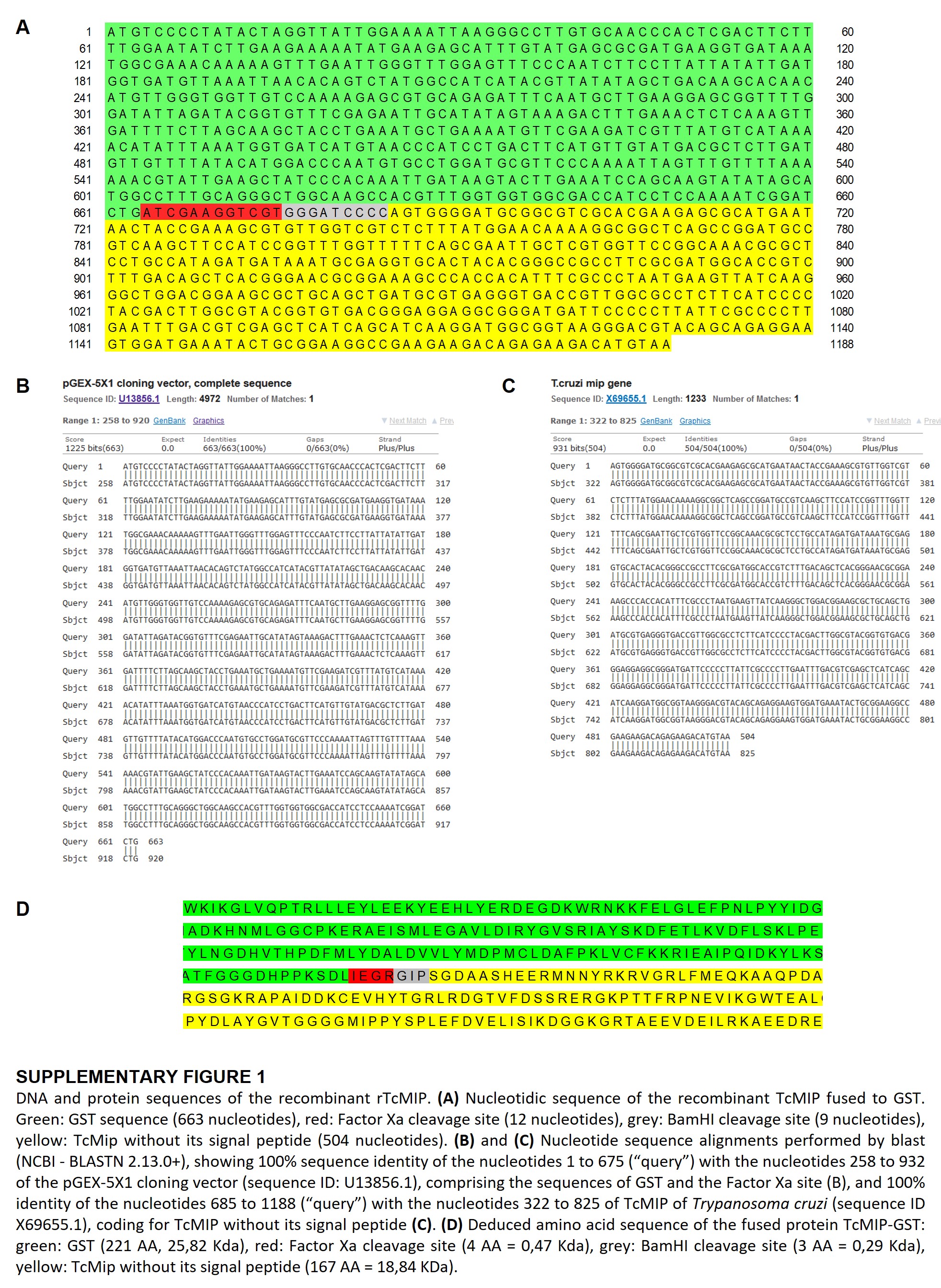

Supplement: Supplementary file 1 [file Image_1.jpg]

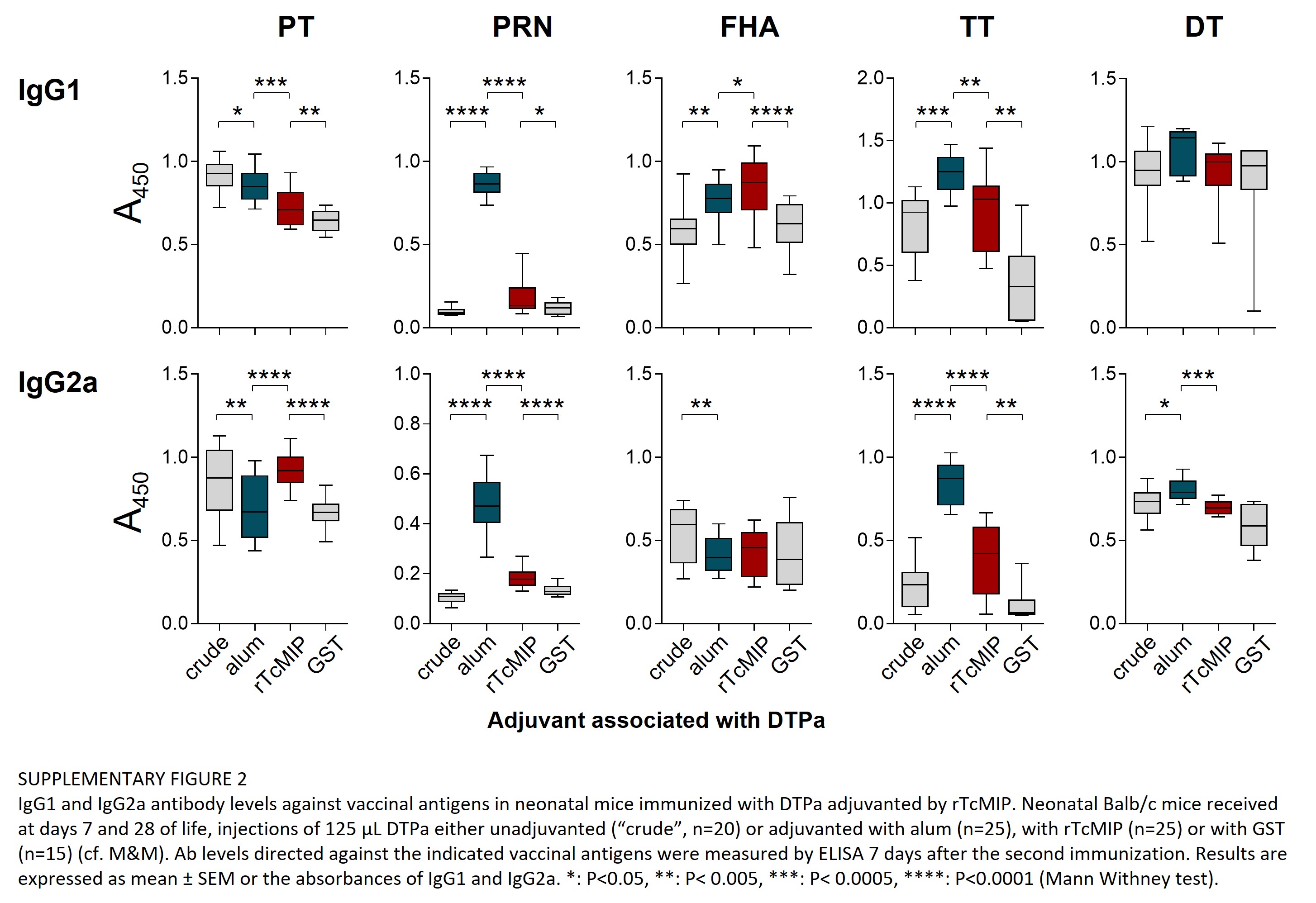

Supplement: Supplementary file 2 [file Image_2.jpg]
