# Supplementary material for: Macrophage-infectivity potentiator of Trypanosoma cruzi (TcMIP) is a new pro-type 1 immuno-stimulating protein for neonatal human cells and vaccines in mice
Source: Front Immunol. 2023 Mar 23;14:1138526. doi: 10.3389/fimmu.2023.1138526 (PMC10077492; doi:10.3389/fimmu.2023.1138526)
Supplement: Supplementary file 3 [file DataSheet_1.pdf]

Mascot Search Results

User : GM
Email : gabriel.mazzucchelli@ulg.ac.be
Search title : Submitted from 081125-OGE33-precip-ZipTip-8467 by Mascot Daemon on MASPEC39
MS data file : F:\DATA\Archives\ESQUIRE\2008-Esquire\Adjuvac\081120-OGE33\081125-OGE33-precip-dig-zipTip_8467.mgf
Database : NCBInr 20081117 (6550153 sequences; 2246496946 residues)
Taxonomy : Other Eukaryota (159618 sequences)
Timestamp : 26 Nov 2008 at 12:43:11 GMT
Significant hits:

gi|3023478	RecName: Full=Chaperonin HSP60, mitochondrial; Short=Protein Cpn60; AltName: Full=groEL protein; Al
gi|50659756	heat shock protein 70 [Trypanosoma cruzi]
gi|71414968	microtubule-associated protein [Trypanosoma cruzi strain CL Brener]
gi|296214	P69 antigen [Trypanosoma congolense]
gi|71413559	hypothetical protein [Trypanosoma cruzi strain CL Brener]
gi|71667211	hypothetical protein [Trypanosoma cruzi strain CL Brener]
gi|71410853	10 kDa heat shock protein [Trypanosoma cruzi strain CL Brener]
gi|3885846	beta tubulin [Trypanosoma cruzi]
gi|71667953	hypothetical protein [Trypanosoma cruzi strain CL Brener]
gi|71399455	surface protein TolT [Trypanosoma cruzi strain CL Brener]
gi|5566209	enolase 1 [Trypanosoma cruzi]
gi|71662736	hypothetical protein [Trypanosoma cruzi strain CL Brener]
gi|1170958	RecName: Full=Macrophage infectivity potentiator; AltName: Full=Peptidyl-prolyl cis-trans isomerase
gi|71413591	hypothetical protein [Trypanosoma cruzi strain CL Brener]
gi|119859	RecName: Full=Flagellar calcium-binding protein; Short=FCABP; AltName: Full=1F8 protein; AltName: F
gi|71425779	I/6 autoantigen [Trypanosoma cruzi strain CL Brener]
gi|71659715	cyclophilin A [Trypanosoma cruzi strain CL Brener]
gi|71406218	COP-coated vesicle membrane protein gp25L precursor [Trypanosoma cruzi strain CL Brener]
gi|1314208	alpha-tubulin
gi|120679	RecName: Full=Glyceraldehyde-3-phosphate dehydrogenase, glycosomal; Short=GAPDH
gi|71404821	trans-sialidase [Trypanosoma cruzi strain CL Brener]
gi|704459	elongation factor 1 alpha [Trypanosoma cruzi]
gi|168830555	translation elongation factor 1 alpha [Andalucia incarcerata]
gi|71659663	hypothetical protein [Trypanosoma cruzi strain CL Brener]
gi|71655052	reiske iron-sulfur protein precursor [Trypanosoma cruzi strain CL Brener]
gi|71663174	succinyl-CoA ligase [GDP-forming] beta-chain [Trypanosoma cruzi]
gi|133068	60S acidic ribosomal protein P2-B (P2B)
gi|71666956	hypothetical protein [Trypanosoma cruzi strain CL Brener]
gi|71648964	hypothetical protein [Trypanosoma cruzi strain CL Brener]
gi|71403861	trans-sialidase [Trypanosoma cruzi strain CL Brener]
gi|71655600	mitochondrial processing peptidase, beta subunit [Trypanosoma cruzi strain CL Brener]
gi|67479249	heat shock protein70, hsp70A2 [Entamoeba histolytica HM-1:IMSS]
gi|33694250	heat shock protein 70 [Euglena gracilis]
gi|84105389	cytosolic heat shock protein 70 [Trimastix marina]

gi|55824404	heat shock protein 70 cytosolic isoform [Cryptobia helicis]
gi|23306650	heat shock protein 70 [Carpediemonas membranifera]
gi|123592	RecName: Full=Heat shock 70 kDa protein
gi|10119899	pyruvate phosphate dikinase 1 [Trypanosoma cruzi]
gi|71409962	calpain-like cysteine peptidase [Trypanosoma cruzi strain CL Brener]
gi|71660675	6-phospho-1-fructokinase [Trypanosoma cruzi strain CL Brener]
gi|9954108	RNA binding protein RGGm [Trypanosoma cruzi]
gi|71656483	hypothetical protein [Trypanosoma cruzi strain CL Brener]
gi|71416147	histone H2A [Trypanosoma cruzi strain CL Brener]
gi|73536838	histone H2A [Leishmania major strain Friedlin]
gi|71407337	hypothetical protein [Trypanosoma cruzi strain CL Brener]
gi|123615	RecName: Full=Heat shock 70 kDa protein, mitochondrial; Flags: Precursor
gi|71420500	cytochrome C oxidase subunit VI [Trypanosoma cruzi strain CL Brener]
gi|167375825	hypothetical protein [Entamoeba dispar SAW760]
gi|71651239	thiolase protein-like protein [Trypanosoma cruzi strain CL Brener]
gi|123401786	hypothetical protein [Trichomonas vaginalis G3]
gi|225587	tubulin alpha
gi|84105377	alpha tubulin 2 [Rhynchopus sp. ATCC 50230]
gi|116222245	alpha tubulin [Thaumatomonas sp. TMT002]
gi|71413646	hypothetical protein [Trypanosoma cruzi strain CL Brener]
gi|154420635	DEAD/DEAH box helicase family protein [Trichomonas vaginalis G3]
gi|158577406	glycosomal glyceraldehyde-3-phosphate dehydrogenase [Crithidia oncopelti]
gi|71413449	RNA-binding protein [Trypanosoma cruzi strain CL Brener]

Probability Based Mowse Score

Ions score is -10*Log(P), where P is the probability that the observed match is a random event.
Individual ions scores > 42 indicate identity or extensive homology (p<0.05).
Protein scores are derived from ions scores as a non-probabilistic basis for ranking protein hits.

Score Distribution

Peptide Summary Report

Format As | Peptide Summary | Help

Significance threshold p< 0.05 | Max. number of hits AUTO

Standard scoring ○ MudPIT scoring ◉ | Ions score cut-off 0 | Show sub-sets ☐

Show pop-ups ◉ Suppress pop-ups ○ | Sort unassigned Decreasing Score | Require bold red ☐

Select All | Select None | Search Selected | ☐ **Error tolerant** | Archive Report

1. gi|3023478 **Mass:** 59374 **Score:** 374 **Queries matched:** 8

RecName: Full=Chaperonin HSP60, mitochondrial; Short=Protein Cpn60; AltName: Full=groEL protein; Al

☐ Check to include this hit in error tolerant search or archive report

	Query	Observed	Mr(expt)	Mr(calc)	Delta	Miss	Score	Expect	Rank	Peptide
☑	1482	557.86	1113.71	1113.64	0.07	0	94	2.1e-07	1	R.AVSAVATTLGPK.G
☑	1673	602.37	1202.73	1202.58	0.15	0	55	0.0018	1	K.VGGGSEVEVNEK.K
☑	1798	659.92	1317.82	1317.69	0.13	0	88	9.7e-07	1	R.NVIIEQSYGAPK.I
☑	1806	670.37	1338.72	1338.61	0.12	0	87	1e-06	1	R.GLIDGETSDYNR.E
☑	1871	728.93	1455.85	1455.80	0.05	0	102	3.2e-08	1	R.AVGVILQSVAEQSR.K
☑	1964	804.04	1606.06	1605.92	0.14	0	22	3.2	1	R.AAVQEGIVPGGGVALLR.A
☑	2020	846.95	1691.88	1691.81	0.07	0	108	8.7e-09	1	K.VLENNDVTVGYDAQR.D
☑	2358	844.48	2530.41	2530.29	0.12	1	59	0.00048	1	R.KVTSTENIVQVATISANGDEELGR.L

Proteins matching the same set of peptides:

gi|71665068 **Mass:** 59129 **Score:** 374 **Queries matched:** 8

chaperonin HSP60, mitochondrial precursor [Trypanosoma cruzi strain CL Brener]

gi|71665064 **Mass:** 59351 **Score:** 374 **Queries matched:** 8

chaperonin HSP60, mitochondrial precursor [Trypanosoma cruzi strain CL Brener]

---

2. gi|50659756 **Mass:** 71330 **Score:** 271 **Queries matched:** 7

heat shock protein 70 [Trypanosoma cruzi]

☐ Check to include this hit in error tolerant search or archive report

	Query	Observed	Mr(expt)	Mr(calc)	Delta	Miss	Score	Expect	Rank	Peptide
☑	1797	658.38	1314.74	1314.62	0.12	0	6	1.2e+02	1	K.FEELNMELFK.G + Oxidation (M)
☑	1862	725.43	1448.85	1448.75	0.10	0	82	3.1e-06	1	R.ITPSVVAFTETER.L
☑	1892	741.99	1481.97	1481.78	0.19	0	70	4.7e-05	1	K.SDIHEIVLVGGSTR.V
☑	1993	553.69	1658.05	1657.90	0.14	0	(8)	84	1	R.IINEPTAAAIAYGLNK.A
☑	1994	830.03	1658.05	1657.90	0.15	0	113	2.3e-09	1	R.IINEPTAAAIAYGLNK.A
☑	1996	832.84	1663.66	1663.83	-0.17	0	7	1.1e+02	1	K.NAVVTVPAYFNDAQR.Q
☑	2028	851.47	1700.92	1700.78	0.14	0	95	1.5e-07	1	R.VEVDSLTEGFDFSEK.I

Proteins matching the same set of peptides:
gi|71415505 **Mass:** 71272 **Score:** 271 **Queries matched:** 7
glucose-regulated protein 78 [Trypanosoma cruzi strain CL Brener]

---

3. gi|71414968 **Mass:** 125435 **Score:** 250 **Queries matched:** 11
microtubule-associated protein [Trypanosoma cruzi strain CL Brener]
☐ Check to include this hit in error tolerant search or archive report

	Query	Observed	Mr(expt)	Mr(calc)	Delta	Miss	Score	Expect	Rank	Peptide
☑	1865	726.93	1451.84	1451.73	0.11	0	79	6e-06	1	R.ALPLEEQEDVGPR.H
☑	1868	727.42	1452.83	1452.71	0.12	0	81	3.7e-06	1	R.ALPLEEEEDVGPR.H
☑	1879	734.40	1466.78	1466.70	0.08	0	69	7.5e-05	1	R.ALPQEEQEDVGPR.H
☑	1880	489.95	1466.83	1466.70	0.13	0	(35)	0.18	1	R.ALPQEEQEDVGPR.H
☑	1881	734.90	1467.78	1467.68	0.10	0	73	2.2e-05	1	R.ALPQEEEEDVGPR.H
☑	1974	542.01	1623.01	1622.80	0.21	1	37	0.087	1	K.RALPQEEQEDVGPR.H
☑	1977	542.32	1623.94	1623.79	0.15	1	40	0.052	1	K.RALPQEEEEDVGPR.H
☑	2068	900.02	1798.02	1797.85	0.16	0	28	0.71	1	R.STTQDAYRPVDPSAYK.R
☑	2069	600.35	1798.02	1797.85	0.17	0	(28)	0.77	1	R.STTQDAYRPVDPSAYK.R
	2072	904.11	1806.21	1805.89	0.32	0	0	5.9e+02	2	R.HVDPSVYTTTNQAVFK.D
☑	2146	652.41	1954.20	1953.95	0.24	1	41	0.041	1	R.STTQDAYRPVDPSAYKR.A

---

4. gi|296214 **Mass:** 70909 **Score:** 178 **Queries matched:** 5
P69 antigen [Trypanosoma congolense]
☐ Check to include this hit in error tolerant search or archive report

Query	Observed	Mr(expt)	Mr(calc)	Delta	Miss	Score	Expect	Rank	Peptide
☑ 1619	**1171.38**	**1170.37**	**1170.64**	**-0.27**	**0**	**8**	**91**	**1**	**K.FELTGIPPAAR.G**
1993	553.69	1658.05	1657.90	0.14	0	(8)	84	1	R.IINEPTAAAIAYGLNK.A
1994	830.03	1658.05	1657.90	0.15	0	113	2.3e-09	1	R.IINEPTAAAIAYGLNK.A
1996	832.84	1663.66	1663.83	-0.17	0	7	1.1e+02	1	K.NAVVTVPAYFNDAQR.Q
2028	851.47	1700.92	1700.78	0.14	0	95	1.5e-07	1	R.VEVDSLTEGFDFSEK.I

5. gi|71413559 **Mass:** 44080 **Score:** 176 **Queries matched:** 9
hypothetical protein [Trypanosoma cruzi strain CL Brener]

☐ Check to include this hit in error tolerant search or archive report

Query	Observed	Mr(expt)	Mr(calc)	Delta	Miss	Score	Expect	Rank	Peptide
☑ 1517	**563.35**	**1124.68**	**1124.63**	**0.05**	**0**	**(16)**	**12**	**1**	**R.QFIPASLPPR.Q**
☑ 1518	**563.37**	**1124.72**	**1124.63**	**0.09**	**0**	**45**	**0.015**	**1**	**R.QFIPASLPPR.Q**
☑ 1519	**563.39**	**1124.77**	**1124.63**	**0.14**	**0**	**(43)**	**0.025**	**1**	**R.QFIPASLPPR.Q**
☑ 201	**568.90**	**1135.78**	**1135.56**	**0.22**	**0**	**41**	**0.08**	**1**	**K.GVEDVEPVHR.K**
☑ 1646	**594.37**	**1186.72**	**1186.63**	**0.09**	**0**	**65**	**0.0002**	**1**	**K.ALTEEWILGR.K**
☑ 77	**480.40**	**1438.19**	**1437.76**	**0.43**	**0**	**45**	**0.04**	**1**	**K.IVHASPSDFLAGPK.E**
☑ 2021	**846.98**	**1691.95**	**1691.85**	**0.10**	**0**	**93**	**2.5e-07**	**1**	**K.ANESLDVLNLGQYTR.E**
☑ 2122	**637.36**	**1909.07**	**1908.97**	**0.10**	**0**	**32**	**0.27**	**1**	**K.DLVEAPARPASANAAAGGSGK.K**
☑ 345	**645.80**	**1934.38**	**1933.99**	**0.40**	**1**	**14**	**1.1e+02**	**1**	**R.NKANESLDVLNLGQYTR.E**

6. gi|71667211 **Mass:** 30381 **Score:** 161 **Queries matched:** 3
hypothetical protein [Trypanosoma cruzi strain CL Brener]

☐ Check to include this hit in error tolerant search or archive report

Query	Observed	Mr(expt)	Mr(calc)	Delta	Miss	Score	Expect	Rank	Peptide
☑ 1952	**787.06**	**1572.10**	**1571.92**	**0.17**	**0**	**96**	**1.4e-07**	**1**	**R.AIGSTITLITNSLIR.Y**
☑ 2265	**1124.62**	**2247.22**	**2247.11**	**0.11**	**0**	**104**	**1.6e-08**	**1**	**R.YEGTLGQIDGPNNTVSLTNVR.V**
☑ 2330	**805.16**	**2412.46**	**2412.24**	**0.21**	**0**	**27**	**0.79**	**1**	**R.GQEAGLAQIPPADQLFDQIVFR.G**

7. gi|71410853 **Mass:** 10694 **Score:** 157 **Queries matched:** 5

10 kDa heat shock protein [Trypanosoma cruzi strain CL Brener]

☐ Check to include this hit in error tolerant search or archive report

	Query	Observed	Mr(expt)	Mr(calc)	Delta	Miss	Score	Expect	Rank	Peptide
☑	1639	591.44	1180.87	1180.68	0.19	0	37	0.086	1	K.AGVLIPEQVAGK.V
☑	1801	665.46	1328.91	1328.73	0.18	0	94	2.1e-07	1	K.VNEGTVVAVAAATK.D
	1995	831.99	1661.96	1661.85	0.11	0	23	2.4	2	K.VDDTVLLPEFGGSSVK.V
	179	555.03	1662.08	1661.85	0.23	0	(17)	31	2	K.VDDTVLLPEFGGSSVK.V
☑	2276	1129.62	2257.23	2257.08	0.15	0	85	1.2e-06	1	K.VEGEEFFLYNEDSLLGVLQG.-

Proteins matching the same set of peptides:

gi|71410857 **Mass:** 20085 **Score:** 157 **Queries matched:** 5
10 kDa heat shock protein [Trypanosoma cruzi strain CL Brener]

---

8. gi|3885846 **Mass:** 9102 **Score:** 142 **Queries matched:** 2
beta tubulin [Trypanosoma cruzi]

☐ Check to include this hit in error tolerant search or archive report

	Query	Observed	Mr(expt)	Mr(calc)	Delta	Miss	Score	Expect	Rank	Peptide
☑	1807	671.36	1340.70	1340.64	0.06	0	81	3.8e-06	1	R.INVYFDEATGGR.Y
☑	2097	924.48	1846.94	1846.87	0.07	0	93	2.5e-07	1	R.EIVCVQAGQCGNQIGSK.F + 2 Carbamidomethyl (C)

Proteins matching the same set of peptides:

gi|91983178 **Mass:** 10597 **Score:** 142 **Queries matched:** 2
beta tubulin [Crithidia deanei]

gi|111115853 **Mass:** 49628 **Score:** 142 **Queries matched:** 2
beta tubulin [Trypanosoma evansi]

gi|115504281 **Mass:** 49672 **Score:** 142 **Queries matched:** 2
beta tubulin [Trypanosoma brucei]

gi|166203636 **Mass:** 24112 **Score:** 142 **Queries matched:** 2
beta tubulin [Trypanosoma evansi]

gi|169125731 **Mass:** 49655 **Score:** 142 **Queries matched:** 2
beta-tubulin [Trypanosoma evansi]

gi|91983201 **Mass:** 42807 **Score:** 142 **Queries matched:** 2

beta tubulin [Trypanosoma grayi]
gi|1220547 Mass: 49410 Score: 142 Queries matched: 2
beta tubulin
gi|3915883 Mass: 49541 Score: 142 Queries matched: 2
Tubulin beta chain (Beta-tubulin)
gi|18568139 Mass: 49668 Score: 142 Queries matched: 2
beta tubulin 1.9 [Trypanosoma cruzi]
gi|71656281 Mass: 49668 Score: 142 Queries matched: 2
beta tubulin [Trypanosoma cruzi strain CL Brener]
gi|74229926 Mass: 49696 Score: 142 Queries matched: 2
beta-tubulin [Trypanosoma danilewskyi]

---

9. gi|71667953 Mass: 45869 Score: 138 Queries matched: 7
hypothetical protein [Trypanosoma cruzi strain CL Brener]
☐ Check to include this hit in error tolerant search or archive report

	Query	Observed	Mr(expt)	Mr(calc)	Delta	Miss	Score	Expect	Rank	Peptide
☑	1335	529.87	1057.72	1057.60	0.12	0	34	0.22	1	K.LQVELLDTK.H
	1447	549.87	1097.72	1097.57	0.15	0	21	3.3	2	K.NVLPEEIER.V
☑	1674	602.88	1203.75	1203.63	0.11	0	50	0.0056	1	R.IQSILSETGEK.E
☑	1763	637.89	1273.77	1273.61	0.16	0	62	0.0003	1	R.VENGELNALCR.V + Carbamidomethyl (C)
☑	1836	462.29	1383.84	1383.64	0.19	0	67	0.0001	1	R.HHSDDEVAAYIK.E
☑	1838	692.95	1383.89	1383.64	0.25	0	(38)	0.077	1	R.HHSDDEVAAYIK.E
☑	1851	714.45	1426.88	1426.73	0.15	0	55	0.0018	1	K.ELPELQVLSEDR.A

---

10. gi|71399455 Mass: 24752 Score: 134 Queries matched: 2
surface protein TolT [Trypanosoma cruzi strain CL Brener]
☐ Check to include this hit in error tolerant search or archive report

	Query	Observed	Mr(expt)	Mr(calc)	Delta	Miss	Score	Expect	Rank	Peptide
☑	1649	594.87	1187.72	1187.64	0.08	0	63	0.00028	1	R.AAEQTVLSLEK.A
☑	1902	750.99	1499.96	1499.83	0.13	0	110	5e-09	1	R.TLAQDVAATASALLR.Q

Proteins matching the same set of peptides:

gi|71402301 **Mass:** 32319 **Score:** 134 **Queries matched:** 2
surface protein TolT [Trypanosoma cruzi strain CL Brener]

---

11. gi|5566209 **Mass:** 37655 **Score:** 106 **Queries matched:** 2
enolase 1 [Trypanosoma cruzi]

☐ Check to include this hit in error tolerant search or archive report

	Query	Observed	Mr(expt)	Mr(calc)	Delta	Miss	Score	Expect	Rank	Peptide
☑	608	787.48	1572.94	1572.81	0.13	0	106	3.7e-08	1	K.AQVVGDDLTVTNVSR.I
☑	2342	823.21	2466.59	2466.30	0.30	1	11	48	1	K.NVNDVLAPALVGKDELQQSTLDK.L

Proteins matching the same set of peptides:
gi|5566210 **Mass:** 37596 **Score:** 106 **Queries matched:** 2
enolase 2 [Trypanosoma cruzi]
gi|71665461 **Mass:** 46415 **Score:** 106 **Queries matched:** 2
enolase [Trypanosoma cruzi strain CL Brener]

---

12. gi|71662736 **Mass:** 42788 **Score:** 94 **Queries matched:** 4
hypothetical protein [Trypanosoma cruzi strain CL Brener]

☐ Check to include this hit in error tolerant search or archive report

	Query	Observed	Mr(expt)	Mr(calc)	Delta	Miss	Score	Expect	Rank	Peptide
☑	961	466.29	930.57	930.48	0.09	0	50	0.0058	1	K.VDLDNLSR.R
☑	1753	631.35	1260.69	1260.56	0.13	0	66	0.00014	1	R.DLNSAEEEAQR.S
☑	1755	632.39	1262.77	1262.65	0.11	0	58	0.00089	1	R.QLDSINVFAEK.Y
	2008	559.68	1676.02	1675.88	0.14	1	4	2e+02	5	R.FVLASIEEKENELR.E

---

13. gi|1170958 **Mass:** 22122 **Score:** 88 **Queries matched:** 2
RecName: Full=Macrophage infectivity potentiator; AltName: Full=Peptidyl-prolyl cis-trans isomerase

☐ Check to include this hit in error tolerant search or archive report

	Query	Observed	Mr(expt)	Mr(calc)	Delta	Miss	Score	Expect	Rank	Peptide
☑	108	508.80	1015.59	1015.58	0.01	0	49	0.016	1	K.LPSGLVFQR.I
☑	1626	587.86	1173.71	1173.59	0.12	0	76	1.3e-05	1	R.TAEEVDEILR.K

Proteins matching the same set of peptides:

gi|21465757 **Mass:** 18822 **Score:** 88 **Queries matched:** 2
Chain A, Trypanosoma Cruzi Macrophage Infectivity Potentiator (Tcmip)

gi|71418561 **Mass:** 22140 **Score:** 88 **Queries matched:** 2
macrophage infectivity potentiator, precursor [Trypanosoma cruzi strain CL Brener]

---

14. gi|71413591 **Mass:** 16740 **Score:** 87 **Queries matched:** 1
hypothetical protein [Trypanosoma cruzi strain CL Brener]

☐ Check to include this hit in error tolerant search or archive report

	Query	Observed	Mr(expt)	Mr(calc)	Delta	Miss	Score	Expect	Rank	Peptide
☑	1891	741.94	1481.87	1481.75	0.12	0	87	9.7e-07	1	K.TETVSSTETVVTTK.N

---

15. gi|119859 **Mass:** 23721 **Score:** 86 **Queries matched:** 3
RecName: Full=Flagellar calcium-binding protein; Short=FCABP; AltName: Full=1F8 protein; AltName: F

☐ Check to include this hit in error tolerant search or archive report

	Query	Observed	Mr(expt)	Mr(calc)	Delta	Miss	Score	Expect	Rank	Peptide
☑	1142	495.32	988.63	988.52	0.10	0	38	0.078	1	K.VEDPAALFK.E
☑	1845	707.87	1413.73	1413.59	0.14	0	49	0.0059	1	K.LDADGDPDNVPESA.-
☑	518	737.99	1473.97	1473.68	0.29	0	71	0.00013	1	K.GSEDFVEFLEFR.L

Proteins matching the same set of peptides:

gi|1552212 **Mass:** 23727 **Score:** 86 **Queries matched:** 3
calcium-binding protein [Trypanosoma cruzi]

gi|1556494 **Mass:** 23627 **Score:** 86 **Queries matched:** 3
24 kDa flagellar calcium-binding protein [Trypanosoma cruzi]

gi|71404399 **Mass:** 11930 **Score:** 86 **Queries matched:** 3
flagellar calcium-binding protein [Trypanosoma cruzi strain CL Brener]

gi|71406002 **Mass:** 23827 **Score:** 86 **Queries matched:** 3
flagellar calcium-binding protein [Trypanosoma cruzi strain CL Brener]

gi|71406004 **Mass:** 23799 **Score:** 86 **Queries matched:** 3
flagellar calcium-binding protein [Trypanosoma cruzi strain CL Brener]

gi|71412217 **Mass:** 23707 **Score:** 86 **Queries matched:** 3
calcium-binding protein [Trypanosoma cruzi strain CL Brener]
gi|71656918 **Mass:** 23713 **Score:** 86 **Queries matched:** 3
flagellar calcium-binding protein [Trypanosoma cruzi strain CL Brener]
gi|487896 **Mass:** 23803 **Score:** 86 **Queries matched:** 3
calcium-binding protein

---

16. gi|71425779 **Mass:** 23211 **Score:** 85 **Queries matched:** 2
I/6 autoantigen [Trypanosoma cruzi strain CL Brener]
☐ Check to include this hit in error tolerant search or archive report

	Query	Observed	Mr(expt)	Mr(calc)	Delta	Miss	Score	Expect	Rank	Peptide
☑	1737	**623.36**	**1244.71**	**1244.60**	**0.11**	**0**	**85**	**1.5e-06**	**1**	**M.PISPAEAFEER.H**
	1939	**776.00**	**1549.98**	**1549.68**	**0.30**	**1**	**4**	**2e+02**	**4**	**K.NPNNCLDARMMAK.H + Carbamidomethyl (C); Oxidation (M)**

Proteins matching the same set of peptides:
gi|71655864 **Mass:** 23105 **Score:** 85 **Queries matched:** 2
I/6 autoantigen [Trypanosoma cruzi strain CL Brener]

---

17. gi|71659715 **Mass:** 18769 **Score:** 85 **Queries matched:** 2
cyclophilin A [Trypanosoma cruzi strain CL Brener]
☐ Check to include this hit in error tolerant search or archive report

	Query	Observed	Mr(expt)	Mr(calc)	Delta	Miss	Score	Expect	Rank	Peptide
☑	1804	**667.91**	**1333.80**	**1333.73**	**0.08**	**0**	**67**	**0.0001**	**1**	**R.VVFELFADAVPK.T**
☑	480	**720.48**	**1438.95**	**1438.72**	**0.23**	**0**	**46**	**0.039**	**1**	**K.VFFDVSIGGQSAGR.V**

---

18. gi|71406218 **Mass:** 36425 **Score:** 83 **Queries matched:** 1
COP-coated vesicle membrane protein gp25L precursor [Trypanosoma cruzi strain CL Brener]
☐ Check to include this hit in error tolerant search or archive report

	Query	Observed	Mr(expt)	Mr(calc)	Delta	Miss	Score	Expect	Rank	Peptide
☑	1925	**763.92**	**1525.83**	**1525.69**	**0.14**	**0**	**83**	**2.3e-06**	**1**	**R.VGSENNDYSELATK.E**

Proteins matching the same set of peptides:
gi|71652059 **Mass:** 24859 **Score:** 83 **Queries matched:** 1
COP-coated vesicle membrane protein gp25L precursor [Trypanosoma cruzi strain CL Brener]

---

19. gi|1314208 **Mass:** 46927 **Score:** 82 **Queries matched:** 2
alpha-tubulin
☐ Check to include this hit in error tolerant search or archive report

	Query	Observed	Mr(expt)	Mr(calc)	Delta	Miss	Score	Expect	Rank	Peptide
☑	2035	858.56	1715.10	1714.91	0.19	0	82	2.8e-06	1	R.AVFLDLEPTVVDEIR.T
☑	2288	765.49	2293.46	2293.20	0.26	1	16	12	1	R.AVFLDLEPTVVDEIRTGTYR.Q

Proteins matching the same set of peptides:
gi|3915082 **Mass:** 49696 **Score:** 82 **Queries matched:** 2
Tubulin alpha chain
gi|71397525 **Mass:** 49768 **Score:** 82 **Queries matched:** 2
alpha tubulin [Trypanosoma cruzi strain CL Brener]
gi|74229924 **Mass:** 49766 **Score:** 82 **Queries matched:** 2
alpha-tubulin [Trypanosoma danilewskyi]
gi|91983193 **Mass:** 38117 **Score:** 82 **Queries matched:** 2
alpha tubulin [Parabodo caudatus]
gi|91983196 **Mass:** 49777 **Score:** 82 **Queries matched:** 2
alpha tubulin [Trypanosoma cyclops]
gi|91983200 **Mass:** 49749 **Score:** 82 **Queries matched:** 2
alpha tubulin [Trypanosoma grayi]

---

20. gi|120679 **Mass:** 39036 **Score:** 74 **Queries matched:** 4
RecName: Full=Glyceraldehyde-3-phosphate dehydrogenase, glycosomal; Short=GAPDH
☐ Check to include this hit in error tolerant search or archive report

	Query	Observed	Mr(expt)	Mr(calc)	Delta	Miss	Score	Expect	Rank	Peptide
☑	1835	692.90	1383.78	1383.77	0.01	0	42	0.03	1	R.AAAVNIIPSTTGAAK.A
☑	1837	692.94	1383.87	1383.77	0.10	0	(40)	0.046	1	R.AAAVNIIPSTTGAAK.A
☑	2075	604.65	1810.92	1810.81	0.11	0	24	1.6	1	K.IVSWYDNEWGYSHR.V
☑	2262	747.89	2240.65	2240.06	0.59	0	67	0.00019	1	K.GILGYTDEELVSADFINDNR.S

Proteins matching the same set of peptides:
gi|71422448 **Mass:** 39008 **Score:** 74 **Queries matched:** 4
glyceraldehyde 3-phosphate dehydrogenase [Trypanosoma cruzi strain CL Brener]
gi|71650185 **Mass:** 38993 **Score:** 74 **Queries matched:** 4
glyceraldehyde 3-phosphate dehydrogenase [Trypanosoma cruzi strain CL Brener]

---

21. gi|71404821 **Mass:** 88843 **Score:** 72 **Queries matched:** 1
trans-sialidase [Trypanosoma cruzi strain CL Brener]
☐ Check to include this hit in error tolerant search or archive report

	Query	Observed	Mr(expt)	Mr(calc)	Delta	Miss	Score	Expect	Rank	Peptide
☑	2153	985.08	1968.14	1967.86	0.29	0	72	3.1e-05	1	K.ATGSSAGEDSESSGAAGTDLAK.G

Proteins matching the same set of peptides:
gi|71393748 **Mass:** 37829 **Score:** 72 **Queries matched:** 1
trans-sialidase [Trypanosoma cruzi strain CL Brener]

---

22. gi|704459 **Mass:** 43546 **Score:** 70 **Queries matched:** 2
elongation factor 1 alpha [Trypanosoma cruzi]
☐ Check to include this hit in error tolerant search or archive report

	Query	Observed	Mr(expt)	Mr(calc)	Delta	Miss	Score	Expect	Rank	Peptide
☑	1249	513.38	1024.74	1024.60	0.14	0	70	4.3e-05	1	K.IGGIGTVPVGR.V
☑	2352	836.44	2506.30	2506.26	0.04	0	17	9	1	R.VETGTMKPGDVVTFAPANVTTEVK.S + Oxidation (M)

Proteins matching the same set of peptides:
gi|1929445 **Mass:** 49018 **Score:** 70 **Queries matched:** 2
elongation factor 1-alpha [Trypanosoma cruzi]
gi|52424046 **Mass:** 49067 **Score:** 70 **Queries matched:** 2
elongation factor alpha G5 [Trypanosoma cruzi]
gi|61207234 **Mass:** 47727 **Score:** 70 **Queries matched:** 2
elongation factor 1-alpha [Trypanosoma cruzi]
gi|61207240 **Mass:** 47728 **Score:** 70 **Queries matched:** 2

elongation factor 1-alpha [Trypanosoma cruzi]
gi|61207250 **Mass:** 47741 **Score:** 70 **Queries matched:** 2
elongation factor 1-alpha [Trypanosoma cruzi]
gi|61207252 **Mass:** 47688 **Score:** 70 **Queries matched:** 2
elongation factor 1-alpha [Trypanosoma cruzi]
gi|61207254 **Mass:** 47727 **Score:** 70 **Queries matched:** 2
elongation factor 1-alpha [Trypanosoma cruzi]
gi|61207256 **Mass:** 47843 **Score:** 70 **Queries matched:** 2
elongation factor 1-alpha [Trypanosoma cruzi]
gi|61207270 **Mass:** 48276 **Score:** 70 **Queries matched:** 2
elongation factor 1-alpha [Trypanosoma cruzi]
gi|61207272 **Mass:** 47767 **Score:** 70 **Queries matched:** 2
elongation factor 1-alpha [Trypanosoma cruzi]
gi|61207276 **Mass:** 47784 **Score:** 70 **Queries matched:** 2
elongation factor 1-alpha [Trypanosoma cruzi]
gi|61207288 **Mass:** 47711 **Score:** 70 **Queries matched:** 2
elongation factor 1-alpha [Trypanosoma cruzi]
gi|61207290 **Mass:** 47769 **Score:** 70 **Queries matched:** 2
elongation factor 1-alpha [Trypanosoma cruzi]
gi|61207296 **Mass:** 47667 **Score:** 70 **Queries matched:** 2
elongation factor 1-alpha [Trypanosoma cruzi]
gi|61207306 **Mass:** 47815 **Score:** 70 **Queries matched:** 2
elongation factor 1-alpha [Trypanosoma cruzi]
gi|61207316 **Mass:** 47797 **Score:** 70 **Queries matched:** 2
elongation factor 1-alpha [Trypanosoma cruzi]
gi|71403912 **Mass:** 48316 **Score:** 70 **Queries matched:** 2
elongation factor 1-alpha (EF-1-alpha) [Trypanosoma cruzi strain CL Brener]
gi|71403914 **Mass:** 49125 **Score:** 70 **Queries matched:** 2
elongation factor 1-alpha (EF-1-alpha) [Trypanosoma cruzi strain CL Brener]
gi|71403916 **Mass:** 30716 **Score:** 70 **Queries matched:** 2
elongation factor 1-alpha (EF-1-alpha) [Trypanosoma cruzi strain CL Brener]
gi|71408910 **Mass:** 49083 **Score:** 70 **Queries matched:** 2
elongation factor 1-alpha (EF-1-alpha) [Trypanosoma cruzi strain CL Brener]
gi|71408922 **Mass:** 42751 **Score:** 70 **Queries matched:** 2
elongation factor 1-alpha (EF-1-alpha) [Trypanosoma cruzi strain CL Brener]
gi|71664927 **Mass:** 49097 **Score:** 70 **Queries matched:** 2

elongation factor 1-alpha (EF-1-alpha) [Trypanosoma cruzi strain CL Brener]

---

23. gi|168830555 **Mass:** 44024 **Score:** 70 **Queries matched:** 2
translation elongation factor 1 alpha [Andalucia incarcerata]
☐ Check to include this hit in error tolerant search or archive report

	Query	Observed	Mr(expt)	Mr(calc)	Delta	Miss	Score	Expect	Rank	Peptide
	1249	513.38	1024.74	1024.60	0.14	0	70	4.3e-05	1	K.IGGIGTVPVGR.V
	66	**467.34**	**1399.00**	**1398.62**	**0.38**	**1**	**3**	**5e+02**	**3**	**K.MDDKSVNYGQAR.F + Oxidation (M)**

---

24. gi|71659663 **Mass:** 31532 **Score:** 70 **Queries matched:** 2
hypothetical protein [Trypanosoma cruzi strain CL Brener]
☐ Check to include this hit in error tolerant search or archive report

	Query	Observed	Mr(expt)	Mr(calc)	Delta	Miss	Score	Expect	Rank	Peptide
	860	**895.65**	**894.64**	**894.50**	**0.14**	**2**	**3**	**8.8e+02**	**2**	**K.RKTVSPAH.-**
☑	1986	**818.38**	**1634.74**	**1634.78**	**-0.05**	**0**	**70**	**5.3e-05**	**1**	**R.MVSENESAINDVVTK.L**

---

25. gi|71655052 **Mass:** 33662 **Score:** 69 **Queries matched:** 3
reiske iron-sulfur protein precursor [Trypanosoma cruzi strain CL Brener]
☐ Check to include this hit in error tolerant search or archive report

	Query	Observed	Mr(expt)	Mr(calc)	Delta	Miss	Score	Expect	Rank	Peptide
☑	1189	**501.88**	**1001.74**	**1001.58**	**0.16**	**0**	**40**	**0.06**	**1**	**R.IETTAGVALK.D**
☑	1644	**593.38**	**1184.75**	**1184.64**	**0.11**	**0**	**37**	**0.093**	**1**	**K.QLEGSNPLTVK.D**
☑	2456	**1004.86**	**3011.56**	**3011.38**	**0.18**	**0**	**48**	**0.0064**	**1**	**K.YSDPALCTVDTSSEVVLNTYPDGAPQGR.I + Carbamidomethyl (C)**

Proteins matching the same set of peptides:
gi|71657437 **Mass:** 33708 **Score:** 69 **Queries matched:** 3
reiske iron-sulfur protein precursor [Trypanosoma cruzi strain CL Brener]

---

26. gi|71663174 **Mass:** 45236 **Score:** 69 **Queries matched:** 1
succinyl-CoA ligase [GDP-forming] beta-chain [Trypanosoma cruzi]

☐ Check to include this hit in error tolerant search or archive report

	Query	Observed	Mr(expt)	Mr(calc)	Delta	Miss	Score	Expect	Rank	Peptide
☑	1589	580.36	1158.70	1158.61	0.08	0	69	7e-05	1	R.TLEEVEAALGK.I

---

27. gi|133068 **Mass:** 10919 **Score:** 68 **Queries matched:** 1
60S acidic ribosomal protein P2-B (P2B)
☐ Check to include this hit in error tolerant search or archive report

	Query	Observed	Mr(expt)	Mr(calc)	Delta	Miss	Score	Expect	Rank	Peptide
☑	1490	558.88	1115.75	1115.62	0.13	0	68	8.4e-05	1	R.SVATLVAEGAAK.M

Proteins matching the same set of peptides:
gi|71405064 **Mass:** 10861 **Score:** 68 **Queries matched:** 1
60S acidic ribosomal protein P2 [Trypanosoma cruzi strain CL Brener]

---

28. gi|71666956 **Mass:** 40800 **Score:** 67 **Queries matched:** 2
hypothetical protein [Trypanosoma cruzi strain CL Brener]
☐ Check to include this hit in error tolerant search or archive report

	Query	Observed	Mr(expt)	Mr(calc)	Delta	Miss	Score	Expect	Rank	Peptide
☑	1803	667.84	1333.66	1333.59	0.07	0	67	0.00011	1	K.QEINECVETGR.E + Carbamidomethyl (C)
☑	1946	522.37	1564.08	1563.83	0.25	1	21	3.8	1	K.IISVADQYVESGRK.I

---

29. gi|71648964 **Mass:** 52877 **Score:** 66 **Queries matched:** 1
hypothetical protein [Trypanosoma cruzi strain CL Brener]
☐ Check to include this hit in error tolerant search or archive report

	Query	Observed	Mr(expt)	Mr(calc)	Delta	Miss	Score	Expect	Rank	Peptide
☑	1985	815.47	1628.92	1628.78	0.15	0	66	0.00014	1	R.QLQEQSENLQQER.A

Proteins matching the same set of peptides:
gi|71659037 **Mass:** 52427 **Score:** 66 **Queries matched:** 1

hypothetical protein [Trypanosoma cruzi strain CL Brener]

---

30. gi|71403861 **Mass:** 76587 **Score:** 65 **Queries matched:** 1
trans-sialidase [Trypanosoma cruzi strain CL Brener]
☐ Check to include this hit in error tolerant search or archive report

	Query	Observed	Mr(expt)	Mr(calc)	Delta	Miss	Score	Expect	Rank	Peptide
☑	2077	**909.05**	**1816.09**	**1815.94**	**0.15**	**0**	**65**	**0.00016**	**1**	**R.SVEELQWVDLFVPQK.T**

Proteins matching the same set of peptides:
gi|71404338 **Mass:** 48533 **Score:** 65 **Queries matched:** 1
trans-sialidase [Trypanosoma cruzi strain CL Brener]

---

31. gi|71655600 **Mass:** 54205 **Score:** 64 **Queries matched:** 2
mitochondrial processing peptidase, beta subunit [Trypanosoma cruzi strain CL Brener]
☐ Check to include this hit in error tolerant search or archive report

	Query	Observed	Mr(expt)	Mr(calc)	Delta	Miss	Score	Expect	Rank	Peptide
☑	155	**536.97**	**1607.89**	**1607.86**	**0.03**	**0**	**25**	**3.8**	**1**	**R.VVVVGSGAVDHTALER.A**
☑	2336	**811.09**	**2430.26**	**2430.09**	**0.17**	**0**	**60**	**0.00039**	**1**	**R.CAFDSTSHGLGTPFYGTETGVAR.V + Carbamidomethyl (C)**

---

32. gi|67479249 **Mass:** 66261 **Score:** 62 **Queries matched:** 3
heat shock protein70, hsp70A2 [Entamoeba histolytica HM-1:IMSS]
☐ Check to include this hit in error tolerant search or archive report

	Query	Observed	Mr(expt)	Mr(calc)	Delta	Miss	Score	Expect	Rank	Peptide
	1619	1171.38	1170.37	1170.64	-0.27	0	8	91	1	R.FELTGIAPAPR.G
☑	1710	**614.89**	**1227.76**	**1227.62**	**0.14**	**0**	**62**	**0.0003**	**1**	**R.VEIIANDQGNR.T**
☑	1895	**744.42**	**1486.82**	**1486.69**	**0.13**	**0**	**28**	**0.76**	**1**	**R.TTPSYVAFTDTER.L**

Proteins matching the same set of peptides:
gi|183232244 **Mass:** 73979 **Score:** 62 **Queries matched:** 3
heat shock protein70, hsp70A2 [Entamoeba histolytica HM-1:IMSS]
gi|183234352 **Mass:** 70515 **Score:** 62 **Queries matched:** 3

heat shock protein 70 [Entamoeba histolytica HM-1:IMSS]
gi|183234972 **Mass:** 70518 **Score:** 62 **Queries matched:** 3
heat shock protein 70 [Entamoeba histolytica HM-1:IMSS]

---

33. gi|33694250 **Mass:** 50799 **Score:** 62 **Queries matched:** 3
heat shock protein 70 [Euglena gracilis]
☐ Check to include this hit in error tolerant search or archive report

Query	Observed	Mr(expt)	Mr(calc)	Delta	Miss	Score	Expect	Rank	Peptide
1710	614.89	1227.76	1227.62	0.14	0	62	0.0003	1	R.VEIIANDQGNR.T
71	**473.00**	**1415.97**	**1415.75**	**0.22**	**0**	**1**	**1.2e+03**	**7**	**K.SDGRPIIQVEFR.G**
1895	744.42	1486.82	1486.69	0.13	0	28	0.76	1	R.TTPSYVAFTDTER.L

---

34. gi|84105389 **Mass:** 67446 **Score:** 62 **Queries matched:** 3
cytosolic heat shock protein 70 [Trimastix marina]
☐ Check to include this hit in error tolerant search or archive report

Query	Observed	Mr(expt)	Mr(calc)	Delta	Miss	Score	Expect	Rank	Peptide
210	**573.28**	**1144.55**	**1144.67**	**-0.12**	**1**	**2**	**9.4e+02**	**7**	**K.NIPPAPRGVPK.I**
1710	614.89	1227.76	1227.62	0.14	0	62	0.0003	1	R.VEIIANDQGNR.T
1895	744.42	1486.82	1486.69	0.13	0	28	0.76	1	R.TTPSYVAFTDTER.L

---

35. gi|55824404 **Mass:** 69318 **Score:** 62 **Queries matched:** 3
heat shock protein 70 cytosolic isoform [Cryptobia helicis]
☐ Check to include this hit in error tolerant search or archive report

Query	Observed	Mr(expt)	Mr(calc)	Delta	Miss	Score	Expect	Rank	Peptide
1710	614.89	1227.76	1227.62	0.14	0	62	0.0003	1	R.VEIIANDQGNR.T
480	720.48	1438.95	1438.68	0.27	1	0	1.5e+03	8	R.MVSDAAKFEAQDK.E
1895	744.42	1486.82	1486.69	0.13	0	28	0.76	1	R.TTPSYVAFTDTER.L

---

36. gi|23306650 **Score:** 62 **Queries matched:** 2
heat shock protein 70 [Carpediemonas membranifera]

☐ Check to include this hit in error tolerant search or archive report

Query	Observed	Mr(expt)	Mr(calc)	Delta	Miss	Score	Expect	Rank	Peptide
1710	614.89	1227.76	1227.62	0.14	0	62	0.0003	1	R.VEIIANDQGNR.T
1972	540.35	1618.03	1617.96	0.07	1	0	4.2e+02	10	K.IHEIVLVGGSTRIPK.V

---

37. gi|123592 **Mass:** 56500 **Score:** 62 **Queries matched:** 1
RecName: Full=Heat shock 70 kDa protein
☐ Check to include this hit in error tolerant search or archive report

Query	Observed	Mr(expt)	Mr(calc)	Delta	Miss	Score	Expect	Rank	Peptide
1710	614.89	1227.76	1227.62	0.14	0	62	0.0003	1	R.LDIIANDQGNR.T

---

38. gi|10119899 **Mass:** 100749 **Score:** 62 **Queries matched:** 2
pyruvate phosphate dikinase 1 [Trypanosoma cruzi]
☐ Check to include this hit in error tolerant search or archive report

	Query	Observed	Mr(expt)	Mr(calc)	Delta	Miss	Score	Expect	Rank	Peptide
	1577	576.94	1151.87	1151.54	0.33	0	3	2.7e+02	4	R.SFGAEGVGLCR.T + Carbamidomethyl (C)
☑	1696	609.39	1216.77	1216.60	0.16	0	62	0.00032	1	K.TAEETLAAAGQR.V

Proteins matching the same set of peptides:
gi|71658999 **Mass:** 100785 **Score:** 62 **Queries matched:** 2
pyruvate phosphate dikinase [Trypanosoma cruzi strain CL Brener]
gi|71666490 **Mass:** 100767 **Score:** 62 **Queries matched:** 2
pyruvate phosphate dikinase [Trypanosoma cruzi strain CL Brener]

---

39. gi|71409962 **Mass:** 12839 **Score:** 61 **Queries matched:** 3
calpain-like cysteine peptidase [Trypanosoma cruzi strain CL Brener]
☐ Check to include this hit in error tolerant search or archive report

	Query	Observed	Mr(expt)	Mr(calc)	Delta	Miss	Score	Expect	Rank	Peptide
	1198	503.29	1004.56	1004.50	0.06	0	6	1.5e+02	2	K.DNGNGLLFR.I
☑	1622	586.80	1171.58	1171.53	0.05	0	41	0.043	1	K.QWAFYNDTK.E

☑ 1997	833.44	1664.87	1664.80	0.06	0	48	0.0078	1	K.YENGQPTFEGPTVVK.C

Proteins matching the same set of peptides:
gi|71411006 **Mass:** 12798 **Score:** 61 **Queries matched:** 3
calpain-like cysteine peptidase [Trypanosoma cruzi strain CL Brener]

---

40. gi|71660675 **Mass:** 53532 **Score:** 60 **Queries matched:** 1
6-phospho-1-fructokinase [Trypanosoma cruzi strain CL Brener]
☐ Check to include this hit in error tolerant search or archive report

Query	Observed	Mr(expt)	Mr(calc)	Delta	Miss	Score	Expect	Rank	Peptide
☑ 1991	551.36	1651.05	1650.82	0.22	0	60	0.00045	1	R.SHAAPLNEVTQEDLK.V

---

41. gi|9954108 **Mass:** 34491 **Score:** 58 **Queries matched:** 2
RNA binding protein RGGm [Trypanosoma cruzi]
☐ Check to include this hit in error tolerant search or archive report

Query	Observed	Mr(expt)	Mr(calc)	Delta	Miss	Score	Expect	Rank	Peptide
☑ 1849	710.43	1418.85	1418.70	0.14	0	52	0.0035	1	R.AVVEFVTPEDASR.A
☑ 2061	596.28	1785.82	1785.89	-0.07	0	34	0.19	1	R.VQVSGVSDETTWHTLK.D

Proteins matching the same set of peptides:
gi|71410145 **Mass:** 34635 **Score:** 58 **Queries matched:** 2
RNA-binding protein RGGm [Trypanosoma cruzi strain CL Brener]
gi|71652462 **Mass:** 33389 **Score:** 58 **Queries matched:** 2
RNA-binding protein RGGm [Trypanosoma cruzi strain CL Brener]

---

42. gi|71656483 **Mass:** 10547 **Score:** 55 **Queries matched:** 1
hypothetical protein [Trypanosoma cruzi strain CL Brener]
☐ Check to include this hit in error tolerant search or archive report

Query	Observed	Mr(expt)	Mr(calc)	Delta	Miss	Score	Expect	Rank	Peptide
☑ 1780	647.32	1292.62	1292.60	0.02	0	55	0.0016	1	R.LEENYNAGVER.E

43. gi|71416147 **Mass:** 14345 **Score:** 54 **Queries matched:** 3
histone H2A [Trypanosoma cruzi strain CL Brener]
☐ Check to include this hit in error tolerant search or archive report

	Query	Observed	Mr(expt)	Mr(calc)	Delta	Miss	Score	Expect	Rank	Peptide
☑	952	**465.32**	**928.62**	**928.55**	**0.07**	**0**	**(24)**	**2.3**	**1**	**K.AGLIFPVGR.V**
☑	953	**465.38**	**928.76**	**928.55**	**0.21**	**0**	**35**	**0.16**	**1**	**K.AGLIFPVGR.V**
☑	1523	**563.87**	**1125.72**	**1125.57**	**0.16**	**0**	**54**	**0.0017**	**1**	**R.HDDDLGTLLK.D**

Proteins matching the same set of peptides:
gi|71416149 **Mass:** 14327 **Score:** 54 **Queries matched:** 3
histone H2A [Trypanosoma cruzi strain CL Brener]

44. gi|73536838 **Mass:** 13814 **Score:** 54 **Queries matched:** 3
histone H2A [Leishmania major strain Friedlin]
☐ Check to include this hit in error tolerant search or archive report

Query	Observed	Mr(expt)	Mr(calc)	Delta	Miss	Score	Expect	Rank	Peptide
952	465.32	928.62	928.55	0.07	0	(24)	2.3	1	K.AGLIFPVGR.V
953	465.38	928.76	928.55	0.21	0	35	0.16	1	K.AGLIFPVGR.V
1523	563.87	1125.72	1125.57	0.16	0	54	0.0017	1	R.HDDDIGTLLK.S

Proteins matching the same set of peptides:
gi|73536842 **Mass:** 13841 **Score:** 54 **Queries matched:** 3
histone H2A [Leishmania major strain Friedlin]

45. gi|71407337 **Mass:** 16350 **Score:** 53 **Queries matched:** 2
hypothetical protein [Trypanosoma cruzi strain CL Brener]
☐ Check to include this hit in error tolerant search or archive report

	Query	Observed	Mr(expt)	Mr(calc)	Delta	Miss	Score	Expect	Rank	Peptide
☑	1602	**583.38**	**1164.76**	**1164.64**	**0.12**	**1**	**42**	**0.026**	**1**	**R.KITLSDFVDK.A**
☑	1901	**750.43**	**1498.85**	**1498.73**	**0.12**	**0**	**47**	**0.011**	**1**	**K.QFTSTDADLLFNK.V**

Proteins matching the same set of peptides:
gi|71651158 **Mass:** 16325 **Score:** 53 **Queries matched:** 2
hypothetical protein [Trypanosoma cruzi strain CL Brener]

---

46. gi|123615 **Mass:** 71102 **Score:** 51 **Queries matched:** 1
RecName: Full=Heat shock 70 kDa protein, mitochondrial; Flags: Precursor
☐ Check to include this hit in error tolerant search or archive report

	Query	Observed	Mr(expt)	Mr(calc)	Delta	Miss	Score	Expect	Rank	Peptide
☑	1355	532.84	1063.67	1063.53	0.14	0	51	0.005	1	R.VLENTEGFR.A

Proteins matching the same set of peptides:
gi|25553516 **Mass:** 71625 **Score:** 51 **Queries matched:** 1
mitochondrial HSP70 [Trypanosoma congolense]
gi|71396677 **Mass:** 20401 **Score:** 51 **Queries matched:** 1
heat shock 70 kDa protein, mitochondrial precursor [Trypanosoma cruzi strain CL Brener]
gi|71407515 **Mass:** 70946 **Score:** 51 **Queries matched:** 1
heat shock 70 kDa protein, mitochondrial precursor [Trypanosoma cruzi strain CL Brener]
gi|71654158 **Mass:** 7863 **Score:** 51 **Queries matched:** 1
70 kDa heat shock protein [Trypanosoma cruzi strain CL Brener]
gi|72390395 **Mass:** 71430 **Score:** 51 **Queries matched:** 1
heat shock 70 kDa protein, mitochondrial precursor [Trypanosoma brucei TREU927]

---

47. gi|71420500 **Mass:** 19088 **Score:** 50 **Queries matched:** 2
cytochrome C oxidase subunit VI [Trypanosoma cruzi strain CL Brener]
☐ Check to include this hit in error tolerant search or archive report

	Query	Observed	Mr(expt)	Mr(calc)	Delta	Miss	Score	Expect	Rank	Peptide
☑	1794	437.29	1308.86	1308.72	0.14	0	48	0.009	1	K.APILHDVFVNGK.K
☑	1949	523.03	1566.06	1565.86	0.20	1	23	2.7	1	R.EKAPILHDVFVNGK.K

---

48. gi|167375825 **Mass:** 24465 **Score:** 49 **Queries matched:** 1
hypothetical protein [Entamoeba dispar SAW760]
☐ Check to include this hit in error tolerant search or archive report

	Query	Observed	Mr(expt)	Mr(calc)	Delta	Miss	Score	Expect	Rank	Peptide
☑	1790	651.91	1301.80	1301.68	0.12	0	49	0.0058	1	K.DVTIDISIAEAR.I

---

49. gi|71651239 **Mass:** 48013 **Score:** 48 **Queries matched:** 2
thiolase protein-like protein [Trypanosoma cruzi strain CL Brener]
☐ Check to include this hit in error tolerant search or archive report

	Query	Observed	Mr(expt)	Mr(calc)	Delta	Miss	Score	Expect	Rank	Peptide
☑	1727	620.37	1238.72	1238.57	0.15	0	48	0.0067	1	K.DEYIQSDIEK.M
☑	1746	418.65	1252.92	1252.64	0.28	0	2	3.1e+02	1	R.ADQEALAIASHK.N

---

50. gi|123401786 **Score:** 46 **Queries matched:** 1
hypothetical protein [Trichomonas vaginalis G3]
☐ Check to include this hit in error tolerant search or archive report

Query	Observed	Mr(expt)	Mr(calc)	Delta	Miss	Score	Expect	Rank	Peptide
1589	580.36	1158.70	1158.61	0.08	0	46	0.013	2	K.TIEDLEAQIK.A

---

51. gi|225587 **Score:** 45 **Queries matched:** 2
tubulin alpha
☐ Check to include this hit in error tolerant search or archive report

Query	Observed	Mr(expt)	Mr(calc)	Delta	Miss	Score	Expect	Rank	Peptide
2035	858.56	1715.10	1714.91	0.19	0	45	0.014	2	K.AVFLDLEPTVIDEVR.T
2288	765.49	2293.46	2293.20	0.26	1	15	16	2	K.AVFLDLEPTVIDEVRTGTYR.Q

Proteins matching the same set of peptides:
gi|1729839 **Score:** 45 **Queries matched:** 2
gi|1755084 **Score:** 45 **Queries matched:** 2

---

52. gi|84105377 **Score:** 45 **Queries matched:** 2
alpha tubulin 2 [Rhynchopus sp. ATCC 50230]

☐ Check to include this hit in error tolerant search or archive report

Query	Observed	Mr(expt)	Mr(calc)	Delta	Miss	Score	Expect	Rank	Peptide
2035	858.56	1715.10	1714.88	0.22	0	45	0.014	2	R.AVMLDLEPTVIDEVR.T + Oxidation (M)
2288	765.49	2293.46	2293.16	0.30	1	15	16	2	R.AVMLDLEPTVIDEVRTGTYR.Q + Oxidation (M)

Proteins matching the same set of peptides:

gi|84105383 **Score:** 45 **Queries matched:** 2

---

53. gi|116222245 **Score:** 45 **Queries matched:** 2

alpha tubulin [Thaumatomonas sp. TMT002]

☐ Check to include this hit in error tolerant search or archive report

Query	Observed	Mr(expt)	Mr(calc)	Delta	Miss	Score	Expect	Rank	Peptide
2035	858.56	1715.10	1714.91	0.19	0	45	0.014	2	R.AVFIDLEPTVIDEVR.T
2288	765.49	2293.46	2293.20	0.26	1	15	16	2	R.AVFIDLEPTVIDEVRTGTYR.Q

Proteins matching the same set of peptides:

gi|116222249 **Score:** 45 **Queries matched:** 2

gi|116222289 **Score:** 45 **Queries matched:** 2

---

54. gi|71413646 **Mass:** 19139 **Score:** 45 **Queries matched:** 1

hypothetical protein [Trypanosoma cruzi strain CL Brener]

☐ Check to include this hit in error tolerant search or archive report

	Query	Observed	Mr(expt)	Mr(calc)	Delta	Miss	Score	Expect	Rank	Peptide
☑	1989	**550.00**	**1646.97**	**1646.75**	**0.22**	**0**	**45**	**0.016**	**1**	**R.RPEEAPEVNGYCAR.L + Carbamidomethyl (C)**

Proteins matching the same set of peptides:

gi|148746664 **Mass:** 19138 **Score:** 45 **Queries matched:** 1

putative surface antigen YASP-A5 [Trypanosoma cruzi]

gi|148746666 **Mass:** 19021 **Score:** 45 **Queries matched:** 1

putative surface antigen YASP-A7 [Trypanosoma cruzi]

gi|148746676 **Mass:** 18880 **Score:** 45 **Queries matched:** 1

putative surface antigen YASP-A6 [Trypanosoma cruzi]
gi|148746682 **Mass:** 18983 **Score:** 45 **Queries matched:** 1
putative surface antigen YASP-A8 [Trypanosoma cruzi]
gi|148746684 **Mass:** 19053 **Score:** 45 **Queries matched:** 1
putative surface antigen YASP-A9 [Trypanosoma cruzi]

---

55. gi|154420635 **Score:** 45 **Queries matched:** 1
DEAD/DEAH box helicase family protein [Trichomonas vaginalis G3]
☐ Check to include this hit in error tolerant search or archive report

Query	Observed	Mr(expt)	Mr(calc)	Delta	Miss	Score	Expect	Rank	Peptide
2077	909.05	1816.09	1815.92	0.17	2	45	0.016	2	K.RMKAMLDIEEEVPQK.M

---

56. gi|158577406 **Mass:** 36564 **Score:** 43 **Queries matched:** 3
glycosomal glyceraldehyde-3-phosphate dehydrogenase [Crithidia oncopelti]
☐ Check to include this hit in error tolerant search or archive report

Query	Observed	Mr(expt)	Mr(calc)	Delta	Miss	Score	Expect	Rank	Peptide
1835	692.90	1383.78	1383.77	0.01	0	42	0.03	1	R.AAAVNIIPSTTGAAK.A
1837	692.94	1383.87	1383.77	0.10	0	(40)	0.046	1	R.AAAVNIIPSTTGAAK.A
2262	747.89	2240.65	2240.06	0.59	0	18	12	2	K.GILGFTDEELVSSDFINDNR.S

---

57. gi|71413449 **Mass:** 31077 **Score:** 43 **Queries matched:** 1
RNA-binding protein [Trypanosoma cruzi strain CL Brener]
☐ Check to include this hit in error tolerant search or archive report

	Query	Observed	Mr(expt)	Mr(calc)	Delta	Miss	Score	Expect	Rank	Peptide
☑	1791	**653.90**	**1305.79**	**1305.63**	**0.16**	**0**	**43**	**0.029**	**1**	**R.ETFQQVGNVER.A**

Proteins matching the same set of peptides:
gi|71417144 **Mass:** 31047 **Score:** 43 **Queries matched:** 1
RNA-binding protein [Trypanosoma cruzi strain CL Brener]

Peptide matches not assigned to protein hits: (no details means no match)

	Query	Observed	Mr(expt)	Mr(calc)	Delta	Miss	Score	Expect	Rank	Peptide
☑	1787	433.33	1296.96	1296.79	0.17	2	41	0.043	1	**RKELELAVALR**
☑	474	718.11	2151.30	2151.13	0.16	0	40	0.21	1	**GFERPSPVQEEAIPVALQGK**
☑	1342	530.84	1059.67	1059.60	0.07	1	38	0.081	1	**DVIDIVKMK**
☑	1958	529.01	1584.01	1583.78	0.23	0	38	0.075	1	**NAPTAANPPHGAVPDR**
☑	2318	790.12	2367.33	2367.20	0.12	1	37	0.087	1	**KIQPSIPEDTTTGVGGLGNVDNR**
☑	1660	597.86	1193.71	1193.59	0.13	0	37	0.11	1	**VLAYNGCLER + Carbamidomethyl (C)**
☑	2032	572.04	1713.09	1712.92	0.17	1	36	0.12	1	**ILLFPEGNQEEIRR**
☑	1812	673.88	1345.76	1345.64	0.12	1	35	0.21	1	**EKLDAAGVETDAQ**
☑	2058	592.03	1773.08	1772.90	0.18	0	35	0.16	1	**VTFQFQVVGGGNHDIR**
☑	1945	521.33	1560.98	1560.75	0.23	0	35	0.15	1	**LPPGTQESDEPHVR**
☑	1158	496.85	991.68	991.56	0.12	1	34	0.27	1	**YSGVAALRR**
☑	2119	949.55	1897.09	1897.00	0.09	0	33	0.2	1	**STLPELSLPDTSGGIPVSK**
☑	230	586.25	1170.48	1170.49	-0.00	0	32	0.76	1	**DFDTVCTEGK + Carbamidomethyl (C)**
☑	1972	540.35	1618.03	1617.92	0.11	0	32	0.3	1	**LHEGKPEVIITGLGR**
☑	1316	526.32	1050.62	1050.57	0.05	0	31	0.4	1	**VVAFLYDPK**
☑	1264	1032.05	1031.04	1031.49	-0.45	0	30	1.2	1	**GDSDLQLER**
☑	252	599.33	1196.65	1196.53	0.12	0	28	1.8	1	**TGDGELSDFTR**
☑	1586	578.92	1155.83	1155.67	0.15	2	28	0.74	1	**KLEIDRINR**
☑	1995	831.99	1661.96	1661.94	0.02	0	28	0.82	1	VTIDTLLIFANLMAK
☑	716	834.18	833.17	833.39	-0.21	0	28	0.91	1	**QDAQSASK**
☑	1631	589.82	1177.63	1177.61	0.02	0	28	0.83	1	**DVINLFGWSK**
☑	2057	887.02	1772.04	1772.02	0.01	1	26	1.1	1	**LNLMLASTIKGLLQNK + Oxidation (M)**
☑	2117	947.01	1892.01	1891.93	0.07	2	26	1.3	1	**DFERRLLQSCDNALGR**
☑	88	490.33	1467.96	1467.75	0.21	0	26	2.8	1	**TPVSAFLHEPSQR**
☑	1884	738.43	1474.85	1474.72	0.13	0	26	1.4	1	**FIDCYLQFLEK + Carbamidomethyl (C)**
☑	315	632.00	1261.98	1261.64	0.33	2	25	6.3	1	**ARQVDAKAMEK + Oxidation (M)**
☑	1257	515.40	1028.78	1028.59	0.19	0	25	1.7	1	**VQVAGTEVVK**
☑	2254	1106.13	2210.24	2210.10	0.14	0	25	1.4	1	**YICDAGVLVEIQINDAYVR + Carbamidomethyl (C)**
☑	2114	941.98	1881.94	1882.03	-0.09	2	25	1.5	1	**TNKRTSLALPPSVGAADGK**
☑	1322	527.39	1052.76	1052.54	0.22	0	24	1.5	1	**LVIMSATMR + 2 Oxidation (M)**
☑	1447	549.87	1097.72	1097.64	0.08	2	24	1.7	1	AAALRRDAVR
☑	273	610.45	1218.88	1218.59	0.29	0	24	6.1	1	**LICGDVVSEER**
☑	234	588.53	587.52	587.34	0.18	0	24	8.8	1	**AITGAR**

☑	264	603.12	602.11	602.34	-0.23	0	24	9.5	1	NASLAK
☑	1096	487.30	972.59	972.60	-0.01	1	24	2.5	1	LELTLTRK
☑	2091	613.36	1837.05	1837.04	0.01	2	24	2	1	ISRGVNGPVVTARVWAR
☑	2331	806.05	2415.12	2415.03	0.09	0	24	1.8	1	AEEPCQHEIEQNYPDSEGVGK + Carbamidomethyl (C)
☑	169	548.35	1642.03	1642.05	-0.02	2	23	6.5	1	ITILPSFITILRKK
☑	1341	530.84	1059.66	1059.49	0.16	1	23	3	1	DGETPRETR
☑	2362	850.50	2548.47	2548.34	0.13	0	22	2.6	1	VSFGGVAPAAGGATAAPAAAAAAAPAAAAAAK
☑	637	799.90	798.89	798.47	0.42	1	22	4.6	1	ARLTNPK
☑	2306	784.50	2350.47	2350.19	0.28	1	22	3.3	1	ANELAEDNRLDILPGGSPNSLR
☑	900	456.36	910.71	910.41	0.29	0	22	3	1	MDLFEEK
☑	2033	572.22	1713.63	1713.99	-0.36	1	22	3.1	1	LIIFLMFELYLRK + Oxidation (M)
☑	1691	608.31	1214.60	1214.70	-0.10	0	22	3.8	1	AVPISFEILAR
☑	361	650.36	649.35	649.31	0.04	0	22	10	1	CASLEK
☑	35	430.72	859.42	859.51	-0.09	0	21	11	1	SISATLIR
☑	1934	772.46	1542.90	1542.81	0.09	1	21	3.4	1	SVGLDRLGALSNGER
☑	2244	725.06	2172.16	2171.96	0.20	0	21	3.3	1	ATANSGTSQPQQPSQEGETPAS
☑	1959	793.90	1585.79	1585.88	-0.10	1	21	4.2	1	ESGVPAAIKFITTPR
☑	1781	431.98	1292.91	1292.67	0.24	0	21	4.6	1	DPYAYLVPIDK
☑	2208	700.41	2098.20	2098.00	0.20	0	21	4.1	1	GLEHYGLDPTDAEVANEIR
☑	1830	690.42	1378.82	1378.65	0.18	0	20	5	1	EAQLAAYSAENGR
☑	1240	511.85	1021.69	1021.35	0.34	0	20	4.7	1	MAMMMTCR + 3 Oxidation (M)
☑	735	421.83	841.64	841.47	0.17	0	20	4.1	1	SLAADIPR
☑	71	473.00	1415.97	1415.75	0.22	2	20	18	1	GVDSHKDKVYIR
☑	1948	522.81	1565.42	1565.67	-0.25	2	19	8	1	ARREEEMASGCDR + Carbamidomethyl (C)
☑	2090	613.02	1836.04	1835.93	0.11	0	19	5.8	1	ICENFSQIIEEGLTLK
☑	1357	532.86	1063.71	1063.51	0.20	0	19	7.6	1	VVAMGDGPFR + Oxidation (M)
☑	1059	480.01	958.01	957.54	0.47	1	19	13	1	WEAKGIVR
☑	2001	835.56	1669.11	1669.01	0.10	2	19	6.2	1	CVREALKALVVTLVR
☑	115	511.99	1021.97	1022.50	-0.53	0	19	17	1	AFVDETVSR
☑	140	529.81	1586.41	1586.63	-0.21	1	19	21	1	MKCEGCCVGWWR + 2 Carbamidomethyl (C); Oxidation (M)
☑	473	717.90	716.90	716.37	0.53	0	19	17	1	AVVEDGK
☑	612	788.72	787.71	787.46	0.26	0	19	8.8	1	IVTVNSR
☑	1278	519.23	1036.44	1036.57	-0.13	0	19	6.2	1	ANIFGFLQK
☑	1526	564.38	1126.74	1126.60	0.14	1	19	6.2	1	SIDRVPSEPK

☑	152	534.09	1599.24	1599.79	-0.56	1	18	23	1	STSMPEDHIIKQAK + Oxidation (M)
☑	1833	691.36	1380.71	1380.70	0.02	0	18	6.5	1	GIQLYVDMLGEK + Oxidation (M)
☑	42	441.80	1322.38	1322.56	-0.18	0	18	15	1	CTTCASNYYLK + Carbamidomethyl (C)
☑	1776	643.91	1285.80	1285.67	0.13	1	18	7.7	1	RLGPSWATWGR
☑	1001	470.36	938.71	938.58	0.13	0	18	7.8	1	LVIPGELAK
☑	1829	689.43	1376.84	1376.72	0.12	1	18	8.3	1	EEIDALMTKISK
☑	1874	731.93	1461.84	1461.71	0.13	1	18	8.9	1	ADPVDAKDIAYER
☑	181	556.78	1111.54	1111.50	0.04	0	18	27	1	DYDLTLGCR + Carbamidomethyl (C)
☑	1301	523.35	1044.68	1044.57	0.11	2	17	10	1	NKAAATRADK
☑	560	764.18	763.18	763.42	-0.25	0	17	9.1	1	NNYIIK
☑	2427	957.95	2870.82	2870.44	0.38	0	17	12	1	ELPFGSTLSLYDVAGAPGVAADLSHIDR
☑	1410	543.35	1084.68	1084.57	0.11	1	17	9.5	1	CKVVIDSHK + Carbamidomethyl (C)
☑	1802	444.34	1330.00	1329.77	0.24	2	17	12	1	MSTIKIPVGEKK
☑	323	635.91	1269.81	1269.72	0.09	0	17	37	1	SVASRPIQSVAR
☑	1978	813.00	1623.99	1623.90	0.09	2	17	10	1	FGVERLVDTGYIKK
☑	2359	845.49	2533.46	2533.28	0.18	1	17	8.6	1	VESGELIPGMMVVFAPAGEKTEVK + Oxidation (M)
☑	179	555.03	1662.08	1661.70	0.38	0	17	31	1	CVCNPLYGGTYCQK + 2 Carbamidomethyl (C)
☑	237	591.30	1770.88	1770.79	0.08	1	16	29	1	DEMDALAAIGMTKMSR + 2 Oxidation (M)
☑	1136	494.58	987.15	987.56	-0.41	0	16	19	1	VISLVNESK
☑	1303	523.36	1044.70	1044.52	0.18	0	16	12	1	AELAAAQSER
☑	36	431.96	1292.85	1292.60	0.24	0	16	39	1	AAMTSSAAAEDLR
☑	1810	672.98	1343.95	1343.64	0.31	1	16	13	1	AAEAERAAAEAER
☑	337	643.39	642.38	642.27	0.11	0	16	12	1	EMYGK + Oxidation (M)
☑	1832	690.90	1379.79	1379.70	0.09	1	16	11	1	VDIDAYQTKTAR
☑	1411	543.35	1084.68	1084.48	0.20	0	16	12	1	MDHLCVDPR
☑	1440	548.83	1095.65	1095.57	0.08	0	16	10	1	GNVGPPGTELR
☑	164	545.88	544.87	544.30	0.57	0	16	16	1	GVVDR
☑	93	499.31	996.60	996.56	0.04	2	16	25	1	RQQRQGPK
☑	1650	594.89	1187.76	1187.60	0.15	2	16	14	1	KALDDWERR
☑	967	932.20	931.20	931.41	-0.21	0	16	53	1	GCLDYSFK
☑	1668	1200.04	1199.03	1198.70	0.33	1	15	45	1	GIDVAALLGSKR
☑	1775	429.50	1285.47	1285.67	-0.20	1	15	14	1	QAQSGGKNVEIR
☑	1786	648.89	1295.77	1295.72	0.05	1	15	13	1	QEIFYKLISR
☑	750	848.89	847.88	848.39	-0.51	0	15	64	1	SWWGASR

☑	1860	724.91	1447.82	1447.74	0.07	0	15	15	1	ISDIICLQMDLK + Carbamidomethyl (C)
☑	920	459.37	916.73	916.52	0.21	0	15	17	1	STDLLLQK
☑	94	499.71	1496.12	1495.66	0.46	1	15	33	1	WMKQCAWEVDK + Carbamidomethyl (C); Oxidation (M)
☑	737	421.94	841.86	841.49	0.37	1	15	19	1	RNANVLR
☑	1754	631.91	1261.80	1261.64	0.16	1	15	15	1	EVPSRSTLAMR + Oxidation (M)
☑	567	767.38	766.37	766.47	-0.10	0	15	40	1	NIAPKPK
☑	2211	701.59	2101.76	2101.22	0.54	0	15	15	1	LIGSVIYFIGFLIYSGVIK
☑	2086	611.63	1831.87	1831.85	0.02	0	15	16	1	EDERPTFPTPAMASGAR
☑	1271	1034.32	2066.63	2066.04	0.59	2	15	56	1	SSSDITPPRGASKTVSSSFR
☑	50	445.27	1332.79	1332.65	0.13	0	15	44	1	TTEQPQTATTAGK
☑	860	895.65	894.64	894.51	0.12	2	15	60	1	RKADLHR
☑	1345	531.06	1060.11	1059.60	0.51	2	15	33	1	KQVVSRTDK
☑	2264	749.73	2246.18	2246.19	-0.01	1	15	16	1	ENVILNQSIKASNMSLTQIK + Oxidation (M)
☑	311	630.04	1887.09	1886.90	0.19	0	15	74	1	IYDDDGNLKPGETTPPR
☑	81	481.83	961.65	961.56	0.08	2	15	37	1	MIKDKISK
☑	2088	612.40	1834.18	1833.95	0.22	1	14	18	1	AINDQIGSMKSESTLLK
☑	39	435.25	1302.74	1302.66	0.08	0	14	32	1	GDVDFASGLVPAR
☑	399	674.10	1346.18	1346.71	-0.53	2	14	81	1	FVERGPGSGASKR
☑	418	684.10	1366.18	1366.68	-0.51	0	14	68	1	THDIDAVVVGADR
☑	57	456.19	1365.56	1365.78	-0.22	1	14	36	1	STLGGTVHALVRR
☑	1869	727.88	1453.75	1453.60	0.15	0	14	19	1	CMPGYTEGDVGPR + Carbamidomethyl (C); Oxidation (M)
☑	1498	559.86	1117.70	1117.60	0.10	0	14	20	1	HPGGASPLVQR
☑	44	443.08	1326.22	1325.73	0.49	1	14	51	1	ILSILMFMKCK
☑	1233	1019.41	3055.20	3054.77	0.43	0	14	62	1	WAVVLIIVSSIICLAVVGLVLACIVFER + Carbamidomethyl (C)
☑	1926	763.97	1525.92	1525.76	0.16	1	14	18	1	VQRNAVTPNDEQR
☑	810	874.09	2619.24	2619.49	-0.26	2	14	80	1	TRDLLLANAIVRVILQMVQFYK + Oxidation (M)
☑	2125	639.03	1914.06	1913.95	0.11	2	14	18	1	LRNMHFNKDRPIDDK + Oxidation (M)
☑	442	699.41	698.40	698.40	0.00	0	14	13	1	ATVPSPK
☑	917	916.84	915.83	916.34	-0.51	0	14	66	1	DGGACYMGK + Oxidation (M)
☑	349	646.72	1937.14	1937.07	0.07	1	14	78	1	QRPLEELERLSGLLER
☑	2281	756.83	2267.47	2267.95	-0.48	0	14	25	1	VHACNNCAGLGNIMCTFCQGR + Carbamidomethyl (C)
☑	945	464.35	926.68	926.49	0.19	0	14	17	1	MPVLGDPAK
☑	76	480.31	958.61	958.47	0.14	0	14	44	1	TVPPVMCR + Carbamidomethyl (C)
☑	1967	537.87	1610.57	1610.79	-0.21	1	14	23	1	HNTPGGGLRSGNSTTR

☑	74	476.36	1426.04	1425.75	0.29	0	13	53	1	ISTVISSVSFMQK
☑	1745	627.44	1252.87	1252.72	0.16	1	13	25	1	AARPGLFHRTK
☑	2222	708.40	2122.18	2122.01	0.16	0	13	21	1	QPWFPAGSTGNEDHIPLEK
☑	1647	396.70	1187.08	1186.77	0.32	1	13	48	1	IISIYPKILK
☑	1883	738.04	1474.06	1473.87	0.19	2	13	26	1	QVLQQSFQIKKK
☑	1841	703.90	1405.79	1405.68	0.11	2	13	28	1	CRCVEELKQNK + Carbamidomethyl (C)
☑	106	508.15	1521.42	1521.75	-0.33	0	13	60	1	MAGLTSDNISIASAR + Oxidation (M)
☑	2329	805.14	2412.41	2412.16	0.25	2	13	21	1	ADGDIAAAATGTAGSGRSCASPPPRR
☑	2397	904.58	2710.73	2710.45	0.28	1	13	30	1	SSEALLPTVAVRAPSSVPDFIIDAAGK
☑	2405	921.46	2761.36	2761.26	0.10	2	13	19	1	MYEYTVCGNPCFISFLEKMKGTK + Carbamidomethyl (C); Oxidation (M)
☑	210	573.28	1144.55	1144.57	-0.02	0	13	75	1	QISANLLSEGN
☑	1066	962.03	2883.07	2883.59	-0.52	2	13	1.1e+02	1	ASGTERIGPIQTISTIELTDLLIMRR
☑	1692	608.31	1214.61	1214.72	-0.11	2	13	29	1	AKNQIAMLKAK
☑	2008	559.68	1676.02	1675.86	0.15	1	13	26	1	VMVLLKEAQDMIDR + Oxidation (M)
☑	721	836.65	835.64	835.49	0.15	0	13	1e+02	1	LLTQHPK
☑	1828	688.95	1375.89	1375.64	0.25	0	13	28	1	LVDMENATAQER
☑	822	877.63	2629.87	2629.32	0.54	1	13	97	1	MQGIDVRIAETLMPGLAEVAWDSK
☑	184	557.92	1113.83	1113.60	0.23	0	13	77	1	CRPLILDER
☑	194	564.95	1127.89	1127.59	0.29	0	13	72	1	VDTGAPNIVSR
☑	2066	897.52	1793.03	1792.91	0.12	1	13	25	1	DFSFFTTAQMITIKK + Oxidation (M)
☑	2055	589.66	1765.96	1766.01	-0.04	1	13	25	1	EVVLSIKHVPQNLYK
☑	2063	597.09	1788.26	1787.87	0.38	1	13	31	1	EKPKEDTVAMPGDSLR + Oxidation (M)
☑	245	597.07	1788.17	1788.01	0.16	2	13	89	1	LEINKLNKDLAYLNK
☑	616	791.09	1580.16	1579.76	0.40	1	12	96	1	IMQQGQRQSDPHR
☑	2287	761.80	2282.38	2282.26	0.12	1	12	24	1	SLLKAISITECYDFVLNLLK
☑	1941	518.35	1552.03	1551.88	0.15	0	12	26	1	LDLVHSVLAPVFSR
☑	277	612.47	1834.39	1834.98	-0.59	0	12	88	1	HNLFVPLMDPVIDAVR
☑	1262	516.33	1030.64	1030.61	0.04	0	12	35	1	VIDLLAPYK
☑	1782	647.49	1292.96	1292.71	0.25	0	12	32	1	IIVVQPNPDGNK
☑	1052	478.93	955.85	956.41	-0.57	1	12	30	1	KCTICDMK + Oxidation (M)
☑	845	888.79	2663.34	2663.18	0.16	0	12	99	1	NVSPVVTMDDEHDDIMLPAHGEDK
☑	2243	725.04	2172.11	2172.01	0.10	2	12	26	1	RSGHGEGALSAAREECSVGSR + Carbamidomethyl (C)
☑	68	471.01	1410.01	1409.79	0.22	0	12	74	1	EKPVDELLLAQR
☑	718	417.93	833.84	833.42	0.42	1	12	50	1	IGKGNCSR

☑	1936	516.65	1546.93	1546.79	0.14	1	12	33	1	ENITLNMWGAVKR + Oxidation (M)
☑	563	765.97	764.96	765.44	-0.48	0	12	46	1	FYVIPK
☑	2000	835.53	1669.05	1669.01	0.04	2	12	30	1	CVREALKALVVTLVR
☑	2018	846.14	1690.28	1689.88	0.39	1	12	47	1	YTILSHIPFNSDRK
☑	1081	967.94	966.93	967.45	-0.52	0	12	1.1e+02	1	TGLCAFGSR + Carbamidomethyl (C)
☑	249	598.97	1195.92	1195.57	0.35	0	12	74	1	QDYVETLSNK
☑	2155	988.75	1975.49	1976.04	-0.55	2	12	49	1	CGIVLCSKCAATKTVIPR + 2 Carbamidomethyl (C)
☑	53	451.79	901.57	901.52	0.04	0	12	71	1	GTLTIELR
☑	1231	509.84	1017.66	1017.62	0.04	2	12	38	1	KEIKQLFI
☑	1917	759.14	1516.27	1515.80	0.46	1	12	64	1	CAKLFFEHGVPLR
☑	297	623.50	1245.00	1244.68	0.32	0	12	1.2e+02	1	MNIQIEQLLK + Oxidation (M)
☑	1866	485.04	1452.10	1451.66	0.44	2	12	43	1	MKTFQMDDKHR + Oxidation (M)
☑	432	694.71	2081.11	2080.78	0.32	1	12	1e+02	1	RWGDSDDDGDAVGSDDEDR
☑	981	935.11	934.10	934.50	-0.40	1	12	1.2e+02	1	RAVFDATR
☑	1771	640.41	1278.81	1278.66	0.15	0	12	35	1	IVLSTEMMIDK
☑	541	749.92	748.91	749.44	-0.53	1	12	77	1	TKFGGLK
☑	1198	503.29	1004.56	1004.53	0.03	1	12	37	1	LDVDKIMR + Oxidation (M)
☑	620	793.02	792.02	792.41	-0.40	0	12	59	1	GYVEGLR
☑	1856	719.88	1437.75	1437.88	-0.13	2	12	32	1	EIIIANRKINVR
☑	705	829.74	828.73	828.48	0.25	1	12	44	1	GDVALARK
☑	2092	614.69	1841.05	1840.96	0.08	0	11	34	1	LHDGSLSLSAIAGTTAATR
☑	428	691.35	2071.03	2071.06	-0.04	2	11	1.1e+02	1	ALWATVPEAEEQLMRRR + Oxidation (M)
☑	1922	762.31	1522.61	1522.75	-0.14	1	11	36	1	CESEITIKQMIR + Carbamidomethyl (C); Oxidation (M)
☑	1847	709.35	1416.68	1416.71	-0.03	0	11	42	1	ESIVEATADLNQK
☑	2139	968.14	1934.26	1934.05	0.21	0	11	36	1	DPTLVAPSWVPVLENLGK
☑	127	522.01	1563.01	1562.81	0.21	0	11	1.1e+02	1	QAITNNTLAQSQFK
☑	2156	661.78	1982.32	1982.04	0.28	2	11	45	1	QIDHCTDKQIVTQLRK + Carbamidomethyl (C)
☑	182	557.41	1669.21	1668.76	0.45	1	11	99	1	SNSDGTVKEGQTATMK + Oxidation (M)
☑	269	608.88	1823.61	1823.84	-0.23	1	11	1.3e+02	1	FNGGFGQDEKAAAAAADGK
☑	1713	616.32	1230.63	1230.68	-0.05	0	11	43	1	FTELWIPAVR
☑	1479	1113.05	1112.04	1111.58	0.46	0	11	68	1	MSLLVHGSPR + Oxidation (M)
☑	811	874.15	2619.43	2619.34	0.09	2	11	1.5e+02	1	MKDPASWWTTQYTLVVDKIPPK + Oxidation (M)
☑	216	576.40	1150.80	1150.45	0.35	0	11	1.2e+02	1	YTEESYCEK
☑	464	714.41	2140.22	2140.11	0.11	2	11	1.1e+02	1	SVKQHLFNPTETAGSAAARR

☑	1704	1225.59	3673.74	3673.78	-0.04	1	11	92	1	NAIISHNIDFVTFLVNEYDLEIDLEMCGRFK + Oxidation (M)
☑	578	773.95	772.95	773.46	-0.52	1	11	1.1e+02	1	EAVLKSK
☑	1767	1278.43	3832.27	3831.89	0.37	2	11	1.3e+02	1	EEAILLLREHRNDPEMEALANEEIGALDEVIER + Oxidation (M)
☑	2349	832.15	2493.42	2493.13	0.29	1	11	38	1	LYTVFQYFREDYMMSETVR + Oxidation (M)
☑	1703	1221.97	2441.93	2442.11	-0.18	1	11	1.2e+02	1	CGEFVRCANLAGYPECIGEIK + 3 Carbamidomethyl (C)
☑	2296	771.46	2311.35	2311.04	0.31	2	11	41	1	TCILTFKSCWSCMKNGFR + 3 Carbamidomethyl (C); Oxidation (M)
☑	1533	1131.77	2261.53	2262.06	-0.53	2	11	1.4e+02	1	GWGMIVVMPPEESADRGCKK + Carbamidomethyl (C); Oxidation (M)
☑	2187	685.38	2053.10	2053.17	-0.07	1	11	43	1	LRPFLQSGRLVHFLSQR
☑	2239	722.41	2164.20	2163.96	0.24	1	10	38	1	VNASGIDSATASEDDRESGQR
☑	208	571.80	1712.39	1712.84	-0.44	0	10	1.5e+02	1	QLWDVLEVPSEDQR
☑	2054	883.94	1765.87	1765.88	-0.01	2	10	42	1	ARMSLEAKMSTEGVTR
☑	2454	999.11	2994.30	2994.57	-0.27	1	10	37	1	NMSHVSTGGGASLELLEGKTLPGVAILTDK
☑	2015	562.07	1683.19	1682.93	0.26	1	10	48	1	EVRPLTRLVEESQK
☑	1085	968.53	1935.05	1934.86	0.19	0	10	1.2e+02	1	MMSNFAQHPDLTLNER + 2 Oxidation (M)
☑	1005	940.70	939.69	939.55	0.14	1	10	36	1	QERLLPGK
☑	833	881.91	1761.81	1761.88	-0.07	1	10	1.8e+02	1	ELMARVVEMNELQGK + Oxidation (M)
☑	2434	968.86	2903.54	2903.47	0.07	2	10	34	1	AFASAANACHEAHQPQRSRASVVVQLR
☑	2406	924.73	2771.17	2771.34	-0.18	2	10	39	1	ASGSRPAMASGSNEAKMAALLSSSRYR + Oxidation (M)
☑	262	601.59	1201.16	1200.63	0.52	1	10	1.9e+02	1	VIDTAKSDPQK
☑	1857	481.65	1441.94	1441.79	0.14	2	10	46	1	RETSKFSFLLSK
☑	2278	755.45	2263.32	2263.04	0.28	2	10	46	1	MYAYSFSMWRTTTTTTKR + 2 Oxidation (M)
☑	988	468.93	935.85	935.45	0.40	0	10	59	1	MASSAVQSR
☑	1778	646.17	1290.33	1290.70	-0.37	1	10	57	1	MKMLGAEVVAVK + Oxidation (M)
☑	684	412.82	823.64	823.54	0.10	1	10	35	1	GLRLIRP
☑	2040	575.14	1722.39	1721.94	0.45	2	10	80	1	EGRPANTRPIRGKDR
☑	1358	1064.89	2127.77	2128.10	-0.33	1	10	1.7e+02	1	LDYERLMSLITSYQQLR
☑	2116	630.08	1887.20	1887.80	-0.59	1	10	58	1	EYTGAKANSQDDEMCVK
☑	303	625.89	624.89	625.32	-0.43	0	10	1.3e+02	1	DHAVGK
☑	23	407.77	1220.28	1220.48	-0.20	0	10	1.1e+02	1	AMGMYVACMR + Carbamidomethyl (C); 2 Oxidation (M)
☑	1919	506.73	1517.16	1516.83	0.32	1	10	70	1	ISDLQTKITEIEK
☑	1665	599.79	1197.57	1197.51	0.06	0	10	47	1	DCGTGADLSFR + Carbamidomethyl (C)
☑	24	418.31	1251.92	1251.69	0.23	1	10	1e+02	1	VRDLPADPITR
☑	767	856.34	1710.66	1710.91	-0.25	2	10	1.3e+02	1	LWRNEALRVFHDR
☑	1045	955.20	1908.38	1908.87	-0.49	1	10	1.6e+02	1	NEALMQQYSKFESYR + Oxidation (M)

2079	607.35	1819.04	1818.76	0.28	0	10	51	1	HMNLWETDTDASNNR + Oxidation (M)
64	464.66	1390.96	1390.64	0.31	1	10	1.1e+02	1	GGSERAMDYLHR
1103	976.21	1950.41	1950.94	-0.54	2	10	1.9e+02	1	VKGSGTNRSGQGAVGNFCR + Carbamidomethyl (C)
190	561.28	1680.82	1680.83	-0.01	1	10	1.5e+02	1	CCIRSLALEEELFR
1935	515.68	1544.01	1543.88	0.13	2	10	62	1	ITNLQMLLMRRR
2132	642.38	1924.12	1923.93	0.19	1	10	49	1	DYLKSTALHNAAYENSK
784	863.35	862.34	862.47	-0.13	1	10	61	1	IVCTKSR + Carbamidomethyl (C)
162	544.10	1086.19	1085.62	0.57	0	10	2.1e+02	1	VITALAANGTR
2126	640.04	1917.10	1916.97	0.14	1	10	48	1	DNIDLMSLSSHKIYGPK
1135	988.09	2961.24	2961.42	-0.19	0	10	2.1e+02	1	MDIVIYLIVLIVCGMVFTGCYGCYK + 2 Carbamidomethyl (C); 2 Oxidation (M)
25	420.66	1258.97	1258.69	0.28	2	10	1.1e+02	1	RWGVREVTTR
203	569.48	1136.94	1137.51	-0.57	0	10	1.8e+02	1	SNSDGTTSEIK
391	669.30	668.29	668.42	-0.13	0	10	31	1	ALPAAVK
1875	733.41	1464.80	1464.71	0.08	0	9	74	1	LASNINTSEEMIK + Oxidation (M)
1317	1051.86	1050.85	1050.60	0.26	1	9	1.6e+02	1	FRLLFNNK
407	679.39	2035.16	2034.92	0.24	1	9	1.9e+02	1	MVDGASATRHDSSVGTCLGR + Oxidation (M)
472	717.90	716.89	717.38	-0.48	1	9	1.5e+02	1	ARGETGK
373	655.62	1963.84	1963.92	-0.07	0	9	1.8e+02	1	AAEMLLESDQLSGASEAAR + Oxidation (M)
1844	1412.95	2823.90	2824.37	-0.47	1	9	1.5e+02	1	RGGGNHLPFQTSVAVPAGVDVGEGNMSR + Oxidation (M)
950	929.38	2785.13	2785.27	-0.14	2	9	1.7e+02	1	SPTCSATAWQWTRCCCIHTKFLSR
863	896.00	894.99	895.43	-0.44	0	9	2.2e+02	1	IDGSFTEK
465	714.43	1426.84	1426.69	0.15	0	9	1.6e+02	1	ATVNDSICPHVSK + Carbamidomethyl (C)
321	633.92	632.91	632.32	0.59	1	9	2.2e+02	1	DKGGTR
893	453.87	905.72	905.40	0.31	0	9	62	1	AWNDWSK
1905	502.34	1504.01	1503.81	0.20	2	9	57	1	DLLNEKKSLMASR
1890	494.23	1479.66	1479.71	-0.04	0	9	64	1	MVVNDTVADMLTR + Oxidation (M)
760	427.79	853.56	853.42	0.14	0	9	47	1	ISASSFDK
295	623.32	1244.63	1244.70	-0.07	1	9	1.7e+02	1	VTSIEKAAEVAK
1913	505.05	1512.11	1511.66	0.45	0	9	72	1	GALSLMGSCSQEEK + Carbamidomethyl (C); Oxidation (M)
2017	563.80	1688.39	1687.80	0.59	1	9	1.1e+02	1	MHRAADAAAVHTHER + Oxidation (M)
2270	751.77	2252.28	2252.26	0.02	2	9	57	1	QRRLVPEPLITELLTAMEK + Oxidation (M)
2095	616.51	1846.51	1847.04	-0.53	2	9	1e+02	1	LDVVKYLISVGADKEAK
1131	494.35	986.69	986.48	0.21	1	9	70	1	TPRNNEEK
1962	800.92	1599.83	1599.77	0.05	0	9	60	1	NEDLQAAAAQLDDVK

☑	300	625.19	1872.55	1872.85	-0.30	0	9	1.8e+02	1	CTICHQEPTKPVVCCR + Carbamidomethyl (C)
☑	1435	1095.71	1094.70	1094.60	0.10	0	9	2.1e+02	1	AQEIAPEPLK
☑	1799	1320.46	1319.45	1319.70	-0.25	0	9	1.9e+02	1	ISELLTEMIQK + Oxidation (M)
☑	1889	494.06	1479.16	1479.69	-0.53	0	9	1.1e+02	1	MQTDDILQMVMR
☑	2472	1055.33	3162.98	3162.42	0.56	2	9	67	1	SACAEPCHNHSAAAEPPQHPRGSPARAEER
☑	846	888.92	2663.73	2663.38	0.36	1	9	3e+02	1	IQGLQAELLETEEAHEQEISKLR
☑	2142	648.78	1943.32	1942.94	0.38	1	9	81	1	ARQIAHEGVTTLESGDYP
☑	420	685.72	2054.13	2054.12	0.01	2	9	1.7e+02	1	VQKKHTVSDELVACVSALK
☑	1979	813.02	1624.04	1623.83	0.21	2	9	62	1	SHYSKETLKFEQK
☑	1969	538.72	1613.13	1612.86	0.27	1	9	89	1	LASDAAMAAVPDKVVR
☑	166	546.52	1091.02	1091.53	-0.50	1	9	2.4e+02	1	VQEKMAENK + Oxidation (M)
☑	212	573.53	1145.04	1144.69	0.36	1	9	2.7e+02	1	FILKLASEPK
☑	317	632.08	1262.14	1262.65	-0.51	0	9	3.1e+02	1	GQVVNRPGHDGK
☑	1260	516.10	1030.19	1030.49	-0.29	0	9	96	1	MNQALDPAR + Oxidation (M)
☑	2189	1028.82	2055.63	2055.16	0.47	2	9	98	1	ISESPVKPLSAMTIVRRR + Oxidation (M)
☑	532	744.43	1486.85	1486.87	-0.02	0	9	2.2e+02	1	LTNRPAFLMAILK
☑	2298	776.13	2325.38	2325.29	0.09	1	9	60	1	TCVGVSLKTHILYLSIYLFR
☑	430	693.80	692.79	692.29	0.50	0	8	1.3e+02	1	CSAQER
☑	1318	526.88	1051.75	1051.48	0.27	1	8	69	1	NYMEDPKR
☑	855	893.30	892.30	892.48	-0.19	0	8	68	1	NIFVCAVK
☑	2335	810.48	2428.41	2428.33	0.08	2	8	62	1	VRPMVTDTTLLVHRYLKENK + Oxidation (M)
☑	360	650.31	1947.91	1948.05	-0.14	1	8	2.1e+02	1	GVSLIKINDDGTISEFLK
☑	2375	873.13	2616.36	2616.14	0.22	0	8	55	1	LYGCGSSIDSQETCYSLTGVGYEK + Carbamidomethyl (C)
☑	375	656.59	655.59	655.33	0.26	1	8	41	1	DKEHK
☑	580	774.11	773.10	773.41	-0.31	1	8	93	1	DGVARTR
☑	682	821.96	2462.86	2463.26	-0.41	2	8	3e+02	1	DYLYDIVVEKGENLPKADIGGR
☑	2081	608.03	1821.07	1820.84	0.23	2	8	74	1	MGEWREKHVFDCLR + Oxidation (M)
☑	2158	664.86	1991.56	1991.80	-0.24	0	8	1e+02	1	NSCTACSAETCYNTPSVK + 2 Carbamidomethyl (C)
☑	2041	862.47	1722.92	1722.75	0.17	0	8	64	1	LFADEVEDCGGGVADAR
☑	142	531.00	1589.96	1589.70	0.26	0	8	2.4e+02	1	MYSQGGSAASGAPGGHR
☑	1796	1312.83	3935.48	3935.07	0.41	2	8	2.1e+02	1	MCVNVTDSGIQMLLNKCTKLLNIVLTGTSVSQQVIR + Oxidation (M)
☑	1582	1154.33	1153.32	1153.58	-0.26	1	8	2.2e+02	1	CKLTDFGVSK + Carbamidomethyl (C)
☑	680	821.02	2460.05	2460.17	-0.12	2	8	2.6e+02	1	RLGRGDADACPVDVAQAIDDFEK
☑	223	582.26	1743.77	1743.98	-0.21	2	8	1.5e+02	1	LRVRLDDTTSLTLNK

☑	1466	1105.91	1104.91	1104.62	0.29	1	8	2.1e+02	1	LTWGGRLFR
☑	1715	617.02	1232.03	1231.69	0.34	2	8	1.2e+02	1	KRETSETIIR
☑	799	871.83	870.82	870.44	0.39	1	8	2.2e+02	1	EAMKQHK
☑	66	467.34	1399.00	1398.68	0.31	0	8	1.6e+02	1	FASSISVCVSSQK + Carbamidomethyl (C)
☑	1543	1137.42	3409.25	3408.82	0.42	1	8	2.2e+02	1	FLKYWFMSLLTSQINVTMGALIGYIIINK + 2 Oxidation (M)
☑	1608	1169.22	2336.42	2336.14	0.28	2	8	2.1e+02	1	REHLQEIHALMEELERCK + Carbamidomethyl (C); Oxidation (M)
☑	1344	530.97	1059.93	1060.53	-0.60	0	8	1.1e+02	1	SAGVAPFNNGK
☑	160	543.42	1627.25	1627.69	-0.44	1	8	2.2e+02	1	CIQGGRCMLSDNMK + Carbamidomethyl (C); Oxidation (M)
☑	422	686.10	685.09	685.43	-0.34	2	8	75	1	KGRGLR
☑	1577	576.94	1151.87	1151.51	0.36	0	8	80	1	EEEASEVGFR
☑	79	480.68	1439.01	1438.73	0.28	1	8	1.7e+02	1	EASTAAAAREGLHR
☑	1481	1114.69	3341.05	3340.71	0.34	1	8	2.3e+02	1	NVVLSASSLLVTNVKAHASSYCGPGFYSTGALK
☑	484	722.38	721.37	721.33	0.04	0	8	64	1	HDSHAR
☑	427	689.26	1376.51	1376.64	-0.13	0	8	2.6e+02	1	MAGAGGDGGLEASLR + Oxidation (M)
☑	2209	700.42	2098.23	2098.15	0.08	2	8	76	1	EPIGEKFMSGFFVLIKIK + Oxidation (M)
☑	2368	860.74	2579.20	2579.32	-0.13	2	8	65	1	EFIVIRCMGDNFPASLLKDQVK + Carbamidomethyl (C)
☑	2343	823.86	2468.54	2468.99	-0.45	2	8	79	1	GCDSEGNGEMEGEEEEKVDNKK + Carbamidomethyl (C)
☑	1687	607.34	1212.67	1212.53	0.14	1	8	77	1	EMGEKFTEDK
☑	1361	1065.17	3192.50	3192.82	-0.32	2	8	2.7e+02	1	LQDSLVRSVVSTIVSLIPVSRHLAESIFK
☑	643	804.23	2409.67	2410.19	-0.51	1	8	3.4e+02	1	SQHNWMFAEIGVNVGHEALKK + Oxidation (M)
☑	1961	800.03	1598.05	1598.56	-0.51	0	8	90	1	YNGCCCEELGYCSR
☑	2334	1212.03	2422.05	2422.05	0.00	1	8	65	1	DTDPIEYNKGNMCYSPYVEK + Carbamidomethyl (C)
☑	1816	1354.62	4060.85	4061.05	-0.20	2	8	1.8e+02	1	HGPALVLDGVRLLSTCFVMTRSTLVCGGCSCAAILVER + 2 Carbamidomethyl (C)
☑	1779	1292.02	2582.03	2582.49	-0.46	2	8	2.2e+02	1	LREMLLWAMVLLSIAPIIKNMK
☑	284	615.58	1843.72	1843.75	-0.03	0	8	3.3e+02	1	GHCEPAEEAEAAADMTR + Carbamidomethyl (C)
☑	389	667.54	1333.06	1332.70	0.37	1	8	3.1e+02	1	ELMNSKEIEIK
☑	922	919.50	918.49	918.48	0.01	0	8	93	1	LMISTNPK + Oxidation (M)
☑	63	464.06	1389.16	1389.75	-0.59	2	8	2.5e+02	1	ESEVRSLDSIKK
☑	562	765.83	1529.65	1529.73	-0.08	2	8	3.1e+02	1	MRCFSTKSTAPTK + Carbamidomethyl (C); Oxidation (M)
☑	1822	1361.92	4082.74	4083.10	-0.37	0	8	2.5e+02	1	QSQTLALPTISLATAAAAEQTVPVTATKPMSSQSSADVPAK + Oxidation (M)
☑	1709	1228.41	1227.40	1227.59	-0.19	0	8	2.8e+02	1	SVFAGSSMSTVR
☑	1404	542.34	1082.66	1082.59	0.06	2	8	71	1	LAPGEERRR
☑	2325	798.55	2392.62	2392.27	0.36	1	8	1.2e+02	1	FKPALPRAIQGASYHLHTEEK
☑	1873	487.68	1460.02	1459.79	0.23	0	8	88	1	ILSVSGNSNILSEK

☑	1414	543.58	1085.15	1085.53	-0.37	1	8	1.4e+02	1	RGDNPWVDK
☑	927	461.29	920.57	920.44	0.13	0	8	85	1	EGIVNMSR + Oxidation (M)
☑	2067	598.79	1793.34	1792.93	0.41	2	8	1.4e+02	1	TQSALWSFHAKKDFK
☑	92	497.54	1489.60	1489.74	-0.14	1	8	2.4e+02	1	RFESPELNEELK
☑	1510	1124.52	3370.53	3370.56	-0.03	2	8	2.2e+02	1	LGNREGRYMYDGVLQCYLDLETNAYVDK + Carbamidomethyl (C); Oxidation (M)
☑	62	464.02	926.02	925.46	0.56	0	8	2.3e+02	1	NGGDVHSLK
☑	1386	539.51	1077.00	1077.52	-0.53	1	8	1.8e+02	1	CKHQNVGHR
☑	2396	902.84	2705.48	2705.32	0.16	1	8	70	1	ARPTTPSRQSAPSQAQDAAAADGGAAPR
☑	1418	544.09	1086.17	1086.52	-0.35	0	7	1.5e+02	1	MMLSVYATR + Oxidation (M)
☑	1434	548.31	1094.61	1094.47	0.14	0	7	1e+02	1	STSCPAGLECK
☑	20	387.91	1160.71	1160.66	0.04	0	7	2.5e+02	1	ATFCLPIIGVK
☑	2089	919.00	1835.99	1835.80	0.19	0	7	86	1	CGSSCGTRPSGPAGGTGATGR
☑	2216	704.40	2110.17	2110.06	0.10	2	7	78	1	AGDAKSAATLLKDVVDMYDK
☑	2332	807.54	2419.59	2419.08	0.51	2	7	1e+02	1	LCGSSSFCFANVCGGRKETPSGR + Carbamidomethyl (C)
☑	1701	611.28	1220.56	1220.72	-0.17	2	7	93	1	RGHSVLLRQR
☑	785	863.36	1724.71	1724.97	-0.26	2	7	2.7e+02	1	GRTVAPISPCASLLKR + Carbamidomethyl (C)
☑	1793	1309.83	3926.46	3926.69	-0.23	2	7	2.7e+02	1	EVFQAVKTCMGCYCMGSDTCQILERIFCDYELR + Carbamidomethyl (C); Oxidation ([illegible]
☑	319	632.88	631.87	631.29	0.58	0	7	2.7e+02	1	VDENR
☑	125	518.80	1553.39	1553.88	-0.50	2	7	2.7e+02	1	AIGRAGARLVAACTPK
☑	2161	668.19	2001.54	2001.98	-0.44	0	7	1.4e+02	1	NQQLSMIHIACMLNNIK + 2 Oxidation (M)
☑	2026	567.36	1699.05	1698.90	0.15	1	7	85	1	TPILHTKEIGMTTNK + Oxidation (M)
☑	58	456.52	911.04	911.63	-0.59	2	7	2.1e+02	1	LKLAKAIR
☑	2437	969.87	2906.59	2906.43	0.16	2	7	77	1	GPACRLNESVRPLTHYTKAYSMPSR + Carbamidomethyl (C); Oxidation (M)
☑	984	936.24	1870.46	1869.89	0.58	2	7	3.3e+02	1	RTTNHQGQSDPNTMKR
☑	265	603.55	1205.08	1205.65	-0.57	2	7	4e+02	1	VRGIRCAFER
☑	228	585.39	1753.16	1752.95	0.21	1	7	2.1e+02	1	KLGGTQLSEGPLQQAAR
☑	1906	753.09	1504.16	1503.72	0.44	2	7	1.3e+02	1	KNENTEEQEKQK
☑	1908	754.40	1506.78	1506.69	0.09	0	7	1.2e+02	1	SPSWPGQGSFCAVK + Carbamidomethyl (C)
☑	324	635.93	1904.76	1904.86	-0.10	0	7	3.2e+02	1	CQEQLQDCINVLEEK + 2 Carbamidomethyl (C)
☑	85	486.31	970.61	970.54	0.07	2	7	1.8e+02	1	EVKNEPKK
☑	207	571.40	1140.79	1140.65	0.15	2	7	2.4e+02	1	NLTARSPARR
☑	1061	960.24	1918.46	1917.99	0.46	1	7	3.6e+02	1	RAISAAQEEFGVIDGSIR
☑	1893	743.45	1484.88	1484.76	0.12	1	7	91	1	GAPPKQDTSSTLQR
☑	677	819.76	2456.27	2456.25	0.02	1	7	3.1e+02	1	LAPHDFDAALLEIVHKYYADR

☑	271	609.71	608.71	608.29	0.41	1	7	2.9e+02	1	AKYNN
☑	40	438.65	1312.92	1312.78	0.14	1	7	2.1e+02	1	SVSAAAKVVAAIAR
☑	2002	557.55	1669.64	1669.88	-0.24	1	7	93	1	ISKEAEKPTDFHLR
☑	1815	1353.21	2704.41	2704.33	0.08	2	7	3.2e+02	1	KRPSDAGWDWGPAFGTMGLWRSVK
☑	1992	553.58	1657.71	1657.84	-0.13	0	7	1e+02	1	CVQSICVLIFGCVFK
☑	2230	1070.55	2139.09	2139.02	0.07	1	7	98	1	SLYDPHYGSTVCLACLNKR
☑	2305	783.46	2347.35	2347.21	0.14	2	7	82	1	STHGSMKSVAAQLKSDWVFIR
☑	1818	678.89	1355.77	1355.64	0.13	2	7	97	1	MPHEDHKKYR + Oxidation (M)
☑	22	396.96	1187.85	1187.44	0.41	0	7	3.4e+02	1	MCMCVGVCGR + 2 Carbamidomethyl (C); Oxidation (M)
☑	1605	1167.69	3500.06	3499.85	0.22	2	7	2.8e+02	1	QTVRVQFTPTVPHCSMTTLIGLCISLKLQR + 2 Carbamidomethyl (C); Oxidation (M)
☑	281	613.77	1838.29	1838.88	-0.59	0	7	2.7e+02	1	SVEGSTVSATASHSGPHTK
☑	1090	971.57	1941.13	1940.99	0.14	1	7	2.7e+02	1	YSRLVMSPSPLHGGSTPR
☑	989	936.88	2807.62	2807.36	0.26	0	7	3.2e+02	1	SCLRPHLGPLATGGMSLMNSAPNSPQR + Oxidation (M)
☑	1774	643.07	1284.12	1284.68	-0.56	1	7	1.8e+02	1	LVEERGDVAAAR
☑	1848	709.41	1416.80	1416.73	0.08	0	7	1.2e+02	1	MLQLENNDIAIK + Oxidation (M)
☑	348	646.72	1291.42	1291.66	-0.24	2	7	3.8e+02	1	MKLFHAMSRR + Oxidation (M)
☑	2275	752.59	2254.74	2255.11	-0.37	2	7	1.3e+02	1	LYEQVLKDMPTNDFCVKR + Carbamidomethyl (C)
☑	639	801.39	1600.77	1600.80	-0.03	1	7	3.1e+02	1	MAVCSRLATLSTLY + Carbamidomethyl (C); Oxidation (M)
☑	267	605.84	604.83	604.32	0.51	1	7	2e+02	1	EKGGSK
☑	699	828.36	2482.07	2482.09	-0.02	0	7	2.9e+02	1	TTHWQLPAEYSMQPPMCSYR + Carbamidomethyl (C)
☑	1572	1151.44	3451.31	3450.89	0.42	2	7	3e+02	1	GVQAAPGLEAIELTAARATSVVNRILTSITSWR
☑	1314	1050.19	1049.18	1049.50	-0.32	1	7	3.1e+02	1	CFERDVPK + Carbamidomethyl (C)
☑	222	581.72	1742.15	1741.88	0.27	0	7	2.7e+02	1	EVMAAVTWQTPPLQR + Oxidation (M)
☑	1825	682.45	1362.88	1362.78	0.11	1	7	1.1e+02	1	RWYGITLISVR
☑	52	448.40	894.78	894.37	0.41	0	7	3e+02	1	CVAEMGGGR + Oxidation (M)
☑	2285	761.54	2281.61	2282.09	-0.48	1	7	1.8e+02	1	SLQWQGMGVCGVPFMIEKEK + Oxidation (M)
☑	869	898.41	2692.20	2692.27	-0.07	1	7	2.8e+02	1	YSDMLNAANSHADLVIAESAAMRAR + Oxidation (M)
☑	2124	638.14	1911.40	1910.89	0.51	1	7	1.8e+02	1	GKAIGCCTESILNISDCK + Carbamidomethyl (C)
☑	1547	570.00	1137.98	1138.47	-0.50	0	7	1.5e+02	1	MEAGTQAEMR + Oxidation (M)
☑	1938	517.59	1549.74	1549.82	-0.08	0	7	1.1e+02	1	YTLTVAGEEVELVK
☑	2356	841.54	2521.61	2522.20	-0.59	1	7	1.2e+02	1	NLENLTSADKSYLFFFDEQNK
☑	347	646.62	1291.22	1291.71	-0.50	1	7	4.7e+02	1	EIYVLTKGDVR
☑	938	463.30	924.59	924.35	0.24	0	7	88	1	EMHSMMK + 2 Oxidation (M)
☑	1747	1254.79	1253.78	1253.71	0.07	1	7	3.2e+02	1	NLPTSLTPKQR

☑	586	775.81	774.80	774.46	0.34	1	7	1.9e+02	1	TKATQVK
☑	1894	496.42	1486.24	1486.77	-0.53	1	7	1.9e+02	1	DTIPMTEQPKIAK + Oxidation (M)
☑	2196	1033.91	2065.82	2066.10	-0.28	1	7	1e+02	1	LMTDEEKAVGAVSLLTYVK
☑	253	599.48	1196.94	1196.57	0.37	0	6	3.4e+02	1	TEYNIDAIMK
☑	1628	588.29	1174.57	1174.56	0.01	0	6	1.3e+02	1	SEGSEQAAGALR
☑	2112	627.09	1878.26	1877.86	0.40	0	6	1.2e+02	1	STAISLYYELAEEDMK + Oxidation (M)
☑	1877	733.89	1465.77	1465.77	0.00	1	6	1.1e+02	1	KEEEILNLYSTK
☑	426	688.29	687.29	687.30	-0.02	0	6	1.3e+02	1	NTCHLT
☑	1529	1129.46	1128.45	1128.56	-0.11	1	6	3.1e+02	1	KLMNYFADK
☑	110	510.32	1018.62	1018.59	0.03	1	6	2.7e+02	1	NLNVKYIR
☑	2388	892.75	2675.22	2675.30	-0.08	0	6	92	1	FTAYVFQSGLVQCNAFHVSAMALR + Oxidation (M)
☑	1393	1079.34	3234.99	3234.41	0.58	2	6	3.7e+02	1	ENTEGENSMCAKCESMSATIASLTKEIER + Carbamidomethyl (C); Oxidation (M)
☑	1470	555.06	1108.10	1108.58	-0.48	0	6	1.9e+02	1	LGSQTEFVTK
☑	1215	1013.66	1012.66	1012.58	0.08	1	6	3.5e+02	1	GSIGLPASRR
☑	854	893.28	892.28	892.52	-0.25	2	6	4e+02	1	VREHPKK
☑	292	622.57	621.56	621.32	0.24	0	6	95	1	ASAAFR
☑	296	623.47	1867.40	1866.90	0.49	2	6	4.2e+02	1	KIDEFARNNYIPCER
☑	2268	751.48	2251.42	2251.12	0.30	2	6	1.3e+02	1	MSGLEAKLQGMLASACQRSVR + Oxidation (M)
☑	879	451.99	901.97	902.56	-0.59	1	6	2.7e+02	1	EIRLIIF
☑	1496	1118.36	3352.04	3351.59	0.45	2	6	3.9e+02	1	LMYYPETDHGPEEFVAIDSPFRCKLPPK + Carbamidomethyl (C); Oxidation (M)
☑	1008	942.71	2825.11	2825.39	-0.29	2	6	3.8e+02	1	ISKSPTNDSFVSPSEFKLDPNLMTR + Oxidation (M)
☑	239	591.93	1772.78	1772.96	-0.18	2	6	3.4e+02	1	TSALEAAKEKTVQLER
☑	590	390.75	779.50	779.38	0.11	1	6	99	1	GKADDFK
☑	1658	1192.27	1191.26	1191.61	-0.35	1	6	3.5e+02	1	SNKSLNTAETK
☑	1218	1014.15	2026.29	2026.75	-0.45	0	6	3.7e+02	1	YGSMSAAQDGHAMACPHMM + 2 Oxidation (M)
☑	505	732.22	1462.43	1461.85	0.58	1	6	4.8e+02	1	KGHGLLCLPAGVIK + Carbamidomethyl (C)
☑	1885	738.88	1475.75	1475.70	0.04	1	6	1.2e+02	1	NSTEPFTYARYK
☑	2181	683.31	2046.90	2047.01	-0.11	1	6	1.1e+02	1	VTDDSLLFFMNNQFKTK
☑	302	625.54	1873.60	1873.86	-0.26	0	6	4.1e+02	1	GLPIDVYHTANNEEMR + Oxidation (M)
☑	1595	581.87	1161.73	1161.56	0.17	0	6	1.2e+02	1	EFNIGGNFHK
☑	1943	778.95	1555.88	1555.71	0.16	0	6	1.5e+02	1	ESGTAHAMLFYWK + Oxidation (M)
☑	161	543.62	1627.85	1627.75	0.09	2	6	3.1e+02	1	GMTKVMVDDSRTEK + 2 Oxidation (M)
☑	436	696.03	695.02	695.35	-0.33	0	6	90	1	EYVASK
☑	2174	677.39	2029.15	2028.99	0.17	1	6	1.2e+02	1	MSSHSKMEHFSKPDLLR

☑	446	701.58	2101.72	2102.08	-0.36	1	6	5e+02	1	AKLEQLQPCADYLQAVANK
☑	1823	681.69	1361.36	1361.65	-0.29	1	6	1.5e+02	1	EPKEIEDMSLR + Oxidation (M)
☑	912	916.13	1830.25	1829.88	0.36	0	6	4.9e+02	1	AQLEICNAIEELDGGAK + Carbamidomethyl (C)
☑	1675	1204.96	3611.85	3611.38	0.46	2	6	3.4e+02	1	SRMAAPGCLDEGCMGCADCVKCDACEGCADVLR + 4 Carbamidomethyl (C); 2 Oxidatio
☑	936	462.90	923.79	923.51	0.28	0	6	1.1e+02	1	AVEVLHEK
☑	2246	1090.13	2178.24	2177.96	0.28	1	6	1.1e+02	1	RNDSTAMANPLDAEAGMWGR + Oxidation (M)
☑	60	459.18	1374.52	1374.87	-0.35	1	6	2.9e+02	1	LTATILRHPLLK
☑	831	881.48	880.48	880.44	0.04	0	6	3.3e+02	1	DIMFIDK
☑	962	931.61	930.60	930.50	0.10	0	6	4.1e+02	1	ITSSADLPK
☑	61	463.92	1388.74	1388.65	0.08	2	6	2.4e+02	1	NEHRRSSSSSSR
☑	2024	848.93	1695.84	1695.81	0.03	0	6	1.2e+02	1	DAIADLTYIMNNNTK
☑	103	503.79	1005.57	1005.66	-0.09	2	6	3.8e+02	1	YLTKKIIK
☑	1882	737.66	1473.31	1473.74	-0.43	2	6	2.2e+02	1	YRGRNSTAEAPPR
☑	450	705.30	2112.87	2112.99	-0.12	0	6	4e+02	1	TFTDCFNCLPVAALIDEK + 2 Carbamidomethyl (C)
☑	1332	1057.49	3169.44	3169.66	-0.22	1	6	3.3e+02	1	STLSAVAPSSPSGATSATDTPLSPFLPRSVLR
☑	1406	1084.96	1083.95	1084.52	-0.57	0	6	1.8e+02	1	SLVWHAGCR + Carbamidomethyl (C)
☑	2085	611.61	1831.81	1831.85	-0.05	0	6	1.4e+02	1	EDERPTFPTPAMASGAR
☑	1951	786.73	1571.45	1571.72	-0.27	2	6	1.5e+02	1	SRCSEFTKAVCER + Carbamidomethyl (C)
☑	1382	1076.66	3226.95	3226.68	0.27	1	6	4.1e+02	1	TVLECPHIRNVLFSLNSTNTVPVCSSLVK + Carbamidomethyl (C)
☑	158	540.58	1079.15	1079.47	-0.33	0	6	3e+02	1	MQNGELMNK + Oxidation (M)
☑	147	532.43	1062.85	1062.48	0.36	0	6	4.2e+02	1	ENMMGQIPK + Oxidation (M)
☑	1809	671.60	1341.18	1340.66	0.52	0	6	2.3e+02	1	NVSSIGSSAFSASK
☑	691	825.42	1648.83	1648.87	-0.03	0	6	3.4e+02	1	QTLAVVTEQLDAYAK
☑	2282	758.57	2272.68	2273.16	-0.48	1	6	1.8e+02	1	KTSSEIFSYLLDACQALISK + Carbamidomethyl (C)
☑	998	469.99	937.97	937.53	0.44	0	6	2.4e+02	1	ILTIGHER
☑	2193	688.11	2061.30	2061.00	0.31	1	6	1.3e+02	1	STSRLLVHIMDMCYQHK
☑	95	500.32	1497.93	1497.79	0.14	0	6	2.5e+02	1	QMVPSILIDVPDR + Oxidation (M)
☑	288	618.11	1234.21	1233.70	0.51	2	6	5.9e+02	1	LLGCRLEFKR
☑	1928	765.25	1528.49	1528.75	-0.25	0	6	1.4e+02	1	FLGEGHVTLPCGSR + Carbamidomethyl (C)
☑	884	903.86	1805.70	1805.94	-0.24	2	6	4.4e+02	1	KKSNCVTLTSNNTLQR
☑	2198	691.83	2072.46	2073.06	-0.60	1	6	2e+02	1	LTDMATIDASVSRLFYVR + Oxidation (M)
☑	261	601.50	1200.98	1200.64	0.34	1	6	5.1e+02	1	NGKTAFHTAVR
☑	1216	507.38	1012.75	1012.59	0.16	2	6	1.1e+02	1	AADGVIRRR
☑	700	828.39	1654.77	1654.87	-0.09	2	6	3.7e+02	1	FQEPRQPSLRAGGGR

☑	724	839.29	2514.86	2514.32	0.54	2	6	4.1e+02	1	MLYKTFPLGDVGHGNGGGKIGVAAR
☑	902	913.14	2736.40	2736.14	0.27	1	6	4.1e+02	1	KGEGNAEMVEGGSDTEESPTHWMAR + 2 Oxidation (M)
☑	931	922.98	2765.92	2766.51	-0.59	0	6	5.4e+02	1	THQVMLLFSAISAQDGVAVVPLTTLR
☑	1108	977.51	976.51	976.55	-0.04	2	6	3.6e+02	1	NDIKKGFR
☑	2038	574.51	1720.50	1720.77	-0.27	1	6	1.8e+02	1	QGNEAHRFYSPCQK + Carbamidomethyl (C)
☑	2240	1083.14	2164.26	2164.12	0.13	2	6	1.2e+02	1	LQDENKLLHEQLADQKSR
☑	2348	829.83	2486.47	2487.05	-0.58	2	6	1.1e+02	1	NMMCAADPRHGRYPTASCMFR + Carbamidomethyl (C); Oxidation (M)
☑	607	787.21	1572.40	1571.86	0.55	1	6	5.6e+02	1	SYLYLSTIIERSK
☑	2103	619.61	1855.81	1855.90	-0.09	2	6	1.2e+02	1	KKYCLIFDNVCPDNK + Carbamidomethyl (C)
☑	2009	559.74	1676.19	1675.93	0.26	2	6	1.6e+02	1	VTQKGANVTKFAIGDK
☑	2304	783.25	2346.73	2346.19	0.54	1	6	2.2e+02	1	LECVVAREMLLTSVEEELQR
☑	1276	1036.51	3106.51	3106.62	-0.10	2	6	3.7e+02	1	KKIQAVFSGVTMGLLSGMRPDGSSPVTSPK + 2 Oxidation (M)
☑	704	829.49	1656.96	1656.83	0.13	1	6	4.3e+02	1	RPWMDGAGRINVNR + Oxidation (M)
☑	340	643.86	642.85	642.42	0.44	1	6	3e+02	1	AAVRVK
☑	533	745.08	1488.14	1488.74	-0.60	0	6	5e+02	1	QESLLLSGDPSSTR
☑	423	686.98	2057.92	2057.96	-0.04	0	5	5.2e+02	1	GHNTNVVAACAPMLPCYAAR
☑	596	782.55	2344.63	2344.14	0.50	0	5	4.5e+02	1	NIVQDLETSGMSVPEYLANHK
☑	231	587.95	1173.89	1173.57	0.32	1	5	5.2e+02	1	QQTDNVGEKR
☑	1149	495.93	989.85	989.49	0.37	0	5	1.9e+02	1	NAPTSLMEK
☑	2293	770.54	2308.60	2308.91	-0.31	1	5	2e+02	1	APVAANPSGGCCDNGGCKCGPSCK + 2 Carbamidomethyl (C)
☑	1515	1125.28	2248.55	2249.10	-0.55	2	5	4.3e+02	1	TNEPKSVTMGRTTIYSSSFK + Oxidation (M)
☑	2213	702.59	2104.75	2105.17	-0.42	2	5	1.4e+02	1	RANHGVTTSGKLELKPELR
☑	528	742.48	2224.43	2224.83	-0.40	2	5	4.4e+02	1	CPKCDNGCYEGCTCMQKR + 3 Carbamidomethyl (C); Oxidation (M)
☑	897	908.42	2722.24	2722.43	-0.19	2	5	3.8e+02	1	KSFLNQEIWSSICGRNNIMIIIK + Oxidation (M)
☑	1485	1115.24	1114.23	1114.54	-0.30	0	5	4.4e+02	1	AQGPDGGLSASR
☑	1557	573.08	1144.15	1144.59	-0.43	1	5	2.8e+02	1	QPDPKELYR
☑	1705	409.65	1225.92	1225.69	0.23	1	5	1.3e+02	1	LEVNAAAGRVAR
☑	1897	746.64	1491.27	1491.72	-0.45	2	5	3e+02	1	NACTGRNISSERK + Carbamidomethyl (C)
☑	2390	895.05	2682.14	2682.58	-0.43	2	5	1.2e+02	1	TVNGRRILVGLIWLVGMFTLSLPK
☑	331	639.10	1276.19	1275.68	0.51	1	5	5.7e+02	1	SFIAAQWAARR
☑	965	931.90	930.89	930.59	0.30	1	5	2.5e+02	1	KLMSVLLK
☑	2464	1040.55	3118.64	3118.57	0.07	2	5	1.1e+02	1	DYNIKFDNVYNTLYELNNLDRTLLR
☑	1261	1031.52	3091.53	3091.53	-0.00	2	5	4.1e+02	1	TGSSYNGQVKDDNVLQLSSYIFMLKER
☑	170	550.05	1647.13	1646.84	0.29	2	5	4.5e+02	1	LQQERSVACGATKEK

☑	1864	726.75	1451.49	1451.77	-0.28	2	5	1.5e+02	1	HTPLSEEKVREK
☑	755	851.63	2551.87	2552.17	-0.31	2	5	5.1e+02	1	TCRVCASLEEQTSQLNSREER + 2 Carbamidomethyl (C)
☑	2093	921.82	1841.63	1841.96	-0.33	1	5	1.5e+02	1	TKGTSAPAARPSASASSPAK
☑	666	814.87	2441.59	2441.15	0.44	1	5	5.4e+02	1	GMLENNIMSSDVSLEELGKMTK + Oxidation (M)
☑	768	857.27	2568.79	2569.01	-0.22	0	5	5.4e+02	1	CLGIEDLEFDEESGEMYCVNCR + Oxidation (M)
☑	867	897.27	896.26	896.45	-0.18	0	5	5.1e+02	1	LMMTTIR + 2 Oxidation (M)
☑	2393	897.49	2689.44	2689.42	0.02	2	5	1.2e+02	1	KWIMILSSIASLGVSTVMYFKGDK + Oxidation (M)
☑	421	685.86	684.85	684.45	0.40	0	5	6.3e+02	1	ILAAAVK
☑	1324	1055.04	2108.06	2107.98	0.08	1	5	5.6e+02	1	SDLQRQSEDLVQTMAESR + Oxidation (M)
☑	1955	789.92	1577.83	1577.77	0.06	0	5	1.5e+02	1	TTNAFDLFGEAPAPK
☑	2361	850.15	2547.43	2547.31	0.12	2	5	1.4e+02	1	WYVSRVQAARSSPPSISVEEATK
☑	415	683.31	1364.61	1364.55	0.06	0	5	3.5e+02	1	MQGEDDAVAEER + Oxidation (M)
☑	1580	1153.54	2305.07	2305.05	0.02	0	5	3.7e+02	1	DASVDIQGDDAVDSTAAAALAACR
☑	2138	645.10	1932.27	1932.83	-0.57	1	5	1.4e+02	1	VCYGDCTGATPFETEGKR
☑	2064	895.52	1789.03	1788.85	0.18	0	5	1.7e+02	1	TVTAGAQSMNFTFVGSR + Oxidation (M)
☑	572	770.84	769.83	769.49	0.35	0	5	1.7e+02	1	KPLVWK
☑	1062	960.38	959.37	959.53	-0.16	0	5	1.9e+02	1	NISGELSIK
☑	1575	1152.14	3453.39	3452.84	0.54	2	5	5.3e+02	1	VAPLGCLEKDNTDAVRLELAALPELSHPGVLR + Carbamidomethyl (C)
☑	1960	530.28	1587.82	1587.74	0.09	0	5	1.4e+02	1	FEFMTGSAVTTNQR
☑	703	829.46	1656.90	1656.76	0.13	1	5	4.7e+02	1	GGNKPANAQQECKQQ + Carbamidomethyl (C)
☑	383	661.31	660.30	660.32	-0.02	0	5	2.5e+02	1	NPSTSR
☑	1250	513.70	1025.38	1025.54	-0.17	0	5	1.2e+02	1	LCHVLGAASR
☑	199	567.18	1698.51	1698.85	-0.34	1	5	4e+02	1	ATKWDNFTHDVLPR
☑	78	480.61	1438.80	1438.49	0.31	0	5	3.3e+02	1	CDSTCNECEHK + 3 Carbamidomethyl (C)
☑	1903	751.52	1501.03	1500.81	0.21	0	5	1.6e+02	1	TLQVTETVGLSPTR
☑	780	860.53	859.52	859.48	0.04	1	5	1.8e+02	1	MAILLKDG
☑	1200	1006.40	3016.18	3016.64	-0.46	1	5	4.3e+02	1	SLTTFQTLSSLIQQKSINHLILTSESK
☑	1352	1062.74	3185.21	3185.51	-0.30	1	5	5.3e+02	1	TTMHMNSPQFRQSFCFVADTSAPGIVAVK + Oxidation (M)
☑	1494	1117.87	3350.60	3350.75	-0.15	2	5	4.6e+02	1	VNFIKSFWPGLTAMIIFYMLICGLRDFR
☑	1800	1327.18	3978.53	3979.12	-0.59	1	5	5.1e+02	1	LLGVESSDGEALVKPLVAGDILMINIGSTHTGCKVMALK
☑	2072	904.11	1806.21	1805.97	0.24	1	5	2e+02	1	GMENYLAVAKPAAVKTK + Oxidation (M)
☑	1080	967.72	2900.14	2900.31	-0.17	1	5	5.3e+02	1	VYDCTDGAAFYGPGNSYAVFAGKEVSR + Carbamidomethyl (C)
☑	625	793.81	1585.61	1585.90	-0.29	0	5	5.5e+02	1	WAFILVAGQEALLR
☑	1610	1169.73	1168.72	1168.57	0.15	0	5	4.4e+02	1	IHNEDAETIK

☑	537	747.60	2239.78	2240.09	-0.31	2	5	6.3e+02	1	NGTGVCASALTKHTRQQQHF + Carbamidomethyl (C)
☑	1840	693.93	1385.85	1385.73	0.13	2	5	1.6e+02	1	EAEPESATLRRK
☑	765	855.44	2563.29	2563.35	-0.07	2	5	4.3e+02	1	EELVGCTNPAKAPPTSLRGLIQNR
☑	1036	951.89	950.89	950.40	0.49	0	5	5.7e+02	1	DEMSGADVK
☑	1067	481.54	961.07	960.48	0.59	0	5	3.2e+02	1	IVMAGAGGGGR + Oxidation (M)
☑	1107	977.18	1952.36	1951.94	0.41	2	5	5e+02	1	AKDDGLSEEGAIRCIYGR
☑	118	513.82	1538.44	1537.88	0.56	2	5	3.2e+02	1	IISKEPCIIRNPR
☑	1765	1275.36	2548.70	2549.28	-0.59	2	5	4.8e+02	1	IANDFVNALKGMAGETARLSACAAR
☑	1690	1214.90	2427.78	2427.37	0.40	2	5	4.5e+02	1	VKLIDSPGIVFSSKSDPASLVLR
☑	2047	873.61	1745.21	1744.79	0.43	0	5	1.8e+02	1	QADHTTAATVEGSENSK
☑	1394	1079.58	1078.58	1078.57	0.00	1	5	1.4e+02	1	SSSSLSSLRR
☑	1272	518.33	1034.64	1034.52	0.12	1	5	1.6e+02	1	MTNRIGESK
☑	876	901.00	899.99	900.36	-0.37	0	5	6.5e+02	1	MSTEDFR + Oxidation (M)
☑	1323	1054.71	3161.10	3161.37	-0.27	0	5	5.5e+02	1	FVENNDIEFCSCIVSDNSIQNVNDSSR + 2 Carbamidomethyl (C)
☑	2108	936.80	1871.58	1871.93	-0.35	1	5	1.9e+02	1	RTSNPLAAVMMVQPGER + Oxidation (M)
☑	2353	837.41	2509.21	2508.96	0.25	2	5	1.7e+02	1	RCCCVLEMTEEEDMRGCSR + 4 Carbamidomethyl (C); 2 Oxidation (M)
☑	148	532.76	531.76	531.25	0.50	0	5	1.6e+02	1	DVEGL
☑	1402	1082.83	3245.48	3245.46	0.02	1	5	4.8e+02	1	EPSYFSMDCAPQVSIHIMCTEALRSSVR + Carbamidomethyl (C); 2 Oxidation (M)
☑	1469	1108.61	1107.61	1107.55	0.05	2	5	4.1e+02	1	TTADDSRSKK
☑	1618	586.05	1170.08	1170.55	-0.47	2	5	2.9e+02	1	KVDGCSFCKK + Carbamidomethyl (C)
☑	1756	633.58	1265.15	1265.51	-0.37	0	5	3.1e+02	1	CGCDGAGGADALTR
☑	727	840.00	2516.99	2517.17	-0.18	2	5	5.3e+02	1	WMLQVTDTMKSDHSAPTERER
☑	2283	758.79	2273.36	2273.26	0.10	2	5	1.3e+02	1	KIYMEKPHFILQQKITEK
☑	2363	853.30	2556.87	2557.31	-0.44	2	5	2e+02	1	SLERGKQQETWNEACLGLNLLR
☑	1288	1041.55	2081.08	2081.15	-0.07	1	5	4e+02	1	MRGSLPLLFNPVLPPSTAR + Oxidation (M)
☑	1843	706.25	1410.50	1410.78	-0.28	2	5	1.6e+02	1	VTAAEKVKHCLR + Carbamidomethyl (C)
☑	204	570.10	1707.27	1707.80	-0.53	0	5	5e+02	1	TVSVCFSDRPEQQGR
☑	382	661.02	660.01	660.34	-0.33	0	5	7.1e+02	1	AETVNK
☑	1741	1250.21	3747.61	3747.46	0.15	1	5	5.2e+02	1	CVDGYFLNSDLMCESCDTSCSTCITDSKTCLSCK + Carbamidomethyl (C); Oxidation (
☑	581	774.45	1546.89	1546.78	0.12	2	5	5.3e+02	1	NNTNDKFKIYYK
☑	2290	765.86	2294.56	2295.12	-0.56	1	5	2.4e+02	1	MRVSEVNVEVPITDSCIFNK + Oxidation (M)
☑	250	599.28	1794.82	1794.89	-0.07	1	5	4e+02	1	KSGGYLVILCDDATQR + Carbamidomethyl (C)
☑	287	618.08	1234.15	1233.68	0.47	2	5	7.4e+02	1	ERAKYVAELR
☑	730	840.83	839.82	839.49	0.33	0	5	1.8e+02	1	LAVDPLGR

☑	1700	1221.36	2440.70	2441.27	-0.57	2	5	5.1e+02	1	LNQTMDAEVAQQQKIVDNIRK
☑	102	503.54	1507.59	1507.64	-0.06	0	5	5.4e+02	1	DVCDAEWLCIER + Carbamidomethyl (C)
☑	440	698.71	2093.10	2092.98	0.12	0	5	4.2e+02	1	QMLIAFYCLGPVNCSFSK + Carbamidomethyl (C); Oxidation (M)
☑	457	708.78	1415.54	1415.75	-0.21	0	5	5.4e+02	1	LANVGTYAPAAAAAR
☑	2326	803.85	2408.52	2408.02	0.50	0	5	1.7e+02	1	VTFSESFYEFDLDASYMSDR
☑	511	734.45	2200.33	2200.02	0.31	1	5	5e+02	1	VTMMRGASSAASAATAAIQCMR + Oxidation (M)
☑	558	763.41	762.41	762.39	0.01	0	5	1.9e+02	1	SPPSTFK
☑	540	749.08	1496.14	1495.67	0.47	1	5	6.5e+02	1	GTADAEDEFVRMR
☑	1051	478.91	955.81	955.43	0.38	0	5	1.6e+02	1	DMEYAAIK + Oxidation (M)
☑	2065	597.83	1790.47	1790.96	-0.49	1	5	3.2e+02	1	ETPLITAFRGMLDTVK
☑	1643	1184.71	3551.10	3550.98	0.12	2	5	4.6e+02	1	NVKILQAWMKPVRPGIYRPFILVGPEGCGKK + Carbamidomethyl (C)
☑	1764	637.92	1273.84	1273.70	0.14	2	5	1.9e+02	1	QQISSAEKQKK
☑	2377	876.94	2627.81	2628.30	-0.49	2	5	2.3e+02	1	TVDRFIYHASESMYADPIRITK + Oxidation (M)
☑	1274	1035.77	3104.28	3104.67	-0.39	2	5	5.8e+02	1	LENCPAIKRLELANVEVLFSSLAYVTR + Carbamidomethyl (C)
☑	1954	788.81	1575.60	1575.73	-0.13	1	5	1.8e+02	1	NMSRSCIPNCIPK + 2 Carbamidomethyl (C)
☑	744	846.61	2536.81	2536.27	0.54	1	5	7e+02	1	MSEQPAPKAPPLEPIIPGCTPYK + Carbamidomethyl (C); Oxidation (M)
☑	1433	1094.36	2186.71	2186.26	0.44	2	5	5.7e+02	1	AIIPDRDPVAAIGINGGIRIR
☑	1438	1096.28	3285.81	3285.54	0.28	2	5	5.3e+02	1	DPSADTDCSAGKITAGLVGFLSGNFSDNKWR + Carbamidomethyl (C)
☑	1947	522.67	1564.98	1564.76	0.22	0	5	1.8e+02	1	YIPSSSIQQSCQPK
☑	534	745.57	744.56	744.42	0.14	1	5	2.3e+02	1	KLQSNR
☑	1574	1151.86	1150.85	1150.52	0.33	2	5	5.5e+02	1	CKEEEEEKK
☑	1689	607.93	1213.84	1213.57	0.27	1	5	1.6e+02	1	TAHTAADDEKR
☑	178	554.64	1107.26	1107.54	-0.28	2	5	3.5e+02	1	RRACSTVCR + Carbamidomethyl (C)
☑	970	932.96	931.95	931.45	0.50	0	5	3.8e+02	1	VSCLGSPNR
☑	2286	1141.87	2281.72	2282.18	-0.47	2	5	2.3e+02	1	QSAGGPPSLSASAAVASSRAARQR
☑	339	643.48	642.47	642.38	0.09	1	5	1.8e+02	1	SKVPGR
☑	1075	482.98	963.95	963.49	0.45	0	5	3.4e+02	1	DIITDMIK + Oxidation (M)
☑	1296	1044.55	1043.55	1043.49	0.06	0	5	4.8e+02	1	NSFCVYVR + Carbamidomethyl (C)
☑	89	491.58	1471.72	1471.78	-0.06	1	4	4e+02	1	NGNLSISGMKIPNK
☑	353	647.48	1292.95	1293.53	-0.57	1	4	6.8e+02	1	ENECSNKNGDK + Carbamidomethyl (C)
☑	463	710.57	709.56	709.42	0.14	0	4	1.3e+02	1	VVISHR
☑	904	913.33	1824.65	1824.92	-0.27	2	4	5e+02	1	TLSEFMTWIREREK
☑	1089	486.20	970.40	970.50	-0.11	1	4	1.6e+02	1	KTMDHALR
☑	1669	1200.46	3598.35	3598.91	-0.56	1	4	5.2e+02	1	DSPALRLILEVDTPLDCVVLQSELHLHFLEK + Carbamidomethyl (C)

☑	1719	618.66	1235.30	1235.57	-0.27	0	4	1.9e+02	1	DLHDIICHDR
☑	1000	939.71	2816.10	2816.32	-0.22	2	4	6.3e+02	1	SLADTNKADEAGDQGGGSGDSSPLAVGKGR
☑	2056	591.39	1771.14	1770.90	0.24	2	4	1.6e+02	1	KCLPGEPSVGFPPASKSA
☑	1273	1035.66	3103.96	3103.74	0.22	2	4	5.5e+02	1	ILYVCVPTVCIKQVRINPHLPISSLNK + Carbamidomethyl (C)
☑	406	678.80	677.79	677.34	0.45	0	4	8.6e+02	1	TPFADK
☑	1556	1144.91	2287.80	2288.14	-0.33	1	4	5.3e+02	1	YTTTFNDLTNLKVENMQLK + Oxidation (M)
☑	1863	725.90	1449.80	1449.72	0.07	1	4	1.7e+02	1	LEQQSRGESAFAK
☑	257	600.07	1198.13	1197.74	0.39	1	4	6.3e+02	1	LSKEGVALLIR
☑	1109	978.22	977.21	977.50	-0.29	0	4	6.1e+02	1	ISICGNIMK
☑	1141	989.61	988.61	988.51	0.09	0	4	5.7e+02	1	VLGGGGIMGGR + Oxidation (M)
☑	1731	1242.73	3725.17	3725.10	0.06	2	4	4.6e+02	1	LLSFLVLQTIVWICSIVILAGYPRDQPLRGWR
☑	1437	1095.87	2189.72	2190.02	-0.30	1	4	5.6e+02	1	FGSPQLTHGRGVACMTWNR + Carbamidomethyl (C); Oxidation (M)
☑	1939	776.00	1549.98	1549.83	0.15	2	4	1.8e+02	1	SSEYAFHIKKAAAK
☑	2438	970.85	2909.52	2909.25	0.27	0	4	1.4e+02	1	MCSCCCEPPVGQWIDIYGGPVRPR + 4 Carbamidomethyl (C); Oxidation (M)
☑	1359	1064.97	2127.92	2128.20	-0.28	1	4	6.9e+02	1	NPLIFVGSSVSLKTNINIGR
☑	507	732.35	2194.04	2193.98	0.06	2	4	6e+02	1	CCPNRHLLGGHPGCKECTR + 2 Carbamidomethyl (C)
☑	1035	951.52	1901.02	1900.84	0.18	1	4	5.4e+02	1	TCQAECRMLTTTTTER + Carbamidomethyl (C)
☑	1718	1235.46	3703.37	3703.69	-0.32	0	4	5.2e+02	1	HLAACHGHEVVVGSASCGIHPAPQGGTSPCSAAPCPK + 3 Carbamidomethyl (C)
☑	1910	503.97	1508.88	1508.73	0.15	0	4	1.8e+02	1	SMDAFADAVNVTLR
☑	424	687.85	2060.54	2060.96	-0.42	0	4	9.4e+02	1	SSIGMHGSSCGLGDLSLPSTR
☑	1640	1182.54	2363.07	2363.08	-0.01	1	4	4.4e+02	1	TSKEEPTSETTFIACTVYNDK
☑	2309	785.05	2352.14	2352.02	0.12	1	4	1.7e+02	1	FMMIWIKYDDDNSGDICVR + 2 Oxidation (M)
☑	488	725.64	2173.91	2174.13	-0.22	1	4	5.8e+02	1	SPPGIALQGASVSDGYRTGLTK
☑	1732	1243.51	3727.51	3726.94	0.57	2	4	4.9e+02	1	VIQILLPYEAGLANDDNQTALIVAALCDKAKACR + 2 Carbamidomethyl (C)
☑	416	683.78	682.77	683.32	-0.55	0	4	1.7e+02	1	EGDVHK
☑	1375	1071.47	3211.39	3211.58	-0.20	2	4	5.1e+02	1	EKHSLDHQEAAKDANCQVLPPLAPATSWR
☑	2170	674.49	2020.46	2019.92	0.54	0	4	2.9e+02	1	EIINESDDEEILDMIDK
☑	571	770.35	2308.04	2307.97	0.07	1	4	4.8e+02	1	GFGQMICGMGVGNCASEDFKK + 2 Carbamidomethyl (C); Oxidation (M)
☑	1757	1269.40	1268.40	1268.75	-0.35	1	4	6.3e+02	1	LDITGKPIKER
☑	467	714.84	1427.66	1427.73	-0.08	0	4	7.7e+02	1	QCIPTWIGGLQGR
☑	1789	650.34	1298.67	1298.77	-0.10	2	4	1.8e+02	1	RVVSARLISAVE
☑	41	439.79	1316.36	1316.77	-0.41	1	4	4.8e+02	1	KALFLEINELK
☑	650	807.91	1613.81	1613.87	-0.07	1	4	9.1e+02	1	QARVELHVGGVHANK
☑	1970	808.10	1614.19	1614.71	-0.52	0	4	2.5e+02	1	CMSYLMPIAEQEK + Carbamidomethyl (C); Oxidation (M)

☑	2030	569.84	1706.50	1706.77	-0.28	2	4	2.3e+02	1	MFRVMRTVGEMMR + 4 Oxidation (M)
☑	1914	758.06	1514.10	1513.70	0.40	1	4	2.2e+02	1	NSAEVKYEDAFNK
☑	105	506.61	1011.21	1011.37	-0.17	0	4	3.8e+02	1	CEGCCVGWR
☑	489	725.72	724.71	725.28	-0.57	0	4	1.1e+02	1	MNPWY + Oxidation (M)
☑	185	557.98	1113.95	1113.49	0.46	0	4	5.6e+02	1	LTCCSVFER + Carbamidomethyl (C)
☑	1662	1196.16	1195.15	1195.66	-0.51	2	4	6e+02	1	DKVPAPGAKGEK
☑	713	832.95	831.94	832.35	-0.40	0	4	5.1e+02	1	ENGDGWR
☑	1184	1001.60	3001.79	3001.31	0.48	2	4	5.5e+02	1	CLVCLNPCKYCAGTIRWCPHCYENR + Carbamidomethyl (C)
☑	1143	495.33	988.65	988.51	0.14	1	4	2.1e+02	1	MTKAFYTK
☑	1632	1178.83	3533.46	3533.77	-0.31	1	4	6.1e+02	1	MATQCSSNFAAVNRMQALAMILYLVVVVVMR + Carbamidomethyl (C); 3 Oxidation (M
☑	2299	777.17	2328.50	2328.10	0.41	1	4	2.1e+02	1	NAHQMRVDAIAVCEEALETR + Carbamidomethyl (C); Oxidation (M)
☑	739	843.30	842.29	842.37	-0.07	0	4	2.3e+02	1	CAYMVEK
☑	1684	605.74	1209.46	1209.70	-0.25	1	4	1.7e+02	1	LPSLCLPRGVR
☑	2022	847.00	1691.98	1691.94	0.04	2	4	2.1e+02	1	LRFLYGPTAVAKDNK
☑	539	748.76	2243.27	2243.21	0.05	2	4	6.4e+02	1	AIEKGCQVISGTPGRIMGLIK + Carbamidomethyl (C); Oxidation (M)
☑	107	508.15	1521.43	1521.74	-0.30	1	4	4.6e+02	1	RPSQNLCKDYNK + Carbamidomethyl (C)
☑	527	740.14	1478.26	1477.71	0.55	1	4	6.8e+02	1	AQGANRDAAGASPHR
☑	688	825.33	824.32	824.37	-0.05	0	4	5.1e+02	1	FINSMVD
☑	1900	750.21	1498.41	1498.73	-0.32	1	4	2.6e+02	1	NSDMGLAGPVPNRR + Oxidation (M)
☑	451	705.30	2112.89	2113.03	-0.14	2	4	6.1e+02	1	RHFYELFKSCEEGEVIK
☑	1512	562.82	1123.62	1123.61	0.01	1	4	1.8e+02	1	RLEGAAVSHGK
☑	2087	612.02	1833.04	1832.96	0.08	2	4	1.8e+02	1	VFLDLVEEERVARCR
☑	294	623.25	1244.48	1244.64	-0.16	1	4	5.8e+02	1	MVKTLECHLR + Oxidation (M)
☑	282	614.10	1839.27	1839.12	0.15	2	4	6.7e+02	1	NHLVILAHAKKLGGQLK
☑	362	650.38	1948.13	1948.01	0.12	1	4	5.9e+02	1	DKVCLTGSAVAALCASGLAAK
☑	1292	1043.17	3126.49	3126.41	0.08	1	4	6.4e+02	1	DKTLFFPSDTTEILEGDEVFFMCEADK
☑	2060	595.91	1784.71	1784.88	-0.17	1	4	1.8e+02	1	NSVCGGKSGGESVAHVIK + Carbamidomethyl (C)
☑	1503	1119.97	3356.89	3356.80	0.10	0	4	6.2e+02	1	NICIIVVVIGVASFISYFMYIGLFLSAGFK + Carbamidomethyl (C); Oxidation (M)
☑	1990	550.28	1647.81	1647.98	-0.17	2	4	2e+02	1	VLRRWFPLLGVHR
☑	388	667.46	1332.91	1332.65	0.26	0	4	5.8e+02	1	QNALSPLSCSGTR
☑	43	442.40	1324.18	1324.64	-0.46	2	4	5.2e+02	1	NKSMALQCMKR + Oxidation (M)
☑	325	636.52	635.52	635.26	0.26	0	4	1.9e+02	1	ECGIGT + Carbamidomethyl (C)
☑	1652	1189.33	3564.98	3564.72	0.26	2	4	6.2e+02	1	DTVVFSCPQCRHMQPKPLTDLCFCGKVSKPK + Carbamidomethyl (C); Oxidation (M)
☑	1708	614.37	1226.72	1226.70	0.02	1	4	2e+02	1	IPNDTTGILRK

☑	926	461.22	920.43	920.51	-0.08	1	4	2e+02	1	ETFRAIGK
☑	2219	1058.36	2114.70	2114.10	0.60	1	4	2.3e+02	1	GTVLSANRILCSFPFFASGK
☑	646	805.89	1609.77	1609.80	-0.03	2	4	9.3e+02	1	SEVSFVCKTEEPKK
☑	298	624.37	1246.72	1246.68	0.04	1	4	5.5e+02	1	SRSPVFWGALK
☑	2229	1068.35	2134.68	2135.04	-0.35	1	4	2.6e+02	1	LEIMTRPDTVDGEKQMTR + Oxidation (M)
☑	285	615.68	1229.36	1229.67	-0.32	1	4	6.3e+02	1	KLGSESQVINR
☑	707	830.83	829.83	830.40	-0.58	0	4	6.9e+02	1	HSGIDFR
☑	1842	705.16	1408.31	1408.71	-0.40	1	4	3.1e+02	1	CGGFLARVCLGTR + Carbamidomethyl (C)
☑	69	471.91	1412.71	1412.69	0.02	0	4	3.9e+02	1	DFVNSIISFESR
☑	468	715.51	2143.52	2143.11	0.41	1	4	7.5e+02	1	QRLFVCLAPESQPELAASK + Carbamidomethyl (C)
☑	1302	1045.70	1044.69	1044.53	0.16	1	4	2.2e+02	1	AESNERAIR
☑	513	734.86	2201.55	2202.11	-0.55	1	4	9e+02	1	EQQEVVRSCALGGSVNASQIK
☑	993	938.04	937.03	937.48	-0.45	0	4	3.5e+02	1	VFLDTESK
☑	1379	1075.51	1074.50	1074.55	-0.05	1	4	2.3e+02	1	AKLVDGVDCR
☑	1477	1111.73	3332.16	3331.60	0.56	1	4	7e+02	1	GHKQCVELLLQEEAGLCNIMGGTALMAAASK + 2 Carbamidomethyl (C); 2 Oxidation
☑	1826	1364.03	4089.06	4088.96	0.10	2	4	5.5e+02	1	SNMSRHLFYSAVLLLLVVMMCCACEAALAEEKSTDPK + Oxidation (M)
☑	1671	1201.60	3601.78	3601.86	-0.07	2	4	5.3e+02	1	IAPVSETSLSTMSRAHVAMPQAVAADGVRQRPPR + Oxidation (M)
☑	411	681.58	1361.15	1360.67	0.48	1	4	7.6e+02	1	FNEPITEAERR
☑	857	893.80	892.79	892.47	0.33	0	4	6.1e+02	1	SPSSIFQK
☑	954	930.02	929.01	929.51	-0.50	0	4	4.7e+02	1	CVITGRPK + Carbamidomethyl (C)
☑	2369	863.54	2587.60	2587.25	0.35	1	4	2e+02	1	MRLTCYECLAMGNAVGMIEVVLR + Oxidation (M)
☑	242	595.47	1783.38	1783.03	0.35	1	4	6.7e+02	1	EQAILTGSAKVLCIPLK
☑	1018	946.30	2835.87	2835.51	0.36	2	4	8.2e+02	1	VNKIISDSEQSSPDKPGTPIQLAQRK
☑	1653	1189.39	2376.76	2376.24	0.52	1	4	6.6e+02	1	HIETATVRVHEIPVEFCALR + Carbamidomethyl (C)
☑	393	671.38	1340.74	1340.71	0.03	1	4	5.4e+02	1	CVCIVAHLEKAR
☑	1009	942.89	2825.66	2825.41	0.25	2	4	6.1e+02	1	NSQLIVHLQNCFVPKDDSNDNLKK + Carbamidomethyl (C)
☑	1034	951.17	1900.32	1899.93	0.39	1	4	6.9e+02	1	LVNGLAMAEMARDPEQR
☑	1175	998.33	1994.65	1994.89	-0.24	0	4	7.3e+02	1	GGEGEVIAPPCTGACSLFCK + Carbamidomethyl (C)
☑	574	772.11	2313.30	2313.06	0.25	0	4	7.9e+02	1	YAMIYTPMYPCGSIGTLVCAK + 2 Oxidation (M)
☑	1338	1059.85	2117.69	2117.98	-0.29	1	4	6.6e+02	1	REEEIENNEMEIIDDIK
☑	1788	433.77	1298.29	1298.53	-0.25	0	4	3e+02	1	CSEEVLCQMK + 2 Carbamidomethyl (C); Oxidation (M)
☑	2404	920.15	2757.43	2757.45	-0.01	2	4	1.7e+02	1	ELSPQFKFNNLISFYKSIGELQR
☑	1859	722.97	1443.92	1443.82	0.10	1	4	2e+02	1	STLPIINDIRFR
☑	888	905.08	1808.15	1807.97	0.18	2	4	7.2e+02	1	RDSVLCHGLIPVRMR + Carbamidomethyl (C)

☑	890	905.18	2712.51	2712.51	-0.00	1	4	7.4e+02	1	FAEALLCIPKILATNASLDAIDLVAK
☑	1527	1128.33	2254.64	2254.11	0.52	1	4	7.2e+02	1	LLVDLSRGSEHSDQYPWPR
☑	338	643.43	1927.28	1926.98	0.30	0	4	6.5e+02	1	GVTSFTVYGLVPAADFQR
☑	611	787.83	2360.45	2359.97	0.48	1	4	7.4e+02	1	ETSVSFQPSVDASDTGDDSRSCA
☑	1349	1061.95	3182.84	3182.63	0.22	0	4	7e+02	1	FFLTDYAFLFYSLLLLMGPFILGMYR + 2 Oxidation (M)
☑	1624	1173.18	3516.51	3516.72	-0.22	2	4	7.3e+02	1	CDLDGDVTVVEMPALPEQRWVHVVTSHARGK + Carbamidomethyl (C); Oxidation (M)
☑	1449	1098.98	3293.93	3293.48	0.45	1	4	6.8e+02	1	FWHNLACFAQNNFNFSNSRELCEEVFK
☑	907	913.58	912.57	912.47	0.10	1	4	1.8e+02	1	VNFKDYK
☑	1617	1171.08	3510.22	3510.65	-0.43	2	4	7.9e+02	1	YQMFTEVVDDKGKQIPELNDCCFIYALQK + Carbamidomethyl (C); Oxidation (M)
☑	343	645.17	1932.49	1932.19	0.30	2	4	9e+02	1	LQILNLAVKLGLREPQK
☑	475	718.29	1434.57	1434.74	-0.17	2	4	7e+02	1	DVVAMAGAMVRRK + 2 Oxidation (M)
☑	633	798.14	2391.40	2391.08	0.31	1	4	7e+02	1	SGMMRTGVLETYPYDQNELR + 2 Oxidation (M)
☑	1097	973.93	2918.76	2919.25	-0.49	2	4	8e+02	1	QAGASRIVMNCPDEMDVKAMCTWMK + Carbamidomethyl (C); 3 Oxidation (M)
☑	414	683.04	1364.06	1363.66	0.40	1	4	5.9e+02	1	AVMQRDLQEMK + Oxidation (M)
☑	803	873.14	872.13	872.39	-0.25	0	4	3.4e+02	1	YGFECVR
☑	1641	592.60	1183.19	1182.70	0.49	1	4	3.2e+02	1	YLVYAVTKVK
☑	1236	1020.49	3058.44	3058.59	-0.15	2	4	6e+02	1	HLAAIGAAQQERLLTCDFLHRYYTLR
☑	460	709.44	708.43	708.33	0.10	0	4	6.3e+02	1	VSLFDE
☑	635	799.19	2394.54	2394.24	0.30	2	4	8.3e+02	1	HAEIEKHLDVMTAQILQMKK + 2 Oxidation (M)
☑	1432	1093.98	3278.93	3278.83	0.10	0	4	7.1e+02	1	LFANLSGPPAQEVALPYALAASLVILAAAATQK
☑	2346	1241.82	2481.63	2482.19	-0.56	1	4	2.8e+02	1	WGASRPSGDWASRGGAVAAAAYYR
☑	1238	1021.20	3060.58	3060.47	0.11	2	4	6.9e+02	1	RMAALQSTKGSSGGEGSRPPEPGADGELYR
☑	2234	716.80	2147.37	2146.99	0.37	0	4	2.1e+02	1	DYIEADNLFSTFEVEDIK
☑	711	832.09	1662.16	1661.71	0.45	0	4	8.3e+02	1	CAGGNAQPIPCSSTDK + 2 Carbamidomethyl (C)
☑	2321	795.09	2382.25	2382.23	0.02	1	4	1.9e+02	1	AAPAPSALPLSPPSSTYASAQSRR
☑	2442	977.41	2929.22	2929.51	-0.29	1	4	1.6e+02	1	AVATCVQAGTAVTSAKCSGLGAPAVSLQVR + 2 Carbamidomethyl (C)
☑	189	560.45	1678.32	1678.88	-0.56	0	3	8e+02	1	QSHSPGVYGHTAVVLK
☑	326	636.54	1271.07	1270.57	0.50	0	3	8.2e+02	1	NLGGSCFPMTTK + Oxidation (M)
☑	925	921.30	2760.88	2760.64	0.24	1	3	7.9e+02	1	VALNVDKPVIVVLNKLDLLSDALTAK
☑	165	546.47	1636.38	1635.87	0.51	0	3	7.7e+02	1	VDNPGTSARPLVPASR
☑	2203	696.76	2087.27	2087.04	0.22	2	3	2e+02	1	RGSGEHSAISLSRMTEELK
☑	1183	1001.14	2000.26	1999.92	0.34	1	3	7.9e+02	1	SLCPRAGLFCIPQHCQN + 2 Carbamidomethyl (C)
☑	487	723.66	2167.97	2168.19	-0.21	0	3	7.9e+02	1	CEVAATLVLGLLLDPSAGTLR + Carbamidomethyl (C)
☑	1698	609.82	1217.62	1217.69	-0.07	0	3	2.4e+02	1	LHALHVNVTSK

☑	1821	1358.39	4072.16	4072.06	0.10	2	3	6.4e+02	1	DANSRLTPTQLSIIALGVAYGMQYLHSQNMVHRDLK + 2 Oxidation (M)
☑	804	873.36	2617.06	2617.31	-0.24	0	3	7.2e+02	1	ETLPVAMAAVTDGCNTVALLLSESR + Carbamidomethyl (C)
☑	1770	640.36	1278.71	1278.62	0.09	1	3	2.3e+02	1	QYKLNDEDVR
☑	2215	703.37	2107.09	2107.22	-0.12	1	3	2.1e+02	1	AALSLPEAANKLFGGPLLAVR
☑	1686	607.33	1212.64	1212.54	0.11	0	3	2.1e+02	1	MTGSMDSLVEK + Oxidation (M)
☑	2096	924.46	1846.91	1846.98	-0.07	2	3	2.4e+02	1	KLTNDTELIPFDKWK
☑	2302	1172.17	2342.33	2342.20	0.13	2	3	2.4e+02	1	EAIRCIERSESVLVSAHTSAGK
☑	1247	1024.38	3070.11	3069.58	0.53	1	3	6.8e+02	1	ITHVINCCLESQSPKYGVPNLACLLLK + 2 Carbamidomethyl (C)
☑	2360	849.06	2544.16	2544.28	-0.12	2	3	2e+02	1	TQVSSPKQEMKWVQLPPQYDR
☑	1152	991.58	2971.71	2971.39	0.32	2	3	6.4e+02	1	TAHVSRVMEKTASDNASTGGPSVEQCPR + Carbamidomethyl (C)
☑	1287	520.85	1039.68	1039.52	0.17	0	3	2.1e+02	1	SLAGASGQGHR
☑	280	613.71	1838.09	1838.02	0.07	1	3	5e+02	1	LRQTMIQIANSIHVAK + Oxidation (M)
☑	806	437.29	872.56	872.52	0.04	1	3	3.3e+02	1	RGTTAVLR
☑	994	938.22	2811.64	2811.38	0.26	1	3	7.9e+02	1	DNVYGMAYLYSTPLSIEHQWRLR
☑	1441	1097.34	3288.99	3288.40	0.58	2	3	7.4e+02	1	YMCAYRVAKAMSSMPDGNSEIPSEFEMR + Carbamidomethyl (C); 2 Oxidation (M)
☑	1759	635.86	1269.71	1269.60	0.11	0	3	2.3e+02	1	SMVYQEISASR
☑	2121	636.89	1907.64	1907.05	0.60	2	3	2.4e+02	1	KAVACLTYIIKNGPFNR
☑	1907	754.08	1506.14	1505.77	0.36	0	3	3e+02	1	YDSPVGAVGSLLSGGK
☑	370	655.13	1962.37	1961.97	0.40	1	3	8.2e+02	1	IEEQLANNEEKYQINK
☑	1427	1091.67	3272.00	3271.61	0.39	0	3	7.2e+02	1	AAAGQATAAAAPEAAQADILIGHTGTGGMTVSTFK + Oxidation (M)
☑	1550	1141.32	3420.94	3420.67	0.27	1	3	8.6e+02	1	VENLQDCPCGNTIGLVGIDQYLVKSGTISDR + 2 Carbamidomethyl (C)
☑	2319	793.39	2377.16	2377.40	-0.24	1	3	1.9e+02	1	VASVALVSAALMLPAPPLSLSKVK + Oxidation (M)
☑	238	591.87	590.86	591.31	-0.45	0	3	3.9e+02	1	ASLACK
☑	1544	1137.73	3410.18	3409.64	0.54	1	3	7.1e+02	1	FYLYAWHLQTSVIRTDIMQNAYFNQCK + Carbamidomethyl (C)
☑	1911	755.45	1508.88	1508.81	0.07	1	3	2.3e+02	1	MEHVEGLLQKGIR
☑	672	816.15	1630.29	1630.82	-0.52	2	3	9.3e+02	1	QALEEVEAKKSEDR
☑	706	829.87	828.86	828.42	0.44	0	3	9.1e+02	1	QGQAGVNR
☑	1819	1357.05	2712.08	2712.24	-0.16	1	3	5.9e+02	1	DYMCVDDGAIKGNGEATELLLASDR + Carbamidomethyl (C)
☑	2385	890.28	2667.82	2667.40	0.42	1	3	3.1e+02	1	QQRLTVALVSSFEPPHDSTSLSLR
☑	153	534.32	1066.62	1066.54	0.08	0	3	5.2e+02	1	AEELLAHER
☑	374	655.84	1309.67	1309.60	0.07	0	3	8.8e+02	1	CPTEEFVSGVSR
☑	1899	498.99	1493.94	1493.66	0.28	0	3	2.2e+02	1	ICCASLSEAQQER + Carbamidomethyl (C)
☑	461	710.22	2127.64	2128.09	-0.44	1	3	8.3e+02	1	EQFVTLYGKYDPLWELK
☑	598	784.04	2349.10	2349.11	-0.01	2	3	6.5e+02	1	YLENRMSTGKVLLYCNSTAN + Carbamidomethyl (C); Oxidation (M)

☑	486	723.52	2167.54	2167.23	0.31	2	3	8.6e+02	1	CVLPGAEVGIVPTRPKKFTR
☑	766	855.58	2563.71	2564.29	-0.58	2	3	8.2e+02	1	YLKALLKCGFYALDCGSFVSPR + 2 Carbamidomethyl (C)
☑	1530	1129.51	1128.50	1128.52	-0.02	1	3	6.4e+02	1	GYSKELCSSR
☑	1006	941.04	940.03	939.46	0.57	1	3	3.3e+02	1	LSMPERHA
☑	2195	1033.76	2065.50	2065.86	-0.36	0	3	3.8e+02	1	NGHQLPQSQSCHCHYDR + Carbamidomethyl (C)
☑	702	828.77	2483.30	2483.13	0.17	1	3	7e+02	1	LSSLREMMMAMNQTYTPYLR + 3 Oxidation (M)
☑	978	933.77	932.76	932.45	0.32	0	3	2.6e+02	1	EEGATIDAK
☑	1219	1014.28	2026.54	2027.01	-0.47	0	3	8.8e+02	1	APPAASPAYGSPTIGNGSVTGR
☑	1211	1012.44	3034.31	3034.71	-0.40	2	3	5.9e+02	1	EIAKVLIAHGANVNAQKLMGEVPLHLAAK
☑	172	551.48	1100.95	1100.67	0.28	2	3	7.8e+02	1	VEKTLTRVR
☑	553	758.95	1515.89	1515.88	0.01	2	3	1.1e+03	1	LIGRMEKPSTKIK + Oxidation (M)
☑	275	610.87	1219.73	1219.69	0.03	2	3	7.8e+02	1	YNKLKPKSDK
☑	664	814.64	2440.91	2441.15	-0.24	0	3	8.5e+02	1	ICYNNSSEPVMQTLLEELSSK + Carbamidomethyl (C)
☑	1243	1023.12	1022.11	1021.53	0.58	2	3	7.3e+02	1	SSCVKSRQK
☑	84	486.29	970.57	970.49	0.08	0	3	4.6e+02	1	MAATPQPQK
☑	1305	1046.27	1045.26	1045.53	-0.27	1	3	8.3e+02	1	ELEESPKSK
☑	2376	875.87	2624.60	2624.21	0.39	1	3	2.1e+02	1	ETMAEAAEYVFGVHEGTVDQKADK
☑	591	781.05	2340.14	2340.23	-0.09	2	3	7.3e+02	1	TALRLYTESLERHPNNVTAR
☑	1606	584.38	1166.75	1166.48	0.27	0	3	2.3e+02	1	MAEPMSWGSR + Oxidation (M)
☑	290	619.64	1855.91	1855.88	0.03	1	3	7.6e+02	1	LKESHATFNMSFDTTK
☑	482	721.00	1440.00	1439.78	0.21	2	3	7.6e+02	1	KSPQEDRPRSLK
☑	1672	601.38	1200.76	1200.63	0.13	0	3	2.6e+02	1	AQALQDLCLAR
☑	592	782.18	2343.53	2344.08	-0.55	1	3	8.8e+02	1	AATEQAASRYCQLFSGDELER
☑	1697	1217.92	2433.83	2433.39	0.44	2	3	7.1e+02	1	VAIYVQGFELPKTETKGTVLLK
☑	305	627.24	1878.70	1878.98	-0.29	2	3	6.4e+02	1	QATRRVTLNVFGTTCR + Carbamidomethyl (C)
☑	70	472.27	942.53	942.44	0.09	0	3	4.9e+02	1	MVCPAPPR + Carbamidomethyl (C); Oxidation (M)
☑	2099	617.90	1850.67	1850.97	-0.30	1	3	2.8e+02	1	FLHPYRSAEIFLSSGK
☑	233	588.38	587.37	587.30	0.07	0	3	2.9e+02	1	AAEAAR
☑	663	813.76	812.76	813.33	-0.57	0	3	6.9e+02	1	TYECNK + Carbamidomethyl (C)
☑	557	762.98	2285.92	2285.92	-0.00	0	3	1e+03	1	CCTLPDDSTENDVGSVSATCR + 2 Carbamidomethyl (C)
☑	619	792.84	791.83	791.38	0.45	0	3	4.7e+02	1	QGTSPFGV
☑	2351	835.26	2502.77	2502.24	0.54	1	3	3.8e+02	1	QDSYIHILTSQNDQLTEQNKK
☑	439	698.14	2091.40	2091.10	0.30	2	3	8.8e+02	1	ARIQYEWVVCLERIASR
☑	1615	1170.56	3508.65	3508.72	-0.07	2	3	6.2e+02	1	RGDATEHSGAGSGGNICFFLAAAEKVSCLVPFLK + Carbamidomethyl (C)

☑	678	820.30	2457.89	2458.22	-0.33	2	3	8.2e+02	1	ESKLFGIFEPEYVAKDIMNGR + Oxidation (M)
☑	1431	1093.36	2184.70	2185.08	-0.38	0	3	8.2e+02	1	QQGGVPLLECSAAFNPIDAQK
☑	798	871.70	2612.09	2612.32	-0.23	1	3	8.8e+02	1	ATVIAHDLAMSVGVCMDPLLTRAGR + Oxidation (M)
☑	120	514.96	1027.90	1027.58	0.32	1	3	5.8e+02	1	EVEVLQRR
☑	1376	1072.27	2142.52	2143.01	-0.50	1	3	8.5e+02	1	EKMAEVEGYSAALAEFVDGK
☑	613	789.89	2366.64	2366.08	0.56	0	3	1.2e+03	1	SSGALAAEDANVMVVGVADCTQSR + Oxidation (M)
☑	695	825.98	824.97	825.41	-0.44	0	3	7.9e+02	1	EAHNSIR
☑	2042	575.60	1723.78	1723.80	-0.02	1	3	2.3e+02	1	SCKGDVCVGGGTTNIWK
☑	2466	1048.02	3141.04	3141.33	-0.29	0	3	2.9e+02	1	SSTEPLSEICHFSGQPQTFTEDNADNCK + Carbamidomethyl (C)
☑	122	515.42	1543.23	1542.88	0.35	0	3	7.8e+02	1	TLLPLDITQFLNR
☑	1334	1058.33	3171.97	3172.51	-0.55	0	3	8.8e+02	1	QQGMTQGTVVGMLQYPWVQGGCTAVQYVK + Oxidation (M)
☑	2481	1073.49	3217.45	3217.62	-0.17	1	3	1.7e+02	1	DFGAILNQYSYNLHPGDIIAGTVFHKEAK
☑	1483	1114.84	2227.66	2228.20	-0.54	2	3	8e+02	1	EVTQPLSRFLPADAVCNKLK
☑	835	882.94	881.93	881.52	0.42	2	3	4.1e+02	1	KGVHKASR
☑	636	799.52	798.51	798.36	0.15	0	3	8.1e+02	1	CIGSEYK
☑	1768	1278.61	2555.20	2555.29	-0.10	1	3	6.2e+02	1	DVVPWYMIGMRTVHHLDLAFR
☑	2140	971.01	1940.01	1940.04	-0.04	1	3	2.3e+02	1	LIVFYSFPRLDENVTK
☑	254	300.29	598.56	598.37	0.19	0	3	1e+02	1	IIEPK
☑	1784	1294.48	3880.42	3879.87	0.54	0	3	7.4e+02	1	LFGWMILLCECIISYSVLEEDVLDSPDLGFLCTK + Oxidation (M)
☑	2407	924.81	2771.40	2771.45	-0.05	1	3	2.1e+02	1	RQLTLPGRPGSAPVPLCHACAICVAK + 2 Carbamidomethyl (C)
☑	1423	1089.27	3264.78	3264.65	0.13	2	3	8.6e+02	1	YISDLEDSYYNSKAIKMKPIIFSQQQR
☑	712	832.61	2494.80	2495.29	-0.49	1	3	1.1e+03	1	GVVTSATPAPARQPQTGPPVHDQGK
☑	894	907.00	2717.98	2718.37	-0.38	2	3	1.1e+03	1	LPDMGSSDIQVSMNRESTILEIKR
☑	696	827.36	1652.71	1652.78	-0.07	1	3	7e+02	1	AEDHNDEGAALAQKGK
☑	740	843.74	2528.20	2528.31	-0.11	1	3	9.1e+02	1	FSNVPKPKASGSSVSYFTTHIFK
☑	763	855.00	853.99	853.41	0.58	0	3	4e+02	1	CTVVGGYR
☑	2274	752.51	2254.50	2254.08	0.42	2	3	3.6e+02	1	YKVHDEMNECKGDYILIK + Carbamidomethyl (C)
☑	215	576.39	1726.16	1725.99	0.17	2	3	7.6e+02	1	EIEELKNIIQKLEK
☑	556	762.70	761.70	761.37	0.32	0	3	9.3e+02	1	AYFFSK
☑	658	810.94	2429.79	2429.24	0.55	1	3	1.1e+03	1	GITSLMYCAEVGNTSMAKLLLSK
☑	856	893.68	2678.02	2678.25	-0.23	1	3	9.3e+02	1	LLNDDETDWWGVLDQNLVMCRK + Oxidation (M)
☑	1192	1004.17	2006.33	2006.01	0.32	2	3	8.5e+02	1	HLCAHRVAETIEERQSK
☑	1206	1008.90	2015.78	2015.92	-0.14	1	3	9.2e+02	1	YVDEHGRCPITGDPLCK + 2 Carbamidomethyl (C)
☑	2327	803.89	2408.66	2408.31	0.35	2	3	3.9e+02	1	RNCISGSRNGPAIAALAELIVAR + Carbamidomethyl (C)

☑	2499	1208.89	3623.66	3623.64	0.02	2	3	1.8e+02	1	QCSGYLRGASVEVISSMDQMDPAIDAYIERCK + Carbamidomethyl (C); 2 Oxidation (
☑	905	913.41	1824.81	1824.94	-0.13	0	3	6.6e+02	1	SVPTPPQVASAMITASPR + Oxidation (M)
☑	246	597.33	1788.97	1788.85	0.12	1	3	6.7e+02	1	QQDAQTEVPGTKSDGTK
☑	732	842.10	841.09	841.41	-0.32	1	3	7.9e+02	1	WRGMHR
☑	1516	1125.50	2248.99	2249.13	-0.14	2	3	6.7e+02	1	VHSYVAMVDLAGCERVKQTK + Oxidation (M)
☑	1761	636.97	1271.93	1271.65	0.29	1	3	2.7e+02	1	DKEGSPGGGSLLR
☑	1546	1138.92	3413.75	3413.75	0.00	2	3	7.2e+02	1	YATDTVQSFLLFSNSTDLLQHLRQFRSAR
☑	2328	804.91	2411.70	2411.16	0.54	0	3	3.9e+02	1	DPQTLSTVLACTLQCLAGFMQR + Oxidation (M)
☑	313	630.78	629.77	630.34	-0.58	1	3	1.3e+03	1	AAGERK
☑	963	931.86	2792.55	2792.37	0.17	2	3	8.2e+02	1	RPEGNMALYNAATGQLLRRCMQAR + Carbamidomethyl (C); Oxidation (M)
☑	1702	1221.79	2441.57	2442.10	-0.53	0	3	8.2e+02	1	AFPDLVGETVVDSFNCAWMER + Carbamidomethyl (C)
☑	2386	890.64	2668.90	2668.39	0.51	2	3	3.1e+02	1	GRTVDFKNTVVIMTSNLGSEIIMK + Oxidation (M)
☑	2460	1024.57	3070.68	3070.64	0.04	2	3	2e+02	1	MTKSLTKQLHQYSATAVTQLPHVFVAR + Oxidation (M)
☑	180	555.54	1109.06	1109.60	-0.54	0	3	8e+02	1	YLETSSVLAK
☑	1567	1149.04	3444.11	3443.66	0.45	1	3	1e+03	1	GVAEFIAATGTFDDVCAPGERGYSDVSLAVEGLK
☑	2371	868.63	2602.87	2603.11	-0.24	2	3	3.4e+02	1	MWSWMMMARLGGGEAEQAGRER + 4 Oxidation (M)
☑	538	748.33	2241.97	2242.19	-0.21	1	3	8.2e+02	1	ANAGFTVALNKLAHLSPAEYR
☑	530	743.21	2226.59	2226.91	-0.32	1	3	1e+03	1	CGPSCTSGIMTESDSPCRSPR + Carbamidomethyl (C)
☑	676	817.34	2449.00	2449.36	-0.36	2	3	8.6e+02	1	RVGPCALVLLLLVRVCAAGCPGR + 2 Carbamidomethyl (C)
☑	1664	599.77	1197.52	1197.60	-0.08	1	3	2.5e+02	1	FSKSVAGSSSNK
☑	2410	925.09	2772.24	2772.35	-0.11	1	3	2.2e+02	1	DKFNSIFGMLMEVNDHAAAQIIHR + Oxidation (M)
☑	236	591.27	590.27	590.25	0.01	0	3	7.2e+02	1	DDVDK
☑	1525	1127.71	3380.10	3379.58	0.52	0	3	8.2e+02	1	MFQLIPLFVASALMAACQPDGNHTATCAQNK + Carbamidomethyl (C); 2 Oxidation (
☑	1953	525.76	1574.25	1574.67	-0.42	0	3	4.7e+02	1	AWSGNACGHEAMLR + Carbamidomethyl (C); Oxidation (M)
☑	2444	980.80	2939.39	2939.44	-0.05	1	3	2.2e+02	1	EIYVPDSVIHIGESCFSNCTKLTSIK + Carbamidomethyl (C)
☑	1598	1163.23	2324.45	2324.14	0.32	2	3	8e+02	1	TSTGRTALMIAARYGHTDVCK + Carbamidomethyl (C); Oxidation (M)
☑	1661	1195.00	3581.97	3581.58	0.39	1	3	7.9e+02	1	VDMSPRETDYFHYYLLEAATHMGDEDSFVK + Oxidation (M)
☑	2383	884.91	2651.72	2651.41	0.31	2	3	3.3e+02	1	GIALRVFHFVAMLASKNHAGEEVR
☑	609	787.72	786.71	787.30	-0.59	0	3	3.2e+02	1	HDSGGCR + Carbamidomethyl (C)
☑	723	838.77	2513.29	2513.27	0.02	2	3	8.7e+02	1	STALQEQIAGATTVAPEPRDMRR + Oxidation (M)
☑	1597	1163.10	3486.29	3486.83	-0.54	1	3	1e+03	1	MQEGVVALMQMANTTSCIESVKQAGIPVIVVIK
☑	946	927.77	1853.53	1854.04	-0.51	2	3	8.3e+02	1	AAIIPTRMELQNLKEK
☑	928	921.79	1841.56	1841.92	-0.36	2	3	8.6e+02	1	VREEATAPSPAMQQRR + Oxidation (M)
☑	1607	1169.18	1168.18	1167.64	0.54	1	3	4.1e+02	1	KYLFITNNR

☑	1744	627.42	1252.83	1252.56	0.27	0	3	3e+02	1	LYGPDGSSEGSGK
☑	90	492.15	982.29	982.52	-0.23	0	2	6e+02	1	FVSAAVAYR
☑	745	846.97	2537.89	2538.34	-0.44	2	2	1.3e+03	1	IIGFGAYGTVCSAVANRSGERVAIK
☑	116	512.64	1534.91	1534.79	0.12	1	2	5.2e+02	1	GQPVSAALDEHQKR
☑	368	654.56	653.55	653.39	0.17	0	2	9.8e+02	1	IIGHSK
☑	1590	1160.80	3479.38	3478.83	0.55	2	2	9e+02	1	SSAQAELDAIAPLIAEAANSVQNIKKSNLDEIR
☑	1381	1076.38	3226.12	3225.52	0.60	1	2	9.7e+02	1	AKSFTATAASSPAVYGGAIDSLSNDGDSGAANPR
☑	1396	1080.88	2159.74	2160.01	-0.27	1	2	7.8e+02	1	DSAGMTSFELACSLGKTESVK
☑	350	646.82	1937.44	1937.95	-0.51	1	2	1.5e+03	1	QLGAEAATATPHACQRTR + Carbamidomethyl (C)
☑	1217	1014.08	1013.07	1012.58	0.50	1	2	9.7e+02	1	EQQLRALR
☑	1425	1090.52	2179.02	2179.25	-0.23	1	2	7.7e+02	1	IIPATHTLFAKQNGSVILTR
☑	1098	974.16	973.15	973.52	-0.37	1	2	9.4e+02	1	DNSILREK
☑	1242	1022.84	1021.84	1022.42	-0.59	0	2	7.9e+02	1	CTDDEEALK
☑	410	681.37	680.36	680.24	0.12	0	2	2e+02	1	CGEGCGR
☑	883	903.58	902.58	902.53	0.05	1	2	9.6e+02	1	DVMKIAVK
☑	2148	979.16	1956.31	1956.85	-0.55	2	2	3.1e+02	1	ECSKCGKQCITCLNEK + 3 Carbamidomethyl (C)
☑	531	743.58	2227.72	2227.15	0.58	1	2	1e+03	1	GWYVHRLLVNAVGMWASPR + Oxidation (M)
☑	1168	995.05	1988.08	1988.06	0.02	0	2	1.1e+03	1	YVLLCLGVLASQCLPPSR + Carbamidomethyl (C)
☑	1642	1184.35	3550.03	3549.91	0.12	1	2	9.3e+02	1	GVPLALQTELGIDLRHSTANILSELSTLSINMK + Oxidation (M)
☑	1667	1199.77	2397.52	2397.28	0.24	1	2	9.1e+02	1	DVDVVVIGEGVDAAAAVVSTLKDR
☑	1966	537.78	1610.33	1609.79	0.54	2	2	4.9e+02	1	GDTVRKFVESDMAR
☑	167	546.57	1636.68	1636.71	-0.03	1	2	9.8e+02	1	SDAPACSCCNKVLR + 3 Carbamidomethyl (C)
☑	316	632.07	1893.19	1892.83	0.36	0	2	1.3e+03	1	MGDDISYYSGGAVVGMAGK + Oxidation (M)
☑	603	785.62	2353.83	2354.10	-0.27	1	2	9.7e+02	1	TPGISSSQCTIGTSDCRSSVVR + 2 Carbamidomethyl (C)
☑	746	847.15	846.14	846.39	-0.25	0	2	3.5e+02	1	TCGPLQTAG
☑	1372	1068.93	3203.77	3203.54	0.24	1	2	8e+02	1	CMRGIYHNVAPNEIPPMYDPKPQQVYK + Oxidation (M)
☑	1736	1245.52	3733.53	3734.04	-0.51	2	2	7.5e+02	1	KLVPSMISLTELATEVAVAVVRGEVLPIDFGHATR + Oxidation (M)
☑	1504	1120.45	3358.34	3358.76	-0.42	2	2	7.8e+02	1	RLFYLSNTVTQCCVAMGAALLELLPYKLR + Carbamidomethyl (C); Oxidation (M)
☑	955	930.29	2787.86	2787.35	0.50	0	2	1.1e+03	1	YITQLSLSNCVMTDIPDPVFCSTIK
☑	2192	687.43	2059.28	2059.12	0.16	1	2	2.9e+02	1	KTTPTSLGNIYDIPAVIEK
☑	2310	786.07	2355.19	2355.22	-0.04	2	2	2.7e+02	1	TINAKEAIQNPNSIFNYYKK
☑	1330	1057.08	3168.22	3168.54	-0.31	0	2	1.1e+03	1	EPTFSMGDDTPLPILSEKPHLIYDYFK + Oxidation (M)
☑	2013	841.81	1681.60	1681.81	-0.21	0	2	2.7e+02	1	LHGAGVGPAGWMTAWR + Oxidation (M)
☑	2019	564.93	1691.77	1691.87	-0.10	1	2	3.2e+02	1	SVQPLVSEDQYAKTK

☑	1088	971.31	2910.90	2911.40	-0.50	2	2	1e+03	1	VCENVANFIMVSSNAVPMKLESSDRR + Oxidation (M)
☑	1620	1171.47	3511.39	3511.55	-0.16	0	2	8.5e+02	1	VVFDDSNAMNSADSQLLCPPSLPSRPCSGEMR + Carbamidomethyl (C); 2 Oxidation (
☑	449	705.16	1408.31	1408.72	-0.41	1	2	1.2e+03	1	GFLNGELSMWKK
☑	1246	1024.09	3069.26	3069.54	-0.29	0	2	9.5e+02	1	GVQLVETQMGFPVEELQAICCSHGLIIR
☑	1681	1209.97	2417.92	2418.23	-0.31	2	2	7.6e+02	1	LYVAATCPFCHRVEIVAREK + 2 Carbamidomethyl (C)
☑	322	635.83	634.83	635.26	-0.43	0	2	5.6e+02	1	ACEEK + Carbamidomethyl (C)
☑	1280	1038.29	2074.56	2074.87	-0.31	1	2	1e+03	1	GNIPYCDVYECESKENGR
☑	1625	1173.48	3517.43	3517.94	-0.51	2	2	8e+02	1	VLVVAEMHNGKVSPATLAAVTAGMKVGPVTALVAGK + 2 Oxidation (M)
☑	1886	739.05	1476.08	1475.70	0.38	1	2	3.4e+02	1	CPMRVEGVTNSAR + Carbamidomethyl (C)
☑	359	650.04	1947.09	1946.91	0.17	2	2	1e+03	1	DYEMKMETELYRVPK + Oxidation (M)
☑	1408	1085.17	3252.49	3252.55	-0.06	2	2	9.8e+02	1	MIVCCSRARFAMVMIGNDGLLEQSTHWK + Carbamidomethyl (C)
☑	1867	485.05	1452.12	1451.65	0.47	0	2	4.2e+02	1	MANNTIAALSCDR + Carbamidomethyl (C); Oxidation (M)
☑	2300	1168.35	2334.69	2335.22	-0.53	2	2	4.2e+02	1	TVSGVEVTELLRSLMERLCK + Carbamidomethyl (C); Oxidation (M)
☑	2475	1060.55	3178.63	3178.64	-0.02	2	2	2.1e+02	1	EGRKTDCTLYTTFAAVGLSNGTLLLHNVGK
☑	1176	998.40	1994.78	1994.99	-0.22	1	2	8.5e+02	1	EQEYINGEAIKAINEFK
☑	1725	1239.15	3714.42	3714.78	-0.37	1	2	9.9e+02	1	RNAQLLTLMGTKPGGVTYPDAATSTATTDEMAASSR + 2 Oxidation (M)
☑	2364	853.36	2557.07	2557.43	-0.37	1	2	2.5e+02	1	SQVQVAANVVKNGTRPLLPSSAPPK
☑	1541	1136.90	3407.67	3407.31	0.35	2	2	8.7e+02	1	GCLRCKSGYYLEDGECWECQGECWECSSK + Carbamidomethyl (C)
☑	1592	1161.67	2321.33	2321.01	0.32	2	2	8.1e+02	1	MLWKVHREMDCGQDSGNQR + 2 Oxidation (M)
☑	1916	758.46	1514.91	1514.88	0.03	1	2	3.1e+02	1	IIPAPKIYSSSAIR
☑	151	533.95	1065.89	1065.46	0.43	0	2	6.7e+02	1	SASDESSEVR
☑	255	599.85	1796.53	1795.98	0.55	1	2	9.6e+02	1	VDKSTIHEVVLVGGSTR
☑	2186	685.17	2052.49	2051.93	0.56	1	2	5e+02	1	NMLTKETTCAVCGDVPGEK + Carbamidomethyl (C)
☑	2392	895.98	2684.92	2685.16	-0.24	2	2	3.7e+02	1	FCYTCQQYKPDDAHHCRVCRR + Carbamidomethyl (C)
☑	244	596.07	1190.13	1189.65	0.48	2	2	9.8e+02	1	SKKILQACDK + Carbamidomethyl (C)
☑	240	592.01	591.00	591.29	-0.29	0	2	2.5e+02	1	STAADK
☑	862	895.95	894.94	895.45	-0.51	0	2	1.2e+03	1	VIEHNER
☑	2400	908.33	2721.97	2721.42	0.56	1	2	3.4e+02	1	GNKLTSVIFECNNSVVVFKPINDK + Carbamidomethyl (C)
☑	671	815.99	814.99	814.47	0.52	1	2	6.3e+02	1	GLPTSGRK
☑	2432	967.78	2900.33	2900.32	0.01	1	2	2.3e+02	1	CIKCKPSYGVVNGTCAQPIEHCMEYK
☑	444	700.23	2097.67	2097.16	0.51	2	2	9.4e+02	1	MKLGINITITDPNKAEAIR
☑	1623	586.90	1171.79	1171.66	0.13	1	2	3.3e+02	1	NIMEIPSIKK
☑	1729	1241.02	3720.04	3719.72	0.32	1	2	9e+02	1	SAMSIAQSAVACELMSQPPPCVVLFHVDDAAKCR + 2 Carbamidomethyl (C); 2 Oxidati
☑	2076	906.68	1811.35	1811.92	-0.57	2	2	4.6e+02	1	KEEVCALRPEEKEPR

☑	2441	976.47	2926.38	2926.33	0.05	0	2	2.4e+02	1	IFISTFGIMSMAYDCHCNIFQSLR + 2 Carbamidomethyl (C); Oxidation (M)
☑	471	717.85	716.84	716.37	0.47	0	2	7.9e+02	1	GSSEIPK
☑	839	884.99	883.98	884.44	-0.46	1	2	4.5e+02	1	ARATHCAR
☑	844	888.04	887.03	887.50	-0.46	0	2	1.3e+03	1	ATVDELLK
☑	919	917.00	915.99	915.50	0.50	1	2	1.4e+03	1	AMKNPSLR
☑	1511	1124.55	3370.63	3370.44	0.20	1	2	7.8e+02	1	KCTLCQNITDSVAIGDCGHSFICCFCSFK + 4 Carbamidomethyl (C)
☑	1633	1179.46	2356.90	2357.13	-0.23	2	2	8.6e+02	1	LMNAQALRDNTMSQYMQAKK + Oxidation (M)
☑	717	834.58	2500.72	2501.30	-0.58	2	2	1.1e+03	1	SDISLSGHSKRQAPLPIPADAADR
☑	579	773.97	772.96	773.39	-0.43	0	2	7.8e+02	1	FFAYAR
☑	1224	1015.48	3043.41	3043.48	-0.07	2	2	8.8e+02	1	LGMCLMPGAQKSQNAKGGTGVSGLLEMHR + Carbamidomethyl (C); Oxidation (M)
☑	1591	1161.32	2320.63	2320.17	0.46	2	2	1e+03	1	SVRRGVMTACGAVGLAGTCWGLR
☑	2339	816.60	2446.79	2447.18	-0.39	0	2	3.8e+02	1	SYVSNPVGSGNTLPGSHGLAGGTYR
☑	266	603.87	1808.59	1808.98	-0.39	2	2	1.5e+03	1	SPLSKDVAKVICEVGHK
☑	2256	1107.51	2213.00	2213.09	-0.09	1	2	2.6e+02	1	IAQERDEPIPGFIYDSAHR
☑	687	825.27	2472.79	2472.19	0.60	1	2	9.8e+02	1	DYCSSIKLCRPTASSLSMKPR + 2 Carbamidomethyl (C); Oxidation (M)
☑	964	931.86	2792.56	2792.61	-0.05	1	2	9.4e+02	1	IGIIIGVVVCIVAIIVFAVSCYICKK + Carbamidomethyl (C)
☑	2098	924.70	1847.38	1846.96	0.42	0	2	5.5e+02	1	AELIADLNNVFNIVMR + Oxidation (M)
☑	2469	1050.29	3147.83	3147.37	0.47	1	2	2.5e+02	1	HVVAGRSDCTASMHSVDGSTSCVFSGHQR + 2 Carbamidomethyl (C); Oxidation (M)
☑	583	774.59	2320.74	2320.15	0.59	2	2	1.2e+03	1	YGKSRFTCEITSPTEFVVTR
☑	314	630.83	1889.47	1889.83	-0.35	1	2	1.9e+03	1	GYIIDDEDICQRCYGK
☑	796	871.20	2610.58	2610.22	0.36	0	2	1.1e+03	1	VPSLVEMDGEVFAVVEAQCTEASK + Carbamidomethyl (C); Oxidation (M)
☑	545	376.60	751.18	751.47	-0.29	1	2	2.7e+02	1	ILKNHK
☑	495	728.29	727.29	727.40	-0.11	0	2	8.4e+02	1	GGAGTPIR
☑	940	925.71	2774.11	2774.16	-0.05	2	2	1e+03	1	DYCFSTCGLMEKKSNHMSYNAWK + 2 Oxidation (M)
☑	1363	1065.68	3194.02	3193.64	0.37	0	2	9.6e+02	1	CAGIAGVALHPSGTLAVAAVQTGQANHADAPVAR
☑	2462	1032.08	3093.22	3092.76	0.46	2	2	2.4e+02	1	QLQSGSGKLLLLPGGIAGAGPEVQHRQLLR
☑	694	825.78	824.77	824.40	0.38	1	2	2.7e+02	1	CFSNRAK
☑	824	877.67	2629.99	2630.36	-0.37	0	2	1.1e+03	1	LNPCMPVHHFGRPLLLASASPTTR + Oxidation (M)
☑	1048	956.37	2866.09	2866.44	-0.35	1	2	8.9e+02	1	TTAGILQYQGSITISGGCLSPMRGVGSR + Carbamidomethyl (C)
☑	2366	855.90	2564.67	2564.29	0.38	2	2	4.2e+02	1	SVNCMGGFKKFGFINNDYLIIK + Carbamidomethyl (C)
☑	492	726.80	2177.38	2176.94	0.44	1	2	9.1e+02	1	CVSPSPSSRCASNGACSPTPR + 2 Carbamidomethyl (C)
☑	206	571.30	1140.58	1140.64	-0.07	1	2	7.4e+02	1	MPAPVVTVRR + Oxidation (M)
☑	2200	1040.25	2078.49	2078.94	-0.45	0	2	4.7e+02	1	VVMSSELEQVFMSMYDGK
☑	2465	1043.88	3128.62	3128.68	-0.06	2	2	2.4e+02	1	CTQSKSRIHKPEPMLVEPGPVNQLLLSK

☑	462	710.38	2128.13	2127.99	0.14	1	2	9e+02	1	TSNLATGSEETMFEGRNLR + Oxidation (M)
☑	515	735.90	2204.69	2204.09	0.60	0	2	1.4e+03	1	MLRPFPHNQLLTMTMSGAK + 2 Oxidation (M)
☑	2340	816.83	2447.48	2447.16	0.32	2	2	2.7e+02	1	VRDNMHVALCMSPVGDQFRTR + Oxidation (M)
☑	371	655.50	654.50	654.33	0.16	0	2	1.1e+03	1	DAPQPK
☑	499	729.90	2186.68	2187.12	-0.44	2	2	1.5e+03	1	NETDAEPSPQVVVDYLKRK
☑	2157	993.66	1985.31	1985.86	-0.54	2	2	3.3e+02	1	ITLSNYCRDHKCDMCK + Carbamidomethyl (C)
☑	2194	1032.14	2062.26	2062.04	0.22	1	2	2.9e+02	1	LDTALNIAQTSGLCDKSQK + Carbamidomethyl (C)
☑	910	915.28	2742.81	2743.36	-0.55	1	2	1.2e+03	1	RLMEQLNEGSSSLPSSVLPCSSPLR + Carbamidomethyl (C)
☑	1918	506.63	1516.86	1516.84	0.02	1	2	3.2e+02	1	TLPPHVRGVTDPTK
☑	1413	1086.02	3255.04	3254.91	0.14	1	2	1.1e+03	1	KVFVPLKPCVEVGIGILIILIFVMISWR + Carbamidomethyl (C); Oxidation (M)
☑	2046	872.14	1742.26	1741.95	0.30	1	2	4.3e+02	1	HYGVSERAILPLIMK + Oxidation (M)
☑	536	746.61	1491.20	1490.87	0.33	1	2	1.4e+03	1	RQQLLLLESHVR
☑	1570	1149.89	2297.78	2298.17	-0.39	2	2	9.1e+02	1	ISSSDMIISRIRSSDMIISR + 2 Oxidation (M)
☑	529	743.20	2226.59	2227.12	-0.53	2	2	1.2e+03	1	RGAGAYNTVVEGNKYVSDVTK
☑	675	817.01	816.00	815.44	0.56	0	2	1.3e+03	1	LLDAQEK
☑	1940	517.70	1550.08	1549.83	0.25	1	2	3.5e+02	1	LISKMVTSQQMLR + Oxidation (M)
☑	73	474.59	947.16	947.58	-0.42	0	2	7.5e+02	1	ITAVILYR
☑	829	880.27	2637.79	2637.23	0.56	2	2	1.2e+03	1	SIMSTPCNSERAYDLPSPGDLRR + Carbamidomethyl (C); Oxidation (M)
☑	1505	1121.86	3362.56	3362.73	-0.17	1	2	1e+03	1	DAAMCVGDEILASARCIASVVTEWIVSSLLLK
☑	2238	720.34	2158.01	2158.08	-0.07	1	2	3.1e+02	1	LVLGSDSEDQEILDNQKQK
☑	2474	1058.48	3172.42	3172.61	-0.20	1	2	2.2e+02	1	STAAPTVLTGGTPTRADANTGAATLSLACTGVAR
☑	762	854.82	2561.44	2561.12	0.32	2	2	9.7e+02	1	SRANMFHCLACCVREQCSVYK + 2 Carbamidomethyl (C)
☑	903	913.18	2736.52	2736.12	0.41	0	2	1.1e+03	1	NGTGWYFFHCADVDECALNTSACR + Carbamidomethyl (C)
☑	1032	951.04	1900.06	1899.99	0.07	1	2	1.3e+03	1	MQDQIQKLLLMGPGGAGK + Oxidation (M)
☑	2461	1029.15	3084.44	3084.57	-0.13	0	2	2.4e+02	1	LGQHFLTLSESQLSTIFSEWEGGLHLR
☑	2490	1123.93	3368.76	3368.70	0.07	1	2	2.1e+02	1	EFIQLNPIWSNKFSQFNIFAPVVCWWR
☑	121	515.34	514.33	514.28	0.05	0	2	7e+02	1	DVPGK
☑	1076	965.54	1929.07	1928.97	0.10	2	2	8.9e+02	1	HQPPHRRATGATAASTDR
☑	1579	1153.51	3457.51	3457.86	-0.35	2	2	7.9e+02	1	VLAAMDSGDIVLVRASTQGIETLSSVRVGLTAGR + Oxidation (M)
☑	1792	1307.73	2613.44	2614.01	-0.57	0	2	8.3e+02	1	SCFCMTEPDVASSDATNMACSISR + Carbamidomethyl (C); 2 Oxidation (M)
☑	433	694.87	2081.57	2082.14	-0.57	1	2	1.5e+03	1	FSLEMSKIPSILLSIFNK + Oxidation (M)
☑	1616	1170.98	3509.92	3509.69	0.23	1	2	8.8e+02	1	YESGVIPYANMGYWDPDYVVKETDVLALFR
☑	1722	1238.29	2474.57	2474.29	0.28	2	2	8.4e+02	1	HMEQLKWRLAAHLTDEELVR
☑	2214	702.91	2105.71	2105.96	-0.25	1	2	3.6e+02	1	FEYLISCEEKGITSECAK + Carbamidomethyl (C)

☑	2471	1053.93	3158.78	3158.50	0.27	1	2	2.4e+02	1	ETVTAGMGEEFLTATTSTVGATTSNIDKNGR
☑	584	775.35	2323.03	2323.00	0.03	1	2	9.9e+02	1	CWFTTTSAKSCTGDGTAMVAR + 2 Carbamidomethyl (C); Oxidation (M)
☑	686	824.96	823.95	823.39	0.56	0	2	1.2e+03	1	TCSLACVK
☑	1049	956.69	2867.04	2867.48	-0.44	1	2	1.1e+03	1	MSGRLTIFNEPIAPWADAMVHSALLK
☑	1509	1123.92	3368.73	3368.59	0.13	2	2	9e+02	1	KLYVCMQKYGVIPSSTQSTSGSNMEALDATK + 2 Oxidation (M)
☑	1872	730.30	1458.59	1458.78	-0.18	1	2	3.1e+02	1	VDCKEIVVNALEK
☑	555	760.77	2279.29	2279.24	0.05	1	2	1.1e+03	1	LDNKLPPFELPLTENIDAIK
☑	1762	1273.48	2544.94	2545.25	-0.31	0	2	9.3e+02	1	FSGNEDAAAVCAALQEGVVPETVLR
☑	693	825.57	824.57	824.45	0.12	0	2	1.1e+03	1	TLICFTK
☑	761	854.71	1707.40	1707.76	-0.35	0	2	1.1e+03	1	LMDMAADNASNLAEAR + Oxidation (M)
☑	1999	835.36	1668.70	1668.90	-0.20	2	2	3.2e+02	1	AKNPKGMPSISSLAPR + Oxidation (M)
☑	270	609.14	1824.40	1824.02	0.39	1	2	1.2e+03	1	KLVTCVGNTIGIIPDPK + Carbamidomethyl (C)
☑	577	773.54	2317.61	2317.14	0.46	0	2	1.2e+03	1	LISCAEIYGDACHPGLLSSLAK + Carbamidomethyl (C)
☑	1457	1101.66	3301.97	3301.52	0.45	1	2	9.3e+02	1	RGASTGSVSVMCFGHPLLDMMATVEEEFLR + 2 Oxidation (M)
☑	1188	1002.04	3003.10	3003.45	-0.35	1	2	1.3e+03	1	TPPTNNKGIPHIIEHSCLSGSDHYTTK + Carbamidomethyl (C)
☑	2228	712.43	2134.28	2133.99	0.30	1	2	2.8e+02	1	TSPKMSCCDNTPLHISSIK + Carbamidomethyl (C); Oxidation (M)
☑	2322	1192.56	2383.11	2383.01	0.09	0	2	3e+02	1	YAAEACVMEDGGLLQDDPDLCR
☑	2341	821.62	2461.84	2462.28	-0.44	2	2	4.7e+02	1	DTGIGMTKADLINNLGTIARSGTK + Oxidation (M)
☑	392	669.89	668.89	669.36	-0.48	0	2	1e+03	1	GICHLK
☑	1095	973.21	2916.60	2916.36	0.24	1	2	1.2e+03	1	ETTEMQPNTPIFNSLSQCLQKNYSK + Oxidation (M)
☑	1333	1057.55	3169.63	3169.80	-0.17	2	2	8.6e+02	1	MTLLMVAEKPLLAESIANFLSLGPVKTRK
☑	1370	1067.94	3200.79	3200.60	0.19	1	2	9.5e+02	1	VAFMRLTGMHFGGEELTYVQDTLTSLLR + Oxidation (M)
☑	2357	844.08	2529.22	2529.29	-0.07	2	2	3e+02	1	LCLLWIESQKLRSTDPEADANK
☑	456	708.75	2123.24	2123.04	0.20	2	2	1e+03	1	RCGLEGPVGEFLRDFICR + Carbamidomethyl (C)
☑	823	439.34	876.66	876.45	0.21	1	2	4.1e+02	1	VCGLDRSK
☑	2173	676.07	2025.18	2025.09	0.10	2	2	2.9e+02	1	NRLADAPVQRQDNLFIR
☑	2422	950.34	2847.98	2848.28	-0.30	2	2	3.9e+02	1	HTSNNTSVNDTNTPTNKRTDNDTSSK
☑	402	676.06	2025.17	2025.03	0.14	2	2	1.2e+03	1	MVYKNISFIYKSEEFK
☑	1471	1109.83	3326.48	3326.54	-0.07	2	2	1e+03	1	HLADYVLVWSTRFGGMYGDDMAKSPHMAR + Oxidation (M)
☑	2171	1012.30	2022.58	2023.00	-0.42	2	2	4.8e+02	1	AARDWNFEYQRAVELR
☑	259	600.47	1198.93	1198.64	0.29	0	2	1.2e+03	1	MAPGGLNADLLK
☑	2365	855.42	2563.24	2563.33	-0.09	2	2	2.8e+02	1	ALLRSPANRLSDGGVGALCPHSSTK + Carbamidomethyl (C)
☑	1461	1103.68	2205.35	2204.91	0.44	0	2	9.9e+02	1	MPCAHDAAFCSAMPTHVSEK + Carbamidomethyl (C); Oxidation (M)
☑	661	813.16	2436.47	2436.15	0.32	1	2	1.1e+03	1	LDMCAGFLVSATLYSKDTCLNR + Oxidation (M)

☑	1084	484.72	967.42	967.50	-0.08	1	2	3e+02	1	QTTEKYAK
☑	2323	795.38	2383.12	2383.18	-0.06	1	2	3.1e+02	1	TTEATDLTLSYVRLLGCSGGGGGR
☑	1181	1000.14	999.14	998.59	0.55	1	2	1e+03	1	NGKKPQLSK
☑	1364	1066.60	2131.18	2131.26	-0.08	2	2	8.3e+02	1	SLITKLEQTLVRAMIFIR
☑	1739	1247.96	3740.86	3740.48	0.37	1	2	1e+03	1	CVACSDTTNGGHEGCSLCSNNNGFKCTDCKPNYK + 3 Carbamidomethyl (C)
☑	2315	1182.37	2362.73	2363.18	-0.45	2	2	5.3e+02	1	HGGVGSHRIRSASATTAAATGENR
☑	413	682.46	2044.36	2043.88	0.47	0	2	9.2e+02	1	SCYSGASGHGIPSTANGHER + Carbamidomethyl (C)
☑	520	738.16	2211.46	2211.06	0.40	2	2	1.3e+03	1	NGQKVNLMQRGSNTSFCVR + Carbamidomethyl (C); Oxidation (M)
☑	1207	1009.63	3025.88	3025.54	0.34	1	2	1.1e+03	1	TEYYEYIISYVDDLTIVSADTKAILK
☑	126	519.36	518.36	518.28	0.08	1	2	1.3e+02	1	SRIGS
☑	396	672.66	1343.31	1343.62	-0.31	0	2	1.2e+03	1	AGGTYCFLDAAQK
☑	476	718.56	717.55	717.32	0.24	0	2	1.4e+03	1	YFMNK + Oxidation (M)
☑	86	486.63	485.62	485.28	0.34	0	2	7e+02	1	VTPVA
☑	552	758.92	757.91	758.41	-0.50	2	2	1.6e+03	1	KRGDGAR
☑	1220	1014.66	3040.96	3040.41	0.55	2	2	1.1e+03	1	THVPMAGNIGEGDCSVYAVNRRGSQHNR + Oxidation (M)
☑	1399	1082.12	1081.11	1080.52	0.59	1	2	1.1e+03	1	TDASGNKFNK
☑	502	730.77	2189.30	2189.09	0.21	2	2	1.2e+03	1	TPDGGQIEIMACKDTGIIRR + Oxidation (M)
☑	1290	1042.11	3123.31	3123.56	-0.25	2	2	1.1e+03	1	FMGHRGPVLAVACSPRANLLASGGHDGYVR + Oxidation (M)
☑	1814	676.60	1351.19	1351.73	-0.55	0	2	6.1e+02	1	EINPIQTPELAK
☑	1553	1143.24	2284.47	2284.18	0.29	2	2	1.1e+03	1	MTDKQYTVVIEEPTAKLYR
☑	1014	944.48	943.48	943.46	0.02	1	2	4e+02	1	DRLYFCK
☑	1475	1111.27	1110.27	1110.59	-0.32	2	2	1.2e+03	1	FIKDAMSKR + Oxidation (M)
☑	1680	1209.74	2417.47	2417.21	0.26	0	2	9.7e+02	1	FTPVEYTDDVPTAAVNPLEALR
☑	135	527.24	1578.70	1578.90	-0.20	0	2	6.6e+02	1	VKPLQPEQIVATEK
☑	435	695.92	2084.75	2084.16	0.60	2	2	1.2e+03	1	KLNWMINVTEDLLLRAR
☑	2395	899.55	2695.64	2695.38	0.26	2	2	3.2e+02	1	ELVDLLSEYYMLLPQRVRDGMR
☑	2428	961.38	2881.11	2881.56	-0.45	2	2	3.1e+02	1	VGNVLSKMNPTTNPENIIKVAELSLGK + Oxidation (M)
☑	582	774.51	1547.01	1546.75	0.26	0	2	1.2e+03	1	FDSSPPSSSPPITTK
☑	1562	1147.21	3438.60	3438.82	-0.22	2	2	1.1e+03	1	FSQVLHVLEPSKEAIAAVVSVAEKTGDLGAMSR
☑	1354	1064.45	3190.32	3190.62	-0.29	2	2	1e+03	1	LAFYVTCMLPSTLMLDREFCLLIRGR + 2 Carbamidomethyl (C); Oxidation (M)
☑	1487	1116.04	3345.08	3345.66	-0.58	0	2	1.3e+03	1	DHEQLLNLGLEAQMTELQQSRPTPQTQPK + Oxidation (M)
☑	2470	1052.09	3153.23	3153.73	-0.49	1	2	3.1e+02	1	VLHHAGLIHGSPLPLSLFLCGSAPSSLRFK
☑	971	933.25	2796.72	2796.31	0.42	1	2	1.3e+03	1	LVVCAQGGSVEAMGQDNSFENIEQKK + Oxidation (M)
☑	1965	536.74	1607.19	1606.76	0.43	1	2	5.1e+02	1	DVERDIETAVASMR + Oxidation (M)

☑	2199	1037.53	2073.04	2073.01	0.03	1	2	3.2e+02	1	GTQHVLCTLMLNSDQRGK + Carbamidomethyl (C); Oxidation (M)
☑	156	537.62	1609.84	1609.75	0.09	0	2	8.6e+02	1	IEWCMQTVLDTAK + Carbamidomethyl (C); Oxidation (M)
☑	1116	980.84	2939.50	2939.61	-0.11	2	2	9.9e+02	1	ILLADLAGSERIKIAQTETGIPFEQAR
☑	786	863.50	2587.48	2587.37	0.12	1	2	1e+03	1	RGGAEPLEVLQGCLQAQVISLHGR + Carbamidomethyl (C)
☑	1054	957.24	1912.46	1912.85	-0.39	1	2	1.3e+03	1	APTTDRCVCCLAGTIDMK + Oxidation (M)
☑	1114	490.61	979.20	979.56	-0.35	0	1	3.3e+02	1	HTLVNQIR
☑	781	860.98	1719.95	1719.93	0.01	1	1	1.6e+03	1	IWESLVYIYAHAKK
☑	1325	1055.05	3162.11	3161.55	0.56	1	1	1.3e+03	1	NGVEKVVISAPSSDAPMFVMGVNHELYQK + Oxidation (M)
☑	1777	644.88	1287.75	1287.71	0.04	2	1	3.8e+02	1	KITKMVPYHR + Oxidation (M)
☑	2255	1106.32	2210.63	2211.16	-0.53	1	1	6.8e+02	1	VACQRLGLPCTGNKPDLLTR + Carbamidomethyl (C)
☑	241	593.61	592.60	592.36	0.24	0	1	2.4e+02	1	IIGYK
☑	1417	543.84	1085.66	1085.54	0.13	0	1	3.8e+02	1	SLDPGNGADIK
☑	1854	718.02	1434.03	1434.58	-0.55	0	1	4.1e+02	1	YCQGVEEEFMK + Carbamidomethyl (C); Oxidation (M)
☑	697	827.83	2480.47	2480.20	0.27	2	1	1.2e+03	1	TMLKKLQGEYYEIQIDCYDK
☑	817	876.99	2627.94	2628.32	-0.38	0	1	1.5e+03	1	HHFVLSEYTLACHMLHVILHGR + Oxidation (M)
☑	1367	1067.14	2132.27	2132.04	0.23	1	1	1.1e+03	1	MKGELNFVGITMEFEMLK + Oxidation (M)
☑	1956	791.19	1580.36	1580.90	-0.54	0	1	6e+02	1	RPQNSGAGGAIILTVK
☑	2347	828.92	2483.75	2484.23	-0.47	2	1	5e+02	1	SGEVKSSSICRIDNLNAAYGVSSK
☑	1178	999.00	2993.98	2994.46	-0.48	2	1	1.5e+03	1	ISVVEGMVLDQGYLSRYFTTDDKNTK + Oxidation (M)
☑	1308	1047.67	2093.34	2093.13	0.21	2	1	1.1e+03	1	MVGVSRGDAQRGLEPIRPR
☑	2010	560.09	1677.25	1677.69	-0.44	0	1	5.1e+02	1	VFMSLAMAEEMEDK + 3 Oxidation (M)
☑	1139	988.83	987.82	987.51	0.31	1	1	1.1e+03	1	LESEARQR
☑	1201	1006.84	3017.49	3017.51	-0.01	2	1	1.1e+03	1	LINDGNVERLIPIVMRACSSDDPYVR + Carbamidomethyl (C); Oxidation (M)
☑	2413	932.51	2794.52	2794.33	0.19	2	1	2.6e+02	1	FSPFTNCCVYAGISKGKISISDTDK + 2 Carbamidomethyl (C)
☑	1659	1194.49	3580.46	3580.66	-0.20	2	1	9.3e+02	1	MMTGRVLLVCALCVLWCGAGGGYAWPYKECK + 3 Carbamidomethyl (C); 2 Oxidation
☑	1752	1260.89	3779.65	3779.70	-0.05	2	1	1.2e+03	1	CGAVFVGTWKDNRIVGQGEFYLSENCDLNNPDY + Carbamidomethyl (C)
☑	1769	1278.92	1277.91	1277.73	0.18	2	1	3.7e+02	1	MFRTGVQLAKK
☑	441	699.02	2094.05	2094.09	-0.04	1	1	9.3e+02	1	RLMEEHLTQGAHVPHALR
☑	1119	981.73	2942.16	2942.31	-0.15	2	1	1.2e+03	1	MLARQMGMMIQQVTVPCDACGGEGRR + Carbamidomethyl (C); 3 Oxidation (M)
☑	1128	985.44	2953.30	2953.39	-0.09	1	1	9.2e+02	1	LRNQEFKPQMINQWCSSIVEDCTK + Carbamidomethyl (C)
☑	1244	1023.28	3066.81	3066.73	0.08	1	1	1.2e+03	1	RNVIHLLPVLAQQLYVLAVYLSNTSSR
☑	777	858.75	857.74	857.47	0.27	1	1	4.4e+02	1	APSKAASAR
☑	2005	838.05	1674.08	1673.93	0.15	2	1	3.6e+02	1	VSAKASVAADAAKAVSTK
☑	2384	889.83	2666.47	2666.51	-0.04	2	1	3e+02	1	NLANSALTATKQIAQISTPKITLNR

☑	263	602.99	1805.95	1805.98	-0.03	2	1	1.3e+03	1	AHQLARLLQERNAMR
☑	1484	1114.96	2227.92	2228.09	-0.18	1	1	1e+03	1	EINDKVAEAEQAEIEINASR
☑	1214	1013.13	3036.38	3036.37	0.01	1	1	1.1e+03	1	DEANTLHSVDSARMDGQVETSVFAAAGDK + Oxidation (M)
☑	1369	1067.85	3200.52	3200.69	-0.17	1	1	1e+03	1	KLLLPQTIQTISECSFSMTTLVNFVFPK + Oxidation (M)
☑	1712	1229.89	3686.65	3686.87	-0.22	2	1	1.1e+03	1	GVQELLLKAQAQYMAENGGVEQDVRHMIGLFSR
☑	307	628.50	1882.47	1882.89	-0.42	2	1	1.2e+03	1	AMVLSGGSGCTLSRCRR + 2 Carbamidomethyl (C); Oxidation (M)
☑	655	808.97	1615.92	1615.61	0.31	0	1	1.3e+03	1	CPACGCQTCCLACVK + 2 Carbamidomethyl (C)
☑	283	614.14	1839.39	1838.91	0.48	1	1	1.3e+03	1	MDLVSQIMEKSTLNSK + Oxidation (M)
☑	843	887.45	2659.34	2659.16	0.18	1	1	1.2e+03	1	WCQRFEAEVCDVNTAQWAFDK + 2 Carbamidomethyl (C)
☑	1182	1000.86	1999.70	1999.97	-0.28	2	1	1.2e+03	1	CPRNSDELANASPETKLR
☑	2423	950.52	2848.54	2848.44	0.10	2	1	2.8e+02	1	ICQSMNEPMAINHPKTITLHEKLK + Carbamidomethyl (C); Oxidation (M)
☑	618	792.50	2374.47	2374.04	0.43	1	1	1.1e+03	1	ASVSAASPEDDSGPAGGSDSGAGRGGR
☑	1740	1249.77	3746.29	3745.76	0.52	2	1	1.1e+03	1	ATSLLMEYCQASALSFAKGGSEVAMTQKIDSYSSR + Oxidation (M)
☑	82	483.70	1448.08	1447.73	0.35	0	1	8.1e+02	1	MYLTTSLFESIK + Oxidation (M)
☑	600	784.38	783.37	783.49	-0.12	0	1	2.3e+02	1	KPLIDAK
☑	738	842.96	2525.85	2526.14	-0.29	0	1	1.5e+03	1	CSLCVVPEGAATPICLEYDNTSK + 2 Carbamidomethyl (C)
☑	865	896.49	895.48	895.49	-0.02	1	1	1e+03	1	MSYVLKR
☑	1281	1038.71	3113.11	3112.66	0.45	2	1	1.2e+03	1	QGPIVCNLCTGLINTLENLLTTKGADKVK + Carbamidomethyl (C)
☑	1312	1049.71	2097.40	2097.06	0.34	2	1	1.2e+03	1	VMLAAECEARNLLHMPKR + Oxidation (M)
☑	1395	1080.67	3238.99	3238.65	0.34	0	1	1e+03	1	TCLVYCDGGVVAATSGAAAAAAVCVVVLLMVGLAT
☑	778	859.42	2575.24	2575.30	-0.06	1	1	1.1e+03	1	GSVVHICALSSSLEKTISQAMVDGK + Oxidation (M)
☑	1716	1234.83	2467.65	2467.10	0.55	0	1	1.2e+03	1	HDAHDGNGGAAGLLCGESLVCSAASR
☑	797	871.46	2611.36	2611.44	-0.08	1	1	9.7e+02	1	HMEGSVIGRETEIQLSIVLLLFK
☑	1931	769.48	1536.94	1536.78	0.16	1	1	4.1e+02	1	TRYTTAVQHSAFR
☑	310	629.96	1886.84	1887.03	-0.19	1	1	1.6e+03	1	KLALGGEPITMQVFLDR
☑	1397	1081.02	3240.05	3240.62	-0.58	2	1	1.4e+03	1	EISFVICTGDMQTLRCESDLVYLKVPPK + Carbamidomethyl (C)
☑	1300	1045.42	3133.24	3133.64	-0.41	1	1	1.1e+03	1	AAGDLVAALMYVADVAMLKATHIISAPHER
☑	1717	1234.92	2467.83	2467.43	0.40	1	1	1.1e+03	1	TGAVLPRAILAWLQSLQLGSFVK
☑	132	524.53	1570.58	1570.77	-0.18	2	1	1.2e+03	1	IADSMEKFETKTR + Oxidation (M)
☑	1551	1141.35	3421.02	3421.30	-0.28	0	1	1.4e+03	1	GCNHMTCANCGYQFCWLCMQEYTSSHFK + 2 Carbamidomethyl (C); Oxidation (M)
☑	1329	1056.66	3166.96	3166.67	0.28	1	1	1.1e+03	1	MRVGGPASSLAGCVPALQSMVLVYYVTILR + Oxidation (M)
☑	1742	626.29	1250.56	1250.71	-0.15	2	1	3.4e+02	1	IHSIKGSPEKR
☑	2225	710.17	2127.50	2127.10	0.40	2	1	6.1e+02	1	IIEQLNNEAKTQTRNNNK
☑	754	850.80	849.79	850.32	-0.53	0	1	4.5e+02	1	ANDESCR + Carbamidomethyl (C)

☑	2482	1078.05	3231.14	3231.47	-0.33	2	1	4.4e+02	1	VVCCEDVGERERCAALTIACSILCDPHR + 3 Carbamidomethyl (C)
☑	2052	588.71	1763.10	1762.96	0.14	1	1	4e+02	1	AALNYSITGASGKAQALK
☑	2320	794.47	2380.40	2380.15	0.25	0	1	3.2e+02	1	YLNIGCNDLGNGCSLALSDIIR + Carbamidomethyl (C)
☑	986	936.55	935.54	935.49	0.05	1	1	1e+03	1	LRELMMK + Oxidation (M)
☑	1666	1199.61	3595.81	3595.77	0.03	2	1	9.9e+02	1	AMHHILGSSRASLLRGALTDLGVCSPTDDTTSEK + Carbamidomethyl (C)
☑	1748	628.44	1254.86	1254.72	0.15	1	1	3.5e+02	1	KIINDSDLLPK
☑	1072	963.23	962.22	962.47	-0.25	0	1	1.4e+03	1	EINMSLEK
☑	1365	1066.99	3197.96	3197.55	0.41	2	1	1.3e+03	1	MYSQTHRLSMESKASASAVPLAASTMPVAK + 3 Oxidation (M)
☑	1522	1126.52	3376.54	3376.71	-0.18	0	1	9.8e+02	1	VCVMMPMMLLLVLSLLAFVSSSYVHVQYR + 3 Oxidation (M)
☑	2049	877.20	1752.38	1752.81	-0.44	1	1	6.2e+02	1	ENIHDMFVENKYAK + Oxidation (M)
☑	801	871.94	870.93	871.50	-0.57	2	1	7.3e+02	1	RAEAAARK
☑	1384	1077.42	3229.24	3228.66	0.58	2	1	1.2e+03	1	ILISHGADINALNENDYTPLDFAIMRGKK
☑	1513	1124.90	1123.90	1123.62	0.27	0	1	1e+03	1	ELGLHIDLSK
☑	1766	1275.65	2549.29	2549.36	-0.07	2	1	9.2e+02	1	AATVTAAKAVPPLQGTASPKQNSADR
☑	1933	514.86	1541.55	1541.69	-0.15	0	1	3.7e+02	1	FTYCLPYYTPMK + Oxidation (M)
☑	2051	586.10	1755.27	1754.76	0.50	0	1	4.7e+02	1	NNFELFGDTENHYR
☑	2134	643.36	1927.05	1926.87	0.18	0	1	3.4e+02	1	MPLYDISNEDVTEMVR + Oxidation (M)
☑	916	916.68	2747.03	2747.10	-0.07	1	1	1.4e+03	1	RDADGGGNGTSSYCADCTPCAFPPETR
☑	1500	1119.24	3354.71	3354.54	0.16	1	1	1.2e+03	1	MPHPSCLPPHGGDEGGGHSTPAWMRSLLSVR + Carbamidomethyl (C); 2 Oxidation (M
☑	2479	1072.20	3213.57	3213.55	0.02	1	1	2.5e+02	1	EMNHIGGIMADHLGMGKTVQMIGLCLVSDK + Oxidation (M)
☑	56	453.91	905.80	905.44	0.36	0	1	8.4e+02	1	TSALQQCR
☑	1094	973.11	2916.30	2916.37	-0.07	2	1	1.4e+03	1	FNAESSKSIIHDNEKIDNQENIDEK
☑	1648	1188.47	3562.39	3562.60	-0.20	2	1	1.1e+03	1	MGMCTDRCVGVCKLNLALMHFVGMGEVCVWR + Carbamidomethyl (C); 3 Oxidation (M
☑	599	784.28	2349.81	2349.21	0.60	0	1	1e+03	1	TVADGGALSLMAGTAMTTAAWLLK
☑	479	360.69	719.36	719.38	-0.03	0	1	4.2e+02	1	MLGMLR
☑	2036	573.59	1717.74	1717.91	-0.18	1	1	3.6e+02	1	SLSQLVTVNVSKANCR
☑	2488	1105.86	3314.56	3314.74	-0.18	1	1	2.5e+02	1	DTNLQILIEAHCIDEFISYVPVVVGLKMR
☑	934	924.27	2769.78	2770.06	-0.28	1	1	1.4e+03	1	TCATFNNSKDCLDMPGCGYCMHTDK + Oxidation (M)
☑	1148	990.71	2969.10	2969.34	-0.24	1	1	1.5e+03	1	YNSDISSCNYVARSYGIHAGMYVNSAK
☑	1846	473.23	1416.66	1416.74	-0.09	2	1	4.5e+02	1	RSVSLTEQERGR
☑	98	502.56	1504.66	1504.74	-0.08	2	1	1.3e+03	1	QESLRRMCGVGGR + Carbamidomethyl (C)
☑	1734	622.59	1243.17	1242.57	0.60	1	1	7.7e+02	1	REWMTFSTSV
☑	1634	1179.55	3535.63	3535.44	0.18	2	1	9.7e+02	1	TMSGSCGCRCAECGYGRDCLPVYLPHVDGCNR + 2 Carbamidomethyl (C)
☑	1636	1181.14	3540.39	3539.98	0.42	2	1	1.3e+03	1	HQLTLLSVCLIAEQLLSRFEVIHSKSIIHR + Carbamidomethyl (C)

☑	516	736.98	2207.91	2208.03	-0.12	1	1	1.4e+03	1	RPSMRGDGAQQPCSHAPSAAK + Carbamidomethyl (C)
☑	1360	1065.08	3192.22	3192.61	-0.39	2	1	1.6e+03	1	RPLSASRASARASAASRPQPHTTVSSDDASR
☑	820	877.33	2628.96	2629.33	-0.37	0	1	1.2e+03	1	LISTYVGHLPMNDFPIFFDIFK + Oxidation (M)
☑	1685	1211.03	2420.05	2420.05	-0.01	2	1	1.1e+03	1	RVMVEQVICTDMYCREGCR + 2 Carbamidomethyl (C); Oxidation (M)
☑	2217	705.16	2112.45	2112.13	0.32	2	1	5.9e+02	1	RVALTGKLCIGDQEHLFR + Carbamidomethyl (C)
☑	136	527.62	526.61	527.20	-0.59	0	1	37	1	DHGAE
☑	685	824.90	823.90	824.39	-0.49	0	1	1.6e+03	1	FLSDMGR
☑	821	877.33	876.33	876.45	-0.12	0	1	1.2e+03	1	VFPSNWK
☑	1528	1128.67	3382.98	3383.45	-0.46	1	1	1.1e+03	1	AQQHYGCKTQAFMELEDTGDIDDASSHWK + Carbamidomethyl (C); Oxidation (M)
☑	689	825.37	824.36	824.49	-0.13	0	1	1e+03	1	GIGGVLPGR
☑	1450	1099.05	3294.13	3293.66	0.46	1	1	1.5e+03	1	CPFGVFLSTATQCLFSMLRYQTDLVGIVK + Carbamidomethyl (C)
☑	1545	1138.73	3413.16	3412.65	0.51	1	1	1.2e+03	1	ISEDCYLLICKNEHQMYSLLLNELQSWK
☑	1564	1147.39	3439.16	3439.55	-0.38	2	1	1.3e+03	1	SSAATGEVKGHYMNVTAATMEQMYERAEFAK + 2 Oxidation (M)
☑	1275	1035.88	3104.63	3105.20	-0.57	0	1	1.1e+03	1	AMDDYCSEGGCIASEVLGACFSQMSGGCNK + Carbamidomethyl (C); Oxidation (M)
☑	1293	1043.27	2084.52	2084.09	0.43	0	1	1.4e+03	1	VATAVVELLDGDTQAWAVAR
☑	2261	747.80	2240.39	2240.07	0.32	0	1	4.3e+02	1	IEGELESLQECEANLHLTR + Carbamidomethyl (C)
☑	458	708.83	707.83	707.38	0.45	0	1	6e+02	1	IWYAR
☑	1693	1216.53	3646.58	3646.90	-0.32	2	1	9.9e+02	1	EDNSPVLENYSVIFLITTSIGERHEGIRAFLK
☑	2220	1059.94	2117.86	2118.03	-0.17	2	1	3.5e+02	1	ADRRWCNVAQDPLHGGPAR
☑	2493	1139.59	3415.75	3415.63	0.12	1	1	2.8e+02	1	ASALSGAATGTEKAIPANVDDGCDFGTITVYVCAK
☑	1401	1082.45	3244.33	3244.59	-0.26	0	1	1e+03	1	EIVGLPSDGIACNGKPFVMCLINLDPSSNK + 2 Carbamidomethyl (C)
☑	1571	1150.46	2298.90	2299.11	-0.21	0	1	1.1e+03	1	AAEVAATLDIGSQLAEAAPCADR + Carbamidomethyl (C)
☑	2059	592.73	1775.17	1774.98	0.19	2	1	4e+02	1	QRRLLASLDLPDNHK
☑	771	857.62	2569.85	2570.21	-0.36	0	1	1.4e+03	1	MMGIYNENNQISSIVLSLNNER + 2 Oxidation (M)
☑	1064	961.39	2881.16	2881.26	-0.10	2	1	1.1e+03	1	QMGMMIQQVTVPCDACGGEGRRMDPR + Oxidation (M)
☑	1730	1241.53	3721.57	3721.66	-0.09	2	1	9.7e+02	1	LCSGFGACEKTGDTYGCLCASYYILVDKDCIPTR + Carbamidomethyl (C)
☑	1982	543.57	1627.69	1627.77	-0.08	0	1	4.2e+02	1	YDMINNAVDYALAR
☑	1111	978.66	2932.96	2932.38	0.58	1	1	1.4e+03	1	NNFRDELEDHLLAHVAGHCYNNPVR
☑	645	805.57	804.56	804.38	0.18	0	1	1.4e+03	1	GAGGPTSMK
☑	802	872.51	1743.02	1742.68	0.33	1	1	1.2e+03	1	MRCQETSAEGDEESR + Oxidation (M)
☑	1151	991.57	1981.13	1980.97	0.16	1	1	1.1e+03	1	ISESIWGQNCATKFLER
☑	1707	1227.52	3679.53	3678.97	0.56	2	1	9.8e+02	1	VTRSFAMLMVCIGILSMVLVFIKTATYQVTATR + Oxidation (M)
☑	2401	914.26	2739.76	2739.35	0.40	1	1	4.5e+02	1	QESLSRMTLPPMTLTIEEMLEFK + Oxidation (M)
☑	728	840.15	2517.43	2517.29	0.15	1	1	1.3e+03	1	QRGSGSILAVSCISSAAADAGAGEVLK

☑	973	933.36	2797.07	2797.40	-0.33	1	1	1.1e+03	1	KSSHQAVITCIVFDATGSLFATTSADK
☑	1492	1117.67	2233.33	2233.21	0.11	2	1	1.1e+03	1	DVNVVFPKGKLTMVIGATGSGK + Oxidation (M)
☑	1524	1127.60	2253.18	2253.14	0.03	2	1	1e+03	1	RRPGGGMLPREEYSGEPPLR
☑	782	861.27	2580.79	2580.26	0.52	2	1	1.6e+03	1	SFPEYHLAYLNLTDNRNKEEK
☑	1091	971.65	2911.93	2911.44	0.50	0	1	1.3e+03	1	CGLSLDDQGALLTCSAVLHGVASRPMAAV + Carbamidomethyl (C)
☑	896	908.05	2721.13	2721.43	-0.29	1	1	1.4e+03	1	GFFPHISEADIQRHVIFELPLEK
☑	1419	1087.48	3259.43	3259.65	-0.23	1	1	1e+03	1	QGTVVFLDDLLRETEFHMSQVLSLNPQK + Oxidation (M)
☑	2179	1020.02	2038.03	2038.02	0.00	1	1	3.7e+02	1	HPSDVGEAPFETLVRAASR
☑	1313	1049.77	3146.28	3146.60	-0.32	2	1	1.4e+03	1	LGDTAPRWDIPVPSGYSFASVDLEAIKSR
☑	1387	1078.08	3231.23	3231.51	-0.28	2	1	1.6e+03	1	RVNAAKMIVSAAEQCAPAMPGAGLGSMHMER + 3 Oxidation (M)
☑	1478	1112.28	3333.82	3333.65	0.17	2	1	1.3e+03	1	CMVVLQHGSHGTHLDLACLSQYLKAKDPR + 2 Carbamidomethyl (C)
☑	2271	1127.47	2252.92	2253.12	-0.20	1	1	3.8e+02	1	VQWHLNGKHCCAPLAGHLR + 2 Carbamidomethyl (C)
☑	452	705.39	704.39	704.35	0.04	1	1	1.3e+03	1	DERASK
☑	195	565.78	564.77	565.22	-0.45	0	1	2.9e+02	1	GMDIM
☑	774	858.03	2571.08	2571.22	-0.14	2	1	1.5e+03	1	EKMRNCDIGFMVVSDPMIEITK + Oxidation (M)
☑	1336	1059.17	3174.49	3174.57	-0.08	2	1	1.4e+03	1	NYYAMWQLATSLNKARWTMLDVISQR + Oxidation (M)
☑	1548	1139.71	1138.70	1138.50	0.20	2	1	1.2e+03	1	CRSSCGVRGAS + Carbamidomethyl (C)
☑	2497	1201.20	3600.57	3600.65	-0.08	1	1	2.8e+02	1	QQMEFYDEENPPPPPPPMPLEEIIKMDVMK + 2 Oxidation (M)
☑	1415	1086.26	1085.26	1085.57	-0.31	1	1	1.3e+03	1	MPPTSEIRR
☑	615	395.64	789.28	789.42	-0.15	1	1	5.1e+02	1	KEIGESK
☑	657	810.54	2428.61	2429.08	-0.47	2	1	1.3e+03	1	LTEMYMRKDQEVLGCAGHAFD + Oxidation (M)
☑	957	930.81	929.81	929.52	0.28	1	1	5.6e+02	1	LAAMRVGGR
☑	2272	751.98	2252.93	2253.02	-0.09	2	1	3.8e+02	1	MEPFVMHVECRTMESAKR + Carbamidomethyl (C); Oxidation (M)
☑	977	933.71	2798.10	2798.33	-0.23	1	1	1.5e+03	1	TTISSCTDDQQISTLSEDNFQKIPK
☑	2044	869.85	1737.68	1737.89	-0.21	2	1	3.6e+02	1	NSAKLSASNESIKIYN
☑	2447	985.31	2952.90	2953.45	-0.55	0	1	4.7e+02	1	GVLVQPSVANTFQWQVTMMPNNGIYK + 2 Oxidation (M)
☑	544	751.07	2250.19	2250.13	0.06	1	1	1.3e+03	1	LNGYDSNIVGIEMKIGALGDR + Oxidation (M)
☑	1351	1062.39	3184.16	3184.49	-0.33	1	1	1.3e+03	1	MASLAGHPMSALPLASLHSACQMCWKGWK + Carbamidomethyl (C); Oxidation (M)
☑	1573	1151.80	3452.38	3452.82	-0.43	2	1	1.4e+03	1	GGVTLCIYSLMCWATFLICFMKIIVSLVKK + Carbamidomethyl (C); Oxidation (M)
☑	1670	1200.51	3598.50	3598.85	-0.35	1	1	1e+03	1	MARPSLPLASSSCAPLPPSPSLSILRCHLGQPSR + Carbamidomethyl (C); Oxidation (
☑	2435	969.62	2905.83	2905.47	0.36	2	1	5.3e+02	1	NLKGKVVDDVELPNWCISSTDFIWK
☑	128	522.85	1043.69	1043.54	0.14	0	1	9.3e+02	1	SAAATAPCKPK
☑	722	838.35	2512.03	2512.23	-0.20	2	1	1.2e+03	1	EAVERGRLPDATQTVFDNGYFK
☑	814	874.30	2619.88	2620.24	-0.36	2	1	1.6e+03	1	MKANGFSDAQIAKYFLCTADDVR + Carbamidomethyl (C)

☑	1630	1176.49	2350.98	2351.37	-0.39	2	1	1.1e+03	1	ELISIFLSILKQIGEHRLSR
☑	2463	1038.14	3111.40	3111.60	-0.20	1	1	2.9e+02	1	LLLSQHTRWLITALETAAMSDEQESIR
☑	1409	1085.52	2169.03	2168.93	0.10	0	1	1.1e+03	1	NGNSLLEICCYYGSAQCFK + Carbamidomethyl (C)
☑	1298	1045.17	3132.49	3132.55	-0.06	1	1	1.3e+03	1	NGDVELVGHCFTSLSLIFENLDIEKQR + Carbamidomethyl (C)
☑	2175	1015.71	2029.41	2028.89	0.52	0	1	5.6e+02	1	AGYYLQSTSCIACHPACK + 2 Carbamidomethyl (C)
☑	150	533.73	1065.44	1065.53	-0.09	1	1	1.2e+03	1	ECVKTSFPR
☑	2180	1024.06	2046.11	2045.98	0.13	1	1	3.6e+02	1	LGGNRAELPSEPFQCCLR + Carbamidomethyl (C)
☑	2445	984.56	2950.66	2950.34	0.31	2	1	3.9e+02	1	VTMGQWAGDDGEGEAFGAPLRRCTPSSR
☑	365	650.66	1299.31	1298.72	0.59	1	1	1.6e+03	1	DGLRQLLLDEK
☑	1331	1057.20	3168.58	3168.46	0.11	2	1	1.3e+03	1	MSHDPSPKQTLDDSRVGTFDCSYLLQR + Carbamidomethyl (C); Oxidation (M)
☑	543	750.54	1499.07	1498.69	0.38	0	1	1.5e+03	1	DMLQGAGAGDFVFR + Oxidation (M)
☑	1721	1238.12	2474.22	2474.00	0.22	1	1	1.4e+03	1	IEDVEMCLCDARNEQCGMQAK + Carbamidomethyl (C); 2 Oxidation (M)
☑	1973	810.12	1618.22	1618.77	-0.55	1	1	5.8e+02	1	AMRGGSHAGVYPAMAK + Oxidation (M)
☑	652	808.53	1615.04	1614.82	0.22	2	1	1.3e+03	1	GEKDVGGASTKGVPEGK
☑	2489	1123.63	3367.88	3367.71	0.17	2	1	3e+02	1	DIAIQCLATNPESRPNAGEIKKMILEEAEK + Carbamidomethyl (C)
☑	1002	940.04	2817.11	2817.51	-0.40	0	1	1.4e+03	1	QLGTVIIMIDLVIGSTAIQSMGIWAR + 2 Oxidation (M)
☑	2048	876.45	1750.88	1751.01	-0.13	2	1	3.8e+02	1	KEHKAYAVAVLGPQLK
☑	2279	1132.84	2263.67	2264.13	-0.46	1	1	6.6e+02	1	NSVLETNGKMSTQEAIASSAVK
☑	605	786.26	2355.77	2356.25	-0.49	2	1	1.4e+03	1	TTMGLPARQLTEQEVAATKGVR
☑	1204	1008.36	3022.05	3022.45	-0.40	1	1	1.5e+03	1	FIQINPVPHCCLCQKDFTSTVSLMK + 3 Carbamidomethyl (C)
☑	1255	514.72	1027.42	1027.47	-0.05	1	1	3.8e+02	1	HVCPECGKR
☑	918	916.90	915.89	915.48	0.42	0	1	7.5e+02	1	SSASAALPGR
☑	2206	1048.05	2094.09	2093.93	0.16	0	1	3.5e+02	1	MEDGVELCAVGLVSCFHGR + Carbamidomethyl (C); Oxidation (M)
☑	2269	1126.78	2251.55	2251.13	0.41	2	1	6.1e+02	1	KMALAAMEAMSMALNGNTKLR
☑	1019	946.30	1890.59	1890.87	-0.29	1	1	1.7e+03	1	EIKELCSTPTQSGGDGGR + Carbamidomethyl (C)
☑	1055	957.36	2869.05	2868.45	0.60	1	1	1.3e+03	1	IVNFEFPSGITKVPSSFFSDCHLLK + Carbamidomethyl (C)
☑	1347	1061.67	3182.00	3182.46	-0.46	2	1	1.3e+03	1	TTVNEGQLGGSSTSDSHPPAADRGSREQEGR
☑	1942	777.75	1553.49	1553.79	-0.30	1	1	4.2e+02	1	EARGMFEYTPLIK
☑	119	514.35	1540.02	1539.74	0.27	2	1	8.4e+02	1	KTRTWTGYEDQR
☑	1601	1164.45	3490.33	3490.78	-0.45	1	1	1.3e+03	1	SLLADLETITGPSTAQVKCQQFTPGSCSIAVLR + Carbamidomethyl (C)
☑	1156	992.16	1982.30	1981.96	0.34	2	1	1.5e+03	1	RCVVASSEGNKTAMISEK + Carbamidomethyl (C); Oxidation (M)
☑	2062	894.47	1786.92	1786.81	0.11	1	1	4.6e+02	1	EEGKNWDQCLAAPNR + Carbamidomethyl (C)
☑	1445	1097.96	3290.85	3290.69	0.15	1	1	1.2e+03	1	VFLFLLDIPPVRSPDCGEEAHVPGIGAVMR + Carbamidomethyl (C)
☑	1750	1256.19	3765.56	3765.92	-0.36	2	1	1.4e+03	1	GAVILLDSTTETPELIWNSEMKNDVGIFIGKMTK + Oxidation (M)

☑	146	532.40	1594.17	1593.87	0.30	0	1	1.2e+03	1	EVATQILEHISNIK
☑	1120	491.84	981.67	981.46	0.20	1	1	3.3e+02	1	SGASRDYAR
☑	1129	987.08	2958.23	2958.39	-0.16	2	1	1.6e+03	1	VIHKKDNQYFEFQNSSNFGMPICGR
☑	1191	1002.79	3005.34	3005.56	-0.21	2	1	1.5e+03	1	VLDGSAEEMGTLTPSPELLLRYAKMLR + Oxidation (M)
☑	1585	1156.27	3465.79	3465.37	0.43	0	1	1.3e+03	1	NPYPSDDPECGGIACMLCCSCYWSTHLTR + 4 Carbamidomethyl (C); Oxidation (M)
☑	173	552.46	1654.36	1654.74	-0.39	1	1	1.3e+03	1	DGVCTACKADNGLFK + 2 Carbamidomethyl (C)
☑	248	598.04	1791.11	1790.76	0.35	0	1	1.4e+03	1	ASFNADDSLLFTCCER
☑	1377	1074.59	2147.16	2147.10	0.06	1	1	1.2e+03	1	SEGIINDRSTGVFPFPGVQK
☑	1682	1210.39	3628.16	3627.64	0.51	0	1	1.3e+03	1	IQCNQYSQDMIQAINCDINIFNYLQYGQSK + Carbamidomethyl (C); Oxidation (M)
☑	783	861.43	860.42	860.49	-0.07	1	1	5.5e+02	1	YVPRAQK
☑	2486	1093.06	3276.15	3275.61	0.54	2	1	4.8e+02	1	FGTLVYVGESDGEVVLRWRSAYACPLCTK + Carbamidomethyl (C)
☑	352	647.40	1939.17	1938.99	0.17	2	1	1.3e+03	1	ACGPLEPEPLARSKASASR
☑	836	883.92	882.91	883.50	-0.59	1	1	1.5e+03	1	HKLAYPR
☑	895	907.82	2720.44	2720.41	0.03	1	1	1.3e+03	1	ITKIAIPDNITTIGDFAFDNCPTLK
☑	2109	939.26	1876.51	1876.75	-0.24	0	1	6.9e+02	1	GSSMSNMNVSSNSGSSGSGK + Oxidation (M)
☑	795	871.02	1740.03	1739.93	0.10	2	1	1.4e+03	1	IEAADQRRDAILNQK
☑	291	619.93	1856.76	1856.99	-0.22	1	1	1.5e+03	1	IEGGSCLAPLLMKQELR
☑	1163	993.76	992.75	992.45	0.30	1	1	3.8e+02	1	MGLCREER
☑	2424	951.17	2850.49	2850.41	0.08	1	1	3.7e+02	1	HCCTAFETDVVTQCLSLLSNVVVGKK + Carbamidomethyl (C)
☑	109	509.19	1524.55	1524.77	-0.22	1	1	9.8e+02	1	VIPPGCIERDANGK + Carbamidomethyl (C)
☑	816	875.56	2623.67	2624.23	-0.56	2	1	1.5e+03	1	NGYSDPFVEFSCPQADPPKVRSK + Carbamidomethyl (C)
☑	1443	1097.61	3289.80	3289.53	0.27	2	1	1.1e+03	1	QLCSEGNNEGMCDVLKTTMNDVVHALAKR + 2 Carbamidomethyl (C)
☑	1984	815.03	1628.06	1627.78	0.28	1	1	4.6e+02	1	ALCGVCGKEILCSFVS
☑	2374	872.73	2615.16	2615.31	-0.15	1	1	3.3e+02	1	PFPSPPSHPHGCAVAARTYTLPQK + Carbamidomethyl (C)
☑	980	934.17	2799.48	2799.47	0.01	2	1	1.4e+03	1	EIIKVKIYILDEFDSNFDPNFLK
☑	2113	941.10	1880.18	1879.96	0.22	2	1	4.8e+02	1	LVQRWDGCHSPTLGRR
☑	2378	877.61	2629.81	2629.29	0.52	2	1	5.6e+02	1	CMLLLPDDGVADVGWRRAGSGAASR + Carbamidomethyl (C)
☑	1383	1077.12	3228.34	3228.51	-0.17	0	1	1.6e+03	1	TYWPILFWFTMACSSLVGTCISDFIDR + Carbamidomethyl (C)
☑	447	702.42	2104.23	2104.07	0.16	0	1	1.4e+03	1	EGEIPASLGVIEASFFDPVK
☑	847	891.36	2671.06	2671.44	-0.39	1	1	1.3e+03	1	EGKPELHYIPPVYMTAPTLKIFK
☑	1337	1059.71	2117.41	2117.12	0.29	2	1	1.5e+03	1	AMVERLLADVHAQRDHLK + Oxidation (M)
☑	1724	619.90	1237.79	1237.59	0.20	0	1	3.9e+02	1	GDDAAASSLLYR
☑	944	927.63	2779.88	2780.35	-0.47	1	1	1.5e+03	1	YNYASGANYVKILEQIGADLTEVSY
☑	1256	1029.49	2056.97	2057.10	-0.13	0	1	1.2e+03	1	TLEQISAALAACAPYLPLR + Carbamidomethyl (C)

☑	1472	1109.91	2217.80	2218.17	-0.38	1	1	1.2e+03	1	RYGATVILHGVTVAEAEAYAK
☑	2226	711.42	2131.23	2131.02	0.21	0	1	3.8e+02	1	EEAYEMPQLMPVLHVVCK + Oxidation (M)
☑	1398	1081.11	3240.30	3240.70	-0.40	2	1	1.5e+03	1	KVVAQKLPAFITVGNYVVDCQSFGQIMASK
☑	1537	1134.82	2267.62	2267.15	0.48	1	0	1.5e+03	1	DFFINFSSSGSTYNSLLIKK
☑	1714	1231.65	2461.30	2461.13	0.16	2	0	1.2e+03	1	QYDMTEWRENIGTYKNEIR + Oxidation (M)
☑	2468	1050.17	3147.48	3147.54	-0.07	1	0	3.2e+02	1	ALEPAHVPDPCQFVKNHISLSYTNEPGK + Carbamidomethyl (C)
☑	987	936.59	2806.75	2806.48	0.27	1	0	1.3e+03	1	CPILTITTIASSSDSYNVIPDRVTLK
☑	2083	609.62	1825.83	1825.77	0.06	2	0	4.2e+02	1	DRYCQKYYQGMPCR + Oxidation (M)
☑	357	649.78	648.77	649.32	-0.55	0	0	8.6e+02	1	ITGGMR + Oxidation (M)
☑	1232	1019.05	3054.12	3054.44	-0.32	1	0	2e+03	1	WSCQFLSDTMWPLERAEVITDVICR + Carbamidomethyl (C)
☑	1266	1032.17	3093.50	3093.51	-0.01	1	0	1.4e+03	1	INLPNLENIDLTSNGNFNIKNDCLYDK
☑	2420	942.75	2825.22	2825.51	-0.29	2	0	3.3e+02	1	VERAVPFNAIVPPPPSIGTDVPVDPRS
☑	218	578.04	1731.11	1730.94	0.16	1	0	1.4e+03	1	ALVLGMMVKTAEAPLR + 2 Oxidation (M)
☑	570	769.32	2304.95	2305.07	-0.12	0	0	1.2e+03	1	MVQNYDLFIMPNTTGFWGR + Oxidation (M)
☑	1827	686.13	1370.25	1369.69	0.56	0	0	7.5e+02	1	NYHINLFFFR
☑	2007	838.90	1675.78	1675.68	0.11	1	0	4.4e+02	1	MDKSQGMANNYDMR + Oxidation (M)
☑	1464	1105.56	3313.64	3313.59	0.05	2	0	1.2e+03	1	VYDVDRVYGTNDSTEYIFYDRVAALVDR
☑	2381	884.41	2650.22	2650.43	-0.21	2	0	3.5e+02	1	DSALPLAVAPPEAVVMLLMDVRRR + 2 Oxidation (M)
☑	37	432.86	863.70	863.40	0.30	0	0	1e+03	1	WDLQMR + Oxidation (M)
☑	494	727.83	2180.45	2180.05	0.41	1	0	1.4e+03	1	APRWASFLEVMTEELEEK + Oxidation (M)
☑	293	622.61	1243.21	1243.65	-0.44	0	0	1.6e+03	1	GSLLAGQSGTTPR
☑	1165	993.87	992.86	992.55	0.31	0	0	4.8e+02	1	FMVSLVAAR
☑	1306	1047.10	3138.26	3138.47	-0.21	2	0	1.8e+03	1	RVAHWNMVDGVALCATHDKVESSAGGESR + Carbamidomethyl (C)
☑	2399	908.20	2721.58	2721.32	0.26	2	0	3.7e+02	1	AATYDTPVDSFFGGLFRETKICAR + Carbamidomethyl (C)
☑	1343	1060.88	3179.62	3180.12	-0.50	0	0	1.3e+03	1	CDDNCTTCYGSATYCMSCSSDTYLSSNR + 2 Carbamidomethyl (C); Oxidation (M)
☑	1621	586.30	1170.60	1170.48	0.11	0	0	4.9e+02	1	GGAGDAQGCHNGK
☑	748	848.57	2542.67	2542.15	0.53	0	0	1.6e+03	1	EQAVGGPCMQPTPWPEIGQMCVK + Carbamidomethyl (C)
☑	683	823.89	822.89	822.36	0.52	0	0	7.9e+02	1	LDCCIEK
☑	2129	960.59	1919.17	1918.95	0.22	1	0	3.9e+02	1	TRPQSGIQGMARMDALR + 2 Oxidation (M)
☑	1436	1095.81	3284.40	3284.57	-0.18	2	0	1.6e+03	1	QGGQEMIRLSEHAIPWNQEFKFYMTTK + Oxidation (M)
☑	2473	1058.27	3171.78	3171.49	0.28	2	0	3.3e+02	1	IEDSQGRELSSGCAVMSEGTEFVIATRSR + Carbamidomethyl (C)
☑	1368	1067.80	2133.58	2133.08	0.50	1	0	1.4e+03	1	LRGVGIGTFCVVSGTVSHGCK + Carbamidomethyl (C)
☑	2003	835.87	1669.72	1669.87	-0.15	2	0	4.3e+02	1	ESNVVPDTRVGVDRK
☑	826	879.54	878.53	878.58	-0.05	1	0	4.2e+02	1	LPRIKPR

☑	871	899.55	2695.62	2695.27	0.35	1	0	1.2e+03	1	THGGCSAADAGSWRNGVPANVQQVWK
☑	958	930.92	2789.74	2789.39	0.35	2	0	1.9e+03	1	MSSAVGAAMSLIGQDSDEARAAVRLQR
☑	975	933.63	2797.88	2797.34	0.53	0	0	1.6e+03	1	GLTDDEQCLPVHATLIGISDSGSSTPK + Carbamidomethyl (C)
☑	1453	1100.21	2198.41	2198.05	0.36	2	0	1.4e+03	1	NFPEDAQKKSSDGEVSFVSK
☑	2094	922.47	1842.92	1842.94	-0.03	2	0	4.4e+02	1	GTATTLPSPSAEGERGKGK
☑	14	369.79	737.56	737.42	0.14	0	0	7.5e+02	1	LANPAPR
☑	690	825.39	2473.16	2473.32	-0.16	2	0	1.2e+03	1	SITEQEIIEELKVHIDFRFK
☑	848	891.84	890.83	891.42	-0.60	1	0	6.7e+02	1	KCVDGSQR
☑	1226	1017.21	3048.61	3048.43	0.19	2	0	1.6e+03	1	KEPGPSNAAICDAYAMRLLQEATEEER + Carbamidomethyl (C)
☑	2183	1025.64	2049.27	2049.03	0.25	1	0	4.9e+02	1	VNGKSLTSEEIHFGNVYR
☑	2487	1104.58	3310.72	3311.22	-0.49	1	0	3.3e+02	1	NTNERCSCPGDCHTCDWNDGAPGACHFCK + 3 Carbamidomethyl (C)
☑	21	390.70	1169.09	1168.61	0.47	0	0	1.1e+03	1	TFSLGAFADLK
☑	668	815.36	2443.05	2443.30	-0.25	1	0	1.4e+03	1	GHKIDNVPELPLVVSDDVEQIK
☑	2029	852.70	1703.38	1702.92	0.46	1	0	8e+02	1	KAFPVLAIGCSAASGVR + Carbamidomethyl (C)
☑	2233	1072.69	2143.37	2143.05	0.32	0	0	4.3e+02	1	QLWWGHQIPAFGFTAPCGK
☑	2311	786.54	2356.60	2357.05	-0.45	2	0	6.2e+02	1	MSCTEGQRAVLCQCTICRK + 4 Carbamidomethyl (C)
☑	2391	895.51	2683.50	2683.35	0.15	1	0	3.7e+02	1	VDLFKPQLIICGGSAYPRDWDYK
☑	2210	1051.76	2101.50	2101.95	-0.45	0	0	7.4e+02	1	CGGSDSNVSSPAPPVNAVAAMR + Oxidation (M)
☑	624	793.47	2377.39	2377.06	0.33	1	0	1.4e+03	1	EEGGPVMAMRPPSGSCRVDASR + Carbamidomethyl (C); 2 Oxidation (M)
☑	1248	1024.96	2047.90	2047.97	-0.07	0	0	1.5e+03	1	QQSFSAAAPAASGDFSHITR
☑	1263	1031.94	3092.79	3092.36	0.43	1	0	1.6e+03	1	GGMWPAIHEDAFNGGGWGGSKNSGGGATDFR
☑	1304	1045.95	3134.82	3134.60	0.22	2	0	1.5e+03	1	GQLGDQVDEHAEREAIMDLLKLSVIEAR
☑	659	406.52	811.03	811.53	-0.50	1	0	4.5e+02	1	VLKIPSR
☑	825	879.06	878.05	878.44	-0.38	1	0	6.7e+02	1	KCLEAMK + Carbamidomethyl (C)
☑	1237	1021.18	3060.52	3060.36	0.16	2	0	1.4e+03	1	TCGKCVDAYFMYKGGCYLAGAAGPGTGTK + 3 Carbamidomethyl (C)
☑	491	726.72	2177.13	2177.03	0.09	2	0	1.2e+03	1	DNVCEDTPLHFAARYNKK + Carbamidomethyl (C)
☑	1374	1071.12	3210.35	3210.41	-0.06	1	0	1.6e+03	1	TMHEMGVYGMDCASVLYLLRLCLCDR + 3 Carbamidomethyl (C); 3 Oxidation (M)
☑	1380	1076.16	3225.45	3225.40	0.04	1	0	1.5e+03	1	YEGLAMQCADGYSGSLCETQDTTRAPTTTR
☑	791	867.17	2598.48	2598.53	-0.04	2	0	1.5e+03	1	LLAFVPSLQFLDGLIVRRSEVVK
☑	1455	1100.83	1099.82	1099.63	0.18	1	0	1.5e+03	1	TAEREILLR
☑	1726	1239.43	3715.26	3715.85	-0.59	1	0	1.4e+03	1	AFTFPSLPLMPTMVAMTCTRLGVPTTFDDVVLR + Carbamidomethyl (C); 2 Oxidation
☑	2006	559.03	1674.08	1673.86	0.22	2	0	4.8e+02	1	RCAHCGAIFRTLAAK + Carbamidomethyl (C)
☑	2045	871.52	1741.02	1740.74	0.28	0	0	4.2e+02	1	EGDCFAPLTTAVSDCR + Carbamidomethyl (C)
☑	906	913.44	912.43	912.54	-0.11	1	0	3.9e+02	1	NLLSPKNK

☑	1452	1099.37	2196.72	2197.11	-0.39	2	0	1.6e+03	1	SQQATDESVHQAKVSLAKDR
☑	1555	1144.21	2286.41	2286.15	0.26	1	0	1.6e+03	1	RLEMGTSEVVNVPLDIQQCR
☑	1651	1189.02	3564.04	3563.98	0.07	2	0	1.5e+03	1	IGTIVVDELHMLGETSRGATLELLLTKLLCLR + Carbamidomethyl (C)
☑	2110	939.32	1876.63	1876.94	-0.32	2	0	5e+02	1	RKGDFVPFMHLMVER + Oxidation (M)
☑	376	658.23	1971.68	1972.02	-0.34	0	0	1.6e+03	1	DVDFEVLQLTPAEVLER
☑	438	697.34	1392.66	1392.67	-0.01	0	0	1.1e+03	1	GSVGDFEVPMALR + Oxidation (M)
☑	1743	626.94	1251.86	1251.57	0.29	1	0	4.1e+02	1	MMLDASERGSR
☑	1491	1117.49	3349.46	3349.59	-0.13	2	0	1.2e+03	1	SDSPNPTVSKEMVKNVVNNLGGIPDYNCSAK + Carbamidomethyl (C); Oxidation (M)
☑	1552	1142.30	3423.88	3423.62	0.26	2	0	1.5e+03	1	YSSIDFFQKLDEIYYMTTQTTTNTQKNK + Oxidation (M)
☑	1760	1272.14	3813.41	3812.86	0.55	2	0	1.7e+03	1	CMEYAIFSYNKDLIMLLANNYKIQIDIDICAR + Oxidation (M)
☑	1988	549.47	1645.37	1644.85	0.52	2	0	9.7e+02	1	EMKALGIRANYHAR + Oxidation (M)
☑	2408	924.96	2771.87	2771.87	-0.00	1	0	6.4e+02	1	DDNGDDDDDDDDDDNDDDCPSRSR + Carbamidomethyl (C)
☑	2412	927.29	2778.85	2779.32	-0.46	1	0	6.2e+02	1	ELSQLQINSYSEYLYQSRTLEES
☑	1388	1078.10	3231.28	3231.58	-0.30	2	0	1.8e+03	1	RLLDNTYIFYSSDHGYHLGQFALMKDK
☑	1950	785.07	1568.12	1567.92	0.20	1	0	5e+02	1	KPDPPAPKPPKQIR
☑	2031	571.25	1710.72	1710.88	-0.16	1	0	4.6e+02	1	ALDRDATSTSLSAIYK
☑	2436	969.80	2906.39	2906.42	-0.03	2	0	3.9e+02	1	EMAECTFQPLLSPGTRAMVRLAQER + Carbamidomethyl (C); Oxidation (M)
☑	144	531.42	1591.24	1591.73	-0.50	1	0	1.6e+03	1	FMLGDCVRMLYR + Carbamidomethyl (C); 2 Oxidation (M)
☑	320	633.76	632.75	632.33	0.42	1	0	1e+03	1	HKTTF
☑	1645	1187.14	1186.13	1185.65	0.49	0	0	1.8e+03	1	NGAVSSNGLLVR
☑	1132	987.72	2960.14	2959.61	0.53	2	0	1.7e+03	1	DGPAYQTGIRLGDVLLRIAGVYVDSIAK
☑	1720	1236.37	3706.09	3705.80	0.29	2	0	1.5e+03	1	ASDSFIHAVPESSALDGTTVPAQPRGQRRPCDQR + Carbamidomethyl (C)
☑	2443	977.76	2930.27	2930.61	-0.34	0	0	3.7e+02	1	VMIPVPLMLLPPVLMNFLFHPANGVR + Oxidation (M)
☑	547	755.42	2263.24	2263.20	0.04	2	0	1.2e+03	1	IVFKGHSASKCDIVVADLYAK
☑	1245	1023.41	3067.21	3067.55	-0.34	2	0	1.3e+03	1	QHSRSRMATVAMLACVVFFVALGLACMR
☑	2004	559.01	1674.00	1673.85	0.16	2	0	4.8e+02	1	QKEEAADKLLGTEDK
☑	2483	1082.64	3244.91	3244.44	0.47	2	0	4e+02	1	TEARSEAETACEADLNQELSMMSVAQRSR + 2 Oxidation (M)
☑	1083	968.40	2902.19	2902.44	-0.25	2	0	1.3e+03	1	EECTVQEIPKQPPLSVAAEKTETYR + Carbamidomethyl (C)
☑	191	563.36	562.35	562.28	0.07	0	0	3e+02	1	AELCK
☑	1017	944.87	2831.57	2831.64	-0.06	1	0	1.5e+03	1	VATRIIMIYIIPILCLIMIVPHHK + 2 Oxidation (M)
☑	1311	1048.50	3142.49	3142.26	0.23	2	0	1.3e+03	1	VTSYDGKNCFDCPWDKDCCGCVLTDK + 4 Carbamidomethyl (C)
☑	1534	1131.85	2261.68	2262.08	-0.40	2	0	1.5e+03	1	DLCQRCRPCTRLPWLSCR + Carbamidomethyl (C)
☑	2455	1002.87	3005.59	3005.55	0.04	2	0	3.7e+02	1	DRKPVPTRGSESQAGGVPQTIASAVSGPEK
☑	504	731.07	2190.20	2190.14	0.05	1	0	1.9e+03	1	DYVGEGPQILRNAAATFGALK

☑	807	873.65	1745.28	1744.74	0.54	0	0	1.9e+03	1	ESCVCAGTVGFNETSGK + Carbamidomethyl (C)
☑	2433	968.52	2902.54	2902.41	0.13	2	0	3.9e+02	1	YTTIFPKNYMSGDIIIMVCFMSKGK + Oxidation (M)
☑	1082	968.05	2901.13	2901.25	-0.13	2	0	1.6e+03	1	QDCQGELQCAVDFMQEEVEKMRR + 2 Carbamidomethyl (C); Oxidation (M)
☑	1458	1102.02	3303.03	3302.48	0.55	0	0	1.8e+03	1	CVQLLYGEINLGGFTMLMYAVACDDYDR + 2 Carbamidomethyl (C); Oxidation (M)
☑	1569	1149.57	3445.69	3445.80	-0.12	2	0	1.2e+03	1	MRDEPAWALFATTVVSHVPMIPTAVLFYKR
☑	1023	946.52	2836.54	2836.45	0.08	2	0	1.5e+03	1	SLEPSISASPPPETHKMAVALEAFRR + Oxidation (M)
☑	2223	1064.42	2126.83	2126.95	-0.13	1	0	4.6e+02	1	SIHQMPIDDDEDDLARNK + Oxidation (M)
☑	1203	1007.55	3019.63	3019.45	0.18	1	0	1.3e+03	1	ATIDEVLSHPYFEKTFSHGIMSPNQR + Oxidation (M)
☑	1554	1143.39	3427.16	3426.81	0.35	1	0	1.7e+03	1	ACRVWYLYGLGLVLLWWLAYAHLTVEYR
☑	2312	788.01	2361.00	2361.20	-0.20	2	0	3.9e+02	1	ADDAAAEVVTEFAGVVATSARRR
☑	2317	1183.82	2365.62	2365.08	0.54	1	0	6.6e+02	1	LSFEGGCNASFARSLIADCYK + 2 Carbamidomethyl (C)
☑	813	874.23	1746.45	1746.85	-0.40	2	0	2e+03	1	EAREAAESDAGAKSLSR
☑	2273	752.21	2253.60	2253.04	0.57	2	0	8e+02	1	MRALEQQASKIEGDGEMNSK + 2 Oxidation (M)
☑	2480	1072.71	3215.10	3214.59	0.51	1	0	5.5e+02	1	VFNSTVLLLLVTMMCCNAGTASAGESNVKK + 2 Carbamidomethyl (C)
☑	757	852.48	851.47	851.41	0.05	0	0	5.1e+02	1	HGTPDLVN
☑	2297	774.69	2321.06	2321.14	-0.08	2	0	4.1e+02	1	CEATKASVKQQTDEMVEIVR + Carbamidomethyl (C)
☑	2418	940.78	2819.33	2819.33	-0.00	1	0	3.7e+02	1	EEFTNVAVGHFGCGLAWPVKDTNSGK + Carbamidomethyl (C)
☑	1194	1004.77	3011.29	3011.73	-0.44	2	0	1.7e+03	1	AINEAVDGKWRLLVYALQYVIVPLIR
☑	1202	1007.35	3019.02	3019.44	-0.42	2	0	1.6e+03	1	FFEWEATSGSQERMNEVITSKMATLK
☑	1476	1111.28	2220.55	2220.94	-0.39	2	0	1.7e+03	1	GCTKCEDGYSETSDRLLCK + 2 Carbamidomethyl (C)
☑	2263	748.78	2243.31	2243.14	0.17	2	0	4.2e+02	1	MKESSQHIMALTESALPRAK + Oxidation (M)
☑	2416	937.00	2807.98	2807.45	0.53	1	0	5.6e+02	1	SEESKDEPLSFWIVFSPLIACLGLK
☑	1506	1122.71	3365.11	3364.51	0.60	2	0	1.5e+03	1	LRAADRQCVTDGATLYPDMFCTDSSDATLR + Carbamidomethyl (C); Oxidation (M)
☑	2398	906.42	2716.25	2716.32	-0.06	2	0	4e+02	1	QQTRWPSPVATASESSNLQSRGDSK
☑	861	895.75	894.74	894.58	0.17	1	0	4.6e+02	1	RLQPILR
☑	1407	1085.06	2168.11	2168.08	0.02	0	0	1.9e+03	1	MSWNTNHVWTVTLTIPPR + Oxidation (M)
☑	225	583.48	1747.43	1746.92	0.51	0	0	1.6e+03	1	INSFIPIICVINNCK + Carbamidomethyl (C)
☑	1420	1088.21	3261.60	3261.60	0.00	1	0	1.6e+03	1	MGSTEDALVISCENVHKTYLLGSEGVPALR + Carbamidomethyl (C); Oxidation (M)
☑	1637	1181.55	3541.64	3541.70	-0.07	2	0	1.1e+03	1	WVPSSDSGAAAERLCGEATSEAPYYSVADKALLK
☑	623	793.42	792.41	792.45	-0.03	1	0	5.5e+02	1	KYIQGGK
☑	1046	955.29	2862.85	2863.37	-0.52	2	0	1.6e+03	1	GYSGNITPEGLRGSVYEIAKDQHGCR + Carbamidomethyl (C)
☑	1699	1219.06	3654.16	3654.65	-0.49	2	0	1.7e+03	1	GHQCIDNGVEKCDGHQLYTVFSASRYCNQLR + 2 Carbamidomethyl (C)
☑	2207	699.60	2095.78	2096.08	-0.29	2	0	5.7e+02	1	LPWQKFLYTIEADKCGK + Carbamidomethyl (C)
☑	1	*285.02*	*284.02*							

2	285.04	284.03
3	285.04	284.03
4	285.04	284.03
5	285.05	284.04
6	285.05	284.04
7	285.05	284.05
8	285.07	284.06
9	285.07	284.06
10	285.07	284.06
11	299.11	298.10
12	329.22	328.21
13	355.10	354.09
15	371.12	370.11
16	371.12	370.12
17	371.12	370.12
18	371.15	370.14
19	371.19	370.18
26	429.08	428.07
27	429.09	428.08
28	429.09	428.08
29	429.09	428.09
30	429.12	428.11
31	429.13	428.13
32	429.14	428.13
33	429.15	428.14
34	429.23	428.22
38	434.65	433.65
45	445.11	444.10
46	445.15	444.14
47	445.16	444.15
48	445.18	444.17
49	445.18	444.17
51	446.33	445.33
54	452.63	451.63

☑	55	453.76	452.75
☑	59	457.31	456.31
☑	65	465.61	464.60
☑	67	469.02	468.01
☑	72	473.35	472.34
☑	75	476.86	475.85
☑	80	481.73	480.73
☑	83	484.32	483.32
☑	87	489.56	488.56
☑	91	496.35	495.34
☑	96	501.50	500.50
☑	97	502.51	501.50
☑	99	502.70	501.69
☑	100	503.31	502.31
☑	101	503.42	502.41
☑	104	504.06	503.05
☑	111	510.45	509.44
☑	112	510.84	509.83
☑	113	511.22	510.21
☑	114	511.79	510.79
☑	117	513.61	512.60
☑	123	518.25	517.24
☑	124	518.72	517.71
☑	129	523.24	522.23
☑	130	523.48	522.47
☑	131	524.28	523.27
☑	133	525.83	524.82
☑	134	526.89	525.89
☑	137	527.84	526.83
☑	138	528.08	527.07
☑	139	528.69	527.68
☑	141	530.02	529.02
☑	143	531.34	530.34
☑	145	531.84	530.83

☑	149	533.47	532.47
☑	154	535.81	534.81
☑	157	537.64	536.63
☑	159	542.33	541.33
☑	163	545.75	544.74
☑	168	547.36	546.35
☑	171	551.30	550.29
☑	174	553.30	552.29
☑	175	553.73	552.72
☑	176	553.76	552.75
☑	177	554.50	553.49
☑	183	557.61	556.60
☑	186	559.29	558.29
☑	187	559.71	558.70
☑	188	560.09	559.08
☑	192	563.47	562.46
☑	193	564.34	563.34
☑	196	566.78	565.77
☑	197	567.06	566.05
☑	198	567.14	566.13
☑	200	568.59	567.58
☑	202	569.20	568.19
☑	205	570.14	569.14
☑	209	573.28	572.27
☑	211	573.45	572.44
☑	213	573.97	572.96
☑	214	575.38	574.37
☑	217	577.78	576.77
☑	219	578.38	577.37
☑	220	578.90	577.89
☑	221	581.00	579.99
☑	224	583.17	582.16
☑	226	583.89	582.88
☑	227	584.36	583.35

☑	229	586.03	585.02
☑	232	587.98	586.97
☑	235	590.65	589.64
☑	243	595.93	594.93
☑	247	597.46	596.45
☑	251	599.31	598.30
☑	256	600.06	599.05
☑	258	600.42	599.41
☑	260	601.19	600.18
☑	268	607.72	606.71
☑	272	610.30	609.29
☑	274	610.46	609.45
☑	276	612.37	611.36
☑	278	613.35	612.34
☑	279	613.38	612.37
☑	286	617.92	616.92
☑	289	618.13	617.12
☑	299	624.82	623.81
☑	301	625.47	624.47
☑	304	626.76	625.76
☑	306	627.25	626.24
☑	308	628.62	627.61
☑	309	628.95	627.94
☑	312	630.47	629.46
☑	318	632.48	631.47
☑	327	637.03	636.02
☑	328	637.66	636.66
☑	329	637.80	636.79
☑	330	638.30	637.29
☑	332	639.55	638.55
☑	333	640.28	639.27
☑	334	641.16	640.15
☑	335	641.38	640.37
☑	336	641.61	640.61

☑	341	644.57	643.57
☑	342	644.63	643.62
☑	344	645.37	644.36
☑	346	645.83	644.82
☑	351	647.16	646.15
☑	354	648.05	647.04
☑	355	648.20	647.19
☑	356	649.18	648.17
☑	358	649.83	648.83
☑	363	650.40	649.39
☑	364	650.50	649.50
☑	366	650.77	649.76
☑	367	652.86	651.85
☑	369	654.72	653.71
☑	372	655.53	654.52
☑	377	658.40	657.39
☑	378	658.69	657.68
☑	379	659.06	658.05
☑	380	659.43	658.43
☑	381	661.02	660.01
☑	384	663.08	662.07
☑	385	663.25	662.25
☑	386	665.41	664.41
☑	387	665.70	664.69
☑	390	668.17	667.16
☑	394	672.20	671.19
☑	395	672.39	671.38
☑	397	673.58	672.57
☑	398	673.96	672.95
☑	400	674.50	673.49
☑	401	675.42	674.42
☑	403	677.55	676.54
☑	404	678.13	677.12
☑	405	678.58	677.57

☑	408	680.49	679.48
☑	409	681.02	680.02
☑	412	682.30	681.30
☑	417	683.92	682.91
☑	419	685.53	684.52
☑	425	688.07	687.06
☑	429	692.15	691.14
☑	431	694.05	693.05
☑	434	695.17	694.16
☑	437	697.29	696.28
☑	443	699.68	698.68
☑	445	700.57	699.57
☑	448	703.21	702.20
☑	453	705.61	704.61
☑	454	705.87	704.86
☑	455	707.13	706.13
☑	459	709.36	708.35
☑	466	714.57	713.56
☑	469	715.63	714.63
☑	470	715.99	714.98
☑	477	718.77	717.76
☑	478	720.34	719.33
☑	481	720.90	719.90
☑	483	722.30	721.29
☑	485	722.71	721.70
☑	490	726.50	725.50
☑	493	726.96	725.95
☑	496	728.42	727.41
☑	497	729.01	728.00
☑	498	729.17	728.16
☑	500	730.40	729.40
☑	501	730.75	729.74
☑	503	730.90	729.89
☑	506	732.23	731.22

☑	508	732.95	731.95
☑	509	733.14	732.14
☑	510	733.33	732.32
☑	512	734.56	733.55
☑	514	735.66	734.65
☑	517	737.12	736.11
☑	519	738.00	736.99
☑	521	738.38	737.37
☑	522	738.84	737.84
☑	523	739.41	738.40
☑	524	739.61	738.60
☑	525	739.63	738.62
☑	526	739.95	738.94
☑	535	745.84	744.83
☑	542	750.32	749.31
☑	546	754.02	753.02
☑	548	755.46	754.45
☑	549	755.71	754.70
☑	550	756.22	755.22
☑	551	757.38	756.37
☑	554	759.89	758.88
☑	559	764.08	763.07
☑	561	764.49	763.48
☑	564	766.12	765.11
☑	565	766.52	765.51
☑	566	766.98	765.97
☑	568	768.39	767.38
☑	569	768.67	767.66
☑	573	771.10	770.09
☑	575	772.46	771.45
☑	576	773.51	772.51
☑	585	775.71	774.70
☑	587	777.26	776.25
☑	588	777.36	776.35

☑	589	777.37	776.37
☑	593	782.26	781.25
☑	594	782.43	781.42
☑	595	782.45	781.44
☑	597	783.71	782.70
☑	601	784.92	783.91
☑	602	785.33	784.32
☑	604	786.07	785.06
☑	606	787.20	786.19
☑	610	787.81	786.81
☑	614	789.89	788.88
☑	617	792.48	791.47
☑	621	793.34	792.33
☑	622	793.42	792.41
☑	626	793.88	792.87
☑	627	794.30	793.29
☑	628	795.15	794.15
☑	629	795.53	794.52
☑	630	796.56	795.55
☑	631	797.11	796.10
☑	632	797.53	796.52
☑	634	798.22	797.21
☑	638	800.35	799.34
☑	640	803.23	802.23
☑	641	803.72	802.71
☑	642	803.81	802.80
☑	644	805.08	804.07
☑	647	806.05	805.04
☑	648	806.49	805.49
☑	649	807.72	806.71
☑	651	808.28	807.27
☑	653	808.76	807.75
☑	654	808.91	807.90
☑	656	809.89	808.88

☑	660	812.36	811.35
☑	662	813.27	812.26
☑	665	814.71	813.70
☑	667	815.14	814.14
☑	669	408.25	814.49
☑	670	815.81	814.81
☑	673	816.48	815.47
☑	674	816.49	815.49
☑	679	820.91	819.91
☑	681	821.49	820.48
☑	692	825.43	824.43
☑	698	827.97	826.96
☑	701	828.47	827.46
☑	708	831.12	830.11
☑	709	416.26	830.50
☑	710	831.81	830.80
☑	714	833.06	832.06
☑	715	834.00	832.99
☑	719	835.27	834.26
☑	720	836.36	835.35
☑	725	839.43	838.42
☑	726	839.75	838.74
☑	729	840.51	839.50
☑	731	841.87	840.86
☑	733	421.59	841.17
☑	734	842.47	841.46
☑	736	842.72	841.71
☑	741	844.58	843.57
☑	742	845.34	844.33
☑	743	845.78	844.77
☑	747	848.26	847.25
☑	749	848.78	847.78
☑	751	849.02	848.01
☑	752	849.40	848.39

☑	753	849.69	848.69
☑	756	851.75	850.74
☑	758	852.60	851.59
☑	759	852.96	851.95
☑	764	855.23	854.22
☑	769	857.36	856.35
☑	770	857.39	856.38
☑	772	857.67	856.66
☑	773	857.71	856.71
☑	775	858.19	857.18
☑	776	858.58	857.57
☑	779	860.36	859.35
☑	787	863.80	862.80
☑	788	865.24	864.23
☑	789	865.27	864.27
☑	790	867.01	866.01
☑	792	867.33	866.33
☑	793	870.12	869.12
☑	794	870.38	869.37
☑	800	871.84	870.83
☑	805	873.45	872.44
☑	808	874.03	873.03
☑	809	874.05	873.04
☑	812	874.16	873.15
☑	815	874.87	873.86
☑	818	877.23	876.22
☑	819	877.24	876.23
☑	827	879.74	878.73
☑	828	880.22	879.21
☑	830	880.51	879.51
☑	832	881.86	880.85
☑	834	882.18	881.17
☑	837	883.93	882.93
☑	838	884.54	883.54

☑	840	885.30	884.29
☑	841	885.36	884.35
☑	842	886.83	885.83
☑	849	892.21	891.20
☑	850	892.28	891.27
☑	851	892.70	891.69
☑	852	893.06	892.06
☑	853	893.25	892.24
☑	858	893.90	892.89
☑	859	895.61	894.61
☑	864	896.43	895.42
☑	866	897.13	896.12
☑	868	897.54	896.53
☑	870	450.15	898.29
☑	872	900.21	899.20
☑	873	900.48	899.47
☑	874	900.73	899.72
☑	875	900.85	899.84
☑	877	902.67	901.67
☑	878	902.69	901.68
☑	880	902.98	901.97
☑	881	903.46	902.45
☑	882	903.57	902.56
☑	885	904.65	903.64
☑	886	904.80	903.79
☑	887	904.88	903.87
☑	889	905.16	904.15
☑	891	906.02	905.02
☑	892	906.53	905.52
☑	898	908.44	907.43
☑	899	908.85	907.84
☑	901	912.18	911.17
☑	908	914.82	913.81
☑	909	914.99	913.98

☑	911	*915.47*	*914.47*
☑	913	*916.14*	*915.13*
☑	914	*916.15*	*915.15*
☑	915	*916.43*	*915.42*
☑	921	*918.40*	*917.39*
☑	923	*919.52*	*918.51*
☑	924	*920.34*	*919.33*
☑	929	*922.27*	*921.26*
☑	930	*922.49*	*921.48*
☑	932	*923.02*	*922.01*
☑	933	*924.15*	*923.14*
☑	935	*924.35*	*923.34*
☑	937	*925.60*	*924.59*
☑	939	*925.68*	*924.67*
☑	941	*925.84*	*924.83*
☑	942	*926.14*	*925.13*
☑	943	*926.55*	*925.54*
☑	947	*928.71*	*927.70*
☑	948	*928.97*	*927.96*
☑	949	*929.22*	*928.21*
☑	951	*929.58*	*928.57*
☑	956	*930.64*	*929.64*
☑	959	*931.16*	*930.15*
☑	960	*931.35*	*930.34*
☑	966	*931.90*	*930.90*
☑	968	*932.39*	*931.39*
☑	969	*932.94*	*931.93*
☑	972	*933.32*	*932.31*
☑	974	*933.54*	*932.54*
☑	976	*933.67*	*932.67*
☑	979	*933.99*	*932.98*
☑	982	*935.65*	*934.64*
☑	983	*935.73*	*934.72*
☑	985	*936.35*	*935.34*

☑	990	937.11	936.10
☑	991	937.83	936.82
☑	992	937.86	936.85
☑	995	938.52	937.51
☑	996	938.61	937.60
☑	997	938.91	937.90
☑	999	939.05	938.05
☑	1003	940.28	939.27
☑	1004	470.82	939.63
☑	1007	942.14	941.13
☑	1010	942.90	941.90
☑	1011	943.19	942.18
☑	1012	943.34	942.33
☑	1013	943.48	942.47
☑	1015	944.48	943.48
☑	1016	944.82	943.81
☑	1020	946.39	945.39
☑	1021	946.44	945.44
☑	1022	946.45	945.44
☑	1024	948.02	947.01
☑	1025	948.21	947.21
☑	1026	948.49	947.48
☑	1027	948.49	947.48
☑	1028	948.78	947.77
☑	1029	949.21	948.21
☑	1030	949.60	948.60
☑	1031	951.00	949.99
☑	1033	951.04	950.04
☑	1037	951.90	950.89
☑	1038	951.90	950.89
☑	1039	952.74	951.73
☑	1040	952.97	951.96
☑	1041	953.66	952.65
☑	1042	954.45	953.44

☑	1043	955.11	954.10
☑	1044	955.19	954.18
☑	1047	956.04	955.04
☑	1050	956.70	955.70
☑	1053	957.00	955.99
☑	1056	957.40	956.39
☑	1057	957.69	956.69
☑	1058	958.37	957.36
☑	1060	959.72	958.72
☑	1063	960.50	959.50
☑	1065	961.96	960.95
☑	1068	962.58	961.57
☑	1069	962.87	961.87
☑	1070	963.22	962.21
☑	1071	963.22	962.21
☑	1073	482.50	962.98
☑	1074	482.61	963.21
☑	1077	966.99	965.98
☑	1078	967.12	966.11
☑	1079	967.49	966.48
☑	1086	484.84	967.67
☑	1087	485.25	968.49
☑	1092	972.20	971.19
☑	1093	972.72	971.71
☑	1099	974.34	973.33
☑	1100	974.56	973.55
☑	1101	975.43	974.42
☑	1102	975.85	974.84
☑	1104	976.23	975.22
☑	1105	976.36	975.35
☑	1106	976.58	975.57
☑	1110	489.78	977.55
☑	1112	490.01	978.01
☑	1113	980.19	979.18

☑	1115	980.60	979.59
☑	1117	981.63	980.63
☑	1118	981.65	980.64
☑	1121	983.08	982.07
☑	1122	983.59	982.58
☑	1123	983.94	982.93
☑	1124	984.53	983.53
☑	1125	985.06	984.05
☑	1126	985.17	984.16
☑	1127	985.25	984.24
☑	1130	987.21	986.20
☑	1133	987.86	986.85
☑	1134	987.91	986.90
☑	1137	988.38	987.37
☑	1138	988.40	987.39
☑	1140	494.99	987.97
☑	1144	990.02	989.01
☑	1145	990.04	989.03
☑	1146	495.82	989.63
☑	1147	990.71	989.70
☑	1150	991.38	990.38
☑	1153	991.70	990.69
☑	1154	991.71	990.70
☑	1155	992.08	991.07
☑	1157	992.58	991.57
☑	1159	992.73	991.73
☑	1160	993.43	992.42
☑	1161	993.45	992.44
☑	1162	993.55	992.55
☑	1164	993.79	992.79
☑	1166	994.21	993.21
☑	1167	994.84	993.84
☑	1169	995.86	994.85
☑	1170	996.00	994.99

1171	996.55	995.54						
1172	996.62	995.61						
1173	997.02	996.02						
1174	998.11	997.10						
1177	998.42	997.41						
1179	999.57	998.56						
1180	999.61	998.60						
1185	1001.83	1000.82						
1186	1002.02	1001.01						
1187	1002.02	1001.01						
1190	1002.77	1001.77						
1193	1004.39	1003.39						
1195	1005.13	1004.12						
1196	503.16	1004.30						
1197	1005.57	1004.56						
1199	1005.99	3014.95	3015.37	-0.42	2	---	1	MYDSSSEHQTGSKAPLSRCSTHHLQR + Carbamidomethyl (C); Oxidation (M)
1205	1008.76	1007.76						
1208	1009.92	1008.91						
1209	1010.52	1009.52						
1210	1011.24	1010.23						
1212	1012.53	1011.52						
1213	1013.02	1012.01						
1221	1015.02	1014.02						
1222	1015.20	1014.19						
1223	1015.36	1014.35						
1225	508.57	1015.13						
1227	1017.75	1016.75						
1228	1018.27	1017.26						
1229	1018.33	1017.32						
1230	1018.49	1017.48						
1234	1020.45	1019.45						
1235	1020.48	1019.47						
1239	1022.28	1021.27						
1241	1022.83	1021.82						

☑	1251	1027.15	1026.14
☑	1252	1027.17	1026.16
☑	1253	1027.49	1026.48
☑	1254	1027.78	1026.77
☑	1258	1030.72	1029.72
☑	1259	1030.73	1029.73
☑	1265	1032.05	1031.04
☑	1267	1032.42	1031.42
☑	1268	1032.58	1031.57
☑	1269	1033.16	1032.15
☑	1270	1033.96	1032.95
☑	1277	1036.87	1035.86
☑	1279	519.62	1037.22
☑	1282	1038.82	1037.81
☑	1283	1039.42	1038.41
☑	1284	1039.58	1038.57
☑	1285	1040.02	1039.01
☑	1286	1040.52	1039.51
☑	1289	1041.61	1040.60
☑	1291	1043.09	1042.08
☑	1294	1043.90	1042.89
☑	1295	1043.94	1042.93
☑	1297	1045.15	1044.14
☑	1299	1045.18	1044.17
☑	1307	1047.49	1046.48
☑	1309	1047.73	1046.72
☑	1310	1048.49	1047.49
☑	1315	1051.36	1050.35
☑	1319	1052.84	1051.83
☑	1320	1053.52	1052.52
☑	1321	1053.76	1052.76
☑	1326	1055.22	1054.21
☑	1327	1055.54	1054.53
☑	1328	1056.42	1055.41

1339	1059.96	1058.95						
1340	1060.60	1059.59						
1346	1061.35	1060.34						
1348	1061.83	1060.82						
1350	1062.35	1061.34						
1353	532.24	1062.47						
1356	1064.71	1063.70						
1362	1065.18	1064.18						
1366	1067.06	1066.05						
1371	1068.55	1067.55						
1373	1070.27	1069.26						
1378	1074.86	1073.85						
1385	1077.46	3229.35	3229.62	-0.27	2	---	1	FIQFIESGNHYKDQRLNNILYPETYK
1389	1078.13	1077.13						
1390	1078.34	1077.34						
1391	1079.32	1078.31						
1392	1079.33	1078.32						
1400	1082.25	1081.24						
1403	1083.61	1082.60						
1405	1084.77	1083.76						
1412	1085.71	1084.71						
1416	1086.41	1085.40						
1421	1088.61	1087.60						
1422	544.90	1087.79						
1424	1089.77	1088.76						
1426	1090.62	1089.61						
1428	546.56	1091.11						
1429	1092.16	1091.16						
1430	546.73	1091.45						
1439	1096.28	1095.27						
1442	1097.43	1096.42						
1444	549.46	1096.90						
1446	549.86	1097.70						
1448	1098.73	1097.73						

☑	1451	1099.23	1098.22
☑	1454	1100.28	1099.27
☑	1456	1100.86	1099.85
☑	1459	1102.68	1101.67
☑	1460	1103.19	1102.18
☑	1462	1104.58	1103.57
☑	1463	1105.54	1104.54
☑	1465	553.31	1104.60
☑	1467	1107.79	1106.78
☑	1468	1108.22	1107.21
☑	1473	1110.81	1109.81
☑	1474	1110.83	1109.82
☑	1480	1113.74	1112.73
☑	1486	1115.98	1114.98
☑	1488	1116.24	1115.23
☑	1489	1116.56	1115.55
☑	1493	1117.83	1116.83
☑	1495	1118.19	1117.18
☑	1497	1118.52	1117.51
☑	1499	1119.06	1118.05
☑	1501	1119.49	1118.48
☑	1502	1119.85	1118.84
☑	1507	561.93	1121.84
☑	1508	1123.04	1122.04
☑	1514	1125.14	1124.14
☑	1520	1126.14	1125.14
☑	1521	1126.45	1125.44
☑	1531	1130.17	1129.16
☑	1532	565.79	1129.57
☑	1535	1132.23	1131.22
☑	1536	1134.58	1133.57
☑	1538	1134.91	1133.90
☑	1539	568.30	1134.59
☑	1540	1135.89	1134.88

☑	1542	1137.24	1136.24
☑	1549	1140.11	1139.10
☑	1558	1145.83	1144.82
☑	1559	1146.25	1145.24
☑	1560	1146.32	1145.31
☑	1561	1146.71	1145.70
☑	1563	1147.35	1146.34
☑	1565	1148.10	1147.09
☑	1566	1148.39	1147.38
☑	1568	1149.44	1148.43
☑	1576	1152.18	1151.17
☑	1578	576.97	1151.93
☑	1581	577.51	1153.00
☑	1583	1154.64	1153.63
☑	1584	1154.77	1153.76
☑	1587	1157.69	1156.68
☑	1588	1158.12	1157.11
☑	1593	1162.25	1161.24
☑	1594	581.70	1161.38
☑	1596	1163.02	1162.01
☑	1599	1163.58	1162.57
☑	1600	1164.22	1163.21
☑	1603	583.92	1165.82
☑	1604	1167.30	1166.29
☑	1609	1169.46	1168.45
☑	1611	1169.74	1168.73
☑	1612	585.49	1168.96
☑	1613	1170.15	1169.14
☑	1614	1170.56	1169.55
☑	1627	1175.21	1174.20
☑	1629	588.38	1174.75
☑	1635	1179.91	1178.90
☑	1638	591.36	1180.71
☑	1654	1189.52	1188.51

☑	1655	1190.08	1189.07
☑	1656	1190.48	1189.48
☑	1657	1190.84	1189.84
☑	1663	599.39	1196.76
☑	1676	603.39	1204.77
☑	1677	1206.12	1205.11
☑	1678	603.58	1205.15
☑	1679	1208.30	1207.29
☑	1683	1210.46	1209.45
☑	1688	1214.00	1212.99
☑	1694	1216.57	1215.56
☑	1695	1216.99	1215.99
☑	1706	1227.11	1226.10
☑	1711	614.92	1227.82
☑	1723	1238.41	1237.40
☑	1728	620.49	1238.97
☑	1733	1243.83	1242.82
☑	1735	1245.52	1244.51
☑	1738	1246.25	1245.25
☑	1749	1256.18	1255.17
☑	1751	628.84	1255.66
☑	1758	635.75	1269.48
☑	1772	640.73	1279.44
☑	1773	1283.80	1282.79
☑	1783	1294.48	1293.47
☑	1785	647.95	1293.89
☑	1795	656.13	1310.24
☑	1805	668.12	1334.23
☑	1808	671.54	1341.07
☑	1811	673.84	1345.67
☑	1813	674.45	1346.89
☑	1817	678.87	1355.73
☑	1820	679.56	1357.10
☑	1824	682.28	1362.55

☑	1831	690.42	1378.83
☑	1834	691.66	1381.31
☑	1839	693.73	1385.45
☑	1850	712.37	1422.72
☑	1852	714.89	1427.77
☑	1853	717.81	1433.61
☑	1855	718.76	1435.51
☑	1858	722.87	1443.72
☑	1861	725.13	1448.25
☑	1870	728.73	1455.44
☑	1876	733.86	1465.70
☑	1878	734.36	1466.70
☑	1887	739.52	1477.02
☑	1888	739.68	1477.36
☑	1896	497.99	1490.95
☑	1898	747.86	1493.71
☑	1904	501.81	1502.42
☑	1909	754.89	1507.77
☑	1912	756.05	1510.08
☑	1915	758.32	1514.62
☑	1920	761.44	1520.87
☑	1921	762.10	1522.19
☑	1923	508.76	1523.27
☑	1924	763.37	1524.72
☑	1927	764.40	1526.79
☑	1929	767.07	1532.12
☑	1930	768.85	1535.69
☑	1932	770.56	1539.10
☑	1937	774.69	1547.36
☑	1944	780.68	1559.34
☑	1957	793.01	1584.00
☑	1963	803.13	1604.25
☑	1968	807.28	1612.55
☑	1971	809.99	1617.96

☑	1975	*812.55*	*1623.09*
☑	1976	*812.71*	*1623.40*
☑	1980	*814.08*	*1626.15*
☑	1981	*543.34*	*1626.99*
☑	1983	*815.03*	*1628.05*
☑	1987	*821.28*	*1640.55*
☑	1998	*834.30*	*1666.59*
☑	2011	*840.11*	*1678.20*
☑	2012	*840.14*	*1678.28*
☑	2014	*842.28*	*1682.55*
☑	2016	*843.11*	*1684.21*
☑	2023	*848.15*	*1694.29*
☑	2025	*849.98*	*1697.94*
☑	2027	*851.44*	*1700.87*
☑	2034	*858.17*	*1714.32*
☑	2037	*860.29*	*1718.57*
☑	2039	*574.51*	*1720.52*
☑	2043	*579.06*	*1734.17*
☑	2050	*877.36*	*1752.70*
☑	2053	*883.11*	*1764.20*
☑	2070	*901.14*	*1800.27*
☑	2071	*601.78*	*1802.31*
☑	2073	*905.48*	*1808.94*
☑	2074	*905.91*	*1809.81*
☑	2078	*909.92*	*1817.83*
☑	2080	*911.24*	*1820.47*
☑	2082	*912.03*	*1822.04*
☑	2084	*914.05*	*1826.09*
☑	2100	*927.08*	*1852.15*
☑	2101	*619.37*	*1855.09*
☑	2102	*928.85*	*1855.68*
☑	2104	*929.98*	*1857.94*
☑	2105	*931.04*	*1860.07*
☑	2106	*931.05*	*1860.09*

☑	2107	935.57	1869.13
☑	2111	939.94	1877.86
☑	2115	942.15	1882.29
☑	2118	632.64	1894.91
☑	2120	953.11	1904.21
☑	2123	955.91	1909.81
☑	2127	960.00	1917.99
☑	2128	960.14	1918.27
☑	2130	962.48	1922.95
☑	2131	962.84	1923.67
☑	2133	963.78	1925.56
☑	2135	965.43	1928.84
☑	2136	965.94	1929.87
☑	2137	966.63	1931.25
☑	2141	971.12	1940.23
☑	2143	974.21	1946.41
☑	2144	974.59	1947.16
☑	2145	974.92	1947.83
☑	2147	979.04	1956.06
☑	2149	980.52	1959.03
☑	2150	982.49	1962.97
☑	2151	982.65	1963.28
☑	2152	982.92	1963.83
☑	2154	985.35	1968.68
☑	2159	997.13	1992.26
☑	2160	997.80	1993.59
☑	2162	1002.82	2003.62
☑	2163	1003.99	2005.97
☑	2164	1005.10	2008.18
☑	2165	1007.11	2012.20
☑	2166	1007.57	2013.13
☑	2167	1007.92	2013.84
☑	2168	1008.75	2015.49
☑	2169	1010.88	2019.74

2172	1013.22	2024.42
2176	1016.14	2030.27
2177	1017.77	2033.53
2178	1018.42	2034.82
2182	1025.50	2048.98
2184	1025.98	2049.96
2185	1026.07	2050.12
2188	1027.60	2053.18
2190	1029.46	2056.91
2191	1029.83	2057.64
2197	689.64	2065.91
2201	1040.89	2079.76
2202	1041.97	2081.92
2204	1045.64	2089.27
2205	1046.50	2090.98
2212	1052.02	2102.02
2218	1057.57	2113.12
2221	1061.05	2120.08
2224	709.96	2126.84
2227	712.10	2133.28
2231	714.52	2140.53
2232	714.66	2140.96
2235	1075.41	2148.81
2236	1076.53	2151.04
2237	1078.96	2155.90
2241	1084.15	2166.28
2242	724.76	2171.25
2245	1088.62	2175.23
2247	1090.74	2179.47
2248	1090.88	2179.75
2249	727.97	2180.89
2250	1091.49	2180.97
2251	1101.30	2200.59
2252	1103.25	2204.48

☑	2253	1105.37	2208.73
☑	2257	1114.70	2227.38
☑	2258	1115.32	2228.63
☑	2259	1118.16	2234.31
☑	2260	1119.60	2237.18
☑	2266	751.14	2250.40
☑	2267	1126.58	2251.15
☑	2277	754.71	2261.09
☑	2280	1134.42	2266.84
☑	2284	1138.69	2275.36
☑	2289	765.64	2293.91
☑	2291	767.07	2298.19
☑	2292	768.25	2301.72
☑	2294	770.79	2309.34
☑	2295	770.98	2309.92
☑	2301	779.90	2336.69
☑	2303	1172.57	2343.12
☑	2307	784.55	2350.63
☑	2308	784.82	2351.44
☑	2313	788.15	2361.44
☑	2314	1182.20	2362.39
☑	2316	789.22	2364.63
☑	2324	795.40	2383.18
☑	2333	807.84	2420.50
☑	2337	814.12	2439.35
☑	2338	816.18	2445.52
☑	2344	825.87	2474.57
☑	2345	826.59	2476.74
☑	2350	833.23	2496.67
☑	2354	839.65	2515.92
☑	2355	840.65	2518.92
☑	2367	860.09	2577.25
☑	2370	866.57	2596.69
☑	2372	868.77	2603.28

☑	2373	872.68	2615.01
☑	2379	878.71	2633.12
☑	2380	882.64	2644.90
☑	2382	884.52	2650.53
☑	2387	892.45	2674.33
☑	2389	893.02	2676.03
☑	2394	897.53	2689.57
☑	2402	914.27	2739.78
☑	2403	918.63	2752.87
☑	2409	925.04	2772.10
☑	2411	926.61	2776.81
☑	2414	935.39	2803.16
☑	2415	935.46	2803.35
☑	2417	937.22	2808.64
☑	2419	941.67	2821.99
☑	2421	949.66	2845.96
☑	2425	952.74	2855.19
☑	2426	957.73	2870.16
☑	2429	962.96	2885.87
☑	2430	962.98	2885.91
☑	2431	965.22	2892.65
☑	2439	973.59	2917.75
☑	2440	975.24	2922.70
☑	2446	984.79	2951.36
☑	2448	987.73	2960.16
☑	2449	991.09	2970.24
☑	2450	994.85	2981.52
☑	2451	995.98	2984.92
☑	2452	996.83	2987.46
☑	2453	997.91	2990.70
☑	2457	1006.93	3017.76
☑	2458	1018.13	3051.38
☑	2459	1021.56	3061.67
☑	2467	1048.26	3141.76

☑	2476	*1066.07*	*3195.20*
☑	2477	*1066.52*	*3196.52*
☑	2478	*1068.94*	*3203.81*
☑	2484	*1090.15*	*3267.42*
☑	2485	*1092.21*	*3273.60*
☑	2491	*1127.70*	*3380.09*
☑	2492	*1137.01*	*3407.99*
☑	2494	*1140.64*	*3418.89*
☑	2495	*1151.46*	*3451.36*
☑	2496	*1159.44*	*3475.31*
☑	2498	*1207.99*	*3620.95*

Search Parameters

```
Type of search         : MS/MS Ion Search
Enzyme                 : Trypsin
Variable modifications : Carbamidomethyl (C),Oxidation (M)
Mass values            : Monoisotopic
Protein Mass           : Unrestricted
Peptide Mass Tolerance : ± 0.6 Da
Fragment Mass Tolerance: ± 0.3 Da
Max Missed Cleavages   : 2
Instrument type        : ESI-TRAP
Number of queries      : 2499
```

Mascot: http://www.matrixscience.com/
