# Supplementary material for: Macrophage-infectivity potentiator of Trypanosoma cruzi (TcMIP) is a new pro-type 1 immuno-stimulating protein for neonatal human cells and vaccines in mice
Source: Front Immunol. 2023 Mar 23;14:1138526. doi: 10.3389/fimmu.2023.1138526 (PMC10077492; doi:10.3389/fimmu.2023.1138526)
Supplement: Supplementary file 4 [file DataSheet_2.pdf]

Mascot Search Results

```
User             : GM
Email            : gabriel.mazzucchelli@ulg.ac.be
Search title     : Submitted from 081125-OGE33-precip-ZipTip-8467-sprot by Mascot Daemon on MASPEC39
MS data file     : F:\DATA\Archives\ESQUIRE\2008-Esquire\Adjuvac\081120-OGE33\081125-OGE33-precip-dig-zipTip_8467.mgf
Database         : Sprot 55.5 (389046 sequences; 139778124 residues)
Taxonomy         : Other Eukaryota (2925 sequences)
Timestamp        : 26 Nov 2008 at 15:33:40 GMT
Significant hits: CH60_TRYCR    Q95046|CH60_TRYCR Chaperonin HSP60, mitochondrial precursor - Trypanosoma cruzi
                  TBB_TRYBR     P04107|TBB_TRYBR Tubulin beta chain - Trypanosoma brucei rhodesiense
                  G3PG_TRYCR    P22513|G3PG_TRYCR Glyceraldehyde-3-phosphate dehydrogenase, glycosomal - Trypanosoma cruzi
                  FCA1_TRYCR    P07749|FCA1_TRYCR Flagellar calcium-binding protein - Trypanosoma cruzi
                  MIP_TRYCR     Q09734|MIP_TRYCR Macrophage infectivity potentiator precursor - Trypanosoma cruzi
                  CH60_TRYBB    Q37683|CH60_TRYBB Chaperonin HSP60, mitochondrial precursor - Trypanosoma brucei brucei
                  TBA_TRYCR     Q27352|TBA_TRYCR Tubulin alpha chain - Trypanosoma cruzi
                  EF1AC_PORPU   P50256|EF1AC_PORPU Elongation factor 1-alpha C - Porphyra purpurea
                  EF1A_TRYBB    P41166|EF1A_TRYBB Elongation factor 1-alpha - Trypanosoma brucei brucei
                  HSP70_BRELC   P16394|HSP70_BRELC Heat shock 70 kDa protein - Bremia lactucae (Lettuce downy mildew)
                  RLA3_TRYCR    P26795|RLA3_TRYCR 60S acidic ribosomal protein P2-B - Trypanosoma cruzi
                  HSP70_LEIMA   P14834|HSP70_LEIMA Heat shock 70 kDa protein - Leishmania major
                  H2A1_LEIIN    P27891|H2A1_LEIIN Histone H2A.1 - Leishmania infantum
                  HSP71_TRYCR   P20583|HSP71_TRYCR Heat shock 70 kDa protein, mitochondrial precursor - Trypanosoma cruzi
                  TBAD_PHYPO    P50258|TBAD_PHYPO Tubulin alpha-1A chain - Physarum polycephalum (Slime mold)
                  H2A_TRYCR     P35066|H2A_TRYCR Histone H2A - Trypanosoma cruzi
                  RLA2_TRYCR    P23632|RLA2_TRYCR 60S acidic ribosomal protein P2-A - Trypanosoma cruzi
                  RT07_ACACA    P46756|RT07_ACACA Mitochondrial ribosomal protein S7 - Acanthamoeba castellanii (Amoeba)
```

Probability Based Mowse Score

Ions score is -10*Log(P), where P is the probability that the observed match is a random event.
Individual ions scores > 23 indicate identity or extensive homology ($p<0.05$).
Protein scores are derived from ions scores as a non-probabilistic basis for ranking protein hits.

Score Distribution

Peptide Summary Report

Format As | Peptide Summary | Help

Significance threshold p< 0.05 | Max. number of hits AUTO

Standard scoring ○ | MudPIT scoring ◉ | Ions score cut-off 0 | Show sub-sets ☐

Show pop-ups ◉ | Suppress pop-ups ○ | Sort unassigned Decreasing Score | Require bold red ☐

Select All | Select None | Search Selected | ☐ **Error tolerant** | Archive Report

1. CH60_TRYCR **Mass:** 59374 **Score:** 471 **Queries matched:** 8

Q95046|CH60_TRYCR Chaperonin HSP60, mitochondrial precursor - Trypanosoma cruzi

☐ Check to include this hit in error tolerant search or archive report

Query	Observed	Mr(expt)	Mr(calc)	Delta	Miss	Score	Expect	Rank	Peptide
☑ 1482	557.86	1113.71	1113.64	0.07	0	94	2.6e-09	1	R.AVSAVATTLGPK.G
☑ 1673	602.37	1202.73	1202.58	0.15	0	55	2.6e-05	1	K.VGGGSEVEVNEK.K
☑ 1798	659.92	1317.82	1317.69	0.13	0	88	1.3e-08	1	R.NVIIEQSYGAPK.I
☑ 1806	670.37	1338.72	1338.61	0.12	0	87	1.2e-08	1	R.GLIDGETSDYNR.E
☑ 1871	728.93	1455.85	1455.80	0.05	0	102	4.6e-10	1	R.AVGVILQSVAEQSR.K
☑ 1964	804.04	1606.06	1605.92	0.14	0	22	0.031	1	R.AAVQEGIVPGGGVALLR.A
☑ 2020	846.95	1691.88	1691.81	0.07	0	108	8.6e-11	1	K.VLENNDVTVGYDAQR.D
☑ 2358	844.48	2530.41	2530.29	0.12	1	59	5.9e-06	1	R.KVTSTENIVQVATISANGDEELGR.L

2. TBB_TRYBR **Mass:** 49672 **Score:** 154 **Queries matched:** 2
P04107|TBB_TRYBR Tubulin beta chain - Trypanosoma brucei rhodesiense
☐ Check to include this hit in error tolerant search or archive report

	Query	Observed	Mr(expt)	Mr(calc)	Delta	Miss	Score	Expect	Rank	Peptide
☑	1807	671.36	1340.70	1340.64	0.06	0	81	4.5e-08	1	R.INVYFDEATGGR.Y
☑	2097	924.48	1846.94	1846.87	0.07	0	93	2.5e-09	1	R.EIVCVQAGQCGNQIGSK.F + 2 Carbamidomethyl (C)

Proteins matching the same set of peptides:
TBB_TRYCR **Mass:** 49541 **Score:** 154 **Queries matched:** 2
P08562|TBB_TRYCR Tubulin beta chain - Trypanosoma cruzi

3. G3PG_TRYCR **Mass:** 39036 **Score:** 115 **Queries matched:** 5
P22513|G3PG_TRYCR Glyceraldehyde-3-phosphate dehydrogenase, glycosomal - Trypanosoma cruzi
☐ Check to include this hit in error tolerant search or archive report

	Query	Observed	Mr(expt)	Mr(calc)	Delta	Miss	Score	Expect	Rank	Peptide
☑	684	412.82	823.64	823.43	0.21	0	1	2.8	1	K.AAAEGHLR.G
☑	1835	692.90	1383.78	1383.77	0.01	0	42	0.00037	1	R.AAAVNIIPSTTGAAK.A
☑	1837	692.94	1383.87	1383.77	0.10	0	(40)	0.00056	1	R.AAAVNIIPSTTGAAK.A
☑	2075	604.65	1810.92	1810.81	0.11	0	24	0.016	1	K.IVSWYDNEWGYSHR.V
☑	2262	747.89	2240.65	2240.06	0.59	0	67	2e-06	1	K.GILGYTDEELVSADFINDNR.S

4. FCA1_TRYCR **Mass:** 23721 **Score:** 114 **Queries matched:** 3
P07749|FCA1_TRYCR Flagellar calcium-binding protein - Trypanosoma cruzi
☐ Check to include this hit in error tolerant search or archive report

	Query	Observed	Mr(expt)	Mr(calc)	Delta	Miss	Score	Expect	Rank	Peptide
☑	1142	495.32	988.63	988.52	0.10	0	38	0.001	1	K.VEDPAALFK.E
☑	1845	707.87	1413.73	1413.59	0.14	0	49	9e-05	1	K.LDADGDPDNVPESA.-
☑	518	737.99	1473.97	1473.68	0.29	0	71	1.7e-06	1	K.GSEDFVEFLEFR.L

5. MIP_TRYCR   Mass: 22122   Score: 103   Queries matched: 2

Q09734|MIP_TRYCR Macrophage infectivity potentiator precursor - Trypanosoma cruzi

☐ Check to include this hit in error tolerant search or archive report

	Query	Observed	Mr(expt)	Mr(calc)	Delta	Miss	Score	Expect	Rank	Peptide
☑	108	508.80	1015.59	1015.58	0.01	0	49	0.00016	1	K.LPSGLVFQR.I
☑	1626	587.86	1173.71	1173.59	0.12	0	76	1.3e-07	1	R.TAEEVDEILR.K

6. CH60_TRYBB   Mass: 59492   Score: 88   Queries matched: 2

Q37683|CH60_TRYBB Chaperonin HSP60, mitochondrial precursor - Trypanosoma brucei brucei

☐ Check to include this hit in error tolerant search or archive report

Query	Observed	Mr(expt)	Mr(calc)	Delta	Miss	Score	Expect	Rank	Peptide
1755	632.39	1262.77	1262.62	0.15	0	2	4	2	K.SIATGTNPIDMK.R + Oxidation (M)
1798	659.92	1317.82	1317.69	0.13	0	88	1.3e-08	1	R.NVIIEQSYGAPK.I

7. TBA_TRYCR   Mass: 49696   Score: 82   Queries matched: 2

Q27352|TBA_TRYCR Tubulin alpha chain - Trypanosoma cruzi

☐ Check to include this hit in error tolerant search or archive report

	Query	Observed	Mr(expt)	Mr(calc)	Delta	Miss	Score	Expect	Rank	Peptide
☑	2035	858.56	1715.10	1714.91	0.19	0	82	3e-08	1	R.AVFLDLEPTVVDEIR.T
☑	2288	765.49	2293.46	2293.20	0.26	1	16	0.11	1	R.AVFLDLEPTVVDEIRTGTYR.Q

8. EF1AC_PORPU   Mass: 49142   Score: 70   Queries matched: 2

P50256|EF1AC_PORPU Elongation factor 1-alpha C - Porphyra purpurea

☐ Check to include this hit in error tolerant search or archive report

	Query	Observed	Mr(expt)	Mr(calc)	Delta	Miss	Score	Expect	Rank	Peptide
☑	1249	513.38	1024.74	1024.60	0.14	0	70	5e-07	1	K.IGGIGTVPVGR.V
☑	1674	602.88	1203.75	1203.65	0.09	2	11	0.5	1	K.KLEDSPKMIK.S + Oxidation (M)

9. EF1A_TRYBB **Mass:** 49003 **Score:** 70 **Queries matched:** 2

P41166|EF1A_TRYBB Elongation factor 1-alpha - Trypanosoma brucei brucei

☐ Check to include this hit in error tolerant search or archive report

	Query	Observed	Mr(expt)	Mr(calc)	Delta	Miss	Score	Expect	Rank	Peptide
	1249	513.38	1024.74	1024.60	0.14	0	70	5e-07	1	K.IGGIGTVPVGR.V
☑	931	**922.98**	**2765.92**	**2766.38**	**-0.46**	**1**	**1**	**18**	**1**	**R.MVPQKPMCVEVFNDYAPLGRFAVR.D**

---

10. HSP70_BRELC **Mass:** 74041 **Score:** 69 **Queries matched:** 2

P16394|HSP70_BRELC Heat shock 70 kDa protein - Bremia lactucae (Lettuce downy mildew)

☐ Check to include this hit in error tolerant search or archive report

	Query	Observed	Mr(expt)	Mr(calc)	Delta	Miss	Score	Expect	Rank	Peptide
☑	1710	**614.89**	**1227.76**	**1227.62**	**0.14**	**0**	**62**	**3.6e-06**	**1**	**R.VEIIANDQGNR.T**
☑	1895	**744.42**	**1486.82**	**1486.69**	**0.13**	**0**	**28**	**0.014**	**1**	**R.TTPSYVAFTDTER.L**

---

11. RLA3_TRYCR **Mass:** 10919 **Score:** 68 **Queries matched:** 1

P26795|RLA3_TRYCR 60S acidic ribosomal protein P2-B - Trypanosoma cruzi

☐ Check to include this hit in error tolerant search or archive report

	Query	Observed	Mr(expt)	Mr(calc)	Delta	Miss	Score	Expect	Rank	Peptide
☑	1490	**558.88**	**1115.75**	**1115.62**	**0.13**	**0**	**68**	**1e-06**	**1**	**R.SVATLVAEGAAK.M**

---

12. HSP70_LEIMA **Mass:** 56500 **Score:** 62 **Queries matched:** 1

P14834|HSP70_LEIMA Heat shock 70 kDa protein - Leishmania major

☐ Check to include this hit in error tolerant search or archive report

Query	Observed	Mr(expt)	Mr(calc)	Delta	Miss	Score	Expect	Rank	Peptide
1710	614.89	1227.76	1227.62	0.14	0	62	3.6e-06	1	R.LDIIANDQGNR.T

---

13. H2A1_LEIIN **Mass:** 13884 **Score:** 54 **Queries matched:** 1

P27891|H2A1_LEIIN Histone H2A.1 - Leishmania infantum

☐ Check to include this hit in error tolerant search or archive report

	Query	Observed	Mr(expt)	Mr(calc)	Delta	Miss	Score	Expect	Rank	Peptide
☑	1523	**563.87**	**1125.72**	**1125.57**	**0.16**	**0**	**54**	**2.4e-05**	**1**	**R.HDDDIGTLLK.N**

Proteins matching the same set of peptides:

H2A2_LEIIN **Mass:** 13811 **Score:** 54 **Queries matched:** 1

P27892|H2A2_LEIIN Histone H2A.2 - Leishmania infantum

---

14. HSP71_TRYCR **Mass:** 71102 **Score:** 51 **Queries matched:** 1

P20583|HSP71_TRYCR Heat shock 70 kDa protein, mitochondrial precursor - Trypanosoma cruzi

☐ Check to include this hit in error tolerant search or archive report

	Query	Observed	Mr(expt)	Mr(calc)	Delta	Miss	Score	Expect	Rank	Peptide
☑	1355	**532.84**	**1063.67**	**1063.53**	**0.14**	**0**	**51**	**5.9e-05**	**1**	**R.VLENTEGFR.A**

---

15. TBAD_PHYPO **Score:** 45 **Queries matched:** 2

P50258|TBAD_PHYPO Tubulin alpha-1A chain - Physarum polycephalum (Slime mold)

☐ Check to include this hit in error tolerant search or archive report

Query	Observed	Mr(expt)	Mr(calc)	Delta	Miss	Score	Expect	Rank	Peptide
2035	858.56	1715.10	1714.91	0.19	0	45	0.00015	2	R.AVFLDLEPTVIDEVR.T
2288	765.49	2293.46	2293.20	0.26	1	15	0.15	2	R.AVFLDLEPTVIDEVRTGTYR.Q

---

16. H2A_TRYCR **Mass:** 14357 **Score:** 40 **Queries matched:** 2

P35066|H2A_TRYCR Histone H2A - Trypanosoma cruzi

☐ Check to include this hit in error tolerant search or archive report

	Query	Observed	Mr(expt)	Mr(calc)	Delta	Miss	Score	Expect	Rank	Peptide
☑	952	**465.32**	**928.62**	**928.55**	**0.07**	**0**	**(24)**	**0.033**	**1**	**K.AGLIFPVGR.V**
☑	953	**465.38**	**928.76**	**928.55**	**0.21**	**0**	**35**	**0.0022**	**1**	**K.AGLIFPVGR.V**

---

17. RLA2_TRYCR **Mass:** 10505 **Score:** 32 **Queries matched:** 1

P23632|RLA2_TRYCR 60S acidic ribosomal protein P2-A - Trypanosoma cruzi

☐ Check to include this hit in error tolerant search or archive report

	Query	Observed	Mr(expt)	Mr(calc)	Delta	Miss	Score	Expect	Rank	Peptide
☑	230	586.25	1170.48	1170.49	-0.00	0	32	0.0095	1	K.DFDTVCTEGK.S + Carbamidomethyl (C)

---

18. RT07_ACACA **Mass:** 41586 **Score:** 26 **Queries matched:** 1

P46756|RT07_ACACA Mitochondrial ribosomal protein S7 - Acanthamoeba castellanii (Amoeba)

☐ Check to include this hit in error tolerant search or archive report

	Query	Observed	Mr(expt)	Mr(calc)	Delta	Miss	Score	Expect	Rank	Peptide
☑	1884	738.43	1474.85	1474.72	0.13	0	26	0.017	1	K.FIDCYLQFLEK.V + Carbamidomethyl (C)

Peptide matches not assigned to protein hits: (no details means no match)

	Query	Observed	Mr(expt)	Mr(calc)	Delta	Miss	Score	Expect	Rank	Peptide
☑	637	799.90	798.89	798.47	0.42	1	22	0.077	1	ARLTNPK
☑	1341	530.84	1059.66	1059.56	0.09	0	19	0.069	1	MGITPTALEK
☑	2362	850.50	2548.47	2548.34	0.13	0	19	0.053	1	VSFGGVAPAAGGATAAPAAAAAAAAPAAAAAK
☑	1902	750.99	1499.96	1499.85	0.11	2	19	0.089	1	ALDMKDVKQAILR
☑	1001	470.36	938.71	938.58	0.13	0	18	0.11	1	LVLPADLAK
☑	361	650.36	649.35	649.38	-0.03	1	15	0.58	1	KFIDK
☑	1804	667.91	1333.80	1333.75	0.06	2	14	0.22	1	SRVVYQIKGER
☑	35	430.72	859.42	859.43	-0.01	1	13	1.1	1	CPTDRLR
☑	1881	734.90	1467.78	1467.86	-0.08	2	13	0.32	1	ALSGGQKQRIAIAR
☑	1262	516.33	1030.64	1030.61	0.04	0	12	0.37	1	VIDLLAPYK
☑	1303	523.36	1044.70	1044.63	0.07	1	12	0.49	1	SLSALAKSIR
☑	1781	431.98	1292.91	1292.67	0.24	1	12	0.39	1	DAFMEILKTIP + Oxidation (M)
☑	1836	462.29	1383.84	1383.72	0.12	1	12	0.4	1	HGIEKVAEQVMK + Oxidation (M)
☑	1901	750.43	1498.85	1498.70	0.15	2	12	0.42	1	ECKTCSLTETKEK
☑	1696	609.39	1216.77	1216.70	0.06	1	11	0.51	1	LASETAKTLVGK
☑	578	773.95	772.95	773.43	-0.48	0	11	1.4	1	IADIQSK
☑	1782	647.49	1292.96	1292.71	0.25	0	11	0.52	1	NNPVLIGEPGVGK
☑	2454	999.11	2994.30	2994.57	-0.27	1	10	0.32	1	NMSHVSTGGGASLELLEGKTLPGVAILTDK

☑	210	573.28	1144.55	1144.63	-0.08	2	10	2	1	GLASANKKSNR
☑	1257	515.40	1028.78	1028.56	0.22	1	9	0.6	1	QVAEKAVER
☑	234	588.53	587.52	587.33	0.19	0	9	3.2	1	AIAGEK
☑	1890	494.23	1479.66	1479.71	-0.04	0	9	0.62	1	MVVNDTVADMLTR + Oxidation (M)
☑	179	555.03	1662.08	1661.88	0.20	2	9	2.1	1	KVPERPTVCNVRGAH
☑	1727	620.37	1238.72	1238.68	0.04	1	9	0.68	1	LLNYSARFQK
☑	961	466.29	930.57	930.50	0.07	0	9	0.94	1	DVITIENK
☑	2090	613.02	1836.04	1835.97	0.07	1	9	0.8	1	SFDLDVLKDSCALILK + Carbamidomethyl (C)
☑	1301	523.35	1044.68	1044.63	0.05	1	9	1	1	SLSALAKSIR
☑	1787	433.33	1296.96	1296.68	0.28	0	9	0.83	1	QTEVLVNGPGAGR
☑	1240	511.85	1021.69	1021.46	0.22	0	9	0.54	1	METLPESSL + Oxidation (M)
☑	2041	862.47	1722.92	1722.94	-0.02	2	8	0.65	1	SICKSLIGSVKSMGIK + Carbamidomethyl (C); Oxidation (M)
☑	1602	583.38	1164.76	1164.67	0.08	2	8	0.74	1	KIHEQNKIR
☑	1776	643.91	1285.80	1285.66	0.14	1	8	1.2	1	MIHIGNNTKSR + Oxidation (M)
☑	1158	496.85	991.68	991.69	-0.01	2	8	1.3	1	IHKKIILK
☑	63	464.06	1389.16	1389.64	-0.48	2	8	3.2	1	KGCGHCSNLRMK + Carbamidomethyl (C)
☑	1622	586.80	1171.58	1171.68	-0.10	2	8	1.1	1	LSPELKEKTK
☑	2091	613.36	1837.05	1836.85	0.20	1	8	1.1	1	IMAMREMILSDDEGAR
☑	1414	543.58	1085.15	1085.53	-0.37	1	8	2	1	RGDNPWVDK
☑	1335	529.87	1057.72	1057.64	0.08	1	7	1.2	1	LIDLKISEK
☑	1841	703.90	1405.79	1405.76	0.03	1	7	1.1	1	NEFTGVLTGKNVK
☑	1833	691.36	1380.71	1380.58	0.13	1	7	1.4	1	SPNCKWEGETCK
☑	1934	772.46	1542.90	1542.75	0.15	1	7	1.1	1	MAPPASASKAGAAEER
☑	1644	593.38	1184.75	1184.72	0.03	2	6	1.7	1	KLTNQVNKIK
☑	319	632.88	631.87	631.35	0.52	0	6	4.6	1	VLMNR
☑	1649	594.87	1187.72	1187.63	0.10	1	6	1.8	1	KLMYTFLEK + Oxidation (M)
☑	162	544.10	1086.19	1085.70	0.50	2	6	6.4	1	LILKTWKGK
☑	76	480.31	958.61	958.57	0.04	0	6	3.2	1	TVIELSGIK
☑	1838	692.95	1383.89	1383.79	0.10	2	6	1.6	1	MLLNLFKFSKK + Oxidation (M)
☑	1790	651.91	1301.80	1301.66	0.13	0	6	2	1	QSVNEPMITGVK
☑	340	643.86	642.85	642.41	0.45	0	6	4.6	1	LNGLVK
☑	323	635.91	1269.81	1269.74	0.07	1	5	7	1	GIEGIKIQISGR
☑	170	550.05	549.04	549.24	-0.19	0	5	5.2	1	DSNSK
☑	560	764.18	763.18	763.46	-0.28	1	5	2.1	1	NVKYLK

☑	2057	887.02	1772.04	1772.00	0.03	1	5	1.6	1	RIIENTGISSSIIIEK
☑	2081	608.03	1821.07	1820.99	0.09	2	5	2	1	LGMAGFDPKGPTVLKFK + Oxidation (M)
☑	1189	501.88	1001.74	1001.52	0.22	0	5	2.1	1	LFHSEEIK
☑	249	598.97	1195.92	1195.60	0.32	1	5	4.6	1	QYDMQVIRK + Oxidation (M)
☑	2032	572.04	1713.09	1712.99	0.10	2	5	1.3	1	IIAQRSKGEIPFNLK
☑	784	863.35	862.34	862.49	-0.14	1	5	2.2	1	VISSKSSR
☑	194	564.95	1691.83	1691.81	0.01	0	5	4.8	1	TGLQDAVQTGTGTMQGK
☑	2270	751.77	2252.28	2252.07	0.21	0	5	1.6	1	NNFFALNHEVCAVGYGVVDGK
☑	2359	845.49	2533.46	2533.31	0.15	1	5	1.6	1	VVIFLICLTMVLCAGGGFFSCKR + Carbamidomethyl (C)
☑	970	932.96	931.95	931.45	0.50	0	5	4.3	1	VSCLGSPNR
☑	152	534.09	533.08	533.30	-0.22	1	4	6.1	1	MGKAK
☑	2038	574.51	1720.50	1719.95	0.55	1	4	3	1	SSLIVGSKVSLNQNFK
☑	1494	1117.87	3350.60	3350.61	-0.01	2	4	5.7	1	VALCYGQMNEPPGARMRVGLTALTMAEHFR + 2 Oxidation (M)
☑	586	775.81	774.80	774.42	0.38	1	4	3.8	1	DNAKTVK
☑	1316	526.32	1050.62	1050.54	0.08	0	4	1.7	1	ISSMISELR + Oxidation (M)
☑	2024	848.93	1695.84	1695.95	-0.11	1	4	1.9	1	AVVVIGDENGKVGVGVGK
☑	739	843.30	842.29	842.38	-0.08	0	4	2.7	1	SHDEVEK
☑	164	545.88	544.87	544.33	0.53	0	4	4.3	1	GVTLR
☑	1753	631.35	1260.69	1260.72	-0.03	2	4	3	1	TFVDKGPTLKR
☑	2125	639.03	1914.06	1914.08	-0.02	2	4	2.3	1	KNLTQASDILAKAEAITK
☑	64	464.66	1390.96	1390.84	0.11	2	4	5.5	1	RLVVKNLQFFK
☑	1771	640.41	1278.81	1278.88	-0.07	1	4	2.6	1	LVNLLVIRVLK
☑	411	681.58	1361.15	1360.67	0.48	1	4	7.8	1	FNEPITEAERR
☑	201	568.90	1135.78	1135.63	0.15	2	4	5.2	1	RVLRGQDHR
☑	78	480.61	1438.80	1438.68	0.13	0	4	6.4	1	EFCQGATSSAPLTK
☑	2077	909.05	1816.09	1815.97	0.12	1	3	2.5	1	QLTKDEVGEIAEIMLK
☑	1692	608.31	1214.61	1214.69	-0.08	1	3	2.9	1	VITKAGEEQLK
☑	222	581.72	1161.43	1161.68	-0.24	1	3	7.2	1	SPISGIVKFSK
☑	2230	1070.55	2139.09	2139.13	-0.04	1	3	2.2	1	VSSGIGFLDHMLTALAKHSR
☑	1650	594.89	1187.76	1187.56	0.20	1	3	3.2	1	KLGEEPQECR
☑	264	603.12	1806.34	1806.77	-0.43	2	3	13	1	CACGGASPSNESCPRRR + Carbamidomethyl (C)
☑	750	848.89	847.88	848.45	-0.57	1	3	12	1	APQYSKR
☑	1639	591.44	1180.87	1180.57	0.30	0	3	2.5	1	LSTGIQQGQSY
☑	1608	1169.22	2336.42	2336.00	0.42	2	3	9.9	1	CQPNGDNSYSCVFEKDKTSSK

☑	1919	506.73	1517.16	1516.79	0.37	1	3	3.8	1	MAACLRDGEIIIR + Carbamidomethyl (C)
☑	705	829.74	828.73	828.52	0.21	2	3	5.1	1	GEIVKRK
☑	1897	746.64	1491.27	1490.81	0.46	2	3	5.8	1	KLVNDANKYLGEK
☑	2099	617.90	1850.67	1850.97	-0.30	1	3	3	1	FLHPYRSAEIFLSSGK
☑	2161	668.19	2001.54	2002.08	-0.53	1	3	3.9	1	SNKIICTIGPSSQSVEVLK
☑	1755	632.39	1262.77	1262.71	0.06	1	3	3.1	1	VVIAQKMIMSK + Oxidation (M)
☑	2126	640.04	1917.10	1917.06	0.05	1	3	2.7	1	AYKILKPTCHITVVMK + Carbamidomethyl (C); Oxidation (M)
☑	1006	941.04	940.03	940.43	-0.40	0	3	5	1	NSVSMIQF + Oxidation (M)
☑	920	459.37	916.73	916.57	0.16	2	3	4	1	TSVKINKK
☑	1856	719.88	1437.75	1437.82	-0.07	1	3	3.1	1	EILEGHKVLSVSK
☑	1926	763.97	1525.92	1525.86	0.07	1	3	3.7	1	ALEAALNTDLRALR
☑	50	445.27	888.52	888.46	0.06	1	2	7.7	1	MNLRNNK
☑	203	569.48	1136.94	1136.61	0.33	0	2	11	1	ISLIHFDHR
☑	2363	853.30	2556.87	2557.30	-0.44	0	2	4.5	1	LCTFTLGTVTLSEITSSLSSLPCK + Carbamidomethyl (C)
☑	563	765.97	764.96	765.43	-0.46	0	2	5	1	LLTEYK
☑	1330	1057.08	3168.22	3168.54	-0.31	0	2	12	1	EPTFSMGDDTPLPILSEKPHLIYDYFK + Oxidation (M)
☑	1260	516.10	1030.19	1030.57	-0.37	0	2	4.8	1	LEIAETLSR
☑	322	635.83	634.83	635.34	-0.51	1	2	8.1	1	KFEGR
☑	1703	1221.97	3662.90	3662.86	0.04	2	2	9.1	1	RDMADIISTFLAVLMGSFGLGFVAPSRTAFTESR
☑	803	873.14	872.13	872.53	-0.40	1	2	6.2	1	FRPLKGR
☑	919	917.00	915.99	915.50	0.50	1	2	16	1	AMKNPSLR
☑	1498	559.86	1117.70	1117.59	0.11	0	2	3.9	1	HGDFFAAILK
☑	349	646.72	1937.14	1936.83	0.31	0	2	13	1	MTDDNPAIVIDNGSGMCK + Carbamidomethyl (C)
☑	1152	991.58	1981.14	1981.06	0.09	0	2	11	1	SVIVVEPTLNLNECGLPK + Carbamidomethyl (C)
☑	1810	672.98	1343.95	1343.70	0.25	1	2	3.9	1	SKEVSPNLSQQK
☑	1623	586.90	1171.79	1171.64	0.15	1	2	4.3	1	NDEIDVVKIK
☑	169	548.35	1642.03	1641.94	0.09	1	2	9.2	1	VDLIIGSKINSTNLR
☑	331	639.10	1914.29	1914.88	-0.59	1	2	14	1	MLAFSDMNTGAGKIENGK + 2 Oxidation (M)
☑	1886	739.05	1476.08	1475.69	0.39	1	2	4.8	1	KSNQVAQDMYHR
☑	1096	487.30	972.59	972.58	0.01	1	2	3.3	1	ELLPFAKR
☑	2040	575.14	1722.39	1721.87	0.52	0	2	5.9	1	AMSIISGTAANMGIQIL + 2 Oxidation (M)
☑	1358	1064.89	2127.77	2128.15	-0.38	2	2	13	1	DAKLNKSDINEIVLVGGSTR
☑	1370	1067.94	3200.79	3200.60	0.19	1	2	10	1	VAFMRLTGMHFGGEELTYVQDTLTSLLR + Oxidation (M)
☑	1989	550.00	1646.97	1646.81	0.16	0	2	3.7	1	VTLVYGQMNEPPGAR + Oxidation (M)

☑	2343	823.86	2468.54	2469.14	-0.60	1	2	3.5	1	GDDMDIVDEAITYYKANVFYK
☑	1646	594.37	1186.72	1186.69	0.03	1	2	5	1	VIRVGINGFGR
☑	241	593.61	592.60	592.36	0.24	0	1	3.3	1	IIGYK
☑	888	905.08	2712.22	2712.24	-0.02	0	1	16	1	INDNILYSTSMACCALSSVYSISK + 2 Carbamidomethyl (C); Oxidation (M)
☑	1691	608.31	1214.60	1214.67	-0.06	1	1	4.6	1	APVPGYKVEAGK
☑	2244	725.06	2172.16	2171.98	0.18	1	1	3.8	1	MKHGNYSLATMNVCSNTTK + Carbamidomethyl (C); Oxidation (M)
☑	2330	805.16	2412.46	2412.21	0.25	0	1	3.4	1	DGVFSEVFHVNEADANLALLPR
☑	1874	731.93	1461.84	1461.87	-0.03	2	1	3.6	1	QLKQGLFMLLKK + Oxidation (M)
☑	140	529.81	1586.41	1586.80	-0.39	1	1	15	1	IDNDEEIEKIEIK
☑	1786	648.89	1295.77	1295.76	0.02	0	1	4.1	1	DLKPQNLLISR
☑	77	480.40	1438.19	1437.82	0.37	1	1	11	1	EILEGHKVLSVSK
☑	1690	1214.90	1213.89	1213.60	0.29	0	1	12	1	YGETNGLVFSK
☑	1281	1038.71	3113.11	3112.66	0.45	2	1	12	1	QGPIVCNLCTGLINTLENLLTTKGADKVK + Carbamidomethyl (C)
☑	1322	527.39	1052.76	1052.53	0.23	0	1	3.3	1	NPNAYSFLK
☑	1481	1114.69	3341.05	3340.64	0.41	0	1	12	1	MLMEGLSNVAAMALGAGIPTSSTIGPMLVDAFK + 3 Oxidation (M)
☑	1865	726.93	1451.84	1451.74	0.10	2	1	3.5	1	YHVEPRRGEGPR
☑	1543	1137.42	3409.25	3409.80	-0.56	2	1	13	1	MTIINKTAQTLFLTELVKGMSLTLDYFFR + Oxidation (M)
☑	245	597.07	1788.17	1788.77	-0.59	0	1	14	1	CESVSVWHMNANEAR + Carbamidomethyl (C)
☑	1873	487.68	1460.02	1459.85	0.17	1	1	5.8	1	LELLSFSNLIRR
☑	488	725.64	2173.91	2174.06	-0.14	0	1	13	1	FVQLCISMSDIIGFMELR + Carbamidomethyl (C); Oxidation (M)
☑	458	708.83	707.83	707.36	0.47	0	1	6.9	1	LDAYAR
☑	185	557.98	1113.95	1113.69	0.26	2	1	13	1	TLQKKIVER
☑	2068	900.02	1798.02	1797.87	0.15	2	1	3.9	1	LRSGATSVNMAMDKSAK + 2 Oxidation (M)
☑	2369	863.54	2587.60	2587.40	0.20	2	1	3.8	1	LFQQLNLGIHWRINSQTYTKK
☑	452	705.39	704.39	704.39	-0.00	0	1	13	1	MLNISK
☑	1045	955.20	2862.57	2862.44	0.13	2	1	15	1	VFDVQMMGAIILHQGKIAEMKTGEGK + 2 Oxidation (M)
☑	615	395.64	789.28	789.42	-0.15	1	1	5.7	1	KEIGESK
☑	2276	1129.62	2257.23	2257.20	0.04	1	1	4.1	1	VDLYDNARTAIVDIINPDIK
☑	1484	1114.96	2227.92	2228.02	-0.11	2	1	14	1	MSYCKIEAPYPEAEAGEKR + Carbamidomethyl (C)
☑	1231	509.84	1017.66	1017.49	0.17	1	1	5.3	1	MFRNHASR
☑	1868	727.42	1452.83	1452.85	-0.02	2	1	4.9	1	ISRRLGDLPGLTR
☑	608	787.48	2359.41	2359.02	0.39	1	1	15	1	GKCETHCPASFLHEHPHCR + 3 Carbamidomethyl (C)
☑	285	615.68	614.68	614.45	0.23	2	1	17	1	KLKVK
☑	2216	704.40	2110.17	2110.17	-0.01	2	1	4	1	TPSKAAVEAVLKAAGVAVDASR

☑	1958	529.01	1584.01	1583.91	0.10	2	1	4.6	1	KRLILPDPIYNSR
☑	691	825.42	2473.25	2473.20	0.05	1	1	13	1	APTEAPHRGGGGGGGHNTPAPTQPPR
☑	36	431.96	861.90	861.40	0.50	0	1	15	1	ADGLCDIR
☑	1410	543.35	1084.68	1084.63	0.05	0	1	5.8	1	NCLLATLIPK
☑	451	705.30	1408.59	1408.66	-0.07	0	1	13	1	MQTTINNGQTSSK
☑	94	499.71	1496.12	1495.88	0.24	1	1	11	1	NCGPIVLDALIKIK
☑	1713	616.32	1230.63	1230.64	-0.01	1	1	5.8	1	MSDPKPAKSVR + Oxidation (M)
☑	74	476.36	1426.04	1425.59	0.46	1	1	12	1	TACTQEADDGKCK + Carbamidomethyl (C)
☑	2018	846.14	1690.28	1689.73	0.55	0	1	7.8	1	GSAMNPVDHPHGGGEGR + Oxidation (M)
☑	1945	521.33	1560.98	1560.77	0.21	2	0	4.8	1	DFRGISYKTFDGR
☑	962	931.61	930.60	930.50	0.10	0	0	17	1	DVITIENK
☑	277	612.47	1834.39	1834.86	-0.47	1	0	14	1	VDIDFCKFEEPPSPR + Carbamidomethyl (C)
☑	1697	1217.92	2433.83	2434.20	-0.37	2	0	15	1	WIVDSRDNSTENRLNFLNNK
☑	24	418.31	834.61	834.39	0.22	0	0	11	1	MTNNLDK
☑	945	464.35	926.68	926.53	0.15	0	0	6.2	1	WVLVPASR
☑	418	684.10	2049.27	2049.86	-0.60	0	0	17	1	SYCIMYEGYLTYGGMAGR + Oxidation (M)
☑	1879	734.40	1466.78	1466.91	-0.13	1	0	4.9	1	ALITRLVLQVWR
☑	426	688.29	687.29	687.36	-0.07	0	0	6.9	1	QPTTNK
☑	1991	551.36	1651.05	1650.85	0.20	0	0	5.5	1	EHPNLVVLDLMMPK + Oxidation (M)
☑	1910	503.97	1508.88	1508.80	0.07	1	0	4.2	1	LFNFTSPWEKIK
☑	92	497.54	993.07	992.52	0.55	0	0	16	1	FTLGEGLEK
☑	231	587.95	586.95	587.31	-0.37	1	0	22	1	AREGR
☑	297	623.50	1867.49	1867.91	-0.41	0	0	18	1	GAGQFYCVIGSLFFFR + Carbamidomethyl (C)
☑	539	748.76	747.76	747.41	0.34	0	0	20	1	ATTTLNK
☑	1631	589.82	1177.63	1177.56	0.06	0	0	4.5	1	AIEQISQDMK + Oxidation (M)
☑	389	667.54	1999.60	1999.15	0.45	2	0	20	1	TTPGRIIFNKIIQESLVA
☑	2021	846.98	1691.95	1692.03	-0.08	1	0	5.2	1	VVQVPLTNINKNILK
☑	184	557.92	1670.74	1670.99	-0.25	2	0	17	1	GQVCSAVILRTIKGVK
☑	1625	1173.48	3517.43	3517.75	-0.31	1	0	17	1	ADFAFPSLRAFSIVVALDMQHGIGDGESIPWR + Oxidation (M)
☑	1736	1245.52	3733.53	3733.75	-0.22	1	0	15	1	FTMRQMFITCSFVASCMCLFLCGIPVFPGVAGK + 2 Carbamidomethyl (C); Oxidation (
☑	317	632.08	1893.20	1892.97	0.24	1	0	27	1	TTYSWKAFGSLAFSTVK
☑	1	*285.02*	*284.02*							
☑	2	*285.04*	*284.03*							
☑	3	*285.04*	*284.03*							

☑	4	285.04	284.03
☑	5	285.05	284.04
☑	6	285.05	284.04
☑	7	285.05	284.05
☑	8	285.07	284.06
☑	9	285.07	284.06
☑	10	285.07	284.06
☑	11	299.11	298.10
☑	12	329.22	328.21
☑	13	355.10	354.09
☑	14	369.79	368.78
☑	15	371.12	370.11
☑	16	371.12	370.12
☑	17	371.12	370.12
☑	18	371.15	370.14
☑	19	371.19	370.18
☑	20	387.91	386.90
☑	21	390.70	389.70
☑	22	396.96	395.95
☑	23	407.77	406.76
☑	25	420.66	419.66
☑	26	429.08	428.07
☑	27	429.09	428.08
☑	28	429.09	428.08
☑	29	429.09	428.09
☑	30	429.12	428.11
☑	31	429.13	428.13
☑	32	429.14	428.13
☑	33	429.15	428.14
☑	34	429.23	428.22
☑	37	432.86	431.85
☑	38	434.65	433.65
☑	39	435.25	434.25
☑	40	438.65	437.64

☑	41	*439.79*	*438.79*
☑	42	*441.80*	*440.79*
☑	43	*442.40*	*441.39*
☑	44	*443.08*	*442.07*
☑	45	*445.11*	*444.10*
☑	46	*445.15*	*444.14*
☑	47	*445.16*	*444.15*
☑	48	*445.18*	*444.17*
☑	49	*445.18*	*444.17*
☑	51	*446.33*	*445.33*
☑	52	*448.40*	*447.39*
☑	53	*451.79*	*450.78*
☑	54	*452.63*	*451.63*
☑	55	*453.76*	*452.75*
☑	56	*453.91*	*452.90*
☑	57	*456.19*	*455.19*
☑	58	*456.52*	*455.52*
☑	59	*457.31*	*456.31*
☑	60	*459.18*	*458.17*
☑	61	*463.92*	*462.91*
☑	62	*464.02*	*463.01*
☑	65	*465.61*	*464.60*
☑	66	*467.34*	*466.33*
☑	67	*469.02*	*468.01*
☑	68	*471.01*	*470.00*
☑	69	*471.91*	*470.90*
☑	70	*472.27*	*471.27*
☑	71	*473.00*	*471.99*
☑	72	*473.35*	*472.34*
☑	73	*474.59*	*473.58*
☑	75	*476.86*	*475.85*
☑	79	*480.68*	*479.67*
☑	80	*481.73*	*480.73*
☑	81	*481.83*	*480.82*

☑	82	483.70	482.69
☑	83	484.32	483.32
☑	84	486.29	485.28
☑	85	486.31	485.31
☑	86	486.63	485.62
☑	87	489.56	488.56
☑	88	490.33	489.32
☑	89	491.58	490.57
☑	90	492.15	491.14
☑	91	496.35	495.34
☑	93	499.31	498.30
☑	95	500.32	499.31
☑	96	501.50	500.50
☑	97	502.51	501.50
☑	98	502.56	501.55
☑	99	502.70	501.69
☑	100	503.31	502.31
☑	101	503.42	502.41
☑	102	503.54	502.53
☑	103	503.79	502.78
☑	104	504.06	503.05
☑	105	506.61	505.60
☑	106	508.15	507.14
☑	107	508.15	507.14
☑	109	509.19	508.18
☑	110	510.32	509.31
☑	111	510.45	509.44
☑	112	510.84	509.83
☑	113	511.22	510.21
☑	114	511.79	510.79
☑	115	511.99	510.99
☑	116	512.64	511.64
☑	117	513.61	512.60
☑	118	513.82	512.81

☑	119	*514.35*	*513.34*
☑	120	*514.96*	*513.95*
☑	121	*515.34*	*514.33*
☑	122	*515.42*	*514.41*
☑	123	*518.25*	*517.24*
☑	124	*518.72*	*517.71*
☑	125	*518.80*	*517.80*
☑	126	*519.36*	*518.36*
☑	127	*522.01*	*521.00*
☑	128	*522.85*	*521.84*
☑	129	*523.24*	*522.23*
☑	130	*523.48*	*522.47*
☑	131	*524.28*	*523.27*
☑	132	*524.53*	*523.53*
☑	133	*525.83*	*524.82*
☑	134	*526.89*	*525.89*
☑	135	*527.24*	*526.23*
☑	136	*527.62*	*526.61*
☑	137	*527.84*	*526.83*
☑	138	*528.08*	*527.07*
☑	139	*528.69*	*527.68*
☑	141	*530.02*	*529.02*
☑	142	*531.00*	*529.99*
☑	143	*531.34*	*530.34*
☑	144	*531.42*	*530.41*
☑	145	*531.84*	*530.83*
☑	146	*532.40*	*531.39*
☑	147	*532.43*	*531.42*
☑	148	*532.76*	*531.76*
☑	149	*533.47*	*532.47*
☑	150	*533.73*	*532.72*
☑	151	*533.95*	*532.94*
☑	153	*534.32*	*533.31*
☑	154	*535.81*	*534.81*

☑	155	536.97	535.96
☑	156	537.62	536.61
☑	157	537.64	536.63
☑	158	540.58	539.57
☑	159	542.33	541.33
☑	160	543.42	542.42
☑	161	543.62	542.62
☑	163	545.75	544.74
☑	165	546.47	545.46
☑	166	546.52	545.51
☑	167	546.57	545.56
☑	168	547.36	546.35
☑	171	551.30	550.29
☑	172	551.48	550.48
☑	173	552.46	551.45
☑	174	553.30	552.29
☑	175	553.73	552.72
☑	176	553.76	552.75
☑	177	554.50	553.49
☑	178	554.64	553.63
☑	180	555.54	554.53
☑	181	556.78	555.77
☑	182	557.41	556.40
☑	183	557.61	556.60
☑	186	559.29	558.29
☑	187	559.71	558.70
☑	188	560.09	559.08
☑	189	560.45	559.44
☑	190	561.28	560.27
☑	191	563.36	562.35
☑	192	563.47	562.46
☑	193	564.34	563.34
☑	195	565.78	564.77
☑	196	566.78	565.77

☑	197	567.06	566.05
☑	198	567.14	566.13
☑	199	567.18	566.17
☑	200	568.59	567.58
☑	202	569.20	568.19
☑	204	570.10	569.09
☑	205	570.14	569.14
☑	206	571.30	570.29
☑	207	571.40	570.40
☑	208	571.80	570.80
☑	209	573.28	572.27
☑	211	573.45	572.44
☑	212	573.53	572.52
☑	213	573.97	572.96
☑	214	575.38	574.37
☑	215	576.39	575.39
☑	216	576.40	575.40
☑	217	577.78	576.77
☑	218	578.04	577.04
☑	219	578.38	577.37
☑	220	578.90	577.89
☑	221	581.00	579.99
☑	223	582.26	581.26
☑	224	583.17	582.16
☑	225	583.48	582.48
☑	226	583.89	582.88
☑	227	584.36	583.35
☑	228	585.39	584.39
☑	229	586.03	585.02
☑	232	587.98	586.97
☑	233	588.38	587.37
☑	235	590.65	589.64
☑	236	591.27	590.27
☑	237	591.30	590.29

☑	238	591.87	590.86
☑	239	591.93	590.93
☑	240	592.01	591.00
☑	242	595.47	594.46
☑	243	595.93	594.93
☑	244	596.07	595.06
☑	246	597.33	596.32
☑	247	597.46	596.45
☑	248	598.04	597.04
☑	250	599.28	598.27
☑	251	599.31	598.30
☑	252	599.33	598.33
☑	253	599.48	598.47
☑	254	300.29	598.56
☑	255	599.85	598.84
☑	256	600.06	599.05
☑	257	600.07	599.07
☑	258	600.42	599.41
☑	259	600.47	599.46
☑	260	601.19	600.18
☑	261	601.50	600.49
☑	262	601.59	600.58
☑	263	602.99	601.98
☑	265	603.55	602.54
☑	266	603.87	602.86
☑	267	605.84	604.83
☑	268	607.72	606.71
☑	269	608.88	607.87
☑	270	609.14	608.13
☑	271	609.71	608.71
☑	272	610.30	609.29
☑	273	610.45	609.44
☑	274	610.46	609.45
☑	275	610.87	609.86

☑	276	612.37	611.36
☑	278	613.35	612.34
☑	279	613.38	612.37
☑	280	613.71	612.70
☑	281	613.77	612.76
☑	282	614.10	613.09
☑	283	614.14	613.13
☑	284	615.58	614.57
☑	286	617.92	616.92
☑	287	618.08	617.08
☑	288	618.11	617.10
☑	289	618.13	617.12
☑	290	619.64	618.64
☑	291	619.93	618.92
☑	292	622.57	621.56
☑	293	622.61	621.61
☑	294	623.25	622.24
☑	295	623.32	622.31
☑	296	623.47	622.47
☑	298	624.37	623.36
☑	299	624.82	623.81
☑	300	625.19	624.18
☑	301	625.47	624.47
☑	302	625.54	624.53
☑	303	625.89	624.89
☑	304	626.76	625.76
☑	305	627.24	626.23
☑	306	627.25	626.24
☑	307	628.50	627.49
☑	308	628.62	627.61
☑	309	628.95	627.94
☑	310	629.96	628.95
☑	311	630.04	629.03
☑	312	630.47	629.46

☑	313	630.78	629.77
☑	314	630.83	629.82
☑	315	632.00	630.99
☑	316	632.07	631.06
☑	318	632.48	631.47
☑	320	633.76	632.75
☑	321	633.92	632.91
☑	324	635.93	634.92
☑	325	636.52	635.52
☑	326	636.54	635.54
☑	327	637.03	636.02
☑	328	637.66	636.66
☑	329	637.80	636.79
☑	330	638.30	637.29
☑	332	639.55	638.55
☑	333	640.28	639.27
☑	334	641.16	640.15
☑	335	641.38	640.37
☑	336	641.61	640.61
☑	337	643.39	642.38
☑	338	643.43	642.43
☑	339	643.48	642.47
☑	341	644.57	643.57
☑	342	644.63	643.62
☑	343	645.17	644.16
☑	344	645.37	644.36
☑	345	645.80	644.79
☑	346	645.83	644.82
☑	347	646.62	645.61
☑	348	646.72	645.71
☑	350	646.82	645.81
☑	351	647.16	646.15
☑	352	647.40	646.39
☑	353	647.48	646.48

☑	354	648.05	647.04
☑	355	648.20	647.19
☑	356	649.18	648.17
☑	357	649.78	648.77
☑	358	649.83	648.83
☑	359	650.04	649.03
☑	360	650.31	649.30
☑	362	650.38	649.38
☑	363	650.40	649.39
☑	364	650.50	649.50
☑	365	650.66	649.65
☑	366	650.77	649.76
☑	367	652.86	651.85
☑	368	654.56	653.55
☑	369	654.72	653.71
☑	370	655.13	654.12
☑	371	655.50	654.50
☑	372	655.53	654.52
☑	373	655.62	654.61
☑	374	655.84	654.83
☑	375	656.59	655.59
☑	376	658.23	657.23
☑	377	658.40	657.39
☑	378	658.69	657.68
☑	379	659.06	658.05
☑	380	659.43	658.43
☑	381	661.02	660.01
☑	382	661.02	660.01
☑	383	661.31	660.30
☑	384	663.08	662.07
☑	385	663.25	662.25
☑	386	665.41	664.41
☑	387	665.70	664.69
☑	388	667.46	666.45

☑	390	668.17	667.16
☑	391	669.30	668.29
☑	392	669.89	668.89
☑	393	671.38	670.37
☑	394	672.20	671.19
☑	395	672.39	671.38
☑	396	672.66	671.65
☑	397	673.58	672.57
☑	398	673.96	672.95
☑	399	674.10	673.09
☑	400	674.50	673.49
☑	401	675.42	674.42
☑	402	676.06	675.06
☑	403	677.55	676.54
☑	404	678.13	677.12
☑	405	678.58	677.57
☑	406	678.80	677.79
☑	407	679.39	678.39
☑	408	680.49	679.48
☑	409	681.02	680.02
☑	410	681.37	680.36
☑	412	682.30	681.30
☑	413	682.46	681.45
☑	414	683.04	682.03
☑	415	683.31	682.31
☑	416	683.78	682.77
☑	417	683.92	682.91
☑	419	685.53	684.52
☑	420	685.72	684.71
☑	421	685.86	684.85
☑	422	686.10	685.09
☑	423	686.98	685.97
☑	424	687.85	686.85
☑	425	688.07	687.06

☑	427	689.26	688.25
☑	428	691.35	690.34
☑	429	692.15	691.14
☑	430	693.80	692.79
☑	431	694.05	693.05
☑	432	694.71	693.70
☑	433	694.87	693.86
☑	434	695.17	694.16
☑	435	695.92	694.92
☑	436	696.03	695.02
☑	437	697.29	696.28
☑	438	697.34	696.33
☑	439	698.14	697.13
☑	440	698.71	697.70
☑	441	699.02	698.02
☑	442	699.41	698.40
☑	443	699.68	698.68
☑	444	700.23	699.22
☑	445	700.57	699.57
☑	446	701.58	700.57
☑	447	702.42	701.41
☑	448	703.21	702.20
☑	449	705.16	704.15
☑	450	705.30	704.29
☑	453	705.61	704.61
☑	454	705.87	704.86
☑	455	707.13	706.13
☑	456	708.75	707.75
☑	457	708.78	707.77
☑	459	709.36	708.35
☑	460	709.44	708.43
☑	461	710.22	709.21
☑	462	710.38	709.38
☑	463	710.57	709.56

☑	464	714.41	713.41
☑	465	714.43	713.42
☑	466	714.57	713.56
☑	467	714.84	713.83
☑	468	715.51	714.51
☑	469	715.63	714.63
☑	470	715.99	714.98
☑	471	717.85	716.84
☑	472	717.90	716.89
☑	473	717.90	716.90
☑	474	718.11	717.10
☑	475	718.29	717.28
☑	476	718.56	717.55
☑	477	718.77	717.76
☑	478	720.34	719.33
☑	479	360.69	719.36
☑	480	720.48	719.47
☑	481	720.90	719.90
☑	482	721.00	720.00
☑	483	722.30	721.29
☑	484	722.38	721.37
☑	485	722.71	721.70
☑	486	723.52	722.51
☑	487	723.66	722.66
☑	489	725.72	724.71
☑	490	726.50	725.50
☑	491	726.72	725.71
☑	492	726.80	725.79
☑	493	726.96	725.95
☑	494	727.83	726.82
☑	495	728.29	727.29
☑	496	728.42	727.41
☑	497	729.01	728.00
☑	498	729.17	728.16

☑	499	729.90	728.89
☑	500	730.40	729.40
☑	501	730.75	729.74
☑	502	730.77	729.77
☑	503	730.90	729.89
☑	504	731.07	730.07
☑	505	732.22	731.21
☑	506	732.23	731.22
☑	507	732.35	731.35
☑	508	732.95	731.95
☑	509	733.14	732.14
☑	510	733.33	732.32
☑	511	734.45	733.44
☑	512	734.56	733.55
☑	513	734.86	733.85
☑	514	735.66	734.65
☑	515	735.90	734.90
☑	516	736.98	735.97
☑	517	737.12	736.11
☑	519	738.00	736.99
☑	520	738.16	737.15
☑	521	738.38	737.37
☑	522	738.84	737.84
☑	523	739.41	738.40
☑	524	739.61	738.60
☑	525	739.63	738.62
☑	526	739.95	738.94
☑	527	740.14	739.13
☑	528	742.48	741.48
☑	529	743.20	742.20
☑	530	743.21	742.20
☑	531	743.58	742.57
☑	532	744.43	743.42
☑	533	745.08	744.07

☑	534	745.57	744.56
☑	535	745.84	744.83
☑	536	746.61	745.60
☑	537	747.60	746.59
☑	538	748.33	747.32
☑	540	749.08	748.07
☑	541	749.92	748.91
☑	542	750.32	749.31
☑	543	750.54	749.53
☑	544	751.07	750.06
☑	545	376.60	751.18
☑	546	754.02	753.02
☑	547	755.42	754.41
☑	548	755.46	754.45
☑	549	755.71	754.70
☑	550	756.22	755.22
☑	551	757.38	756.37
☑	552	758.92	757.91
☑	553	758.95	757.95
☑	554	759.89	758.88
☑	555	760.77	759.76
☑	556	762.70	761.70
☑	557	762.98	761.97
☑	558	763.41	762.41
☑	559	764.08	763.07
☑	561	764.49	763.48
☑	562	765.83	764.82
☑	564	766.12	765.11
☑	565	766.52	765.51
☑	566	766.98	765.97
☑	567	767.38	766.37
☑	568	768.39	767.38
☑	569	768.67	767.66
☑	570	769.32	768.32

☑	571	770.35	769.35
☑	572	770.84	769.83
☑	573	771.10	770.09
☑	574	772.11	771.10
☑	575	772.46	771.45
☑	576	773.51	772.51
☑	577	773.54	772.54
☑	579	773.97	772.96
☑	580	774.11	773.10
☑	581	774.45	773.45
☑	582	774.51	773.50
☑	583	774.59	773.58
☑	584	775.35	774.34
☑	585	775.71	774.70
☑	587	777.26	776.25
☑	588	777.36	776.35
☑	589	777.37	776.37
☑	590	390.75	779.50
☑	591	781.05	780.05
☑	592	782.18	781.18
☑	593	782.26	781.25
☑	594	782.43	781.42
☑	595	782.45	781.44
☑	596	782.55	781.54
☑	597	783.71	782.70
☑	598	784.04	783.03
☑	599	784.28	783.27
☑	600	784.38	783.37
☑	601	784.92	783.91
☑	602	785.33	784.32
☑	603	785.62	784.61
☑	604	786.07	785.06
☑	605	786.26	785.26
☑	606	787.20	786.19

☑	607	787.21	786.20
☑	609	787.72	786.71
☑	610	787.81	786.81
☑	611	787.83	786.82
☑	612	788.72	787.71
☑	613	789.89	788.88
☑	614	789.89	788.88
☑	616	791.09	790.08
☑	617	792.48	791.47
☑	618	792.50	791.49
☑	619	792.84	791.83
☑	620	793.02	792.02
☑	621	793.34	792.33
☑	622	793.42	792.41
☑	623	793.42	792.41
☑	624	793.47	792.46
☑	625	793.81	792.80
☑	626	793.88	792.87
☑	627	794.30	793.29
☑	628	795.15	794.15
☑	629	795.53	794.52
☑	630	796.56	795.55
☑	631	797.11	796.10
☑	632	797.53	796.52
☑	633	798.14	797.13
☑	634	798.22	797.21
☑	635	799.19	798.18
☑	636	799.52	798.51
☑	638	800.35	799.34
☑	639	801.39	800.38
☑	640	803.23	802.23
☑	641	803.72	802.71
☑	642	803.81	802.80
☑	643	804.23	803.22

☑	644	805.08	804.07
☑	645	805.57	804.56
☑	646	805.89	804.88
☑	647	806.05	805.04
☑	648	806.49	805.49
☑	649	807.72	806.71
☑	650	807.91	806.90
☑	651	808.28	807.27
☑	652	808.53	807.52
☑	653	808.76	807.75
☑	654	808.91	807.90
☑	655	808.97	807.96
☑	656	809.89	808.88
☑	657	810.54	809.54
☑	658	810.94	809.93
☑	659	406.52	811.03
☑	660	812.36	811.35
☑	661	813.16	812.16
☑	662	813.27	812.26
☑	663	813.76	812.76
☑	664	814.64	813.64
☑	665	814.71	813.70
☑	666	814.87	813.86
☑	667	815.14	814.14
☑	668	815.36	814.35
☑	669	408.25	814.49
☑	670	815.81	814.81
☑	671	815.99	814.99
☑	672	816.15	815.15
☑	673	816.48	815.47
☑	674	816.49	815.49
☑	675	817.01	816.00
☑	676	817.34	816.33
☑	677	819.76	818.76

☑	678	820.30	819.30
☑	679	820.91	819.91
☑	680	821.02	820.02
☑	681	821.49	820.48
☑	682	821.96	820.95
☑	683	823.89	822.89
☑	685	824.90	823.90
☑	686	824.96	823.95
☑	687	825.27	824.26
☑	688	825.33	824.32
☑	689	825.37	824.36
☑	690	825.39	824.39
☑	692	825.43	824.43
☑	693	825.57	824.57
☑	694	825.78	824.77
☑	695	825.98	824.97
☑	696	827.36	826.35
☑	697	827.83	826.82
☑	698	827.97	826.96
☑	699	828.36	827.36
☑	700	828.39	827.39
☑	701	828.47	827.46
☑	702	828.77	827.77
☑	703	829.46	828.45
☑	704	829.49	828.48
☑	706	829.87	828.86
☑	707	830.83	829.83
☑	708	831.12	830.11
☑	709	416.26	830.50
☑	710	831.81	830.80
☑	711	832.09	831.08
☑	712	832.61	831.60
☑	713	832.95	831.94
☑	714	833.06	832.06

715	834.00	832.99
716	834.18	833.17
717	834.58	833.57
718	417.93	833.84
719	835.27	834.26
720	836.36	835.35
721	836.65	835.64
722	838.35	837.34
723	838.77	837.76
724	839.29	838.29
725	839.43	838.42
726	839.75	838.74
727	840.00	839.00
728	840.15	839.14
729	840.51	839.50
730	840.83	839.82
731	841.87	840.86
732	842.10	841.09
733	421.59	841.17
734	842.47	841.46
735	421.83	841.64
736	842.72	841.71
737	421.94	841.86
738	842.96	841.95
740	843.74	842.73
741	844.58	843.57
742	845.34	844.33
743	845.78	844.77
744	846.61	845.60
745	846.97	845.96
746	847.15	846.14
747	848.26	847.25
748	848.57	847.56
749	848.78	847.78

☑	751	849.02	848.01
☑	752	849.40	848.39
☑	753	849.69	848.69
☑	754	850.80	849.79
☑	755	851.63	850.62
☑	756	851.75	850.74
☑	757	852.48	851.47
☑	758	852.60	851.59
☑	759	852.96	851.95
☑	760	427.79	853.56
☑	761	854.71	853.70
☑	762	854.82	853.81
☑	763	855.00	853.99
☑	764	855.23	854.22
☑	765	855.44	854.43
☑	766	855.58	854.57
☑	767	856.34	855.33
☑	768	857.27	856.26
☑	769	857.36	856.35
☑	770	857.39	856.38
☑	771	857.62	856.62
☑	772	857.67	856.66
☑	773	857.71	856.71
☑	774	858.03	857.03
☑	775	858.19	857.18
☑	776	858.58	857.57
☑	777	858.75	857.74
☑	778	859.42	858.41
☑	779	860.36	859.35
☑	780	860.53	859.52
☑	781	860.98	859.97
☑	782	861.27	860.26
☑	783	861.43	860.42
☑	785	863.36	862.35

☑	786	863.50	862.49
☑	787	863.80	862.80
☑	788	865.24	864.23
☑	789	865.27	864.27
☑	790	867.01	866.01
☑	791	867.17	866.16
☑	792	867.33	866.33
☑	793	870.12	869.12
☑	794	870.38	869.37
☑	795	871.02	870.01
☑	796	871.20	870.19
☑	797	871.46	870.45
☑	798	871.70	870.70
☑	799	871.83	870.82
☑	800	871.84	870.83
☑	801	871.94	870.93
☑	802	872.51	871.51
☑	804	873.36	872.35
☑	805	873.45	872.44
☑	806	437.29	872.56
☑	807	873.65	872.64
☑	808	874.03	873.03
☑	809	874.05	873.04
☑	810	874.09	873.08
☑	811	874.15	873.14
☑	812	874.16	873.15
☑	813	874.23	873.22
☑	814	874.30	873.29
☑	815	874.87	873.86
☑	816	875.56	874.56
☑	817	876.99	875.98
☑	818	877.23	876.22
☑	819	877.24	876.23
☑	820	877.33	876.32

☑	821	877.33	876.33
☑	822	877.63	876.62
☑	823	439.34	876.66
☑	824	877.67	876.66
☑	825	879.06	878.05
☑	826	879.54	878.53
☑	827	879.74	878.73
☑	828	880.22	879.21
☑	829	880.27	879.26
☑	830	880.51	879.51
☑	831	881.48	880.48
☑	832	881.86	880.85
☑	833	881.91	880.90
☑	834	882.18	881.17
☑	835	882.94	881.93
☑	836	883.92	882.91
☑	837	883.93	882.93
☑	838	884.54	883.54
☑	839	884.99	883.98
☑	840	885.30	884.29
☑	841	885.36	884.35
☑	842	886.83	885.83
☑	843	887.45	886.45
☑	844	888.04	887.03
☑	845	888.79	887.78
☑	846	888.92	887.91
☑	847	891.36	890.35
☑	848	891.84	890.83
☑	849	892.21	891.20
☑	850	892.28	891.27
☑	851	892.70	891.69
☑	852	893.06	892.06
☑	853	893.25	892.24
☑	854	893.28	892.28

☑	855	893.30	892.30
☑	856	893.68	892.67
☑	857	893.80	892.79
☑	858	893.90	892.89
☑	859	895.61	894.61
☑	860	895.65	894.64
☑	861	895.75	894.74
☑	862	895.95	894.94
☑	863	896.00	894.99
☑	864	896.43	895.42
☑	865	896.49	895.48
☑	866	897.13	896.12
☑	867	897.27	896.26
☑	868	897.54	896.53
☑	869	898.41	897.40
☑	870	450.15	898.29
☑	871	899.55	898.54
☑	872	900.21	899.20
☑	873	900.48	899.47
☑	874	900.73	899.72
☑	875	900.85	899.84
☑	876	901.00	899.99
☑	877	902.67	901.67
☑	878	902.69	901.68
☑	879	451.99	901.97
☑	880	902.98	901.97
☑	881	903.46	902.45
☑	882	903.57	902.56
☑	883	903.58	902.58
☑	884	903.86	902.85
☑	885	904.65	903.64
☑	886	904.80	903.79
☑	887	904.88	903.87
☑	889	905.16	904.15

☑	890	905.18	904.17
☑	891	906.02	905.02
☑	892	906.53	905.52
☑	893	453.87	905.72
☑	894	907.00	905.99
☑	895	907.82	906.81
☑	896	908.05	907.04
☑	897	908.42	907.41
☑	898	908.44	907.43
☑	899	908.85	907.84
☑	900	456.36	910.71
☑	901	912.18	911.17
☑	902	913.14	912.13
☑	903	913.18	912.17
☑	904	913.33	912.32
☑	905	913.41	912.41
☑	906	913.44	912.43
☑	907	913.58	912.57
☑	908	914.82	913.81
☑	909	914.99	913.98
☑	910	915.28	914.27
☑	911	915.47	914.47
☑	912	916.13	915.12
☑	913	916.14	915.13
☑	914	916.15	915.15
☑	915	916.43	915.42
☑	916	916.68	915.68
☑	917	916.84	915.83
☑	918	916.90	915.89
☑	921	918.40	917.39
☑	922	919.50	918.49
☑	923	919.52	918.51
☑	924	920.34	919.33
☑	925	921.30	920.29

☑	926	461.22	920.43
☑	927	461.29	920.57
☑	928	921.79	920.78
☑	929	922.27	921.26
☑	930	922.49	921.48
☑	932	923.02	922.01
☑	933	924.15	923.14
☑	934	924.27	923.26
☑	935	924.35	923.34
☑	936	462.90	923.79
☑	937	925.60	924.59
☑	938	463.30	924.59
☑	939	925.68	924.67
☑	940	925.71	924.70
☑	941	925.84	924.83
☑	942	926.14	925.13
☑	943	926.55	925.54
☑	944	927.63	926.63
☑	946	927.77	926.76
☑	947	928.71	927.70
☑	948	928.97	927.96
☑	949	929.22	928.21
☑	950	929.38	928.38
☑	951	929.58	928.57
☑	954	930.02	929.01
☑	955	930.29	929.29
☑	956	930.64	929.64
☑	957	930.81	929.81
☑	958	930.92	929.91
☑	959	931.16	930.15
☑	960	931.35	930.34
☑	963	931.86	930.85
☑	964	931.86	930.85
☑	965	931.90	930.89

☑	966	931.90	930.90
☑	967	932.20	931.20
☑	968	932.39	931.39
☑	969	932.94	931.93
☑	971	933.25	932.24
☑	972	933.32	932.31
☑	973	933.36	932.36
☑	974	933.54	932.54
☑	975	933.63	932.63
☑	976	933.67	932.67
☑	977	933.71	932.70
☑	978	933.77	932.76
☑	979	933.99	932.98
☑	980	934.17	933.16
☑	981	935.11	934.10
☑	982	935.65	934.64
☑	983	935.73	934.72
☑	984	936.24	935.23
☑	985	936.35	935.34
☑	986	936.55	935.54
☑	987	936.59	935.58
☑	988	468.93	935.85
☑	989	936.88	935.87
☑	990	937.11	936.10
☑	991	937.83	936.82
☑	992	937.86	936.85
☑	993	938.04	937.03
☑	994	938.22	937.21
☑	995	938.52	937.51
☑	996	938.61	937.60
☑	997	938.91	937.90
☑	998	469.99	937.97
☑	999	939.05	938.05
☑	1000	939.71	938.70

☑	1002	940.04	939.04
☑	1003	940.28	939.27
☑	1004	470.82	939.63
☑	1005	940.70	939.69
☑	1007	942.14	941.13
☑	1008	942.71	941.70
☑	1009	942.89	941.89
☑	1010	942.90	941.90
☑	1011	943.19	942.18
☑	1012	943.34	942.33
☑	1013	943.48	942.47
☑	1014	944.48	943.48
☑	1015	944.48	943.48
☑	1016	944.82	943.81
☑	1017	944.87	943.86
☑	1018	946.30	945.29
☑	1019	946.30	945.29
☑	1020	946.39	945.39
☑	1021	946.44	945.44
☑	1022	946.45	945.44
☑	1023	946.52	945.51
☑	1024	948.02	947.01
☑	1025	948.21	947.21
☑	1026	948.49	947.48
☑	1027	948.49	947.48
☑	1028	948.78	947.77
☑	1029	949.21	948.21
☑	1030	949.60	948.60
☑	1031	951.00	949.99
☑	1032	951.04	950.03
☑	1033	951.04	950.04
☑	1034	951.17	950.16
☑	1035	951.52	950.51
☑	1036	951.89	950.89

☑	1037	951.90	950.89
☑	1038	951.90	950.89
☑	1039	952.74	951.73
☑	1040	952.97	951.96
☑	1041	953.66	952.65
☑	1042	954.45	953.44
☑	1043	955.11	954.10
☑	1044	955.19	954.18
☑	1046	955.29	954.28
☑	1047	956.04	955.04
☑	1048	956.37	955.36
☑	1049	956.69	955.68
☑	1050	956.70	955.70
☑	1051	478.91	955.81
☑	1052	478.93	955.85
☑	1053	957.00	955.99
☑	1054	957.24	956.23
☑	1055	957.36	956.35
☑	1056	957.40	956.39
☑	1057	957.69	956.69
☑	1058	958.37	957.36
☑	1059	480.01	958.01
☑	1060	959.72	958.72
☑	1061	960.24	959.23
☑	1062	960.38	959.37
☑	1063	960.50	959.50
☑	1064	961.39	960.39
☑	1065	961.96	960.95
☑	1066	962.03	961.02
☑	1067	481.54	961.07
☑	1068	962.58	961.57
☑	1069	962.87	961.87
☑	1070	963.22	962.21
☑	1071	963.22	962.21

☑	1072	963.23	962.22
☑	1073	482.50	962.98
☑	1074	482.61	963.21
☑	1075	482.98	963.95
☑	1076	965.54	964.53
☑	1077	966.99	965.98
☑	1078	967.12	966.11
☑	1079	967.49	966.48
☑	1080	967.72	966.71
☑	1081	967.94	966.93
☑	1082	968.05	967.04
☑	1083	968.40	967.40
☑	1084	484.72	967.42
☑	1085	968.53	967.52
☑	1086	484.84	967.67
☑	1087	485.25	968.49
☑	1088	971.31	970.30
☑	1089	486.20	970.40
☑	1090	971.57	970.56
☑	1091	971.65	970.64
☑	1092	972.20	971.19
☑	1093	972.72	971.71
☑	1094	973.11	972.10
☑	1095	973.21	972.20
☑	1097	973.93	972.92
☑	1098	974.16	973.15
☑	1099	974.34	973.33
☑	1100	974.56	973.55
☑	1101	975.43	974.42
☑	1102	975.85	974.84
☑	1103	976.21	975.20
☑	1104	976.23	975.22
☑	1105	976.36	975.35
☑	1106	976.58	975.57

☑	1107	977.18	976.18
☑	1108	977.51	976.51
☑	1109	978.22	977.21
☑	1110	489.78	977.55
☑	1111	978.66	977.65
☑	1112	490.01	978.01
☑	1113	980.19	979.18
☑	1114	490.61	979.20
☑	1115	980.60	979.59
☑	1116	980.84	979.83
☑	1117	981.63	980.63
☑	1118	981.65	980.64
☑	1119	981.73	980.72
☑	1120	491.84	981.67
☑	1121	983.08	982.07
☑	1122	983.59	982.58
☑	1123	983.94	982.93
☑	1124	984.53	983.53
☑	1125	985.06	984.05
☑	1126	985.17	984.16
☑	1127	985.25	984.24
☑	1128	985.44	984.43
☑	1129	987.08	986.08
☑	1130	987.21	986.20
☑	1131	494.35	986.69
☑	1132	987.72	986.71
☑	1133	987.86	986.85
☑	1134	987.91	986.90
☑	1135	988.09	987.08
☑	1136	494.58	987.15
☑	1137	988.38	987.37
☑	1138	988.40	987.39
☑	1139	988.83	987.82
☑	1140	494.99	987.97

☑	1141	989.61	988.61
☑	1143	495.33	988.65
☑	1144	990.02	989.01
☑	1145	990.04	989.03
☑	1146	495.82	989.63
☑	1147	990.71	989.70
☑	1148	990.71	989.70
☑	1149	495.93	989.85
☑	1150	991.38	990.38
☑	1151	991.57	990.57
☑	1153	991.70	990.69
☑	1154	991.71	990.70
☑	1155	992.08	991.07
☑	1156	992.16	991.15
☑	1157	992.58	991.57
☑	1159	992.73	991.73
☑	1160	993.43	992.42
☑	1161	993.45	992.44
☑	1162	993.55	992.55
☑	1163	993.76	992.75
☑	1164	993.79	992.79
☑	1165	993.87	992.86
☑	1166	994.21	993.21
☑	1167	994.84	993.84
☑	1168	995.05	994.04
☑	1169	995.86	994.85
☑	1170	996.00	994.99
☑	1171	996.55	995.54
☑	1172	996.62	995.61
☑	1173	997.02	996.02
☑	1174	998.11	997.10
☑	1175	998.33	997.33
☑	1176	998.40	997.39
☑	1177	998.42	997.41

☑	1178	999.00	997.99
☑	1179	999.57	998.56
☑	1180	999.61	998.60
☑	1181	1000.14	999.14
☑	1182	1000.86	999.85
☑	1183	1001.14	1000.13
☑	1184	1001.60	1000.60
☑	1185	1001.83	1000.82
☑	1186	1002.02	1001.01
☑	1187	1002.02	1001.01
☑	1188	1002.04	1001.03
☑	1190	1002.77	1001.77
☑	1191	1002.79	1001.78
☑	1192	1004.17	1003.17
☑	1193	1004.39	1003.39
☑	1194	1004.77	1003.76
☑	1195	1005.13	1004.12
☑	1196	503.16	1004.30
☑	1197	1005.57	1004.56
☑	1198	503.29	1004.56
☑	1199	1005.99	1004.98
☑	1200	1006.40	1005.39
☑	1201	1006.84	1005.83
☑	1202	1007.35	1006.34
☑	1203	1007.55	1006.54
☑	1204	1008.36	1007.35
☑	1205	1008.76	1007.76
☑	1206	1008.90	1007.89
☑	1207	1009.63	1008.63
☑	1208	1009.92	1008.91
☑	1209	1010.52	1009.52
☑	1210	1011.24	1010.23
☑	1211	1012.44	1011.44
☑	1212	1012.53	1011.52

☑	1213	1013.02	1012.01
☑	1214	1013.13	1012.13
☑	1215	1013.66	1012.66
☑	1216	507.38	1012.75
☑	1217	1014.08	1013.07
☑	1218	1014.15	1013.15
☑	1219	1014.28	1013.27
☑	1220	1014.66	1013.65
☑	1221	1015.02	1014.02
☑	1222	1015.20	1014.19
☑	1223	1015.36	1014.35
☑	1224	1015.48	1014.47
☑	1225	508.57	1015.13
☑	1226	1017.21	1016.20
☑	1227	1017.75	1016.75
☑	1228	1018.27	1017.26
☑	1229	1018.33	1017.32
☑	1230	1018.49	1017.48
☑	1232	1019.05	1018.04
☑	1233	1019.41	1018.40
☑	1234	1020.45	1019.45
☑	1235	1020.48	1019.47
☑	1236	1020.49	1019.48
☑	1237	1021.18	1020.17
☑	1238	1021.20	1020.19
☑	1239	1022.28	1021.27
☑	1241	1022.83	1021.82
☑	1242	1022.84	1021.84
☑	1243	1023.12	1022.11
☑	1244	1023.28	1022.27
☑	1245	1023.41	1022.40
☑	1246	1024.09	1023.09
☑	1247	1024.38	1023.37
☑	1248	1024.96	1023.95

☑	1250	513.70	1025.38
☑	1251	1027.15	1026.14
☑	1252	1027.17	1026.16
☑	1253	1027.49	1026.48
☑	1254	1027.78	1026.77
☑	1255	514.72	1027.42
☑	1256	1029.49	1028.49
☑	1258	1030.72	1029.72
☑	1259	1030.73	1029.73
☑	1261	1031.52	1030.51
☑	1263	1031.94	1030.93
☑	1264	1032.05	1031.04
☑	1265	1032.05	1031.04
☑	1266	1032.17	1031.17
☑	1267	1032.42	1031.42
☑	1268	1032.58	1031.57
☑	1269	1033.16	1032.15
☑	1270	1033.96	1032.95
☑	1271	1034.32	1033.32
☑	1272	518.33	1034.64
☑	1273	1035.66	1034.65
☑	1274	1035.77	1034.76
☑	1275	1035.88	1034.88
☑	1276	1036.51	1035.50
☑	1277	1036.87	1035.86
☑	1278	519.23	1036.44
☑	1279	519.62	1037.22
☑	1280	1038.29	1037.28
☑	1282	1038.82	1037.81
☑	1283	1039.42	1038.41
☑	1284	1039.58	1038.57
☑	1285	1040.02	1039.01
☑	1286	1040.52	1039.51
☑	1287	520.85	1039.68

☑	1288	1041.55	1040.54
☑	1289	1041.61	1040.60
☑	1290	1042.11	1041.10
☑	1291	1043.09	1042.08
☑	1292	1043.17	1042.16
☑	1293	1043.27	1042.26
☑	1294	1043.90	1042.89
☑	1295	1043.94	1042.93
☑	1296	1044.55	1043.55
☑	1297	1045.15	1044.14
☑	1298	1045.17	1044.16
☑	1299	1045.18	1044.17
☑	1300	1045.42	1044.41
☑	1302	1045.70	1044.69
☑	1304	1045.95	1044.94
☑	1305	1046.27	1045.26
☑	1306	1047.10	1046.09
☑	1307	1047.49	1046.48
☑	1308	1047.67	1046.67
☑	1309	1047.73	1046.72
☑	1310	1048.49	1047.49
☑	1311	1048.50	1047.50
☑	1312	1049.71	1048.70
☑	1313	1049.77	1048.76
☑	1314	1050.19	1049.18
☑	1315	1051.36	1050.35
☑	1317	1051.86	1050.85
☑	1318	526.88	1051.75
☑	1319	1052.84	1051.83
☑	1320	1053.52	1052.52
☑	1321	1053.76	1052.76
☑	1323	1054.71	1053.70
☑	1324	1055.04	1054.03
☑	1325	1055.05	1054.04

☑	1326	1055.22	1054.21
☑	1327	1055.54	1054.53
☑	1328	1056.42	1055.41
☑	1329	1056.66	1055.65
☑	1331	1057.20	1056.19
☑	1332	1057.49	1056.48
☑	1333	1057.55	1056.54
☑	1334	1058.33	1057.32
☑	1336	1059.17	1058.16
☑	1337	1059.71	1058.70
☑	1338	1059.85	1058.85
☑	1339	1059.96	1058.95
☑	1340	1060.60	1059.59
☑	1342	530.84	1059.67
☑	1343	1060.88	1059.87
☑	1344	530.97	1059.93
☑	1345	531.06	1060.11
☑	1346	1061.35	1060.34
☑	1347	1061.67	1060.67
☑	1348	1061.83	1060.82
☑	1349	1061.95	1060.95
☑	1350	1062.35	1061.34
☑	1351	1062.39	1061.39
☑	1352	1062.74	1061.74
☑	1353	532.24	1062.47
☑	1354	1064.45	1063.44
☑	1356	1064.71	1063.70
☑	1357	532.86	1063.71
☑	1359	1064.97	1063.96
☑	1360	1065.08	1064.07
☑	1361	1065.17	1064.17
☑	1362	1065.18	1064.18
☑	1363	1065.68	1064.67
☑	1364	1066.60	1065.59

☑	1365	1066.99	1065.99
☑	1366	1067.06	1066.05
☑	1367	1067.14	1066.13
☑	1368	1067.80	1066.79
☑	1369	1067.85	1066.84
☑	1371	1068.55	1067.55
☑	1372	1068.93	1067.92
☑	1373	1070.27	1069.26
☑	1374	1071.12	1070.12
☑	1375	1071.47	1070.46
☑	1376	1072.27	1071.26
☑	1377	1074.59	1073.58
☑	1378	1074.86	1073.85
☑	1379	1075.51	1074.50
☑	1380	1076.16	1075.15
☑	1381	1076.38	1075.37
☑	1382	1076.66	1075.65
☑	1383	1077.12	1076.11
☑	1384	1077.42	1076.41
☑	1385	1077.46	1076.45
☑	1386	539.51	1077.00
☑	1387	1078.08	1077.08
☑	1388	1078.10	1077.09
☑	1389	1078.13	1077.13
☑	1390	1078.34	1077.34
☑	1391	1079.32	1078.31
☑	1392	1079.33	1078.32
☑	1393	1079.34	1078.33
☑	1394	1079.58	1078.58
☑	1395	1080.67	1079.66
☑	1396	1080.88	1079.87
☑	1397	1081.02	1080.02
☑	1398	1081.11	1080.10
☑	1399	1082.12	1081.11

1400	1082.25	1081.24
1401	1082.45	1081.44
1402	1082.83	1081.83
1403	1083.61	1082.60
1404	542.34	1082.66
1405	1084.77	1083.76
1406	1084.96	1083.95
1407	1085.06	1084.05
1408	1085.17	1084.16
1409	1085.52	1084.52
1411	543.35	1084.68
1412	1085.71	1084.71
1413	1086.02	1085.01
1415	1086.26	1085.26
1416	1086.41	1085.40
1417	543.84	1085.66
1418	544.09	1086.17
1419	1087.48	1086.48
1420	1088.21	1087.20
1421	1088.61	1087.60
1422	544.90	1087.79
1423	1089.27	1088.26
1424	1089.77	1088.76
1425	1090.52	1089.51
1426	1090.62	1089.61
1427	1091.67	1090.67
1428	546.56	1091.11
1429	1092.16	1091.16
1430	546.73	1091.45
1431	1093.36	1092.35
1432	1093.98	1092.98
1433	1094.36	1093.35
1434	548.31	1094.61
1435	1095.71	1094.70

☑	1436	1095.81	1094.80
☑	1437	1095.87	1094.86
☑	1438	1096.28	1095.27
☑	1439	1096.28	1095.27
☑	1440	548.83	1095.65
☑	1441	1097.34	1096.33
☑	1442	1097.43	1096.42
☑	1443	1097.61	1096.60
☑	1444	549.46	1096.90
☑	1445	1097.96	1096.95
☑	1446	549.86	1097.70
☑	1447	549.87	1097.72
☑	1448	1098.73	1097.73
☑	1449	1098.98	1097.98
☑	1450	1099.05	1098.04
☑	1451	1099.23	1098.22
☑	1452	1099.37	1098.36
☑	1453	1100.21	1099.20
☑	1454	1100.28	1099.27
☑	1455	1100.83	1099.82
☑	1456	1100.86	1099.85
☑	1457	1101.66	1100.66
☑	1458	1102.02	1101.01
☑	1459	1102.68	1101.67
☑	1460	1103.19	1102.18
☑	1461	1103.68	1102.67
☑	1462	1104.58	1103.57
☑	1463	1105.54	1104.54
☑	1464	1105.56	1104.55
☑	1465	553.31	1104.60
☑	1466	1105.91	1104.91
☑	1467	1107.79	1106.78
☑	1468	1108.22	1107.21
☑	1469	1108.61	1107.61

☑	1470	555.06	1108.10
☑	1471	1109.83	1108.83
☑	1472	1109.91	1108.90
☑	1473	1110.81	1109.81
☑	1474	1110.83	1109.82
☑	1475	1111.27	1110.27
☑	1476	1111.28	1110.28
☑	1477	1111.73	1110.72
☑	1478	1112.28	1111.27
☑	1479	1113.05	1112.04
☑	1480	1113.74	1112.73
☑	1483	1114.84	1113.83
☑	1485	1115.24	1114.23
☑	1486	1115.98	1114.98
☑	1487	1116.04	1115.03
☑	1488	1116.24	1115.23
☑	1489	1116.56	1115.55
☑	1491	1117.49	1116.49
☑	1492	1117.67	1116.66
☑	1493	1117.83	1116.83
☑	1495	1118.19	1117.18
☑	1496	1118.36	1117.35
☑	1497	1118.52	1117.51
☑	1499	1119.06	1118.05
☑	1500	1119.24	1118.24
☑	1501	1119.49	1118.48
☑	1502	1119.85	1118.84
☑	1503	1119.97	1118.96
☑	1504	1120.45	1119.45
☑	1505	1121.86	1120.85
☑	1506	1122.71	1121.70
☑	1507	561.93	1121.84
☑	1508	1123.04	1122.04
☑	1509	1123.92	1122.91

☑	1510	1124.52	1123.51
☑	1511	1124.55	1123.54
☑	1512	562.82	1123.62
☑	1513	1124.90	1123.90
☑	1514	1125.14	1124.14
☑	1515	1125.28	1124.27
☑	1516	1125.50	1124.50
☑	1517	563.35	1124.68
☑	1518	563.37	1124.72
☑	1519	563.39	1124.77
☑	1520	1126.14	1125.14
☑	1521	1126.45	1125.44
☑	1522	1126.52	1125.51
☑	1524	1127.60	1126.59
☑	1525	1127.71	1126.70
☑	1526	564.38	1126.74
☑	1527	1128.33	1127.32
☑	1528	1128.67	1127.66
☑	1529	1129.46	1128.45
☑	1530	1129.51	1128.50
☑	1531	1130.17	1129.16
☑	1532	565.79	1129.57
☑	1533	1131.77	1130.76
☑	1534	1131.85	1130.84
☑	1535	1132.23	1131.22
☑	1536	1134.58	1133.57
☑	1537	1134.82	1133.81
☑	1538	1134.91	1133.90
☑	1539	568.30	1134.59
☑	1540	1135.89	1134.88
☑	1541	1136.90	1135.89
☑	1542	1137.24	1136.24
☑	1544	1137.73	1136.73
☑	1545	1138.73	1137.72

☑	1546	1138.92	1137.92
☑	1547	570.00	1137.98
☑	1548	1139.71	1138.70
☑	1549	1140.11	1139.10
☑	1550	1141.32	1140.31
☑	1551	1141.35	1140.34
☑	1552	1142.30	1141.29
☑	1553	1143.24	1142.23
☑	1554	1143.39	1142.39
☑	1555	1144.21	1143.20
☑	1556	1144.91	1143.90
☑	1557	573.08	1144.15
☑	1558	1145.83	1144.82
☑	1559	1146.25	1145.24
☑	1560	1146.32	1145.31
☑	1561	1146.71	1145.70
☑	1562	1147.21	1146.20
☑	1563	1147.35	1146.34
☑	1564	1147.39	1146.39
☑	1565	1148.10	1147.09
☑	1566	1148.39	1147.38
☑	1567	1149.04	1148.04
☑	1568	1149.44	1148.43
☑	1569	1149.57	1148.56
☑	1570	1149.89	1148.89
☑	1571	1150.46	1149.45
☑	1572	1151.44	1150.44
☑	1573	1151.80	1150.79
☑	1574	1151.86	1150.85
☑	1575	1152.14	1151.13
☑	1576	1152.18	1151.17
☑	1577	576.94	1151.87
☑	1578	576.97	1151.93
☑	1579	1153.51	1152.50

☑	1580	1153.54	1152.53
☑	1581	577.51	1153.00
☑	1582	1154.33	1153.32
☑	1583	1154.64	1153.63
☑	1584	1154.77	1153.76
☑	1585	1156.27	1155.26
☑	1586	578.92	1155.83
☑	1587	1157.69	1156.68
☑	1588	1158.12	1157.11
☑	1589	580.36	1158.70
☑	1590	1160.80	1159.79
☑	1591	1161.32	1160.32
☑	1592	1161.67	1160.66
☑	1593	1162.25	1161.24
☑	1594	581.70	1161.38
☑	1595	581.87	1161.73
☑	1596	1163.02	1162.01
☑	1597	1163.10	1162.10
☑	1598	1163.23	1162.23
☑	1599	1163.58	1162.57
☑	1600	1164.22	1163.21
☑	1601	1164.45	1163.44
☑	1603	583.92	1165.82
☑	1604	1167.30	1166.29
☑	1605	1167.69	1166.69
☑	1606	584.38	1166.75
☑	1607	1169.18	1168.18
☑	1609	1169.46	1168.45
☑	1610	1169.73	1168.72
☑	1611	1169.74	1168.73
☑	1612	585.49	1168.96
☑	1613	1170.15	1169.14
☑	1614	1170.56	1169.55
☑	1615	1170.56	1169.55

☑	1616	1170.98	1169.97
☑	1617	1171.08	1170.07
☑	1618	586.05	1170.08
☑	1619	1171.38	1170.37
☑	1620	1171.47	1170.46
☑	1621	586.30	1170.60
☑	1624	1173.18	1172.17
☑	1627	1175.21	1174.20
☑	1628	588.29	1174.57
☑	1629	588.38	1174.75
☑	1630	1176.49	1175.49
☑	1632	1178.83	1177.82
☑	1633	1179.46	1178.45
☑	1634	1179.55	1178.54
☑	1635	1179.91	1178.90
☑	1636	1181.14	1180.13
☑	1637	1181.55	1180.55
☑	1638	591.36	1180.71
☑	1640	1182.54	1181.53
☑	1641	592.60	1183.19
☑	1642	1184.35	1183.34
☑	1643	1184.71	1183.70
☑	1645	1187.14	1186.13
☑	1647	396.70	1187.08
☑	1648	1188.47	1187.46
☑	1651	1189.02	1188.01
☑	1652	1189.33	1188.33
☑	1653	1189.39	1188.38
☑	1654	1189.52	1188.51
☑	1655	1190.08	1189.07
☑	1656	1190.48	1189.48
☑	1657	1190.84	1189.84
☑	1658	1192.27	1191.26
☑	1659	1194.49	1193.49

☑	1660	597.86	1193.71
☑	1661	1195.00	1193.99
☑	1662	1196.16	1195.15
☑	1663	599.39	1196.76
☑	1664	599.77	1197.52
☑	1665	599.79	1197.57
☑	1666	1199.61	1198.60
☑	1667	1199.77	1198.76
☑	1668	1200.04	1199.03
☑	1669	1200.46	1199.45
☑	1670	1200.51	1199.50
☑	1671	1201.60	1200.59
☑	1672	601.38	1200.76
☑	1675	1204.96	1203.95
☑	1676	603.39	1204.77
☑	1677	1206.12	1205.11
☑	1678	603.58	1205.15
☑	1679	1208.30	1207.29
☑	1680	1209.74	1208.74
☑	1681	1209.97	1208.96
☑	1682	1210.39	1209.39
☑	1683	1210.46	1209.45
☑	1684	605.74	1209.46
☑	1685	1211.03	1210.02
☑	1686	607.33	1212.64
☑	1687	607.34	1212.67
☑	1688	1214.00	1212.99
☑	1689	607.93	1213.84
☑	1693	1216.53	1215.53
☑	1694	1216.57	1215.56
☑	1695	1216.99	1215.99
☑	1698	609.82	1217.62
☑	1699	1219.06	1218.05
☑	1700	1221.36	1220.35

☑	1701	611.28	1220.56
☑	1702	1221.79	1220.79
☑	1704	1225.59	1224.58
☑	1705	409.65	1225.92
☑	1706	1227.11	1226.10
☑	1707	1227.52	1226.51
☑	1708	614.37	1226.72
☑	1709	1228.41	1227.40
☑	1711	614.92	1227.82
☑	1712	1229.89	1228.88
☑	1714	1231.65	1230.65
☑	1715	617.02	1232.03
☑	1716	1234.83	1233.82
☑	1717	1234.92	1233.91
☑	1718	1235.46	1234.46
☑	1719	618.66	1235.30
☑	1720	1236.37	1235.36
☑	1721	1238.12	1237.11
☑	1722	1238.29	1237.28
☑	1723	1238.41	1237.40
☑	1724	619.90	1237.79
☑	1725	1239.15	1238.14
☑	1726	1239.43	1238.42
☑	1728	620.49	1238.97
☑	1729	1241.02	1240.01
☑	1730	1241.53	1240.52
☑	1731	1242.73	1241.72
☑	1732	1243.51	1242.50
☑	1733	1243.83	1242.82
☑	1734	622.59	1243.17
☑	1735	1245.52	1244.51
☑	1737	623.36	1244.71
☑	1738	1246.25	1245.25
☑	1739	1247.96	1246.95

☑	1740	1249.77	1248.76
☑	1741	1250.21	1249.20
☑	1742	626.29	1250.56
☑	1743	626.94	1251.86
☑	1744	627.42	1252.83
☑	1745	627.44	1252.87
☑	1746	418.65	1252.92
☑	1747	1254.79	1253.78
☑	1748	628.44	1254.86
☑	1749	1256.18	1255.17
☑	1750	1256.19	1255.19
☑	1751	628.84	1255.66
☑	1752	1260.89	1259.88
☑	1754	631.91	1261.80
☑	1756	633.58	1265.15
☑	1757	1269.40	1268.40
☑	1758	635.75	1269.48
☑	1759	635.86	1269.71
☑	1760	1272.14	1271.14
☑	1761	636.97	1271.93
☑	1762	1273.48	1272.47
☑	1763	637.89	1273.77
☑	1764	637.92	1273.84
☑	1765	1275.36	1274.35
☑	1766	1275.65	1274.64
☑	1767	1278.43	1277.42
☑	1768	1278.61	1277.60
☑	1769	1278.92	1277.91
☑	1770	640.36	1278.71
☑	1772	640.73	1279.44
☑	1773	1283.80	1282.79
☑	1774	643.07	1284.12
☑	1775	429.50	1285.47
☑	1777	644.88	1287.75

☑	1778	646.17	1290.33
☑	1779	1292.02	1291.01
☑	1780	647.32	1292.62
☑	1783	1294.48	1293.47
☑	1784	1294.48	1293.47
☑	1785	647.95	1293.89
☑	1788	433.77	1298.29
☑	1789	650.34	1298.67
☑	1791	653.90	1305.79
☑	1792	1307.73	1306.72
☑	1793	1309.83	1308.82
☑	1794	437.29	1308.86
☑	1795	656.13	1310.24
☑	1796	1312.83	1311.83
☑	1797	658.38	1314.74
☑	1799	1320.46	1319.45
☑	1800	1327.18	1326.18
☑	1801	665.46	1328.91
☑	1802	444.34	1330.00
☑	1803	667.84	1333.66
☑	1805	668.12	1334.23
☑	1808	671.54	1341.07
☑	1809	671.60	1341.18
☑	1811	673.84	1345.67
☑	1812	673.88	1345.76
☑	1813	674.45	1346.89
☑	1814	676.60	1351.19
☑	1815	1353.21	1352.21
☑	1816	1354.62	1353.62
☑	1817	678.87	1355.73
☑	1818	678.89	1355.77
☑	1819	1357.05	1356.04
☑	1820	679.56	1357.10
☑	1821	1358.39	1357.39

☑	1822	1361.92	1360.91
☑	1823	681.69	1361.36
☑	1824	682.28	1362.55
☑	1825	682.45	1362.88
☑	1826	1364.03	1363.02
☑	1827	686.13	1370.25
☑	1828	688.95	1375.89
☑	1829	689.43	1376.84
☑	1830	690.42	1378.82
☑	1831	690.42	1378.83
☑	1832	690.90	1379.79
☑	1834	691.66	1381.31
☑	1839	693.73	1385.45
☑	1840	693.93	1385.85
☑	1842	705.16	1408.31
☑	1843	706.25	1410.50
☑	1844	1412.95	1411.95
☑	1846	473.23	1416.66
☑	1847	709.35	1416.68
☑	1848	709.41	1416.80
☑	1849	710.43	1418.85
☑	1850	712.37	1422.72
☑	1851	714.45	1426.88
☑	1852	714.89	1427.77
☑	1853	717.81	1433.61
☑	1854	718.02	1434.03
☑	1855	718.76	1435.51
☑	1857	481.65	1441.94
☑	1858	722.87	1443.72
☑	1859	722.97	1443.92
☑	1860	724.91	1447.82
☑	1861	725.13	1448.25
☑	1862	725.43	1448.85
☑	1863	725.90	1449.80

☑	1864	726.75	1451.49
☑	1866	485.04	1452.10
☑	1867	485.05	1452.12
☑	1869	727.88	1453.75
☑	1870	728.73	1455.44
☑	1872	730.30	1458.59
☑	1875	733.41	1464.80
☑	1876	733.86	1465.70
☑	1877	733.89	1465.77
☑	1878	734.36	1466.70
☑	1880	489.95	1466.83
☑	1882	737.66	1473.31
☑	1883	738.04	1474.06
☑	1885	738.88	1475.75
☑	1887	739.52	1477.02
☑	1888	739.68	1477.36
☑	1889	494.06	1479.16
☑	1891	741.94	1481.87
☑	1892	741.99	1481.97
☑	1893	743.45	1484.88
☑	1894	496.42	1486.24
☑	1896	497.99	1490.95
☑	1898	747.86	1493.71
☑	1899	498.99	1493.94
☑	1900	750.21	1498.41
☑	1903	751.52	1501.03
☑	1904	501.81	1502.42
☑	1905	502.34	1504.01
☑	1906	753.09	1504.16
☑	1907	754.08	1506.14
☑	1908	754.40	1506.78
☑	1909	754.89	1507.77
☑	1911	755.45	1508.88
☑	1912	756.05	1510.08

☑	1913	505.05	1512.11
☑	1914	758.06	1514.10
☑	1915	758.32	1514.62
☑	1916	758.46	1514.91
☑	1917	759.14	1516.27
☑	1918	506.63	1516.86
☑	1920	761.44	1520.87
☑	1921	762.10	1522.19
☑	1922	762.31	1522.61
☑	1923	508.76	1523.27
☑	1924	763.37	1524.72
☑	1925	763.92	1525.83
☑	1927	764.40	1526.79
☑	1928	765.25	1528.49
☑	1929	767.07	1532.12
☑	1930	768.85	1535.69
☑	1931	769.48	1536.94
☑	1932	770.56	1539.10
☑	1933	514.86	1541.55
☑	1935	515.68	1544.01
☑	1936	516.65	1546.93
☑	1937	774.69	1547.36
☑	1938	517.59	1549.74
☑	1939	776.00	1549.98
☑	1940	517.70	1550.08
☑	1941	518.35	1552.03
☑	1942	777.75	1553.49
☑	1943	778.95	1555.88
☑	1944	780.68	1559.34
☑	1946	522.37	1564.08
☑	1947	522.67	1564.98
☑	1948	522.81	1565.42
☑	1949	523.03	1566.06
☑	1950	785.07	1568.12

☑	1951	786.73	1571.45
☑	1952	787.06	1572.10
☑	1953	525.76	1574.25
☑	1954	788.81	1575.60
☑	1955	789.92	1577.83
☑	1956	791.19	1580.36
☑	1957	793.01	1584.00
☑	1959	793.90	1585.79
☑	1960	530.28	1587.82
☑	1961	800.03	1598.05
☑	1962	800.92	1599.83
☑	1963	803.13	1604.25
☑	1965	536.74	1607.19
☑	1966	537.78	1610.33
☑	1967	537.87	1610.57
☑	1968	807.28	1612.55
☑	1969	538.72	1613.13
☑	1970	808.10	1614.19
☑	1971	809.99	1617.96
☑	1972	540.35	1618.03
☑	1973	810.12	1618.22
☑	1974	542.01	1623.01
☑	1975	812.55	1623.09
☑	1976	812.71	1623.40
☑	1977	542.32	1623.94
☑	1978	813.00	1623.99
☑	1979	813.02	1624.04
☑	1980	814.08	1626.15
☑	1981	543.34	1626.99
☑	1982	543.57	1627.69
☑	1983	815.03	1628.05
☑	1984	815.03	1628.06
☑	1985	815.47	1628.92
☑	1986	818.38	1634.74

☑	1987	821.28	1640.55
☑	1988	549.47	1645.37
☑	1990	550.28	1647.81
☑	1992	553.58	1657.71
☑	1993	553.69	1658.05
☑	1994	830.03	1658.05
☑	1995	831.99	1661.96
☑	1996	832.84	1663.66
☑	1997	833.44	1664.87
☑	1998	834.30	1666.59
☑	1999	835.36	1668.70
☑	2000	835.53	1669.05
☑	2001	835.56	1669.11
☑	2002	557.55	1669.64
☑	2003	835.87	1669.72
☑	2004	559.01	1674.00
☑	2005	838.05	1674.08
☑	2006	559.03	1674.08
☑	2007	838.90	1675.78
☑	2008	559.68	1676.02
☑	2009	559.74	1676.19
☑	2010	560.09	1677.25
☑	2011	840.11	1678.20
☑	2012	840.14	1678.28
☑	2013	841.81	1681.60
☑	2014	842.28	1682.55
☑	2015	562.07	1683.19
☑	2016	843.11	1684.21
☑	2017	563.80	1688.39
☑	2019	564.93	1691.77
☑	2022	847.00	1691.98
☑	2023	848.15	1694.29
☑	2025	849.98	1697.94
☑	2026	567.36	1699.05

☑	2027	851.44	1700.87
☑	2028	851.47	1700.92
☑	2029	852.70	1703.38
☑	2030	569.84	1706.50
☑	2031	571.25	1710.72
☑	2033	572.22	1713.63
☑	2034	858.17	1714.32
☑	2036	573.59	1717.74
☑	2037	860.29	1718.57
☑	2039	574.51	1720.52
☑	2042	575.60	1723.78
☑	2043	579.06	1734.17
☑	2044	869.85	1737.68
☑	2045	871.52	1741.02
☑	2046	872.14	1742.26
☑	2047	873.61	1745.21
☑	2048	876.45	1750.88
☑	2049	877.20	1752.38
☑	2050	877.36	1752.70
☑	2051	586.10	1755.27
☑	2052	588.71	1763.10
☑	2053	883.11	1764.20
☑	2054	883.94	1765.87
☑	2055	589.66	1765.96
☑	2056	591.39	1771.14
☑	2058	592.03	1773.08
☑	2059	592.73	1775.17
☑	2060	595.91	1784.71
☑	2061	596.28	1785.82
☑	2062	894.47	1786.92
☑	2063	597.09	1788.26
☑	2064	895.52	1789.03
☑	2065	597.83	1790.47
☑	2066	897.52	1793.03

☑	2067	598.79	1793.34
☑	2069	600.35	1798.02
☑	2070	901.14	1800.27
☑	2071	601.78	1802.31
☑	2072	904.11	1806.21
☑	2073	905.48	1808.94
☑	2074	905.91	1809.81
☑	2076	906.68	1811.35
☑	2078	909.92	1817.83
☑	2079	607.35	1819.04
☑	2080	911.24	1820.47
☑	2082	912.03	1822.04
☑	2083	609.62	1825.83
☑	2084	914.05	1826.09
☑	2085	611.61	1831.81
☑	2086	611.63	1831.87
☑	2087	612.02	1833.04
☑	2088	612.40	1834.18
☑	2089	919.00	1835.99
☑	2092	614.69	1841.05
☑	2093	921.82	1841.63
☑	2094	922.47	1842.92
☑	2095	616.51	1846.51
☑	2096	924.46	1846.91
☑	2098	924.70	1847.38
☑	2100	927.08	1852.15
☑	2101	619.37	1855.09
☑	2102	928.85	1855.68
☑	2103	619.61	1855.81
☑	2104	929.98	1857.94
☑	2105	931.04	1860.07
☑	2106	931.05	1860.09
☑	2107	935.57	1869.13
☑	2108	936.80	1871.58

☑	2109	939.26	1876.51
☑	2110	939.32	1876.63
☑	2111	939.94	1877.86
☑	2112	627.09	1878.26
☑	2113	941.10	1880.18
☑	2114	941.98	1881.94
☑	2115	942.15	1882.29
☑	2116	630.08	1887.20
☑	2117	947.01	1892.01
☑	2118	632.64	1894.91
☑	2119	949.55	1897.09
☑	2120	953.11	1904.21
☑	2121	636.89	1907.64
☑	2122	637.36	1909.07
☑	2123	955.91	1909.81
☑	2124	638.14	1911.40
☑	2127	960.00	1917.99
☑	2128	960.14	1918.27
☑	2129	960.59	1919.17
☑	2130	962.48	1922.95
☑	2131	962.84	1923.67
☑	2132	642.38	1924.12
☑	2133	963.78	1925.56
☑	2134	643.36	1927.05
☑	2135	965.43	1928.84
☑	2136	965.94	1929.87
☑	2137	966.63	1931.25
☑	2138	645.10	1932.27
☑	2139	968.14	1934.26
☑	2140	971.01	1940.01
☑	2141	971.12	1940.23
☑	2142	648.78	1943.32
☑	2143	974.21	1946.41
☑	2144	974.59	1947.16

2145	974.92	1947.83
2146	652.41	1954.20
2147	979.04	1956.06
2148	979.16	1956.31
2149	980.52	1959.03
2150	982.49	1962.97
2151	982.65	1963.28
2152	982.92	1963.83
2153	985.08	1968.14
2154	985.35	1968.68
2155	988.75	1975.49
2156	661.78	1982.32
2157	993.66	1985.31
2158	664.86	1991.56
2159	997.13	1992.26
2160	997.80	1993.59
2162	1002.82	2003.62
2163	1003.99	2005.97
2164	1005.10	2008.18
2165	1007.11	2012.20
2166	1007.57	2013.13
2167	1007.92	2013.84
2168	1008.75	2015.49
2169	1010.88	2019.74
2170	674.49	2020.46
2171	1012.30	2022.58
2172	1013.22	2024.42
2173	676.07	2025.18
2174	677.39	2029.15
2175	1015.71	2029.41
2176	1016.14	2030.27
2177	1017.77	2033.53
2178	1018.42	2034.82
2179	1020.02	2038.03

☑	2180	1024.06	2046.11
☑	2181	683.31	2046.90
☑	2182	1025.50	2048.98
☑	2183	1025.64	2049.27
☑	2184	1025.98	2049.96
☑	2185	1026.07	2050.12
☑	2186	685.17	2052.49
☑	2187	685.38	2053.10
☑	2188	1027.60	2053.18
☑	2189	1028.82	2055.63
☑	2190	1029.46	2056.91
☑	2191	1029.83	2057.64
☑	2192	687.43	2059.28
☑	2193	688.11	2061.30
☑	2194	1032.14	2062.26
☑	2195	1033.76	2065.50
☑	2196	1033.91	2065.82
☑	2197	689.64	2065.91
☑	2198	691.83	2072.46
☑	2199	1037.53	2073.04
☑	2200	1040.25	2078.49
☑	2201	1040.89	2079.76
☑	2202	1041.97	2081.92
☑	2203	696.76	2087.27
☑	2204	1045.64	2089.27
☑	2205	1046.50	2090.98
☑	2206	1048.05	2094.09
☑	2207	699.60	2095.78
☑	2208	700.41	2098.20
☑	2209	700.42	2098.23
☑	2210	1051.76	2101.50
☑	2211	701.59	2101.76
☑	2212	1052.02	2102.02
☑	2213	702.59	2104.75

☑	2214	702.91	2105.71
☑	2215	703.37	2107.09
☑	2217	705.16	2112.45
☑	2218	1057.57	2113.12
☑	2219	1058.36	2114.70
☑	2220	1059.94	2117.86
☑	2221	1061.05	2120.08
☑	2222	708.40	2122.18
☑	2223	1064.42	2126.83
☑	2224	709.96	2126.84
☑	2225	710.17	2127.50
☑	2226	711.42	2131.23
☑	2227	712.10	2133.28
☑	2228	712.43	2134.28
☑	2229	1068.35	2134.68
☑	2231	714.52	2140.53
☑	2232	714.66	2140.96
☑	2233	1072.69	2143.37
☑	2234	716.80	2147.37
☑	2235	1075.41	2148.81
☑	2236	1076.53	2151.04
☑	2237	1078.96	2155.90
☑	2238	720.34	2158.01
☑	2239	722.41	2164.20
☑	2240	1083.14	2164.26
☑	2241	1084.15	2166.28
☑	2242	724.76	2171.25
☑	2243	725.04	2172.11
☑	2245	1088.62	2175.23
☑	2246	1090.13	2178.24
☑	2247	1090.74	2179.47
☑	2248	1090.88	2179.75
☑	2249	727.97	2180.89
☑	2250	1091.49	2180.97

☑	2251	1101.30	2200.59
☑	2252	1103.25	2204.48
☑	2253	1105.37	2208.73
☑	2254	1106.13	2210.24
☑	2255	1106.32	2210.63
☑	2256	1107.51	2213.00
☑	2257	1114.70	2227.38
☑	2258	1115.32	2228.63
☑	2259	1118.16	2234.31
☑	2260	1119.60	2237.18
☑	2261	747.80	2240.39
☑	2263	748.78	2243.31
☑	2264	749.73	2246.18
☑	2265	1124.62	2247.22
☑	2266	751.14	2250.40
☑	2267	1126.58	2251.15
☑	2268	751.48	2251.42
☑	2269	1126.78	2251.55
☑	2271	1127.47	2252.92
☑	2272	751.98	2252.93
☑	2273	752.21	2253.60
☑	2274	752.51	2254.50
☑	2275	752.59	2254.74
☑	2277	754.71	2261.09
☑	2278	755.45	2263.32
☑	2279	1132.84	2263.67
☑	2280	1134.42	2266.84
☑	2281	756.83	2267.47
☑	2282	758.57	2272.68
☑	2283	758.79	2273.36
☑	2284	1138.69	2275.36
☑	2285	761.54	2281.61
☑	2286	1141.87	2281.72
☑	2287	761.80	2282.38

☑	2289	765.64	2293.91
☑	2290	765.86	2294.56
☑	2291	767.07	2298.19
☑	2292	768.25	2301.72
☑	2293	770.54	2308.60
☑	2294	770.79	2309.34
☑	2295	770.98	2309.92
☑	2296	771.46	2311.35
☑	2297	774.69	2321.06
☑	2298	776.13	2325.38
☑	2299	777.17	2328.50
☑	2300	1168.35	2334.69
☑	2301	779.90	2336.69
☑	2302	1172.17	2342.33
☑	2303	1172.57	2343.12
☑	2304	783.25	2346.73
☑	2305	783.46	2347.35
☑	2306	784.50	2350.47
☑	2307	784.55	2350.63
☑	2308	784.82	2351.44
☑	2309	785.05	2352.14
☑	2310	786.07	2355.19
☑	2311	786.54	2356.60
☑	2312	788.01	2361.00
☑	2313	788.15	2361.44
☑	2314	1182.20	2362.39
☑	2315	1182.37	2362.73
☑	2316	789.22	2364.63
☑	2317	1183.82	2365.62
☑	2318	790.12	2367.33
☑	2319	793.39	2377.16
☑	2320	794.47	2380.40
☑	2321	795.09	2382.25
☑	2322	1192.56	2383.11

2323	795.38	2383.12
2324	795.40	2383.18
2325	798.55	2392.62
2326	803.85	2408.52
2327	803.89	2408.66
2328	804.91	2411.70
2329	805.14	2412.41
2331	806.05	2415.12
2332	807.54	2419.59
2333	807.84	2420.50
2334	1212.03	2422.05
2335	810.48	2428.41
2336	811.09	2430.26
2337	814.12	2439.35
2338	816.18	2445.52
2339	816.60	2446.79
2340	816.83	2447.48
2341	821.62	2461.84
2342	823.21	2466.59
2344	825.87	2474.57
2345	826.59	2476.74
2346	1241.82	2481.63
2347	828.92	2483.75
2348	829.83	2486.47
2349	832.15	2493.42
2350	833.23	2496.67
2351	835.26	2502.77
2352	836.44	2506.30
2353	837.41	2509.21
2354	839.65	2515.92
2355	840.65	2518.92
2356	841.54	2521.61
2357	844.08	2529.22
2360	849.06	2544.16

☑	2361	850.15	2547.43
☑	2364	853.36	2557.07
☑	2365	855.42	2563.24
☑	2366	855.90	2564.67
☑	2367	860.09	2577.25
☑	2368	860.74	2579.20
☑	2370	866.57	2596.69
☑	2371	868.63	2602.87
☑	2372	868.77	2603.28
☑	2373	872.68	2615.01
☑	2374	872.73	2615.16
☑	2375	873.13	2616.36
☑	2376	875.87	2624.60
☑	2377	876.94	2627.81
☑	2378	877.61	2629.81
☑	2379	878.71	2633.12
☑	2380	882.64	2644.90
☑	2381	884.41	2650.22
☑	2382	884.52	2650.53
☑	2383	884.91	2651.72
☑	2384	889.83	2666.47
☑	2385	890.28	2667.82
☑	2386	890.64	2668.90
☑	2387	892.45	2674.33
☑	2388	892.75	2675.22
☑	2389	893.02	2676.03
☑	2390	895.05	2682.14
☑	2391	895.51	2683.50
☑	2392	895.98	2684.92
☑	2393	897.49	2689.44
☑	2394	897.53	2689.57
☑	2395	899.55	2695.64
☑	2396	902.84	2705.48
☑	2397	904.58	2710.73

☑	2398	906.42	2716.25
☑	2399	908.20	2721.58
☑	2400	908.33	2721.97
☑	2401	914.26	2739.76
☑	2402	914.27	2739.78
☑	2403	918.63	2752.87
☑	2404	920.15	2757.43
☑	2405	921.46	2761.36
☑	2406	924.73	2771.17
☑	2407	924.81	2771.40
☑	2408	924.96	2771.87
☑	2409	925.04	2772.10
☑	2410	925.09	2772.24
☑	2411	926.61	2776.81
☑	2412	927.29	2778.85
☑	2413	932.51	2794.52
☑	2414	935.39	2803.16
☑	2415	935.46	2803.35
☑	2416	937.00	2807.98
☑	2417	937.22	2808.64
☑	2418	940.78	2819.33
☑	2419	941.67	2821.99
☑	2420	942.75	2825.22
☑	2421	949.66	2845.96
☑	2422	950.34	2847.98
☑	2423	950.52	2848.54
☑	2424	951.17	2850.49
☑	2425	952.74	2855.19
☑	2426	957.73	2870.16
☑	2427	957.95	2870.82
☑	2428	961.38	2881.11
☑	2429	962.96	2885.87
☑	2430	962.98	2885.91
☑	2431	965.22	2892.65

☑	2432	967.78	2900.33
☑	2433	968.52	2902.54
☑	2434	968.86	2903.54
☑	2435	969.62	2905.83
☑	2436	969.80	2906.39
☑	2437	969.87	2906.59
☑	2438	970.85	2909.52
☑	2439	973.59	2917.75
☑	2440	975.24	2922.70
☑	2441	976.47	2926.38
☑	2442	977.41	2929.22
☑	2443	977.76	2930.27
☑	2444	980.80	2939.39
☑	2445	984.56	2950.66
☑	2446	984.79	2951.36
☑	2447	985.31	2952.90
☑	2448	987.73	2960.16
☑	2449	991.09	2970.24
☑	2450	994.85	2981.52
☑	2451	995.98	2984.92
☑	2452	996.83	2987.46
☑	2453	997.91	2990.70
☑	2455	1002.87	3005.59
☑	2456	1004.86	3011.56
☑	2457	1006.93	3017.76
☑	2458	1018.13	3051.38
☑	2459	1021.56	3061.67
☑	2460	1024.57	3070.68
☑	2461	1029.15	3084.44
☑	2462	1032.08	3093.22
☑	2463	1038.14	3111.40
☑	2464	1040.55	3118.64
☑	2465	1043.88	3128.62
☑	2466	1048.02	3141.04

☑	2467	*1048.26*	*3141.76*
☑	2468	*1050.17*	*3147.48*
☑	2469	*1050.29*	*3147.83*
☑	2470	*1052.09*	*3153.23*
☑	2471	*1053.93*	*3158.78*
☑	2472	*1055.33*	*3162.98*
☑	2473	*1058.27*	*3171.78*
☑	2474	*1058.48*	*3172.42*
☑	2475	*1060.55*	*3178.63*
☑	2476	*1066.07*	*3195.20*
☑	2477	*1066.52*	*3196.52*
☑	2478	*1068.94*	*3203.81*
☑	2479	*1072.20*	*3213.57*
☑	2480	*1072.71*	*3215.10*
☑	2481	*1073.49*	*3217.45*
☑	2482	*1078.05*	*3231.14*
☑	2483	*1082.64*	*3244.91*
☑	2484	*1090.15*	*3267.42*
☑	2485	*1092.21*	*3273.60*
☑	2486	*1093.06*	*3276.15*
☑	2487	*1104.58*	*3310.72*
☑	2488	*1105.86*	*3314.56*
☑	2489	*1123.63*	*3367.88*
☑	2490	*1123.93*	*3368.76*
☑	2491	*1127.70*	*3380.09*
☑	2492	*1137.01*	*3407.99*
☑	2493	*1139.59*	*3415.75*
☑	2494	*1140.64*	*3418.89*
☑	2495	*1151.46*	*3451.36*
☑	2496	*1159.44*	*3475.31*
☑	2497	*1201.20*	*3600.57*
☑	2498	*1207.99*	*3620.95*
☑	2499	*1208.89*	*3623.66*

Search Parameters

```
Type of search         : MS/MS Ion Search
Enzyme                 : Trypsin
Variable modifications : Carbamidomethyl (C),Oxidation (M)
Mass values            : Monoisotopic
Protein Mass           : Unrestricted
Peptide Mass Tolerance : ± 0.6 Da
Fragment Mass Tolerance: ± 0.3 Da
Max Missed Cleavages   : 2
Instrument type        : ESI-TRAP
Number of queries      : 2499
```

Mascot: http://www.matrixscience.com/
