# Supplementary material for: Macrophage-infectivity potentiator of Trypanosoma cruzi (TcMIP) is a new pro-type 1 immuno-stimulating protein for neonatal human cells and vaccines in mice
Source: Front Immunol. 2023 Mar 23;14:1138526. doi: 10.3389/fimmu.2023.1138526 (PMC10077492; doi:10.3389/fimmu.2023.1138526)
Supplement: Supplementary file 5 [file DataSheet_3.pdf]

Mascot Search Results

User	: GM	
Email	: gabriel.mazzucchelli@ulg.ac.be	
Search title	: Submitted from 081205-adj-OGE34-8558-other Euc-NCBI by Mascot Daemon on MASPEC39	
MS data file	: F:\DATA\Archives\ESQUIRE\2008-Esquire\Adjuvac\081205-OGE34-140min\081205-adj-OGE34_8558.mgf	
Database	: NCBInr 20081117 (6550153 sequences; 2246496946 residues)	
Taxonomy	: Other Eukaryota (159618 sequences)	
Timestamp	: 8 Dec 2008 at 08:18:13 GMT	
Significant hits:	gi|71399455	surface protein TolT [Trypanosoma cruzi strain CL Brener]
	gi|295363	heat shock protein 60
	gi|349838	heat shock protein
	gi|71414968	microtubule-associated protein [Trypanosoma cruzi strain CL Brener]
	gi|71662736	hypothetical protein [Trypanosoma cruzi strain CL Brener]
	gi|71410853	10 kDa heat shock protein [Trypanosoma cruzi strain CL Brener]
	gi|71413559	hypothetical protein [Trypanosoma cruzi strain CL Brener]
	gi|111115853	beta tubulin [Trypanosoma evansi]
	gi|119859	RecName: Full=Flagellar calcium-binding protein; Short=FCABP; AltName: Full=1F8 protein; AltName: F
	gi|91983201	beta tubulin [Trypanosoma grayi]
	gi|10673	unnamed protein product [Trypanosoma cruzi]
	gi|71405064	60S acidic ribosomal protein P2 [Trypanosoma cruzi strain CL Brener]
	gi|162030	calmodulin A
	gi|120679	RecName: Full=Glyceraldehyde-3-phosphate dehydrogenase, glycosomal; Short=GAPDH
	gi|71649650	surface protein TolT [Trypanosoma cruzi strain CL Brener]
	gi|71659663	hypothetical protein [Trypanosoma cruzi strain CL Brener]
	gi|18277736	RecName: Full=Chaperonin HSP60, mitochondrial; Short=Protein Cpn60; AltName: Full=groEL protein; Al
	gi|71667953	hypothetical protein [Trypanosoma cruzi strain CL Brener]
	gi|71398774	hypothetical protein [Trypanosoma cruzi strain CL Brener]
	gi|2961256	putative malate dehydrogenase [Trypanosoma cruzi]
	gi|295361	BiP/GRP78
	gi|71402480	hypothetical protein [Trypanosoma cruzi strain CL Brener]
	gi|438910	elongation factor 1-alpha
	gi|91983200	alpha tubulin [Trypanosoma grayi]
	gi|133055	60S acidic ribosomal protein P1
	gi|71418715	60S acidic ribosomal protein [Trypanosoma cruzi strain CL Brener]
	gi|121960	Histone H2A.1
	gi|71416147	histone H2A [Trypanosoma cruzi strain CL Brener]
	gi|85822967	elongation factor 1-alpha [Schizochytrium aggregatum]
	gi|156987940	translation elongation factor 1-alpha [Phytophthora primulae]
	gi|114865174	translation elongation factor 1-alpha [Pythium sp. quercum]
	gi|85822985	elongation factor 1-alpha [Thraustochytriidae sp. P19]
	gi|89329735	translation elongation factor 1-alpha [Capsaspora owczarzaki]
	gi|146083153	elongation factor 1-alpha [Leishmania infantum JPCM5]

gi|56156664	elongation factor 1A [Cribraria cancellata]
gi|59859756	elongation factor 1 alpha [Cyclotella cryptica]
gi|704459	elongation factor 1 alpha [Trypanosoma cruzi]
gi|71663174	succinyl-CoA ligase [GDP-forming] beta-chain [Trypanosoma cruzi]
gi|71656402	hypothetical protein [Trypanosoma cruzi strain CL Brener]
gi|1781355	histone H2A [Trypanosoma cruzi]
gi|13384081	histone H2A [Leishmania infantum]
gi|89329739	heat shock protein 70 [Capsaspora owczarzaki]
gi|183232119	heat shock protein70, hsp70A2 [Entamoeba histolytica HM-1:IMSS]
gi|10626	Heat shock protein 70 [Trypanosoma cruzi]
gi|123603	RecName: Full=Heat shock 70 kDa protein
gi|58042864	PPAT5 [Hyaloperonospora parasitica]
gi|123592	RecName: Full=Heat shock 70 kDa protein
gi|71650885	ATP-dependent DEAD/H RNA helicase [Trypanosoma cruzi strain CL Brener]
gi|5139798	elongation factor 1 alpha [Pyrsonympha grandis]
gi|118766700	elongation factor-1 alpha [Oxymonadida environmental sample]
gi|71401749	hypothetical protein [Trypanosoma cruzi strain CL Brener]
gi|438596	elongation factor 1-alpha
gi|71401492	fatty acid desaturase [Trypanosoma cruzi strain CL Brener]
gi|167375825	hypothetical protein [Entamoeba dispar SAW760]
gi|71651158	hypothetical protein [Trypanosoma cruzi strain CL Brener]
gi|9954108	RNA binding protein RGGm [Trypanosoma cruzi]
gi|15488542	centrin [Leishmania donovani]
gi|71412664	hypothetical protein [Trypanosoma cruzi strain CL Brener]
gi|123436584	hypothetical protein [Trichomonas vaginalis G3]
gi|71407337	hypothetical protein [Trypanosoma cruzi strain CL Brener]
gi|5566209	enolase 1 [Trypanosoma cruzi]
gi|160330959	hypothetical protein HAN_1g14 [Hemiselmis andersenii]
gi|91983209	beta tubulin [Leishmania mexicana]
gi|71412308	hypothetical protein [Trypanosoma cruzi strain CL Brener]
gi|58414949	polyubiquitin [Stauracon pallidus]
gi|154411942	hypothetical protein [Trichomonas vaginalis G3]
gi|11467023	RNA polymerase alpha chain [Euglena gracilis]
gi|123437981	hypothetical protein [Trichomonas vaginalis G3]
gi|123456564	hypothetical protein [Trichomonas vaginalis G3]
gi|146089176	vacuolar protein sorting-like protein [Leishmania infantum JPCM5]
gi|123416964	hypothetical protein [Trichomonas vaginalis G3]
gi|71659778	poly(A)-binding protein [Trypanosoma cruzi strain CL Brener]
gi|71420441	hypothetical protein [Trypanosoma cruzi strain CL Brener]
gi|225587	tubulin alpha
gi|84105377	alpha tubulin 2 [Rhynchopus sp. ATCC 50230]
gi|116222245	alpha tubulin [Thaumatomonas sp. TMT002]
gi|914057	KMP-11=kinetoplastid membrane protein-11 [Leishmania donovani, LD3, promastigotes, Peptide Kinetopl
gi|84105385	cytosolic heat shock protein 70 [Malawimonas jakobiformis]
gi|53829566	HSP70 [Nuclearia simplex]

gi|8918240	hsp70 [Blastocystis hominis]
gi|71409962	calpain-like cysteine peptidase [Trypanosoma cruzi strain CL Brener]
gi|71411263	hypothetical protein Tc00.1047053507389.20 [Trypanosoma cruzi strain CL Brener]
gi|71407149	hypothetical protein [Trypanosoma cruzi strain CL Brener]
gi|71404920	hypothetical protein [Trypanosoma cruzi strain CL Brener]
gi|27734387	polyubiquitin [Euglypha rotunda]

Probability Based Mowse Score

Ions score is -10*Log(P), where P is the probability that the observed match is a random event.
Individual ions scores > 42 indicate identity or extensive homology (p<0.05).
Protein scores are derived from ions scores as a non-probabilistic basis for ranking protein hits.

Score Distribution

Peptide Summary Report

Format As | Peptide Summary | Help

Significance threshold p< 0.05 | Max. number of hits AUTO

Standard scoring ○ MudPIT scoring ● | Ions score cut-off 0 | Show sub-sets ☐

Show pop-ups ● Suppress pop-ups ○ | Sort unassigned Decreasing Score | Require bold red ☐

Select All | Select None | Search Selected | ☐ **Error tolerant** | Archive Report

1. gi|71399455 **Mass:** 24752 **Score:** 306 **Queries matched:** 6
surface protein TolT [Trypanosoma cruzi strain CL Brener]

☐ Check to include this hit in error tolerant search or archive report

	Query	Observed	Mr(expt)	Mr(calc)	Delta	Miss	Score	Expect	Rank	Peptide
☑	1343	1188.70	1187.69	1187.64	0.05	0	(15)	18	1	R.AAEQTVLSLEK.A
☑	1344	594.90	1187.79	1187.64	0.15	0	68	9e-05	1	R.AAEQTVLSLEK.A
☑	1345	594.94	1187.87	1187.64	0.23	0	(60)	0.0005	1	R.AAEQTVLSLEK.A
☑	1615	751.04	1500.07	1499.83	0.23	0	110	5.3e-09	1	R.TLAQDVAATASALLR.Q
☑	1925	720.10	2157.28	2157.10	0.18	0	(83)	2.3e-06	1	K.AAEEAAQAAGIMTLDAVGEVLK.H
☑	1926	1079.67	2157.33	2157.10	0.23	0	131	3.7e-11	1	K.AAEEAAQAAGIMTLDAVGEVLK.H

Proteins matching the same set of peptides:

gi|71402301 **Mass:** 32319 **Score:** 306 **Queries matched:** 6

surface protein TolT [Trypanosoma cruzi strain CL Brener]

---

2. gi|295363 **Mass:** 59349 **Score:** 200 **Queries matched:** 5

heat shock protein 60

☐ Check to include this hit in error tolerant search or archive report

	Query	Observed	Mr(expt)	Mr(calc)	Delta	Miss	Score	Expect	Rank	Peptide
☑	1503	659.93	1317.85	1317.69	0.15	0	87	1.1e-06	1	R.NVIIEQSYGAPK.I
☑	1512	670.41	1338.81	1338.61	0.20	0	75	1.7e-05	1	R.GLIDGETSDYNR.E
☑	380	696.47	1390.93	1390.73	0.19	0	50	0.013	1	K.AELEDAFVLVSAK.K
☑	1667	804.02	1606.03	1605.92	0.11	0	26	1.1	1	R.AAVQEGIVPGGGVALLR.A
☑	1709	846.99	1691.97	1691.81	0.16	0	89	7.1e-07	1	K.VLENNDVTVGYDAQR.D

Proteins matching the same set of peptides:

gi|3023478 **Mass:** 59374 **Score:** 200 **Queries matched:** 5

RecName: Full=Chaperonin HSP60, mitochondrial; Short=Protein Cpn60; AltName: Full=groEL protein; Al

gi|71665068 **Mass:** 59129 **Score:** 200 **Queries matched:** 5

chaperonin HSP60, mitochondrial precursor [Trypanosoma cruzi strain CL Brener]

gi|71665064 **Mass:** 59351 **Score:** 200 **Queries matched:** 5

chaperonin HSP60, mitochondrial precursor [Trypanosoma cruzi strain CL Brener]

---

3. gi|349838 **Mass:** 71488 **Score:** 198 **Queries matched:** 5

heat shock protein

Check to include this hit in error tolerant search or archive report

Query	Observed	Mr(expt)	Mr(calc)	Delta	Miss	Score	Expect	Rank	Peptide
694	435.27	868.53	868.51	0.01	0	25	1.3	2	K.GTLVPVQR.V
1477	650.40	1298.79	1298.62	0.17	0	62	0.00032	1	K.FEELNMELFK.G
1584	719.42	1436.83	1436.68	0.15	0	42	0.029	1	K.GDVHIIPNDMGNR.I
1592	725.45	1448.88	1448.75	0.12	0	102	3e-08	1	R.ITPSVVAFTETER.L
1716	851.43	1700.84	1700.78	0.06	0	88	7.2e-07	1	R.VEVDSLTEGFDFSEK.I

Proteins matching the same set of peptides:

gi|50659756 **Mass:** 71330 **Score:** 198 **Queries matched:** 5

heat shock protein 70 [Trypanosoma cruzi]

gi|71415505 **Mass:** 71272 **Score:** 198 **Queries matched:** 5

glucose-regulated protein 78 [Trypanosoma cruzi strain CL Brener]

---

4. gi|71414968 **Mass:** 125435 **Score:** 194 **Queries matched:** 6

microtubule-associated protein [Trypanosoma cruzi strain CL Brener]

Check to include this hit in error tolerant search or archive report

Query	Observed	Mr(expt)	Mr(calc)	Delta	Miss	Score	Expect	Rank	Peptide
1595	726.94	1451.87	1451.73	0.15	0	74	1.9e-05	1	R.ALPLEEQEDVGPR.H
1596	727.46	1452.90	1452.71	0.19	0	72	3.1e-05	1	R.ALPLEEEEDVGPR.H
1600	734.41	1466.81	1466.70	0.11	0	67	0.00011	1	R.ALPQEEQEDVGPR.H
1601	734.95	1467.89	1467.68	0.20	0	65	0.00014	1	R.ALPQEEEEDVGPR.H
1761	900.05	1798.09	1797.85	0.24	0	30	0.45	1	R.STTQDAYRPVDPSAYK.R
1762	600.38	1798.13	1797.85	0.28	0	(18)	7.7	1	R.STTQDAYRPVDPSAYK.R

Proteins matching the same set of peptides:

gi|71400377 **Mass:** 85164 **Score:** 194 **Queries matched:** 6

microtubule-associated protein [Trypanosoma cruzi strain CL Brener]

---

5. gi|71662736 **Mass:** 42788 **Score:** 176 **Queries matched:** 7

hypothetical protein [Trypanosoma cruzi strain CL Brener]

☐ Check to include this hit in error tolerant search or archive report

	Query	Observed	Mr(expt)	Mr(calc)	Delta	Miss	Score	Expect	Rank	Peptide
☑	1072	518.42	1034.83	1034.56	0.27	0	40	0.045	1	R.FVLASIEEK.E
☑	259	620.39	1238.76	1238.56	0.20	0	55	0.0049	1	K.APEAAAEFDYR.E
☑	1435	631.37	1260.73	1260.56	0.17	0	67	0.00012	1	R.DLNSAEEEAQR.S
☑	1474	649.40	1296.78	1296.60	0.18	0	66	0.00014	1	K.VYASQEDMINK.N
☑	1753	896.02	1790.02	1789.96	0.06	0	63	0.00027	1	K.AVESQLQVYSEVIGLR.K
☑	1318	1178.00	2353.98	2354.07	-0.08	0	40	0.14	1	R.ELGEAAVQEFEEYSSASGAAPGR.R
☑	2003	785.75	2354.24	2354.07	0.17	0	(13)	23	1	R.ELGEAAVQEFEEYSSASGAAPGR.R

6. gi|71410853 **Mass:** 10694 **Score:** 160 **Queries matched:** 5
10 kDa heat shock protein [Trypanosoma cruzi strain CL Brener]
☐ Check to include this hit in error tolerant search or archive report

	Query	Observed	Mr(expt)	Mr(calc)	Delta	Miss	Score	Expect	Rank	Peptide
☑	1325	591.43	1180.85	1180.68	0.17	0	45	0.015	1	K.AGVLIPEQVAGK.V
☑	1508	665.49	1328.97	1328.73	0.24	0	102	3.5e-08	1	K.VNEGTVVAVAAATK.D
☑	70	444.02	1329.03	1328.73	0.30	0	(37)	0.36	1	K.VNEGTVVAVAAATK.D
☑	1690	832.02	1662.02	1661.85	0.17	0	35	0.16	1	K.VDDTVLLPEFGGSSVK.V
☑	1965	1129.63	2257.24	2257.08	0.16	0	79	5e-06	1	K.VEGEEFFLYNEDSLLGVLQG.-

Proteins matching the same set of peptides:
gi|71410857 **Mass:** 20085 **Score:** 160 **Queries matched:** 5
10 kDa heat shock protein [Trypanosoma cruzi strain CL Brener]

7. gi|71413559 **Mass:** 44080 **Score:** 158 **Queries matched:** 4
hypothetical protein [Trypanosoma cruzi strain CL Brener]
☐ Check to include this hit in error tolerant search or archive report

	Query	Observed	Mr(expt)	Mr(calc)	Delta	Miss	Score	Expect	Rank	Peptide
☑	1341	594.43	1186.84	1186.63	0.21	0	63	0.0003	1	K.ALTEEWILGR.K
☑	1582	717.95	1433.89	1433.71	0.19	0	69	6.6e-05	1	R.FHEQTSVNLFGR.A
☑	91	482.12	1443.33	1442.84	0.49	0	20	14	1	R.VLALNNPPPRPQK.A

	Query	Observed	Mr(expt)	Mr(calc)	Delta	Miss	Score	Expect	Rank	Peptide
☑	1708	846.96	1691.91	1691.85	0.06	0	94	2e-07	1	K.ANESLDVLNLGQYTR.E

---

8. gi|111115853 **Mass:** 49628 **Score:** 157 **Queries matched:** 4
beta tubulin [Trypanosoma evansi]
☐ Check to include this hit in error tolerant search or archive report

	Query	Observed	Mr(expt)	Mr(calc)	Delta	Miss	Score	Expect	Rank	Peptide
	274	627.12	1252.24	1252.77	-0.53	1	6	4e+02	2	R.KLAVNLVPFPR.L
☑	1516	671.43	1340.85	1340.64	0.21	0	87	8.8e-07	1	R.INVYFDEATGGR.Y
☑	1589	723.93	1445.85	1445.68	0.17	0	41	0.038	1	K.EVDEQMLNVQNK.N
☑	1785	924.50	1846.98	1846.87	0.11	0	90	5.4e-07	1	R.EIVCVQAGQCGNQIGSK.F + 2 Carbamidomethyl (C)

Proteins matching the same set of peptides:
gi|115504281 **Mass:** 49672 **Score:** 157 **Queries matched:** 4
beta tubulin [Trypanosoma brucei]
gi|169125731 **Mass:** 49655 **Score:** 157 **Queries matched:** 4
beta-tubulin [Trypanosoma evansi]
gi|1220547 **Mass:** 49410 **Score:** 157 **Queries matched:** 4
beta tubulin
gi|3915883 **Mass:** 49541 **Score:** 157 **Queries matched:** 4
Tubulin beta chain (Beta-tubulin)
gi|18568139 **Mass:** 49668 **Score:** 157 **Queries matched:** 4
beta tubulin 1.9 [Trypanosoma cruzi]
gi|71656281 **Mass:** 49668 **Score:** 157 **Queries matched:** 4
beta tubulin [Trypanosoma cruzi strain CL Brener]

---

9. gi|119859 **Mass:** 23721 **Score:** 154 **Queries matched:** 7
RecName: Full=Flagellar calcium-binding protein; Short=FCABP; AltName: Full=1F8 protein; AltName: F
☐ Check to include this hit in error tolerant search or archive report

	Query	Observed	Mr(expt)	Mr(calc)	Delta	Miss	Score	Expect	Rank	Peptide
☑	721	439.33	876.65	876.43	0.22	0	46	0.014	1	K.LDEFTPR.V
☑	970	495.35	988.69	988.52	0.16	0	45	0.016	1	K.VEDPAALFK.E
☑	1564	707.88	1413.75	1413.59	0.16	0	51	0.0036	1	K.LDADGDPDNVPESA.-

☑	1565	707.88	1413.75	1413.59	0.16	0	(37)	0.099	1	K.LDADGDPDNVPESA.-
☑	1603	737.93	1473.84	1473.68	0.17	0	78	8.4e-06	1	K.GSEDFVEFLEFR.L
☑	1768	607.92	1820.74	1820.84	-0.11	0	27	1	1	K.LCYDEVHSGCLEVLK.L + 2 Carbamidomethyl (C)
☑	1832	979.63	1957.24	1956.92	0.32	0	62	0.00027	1	K.NGTGSVTFDEFAAWASAVK.L

Proteins matching the same set of peptides:

gi|1552212 **Mass:** 23727 **Score:** 154 **Queries matched:** 7
calcium-binding protein [Trypanosoma cruzi]

gi|71406002 **Mass:** 23827 **Score:** 154 **Queries matched:** 7
flagellar calcium-binding protein [Trypanosoma cruzi strain CL Brener]

gi|71406004 **Mass:** 23799 **Score:** 154 **Queries matched:** 7
flagellar calcium-binding protein [Trypanosoma cruzi strain CL Brener]

gi|71412217 **Mass:** 23707 **Score:** 154 **Queries matched:** 7
calcium-binding protein [Trypanosoma cruzi strain CL Brener]

gi|71656918 **Mass:** 23713 **Score:** 154 **Queries matched:** 7
flagellar calcium-binding protein [Trypanosoma cruzi strain CL Brener]

---

10. gi|91983201 **Mass:** 42807 **Score:** 146 **Queries matched:** 4
beta tubulin [Trypanosoma grayi]

☐ Check to include this hit in error tolerant search or archive report

	Query	Observed	Mr(expt)	Mr(calc)	Delta	Miss	Score	Expect	Rank	Peptide
	274	627.12	1252.24	1252.77	-0.53	1	6	4e+02	2	R.KLAVNLVPFPR.L
	1516	671.43	1340.85	1340.64	0.21	0	87	8.8e-07	1	R.INVYFDEATGGR.Y
	1785	924.50	1846.98	1846.87	0.11	0	90	5.4e-07	1	R.EIVCVQAGQCGNQIGSK.F + 2 Carbamidomethyl (C)
☑	850	937.23	1872.45	1871.98	0.48	0	1	1.4e+03	1	**R.IAHSLGGGTGSGMGTLLISK.L + Oxidation (M)**

---

11. gi|10673 **Mass:** 14649 **Score:** 144 **Queries matched:** 5
unnamed protein product [Trypanosoma cruzi]

☐ Check to include this hit in error tolerant search or archive report

	Query	Observed	Mr(expt)	Mr(calc)	Delta	Miss	Score	Expect	Rank	Peptide
☑	1120	533.36	1064.70	1064.55	0.15	0	(31)	0.33	1	R.TLADYNIQK.E
☑	138	533.40	1064.79	1064.55	0.24	0	44	0.054	1	R.TLADYNIQK.E

☑	1126	534.41	1066.81	1066.61	0.20	0	46	0.012	1	K.ESTLHLVLR.L
☑	1734	874.52	1747.02	1746.89	0.13	0	99	5.8e-08	1	K.TIALEVESSDTIENVK.A
☑	1735	583.36	1747.07	1746.89	0.18	0	(66)	0.00013	1	K.TIALEVESSDTIENVK.A

Proteins matching the same set of peptides:

gi|10674 **Mass:** 8763 **Score:** 144 **Queries matched:** 5
unnamed protein product [Trypanosoma cruzi]

gi|136677 **Mass:** 8504 **Score:** 144 **Queries matched:** 5
RecName: Full=Ubiquitin

gi|162337 **Mass:** 40106 **Score:** 144 **Queries matched:** 5
ubiquitin precursor

---

12. gi|71405064 **Mass:** 10861 **Score:** 133 **Queries matched:** 3
60S acidic ribosomal protein P2 [Trypanosoma cruzi strain CL Brener]

☐ Check to include this hit in error tolerant search or archive report

	Query	Observed	Mr(expt)	Mr(calc)	Delta	Miss	Score	Expect	Rank	Peptide
☑	1222	558.90	1115.78	1115.62	0.16	0	92	3.2e-07	1	R.SVATLVAEGAAK.M
☑	1810	952.92	1903.84	1903.73	0.10	1	68	8.2e-05	1	K.KEEEEEDDDMGFGLFD.-
☑	2104	902.56	2704.66	2704.36	0.30	0	25	1.3	1	K.MSAVAVSAAPAAGGAAAPAAAAGGAAAPAAADAK.K

---

13. gi|162030 **Mass:** 16829 **Score:** 130 **Queries matched:** 2
calmodulin A

☐ Check to include this hit in error tolerant search or archive report

	Query	Observed	Mr(expt)	Mr(calc)	Delta	Miss	Score	Expect	Rank	Peptide
☑	1524	675.46	1348.90	1348.62	0.28	0	87	1e-06	1	K.LTDEEVDEMIR.E
☑	1819	964.50	1926.98	1926.85	0.13	0	73	2.3e-05	1	R.EADVDGDGQINYEEFVK.M

Proteins matching the same set of peptides:

gi|71405209 **Mass:** 16814 **Score:** 130 **Queries matched:** 2
calmodulin [Trypanosoma cruzi strain CL Brener]

gi|71411702 **Mass:** 23555 **Score:** 130 **Queries matched:** 2

calmodulin [Trypanosoma cruzi strain CL Brener]
gi|71411704 **Mass:** 16824 **Score:** 130 **Queries matched:** 2
calmodulin [Trypanosoma cruzi strain CL Brener]
gi|74025586 **Mass:** 16828 **Score:** 130 **Queries matched:** 2
calmodulin [Trypanosoma brucei TREU927]
gi|122063212 **Mass:** 16845 **Score:** 130 **Queries matched:** 2
Calmodulin (CaM)

---

14. gi|120679 **Mass:** 39036 **Score:** 128 **Queries matched:** 5
RecName: Full=Glyceraldehyde-3-phosphate dehydrogenase, glycosomal; Short=GAPDH
☐ Check to include this hit in error tolerant search or archive report

	Query	Observed	Mr(expt)	Mr(calc)	Delta	Miss	Score	Expect	Rank	Peptide
	580	406.29	810.57	810.41	0.17	0	15	11	5	K.LTGMSFR.V
	606	412.81	823.61	823.43	0.18	0	3	1.8e+02	8	K.AAAEGHLR.G
☑	1547	692.96	1383.90	1383.77	0.13	0	28	0.74	1	R.AAAVNIIPSTTGAAK.A
☑	205	596.60	1786.79	1786.95	-0.16	0	(59)	0.002	1	R.VPTPDVSVVDLTFTAAR.D
☑	1751	894.56	1787.10	1786.95	0.15	0	102	3.2e-08	1	R.VPTPDVSVVDLTFTAAR.D

Proteins matching the same set of peptides:
gi|53849823 **Mass:** 35106 **Score:** 128 **Queries matched:** 5
glycosomal glyceraldehyde phosphate dehydrogenase [Trypanosoma cruzi]
gi|53849825 **Mass:** 32281 **Score:** 128 **Queries matched:** 5
glycosomal glyceraldehyde phosphate dehydrogenase [Trypanosoma cruzi]
gi|71422448 **Mass:** 39008 **Score:** 128 **Queries matched:** 5
glyceraldehyde 3-phosphate dehydrogenase [Trypanosoma cruzi strain CL Brener]
gi|71650185 **Mass:** 38993 **Score:** 128 **Queries matched:** 5
glyceraldehyde 3-phosphate dehydrogenase [Trypanosoma cruzi strain CL Brener]
gi|159157547 **Mass:** 34955 **Score:** 128 **Queries matched:** 5
glycosomal glyceraldehyde-3-phosphate dehydrogenase [Trypanosoma cruzi]
gi|169264614 **Mass:** 31557 **Score:** 128 **Queries matched:** 5
glycosomal glyceraldehyde-3-phosphate dehydrogenase [uncultured kinetoplastid]
gi|169264616 **Mass:** 29907 **Score:** 128 **Queries matched:** 5
glycosomal glyceraldehyde-3-phosphate dehydrogenase [uncultured kinetoplastid]

---

15. gi|71649650 **Mass:** 33266 **Score:** 110 **Queries matched:** 2
surface protein TolT [Trypanosoma cruzi strain CL Brener]
☐ Check to include this hit in error tolerant search or archive report

Query	Observed	Mr(expt)	Mr(calc)	Delta	Miss	Score	Expect	Rank	Peptide
1161	**544.84**	**1087.66**	**1087.62**	**0.04**	**1**	**16**	**15**	**5**	**K.LSATKAAGLEK.E**
1615	751.04	1500.07	1499.83	0.23	0	110	5.3e-09	1	R.TLAQDVAATASALLR.Q

Proteins matching the same set of peptides:
gi|71411130 **Mass:** 33092 **Score:** 110 **Queries matched:** 2
surface protein TolT [Trypanosoma cruzi strain CL Brener]
gi|71411132 **Mass:** 33020 **Score:** 110 **Queries matched:** 2
surface protein TolT [Trypanosoma cruzi strain CL Brener]

---

16. gi|71659663 **Mass:** 31532 **Score:** 110 **Queries matched:** 1
hypothetical protein [Trypanosoma cruzi strain CL Brener]
☐ Check to include this hit in error tolerant search or archive report

Query	Observed	Mr(expt)	Mr(calc)	Delta	Miss	Score	Expect	Rank	Peptide
☑ 1681	**818.47**	**1634.93**	**1634.78**	**0.15**	**0**	**110**	**5.2e-09**	**1**	**R.MVSENESAINDVVTK.L**

---

17. gi|18277736 **Mass:** 59492 **Score:** 106 **Queries matched:** 3
RecName: Full=Chaperonin HSP60, mitochondrial; Short=Protein Cpn60; AltName: Full=groEL protein; Al
☐ Check to include this hit in error tolerant search or archive report

Query	Observed	Mr(expt)	Mr(calc)	Delta	Miss	Score	Expect	Rank	Peptide
1503	659.93	1317.85	1317.69	0.15	0	87	1.1e-06	1	R.NVIIEQSYGAPK.I
380	696.47	1390.93	1390.73	0.19	0	50	0.013	1	K.AELEDAFVLVSAK.K
827	**925.21**	**1848.41**	**1848.89**	**-0.48**	**0**	**1**	**1.4e+03**	**2**	**K.VLENTDAAVGYDAQLDR.Y**

Proteins matching the same set of peptides:
gi|71747654 **Mass:** 59466 **Score:** 106 **Queries matched:** 3
chaperonin Hsp60 [Trypanosoma brucei TREU927]

---

18. gi|71667953 **Mass:** 45869 **Score:** 91 **Queries matched:** 4
hypothetical protein [Trypanosoma cruzi strain CL Brener]
☐ Check to include this hit in error tolerant search or archive report

	Query	Observed	Mr(expt)	Mr(calc)	Delta	Miss	Score	Expect	Rank	Peptide
☑	1182	549.90	1097.79	1097.57	0.22	0	29	0.54	1	K.NVLPEEIER.V
☑	1372	602.83	1203.64	1203.63	0.00	0	41	0.046	1	R.IQSILSETGEK.E
☑	1548	462.31	1383.91	1383.64	0.27	0	66	0.00011	1	R.HHSDDEVAAYIK.E
☑	1577	714.47	1426.92	1426.73	0.19	0	54	0.002	1	K.ELPELQVLSEDR.A

---

19. gi|71398774 **Mass:** 29630 **Score:** 90 **Queries matched:** 1
hypothetical protein [Trypanosoma cruzi strain CL Brener]
☐ Check to include this hit in error tolerant search or archive report

	Query	Observed	Mr(expt)	Mr(calc)	Delta	Miss	Score	Expect	Rank	Peptide
☑	1327	591.90	1181.78	1181.57	0.21	0	90	3.6e-07	1	K.FSVDVEYAPR.C

Proteins matching the same set of peptides:
gi|71398776 **Mass:** 27357 **Score:** 90 **Queries matched:** 1
hypothetical protein [Trypanosoma cruzi strain CL Brener]
gi|71398971 **Mass:** 29609 **Score:** 90 **Queries matched:** 1
hypothetical protein [Trypanosoma cruzi strain CL Brener]
gi|71404900 **Mass:** 29621 **Score:** 90 **Queries matched:** 1
hypothetical protein [Trypanosoma cruzi strain CL Brener]
gi|71661320 **Mass:** 29622 **Score:** 90 **Queries matched:** 1
hypothetical protein [Trypanosoma cruzi strain CL Brener]
gi|71668366 **Mass:** 29617 **Score:** 90 **Queries matched:** 1
hypothetical protein [Trypanosoma cruzi strain CL Brener]

---

20. gi|2961256 **Mass:** 34112 **Score:** 90 **Queries matched:** 3
putative malate dehydrogenase [Trypanosoma cruzi]
☐ Check to include this hit in error tolerant search or archive report

Query	Observed	Mr(expt)	Mr(calc)	Delta	Miss	Score	Expect	Rank	Peptide

Query	Observed	Mr(expt)	Mr(calc)	Delta	Miss	Score	Expect	Rank	Peptide
117	515.43	1028.85	1028.59	0.26	0	19	21	2	R.VQVAGTEVVK.A
1281	578.46	1154.91	1154.70	0.21	0	77	1.2e-05	1	K.LLGVSLLDGLR.A
1368	602.39	1202.78	1202.62	0.15	0	54	0.0024	1	K.EMLEEAVGVVK.K

Proteins matching the same set of peptides:

gi|71423452 **Mass:** 34063 **Score:** 90 **Queries matched:** 3
glycosomal malate dehydrogenase [Trypanosoma cruzi strain CL Brener]

gi|71656813 **Mass:** 34046 **Score:** 90 **Queries matched:** 3
glycosomal malate dehydrogenase [Trypanosoma cruzi strain CL Brener]

---

21. gi|295361 **Mass:** 71419 **Score:** 88 **Queries matched:** 3
BiP/GRP78

☐ Check to include this hit in error tolerant search or archive report

Query	Observed	Mr(expt)	Mr(calc)	Delta	Miss	Score	Expect	Rank	Peptide
694	435.27	868.53	868.51	0.01	0	25	1.3	2	K.GTLVPVQR.V
1716	851.43	1700.84	1700.78	0.06	0	88	7.2e-07	1	R.VEVDSLTEGFDFSEK.I
1954	746.80	2237.39	2237.20	0.19	2	1	3.7e+02	4	K.LGGKLDPNDKAAVETAVAEAIR.F

Proteins matching the same set of peptides:

gi|71755837 **Mass:** 71391 **Score:** 88 **Queries matched:** 3
glucose-regulated protein 78 [Trypanosoma brucei TREU927]

---

22. gi|71402480 **Mass:** 37891 **Score:** 86 **Queries matched:** 1
hypothetical protein [Trypanosoma cruzi strain CL Brener]

☐ Check to include this hit in error tolerant search or archive report

Query	Observed	Mr(expt)	Mr(calc)	Delta	Miss	Score	Expect	Rank	Peptide
1426	626.96	1251.91	1251.68	0.22	0	86	1e-06	1	R.HAELVQLSDLK.E

Proteins matching the same set of peptides:

gi|71418019 **Mass:** 37850 **Score:** 86 **Queries matched:** 1
hypothetical protein [Trypanosoma cruzi strain CL Brener]

---

23. gi|438910 Mass: 43750 Score: 83 Queries matched: 3

elongation factor 1-alpha

☐ Check to include this hit in error tolerant search or archive report

Query	Observed	Mr(expt)	Mr(calc)	Delta	Miss	Score	Expect	Rank	Peptide
☑ 941	488.38	974.76	974.54	0.21	0	59	0.00095	1	R.LPLQDVYK.I
☑ 1047	513.39	1024.76	1024.60	0.16	0	62	0.00026	1	K.IGGIGTVPVGR.V
☑ 644	841.79	2522.36	2522.19	0.18	0	31	0.96	1	K.SIEMHHEQLAEATPGDNVGFNVK.N

Proteins matching the same set of peptides:

gi|61207242 Mass: 47755 Score: 83 Queries matched: 3

elongation factor 1-alpha [Trypanosoma cruzi]

gi|61207387 Mass: 49063 Score: 83 Queries matched: 3

elongation factor 1-alpha [Trypanosoma rangeli]

gi|71746818 Mass: 37784 Score: 83 Queries matched: 3

elongation factor 1-alpha [Trypanosoma brucei TREU927]

gi|71746820 Mass: 49075 Score: 83 Queries matched: 3

elongation factor 1-alpha [Trypanosoma brucei TREU927]

---

24. gi|91983200 Mass: 49749 Score: 81 Queries matched: 3

alpha tubulin [Trypanosoma grayi]

☐ Check to include this hit in error tolerant search or archive report

Query	Observed	Mr(expt)	Mr(calc)	Delta	Miss	Score	Expect	Rank	Peptide
☑ 1721	572.73	1715.17	1714.91	0.26	0	(45)	0.015	1	R.AVFLDLEPTVVDEIR.T
☑ 1722	858.61	1715.21	1714.91	0.30	0	76	1.3e-05	1	R.AVFLDLEPTVVDEIR.T
☑ 925	971.42	2911.25	2911.34	-0.09	1	3	6.7e+02	1	K.YMACCLMYRGDVVPYDVNAAVATIK.T + 2 Carbamidomethyl (C); 2 Oxidation (M

---

25. gi|133055 Mass: 10747 Score: 81 Queries matched: 3

60S acidic ribosomal protein P1

☐ Check to include this hit in error tolerant search or archive report

Query	Observed	Mr(expt)	Mr(calc)	Delta	Miss	Score	Expect	Rank	Peptide
☑ 1220	558.86	1115.70	1115.58	0.11	0	24	1.9	1	K.NVDINDVLSK.V

1810	952.92	1903.84	1903.73	0.10	1	68	8.2e-05	1	K.KEEEEEDDDMGFGLFD.-
☑ 2072	**850.54**	**2548.60**	**2548.34**	**0.26**	**0**	**34**	**0.22**	**1**	**K.VSFGGVAPAAGGATAAPAAAAAAAAPAAAAAK.K**

---

26. gi\|71418715 **Mass:** 10747 **Score:** 81 **Queries matched:** 3
60S acidic ribosomal protein [Trypanosoma cruzi strain CL Brener]
☐ Check to include this hit in error tolerant search or archive report

Query	Observed	Mr(expt)	Mr(calc)	Delta	Miss	Score	Expect	Rank	Peptide
1220	558.86	1115.70	1115.58	0.11	0	24	1.9	1	K.NVDINDVLSK.V
1810	952.92	1903.84	1903.73	0.10	1	68	8.2e-05	1	K.KEEEEEDDDMGFGLFD.-
2072	850.54	2548.60	2548.34	0.26	0	34	0.22	1	K.VSFGGVAPAAGGATAAPAAAAAAAPAAAAAAK.K

---

27. gi\|121960 **Mass:** 13884 **Score:** 81 **Queries matched:** 1
Histone H2A.1
☐ Check to include this hit in error tolerant search or archive report

Query	Observed	Mr(expt)	Mr(calc)	Delta	Miss	Score	Expect	Rank	Peptide
☑ 1239	**563.90**	**1125.78**	**1125.57**	**0.21**	**0**	**81**	**3.6e-06**	**1**	**R.HDDDIGTLLK.N**

Proteins matching the same set of peptides:
gi\|121969 **Mass:** 13811 **Score:** 81 **Queries matched:** 1
Histone H2A.2
gi\|16973360 **Mass:** 13883 **Score:** 81 **Queries matched:** 1
histone H2A [Leishmania infantum]
gi\|73536838 **Mass:** 13814 **Score:** 81 **Queries matched:** 1
histone H2A [Leishmania major strain Friedlin]
gi\|73536842 **Mass:** 13841 **Score:** 81 **Queries matched:** 1
histone H2A [Leishmania major strain Friedlin]

---

28. gi\|71416147 **Score:** 81 **Queries matched:** 1
histone H2A [Trypanosoma cruzi strain CL Brener]
☐ Check to include this hit in error tolerant search or archive report

Query	Observed	Mr(expt)	Mr(calc)	Delta	Miss	Score	Expect	Rank	Peptide

1239	563.90	1125.78	1125.57	0.21	0	81	3.6e-06	1	R.HDDDLGTLLK.D

Proteins matching the same set of peptides:
gi|71416149 **Score:** 81 **Queries matched:** 1

---

29. gi|85822967 **Mass:** 34361 **Score:** 81 **Queries matched:** 3
elongation factor 1-alpha [Schizochytrium aggregatum]
☐ Check to include this hit in error tolerant search or archive report

Query	Observed	Mr(expt)	Mr(calc)	Delta	Miss	Score	Expect	Rank	Peptide
941	488.38	974.76	974.54	0.21	0	59	0.00095	1	R.LPLQDVYK.I
1047	513.39	1024.76	1024.60	0.16	0	62	0.00026	1	K.IGGIGTVPVGR.V
565	**802.18**	**1602.34**	**1602.83**	**-0.49**	**1**	**12**	**1.3e+02**	**2**	**K.ALKSGDSGMIPSIPSK.R + Oxidation (M)**

---

30. gi|156987940 **Mass:** 34902 **Score:** 81 **Queries matched:** 3
translation elongation factor 1-alpha [Phytophthora primulae]
☐ Check to include this hit in error tolerant search or archive report

Query	Observed	Mr(expt)	Mr(calc)	Delta	Miss	Score	Expect	Rank	Peptide
941	488.38	974.76	974.54	0.21	0	59	0.00095	1	R.LPLQDVYK.I
1047	513.39	1024.76	1024.60	0.16	0	62	0.00026	1	K.IGGIGTVPVGR.V
1563	**703.96**	**1405.90**	**1405.73**	**0.17**	**2**	**9**	**78**	**3**	**K.ELRRGFVASDEK.N**

---

31. gi|114865174 **Mass:** 33796 **Score:** 81 **Queries matched:** 3
translation elongation factor 1-alpha [Pythium sp. quercum]
☐ Check to include this hit in error tolerant search or archive report

Query	Observed	Mr(expt)	Mr(calc)	Delta	Miss	Score	Expect	Rank	Peptide
941	488.38	974.76	974.54	0.21	0	59	0.00095	1	R.LPLQDVYK.I
1047	513.39	1024.76	1024.60	0.16	0	62	0.00026	1	K.IGGIGTVPVGR.V
☑ 399	**709.52**	**1417.03**	**1416.76**	**0.27**	**1**	**8**	**2.5e+02**	**1**	**R.SGEVLEANPKFVK.S**

---

32. gi|85822985 **Mass:** 28912 **Score:** 81 **Queries matched:** 3

elongation factor 1-alpha [Thraustochytriidae sp. P19]

Check to include this hit in error tolerant search or archive report

Query	Observed	Mr(expt)	Mr(calc)	Delta	Miss	Score	Expect	Rank	Peptide
941	488.38	974.76	974.54	0.21	0	59	0.00095	1	R.LPLQDVYK.I
1047	513.39	1024.76	1024.60	0.16	0	62	0.00026	1	K.IGGIGTVPVGR.V
644	841.79	2522.36	2522.15	0.21	0	8	2.1e+02	3	K.SVEMHHESVPEAQPGDNVGFNVK.N + Oxidation (M)

---

33. gi|89329735 **Mass:** 50628 **Score:** 81 **Queries matched:** 3

translation elongation factor 1-alpha [Capsaspora owczarzaki]

Check to include this hit in error tolerant search or archive report

Query	Observed	Mr(expt)	Mr(calc)	Delta	Miss	Score	Expect	Rank	Peptide
941	488.38	974.76	974.54	0.21	0	59	0.00095	1	R.LPLQDVYK.I
1047	513.39	1024.76	1024.60	0.16	0	62	0.00026	1	K.IGGIGTVPVGR.V
644	841.79	2522.36	2522.15	0.21	0	8	2.1e+02	3	K.SVEMHHESLPEANPGDNVGFNVK.N + Oxidation (M)

---

34. gi|146083153 **Mass:** 49097 **Score:** 81 **Queries matched:** 3

elongation factor 1-alpha [Leishmania infantum JPCM5]

Check to include this hit in error tolerant search or archive report

Query	Observed	Mr(expt)	Mr(calc)	Delta	Miss	Score	Expect	Rank	Peptide
941	488.38	974.76	974.54	0.21	0	59	0.00095	1	R.LPLQDVYK.I
1047	513.39	1024.76	1024.60	0.16	0	62	0.00026	1	K.IGGIGTVPVGR.V
177	**576.90**	**1727.67**	**1727.97**	**-0.31**	**2**	**3**	**8e+02**	**3**	**R.DMRQTVAVGIIKGVNK.K**

Proteins matching the same set of peptides:

gi|157867408 **Mass:** 49085 **Score:** 81 **Queries matched:** 3

elongation factor 1-alpha [Leishmania major]

gi|157867412 **Mass:** 49027 **Score:** 81 **Queries matched:** 3

elongation factor 1-alpha [Leishmania major]

---

35. gi|56156664 **Mass:** 42035 **Score:** 81 **Queries matched:** 2

elongation factor 1A [Cribraria cancellata]

☐ Check to include this hit in error tolerant search or archive report

Query	Observed	Mr(expt)	Mr(calc)	Delta	Miss	Score	Expect	Rank	Peptide
941	488.38	974.76	974.54	0.21	0	59	0.00095	1	R.IPLQDVYK.I
1047	513.39	1024.76	1024.60	0.16	0	62	0.00026	1	K.IGGIGTVPVGR.V

Proteins matching the same set of peptides:

gi|56156697 **Mass:** 44176 **Score:** 81 **Queries matched:** 2
elongation factor 1A [Trichia persimilis]

gi|61661537 **Mass:** 43522 **Score:** 81 **Queries matched:** 2
elongation factor 1F-alpha [Echinostelium arboreum]

gi|50660706 **Mass:** 32133 **Score:** 81 **Queries matched:** 2
translation elongation factor 1 alpha [Phytophthora andina]

---

36. gi|59859756 **Mass:** 35861 **Score:** 81 **Queries matched:** 3
elongation factor 1 alpha [Cyclotella cryptica]

☐ Check to include this hit in error tolerant search or archive report

Query	Observed	Mr(expt)	Mr(calc)	Delta	Miss	Score	Expect	Rank	Peptide
941	488.38	974.76	974.54	0.21	0	59	0.00095	1	R.LPLQDVYK.I
1047	513.39	1024.76	1024.60	0.16	0	62	0.00026	1	K.IGGIGTVPVGR.V
614	**828.84**	**2483.49**	**2483.12**	**0.37**	**0**	**1**	**1.2e+03**	**4**	**K.SVEMHHEALAEALPGDNVGYNCK.N**

---

37. gi|704459 **Mass:** 43546 **Score:** 80 **Queries matched:** 5
elongation factor 1 alpha [Trypanosoma cruzi]

☐ Check to include this hit in error tolerant search or archive report

Query	Observed	Mr(expt)	Mr(calc)	Delta	Miss	Score	Expect	Rank	Peptide
941	488.38	974.76	974.54	0.21	0	59	0.00095	1	R.LPLQDVYK.I
1047	513.39	1024.76	1024.60	0.16	0	62	0.00026	1	K.IGGIGTVPVGR.V
☑ 2053	**831.17**	**2490.49**	**2490.27**	**0.23**	**0**	**23**	**2.1**	**1**	**R.VETGTMKPGDVVTFAPANVTTEVK.S**
☑ 2056	**836.50**	**2506.46**	**2506.26**	**0.20**	**0**	**(23)**	**2.2**	**1**	**R.VETGTMKPGDVVTFAPANVTTEVK.S + Oxidation (M)**
644	841.79	2522.36	2522.19	0.18	0	31	0.96	1	K.SIEMHHEQLAEATPGDNVGFNVK.N

Proteins matching the same set of peptides:

gi|1929445 **Mass:** 49018 **Score:** 80 **Queries matched:** 5
elongation factor 1-alpha [Trypanosoma cruzi]
gi|52424046 **Mass:** 49067 **Score:** 80 **Queries matched:** 5
elongation factor alpha G5 [Trypanosoma cruzi]
gi|61207234 **Mass:** 47727 **Score:** 80 **Queries matched:** 5
elongation factor 1-alpha [Trypanosoma cruzi]
gi|61207240 **Mass:** 47728 **Score:** 80 **Queries matched:** 5
elongation factor 1-alpha [Trypanosoma cruzi]
gi|61207250 **Mass:** 47741 **Score:** 80 **Queries matched:** 5
elongation factor 1-alpha [Trypanosoma cruzi]
gi|61207254 **Mass:** 47727 **Score:** 80 **Queries matched:** 5
elongation factor 1-alpha [Trypanosoma cruzi]
gi|61207256 **Mass:** 47843 **Score:** 80 **Queries matched:** 5
elongation factor 1-alpha [Trypanosoma cruzi]
gi|61207272 **Mass:** 47767 **Score:** 80 **Queries matched:** 5
elongation factor 1-alpha [Trypanosoma cruzi]
gi|61207288 **Mass:** 47711 **Score:** 80 **Queries matched:** 5
elongation factor 1-alpha [Trypanosoma cruzi]
gi|61207296 **Mass:** 47667 **Score:** 80 **Queries matched:** 5
elongation factor 1-alpha [Trypanosoma cruzi]
gi|71408910 **Mass:** 49083 **Score:** 80 **Queries matched:** 5
elongation factor 1-alpha (EF-1-alpha) [Trypanosoma cruzi strain CL Brener]
gi|71408922 **Mass:** 42751 **Score:** 80 **Queries matched:** 5
elongation factor 1-alpha (EF-1-alpha) [Trypanosoma cruzi strain CL Brener]
gi|71664927 **Mass:** 49097 **Score:** 80 **Queries matched:** 5
elongation factor 1-alpha (EF-1-alpha) [Trypanosoma cruzi strain CL Brener]

---

38. gi|71663174 **Mass:** 45236 **Score:** 76 **Queries matched:** 1
succinyl-CoA ligase [GDP-forming] beta-chain [Trypanosoma cruzi]
☐ Check to include this hit in error tolerant search or archive report

	Query	Observed	Mr(expt)	Mr(calc)	Delta	Miss	Score	Expect	Rank	Peptide
☑	1291	**580.45**	**1158.89**	**1158.61**	**0.28**	**0**	**76**	**1.3e-05**	**1**	**R.TLEEVEAALGK.I**

---

39. gi|71656402 **Mass:** 143742 **Score:** 76 **Queries matched:** 1

hypothetical protein [Trypanosoma cruzi strain CL Brener]

☐ Check to include this hit in error tolerant search or archive report

	Query	Observed	Mr(expt)	Mr(calc)	Delta	Miss	Score	Expect	Rank	Peptide
☑	1664	801.57	1601.12	1600.77	0.35	0	76	1.4e-05	1	R.GLQEVSEQAEDLQR.Q

Proteins matching the same set of peptides:

gi|71409302 **Mass:** 132032 **Score:** 76 **Queries matched:** 1

hypothetical protein [Trypanosoma cruzi strain CL Brener]

---

40. gi|1781355 **Score:** 75 **Queries matched:** 1

histone H2A [Trypanosoma cruzi]

☐ Check to include this hit in error tolerant search or archive report

	Query	Observed	Mr(expt)	Mr(calc)	Delta	Miss	Score	Expect	Rank	Peptide
☑	1283	578.90	1155.79	1155.56	0.23	0	75	1.6e-05	1	R.HDDDLGMLLK.D

Proteins matching the same set of peptides:

gi|2222802 **Score:** 75 **Queries matched:** 1

gi|18266856 **Score:** 75 **Queries matched:** 1

gi|71409700 **Score:** 75 **Queries matched:** 1

gi|71409702 **Score:** 75 **Queries matched:** 1

gi|71649895 **Score:** 75 **Queries matched:** 1

gi|71664770 **Score:** 75 **Queries matched:** 1

gi|71664802 **Score:** 75 **Queries matched:** 1

---

41. gi|13384081 **Mass:** 13942 **Score:** 75 **Queries matched:** 1

histone H2A [Leishmania infantum]

☐ Check to include this hit in error tolerant search or archive report

Query	Observed	Mr(expt)	Mr(calc)	Delta	Miss	Score	Expect	Rank	Peptide
1283	578.90	1155.79	1155.56	0.23	0	75	1.6e-05	1	R.HDDDIGMLLK.N

Proteins matching the same set of peptides:

gi|16973355 **Mass:** 13841 **Score:** 75 **Queries matched:** 1
histone H2A [Leishmania infantum]
gi|16973359 **Mass:** 13872 **Score:** 75 **Queries matched:** 1
histone H2A [Leishmania infantum]

---

42. gi|89329739 **Mass:** 53354 **Score:** 73 **Queries matched:** 3
heat shock protein 70 [Capsaspora owczarzaki]
☐ Check to include this hit in error tolerant search or archive report

	Query	Observed	Mr(expt)	Mr(calc)	Delta	Miss	Score	Expect	Rank	Peptide
☑	1404	**614.92**	**1227.83**	**1227.62**	**0.21**	**0**	**69**	**6.8e-05**	**1**	**K.VEIIANDQGNR.T**
	1477	650.40	1298.79	1298.60	0.19	0	15	14	8	R.FEELCADLFR.G + Carbamidomethyl (C)
☑	1609	**744.47**	**1486.92**	**1486.69**	**0.23**	**0**	**44**	**0.021**	**1**	**R.TTPSYVAFTDTER.L**

---

43. gi|183232119 **Mass:** 24772 **Score:** 73 **Queries matched:** 3
heat shock protein70, hsp70A2 [Entamoeba histolytica HM-1:IMSS]
☐ Check to include this hit in error tolerant search or archive report

Query	Observed	Mr(expt)	Mr(calc)	Delta	Miss	Score	Expect	Rank	Peptide
1404	614.92	1227.83	1227.62	0.21	0	69	6.8e-05	1	R.VEIIANDQGNR.T
1439	**633.38**	**1264.74**	**1264.59**	**0.15**	**0**	**5**	**1.8e+02**	**7**	**R.FSDPTIQNDTK.H**
1609	744.47	1486.92	1486.69	0.23	0	44	0.021	1	R.TTPSYVAFTDTER.L

Proteins matching the same set of peptides:
gi|183232244 **Mass:** 73979 **Score:** 73 **Queries matched:** 3
heat shock protein70, hsp70A2 [Entamoeba histolytica HM-1:IMSS]

---

44. gi|10626 **Mass:** 74234 **Score:** 73 **Queries matched:** 3
Heat shock protein 70 [Trypanosoma cruzi]
☐ Check to include this hit in error tolerant search or archive report

Query	Observed	Mr(expt)	Mr(calc)	Delta	Miss	Score	Expect	Rank	Peptide
1404	614.92	1227.83	1227.62	0.21	0	69	6.8e-05	1	R.VEIIANDQGNR.T
1609	744.47	1486.92	1486.69	0.23	0	44	0.021	1	R.TTPSYVAFTDTER.L
469	**751.58**	**1501.15**	**1500.77**	**0.37**	**1**	**6**	**4.1e+02**	**6**	**R.GRIPEDVLMAASAR.E + Oxidation (M)**

45. gi|123603 **Score:** 69 **Queries matched:** 2
RecName: Full=Heat shock 70 kDa protein
☐ Check to include this hit in error tolerant search or archive report

Query	Observed	Mr(expt)	Mr(calc)	Delta	Miss	Score	Expect	Rank	Peptide
1404	614.92	1227.83	1227.62	0.21	0	69	6.8e-05	1	R.VEIIANDQGNR.T
1477	650.40	1298.79	1298.60	0.19	0	15	14	8	R.FEELCGELFR.G + Carbamidomethyl (C)

46. gi|58042864 **Score:** 69 **Queries matched:** 2
PPAT5 [Hyaloperonospora parasitica]
☐ Check to include this hit in error tolerant search or archive report

Query	Observed	Mr(expt)	Mr(calc)	Delta	Miss	Score	Expect	Rank	Peptide
1404	614.92	1227.83	1227.62	0.21	0	69	6.8e-05	1	K.VEIIANDQGNR.I
533	**788.04**	**2361.09**	**2361.24**	**-0.15**	**1**	**0**	**1.8e+03**	**2**	**M.NLAKICFLAAAALANAAVSGEDTK.G**

Proteins matching the same set of peptides:
gi|58042866 **Score:** 69 **Queries matched:** 2

47. gi|123592 **Mass:** 56500 **Score:** 69 **Queries matched:** 1
RecName: Full=Heat shock 70 kDa protein
☐ Check to include this hit in error tolerant search or archive report

Query	Observed	Mr(expt)	Mr(calc)	Delta	Miss	Score	Expect	Rank	Peptide
1404	614.92	1227.83	1227.62	0.21	0	69	6.8e-05	1	R.LDIIANDQGNR.T

48. gi|71650885 **Mass:** 46722 **Score:** 67 **Queries matched:** 3
ATP-dependent DEAD/H RNA helicase [Trypanosoma cruzi strain CL Brener]
☐ Check to include this hit in error tolerant search or archive report

	Query	Observed	Mr(expt)	Mr(calc)	Delta	Miss	Score	Expect	Rank	Peptide
☑	148	**547.88**	**1093.76**	**1093.55**	**0.21**	**0**	**30**	**1.4**	**1**	**R.ELQMGIFEK.G**

☑	1367	602.38	1202.75	1202.69	0.06	0	54	0.0021	1	K.TASFVIPVLEK.V
☑	1921	718.13	2151.38	2151.13	0.25	0	37	0.11	1	K.GFERPSPVQEEAIPVALQGK.D

Proteins matching the same set of peptides:
gi|71747184 **Mass:** 46439 **Score:** 67 **Queries matched:** 3
ATP-dependent DEAD-box RNA helicase [Trypanosoma brucei TREU927]

---

49. gi|5139798 **Mass:** 38369 **Score:** 62 **Queries matched:** 2
elongation factor 1 alpha [Pyrsonympha grandis]
☐ Check to include this hit in error tolerant search or archive report

	Query	Observed	Mr(expt)	Mr(calc)	Delta	Miss	Score	Expect	Rank	Peptide
	1047	513.39	1024.76	1024.60	0.16	0	62	0.00026	1	K.IGGIGTVPVGR.V
☑	1496	658.43	1314.84	1314.66	0.18	1	27	1.1	1	R.GFVAGDAKNDPPK.E

Proteins matching the same set of peptides:
gi|5139800 **Mass:** 38387 **Score:** 62 **Queries matched:** 2
elongation factor 1 alpha [Pyrsonympha grandis]
gi|7417248 **Mass:** 43987 **Score:** 62 **Queries matched:** 2
elongation factor 1 alpha [Dinenympha exilis]

---

50. gi|118766700 **Mass:** 44258 **Score:** 62 **Queries matched:** 2
elongation factor-1 alpha [Oxymonadida environmental sample]
☐ Check to include this hit in error tolerant search or archive report

Query	Observed	Mr(expt)	Mr(calc)	Delta	Miss	Score	Expect	Rank	Peptide
1047	513.39	1024.76	1024.60	0.16	0	62	0.00026	1	K.IGGIGTVPVGR.V
1169	545.84	1089.67	1089.55	0.12	1	4	1.9e+02	7	K.CGGIDKGAIEK.F

---

51. gi|71401749 **Mass:** 26271 **Score:** 61 **Queries matched:** 1
hypothetical protein [Trypanosoma cruzi strain CL Brener]
☐ Check to include this hit in error tolerant search or archive report

Query	Observed	Mr(expt)	Mr(calc)	Delta	Miss	Score	Expect	Rank	Peptide

1920	718.00	2150.98	2150.91	0.07	0	61	0.00047	1	R.LADHCLASAGNQNTCSSMSK.L + 2 Carbamidomethyl (C)

Proteins matching the same set of peptides:
gi|71415005 **Mass:** 26250 **Score:** 61 **Queries matched:** 1
hypothetical protein [Trypanosoma cruzi strain CL Brener]

---

52. gi|438596 **Score:** 59 **Queries matched:** 1
elongation factor 1-alpha
☐ Check to include this hit in error tolerant search or archive report

Query	Observed	Mr(expt)	Mr(calc)	Delta	Miss	Score	Expect	Rank	Peptide
941	488.38	974.76	974.54	0.21	0	59	0.00095	1	R.LPIQDVYK.I

Proteins matching the same set of peptides:

gi|585082	**Score:** 59	**Queries matched:** 1
gi|1322226	**Score:** 59	**Queries matched:** 1
gi|2147344	**Score:** 59	**Queries matched:** 1
gi|2522342	**Score:** 59	**Queries matched:** 1
gi|6017764	**Score:** 59	**Queries matched:** 1
gi|6017766	**Score:** 59	**Queries matched:** 1
gi|6017768	**Score:** 59	**Queries matched:** 1
gi|6017770	**Score:** 59	**Queries matched:** 1
gi|6017772	**Score:** 59	**Queries matched:** 1
gi|6017774	**Score:** 59	**Queries matched:** 1
gi|6017776	**Score:** 59	**Queries matched:** 1
gi|6017778	**Score:** 59	**Queries matched:** 1
gi|6017780	**Score:** 59	**Queries matched:** 1
gi|6017782	**Score:** 59	**Queries matched:** 1
gi|7417250	**Score:** 59	**Queries matched:** 1
gi|34596984	**Score:** 59	**Queries matched:** 1
gi|159108518	**Score:** 59	**Queries matched:** 1

---

53. gi|71401492 **Mass:** 46811 **Score:** 56 **Queries matched:** 1
fatty acid desaturase [Trypanosoma cruzi strain CL Brener]
☐ Check to include this hit in error tolerant search or archive report

	Query	Observed	Mr(expt)	Mr(calc)	Delta	Miss	Score	Expect	Rank	Peptide
☑	1638	776.96	1551.91	1551.80	0.11	0	56	0.001	1	R.VDNLTVAPGPPDVMK.A

54. gi|167375825 **Mass:** 24465 **Score:** 56 **Queries matched:** 1
hypothetical protein [Entamoeba dispar SAW760]
☐ Check to include this hit in error tolerant search or archive report

	Query	Observed	Mr(expt)	Mr(calc)	Delta	Miss	Score	Expect	Rank	Peptide
☑	1486	651.98	1301.94	1301.68	0.25	0	56	0.0013	1	K.DVTIDISIAEAR.I

55. gi|71651158 **Mass:** 16325 **Score:** 54 **Queries matched:** 5
hypothetical protein [Trypanosoma cruzi strain CL Brener]
☐ Check to include this hit in error tolerant search or archive report

	Query	Observed	Mr(expt)	Mr(calc)	Delta	Miss	Score	Expect	Rank	Peptide
	938	975.52	974.51	974.45	0.06	0	6	3.6e+02	2	K.NMYTGVYK.A
☑	1169	545.84	1089.67	1089.54	0.12	0	14	20	1	R.NSGSLSGVVDR.R
	1171	545.86	1089.70	1089.54	0.16	0	(10)	53	5	R.NSGSLSGVVDR.R
☑	1614	750.46	1498.90	1498.73	0.17	0	51	0.0042	1	K.QFTSTDADLLFNK.V
☑	1792	931.94	1861.86	1861.87	-0.02	0	35	0.16	1	K.SVEELIADISSCSPEAR.A + Carbamidomethyl (C)

56. gi|9954108 **Mass:** 34491 **Score:** 53 **Queries matched:** 2
RNA binding protein RGGm [Trypanosoma cruzi]
☐ Check to include this hit in error tolerant search or archive report

	Query	Observed	Mr(expt)	Mr(calc)	Delta	Miss	Score	Expect	Rank	Peptide
☑	1569	710.44	1418.86	1418.70	0.16	0	53	0.0027	1	R.AVVEFVTPEDASR.A
☑	1913	714.07	2139.20	2139.17	0.03	2	3	2.2e+02	1	K.RTSLRVLHVTAALMQGWGK.G + Oxidation (M)

Proteins matching the same set of peptides:
gi|71410145 **Mass:** 34635 **Score:** 53 **Queries matched:** 2
RNA-binding protein RGGm [Trypanosoma cruzi strain CL Brener]
gi|71652462 **Mass:** 33389 **Score:** 53 **Queries matched:** 2

RNA-binding protein RGGm [Trypanosoma cruzi strain CL Brener]

---

57. gi|15488542 **Mass:** 16446 **Score:** 53 **Queries matched:** 1
centrin [Leishmania donovani]
☐ Check to include this hit in error tolerant search or archive report

	Query	Observed	Mr(expt)	Mr(calc)	Delta	Miss	Score	Expect	Rank	Peptide
☑	2060	839.42	2515.25	2515.12	0.12	0	53	0.0022	1	R.EAFNLFDADGSGAIDAEEMALAMK.G

Proteins matching the same set of peptides:
gi|71425751 **Mass:** 16513 **Score:** 53 **Queries matched:** 1
centrin [Trypanosoma cruzi strain CL Brener]
gi|72391340 **Mass:** 16542 **Score:** 53 **Queries matched:** 1
centrin [Trypanosoma brucei TREU927]
gi|146086774 **Mass:** 16465 **Score:** 53 **Queries matched:** 1
Ca2+-binding EF-hand protein [Leishmania infantum JPCM5]
gi|154337629 **Mass:** 16487 **Score:** 53 **Queries matched:** 1
Ca2+-binding EF-hand protein [Leishmania braziliensis MHOM/BR/75/M2904]
gi|157869481 **Mass:** 16437 **Score:** 53 **Queries matched:** 1
Ca2+-binding EF-hand protein; centrin [Leishmania major strain Friedlin]

---

58. gi|71412664 **Mass:** 60479 **Score:** 51 **Queries matched:** 1
hypothetical protein [Trypanosoma cruzi strain CL Brener]
☐ Check to include this hit in error tolerant search or archive report

	Query	Observed	Mr(expt)	Mr(calc)	Delta	Miss	Score	Expect	Rank	Peptide
☑	1648	788.98	1575.95	1575.78	0.18	0	51	0.004	1	R.SPGFNPAAVPYTPMK.T

---

59. gi|123436584 **Mass:** 114954 **Score:** 51 **Queries matched:** 1
hypothetical protein [Trichomonas vaginalis G3]
☐ Check to include this hit in error tolerant search or archive report

	Query	Observed	Mr(expt)	Mr(calc)	Delta	Miss	Score	Expect	Rank	Peptide
☑	1259	572.40	1142.78	1142.58	0.20	0	51	0.0043	1	R.SPELEEALQK.Y

60. gi|71407337 **Mass:** 16350 **Score:** 50 **Queries matched:** 5
hypothetical protein [Trypanosoma cruzi strain CL Brener]
☐ Check to include this hit in error tolerant search or archive report

	Query	Observed	Mr(expt)	Mr(calc)	Delta	Miss	Score	Expect	Rank	Peptide
	938	975.52	974.51	974.45	0.06	0	6	3.6e+02	2	K.NMYTGVYK.A
	1169	545.84	1089.67	1089.54	0.12	0	14	20	1	R.NSGSLSGVVDR.R
	1171	545.86	1089.70	1089.54	0.16	0	(10)	53	5	R.NSGSLSGVVDR.R
	1614	750.46	1498.90	1498.73	0.17	0	51	0.0042	1	K.QFTSTDADLLFNK.V
☑	**1774**	**612.35**	**1834.01**	**1833.84**	**0.17**	**0**	**27**	**1**	**1**	**K.SAEELIADISSCSPEAR.A + Carbamidomethyl (C)**

61. gi|5566209 **Mass:** 37655 **Score:** 50 **Queries matched:** 2
enolase 1 [Trypanosoma cruzi]
☐ Check to include this hit in error tolerant search or archive report

	Query	Observed	Mr(expt)	Mr(calc)	Delta	Miss	Score	Expect	Rank	Peptide
☑	**905**	**482.81**	**963.60**	**963.48**	**0.12**	**0**	**29**	**0.71**	**1**	**K.AGSFNEALR.M**
☑	**1493**	**655.47**	**1308.92**	**1308.74**	**0.18**	**0**	**50**	**0.005**	**1**	**K.NVNDVLAPALVGK.D**

Proteins matching the same set of peptides:
gi|5566210 **Mass:** 37596 **Score:** 50 **Queries matched:** 2
enolase 2 [Trypanosoma cruzi]
gi|71665461 **Mass:** 46415 **Score:** 50 **Queries matched:** 2
enolase [Trypanosoma cruzi strain CL Brener]

62. gi|160330959 **Mass:** 244710 **Score:** 50 **Queries matched:** 2
hypothetical protein HAN_1g14 [Hemiselmis andersenii]
☐ Check to include this hit in error tolerant search or archive report

	Query	Observed	Mr(expt)	Mr(calc)	Delta	Miss	Score	Expect	Rank	Peptide
	1578	476.65	1426.93	1426.84	0.08	2	(16)	12	4	K.IVCGNLKNLKLR.E + Carbamidomethyl (C)
☑	**1579**	**714.49**	**1426.96**	**1426.84**	**0.12**	**2**	**50**	**0.0052**	**1**	**K.IVCGNLKNLKLR.E + Carbamidomethyl (C)**

63. gi|91983209 **Mass:** 12465 **Score:** 47 **Queries matched:** 1
beta tubulin [Leishmania mexicana]
☐ Check to include this hit in error tolerant search or archive report

	Query	Observed	Mr(expt)	Mr(calc)	Delta	Miss	Score	Expect	Rank	Peptide
☑	2164	**1032.25**	**3093.72**	**3093.43**	**0.28**	**1**	**47**	**0.0061**	**1**	**K.FWEVIADEHGVDKTGSYQGDSDLQLER.I**

64. gi|71412308 **Mass:** 152202 **Score:** 46 **Queries matched:** 1
hypothetical protein [Trypanosoma cruzi strain CL Brener]
☐ Check to include this hit in error tolerant search or archive report

	Query	Observed	Mr(expt)	Mr(calc)	Delta	Miss	Score	Expect	Rank	Peptide
☑	1767	**607.43**	**1819.26**	**1818.95**	**0.31**	**0**	**46**	**0.014**	**1**	**R.LVVETELHPADAELQR.R**

Proteins matching the same set of peptides:
gi|71649424 **Mass:** 151616 **Score:** 46 **Queries matched:** 1
hypothetical protein [Trypanosoma cruzi strain CL Brener]

65. gi|58414949 **Score:** 46 **Queries matched:** 3
polyubiquitin [Stauracon pallidus]
☐ Check to include this hit in error tolerant search or archive report

Query	Observed	Mr(expt)	Mr(calc)	Delta	Miss	Score	Expect	Rank	Peptide
1126	534.41	1066.81	1066.61	0.20	0	46	0.012	1	K.ESTLHLVLR.L
1734	874.52	1747.02	1746.89	0.13	0	20	5.4	3	K.TITLEVEASDSIENVK.A
1735	583.36	1747.07	1746.89	0.18	0	(8)	89	6	K.TITLEVEASDSIENVK.A

66. gi|154411942 **Mass:** 27817 **Score:** 46 **Queries matched:** 1
hypothetical protein [Trichomonas vaginalis G3]
☐ Check to include this hit in error tolerant search or archive report

Query	Observed	Mr(expt)	Mr(calc)	Delta	Miss	Score	Expect	Rank	Peptide
1126	534.41	1066.81	1066.61	0.20	0	46	0.012	1	R.DTTIHLVLR.C

67. gi|11467023 **Mass:** 18594 **Score:** 46 **Queries matched:** 1
RNA polymerase alpha chain [Euglena gracilis]
☐ Check to include this hit in error tolerant search or archive report

Query	Observed	Mr(expt)	Mr(calc)	Delta	Miss	Score	Expect	Rank	Peptide
☑ 1108	**528.42**	**1054.82**	**1054.71**	**0.11**	**2**	**46**	**0.01**	**1**	**K.ILLKIEAKK.A**

Proteins matching the same set of peptides:
gi|24638474 **Mass:** 25348 **Score:** 46 **Queries matched:** 1
RecName: Full=DNA-directed RNA polymerase subunit alpha; Short=PEP; AltName: Full=Plastid-encoded R

68. gi|123437981 **Score:** 46 **Queries matched:** 1
hypothetical protein [Trichomonas vaginalis G3]
☐ Check to include this hit in error tolerant search or archive report

Query	Observed	Mr(expt)	Mr(calc)	Delta	Miss	Score	Expect	Rank	Peptide
1108	528.42	1054.82	1054.68	0.15	0	46	0.01	1	K.LLLQIQSLK.N

69. gi|123456564 **Score:** 46 **Queries matched:** 1
hypothetical protein [Trichomonas vaginalis G3]
☐ Check to include this hit in error tolerant search or archive report

Query	Observed	Mr(expt)	Mr(calc)	Delta	Miss	Score	Expect	Rank	Peptide
1108	528.42	1054.82	1054.75	0.08	2	46	0.01	1	K.LLLKIKSLK.N

70. gi|146089176 **Score:** 46 **Queries matched:** 1
vacuolar protein sorting-like protein [Leishmania infantum JPCM5]
☐ Check to include this hit in error tolerant search or archive report

Query	Observed	Mr(expt)	Mr(calc)	Delta	Miss	Score	Expect	Rank	Peptide
1108	528.42	1054.82	1054.69	0.14	1	46	0.01	1	R.LLLKLQTAR.F

Proteins matching the same set of peptides:
gi|157870858 **Score:** 46 **Queries matched:** 1

---

71. gi|123416964 **Score:** 45 **Queries matched:** 1
hypothetical protein [Trichomonas vaginalis G3]
☐ Check to include this hit in error tolerant search or archive report

Query	Observed	Mr(expt)	Mr(calc)	Delta	Miss	Score	Expect	Rank	Peptide
1108	528.42	1054.82	1054.65	0.17	1	45	0.011	5	R.ILLKNNVNK.K

---

72. gi|71659778 **Mass:** 61371 **Score:** 45 **Queries matched:** 1
poly(A)-binding protein [Trypanosoma cruzi strain CL Brener]
☐ Check to include this hit in error tolerant search or archive report

	Query	Observed	Mr(expt)	Mr(calc)	Delta	Miss	Score	Expect	Rank	Peptide
☑	1310	**585.90**	**1169.79**	**1169.64**	**0.15**	**0**	**45**	**0.013**	**1**	**K.VQEALEVLNR.H**

Proteins matching the same set of peptides:
gi|159162751 **Mass:** 9085 **Score:** 45 **Queries matched:** 1
Chain A, Solution Structure Of C-Terminal Domain From Trypanosoma Cruzi Poly(A)-Binding Protein

---

73. gi|71420441 **Mass:** 7307 **Score:** 45 **Queries matched:** 1
hypothetical protein [Trypanosoma cruzi strain CL Brener]
☐ Check to include this hit in error tolerant search or archive report

	Query	Observed	Mr(expt)	Mr(calc)	Delta	Miss	Score	Expect	Rank	Peptide
☑	1802	**630.03**	**1887.06**	**1886.90**	**0.16**	**0**	**45**	**0.018**	**1**	**R.IYDDDGNLKPGETTPPR.N**

Proteins matching the same set of peptides:
gi|71663656 **Mass:** 35448 **Score:** 45 **Queries matched:** 1
hypothetical protein [Trypanosoma cruzi strain CL Brener]

---

74. gi|225587 **Score:** 45 **Queries matched:** 2

tubulin alpha

☐ Check to include this hit in error tolerant search or archive report

Query	Observed	Mr(expt)	Mr(calc)	Delta	Miss	Score	Expect	Rank	Peptide
1721	572.73	1715.17	1714.91	0.26	0	(28)	0.86	2	K.AVFLDLEPTVIDEVR.T
1722	858.61	1715.21	1714.91	0.30	0	45	0.018	2	K.AVFLDLEPTVIDEVR.T

Proteins matching the same set of peptides:

gi|1729839 **Score:** 45 **Queries matched:** 2

gi|1755084 **Score:** 45 **Queries matched:** 2

---

75. gi|84105377 **Score:** 45 **Queries matched:** 2

alpha tubulin 2 [Rhynchopus sp. ATCC 50230]

☐ Check to include this hit in error tolerant search or archive report

Query	Observed	Mr(expt)	Mr(calc)	Delta	Miss	Score	Expect	Rank	Peptide
1721	572.73	1715.17	1714.88	0.29	0	(28)	0.86	2	R.AVMLDLEPTVIDEVR.T + Oxidation (M)
1722	858.61	1715.21	1714.88	0.33	0	45	0.018	2	R.AVMLDLEPTVIDEVR.T + Oxidation (M)

Proteins matching the same set of peptides:

gi|84105383 **Score:** 45 **Queries matched:** 2

---

76. gi|116222245 **Score:** 45 **Queries matched:** 2

alpha tubulin [Thaumatomonas sp. TMT002]

☐ Check to include this hit in error tolerant search or archive report

Query	Observed	Mr(expt)	Mr(calc)	Delta	Miss	Score	Expect	Rank	Peptide
1721	572.73	1715.17	1714.91	0.26	0	(28)	0.86	2	R.AVFIDLEPTVIDEVR.T
1722	858.61	1715.21	1714.91	0.30	0	45	0.018	2	R.AVFIDLEPTVIDEVR.T

Proteins matching the same set of peptides:

gi|116222249 **Score:** 45 **Queries matched:** 2

gi|116222289 **Score:** 45 **Queries matched:** 2

gi|156066414 **Score:** 45 **Queries matched:** 2

---

77. gi|914057 **Mass:** 11254 **Score:** 45 **Queries matched:** 1
KMP-11=kinetoplastid membrane protein-11 [Leishmania donovani, LD3, promastigotes, Peptide Kinetopl

☐ Check to include this hit in error tolerant search or archive report

	Query	Observed	Mr(expt)	Mr(calc)	Delta	Miss	Score	Expect	Rank	Peptide
☑	1194	553.38	1104.75	1104.58	0.17	0	45	0.018	1	K.FAELLEQQK.A

Proteins matching the same set of peptides:

gi|1078694 **Mass:** 11255 **Score:** 45 **Queries matched:** 1
kinetoplastid membrane protein-11 - Leishmania donovani
gi|2736232 **Mass:** 11106 **Score:** 45 **Queries matched:** 1
kinetoplastid membrane protein-11 [Leishmania braziliensis]
gi|2736234 **Mass:** 11178 **Score:** 45 **Queries matched:** 1
kinetoplastid membrane protein-11 [Leishmania braziliensis]
gi|2736240 **Mass:** 11151 **Score:** 45 **Queries matched:** 1
kinetoplastid membrane protein-11 [Leishmania braziliensis]
gi|2736242 **Mass:** 11192 **Score:** 45 **Queries matched:** 1
kinetoplastid membrane protein-11 [Leishmania guyanensis]
gi|2736244 **Mass:** 11148 **Score:** 45 **Queries matched:** 1
kinetoplastid membrane protein-11 [Leishmania guyanensis]
gi|2736248 **Mass:** 11192 **Score:** 45 **Queries matched:** 1
kinetoplastid membrane protein-11 [Leishmania guyanensis]
gi|2736250 **Mass:** 11162 **Score:** 45 **Queries matched:** 1
kinetoplastid membrane protein-11 [Leishmania guyanensis]
gi|2736252 **Mass:** 11144 **Score:** 45 **Queries matched:** 1
kinetoplastid membrane protein-11 [Leishmania guyanensis]
gi|3668342 **Mass:** 11220 **Score:** 45 **Queries matched:** 1
kinetoplastid membrane protein-11 [Leishmania panamensis]
gi|3668346 **Mass:** 11118 **Score:** 45 **Queries matched:** 1
kinetoplastid membrane protein-11 [Leishmania panamensis]
gi|3668348 **Mass:** 11144 **Score:** 45 **Queries matched:** 1
kinetoplastid membrane protein-11 [Leishmania panamensis]
gi|4731407 **Mass:** 11117 **Score:** 45 **Queries matched:** 1
kinetoplastid membrane protein-11 [Leishmania braziliensis]
gi|6166388 **Mass:** 9924 **Score:** 45 **Queries matched:** 1

KMP11 [Trypanosoma cruzi]
gi|11225132 **Mass:** 11213 **Score:** 45 **Queries matched:** 1
kinetoplast membrane protein 11 [Leishmania amazonensis]
gi|24286543 **Mass:** 11048 **Score:** 45 **Queries matched:** 1
kinetoplastid membrane protein 11 [Trypanosoma rangeli]
gi|32892041 **Mass:** 11035 **Score:** 45 **Queries matched:** 1
kinetoplastid membrane protein 11 [Trypanosoma rangeli]
gi|32892042 **Mass:** 11003 **Score:** 45 **Queries matched:** 1
kinetoplastid membrane protein 11 [Trypanosoma rangeli]
gi|34922482 **Mass:** 11169 **Score:** 45 **Queries matched:** 1
RecName: Full=Kinetoplastid membrane protein 11B
gi|34922485 **Mass:** 11255 **Score:** 45 **Queries matched:** 1
RecName: Full=Kinetoplastid membrane protein 11C
gi|34922492 **Mass:** 11254 **Score:** 45 **Queries matched:** 1
RecName: Full=Kinetoplastid membrane protein 11; Short=KMP-11; AltName: Full=Lipophosphoglycan-asso
gi|71413455 **Mass:** 10876 **Score:** 45 **Queries matched:** 1
kinetoplastid membrane protein KMP-11 [Trypanosoma cruzi strain CL Brener]
gi|71417146 **Mass:** 11004 **Score:** 45 **Queries matched:** 1
kinetoplastid membrane protein KMP-11 [Trypanosoma cruzi strain CL Brener]
gi|71745968 **Mass:** 11069 **Score:** 45 **Queries matched:** 1
kinetoplastid membrane protein KMP-11 [Trypanosoma brucei TREU927]
gi|72548013 **Mass:** 11227 **Score:** 45 **Queries matched:** 1
kinetoplastid membrane protein-11 [Leishmania major strain Friedlin]
gi|76365105 **Mass:** 11077 **Score:** 45 **Queries matched:** 1
kinetoplastid membrane protein 11 [Trypanosoma rangeli]
gi|118603314 **Mass:** 11030 **Score:** 45 **Queries matched:** 1
kinetoplastid membrane protein 11 [Trypanosoma rangeli]
gi|146101120 **Mass:** 11228 **Score:** 45 **Queries matched:** 1
kinetoplastid membrane protein-11 [Leishmania infantum]
gi|154344763 **Mass:** 11162 **Score:** 45 **Queries matched:** 1
kinetoplastid membrane protein-11 [Leishmania braziliensis MHOM/BR/75/M2904]
gi|3668340 **Mass:** 11211 **Score:** 45 **Queries matched:** 1
kinetoplastid membrane protein-11 [Leishmania panamensis]

---

78. gi|84105385 **Mass:** 68015 **Score:** 44 **Queries matched:** 2
cytosolic heat shock protein 70 [Malawimonas jakobiformis]

Check to include this hit in error tolerant search or archive report

Query	Observed	Mr(expt)	Mr(calc)	Delta	Miss	Score	Expect	Rank	Peptide
1477	650.40	1298.79	1298.60	0.19	0	15	14	8	R.FEELCIDQFR.K
1609	744.47	1486.92	1486.69	0.23	0	44	0.021	1	R.TTPSYVAFTDTER.L

---

79. gi\|53829566 **Mass:** 51780 **Score:** 44 **Queries matched:** 2

HSP70 [Nuclearia simplex]

Check to include this hit in error tolerant search or archive report

Query	Observed	Mr(expt)	Mr(calc)	Delta	Miss	Score	Expect	Rank	Peptide
**89**	**472.04**	**1413.08**	**1413.64**	**-0.56**	**1**	**14**	**59**	**1**	**R.KFTDAEVQSDMK.H + Oxidation (M)**
1609	744.47	1486.92	1486.69	0.23	0	44	0.021	1	R.TTPSYVAFTDTER.L

---

80. gi\|8918240 **Mass:** 71807 **Score:** 44 **Queries matched:** 2

hsp70 [Blastocystis hominis]

Check to include this hit in error tolerant search or archive report

Query	Observed	Mr(expt)	Mr(calc)	Delta	Miss	Score	Expect	Rank	Peptide
1609	744.47	1486.92	1486.69	0.23	0	44	0.021	1	R.TTPSYVAFTDTER.L
729	**881.13**	**1760.25**	**1759.93**	**0.32**	**1**	**0**	**1.4e+03**	**2**	**K.LSKSEVDEVVLVGGSSR.I**

---

81. gi\|71409962 **Mass:** 12839 **Score:** 43 **Queries matched:** 2

calpain-like cysteine peptidase [Trypanosoma cruzi strain CL Brener]

Check to include this hit in error tolerant search or archive report

Query	Observed	Mr(expt)	Mr(calc)	Delta	Miss	Score	Expect	Rank	Peptide
1482	**650.94**	**1299.86**	**1299.62**	**0.23**	**1**	**24**	**2.5**	**1**	**K.KQWAFYNDTK.E**
624	**833.44**	**1664.86**	**1664.80**	**0.05**	**0**	**43**	**0.072**	**1**	**K.YENGQPTFEGPTVVK.C**

Proteins matching the same set of peptides:

gi\|71411006 **Mass:** 12798 **Score:** 43 **Queries matched:** 2

calpain-like cysteine peptidase [Trypanosoma cruzi strain CL Brener]

82. gi|71411263 **Mass:** 15292 **Score:** 43 **Queries matched:** 1
hypothetical protein Tc00.1047053507389.20 [Trypanosoma cruzi strain CL Brener]
☐ Check to include this hit in error tolerant search or archive report

	Query	Observed	Mr(expt)	Mr(calc)	Delta	Miss	Score	Expect	Rank	Peptide
☑	1805	949.54	1897.07	1897.00	0.07	0	43	0.021	1	R.STLPELSLPDTSGGIPVSK.N

Proteins matching the same set of peptides:
gi|71648856 **Mass:** 12540 **Score:** 43 **Queries matched:** 1
hypothetical protein [Trypanosoma cruzi strain CL Brener]

83. gi|71407149 **Mass:** 46733 **Score:** 42 **Queries matched:** 1
hypothetical protein [Trypanosoma cruzi strain CL Brener]
☐ Check to include this hit in error tolerant search or archive report

	Query	Observed	Mr(expt)	Mr(calc)	Delta	Miss	Score	Expect	Rank	Peptide
☑	96	491.34	980.67	980.54	0.13	0	42	0.06	1	K.HDILQSLR.D

84. gi|71404920 **Mass:** 25087 **Score:** 42 **Queries matched:** 3
hypothetical protein [Trypanosoma cruzi strain CL Brener]
☐ Check to include this hit in error tolerant search or archive report

	Query	Observed	Mr(expt)	Mr(calc)	Delta	Miss	Score	Expect	Rank	Peptide
☑	1442	634.40	1266.78	1266.66	0.12	0	42	0.028	1	R.ASVADQEGHILK.E
☑	1746	592.03	1773.08	1772.90	0.18	0	13	27	1	R.VTFQFQVVGGGNHDIR.A
☑	1777	919.04	1836.06	1835.89	0.17	0	7	88	1	K.AIVACLDNTYAHYTPK.L + Carbamidomethyl (C)

85. gi|27734387 **Score:** 42 **Queries matched:** 2
polyubiquitin [Euglypha rotunda]
☐ Check to include this hit in error tolerant search or archive report

Query	Observed	Mr(expt)	Mr(calc)	Delta	Miss	Score	Expect	Rank	Peptide
1734	874.52	1747.02	1746.89	0.13	0	42	0.032	2	K.TITLDVEASDTIENVK.Q

1735	583.36	1747.07	1746.89	0.18	0	(25)	1.5	2	K.TITLDVEASDTIENVK.Q

Proteins matching the same set of peptides:
gi|27734383 **Score:** 42 **Queries matched:** 2

Peptide matches not assigned to protein hits: (no details means no match)

Query	Observed	Mr(expt)	Mr(calc)	Delta	Miss	Score	Expect	Rank	Peptide
2111	905.76	2714.27	2714.05	0.22	1	41	0.029	1	YRDEEEMDDEGGLEEAQEGNDER
301	645.87	1289.73	1289.65	0.08	0	41	0.2	1	DGATFLDDPVIK
1578	476.65	1426.93	1426.85	0.08	1	41	0.042	1	ISLTNELIKQIR
1765	909.04	1816.06	1815.94	0.12	0	41	0.041	1	SVEELQWVDLFVPQK
1507	665.48	1328.94	1328.77	0.18	1	41	0.043	1	VKTLESLLAAER
1646	787.03	1572.04	1571.92	0.12	0	39	0.063	1	AIGSTITLITNSLIR
364	684.44	683.43	683.42	0.01	0	38	0.058	1	EPVVLK
857	470.42	938.83	938.58	0.25	0	38	0.092	1	LVIPGELAK
1930	722.13	2163.36	2163.03	0.33	0	37	0.11	1	LMEQLLDENPHDIAADPSR
711	437.80	873.58	873.53	0.05	0	37	0.16	1	VTFLPLGK
999	500.91	999.81	999.63	0.17	2	36	0.13	1	IKEIEKIK
1250	570.34	1138.66	1138.62	0.04	2	36	0.12	1	KCKMLEFLK
932	487.38	972.74	972.55	0.19	0	36	0.16	1	LEISALEAK
1061	516.42	1030.83	1030.55	0.29	0	35	0.19	1	VLMPLAESR + Oxidation (M)
105	507.03	1518.06	1517.85	0.21	1	35	0.46	1	LFLGGTKPSKEDVK
1807	951.01	1900.01	1899.87	0.14	0	35	0.14	1	GGWAPAGSDVEGLQQSDAR
1225	559.38	1116.74	1116.64	0.10	0	33	0.24	1	TLIDNTLVTK
694	435.27	868.53	868.49	0.04	1	32	0.26	1	RDAGVVPR
559	797.75	796.74	796.47	0.27	0	31	1.1	1	ILLDLNP
1303	583.40	1164.79	1164.57	0.22	0	31	0.35	1	LENELMATTK + Oxidation (M)
1669	537.01	1608.01	1607.86	0.15	0	31	0.38	1	VVVVGSGAVDHTALER
1541	690.45	1378.88	1378.65	0.23	0	31	0.46	1	EAQLAAYSAENGR
155	558.93	1115.84	1115.55	0.29	0	31	1.5	1	QDDILLDER
1900	700.41	2098.21	2098.00	0.22	0	30	0.44	1	GLEHYGLDPTDAEVANEIR
647	421.85	841.68	841.50	0.18	0	30	0.36	1	VASILSPR
1597	730.98	1459.95	1459.74	0.21	1	30	0.49	1	YSVGEIYTKCIK + Carbamidomethyl (C)
1332	592.94	1183.87	1183.67	0.20	1	30	0.54	1	NVELQRQGLK

☑	1199	554.36	1106.71	1106.50	0.21	0	30	0.52	1	MTNEELQAR + Oxidation (M)
☑	537	789.85	1577.69	1577.85	-0.16	1	29	2.4	1	MKTEFLIQLDGVGK
☑	761	449.28	896.54	896.37	0.17	0	28	0.63	1	MTVDEMR + Oxidation (M)
☑	1551	695.41	1388.81	1388.73	0.08	0	28	0.76	1	EISEVVLVGGMTR
☑	1241	564.39	1126.77	1126.56	0.21	0	28	0.76	1	GDISALHGAMR
☑	180	579.06	1156.11	1155.53	0.58	0	28	2.8	1	GAYEFWLDR
☑	2023	802.15	2403.43	2403.18	0.26	0	27	0.92	1	SVVMEVLGTALSVGCTIDGENPR + Carbamidomethyl (C)
☑	1193	1104.65	1103.65	1103.59	0.05	2	27	1.1	1	DNTIKETKR
☑	1161	544.84	1087.66	1087.59	0.07	0	27	1.2	1	SISEILANNK
☑	1304	583.90	1165.78	1165.53	0.25	0	27	0.9	1	WIEAEFDEK
☑	1104	527.39	1052.77	1052.62	0.15	0	27	0.88	1	LVLQPEGLGK
☑	1956	747.51	2239.50	2239.11	0.39	0	27	1.4	1	IQPSIPEDTTTGVGGLGNVDNR
☑	1170	545.85	1089.68	1089.60	0.08	1	26	1.3	1	LSMQVKSAAR
☑	1350	597.38	1192.75	1192.63	0.11	1	26	1.1	1	GLSGYGQVTRR
☑	366	686.48	1370.95	1370.73	0.22	0	26	4.4	1	NIDIEIDELVAK
☑	1254	571.33	1140.64	1140.63	0.01	1	26	1.4	1	FLYSLRNTK
☑	1757	899.11	1796.21	1795.97	0.24	2	26	1.4	1	TTKIKSPEGASPGDLPAK
☑	1870	1011.56	2021.10	2020.96	0.14	1	26	1.2	1	NGREAATAAAASSAGVCTTGAK + Carbamidomethyl (C)
☑	1322	590.39	1178.78	1178.58	0.20	1	26	1.3	1	CPDAFEITKR
☑	1076	519.34	1036.67	1036.54	0.13	0	25	1.3	1	YLGDITIDK
☑	1605	738.46	1474.90	1474.71	0.19	1	25	1.5	1	QELEEMRAVQDK
☑	913	484.35	966.69	966.50	0.19	1	25	1.3	1	LKDYSDVK
☑	163	565.23	1128.45	1128.60	-0.15	0	25	4.3	1	LLQELEAGEK
☑	1397	611.44	1220.87	1220.59	0.28	0	25	1.7	1	ENHISFLSMK + Oxidation (M)
☑	1695	836.37	1670.73	1670.75	-0.02	0	24	1.9	1	NEDQQTGDAVGDAVPR
☑	1449	638.43	1274.84	1274.71	0.13	1	24	2	1	DVALAYLRAGAR
☑	1489	654.42	1306.82	1306.67	0.15	2	24	2.1	1	KIMEAGSWKNK + Oxidation (M)
☑	1315	588.36	1174.70	1174.63	0.07	1	24	2.4	1	DVPSQKNFIK
☑	1633	772.43	1542.85	1542.75	0.09	1	23	2.1	1	SAASYEARHGPIER
☑	1233	561.40	1120.79	1120.67	0.12	1	23	2.4	1	YLLCKIIAK + Carbamidomethyl (C)
☑	1102	526.38	1050.76	1050.57	0.18	0	23	2.4	1	VVAFLYDPK
☑	528	785.59	784.58	784.48	0.10	0	23	8.8	1	VLAVIDR
☑	1500	658.91	1315.81	1315.73	0.08	1	23	2.6	1	DVFGIHYKIPK
☑	1652	793.15	1584.29	1584.88	-0.58	0	23	4.7	1	GLPEEYAPLIASVVK

☑	1376	603.91	1205.80	1205.65	0.15	1	23	3	1	ARLSIFENTR
☑	1527	679.40	1356.78	1356.72	0.06	1	22	3	1	NLDDEIRILEK
☑	1851	994.54	1987.06	1987.06	0.00	1	22	2.5	1	SSVNELSLQRGDVIEITK
☑	1253	571.32	1140.62	1140.63	-0.01	1	22	3.2	1	TPRVVEDVAR
☑	1339	593.94	1185.87	1185.62	0.25	0	22	3	1	EIDVIEEIAR
☑	1542	691.42	1380.82	1380.72	0.09	2	22	2.7	1	KNAEYKIDSVSK
☑	977	496.84	991.66	991.56	0.11	1	22	4.5	1	YSGVAALRR
☑	1412	412.31	1233.92	1233.61	0.30	1	22	3.1	1	SRTGMDEVALR
☑	469	751.58	1501.15	1500.72	0.42	0	22	12	1	VNLPCVNADGTVDK + Carbamidomethyl (C)
☑	1484	651.42	1300.82	1300.70	0.12	1	22	4.4	1	DQINNKTIEVK
☑	1465	645.31	1288.61	1288.61	-0.00	0	22	3.2	1	EVSVVNDAEAEK
☑	1782	920.06	1838.11	1837.78	0.34	1	21	3.1	1	AEEAGEACPISCSACKR + 2 Carbamidomethyl (C)
☑	1741	881.51	1761.01	1760.86	0.16	0	21	3.4	1	EVPGTENFEVVETGVR
☑	989	499.42	996.82	996.62	0.19	0	21	3.4	1	LVLVVEPTK
☑	1674	540.46	1618.35	1617.85	0.50	2	21	6.6	1	PIGYPLVKAMDKDR + Oxidation (M)
☑	1675	811.98	1621.95	1621.82	0.13	1	21	3.6	1	NTKSLLETMIEAEK + Oxidation (M)
☑	580	406.29	810.57	810.50	0.08	0	21	3.1	1	LTLSHLK
☑	277	629.36	628.35	628.33	0.03	0	21	3.8	1	GVGGPSR
☑	1855	665.77	1994.28	1994.01	0.27	1	21	4	1	EQAEGDSFIPGSKTIFLR
☑	1235	561.87	1121.72	1121.55	0.17	0	21	4.1	1	ELQNQTYAR
☑	1749	596.02	1785.05	1784.89	0.16	0	21	3.9	1	LQQESKPEQLDSVER
☑	980	497.34	992.66	992.52	0.14	1	21	3.9	1	YERWALR
☑	1934	1086.11	2170.20	2170.09	0.11	2	21	3.6	1	ACRGMQYVIDHEVPALRR + Carbamidomethyl (C)
☑	1064	517.35	1032.69	1032.52	0.17	1	20	5	1	DVGNAKNSTK
☑	1364	601.40	1200.78	1200.61	0.17	0	20	5.1	1	NVTADADAVVAR
☑	1463	644.99	1287.97	1287.64	0.33	1	20	5.6	1	DESREVNIAAGK
☑	1499	658.88	1315.76	1315.66	0.10	0	20	4.9	1	AYFADIAINYR
☑	1654	793.98	1585.94	1585.81	0.14	1	20	5.2	1	AREQQQAEESLGIK
☑	1455	640.42	1278.82	1278.70	0.12	1	20	5.2	1	VLINGHDRDLK
☑	1691	556.50	1666.48	1666.71	-0.23	1	20	5.6	1	FMGCGPSSSTSSGYRK + Oxidation (M)
☑	1354	597.85	1193.69	1193.56	0.13	0	20	5.1	1	ICNFQAGTQR + Carbamidomethyl (C)
☑	42	300.46	598.91	598.34	0.57	0	20	9.3	1	DVLPR
☑	1534	683.41	1364.81	1364.62	0.19	0	20	4.8	1	DFLMLPCEGANR
☑	1553	695.94	1389.86	1389.72	0.14	1	20	6	1	KSIEALMEENVK

☑	320	327.81	653.61	653.37	0.24	1	20	3	1	SRVHR
☑	1665	803.43	1604.85	1604.86	-0.01	1	20	5.8	1	IICTNVINLEKSMK
☑	2009	1184.27	2366.53	2366.13	0.40	1	20	5.9	1	LCEELKSMTNEMINQLNNVK + Oxidation (M)
☑	414	717.89	716.88	716.37	0.51	0	20	15	1	AVVEDGK
☑	1461	643.95	1285.88	1285.67	0.21	1	19	5.7	1	RLGPSWATWGR
☑	19	269.25	804.74	804.47	0.27	2	19	7.7	1	AARGFRK
☑	597	819.27	818.26	818.43	-0.17	0	19	21	1	VVQVSCGK
☑	282	633.95	1265.88	1265.67	0.20	1	19	20	1	TQYSKSGLLNR
☑	772	903.23	902.22	902.49	-0.27	1	19	25	1	NSKATNLR
☑	1801	943.04	1884.06	1883.88	0.18	2	19	5.9	1	SNKSSSDQCYPRAAVGSK
☑	117	515.43	1028.85	1028.61	0.24	1	19	21	1	LNVSITRAR
☑	1549	694.37	1386.72	1386.84	-0.12	2	19	7.2	1	MRLIRSLILEK + Oxidation (M)
☑	1428	627.89	1253.77	1253.53	0.24	0	19	6.2	1	DSASGISGSACGSR
☑	1857	999.06	1996.10	1996.10	0.00	1	19	8.7	1	GVAKALFDLDIQIEHSIK
☑	1655	529.66	1585.95	1585.84	0.11	2	18	7.5	1	LNASRGAAGGSVAGRSR
☑	1478	650.41	1298.81	1298.72	0.09	1	18	6.8	1	QLEIEAKALER
☑	1208	556.40	1110.80	1110.63	0.17	1	18	8.7	1	ISLASRHATR
☑	1872	1011.99	2021.97	2022.03	-0.07	2	18	6.7	1	AFSFAFRSASSLALRAFSS
☑	213	600.15	1198.28	1198.52	-0.25	1	18	27	1	FMWRDPQGY
☑	334	666.33	665.32	665.31	0.02	0	18	6.4	1	DMIGSK + Oxidation (M)
☑	1795	937.40	1872.79	1872.97	-0.18	1	18	6.7	1	GLPAESADRIGEYVQLR
☑	1509	665.92	1329.83	1329.76	0.07	2	18	8.7	1	NKLLNSVKMQR
☑	1538	687.40	1372.78	1372.63	0.15	1	18	7.5	1	KAENGTSSSAEHR
☑	1396	610.92	1219.82	1219.70	0.13	0	18	7.9	1	VFPGAIIQFTK
☑	20	283.12	846.34	846.41	-0.08	1	18	14	1	GPRGASMR + Oxidation (M)
☑	50	371.11	1110.30	1110.58	-0.28	1	18	15	1	SQAHIDGGAKK
☑	87	460.58	1378.72	1378.66	0.06	0	18	19	1	CVMVSEPTWVTK
☑	1581	717.54	1433.06	1432.70	0.36	2	18	10	1	HEARRLMDSFR + Oxidation (M)
☑	799	913.26	912.25	912.46	-0.21	0	18	29	1	FHPPYPR
☑	935	974.81	973.80	974.40	-0.60	0	18	27	1	DFFPMMR + 2 Oxidation (M)
☑	190	586.39	1170.77	1170.49	0.28	0	18	23	1	DFDTVCTEGK + Carbamidomethyl (C)
☑	1288	579.86	1157.70	1157.62	0.08	2	18	8.9	1	RKDVEDALGR
☑	211	599.80	1796.37	1796.91	-0.54	1	18	29	1	VPCFDIRQIGMFLSR + Oxidation (M)
☑	1958	1126.16	2250.30	2250.02	0.28	1	18	8	1	NEWMSSGQGLKVVDEDGEVR + Oxidation (M)

☑	328	660.89	659.89	659.35	0.54	0	18	26	1	GEEVVK
☑	124	519.25	1036.48	1036.57	-0.09	0	18	20	1	ANIFGFLQK
☑	1453	639.42	1276.83	1276.62	0.21	0	17	9.1	1	AGGQVLQGGGDYR
☑	1298	582.84	1163.67	1163.59	0.08	0	17	11	1	MGSELVQAVSK + Oxidation (M)
☑	1166	1090.01	1089.00	1089.55	-0.55	2	17	34	1	MRNNDLRR + Oxidation (M)
☑	581	811.76	1621.50	1621.73	-0.24	0	17	25	1	AQASTAEEPHAEEPR
☑	588	814.98	1627.95	1627.83	0.12	1	17	35	1	EPKLETEANEEVLK
☑	612	828.20	1654.39	1654.73	-0.33	0	17	34	1	DNDPMPNELDPINR + Oxidation (M)
☑	745	889.03	2664.07	2664.30	-0.22	1	17	41	1	RATAAAPTAVDGAIDDTTVGAAAYNSSK
☑	1967	1135.43	2268.85	2269.18	-0.33	2	17	9.4	1	LFLFKLETKSWVECGVGDAK
☑	1114	1060.64	1059.63	1059.54	0.10	1	17	10	1	CIKPNDDKK
☑	338	666.95	1331.89	1331.76	0.13	1	17	26	1	YNTPATVGLLRK
☑	416	718.83	717.82	718.31	-0.50	0	17	21	1	DGVDDAK
☑	1204	555.32	1108.63	1108.61	0.01	1	17	8.3	1	LSADTFSKLK
☑	1737	876.44	1750.86	1750.83	0.03	1	17	8.6	1	AVQEDNVERFEMLR + Oxidation (M)
☑	1359	599.90	1197.78	1197.69	0.09	0	17	8.9	1	QPFSLPQLLR
☑	1369	602.42	1202.83	1202.62	0.21	2	17	11	1	RDEMKQQIR
☑	1606	738.47	1474.93	1474.81	0.13	2	17	9.6	1	ELLKNMTELKEK
☑	553	796.37	795.36	795.44	-0.07	1	17	7.9	1	HEKINR
☑	1441	633.92	1265.82	1265.62	0.20	0	17	9.4	1	CIINCEISMIK
☑	931	973.28	1944.56	1944.07	0.48	2	17	40	1	MRDYSQVPSVLKRPLR
☑	1122	533.86	1065.71	1065.56	0.15	1	17	9.1	1	LYNMKEIR
☑	1610	744.91	1487.81	1487.82	-0.01	1	17	9.8	1	AKDVSEVVLVGGSTK
☑	122	519.17	1036.33	1036.57	-0.24	0	17	25	1	ANIFGFLQK
☑	1196	1106.12	1105.12	1105.53	-0.42	0	17	38	1	EVVDLMEQK + Oxidation (M)
☑	1798	939.65	1877.28	1876.98	0.30	0	17	12	1	MFLPSQILEVICNVAK + Carbamidomethyl (C); Oxidation (M)
☑	1480	650.83	1299.64	1299.58	0.06	1	17	14	1	KVEDMQTDYR + Oxidation (M)
☑	295	642.60	1924.78	1925.09	-0.32	1	17	35	1	DKSPTQVIAITLSGAVAVR
☑	342	672.18	2013.53	2014.05	-0.52	0	17	41	1	LTGYQTNTTPIILSHAER
☑	361	683.35	2047.03	2047.06	-0.02	1	17	25	1	RIEAEGDFELIASALTGQK
☑	370	689.95	2066.84	2067.03	-0.19	1	17	48	1	EFVEDTLQSFEVLEARR
☑	382	698.03	2091.07	2090.93	0.14	1	17	28	1	IAFGNSFKYAGCYSTFCR + Carbamidomethyl (C)
☑	1506	665.41	1328.81	1328.72	0.09	2	17	10	1	LDGNGALKRTER
☑	1878	1016.09	2030.16	2030.12	0.04	2	17	11	1	RHLVLPVVSCPRAELER + Carbamidomethyl (C)

☑	160	563.04	1124.06	1123.67	0.39	0	17	35	1	LSLSSILVHR
☑	156	559.66	1117.32	1117.70	-0.38	2	17	31	1	VINKKFITR
☑	270	624.81	1247.61	1247.61	-0.01	0	17	42	1	IDAEQEVAAFR
☑	197	591.36	1771.07	1770.92	0.16	1	17	29	1	DEHQGLLKYELLGEK
☑	1381	604.91	1207.80	1207.58	0.22	0	16	11	1	GDATLVQYADR
☑	1539	689.44	1376.87	1376.73	0.15	2	16	11	1	KARTTLSETNEK
☑	1991	770.42	2308.25	2308.20	0.05	2	16	10	1	IIEMVGMPSTQILDKGSKTTK + 2 Oxidation (M)
☑	1604	738.43	1474.84	1474.68	0.16	0	16	11	1	IFDEEHVQAEMK
☑	454	741.92	1481.83	1481.67	0.16	1	16	41	1	AMRSGTAADGQDFR
☑	610	827.32	2478.95	2479.17	-0.22	2	16	34	1	YTDVPIMTDPARHDYMNIRR + Oxidation (M)
☑	1256	571.40	1140.80	1140.48	0.32	0	16	14	1	DFDTVCAEGK + Carbamidomethyl (C)
☑	207	598.92	1195.82	1195.53	0.28	0	16	31	1	CTQDMATIASR
☑	1982	762.49	2284.46	2284.22	0.24	2	16	14	1	EYSFRREEIISNIFQLLK
☑	330	663.36	1987.06	1987.09	-0.03	0	16	42	1	QGVLPNVVSYNTLLCLVR
☑	475	754.10	2259.27	2259.11	0.16	0	16	43	1	QCAYVATGMILIQAQNTHPK + Carbamidomethyl (C); Oxidation (M)
☑	1789	929.09	1856.17	1855.87	0.30	0	16	12	1	MQETEEQSLQLHEVR
☑	1974	761.59	2281.76	2282.05	-0.28	0	16	15	1	HDEEPTVTQQFDVEPENLR
☑	149	548.55	1642.62	1642.81	-0.18	2	16	42	1	DKIGNYAESMKMIK + Oxidation (M)
☑	1466	645.34	1288.66	1288.60	0.06	0	16	13	1	MNEYIDFITK + Oxidation (M)
☑	620	833.15	1664.28	1663.81	0.47	0	16	60	1	TMEDAATVVGEIFGPK
☑	147	547.83	1640.48	1640.94	-0.46	2	16	48	1	LARKEQCIALGAQLK
☑	1624	755.92	1509.82	1509.94	-0.12	0	15	14	1	EIIILITNLVIEK
☑	1994	776.47	2326.38	2326.18	0.19	2	15	14	1	GEEEMLIVGKQHSDEVAAIKK + Oxidation (M)
☑	268	623.54	1245.07	1245.64	-0.57	0	15	51	1	ICQISDEALVR
☑	1634	773.49	1544.97	1544.84	0.12	0	15	14	1	IFTPLLNEIETQK
☑	1545	692.42	1382.83	1382.82	0.00	1	15	14	1	ISRPLLNSLKDK
☑	2093	882.16	2643.46	2643.20	0.26	2	15	11	1	VDNNEEHKDNKTTNTSESKPDNK
☑	182	580.55	1159.09	1158.61	0.48	1	15	52	1	ANSNKVVASNR
☑	2028	807.85	2420.53	2421.07	-0.54	1	15	16	1	MLMDECTELINQCSEVKTFR + 2 Oxidation (M)
☑	177	576.90	1727.67	1727.90	-0.24	1	15	50	1	HKLGGMMILCIPPYR
☑	1897	1048.64	2095.27	2095.05	0.22	0	15	14	1	TFVSTLDEICELSNQVLK + Carbamidomethyl (C)
☑	1868	1011.10	2020.18	2019.92	0.26	0	15	14	1	THAAPSSGGGYSDDVLDMLK
☑	2047	826.29	2475.86	2476.25	-0.39	1	15	16	1	SPRDPDLLAVGGLDDSIYFVEAK
☑	864	943.13	1884.25	1884.04	0.21	1	15	49	1	AEIINAMVNVLLRSWR

☑	231	608.36	1214.70	1214.68	0.02	1	15	46	1	KLCGDALNILR
☑	1775	613.02	1836.04	1835.93	0.11	0	15	15	1	ICENFSQIIEEGLTLK
☑	732	882.63	2644.88	2645.14	-0.26	2	15	56	1	AECVCVCVCVCVCVCVRRR + 6 Carbamidomethyl (C)
☑	1091	523.38	1044.74	1044.64	0.10	2	15	17	1	SLSLRKVSR
☑	1528	679.48	1356.94	1356.72	0.21	1	15	18	1	NLDDEIRILEK
☑	1636	775.33	1548.65	1548.85	-0.21	1	15	16	1	SGVLISIFRTAEEK
☑	1657	794.48	1586.95	1586.79	0.16	1	15	19	1	LNALSNQEKANEEK
☑	1563	703.96	1405.90	1405.60	0.30	1	15	19	1	EEFKDDFDFSK
☑	1479	650.42	1298.82	1298.61	0.21	1	15	16	1	MMARSSSSSISR
☑	1778	613.07	1836.17	1836.00	0.17	0	15	17	1	SATNVYGILDLPSVFIK
☑	1887	687.40	2059.19	2059.05	0.14	2	15	16	1	TRTLGTNKDLTEEDNKPK
☑	291	641.16	640.16	640.25	-0.09	1	15	8.2	1	CCRIM + Oxidation (M)
☑	902	963.48	962.47	962.49	-0.02	0	15	50	1	MSSNNLVAK
☑	2063	840.87	2519.59	2519.00	0.59	0	15	20	1	CNACGTCQDLWSCLVCGWVGCGK + 2 Carbamidomethyl (C)
☑	1959	751.14	2250.40	2250.02	0.37	0	15	17	1	GDLYELVEDGELHEQYWR
☑	1346	595.89	1189.76	1189.67	0.09	1	14	19	1	NLKDVITIMK + Oxidation (M)
☑	804	915.22	1828.42	1828.84	-0.42	1	14	67	1	EFPNEMAKHSPVNDSK
☑	208	599.13	1794.38	1793.92	0.46	1	14	60	1	CDVVLQGAICVKAFSK + 2 Carbamidomethyl (C)
☑	1977	761.82	2282.43	2282.05	0.37	1	14	16	1	NMERITLCCGGSAVCALDELK + Carbamidomethyl (C)
☑	267	623.47	1867.39	1866.94	0.45	0	14	66	1	RPIPPYTPQQGDANWK
☑	1639	778.49	1554.96	1554.88	0.08	2	14	18	1	ELSEVNRQIRALK
☑	238	612.02	1833.05	1832.96	0.09	2	14	53	1	KYLGLKCPHAQFTEAK
☑	1437	632.43	1262.85	1262.60	0.25	0	14	20	1	NINPANQSSYR
☑	1677	542.39	1624.15	1623.80	0.35	1	14	21	1	RATTGEHHSYRPGR
☑	1797	626.76	1877.27	1876.95	0.32	1	14	21	1	LTDLKDMIEMLPDTVK + Oxidation (M)
☑	1218	558.37	1114.73	1114.63	0.10	1	14	18	1	LEESIRLQK
☑	1723	859.03	1716.05	1715.86	0.19	0	14	19	1	LLLEAETELSDAQER
☑	1980	762.15	2283.44	2283.17	0.27	1	14	20	1	NYSKELPEINITHQEDILK
☑	1012	1006.05	3015.13	3015.48	-0.34	1	14	76	1	MEVFDPFTSQLGMIPSRILTTMDTASK
☑	1510	666.32	1330.62	1330.61	0.01	0	14	20	1	FDLPDMLSSYK + Oxidation (M)
☑	681	863.43	2587.26	2587.22	0.04	1	14	58	1	GHYMNVTIATIENMYERAEFAK
☑	2014	793.15	2376.42	2376.28	0.14	2	14	17	1	LYLSLADIEEGKIIKVENDSK
☑	1612	747.45	1492.88	1492.67	0.21	1	14	19	1	MFGSGMKTMTGSVK + 2 Oxidation (M)
☑	749	890.48	889.47	889.40	0.07	0	14	26	1	HCCVIMK + Carbamidomethyl (C)

☑	232	609.38	608.38	608.23	0.15	0	14	15	1	FPDAC + Carbamidomethyl (C)
☑	81	447.67	1339.98	1339.72	0.26	2	14	42	1	ARLRGEVADPEK
☑	611	827.51	826.50	826.28	0.22	0	14	19	1	CAECNCK + Carbamidomethyl (C)
☑	385	699.87	1397.73	1397.77	-0.05	2	14	79	1	DLLRNVRDEIR
☑	1535	683.51	1365.01	1364.68	0.33	1	14	22	1	MMGLGTELKQNK + Oxidation (M)
☑	104	505.30	1512.87	1512.79	0.08	1	14	46	1	LSTPPAVADGRHHR
☑	788	908.96	907.95	907.50	0.45	2	14	85	1	RKSFSQR
☑	1121	533.43	1064.86	1064.73	0.12	1	14	22	1	LPAVKLLLAK
☑	1440	633.40	1264.79	1264.68	0.12	1	14	24	1	TIFASSENLRK
☑	101	500.44	998.87	999.46	-0.59	0	13	64	1	NDMHAQLR + Oxidation (M)
☑	1205	555.35	1108.69	1108.54	0.15	1	13	20	1	KEVFSEGGEK
☑	1883	679.96	2036.85	2036.91	-0.06	1	13	21	1	DTTVQSACCAREDAAPLMR
☑	613	414.79	827.56	827.45	0.11	0	13	21	1	ESGAVLPR
☑	344	672.66	1343.31	1342.73	0.58	1	13	79	1	EKGCKPVPLSTK + Carbamidomethyl (C)
☑	236	611.93	1221.84	1221.78	0.06	1	13	56	1	LVVAKLDVPLR
☑	1095	1048.71	1047.71	1047.50	0.20	0	13	87	1	ETLNDAVMR
☑	2078	855.84	2564.50	2564.37	0.12	2	13	23	1	RRPGLGPIVLTSGEAERETPALMV + Oxidation (M)
☑	590	815.38	814.37	814.48	-0.11	1	13	70	1	ALRASAAR
☑	242	614.86	1841.56	1842.06	-0.50	2	13	95	1	LDIEPVKFAIVTKNGAK
☑	1289	579.88	1157.75	1157.57	0.18	1	13	25	1	QRNYHPTSR
☑	529	785.74	784.73	784.47	0.26	2	13	68	1	RTKAGPR
☑	239	612.80	1835.39	1835.96	-0.57	2	13	83	1	SKDEFVNDVIKSISQK
☑	1758	599.75	1796.23	1796.82	-0.59	1	13	26	1	TRSELDAAQGMCMALK + Carbamidomethyl (C); Oxidation (M)
☑	1744	883.98	1765.95	1766.01	-0.06	2	13	22	1	FTFAKLSKLQALGNTK
☑	335	666.65	1996.93	1997.06	-0.12	0	13	81	1	CFWALHILEPASTGALLR
☑	336	666.82	1331.62	1331.77	-0.15	0	13	96	1	IELILVYVENK
☑	393	703.96	2108.85	2109.08	-0.23	1	13	1.1e+02	1	TPTPTTAAATATAAVSGEHAKR
☑	440	733.40	2197.17	2197.14	0.02	2	13	84	1	IKAYNKLHASQPPAQSMASR
☑	674	860.90	2579.69	2579.22	0.47	2	13	1.1e+02	1	GSQHRYATTQATGDGAYRNEAAVR
☑	1133	1071.72	2141.43	2140.99	0.44	2	13	85	1	VGVKWLADSCMQCELCRK + Carbamidomethyl (C); Oxidation (M)
☑	1272	1150.74	3449.19	3448.84	0.35	2	13	76	1	AERSFSYKPLPIEPEIVEIGHGEIWSKIPK
☑	2036	814.34	2440.01	2440.08	-0.07	2	13	20	1	DMEKMLEKVDCSQVHEQFR + Carbamidomethyl (C); 2 Oxidation (M)
☑	299	644.36	643.35	643.30	0.05	0	13	75	1	GNNSPR
☑	1084	521.39	1040.76	1040.54	0.22	0	13	21	1	PAPTYTHVR

☑	127	523.39	1567.14	1566.79	0.35	0	13	59	1	VCGSLQFMATELLR
☑	1285	578.95	1155.88	1155.64	0.24	1	13	24	1	QRNALDAQLK
☑	224	604.72	1811.14	1810.90	0.24	1	13	95	1	HASMALIHELMQRMK + Oxidation (M)
☑	626	834.43	833.42	833.50	-0.08	1	13	72	1	ALEKFVK
☑	740	886.48	2656.42	2656.30	0.12	2	13	70	1	RASQSATLHVAPPSCVKNNMQVMR + 2 Oxidation (M)
☑	650	423.27	844.52	844.50	0.02	1	13	34	1	VALDKTAK
☑	1941	1094.09	2186.16	2186.10	0.06	2	13	23	1	DILHNYKPTQKKEENDSK
☑	1447	636.91	1271.81	1271.71	0.10	2	13	27	1	AVAGSGGKRGTVGR
☑	1689	830.93	1659.85	1659.71	0.14	1	13	26	1	CRADVWFMPCFR + 2 Carbamidomethyl (C); Oxidation (M)
☑	398	706.69	705.68	706.23	-0.55	0	13	27	1	GHCCCK + Carbamidomethyl (C)
☑	1521	674.97	1347.92	1347.79	0.13	2	13	28	1	GKNTIKVQFSVK
☑	1265	574.01	1146.02	1146.51	-0.49	0	13	54	1	QCIGCWPAR + 2 Carbamidomethyl (C)
☑	1497	658.58	1315.15	1315.70	-0.55	2	13	52	1	SGEKAHPHKTPK
☑	34	297.16	888.47	888.51	-0.04	0	13	60	1	EYPVLIR
☑	565	802.18	1602.34	1601.82	0.53	0	13	1.2e+02	1	SQTTLTITWTASHR
☑	1176	1092.64	1091.63	1091.59	0.04	0	12	76	1	FLISEENIK
☑	1590	724.56	1447.11	1447.68	-0.57	0	12	42	1	CTHSISAFEQTPK
☑	1171	545.86	1089.70	1089.56	0.14	1	12	30	1	AKVCGWIEGK
☑	1090	1045.65	1044.64	1044.61	0.03	1	12	89	1	KGFAVPASLR
☑	544	792.57	2374.68	2375.18	-0.50	0	12	1.1e+02	1	LDFHCHLNGSVSASLLAHLER + Carbamidomethyl (C)
☑	1490	436.65	1306.92	1306.68	0.24	0	12	32	1	FVVSSEEELLR
☑	308	650.46	1948.35	1947.90	0.45	2	12	1e+02	1	LPKRCIYDCESLYSCR
☑	1884	682.25	2043.74	2043.98	-0.24	1	12	26	1	EGCINVSNFFSLKMASPK + Carbamidomethyl (C); Oxidation (M)
☑	2057	836.60	2506.77	2506.26	0.51	1	12	44	1	AVDGDTSCVVNQTLTKIALNMWK
☑	1488	653.01	1304.01	1303.80	0.21	0	12	40	1	ILHALMLVGPIK
☑	1004	1003.72	3008.15	3007.60	0.55	2	12	1.2e+02	1	LPIKANQYKTIGMIAGGTGITPMYQVAR + Oxidation (M)
☑	871	473.87	945.72	945.47	0.25	0	12	43	1	AFDPAAQAR
☑	1299	1165.07	3492.19	3492.49	-0.31	2	12	1.2e+02	1	NMCPSLEYDSMSCVTLTQRICQEHTDCKK + 2 Carbamidomethyl (C); Oxidation (M)
☑	269	624.79	1247.57	1247.65	-0.08	2	12	1.1e+02	1	LIEGRMKEEK + Oxidation (M)
☑	1555	697.51	1393.01	1392.67	0.34	0	12	31	1	EMSLLNEAMAIR + Oxidation (M)
☑	1498	658.86	1315.70	1315.71	-0.01	1	12	31	1	LIQNTIERSDK
☑	1336	1186.36	1185.35	1185.67	-0.31	0	12	1e+02	1	FVFTAVLPHR
☑	1366	601.90	1201.79	1201.68	0.11	1	12	37	1	NNARSSIILSK
☑	286	637.46	636.45	636.27	0.18	0	12	92	1	MAEMR

☑	1704	844.48	1686.94	1686.93	0.01	2	12	28	1	DIKKQIQTFPDINK
☑	1439	633.38	1264.74	1264.64	0.10	2	12	34	1	FSDLNEKERK
☑	1443	1270.15	1269.14	1268.81	0.33	2	12	1.2e+02	1	KILLKGLLDEK
☑	83	449.69	897.37	897.54	-0.17	1	12	76	1	GQPKVLTR
☑	1333	593.08	1184.14	1184.63	-0.48	2	12	57	1	TPREDGGVARK
☑	1867	1011.06	2020.10	2020.07	0.03	2	12	29	1	DKYVLMTLAVYTKFQGK + Oxidation (M)
☑	1950	741.98	2222.92	2222.95	-0.03	2	12	28	1	EEDNQGDKETKESETNSQR
☑	1198	1106.97	1105.96	1105.59	0.37	1	12	97	1	GLLAMSTRNK + Oxidation (M)
☑	1511	667.86	1333.71	1333.63	0.08	1	12	34	1	ELNEDKAAMAAR + Oxidation (M)
☑	1858	999.54	1997.07	1996.93	0.13	1	12	29	1	GVFSTCSDICSPSPSLRK + 2 Carbamidomethyl (C)
☑	1365	601.87	1201.73	1201.64	0.09	1	12	38	1	KSENAASLVQR
☑	1712	850.06	1698.10	1697.77	0.34	1	12	31	1	KMLDYAANSSEDAQR
☑	271	625.33	1248.65	1248.68	-0.03	1	12	79	1	TGNQKSLIFNK
☑	448	739.63	1477.24	1477.69	-0.45	0	12	1.2e+02	1	IMLDGPQQACTTK + Carbamidomethyl (C); Oxidation (M)
☑	1666	803.45	1604.89	1604.79	0.10	1	12	36	1	EVNTLFFFKDSMK
☑	1546	692.72	1383.43	1383.74	-0.31	1	12	33	1	AIIERPMRNER
☑	819	922.20	2763.58	2763.12	0.46	1	12	1.1e+02	1	ARCFPDGMDATEPDNTFWMEWR + Carbamidomethyl (C); 2 Oxidation (M)
☑	1323	1179.87	2357.72	2358.26	-0.54	2	12	99	1	AWPLWPFIAAVNCKKSASIEK
☑	522	782.74	2345.20	2345.34	-0.13	2	12	85	1	TLLMLACQRGQLSLVMALLKK + Oxidation (M)
☑	1203	1109.59	1108.58	1108.55	0.03	2	12	80	1	EEGEKFSKR
☑	62	427.40	852.79	852.37	0.42	0	12	94	1	SHSHEEK
☑	204	596.56	1191.10	1190.63	0.47	1	12	1.1e+02	1	LVNGIKDSSMK
☑	1671	807.92	1613.84	1613.81	0.02	2	12	34	1	DEVLKDGQERAAQR
☑	132	528.38	1054.75	1054.54	0.21	1	12	67	1	DRFTEIFK
☑	596	817.30	816.29	816.51	-0.22	1	12	1.1e+02	1	LLAKSSAK
☑	826	924.86	923.85	923.42	0.43	0	12	1e+02	1	SVVDCVMR + Oxidation (M)
☑	1591	724.93	1447.84	1447.74	0.10	0	12	34	1	ISDIICLQMDLK + Carbamidomethyl (C)
☑	1044	1024.15	1023.14	1023.52	-0.38	2	12	97	1	GRIKEMMK + 2 Oxidation (M)
☑	1245	567.37	1132.72	1132.62	0.10	1	12	38	1	AFRVADDVLK
☑	1195	1106.06	2210.10	2210.06	0.04	2	12	1.4e+02	1	GIMVGSGQKDEYVGDAAMARR
☑	1392	607.88	1213.75	1213.71	0.04	2	12	33	1	VLKSKDQAAVR
☑	1588	1446.15	2890.30	2890.43	-0.13	2	12	1.1e+02	1	FYIPTKCLHIDCIHRELEDIYR + 2 Carbamidomethyl (C)
☑	882	955.21	954.20	954.39	-0.19	0	12	28	1	SSSMMAGER
☑	130	524.81	1571.40	1571.84	-0.44	2	11	92	1	KRQCQTPLMVAAAR

☑	498	762.96	761.95	761.39	0.57	0	11	1.5e+02	1	VSAAVCR + Carbamidomethyl (C)
☑	918	968.86	2903.55	2903.47	0.08	2	11	96	1	AFASAANACHEAHQPQRSRASVVVQLR
☑	1544	692.41	1382.80	1382.68	0.12	0	11	34	1	EAAYSLLSCALDK
☑	1494	656.47	1310.92	1310.78	0.14	2	11	31	1	DVRSILPTVRR
☑	1853	997.59	1993.17	1992.85	0.32	1	11	31	1	MNAMSAADAGTSYKCPYR + Carbamidomethyl (C)
☑	234	609.79	1826.36	1826.87	-0.51	0	11	1.4e+02	1	LIELMNNPENCDPLR + Carbamidomethyl (C)
☑	248	617.62	1233.22	1232.62	0.59	1	11	1.4e+02	1	AEIEDKNSSIK
☑	188	585.41	1168.81	1168.58	0.23	1	11	85	1	SFIKAEDMTK
☑	1914	1070.64	2139.26	2139.03	0.23	2	11	36	1	TRVMEQDDAYEQLRELK + Oxidation (M)
☑	562	799.43	2395.27	2395.21	0.06	0	11	1e+02	1	CLLFILEIIVMACVAQAETCR + Carbamidomethyl (C)
☑	1717	851.59	1701.16	1700.81	0.35	0	11	40	1	ELLAALMDAYYAADR + Oxidation (M)
☑	1790	621.15	1860.43	1860.07	0.36	0	11	68	1	VMATGQLLHIPVGAGVLGK
☑	312	651.33	1300.64	1300.64	-0.01	1	11	1.1e+02	1	EQASTLEKHMK
☑	322	656.08	1310.14	1310.60	-0.46	0	11	1.2e+02	1	DAMSAVAQPFMK + Oxidation (M)
☑	526	785.11	1568.20	1568.78	-0.58	1	11	1.2e+02	1	LSCLEFFRIDNR + Carbamidomethyl (C)
☑	651	845.95	2534.83	2535.33	-0.50	1	11	1.8e+02	1	AQHGMCLLRPINIDAVLSRCLR + Carbamidomethyl (C)
☑	838	465.86	929.71	929.48	0.23	0	11	50	1	VDLNVENK
☑	92	483.11	964.20	964.44	-0.25	0	11	1.1e+02	1	GCTYLHSK + Carbamidomethyl (C)
☑	219	603.76	1205.51	1205.65	-0.14	2	11	1.8e+02	1	IDLAGDKYRR
☑	1576	713.42	1424.82	1424.72	0.10	1	11	38	1	ISRYPYIPMNR + Oxidation (M)
☑	2120	914.87	2741.60	2741.37	0.23	2	11	31	1	TAADVERNGAVGGCSPEVCVAARLALR + Carbamidomethyl (C)
☑	1016	1007.71	3020.10	3019.54	0.56	2	11	1.3e+02	1	SPKPVASCAMPVMDNMKIFTNTPLVKK + Carbamidomethyl (C); Oxidation (M)
☑	1935	724.41	2170.22	2170.08	0.14	2	11	32	1	APDSWKMSHAQIVEIMRR + Oxidation (M)
☑	1947	737.81	2210.40	2210.11	0.29	0	11	42	1	TAEHSALRPASVAEAADVMLR + Oxidation (M)
☑	1854	665.41	1993.22	1992.99	0.23	2	11	34	1	MRNRAACYFHATGVALR + Carbamidomethyl (C)
☑	1747	888.05	1774.09	1773.95	0.14	2	11	38	1	LYLFSKVTAMCIKNK + Oxidation (M)
☑	878	952.13	2853.38	2853.51	-0.13	2	11	1.2e+02	1	VSSSGSARLQSPAASTAALAPAVTSSAPRR
☑	432	728.85	2183.52	2184.09	-0.57	0	11	1.5e+02	1	HLLHNGWMEYQEVILFR
☑	854	939.22	2814.65	2814.31	0.34	2	11	1.4e+02	1	DIKKENPELIAHCSQSSQNSESEGK + Carbamidomethyl (C)
☑	896	961.03	2880.07	2880.30	-0.23	0	11	1.7e+02	1	MFCPVFLNPEASLGGLAVCGAHCAMR + 3 Carbamidomethyl (C); Oxidation (M)
☑	1257	571.42	1140.83	1140.60	0.23	1	11	44	1	RSVGPGQDVAR
☑	737	885.21	884.20	884.50	-0.30	0	11	34	1	SGAAPIELK
☑	1599	731.96	1461.91	1461.72	0.19	1	11	42	1	TIPANGKFSEDQR
☑	830	926.26	925.26	925.46	-0.20	1	11	33	1	EKYMVEK

☑	1242	564.90	1127.79	1127.59	0.20	2	11	40	1	AKAEAMKAHR + Oxidation (M)
☑	2022	799.14	2394.39	2394.26	0.13	2	11	38	1	IPRQYIIKVDLLFQSCCNK + 2 Carbamidomethyl (C)
☑	384	698.79	697.78	698.37	-0.59	0	11	1.2e+02	1	HSTNLK
☑	991	998.45	997.45	997.47	-0.03	0	11	34	1	SYDLNTTGK
☑	167	568.70	1135.38	1135.69	-0.31	0	11	86	1	ELIDPIVLPK
☑	675	861.04	860.03	860.49	-0.46	2	11	92	1	KKMELGR
☑	1623	755.90	1509.78	1509.94	-0.16	0	11	43	1	EIIILITNLVIEK
☑	266	623.27	1244.52	1244.70	-0.18	1	11	1.3e+02	1	SVIGKADLSEVK
☑	481	756.67	2267.00	2267.05	-0.05	0	11	1.4e+02	1	YESVMSSVMMIGGIFVDSTAK + Oxidation (M)
☑	212	599.89	1197.78	1197.60	0.17	0	11	1.2e+02	1	YIFDSLDAVR
☑	129	524.34	1046.66	1046.55	0.10	1	11	88	1	LNSGMRELK
☑	858	939.90	2816.68	2816.36	0.33	2	10	1.5e+02	1	LNNLLKHKMIHHENTMYDGYAMK + Oxidation (M)
☑	146	546.82	1091.62	1091.60	0.02	0	10	1.7e+02	1	DVFTLSAALR
☑	215	602.84	1805.50	1804.96	0.54	1	10	2.4e+02	1	SITFPVDSKLQTINDK
☑	245	615.75	614.75	615.33	-0.59	0	10	67	1	IAEGAR
☑	516	774.55	773.55	773.40	0.15	0	10	59	1	FVPPDVT
☑	1963	1127.34	2252.66	2253.07	-0.41	0	10	69	1	EGAEEVVVAAFYASSHVDAFR
☑	229	608.07	1821.19	1820.92	0.27	1	10	1.8e+02	1	LVHEFFRTCCLQLR + Carbamidomethyl (C)
☑	1302	1165.75	1164.75	1164.55	0.20	0	10	42	1	STPACYEPLK + Carbamidomethyl (C)
☑	1703	843.65	1685.29	1684.77	0.51	2	10	73	1	EAEMEFDMMLRRK
☑	755	893.89	2678.64	2678.42	0.23	2	10	1.7e+02	1	NGTIIKECLLLKNQMGQMIITTK + Carbamidomethyl (C); 2 Oxidation (M)
☑	820	922.53	2764.58	2764.34	0.24	0	10	1.3e+02	1	DEDNQLLELVQMYGVGNWNIIASK + Oxidation (M)
☑	192	586.89	585.89	586.29	-0.40	0	10	80	1	AMHTK
☑	1693	835.56	1669.10	1668.81	0.29	1	10	48	1	SIDATSVATTMASRSR + Oxidation (M)
☑	1985	766.07	2295.20	2295.16	0.04	2	10	47	1	YAKWNREIMLQNVLQAMR + 2 Oxidation (M)
☑	244	615.37	614.36	614.34	0.02	0	10	52	1	NVPTGK
☑	691	866.80	865.79	866.37	-0.57	0	10	56	1	MSEEEVK + Oxidation (M)
☑	566	802.48	801.47	801.47	-0.01	0	10	62	1	APIINFK
☑	1046	1025.63	1024.62	1024.56	0.06	0	10	1.4e+02	1	ENYVFIIK
☑	1698	837.56	1673.10	1672.80	0.30	1	10	54	1	MNMAVQEYLTAKMK + Oxidation (M)
☑	1530	681.43	1360.84	1360.67	0.17	0	10	49	1	AAMAGISEDDLLR
☑	929	486.67	971.33	971.57	-0.24	1	10	52	1	LAKADVLDK
☑	1018	1008.34	3022.00	3021.66	0.34	2	10	1.9e+02	1	QAMSFTRQRGCSLPILLLSSLLFLTAR
☑	397	705.59	1409.16	1409.64	-0.48	1	10	2e+02	1	VNRMSGMEDALR + 2 Oxidation (M)

☑	928	972.15	971.14	970.66	0.48	1	10	1.6e+02	1	IIKIPFLK
☑	158	561.52	1121.02	1120.48	0.54	0	10	1.9e+02	1	CGGCGSAGPSLR + Carbamidomethyl (C)
☑	1586	720.97	1439.93	1439.75	0.17	0	10	49	1	TYEELVPIVYSK
☑	133	528.99	1583.96	1583.78	0.18	0	10	1.4e+02	1	NAPTAANPPHGAVPDR
☑	143	542.61	1624.81	1624.93	-0.12	2	10	1.2e+02	1	ARLLEVGKELDNLR
☑	1754	896.42	1790.82	1790.75	0.08	0	10	62	1	YDTDANCAGTSTSGVCVK
☑	972	990.99	1979.97	1979.89	0.08	0	10	2.5e+02	1	HCGVENMFIDEVLFGDR
☑	1668	804.98	1607.94	1607.81	0.13	2	10	49	1	SRMQPYVDELKSR
☑	1953	1117.09	2232.16	2232.13	0.03	0	10	46	1	VYFALNLMNEDLFEFIVR
☑	1954	746.80	2237.39	2237.31	0.08	2	10	53	1	VNAKPLASLGKVTSEKPSGKVK
☑	622	833.29	2496.85	2496.37	0.47	2	10	2e+02	1	LVSVPTMPRVPRQELMDFLLR
☑	189	586.33	1170.65	1170.67	-0.02	2	10	1.4e+02	1	ELRRLVEEK
☑	1431	628.99	1255.96	1255.67	0.29	1	10	61	1	TLPGVAVLDDKE
☑	1952	744.15	2229.42	2230.00	-0.58	1	10	53	1	ASATCDAFKCAVAMYGFVFGR + Oxidation (M)
☑	2109	905.09	2712.25	2712.28	-0.03	0	10	40	1	QPSYFMSAIVSLYACLNGYLDDVK + Oxidation (M)
☑	968	988.90	2963.67	2963.41	0.26	0	10	1.7e+02	1	GHGLQQNPDQTTLNLSPTIEDENDSLK
☑	1922	719.02	2154.04	2154.05	-0.02	0	10	46	1	DGTSQSEALFCSGVASISVILA
☑	400	710.08	2127.23	2126.99	0.23	1	10	1.9e+02	1	ACQHCLRSLESPQETVNR + Carbamidomethyl (C)
☑	478	755.77	2264.29	2264.12	0.17	2	10	1.4e+02	1	IRRVDESLMCAFHFAVEGK + Carbamidomethyl (C)
☑	489	759.42	2275.24	2275.23	0.01	1	10	1.8e+02	1	EIAALTLGPARDPETGLPIWR
☑	1099	1050.86	1049.85	1049.52	0.33	2	10	1.6e+02	1	FFCKFCKK
☑	1417	622.39	1242.76	1242.54	0.22	1	10	57	1	CAEKEHENAR + Carbamidomethyl (C)
☑	2026	1203.60	2405.18	2405.26	-0.08	1	9	46	1	VTDLLEFFGVQGITAASPREQK
☑	1062	1031.95	3092.82	3093.32	-0.51	0	9	2e+02	1	ALSESGDGDNYSVDMDLEDAAPPMFYIR + Oxidation (M)
☑	699	871.04	870.03	870.53	-0.50	2	9	78	1	KKPKENK
☑	1518	672.94	1343.87	1343.63	0.23	1	9	60	1	TPEELDNENRK
☑	790	909.57	1817.13	1816.98	0.15	0	9	1.7e+02	1	YLVELQPLTEAITEAK
☑	1209	1111.87	3332.58	3332.60	-0.02	2	9	1.8e+02	1	GKEETMKGADTSTTEVISAGNEEPPIISSGAAR
☑	922	970.80	2909.38	2909.32	0.06	0	9	1.7e+02	1	MPIMNELNVCVAQLQPSEFQCWGEK + Oxidation (M)
☑	337	666.89	1331.76	1331.72	0.04	2	9	2e+02	1	EQMAALEKLRK + Oxidation (M)
☑	916	968.09	967.08	967.50	-0.42	0	9	1.8e+02	1	GVNLLHCR + Carbamidomethyl (C)
☑	1042	1023.53	1022.52	1022.46	0.06	0	9	52	1	MYTTQHSR
☑	1650	792.50	1582.98	1582.73	0.25	1	9	53	1	TGEGVKEAFENMQK + Oxidation (M)
☑	938	975.52	974.51	974.58	-0.06	1	9	1.8e+02	1	TGGSTKVVVK

☑	1278	1152.52	3454.54	3454.80	-0.26	1	9	1.5e+02	1	TQYTLFTAMIQENIQEQLTLASTILGDIRK + Oxidation (M)
☑	423	722.42	1442.83	1442.83	-0.00	1	9	1.6e+02	1	VALGGSLQKALCVK + Carbamidomethyl (C)
☑	640	840.16	839.15	839.36	-0.21	0	9	2e+02	1	AACGEFSR
☑	503	766.01	1530.00	1529.70	0.30	0	9	2e+02	1	SSQNSEVPQNSEPK
☑	1338	593.94	1185.86	1185.60	0.26	0	9	60	1	LNAEADDGLIR
☑	813	921.72	2762.13	2762.51	-0.38	1	9	2.1e+02	1	RGPSAEAMPNGLLLAIASTLADKPTLR
☑	556	797.23	2388.65	2389.04	-0.38	2	9	2.1e+02	1	LCCFDPLMCCREHPGSHRGTK
☑	1267	1148.29	3441.84	3441.58	0.26	1	9	2e+02	1	DSFEQVQHWMSEIDNHASQDVCRLLVGNK + Carbamidomethyl (C)
☑	371	690.02	689.01	689.32	-0.31	0	9	83	1	DIEEGK
☑	377	693.91	692.90	693.37	-0.47	0	9	1.1e+02	1	SMVITK + Oxidation (M)
☑	408	713.84	2138.48	2139.05	-0.57	1	9	2.1e+02	1	GSICLSVLQRVQTMCVSDK + Carbamidomethyl (C); Oxidation (M)
☑	373	691.96	690.96	691.38	-0.42	1	9	1.1e+02	1	FGGRQK
☑	831	464.38	926.75	926.49	0.26	0	9	50	1	YLYINNK
☑	351	677.42	1352.83	1352.75	0.08	2	9	1.9e+02	1	HFVAGGAARRVGR
☑	521	780.59	2338.73	2338.19	0.55	2	9	2.2e+02	1	LCIWASAAYARRGCLMLAQK + 2 Carbamidomethyl (C)
☑	1748	892.88	1783.74	1783.97	-0.22	2	9	60	1	ASVPSEQSGRLTRQLR
☑	738	885.30	2652.87	2652.37	0.50	1	9	1.9e+02	1	LAYRPVHMETLTNEVDSIPPAKR + Oxidation (M)
☑	664	854.28	1706.55	1706.84	-0.29	2	9	2.1e+02	1	KCKYLHVCPYSPR + 2 Carbamidomethyl (C)
☑	329	661.97	1321.93	1322.51	-0.58	0	9	2.5e+02	1	CQGAACGENGGGSR + Carbamidomethyl (C)
☑	806	459.33	916.65	916.40	0.25	0	9	73	1	VEYAYMAA
☑	323	657.11	1968.29	1968.04	0.26	2	9	2.5e+02	1	KVLLSGPPDCANWSRLR + Carbamidomethyl (C)
☑	1705	563.59	1687.74	1687.87	-0.13	0	9	66	1	NDPSIIMVDHLGLHK
☑	1401	1225.89	2449.77	2450.16	-0.39	2	9	1.8e+02	1	TSPALHDRESHSTRTEGSAAGQR
☑	430	724.96	1447.90	1447.63	0.26	0	9	1.8e+02	1	QGDASEVEEDIEK
☑	741	444.23	886.44	886.49	-0.05	0	9	85	1	ALIGTEAGR
☑	153	554.04	1106.07	1106.64	-0.57	1	9	2e+02	1	IICHLPKQR
☑	223	604.10	1809.29	1808.96	0.33	1	9	2.9e+02	1	AEVASILSIKGYSSQTR
☑	1990	767.54	2299.61	2299.14	0.47	1	9	98	1	HRSLPYHELMSTTVQELSR + Oxidation (M)
☑	974	496.34	990.66	990.60	0.06	1	9	72	1	RALVTIYR
☑	2174	1057.24	3168.69	3168.47	0.22	1	9	46	1	SHQTNCLCFLLPSYSHPSPSQIKFCMK + Carbamidomethyl (C); Oxidation (M)
☑	984	994.42	2980.24	2980.48	-0.25	1	9	1.8e+02	1	ININLEKDIISNNFTSSVDEMTTVQR
☑	263	621.92	1241.82	1241.68	0.13	1	9	2.2e+02	1	TLLAREANQAR
☑	1780	919.65	1837.28	1836.87	0.41	1	9	81	1	CAVACKIMTPLCNAASK + 2 Carbamidomethyl (C)
☑	316	654.00	652.99	653.32	-0.33	0	9	1.8e+02	1	FCSAVK

☑	151	552.39	1102.76	1102.59	0.17	0	9	1.5e+02	1	EPELLDFLK
☑	784	454.40	906.79	906.54	0.25	0	9	74	1	HALLQAVR
☑	194	587.67	586.67	586.34	0.32	0	9	69	1	GEIIR
☑	1008	502.91	1003.81	1003.52	0.29	1	9	76	1	APSMSLRAR + Oxidation (M)
☑	1608	740.71	1479.40	1479.83	-0.43	0	9	88	1	VLSVVSSSSLFSLR
☑	702	872.05	2613.13	2613.35	-0.22	0	9	2.5e+02	1	GGVVEIQRPDFLLVTFGAHSNMAR
☑	417	718.96	1435.90	1435.76	0.15	1	9	2.8e+02	1	NRFVDAFVAIER
☑	1152	1084.22	2166.42	2166.25	0.17	0	9	2e+02	1	IFILCVLLVIHTNALIDEK
☑	942	976.08	2925.21	2925.57	-0.36	2	9	2.8e+02	1	IKTIYHAADVSLIFSQLDYSLCIKK + Carbamidomethyl (C)
☑	367	686.84	685.83	686.43	-0.60	1	9	1.4e+02	1	AEVLKK
☑	1575	712.42	1422.83	1422.82	0.01	2	9	70	1	ARAAAPLVQRNTR
☑	868	945.81	944.80	944.53	0.27	1	9	2.4e+02	1	GLPSKQTSK
☑	1556	698.01	1394.00	1394.57	-0.57	1	8	68	1	CNGPGTSKGCGSDR + Carbamidomethyl (C)
☑	1191	1103.19	3306.56	3306.68	-0.12	2	8	2.2e+02	1	HTLFAATTSLRGSMASSLRSTSGSPMLPLTGR + Oxidation (M)
☑	2067	843.16	2526.45	2526.18	0.26	1	8	57	1	FAVDAAMAAPQVDAGTRHENYHR
☑	1138	1073.09	3216.26	3216.72	-0.46	1	8	2.7e+02	1	APVRCVAFSSNADQLISASIDGTIILLSLSR
☑	455	742.47	1482.93	1482.64	0.28	1	8	2.1e+02	1	GVPRMQDSESSMK + 2 Oxidation (M)
☑	480	756.39	2266.15	2266.13	0.01	0	8	1.8e+02	1	FIVNSDGLNSINIWSVETMK
☑	1557	698.49	1394.96	1394.82	0.14	1	8	69	1	EVGIAALQGKPGKK
☑	646	842.57	841.56	841.47	0.09	0	8	55	1	AALTPVDR
☑	1607	1478.64	4432.90	4433.30	-0.40	2	8	1.7e+02	1	MKPMSCSKQSAASVLLAVLLATTGTEGAANMGLLKGTWVPLCK + Carbamidomethyl (C)
☑	1804	631.85	1892.52	1892.75	-0.22	0	8	1.2e+02	1	MFTSSGCSGTCSGPTNAR + 2 Carbamidomethyl (C); Oxidation (M)
☑	88	461.73	1382.17	1381.79	0.38	0	8	1.9e+02	1	LLSTYIVPSYVK
☑	1517	671.99	1341.97	1342.53	-0.56	0	8	73	1	CFEGCPAPYDK + 2 Carbamidomethyl (C)
☑	466	747.51	746.50	746.40	0.10	0	8	2.6e+02	1	IDLVCK + Carbamidomethyl (C)
☑	59	379.48	756.94	757.38	-0.44	0	8	2e+02	1	FVVCYK
☑	85	453.91	1358.72	1358.74	-0.02	0	8	1.6e+02	1	SYQMLLLHVQK
☑	1719	854.47	1706.93	1706.84	0.09	2	8	71	1	CQDKTSSDKPSLRSR
☑	246	616.56	615.56	615.33	0.22	0	8	3.6e+02	1	IDGGVR
☑	1570	710.47	1418.92	1418.63	0.29	1	8	84	1	CGNTERRPNGGCR
☑	162	563.58	1125.15	1125.63	-0.48	1	8	2.4e+02	1	LYRLPPPDR
☑	2207	1187.16	3558.47	3558.88	-0.42	1	8	56	1	ILMLVFNIMTISCPFYVKNAANICLSNLLLR + 2 Oxidation (M)
☑	1287	1158.42	1157.42	1157.57	-0.15	0	8	2.4e+02	1	LMSYYGRPR + Oxidation (M)
☑	1063	1032.56	3094.66	3094.48	0.17	2	8	2.3e+02	1	RTEPWGSVSGILYTDADGDAQRLTTEEK

☑	412	716.16	715.16	715.34	-0.19	0	8	94	1	HATTMR
☑	157	560.64	1678.89	1678.75	0.13	1	8	2.2e+02	1	TILSYCGNICTKCF + 2 Carbamidomethyl (C)
☑	159	562.97	1685.89	1685.73	0.15	0	8	2.1e+02	1	IGSSATTVSCSNDVCSR
☑	1110	1056.46	1055.46	1055.52	-0.07	0	8	2.1e+02	1	ASMFAFVQR
☑	1583	718.87	1435.73	1435.74	-0.01	2	8	77	1	SSNNVNRHPRQK
☑	1594	725.91	1449.81	1449.67	0.14	1	8	76	1	NAGCVVNDMLDRK + Oxidation (M)
☑	492	760.32	759.31	759.42	-0.11	1	8	2.6e+02	1	DKASALR
☑	532	787.53	786.53	786.39	0.14	0	8	90	1	NPTPGSSK
☑	1680	815.05	1628.09	1627.89	0.20	1	8	86	1	RQPGVTIVHGHVGSGK
☑	777	904.71	2711.10	2711.42	-0.32	2	8	3.1e+02	1	LGEGTYGEVCKAVDILSNTFVALKK + Carbamidomethyl (C)
☑	1886	686.78	2057.33	2057.04	0.28	1	8	85	1	AGAFRRPPTAFHDTIGSEK
☑	319	327.77	653.52	653.32	0.19	0	8	45	1	DGVAHR
☑	1433	1258.87	3773.58	3773.80	-0.22	2	8	2.5e+02	1	KPNDNDFYFVHSHSPQYFSFRESKAWINSVK
☑	1656	794.45	1586.89	1586.88	0.01	1	8	94	1	VQAIKGTLIVCEGTR
☑	2147	999.61	2995.81	2996.38	-0.57	1	8	73	1	AFCQFCLDPIYQIFDAVMTEKQEK + 2 Carbamidomethyl (C); Oxidation (M)
☑	774	903.86	2708.56	2708.34	0.22	2	8	2.7e+02	1	LRHESAAYALAQLRQAMSTMTSEK + Oxidation (M)
☑	97	491.35	980.68	980.43	0.25	0	8	1.7e+02	1	GFASDNQSR
☑	1377	603.91	1205.81	1205.57	0.24	1	8	89	1	VASAEDEMAKR
☑	720	877.66	876.65	876.45	0.21	0	8	1e+02	1	NFAQIER
☑	948	979.46	1956.90	1957.02	-0.12	1	8	2.1e+02	1	CHILSGGTRLTFGNSPLK + Carbamidomethyl (C)
☑	1833	979.93	1957.86	1957.98	-0.12	2	8	70	1	HDCELAALRFQTVDRK + Carbamidomethyl (C)
☑	90	479.31	1434.91	1434.69	0.22	1	8	1.8e+02	1	SRSGSDPNIMTVR + Oxidation (M)
☑	636	420.32	838.63	838.44	0.19	1	8	76	1	VRQGHDK
☑	849	936.95	935.94	935.42	0.52	0	8	3.3e+02	1	WELSCGGGK
☑	764	898.24	2691.68	2691.17	0.51	0	8	2.9e+02	1	GEEWEHIIFYCCFLMMIQNNR + Oxidation (M)
☑	418	720.05	2157.12	2156.93	0.19	1	8	2.6e+02	1	MSYPTSQGEEASKEAGDNTR
☑	1960	751.43	2251.27	2251.04	0.23	1	8	71	1	VMVQLAACAQEDGTECGRVAK + Carbamidomethyl (C); Oxidation (M)
☑	119	518.93	1553.76	1553.72	0.04	0	8	2e+02	1	MSSVTAAISEAASACR
☑	747	889.33	888.32	888.38	-0.06	0	8	2.9e+02	1	GCAGDDPVR
☑	1532	682.93	1363.85	1363.61	0.24	1	8	87	1	NEATPXAGNKXLK
☑	523	782.88	781.88	781.29	0.59	0	8	1.1e+02	1	MESEDR + Oxidation (M)
☑	949	979.46	2935.37	2935.35	0.02	2	8	2.3e+02	1	ARQRQQEEQSSITETTMPADEDELK + Oxidation (M)
☑	579	811.55	810.54	810.39	0.15	1	8	66	1	EKTYDR
☑	595	817.24	816.23	816.47	-0.23	2	8	3.4e+02	1	RRTSAAR

☑	139	534.36	1066.71	1066.44	0.28	0	8	1.9e+02	1	NMMCAATPR + Carbamidomethyl (C); Oxidation (M)
☑	1598	488.30	1461.89	1461.69	0.20	1	8	91	1	AADASKAAETAEAEK
☑	314	653.18	1956.53	1956.14	0.39	1	8	2.8e+02	1	IQGNVLLNVAVTGGFTLKI
☑	1682	547.42	1639.23	1638.76	0.47	1	7	1.2e+02	1	VADMGVCRLNFDAR + Carbamidomethyl (C); Oxidation (M)
☑	1898	699.45	2095.32	2095.18	0.14	0	7	85	1	GTLFLLLSCSVQINSIFLK
☑	206	597.98	1193.94	1193.60	0.35	2	7	2.3e+02	1	KTMKEESVDK
☑	1729	580.78	1739.32	1738.85	0.47	0	7	1.4e+02	1	GFCQGGGPISLNFAPFK
☑	2051	830.56	2488.65	2488.30	0.35	2	7	1.1e+02	1	KDQRTTILDSVLDTIGSTPCIR + Carbamidomethyl (C)
☑	150	550.06	1098.11	1097.63	0.48	0	7	2.9e+02	1	LTLLVPEDAK
☑	1827	973.64	1945.28	1944.95	0.33	2	7	95	1	SYTGTFSACAKDNAVVRR
☑	135	531.33	1590.96	1590.80	0.16	1	7	2.5e+02	1	SAQLPSKLSSAAGSCK + Carbamidomethyl (C)
☑	1660	795.13	1588.24	1587.91	0.33	1	7	1.6e+02	1	ARFTTAIQQIVNVK
☑	93	486.09	1455.26	1455.80	-0.55	2	7	2.7e+02	1	KNIQKLNGDLGEK
☑	914	967.72	966.71	966.54	0.17	1	7	78	1	WLHVKER
☑	969	494.99	987.97	988.51	-0.54	0	7	1.9e+02	1	EVDGSILEK
☑	1687	829.46	1656.91	1656.77	0.14	1	7	93	1	FREMLLLMDCNASV + Oxidation (M)
☑	341	669.45	1336.89	1336.61	0.28	1	7	2.4e+02	1	NACAEVMASEKK + Carbamidomethyl (C)
☑	1080	1040.02	2078.03	2078.09	-0.06	2	7	3.4e+02	1	MTKATKPSSYISKQFAYK
☑	1074	1036.35	2070.68	2070.10	0.58	0	7	3.2e+02	1	FLAAAEAAHAQIEHHLITK
☑	1834	980.03	1958.04	1958.09	-0.06	2	7	82	1	EFLNVRGKASVLQVSSPK
☑	241	613.87	1838.57	1838.01	0.56	1	7	2.9e+02	1	GQSQLRVLLARPSEER
☑	1502	1317.49	3949.45	3949.79	-0.33	2	7	3.1e+02	1	MTTCRLMCALLVLALCYCPSVCAEETPKDTEAER + 3 Carbamidomethyl (C); Oxidatior
☑	1850	994.26	1986.51	1987.00	-0.50	1	7	1.5e+02	1	VTKIADNHSEEYALELR
☑	345	673.44	672.44	672.34	0.10	0	7	1.4e+02	1	IGGCAPR
☑	313	652.35	1302.69	1302.52	0.16	0	7	2.6e+02	1	YSVDECMTAER
☑	460	745.90	2234.67	2235.00	-0.33	1	7	5.1e+02	1	SPSTPVEGSMREAICAEYHR + Oxidation (M)
☑	261	620.74	1859.21	1858.86	0.34	1	7	3.3e+02	1	DWTRVSPVMPANAEDR + Oxidation (M)
☑	1755	597.96	1790.86	1790.96	-0.09	1	7	1.2e+02	1	RMHERPIGDLVEALR
☑	926	971.87	1941.73	1942.01	-0.28	2	7	2.7e+02	1	AIAESTVDPTINKDNRAK
☑	1733	873.09	1744.17	1743.77	0.40	2	7	99	1	MKEPLKCSTTTGMNM + Carbamidomethyl (C); Oxidation (M)
☑	1939	726.41	2176.21	2176.18	0.02	2	7	86	1	TVGMAATCISSINVSLGRIKR
☑	128	523.83	522.82	522.24	0.58	0	7	14	1	FGGDK
☑	2193	1117.72	3350.13	3350.68	-0.55	2	7	1.1e+02	1	AENVLLVVIRCNTCHSVGTVCHLCDVRVK + 3 Carbamidomethyl (C)
☑	2029	808.41	2422.22	2422.16	0.06	0	7	81	1	GASALGAHLGVGDSAAAAVGTDGGGGGGGR

☑	354	679.07	2034.19	2033.99	0.20	0	7	4e+02	1	IIPEDLNAADFGDGYSIPK
☑	1184	1099.89	1098.89	1098.48	0.40	0	7	2.8e+02	1	CGYAEACVGVK
☑	333	665.38	664.37	664.32	0.05	0	7	82	1	CTAAATK
☑	1223	1116.84	3347.49	3346.89	0.60	2	7	3.4e+02	1	ALDKKDYQSLELQLHGTLLATVNVGPVAIIK
☑	1504	660.45	1318.88	1318.60	0.28	0	7	1e+02	1	AMNNTIYGTYR + Oxidation (M)
☑	1627	764.93	1527.85	1527.81	0.04	0	7	1.1e+02	1	LLQSAVCAAPTIER + Carbamidomethyl (C)
☑	1495	1315.70	3944.07	3943.88	0.19	2	7	2.4e+02	1	MSWSSLFALLIIAMHAHDEVCARCLHDKCLQTR + 2 Carbamidomethyl (C); 2 Oxidatio
☑	249	618.54	1235.07	1235.61	-0.54	0	7	4.7e+02	1	AVVSSDFIGDAR
☑	318	654.29	1306.57	1306.64	-0.07	0	7	2.7e+02	1	NYKPGNAPQYR
☑	1864	1008.83	2015.64	2016.06	-0.42	2	7	1.3e+02	1	MNFAKACLHLDINTIKK + Carbamidomethyl (C)
☑	1039	1022.55	1021.54	1021.52	0.02	1	7	2.9e+02	1	AGGAGSVMKTK + Oxidation (M)
☑	501	765.02	2292.04	2292.20	-0.16	2	7	3.3e+02	1	ECQLEVVKYLISIGADKEAK + Carbamidomethyl (C)
☑	1386	606.39	1210.77	1210.71	0.05	1	7	88	1	QAAELAARIIR
☑	907	964.73	963.72	963.62	0.10	0	7	1.2e+02	1	LIPVAAKPR
☑	2059	837.47	2509.40	2509.30	0.09	2	7	1.1e+02	1	EIEIKEENKENNIENLPLSVR
☑	164	567.17	1698.48	1698.81	-0.33	0	7	2.9e+02	1	DSEAVLAAEDGVGDVPR
☑	1357	599.43	1196.84	1196.77	0.07	2	7	98	1	KPVRKVTITR
☑	1117	1061.90	3182.68	3183.22	-0.55	2	7	3e+02	1	KCLKCDSNCETCYGISTYCMSCSNDK + 5 Carbamidomethyl (C)
☑	1043	1023.72	3068.13	3068.60	-0.47	2	7	3.5e+02	1	YHERIHDIVAEASSAQVYLQMRILAR
☑	1572	710.99	1419.96	1419.73	0.24	0	7	1e+02	1	MVLWELMDIVR + Oxidation (M)
☑	568	804.06	803.05	802.51	0.54	0	7	1.9e+02	1	AIALFIR
☑	1232	1121.67	1120.66	1120.57	0.09	1	7	3.1e+02	1	EKGYQIMPR
☑	485	758.45	757.44	757.36	0.08	0	7	1.2e+02	1	TFDFTK
☑	1841	656.69	1967.04	1967.12	-0.07	2	7	1.1e+02	1	RIVENAGNNGSIIIEKIK
☑	274	627.12	1252.24	1252.54	-0.30	0	7	3.8e+02	1	ALQECNGDFEK
☑	386	700.49	699.48	699.39	0.09	0	7	3.6e+02	1	DTVPIR
☑	1027	1014.33	2026.65	2027.00	-0.35	0	7	3.9e+02	1	IEMEAIDGWDDLPLVVGR
☑	1837	982.61	1963.21	1962.91	0.30	2	7	1.2e+02	1	ACVEDNLSAPDLDRKCSK
☑	2149	1002.72	3005.13	3005.58	-0.45	1	7	1.1e+02	1	CFNLVILKHIEIIDEIYEIIEDFK + Carbamidomethyl (C)
☑	1307	1167.59	1166.59	1166.51	0.07	1	7	2.8e+02	1	YTRNMDHSK + Oxidation (M)
☑	1353	1194.55	1193.54	1193.59	-0.05	1	7	2.7e+02	1	GAAFSASRSGQR
☑	326	657.43	1969.27	1968.95	0.33	0	7	3.4e+02	1	NVNIPHLQTINECGFDR
☑	1160	1088.34	3262.01	3261.58	0.44	1	7	3.9e+02	1	TSTMDKDAEPEQLVFNFVSDYVIELLSR + Oxidation (M)
☑	1383	1209.77	1208.76	1208.71	0.05	0	6	3.3e+02	1	ELGLPVLAELR

884	956.56	955.55	955.45	0.10	0	6	1e+02	1	INGHDCIK + Carbamidomethyl (C)
1871	674.81	2021.40	2021.98	-0.59	0	6	1.5e+02	1	SNYLSFLENIYNINYR
1525	676.02	1350.03	1349.65	0.39	1	6	1.3e+02	1	FGSQADIDWRR
1938	1088.80	2175.59	2176.18	-0.59	2	6	1.7e+02	1	AALAQAQRELSELRTVHQR
1481	650.89	1299.76	1299.79	-0.02	2	6	1.5e+02	1	SREKNLTVILK
1909	707.26	2118.76	2119.14	-0.39	2	6	1.2e+02	1	DERAALGLLGGWLPPGRWR
451	371.14	740.27	740.38	-0.11	1	6	65	1	EPKDPR
963	985.06	984.06	984.53	-0.47	0	6	4e+02	1	IYASQYLK
1424	1252.21	1251.21	1251.60	-0.40	1	6	3.3e+02	1	EANAREMFLR + Oxidation (M)
606	412.81	823.61	823.41	0.20	0	6	83	1	VATGSFDK
1927	720.13	2157.36	2157.05	0.31	0	6	1.1e+02	1	APVISEQSPEEMTLEELQK
956	982.49	981.48	981.42	0.06	0	6	3.2e+02	1	NNPDMTYK
185	582.95	1745.82	1745.87	-0.04	2	6	3e+02	1	TWCRFAVTTYRQSK
1988	767.23	2298.67	2299.17	-0.50	0	6	1.7e+02	1	TSLANQFSQTLLSFISQSEAK
591	816.09	2445.25	2445.41	-0.16	2	6	4.3e+02	1	TGSGKTLAFLIPIVEIISRAGFR
1670	538.63	1612.86	1612.73	0.14	0	6	1.4e+02	1	HNEELQSQLCEAR + Carbamidomethyl (C)
1714	850.19	1698.37	1698.83	-0.46	1	6	2e+02	1	CPRVDIVHMIGGSSR + Carbamidomethyl (C); Oxidation (M)
196	590.36	1768.06	1767.83	0.23	2	6	3.5e+02	1	EVCEEACKEFKGLAR + Carbamidomethyl (C)
1763	905.63	1809.24	1808.92	0.33	1	6	1.3e+02	1	RTWEINNILGEHEAK
216	603.38	1807.12	1807.03	0.09	1	6	4.3e+02	1	AHLLSKSELVVAAVSQR
1825	648.43	1942.26	1942.82	-0.56	0	6	1.1e+02	1	CWVFDCAAPGLYCEPR + 2 Carbamidomethyl (C)
260	620.48	1238.95	1238.77	0.18	1	6	4.6e+02	1	VINPSKVTLLR
1949	740.74	2219.19	2219.05	0.14	1	6	1.1e+02	1	ERELAEASQQLQQSEAMAGK + Oxidation (M)
227	607.39	1819.14	1818.86	0.28	0	6	3.4e+02	1	QHTCPICMEHLPSVPK
856	939.53	2815.58	2815.27	0.31	2	6	3.3e+02	1	LETCSFRPVASDDDDDEFRRIACR
1423	1251.41	1250.41	1250.67	-0.26	1	6	3.7e+02	1	MAEKYQQLIK
744	888.92	1775.84	1776.00	-0.16	2	6	6e+02	1	AKDIYTLARCATLLPK
594	816.56	2446.66	2447.19	-0.54	1	6	4.5e+02	1	GASAMAATASCPAGAHVRALPHTQR + Oxidation (M)
1529	680.34	1358.67	1358.68	-0.01	1	6	1.3e+02	1	ASSGFVSTFKNSK
2082	858.55	2572.63	2572.24	0.39	1	6	1.3e+02	1	MGNIHISQNQEFPCPKLFGMGPK
264	622.11	1242.21	1241.71	0.50	2	6	5.2e+02	1	VQGEALGKNAKK
1071	1035.66	1034.66	1034.59	0.07	0	6	4e+02	1	LGKPQHDLK
1889	689.39	2065.14	2065.01	0.13	2	6	1.1e+02	1	ETLFNGKLAVGGRDQESTGS
892	480.49	958.96	958.47	0.49	0	6	2.9e+02	1	AEADDGLIR

☑	1349	1193.69	1192.68	1192.72	-0.04	1	6	3.2e+02	1	FKILVELFGK
☑	605	824.57	823.57	823.44	0.12	1	6	92	1	HERGLGR
☑	836	929.90	928.89	928.56	0.33	2	6	4.5e+02	1	SKLAQARR
☑	719	877.59	2629.73	2629.15	0.59	1	6	4.4e+02	1	VQHMCIVSCTLQYCARCGEWAR + Carbamidomethyl (C); Oxidation (M)
☑	1815	640.72	1919.14	1918.98	0.16	2	6	1.1e+02	1	LPKYDRVGISAELCCPR
☑	353	678.54	2032.61	2033.13	-0.52	1	6	5.6e+02	1	DINALVTFALSLVRTLCK + Carbamidomethyl (C)
☑	1269	1149.95	3446.83	3446.32	0.50	0	6	3.7e+02	1	CLCGCPHCDPTTCDDLVNQEFESLCTCR + 6 Carbamidomethyl (C)
☑	1630	770.00	1537.98	1537.71	0.27	0	6	1.3e+02	1	IIDEFCSQNNVEK
☑	1799	942.23	1882.45	1882.73	-0.28	1	6	2.2e+02	1	SSGTSRDNDDEEEEADK
☑	1720	857.83	1713.64	1713.86	-0.22	1	6	1.2e+02	1	CCVAYLIEKEFLQR
☑	1852	997.56	1993.10	1993.22	-0.12	2	6	1.2e+02	1	LVRIHRIITISPFLTSK
☑	1215	557.42	1112.83	1112.72	0.12	0	6	1.4e+02	1	ILTVAVSGLLK
☑	172	571.70	1712.08	1711.95	0.13	2	6	3e+02	1	EKSVDKSAHGSLLTLK
☑	775	903.92	902.91	903.45	-0.54	0	6	2.9e+02	1	GCGALLQDK
☑	1317	1177.85	1176.85	1176.65	0.19	2	6	4.4e+02	1	KISEGIKGMSK
☑	1476	650.40	1298.78	1298.68	0.10	1	6	1.3e+02	1	GDNVRHVFLSR
☑	986	498.29	994.56	994.43	0.13	1	6	1.2e+02	1	CSNEKMAR + Carbamidomethyl (C)
☑	1054	1029.63	3085.88	3086.46	-0.58	0	6	4.1e+02	1	DYCPAQLCLDAAPASLPFVQQLYNAYR + Carbamidomethyl (C)
☑	1620	753.48	1504.94	1504.69	0.24	1	6	1.3e+02	1	CQKEGKPCVPNMR + Oxidation (M)
☑	1625	756.11	1510.21	1509.76	0.45	2	6	2.4e+02	1	CVYVKENTNGKAK + Carbamidomethyl (C)
☑	444	735.89	2204.65	2204.13	0.51	2	6	6.2e+02	1	SFNSTKCYPVFPFIAIKDK
☑	865	943.56	942.55	942.55	0.00	2	6	1.3e+02	1	DKQSPKLK
☑	889	958.89	2873.65	2873.16	0.49	0	6	4.2e+02	1	EGGAGGGGGGGGGGGGGGGGGGGGGGGGGGGGGGGFGAGGGR
☑	154	554.11	1659.30	1658.81	0.49	1	6	4.2e+02	1	KVNDIPVDDGWVMR + Oxidation (M)
☑	1487	1304.33	2606.64	2606.08	0.56	1	6	4.2e+02	1	IDVAGCDDCDRDIYCYAPGTASAR + Carbamidomethyl (C)
☑	279	630.99	629.98	630.33	-0.35	0	6	6.5e+02	1	DIDIR
☑	625	833.61	832.60	832.41	0.19	0	6	5.5e+02	1	FGPQGEAK
☑	973	991.54	990.53	990.48	0.05	0	5	1.5e+02	1	CTLVEAEAR
☑	1519	673.10	1344.19	1343.74	0.44	0	5	2.7e+02	1	SIYIHEASALLK
☑	456	743.48	742.47	742.40	0.07	0	5	4e+02	1	LSHTSAK
☑	262	621.32	1860.95	1860.84	0.11	1	5	3.8e+02	1	KAVHELDSDAPCYSNR + Carbamidomethyl (C)
☑	332	665.11	1328.20	1328.77	-0.56	1	5	5.1e+02	1	EILLLATGESKR
☑	617	829.82	828.81	828.46	0.36	1	5	2e+02	1	SPRSLGGR
☑	1029	1015.96	3044.85	3044.61	0.24	2	5	5.4e+02	1	SQIMIIGMKGITLDSIGYKNCNILIYK + Oxidation (M)

649	844.41	1686.80	1686.73	0.07	0	5	4.2e+02	1	DPAAHPASSEMTSAGMK
302	646.35	1936.02	1935.96	0.07	2	5	4.9e+02	1	RDDVGGDFFNALRDAIR
846	935.11	2802.31	2802.47	-0.16	2	5	5.1e+02	1	LRRPCVAAVDMFAGASSTIVAVSPRR + Carbamidomethyl (C); Oxidation (M)
2154	1017.51	3049.50	3049.45	0.05	0	5	1.1e+02	1	GAEAAAAAAEQLEEEVVDDGQGQTGPAAAPVK
1319	1178.09	3531.25	3531.75	-0.50	1	5	5.4e+02	1	LPRFYFISDEDMLEMLGHSQSPSVIQVHLK + Oxidation (M)
1308	1168.70	1167.69	1167.64	0.06	0	5	1.7e+02	1	MFLFGPLISK + Oxidation (M)
1580	714.93	1427.84	1427.68	0.16	0	5	1.6e+02	1	VDVNDLPDDLGEK
1561	703.37	1404.73	1404.78	-0.05	2	5	1.6e+02	1	LEIRRNISNYK
593	816.51	1631.01	1630.82	0.19	0	5	4.6e+02	1	VGTTLSGEGGDLAVEAR
1436	1262.26	3783.77	3783.85	-0.08	2	5	4.3e+02	1	MRIFKDILTNAEVVCDNDRPMDVEDEIVYVVK + Oxidation (M)
754	893.53	1785.04	1784.88	0.16	2	5	4e+02	1	RQLDEIENQERAER
1340	594.40	1186.79	1186.63	0.15	0	5	1.7e+02	1	VVYQSPEPIR
1781	613.61	1837.81	1837.90	-0.09	0	5	1.3e+02	1	DATLELPNVHVSCVGER
112	512.26	1022.51	1022.50	0.01	0	5	2.8e+02	1	QYGQVVDSK
703	872.51	871.50	871.44	0.06	1	5	4.3e+02	1	GEPTREAI
765	898.44	897.43	897.40	0.02	0	5	3.9e+02	1	MMVTASSR + Oxidation (M)
1976	761.75	2282.24	2282.41	-0.17	0	5	1.2e+02	1	LYAISLIIIVVLFGYVVFLK
1776	919.03	1836.05	1835.79	0.27	0	5	1.4e+02	1	SPSPMLCMQDLEEGAR + Carbamidomethyl (C); Oxidation (M)
601	820.48	2458.41	2458.20	0.21	0	5	4.5e+02	1	MNNPSNIKPIFYLKPSSFCDK + Oxidation (M)
1543	691.99	1381.96	1381.75	0.21	2	5	1.3e+02	1	QPIEGRSRGGGLR
654	848.97	847.96	847.49	0.48	2	5	3.7e+02	1	KRTQSTK
877	950.12	1898.22	1897.98	0.24	2	5	4.6e+02	1	CLAAFDSGFSLASRKIR + Carbamidomethyl (C)
2199	1142.61	3424.81	3424.76	0.05	1	5	1.1e+02	1	NAEDDLLENLIINHGNDPAKVEIVQAPSLQR
494	761.10	2280.29	2280.05	0.23	2	5	5.8e+02	1	AWARCCCGGQAAPGVACKYIPR
1015	1006.98	3017.90	3018.26	-0.35	1	5	6.1e+02	1	QNSMCAESNNASYAVRCCADEADPALTK + Carbamidomethyl (C)
2142	991.19	2970.56	2970.56	-0.01	1	5	1.1e+02	1	IEFEILWIGQSELFYKGIESTLIDK
1640	522.09	1563.25	1562.81	0.45	2	5	2.7e+02	1	GRFDAALDTAKEAAK
178	578.40	577.39	577.22	0.17	0	5	87	1	ESDDI
2080	856.19	2565.55	2565.26	0.29	0	5	1.4e+02	1	VYPTYDFACPIVDSVEGITHALR
317	654.09	1306.17	1306.66	-0.50	1	5	5.6e+02	1	SNDAIYEARLR
530	786.99	785.98	785.48	0.50	1	5	3.2e+02	1	KALADIR
824	924.21	1846.41	1846.98	-0.57	0	5	5.6e+02	1	VWAGVTGCDFLVALLAR + Carbamidomethyl (C)
1144	1079.05	2156.09	2156.02	0.07	1	5	6e+02	1	AQLAQKLTQNMDVMFSCR + Carbamidomethyl (C); Oxidation (M)
173	572.28	571.27	571.37	-0.10	0	5	83	1	IAGAIK

☑	945	489.67	977.33	977.55	-0.23	1	5	1.7e+02	1	EQAVKYIK
☑	1009	1005.09	1004.08	1003.61	0.48	1	5	5.6e+02	1	IDGFIAKLK
☑	912	484.30	966.58	966.45	0.13	0	5	1.3e+02	1	GICTYDPAK
☑	348	675.62	1349.23	1349.53	-0.31	0	5	6.3e+02	1	GTSCSCTSSGTFR + Carbamidomethyl (C)
☑	1856	665.85	1994.53	1994.85	-0.32	2	5	2.7e+02	1	GCRSDEAESGTEAPMRAR + Carbamidomethyl (C); Oxidation (M)
☑	657	850.65	2548.92	2548.36	0.56	1	5	5.5e+02	1	VANSAERPVDAVVAGYLLMVYRR
☑	1103	1052.32	3153.92	3153.51	0.41	0	5	5.4e+02	1	TPTTSVSSTLWWSEVACPTLAVTTTAACR + 2 Carbamidomethyl (C)
☑	1859	999.58	1997.14	1997.09	0.05	1	5	1.5e+02	1	HLAVHGRLMIIGSISSYK + Oxidation (M)
☑	1371	1204.38	3610.13	3609.80	0.33	2	5	5.6e+02	1	GWCALDGALHCVRLASPDTRASPMSALVSLVQR + 2 Carbamidomethyl (C); Oxidation
☑	256	619.65	618.65	618.32	0.33	0	5	1.8e+02	1	ECIVR
☑	1562	703.88	1405.75	1405.79	-0.04	2	5	1.9e+02	1	MSKVSEIDLLKK + Oxidation (M)
☑	2068	845.12	2532.34	2532.17	0.17	2	5	1.3e+02	1	TAALHSDNMTMEERIEENWKK
☑	615	828.89	827.88	827.32	0.57	0	5	2.8e+02	1	ADCSSMSK
☑	470	751.89	2252.65	2252.19	0.46	1	5	7.1e+02	1	RVQDTLDSQGGVILSGQVPQR
☑	891	959.70	2876.09	2875.55	0.54	1	5	5.7e+02	1	EMLHAAGAVTAVGSTLSFLHNRVLLPR + Oxidation (M)
☑	1111	1058.39	2114.77	2115.12	-0.36	2	5	4.9e+02	1	ALHPAVRASSHVNRVCVSR + Carbamidomethyl (C)
☑	1957	750.19	2247.55	2247.02	0.53	1	5	2.3e+02	1	EHHLCSVCMLPASYRCLR + 2 Carbamidomethyl (C); Oxidation (M)
☑	527	785.53	1569.05	1568.73	0.33	2	5	5.1e+02	1	RIFDDMDVDRSGK + Oxidation (M)
☑	840	931.46	2791.34	2791.48	-0.14	2	5	4.6e+02	1	EGTALMVAAVLGHVETVKLLMRHER + 2 Oxidation (M)
☑	186	584.38	1166.74	1166.55	0.19	1	5	3.5e+02	1	MVSEEDTKTK
☑	1282	578.89	1155.76	1155.49	0.27	0	5	1.5e+02	1	LYDSECVDR + Carbamidomethyl (C)
☑	2114	906.48	2716.41	2716.36	0.05	1	5	1.3e+02	1	DGFEAIPAVTRLIETDQGWGCLLR + Carbamidomethyl (C)
☑	1168	545.78	1089.55	1089.52	0.04	0	5	1.8e+02	1	TYMLAFNSK + Oxidation (M)
☑	1373	1205.30	1204.29	1204.62	-0.32	0	5	5e+02	1	VISGPLCPEYK
☑	1770	611.08	1830.22	1829.89	0.32	2	5	2.3e+02	1	DILKETNKHLSDMDR + Oxidation (M)
☑	827	925.21	1848.41	1849.00	-0.59	2	5	5.8e+02	1	DWQLEVHKAVLAERR
☑	520	780.37	1558.72	1558.80	-0.08	1	5	4.5e+02	1	TALLPAHCHSPKER
☑	648	843.82	2528.44	2528.22	0.23	1	5	5.5e+02	1	SGHIQALAECLMKNSYVWSTYK
☑	1106	1054.08	2106.14	2105.89	0.24	1	5	5.9e+02	1	MPCAHGAAACCTMPTRVNEK + Oxidation (M)
☑	1454	1278.52	3832.54	3832.83	-0.29	2	5	4.3e+02	1	FDGSIELRILTCQAIMKLADLSNTCRPFDDSCK + 2 Carbamidomethyl (C); Oxidation
☑	1458	1281.89	1280.88	1280.56	0.32	0	5	4.9e+02	1	AEMELGEDMIK + Oxidation (M)
☑	1961	751.48	2251.41	2251.00	0.41	1	5	1.6e+02	1	SRATEDACIMCGVVSGIHCSR + Carbamidomethyl (C)
☑	2115	906.94	2717.79	2718.20	-0.41	2	5	2.3e+02	1	EDTSMPTFTAFGEEPKKEDTSMPK + Oxidation (M)
☑	834	928.99	2783.95	2783.52	0.43	1	5	6.9e+02	1	RELHVATLSVQVFTSSDVVDSLLLR

☑	866	945.48	2833.43	2833.38	0.05	0	5	5e+02	1	NHIMFGGFGAWYIQSLAGLQQAPGDR
☑	887	958.10	957.09	957.51	-0.43	0	5	3.2e+02	1	QAVVTPESK
☑	927	972.10	971.09	970.50	0.60	0	5	2.8e+02	1	HPSGLSFAR
☑	1207	1109.86	3326.54	3326.46	0.08	1	5	5e+02	1	SVGYSSDGDLVMMREDYSEPSDVDNGFVLK + Oxidation (M)
☑	2096	890.64	2668.90	2669.28	-0.39	1	5	2e+02	1	QAAFLCESFKLCEPGIPIMCLSGR + Carbamidomethyl (C)
☑	1000	1001.65	3001.94	3001.51	0.44	2	5	5.7e+02	1	MFRHVSAASASSLVAARSFSSTNVYLDK
☑	1574	711.45	1420.88	1420.63	0.26	0	5	2e+02	1	GPPDTPFEGGEYR
☑	1713	850.08	1698.14	1697.86	0.28	1	5	1.7e+02	1	GIVRCMMTFLVLCR + Carbamidomethyl (C)
☑	1175	1092.38	2182.75	2182.17	0.57	1	5	5.7e+02	1	EQNQELVRQLGELTVALSR
☑	1351	1193.88	3578.62	3578.86	-0.25	2	5	5e+02	1	MIRKVWLLCFAGVVYIAFTISVFLCADNWK + Carbamidomethyl (C); Oxidation (M)
☑	1456	641.04	1280.07	1279.55	0.52	0	5	2.4e+02	1	QCDMLLAADER + Oxidation (M)
☑	1764	604.12	1809.34	1809.87	-0.54	0	5	2.8e+02	1	ENNTLTIQVYQGESSK
☑	1874	1013.60	2025.19	2025.02	0.17	2	5	1.5e+02	1	RMNSIVAKMLLDMTSGSR + Oxidation (M)
☑	722	877.66	1753.31	1752.96	0.35	0	5	6.4e+02	1	LMGERPLQHAPLHVR
☑	350	676.74	2027.19	2027.01	0.17	1	5	6.2e+02	1	YSSGMPQTSKPAFDRLVK + Oxidation (M)
☑	2128	943.04	2826.10	2826.41	-0.31	0	5	1.5e+02	1	DVGSTICMIDLVIGYTAIQSMAIWAR
☑	61	423.63	1267.88	1268.44	-0.56	0	5	3.9e+02	1	CVCGCGYCYK + 3 Carbamidomethyl (C)
☑	1844	985.52	1969.04	1969.05	-0.01	0	5	1.6e+02	1	SNLDNINIDNAVILLNSK
☑	2155	1018.26	3051.75	3051.54	0.21	2	5	1.5e+02	1	VEMDIIQSFLEEVCLKLDNLANRMTK
☑	2211	1231.51	3691.50	3691.81	-0.31	0	5	1.2e+02	1	TPTMPANSNPAVAPLSLPQQLPASMMAPNGAMLTPK + 3 Oxidation (M)
☑	324	657.27	1312.52	1312.81	-0.29	2	5	5.3e+02	1	KFHRPIFLKK
☑	639	840.02	2517.04	2517.14	-0.10	2	4	5.5e+02	1	CKTSTQAPLNGDCAASSRATFCTK + Carbamidomethyl (C)
☑	550	794.39	1586.76	1586.83	-0.06	0	4	5.1e+02	1	GAMDAANILKPMLSR
☑	1167	1090.29	2178.56	2178.94	-0.39	1	4	6e+02	1	CRCEQDGTETDAVSPLWR + 2 Carbamidomethyl (C)
☑	554	796.64	2386.89	2387.18	-0.29	2	4	6.2e+02	1	GMFACVVLVENIRTNEKFACK + Oxidation (M)
☑	1380	1208.51	3622.50	3622.75	-0.25	1	4	4.4e+02	1	LTCDIYSMQCFDAVQAPKITVTHETKPNIDK + 2 Carbamidomethyl (C)
☑	1619	753.08	1504.14	1503.75	0.39	0	4	2.3e+02	1	CLPDLGTAVANAFR + Carbamidomethyl (C)
☑	800	914.02	2739.04	2739.18	-0.14	2	4	6.1e+02	1	YICDAEVLNEMQASDCYKRMER + Carbamidomethyl (C); Oxidation (M)
☑	1081	1040.43	2078.84	2078.91	-0.07	2	4	4.8e+02	1	ELRRCEGLGMTAEACHCK + Carbamidomethyl (C); Oxidation (M)
☑	82	448.89	895.77	895.44	0.33	0	4	3.5e+02	1	SIESSAFR
☑	1882	679.22	2034.63	2034.07	0.56	0	4	2.7e+02	1	YWSAVELDVHHLILPSR
☑	2169	1043.54	3127.60	3127.49	0.12	1	4	1.3e+02	1	WMTMESRIQHMVASTSSYVARPEVGVK + 3 Oxidation (M)
☑	743	888.39	1774.76	1774.92	-0.16	2	4	5.4e+02	1	YLTVAAIFRGRMSMK + 2 Oxidation (M)
☑	2008	789.54	2365.60	2366.14	-0.54	2	4	2.4e+02	1	LDTERTGIMDANVCQVVACKK + Carbamidomethyl (C); Oxidation (M)

☑	671	858.63	2572.88	2572.37	0.51	2	4	6.6e+02	1	GGRGGGAGAKVVVEPHMLHPGVFISK + Oxidation (M)
☑	1228	1119.09	3354.25	3354.60	-0.35	1	4	7.3e+02	1	VEQNEENMITKFGCSEGEIISVSNPLSISK + Carbamidomethyl (C); Oxidation (M)
☑	346	673.84	2018.51	2018.15	0.36	2	4	1e+03	1	VNRQSPIPSAGGILQGRIR
☑	462	746.20	2235.57	2236.03	-0.47	1	4	8.5e+02	1	REAAASGGGGANMSSLAEMVAQR + Oxidation (M)
☑	960	492.70	983.38	983.50	-0.12	1	4	1.7e+02	1	MHKVNNNK
☑	519	780.13	779.12	779.42	-0.29	0	4	1.6e+02	1	TYEIVR
☑	1694	836.10	1670.18	1669.98	0.20	2	4	2.2e+02	1	IKLSKTICIAPDALK + Carbamidomethyl (C)
☑	360	683.34	682.33	682.45	-0.12	1	4	93	1	VPAIRK
☑	2018	1195.72	2389.42	2389.17	0.25	1	4	1.6e+02	1	GEYARQIFCDSFTVALQAAATK
☑	1928	720.16	2157.47	2157.03	0.44	1	4	2.4e+02	1	RAVLVGFYMEAQCEALER + Carbamidomethyl (C); Oxidation (M)
☑	1002	1002.76	1001.75	1001.60	0.15	2	4	6.8e+02	1	QLTTARKGK
☑	1644	786.06	1570.10	1569.65	0.45	0	4	2e+02	1	CCTAEETCQVLEK + 2 Carbamidomethyl (C)
☑	1893	1041.03	2080.05	2080.09	-0.04	1	4	2.5e+02	1	DVDQHIVATISEIDRTLR
☑	2210	1210.71	3629.12	3629.69	-0.58	0	4	1.5e+02	1	ATNVIAGQLTLADLDQTPANVPYFEHDNEGECK + Carbamidomethyl (C)
☑	966	987.83	986.83	986.54	0.29	1	4	6e+02	1	KGVAADEVAK
☑	1438	1265.09	3792.24	3792.71	-0.47	2	4	6.6e+02	1	TQCSQPACKHEPMQVIGQVWMDYAECRLQQR + 2 Carbamidomethyl (C); Oxidation (M
☑	199	592.60	1183.19	1182.63	0.56	2	4	6.3e+02	1	KEEFLDKFK
☑	1986	1148.69	2295.37	2295.08	0.29	1	4	1.9e+02	1	EHAEGQRTGCVSGSVSTHAGVAR
☑	2148	1001.63	3001.86	3002.28	-0.42	1	4	2.1e+02	1	FRPVACGACDADVHCFAAATRAMSGSCR + 2 Carbamidomethyl (C); Oxidation (M)
☑	763	898.12	2691.34	2691.35	-0.00	1	4	5.9e+02	1	VFKSYLEPTPQNYIVNLDQMHR
☑	114	513.71	1025.41	1025.53	-0.12	0	4	3.9e+02	1	NILHVACEK
☑	2118	911.04	2730.10	2730.26	-0.16	2	4	1.6e+02	1	HHHHHHSSGLVPRGSHMRNYCTK + Oxidation (M)
☑	1394	1217.35	1216.34	1216.68	-0.33	1	4	6.3e+02	1	SRSLLEQTVGK
☑	1316	1177.72	2353.43	2353.08	0.35	0	4	5.8e+02	1	AEPNYSVLSAEMNASIAACPMK + Carbamidomethyl (C)
☑	1672	540.23	1617.67	1617.73	-0.07	1	4	1.8e+02	1	AKMATMGAASSSTSSSK + Oxidation (M)
☑	1296	1164.43	1163.42	1163.60	-0.18	1	4	6.5e+02	1	NISCTISKNK + Carbamidomethyl (C)
☑	1558	1399.55	2797.09	2797.37	-0.29	2	4	5.1e+02	1	RQQLETGITTLGVDCVFAGTNDMKK + Carbamidomethyl (C); Oxidation (M)
☑	363	683.64	1365.26	1365.72	-0.46	1	4	6.1e+02	1	LSLSRFVGQMGR + Oxidation (M)
☑	663	853.77	852.76	852.36	0.40	0	4	1.8e+02	1	EFDEASR
☑	2131	958.23	2871.67	2871.14	0.53	0	4	1.8e+02	1	NNNNNNNTSSGNNNNNAHSNNNNNMR
☑	109	509.85	1017.68	1017.55	0.14	1	4	4.4e+02	1	VNISSNKEK
☑	706	873.16	872.16	872.47	-0.31	1	4	7.6e+02	1	GEPSTVKR
☑	1049	1026.70	2051.40	2051.00	0.39	2	4	6.4e+02	1	MESSMNSQLFHKSTRLR
☑	1784	615.93	1844.77	1844.95	-0.18	2	4	2.1e+02	1	AMLRTREEIQLAQDR + Oxidation (M)

1984	763.08	2286.22	2286.21	0.02	2	4	2.1e+02	1	REPRVALIVVQTGYSPGWCR
136	531.45	530.45	530.27	0.18	0	4	1.1e+02	1	ADPTK
874	949.31	948.30	948.44	-0.14	1	4	2.1e+02	1	EMAASERR
510	769.89	1537.77	1537.71	0.06	1	4	7.3e+02	1	DKDTSPLTCMSVGK + Carbamidomethyl (C)
2077	855.80	2564.38	2564.25	0.12	2	4	1.9e+02	1	AFKEAAAGCVTAEEEAAQLDKTVR + Carbamidomethyl (C)
2042	823.38	2467.12	2467.17	-0.05	0	4	1.9e+02	1	ALMNHDIEYYDQTETGVIIAR + Oxidation (M)
783	907.43	906.43	906.48	-0.05	0	4	5.7e+02	1	AFAELQTK
637	839.66	2515.95	2515.43	0.52	1	4	7.4e+02	1	VRAIMMIVFLLPLLPLSCCVLR + Oxidation (M)
2065	842.51	2524.52	2525.06	-0.53	0	4	1.9e+02	1	MWHPDAMQAGMHGWSPAQGWGR + 2 Oxidation (M)
1165	1089.83	1088.82	1088.54	0.28	2	4	6.8e+02	1	KDSQPRACK + Carbamidomethyl (C)
1328	1182.80	3545.38	3545.62	-0.24	1	4	6.2e+02	1	SNDMIDTPPGFGFNPNSPPGFEKIPDAPPGFDR + Oxidation (M)
1725	860.53	1719.04	1718.64	0.40	0	4	2.1e+02	1	NSPDCGCSSDFFNGR + 2 Carbamidomethyl (C)
542	792.27	791.26	791.34	-0.08	0	4	2.4e+02	1	EAAGDSSR
1079	1039.28	3114.81	3115.40	-0.59	0	4	7e+02	1	MMFDIFLSNCSYIESTSWADPTTVLR + Carbamidomethyl (C); 2 Oxidation (M)
1936	724.44	2170.30	2170.17	0.12	0	4	1.8e+02	1	DQAQKPIDAPAVPAAPVDVLR
486	758.84	757.84	758.41	-0.57	0	4	4.1e+02	1	VVMPSAR
1273	1151.00	3449.97	3449.67	0.29	1	4	6.6e+02	1	EAKLMAEVAAQQQMFLASQALSASCLHSSGSAK + Carbamidomethyl (C)
1505	1321.07	3960.19	3959.89	0.29	2	4	6.3e+02	1	HGIMVVGPAMCGKTQCYDVMTETLSRTTVPHQQLR + Carbamidomethyl (C); Oxidation
1676	812.81	1623.60	1623.78	-0.19	0	4	2e+02	1	AHDGIMIWAAGGNPSK
1836	982.58	1963.14	1963.00	0.14	1	4	2.3e+02	1	YFLYAFHKNTYLIDR
1879	678.14	2031.40	2031.10	0.29	2	4	2.8e+02	1	LNIKYNINIINCRIDR + Carbamidomethyl (C)
214	601.92	1802.73	1802.84	-0.11	1	4	9.7e+02	1	CKLDLQSFTFACQSR + Carbamidomethyl (C)
2173	1054.56	3160.66	3160.68	-0.02	1	4	1.5e+02	1	IVAAEESILFILNDIQNIYRNALSNEAK
221	604.05	1809.14	1808.90	0.24	2	4	9.1e+02	1	KTKNLSMSNFFFSMK
1020	1011.03	2020.04	2020.07	-0.03	2	4	7.6e+02	1	GTLAPCRTQPLRQVCLPPA
1192	1103.59	3307.76	3307.69	0.07	1	4	5.5e+02	1	GTSIVIICVTSALFEPMSAVGRTATTPASSQR + Carbamidomethyl (C)
2083	1287.67	2573.33	2573.25	0.08	0	4	1.7e+02	1	STIPPHTIELVDADGDPYYTLEK
2209	1190.06	3567.15	3566.75	0.40	1	4	1.9e+02	1	VDVVARSYTLQHVLMCSANMTGMLSPLSPFAR + Carbamidomethyl (C); Oxidation (M)
2177	1074.53	3220.56	3220.55	0.01	2	4	1.4e+02	1	DSSDGNVSAYNGTFSAKPGCVFISKIDKTR + Carbamidomethyl (C)
1446	1272.71	1271.70	1271.68	0.02	1	4	5.1e+02	1	NGLQMRVDALR
1880	678.15	2031.44	2031.07	0.36	0	4	3.2e+02	1	LIIGTHDVATQHGEAQTLK
1942	729.74	2186.19	2185.97	0.22	2	4	1.8e+02	1	CFQVQNDWSRKEMDCLK + Carbamidomethyl (C)
802	457.89	913.77	913.52	0.25	1	4	2.1e+02	1	VYRALHR
573	805.23	2412.67	2413.14	-0.47	0	4	8.1e+02	1	AIQYAVGFLCQMDPSHTELYK

☑	923	971.04	970.03	969.59	0.44	1	4	3.6e+02	1	GIKLTVPDK
☑	1148	1080.15	3237.44	3237.49	-0.05	1	4	6.6e+02	1	ALTFTGYVQPDDHCKLCSPAETNNCVAVK + 2 Carbamidomethyl (C)
☑	577	810.28	809.28	809.32	-0.04	0	4	2.2e+02	1	CCSANQK + Carbamidomethyl (C)
☑	2100	901.58	2701.71	2701.36	0.35	1	4	2.5e+02	1	LGTVQQELMCLTAVQQRTQQDVR + Carbamidomethyl (C)
☑	1055	1030.18	3087.52	3087.57	-0.05	2	3	7e+02	1	FQNEIFPLIREGHSITLLCTEEEGKK + Carbamidomethyl (C)
☑	1389	1214.17	3639.48	3638.91	0.56	1	3	7.5e+02	1	DTPGCSQISFAALRPGAFVEWLHGLLASLRTLR + Carbamidomethyl (C)
☑	2181	1082.46	3244.37	3244.50	-0.14	2	3	1.6e+02	1	FFGNLMDAGRLSLCGEESFGTGSNHIREK + Carbamidomethyl (C); Oxidation (M)
☑	2016	795.42	2383.24	2383.05	0.19	2	3	2.1e+02	1	CAGSKETCTDCLAGYYKTIDNK
☑	287	637.73	1910.18	1910.10	0.08	1	3	7e+02	1	LGIAMHLILLDSLTTKR + Oxidation (M)
☑	439	733.20	1464.39	1464.64	-0.25	0	3	9.7e+02	1	AAAVAWACCACGVR + 2 Carbamidomethyl (C)
☑	1127	1068.06	3201.16	3201.32	-0.16	2	3	7.9e+02	1	YADKCYNFCSEAADNLYQSGSICYKCDK
☑	359	682.55	681.55	681.27	0.27	0	3	1.5e+02	1	CPFCR + Carbamidomethyl (C)
☑	809	919.57	1837.12	1837.00	0.13	2	3	6.8e+02	1	TAGKRAILCGILPYMSK + Oxidation (M)
☑	825	924.30	2769.89	2770.29	-0.40	1	3	7.7e+02	1	YAEMGYWDANYTIKDTDVLALFR + Oxidation (M)
☑	1707	846.50	1690.99	1690.85	0.14	0	3	2.1e+02	1	LASTITSIDDFAFYK
☑	812	920.33	2757.97	2758.36	-0.39	2	3	7.4e+02	1	RCEDFSLVASVGWEMVKFLVMQK + Carbamidomethyl (C)
☑	1975	761.61	2281.82	2282.24	-0.43	2	3	2.2e+02	1	CSHQRLGRATVFAGIADILVR
☑	1216	1114.67	1113.67	1113.56	0.10	0	3	6.3e+02	1	FVSMLAFQR + Oxidation (M)
☑	2117	910.45	2728.32	2728.26	0.06	1	3	1.8e+02	1	MAMMMAGRVLLVCALCVLWCGDGGR + Carbamidomethyl (C); Oxidation (M)
☑	2138	985.44	2953.31	2953.21	0.10	2	3	1.7e+02	1	RHGSPPNRPPCCCEKQCTCTYCSR + 4 Carbamidomethyl (C)
☑	552	795.23	2382.67	2383.15	-0.48	1	3	8.8e+02	1	GYIIYDAALACRQTPACLPCK + 2 Carbamidomethyl (C)
☑	2041	820.89	2459.65	2459.08	0.57	2	3	3e+02	1	FMFMCDTRRTGAISIESMMK + Carbamidomethyl (C); 3 Oxidation (M)
☑	875	949.40	2845.17	2845.38	-0.21	1	3	6.5e+02	1	AIDCFMLLARDFGSDGFASNVIQIDK
☑	899	962.73	1923.45	1922.85	0.59	0	3	8.3e+02	1	MNDEVQALVIDNGSGMCK
☑	685	864.12	1726.23	1725.94	0.29	1	3	8.1e+02	1	ISENLEARIQDAIVR
☑	1279	576.77	1151.52	1151.51	0.01	1	3	2.3e+02	1	MSEEDERIK + Oxidation (M)
☑	1537	684.44	1366.86	1366.71	0.15	1	3	2.1e+02	1	GCQQKVVHQAAAK
☑	1727	870.08	1738.15	1737.84	0.31	1	3	2.1e+02	1	NTHASDATPQLNADRK
☑	453	741.58	2221.71	2222.05	-0.34	2	3	8.4e+02	1	TSCVPCTKKTCPPPPPPPCR + 2 Carbamidomethyl (C)
☑	1571	710.84	1419.67	1419.76	-0.09	2	3	2.2e+02	1	RNRDIYEVLSR
☑	1149	1081.89	3242.66	3242.61	0.05	1	3	6.3e+02	1	EMESCDCPQGLRITHSLGGGTGSGLGTLILIK
☑	1157	1086.17	3255.50	3255.59	-0.09	1	3	7.4e+02	1	MGDSAVASTWEKAPSVMGLGSAQSAPFVVFTK
☑	472	752.74	751.73	752.28	-0.55	0	3	6.7e+02	1	AEDCMK + Carbamidomethyl (C)
☑	539	791.63	2371.87	2372.25	-0.38	2	3	9.5e+02	1	KIELRPAAVTETSSLPMPRYN

☑	910	966.26	1930.51	1930.00	0.50	0	3	8.4e+02	1	GLQTLPDSADYLALGATPK
☑	2184	1088.94	3263.79	3263.68	0.11	2	3	1.6e+02	1	FDKPCNEKTIQMSIIFEVMSIVIKHEK + Carbamidomethyl (C)
☑	2198	1141.06	3420.16	3420.61	-0.46	2	3	2.8e+02	1	SAFYKFTLKQNMPIEISTCNTYTNYNTR + Carbamidomethyl (C); Oxidation (M)
☑	2103	902.46	2704.37	2704.37	-0.00	2	3	1.9e+02	1	IELLFSAIGQAMRLMMTGNFSKAK + 3 Oxidation (M)
☑	1772	916.60	1831.18	1830.79	0.39	0	3	2.3e+02	1	VTERPTMSEMAMSSSR + 2 Oxidation (M)
☑	1513	1340.27	2678.52	2678.28	0.24	1	3	7.1e+02	1	VSAQEGWCRPEIVAPGAGCAARHEK + Carbamidomethyl (C)
☑	604	822.90	1643.78	1643.82	-0.04	0	3	1e+03	1	MLQLGETPTADEALR
☑	1070	1034.62	3100.85	3100.46	0.39	1	3	7.3e+02	1	EPVVVAGCAEQYTHGSGCGVGPSWTQGRLR
☑	441	734.32	733.31	733.41	-0.10	0	3	2.4e+02	1	EIVAFR
☑	1865	672.95	2015.83	2015.89	-0.06	0	3	2.3e+02	1	AGAALTDGTGGGGGGSGETGSSHR
☑	735	883.48	882.47	882.53	-0.06	1	3	5.9e+02	1	SVAARVLPA
☑	1069	1034.60	3100.76	3100.53	0.23	1	3	6.9e+02	1	VCSLRNPALIYQWAYIHTTGDATGHTR + Carbamidomethyl (C)
☑	514	773.70	2318.08	2318.02	0.06	0	3	8.9e+02	1	SQAVAADAVDGACHSISNESSSR + Carbamidomethyl (C)
☑	789	909.17	2724.49	2724.31	0.18	2	3	7.6e+02	1	KCFLIDNASMFSSLDSGMARAFLK + Carbamidomethyl (C); Oxidation (M)
☑	2202	1155.62	3463.83	3463.71	0.12	1	3	1.6e+02	1	EVTIPPFYSQHLGAVWQPFVERDWPYFR
☑	904	482.60	963.18	962.58	0.60	1	3	2.8e+02	1	DKIIYALK
☑	578	810.51	809.50	809.35	0.15	0	3	2.5e+02	1	MDFQNR
☑	2175	1057.98	3170.92	3171.50	-0.58	2	3	2.3e+02	1	GHEDKVYCVKYNATGELLVSASCDTTVR + 2 Carbamidomethyl (C)
☑	859	940.44	2818.31	2818.35	-0.04	2	3	6.7e+02	1	MDCKTVNLYTTSMSANELAACLRVK + Carbamidomethyl (C)
☑	908	965.40	2893.18	2893.33	-0.15	2	3	6.9e+02	1	WTLLCGDGKNTEHSSTWETQKDTTR
☑	1003	502.27	1002.52	1002.55	-0.02	2	3	3.5e+02	1	DNKETLKR
☑	1701	841.91	1681.80	1681.88	-0.08	2	3	2.2e+02	1	MESQTKIGKYNVIR + Oxidation (M)
☑	321	654.70	1961.08	1961.15	-0.06	1	3	6.4e+02	1	APAFLPALPLLSSARVGGPK
☑	325	657.32	1968.94	1969.07	-0.13	1	3	7.5e+02	1	TVRPFQFIKFESTLEK
☑	632	837.56	2509.67	2509.39	0.28	2	3	8.7e+02	1	YVFEVPRHTLRPAVAAALKESR
☑	1560	702.46	1402.90	1402.81	0.09	2	3	2.9e+02	1	FMRDPIRILVK + Oxidation (M)
☑	762	897.63	2689.86	2690.44	-0.57	1	3	8.5e+02	1	QIEIILYPSMKPKFINEGDVNVK + Oxidation (M)
☑	482	757.15	1512.30	1511.73	0.57	1	3	9.2e+02	1	EVEGQICSTSFKGK
☑	712	874.71	873.70	873.46	0.25	0	3	3.7e+02	1	DSSLSPLR
☑	955	982.23	2943.66	2943.55	0.11	2	3	8.5e+02	1	VSLPVSGRLVTPPRMQFGRPTSGFDAR + Oxidation (M)
☑	1451	638.78	1275.54	1275.71	-0.16	0	3	3.3e+02	1	LTLDQIDAFLK
☑	695	869.61	868.60	868.41	0.19	0	3	1.9e+02	1	GFDFVER
☑	2025	802.72	2405.14	2405.16	-0.03	0	3	2.1e+02	1	LADDYLSTNPGSSFSYLVSLEK
☑	457	743.89	2228.65	2229.07	-0.42	1	3	1.1e+03	1	CANAAMVTFVERYTWPVAK + Carbamidomethyl (C); Oxidation (M)

1006	1004.58	2007.15	2007.03	0.11	2	3	7.2e+02	1	MMRFCARAGILLGAPSAAR + Oxidation (M)
1290	1159.70	3476.08	3475.61	0.46	1	3	7.5e+02	1	IFLEVWGAQGGKSTAYSYCTANGGFGGYSSGVNK
1658	794.64	1587.26	1586.83	0.43	1	3	4.8e+02	1	AQVSRAMGLQQQVR + Oxidation (M)
1745	592.01	1773.01	1772.89	0.13	1	3	2.7e+02	1	DTCRILGSVHEYVPK + Carbamidomethyl (C)
692	868.14	1734.27	1734.87	-0.60	1	3	8e+02	1	VCAPRELFPSATTTSR
2048	828.46	2482.37	2482.27	0.10	2	3	2.2e+02	1	TFKECEEIARAIGSAFYHIGLK
855	939.53	938.52	938.46	0.06	1	3	2.4e+02	1	ATDFMAKR
1826	972.60	1943.19	1943.02	0.16	1	3	2.7e+02	1	KADAAGAQDLLDDVTSLLK
656	850.21	849.20	849.48	-0.28	0	3	2.6e+02	1	IGIGFCLK
461	746.03	745.02	745.43	-0.41	1	3	5e+02	1	TALKDAK
1526	676.95	1351.89	1351.78	0.11	0	3	2.6e+02	1	QLPATLLSPLGSR
535	788.95	787.94	787.40	0.54	2	3	6.3e+02	1	RDGRER
1096	1049.17	3144.49	3144.40	0.09	2	3	8.6e+02	1	QGSSSGGGMQRQGSLAGGGMQRQGSFAGGGMSR + Oxidation (M)
1118	1062.48	3184.41	3184.62	-0.22	2	3	7.2e+02	1	RLTQGCFSFLPDLTDAQIKAQIDYAVSK + Carbamidomethyl (C)
1408	1234.04	3699.09	3698.88	0.21	1	3	8.1e+02	1	HPFYSAVLLLLLVVMMCCGTGVAHAEVQPSQKK + 2 Carbamidomethyl (C); Oxidation
2017	1192.63	2383.25	2383.21	0.04	1	3	2.4e+02	1	LQMVAYFVGGSTTYVFDKTIK + Oxidation (M)
2187	1095.87	3284.59	3284.66	-0.07	2	3	1.8e+02	1	EDSLLRAAILCERYGLYSTIEVDGVDTVK + Carbamidomethyl (C)
541	791.98	2372.91	2373.19	-0.28	1	3	1.1e+03	1	LEAWCANCDAQITAIQRILK + 2 Carbamidomethyl (C)
1448	638.25	1274.49	1274.65	-0.17	1	3	2.7e+02	1	EVTGKDYPAPAK
1738	584.83	1751.46	1751.86	-0.40	1	3	3.7e+02	1	RVTLEHVMEEPQER
2161	1027.09	3078.24	3078.47	-0.23	2	3	2.3e+02	1	GDFFFMSTYRFLFAIWLRNHGYMK + 2 Oxidation (M)
307	649.91	1946.72	1946.93	-0.21	2	3	1e+03	1	IARESLGTDDMEKEEPK
1602	736.48	1470.95	1470.62	0.33	0	3	2.6e+02	1	EHCGGKPDDDVSR + Carbamidomethyl (C)
1838	655.82	1964.45	1964.04	0.41	1	3	5.2e+02	1	ENELVFFVKAAAADVVSR
1773	611.79	1832.34	1832.08	0.26	2	3	4.3e+02	1	IGLRYAIPGIRELHPK
401	710.70	709.69	710.25	-0.55	1	3	2.1e+02	1	CRCGTD + Carbamidomethyl (C)
499	763.48	2287.41	2287.11	0.30	2	3	8.3e+02	1	LGRHPEKVVCGAAMLPSCTMR + 2 Oxidation (M)
134	530.88	529.87	530.31	-0.43	0	3	2.2e+02	1	TLLNA
1896	699.41	2095.21	2095.00	0.20	1	3	2.5e+02	1	YIIMNDQIDMFKEYIK + 2 Oxidation (M)
953	491.11	980.21	980.53	-0.32	0	3	2.5e+02	1	IPAYFVSGK
631	836.77	2507.29	2507.29	-0.01	2	3	8.8e+02	1	CLLQLVDDFEGITESTEKLARK
1128	1068.43	3202.27	3202.54	-0.26	0	3	7.1e+02	1	ADGALAAIDGSGTNTPAASPLPSLVANMFGCAAR + Oxidation (M)
1271	1150.32	2298.63	2298.11	0.52	2	3	8.5e+02	1	TMLESFEACVNRVLNSARDK + Oxidation (M)
404	712.57	1423.12	1422.69	0.43	0	3	9.1e+02	1	DEDSTELLNFIK

☑	1057	1030.96	3089.86	3089.50	0.36	1	3	9.8e+02	1	TTYRLLCALLVFALCCCPSVCVTANGNK + 2 Carbamidomethyl (C)
☑	1816	641.09	1920.26	1919.94	0.32	1	3	2.6e+02	1	GSNVNTSRLLGICTDGEK + Carbamidomethyl (C)
☑	419	720.24	719.24	719.42	-0.19	0	3	2.9e+02	1	IEIFAK
☑	2045	825.51	2473.51	2473.33	0.18	1	3	2.6e+02	1	YSVKAIIGIVGMLVFGTGTMVSSK + Oxidation (M)
☑	1226	1118.06	3351.15	3351.63	-0.48	1	3	1.1e+03	1	GRTSPMGVLGIESFPTSGDADDPLSGFVASQLK + Oxidation (M)
☑	1559	1401.68	2801.34	2801.43	-0.10	1	3	6.3e+02	1	AAEATLDLAAQANTFLSEQAPWSRIK
☑	531	787.01	1572.01	1571.78	0.23	0	3	1.1e+03	1	QQVAEDVEEVASLR
☑	1923	1078.81	2155.61	2156.11	-0.50	0	3	4.4e+02	1	DAVSAASPMVPAVSVTGGGVGTVK
☑	2088	868.52	2602.53	2602.37	0.16	2	3	2.4e+02	1	ATAPLFGAPTTTTPGSAAAAATTSKKGR
☑	1622	755.88	1509.75	1509.85	-0.10	2	3	2.7e+02	1	LFLFKERMSAIR
☑	993	999.06	2994.16	2994.36	-0.20	1	3	1e+03	1	DGISICRNASTVVMCLEDVCDFTVYK + 2 Carbamidomethyl (C)
☑	688	865.83	2594.47	2594.35	0.12	1	3	8.8e+02	1	QYLGQVAGAEVAANTRILYGGSASAK
☑	311	651.16	1950.45	1950.99	-0.55	1	3	1.2e+03	1	MSTQEEPPQHTRLLLR + Oxidation (M)
☑	297	643.54	1285.08	1285.61	-0.53	0	2	1e+03	1	EFEIAAANHER
☑	533	788.04	2361.09	2360.99	0.10	1	2	1.1e+03	1	MCPSKSDVESASPENACSSTFK + Carbamidomethyl (C)
☑	2073	850.85	2549.52	2549.46	0.06	1	2	2.4e+02	1	QIYKTLLLIEHLLINASPDTVR
☑	1791	621.32	1860.93	1861.02	-0.10	0	2	2.7e+02	1	LTALNVRPSVLTMQYR
☑	46	355.28	1062.83	1062.48	0.35	0	2	5e+02	1	MDDWELVR
☑	574	806.12	2415.34	2415.21	0.14	0	2	9.8e+02	1	GYATLMSEMLALGFSGAELVEVK
☑	897	961.11	2880.31	2880.30	0.01	2	2	9.9e+02	1	LKACDFCTFASSEDGEQLAKSMIEK + 2 Carbamidomethyl (C); Oxidation (M)
☑	1860	1003.51	2005.01	2005.00	0.01	0	2	2.4e+02	1	MNCTQLFDLVAELNIPK + Carbamidomethyl (C)
☑	2116	907.41	2719.21	2719.03	0.18	1	2	2.4e+02	1	SFMNMAAFEESDDDKCMNGGIMR + Carbamidomethyl (C); 4 Oxidation (M)
☑	1202	555.26	1108.51	1108.61	-0.10	2	2	2.5e+02	1	GKLPGDPNRR
☑	1906	703.05	2106.12	2106.14	-0.02	2	2	2.6e+02	1	EICLLSEPKYRHIIHAK + Carbamidomethyl (C)
☑	603	821.84	820.83	820.42	0.41	0	2	1e+03	1	EAHSIHK
☑	1262	1145.61	3433.80	3433.74	0.06	1	2	7.5e+02	1	DIKPQNILLTSDGDVKLSDFGASIVVDNSAMR + Oxidation (M)
☑	251	618.82	1853.43	1853.87	-0.44	0	2	1.5e+03	1	SYSLLMCCGLFVFICR
☑	885	956.71	955.70	955.56	0.14	1	2	2.5e+02	1	AVNLERVR
☑	1025	1014.03	3039.07	3039.57	-0.49	2	2	1.1e+03	1	KCIYNEGIRAQYGGVIFLGVVSNPSMPK
☑	1890	689.92	2066.73	2066.98	-0.25	0	2	3.1e+02	1	STSLVEEVELQCEEFLR + Carbamidomethyl (C)
☑	403	711.28	2130.81	2131.04	-0.23	1	2	7.4e+02	1	FLFAPVGSNICKCIFMADR
☑	828	925.52	2773.54	2773.31	0.23	0	2	7.4e+02	1	LSCPSPSGIPSPSFWHSESFLPTEK + Carbamidomethyl (C)
☑	803	915.05	2742.14	2742.22	-0.09	1	2	1e+03	1	SHASGYSGGLRSSSMSGCHTAAGIYNGK
☑	1983	1144.06	2286.10	2286.16	-0.06	1	2	2.9e+02	1	IPMILSCGEYKQTYLLTEK + Carbamidomethyl (C)

☑	1146	1079.82	2157.63	2158.16	-0.53	2	2	9e+02	1	KFNLSPPKGVLLYGPPGCSK + Carbamidomethyl (C)
☑	787	908.70	1815.39	1815.93	-0.53	1	2	9.6e+02	1	CSTAVLARGTAPQNATAK + Carbamidomethyl (C)
☑	2160	1026.11	3075.29	3075.57	-0.27	2	2	2e+02	1	MLGCRDLLWGDGTAAQTGLPLKMSLSLR + Carbamidomethyl (C); Oxidation (M)
☑	309	650.50	649.49	649.38	0.11	1	2	1.1e+03	1	KALGYV
☑	614	828.84	2483.49	2483.31	0.19	2	2	8.8e+02	1	MLATFVLATSMIRMIGSQGSKNK
☑	555	796.69	795.68	795.41	0.28	1	2	2.3e+02	1	SKMFQR
☑	801	914.22	2739.64	2739.31	0.33	1	2	1e+03	1	LHLVERATGQSESEGQATQSEPETR
☑	1078	1038.73	3113.16	3113.49	-0.33	0	2	9.6e+02	1	YGDEYFATNFMVVENVVGTLLGMPFLR + 2 Oxidation (M)
☑	111	512.09	1533.23	1532.66	0.57	1	2	8.5e+02	1	CAYCQASNFRTR + 2 Carbamidomethyl (C)
☑	2058	1255.67	2509.32	2509.22	0.10	2	2	3.1e+02	1	ARTSSPCIVFFDELDALCPRR + 2 Carbamidomethyl (C)
☑	1036	1019.61	3055.80	3055.42	0.38	2	2	9.2e+02	1	EVVQTMLIACCGEPGGGKNEMTPRFTSK + Carbamidomethyl (C); Oxidation (M)
☑	2090	876.22	2625.63	2625.38	0.24	2	2	2.8e+02	1	YVNNVGGRKITAWVFMQLSSLSR
☑	2110	905.17	2712.49	2712.20	0.29	2	2	2.2e+02	1	CIKGLLEPKCNPYPNTDGSCTDCK + 2 Carbamidomethyl (C)
☑	209	599.44	1196.87	1196.61	0.26	0	2	8.5e+02	1	VDALFTEFAGK
☑	1024	1013.70	3038.08	3038.40	-0.32	0	2	9.5e+02	1	DLGMPIIMHDYITGGFTSNTSLAYYCR
☑	1830	975.71	1949.41	1948.99	0.42	0	2	4.7e+02	1	TSIVEYLADITGHEFVR
☑	1040	1023.16	2044.31	2043.99	0.32	2	2	8.1e+02	1	KTYPRYILMDGDLNSDK + Oxidation (M)
☑	1937	725.56	2173.66	2174.22	-0.56	1	2	4.1e+02	1	SIYQSVAASLLTQLLRQQR
☑	1221	1116.72	3347.13	3346.90	0.23	2	2	9.6e+02	1	MITTVLASKTEILLNILELMSNLLNIRFK + Oxidation (M)
☑	1385	1211.02	3630.04	3630.57	-0.53	2	2	8.1e+02	1	AMRVSAKTEAEATQDCDENAADAEEPSDFLTSR + Carbamidomethyl (C); Oxidation (M
☑	1732	872.54	1743.06	1742.94	0.12	0	2	2.8e+02	1	ILNLNATSEQVAITEK
☑	766	898.70	2693.07	2693.50	-0.43	2	2	1e+03	1	LRLLLAERCATQLLVASGAPLSDAR + Carbamidomethyl (C)
☑	1866	673.22	2016.63	2017.01	-0.39	1	2	4.2e+02	1	FLHAAVEGRPCQFGVKCR
☑	659	852.06	2553.17	2553.30	-0.13	1	2	9.7e+02	1	LTLELGGQPFQGRVPNFFDQYK
☑	1817	641.45	1921.32	1920.98	0.34	1	2	3.4e+02	1	NKSPPETIAVHCVSGLGR + Carbamidomethyl (C)
☑	1908	706.03	2115.08	2115.12	-0.04	2	2	2.6e+02	1	LVAETVTEAGGAPVETMKGKK
☑	2123	926.97	2777.87	2777.34	0.54	1	2	3.8e+02	1	MFTQLEGGYIAVIQKYPYVDGDDR
☑	705	873.04	2616.10	2616.32	-0.21	1	2	1.2e+03	1	CVSIHTDRPTTKLGQLSTTMGWK + Carbamidomethyl (C)
☑	1234	1122.40	1121.40	1121.50	-0.10	0	2	9.5e+02	1	ACAGVPGSTMR + Carbamidomethyl (C); Oxidation (M)
☑	1432	629.38	1256.74	1256.66	0.08	2	2	2.9e+02	1	RTPSQRAAADGK
☑	2013	1188.49	2374.97	2375.20	-0.23	0	2	2.8e+02	1	GWYAGAVGALHFDGSLNTGLTLR
☑	1688	829.93	1657.86	1657.80	0.06	0	2	3e+02	1	ESTPLDAECTLPIGR + Carbamidomethyl (C)
☑	1312	1173.56	3517.67	3517.48	0.19	1	2	7.8e+02	1	EMLTMCSDAHDSLDNGSGVGGGAMSASAGTGTAARK + Carbamidomethyl (C); 3 Oxidati
☑	2153	1016.96	3047.85	3048.34	-0.49	2	2	3.5e+02	1	EGSYNASPQALCGLAMCAMASKDSKEAQR + 2 Oxidation (M)

☑	507	767.46	766.45	766.39	0.06	1	2	8e+02	1	AVCRYR
☑	525	784.99	2351.95	2352.21	-0.27	2	2	9.6e+02	1	DIMRIVDALFEWGKTSLTNK + Oxidation (M)
☑	1472	1295.67	3883.98	3883.66	0.33	1	2	7.2e+02	1	HEQVVHCGEAYMEMFALEVMMDETQACTDPRAR + Carbamidomethyl (C)
☑	202	594.45	1186.89	1186.70	0.19	2	2	9.7e+02	1	IISGGRDKTIK
☑	1037	1020.21	3057.61	3057.34	0.27	1	2	9.9e+02	1	RLCGHAVLCQDDGTWSAEAGSSDTHAASR + Carbamidomethyl (C)
☑	1131	1070.09	3207.26	3207.62	-0.36	2	2	1e+03	1	REFLTHVPHPTANASAPAMHEHLLTMRK + Oxidation (M)
☑	2124	929.51	2785.50	2785.38	0.12	0	2	2.5e+02	1	FHVDYQSVAGPEALDPVESLVESVAK
☑	476	755.39	754.39	754.43	-0.05	0	2	7.8e+02	1	TPKPAGGK
☑	1413	1236.05	1235.04	1235.62	-0.58	0	2	9.5e+02	1	VLENENFMLK
☑	94	486.95	971.88	972.40	-0.52	0	2	7.9e+02	1	CSADEPHSK
☑	1514	670.87	1339.72	1339.70	0.02	0	2	2.8e+02	1	AIDYILDLYNK
☑	378	694.79	2081.34	2081.13	0.20	2	2	1.2e+03	1	LFSLRLTNEEIFCIAKK + Carbamidomethyl (C)
☑	240	613.79	1838.36	1838.70	-0.35	0	2	9.6e+02	1	SDDENDEATNETNETR
☑	687	865.10	1728.19	1727.95	0.23	2	2	9.2e+02	1	SVVTETEKLLRSPNR
☑	1400	1222.61	3664.82	3664.59	0.23	1	2	8.1e+02	1	SGSEVSMCGGSPTGSPQALSFPTKSEMSGDGSLSSSR + Carbamidomethyl (C); Oxidatio
☑	1910	708.89	2123.65	2123.13	0.53	1	2	4.2e+02	1	NQFPKLFTILSDIHGTHR
☑	1554	697.47	1392.92	1392.80	0.12	1	2	3e+02	1	AVSKHQKPMIVR
☑	1628	765.01	1528.00	1527.79	0.21	1	2	3.3e+02	1	SGKPADLNKDLENK
☑	1788	619.09	1854.24	1853.98	0.26	1	2	3.4e+02	1	QQQQPVIVLNQKMER + Oxidation (M)
☑	786	908.39	2722.15	2722.46	-0.30	0	2	8.3e+02	1	HQVSGAVFLPGVFIRPGESLSGAIER
☑	376	693.79	1385.57	1385.80	-0.23	1	2	1.2e+03	1	EIVKIQMGEVIK
☑	1014	1006.39	2010.77	2011.03	-0.25	0	2	9e+02	1	NQAIPFPQKPEANSPAFR
☑	1109	1056.36	3166.06	3165.53	0.52	2	2	1e+03	1	DWHALAPSAAIHLPMTLCGGQCFRWRR + Carbamidomethyl (C); Oxidation (M)
☑	1649	789.79	1577.56	1577.73	-0.17	2	2	3.1e+02	1	LPEKKCNEMGSNGR + Oxidation (M)
☑	987	997.34	1992.67	1993.04	-0.36	2	2	9.9e+02	1	KNVYCDLMKVGIIDPTK + Carbamidomethyl (C)
☑	437	732.23	731.22	731.33	-0.11	0	2	3.7e+02	1	DEENII
☑	1244	1133.10	3396.29	3396.75	-0.46	2	2	1.3e+03	1	LVLLGTGPSTCVPNIGCLLGLRGERSPCAVCK + 3 Carbamidomethyl (C)
☑	1224	1117.02	2232.03	2232.07	-0.04	1	2	1.2e+03	1	YQKAMCLIASGIEQDPYFR
☑	2046	826.14	2475.40	2475.93	-0.53	0	2	2.7e+02	1	GNCNQQPNPSTCSHNCDECPSK + 2 Carbamidomethyl (C)
☑	436	731.28	2190.83	2191.09	-0.25	1	2	1.1e+03	1	YDSMQASLLADELKYLGFK
☑	1060	1031.66	1030.65	1030.46	0.19	1	2	3.9e+02	1	KSYEEMTK + Oxidation (M)
☑	704	872.59	2614.75	2615.34	-0.58	1	2	1.1e+03	1	IVLLDNTETDKATSTSEALPIPCK + Carbamidomethyl (C)
☑	255	619.09	1854.26	1854.05	0.21	1	2	1.3e+03	1	ALTKAILDAEQNVLDIK
☑	920	970.37	2908.07	2908.42	-0.35	2	2	9.4e+02	1	NFADLMDAEYQSRRVGANISKPPSAR + Oxidation (M)

☑	861	941.61	940.60	940.55	0.05	0	2	2.6e+02	1	FVLFLFR
☑	1045	1025.08	2048.14	2048.12	0.02	2	2	1.1e+03	1	TASLIHASVAFYRTLRDK
☑	1522	675.39	1348.76	1348.78	-0.02	2	2	3.2e+02	1	HVSKTRHLLMK
☑	1178	1097.08	3288.23	3288.53	-0.31	1	2	1.1e+03	1	ETAEILISNGADINAKGGDGCTALHSAAMNNSK + Oxidation (M)
☑	1631	514.64	1540.91	1540.77	0.14	1	2	3.5e+02	1	RILDEVVNDPDEK
☑	1779	613.08	1836.23	1835.86	0.37	1	2	3.5e+02	1	NSASLYPTEGIKGDGGDR
☑	2188	1100.42	3298.25	3298.33	-0.08	0	2	3e+02	1	FSEQSLVDCVTSDYSCQGCSGGWPDQAMK + 3 Carbamidomethyl (C)
☑	1822	969.77	1937.52	1937.03	0.49	1	2	5.6e+02	1	DEKISQVFVDTLFQIR
☑	2037	814.85	2441.53	2441.27	0.26	2	2	3.2e+02	1	FELCEALTHPWMRQGALLAKK
☑	2132	964.35	2890.04	2889.57	0.47	2	2	3.8e+02	1	TLTQQELLHLQSKGINTKYIFSATR
☑	1134	1072.13	2142.25	2142.08	0.17	1	2	1.2e+03	1	KLEGPNVSLDLIENVPMCR + Oxidation (M)
☑	1445	1271.61	2541.20	2541.34	-0.15	1	2	7.5e+02	1	AIEQQLTGIYTKHATEDILAAQK
☑	2076	854.59	2560.76	2560.38	0.38	2	2	5e+02	1	NTALFGDLLRQGRQIDLLFENK
☑	296	643.04	1926.10	1926.00	0.11	0	2	1.2e+03	1	GSLSAYNAVTTVTRPFSR
☑	1814	960.26	1918.51	1918.03	0.48	1	2	5e+02	1	SAPHSIATFVSLEYLRK
☑	1840	656.41	1966.20	1966.03	0.16	2	2	3.5e+02	1	IYVDNMLSKNKYLTHK
☑	906	964.63	2890.87	2891.35	-0.48	1	2	1.1e+03	1	LSGLSLHRIGYEDMDELDEESEQVK
☑	1818	963.07	1924.12	1923.79	0.33	1	2	3e+02	1	CPLCDTFYCSVECKEK + Carbamidomethyl (C)
☑	997	999.91	2996.71	2996.61	0.10	1	2	9.4e+02	1	SQNPNPVIIQQAPSTPQPKDIFHLSIK
☑	1943	732.67	2194.99	2194.97	0.02	2	2	4.3e+02	1	RDCYRCCYYLLKPDDR + 2 Carbamidomethyl (C)
☑	2165	1034.91	3101.70	3101.65	0.06	2	2	2.6e+02	1	GCCNIQNGLDKLGSLPKLSVLLLSETTTK + Carbamidomethyl (C)
☑	1460	642.49	1282.96	1282.62	0.34	0	2	3.4e+02	1	ASSITFVDMQGK
☑	2105	903.77	2708.28	2708.40	-0.12	1	2	2.5e+02	1	VALGDTLMAADHFLLALHESKEVGR + Oxidation (M)
☑	1073	1035.92	1034.91	1034.46	0.45	1	2	1e+03	1	NSEEKSDAR
☑	1156	1085.54	3253.60	3253.54	0.06	1	2	8.9e+02	1	MSHIHHTYHGRAGSSITTLSCSSLAAQGSEK
☑	1348	1192.27	1191.26	1191.62	-0.36	1	2	1e+03	1	YGRSTIPEAAK
☑	2119	913.75	2738.24	2738.35	-0.11	2	2	2.5e+02	1	ESEYLVSIRSGPAEIPTMDKIMEK + Oxidation (M)
☑	1030	1016.53	3046.58	3046.51	0.07	2	2	9.7e+02	1	GQRRCVEALVAVYTQYTSSILQSMMR + Carbamidomethyl (C)
☑	1915	714.48	2140.41	2140.90	-0.49	0	2	3.7e+02	1	KPCGEGGCGGCAVAVAEYDNK + 2 Carbamidomethyl (C)
☑	797	912.68	911.67	911.46	0.21	0	2	2.6e+02	1	DVLAHVCR
☑	374	692.53	2074.58	2074.05	0.54	2	2	1.3e+03	1	MGGNEFLCASVKRDLLHK + Carbamidomethyl (C)
☑	642	840.48	1678.94	1678.88	0.06	1	2	8.7e+02	1	TCDIIRYTAGQLIR + Carbamidomethyl (C)
☑	1313	1173.58	3517.71	3517.72	-0.02	2	2	8.8e+02	1	RTRSGITDLPASFGTPAGTTSSGDSVLEHGVAAMR + Oxidation (M)
☑	1726	864.03	1726.05	1725.82	0.23	0	2	3.2e+02	1	NYLASEFAAVEEEVR

☑	2122	924.49	2770.43	2770.44	-0.01	2	2	2.7e+02	1	AFIIGTTSAYRVELAKEMMENLGVK
☑	964	985.82	2954.44	2954.52	-0.09	1	2	9.8e+02	1	ISNIEDKLHEATGNATISPIDAIEYLK
☑	508	768.96	2303.85	2304.22	-0.37	2	2	1e+03	1	NPSFAIGKTVLEVDNFLRER
☑	1097	1049.27	3144.78	3144.50	0.28	1	2	1.2e+03	1	AKLNIMGIVENMSGFVCPNCGSGSFIFPR + Carbamidomethyl (C)
☑	1821	967.47	1932.93	1932.99	-0.06	1	2	3.5e+02	1	GAPPGERASRPTTPQQQR
☑	1987	766.15	2295.43	2295.27	0.16	1	2	3.7e+02	1	QKLVVGQFSILNNAGQIQPGGK
☑	2055	835.60	2503.77	2503.45	0.32	2	2	5.3e+02	1	CLVAIKKVYSELLTAPALISLMK
☑	515	774.30	2319.89	2320.02	-0.13	2	2	1.1e+03	1	KCLEMFAELCEDNEKYNK + 2 Carbamidomethyl (C)
☑	769	899.48	898.47	898.39	0.09	0	2	8.9e+02	1	YTQACDAK
☑	2176	1066.97	3197.89	3198.47	-0.59	2	2	3.1e+02	1	NNGMRLNGTLGTSTTDPMDENGTLMRLNK + 3 Oxidation (M)
☑	496	762.27	761.26	761.40	-0.14	1	2	4.2e+02	1	KGSNASAK
☑	752	892.96	2675.85	2675.25	0.60	1	2	1.5e+03	1	LALLSFCSDGGQEVMDHRPRSER + Carbamidomethyl (C); Oxidation (M)
☑	1469	1291.27	3870.78	3870.90	-0.12	2	2	1.1e+03	1	ISLHNAANDTMRTSSAVREHPAAASPSSSLAVTAHGEK
☑	1276	576.46	1150.91	1150.74	0.17	0	1	4.4e+02	1	LILGALPVVTR
☑	1618	752.46	1502.91	1502.74	0.17	0	1	3.5e+02	1	EMALVDQVNALER + Oxidation (M)
☑	2032	812.03	2433.07	2433.11	-0.03	2	1	2.8e+02	1	CCGVCCSRLSHSYRWPPLPR + 2 Carbamidomethyl (C)
☑	1621	755.45	1508.88	1508.86	0.02	1	1	3.5e+02	1	NKIIHLSIDEVTK
☑	1211	1112.05	3333.13	3333.37	-0.24	1	1	1.4e+03	1	SGASGAHNGGSMAGAGGTMADQSRWPTCHFCHR + 2 Oxidation (M)
☑	1685	821.17	1640.32	1640.80	-0.48	0	1	6.5e+02	1	DTAAPAAAPAEQAAATSK
☑	921	970.76	969.75	969.56	0.19	0	1	3e+02	1	PVIGLGTWK
☑	428	724.31	2169.90	2170.02	-0.12	2	1	9.5e+02	1	SFPPSELMKFMHRCMGLK + 2 Oxidation (M)
☑	623	833.41	2497.22	2497.29	-0.07	2	1	1.1e+03	1	TCQIINKVIKFTKPEEDNSYK
☑	1101	1051.73	2101.44	2101.05	0.39	0	1	1.2e+03	1	LNTSLSSLVYGDIGAFVCSR
☑	814	922.07	2763.20	2763.18	0.02	0	1	1.2e+03	1	LPTEEDEEPVQDPMMMITTDDPSK + Oxidation (M)
☑	954	982.13	981.12	981.48	-0.36	1	1	4e+02	1	RFGGSGVMR + Oxidation (M)
☑	1145	540.06	1078.10	1078.52	-0.42	2	1	6.4e+02	1	TGRERMAVSG + Oxidation (M)
☑	372	690.90	689.89	689.31	0.58	0	1	1.7e+03	1	CHGAFR
☑	166	567.41	1699.20	1698.90	0.29	0	1	9.3e+02	1	TFTLVVMAIVHDEPK
☑	561	799.37	798.36	798.39	-0.03	0	1	3.1e+02	1	IYSSVCK
☑	1329	1183.05	3546.14	3546.73	-0.59	1	1	1.2e+03	1	TESASTATAAPNLMATSPMTSVKSVHSALATAEASR
☑	424	723.33	722.33	722.28	0.04	0	1	2.6e+02	1	CCGADVR
☑	1750	894.06	1786.10	1785.79	0.31	1	1	3.4e+02	1	EIQQSMMAMSKEMAR + Oxidation (M)
☑	198	591.90	590.89	591.24	-0.35	0	1	5.3e+02	1	CEAGR + Carbamidomethyl (C)
☑	230	608.31	607.31	607.30	0.01	0	1	3.2e+02	1	TGLSCK

☑	1048	1026.64	3076.91	3076.47	0.45	0	1	1.1e+03	1	LHEAGALSCEETVFIDAAVIFAMYFTR + Carbamidomethyl (C); Oxidation (M)
☑	1402	1226.83	2451.64	2451.93	-0.29	0	1	1.1e+03	1	YCQLAAFACADEFCHDCSADR + 2 Carbamidomethyl (C)
☑	1075	1036.80	3107.38	3107.48	-0.10	1	1	1.2e+03	1	CWIMGTEEVANGGAGSFIVGTTGGFVTYRK
☑	2127	937.37	2809.09	2809.28	-0.19	1	1	3.1e+02	1	EDHPVSDAMQAFSLMTNEMVTLRR + 2 Oxidation (M)
☑	1087	1043.01	2084.01	2084.12	-0.12	0	1	1.4e+03	1	LLLLFDGLGCAVLQNPWR + Carbamidomethyl (C)
☑	1783	923.13	1844.26	1843.92	0.33	1	1	4.7e+02	1	WLGQSLSRSTLYDYR
☑	1306	1167.36	3499.07	3499.62	-0.56	1	1	1.2e+03	1	LTCDYFLMHGSDIVADNMVSNGKYIFQYAK + Carbamidomethyl (C)
☑	1421	1249.64	3745.91	3745.84	0.07	2	1	9.5e+02	1	TLLAEETHSPPLTALFGLSGTPKVSSQDSSMCRNR + Oxidation (M)
☑	276	628.65	1255.28	1254.74	0.54	2	1	1.1e+03	1	DQLIKNIKQR
☑	1088	1043.77	1042.76	1042.57	0.19	1	1	4.3e+02	1	KYSSVVVYV
☑	1150	1082.13	1081.12	1081.52	-0.40	1	1	5.6e+02	1	EKVYVEGCR
☑	1229	1119.40	1118.39	1118.55	-0.16	0	1	1.2e+03	1	DVDSQTDIVK
☑	1411	1234.54	3700.59	3700.04	0.55	2	1	9.3e+02	1	QIENAVQKGQVVLLEDVGETLDAALDPVLLKQVR
☑	1270	1150.19	3447.54	3447.72	-0.18	1	1	1.1e+03	1	TLSSKNLSALHICAAFDSIDCFIYIQNYLK + Carbamidomethyl (C)
☑	876	949.53	2845.58	2845.52	0.06	2	1	1e+03	1	NYPHEVIEIPGVLIDVRTGQVDRAR
☑	959	983.55	2947.63	2947.50	0.14	2	1	1e+03	1	EDDVVLSNELTTTWERNVLINTKMK
☑	1051	1028.77	2055.53	2055.06	0.47	1	1	1.2e+03	1	LSELKQVNEQEVNELQR
☑	1637	775.47	1548.93	1548.76	0.17	1	1	3.6e+02	1	AMVEAAAMMNVGAKR
☑	742	888.13	887.12	886.53	0.59	1	1	5.9e+02	1	GRLATLTR
☑	1881	678.38	2032.13	2032.03	0.10	2	1	3.7e+02	1	SDKNGPSDTLSVVDIARMK
☑	1158	1087.72	1086.72	1086.69	0.03	2	1	1.2e+03	1	RTLILSKTR
☑	1337	1186.50	3556.47	3556.71	-0.24	2	1	9.9e+02	1	YYLVVYQYVDRFNSDMVGRAIQELNPGMGK + 2 Oxidation (M)
☑	653	848.48	2542.42	2542.20	0.22	1	1	1.1e+03	1	TVNECLGLSACKSSFPGIVTCSR + 3 Carbamidomethyl (C)
☑	1052	1029.20	2056.39	2056.06	0.33	1	1	1.2e+03	1	ACLDGALRGDAEVLTTLPGK + Carbamidomethyl (C)
☑	2178	1075.17	3222.50	3222.65	-0.15	1	1	2.6e+02	1	TGHGVQGGFVSAMIDGQKYILVSRPVYAEK + Oxidation (M)
☑	388	702.55	2104.62	2105.05	-0.43	1	1	1.5e+03	1	NHQFFHAVVMAHGSVPRR + Oxidation (M)
☑	1083	1041.10	2080.18	2080.05	0.13	2	1	1.4e+03	1	RNVVFAEALRCACIVMR + 2 Carbamidomethyl (C); Oxidation (M)
☑	1119	1064.65	2127.28	2127.06	0.23	1	1	1.2e+03	1	LNNSQQEQQTKQLPENTK
☑	901	962.78	1923.54	1922.97	0.56	0	1	1.3e+03	1	SIESGVSSFAVPLAQFER
☑	576	807.27	1612.53	1612.66	-0.14	1	1	1.5e+03	1	NGDKCVACDSSCLK + 3 Carbamidomethyl (C)
☑	975	991.91	990.90	990.48	0.42	0	1	6.3e+02	1	THYQDLSK
☑	315	653.95	1305.88	1305.65	0.23	2	1	1.1e+03	1	KGKNGTTGTEADK
☑	1309	1169.04	3504.09	3503.71	0.38	1	1	1.3e+03	1	VEICNFLVDLCANLSKQGAFCYVVCNNILK + 3 Carbamidomethyl (C)
☑	1130	1069.60	2137.18	2137.05	0.13	2	1	1e+03	1	NYYPGNFSVYVKSTDGAKK

☑	427	723.62	722.62	722.33	0.28	0	1	2.8e+02	1	AFNTDR
☑	1137	1072.91	2143.81	2144.07	-0.26	1	1	1.1e+03	1	HYPLGCAVFTLPPGMKNSR + Carbamidomethyl (C)
☑	1700	838.46	1674.90	1674.81	0.09	2	1	3.5e+02	1	LEGKWRGPVSEMDR + Oxidation (M)
☑	700	871.40	1740.78	1740.96	-0.18	0	1	1e+03	1	QTGELDDSILLILGVR
☑	710	874.47	2620.39	2620.39	0.00	2	1	1.3e+03	1	TNVKVLGRPTQPTGTFFLRSMDR
☑	1188	1101.27	2200.52	2200.02	0.50	1	1	1.2e+03	1	NMAGQGNSYSTICAKLCAVR + 2 Carbamidomethyl (C)
☑	1248	1135.68	3404.01	3403.61	0.40	2	1	1.1e+03	1	ALEQRQVIICCARMEGLLCTTEMEYNGLK + Carbamidomethyl (C); 2 Oxidation (M)
☑	1464	1289.57	2577.12	2577.33	-0.21	1	1	9.1e+02	1	DIFIQPCIDYSRIAIILNNDGK + Carbamidomethyl (C)
☑	883	955.54	1909.07	1908.82	0.25	1	1	9.7e+02	1	EGDGFPKGCPCGCDLLK + 3 Carbamidomethyl (C)
☑	2191	1117.47	3349.39	3349.75	-0.36	1	1	2.8e+02	1	AGDEIYYALNILYFVSLVLDPYIPEFGKK
☑	1067	1033.91	3098.71	3098.66	0.04	2	1	1.2e+03	1	VNKQLSGGASIGFPPRLTNEHIVPPSNAAK
☑	1863	1006.47	2010.92	2011.04	-0.12	1	1	3.6e+02	1	CLVELTIHASRDDELLK + Carbamidomethyl (C)
☑	2157	1021.02	3060.03	3060.50	-0.47	0	1	4.9e+02	1	DTAGVNSPPTTVASAPAPATPHFSALCEPVR
☑	1429	1255.05	3762.13	3761.88	0.25	2	1	1.2e+03	1	DNNGRTPLHLAASKGHLDVVQFLCCLPTINVCEK + Carbamidomethyl (C)
☑	2027	1209.86	2417.70	2417.17	0.52	2	1	6e+02	1	DESVHRPGAACARAPRLLHCCR
☑	776	904.54	1807.07	1807.03	0.04	2	1	1.3e+03	1	NLMYNELVKSKILVK + Oxidation (M)
☑	1213	1113.50	3337.48	3337.85	-0.36	2	1	1e+03	1	MSVRMLPFMLLLLLFRLSGFIEDVAAISR
☑	2084	1290.81	2579.60	2579.25	0.35	0	1	4.4e+02	1	ALPEMSCIVSVGSGVGYVEHIFNR + Oxidation (M)
☑	796	912.08	1822.15	1822.74	-0.59	1	1	1.2e+03	1	KGVFCESGGGECCSFTR + Carbamidomethyl (C)
☑	504	766.28	1530.55	1530.77	-0.22	0	1	1.2e+03	1	IENALECVQGLGTK + Carbamidomethyl (C)
☑	2152	1009.43	3025.27	3025.57	-0.30	2	1	3.3e+02	1	WFTACVATLSVAAFARGSALLRCVATAGR + Carbamidomethyl (C)
☑	1181	1098.24	2194.47	2195.02	-0.55	1	1	1.3e+03	1	TSDKFNCFVISLYMPMQR + Oxidation (M)
☑	170	571.03	570.02	570.31	-0.29	0	1	1.2e+03	1	IDAPR
☑	781	905.77	2714.29	2714.39	-0.11	1	1	1.3e+03	1	TPLLGATLGDTNSPHTMGILYTADKK
☑	1251	1140.66	3418.96	3418.60	0.36	2	1	1.1e+03	1	AGKGRSWMVTMCYLTTFGGMSFAEAVEFIR + Carbamidomethyl (C); Oxidation (M)
☑	1736	875.23	1748.44	1748.75	-0.31	0	1	6.5e+02	1	QSPESSPEALMADSER + Oxidation (M)
☑	1201	1108.63	3322.86	3322.80	0.05	2	1	1e+03	1	MPQQTAKPKGPTSLPPRPVNAGGLQTPQRVR + Oxidation (M)
☑	1268	1148.83	3443.46	3443.48	-0.02	2	1	1.5e+03	1	MTMKDFVRNGNECVAVPFPEDVMCEMYTR + 2 Oxidation (M)
☑	569	804.14	1606.26	1605.84	0.42	1	1	1.6e+03	1	ILGYDLLAKCTDGPK
☑	1330	1183.84	3548.49	3548.81	-0.32	2	1	1.2e+03	1	NITVLQACNSLNIQIPKFCFQENLKIAGNCR + Carbamidomethyl (C)
☑	1462	1287.25	3858.73	3858.86	-0.13	2	1	1.2e+03	1	ATAEGAPLPTDASAEESAATVDFLRHRQEQMQLYR
☑	2126	936.83	2807.47	2807.47	-0.01	2	1	3e+02	1	CIQALSPRTKPSGGAPAAAGEGARVLSDK
☑	1050	1028.37	1027.36	1027.46	-0.10	1	1	1.3e+03	1	CDHLDGRR + Carbamidomethyl (C)
☑	2097	1345.27	2688.53	2688.35	0.18	2	1	3.3e+02	1	MRSIQLQLSVMRNIVPSGEQDCK + Carbamidomethyl (C)

☑	2145	995.46	2983.36	2983.59	-0.24	2	1	3.7e+02	1	KILMAPALGIAAGVNVIGGHSQCFVMSRK + Oxidation (M)
☑	2052	1245.41	2488.81	2489.29	-0.48	1	1	5.3e+02	1	ANGGEVTELTVWRLLIYDDIGR
☑	2079	1283.60	2565.19	2565.32	-0.14	1	1	3.4e+02	1	NLKHQPCTSVPASTSELLPISTSR
☑	1907	1057.17	2112.33	2112.09	0.24	0	1	4.5e+02	1	ISFIEFNEQDVVSILGFR
☑	1718	568.61	1702.81	1702.91	-0.11	0	1	4e+02	1	MSILSPVSVCRPSSLK
☑	1989	767.47	2299.39	2298.94	0.45	1	1	3.8e+02	1	EFCGCGMAHWGSDERGVMATR
☑	1028	1015.39	3043.14	3043.55	-0.41	2	1	1.3e+03	1	NIRFNVVAPGLIDGEGMATSLNEAQKQR + Oxidation (M)
☑	1876	677.53	2029.58	2029.00	0.59	2	1	6.5e+02	1	GPKGPAGPAGADGEEGPAGPRGK
☑	118	517.00	1547.97	1547.78	0.19	0	1	1.5e+03	1	NFTIDQVELDNIK
☑	1387	1212.47	3634.40	3634.84	-0.44	2	1	1.3e+03	1	HVPRIMEAMLNDRVLFHLMDTSPLLPLSDSR + 2 Oxidation (M)
☑	793	910.57	909.56	909.51	0.05	1	1	3.3e+02	1	AIFERFK
☑	1800	942.69	1883.37	1883.86	-0.49	0	1	6.1e+02	1	ASGVPATFAAGCFWGTER + Carbamidomethyl (C)
☑	1919	1075.71	2149.40	2148.97	0.43	2	1	4.5e+02	1	HDDRNASLQKMVDCCSISK
☑	1356	1196.82	2391.63	2391.23	0.40	0	1	1.3e+03	1	LLQNETFNFEELVSKPIDQK
☑	1533	1365.61	4093.79	4093.70	0.10	2	1	9.8e+02	1	CDKEINIEHGHGGYECGDNGEWSTKCVLAYCDSGYK + 2 Carbamidomethyl (C)
☑	1536	1366.22	2730.43	2730.22	0.21	1	1	1.4e+03	1	TTDEEAPAWDLSEGAVTMVYDMKR + Oxidation (M)
☑	2206	1183.24	3546.70	3546.64	0.06	2	1	2.6e+02	1	ELEKCTNSMVDLVGDSKQLTEMITMCEASLR + Carbamidomethyl (C); Oxidation (M)
☑	873	948.03	2841.08	2841.18	-0.10	2	1	1.6e+03	1	DGYYASNGICKKCDENCGSCVINSK + 3 Carbamidomethyl (C)
☑	1450	1276.15	3825.42	3825.76	-0.34	2	1	1.6e+03	1	EGRCWARPGRGTGCAGGVQYCPLFIPSTCNELEK + 3 Carbamidomethyl (C)
☑	2107	904.73	2711.18	2711.17	0.01	2	1	3.3e+02	1	DDEEPSGSRGAAGSSAAQRMVWEMR + 2 Oxidation (M)
☑	171	571.61	1711.82	1711.97	-0.15	2	1	1.1e+03	1	RGPPLRLLSLQFCR + Carbamidomethyl (C)
☑	689	865.96	864.95	865.52	-0.57	2	1	8.4e+02	1	VRLHRSV
☑	794	910.86	909.85	910.43	-0.57	0	1	1.3e+03	1	NVSSDHPR
☑	841	931.64	2791.91	2791.42	0.49	2	1	1.5e+03	1	SYYGTHRIGPFHKTFTAVIGPNGSGK
☑	1082	1041.08	3120.23	3120.58	-0.35	2	1	1.6e+03	1	IAMESLDRPLEQEVRLSFGAFFERHK + Oxidation (M)
☑	728	880.71	2639.10	2639.17	-0.07	0	1	1.6e+03	1	QPGSPSVSSPQCGMDPQTNHSAGVGAK + Oxidation (M)
☑	1787	926.59	1851.17	1850.90	0.27	2	1	4.8e+02	1	SLEEKVNSFEEQREK
☑	505	766.53	765.52	765.41	0.12	1	1	3.8e+02	1	MKSGSLK + Oxidation (M)
☑	1058	1031.32	3090.94	3090.64	0.30	1	1	1.6e+03	1	MMLQIIVGTAFSCPSEPLAKAFQLVIAR + Carbamidomethyl (C)
☑	1140	1074.65	3220.93	3220.50	0.43	2	1	1.3e+03	1	VTDVVRHACEDHIEFCPYEQFEADLKK
☑	1264	1146.73	1145.73	1145.61	0.12	1	1	1.4e+03	1	IGVDTPSVKCK
☑	1324	1181.20	2360.39	2359.97	0.43	2	1	1.3e+03	1	TEDGGGSGMGQAMGPRCPYCSKV + Carbamidomethyl (C); Oxidation (M)
☑	716	876.87	2627.58	2627.13	0.45	1	1	1.5e+03	1	CHGSANCPVCIVVEELHCECRR + 3 Carbamidomethyl (C)
☑	1740	879.80	1757.58	1756.99	0.59	0	1	4.6e+02	1	DVGALAIAAVIANHPGLR

☑	1869	674.64	2020.90	2020.81	0.09	1	1	3.9e+02	1	MFCAQCEETNNNTGCTKK
☑	1059	1031.64	3091.90	3091.34	0.56	1	1	1.4e+03	1	VRPVACSVCDADVSCFAAATRAMSESCR + 3 Carbamidomethyl (C); Oxidation (M)
☑	2179	1075.45	3223.33	3223.68	-0.35	1	1	3.2e+02	1	AAAAPVRSESSTSKPSVAQLRPTNIDDVAGTK
☑	513	773.46	772.45	772.37	0.09	0	0	5.9e+02	1	CISHTR + Carbamidomethyl (C)
☑	1806	949.92	1897.82	1897.91	-0.09	1	0	3.9e+02	1	SPGHLCRNEEVSSSALR + Carbamidomethyl (C)
☑	618	831.67	1661.32	1661.86	-0.54	1	0	1.8e+03	1	LIDVFVNECAKHFK
☑	1180	1097.33	2192.64	2193.04	-0.40	2	0	1.4e+03	1	LYFMDCKTCGASRSVASIK + 2 Carbamidomethyl (C)
☑	1247	1134.38	2266.74	2267.16	-0.43	2	0	1.5e+03	1	ASPSGGGAAEDGASAAATRLRIPGK
☑	1214	1113.64	3337.91	3337.72	0.19	2	0	1.2e+03	1	EFFSIVDVVFAGTMGPPGGGRHFITQRFLR
☑	464	746.72	745.72	745.41	0.31	1	0	6.3e+02	1	AEVRSGK
☑	607	413.34	824.67	824.51	0.16	0	0	3.4e+02	1	LVDPIIR
☑	1230	1120.16	3357.46	3357.57	-0.12	0	0	1.5e+03	1	NAYWELWGLPLFDIQDPAALMYELGECK + Carbamidomethyl (C); Oxidation (M)
☑	2170	1045.45	3133.33	3133.57	-0.25	1	0	3.5e+02	1	KPSAAAGPIDGGAVVVGSTNGGLDTGMQTSYKK
☑	1206	1109.70	3326.07	3325.56	0.51	1	0	1.3e+03	1	EEGGETDDDNITSPEDEFVGRVIGLAHPLSK
☑	1452	1277.77	2553.53	2553.20	0.33	1	0	1.3e+03	1	RCEGLGMTAEACHCTIYAGLILR + Carbamidomethyl (C); Oxidation (M)
☑	1645	786.30	1570.59	1570.73	-0.13	2	0	4.6e+02	1	CCKRAGSLTETSMK + Carbamidomethyl (C)
☑	1321	1178.84	3533.49	3533.67	-0.18	1	0	1.4e+03	1	ALSGEKGVTVCTYVESSVEPSCTFFSSHVELGK + Carbamidomethyl (C)
☑	1335	1185.71	2369.41	2369.15	0.26	1	0	1.3e+03	1	VIPHATGCARAEGYYFVSAETK
☑	517	776.54	2326.61	2326.24	0.37	1	0	1.7e+03	1	TLLCGQRILNVSVLPDTANGSR
☑	1135	1072.21	2142.40	2142.00	0.40	2	0	1.4e+03	1	REEHQLAVQQYCDDPRR
☑	2146	996.91	2987.71	2987.63	0.09	2	0	3.4e+02	1	RAAVTVPLSSDSSGSRVRPSVATPAVPPAR
☑	570	804.53	803.53	803.48	0.05	0	0	5.8e+02	1	VILTSGSK
☑	729	881.13	1760.25	1759.98	0.26	1	0	1.3e+03	1	IDSFVIITEAQRLQK
☑	934	974.75	1947.49	1947.82	-0.33	0	0	1.6e+03	1	DESGEGVIDLDDDEVGER
☑	1955	1119.90	2237.78	2238.07	-0.29	0	0	5.3e+02	1	CLELSFLGGNPEIMLECLK + 2 Carbamidomethyl (C); Oxidation (M)
☑	2189	1102.84	3305.49	3305.68	-0.18	2	0	3.2e+02	1	LDTNFYRYPPSEVIKQQIAMTAPPIEER
☑	1399	611.67	1221.33	1220.75	0.58	2	0	5e+02	1	FKSTLGKIVTK
☑	1444	1270.52	3808.54	3807.95	0.59	2	0	1.2e+03	1	VYKMFIGNNEICLHALAAIEKSPIVVSFLEQCR + Carbamidomethyl (C); Oxidation (M
☑	678	862.54	861.53	861.46	0.07	1	0	5.9e+02	1	MPTLKEK + Oxidation (M)
☑	1032	1017.24	2032.46	2031.89	0.57	0	0	1.7e+03	1	NLFQMASLYDCPMASER + Carbamidomethyl (C)
☑	169	569.84	568.83	568.30	0.53	0	0	1.7e+02	1	AHDVK
☑	500	764.03	2289.06	2289.18	-0.13	2	0	1.4e+03	1	LPVKWCAIEALDDRIFSEK + Carbamidomethyl (C)
☑	2054	1249.53	2497.05	2497.39	-0.34	2	0	3.9e+02	1	SGEMLAILGPSGAGKSTLLDILAKR
☑	791	909.77	2726.28	2726.12	0.16	0	0	1.5e+03	1	SLCGCAQSVYSYSMNDYNAHCLPK + Carbamidomethyl (C); Oxidation (M)

☑	1154	1084.99	2167.96	2168.17	-0.21	1	0	1.5e+03	1	KLANGIVNLYNEGTVTLGHR
☑	821	922.79	2765.34	2765.41	-0.07	2	0	1.4e+03	1	YTVRAVYLATTDSSEQHVAQRATAK
☑	1998	1170.72	2339.43	2339.21	0.22	2	0	4.5e+02	1	AAMKNQTLQFITIDISRMSR + Oxidation (M)
☑	406	713.71	2138.10	2137.94	0.15	2	0	1.2e+03	1	GCNEDTGEEAKRAPAYQSR + Carbamidomethyl (C)
☑	1420	1247.69	2493.36	2493.13	0.23	1	0	1.3e+03	1	MGYWDPEYCVKDTDVLALFR + Carbamidomethyl (C); Oxidation (M)
☑	1932	1083.12	2164.24	2164.11	0.13	2	0	4e+02	1	TEINTVQEMQFGKRAQLR + Oxidation (M)
☑	1995	1166.66	2331.31	2331.89	-0.58	1	0	3.8e+02	1	NSSVTCGARCQAAAADCDSCEDR
☑	602	821.27	2460.79	2460.28	0.51	2	0	1.6e+03	1	VSELEMFWRSLLKSSVHLGDK
☑	990	998.26	997.26	997.53	-0.28	0	0	1.7e+03	1	IFTDPLHR
☑	1739	877.09	1752.16	1751.96	0.19	1	0	4.5e+02	1	AAAVGQQAKAAGANAAAAIK
☑	1902	1050.71	2099.41	2098.97	0.44	1	0	5.8e+02	1	NHIMCEFIPKICEFMTK + Oxidation (M)
☑	911	966.91	2897.70	2897.60	0.09	0	0	1.5e+03	1	QLGAVIIMIHLVIGYTAIQTMAIWAR + Oxidation (M)
☑	1999	783.87	2348.60	2349.00	-0.40	2	0	6.7e+02	1	ENDMLXHXHRAGNSTYARQK
☑	1085	1041.83	3122.47	3122.64	-0.17	2	0	1.4e+03	1	AHMLQPGNIEVVRHILGVLHGECMRLK + Carbamidomethyl (C); Oxidation (M)
☑	1550	1388.71	4163.10	4163.03	0.07	2	0	1.1e+03	1	LAMQEFMILPVGATSFTESMQIGCEVYHCLKKVINK + Carbamidomethyl (C); 3 Oxidati
☑	1862	1004.06	2006.11	2005.94	0.17	0	0	4.3e+02	1	WLMDHDIIADEAPPPMR
☑	176	576.38	1726.10	1725.84	0.26	1	0	1.3e+03	1	SEAWVAGKLGMSMLSK + 2 Oxidation (M)
☑	184	582.10	1743.28	1742.73	0.55	1	0	1.5e+03	1	QCWRATCADCSTSVR + Carbamidomethyl (C)
☑	837	930.35	2788.02	2787.46	0.56	2	0	1.6e+03	1	VMPVGSRTAIGVGTRQVSSSFGGVPWR
☑	586	814.05	1626.09	1625.89	0.19	1	0	1.6e+03	1	KQFYAGQLVQFIGK
☑	981	993.77	992.76	992.53	0.23	1	0	1.6e+03	1	RVVEAYEK
☑	988	997.42	996.41	996.50	-0.09	1	0	4e+02	1	MMRASFVR
☑	2133	968.20	2901.58	2901.47	0.10	1	0	3.8e+02	1	ETPTPPQPVVKSGVGTGASTTGSGVTSFQK
☑	142	538.28	1611.82	1611.76	0.06	1	0	1e+03	1	SPCFKDLDFVEQK + Carbamidomethyl (C)
☑	1410	1234.48	3700.41	3700.01	0.41	2	0	1.3e+03	1	TPVAVICAGVKSILDIPHVDAAIEAGLAAAKASNVMGK
☑	1415	1240.17	3717.49	3716.90	0.60	2	0	1.5e+03	1	LDCPGAEQRLMKPQHPLQEATVKSLMPLGATWR + Oxidation (M)
☑	1728	870.41	1738.81	1738.86	-0.05	0	0	4.9e+02	1	IWEEGGITCSVRPHR
☑	1861	1003.71	2005.40	2005.88	-0.48	1	0	5.9e+02	1	RNVCHPFQGECVDVMEK + Oxidation (M)
☑	2158	1021.05	3060.13	3060.69	-0.56	1	0	4.8e+02	1	CTHASMALVAAVALVLVLPVSRARPVGMR + Carbamidomethyl (C); Oxidation (M)
☑	289	639.55	638.55	638.30	0.24	1	0	3.7e+02	1	YNKTN
☑	567	803.90	2408.67	2408.08	0.59	0	0	2.4e+03	1	TCMADVLMSEASEWAGIAGLGHK + 2 Oxidation (M)
☑	585	813.34	1624.66	1624.75	-0.09	0	0	1.3e+03	1	VSSASCSVNYAPLDR + Carbamidomethyl (C)
☑	1405	1231.35	3691.02	3690.60	0.42	2	0	1.6e+03	1	MDNEYKDSIDDQAQDQNSHEEIHRTLEDTSK
☑	1888	688.20	2061.57	2061.99	-0.42	2	0	7.4e+02	1	ARMNEQCAIIKNCANDIR

☑	2121	918.85	2753.53	2753.43	0.11	1	0	3.9e+02	1	NEIQRVSVTSCVSALPNVVHFLADR
☑	2213	1246.90	3737.68	3737.79	-0.11	2	0	2.7e+02	1	NTFPNEQRTPHSHSTLTQSISESKSENEIPQSK
☑	2156	1020.47	3058.39	3058.52	-0.13	1	0	3.9e+02	1	LHDESDLADERPLHHVTLMVIAMEKK + 2 Oxidation (M)
☑	1292	1161.08	2320.15	2320.09	0.06	2	0	1.9e+03	1	MVPQGKETPDTCFDAVKANNR
☑	2044	1237.19	2472.37	2472.32	0.05	1	0	4e+02	1	DPQTVHLNPKSALPLLDLQSMR
☑	2130	954.79	2861.36	2861.37	-0.01	0	0	3.7e+02	1	LGLDYNEPSPFSDNQSLGEALLEPTR
☑	563	799.47	2395.39	2395.05	0.34	0	0	1.4e+03	1	ESSGMGGMGSLANTNGVGGQAGDGIR + Oxidation (M)
☑	1252	1140.79	2279.57	2279.25	0.32	2	0	1.6e+03	1	SPLPLPHPSPPKARQSTVQSR
☑	2140	988.46	2962.34	2962.39	-0.05	1	0	4.2e+02	1	LCGNGYFLYKGGCYSITHGAPGNLICK + 2 Carbamidomethyl (C)
☑	1794	933.86	1865.70	1865.94	-0.23	1	0	4.5e+02	1	MFDKATAAALEAIDMLR
☑	375	693.12	1384.23	1384.81	-0.59	1	0	1.8e+03	1	LFNFLYKILSK
☑	1210	1111.90	3332.69	3332.71	-0.02	1	0	1.5e+03	1	VNTIQTSTLGSLAMTERLLNDTETVGIDAAAK
☑	1492	654.95	1307.89	1307.65	0.25	0	0	4.8e+02	1	AAPAADAPAPGNASK
☑	1808	635.02	1902.04	1901.97	0.07	0	0	4.9e+02	1	ANATASVLFTEPPMGAGLR
☑	1891	1036.75	2071.49	2071.03	0.46	2	0	8.1e+02	1	CAAHSVGMDTELEVLRKR + Carbamidomethyl (C)
☑	1136	1072.40	1071.39	1071.43	-0.04	0	0	1.5e+03	1	MCSVPFCR + 2 Carbamidomethyl (C); Oxidation (M)
☑	1334	1185.34	3553.00	3552.90	0.10	2	0	1.7e+03	1	KLIAPVHTSILMELNKLAVSMDAFGVVEQEVR + Oxidation (M)
☑	1485	1302.33	3903.98	3903.74	0.24	2	0	1.5e+03	1	MVDGASVTYHDGPVGTCLGWWMWCAVGACPRGRHR + Carbamidomethyl (C); Oxidation
☑	1491	1308.67	3922.99	3922.84	0.16	0	0	1.1e+03	1	SLFIMYGGATSFDEDIEQPSPSLSSHLQFFSSGGLR + Oxidation (M)
☑	1661	795.36	1588.70	1588.74	-0.04	2	0	5.4e+02	1	KDRNGMPGGSGDLNR + Oxidation (M)
☑	1724	859.51	1717.00	1716.83	0.17	0	0	4.7e+02	1	GSNALDSANQLDASVQK
☑	1981	762.36	2284.06	2284.10	-0.05	1	0	4.6e+02	1	RCLQLSFLGGNQEIMSECLK + Oxidation (M)
☑	1	*217.30*	*216.29*							
☑	2	*221.12*	*220.11*							
☑	3	*226.23*	*225.22*							
☑	4	*227.03*	*226.02*							
☑	5	*228.35*	*227.35*							
☑	6	*229.07*	*228.06*							
☑	7	*229.18*	*228.17*							
☑	8	*229.24*	*228.23*							
☑	9	*235.22*	*234.21*							
☑	10	*237.29*	*236.28*							
☑	11	*239.15*	*238.14*							
☑	12	*239.18*	*238.17*							

☑	13	247.10	246.09
☑	14	256.26	255.25
☑	15	257.30	256.29
☑	16	269.03	268.03
☑	17	269.04	268.03
☑	18	269.05	268.04
☑	21	285.03	284.02
☑	22	285.03	284.02
☑	23	285.03	284.02
☑	24	285.06	284.05
☑	25	285.06	284.05
☑	26	285.08	284.07
☑	27	285.08	284.08
☑	28	285.13	284.12
☑	29	288.35	287.34
☑	30	296.90	295.90
☑	31	297.04	296.04
☑	32	297.10	296.09
☑	33	297.12	296.11
☑	35	297.18	296.18
☑	36	298.27	297.26
☑	37	299.09	298.08
☑	38	299.09	298.09
☑	39	299.16	298.15
☑	40	299.20	298.20
☑	41	300.25	299.25
☑	43	351.00	349.99
☑	44	355.14	354.13
☑	45	355.14	354.13
☑	47	371.07	370.06
☑	48	371.08	370.08
☑	49	371.09	370.09
☑	51	371.16	370.15
☑	52	371.16	370.15

☑	53	371.17	370.17
☑	54	371.18	370.17
☑	55	371.18	370.17
☑	56	371.19	370.18
☑	57	371.19	370.18
☑	58	371.32	370.31
☑	60	388.99	387.98
☑	63	429.03	428.02
☑	64	429.15	428.15
☑	65	429.17	428.17
☑	66	429.18	428.17
☑	67	429.21	428.20
☑	68	429.21	428.21
☑	69	429.22	428.21
☑	71	445.16	444.15
☑	72	445.17	444.16
☑	73	445.18	444.17
☑	74	445.19	444.18
☑	75	445.21	444.20
☑	76	445.25	444.24
☑	77	445.25	444.24
☑	78	445.27	444.26
☑	79	445.27	444.27
☑	80	446.11	445.10
☑	84	449.97	448.96
☑	86	455.25	454.24
☑	95	488.07	487.06
☑	98	492.45	491.44
☑	99	494.36	493.35
☑	100	496.42	495.41
☑	102	503.09	502.08
☑	103	503.17	502.16
☑	106	507.11	506.10
☑	107	507.67	506.67

☑	108	508.39	507.38
☑	110	510.67	509.66
☑	113	257.24	512.46
☑	115	514.43	513.43
☑	116	515.40	514.39
☑	120	519.15	518.15
☑	121	519.16	518.16
☑	123	519.20	518.19
☑	125	519.25	518.24
☑	126	519.35	518.34
☑	131	525.08	524.07
☑	137	533.33	532.32
☑	140	536.58	535.57
☑	141	538.19	537.18
☑	144	543.96	542.95
☑	145	544.02	543.01
☑	152	552.64	551.64
☑	161	563.35	562.34
☑	165	567.30	566.30
☑	168	569.00	567.99
☑	174	572.91	571.90
☑	175	573.09	572.08
☑	179	578.98	577.97
☑	181	579.87	578.86
☑	183	582.09	581.08
☑	187	584.42	583.41
☑	191	586.84	585.83
☑	193	587.39	586.38
☑	195	589.31	588.30
☑	200	593.14	592.13
☑	201	594.31	593.30
☑	203	595.03	594.03
☑	210	599.75	598.74
☑	217	603.61	602.60

☑	218	603.66	602.65
☑	220	603.95	602.95
☑	222	604.07	603.06
☑	225	606.98	605.97
☑	226	606.98	605.97
☑	228	608.02	607.01
☑	233	609.64	608.63
☑	235	611.09	610.08
☑	237	611.94	610.93
☑	243	615.02	614.02
☑	247	617.29	616.29
☑	250	618.72	617.71
☑	252	618.86	617.85
☑	253	619.01	618.00
☑	254	619.02	618.01
☑	257	619.89	618.89
☑	258	620.26	619.26
☑	265	622.80	621.80
☑	272	626.46	625.45
☑	273	627.08	626.07
☑	275	628.01	627.00
☑	278	630.64	629.64
☑	280	632.08	631.07
☑	281	633.20	632.19
☑	283	635.01	634.01
☑	284	637.34	636.33
☑	285	637.37	636.36
☑	288	639.09	638.08
☑	290	640.96	639.96
☑	292	641.92	640.91
☑	293	641.95	640.94
☑	294	642.38	641.37
☑	298	644.21	643.20
☑	300	645.03	644.02

☑	303	646.60	645.60
☑	304	647.47	646.47
☑	305	647.66	646.65
☑	306	649.74	648.73
☑	310	650.82	649.82
☑	327	658.06	657.06
☑	331	664.47	663.46
☑	339	667.13	666.12
☑	340	667.27	666.27
☑	343	672.31	671.31
☑	347	675.33	674.32
☑	349	675.77	674.77
☑	352	678.38	677.37
☑	355	681.52	680.52
☑	356	682.11	681.10
☑	357	682.16	681.15
☑	358	682.41	681.40
☑	362	683.40	682.40
☑	365	685.73	684.72
☑	368	687.62	686.61
☑	369	345.03	688.05
☑	379	694.84	693.83
☑	381	697.46	696.45
☑	383	698.42	697.41
☑	387	701.02	700.02
☑	389	702.84	701.83
☑	390	703.18	702.18
☑	391	703.57	702.56
☑	392	235.24	702.70
☑	394	704.55	703.54
☑	395	704.71	703.70
☑	396	705.30	704.29
☑	402	711.10	710.09
☑	405	713.23	712.23

☑	407	713.78	712.77
☑	409	714.77	713.76
☑	410	715.14	714.13
☑	411	239.08	714.23
☑	413	717.60	716.59
☑	415	718.47	717.46
☑	420	720.29	719.29
☑	421	720.40	719.40
☑	422	722.16	721.15
☑	425	723.36	722.35
☑	426	723.52	722.51
☑	429	724.38	723.37
☑	431	728.32	727.31
☑	433	729.26	728.26
☑	434	729.54	728.53
☑	435	730.05	729.04
☑	438	732.57	731.56
☑	442	735.01	734.00
☑	443	735.18	734.17
☑	445	737.47	736.46
☑	446	737.69	736.68
☑	447	738.28	737.27
☑	449	740.06	739.05
☑	450	371.14	740.26
☑	452	741.45	740.44
☑	458	744.04	743.03
☑	459	744.08	743.07
☑	463	746.31	745.30
☑	465	747.12	746.11
☑	467	747.70	746.69
☑	468	749.56	748.55
☑	471	752.50	751.49
☑	473	753.00	751.99
☑	474	753.18	752.17

☑	477	755.68	754.68
☑	479	755.80	754.79
☑	483	757.43	756.43
☑	484	757.71	756.70
☑	487	759.06	758.05
☑	488	759.33	758.32
☑	490	759.52	758.52
☑	491	760.22	759.21
☑	493	760.86	759.85
☑	495	761.52	760.51
☑	497	762.52	761.51
☑	502	765.79	764.79
☑	506	766.90	765.89
☑	509	769.55	768.55
☑	511	770.47	769.47
☑	512	771.98	770.97
☑	518	777.42	776.41
☑	524	784.55	783.55
☑	534	788.92	787.91
☑	536	789.78	788.78
☑	538	790.38	789.37
☑	540	791.98	790.97
☑	543	792.52	791.52
☑	545	793.47	792.46
☑	546	793.55	792.54
☑	547	793.60	792.59
☑	548	793.96	792.95
☑	549	793.97	792.96
☑	551	795.05	794.04
☑	557	797.31	796.30
☑	558	797.49	796.49
☑	560	798.54	797.53
☑	564	799.88	798.87
☑	571	804.59	803.58

☑	572	269.00	803.97
☑	575	807.18	806.17
☑	582	812.41	811.40
☑	583	812.89	811.88
☑	584	813.05	812.05
☑	587	814.84	813.84
☑	589	815.00	813.99
☑	592	816.50	815.49
☑	598	819.60	818.60
☑	599	819.89	818.89
☑	600	820.03	819.03
☑	608	826.15	825.14
☑	609	826.41	825.40
☑	616	829.33	828.32
☑	619	831.84	830.83
☑	621	833.20	832.19
☑	627	835.00	834.00
☑	628	835.44	834.44
☑	629	836.61	835.60
☑	630	836.68	835.68
☑	633	837.81	836.80
☑	634	838.15	837.14
☑	635	839.47	838.46
☑	638	839.67	838.67
☑	641	840.47	839.46
☑	643	840.52	839.51
☑	645	842.49	841.49
☑	652	847.46	846.45
☑	655	849.61	848.60
☑	658	851.20	850.19
☑	660	285.02	852.04
☑	661	285.06	852.16
☑	662	285.13	852.37
☑	665	855.47	854.46

666	855.73	854.72
667	856.73	855.72
668	857.18	856.17
669	429.19	856.37
670	857.87	856.86
672	860.01	859.00
673	860.68	859.68
676	861.45	860.44
677	861.72	860.71
679	862.76	861.75
680	863.38	862.37
682	863.52	862.51
683	863.54	862.54
684	432.33	862.64
686	864.15	863.14
690	866.44	865.44
693	869.30	868.30
696	869.94	868.93
697	870.79	869.79
698	870.80	869.79
701	871.54	870.53
707	874.06	873.06
708	874.08	873.07
709	874.31	873.31
713	875.33	874.32
714	876.38	875.38
715	876.60	875.59
717	876.91	875.90
718	877.24	876.24
723	878.26	877.25
724	878.27	877.26
725	879.05	878.05
726	879.75	878.74
727	880.38	879.38

☑	730	881.29	880.28
☑	731	881.46	880.45
☑	733	882.65	881.64
☑	734	883.34	882.33
☑	736	883.55	882.55
☑	739	885.55	884.54
☑	746	889.25	888.24
☑	748	890.34	889.33
☑	750	890.57	889.57
☑	751	892.39	891.39
☑	753	893.37	892.37
☑	756	893.94	892.93
☑	757	894.84	893.84
☑	758	895.56	894.55
☑	759	896.10	895.09
☑	760	896.58	895.57
☑	767	899.21	898.20
☑	768	899.38	898.37
☑	770	902.67	901.66
☑	771	902.89	901.89
☑	773	903.37	902.37
☑	778	904.81	903.80
☑	779	905.10	904.09
☑	780	905.58	904.57
☑	782	906.13	905.13
☑	785	907.85	906.84
☑	792	910.23	909.22
☑	795	911.92	910.92
☑	798	912.73	911.72
☑	805	915.76	914.76
☑	807	459.61	917.21
☑	808	918.39	917.39
☑	810	919.81	918.81
☑	811	920.17	919.17

☑	815	922.10	921.09
☑	816	922.12	921.11
☑	817	922.12	921.12
☑	818	922.14	921.13
☑	822	923.05	922.04
☑	823	924.00	923.00
☑	829	925.67	924.67
☑	832	928.11	927.10
☑	833	928.41	927.40
☑	835	929.22	928.21
☑	839	931.00	929.99
☑	842	931.87	930.86
☑	843	931.93	930.93
☑	844	932.73	931.72
☑	845	933.36	932.36
☑	847	935.13	934.13
☑	848	936.56	935.55
☑	851	937.32	936.31
☑	852	937.63	936.62
☑	853	938.45	937.44
☑	860	941.51	940.51
☑	862	941.66	940.65
☑	863	942.93	941.93
☑	867	945.61	944.60
☑	869	946.23	945.22
☑	870	946.34	945.33
☑	872	947.28	946.28
☑	879	952.22	951.21
☑	880	954.21	953.20
☑	881	954.71	953.70
☑	886	957.28	956.27
☑	888	958.35	957.34
☑	890	959.10	958.09
☑	893	960.08	959.07

☑	894	960.26	959.25
☑	895	960.45	959.45
☑	898	961.26	960.26
☑	900	962.76	961.76
☑	903	964.06	963.05
☑	909	965.65	964.64
☑	915	967.84	966.83
☑	917	968.81	967.80
☑	919	969.46	968.45
☑	924	971.41	970.41
☑	930	973.17	972.17
☑	933	973.77	972.77
☑	936	975.11	974.10
☑	937	975.17	974.17
☑	939	975.57	974.56
☑	940	975.73	974.72
☑	943	976.75	975.74
☑	944	977.49	976.48
☑	946	978.71	977.70
☑	947	978.98	977.97
☑	950	979.49	978.48
☑	951	980.07	979.06
☑	952	981.05	980.05
☑	957	982.70	981.69
☑	958	983.36	982.36
☑	961	984.42	983.42
☑	962	984.92	983.92
☑	965	986.70	985.69
☑	967	988.65	987.64
☑	971	990.19	989.18
☑	976	992.61	991.60
☑	978	993.45	992.44
☑	979	993.66	992.66
☑	982	993.85	992.84

☑	983	993.99	992.98
☑	985	995.16	994.16
☑	992	998.46	997.45
☑	994	999.15	998.15
☑	995	999.52	998.51
☑	996	999.67	998.67
☑	998	1000.21	999.20
☑	1001	1002.07	1001.06
☑	1005	1004.43	1003.43
☑	1007	1004.64	1003.63
☑	1010	1005.51	1004.50
☑	1011	1005.91	1004.90
☑	1013	1006.09	1005.08
☑	1017	1008.33	1007.32
☑	1019	1010.40	1009.40
☑	1021	1011.34	1010.33
☑	1022	1011.48	1010.48
☑	1023	1011.54	1010.53
☑	1026	1014.14	1013.13
☑	1031	1016.99	1015.98
☑	1033	1017.48	1016.48
☑	1034	1017.85	1016.85
☑	1035	1018.49	1017.48
☑	1038	1022.15	1021.14
☑	1041	1023.20	1022.19
☑	1053	1029.36	1028.36
☑	1056	1030.53	1029.53
☑	1065	1033.82	1032.82
☑	1066	1033.85	1032.84
☑	1068	1034.25	1033.24
☑	1077	1038.51	1037.50
☑	1086	1042.03	1041.02
☑	1089	1044.05	1043.04
☑	1092	1046.59	1045.59

☑	1093	1048.15	1047.15
☑	1094	1048.37	1047.36
☑	1098	1050.07	1049.06
☑	1100	1051.44	1050.43
☑	1105	1054.02	1053.01
☑	1107	1054.27	1053.26
☑	1112	1058.62	1057.62
☑	1113	1060.04	1059.03
☑	1115	1061.13	1060.13
☑	1116	1061.31	1060.30
☑	1123	1066.96	1065.95
☑	1124	1067.19	1066.19
☑	1125	1067.20	1066.19
☑	1129	1068.68	1067.67
☑	1132	1071.36	1070.35
☑	1139	1074.57	1073.56
☑	1141	1075.72	1074.71
☑	1142	1076.12	1075.11
☑	1143	1078.00	1077.00
☑	1147	1079.87	1078.86
☑	1151	1083.55	1082.55
☑	1153	1084.74	1083.73
☑	1155	1085.18	1084.17
☑	1159	1087.99	1086.98
☑	1162	1089.14	1088.14
☑	1163	1089.19	1088.19
☑	1164	1089.73	1088.72
☑	1172	1090.87	1089.86
☑	1173	1091.12	1090.11
☑	1174	1091.17	1090.16
☑	1177	1093.54	1092.53
☑	1179	1097.20	1096.19
☑	1183	1099.24	1098.23
☑	1185	550.53	1099.04

☑	1186	1100.61	1099.60
☑	1187	550.87	1099.72
☑	1189	1101.83	1100.82
☑	1190	1102.24	1101.23
☑	1197	1106.65	1105.64
☑	1200	1108.42	1107.42
☑	1212	1113.42	1112.41
☑	1217	1115.45	1114.45
☑	1219	1116.25	1115.24
☑	1227	1118.76	1117.75
☑	1231	1121.04	1120.03
☑	1236	1123.67	1122.66
☑	1237	1124.18	1123.17
☑	1238	563.16	1124.32
☑	1240	1127.30	1126.29
☑	1243	1132.85	1131.84
☑	1246	1133.82	1132.82
☑	1249	1138.65	1137.64
☑	1255	1141.70	1140.69
☑	1258	1142.65	1141.65
☑	1260	572.61	1143.20
☑	1261	1144.86	1143.85
☑	1263	1146.70	1145.69
☑	1266	1147.06	1146.05
☑	1274	1151.65	1150.65
☑	1275	1151.86	1150.85
☑	1277	576.75	1151.48
☑	1280	577.38	1152.75
☑	1284	1156.83	1155.83
☑	1286	1157.54	1156.53
☑	1293	1161.74	1160.74
☑	1294	1161.83	1160.82
☑	1295	581.96	1161.91
☑	1297	1164.59	1163.58

☑	1300	1165.37	1164.36
☑	1301	1165.47	1164.46
☑	1305	1166.89	1165.88
☑	1311	1171.90	1170.89
☑	1314	1174.73	1173.73
☑	1320	1178.24	1177.23
☑	1326	1182.52	1181.51
☑	1331	592.56	1183.10
☑	1342	1188.01	1187.00
☑	1347	1192.17	1191.16
☑	1352	1194.14	1193.14
☑	1355	1196.07	1195.06
☑	1358	1198.31	1197.30
☑	1360	1199.39	1198.39
☑	1361	600.20	1198.39
☑	1362	1200.78	1199.77
☑	1363	1201.63	1200.62
☑	1370	1204.00	1203.00
☑	1374	1205.79	1204.78
☑	1375	1205.95	1204.95
☑	1378	1207.39	1206.38
☑	1379	1207.60	1206.59
☑	1382	604.96	1207.91
☑	1384	1210.52	1209.52
☑	1388	1213.51	1212.50
☑	1390	1214.32	1213.31
☑	1391	1214.72	1213.71
☑	1393	1215.85	1214.84
☑	1395	1219.90	1218.89
☑	1398	1221.96	1220.96
☑	1403	1227.31	1226.30
☑	1406	1231.47	1230.46
☑	1407	617.00	1231.99
☑	1409	1234.27	1233.26

☑	1414	1236.46	1235.45
☑	1416	1243.24	1242.23
☑	1418	1244.53	1243.52
☑	1419	1245.27	1244.26
☑	1422	1250.12	1249.11
☑	1425	1252.51	1251.50
☑	1427	1254.61	1253.60
☑	1430	1255.57	1254.56
☑	1434	1260.85	1259.84
☑	1457	1281.58	1280.57
☑	1459	1282.77	1281.76
☑	1467	645.56	1289.11
☑	1468	1290.37	1289.36
☑	1470	1291.72	1290.71
☑	1471	1293.94	1292.93
☑	1473	1296.12	1295.11
☑	1475	1297.83	1296.82
☑	1483	1301.23	1300.22
☑	1501	1317.48	1316.47
☑	1515	1340.99	1339.98
☑	1520	1347.63	1346.62
☑	1523	1349.77	1348.76
☑	1531	1363.67	1362.66
☑	1540	1379.77	1378.76
☑	1552	1390.44	1389.43
☑	1566	708.42	1414.82
☑	1567	709.96	1417.91
☑	1568	710.37	1418.73
☑	1573	711.34	1420.67
☑	1585	719.52	1437.03
☑	1587	722.44	1442.86
☑	1593	725.82	1449.62
☑	1611	747.20	1492.39
☑	1613	748.97	1495.93

☑	1616	751.31	1500.60
☑	1617	751.55	1501.09
☑	1626	760.49	1518.96
☑	1629	765.80	1529.59
☑	1632	771.84	1541.67
☑	1635	774.25	1546.49
☑	1641	782.75	1563.50
☑	1642	784.28	1566.55
☑	1643	785.92	1569.83
☑	1647	788.55	1575.08
☑	1651	793.04	1584.07
☑	1653	793.89	1585.76
☑	1659	794.86	1587.71
☑	1662	800.05	1598.08
☑	1663	800.83	1599.65
☑	1673	809.91	1617.80
☑	1678	813.63	1625.25
☑	1679	814.53	1627.04
☑	1683	820.84	1639.67
☑	1684	820.96	1639.91
☑	1686	821.44	1640.87
☑	1692	834.95	1667.89
☑	1696	836.54	1671.07
☑	1697	836.60	1671.18
☑	1699	838.00	1673.98
☑	1702	842.30	1682.60
☑	1706	845.28	1688.55
☑	1710	847.89	1693.77
☑	1711	849.06	1696.10
☑	1715	850.74	1699.46
☑	1730	581.79	1742.34
☑	1731	872.53	1743.05
☑	1742	881.53	1761.05
☑	1743	883.45	1764.88

☑	1752	896.00	1789.98
☑	1756	898.44	1794.87
☑	1759	899.34	1796.67
☑	1760	899.87	1797.72
☑	1766	909.33	1816.65
☑	1769	915.35	1828.68
☑	1771	916.32	1830.62
☑	1786	926.24	1850.46
☑	1793	932.30	1862.60
☑	1796	938.83	1875.64
☑	1803	944.60	1887.19
☑	1809	952.40	1902.78
☑	1811	955.47	1908.92
☑	1812	955.64	1909.26
☑	1813	960.25	1918.48
☑	1820	966.72	1931.43
☑	1823	970.48	1938.94
☑	1824	971.93	1941.85
☑	1828	974.07	1946.12
☑	1829	975.42	1948.82
☑	1831	979.42	1956.82
☑	1835	980.56	1959.11
☑	1839	983.69	1965.37
☑	1842	984.55	1967.09
☑	1843	984.78	1967.56
☑	1845	987.74	1973.46
☑	1846	991.45	1980.89
☑	1847	992.26	1982.50
☑	1848	993.61	1985.21
☑	1849	994.08	1986.15
☑	1873	1013.00	2023.99
☑	1875	676.77	2027.29
☑	1877	1015.89	2029.76
☑	1885	1027.52	2053.03

☑	1892	1040.09	2078.16
☑	1894	1042.44	2082.86
☑	1895	1046.25	2090.49
☑	1899	1048.91	2095.81
☑	1901	1050.18	2098.34
☑	1903	1051.44	2100.88
☑	1904	1051.61	2101.20
☑	1905	1053.04	2104.07
☑	1911	1064.13	2126.25
☑	1912	1067.10	2132.19
☑	1916	1071.72	2141.43
☑	1917	1073.41	2144.81
☑	1918	1075.28	2148.54
☑	1924	1079.02	2156.02
☑	1929	1081.47	2160.93
☑	1931	1082.90	2163.78
☑	1933	1084.79	2167.57
☑	1940	728.11	2181.31
☑	1944	1100.90	2199.78
☑	1945	1101.45	2200.89
☑	1946	1103.51	2205.00
☑	1948	1109.32	2216.62
☑	1951	1114.91	2227.82
☑	1962	1126.99	2251.96
☑	1964	753.03	2256.07
☑	1966	1132.72	2263.43
☑	1968	1136.32	2270.62
☑	1969	1138.10	2274.19
☑	1970	1140.53	2279.05
☑	1971	1140.88	2279.74
☑	1972	1141.25	2280.49
☑	1973	761.54	2281.61
☑	1978	1142.34	2282.66
☑	1979	762.12	2283.33

☑	1992	1162.04	2322.07
☑	1993	1162.64	2323.27
☑	1996	1167.62	2333.22
☑	1997	1168.64	2335.27
☑	2000	1175.80	2349.59
☑	2001	1177.02	2352.02
☑	2002	1177.64	2353.26
☑	2004	1181.56	2361.11
☑	2005	788.77	2363.29
☑	2006	789.23	2364.68
☑	2007	1183.69	2365.37
☑	2010	1184.30	2366.58
☑	2011	1188.18	2374.34
☑	2012	792.48	2374.43
☑	2015	1192.06	2382.11
☑	2019	1195.95	2389.90
☑	2020	798.33	2391.98
☑	2021	1197.08	2392.15
☑	2024	1202.83	2403.64
☑	2030	808.54	2422.60
☑	2031	1215.93	2429.86
☑	2033	812.47	2434.39
☑	2034	1219.94	2437.87
☑	2035	1220.06	2438.11
☑	2038	1227.28	2452.55
☑	2039	1227.73	2453.44
☑	2040	818.86	2453.56
☑	2043	824.74	2471.20
☑	2049	1243.39	2484.78
☑	2050	1243.75	2485.48
☑	2061	839.49	2515.43
☑	2062	1258.75	2515.49
☑	2064	842.39	2524.16
☑	2066	1263.54	2525.06

2069	846.16	2535.47
2070	849.20	2544.57
2071	1274.09	2546.16
2074	852.73	2555.16
2075	1280.13	2558.24
2081	857.13	2568.37
2085	863.71	2588.12
2086	866.88	2597.62
2087	867.17	2598.49
2089	875.78	2624.32
2091	876.84	2627.50
2092	877.60	2629.79
2094	882.85	2645.51
2095	889.37	2665.08
2098	899.44	2695.29
2099	900.66	2698.96
2101	901.73	2702.16
2102	902.36	2704.05
2106	904.67	2711.00
2108	904.99	2711.93
2112	905.78	2714.31
2113	906.47	2716.39
2125	931.65	2791.93
2129	947.10	2838.28
2134	968.63	2902.87
2135	969.26	2904.75
2136	977.13	2928.38
2137	984.33	2949.98
2139	988.17	2961.49
2141	990.77	2969.29
2143	992.73	2975.16
2144	995.45	2983.33
2150	1007.27	3018.78
2151	1007.89	3020.64

☑	2159	*1021.79*	*3062.35*
☑	2162	*1030.23*	*3087.68*
☑	2163	*1031.68*	*3092.02*
☑	2166	*1038.30*	*3111.86*
☑	2167	*1038.89*	*3113.66*
☑	2168	*1040.68*	*3119.02*
☑	2171	*1046.70*	*3137.07*
☑	2172	*1047.83*	*3140.47*
☑	2180	*1079.50*	*3235.48*
☑	2182	*1084.11*	*3249.31*
☑	2183	*1088.92*	*3263.73*
☑	2185	*1095.52*	*3283.53*
☑	2186	*1095.77*	*3284.30*
☑	2190	*1104.87*	*3311.60*
☑	2192	*1117.67*	*3350.00*
☑	2194	*1121.69*	*3362.05*
☑	2195	*1121.73*	*3362.18*
☑	2196	*1130.15*	*3387.44*
☑	2197	*1131.55*	*3391.63*
☑	2200	*1146.78*	*3437.31*
☑	2201	*1151.24*	*3450.69*
☑	2203	*1158.78*	*3473.30*
☑	2204	*1180.13*	*3537.38*
☑	2205	*1180.22*	*3537.65*
☑	2208	*1188.93*	*3563.76*
☑	2212	*1240.37*	*3718.09*

Search Parameters

```
Type of search         : MS/MS Ion Search
Enzyme                 : Trypsin
Variable modifications : Carbamidomethyl (C),Oxidation (M)
Mass values            : Monoisotopic
Protein Mass           : Unrestricted
Peptide Mass Tolerance : ± 0.6 Da
Fragment Mass Tolerance: ± 0.3 Da
```

```
Max Missed Cleavages   : 2
Instrument type        : ESI-TRAP
Number of queries      : 2213
```

Mascot: http://www.matrixscience.com/
