# Supplementary material for: Macrophage-infectivity potentiator of Trypanosoma cruzi (TcMIP) is a new pro-type 1 immuno-stimulating protein for neonatal human cells and vaccines in mice
Source: Front Immunol. 2023 Mar 23;14:1138526. doi: 10.3389/fimmu.2023.1138526 (PMC10077492; doi:10.3389/fimmu.2023.1138526)
Supplement: Supplementary file 6 [file DataSheet_4.pdf]

Mascot Search Results

User : GM
Email : gabriel.mazzucchelli@ulg.ac.be
Search title : Submitted from 081205-adj-OGE34-8558-other Euc-Sprot by Mascot Daemon on MASPEC39
MS data file : F:\DATA\Archives\ESQUIRE\2008-Esquire\Adjuvac\081205-OGE34-140min\081205-adj-OGE34_8558.mgf
Database : Sprot 55.5 (389046 sequences; 139778124 residues)
Taxonomy : Other Eukaryota (2925 sequences)
Timestamp : 8 Dec 2008 at 08:25:19 GMT
Significant hits:

CH60_TRYCR	Q95046|CH60_TRYCR	Chaperonin HSP60, mitochondrial precursor - Trypanosoma cruzi
FCA1_TRYCR	P07749|FCA1_TRYCR	Flagellar calcium-binding protein - Trypanosoma cruzi
UBIQ_TRYCR	P08565|UBIQ_TRYCR	Ubiquitin - Trypanosoma cruzi
TBB_TRYBR	P04107|TBB_TRYBR	Tubulin beta chain - Trypanosoma brucei rhodesiense
G3PG_TRYCR	P22513|G3PG_TRYCR	Glyceraldehyde-3-phosphate dehydrogenase, glycosomal - Trypanosoma cruzi
RLA3_TRYCR	P26795|RLA3_TRYCR	60S acidic ribosomal protein P2-B - Trypanosoma cruzi
CALM_EUGGR	P11118|CALM_EUGGR	Calmodulin - Euglena gracilis
CH60_TRYBB	Q37683|CH60_TRYBB	Chaperonin HSP60, mitochondrial precursor - Trypanosoma brucei brucei
TBA_TRYCR	Q27352|TBA_TRYCR	Tubulin alpha chain - Trypanosoma cruzi
EF1A_EUGGR	P14963|EF1A_EUGGR	Elongation factor 1-alpha - Euglena gracilis
EF1AC_PORPU	P50256|EF1AC_PORPU	Elongation factor 1-alpha C - Porphyra purpurea
HSP70_BRELC	P16394|HSP70_BRELC	Heat shock 70 kDa protein - Bremia lactucae (Lettuce downy mildew)
RLA1_TRYCR	P26643|RLA1_TRYCR	60S acidic ribosomal protein P1 - Trypanosoma cruzi
H2A1_LEIIN	P27891|H2A1_LEIIN	Histone H2A.1 - Leishmania infantum
H2A_TRYCR	P35066|H2A_TRYCR	Histone H2A - Trypanosoma cruzi
EF1A_TRYBB	P41166|EF1A_TRYBB	Elongation factor 1-alpha - Trypanosoma brucei brucei
HSP70_TRYCR	P05456|HSP70_TRYCR	Heat shock 70 kDa protein - Trypanosoma cruzi
HSP70_ACHKL	P41753|HSP70_ACHKL	Heat shock 70 kDa protein - Achlya klebsiana
HSP70_LEIMA	P14834|HSP70_LEIMA	Heat shock 70 kDa protein - Leishmania major
EF1A_ENTHI	P31018|EF1A_ENTHI	Elongation factor 1-alpha - Entamoeba histolytica
EF1A_GIALA	Q08046|EF1A_GIALA	Elongation factor 1-alpha - Giardia lamblia (Giardia intestinalis)
TBAD_PHYPO	P50258|TBAD_PHYPO	Tubulin alpha-1A chain - Physarum polycephalum (Slime mold)
RPOA_EUGGR	P48337|RPOA_EUGGR	DNA-directed RNA polymerase subunit alpha - Euglena gracilis
KM11B_LEIIN	Q25297|KM11B_LEIIN	Kinetoplastid membrane protein 11B - Leishmania infantum
H2B_TRYCR	P27795|H2B_TRYCR	Histone H2B - Trypanosoma cruzi
EF1B_TRYCR	P34827|EF1B_TRYCR	25 kDa elongation factor 1-beta - Trypanosoma cruzi
EFTS_GALSU	P35019|EFTS_GALSU	Elongation factor Ts - Galdieria sulphuraria (Red alga)
MYSN_ACACA	P05659|MYSN_ACACA	Myosin-2 heavy chain, non muscle - Acanthamoeba castellanii (Amoeba)
YCF55_PORYE	Q1XDT4|YCF55_PORYE	Uncharacterized protein ycf55 - Porphyra yezoensis
HSP71_TRYCR	P20583|HSP71_TRYCR	Heat shock 70 kDa protein, mitochondrial precursor - Trypanosoma cruzi
CH60_GUITH	O78419|CH60_GUITH	60 kDa chaperonin - Guillardia theta (Cryptomonas phi)
NDUS1_DICCI	Q2LCP5|NDUS1_DICCI	NADH-ubiquinone oxidoreductase 75 kDa subunit - Dictyostelium citrinum (Slime mo

Probability Based Mowse Score

Ions score is -10*Log(P), where P is the probability that the observed match is a random event.
Individual ions scores > 23 indicate identity or extensive homology (p<0.05).
Protein scores are derived from ions scores as a non-probabilistic basis for ranking protein hits.

Score Distribution

Peptide Summary Report

Format As | Peptide Summary | Help

Significance threshold p< 0.05 | Max. number of hits AUTO

Standard scoring ○ MudPIT scoring ◉ | Ions score cut-off 0 | Show sub-sets ☐

Show pop-ups ◉ Suppress pop-ups ○ | Sort unassigned Decreasing Score | Require bold red ☐

Select All | Select None | Search Selected | ☐ **Error tolerant** | Archive Report

1. CH60_TRYCR **Mass:** 59374 **Score:** 243 **Queries matched:** 5
Q95046|CH60_TRYCR Chaperonin HSP60, mitochondrial precursor - Trypanosoma cruzi
☐ Check to include this hit in error tolerant search or archive report

	Query	Observed	Mr(expt)	Mr(calc)	Delta	Miss	Score	Expect	Rank	Peptide
☑	1503	**659.93**	**1317.85**	**1317.69**	**0.15**	**0**	**87**	**1.5e-08**	**1**	**R.NVIIEQSYGAPK.I**
☑	1512	**670.41**	**1338.81**	**1338.61**	**0.20**	**0**	**75**	**2e-07**	**1**	**R.GLIDGETSDYNR.E**

Query	Observed	Mr(expt)	Mr(calc)	Delta	Miss	Score	Expect	Rank	Peptide
380	696.47	1390.93	1390.73	0.19	0	50	0.00017	1	K.AELEDAFVLVSAK.K
1667	804.02	1606.03	1605.92	0.11	0	26	0.012	1	R.AAVQEGIVPGGGVALLR.A
1709	846.99	1691.97	1691.81	0.16	0	89	7e-09	1	K.VLENNDVTVGYDAQR.D

2. FCA1_TRYCR **Mass:** 23721 **Score:** 226 **Queries matched:** 7
P07749|FCA1_TRYCR Flagellar calcium-binding protein - Trypanosoma cruzi
☐ Check to include this hit in error tolerant search or archive report

Query	Observed	Mr(expt)	Mr(calc)	Delta	Miss	Score	Expect	Rank	Peptide
721	439.33	876.65	876.43	0.22	0	46	0.00019	1	K.LDEFTPR.V
970	495.35	988.69	988.52	0.16	0	45	0.00021	1	K.VEDPAALFK.E
1564	707.88	1413.75	1413.59	0.16	0	51	5.4e-05	1	K.LDADGDPDNVPESA.-
1565	707.88	1413.75	1413.59	0.16	0	(37)	0.0015	1	K.LDADGDPDNVPESA.-
1603	737.93	1473.84	1473.68	0.17	0	78	1.2e-07	1	K.GSEDFVEFLEFR.L
1768	607.92	1820.74	1820.84	-0.11	0	27	0.014	1	K.LCYDEVHSGCLEVLK.L + 2 Carbamidomethyl (C)
1832	979.63	1957.24	1956.92	0.32	0	62	3e-06	1	K.NGTGSVTFDEFAAWASAVK.L

3. UBIQ_TRYCR **Mass:** 8504 **Score:** 209 **Queries matched:** 5
P08565|UBIQ_TRYCR Ubiquitin - Trypanosoma cruzi
☐ Check to include this hit in error tolerant search or archive report

Query	Observed	Mr(expt)	Mr(calc)	Delta	Miss	Score	Expect	Rank	Peptide
1120	533.36	1064.70	1064.55	0.15	0	(31)	0.0041	1	R.TLADYNIQK.E
138	533.40	1064.79	1064.55	0.24	0	44	0.00062	1	R.TLADYNIQK.E
1126	534.41	1066.81	1066.61	0.20	0	46	0.00015	1	K.ESTLHLVLR.L
1734	874.52	1747.02	1746.89	0.13	0	99	6.8e-10	1	K.TIALEVESSDTIENVK.A
1735	583.36	1747.07	1746.89	0.18	0	(66)	1.6e-06	1	K.TIALEVESSDTIENVK.A

4. TBB_TRYBR **Mass:** 49672 **Score:** 179 **Queries matched:** 4
P04107|TBB_TRYBR Tubulin beta chain - Trypanosoma brucei rhodesiense
☐ Check to include this hit in error tolerant search or archive report

Query	Observed	Mr(expt)	Mr(calc)	Delta	Miss	Score	Expect	Rank	Peptide
274	627.12	1252.24	1252.77	-0.53	1	6	4.3	2	R.KLAVNLVPFPR.L
☑ 1516	671.43	1340.85	1340.64	0.21	0	87	1e-08	1	R.INVYFDEATGGR.Y
☑ 1589	723.93	1445.85	1445.68	0.17	0	41	0.0004	1	K.EVDEQMLNVQNK.N
☑ 1785	924.50	1846.98	1846.87	0.11	0	90	5.3e-09	1	R.EIVCVQAGQCGNQIGSK.F + 2 Carbamidomethyl (C)

Proteins matching the same set of peptides:

TBB_TRYCR **Mass:** 49541 **Score:** 179 **Queries matched:** 4

P08562|TBB_TRYCR Tubulin beta chain - Trypanosoma cruzi

---

5. G3PG_TRYCR **Mass:** 39036 **Score:** 149 **Queries matched:** 5

P22513|G3PG_TRYCR Glyceraldehyde-3-phosphate dehydrogenase, glycosomal - Trypanosoma cruzi

☐ Check to include this hit in error tolerant search or archive report

Query	Observed	Mr(expt)	Mr(calc)	Delta	Miss	Score	Expect	Rank	Peptide
☑ 580	406.29	810.57	810.41	0.17	0	15	0.14	1	K.LTGMSFR.V
☑ 606	412.81	823.61	823.43	0.18	0	3	2	1	K.AAAEGHLR.G
☑ 1547	692.96	1383.90	1383.77	0.13	0	28	0.009	1	R.AAAVNIIPSTTGAAK.A
☑ 205	596.60	1786.79	1786.95	-0.16	0	(59)	2.1e-05	1	R.VPTPDVSVVDLTFTAAR.D
☑ 1751	894.56	1787.10	1786.95	0.15	0	102	3.1e-10	1	R.VPTPDVSVVDLTFTAAR.D

---

6. RLA3_TRYCR **Mass:** 10919 **Score:** 139 **Queries matched:** 3

P26795|RLA3_TRYCR 60S acidic ribosomal protein P2-B - Trypanosoma cruzi

☐ Check to include this hit in error tolerant search or archive report

Query	Observed	Mr(expt)	Mr(calc)	Delta	Miss	Score	Expect	Rank	Peptide
☑ 1222	558.90	1115.78	1115.62	0.16	0	92	3.9e-09	1	R.SVATLVAEGAAK.M
155	558.93	1115.84	1115.62	0.22	0	(8)	3	2	R.SVATLVAEGAAK.M
☑ 1810	952.92	1903.84	1903.73	0.10	1	68	9.2e-07	1	K.KEEEEEDDDMGFGLFD.-

---

7. CALM_EUGGR **Mass:** 16845 **Score:** 139 **Queries matched:** 2

P11118|CALM_EUGGR Calmodulin - Euglena gracilis

☐ Check to include this hit in error tolerant search or archive report

	Query	Observed	Mr(expt)	Mr(calc)	Delta	Miss	Score	Expect	Rank	Peptide
☑	1524	**675.46**	**1348.90**	**1348.62**	**0.28**	**0**	**87**	**1.3e-08**	**1**	**K.LTDEEVDEMIR.E**
☑	1819	**964.50**	**1926.98**	**1926.85**	**0.13**	**0**	**73**	**3.3e-07**	**1**	**R.EADVDGDGQINYEEFVK.M**

Proteins matching the same set of peptides:

CALM_TRYBB **Mass:** 16828 **Score:** 139 **Queries matched:** 2
P69097|CALM_TRYBB Calmodulin - Trypanosoma brucei brucei

CALM_TRYBG **Mass:** 16828 **Score:** 139 **Queries matched:** 2
P69098|CALM_TRYBG Calmodulin - Trypanosoma brucei gambiense

CALM_TRYCR **Mass:** 16814 **Score:** 139 **Queries matched:** 2
P18061|CALM_TRYCR Calmodulin - Trypanosoma cruzi

---

8. CH60_TRYBB **Mass:** 59492 **Score:** 114 **Queries matched:** 3
Q37683|CH60_TRYBB Chaperonin HSP60, mitochondrial precursor - Trypanosoma brucei brucei

☐ Check to include this hit in error tolerant search or archive report

	Query	Observed	Mr(expt)	Mr(calc)	Delta	Miss	Score	Expect	Rank	Peptide
	1503	659.93	1317.85	1317.69	0.15	0	87	1.5e-08	1	R.NVIIEQSYGAPK.I
	380	696.47	1390.93	1390.73	0.19	0	50	0.00017	1	K.AELEDAFVLVSAK.K
☑	827	**925.21**	**1848.41**	**1848.89**	**-0.48**	**0**	**1**	**14**	**1**	**K.VLENTDAAVGYDAQLDR.Y**

---

9. TBA_TRYCR **Mass:** 49696 **Score:** 101 **Queries matched:** 2
Q27352|TBA_TRYCR Tubulin alpha chain - Trypanosoma cruzi

☐ Check to include this hit in error tolerant search or archive report

	Query	Observed	Mr(expt)	Mr(calc)	Delta	Miss	Score	Expect	Rank	Peptide
☑	1721	**572.73**	**1715.17**	**1714.91**	**0.26**	**0**	**(45)**	**0.00016**	**1**	**R.AVFLDLEPTVVDEIR.T**
☑	1722	**858.61**	**1715.21**	**1714.91**	**0.30**	**0**	**76**	**1.4e-07**	**1**	**R.AVFLDLEPTVVDEIR.T**

---

10. EF1A_EUGGR **Mass:** 48582 **Score:** 100 **Queries matched:** 3
P14963|EF1A_EUGGR Elongation factor 1-alpha - Euglena gracilis

☐ Check to include this hit in error tolerant search or archive report

Query	Observed	Mr(expt)	Mr(calc)	Delta	Miss	Score	Expect	Rank	Peptide
☑ 941	**488.38**	**974.76**	**974.54**	**0.21**	**0**	**59**	**1.1e-05**	**1**	**R.LPLQDVYK.I**
☑ 1047	**513.39**	**1024.76**	**1024.60**	**0.16**	**0**	**62**	**3e-06**	**1**	**K.IGGIGTVPVGR.V**
1368	602.39	1202.78	1202.65	0.12	1	7	1.7	2	K.ELEAEPKFIK.S

11. EF1AC_PORPU **Mass:** 49142 **Score:** 100 **Queries matched:** 3
P50256|EF1AC_PORPU Elongation factor 1-alpha C - Porphyra purpurea
☐ Check to include this hit in error tolerant search or archive report

Query	Observed	Mr(expt)	Mr(calc)	Delta	Miss	Score	Expect	Rank	Peptide
941	488.38	974.76	974.54	0.21	0	59	1.1e-05	1	R.LPLQDVYK.I
1047	513.39	1024.76	1024.60	0.16	0	62	3e-06	1	K.IGGIGTVPVGR.V
☑ 1372	**602.83**	**1203.64**	**1203.65**	**-0.02**	**2**	**4**	**2.5**	**1**	**K.KLEDSPKMIK.S + Oxidation (M)**

12. HSP70_BRELC **Mass:** 74041 **Score:** 94 **Queries matched:** 2
P16394|HSP70_BRELC Heat shock 70 kDa protein - Bremia lactucae (Lettuce downy mildew)
☐ Check to include this hit in error tolerant search or archive report

Query	Observed	Mr(expt)	Mr(calc)	Delta	Miss	Score	Expect	Rank	Peptide
☑ 1404	**614.92**	**1227.83**	**1227.62**	**0.21**	**0**	**69**	**8.1e-07**	**1**	**R.VEIIANDQGNR.T**
☑ 1609	**744.47**	**1486.92**	**1486.69**	**0.23**	**0**	**44**	**0.00038**	**1**	**R.TTPSYVAFTDTER.L**

13. RLA1_TRYCR **Mass:** 10747 **Score:** 90 **Queries matched:** 3
P26643|RLA1_TRYCR 60S acidic ribosomal protein P1 - Trypanosoma cruzi
☐ Check to include this hit in error tolerant search or archive report

Query	Observed	Mr(expt)	Mr(calc)	Delta	Miss	Score	Expect	Rank	Peptide
☑ 1220	**558.86**	**1115.70**	**1115.58**	**0.11**	**0**	**24**	**0.024**	**1**	**K.NVDINDVLSK.V**
1810	952.92	1903.84	1903.73	0.10	1	68	9.2e-07	1	K.KEEEEEDDDMGFGLFD.-
☑ 2072	**850.54**	**2548.60**	**2548.34**	**0.26**	**0**	**34**	**0.0019**	**1**	**K.VSFGGVAPAAGGATAAPAAAAAAAPAAAAK.K**

14. H2A1_LEIIN Mass: 13884 Score: 81 Queries matched: 2
P27891|H2A1_LEIIN Histone H2A.1 - Leishmania infantum
☐ Check to include this hit in error tolerant search or archive report

	Query	Observed	Mr(expt)	Mr(calc)	Delta	Miss	Score	Expect	Rank	Peptide
☑	489	759.42	758.41	758.43	-0.01	2	0	17	1	K.KGKATPSA.-
☑	1239	563.90	1125.78	1125.57	0.21	0	81	5e-08	1	R.HDDDIGTLLK.N

---

15. H2A_TRYCR Mass: 14357 Score: 75 Queries matched: 2
P35066|H2A_TRYCR Histone H2A - Trypanosoma cruzi
☐ Check to include this hit in error tolerant search or archive report

	Query	Observed	Mr(expt)	Mr(calc)	Delta	Miss	Score	Expect	Rank	Peptide
☑	1166	1090.01	1089.00	1088.60	0.40	2	1	16	1	-.MATPKQAAKK.A + Oxidation (M)
☑	1283	578.90	1155.79	1155.56	0.23	0	75	2.3e-07	1	R.HDDDLGMLLK.D

---

16. EF1A_TRYBB Mass: 49003 Score: 72 Queries matched: 2
P41166|EF1A_TRYBB Elongation factor 1-alpha - Trypanosoma brucei brucei
☐ Check to include this hit in error tolerant search or archive report

	Query	Observed	Mr(expt)	Mr(calc)	Delta	Miss	Score	Expect	Rank	Peptide
	1047	513.39	1024.76	1024.60	0.16	0	62	3e-06	1	K.IGGIGTVPVGR.V
☑	644	841.79	2522.36	2522.19	0.18	0	31	0.011	1	K.SIEMHHEQLAEATPGDNVGFNVK.N

---

17. HSP70_TRYCR Mass: 73762 Score: 69 Queries matched: 2
P05456|HSP70_TRYCR Heat shock 70 kDa protein - Trypanosoma cruzi
☐ Check to include this hit in error tolerant search or archive report

	Query	Observed	Mr(expt)	Mr(calc)	Delta	Miss	Score	Expect	Rank	Peptide
	1404	614.92	1227.83	1227.62	0.21	0	69	8.1e-07	1	R.VEIIANDQGNR.T
☑	1477	650.40	1298.79	1298.60	0.19	0	15	0.19	1	R.FEELCGELFR.G + Carbamidomethyl (C)

---

18. HSP70_ACHKL Mass: 71222 Score: 69 Queries matched: 2
P41753|HSP70_ACHKL Heat shock 70 kDa protein - Achlya klebsiana
☐ Check to include this hit in error tolerant search or archive report

	Query	Observed	Mr(expt)	Mr(calc)	Delta	Miss	Score	Expect	Rank	Peptide
☑	1364	**601.40**	**1200.78**	**1200.65**	**0.13**	**1**	**2**	**3.5**	**1**	**K.TMEPVEKVLR.D**
	1404	614.92	1227.83	1227.62	0.21	0	69	8.1e-07	1	R.VEIIANDQGNR.T

19. HSP70_LEIMA Mass: 56500 Score: 69 Queries matched: 1
P14834|HSP70_LEIMA Heat shock 70 kDa protein - Leishmania major
☐ Check to include this hit in error tolerant search or archive report

Query	Observed	Mr(expt)	Mr(calc)	Delta	Miss	Score	Expect	Rank	Peptide
1404	614.92	1227.83	1227.62	0.21	0	69	8.1e-07	1	R.LDIIANDQGNR.T

20. EF1A_ENTHI Mass: 47234 Score: 59 Queries matched: 4
P31018|EF1A_ENTHI Elongation factor 1-alpha - Entamoeba histolytica
☐ Check to include this hit in error tolerant search or archive report

	Query	Observed	Mr(expt)	Mr(calc)	Delta	Miss	Score	Expect	Rank	Peptide
	941	488.38	974.76	974.54	0.21	0	59	1.1e-05	1	R.LPLQDVYK.I
	148	**547.88**	**1093.76**	**1093.54**	**0.22**	**1**	**2**	**11**	**2**	**K.QERYEEIK.K**
	1233	**561.40**	**1120.79**	**1120.59**	**0.20**	**0**	**1**	**4.3**	**8**	**K.YAWVLDNLK.A**
☑	1474	**649.40**	**1296.78**	**1296.61**	**0.17**	**1**	**1**	**4.9**	**1**	**K.MDAIQYKQER.Y + Oxidation (M)**

21. EF1A_GIALA Mass: 43922 Score: 59 Queries matched: 1
Q08046|EF1A_GIALA Elongation factor 1-alpha - Giardia lamblia (Giardia intestinalis)
☐ Check to include this hit in error tolerant search or archive report

Query	Observed	Mr(expt)	Mr(calc)	Delta	Miss	Score	Expect	Rank	Peptide
941	488.38	974.76	974.54	0.21	0	59	1.1e-05	1	R.LPIQDVYK.I

22. TBAD_PHYPO Score: 53 Queries matched: 2

P50258|TBAD_PHYPO Tubulin alpha-1A chain - Physarum polycephalum (Slime mold)

☐ Check to include this hit in error tolerant search or archive report

	Query	Observed	Mr(expt)	Mr(calc)	Delta	Miss	Score	Expect	Rank	Peptide
	1721	572.73	1715.17	1714.91	0.26	0	(28)	0.0093	2	R.AVFLDLEPTVIDEVR.T
	1722	858.61	1715.21	1714.91	0.30	0	45	0.00019	2	R.AVFLDLEPTVIDEVR.T

---

23. RPOA_EUGGR **Mass:** 25348 **Score:** 46 **Queries matched:** 2

P48337|RPOA_EUGGR DNA-directed RNA polymerase subunit alpha - Euglena gracilis

☐ Check to include this hit in error tolerant search or archive report

	Query	Observed	Mr(expt)	Mr(calc)	Delta	Miss	Score	Expect	Rank	Peptide
☑	1108	**528.42**	**1054.82**	**1054.71**	**0.11**	**2**	**46**	**0.00014**	**1**	**K.ILLKIEAKK.A**
	1126	534.41	1066.81	1066.65	0.16	1	9	0.72	2	K.YLKIYVLR.S

---

24. KM11B_LEIIN **Mass:** 11169 **Score:** 45 **Queries matched:** 1

Q25297|KM11B_LEIIN Kinetoplastid membrane protein 11B - Leishmania infantum

☐ Check to include this hit in error tolerant search or archive report

	Query	Observed	Mr(expt)	Mr(calc)	Delta	Miss	Score	Expect	Rank	Peptide
☑	1194	**553.38**	**1104.75**	**1104.58**	**0.17**	**0**	**45**	**0.00023**	**1**	**K.FAELLEQQK.A**

Proteins matching the same set of peptides:

KM11C_LEIIN **Mass:** 11255 **Score:** 45 **Queries matched:** 1

Q25298|KM11C_LEIIN Kinetoplastid membrane protein 11C - Leishmania infantum

KM11_LEIDO **Mass:** 11254 **Score:** 45 **Queries matched:** 1

Q36736|KM11_LEIDO Kinetoplastid membrane protein 11 - Leishmania donovani

KM11_LEITR **Mass:** 11227 **Score:** 45 **Queries matched:** 1

O21436|KM11_LEITR Kinetoplastid membrane protein 11 - Leishmania tropica

KM11_TRYBB **Mass:** 11069 **Score:** 45 **Queries matched:** 1

P69300|KM11_TRYBB Kinetoplastid membrane protein 11 - Trypanosoma brucei brucei

KM11_TRYBR **Mass:** 11069 **Score:** 45 **Queries matched:** 1

P69301|KM11_TRYBR Kinetoplastid membrane protein 11 - Trypanosoma brucei rhodesiense

KM11_TRYCR Mass: 11004 Score: 45 Queries matched: 1
Q9U6Z1|KM11_TRYCR Kinetoplastid membrane protein 11 - Trypanosoma cruzi

---

25. H2B_TRYCR Mass: 12368 Score: 36 Queries matched: 1
P27795|H2B_TRYCR Histone H2B - Trypanosoma cruzi
☐ Check to include this hit in error tolerant search or archive report

	Query	Observed	Mr(expt)	Mr(calc)	Delta	Miss	Score	Expect	Rank	Peptide
☑	857	470.42	938.83	938.58	0.25	0	36	0.0016	1	R.LVLPADLAK.H

---

26. EF1B_TRYCR Mass: 24450 Score: 35 Queries matched: 1
P34827|EF1B_TRYCR 25 kDa elongation factor 1-beta - Trypanosoma cruzi
☐ Check to include this hit in error tolerant search or archive report

	Query	Observed	Mr(expt)	Mr(calc)	Delta	Miss	Score	Expect	Rank	Peptide
☑	105	507.03	1518.06	1517.85	0.21	1	35	0.0053	1	K.LFLGGTKPSKEDVK.L

---

27. EFTS_GALSU Score: 31 Queries matched: 1
P35019|EFTS_GALSU Elongation factor Ts - Galdieria sulphuraria (Red alga)
☐ Check to include this hit in error tolerant search or archive report

Query	Observed	Mr(expt)	Mr(calc)	Delta	Miss	Score	Expect	Rank	Peptide
1194	553.38	1104.75	1104.58	0.16	0	31	0.005	2	-.MSEISAQLVK.E

---

28. MYSN_ACACA Mass: 171097 Score: 29 Queries matched: 2
P05659|MYSN_ACACA Myosin-2 heavy chain, non muscle - Acanthamoeba castellanii (Amoeba)
☐ Check to include this hit in error tolerant search or archive report

	Query	Observed	Mr(expt)	Mr(calc)	Delta	Miss	Score	Expect	Rank	Peptide
☑	999	500.91	999.81	999.60	0.21	1	29	0.0083	1	K.LEKDLAALK.L
☑	1480	650.83	1299.64	1299.71	-0.07	1	12	0.42	1	K.CNGVLEGIRIAR.K

---

29. YCF55_PORYE Score: 29 Queries matched: 1
Q1XDT4|YCF55_PORYE Uncharacterized protein ycf55 - Porphyra yezoensis
☐ Check to include this hit in error tolerant search or archive report

Query	Observed	Mr(expt)	Mr(calc)	Delta	Miss	Score	Expect	Rank	Peptide
1404	614.92	1227.83	1227.70	0.13	0	29	0.0087	3	K.NNLIWNTIIK.N

---

30. HSP71_TRYCR Mass: 71102 Score: 28 Queries matched: 1
P20583|HSP71_TRYCR Heat shock 70 kDa protein, mitochondrial precursor - Trypanosoma cruzi
☐ Check to include this hit in error tolerant search or archive report

Query	Observed	Mr(expt)	Mr(calc)	Delta	Miss	Score	Expect	Rank	Peptide
☑ 1551	**695.41**	**1388.81**	**1388.73**	**0.08**	**0**	**28**	**0.009**	**1**	**K.EISEVVLVGGMTR.M**

---

31. CH60_GUITH Mass: 57367 Score: 27 Queries matched: 1
O78419|CH60_GUITH 60 kDa chaperonin - Guillardia theta (Cryptomonas phi)
☐ Check to include this hit in error tolerant search or archive report

Query	Observed	Mr(expt)	Mr(calc)	Delta	Miss	Score	Expect	Rank	Peptide
☑ 1193	**1104.65**	**1103.65**	**1103.56**	**0.09**	**2**	**27**	**0.014**	**1**	**K.SRCEQIRR.Q + Carbamidomethyl (C)**

---

32. NDUS1_DICCI Mass: 79554 Score: 24 Queries matched: 3
Q2LCP5|NDUS1_DICCI NADH-ubiquinone oxidoreductase 75 kDa subunit - Dictyostelium citrinum (Slime mo
☐ Check to include this hit in error tolerant search or archive report

Query	Observed	Mr(expt)	Mr(calc)	Delta	Miss	Score	Expect	Rank	Peptide
1199	554.36	1106.71	1106.65	0.06	1	4	2.1	2	K.MVFKVLLNK.K + Oxidation (M)
☑ 1489	**654.42**	**1306.82**	**1306.67**	**0.15**	**2**	**24**	**0.02**	**1**	**K.KIMEAGSWKNK.T + Oxidation (M)**
☑ 1490	**436.65**	**1306.92**	**1306.67**	**0.25**	**2**	**(1)**	**3.8**	**1**	**K.KIMEAGSWKNK.T + Oxidation (M)**

Peptide matches not assigned to protein hits: (no details means no match)

Query	Observed	Mr(expt)	Mr(calc)	Delta	Miss	Score	Expect	Rank	Peptide

☑	1339	593.94	1185.87	1185.62	0.25	0	22	0.039	1	EIDVIEEIAR
☑	711	437.80	873.58	873.48	0.10	0	22	0.056	1	VTVEDIAK
☑	1225	559.38	1116.74	1116.61	0.12	2	21	0.037	1	DREKEIISK
☑	1061	516.42	1030.83	1030.61	0.23	0	21	0.051	1	VIDLLAPYK
☑	1674	540.46	1618.35	1617.85	0.50	2	21	0.072	1	PIGYPLVKAMDKDR + Oxidation (M)
☑	1315	588.36	1174.70	1174.61	0.09	0	20	0.06	1	LTQESEDLLK
☑	1500	658.91	1315.81	1315.73	0.08	1	20	0.059	1	RTMFPVLDLPK
☑	1757	899.11	1796.21	1796.03	0.18	2	20	0.056	1	SRSIHKGPFIDVSLLK
☑	1478	650.41	1298.81	1298.72	0.09	1	18	0.093	1	QLEIEAKALER
☑	1259	572.40	1142.78	1142.63	0.15	2	18	0.082	1	SPEGKEKLQK
☑	20	283.12	846.34	846.39	-0.06	0	18	0.16	1	MYGYGLK + Oxidation (M)
☑	190	586.39	1170.77	1170.49	0.28	0	18	0.28	1	DFDTVCTEGK + Carbamidomethyl (C)
☑	1605	738.46	1474.90	1474.72	0.19	0	18	0.11	1	FIDCYLQFLEK + Carbamidomethyl (C)
☑	612	828.20	1654.39	1653.86	0.54	1	17	0.39	1	KLMVTGAQYMDLIR + Oxidation (M)
☑	1675	811.98	1621.95	1621.90	0.06	0	17	0.11	1	YGLPLIFEIETISK
☑	1615	751.04	1500.07	1499.85	0.22	2	16	0.18	1	ALDMKDVKQAILR
☑	147	547.83	1640.48	1640.94	-0.46	2	16	0.64	1	LARKEQCIALGAQLK
☑	1648	788.98	1575.95	1575.83	0.12	2	15	0.12	1	ASDVIGAVKKGVSDCK
☑	1601	734.95	1467.89	1467.86	0.02	2	15	0.18	1	ALSGGQKQRIAIAR
☑	902	963.48	962.47	962.49	-0.02	0	15	0.55	1	MSSNNLVAK
☑	1597	730.98	1459.95	1459.71	0.24	1	14	0.28	1	MEKEMMLYLLK + 2 Oxidation (M)
☑	1253	571.32	1140.62	1140.60	0.02	1	14	0.17	1	RSVGPGQDVAR
☑	1325	591.43	1180.85	1180.54	0.31	1	14	0.21	1	SIAPCKQCMK + Carbamidomethyl (C); Oxidation (M)
☑	611	827.51	826.50	826.39	0.11	0	14	0.23	1	SSLFMDK
☑	932	487.38	972.74	972.58	0.16	1	14	0.22	1	ELLPFAKR
☑	1542	691.42	1380.82	1380.69	0.12	1	13	0.31	1	ELESKGYEWLK
☑	1332	592.94	1183.87	1183.66	0.22	0	13	0.32	1	NVTAGANPISIK
☑	2036	814.34	2440.01	2440.20	-0.19	1	13	0.19	1	DPLNSIYMMAFSGARANISQVR
☑	1441	633.92	1265.82	1265.58	0.24	0	13	0.25	1	ICINLECIDCK
☑	1233	561.40	1120.79	1120.66	0.13	2	13	0.32	1	RNRHNIIAK
☑	155	558.93	1115.84	1115.67	0.17	1	13	1.1	1	RYVILLDPK
☑	295	642.60	1283.18	1283.69	-0.51	1	13	1.1	1	NLAREGNEIIR
☑	1171	545.86	1089.70	1089.56	0.14	1	12	0.42	1	AKVCGWIEGK
☑	1368	602.39	1202.78	1202.60	0.18	1	12	0.53	1	KINESMEPQK

☑	1412	412.31	1233.92	1233.73	0.19	2	12	0.37	1	LSSAYILRRR
☑	1753	896.02	1790.02	1790.02	0.00	2	12	0.36	1	SFALTREASLRTLGLR
☑	1350	597.38	1192.75	1192.60	0.14	1	12	0.41	1	NICKISSSWR
☑	1548	462.31	1383.91	1383.72	0.19	1	12	0.4	1	HGIEKVAEQVMK + Oxidation (M)
☑	1161	544.84	1087.66	1087.63	0.03	2	11	0.41	1	LASSKRISIN
☑	416	718.83	717.82	718.39	-0.57	0	11	1.1	1	LASETAK
☑	92	483.11	964.20	964.49	-0.29	0	11	1.3	1	IADAIYDGK
☑	1496	658.43	1314.84	1314.66	0.18	1	11	0.49	1	WLRDVPSDEAK
☑	1016	1007.71	3020.10	3019.54	0.56	2	11	1.5	1	SPKPVASCAMPVMDNMKIFTNTPLVKK + Carbamidomethyl (C); Oxidation (M)
☑	1440	633.40	1264.79	1264.70	0.09	1	11	0.55	1	TNSKVLDFTIK
☑	1257	571.42	1140.83	1140.60	0.23	1	11	0.35	1	RSVGPGQDVAR
☑	1250	570.34	1138.66	1138.60	0.06	1	11	0.51	1	NTSSTNKFIK
☑	1091	523.38	1044.74	1044.63	0.11	1	11	0.69	1	SLSALAKSIR
☑	2014	793.15	2376.42	2376.15	0.27	1	10	0.46	1	IIVDTYGGWGAHGGGAFSGKDPSK
☑	1549	694.37	1386.72	1386.74	-0.02	2	10	0.61	1	RARDVEMLELR
☑	1778	613.07	1836.17	1835.97	0.20	1	10	0.6	1	SFDLDVLKDSCALILK + Carbamidomethyl (C)
☑	1664	801.57	1601.12	1600.88	0.25	1	10	0.76	1	MLQIELQKWIQR + Oxidation (M)
☑	928	972.15	971.14	970.65	0.48	2	10	1.9	1	LITGAKKIK
☑	1365	601.87	1201.73	1201.67	0.06	1	10	0.7	1	ESKINTEILR
☑	1245	567.37	1132.72	1132.61	0.11	1	10	0.65	1	KTSEVTEALR
☑	622	833.29	2496.85	2496.37	0.47	2	10	2.2	1	LVSVPTMPRVPRQELMDFLLR
☑	1431	628.99	1255.96	1255.67	0.29	1	10	0.68	1	TLPGVAVLDDKE
☑	182	580.55	1159.09	1158.65	0.45	1	9	2.1	1	IQRTLTGTNR
☑	838	465.86	929.71	929.55	0.16	1	9	0.8	1	TLIEKAQK
☑	1396	610.92	1219.82	1219.68	0.14	0	9	0.61	1	SLLSNMISIVK + Oxidation (M)
☑	1614	750.46	1498.90	1498.70	0.20	2	9	0.7	1	ECKTCSLTETKEK
☑	1046	1025.63	1024.62	1024.63	-0.01	1	9	1.7	1	KYITIFLK
☑	1199	554.36	1106.71	1106.59	0.12	1	9	0.64	1	MPLYVNSKR
☑	1072	518.42	1034.83	1034.56	0.27	0	9	0.8	1	MDLFLQIR
☑	1235	561.87	1121.72	1121.64	0.08	1	9	0.73	1	RTINQVVHR
☑	91	482.12	1443.33	1442.85	0.48	0	9	2.1	1	VLSQLGSSLVTALR
☑	784	454.40	906.79	906.53	0.26	1	9	0.79	1	KGTVSIFR
☑	367	686.84	685.83	686.43	-0.60	0	9	1.7	1	ISVLQK
☑	1758	599.75	1796.23	1796.04	0.19	1	9	0.83	1	IKIAEIINDQATNILK

☑	1241	564.39	1126.77	1126.59	0.18	0	8	0.88	1	GDLGVEIPAEK
☑	1170	545.85	1089.68	1089.62	0.05	2	8	1.1	1	MAADLLKGKK + Oxidation (M)
☑	1886	686.78	2057.33	2057.92	-0.59	1	8	0.84	1	QEANTIVKNSSTDDDFMK + Oxidation (M)
☑	1656	794.45	1586.89	1586.88	0.01	1	8	0.92	1	VQAIKGTLIVCEGTR
☑	97	491.35	980.68	980.42	0.26	0	8	2.3	1	GMLCNAGSR + Carbamidomethyl (C); Oxidation (M)
☑	764	898.24	2691.68	2692.27	-0.59	0	8	3.3	1	VPALVEAGADVLCIDSSDGFSEWQK + Carbamidomethyl (C)
☑	330	663.36	1324.71	1324.68	0.03	1	8	3.4	1	FASKIMGTTDVR
☑	124	519.25	1554.72	1554.85	-0.13	1	8	2	1	MSDILFQLGYIKK
☑	1442	634.40	1266.78	1266.71	0.07	2	8	1.1	1	MKFSQRTLLK + Oxidation (M)
☑	1486	651.98	1301.94	1301.66	0.27	0	7	1.4	1	QSVNEPMITGVK
☑	1604	738.43	1474.84	1474.72	0.12	0	7	1.2	1	FIDCYLQFLEK + Carbamidomethyl (C)
☑	1090	1045.65	1044.64	1044.63	0.01	1	7	3.4	1	SLSALAKSIR
☑	1435	631.37	1260.73	1260.66	0.07	0	7	1.5	1	QCLTAMPAGLIK + Oxidation (M)
☑	197	591.36	1180.71	1180.66	0.05	0	7	3	1	FAVFASLLASR
☑	905	482.81	963.60	963.43	0.17	0	7	1.4	1	TNFDQDPK
☑	1532	682.93	1363.85	1363.67	0.19	1	7	1.2	1	LQEQLKEMSDK + Oxidation (M)
☑	59	379.48	756.94	756.43	0.52	0	7	3.8	1	LHGVGFK
☑	101	500.44	1498.30	1498.79	-0.49	1	7	3.6	1	DNQNIKIYNHLK
☑	1484	651.42	1300.82	1300.72	0.10	1	7	1.3	1	VLVNEKLMEAR
☑	1792	931.94	1861.86	1862.00	-0.14	2	7	1.1	1	AMRLSQHVIRYMTIK + Oxidation (M)
☑	1254	571.33	1140.64	1140.56	0.08	2	7	0.94	1	YDKRTDCIK
☑	1841	656.69	1967.04	1967.12	-0.07	2	7	1.1	1	RIVENAGNNGSIIIEKIK
☑	274	627.12	1252.24	1252.54	-0.30	0	7	4.2	1	ALQECNGDFEK
☑	1855	665.77	1994.28	1994.05	0.22	2	6	1.1	1	EQYRSISSSLAQKTIQR
☑	1938	1088.80	2175.59	2175.22	0.37	2	6	1.7	1	IQTRNAKLKPYDNINLFK
☑	826	924.86	923.85	923.42	0.43	0	6	3.8	1	AFCVGGDQK
☑	451	371.14	740.27	740.53	-0.26	1	6	0.79	1	ILKVIR
☑	1392	607.88	1213.75	1213.61	0.15	0	6	1.4	1	VDIIANDQGNR
☑	948	979.46	2935.35	2935.52	-0.17	1	6	3.7	1	GEDVVCVYVAVGQKAATVASIVTTLEEK + Carbamidomethyl (C)
☑	1775	613.02	1836.04	1835.97	0.07	1	6	1.5	1	SFDLDVLKDSCALILK + Carbamidomethyl (C)
☑	1298	582.84	1163.67	1163.65	0.01	2	6	1.7	1	KEYQLVEKK
☑	1204	555.32	1108.63	1108.61	0.02	1	6	1.3	1	EGISIRHAAR
☑	1553	695.94	1389.86	1389.67	0.19	1	6	1.6	1	MQSDSEPVVSRR
☑	1521	674.97	1347.92	1347.78	0.14	1	6	1.6	1	ADLTKFIVVTNK

☑	1289	579.88	1157.75	1157.68	0.07	0	6	1.8	1	ANVPFIVAISK
☑	1344	594.90	1187.79	1187.57	0.23	1	6	1.8	1	QMVTKFGMSK + 2 Oxidation (M)
☑	799	913.26	2736.76	2736.32	0.44	2	6	5.6	1	MQHNLIDNYQLRARSFGEEPYR
☑	1269	1149.95	3446.83	3446.82	0.00	1	6	3.9	1	GPTAKHLSSIICHFQQLNTIISTAHAIYLGR + Carbamidomethyl (C)
☑	1650	792.50	1582.98	1582.81	0.17	1	6	1.6	1	FPKTNSMIEELFK
☑	364	684.44	683.43	683.33	0.10	0	6	0.85	1	GMGYLK + Oxidation (M)
☑	1763	905.63	1809.24	1808.99	0.25	2	6	1.7	1	APGSIGMGTSPGRVLPGKK
☑	332	665.11	1328.20	1328.77	-0.56	1	5	6.8	1	EILLLATGESKR
☑	1780	919.65	1837.28	1836.91	0.37	2	5	2.2	1	GHSLADPDELRSRQEK
☑	334	666.33	665.32	665.34	-0.02	0	5	1.1	1	MTISSK
☑	245	615.75	614.75	615.33	-0.59	0	5	3.6	1	LEAQR
☑	1639	778.49	1554.96	1554.87	0.09	1	5	1.5	1	EGGRTVGAGVVLSIIQ
☑	1509	665.92	1329.83	1329.73	0.10	1	5	1.8	1	IKNENPLDIFK
☑	366	686.48	1370.95	1370.80	0.15	2	5	5.8	1	NVIMLLSPKKVN + Oxidation (M)
☑	2142	991.19	2970.56	2970.56	-0.01	1	5	1.3	1	IEFEILWIGQSELFYKGIESTLIDK
☑	189	586.33	1170.65	1170.67	-0.02	2	5	5.1	1	QLQKRLEEK
☑	173	572.28	571.27	571.37	-0.10	0	5	1.5	1	LAGALK
☑	1538	687.40	1372.78	1372.81	-0.03	1	5	2.1	1	VLGGKGISLAAMIK + Oxidation (M)
☑	1508	665.49	1328.97	1328.75	0.22	2	5	1.9	1	FYKSSVRTLTK
☑	1461	643.95	1285.88	1285.66	0.22	1	5	2.4	1	MIHIGNNTKSR + Oxidation (M)
☑	1443	1270.15	3807.42	3806.99	0.43	1	5	6.1	1	IHMDPLEIDLIILATSSPNDLFGSASQVQAKIGAAR + Oxidation (M)
☑	1099	1050.86	2099.71	2099.13	0.58	2	5	5.2	1	GLINQRIHSQDIICKFSK
☑	1463	644.99	1287.97	1287.69	0.28	1	5	1.9	1	LMCIRVLGGGNR
☑	192	586.89	585.89	586.29	-0.40	0	5	3.4	1	VCHTK
☑	1369	602.42	1202.83	1202.67	0.16	0	5	2.8	1	ESTLIMQLLR
☑	650	423.27	844.52	844.47	0.06	0	5	2.7	1	IIEQASGK
☑	1499	658.88	1315.76	1315.65	0.10	1	5	2.2	1	GISAGYSKNTYR
☑	444	735.89	2204.65	2205.18	-0.53	2	5	9.9	1	SCCVRIQHVHRNVFLLPK + Carbamidomethyl (C)
☑	242	614.86	1841.56	1840.99	0.57	2	4	10	1	ARNIAALPVSMGGCGIRR
☑	266	623.27	1866.78	1866.90	-0.12	0	4	5.9	1	EMYNSLGTPISGVAEGVK + Oxidation (M)
☑	1713	850.08	1698.14	1697.97	0.17	2	4	2.1	1	MASNQQLKIRIQLR
☑	1797	626.76	1877.27	1877.01	0.26	2	4	2.5	1	KNSLTVATFMLSKFYK
☑	148	547.88	1093.76	1093.58	0.18	1	4	7	1	ELKVDFSTR
☑	160	563.04	1686.09	1685.97	0.12	1	4	6.7	1	MGMSLQLLGINLRIK

☑	277	629.36	628.35	628.40	-0.05	2	4	2.7	1	RKTPK
☑	1481	650.89	1299.76	1299.76	-0.00	0	4	2.5	1	SLTLLEAAELIK
☑	1577	714.47	1426.92	1426.73	0.19	0	4	2.4	1	LIIQIGCYSDFR
☑	523	782.88	781.88	781.32	0.55	0	4	2.2	1	EFSDER
☑	1654	793.98	1585.94	1585.89	0.06	2	4	1.9	1	IIKVSEIMHGKTSK + Oxidation (M)
☑	146	546.82	545.81	546.28	-0.47	0	4	10	1	TLGWA
☑	373	691.96	690.96	690.41	0.55	1	4	4.9	1	VYPGKK
☑	537	789.85	788.84	789.42	-0.57	0	4	9.3	1	NQLLMR + Oxidation (M)
☑	559	797.75	1593.48	1593.91	-0.43	0	4	6.5	1	LNNVDIQKPDVVIK
☑	1465	645.31	1288.61	1288.71	-0.09	0	4	2.6	1	ITSLVLDQCLK + Carbamidomethyl (C)
☑	136	531.45	530.45	530.34	0.10	0	4	1.7	1	AIVTK
☑	1482	650.94	1299.86	1299.65	0.21	2	4	2.7	1	KRDLHSNSSEK
☑	412	716.16	715.16	715.36	-0.20	0	4	3.3	1	EGGNALR
☑	1506	665.41	1328.81	1328.72	0.09	1	4	2.5	1	QVDVFVKGPGAGR
☑	1527	679.40	1356.78	1356.76	0.02	0	4	2.9	1	SAVLQNSPSTIIK
☑	328	660.89	659.89	659.39	0.50	0	4	8	1	SIDVVK
☑	1377	603.91	1205.81	1205.68	0.13	2	4	2.7	1	KTEAAGFKAGVK
☑	763	898.12	1794.23	1794.82	-0.59	1	4	7.3	1	FWSSCHAAVKAAQDMK + Oxidation (M)
☑	279	630.99	629.98	630.31	-0.33	0	4	12	1	DNNLR
☑	454	741.92	2222.74	2223.06	-0.32	1	4	9.2	1	AMIDNRMNQSILISGESGAGK + 2 Oxidation (M)
☑	1633	772.43	1542.85	1542.74	0.11	0	4	2.3	1	GYISPYFVTDPER
☑	1454	1278.52	3832.54	3832.65	-0.12	0	3	6.2	1	MSTAQSDALTFECETGNYHTFCPISCVSWLYQK + Carbamidomethyl (C); Oxidation (M)
☑	1081	1040.43	2078.84	2079.08	-0.24	2	3	6	1	LDIGKLFGGLVGESESRMR + Oxidation (M)
☑	1497	658.58	1315.15	1315.56	-0.40	2	3	5.2	1	DKTGAGMMDCKK + 2 Oxidation (M)
☑	70	444.02	886.02	886.51	-0.49	2	3	9.3	1	KQATQRR
☑	456	743.48	742.47	742.48	-0.01	1	3	8.2	1	LVRTVR
☑	1191	1103.19	3306.56	3306.59	-0.03	2	3	8.3	1	MSGGDHLHSGTVVGKLEGDRAGTYGFINLMR + 2 Oxidation (M)
☑	1063	1032.56	3094.66	3094.57	0.08	0	3	7.1	1	VAVVDVQTVHGPPFFAALGGTCVNVGCVPK + 2 Carbamidomethyl (C)
☑	1265	574.01	1146.02	1145.68	0.33	2	3	5.3	1	VKDLPGVKYK
☑	1322	590.39	1178.78	1178.56	0.22	1	3	2.2	1	KNWCLIDCK + Carbamidomethyl (C)
☑	1634	773.49	1544.97	1544.78	0.19	0	3	3.2	1	HSCLIINFESNLR
☑	1196	1106.12	1105.12	1104.63	0.49	1	3	10	1	NITKIVFDR
☑	1703	843.65	1685.29	1684.87	0.42	0	3	4.2	1	ELFSLLSDFYLNPK
☑	1579	714.49	1426.96	1426.85	0.11	2	3	3.1	1	LDSIKLDVLGKAR

☑	1285	578.95	1155.88	1155.68	0.20	2	3	3.3	1	CRIPVEKALK
☑	1345	594.94	1187.87	1187.57	0.30	1	3	3.4	1	QMVTKFGMSK + 2 Oxidation (M)
☑	802	457.89	913.77	913.52	0.25	0	3	3.8	1	LAQAELAAK
☑	61	423.63	1267.88	1267.71	0.17	0	3	8	1	ADPAAETLVLIR
☑	1507	665.48	1328.94	1328.79	0.15	2	3	3	1	IVSRRSNGLTVK
☑	180	579.06	1156.11	1155.62	0.49	2	3	11	1	NSAPVRSRNR
☑	1366	601.90	1201.79	1201.58	0.21	1	3	3.5	1	TNMFSKLSMK + Oxidation (M)
☑	1341	594.43	1186.84	1186.69	0.15	1	3	3.8	1	VIRVGINGFGR
☑	2093	882.16	2643.46	2643.19	0.27	1	3	2.4	1	SLIENYDCYEVKTVGDSFMIACK + Oxidation (M)
☑	301	645.87	644.87	644.39	0.48	0	3	18	1	GNTLLK
☑	314	653.18	1956.53	1956.98	-0.45	1	3	10	1	FRQYVNQGLNLYDATR
☑	1288	579.86	1157.70	1157.68	0.02	0	3	3.7	1	ANVPFIVAISK
☑	134	530.88	529.87	530.31	-0.43	0	3	4.3	1	TLLNA
☑	313	652.35	651.34	651.37	-0.03	1	3	7.9	1	YSVRK
☑	703	872.51	1743.00	1742.84	0.16	0	3	8.5	1	IVTEALNALSCMEHR + Carbamidomethyl (C)
☑	1281	578.46	1154.91	1154.74	0.17	1	3	3.6	1	IILETAKVIR
☑	1346	595.89	1189.76	1189.59	0.16	1	2	3.5	1	KVLCMGVGGGNV + Carbamidomethyl (C)
☑	81	447.67	1339.98	1339.78	0.20	1	2	6.7	1	EIVDIVLDRIR
☑	1982	762.49	2284.46	2284.18	0.28	1	2	3.2	1	YFTAASHALLMNVFASRTLR + Oxidation (M)
☑	520	780.37	2338.08	2338.24	-0.15	0	2	8.5	1	ICPIETPEGPNAGLIGSLAIFAR
☑	219	603.76	602.76	602.33	0.43	0	2	16	1	DLIDK
☑	1681	818.47	1634.93	1634.83	0.10	1	2	3	1	MVSSLANELREGTTK
☑	377	693.91	692.90	693.37	-0.47	0	2	7.6	1	FEGLTK
☑	565	802.18	2403.51	2403.21	0.31	1	2	14	1	VQSSEGTITCEVQQLLGDNKVR
☑	1074	1036.35	2070.68	2071.14	-0.47	1	2	12	1	QAVPLIRASKPYVSTGQEK
☑	1176	1092.64	1091.63	1091.59	0.04	1	2	9.7	1	NLDKTFIEL
☑	299	644.36	1286.71	1286.71	-0.00	1	2	12	1	IANQQVMKALR + Oxidation (M)
☑	755	893.89	1785.76	1785.90	-0.14	1	2	12	1	MKTPITEAIAAADNQGR
☑	806	459.33	916.65	916.57	0.08	2	2	4.6	1	VTKVTKGGK
☑	1479	650.42	1298.82	1298.62	0.20	0	2	4.1	1	MSVSQVTDASFK
☑	1203	1109.59	1108.58	1108.45	0.13	0	2	8.9	1	DVNDEQSMR + Oxidation (M)
☑	1209	1111.87	3332.58	3332.45	0.13	1	2	10	1	IYDTCIGCTQCVRACPTDVLEMVPWDGCK + 2 Carbamidomethyl (C)
☑	337	666.89	1997.65	1998.05	-0.40	1	2	12	1	MTIQKFITYTPLDLNGK + Oxidation (M)
☑	980	497.34	992.66	992.52	0.14	1	2	4.6	1	EYPELSKK

☑	1770	611.08	1830.22	1829.96	0.26	2	2	4.1	1	QGVEEELAMLKEKQAK
☑	1622	755.88	1509.75	1509.91	-0.15	1	2	4	1	VLPNKLLGVLAMAR + Oxidation (M)
☑	270	624.81	1247.61	1247.61	-0.01	0	2	15	1	LDEFQEQLAR
☑	436	731.28	2190.83	2191.09	-0.25	1	2	14	1	YDSMQASLLADELKYLGFK
☑	1449	638.43	1274.84	1274.68	0.16	1	2	3.7	1	DGLIDTAVKTSR
☑	1755	597.96	1790.86	1791.00	-0.14	1	2	3.9	1	VPVKVEVVGAPAAVDWR
☑	1844	985.52	1969.04	1968.95	0.09	0	2	3.4	1	LMSFSSLPLWTDASAEAK + Oxidation (M)
☑	1889	689.39	2065.14	2065.06	0.08	1	2	3.5	1	TDIERGMVLAQPGTISPHK + Oxidation (M)
☑	968	988.90	2963.67	2963.41	0.26	2	2	12	1	MPKSAIHPDWYPNAKVYCDGQLVMK + Carbamidomethyl (C); Oxidation (M)
☑	1267	1148.29	2294.56	2294.10	0.46	1	2	12	1	QGDIVPGNSHFDTTKGHIESR
☑	1583	718.87	1435.73	1435.74	-0.02	1	2	3.5	1	STITVELMNLMKG
☑	1545	692.42	1382.83	1382.80	0.02	2	2	3.9	1	VHDGIIFQAKKK
☑	640	840.16	839.15	839.44	-0.29	1	2	11	1	RAGFAYR
☑	1576	713.42	1424.82	1424.71	0.11	0	2	3.4	1	FSNSPIFLVCNK + Carbamidomethyl (C)
☑	1291	580.45	1158.89	1158.70	0.19	2	2	4	1	TLKLSDLKNK
☑	1205	555.35	1108.69	1108.61	0.08	1	2	3.6	1	EGISIRHAAR
☑	1069	1034.60	3100.76	3100.43	0.33	1	2	10	1	CNWGIDGTTGKLVDMEEFGVWEPFSVK + Carbamidomethyl (C)
☑	977	496.84	991.66	991.69	-0.03	2	2	5.4	1	IHKKIILK
☑	1376	603.91	1205.80	1205.63	0.16	0	2	4.5	1	QVVTLCSITDK
☑	1528	679.48	1356.94	1356.67	0.27	1	2	4.8	1	NLDFKSVDTYR
☑	1114	1060.64	1059.63	1059.57	0.07	1	2	4.1	1	SVQKLQDSR
☑	139	534.36	1066.71	1066.48	0.24	0	2	9.4	1	EMAQGPSGFK + Oxidation (M)
☑	929	486.67	971.33	971.53	-0.21	1	1	4.3	1	INTCRLPR
☑	1994	776.47	2326.38	2326.15	0.22	1	1	3.3	1	METHFHEWQKSLQLIQQK + Oxidation (M)
☑	1815	640.72	1919.14	1919.03	0.10	2	1	3.3	1	KLLNQMELLLSTCKNR + Oxidation (M)
☑	908	965.40	2893.18	2893.28	-0.10	0	1	11	1	EGIEQVECASTVCQNDNSCPIIADVEK
☑	1541	690.45	1378.88	1378.72	0.16	2	1	3.9	1	VSPGRDRHGVSGR
☑	913	484.35	966.69	966.50	0.19	1	1	3.5	1	DKLSYVDK
☑	1096	1049.17	3144.49	3144.60	-0.11	2	1	14	1	VFWRGVCEELIWFLRGETNAHVLADK + Carbamidomethyl (C)
☑	1367	602.38	1202.75	1202.63	0.12	0	1	6.2	1	MLAQLASNDIK
☑	1299	1165.07	3492.19	3492.27	-0.08	0	1	16	1	CTCPMCCENDCFYTSCDVETGSCIPWPK + 6 Carbamidomethyl (C); Oxidation (M)
☑	1668	804.98	1607.94	1607.83	0.10	1	1	4.4	1	MEIILFDNKINSR + Oxidation (M)
☑	2153	1016.96	3047.85	3047.54	0.31	1	1	3.1	1	VYYPGEIIFDNIKITQPSLCEHINNK
☑	1118	1062.48	3184.41	3184.43	-0.03	2	1	11	1	ILASFFSFNHDQKNDNSEDNRSEDDLK

☑	1303	583.40	1164.79	1164.64	0.16	1	1	3.7	1	ELRHENIVR
☑	1169	545.84	1089.67	1089.65	0.01	2	1	5.6	1	AKEIFIKNK
☑	1599	731.96	1461.91	1461.87	0.03	2	1	3.8	1	QLKQGLFMLLKK + Oxidation (M)
☑	896	961.03	1920.05	1919.91	0.13	1	1	20	1	KELDPNSESEVIEEFR
☑	1343	1188.70	1187.69	1187.63	0.06	1	1	5.2	1	KLMYTFLEK + Oxidation (M)
☑	1874	1013.60	2025.19	2025.09	0.10	1	1	3.6	1	KSHIIYDSPNIELEVLR
☑	263	621.92	620.91	620.31	0.60	0	1	15	1	TSGQTK
☑	1054	1029.63	3085.88	3085.39	0.49	0	1	12	1	NIIGSDAEDVCMCGPEGMIEYACKPALLK + Oxidation (M)
☑	1744	883.98	1765.95	1765.91	0.03	2	1	4.4	1	QQVTEFERTKLMTR
☑	787	908.70	907.70	907.40	0.30	0	1	15	1	GMLCNAGSR
☑	96	491.34	1471.00	1470.71	0.29	1	1	12	1	RFCTGGMSLGALSR + Oxidation (M)
☑	775	903.92	902.91	903.38	-0.47	0	1	8.7	1	DYFATCK + Carbamidomethyl (C)
☑	1569	710.44	1418.86	1418.74	0.12	1	1	4.5	1	VEICDTGIGISKK + Carbamidomethyl (C)
☑	1122	533.86	1065.71	1065.57	0.14	1	1	5.2	1	YLTIKADDK
☑	1790	621.15	1860.43	1859.88	0.55	2	1	7.5	1	MVQEAEKNAAQDKENR
☑	1612	747.45	1492.88	1492.79	0.09	0	1	3.8	1	VQINASNIVSFSSK
☑	1024	1013.70	3038.08	3038.52	-0.44	0	1	15	1	VAVIDVQTHHGPPHYAALGGTCVNVGCVPK
☑	246	616.56	615.56	615.32	0.24	0	1	26	1	CAIPR + Carbamidomethyl (C)
☑	878	952.13	2853.38	2853.31	0.06	1	1	15	1	YFPIFCGWMVGDEFGGVVVREDTSK + Oxidation (M)
☑	1595	726.94	1451.87	1451.90	-0.03	2	1	3.9	1	LAPLIKILWRVM
☑	336	666.82	1331.62	1331.75	-0.13	2	1	17	1	KLIVLDKTCDK + Carbamidomethyl (C)
☑	1868	1011.10	2020.18	2020.00	0.18	1	1	3.4	1	VDDGTNAPGRGAPAGGIPSSPK
☑	722	877.66	2629.96	2630.25	-0.29	2	1	18	1	DFMLNKSNIRNMCVIAHVDHGK + Carbamidomethyl (C); 2 Oxidation (M)
☑	2051	830.56	2488.65	2488.21	0.44	2	1	6.3	1	DGMSIRFDDNAAVIINKENNPR
☑	1600	734.41	1466.81	1466.91	-0.10	1	1	4.7	1	ALITRLVLQVWR
☑	1958	1126.16	2250.30	2250.09	0.21	2	0	3.1	1	AASGGFEKGLDRENEPVLSMK + Oxidation (M)
☑	1307	1167.59	3499.76	3499.76	-0.00	2	0	11	1	QSVCEPMQTGITAIDSMIPIGRGQRELIIGDR + Oxidation (M)
☑	914	967.72	966.71	966.48	0.24	0	0	4.2	1	ADISYATAR
☑	1825	648.43	1942.26	1941.89	0.37	1	0	4.6	1	MAEPFSTILGTDGSGGRCK + Oxidation (M)
☑	1689	830.93	1659.85	1659.89	-0.04	2	0	5.6	1	VNGRLFEVDKVQEK
☑	207	598.92	1195.82	1195.63	0.19	1	0	13	1	KIGMTQIFDK + Oxidation (M)
☑	1060	1031.66	1030.65	1030.61	0.05	0	0	6.1	1	VIDLLAPYK
☑	1655	529.66	1585.95	1585.89	0.06	2	0	4.5	1	IIKVSEIMHGKTSK + Oxidation (M)
☑	188	585.41	1168.81	1168.62	0.19	1	0	15	1	LYKAYTPGTR

☑	262	621.32	1860.95	1860.87	0.08	1	0	15	1	MSTKEVDEQMHAVQTK
☑	408	713.84	712.83	712.42	0.40	0	0	18	1	ISLAPGR
☑	1167	1090.29	3267.83	3267.64	0.19	1	0	19	1	SIKLHPLVCSAFNADFDGDQMGVHIPLSLK + Oxidation (M)
☑	1535	683.51	1365.01	1364.64	0.36	0	0	4.6	1	IACLECGSSQLNK
☑	1218	558.37	1114.73	1114.67	0.06	1	0	5.7	1	TIAKQINSLK
☑	626	834.43	833.42	833.46	-0.04	0	0	16	1	QVDVFVK
☑	158	561.52	1121.02	1120.59	0.43	0	0	23	1	SILQSDGVFR
☑	1076	519.34	1036.67	1036.53	0.14	1	0	4.5	1	TFISDGNKR
☑	1928	720.16	2157.47	2157.99	-0.51	1	0	5.8	1	YGLMGGEGVTMDSAKMQQQK
☑	397	705.59	1409.16	1408.70	0.46	1	0	20	1	LMRVCGLEMSVR + Oxidation (M)
☑	1354	597.85	1193.69	1193.57	0.12	1	0	3.6	1	QRCFLSNNGR
☑	2110	905.17	2712.49	2712.29	0.20	2	0	4.8	1	KRAYDCDITYITNSELGFDYLR + Carbamidomethyl (C)
☑	1638	776.96	1551.91	1551.84	0.07	0	0	5.9	1	GMVLAKPGSITPHTK + Oxidation (M)
☑	1677	542.39	1624.15	1623.87	0.28	2	0	5.8	1	MNLLKHCVVNGPRK + Oxidation (M)
☑	1505	1321.07	1320.06	1320.59	-0.53	2	0	15	1	GGGDRGGRGTGGFGGG
☑	1	*217.30*	*216.29*							
☑	2	*221.12*	*220.11*							
☑	3	*226.23*	*225.22*							
☑	4	*227.03*	*226.02*							
☑	5	*228.35*	*227.35*							
☑	6	*229.07*	*228.06*							
☑	7	*229.18*	*228.17*							
☑	8	*229.24*	*228.23*							
☑	9	*235.22*	*234.21*							
☑	10	*237.29*	*236.28*							
☑	11	*239.15*	*238.14*							
☑	12	*239.18*	*238.17*							
☑	13	*247.10*	*246.09*							
☑	14	*256.26*	*255.25*							
☑	15	*257.30*	*256.29*							
☑	16	*269.03*	*268.03*							
☑	17	*269.04*	*268.03*							
☑	18	*269.05*	*268.04*							
☑	19	*269.25*	*268.25*							

☑	21	285.03	284.02
☑	22	285.03	284.02
☑	23	285.03	284.02
☑	24	285.06	284.05
☑	25	285.06	284.05
☑	26	285.08	284.07
☑	27	285.08	284.08
☑	28	285.13	284.12
☑	29	288.35	287.34
☑	30	296.90	295.90
☑	31	297.04	296.04
☑	32	297.10	296.09
☑	33	297.12	296.11
☑	34	297.16	296.16
☑	35	297.18	296.18
☑	36	298.27	297.26
☑	37	299.09	298.08
☑	38	299.09	298.09
☑	39	299.16	298.15
☑	40	299.20	298.20
☑	41	300.25	299.25
☑	42	300.46	299.46
☑	43	351.00	349.99
☑	44	355.14	354.13
☑	45	355.14	354.13
☑	46	355.28	354.28
☑	47	371.07	370.06
☑	48	371.08	370.08
☑	49	371.09	370.09
☑	50	371.11	370.10
☑	51	371.16	370.15
☑	52	371.16	370.15
☑	53	371.17	370.17
☑	54	371.18	370.17

☑	55	*371.18*	*370.17*
☑	56	*371.19*	*370.18*
☑	57	*371.19*	*370.18*
☑	58	*371.32*	*370.31*
☑	60	*388.99*	*387.98*
☑	62	*427.40*	*426.40*
☑	63	*429.03*	*428.02*
☑	64	*429.15*	*428.15*
☑	65	*429.17*	*428.17*
☑	66	*429.18*	*428.17*
☑	67	*429.21*	*428.20*
☑	68	*429.21*	*428.21*
☑	69	*429.22*	*428.21*
☑	71	*445.16*	*444.15*
☑	72	*445.17*	*444.16*
☑	73	*445.18*	*444.17*
☑	74	*445.19*	*444.18*
☑	75	*445.21*	*444.20*
☑	76	*445.25*	*444.24*
☑	77	*445.25*	*444.24*
☑	78	*445.27*	*444.26*
☑	79	*445.27*	*444.27*
☑	80	*446.11*	*445.10*
☑	82	*448.89*	*447.88*
☑	83	*449.69*	*448.68*
☑	84	*449.97*	*448.96*
☑	85	*453.91*	*452.91*
☑	86	*455.25*	*454.24*
☑	87	*460.58*	*459.57*
☑	88	*461.73*	*460.72*
☑	89	*472.04*	*471.03*
☑	90	*479.31*	*478.30*
☑	93	*486.09*	*485.09*
☑	94	*486.95*	*485.94*

☑	95	488.07	487.06
☑	98	492.45	491.44
☑	99	494.36	493.35
☑	100	496.42	495.41
☑	102	503.09	502.08
☑	103	503.17	502.16
☑	104	505.30	504.29
☑	106	507.11	506.10
☑	107	507.67	506.67
☑	108	508.39	507.38
☑	109	509.85	508.84
☑	110	510.67	509.66
☑	111	512.09	511.08
☑	112	512.26	511.25
☑	113	257.24	512.46
☑	114	513.71	512.71
☑	115	514.43	513.43
☑	116	515.40	514.39
☑	117	515.43	514.42
☑	118	517.00	515.99
☑	119	518.93	517.92
☑	120	519.15	518.15
☑	121	519.16	518.16
☑	122	519.17	518.16
☑	123	519.20	518.19
☑	125	519.25	518.24
☑	126	519.35	518.34
☑	127	523.39	522.38
☑	128	523.83	522.82
☑	129	524.34	523.33
☑	130	524.81	523.80
☑	131	525.08	524.07
☑	132	528.38	527.38
☑	133	528.99	527.99

☑	135	531.33	530.32
☑	137	533.33	532.32
☑	140	536.58	535.57
☑	141	538.19	537.18
☑	142	538.28	537.27
☑	143	542.61	541.60
☑	144	543.96	542.95
☑	145	544.02	543.01
☑	149	548.55	547.54
☑	150	550.06	549.06
☑	151	552.39	551.38
☑	152	552.64	551.64
☑	153	554.04	553.03
☑	154	554.11	553.10
☑	156	559.66	558.66
☑	157	560.64	559.63
☑	159	562.97	561.96
☑	161	563.35	562.34
☑	162	563.58	562.57
☑	163	565.23	564.23
☑	164	567.17	566.16
☑	165	567.30	566.30
☑	166	567.41	566.40
☑	167	568.70	567.69
☑	168	569.00	567.99
☑	169	569.84	568.83
☑	170	571.03	570.02
☑	171	571.61	570.61
☑	172	571.70	570.69
☑	174	572.91	571.90
☑	175	573.09	572.08
☑	176	576.38	575.37
☑	177	576.90	575.89
☑	178	578.40	577.39

☑	179	578.98	577.97
☑	181	579.87	578.86
☑	183	582.09	581.08
☑	184	582.10	581.09
☑	185	582.95	581.94
☑	186	584.38	583.37
☑	187	584.42	583.41
☑	191	586.84	585.83
☑	193	587.39	586.38
☑	194	587.67	586.67
☑	195	589.31	588.30
☑	196	590.36	589.35
☑	198	591.90	590.89
☑	199	592.60	591.59
☑	200	593.14	592.13
☑	201	594.31	593.30
☑	202	594.45	593.45
☑	203	595.03	594.03
☑	204	596.56	595.55
☑	206	597.98	596.97
☑	208	599.13	598.13
☑	209	599.44	598.44
☑	210	599.75	598.74
☑	211	599.80	598.79
☑	212	599.89	598.89
☑	213	600.15	599.14
☑	214	601.92	600.91
☑	215	602.84	601.83
☑	216	603.38	602.37
☑	217	603.61	602.60
☑	218	603.66	602.65
☑	220	603.95	602.95
☑	221	604.05	603.05
☑	222	604.07	603.06

☑	223	604.10	603.10
☑	224	604.72	603.71
☑	225	606.98	605.97
☑	226	606.98	605.97
☑	227	607.39	606.38
☑	228	608.02	607.01
☑	229	608.07	607.06
☑	230	608.31	607.31
☑	231	608.36	607.35
☑	232	609.38	608.38
☑	233	609.64	608.63
☑	234	609.79	608.79
☑	235	611.09	610.08
☑	236	611.93	610.92
☑	237	611.94	610.93
☑	238	612.02	611.02
☑	239	612.80	611.80
☑	240	613.79	612.79
☑	241	613.87	612.86
☑	243	615.02	614.02
☑	244	615.37	614.36
☑	247	617.29	616.29
☑	248	617.62	616.61
☑	249	618.54	617.54
☑	250	618.72	617.71
☑	251	618.82	617.81
☑	252	618.86	617.85
☑	253	619.01	618.00
☑	254	619.02	618.01
☑	255	619.09	618.09
☑	256	619.65	618.65
☑	257	619.89	618.89
☑	258	620.26	619.26
☑	259	620.39	619.38

☑	260	620.48	619.48
☑	261	620.74	619.74
☑	264	622.11	621.10
☑	265	622.80	621.80
☑	267	623.47	622.46
☑	268	623.54	622.53
☑	269	624.79	623.79
☑	271	625.33	624.33
☑	272	626.46	625.45
☑	273	627.08	626.07
☑	275	628.01	627.00
☑	276	628.65	627.64
☑	278	630.64	629.64
☑	280	632.08	631.07
☑	281	633.20	632.19
☑	282	633.95	632.94
☑	283	635.01	634.01
☑	284	637.34	636.33
☑	285	637.37	636.36
☑	286	637.46	636.45
☑	287	637.73	636.73
☑	288	639.09	638.08
☑	289	639.55	638.55
☑	290	640.96	639.96
☑	291	641.16	640.16
☑	292	641.92	640.91
☑	293	641.95	640.94
☑	294	642.38	641.37
☑	296	643.04	642.03
☑	297	643.54	642.54
☑	298	644.21	643.20
☑	300	645.03	644.02
☑	302	646.35	645.34
☑	303	646.60	645.60

☑	304	647.47	646.47
☑	305	647.66	646.65
☑	306	649.74	648.73
☑	307	649.91	648.91
☑	308	650.46	649.45
☑	309	650.50	649.49
☑	310	650.82	649.82
☑	311	651.16	650.15
☑	312	651.33	650.32
☑	315	653.95	652.94
☑	316	654.00	652.99
☑	317	654.09	653.08
☑	318	654.29	653.29
☑	319	327.77	653.52
☑	320	327.81	653.61
☑	321	654.70	653.69
☑	322	656.08	655.07
☑	323	657.11	656.10
☑	324	657.27	656.26
☑	325	657.32	656.31
☑	326	657.43	656.42
☑	327	658.06	657.06
☑	329	661.97	660.96
☑	331	664.47	663.46
☑	333	665.38	664.37
☑	335	666.65	665.64
☑	338	666.95	665.94
☑	339	667.13	666.12
☑	340	667.27	666.27
☑	341	669.45	668.45
☑	342	672.18	671.18
☑	343	672.31	671.31
☑	344	672.66	671.65
☑	345	673.44	672.44

☑	346	673.84	672.84
☑	347	675.33	674.32
☑	348	675.62	674.61
☑	349	675.77	674.77
☑	350	676.74	675.73
☑	351	677.42	676.42
☑	352	678.38	677.37
☑	353	678.54	677.54
☑	354	679.07	678.06
☑	355	681.52	680.52
☑	356	682.11	681.10
☑	357	682.16	681.15
☑	358	682.41	681.40
☑	359	682.55	681.55
☑	360	683.34	682.33
☑	361	683.35	682.34
☑	362	683.40	682.40
☑	363	683.64	682.63
☑	365	685.73	684.72
☑	368	687.62	686.61
☑	369	345.03	688.05
☑	370	689.95	688.95
☑	371	690.02	689.01
☑	372	690.90	689.89
☑	374	692.53	691.53
☑	375	693.12	692.11
☑	376	693.79	692.78
☑	378	694.79	693.78
☑	379	694.84	693.83
☑	381	697.46	696.45
☑	382	698.03	697.02
☑	383	698.42	697.41
☑	384	698.79	697.78
☑	385	699.87	698.86

☑	386	700.49	699.48
☑	387	701.02	700.02
☑	388	702.55	701.54
☑	389	702.84	701.83
☑	390	703.18	702.18
☑	391	703.57	702.56
☑	392	235.24	702.70
☑	393	703.96	702.95
☑	394	704.55	703.54
☑	395	704.71	703.70
☑	396	705.30	704.29
☑	398	706.69	705.68
☑	399	709.52	708.52
☑	400	710.08	709.08
☑	401	710.70	709.69
☑	402	711.10	710.09
☑	403	711.28	710.27
☑	404	712.57	711.56
☑	405	713.23	712.23
☑	406	713.71	712.70
☑	407	713.78	712.77
☑	409	714.77	713.76
☑	410	715.14	714.13
☑	411	239.08	714.23
☑	413	717.60	716.59
☑	414	717.89	716.88
☑	415	718.47	717.46
☑	417	718.96	717.95
☑	418	720.05	719.04
☑	419	720.24	719.24
☑	420	720.29	719.29
☑	421	720.40	719.40
☑	422	722.16	721.15
☑	423	722.42	721.41

☑	424	723.33	722.33
☑	425	723.36	722.35
☑	426	723.52	722.51
☑	427	723.62	722.62
☑	428	724.31	723.30
☑	429	724.38	723.37
☑	430	724.96	723.95
☑	431	728.32	727.31
☑	432	728.85	727.84
☑	433	729.26	728.26
☑	434	729.54	728.53
☑	435	730.05	729.04
☑	437	732.23	731.22
☑	438	732.57	731.56
☑	439	733.20	732.20
☑	440	733.40	732.39
☑	441	734.32	733.31
☑	442	735.01	734.00
☑	443	735.18	734.17
☑	445	737.47	736.46
☑	446	737.69	736.68
☑	447	738.28	737.27
☑	448	739.63	738.62
☑	449	740.06	739.05
☑	450	371.14	740.26
☑	452	741.45	740.44
☑	453	741.58	740.57
☑	455	742.47	741.46
☑	457	743.89	742.88
☑	458	744.04	743.03
☑	459	744.08	743.07
☑	460	745.90	744.89
☑	461	746.03	745.02
☑	462	746.20	745.19

☑	463	746.31	745.30
☑	464	746.72	745.72
☑	465	747.12	746.11
☑	466	747.51	746.50
☑	467	747.70	746.69
☑	468	749.56	748.55
☑	469	751.58	750.57
☑	470	751.89	750.88
☑	471	752.50	751.49
☑	472	752.74	751.73
☑	473	753.00	751.99
☑	474	753.18	752.17
☑	475	754.10	753.09
☑	476	755.39	754.39
☑	477	755.68	754.68
☑	478	755.77	754.76
☑	479	755.80	754.79
☑	480	756.39	755.38
☑	481	756.67	755.67
☑	482	757.15	756.15
☑	483	757.43	756.43
☑	484	757.71	756.70
☑	485	758.45	757.44
☑	486	758.84	757.84
☑	487	759.06	758.05
☑	488	759.33	758.32
☑	490	759.52	758.52
☑	491	760.22	759.21
☑	492	760.32	759.31
☑	493	760.86	759.85
☑	494	761.10	760.10
☑	495	761.52	760.51
☑	496	762.27	761.26
☑	497	762.52	761.51

☑	498	762.96	761.95
☑	499	763.48	762.47
☑	500	764.03	763.02
☑	501	765.02	764.01
☑	502	765.79	764.79
☑	503	766.01	765.00
☑	504	766.28	765.27
☑	505	766.53	765.52
☑	506	766.90	765.89
☑	507	767.46	766.45
☑	508	768.96	767.95
☑	509	769.55	768.55
☑	510	769.89	768.89
☑	511	770.47	769.47
☑	512	771.98	770.97
☑	513	773.46	772.45
☑	514	773.70	772.69
☑	515	774.30	773.30
☑	516	774.55	773.55
☑	517	776.54	775.54
☑	518	777.42	776.41
☑	519	780.13	779.12
☑	521	780.59	779.58
☑	522	782.74	781.73
☑	524	784.55	783.55
☑	525	784.99	783.98
☑	526	785.11	784.10
☑	527	785.53	784.53
☑	528	785.59	784.58
☑	529	785.74	784.73
☑	530	786.99	785.98
☑	531	787.01	786.00
☑	532	787.53	786.53
☑	533	788.04	787.03

☑	534	788.92	787.91
☑	535	788.95	787.94
☑	536	789.78	788.78
☑	538	790.38	789.37
☑	539	791.63	790.62
☑	540	791.98	790.97
☑	541	791.98	790.97
☑	542	792.27	791.26
☑	543	792.52	791.52
☑	544	792.57	791.56
☑	545	793.47	792.46
☑	546	793.55	792.54
☑	547	793.60	792.59
☑	548	793.96	792.95
☑	549	793.97	792.96
☑	550	794.39	793.38
☑	551	795.05	794.04
☑	552	795.23	794.22
☑	553	796.37	795.36
☑	554	796.64	795.63
☑	555	796.69	795.68
☑	556	797.23	796.22
☑	557	797.31	796.30
☑	558	797.49	796.49
☑	560	798.54	797.53
☑	561	799.37	798.36
☑	562	799.43	798.42
☑	563	799.47	798.46
☑	564	799.88	798.87
☑	566	802.48	801.47
☑	567	803.90	802.89
☑	568	804.06	803.05
☑	569	804.14	803.13
☑	570	804.53	803.53

☑	571	804.59	803.58
☑	572	269.00	803.97
☑	573	805.23	804.22
☑	574	806.12	805.11
☑	575	807.18	806.17
☑	576	807.27	806.26
☑	577	810.28	809.28
☑	578	810.51	809.50
☑	579	811.55	810.54
☑	581	811.76	810.75
☑	582	812.41	811.40
☑	583	812.89	811.88
☑	584	813.05	812.05
☑	585	813.34	812.33
☑	586	814.05	813.04
☑	587	814.84	813.84
☑	588	814.98	813.98
☑	589	815.00	813.99
☑	590	815.38	814.37
☑	591	816.09	815.08
☑	592	816.50	815.49
☑	593	816.51	815.50
☑	594	816.56	815.55
☑	595	817.24	816.23
☑	596	817.30	816.29
☑	597	819.27	818.26
☑	598	819.60	818.60
☑	599	819.89	818.89
☑	600	820.03	819.03
☑	601	820.48	819.47
☑	602	821.27	820.26
☑	603	821.84	820.83
☑	604	822.90	821.89
☑	605	824.57	823.57

☑	607	413.34	824.67
☑	608	826.15	825.14
☑	609	826.41	825.40
☑	610	827.32	826.32
☑	613	414.79	827.56
☑	614	828.84	827.83
☑	615	828.89	827.88
☑	616	829.33	828.32
☑	617	829.82	828.81
☑	618	831.67	830.66
☑	619	831.84	830.83
☑	620	833.15	832.14
☑	621	833.20	832.19
☑	623	833.41	832.41
☑	624	833.44	832.43
☑	625	833.61	832.60
☑	627	835.00	834.00
☑	628	835.44	834.44
☑	629	836.61	835.60
☑	630	836.68	835.68
☑	631	836.77	835.76
☑	632	837.56	836.56
☑	633	837.81	836.80
☑	634	838.15	837.14
☑	635	839.47	838.46
☑	636	420.32	838.63
☑	637	839.66	838.65
☑	638	839.67	838.67
☑	639	840.02	839.01
☑	641	840.47	839.46
☑	642	840.48	839.47
☑	643	840.52	839.51
☑	645	842.49	841.49
☑	646	842.57	841.56

☑	647	421.85	841.68
☑	648	843.82	842.81
☑	649	844.41	843.40
☑	651	845.95	844.94
☑	652	847.46	846.45
☑	653	848.48	847.47
☑	654	848.97	847.96
☑	655	849.61	848.60
☑	656	850.21	849.20
☑	657	850.65	849.64
☑	658	851.20	850.19
☑	659	852.06	851.06
☑	660	285.02	852.04
☑	661	285.06	852.16
☑	662	285.13	852.37
☑	663	853.77	852.76
☑	664	854.28	853.27
☑	665	855.47	854.46
☑	666	855.73	854.72
☑	667	856.73	855.72
☑	668	857.18	856.17
☑	669	429.19	856.37
☑	670	857.87	856.86
☑	671	858.63	857.63
☑	672	860.01	859.00
☑	673	860.68	859.68
☑	674	860.90	859.90
☑	675	861.04	860.03
☑	676	861.45	860.44
☑	677	861.72	860.71
☑	678	862.54	861.53
☑	679	862.76	861.75
☑	680	863.38	862.37
☑	681	863.43	862.42

☑	682	863.52	862.51
☑	683	863.54	862.54
☑	684	432.33	862.64
☑	685	864.12	863.11
☑	686	864.15	863.14
☑	687	865.10	864.09
☑	688	865.83	864.82
☑	689	865.96	864.95
☑	690	866.44	865.44
☑	691	866.80	865.79
☑	692	868.14	867.14
☑	693	869.30	868.30
☑	694	435.27	868.53
☑	695	869.61	868.60
☑	696	869.94	868.93
☑	697	870.79	869.79
☑	698	870.80	869.79
☑	699	871.04	870.03
☑	700	871.40	870.39
☑	701	871.54	870.53
☑	702	872.05	871.04
☑	704	872.59	871.58
☑	705	873.04	872.03
☑	706	873.16	872.16
☑	707	874.06	873.06
☑	708	874.08	873.07
☑	709	874.31	873.31
☑	710	874.47	873.46
☑	712	874.71	873.70
☑	713	875.33	874.32
☑	714	876.38	875.38
☑	715	876.60	875.59
☑	716	876.87	875.86
☑	717	876.91	875.90

☑	718	877.24	876.24
☑	719	877.59	876.58
☑	720	877.66	876.65
☑	723	878.26	877.25
☑	724	878.27	877.26
☑	725	879.05	878.05
☑	726	879.75	878.74
☑	727	880.38	879.38
☑	728	880.71	879.70
☑	729	881.13	880.12
☑	730	881.29	880.28
☑	731	881.46	880.45
☑	732	882.63	881.63
☑	733	882.65	881.64
☑	734	883.34	882.33
☑	735	883.48	882.47
☑	736	883.55	882.55
☑	737	885.21	884.20
☑	738	885.30	884.29
☑	739	885.55	884.54
☑	740	886.48	885.47
☑	741	444.23	886.44
☑	742	888.13	887.12
☑	743	888.39	887.38
☑	744	888.92	887.92
☑	745	889.03	888.02
☑	746	889.25	888.24
☑	747	889.33	888.32
☑	748	890.34	889.33
☑	749	890.48	889.47
☑	750	890.57	889.57
☑	751	892.39	891.39
☑	752	892.96	891.95
☑	753	893.37	892.37

☑	754	893.53	892.52
☑	756	893.94	892.93
☑	757	894.84	893.84
☑	758	895.56	894.55
☑	759	896.10	895.09
☑	760	896.58	895.57
☑	761	449.28	896.54
☑	762	897.63	896.62
☑	765	898.44	897.43
☑	766	898.70	897.69
☑	767	899.21	898.20
☑	768	899.38	898.37
☑	769	899.48	898.47
☑	770	902.67	901.66
☑	771	902.89	901.89
☑	772	903.23	902.22
☑	773	903.37	902.37
☑	774	903.86	902.85
☑	776	904.54	903.53
☑	777	904.71	903.70
☑	778	904.81	903.80
☑	779	905.10	904.09
☑	780	905.58	904.57
☑	781	905.77	904.76
☑	782	906.13	905.13
☑	783	907.43	906.43
☑	785	907.85	906.84
☑	786	908.39	907.38
☑	788	908.96	907.95
☑	789	909.17	908.16
☑	790	909.57	908.57
☑	791	909.77	908.76
☑	792	910.23	909.22
☑	793	910.57	909.56

☑	794	910.86	909.85
☑	795	911.92	910.92
☑	796	912.08	911.07
☑	797	912.68	911.67
☑	798	912.73	911.72
☑	800	914.02	913.01
☑	801	914.22	913.21
☑	803	915.05	914.05
☑	804	915.22	914.21
☑	805	915.76	914.76
☑	807	459.61	917.21
☑	808	918.39	917.39
☑	809	919.57	918.56
☑	810	919.81	918.81
☑	811	920.17	919.17
☑	812	920.33	919.32
☑	813	921.72	920.71
☑	814	922.07	921.07
☑	815	922.10	921.09
☑	816	922.12	921.11
☑	817	922.12	921.12
☑	818	922.14	921.13
☑	819	922.20	921.19
☑	820	922.53	921.53
☑	821	922.79	921.78
☑	822	923.05	922.04
☑	823	924.00	923.00
☑	824	924.21	923.20
☑	825	924.30	923.30
☑	828	925.52	924.51
☑	829	925.67	924.67
☑	830	926.26	925.26
☑	831	464.38	926.75
☑	832	928.11	927.10

☑	833	928.41	927.40
☑	834	928.99	927.98
☑	835	929.22	928.21
☑	836	929.90	928.89
☑	837	930.35	929.34
☑	839	931.00	929.99
☑	840	931.46	930.45
☑	841	931.64	930.64
☑	842	931.87	930.86
☑	843	931.93	930.93
☑	844	932.73	931.72
☑	845	933.36	932.36
☑	846	935.11	934.10
☑	847	935.13	934.13
☑	848	936.56	935.55
☑	849	936.95	935.94
☑	850	937.23	936.23
☑	851	937.32	936.31
☑	852	937.63	936.62
☑	853	938.45	937.44
☑	854	939.22	938.22
☑	855	939.53	938.52
☑	856	939.53	938.53
☑	858	939.90	938.89
☑	859	940.44	939.44
☑	860	941.51	940.51
☑	861	941.61	940.60
☑	862	941.66	940.65
☑	863	942.93	941.93
☑	864	943.13	942.13
☑	865	943.56	942.55
☑	866	945.48	944.48
☑	867	945.61	944.60
☑	868	945.81	944.80

☑	869	946.23	945.22
☑	870	946.34	945.33
☑	871	473.87	945.72
☑	872	947.28	946.28
☑	873	948.03	947.03
☑	874	949.31	948.30
☑	875	949.40	948.39
☑	876	949.53	948.53
☑	877	950.12	949.11
☑	879	952.22	951.21
☑	880	954.21	953.20
☑	881	954.71	953.70
☑	882	955.21	954.20
☑	883	955.54	954.54
☑	884	956.56	955.55
☑	885	956.71	955.70
☑	886	957.28	956.27
☑	887	958.10	957.09
☑	888	958.35	957.34
☑	889	958.89	957.88
☑	890	959.10	958.09
☑	891	959.70	958.70
☑	892	480.49	958.96
☑	893	960.08	959.07
☑	894	960.26	959.25
☑	895	960.45	959.45
☑	897	961.11	960.10
☑	898	961.26	960.26
☑	899	962.73	961.72
☑	900	962.76	961.76
☑	901	962.78	961.77
☑	903	964.06	963.05
☑	904	482.60	963.18
☑	906	964.63	963.62

☑	907	964.73	963.72
☑	909	965.65	964.64
☑	910	966.26	965.25
☑	911	966.91	965.90
☑	912	484.30	966.58
☑	915	967.84	966.83
☑	916	968.09	967.08
☑	917	968.81	967.80
☑	918	968.86	967.85
☑	919	969.46	968.45
☑	920	970.37	969.36
☑	921	970.76	969.75
☑	922	970.80	969.79
☑	923	971.04	970.03
☑	924	971.41	970.41
☑	925	971.42	970.42
☑	926	971.87	970.86
☑	927	972.10	971.09
☑	930	973.17	972.17
☑	931	973.28	972.28
☑	933	973.77	972.77
☑	934	974.75	973.74
☑	935	974.81	973.80
☑	936	975.11	974.10
☑	937	975.17	974.17
☑	938	975.52	974.51
☑	939	975.57	974.56
☑	940	975.73	974.72
☑	942	976.08	975.07
☑	943	976.75	975.74
☑	944	977.49	976.48
☑	945	489.67	977.33
☑	946	978.71	977.70
☑	947	978.98	977.97

☑	949	979.46	978.46
☑	950	979.49	978.48
☑	951	980.07	979.06
☑	952	981.05	980.05
☑	953	491.11	980.21
☑	954	982.13	981.12
☑	955	982.23	981.22
☑	956	982.49	981.48
☑	957	982.70	981.69
☑	958	983.36	982.36
☑	959	983.55	982.54
☑	960	492.70	983.38
☑	961	984.42	983.42
☑	962	984.92	983.92
☑	963	985.06	984.06
☑	964	985.82	984.81
☑	965	986.70	985.69
☑	966	987.83	986.83
☑	967	988.65	987.64
☑	969	494.99	987.97
☑	971	990.19	989.18
☑	972	990.99	989.98
☑	973	991.54	990.53
☑	974	496.34	990.66
☑	975	991.91	990.90
☑	976	992.61	991.60
☑	978	993.45	992.44
☑	979	993.66	992.66
☑	981	993.77	992.76
☑	982	993.85	992.84
☑	983	993.99	992.98
☑	984	994.42	993.41
☑	985	995.16	994.16
☑	986	498.29	994.56

☑	987	997.34	996.34
☑	988	997.42	996.41
☑	989	499.42	996.82
☑	990	998.26	997.26
☑	991	998.45	997.45
☑	992	998.46	997.45
☑	993	999.06	998.05
☑	994	999.15	998.15
☑	995	999.52	998.51
☑	996	999.67	998.67
☑	997	999.91	998.90
☑	998	1000.21	999.20
☑	1000	1001.65	1000.65
☑	1001	1002.07	1001.06
☑	1002	1002.76	1001.75
☑	1003	502.27	1002.52
☑	1004	1003.72	1002.72
☑	1005	1004.43	1003.43
☑	1006	1004.58	1003.57
☑	1007	1004.64	1003.63
☑	1008	502.91	1003.81
☑	1009	1005.09	1004.08
☑	1010	1005.51	1004.50
☑	1011	1005.91	1004.90
☑	1012	1006.05	1005.04
☑	1013	1006.09	1005.08
☑	1014	1006.39	1005.39
☑	1015	1006.98	1005.97
☑	1017	1008.33	1007.32
☑	1018	1008.34	1007.33
☑	1019	1010.40	1009.40
☑	1020	1011.03	1010.02
☑	1021	1011.34	1010.33
☑	1022	1011.48	1010.48

☑	1023	1011.54	1010.53
☑	1025	1014.03	1013.02
☑	1026	1014.14	1013.13
☑	1027	1014.33	1013.32
☑	1028	1015.39	1014.38
☑	1029	1015.96	1014.95
☑	1030	1016.53	1015.53
☑	1031	1016.99	1015.98
☑	1032	1017.24	1016.23
☑	1033	1017.48	1016.48
☑	1034	1017.85	1016.85
☑	1035	1018.49	1017.48
☑	1036	1019.61	1018.60
☑	1037	1020.21	1019.20
☑	1038	1022.15	1021.14
☑	1039	1022.55	1021.54
☑	1040	1023.16	1022.16
☑	1041	1023.20	1022.19
☑	1042	1023.53	1022.52
☑	1043	1023.72	1022.71
☑	1044	1024.15	1023.14
☑	1045	1025.08	1024.07
☑	1048	1026.64	1025.64
☑	1049	1026.70	1025.70
☑	1050	1028.37	1027.36
☑	1051	1028.77	1027.77
☑	1052	1029.20	1028.19
☑	1053	1029.36	1028.36
☑	1055	1030.18	1029.17
☑	1056	1030.53	1029.53
☑	1057	1030.96	1029.95
☑	1058	1031.32	1030.31
☑	1059	1031.64	1030.63
☑	1062	1031.95	1030.94

☑	1064	517.35	1032.69
☑	1065	1033.82	1032.82
☑	1066	1033.85	1032.84
☑	1067	1033.91	1032.90
☑	1068	1034.25	1033.24
☑	1070	1034.62	1033.62
☑	1071	1035.66	1034.66
☑	1073	1035.92	1034.91
☑	1075	1036.80	1035.79
☑	1077	1038.51	1037.50
☑	1078	1038.73	1037.72
☑	1079	1039.28	1038.27
☑	1080	1040.02	1039.01
☑	1082	1041.08	1040.08
☑	1083	1041.10	1040.09
☑	1084	521.39	1040.76
☑	1085	1041.83	1040.82
☑	1086	1042.03	1041.02
☑	1087	1043.01	1042.00
☑	1088	1043.77	1042.76
☑	1089	1044.05	1043.04
☑	1092	1046.59	1045.59
☑	1093	1048.15	1047.15
☑	1094	1048.37	1047.36
☑	1095	1048.71	1047.71
☑	1097	1049.27	1048.26
☑	1098	1050.07	1049.06
☑	1100	1051.44	1050.43
☑	1101	1051.73	1050.72
☑	1102	526.38	1050.76
☑	1103	1052.32	1051.31
☑	1104	527.39	1052.77
☑	1105	1054.02	1053.01
☑	1106	1054.08	1053.07

☑	1107	1054.27	1053.26
☑	1109	1056.36	1055.35
☑	1110	1056.46	1055.46
☑	1111	1058.39	1057.38
☑	1112	1058.62	1057.62
☑	1113	1060.04	1059.03
☑	1115	1061.13	1060.13
☑	1116	1061.31	1060.30
☑	1117	1061.90	1060.89
☑	1119	1064.65	1063.64
☑	1121	533.43	1064.86
☑	1123	1066.96	1065.95
☑	1124	1067.19	1066.19
☑	1125	1067.20	1066.19
☑	1127	1068.06	1067.05
☑	1128	1068.43	1067.42
☑	1129	1068.68	1067.67
☑	1130	1069.60	1068.59
☑	1131	1070.09	1069.09
☑	1132	1071.36	1070.35
☑	1133	1071.72	1070.72
☑	1134	1072.13	1071.12
☑	1135	1072.21	1071.20
☑	1136	1072.40	1071.39
☑	1137	1072.91	1071.90
☑	1138	1073.09	1072.09
☑	1139	1074.57	1073.56
☑	1140	1074.65	1073.64
☑	1141	1075.72	1074.71
☑	1142	1076.12	1075.11
☑	1143	1078.00	1077.00
☑	1144	1079.05	1078.04
☑	1145	540.06	1078.10
☑	1146	1079.82	1078.81

☑	1147	1079.87	1078.86
☑	1148	1080.15	1079.15
☑	1149	1081.89	1080.89
☑	1150	1082.13	1081.12
☑	1151	1083.55	1082.55
☑	1152	1084.22	1083.21
☑	1153	1084.74	1083.73
☑	1154	1084.99	1083.98
☑	1155	1085.18	1084.17
☑	1156	1085.54	1084.53
☑	1157	1086.17	1085.17
☑	1158	1087.72	1086.72
☑	1159	1087.99	1086.98
☑	1160	1088.34	1087.34
☑	1162	1089.14	1088.14
☑	1163	1089.19	1088.19
☑	1164	1089.73	1088.72
☑	1165	1089.83	1088.82
☑	1168	545.78	1089.55
☑	1172	1090.87	1089.86
☑	1173	1091.12	1090.11
☑	1174	1091.17	1090.16
☑	1175	1092.38	1091.37
☑	1177	1093.54	1092.53
☑	1178	1097.08	1096.08
☑	1179	1097.20	1096.19
☑	1180	1097.33	1096.32
☑	1181	1098.24	1097.24
☑	1182	549.90	1097.79
☑	1183	1099.24	1098.23
☑	1184	1099.89	1098.89
☑	1185	550.53	1099.04
☑	1186	1100.61	1099.60
☑	1187	550.87	1099.72

☑	1188	1101.27	1100.26
☑	1189	1101.83	1100.82
☑	1190	1102.24	1101.23
☑	1192	1103.59	1102.59
☑	1195	1106.06	1105.05
☑	1197	1106.65	1105.64
☑	1198	1106.97	1105.96
☑	1200	1108.42	1107.42
☑	1201	1108.63	1107.62
☑	1202	555.26	1108.51
☑	1206	1109.70	1108.69
☑	1207	1109.86	1108.85
☑	1208	556.40	1110.80
☑	1210	1111.90	1110.90
☑	1211	1112.05	1111.04
☑	1212	1113.42	1112.41
☑	1213	1113.50	1112.49
☑	1214	1113.64	1112.64
☑	1215	557.42	1112.83
☑	1216	1114.67	1113.67
☑	1217	1115.45	1114.45
☑	1219	1116.25	1115.24
☑	1221	1116.72	1115.71
☑	1223	1116.84	1115.83
☑	1224	1117.02	1116.02
☑	1226	1118.06	1117.05
☑	1227	1118.76	1117.75
☑	1228	1119.09	1118.08
☑	1229	1119.40	1118.39
☑	1230	1120.16	1119.15
☑	1231	1121.04	1120.03
☑	1232	1121.67	1120.66
☑	1234	1122.40	1121.40
☑	1236	1123.67	1122.66

☑	1237	1124.18	1123.17
☑	1238	563.16	1124.32
☑	1240	1127.30	1126.29
☑	1242	564.90	1127.79
☑	1243	1132.85	1131.84
☑	1244	1133.10	1132.10
☑	1246	1133.82	1132.82
☑	1247	1134.38	1133.37
☑	1248	1135.68	1134.67
☑	1249	1138.65	1137.64
☑	1251	1140.66	1139.65
☑	1252	1140.79	1139.78
☑	1255	1141.70	1140.69
☑	1256	571.40	1140.80
☑	1258	1142.65	1141.65
☑	1260	572.61	1143.20
☑	1261	1144.86	1143.85
☑	1262	1145.61	1144.60
☑	1263	1146.70	1145.69
☑	1264	1146.73	1145.73
☑	1266	1147.06	1146.05
☑	1268	1148.83	1147.82
☑	1270	1150.19	1149.18
☑	1271	1150.32	1149.31
☑	1272	1150.74	1149.73
☑	1273	1151.00	1149.99
☑	1274	1151.65	1150.65
☑	1275	1151.86	1150.85
☑	1276	576.46	1150.91
☑	1277	576.75	1151.48
☑	1278	1152.52	1151.51
☑	1279	576.77	1151.52
☑	1280	577.38	1152.75
☑	1282	578.89	1155.76

☑	1284	1156.83	1155.83
☑	1286	1157.54	1156.53
☑	1287	1158.42	1157.42
☑	1290	1159.70	1158.69
☑	1292	1161.08	1160.08
☑	1293	1161.74	1160.74
☑	1294	1161.83	1160.82
☑	1295	581.96	1161.91
☑	1296	1164.43	1163.42
☑	1297	1164.59	1163.58
☑	1300	1165.37	1164.36
☑	1301	1165.47	1164.46
☑	1302	1165.75	1164.75
☑	1304	583.90	1165.78
☑	1305	1166.89	1165.88
☑	1306	1167.36	1166.36
☑	1308	1168.70	1167.69
☑	1309	1169.04	1168.03
☑	1310	585.90	1169.79
☑	1311	1171.90	1170.89
☑	1312	1173.56	1172.56
☑	1313	1173.58	1172.57
☑	1314	1174.73	1173.73
☑	1316	1177.72	1176.71
☑	1317	1177.85	1176.85
☑	1318	1178.00	1176.99
☑	1319	1178.09	1177.08
☑	1320	1178.24	1177.23
☑	1321	1178.84	1177.83
☑	1323	1179.87	1178.86
☑	1324	1181.20	1180.20
☑	1326	1182.52	1181.51
☑	1327	591.90	1181.78
☑	1328	1182.80	1181.79

☑	1329	1183.05	1182.05
☑	1330	1183.84	1182.83
☑	1331	592.56	1183.10
☑	1333	593.08	1184.14
☑	1334	1185.34	1184.33
☑	1335	1185.71	1184.71
☑	1336	1186.36	1185.35
☑	1337	1186.50	1185.49
☑	1338	593.94	1185.86
☑	1340	594.40	1186.79
☑	1342	1188.01	1187.00
☑	1347	1192.17	1191.16
☑	1348	1192.27	1191.26
☑	1349	1193.69	1192.68
☑	1351	1193.88	1192.87
☑	1352	1194.14	1193.14
☑	1353	1194.55	1193.54
☑	1355	1196.07	1195.06
☑	1356	1196.82	1195.82
☑	1357	599.43	1196.84
☑	1358	1198.31	1197.30
☑	1359	599.90	1197.78
☑	1360	1199.39	1198.39
☑	1361	600.20	1198.39
☑	1362	1200.78	1199.77
☑	1363	1201.63	1200.62
☑	1370	1204.00	1203.00
☑	1371	1204.38	1203.38
☑	1373	1205.30	1204.29
☑	1374	1205.79	1204.78
☑	1375	1205.95	1204.95
☑	1378	1207.39	1206.38
☑	1379	1207.60	1206.59
☑	1380	1208.51	1207.50

☑	1381	604.91	1207.80
☑	1382	604.96	1207.91
☑	1383	1209.77	1208.76
☑	1384	1210.52	1209.52
☑	1385	1211.02	1210.01
☑	1386	606.39	1210.77
☑	1387	1212.47	1211.47
☑	1388	1213.51	1212.50
☑	1389	1214.17	1213.16
☑	1390	1214.32	1213.31
☑	1391	1214.72	1213.71
☑	1393	1215.85	1214.84
☑	1394	1217.35	1216.34
☑	1395	1219.90	1218.89
☑	1397	611.44	1220.87
☑	1398	1221.96	1220.96
☑	1399	611.67	1221.33
☑	1400	1222.61	1221.61
☑	1401	1225.89	1224.89
☑	1402	1226.83	1225.82
☑	1403	1227.31	1226.30
☑	1405	1231.35	1230.34
☑	1406	1231.47	1230.46
☑	1407	617.00	1231.99
☑	1408	1234.04	1233.03
☑	1409	1234.27	1233.26
☑	1410	1234.48	1233.47
☑	1411	1234.54	1233.53
☑	1413	1236.05	1235.04
☑	1414	1236.46	1235.45
☑	1415	1240.17	1239.16
☑	1416	1243.24	1242.23
☑	1417	622.39	1242.76
☑	1418	1244.53	1243.52

☑	1419	1245.27	1244.26
☑	1420	1247.69	1246.68
☑	1421	1249.64	1248.64
☑	1422	1250.12	1249.11
☑	1423	1251.41	1250.41
☑	1424	1252.21	1251.21
☑	1425	1252.51	1251.50
☑	1426	626.96	1251.91
☑	1427	1254.61	1253.60
☑	1428	627.89	1253.77
☑	1429	1255.05	1254.04
☑	1430	1255.57	1254.56
☑	1432	629.38	1256.74
☑	1433	1258.87	1257.86
☑	1434	1260.85	1259.84
☑	1436	1262.26	1261.26
☑	1437	632.43	1262.85
☑	1438	1265.09	1264.08
☑	1439	633.38	1264.74
☑	1444	1270.52	1269.51
☑	1445	1271.61	1270.60
☑	1446	1272.71	1271.70
☑	1447	636.91	1271.81
☑	1448	638.25	1274.49
☑	1450	1276.15	1275.14
☑	1451	638.78	1275.54
☑	1452	1277.77	1276.76
☑	1453	639.42	1276.83
☑	1455	640.42	1278.82
☑	1456	641.04	1280.07
☑	1457	1281.58	1280.57
☑	1458	1281.89	1280.88
☑	1459	1282.77	1281.76
☑	1460	642.49	1282.96

☑	1462	1287.25	1286.24
☑	1464	1289.57	1288.56
☑	1466	645.34	1288.66
☑	1467	645.56	1289.11
☑	1468	1290.37	1289.36
☑	1469	1291.27	1290.26
☑	1470	1291.72	1290.71
☑	1471	1293.94	1292.93
☑	1472	1295.67	1294.66
☑	1473	1296.12	1295.11
☑	1475	1297.83	1296.82
☑	1476	650.40	1298.78
☑	1483	1301.23	1300.22
☑	1485	1302.33	1301.33
☑	1487	1304.33	1303.32
☑	1488	653.01	1304.01
☑	1491	1308.67	1307.66
☑	1492	654.95	1307.89
☑	1493	655.47	1308.92
☑	1494	656.47	1310.92
☑	1495	1315.70	1314.69
☑	1498	658.86	1315.70
☑	1501	1317.48	1316.47
☑	1502	1317.49	1316.48
☑	1504	660.45	1318.88
☑	1510	666.32	1330.62
☑	1511	667.86	1333.71
☑	1513	1340.27	1339.26
☑	1514	670.87	1339.72
☑	1515	1340.99	1339.98
☑	1517	671.99	1341.97
☑	1518	672.94	1343.87
☑	1519	673.10	1344.19
☑	1520	1347.63	1346.62

☑	1522	675.39	1348.76
☑	1523	1349.77	1348.76
☑	1525	676.02	1350.03
☑	1526	676.95	1351.89
☑	1529	680.34	1358.67
☑	1530	681.43	1360.84
☑	1531	1363.67	1362.66
☑	1533	1365.61	1364.60
☑	1534	683.41	1364.81
☑	1536	1366.22	1365.21
☑	1537	684.44	1366.86
☑	1539	689.44	1376.87
☑	1540	1379.77	1378.76
☑	1543	691.99	1381.96
☑	1544	692.41	1382.80
☑	1546	692.72	1383.43
☑	1550	1388.71	1387.70
☑	1552	1390.44	1389.43
☑	1554	697.47	1392.92
☑	1555	697.51	1393.01
☑	1556	698.01	1394.00
☑	1557	698.49	1394.96
☑	1558	1399.55	1398.54
☑	1559	1401.68	1400.67
☑	1560	702.46	1402.90
☑	1561	703.37	1404.73
☑	1562	703.88	1405.75
☑	1563	703.96	1405.90
☑	1566	708.42	1414.82
☑	1567	709.96	1417.91
☑	1568	710.37	1418.73
☑	1570	710.47	1418.92
☑	1571	710.84	1419.67
☑	1572	710.99	1419.96

☑	1573	711.34	1420.67
☑	1574	711.45	1420.88
☑	1575	712.42	1422.83
☑	1578	476.65	1426.93
☑	1580	714.93	1427.84
☑	1581	717.54	1433.06
☑	1582	717.95	1433.89
☑	1584	719.42	1436.83
☑	1585	719.52	1437.03
☑	1586	720.97	1439.93
☑	1587	722.44	1442.86
☑	1588	1446.15	1445.15
☑	1590	724.56	1447.11
☑	1591	724.93	1447.84
☑	1592	725.45	1448.88
☑	1593	725.82	1449.62
☑	1594	725.91	1449.81
☑	1596	727.46	1452.90
☑	1598	488.30	1461.89
☑	1602	736.48	1470.95
☑	1606	738.47	1474.93
☑	1607	1478.64	1477.63
☑	1608	740.71	1479.40
☑	1610	744.91	1487.81
☑	1611	747.20	1492.39
☑	1613	748.97	1495.93
☑	1616	751.31	1500.60
☑	1617	751.55	1501.09
☑	1618	752.46	1502.91
☑	1619	753.08	1504.14
☑	1620	753.48	1504.94
☑	1621	755.45	1508.88
☑	1623	755.90	1509.78
☑	1624	755.92	1509.82

☑	1625	756.11	1510.21
☑	1626	760.49	1518.96
☑	1627	764.93	1527.85
☑	1628	765.01	1528.00
☑	1629	765.80	1529.59
☑	1630	770.00	1537.98
☑	1631	514.64	1540.91
☑	1632	771.84	1541.67
☑	1635	774.25	1546.49
☑	1636	775.33	1548.65
☑	1637	775.47	1548.93
☑	1640	522.09	1563.25
☑	1641	782.75	1563.50
☑	1642	784.28	1566.55
☑	1643	785.92	1569.83
☑	1644	786.06	1570.10
☑	1645	786.30	1570.59
☑	1646	787.03	1572.04
☑	1647	788.55	1575.08
☑	1649	789.79	1577.56
☑	1651	793.04	1584.07
☑	1652	793.15	1584.29
☑	1653	793.89	1585.76
☑	1657	794.48	1586.95
☑	1658	794.64	1587.26
☑	1659	794.86	1587.71
☑	1660	795.13	1588.24
☑	1661	795.36	1588.70
☑	1662	800.05	1598.08
☑	1663	800.83	1599.65
☑	1665	803.43	1604.85
☑	1666	803.45	1604.89
☑	1669	537.01	1608.01
☑	1670	538.63	1612.86

☑	1671	807.92	1613.84
☑	1672	540.23	1617.67
☑	1673	809.91	1617.80
☑	1676	812.81	1623.60
☑	1678	813.63	1625.25
☑	1679	814.53	1627.04
☑	1680	815.05	1628.09
☑	1682	547.42	1639.23
☑	1683	820.84	1639.67
☑	1684	820.96	1639.91
☑	1685	821.17	1640.32
☑	1686	821.44	1640.87
☑	1687	829.46	1656.91
☑	1688	829.93	1657.86
☑	1690	832.02	1662.02
☑	1691	556.50	1666.48
☑	1692	834.95	1667.89
☑	1693	835.56	1669.10
☑	1694	836.10	1670.18
☑	1695	836.37	1670.73
☑	1696	836.54	1671.07
☑	1697	836.60	1671.18
☑	1698	837.56	1673.10
☑	1699	838.00	1673.98
☑	1700	838.46	1674.90
☑	1701	841.91	1681.80
☑	1702	842.30	1682.60
☑	1704	844.48	1686.94
☑	1705	563.59	1687.74
☑	1706	845.28	1688.55
☑	1707	846.50	1690.99
☑	1708	846.96	1691.91
☑	1710	847.89	1693.77
☑	1711	849.06	1696.10

☑	1712	850.06	1698.10
☑	1714	850.19	1698.37
☑	1715	850.74	1699.46
☑	1716	851.43	1700.84
☑	1717	851.59	1701.16
☑	1718	568.61	1702.81
☑	1719	854.47	1706.93
☑	1720	857.83	1713.64
☑	1723	859.03	1716.05
☑	1724	859.51	1717.00
☑	1725	860.53	1719.04
☑	1726	864.03	1726.05
☑	1727	870.08	1738.15
☑	1728	870.41	1738.81
☑	1729	580.78	1739.32
☑	1730	581.79	1742.34
☑	1731	872.53	1743.05
☑	1732	872.54	1743.06
☑	1733	873.09	1744.17
☑	1736	875.23	1748.44
☑	1737	876.44	1750.86
☑	1738	584.83	1751.46
☑	1739	877.09	1752.16
☑	1740	879.80	1757.58
☑	1741	881.51	1761.01
☑	1742	881.53	1761.05
☑	1743	883.45	1764.88
☑	1745	592.01	1773.01
☑	1746	592.03	1773.08
☑	1747	888.05	1774.09
☑	1748	892.88	1783.74
☑	1749	596.02	1785.05
☑	1750	894.06	1786.10
☑	1752	896.00	1789.98

☑	1754	896.42	1790.82
☑	1756	898.44	1794.87
☑	1759	899.34	1796.67
☑	1760	899.87	1797.72
☑	1761	900.05	1798.09
☑	1762	600.38	1798.13
☑	1764	604.12	1809.34
☑	1765	909.04	1816.06
☑	1766	909.33	1816.65
☑	1767	607.43	1819.26
☑	1769	915.35	1828.68
☑	1771	916.32	1830.62
☑	1772	916.60	1831.18
☑	1773	611.79	1832.34
☑	1774	612.35	1834.01
☑	1776	919.03	1836.05
☑	1777	919.04	1836.06
☑	1779	613.08	1836.23
☑	1781	613.61	1837.81
☑	1782	920.06	1838.11
☑	1783	923.13	1844.26
☑	1784	615.93	1844.77
☑	1786	926.24	1850.46
☑	1787	926.59	1851.17
☑	1788	619.09	1854.24
☑	1789	929.09	1856.17
☑	1791	621.32	1860.93
☑	1793	932.30	1862.60
☑	1794	933.86	1865.70
☑	1795	937.40	1872.79
☑	1796	938.83	1875.64
☑	1798	939.65	1877.28
☑	1799	942.23	1882.45
☑	1800	942.69	1883.37

☑	1801	943.04	1884.06
☑	1802	630.03	1887.06
☑	1803	944.60	1887.19
☑	1804	631.85	1892.52
☑	1805	949.54	1897.07
☑	1806	949.92	1897.82
☑	1807	951.01	1900.01
☑	1808	635.02	1902.04
☑	1809	952.40	1902.78
☑	1811	955.47	1908.92
☑	1812	955.64	1909.26
☑	1813	960.25	1918.48
☑	1814	960.26	1918.51
☑	1816	641.09	1920.26
☑	1817	641.45	1921.32
☑	1818	963.07	1924.12
☑	1820	966.72	1931.43
☑	1821	967.47	1932.93
☑	1822	969.77	1937.52
☑	1823	970.48	1938.94
☑	1824	971.93	1941.85
☑	1826	972.60	1943.19
☑	1827	973.64	1945.28
☑	1828	974.07	1946.12
☑	1829	975.42	1948.82
☑	1830	975.71	1949.41
☑	1831	979.42	1956.82
☑	1833	979.93	1957.86
☑	1834	980.03	1958.04
☑	1835	980.56	1959.11
☑	1836	982.58	1963.14
☑	1837	982.61	1963.21
☑	1838	655.82	1964.45
☑	1839	983.69	1965.37

☑	1840	656.41	1966.20
☑	1842	984.55	1967.09
☑	1843	984.78	1967.56
☑	1845	987.74	1973.46
☑	1846	991.45	1980.89
☑	1847	992.26	1982.50
☑	1848	993.61	1985.21
☑	1849	994.08	1986.15
☑	1850	994.26	1986.51
☑	1851	994.54	1987.06
☑	1852	997.56	1993.10
☑	1853	997.59	1993.17
☑	1854	665.41	1993.22
☑	1856	665.85	1994.53
☑	1857	999.06	1996.10
☑	1858	999.54	1997.07
☑	1859	999.58	1997.14
☑	1860	1003.51	2005.01
☑	1861	1003.71	2005.40
☑	1862	1004.06	2006.11
☑	1863	1006.47	2010.92
☑	1864	1008.83	2015.64
☑	1865	672.95	2015.83
☑	1866	673.22	2016.63
☑	1867	1011.06	2020.10
☑	1869	674.64	2020.90
☑	1870	1011.56	2021.10
☑	1871	674.81	2021.40
☑	1872	1011.99	2021.97
☑	1873	1013.00	2023.99
☑	1875	676.77	2027.29
☑	1876	677.53	2029.58
☑	1877	1015.89	2029.76
☑	1878	1016.09	2030.16

1879	678.14	2031.40
1880	678.15	2031.44
1881	678.38	2032.13
1882	679.22	2034.63
1883	679.96	2036.85
1884	682.25	2043.74
1885	1027.52	2053.03
1887	687.40	2059.19
1888	688.20	2061.57
1890	689.92	2066.73
1891	1036.75	2071.49
1892	1040.09	2078.16
1893	1041.03	2080.05
1894	1042.44	2082.86
1895	1046.25	2090.49
1896	699.41	2095.21
1897	1048.64	2095.27
1898	699.45	2095.32
1899	1048.91	2095.81
1900	700.41	2098.21
1901	1050.18	2098.34
1902	1050.71	2099.41
1903	1051.44	2100.88
1904	1051.61	2101.20
1905	1053.04	2104.07
1906	703.05	2106.12
1907	1057.17	2112.33
1908	706.03	2115.08
1909	707.26	2118.76
1910	708.89	2123.65
1911	1064.13	2126.25
1912	1067.10	2132.19
1913	714.07	2139.20
1914	1070.64	2139.26

☑	1915	714.48	2140.41
☑	1916	1071.72	2141.43
☑	1917	1073.41	2144.81
☑	1918	1075.28	2148.54
☑	1919	1075.71	2149.40
☑	1920	718.00	2150.98
☑	1921	718.13	2151.38
☑	1922	719.02	2154.04
☑	1923	1078.81	2155.61
☑	1924	1079.02	2156.02
☑	1925	720.10	2157.28
☑	1926	1079.67	2157.33
☑	1927	720.13	2157.36
☑	1929	1081.47	2160.93
☑	1930	722.13	2163.36
☑	1931	1082.90	2163.78
☑	1932	1083.12	2164.24
☑	1933	1084.79	2167.57
☑	1934	1086.11	2170.20
☑	1935	724.41	2170.22
☑	1936	724.44	2170.30
☑	1937	725.56	2173.66
☑	1939	726.41	2176.21
☑	1940	728.11	2181.31
☑	1941	1094.09	2186.16
☑	1942	729.74	2186.19
☑	1943	732.67	2194.99
☑	1944	1100.90	2199.78
☑	1945	1101.45	2200.89
☑	1946	1103.51	2205.00
☑	1947	737.81	2210.40
☑	1948	1109.32	2216.62
☑	1949	740.74	2219.19
☑	1950	741.98	2222.92

☑	1951	1114.91	2227.82
☑	1952	744.15	2229.42
☑	1953	1117.09	2232.16
☑	1954	746.80	2237.39
☑	1955	1119.90	2237.78
☑	1956	747.51	2239.50
☑	1957	750.19	2247.55
☑	1959	751.14	2250.40
☑	1960	751.43	2251.27
☑	1961	751.48	2251.41
☑	1962	1126.99	2251.96
☑	1963	1127.34	2252.66
☑	1964	753.03	2256.07
☑	1965	1129.63	2257.24
☑	1966	1132.72	2263.43
☑	1967	1135.43	2268.85
☑	1968	1136.32	2270.62
☑	1969	1138.10	2274.19
☑	1970	1140.53	2279.05
☑	1971	1140.88	2279.74
☑	1972	1141.25	2280.49
☑	1973	761.54	2281.61
☑	1974	761.59	2281.76
☑	1975	761.61	2281.82
☑	1976	761.75	2282.24
☑	1977	761.82	2282.43
☑	1978	1142.34	2282.66
☑	1979	762.12	2283.33
☑	1980	762.15	2283.44
☑	1981	762.36	2284.06
☑	1983	1144.06	2286.10
☑	1984	763.08	2286.22
☑	1985	766.07	2295.20
☑	1986	1148.69	2295.37

1987	766.15	2295.43
1988	767.23	2298.67
1989	767.47	2299.39
1990	767.54	2299.61
1991	770.42	2308.25
1992	1162.04	2322.07
1993	1162.64	2323.27
1995	1166.66	2331.31
1996	1167.62	2333.22
1997	1168.64	2335.27
1998	1170.72	2339.43
1999	783.87	2348.60
2000	1175.80	2349.59
2001	1177.02	2352.02
2002	1177.64	2353.26
2003	785.75	2354.24
2004	1181.56	2361.11
2005	788.77	2363.29
2006	789.23	2364.68
2007	1183.69	2365.37
2008	789.54	2365.60
2009	1184.27	2366.53
2010	1184.30	2366.58
2011	1188.18	2374.34
2012	792.48	2374.43
2013	1188.49	2374.97
2015	1192.06	2382.11
2016	795.42	2383.24
2017	1192.63	2383.25
2018	1195.72	2389.42
2019	1195.95	2389.90
2020	798.33	2391.98
2021	1197.08	2392.15
2022	799.14	2394.39

☑	2023	*802.15*	*2403.43*
☑	2024	*1202.83*	*2403.64*
☑	2025	*802.72*	*2405.14*
☑	2026	*1203.60*	*2405.18*
☑	2027	*1209.86*	*2417.70*
☑	2028	*807.85*	*2420.53*
☑	2029	*808.41*	*2422.22*
☑	2030	*808.54*	*2422.60*
☑	2031	*1215.93*	*2429.86*
☑	2032	*812.03*	*2433.07*
☑	2033	*812.47*	*2434.39*
☑	2034	*1219.94*	*2437.87*
☑	2035	*1220.06*	*2438.11*
☑	2037	*814.85*	*2441.53*
☑	2038	*1227.28*	*2452.55*
☑	2039	*1227.73*	*2453.44*
☑	2040	*818.86*	*2453.56*
☑	2041	*820.89*	*2459.65*
☑	2042	*823.38*	*2467.12*
☑	2043	*824.74*	*2471.20*
☑	2044	*1237.19*	*2472.37*
☑	2045	*825.51*	*2473.51*
☑	2046	*826.14*	*2475.40*
☑	2047	*826.29*	*2475.86*
☑	2048	*828.46*	*2482.37*
☑	2049	*1243.39*	*2484.78*
☑	2050	*1243.75*	*2485.48*
☑	2052	*1245.41*	*2488.81*
☑	2053	*831.17*	*2490.49*
☑	2054	*1249.53*	*2497.05*
☑	2055	*835.60*	*2503.77*
☑	2056	*836.50*	*2506.46*
☑	2057	*836.60*	*2506.77*
☑	2058	*1255.67*	*2509.32*

☑	2059	837.47	2509.40
☑	2060	839.42	2515.25
☑	2061	839.49	2515.43
☑	2062	1258.75	2515.49
☑	2063	840.87	2519.59
☑	2064	842.39	2524.16
☑	2065	842.51	2524.52
☑	2066	1263.54	2525.06
☑	2067	843.16	2526.45
☑	2068	845.12	2532.34
☑	2069	846.16	2535.47
☑	2070	849.20	2544.57
☑	2071	1274.09	2546.16
☑	2073	850.85	2549.52
☑	2074	852.73	2555.16
☑	2075	1280.13	2558.24
☑	2076	854.59	2560.76
☑	2077	855.80	2564.38
☑	2078	855.84	2564.50
☑	2079	1283.60	2565.19
☑	2080	856.19	2565.55
☑	2081	857.13	2568.37
☑	2082	858.55	2572.63
☑	2083	1287.67	2573.33
☑	2084	1290.81	2579.60
☑	2085	863.71	2588.12
☑	2086	866.88	2597.62
☑	2087	867.17	2598.49
☑	2088	868.52	2602.53
☑	2089	875.78	2624.32
☑	2090	876.22	2625.63
☑	2091	876.84	2627.50
☑	2092	877.60	2629.79
☑	2094	882.85	2645.51

☑	2095	889.37	2665.08
☑	2096	890.64	2668.90
☑	2097	1345.27	2688.53
☑	2098	899.44	2695.29
☑	2099	900.66	2698.96
☑	2100	901.58	2701.71
☑	2101	901.73	2702.16
☑	2102	902.36	2704.05
☑	2103	902.46	2704.37
☑	2104	902.56	2704.66
☑	2105	903.77	2708.28
☑	2106	904.67	2711.00
☑	2107	904.73	2711.18
☑	2108	904.99	2711.93
☑	2109	905.09	2712.25
☑	2111	905.76	2714.27
☑	2112	905.78	2714.31
☑	2113	906.47	2716.39
☑	2114	906.48	2716.41
☑	2115	906.94	2717.79
☑	2116	907.41	2719.21
☑	2117	910.45	2728.32
☑	2118	911.04	2730.10
☑	2119	913.75	2738.24
☑	2120	914.87	2741.60
☑	2121	918.85	2753.53
☑	2122	924.49	2770.43
☑	2123	926.97	2777.87
☑	2124	929.51	2785.50
☑	2125	931.65	2791.93
☑	2126	936.83	2807.47
☑	2127	937.37	2809.09
☑	2128	943.04	2826.10
☑	2129	947.10	2838.28

☑	2130	954.79	2861.36
☑	2131	958.23	2871.67
☑	2132	964.35	2890.04
☑	2133	968.20	2901.58
☑	2134	968.63	2902.87
☑	2135	969.26	2904.75
☑	2136	977.13	2928.38
☑	2137	984.33	2949.98
☑	2138	985.44	2953.31
☑	2139	988.17	2961.49
☑	2140	988.46	2962.34
☑	2141	990.77	2969.29
☑	2143	992.73	2975.16
☑	2144	995.45	2983.33
☑	2145	995.46	2983.36
☑	2146	996.91	2987.71
☑	2147	999.61	2995.81
☑	2148	1001.63	3001.86
☑	2149	1002.72	3005.13
☑	2150	1007.27	3018.78
☑	2151	1007.89	3020.64
☑	2152	1009.43	3025.27
☑	2154	1017.51	3049.50
☑	2155	1018.26	3051.75
☑	2156	1020.47	3058.39
☑	2157	1021.02	3060.03
☑	2158	1021.05	3060.13
☑	2159	1021.79	3062.35
☑	2160	1026.11	3075.29
☑	2161	1027.09	3078.24
☑	2162	1030.23	3087.68
☑	2163	1031.68	3092.02
☑	2164	1032.25	3093.72
☑	2165	1034.91	3101.70

☑	2166	*1038.30*	*3111.86*
☑	2167	*1038.89*	*3113.66*
☑	2168	*1040.68*	*3119.02*
☑	2169	*1043.54*	*3127.60*
☑	2170	*1045.45*	*3133.33*
☑	2171	*1046.70*	*3137.07*
☑	2172	*1047.83*	*3140.47*
☑	2173	*1054.56*	*3160.66*
☑	2174	*1057.24*	*3168.69*
☑	2175	*1057.98*	*3170.92*
☑	2176	*1066.97*	*3197.89*
☑	2177	*1074.53*	*3220.56*
☑	2178	*1075.17*	*3222.50*
☑	2179	*1075.45*	*3223.33*
☑	2180	*1079.50*	*3235.48*
☑	2181	*1082.46*	*3244.37*
☑	2182	*1084.11*	*3249.31*
☑	2183	*1088.92*	*3263.73*
☑	2184	*1088.94*	*3263.79*
☑	2185	*1095.52*	*3283.53*
☑	2186	*1095.77*	*3284.30*
☑	2187	*1095.87*	*3284.59*
☑	2188	*1100.42*	*3298.25*
☑	2189	*1102.84*	*3305.49*
☑	2190	*1104.87*	*3311.60*
☑	2191	*1117.47*	*3349.39*
☑	2192	*1117.67*	*3350.00*
☑	2193	*1117.72*	*3350.13*
☑	2194	*1121.69*	*3362.05*
☑	2195	*1121.73*	*3362.18*
☑	2196	*1130.15*	*3387.44*
☑	2197	*1131.55*	*3391.63*
☑	2198	*1141.06*	*3420.16*
☑	2199	*1142.61*	*3424.81*

☑	2200	*1146.78*	*3437.31*
☑	2201	*1151.24*	*3450.69*
☑	2202	*1155.62*	*3463.83*
☑	2203	*1158.78*	*3473.30*
☑	2204	*1180.13*	*3537.38*
☑	2205	*1180.22*	*3537.65*
☑	2206	*1183.24*	*3546.70*
☑	2207	*1187.16*	*3558.47*
☑	2208	*1188.93*	*3563.76*
☑	2209	*1190.06*	*3567.15*
☑	2210	*1210.71*	*3629.12*
☑	2211	*1231.51*	*3691.50*
☑	2212	*1240.37*	*3718.09*
☑	2213	*1246.90*	*3737.68*

Search Parameters

```
Type of search         : MS/MS Ion Search
Enzyme                 : Trypsin
Variable modifications : Carbamidomethyl (C),Oxidation (M)
Mass values            : Monoisotopic
Protein Mass           : Unrestricted
Peptide Mass Tolerance : ± 0.6 Da
Fragment Mass Tolerance: ± 0.3 Da
Max Missed Cleavages   : 2
Instrument type        : ESI-TRAP
Number of queries      : 2213
```

Mascot: http://www.matrixscience.com/
