# Supplementary material for: Macrophage-infectivity potentiator of Trypanosoma cruzi (TcMIP) is a new pro-type 1 immuno-stimulating protein for neonatal human cells and vaccines in mice
Source: Front Immunol. 2023 Mar 23;14:1138526. doi: 10.3389/fimmu.2023.1138526 (PMC10077492; doi:10.3389/fimmu.2023.1138526)
Supplement: Supplementary file 7 [file DataSheet_5.pdf]

Mascot Search Results

User : GM
Email : gabriel.mazzucchelli@ulg.ac.be
Search title : Submitted from 090116-Adj-OGE39-8923-otherEuk-NCBi by Mascot Daemon on MASPEC39
MS data file : F:\DATA\Archives\ESQUIRE\2009 esquire\Adjuvac\090114\090116-ADJ-OGE39_8923.mgf
Database : NCBInr 20081117 (6550153 sequences; 2246496946 residues)
Taxonomy : Other Eukaryota (159618 sequences)
Timestamp : 19 Jan 2009 at 09:03:09 GMT
Significant hits:

gi|3023478	RecName: Full=Chaperonin HSP60, mitochondrial; Short=Protein Cpn60; AltName: Full=groEL protein; Al
gi|71402301	surface protein TolT [Trypanosoma cruzi strain CL Brener]
gi|71651556	hypothetical protein [Trypanosoma cruzi strain CL Brener]
gi|71662736	hypothetical protein [Trypanosoma cruzi strain CL Brener]
gi|71400377	microtubule-associated protein [Trypanosoma cruzi strain CL Brener]
gi|71401264	hypothetical protein [Trypanosoma cruzi strain CL Brener]
gi|71425779	I/6 autoantigen [Trypanosoma cruzi strain CL Brener]
gi|71413559	hypothetical protein [Trypanosoma cruzi strain CL Brener]
gi|349838	heat shock protein
gi|18568139	beta tubulin 1.9 [Trypanosoma cruzi]
gi|71655864	I/6 autoantigen [Trypanosoma cruzi strain CL Brener]
gi|71407337	hypothetical protein [Trypanosoma cruzi strain CL Brener]
gi|71667953	hypothetical protein [Trypanosoma cruzi strain CL Brener]
gi|71651158	hypothetical protein [Trypanosoma cruzi strain CL Brener]
gi|120679	RecName: Full=Glyceraldehyde-3-phosphate dehydrogenase, glycosomal; Short=GAPDH
gi|71659715	cyclophilin A [Trypanosoma cruzi strain CL Brener]
gi|71659663	hypothetical protein [Trypanosoma cruzi strain CL Brener]
gi|71667211	hypothetical protein [Trypanosoma cruzi strain CL Brener]
gi|71656813	glycosomal malate dehydrogenase [Trypanosoma cruzi strain CL Brener]
gi|2961256	putative malate dehydrogenase [Trypanosoma cruzi]
gi|71664003	ATP synthase, epsilon chain [Trypanosoma cruzi strain CL Brener]
gi|704459	elongation factor 1 alpha [Trypanosoma cruzi]
gi|1929445	elongation factor 1-alpha [Trypanosoma cruzi]
gi|71413449	RNA-binding protein [Trypanosoma cruzi strain CL Brener]
gi|91983204	beta tubulin [Trypanosoma pestanai]
gi|154345814	chaperonin Hsp60, mitochondrial precursor [Leishmania braziliensis MHOM/BR/75/M2904]
gi|71659763	dynein light chain [Trypanosoma cruzi strain CL Brener]
gi|89329739	heat shock protein 70 [Capsaspora owczarzaki]
gi|55824400	heat shock protein 70 cytosolic isoform [Rhynchobodo ATCC50359]
gi|4389144	Chain A, Trypanosoma Cruzi Triosephosphate Isomerase
gi|119859	RecName: Full=Flagellar calcium-binding protein; Short=FCABP; AltName: Full=1F8 protein; AltName: F
gi|71407149	hypothetical protein [Trypanosoma cruzi strain CL Brener]
gi|10673	unnamed protein product [Trypanosoma cruzi]
gi|71405209	calmodulin [Trypanosoma cruzi strain CL Brener]

gi|71409213	elongation factor 1-gamma (EF-1-gamma) [Trypanosoma cruzi strain CL Brener]
gi|71407515	heat shock 70 kDa protein, mitochondrial precursor [Trypanosoma cruzi strain CL Brener]
gi|71423525	vesicle-associated membrane protein [Trypanosoma cruzi strain CL Brener]
gi|1170958	RecName: Full=Macrophage infectivity potentiator; AltName: Full=Peptidyl-prolyl cis-trans isomerase
gi|58177523	Chain A, Crystal Structure Of Cyclophilin From Trypanosoma Cruzi
gi|71422090	electron-transfer-flavoprotein, alpha polypeptide [Trypanosoma cruzi strain CL Brener]
gi|71425268	protein disulfide isomerase [Trypanosoma cruzi strain CL Brener]
gi|71406218	COP-coated vesicle membrane protein gp25L precursor [Trypanosoma cruzi strain CL Brener]
gi|84105385	cytosolic heat shock protein 70 [Malawimonas jakobiformis]
gi|71665461	enolase [Trypanosoma cruzi strain CL Brener]
gi|12007351	heat shock protein Hsp70 [Monosiga ovata]
gi|123592	RecName: Full=Heat shock 70 kDa protein
gi|71404821	trans-sialidase [Trypanosoma cruzi strain CL Brener]
gi|71416386	hypothetical protein [Trypanosoma cruzi strain CL Brener]
gi|123603	RecName: Full=Heat shock 70 kDa protein
gi|58042864	PPAT5 [Hyaloperonospora parasitica]
gi|57903381	heat shock protein 70 [Spumella uniguttata]
gi|71410853	10 kDa heat shock protein [Trypanosoma cruzi strain CL Brener]
gi|85822985	elongation factor 1-alpha [Thraustochytriidae sp. P19]
gi|89329735	translation elongation factor 1-alpha [Capsaspora owczarzaki]
gi|156987872	translation elongation factor 1-alpha [Phytophthora quercina]
gi|50660750	translation elongation factor 1 alpha [Phytophthora hibernalis]
gi|59859762	elongation factor 1 alpha [Phaeodactylum tricornutum]
gi|156987628	translation elongation factor 1-alpha [Phytophthora brassicae]
gi|56156697	elongation factor 1A [Trichia persimilis]
gi|1706590	Elongation factor 1-alpha S (EF-1-alpha S) (Sporophyte-specific EF-1-alpha)
gi|50660760	translation elongation factor 1 alpha [Phytophthora insolita]
gi|71425263	10 kDa heat shock protein [Trypanosoma cruzi strain CL Brener]
gi|1314208	alpha-tubulin
gi|71425751	centrin [Trypanosoma cruzi strain CL Brener]
gi|71414147	cytochrome c [Trypanosoma cruzi strain CL Brener]
gi|71408270	calmodulin [Trypanosoma cruzi strain CL Brener]
gi|71664019	hypothetical protein [Trypanosoma cruzi strain CL Brener]
gi|71658999	pyruvate phosphate dikinase [Trypanosoma cruzi strain CL Brener]
gi|71413591	hypothetical protein [Trypanosoma cruzi strain CL Brener]
gi|19171192	phosphoenolpyruvate mutase [Trypanosoma cruzi]
gi|71414193	adenylate kinase [Trypanosoma cruzi strain CL Brener]
gi|28195111	protein kinase A regulatory subunit [Trypanosoma cruzi]
gi|71666956	hypothetical protein [Trypanosoma cruzi strain CL Brener]
gi|71651382	thioredoxin [Trypanosoma cruzi strain CL Brener]
gi|71654654	kinetoplast DNA-associated protein [Trypanosoma cruzi strain CL Brener]
gi|71650885	ATP-dependent DEAD/H RNA helicase [Trypanosoma cruzi strain CL Brener]
gi|71414729	ras-related protein rab-5 [Trypanosoma cruzi strain CL Brener]
gi|167538218	hypothetical protein [Monosiga brevicollis MX1]
gi|71403985	t-complex protein 1, delta subunit [Trypanosoma cruzi strain CL Brener]

gi|71746618	t-complex protein 1 subunit delta [Trypanosoma brucei TREU927]
gi|71659870	hypothetical protein [Trypanosoma cruzi strain CL Brener]
gi|167539657	hypothetical protein [Monosiga brevicollis MX1]
gi|71662790	trans-sialidase [Trypanosoma cruzi strain CL Brener]
gi|3004649	putative GTP-binding protein RAB11 [Trypanosoma cruzi]
gi|71659614	hypothetical protein [Trypanosoma cruzi strain CL Brener]
gi|84105387	cytosolic heat shock protein 70 [Reclinomonas americana]
gi|168830545	cytosolic heat shock protein 70 [Andalucia godoyi]
gi|468014	cytoplasmic 70 kDa heat shock protein
gi|88770694	70 kDa heat shock protein [Rhodomonas salina]
gi|194476867	molecular chaperone DnaK [Paulinella chromatophora]
gi|409155	ADG1
gi|71409831	basic transcription factor 3a [Trypanosoma cruzi strain CL Brener]
gi|71663174	succinyl-CoA ligase [GDP-forming] beta-chain [Trypanosoma cruzi]
gi|10119899	pyruvate phosphate dikinase 1 [Trypanosoma cruzi]
gi|71649977	hypothetical protein [Trypanosoma cruzi strain CL Brener]
gi|71404111	40S ribosomal protein S15 [Trypanosoma cruzi strain CL Brener]
gi|10632	TcP2a-RA [Trypanosoma cruzi]
gi|118766644	elongation factor-1 alpha [Oxymonadida environmental sample]
gi|56156659	elongation factor 1A [Echinostelium minutum]
gi|71663474	dynein [Trypanosoma cruzi strain CL Brener]
gi|1781355	histone H2A [Trypanosoma cruzi]
gi|13384081	histone H2A [Leishmania infantum]
gi|71659778	poly(A)-binding protein [Trypanosoma cruzi strain CL Brener]
gi|37727511	adenylate kinase [Trypanosoma cruzi]
gi|71664627	hypothetical protein [Trypanosoma cruzi strain CL Brener]
gi|53849793	glycosomal glyceraldehyde phosphate dehydrogenase [Trypanosoma mega]
gi|158577406	glycosomal glyceraldehyde-3-phosphate dehydrogenase [Crithidia oncopelti]
gi|71660723	hypothetical protein [Trypanosoma cruzi strain CL Brener]
gi|71410849	protein disulfide isomerase [Trypanosoma cruzi strain CL Brener]
gi|71405387	hypothetical protein [Trypanosoma cruzi strain CL Brener]
gi|71424867	ATP-dependent zinc metallopeptidase [Trypanosoma cruzi strain CL Brener]
gi|71403861	trans-sialidase [Trypanosoma cruzi strain CL Brener]
gi|71420685	intraflagellar transport (IFT) protein [Trypanosoma cruzi strain CL Brener]
gi|123495829	small GTP-binding protein [Trichomonas vaginalis G3]
gi|23306650	heat shock protein 70 [Carpediemonas membranifera]
gi|1322228	elongation factor 1 alpha
gi|438596	elongation factor 1-alpha
gi|71404920	hypothetical protein [Trypanosoma cruzi strain CL Brener]
gi|71420832	NUP-1 protein [Trypanosoma cruzi strain CL Brener]
gi|71402229	ubiquitin-like protein [Trypanosoma cruzi strain CL Brener]
gi|71420441	hypothetical protein [Trypanosoma cruzi strain CL Brener]
gi|71661916	RNA-binding protein [Trypanosoma cruzi strain CL Brener]
gi|167375825	hypothetical protein [Entamoeba dispar SAW760]
gi|71405064	60S acidic ribosomal protein P2 [Trypanosoma cruzi strain CL Brener]

gi|154341937	glyceraldehyde 3-phosphate dehydrogenase,glycosomal [Leishmania braziliensis MHOM/BR/75/M2904]
gi|71656526	hypothetical protein [Trypanosoma cruzi strain CL Brener]
gi|9954108	RNA binding protein RGGm [Trypanosoma cruzi]
gi|12083381	antigen 38 [Trypanosoma cruzi]
gi|4323557	chaperonin 60.2 precursor [Leishmania donovani]
gi|1217626	heat shock protein 60 [Euglena gracilis]
gi|123438714	small GTP-binding protein [Trichomonas vaginalis G3]
gi|167527390	hypothetical protein [Monosiga brevicollis MX1]
gi|71409962	calpain-like cysteine peptidase [Trypanosoma cruzi strain CL Brener]
gi|71407010	cytochrome c [Trypanosoma cruzi strain CL Brener]
gi|10638	ribosomal protein P-JL5 [Trypanosoma cruzi]
gi|1729846	Tubulin beta-3 chain (Beta-3-tubulin)
gi|71412308	hypothetical protein [Trypanosoma cruzi strain CL Brener]
gi|71398774	hypothetical protein [Trypanosoma cruzi strain CL Brener]
gi|71403995	mucin-like glycoprotein [Trypanosoma cruzi strain CL Brener]
gi|163881518	heat shock protein 70 [Trimastix pyriformis]
gi|71401492	fatty acid desaturase [Trypanosoma cruzi strain CL Brener]
gi|28779307	beta-tubulin [Streblomastix strix]
gi|156988212	beta-tubulin [Phytophthora polonica]
gi|1729847	Tubulin beta-4 chain (Beta-4-tubulin)
gi|55274319	beta-tubulin [Phytophthora ramorum]
gi|188766724	beta-tubulin [Phytophthora ramorum]
gi|11596164	beta-tubulin [Acrasis rosea]
gi|3790449	beta-tubulin-1 [Chlorarachnion CCMP621]
gi|157887509	beta tubulin [Plasmodiophora brassicae]
gi|168830539	beta-tubulin [Andalucia godoyi]
gi|55274341	beta-tubulin [Phytophthora ramorum]
gi|3790456	beta-tubulin-5 [Chlorarachnion CCMP621]
gi|71656799	hypothetical protein [Trypanosoma cruzi strain CL Brener]
gi|71409309	hypothetical protein [Trypanosoma cruzi strain CL Brener]
gi|123487079	IBR domain containing protein [Trichomonas vaginalis G3]
gi|71655671	hypothetical protein [Trypanosoma cruzi strain CL Brener]
gi|71411394	fructose-bisphosphate aldolase, glycosomal [Trypanosoma cruzi strain CL Brener]
gi|154346376	hypothetical protein [Leishmania braziliensis MHOM/BR/75/M2904]
gi|71410728	nuclear transport factor 2 [Trypanosoma cruzi strain CL Brener]
gi|71665043	hypothetical protein [Trypanosoma cruzi strain CL Brener]
gi|71422058	mitochondrial RNA binding protein [Trypanosoma cruzi strain CL Brener]
gi|58414949	polyubiquitin [Stauracon pallidus]
gi|33358312	ubiquitin-like protein Ublp94.4 [Acanthamoeba castellanii]
gi|154411942	hypothetical protein [Trichomonas vaginalis G3]
gi|71402841	60S ribosomal protein L12 [Trypanosoma cruzi strain CL Brener]
gi|133055	60S acidic ribosomal protein P1
gi|71412664	hypothetical protein [Trypanosoma cruzi strain CL Brener]
gi|71411561	hypothetical protein [Trypanosoma cruzi strain CL Brener]
gi|154420635	DEAD/DEAH box helicase family protein [Trichomonas vaginalis G3]

gi|71659535	COP-coated vesicle membrane protein erv25 precursor [Trypanosoma cruzi strain CL Brener]
gi|11055687	ATPase alpha subunit [Trypanosoma brucei brucei]
gi|33867787	21 kDa cyclophilin [Trypanosoma cruzi]
gi|71404616	hypothetical protein [Trypanosoma cruzi strain CL Brener]
gi|123420403	hypothetical protein [Trichomonas vaginalis G3]
gi|167516814	hypothetical protein [Monosiga brevicollis MX1]
gi|27734387	polyubiquitin [Euglypha rotunda]
gi|461992	25 kDa elongation factor 1-beta (EF-1-beta)
gi|71402893	25 kDa translation elongation factor 1-beta [Trypanosoma cruzi strain CL Brener]
gi|225587	tubulin alpha
gi|84105377	alpha tubulin 2 [Rhynchopus sp. ATCC 50230]
gi|116222245	alpha tubulin [Thaumatomonas sp. TMT002]
gi|146081753	hypothetical protein [Leishmania infantum JPCM5]

Probability Based Mowse Score

Ions score is -10*Log(P), where P is the probability that the observed match is a random event.
Individual ions scores > 42 indicate identity or extensive homology (p<0.05).
Protein scores are derived from ions scores as a non-probabilistic basis for ranking protein hits.

Score Distribution

Peptide Summary Report

Format As | Peptide Summary | Help

Significance threshold p< 0.05 | Max. number of hits AUTO

Standard scoring ○ MudPIT scoring ◉ Ions score cut-off 0 | Show sub-sets ☐

Show pop-ups ◉ Suppress pop-ups ○ Sort unassigned [Decreasing Score] Require bold red ☐

[Select All] [Select None] [Search Selected] ☐ **Error tolerant** [Archive Report]

1. gi|3023478 **Mass:** 59374 **Score:** 851 **Queries matched:** 27

RecName: Full=Chaperonin HSP60, mitochondrial; Short=Protein Cpn60; AltName: Full=groEL protein; Al

☐ Check to include this hit in error tolerant search or archive report

	Query	Observed	Mr(expt)	Mr(calc)	Delta	Miss	Score	Expect	Rank	Peptide
☑	588	422.26	842.51	842.52	-0.02	0	56	0.0015	1	K.LSGGVAVIK.V
☑	1013	518.74	1035.47	1035.50	-0.03	0	42	0.033	1	R.ITDALCSTR.A + Carbamidomethyl (C)
☑	1169	557.79	1113.57	1113.64	-0.07	0	82	2.9e-06	1	R.AVSAVATTLGPK.G
☑	1336	602.32	1202.62	1202.58	0.04	0	105	1.8e-08	1	K.VGGGSEVEVNEK.K
☑	1389	617.34	1232.66	1232.72	-0.07	0	31	0.42	1	R.LPAHTIVLNAGK.E
☑	1510	659.84	1317.67	1317.69	-0.02	0	88	9.8e-07	1	R.NVIIEQSYGAPK.I
☑	1512	660.81	1319.61	1319.62	-0.01	0	50	0.0051	1	R.CIATGTNPIDMK.R + Carbamidomethyl (C)
☑	1529	670.30	1338.59	1338.61	-0.01	0	85	1.4e-06	1	R.GLIDGETSDYNR.E
☑	1550	680.82	1359.63	1359.67	-0.04	0	58	0.00083	1	R.GYISPYFVTDAK.A
☑	1584	696.41	1390.81	1390.73	0.08	0	103	2.6e-08	1	K.AELEDAFVLVSAK.K
☑	1644	728.88	1455.75	1455.80	-0.05	0	99	5.9e-08	1	R.AVGVILQSVAEQSR.K
☑	1764	803.94	1605.86	1605.92	-0.06	0	53	0.0024	1	R.AAVQEGIVPGGGVALLR.A
☑	575	831.42	1660.84	1660.83	0.01	0	77	3.5e-05	1	K.ALDSLLGDSSLTADQR.T
☑	1828	564.89	1691.66	1691.81	-0.15	0	(29)	0.71	1	K.VLENNDVTVGYDAQR.D
☑	1829	846.88	1691.75	1691.81	-0.07	0	108	9.3e-09	1	K.VLENNDVTVGYDAQR.D
	1909	905.79	1809.57	1809.85	-0.28	0	(1)	4.4e+02	2	K.TMTTELEVVEGMSIDR.G
	702	905.82	1809.63	1809.85	-0.22	0	(6)	3.5e+02	2	K.TMTTELEVVEGMSIDR.G
☑	1911	905.88	1809.74	1809.85	-0.11	0	(35)	0.17	1	K.TMTTELEVVEGMSIDR.G
☑	227	604.28	1809.83	1809.85	-0.02	0	(33)	0.79	1	K.TMTTELEVVEGMSIDR.G
☑	1912	604.29	1809.86	1809.85	0.01	0	(45)	0.015	1	K.TMTTELEVVEGMSIDR.G
☑	1925	913.89	1825.77	1825.84	-0.07	0	71	4e-05	1	K.TMTTELEVVEGMSIDR.G + Oxidation (M)
☑	1997	655.69	1964.05	1963.98	0.07	1	53	0.0031	1	K.AIEFKDPFENMGAQLVR.Q
☑	2294	837.71	2510.11	2510.25	-0.14	0	(63)	0.00021	1	K.TNDLAGDGTTTSAVLVASVFSESLR.C
☑	2295	1256.10	2510.19	2510.25	-0.06	0	115	1.3e-09	1	K.TNDLAGDGTTTSAVLVASVFSESLR.C
☑	2321	1287.17	2572.33	2572.29	0.04	0	(67)	7.5e-05	1	R.LVGEEGSGLELDAENFDPAILGTVK.K
☑	2322	858.46	2572.35	2572.29	0.06	0	73	2.2e-05	1	R.LVGEEGSGLELDAENFDPAILGTVK.K

☑ 2323	858.63	2572.87	2572.29	0.58	0	(1)	4.7e+02	1	R.LVGEEGSGLELDAENFDPAILGTVK.K

Proteins matching the same set of peptides:
gi|71665068 **Mass:** 59129 **Score:** 851 **Queries matched:** 27
chaperonin HSP60, mitochondrial precursor [Trypanosoma cruzi strain CL Brener]

---

2. gi|71402301 **Mass:** 32319 **Score:** 367 **Queries matched:** 6
surface protein TolT [Trypanosoma cruzi strain CL Brener]
☐ Check to include this hit in error tolerant search or archive report

Query	Observed	Mr(expt)	Mr(calc)	Delta	Miss	Score	Expect	Rank	Peptide
☑ 1267	583.74	1165.47	1165.50	-0.03	0	59	0.00056	1	K.AQEYADEANR.H
☑ 209	594.79	1187.56	1187.64	-0.08	0	68	0.00026	1	R.AAEQTVLSLEK.A
☑ 1680	750.92	1499.83	1499.83	0.00	0	119	5.4e-10	1	R.TLAQDVAATASALLR.Q
☑ 1681	500.98	1499.93	1499.83	0.10	0	(56)	0.0013	1	R.TLAQDVAATASALLR.Q
☑ 2115	1079.54	2157.06	2157.10	-0.04	0	144	1.5e-12	1	K.AAEEAAQAAGIMTLDAVGEVLK.H
☑ 2117	720.11	2157.30	2157.10	0.20	0	(98)	6.6e-08	1	K.AAEEAAQAAGIMTLDAVGEVLK.H

---

3. gi|71651556 **Mass:** 60535 **Score:** 309 **Queries matched:** 9
hypothetical protein [Trypanosoma cruzi strain CL Brener]
☐ Check to include this hit in error tolerant search or archive report

Query	Observed	Mr(expt)	Mr(calc)	Delta	Miss	Score	Expect	Rank	Peptide
☑ 1012	518.28	1034.56	1034.56	-0.01	0	40	0.052	1	R.FVLASIEEK.E
☑ 1399	620.26	1238.50	1238.56	-0.05	0	83	2.2e-06	1	K.APEAAAEFDYR.E
☑ 1400	620.26	1238.51	1238.56	-0.05	0	(40)	0.047	1	K.APEAAAEFDYR.E
☑ 1437	631.78	1261.54	1261.54	-0.00	0	74	2.1e-05	1	R.DLDSAEEEAQR.S
☑ 1441	632.31	1262.61	1262.65	-0.04	0	57	0.00099	1	R.QLDSINVFAEK.Y
☑ 1485	649.30	1296.58	1296.60	-0.02	0	99	6.9e-08	1	K.VYASQEDMINK.N
☑ 1670	743.83	1485.65	1485.71	-0.07	0	89	5.7e-07	1	R.SHVQQVWTSVCR.E + Carbamidomethyl (C)
☑ 1671	496.23	1485.67	1485.71	-0.05	0	(23)	2.6	1	R.SHVQQVWTSVCR.E + Carbamidomethyl (C)
☑ 1896	896.02	1790.02	1789.96	0.06	0	86	1.2e-06	1	K.AVESQLQVYSEVIGLR.K

Proteins matching the same set of peptides:

gi|71651558 **Mass:** 42796 **Score:** 309 **Queries matched:** 9
hypothetical protein [Trypanosoma cruzi strain CL Brener]

---

4. gi|71662736 **Mass:** 42788 **Score:** 305 **Queries matched:** 9
hypothetical protein [Trypanosoma cruzi strain CL Brener]

☐ Check to include this hit in error tolerant search or archive report

	Query	Observed	Mr(expt)	Mr(calc)	Delta	Miss	Score	Expect	Rank	Peptide
	1012	518.28	1034.56	1034.56	-0.01	0	40	0.052	1	R.FVLASIEEK.E
	1399	620.26	1238.50	1238.56	-0.05	0	83	2.2e-06	1	K.APEAAAEFDYR.E
	1400	620.26	1238.51	1238.56	-0.05	0	(40)	0.047	1	K.APEAAAEFDYR.E
☑	**1436**	**631.27**	**1260.53**	**1260.56**	**-0.03**	**0**	**70**	**5.5e-05**	**1**	**R.DLNSAEEEAQR.S**
	1441	632.31	1262.61	1262.65	-0.04	0	57	0.00099	1	R.QLDSINVFAEK.Y
	1485	649.30	1296.58	1296.60	-0.02	0	99	6.9e-08	1	K.VYASQEDMINK.N
	1670	743.83	1485.65	1485.71	-0.07	0	89	5.7e-07	1	R.SHVQQVWTSVCR.E + Carbamidomethyl (C)
	1671	496.23	1485.67	1485.71	-0.05	0	(23)	2.6	1	R.SHVQQVWTSVCR.E + Carbamidomethyl (C)
	1896	896.02	1790.02	1789.96	0.06	0	86	1.2e-06	1	K.AVESQLQVYSEVIGLR.K

---

5. gi|71400377 **Mass:** 85164 **Score:** 283 **Queries matched:** 10
microtubule-associated protein [Trypanosoma cruzi strain CL Brener]

☐ Check to include this hit in error tolerant search or archive report

	Query	Observed	Mr(expt)	Mr(calc)	Delta	Miss	Score	Expect	Rank	Peptide
☑	**1639**	**726.84**	**1451.67**	**1451.73**	**-0.06**	**0**	**84**	**2e-06**	**1**	**R.ALPLEEQEDVGPR.H**
☑	**1640**	**727.34**	**1452.67**	**1452.71**	**-0.04**	**0**	**81**	**3.3e-06**	**1**	**R.ALPLEEEEDVGPR.H**
☑	**1653**	**734.34**	**1466.67**	**1466.70**	**-0.03**	**0**	**74**	**2.1e-05**	**1**	**R.ALPQEEQEDVGPR.H**
☑	**1654**	**734.81**	**1467.61**	**1467.68**	**-0.07**	**0**	**65**	**0.00013**	**1**	**R.ALPQEEEEDVGPR.H**
☑	**1657**	**735.32**	**1468.62**	**1468.74**	**-0.12**	**0**	**25**	**1.4**	**1**	**R.ALLLEEEEDVGPR.H**
☑	**1781**	**541.94**	**1622.81**	**1622.80**	**0.01**	**1**	**61**	**0.00035**	**1**	**K.RALPQEEQEDVGPR.H**
☑	**1782**	**542.29**	**1623.85**	**1623.79**	**0.07**	**1**	**54**	**0.002**	**1**	**K.RALPQEEEEDVGPR.H**
☑	**1902**	**600.27**	**1797.79**	**1797.85**	**-0.06**	**0**	**(21)**	**4.2**	**1**	**R.STTQDAYRPVDPSAYK.R**
☑	**1903**	**899.91**	**1797.80**	**1797.85**	**-0.05**	**0**	**41**	**0.04**	**1**	**R.STTQDAYRPVDPSAYK.R**
☑	**1904**	**600.28**	**1797.82**	**1797.85**	**-0.03**	**0**	**(20)**	**5.2**	**1**	**R.STTQDAYRPVDPSAYK.R**

6. gi|71401264 **Mass:** 30868 **Score:** 273 **Queries matched:** 7

hypothetical protein [Trypanosoma cruzi strain CL Brener]

☐ Check to include this hit in error tolerant search or archive report

	Query	Observed	Mr(expt)	Mr(calc)	Delta	Miss	Score	Expect	Rank	Peptide
☑	1114	544.73	1087.45	1087.49	-0.04	0	52	0.0041	1	R.HCVQAGMSAK.N + Carbamidomethyl (C)
☑	1115	544.73	1087.45	1087.49	-0.04	0	(27)	1.1	1	R.HCVQAGMSAK.N + Carbamidomethyl (C)
☑	1116	544.74	1087.47	1087.49	-0.02	0	(25)	2	1	R.HCVQAGMSAK.N + Carbamidomethyl (C)
	1267	583.74	1165.47	1165.50	-0.03	0	59	0.00056	1	K.AQEYADEANR.H
	209	594.79	1187.56	1187.64	-0.08	0	68	0.00026	1	R.AAEQTVLSLEK.A
	2115	1079.54	2157.06	2157.10	-0.04	0	144	1.5e-12	1	K.AAEEAAQAAGIMTLDAVGEVLK.H
	2117	720.11	2157.30	2157.10	0.20	0	(98)	6.6e-08	1	K.AAEEAAQAAGIMTLDAVGEVLK.H

7. gi|71425779 **Mass:** 23211 **Score:** 245 **Queries matched:** 4

I/6 autoantigen [Trypanosoma cruzi strain CL Brener]

☐ Check to include this hit in error tolerant search or archive report

	Query	Observed	Mr(expt)	Mr(calc)	Delta	Miss	Score	Expect	Rank	Peptide
☑	1415	623.31	1244.61	1244.60	0.00	0	90	5.7e-07	1	M.PISPAEAFEER.H
☑	1872	585.35	1753.02	1752.90	0.11	1	56	0.0011	1	K.KEEPTSPPPPPPPQQK.K
☑	655	878.55	1755.08	1754.99	0.09	0	72	9.1e-05	1	K.VIPPSLALVAALESGYR.F
☑	2222	1169.51	2337.01	2337.10	-0.09	0	119	5.1e-10	1	K.TGSSQDALSADEIDALFDVLDR.E

8. gi|71413559 **Mass:** 44080 **Score:** 244 **Queries matched:** 13

hypothetical protein [Trypanosoma cruzi strain CL Brener]

☐ Check to include this hit in error tolerant search or archive report

	Query	Observed	Mr(expt)	Mr(calc)	Delta	Miss	Score	Expect	Rank	Peptide
☑	1187	563.31	1124.60	1124.63	-0.03	0	(40)	0.054	1	R.QFIPASLPPR.Q
☑	1188	563.31	1124.61	1124.63	-0.02	0	43	0.022	1	R.QFIPASLPPR.Q
☑	1209	568.79	1135.58	1135.56	0.01	0	51	0.0033	1	K.GVEDVEPVHR.K
☑	1311	594.32	1186.62	1186.63	-0.01	0	63	0.00027	1	K.ALTEEWILGR.K

	299	645.77	1289.54	1289.64	-0.10	0	12	1.4e+02	3	K.EDDPVPQHVVR.Q
☑	1614	717.85	1433.68	1433.71	-0.03	0	63	0.00024	1	R.FHEQTSVNLFGR.A
☑	1615	478.92	1433.74	1433.71	0.04	0	(13)	23	1	R.FHEQTSVNLFGR.A
☑	1623	719.90	1437.78	1437.76	0.02	0	37	0.1	1	K.IVHASPSDFLAGPK.E
☑	1629	482.01	1443.02	1442.84	0.18	0	27	0.94	1	R.VLALNNPPPRPQK.A
☑	1734	783.92	1565.82	1565.86	-0.04	1	55	0.0014	1	K.KIVHASPSDFLAGPK.E
☑	1756	530.97	1589.89	1589.81	0.08	1	58	0.00079	1	R.RFHEQTSVNLFGR.A
☑	1830	846.93	1691.84	1691.85	-0.00	0	87	1.2e-06	1	K.ANESLDVLNLGQYTR.E
☑	281	637.33	1908.97	1908.97	0.01	0	42	0.075	1	K.DLVEAPARPASANAAAGGSGK.K

---

9. gi|349838 **Mass:** 71488 **Score:** 238 **Queries matched:** 14

heat shock protein

☐ Check to include this hit in error tolerant search or archive report

	Query	Observed	Mr(expt)	Mr(calc)	Delta	Miss	Score	Expect	Rank	Peptide
	637	435.28	868.54	868.51	0.03	0	25	1.2	2	K.GTLVPVQR.V
☑	729	458.24	914.46	914.46	0.00	0	30	0.58	1	R.YFVDMLK.K
☑	936	502.78	1003.55	1003.48	0.06	0	20	6	1	R.LSEEEIER.M
	186	583.83	1165.64	1165.55	0.09	1	3	7.5e+02	3	R.DFFGGKEPNR.G
☑	1487	650.37	1298.72	1298.62	0.10	0	56	0.0011	1	K.FEELNMELFK.G
	1508	658.31	1314.61	1314.62	-0.00	0	(19)	6.7	3	K.FEELNMELFK.G + Oxidation (M)
☑	1620	719.32	1436.63	1436.68	-0.05	0	38	0.085	1	K.GDVHIIPNDMGNR.I
☑	1621	479.89	1436.64	1436.68	-0.04	0	(33)	0.23	1	K.GDVHIIPNDMGNR.I
☑	1635	725.38	1448.75	1448.75	-0.01	0	85	1.4e-06	1	R.ITPSVVAFTETER.L
☑	1666	741.79	1481.56	1481.78	-0.22	0	60	0.0005	1	K.SDIHEIVLVGGSTR.V
☑	1678	500.31	1497.90	1497.75	0.15	1	47	0.0089	1	R.AKFEELNMELFK.G
☑	1842	851.34	1700.67	1700.78	-0.10	0	94	1.9e-07	1	R.VEVDSLTEGFDFSEK.I
☑	2166	747.14	2238.39	2238.29	0.10	0	37	0.097	1	R.VVLVDVIPLSLGIETVGGVMTK.L
☑	2354	901.16	2700.45	2700.34	0.12	0	48	0.0055	1	R.GINPDEAVAYGAAVQAAVLTGESEVGGR.V

Proteins matching the same set of peptides:

gi|50659756 **Mass:** 71330 **Score:** 238 **Queries matched:** 14

heat shock protein 70 [Trypanosoma cruzi]

gi|71415505 **Mass:** 71272 **Score:** 238 **Queries matched:** 14

glucose-regulated protein 78 [Trypanosoma cruzi strain CL Brener]

---

10. gi|18568139 **Mass:** 49668 **Score:** 227 **Queries matched:** 6
beta tubulin 1.9 [Trypanosoma cruzi]

☐ Check to include this hit in error tolerant search or archive report

	Query	Observed	Mr(expt)	Mr(calc)	Delta	Miss	Score	Expect	Rank	Peptide
☑	1007	517.26	1032.50	1032.45	0.06	0	46	0.013	1	K.NMMQAADPR.H
☑	1531	671.28	1340.55	1340.64	-0.09	0	87	1e-06	1	R.INVYFDEATGGR.Y
☑	1630	723.83	1445.65	1445.68	-0.03	0	51	0.0039	1	K.EVDEQMLNVQNK.N
☑	1773	808.43	1614.85	1614.83	0.02	0	53	0.0024	1	R.AVLIDLEPGTMDSVR.A
☑	1946	924.44	1846.86	1846.87	-0.01	0	87	1e-06	1	R.EIVCVQAGQCGNQIGSK.F + 2 Carbamidomethyl (C)
☑	2433	1031.78	3092.33	3092.40	-0.07	0	81	3.3e-06	1	K.FWEVISDEHGVDPTGTYQGDSDLQLER.I

Proteins matching the same set of peptides:
gi|71656281 **Mass:** 49668 **Score:** 227 **Queries matched:** 6
beta tubulin [Trypanosoma cruzi strain CL Brener]

---

11. gi|71655864 **Mass:** 23105 **Score:** 226 **Queries matched:** 4
I/6 autoantigen [Trypanosoma cruzi strain CL Brener]

☐ Check to include this hit in error tolerant search or archive report

	Query	Observed	Mr(expt)	Mr(calc)	Delta	Miss	Score	Expect	Rank	Peptide
	1415	623.31	1244.61	1244.60	0.00	0	90	5.7e-07	1	M.PISPAEAFEER.H
	655	878.55	1755.08	1754.99	0.09	0	72	9.1e-05	1	K.VIPPSLALVAALESGYR.F
☑	1880	588.66	1762.95	1762.93	0.02	1	35	0.16	1	K.KEEPTPPPPPPPPQQK.K
	2222	1169.51	2337.01	2337.10	-0.09	0	119	5.1e-10	1	K.TGSSQDALSADEIDALFDVLDR.E

---

12. gi|71407337 **Mass:** 16350 **Score:** 218 **Queries matched:** 10
hypothetical protein [Trypanosoma cruzi strain CL Brener]

☐ Check to include this hit in error tolerant search or archive report

	Query	Observed	Mr(expt)	Mr(calc)	Delta	Miss	Score	Expect	Rank	Peptide
☑	864	488.24	974.46	974.45	0.00	0	22	4.8	1	K.NMYTGVYK.A

Query	Observed	Mr(expt)	Mr(calc)	Delta	Miss	Score	Expect	Rank	Peptide
☑ 1017	519.30	1036.58	1036.54	0.04	0	39	0.057	1	K.ITLSDFVDK.A
☑ 1124	545.76	1089.51	1089.54	-0.03	0	71	4.1e-05	1	R.NSGSLSGVVDR.R
☑ 1125	545.84	1089.67	1089.54	0.13	0	(17)	11	1	R.NSGSLSGVVDR.R
☑ 1265	583.31	1164.61	1164.64	-0.03	1	65	0.00015	1	R.KITLSDFVDK.A
☑ 1679	750.34	1498.67	1498.73	-0.06	0	42	0.03	1	K.QFTSTDADLLFNK.V
☑ 1775	809.33	1616.65	1616.73	-0.08	1	79	6e-06	1	K.FHDDKNMYTGVYK.A
☑ 1776	539.91	1616.71	1616.73	-0.02	1	(50)	0.0051	1	K.FHDDKNMYTGVYK.A
☑ 1932	917.90	1833.78	1833.84	-0.06	0	82	3.1e-06	1	K.SAEELIADISSCSPEAR.A + Carbamidomethyl (C)
☑ 1995	655.07	1962.19	1961.94	0.25	1	12	34	1	K.KSAEELIADISSCSPEAR.A + Carbamidomethyl (C)

---

13. gi|71667953 **Mass:** 45869 **Score:** 194 **Queries matched:** 12

hypothetical protein [Trypanosoma cruzi strain CL Brener]

☐ Check to include this hit in error tolerant search or archive report

Query	Observed	Mr(expt)	Mr(calc)	Delta	Miss	Score	Expect	Rank	Peptide
☑ 722	456.74	911.46	911.41	0.04	0	41	0.029	1	R.FEEFANR.A
925	500.36	998.70	998.65	0.05	2	1	3.2e+02	5	R.LKKADLIAK.I
☑ 1052	529.83	1057.65	1057.60	0.05	0	60	0.00052	1	K.LQVELLDTK.H
1138	549.77	1097.53	1097.57	-0.04	0	(6)	1e+02	3	K.NVLPEEIER.V
1140	549.83	1097.64	1097.57	0.07	0	10	40	5	K.NVLPEEIER.V
☑ 1338	602.83	1203.65	1203.63	0.02	0	43	0.024	1	R.IQSILSETGEK.E
☑ 1401	620.31	1238.61	1238.54	0.07	0	31	0.34	1	K.ETQEYVDEAR.L
☑ 1492	650.86	1299.71	1299.70	0.01	0	99	8.1e-08	1	R.LEELQAELLSR.Q
☑ 1575	462.20	1383.58	1383.64	-0.06	0	58	0.00075	1	R.HHSDDEVAAYIK.E
☑ 1576	692.82	1383.62	1383.64	-0.02	0	(53)	0.0023	1	R.HHSDDEVAAYIK.E
☑ 1611	714.39	1426.76	1426.73	0.03	0	51	0.0042	1	K.ELPELQVLSEDR.A
☑ 1612	714.39	1426.77	1426.73	0.04	0	(34)	0.22	1	K.ELPELQVLSEDR.A

---

14. gi|71651158 **Mass:** 16325 **Score:** 189 **Queries matched:** 9

hypothetical protein [Trypanosoma cruzi strain CL Brener]

☐ Check to include this hit in error tolerant search or archive report

Query	Observed	Mr(expt)	Mr(calc)	Delta	Miss	Score	Expect	Rank	Peptide
864	488.24	974.46	974.45	0.00	0	22	4.8	1	K.NMYTGVYK.A

	Query	Observed	Mr(expt)	Mr(calc)	Delta	Miss	Score	Expect	Rank	Peptide
	1017	519.30	1036.58	1036.54	0.04	0	39	0.057	1	K.ITLSDFVDK.A
	1124	545.76	1089.51	1089.54	-0.03	0	71	4.1e-05	1	R.NSGSLSGVVDR.R
	1125	545.84	1089.67	1089.54	0.13	0	(17)	11	1	R.NSGSLSGVVDR.R
	1265	583.31	1164.61	1164.64	-0.03	1	65	0.00015	1	R.KITLSDFVDK.A
	1679	750.34	1498.67	1498.73	-0.06	0	42	0.03	1	K.QFTSTDADLLFNK.V
	1775	809.33	1616.65	1616.73	-0.08	1	79	6e-06	1	K.FHDDKNMYTGVYK.A
	1776	539.91	1616.71	1616.73	-0.02	1	(50)	0.0051	1	K.FHDDKNMYTGVYK.A
☑	255	**621.64**	**1861.90**	**1861.87**	**0.03**	**0**	**45**	**0.042**	**1**	**K.SVEELIADISSCSPEAR.A + Carbamidomethyl (C)**

15. gi|120679 **Mass:** 39036 **Score:** 178 **Queries matched:** 9
RecName: Full=Glyceraldehyde-3-phosphate dehydrogenase, glycosomal; Short=GAPDH
☐ Check to include this hit in error tolerant search or archive report

	Query	Observed	Mr(expt)	Mr(calc)	Delta	Miss	Score	Expect	Rank	Peptide
	540	**406.22**	**810.43**	**810.41**	**0.02**	**0**	**17**	**7.7**	**5**	**K.LTGMSFR.V**
☑	1312	**594.34**	**1186.66**	**1186.64**	**0.02**	**0**	**34**	**0.21**	**1**	**K.AVGMVIPSTQGK.L**
☑	1577	**692.86**	**1383.71**	**1383.77**	**-0.06**	**0**	**59**	**0.0006**	**1**	**R.AAAVNIIPSTTGAAK.A**
☑	1647	**487.27**	**1458.80**	**1458.77**	**0.03**	**1**	**38**	**0.077**	**1**	**R.DTSIQEIDAALKR.A**
☑	1892	**894.48**	**1786.94**	**1786.95**	**-0.00**	**0**	**(16)**	**13**	**1**	**R.VPTPDVSVVDLTFTAAR.D**
☑	1893	**894.49**	**1786.96**	**1786.95**	**0.01**	**0**	**102**	**3.3e-08**	**1**	**R.VPTPDVSVVDLTFTAAR.D**
☑	1894	**596.72**	**1787.14**	**1786.95**	**0.19**	**0**	**(52)**	**0.0032**	**1**	**R.VPTPDVSVVDLTFTAAR.D**
	1913	**604.59**	**1810.74**	**1810.81**	**-0.06**	**0**	**13**	**21**	**2**	**K.IVSWYDNEWGYSHR.V**
☑	2168	**1121.07**	**2240.12**	**2240.06**	**0.06**	**0**	**33**	**0.25**	**1**	**K.GILGYTDEELVSADFINDNR.S**

Proteins matching the same set of peptides:
gi|71422448 **Mass:** 39008 **Score:** 178 **Queries matched:** 9
glyceraldehyde 3-phosphate dehydrogenase [Trypanosoma cruzi strain CL Brener]
gi|71650185 **Mass:** 38993 **Score:** 178 **Queries matched:** 9
glyceraldehyde 3-phosphate dehydrogenase [Trypanosoma cruzi strain CL Brener]

16. gi|71659715 **Mass:** 18769 **Score:** 174 **Queries matched:** 6
cyclophilin A [Trypanosoma cruzi strain CL Brener]
☐ Check to include this hit in error tolerant search or archive report

	Query	Observed	Mr(expt)	Mr(calc)	Delta	Miss	Score	Expect	Rank	Peptide
☑	852	486.23	970.45	970.44	0.01	0	55	0.0014	1	K.FADESFAGK.A
☑	1091	539.75	1077.48	1077.51	-0.03	0	41	0.041	1	K.AMEAVGSQTGK.T
☑	1524	667.89	1333.76	1333.73	0.03	0	44	0.02	1	R.VVFELFADAVPK.T
☑	1624	720.38	1438.75	1438.72	0.03	0	79	6e-06	1	K.VFFDVSIGGQSAGR.V
☑	1801	551.66	1651.95	1651.93	0.02	0	(51)	0.0044	1	K.HVVFGQVLEGIEVVK.A
☑	1802	551.67	1652.00	1651.93	0.07	0	72	3.1e-05	1	K.HVVFGQVLEGIEVVK.A

---

17. gi|71659663 **Mass:** 31532 **Score:** 171 **Queries matched:** 3
hypothetical protein [Trypanosoma cruzi strain CL Brener]
☐ Check to include this hit in error tolerant search or archive report

	Query	Observed	Mr(expt)	Mr(calc)	Delta	Miss	Score	Expect	Rank	Peptide
☑	1790	818.35	1634.69	1634.78	-0.10	0	125	1.7e-10	1	R.MVSENESAINDVVTK.L
☑	1807	828.46	1654.90	1654.94	-0.03	0	66	0.00011	1	R.IVIDPSVPLLMPFSK.E
☑	1949	927.15	1852.29	1851.96	0.33	0	45	0.023	1	R.QSVPVSQELSFIYDLK.R

---

18. gi|71667211 **Mass:** 30381 **Score:** 163 **Queries matched:** 8
hypothetical protein [Trypanosoma cruzi strain CL Brener]
☐ Check to include this hit in error tolerant search or archive report

	Query	Observed	Mr(expt)	Mr(calc)	Delta	Miss	Score	Expect	Rank	Peptide
☑	1592	703.85	1405.69	1405.60	0.09	1	68	0.0001	1	K.EEFKDDFDFSK.S
☑	1743	786.98	1571.94	1571.92	0.01	0	61	0.00041	1	R.AIGSTITLITNSLIR.Y
☑	1744	525.04	1572.10	1571.92	0.17	0	(26)	1.5	1	R.AIGSTITLITNSLIR.Y
	1861	576.63	1726.87	1726.84	0.03	0	9	56	2	R.VDGHTGQDFRPATGAAK.E
☑	2174	750.04	2247.10	2247.11	-0.01	0	(56)	0.0011	1	R.YEGTLGQIDGPNNTVSLTNVR.V
☑	2175	1124.56	2247.11	2247.11	-0.00	0	72	2.8e-05	1	R.YEGTLGQIDGPNNTVSLTNVR.V
☑	2256	805.09	2412.23	2412.24	-0.01	0	25	1.4	1	R.GQEAGLAQIPPADQLFDQIVFR.G
☑	2341	880.13	2637.36	2637.29	0.07	0	20	3.4	1	K.ELTVFEEPHNAMMDPAVVTALPAR.N

---

19. gi|71656813 **Mass:** 34046 **Score:** 156 **Queries matched:** 6
glycosomal malate dehydrogenase [Trypanosoma cruzi strain CL Brener]

☐ Check to include this hit in error tolerant search or archive report

	Query	Observed	Mr(expt)	Mr(calc)	Delta	Miss	Score	Expect	Rank	Peptide
☑	994	515.29	1028.57	1028.59	-0.02	0	42	0.036	1	R.VQVAGTEVVK.A
☑	1249	578.39	1154.76	1154.70	0.06	0	78	7.8e-06	1	K.LLGVSLLDGLR.A
☑	1335	602.31	1202.61	1202.62	-0.01	0	49	0.0078	1	K.EMLEEAVGVVK.K
☑	1514	441.91	1322.70	1322.79	-0.09	1	(31)	0.35	1	R.RLPIGPITTVEK.E
☑	1515	662.38	1322.75	1322.79	-0.05	1	32	0.31	1	R.RLPIGPITTVEK.E
☑	2096	707.79	2120.35	2120.27	0.08	0	93	2.3e-07	1	M.VNVAVIGAAGGIGQSLSLLLLR.E

---

20. gi|2961256 **Mass:** 34112 **Score:** 156 **Queries matched:** 7
putative malate dehydrogenase [Trypanosoma cruzi]
☐ Check to include this hit in error tolerant search or archive report

	Query	Observed	Mr(expt)	Mr(calc)	Delta	Miss	Score	Expect	Rank	Peptide
	994	515.29	1028.57	1028.59	-0.02	0	42	0.036	1	R.VQVAGTEVVK.A
	1249	578.39	1154.76	1154.70	0.06	0	78	7.8e-06	1	K.LLGVSLLDGLR.A
	1335	602.31	1202.61	1202.62	-0.01	0	49	0.0078	1	K.EMLEEAVGVVK.K
	1514	441.91	1322.70	1322.79	-0.09	1	(31)	0.35	1	R.RLPIGPITTVEK.E
	1515	662.38	1322.75	1322.79	-0.05	1	32	0.31	1	R.RLPIGPITTVEK.E
	2096	707.79	2120.35	2120.27	0.08	0	93	2.3e-07	1	M.VNVAVIGAAGGIGQSLSLLLLR.E
☑	2389	957.84	2870.49	2870.44	0.05	0	19	5.2	1	R.ELPFGSTLSLYDVAGAPGVAADLSHIDR.A

Proteins matching the same set of peptides:
gi|71423452 **Mass:** 34063 **Score:** 156 **Queries matched:** 7
glycosomal malate dehydrogenase [Trypanosoma cruzi strain CL Brener]

---

21. gi|71664003 **Mass:** 20233 **Score:** 154 **Queries matched:** 2
ATP synthase, epsilon chain [Trypanosoma cruzi strain CL Brener]
☐ Check to include this hit in error tolerant search or archive report

	Query	Observed	Mr(expt)	Mr(calc)	Delta	Miss	Score	Expect	Rank	Peptide
☑	1356	608.33	1214.64	1214.66	-0.02	0	88	7.8e-07	1	K.ALAAQQASLGSAK.D
☑	1410	621.91	1241.81	1241.76	0.05	0	105	1.4e-08	1	R.ISVLESVIAALK.H

22. gi|704459 **Mass:** 43546 **Score:** 149 **Queries matched:** 8
elongation factor 1 alpha [Trypanosoma cruzi]
☐ Check to include this hit in error tolerant search or archive report

	Query	Observed	Mr(expt)	Mr(calc)	Delta	Miss	Score	Expect	Rank	Peptide
☑	867	**488.28**	**974.55**	**974.54**	**0.01**	**0**	**63**	**0.00038**	**1**	**R.LPLQDVYK.I**
☑	985	**513.31**	**1024.61**	**1024.60**	**0.01**	**0**	**71**	**3.7e-05**	**1**	**K.IGGIGTVPVGR.V**
☑	2228	**784.39**	**2350.15**	**2350.13**	**0.02**	**0**	**22**	**2.6**	**1**	**R.MVPQKPMCVEVFNDYAPLGR.F + Carbamidomethyl (C)**
☑	2282	**831.05**	**2490.12**	**2490.27**	**-0.15**	**0**	**(25)**	**1.4**	**1**	**R.VETGTMKPGDVVTFAPANVTTEVK.S**
☑	2283	**1246.11**	**2490.20**	**2490.27**	**-0.07**	**0**	**(24)**	**1.5**	**1**	**R.VETGTMKPGDVVTFAPANVTTEVK.S**
☑	2284	**831.27**	**2490.79**	**2490.27**	**0.52**	**0**	**(14)**	**30**	**1**	**R.VETGTMKPGDVVTFAPANVTTEVK.S**
☑	2291	**836.41**	**2506.21**	**2506.26**	**-0.06**	**0**	**33**	**0.2**	**1**	**R.VETGTMKPGDVVTFAPANVTTEVK.S + Oxidation (M)**
☑	2298	**841.69**	**2522.05**	**2522.19**	**-0.14**	**0**	**83**	**1.8e-06**	**1**	**K.SIEMHHEQLAEATPGDNVGFNVK.N**

23. gi|1929445 **Mass:** 49018 **Score:** 149 **Queries matched:** 9
elongation factor 1-alpha [Trypanosoma cruzi]
☐ Check to include this hit in error tolerant search or archive report

	Query	Observed	Mr(expt)	Mr(calc)	Delta	Miss	Score	Expect	Rank	Peptide
☑	762	**464.80**	**927.58**	**927.58**	**0.00**	**0**	**24**	**2**	**1**	**R.QTVAVGIIK.A**
	867	488.28	974.55	974.54	0.01	0	63	0.00038	1	R.LPLQDVYK.I
	985	513.31	1024.61	1024.60	0.01	0	71	3.7e-05	1	K.IGGIGTVPVGR.V
	2228	784.39	2350.15	2350.13	0.02	0	22	2.6	1	R.MVPQKPMCVEVFNDYAPLGR.F + Carbamidomethyl (C)
	2282	831.05	2490.12	2490.27	-0.15	0	(25)	1.4	1	R.VETGTMKPGDVVTFAPANVTTEVK.S
	2283	1246.11	2490.20	2490.27	-0.07	0	(24)	1.5	1	R.VETGTMKPGDVVTFAPANVTTEVK.S
	2284	831.27	2490.79	2490.27	0.52	0	(14)	30	1	R.VETGTMKPGDVVTFAPANVTTEVK.S
	2291	836.41	2506.21	2506.26	-0.06	0	33	0.2	1	R.VETGTMKPGDVVTFAPANVTTEVK.S + Oxidation (M)
	2298	841.69	2522.05	2522.19	-0.14	0	83	1.8e-06	1	K.SIEMHHEQLAEATPGDNVGFNVK.N

Proteins matching the same set of peptides:
gi|52424046 **Mass:** 49067 **Score:** 149 **Queries matched:** 9
elongation factor alpha G5 [Trypanosoma cruzi]

gi|61207234 **Mass:** 47727 **Score:** 149 **Queries matched:** 9
elongation factor 1-alpha [Trypanosoma cruzi]
gi|61207240 **Mass:** 47728 **Score:** 149 **Queries matched:** 9
elongation factor 1-alpha [Trypanosoma cruzi]
gi|61207250 **Mass:** 47741 **Score:** 149 **Queries matched:** 9
elongation factor 1-alpha [Trypanosoma cruzi]
gi|61207254 **Mass:** 47727 **Score:** 149 **Queries matched:** 9
elongation factor 1-alpha [Trypanosoma cruzi]
gi|61207256 **Mass:** 47843 **Score:** 149 **Queries matched:** 9
elongation factor 1-alpha [Trypanosoma cruzi]
gi|61207272 **Mass:** 47767 **Score:** 149 **Queries matched:** 9
elongation factor 1-alpha [Trypanosoma cruzi]
gi|61207288 **Mass:** 47711 **Score:** 149 **Queries matched:** 9
elongation factor 1-alpha [Trypanosoma cruzi]
gi|61207296 **Mass:** 47667 **Score:** 149 **Queries matched:** 9
elongation factor 1-alpha [Trypanosoma cruzi]
gi|71408910 **Mass:** 49083 **Score:** 149 **Queries matched:** 9
elongation factor 1-alpha (EF-1-alpha) [Trypanosoma cruzi strain CL Brener]
gi|71664927 **Mass:** 49097 **Score:** 149 **Queries matched:** 9
elongation factor 1-alpha (EF-1-alpha) [Trypanosoma cruzi strain CL Brener]

---

24. gi|71413449 **Mass:** 31077 **Score:** 147 **Queries matched:** 3
RNA-binding protein [Trypanosoma cruzi strain CL Brener]
☐ Check to include this hit in error tolerant search or archive report

	Query	Observed	Mr(expt)	Mr(calc)	Delta	Miss	Score	Expect	Rank	Peptide
☑	277	631.20	1260.39	1260.51	-0.12	0	58	0.0031	1	R.MYNETDFEGR.R
☑	1499	653.82	1305.62	1305.63	-0.01	0	69	7.2e-05	1	R.ETFQQVGNVER.A
☑	1765	804.35	1606.68	1606.77	-0.09	0	96	1.4e-07	1	R.LAIEEFDGIEMANR.A

Proteins matching the same set of peptides:
gi|71417144 **Mass:** 31047 **Score:** 147 **Queries matched:** 3
RNA-binding protein [Trypanosoma cruzi strain CL Brener]

---

25. gi|91983204 **Mass:** 14516 **Score:** 143 **Queries matched:** 4

beta tubulin [Trypanosoma pestanai]

☐ Check to include this hit in error tolerant search or archive report

Query	Observed	Mr(expt)	Mr(calc)	Delta	Miss	Score	Expect	Rank	Peptide
1515	662.38	1322.75	1322.50	0.24	1	11	36	4	R.KEAEFCDCHQG.- + Carbamidomethyl (C)
1531	671.28	1340.55	1340.64	-0.09	0	87	1e-06	1	R.INVYFDEATGGR.Y
1773	808.43	1614.85	1614.83	0.02	0	53	0.0024	1	R.AVLIDLEPGTMDSVR.A
2433	1031.78	3092.33	3092.40	-0.07	0	81	3.3e-06	1	K.FWEVISDEHGVDPTGTYQGDSDLQLER.I

---

26. gi|154345814 **Mass:** 60177 **Score:** 143 **Queries matched:** 5

chaperonin Hsp60, mitochondrial precursor [Leishmania braziliensis MHOM/BR/75/M2904]

☐ Check to include this hit in error tolerant search or archive report

Query	Observed	Mr(expt)	Mr(calc)	Delta	Miss	Score	Expect	Rank	Peptide
588	422.26	842.51	842.52	-0.02	0	56	0.0015	1	K.LSGGVAVIK.V
1336	602.32	1202.62	1202.58	0.04	0	105	1.8e-08	1	K.VGGGSEVEVNEK.K
1997	655.69	1964.05	1963.98	0.07	1	53	0.0031	1	K.AIEFKDPFENMGAQLVR.Q
2294	837.71	2510.11	2510.24	-0.12	0	(19)	5.2	2	K.TNDLAGDGTTTSAVLVDSIFSEGLK.S
2295	1256.10	2510.19	2510.24	-0.05	0	34	0.15	2	K.TNDLAGDGTTTSAVLVDSIFSEGLK.S

Proteins matching the same set of peptides:

gi|154345816 **Mass:** 59476 **Score:** 143 **Queries matched:** 5

chaperonin Hsp60, mitochondrial precursor [Leishmania braziliensis MHOM/BR/75/M2904]

---

27. gi|71659763 **Mass:** 10380 **Score:** 138 **Queries matched:** 2

dynein light chain [Trypanosoma cruzi strain CL Brener]

☐ Check to include this hit in error tolerant search or archive report

	Query	Observed	Mr(expt)	Mr(calc)	Delta	Miss	Score	Expect	Rank	Peptide
☑	307	**650.27**	**649.27**	**649.34**	**-0.08**	**0**	**16**	**14**	**1**	**K.FNIEK.D**
☑	2259	**1210.54**	**2419.06**	**2419.07**	**-0.01**	**0**	**138**	**6.2e-12**	**1**	**K.NADMPEDMQADAIEVALQAMEK.F**

---

28. gi|89329739 **Mass:** 53354 **Score:** 132 **Queries matched:** 4

heat shock protein 70 [Capsaspora owczarzaki]

☐ Check to include this hit in error tolerant search or archive report

	Query	Observed	Mr(expt)	Mr(calc)	Delta	Miss	Score	Expect	Rank	Peptide
	1379	**614.82**	**1227.62**	**1227.62**	**0.00**	**0**	**63**	**0.00029**	**2**	**K.VEIIANDQGNR.T**
	1487	650.37	1298.72	1298.60	0.12	0	24	1.9	2	R.FEELCADLFR.G + Carbamidomethyl (C)
☑	1672	**744.34**	**1486.67**	**1486.69**	**-0.03**	**0**	**68**	**7.3e-05**	**1**	**R.TTPSYVAFTDTER.L**
☑	1808	**830.46**	**1658.90**	**1658.89**	**0.01**	**0**	**74**	**1.8e-05**	**1**	**R.IINEPTAAAIAYGLDK.K**

---

29. gi|55824400 **Mass:** 66906 **Score:** 132 **Queries matched:** 4
heat shock protein 70 cytosolic isoform [Rhynchobodo ATCC50359]

☐ Check to include this hit in error tolerant search or archive report

Query	Observed	Mr(expt)	Mr(calc)	Delta	Miss	Score	Expect	Rank	Peptide
1379	614.82	1227.62	1227.62	0.00	0	63	0.00029	2	R.VEIIANDQGNR.T
1665	**494.61**	**1480.81**	**1480.81**	**0.00**	**1**	**2**	**3.6e+02**	**5**	**K.RSVHDVVLVGGSTR.I**
1672	744.34	1486.67	1486.69	-0.03	0	68	7.3e-05	1	R.TTPSYVAFTDTER.L
1808	830.46	1658.90	1658.89	0.01	0	74	1.8e-05	1	R.IINEPTAAAIAYGLDK.M

---

30. gi|4389144 **Mass:** 27110 **Score:** 130 **Queries matched:** 4
Chain A, Trypanosoma Cruzi Triosephosphate Isomerase

☐ Check to include this hit in error tolerant search or archive report

	Query	Observed	Mr(expt)	Mr(calc)	Delta	Miss	Score	Expect	Rank	Peptide
☑	1386	**616.84**	**1231.67**	**1231.67**	**0.00**	**0**	**95**	**1.9e-07**	**1**	**K.FQIAAQNAITR.S**
	1634	**725.33**	**1448.65**	**1448.79**	**-0.14**	**0**	**31**	**0.36**	**2**	**R.SGAFTGEVSLQILK.D**
☑	1707	**764.84**	**1527.66**	**1527.75**	**-0.09**	**0**	**72**	**3.4e-05**	**1**	**R.LYYGETNEIVAEK.V**
☑	1857	**573.63**	**1717.87**	**1717.91**	**-0.05**	**0**	**25**	**1.6**	**1**	**K.VATPQQAQEVHELLR.R**

Proteins matching the same set of peptides:

gi|160877706 **Mass:** 27189 **Score:** 130 **Queries matched:** 4
Chain A, Crystallographic Analysis Of A Chemically Modified Triosephosphate Isomerase From Trypanos

gi|1730005 **Mass:** 27312 **Score:** 130 **Queries matched:** 4
RecName: Full=Triosephosphate isomerase, glycosomal; Short=TIM; Short=Triose-phosphate isomerase

gi|71662494 **Mass:** 27288 **Score:** 130 **Queries matched:** 4
triosephosphate isomerase [Trypanosoma cruzi strain CL Brener]

---

31. gi|119859 **Mass:** 23721 **Score:** 130 **Queries matched:** 5
RecName: Full=Flagellar calcium-binding protein; Short=FCABP; AltName: Full=1F8 protein; AltName: F
☐ Check to include this hit in error tolerant search or archive report

	Query	Observed	Mr(expt)	Mr(calc)	Delta	Miss	Score	Expect	Rank	Peptide
☑	653	439.24	876.47	876.43	0.03	0	43	0.028	1	K.LDEFTPR.V
☑	899	495.30	988.58	988.52	0.06	0	31	0.4	1	K.VEDPAALFK.E
☑	1598	707.77	1413.53	1413.59	-0.06	0	55	0.0015	1	K.LDADGDPDNVPESA.-
☑	1662	737.90	1473.78	1473.68	0.11	0	90	5.1e-07	1	K.GSEDFVEFLEFR.L
☑	1924	607.96	1820.84	1820.84	-0.00	0	47	0.0098	1	K.LCYDEVHSGCLEVLK.L + 2 Carbamidomethyl (C)

Proteins matching the same set of peptides:
gi|1552212 **Mass:** 23727 **Score:** 130 **Queries matched:** 5
calcium-binding protein [Trypanosoma cruzi]
gi|71406002 **Mass:** 23827 **Score:** 130 **Queries matched:** 5
flagellar calcium-binding protein [Trypanosoma cruzi strain CL Brener]
gi|71406004 **Mass:** 23799 **Score:** 130 **Queries matched:** 5
flagellar calcium-binding protein [Trypanosoma cruzi strain CL Brener]
gi|71412217 **Mass:** 23707 **Score:** 130 **Queries matched:** 5
calcium-binding protein [Trypanosoma cruzi strain CL Brener]
gi|71656918 **Mass:** 23713 **Score:** 130 **Queries matched:** 5
flagellar calcium-binding protein [Trypanosoma cruzi strain CL Brener]

---

32. gi|71407149 **Mass:** 46733 **Score:** 128 **Queries matched:** 3
hypothetical protein [Trypanosoma cruzi strain CL Brener]
☐ Check to include this hit in error tolerant search or archive report

	Query	Observed	Mr(expt)	Mr(calc)	Delta	Miss	Score	Expect	Rank	Peptide
☑	570	414.76	827.51	827.51	0.00	0	32	0.28	1	R.GLLADLVK.S
☑	1876	881.38	1760.75	1760.88	-0.13	0	59	0.00062	1	R.SSAAATEVVNELNTDLK.Q
☑	2093	1061.07	2120.13	2119.94	0.19	0	95	1.3e-07	1	K.ELEMEQMAAEQEIGDIER.E

---

33. gi|10673 **Mass:** 14649 **Score:** 128 **Queries matched:** 5
unnamed protein product [Trypanosoma cruzi]
☐ Check to include this hit in error tolerant search or archive report

	Query	Observed	Mr(expt)	Mr(calc)	Delta	Miss	Score	Expect	Rank	Peptide
	459	383.22	764.43	764.43	0.01	0	25	1.4	4	-.MQIFVK.T
☑	1066	533.27	1064.53	1064.55	-0.02	0	59	0.00061	1	R.TLADYNIQK.E
☑	1070	534.32	1066.62	1066.61	0.00	0	48	0.0086	1	K.ESTLHLVLR.L
☑	1701	508.62	1522.83	1522.77	0.06	1	23	2.7	1	K.IQDKEGIPPDQQR.L
☑	1867	874.42	1746.83	1746.89	-0.06	0	100	4.9e-08	1	K.TIALEVESSDTIENVK.A

Proteins matching the same set of peptides:
gi|10674 **Mass:** 8763 **Score:** 128 **Queries matched:** 5
unnamed protein product [Trypanosoma cruzi]
gi|136677 **Mass:** 8504 **Score:** 128 **Queries matched:** 5
RecName: Full=Ubiquitin
gi|162337 **Mass:** 40106 **Score:** 128 **Queries matched:** 5
ubiquitin precursor

---

34. gi|71405209 **Mass:** 16814 **Score:** 127 **Queries matched:** 5
calmodulin [Trypanosoma cruzi strain CL Brener]
☐ Check to include this hit in error tolerant search or archive report

	Query	Observed	Mr(expt)	Mr(calc)	Delta	Miss	Score	Expect	Rank	Peptide
	991	514.79	1027.58	1027.51	0.06	0	3	2.3e+02	6	R.HVMTNLGEK.L
☑	1539	675.27	1348.53	1348.62	-0.09	0	87	9.6e-07	1	K.LTDEEVDEMIR.E
☑	740	922.87	1843.73	1843.88	-0.16	1	62	0.00098	1	K.EAFSLFDKDGDGTITTK.E
☑	1944	615.63	1843.88	1843.88	-0.00	1	(32)	0.34	1	K.EAFSLFDKDGDGTITTK.E
☑	1945	615.63	1843.88	1843.88	-0.00	1	(23)	2.4	1	K.EAFSLFDKDGDGTITTK.E

Proteins matching the same set of peptides:
gi|71411702 **Mass:** 23555 **Score:** 127 **Queries matched:** 5
calmodulin [Trypanosoma cruzi strain CL Brener]
gi|71411704 **Mass:** 16824 **Score:** 127 **Queries matched:** 5

calmodulin [Trypanosoma cruzi strain CL Brener]
gi|122063212 **Mass:** 16845 **Score:** 127 **Queries matched:** 5
Calmodulin (CaM)
gi|167534692 **Mass:** 16851 **Score:** 127 **Queries matched:** 5
hypothetical protein [Monosiga brevicollis MX1]

---

35. gi|71409213 **Mass:** 46555 **Score:** 126 **Queries matched:** 2
elongation factor 1-gamma (EF-1-gamma) [Trypanosoma cruzi strain CL Brener]
☐ Check to include this hit in error tolerant search or archive report

	Query	Observed	Mr(expt)	Mr(calc)	Delta	Miss	Score	Expect	Rank	Peptide
☑	1795	822.35	1642.68	1642.71	-0.03	0	126	1.3e-10	1	K.AAAAAADGAEEEDEAPR.E
☑	279	634.31	1899.90	1899.84	0.05	1	20	15	1	K.AAAAAADGAEEEDEAPREK.K

---

36. gi|71407515 **Mass:** 70946 **Score:** 124 **Queries matched:** 4
heat shock 70 kDa protein, mitochondrial precursor [Trypanosoma cruzi strain CL Brener]
☐ Check to include this hit in error tolerant search or archive report

	Query	Observed	Mr(expt)	Mr(calc)	Delta	Miss	Score	Expect	Rank	Peptide
☑	807	475.27	948.52	948.53	-0.00	0	23	2.4	1	R.TTPSVVAFK.G
☑	1064	532.77	1063.52	1063.53	-0.01	0	56	0.0014	1	R.VLENTEGFR.T
☑	1787	816.38	1630.75	1630.86	-0.11	0	96	1.2e-07	1	R.VVNEPTAAALAYGLDK.T
☑	2479	1149.18	3444.53	3444.45	0.07	0	27	0.64	1	R.TEYQQAAAANSSSSSGNTDSSQGEQQQQGDQQK.Q

---

37. gi|71423525 **Mass:** 24104 **Score:** 122 **Queries matched:** 3
vesicle-associated membrane protein [Trypanosoma cruzi strain CL Brener]
☐ Check to include this hit in error tolerant search or archive report

	Query	Observed	Mr(expt)	Mr(calc)	Delta	Miss	Score	Expect	Rank	Peptide
☑	1757	795.97	1589.93	1589.88	0.04	0	61	0.00037	1	R.TVFAFLEAVEPLVR.G
☑	2251	1197.52	2393.03	2393.09	-0.06	0	(57)	0.0008	1	K.ITALNDDINQVVDVMMDNMDK.V
☑	2252	798.73	2393.15	2393.09	0.06	0	57	0.00075	1	K.ITALNDDINQVVDVMMDNMDK.V

---

38. gi|1170958 **Mass:** 22122 **Score:** 122 **Queries matched:** 6
RecName: Full=Macrophage infectivity potentiator; AltName: Full=Peptidyl-prolyl cis-trans isomerase
☐ Check to include this hit in error tolerant search or archive report

	Query	Observed	Mr(expt)	Mr(calc)	Delta	Miss	Score	Expect	Rank	Peptide
☑	961	508.77	1015.54	1015.58	-0.05	0	(37)	0.13	1	K.LPSGLVFQR.I
☑	962	508.78	1015.54	1015.58	-0.04	0	51	0.0051	1	K.LPSGLVFQR.I
☑	963	508.87	1015.72	1015.58	0.14	0	(44)	0.023	1	K.LPSGLVFQR.I
☑	1286	587.80	1173.60	1173.59	0.01	0	63	0.00025	1	R.TAEEVDEILR.K
☑	1339	602.86	1203.71	1203.61	0.10	0	68	8.4e-05	1	K.GWTEALQLMR.E
	1900	599.77	1796.28	1795.99	0.28	1	2	3.9e+02	2	K.AAQPDAVKLPSGLVFQR.I

Proteins matching the same set of peptides:
gi|21465757 **Mass:** 18822 **Score:** 122 **Queries matched:** 6
Chain A, Trypanosoma Cruzi Macrophage Infectivity Potentiator (Tcmip)
gi|71418561 **Mass:** 22140 **Score:** 122 **Queries matched:** 6
macrophage infectivity potentiator, precursor [Trypanosoma cruzi strain CL Brener]

---

39. gi|58177523 **Mass:** 18023 **Score:** 116 **Queries matched:** 3
Chain A, Crystal Structure Of Cyclophilin From Trypanosoma Cruzi
☐ Check to include this hit in error tolerant search or archive report

	Query	Observed	Mr(expt)	Mr(calc)	Delta	Miss	Score	Expect	Rank	Peptide
☑	865	488.26	974.51	974.51	0.00	0	42	0.045	1	K.VVEGMDVVK.K
☑	1430	629.37	1256.73	1256.71	0.02	0	56	0.0011	1	R.VVIGLFGNDVPK.T
☑	1970	953.42	1904.84	1904.84	-0.00	0	76	1e-05	1	R.NFMIQGGDFTNFDGTGGK.S

Proteins matching the same set of peptides:
gi|71654523 **Mass:** 21104 **Score:** 116 **Queries matched:** 3
cyclophilin [Trypanosoma cruzi strain CL Brener]
gi|71655246 **Mass:** 21089 **Score:** 116 **Queries matched:** 3
cyclophilin [Trypanosoma cruzi strain CL Brener]

---

40. gi|71422090 **Mass:** 33476 **Score:** 112 **Queries matched:** 3

electron-transfer-flavoprotein, alpha polypeptide [Trypanosoma cruzi strain CL Brener]

☐ Check to include this hit in error tolerant search or archive report

	Query	Observed	Mr(expt)	Mr(calc)	Delta	Miss	Score	Expect	Rank	Peptide
☑	948	506.33	1010.64	1010.61	0.03	0	87	8.3e-07	1	K.VGPVTALVAGK.D
☑	1215	570.31	1138.60	1138.66	-0.06	0	64	0.00017	1	K.ILNELAEPLK.A
	2137	732.64	2194.89	2195.04	-0.15	1	3	2.8e+02	5	K.ADAMPISDVTEVKDDSTFVR.L

Proteins matching the same set of peptides:

gi|71649252 **Mass:** 33464 **Score:** 112 **Queries matched:** 3

electron-transfer-flavoprotein, alpha polypeptide [Trypanosoma cruzi strain CL Brener]

---

41. gi|71425268 **Mass:** 41776 **Score:** 111 **Queries matched:** 4

protein disulfide isomerase [Trypanosoma cruzi strain CL Brener]

☐ Check to include this hit in error tolerant search or archive report

	Query	Observed	Mr(expt)	Mr(calc)	Delta	Miss	Score	Expect	Rank	Peptide
☑	1396	619.78	1237.55	1237.56	-0.01	0	66	0.00012	1	R.NFDAVVMDEAK.D
☑	1397	619.81	1237.61	1237.65	-0.03	0	77	9.2e-06	1	R.EAAAFLSFLNR.Q
☑	1737	523.90	1568.69	1568.79	-0.10	0	37	0.098	1	K.AAADLTGVEAVHYPR.I
☑	2210	768.08	2301.22	2301.21	0.00	0	11	38	1	R.QVPGLNIGVPHEHTYAVELTK.R

---

42. gi|71406218 **Mass:** 36425 **Score:** 109 **Queries matched:** 2

COP-coated vesicle membrane protein gp25L precursor [Trypanosoma cruzi strain CL Brener]

☐ Check to include this hit in error tolerant search or archive report

	Query	Observed	Mr(expt)	Mr(calc)	Delta	Miss	Score	Expect	Rank	Peptide
☑	1186	562.81	1123.61	1123.67	-0.06	0	41	0.033	1	K.LRPIEVELR.V
☑	1704	763.82	1525.63	1525.69	-0.06	0	102	3e-08	1	R.VGSENNDYSELATK.E

Proteins matching the same set of peptides:

gi|71652059 **Mass:** 24859 **Score:** 109 **Queries matched:** 2

COP-coated vesicle membrane protein gp25L precursor [Trypanosoma cruzi strain CL Brener]

---

43. gi|84105385 **Mass:** 68015 **Score:** 108 **Queries matched:** 3

cytosolic heat shock protein 70 [Malawimonas jakobiformis]

☐ Check to include this hit in error tolerant search or archive report

Query	Observed	Mr(expt)	Mr(calc)	Delta	Miss	Score	Expect	Rank	Peptide
1487	650.37	1298.72	1298.60	0.12	0	24	1.9	2	R.FEELCIDQFR.K
1672	744.34	1486.67	1486.69	-0.03	0	68	7.3e-05	1	R.TTPSYVAFTDTER.L
1808	830.46	1658.90	1658.89	0.01	0	74	1.8e-05	1	R.IINEPTAAAIAYGLDK.K

---

44. gi|71665461 **Mass:** 46415 **Score:** 106 **Queries matched:** 5

enolase [Trypanosoma cruzi strain CL Brener]

☐ Check to include this hit in error tolerant search or archive report

	Query	Observed	Mr(expt)	Mr(calc)	Delta	Miss	Score	Expect	Rank	Peptide
☑	837	**483.27**	**964.53**	**964.52**	**0.01**	**0**	**25**	**1.5**	**1**	**R.YLAELAGTK.E**
☑	1118	**545.27**	**1088.54**	**1088.55**	**-0.01**	**0**	**37**	**0.11**	**1**	**K.SPEATWVTAK.Q**
☑	1504	**655.38**	**1308.75**	**1308.74**	**0.01**	**0**	**47**	**0.011**	**1**	**K.NVNDVLAPALVGK.D**
☑	1745	**787.37**	**1572.73**	**1572.81**	**-0.08**	**0**	**97**	**1.3e-07**	**1**	**K.AQVVGDDLTVTNVSR.I**
☑	1943	**614.63**	**1840.87**	**1840.87**	**-0.01**	**0**	**23**	**2.3**	**1**	**R.SAVPSGASTGIHEACELR.D + Carbamidomethyl (C)**

---

45. gi|12007351 **Mass:** 51105 **Score:** 106 **Queries matched:** 3

heat shock protein Hsp70 [Monosiga ovata]

☐ Check to include this hit in error tolerant search or archive report

Query	Observed	Mr(expt)	Mr(calc)	Delta	Miss	Score	Expect	Rank	Peptide
1487	650.37	1298.72	1298.60	0.12	0	24	1.9	2	R.FEELCADLFR.G + Carbamidomethyl (C)
1666	741.79	1481.56	1481.78	-0.22	0	60	0.0005	1	K.SDIHEIVLVGGSTR.I
1808	830.46	1658.90	1658.89	0.01	0	74	1.8e-05	1	R.IINEPTAAAIAYGLDK.K

---

46. gi|123592 **Mass:** 56500 **Score:** 105 **Queries matched:** 3

RecName: Full=Heat shock 70 kDa protein

☐ Check to include this hit in error tolerant search or archive report

Query	Observed	Mr(expt)	Mr(calc)	Delta	Miss	Score	Expect	Rank	Peptide
1379	614.82	1227.62	1227.62	0.00	0	66	0.00013	1	R.LDIIANDQGNR.T
1665	494.61	1480.81	1480.81	0.00	1	2	3.6e+02	5	K.RSVHDVVLVGGSTR.I
1808	830.46	1658.90	1658.89	0.01	0	74	1.8e-05	1	R.IINEPTAAAIAYGLDK.G

47. gi|71404821 **Mass:** 88843 **Score:** 102 **Queries matched:** 2
trans-sialidase [Trypanosoma cruzi strain CL Brener]
Check to include this hit in error tolerant search or archive report

Query	Observed	Mr(expt)	Mr(calc)	Delta	Miss	Score	Expect	Rank	Peptide
2000	**984.93**	**1967.85**	**1967.86**	**-0.00**	**0**	**102**	**2.7e-08**	**1**	**K.ATGSSAGEDSESSGAAGTDLAK.G**
2001	**656.97**	**1967.87**	**1967.86**	**0.02**	**0**	**(21)**	**4.1**	**1**	**K.ATGSSAGEDSESSGAAGTDLAK.G**

Proteins matching the same set of peptides:
gi|71393748 **Mass:** 37829 **Score:** 102 **Queries matched:** 2
trans-sialidase [Trypanosoma cruzi strain CL Brener]

48. gi|71416386 **Mass:** 133169 **Score:** 102 **Queries matched:** 3
hypothetical protein [Trypanosoma cruzi strain CL Brener]
Check to include this hit in error tolerant search or archive report

Query	Observed	Mr(expt)	Mr(calc)	Delta	Miss	Score	Expect	Rank	Peptide
1461	**640.34**	**1278.66**	**1278.63**	**0.04**	**0**	**(68)**	**7.5e-05**	**1**	**R.IMQLDIFDER.A**
1462	**640.38**	**1278.74**	**1278.63**	**0.11**	**0**	**69**	**6.6e-05**	**1**	**R.IMQLDIFDER.A**
1549	**453.58**	**1357.71**	**1357.70**	**0.01**	**0**	**34**	**0.18**	**1**	**K.SPSRPEQFSVPK.V**

49. gi|123603 **Score:** 102 **Queries matched:** 3
RecName: Full=Heat shock 70 kDa protein
Check to include this hit in error tolerant search or archive report

Query	Observed	Mr(expt)	Mr(calc)	Delta	Miss	Score	Expect	Rank	Peptide
1379	614.82	1227.62	1227.62	0.00	0	63	0.00029	2	R.VEIIANDQGNR.T
1487	650.37	1298.72	1298.60	0.12	0	24	1.9	2	R.FEELCGELFR.G + Carbamidomethyl (C)
1808	830.46	1658.90	1658.89	0.01	0	74	1.8e-05	1	R.IINEPTAAAIAYGLDK.V

50. gi|58042864 **Mass:** 72742 **Score:** 102 **Queries matched:** 3

PPAT5 [Hyaloperonospora parasitica]

☐ Check to include this hit in error tolerant search or archive report

Query	Observed	Mr(expt)	Mr(calc)	Delta	Miss	Score	Expect	Rank	Peptide
1078	**536.29**	**1070.58**	**1070.59**	**-0.02**	**1**	**15**	**17**	**3**	**K.RALSAQAQAR.L**
1379	614.82	1227.62	1227.62	0.00	0	63	0.00029	2	K.VEIIANDQGNR.I
1808	830.46	1658.90	1658.89	0.01	0	74	1.8e-05	1	R.IINEPTAAAIAYGIDK.K

Proteins matching the same set of peptides:

gi|58042866 **Mass:** 72801 **Score:** 102 **Queries matched:** 3

PPAT5 [Hyaloperonospora parasitica]

gi|429118 **Mass:** 72636 **Score:** 102 **Queries matched:** 3

glucose regulated protein /BiP [Phytophthora cinnamomi]

---

51. gi|57903381 **Score:** 102 **Queries matched:** 3

heat shock protein 70 [Spumella uniguttata]

☐ Check to include this hit in error tolerant search or archive report

Query	Observed	Mr(expt)	Mr(calc)	Delta	Miss	Score	Expect	Rank	Peptide
1379	614.82	1227.62	1227.62	0.00	0	63	0.00029	2	R.VEIIANDQGNR.T
1665	494.61	1480.81	1480.80	0.01	0	2	4.1e+02	6	K.SQVHEIVLVGGSTR.I
1808	830.46	1658.90	1658.89	0.01	0	74	1.8e-05	1	R.IINEPTAAAIAYGLDK.K

---

52. gi|71410853 **Mass:** 10694 **Score:** 96 **Queries matched:** 4

10 kDa heat shock protein [Trypanosoma cruzi strain CL Brener]

☐ Check to include this hit in error tolerant search or archive report

	Query	Observed	Mr(expt)	Mr(calc)	Delta	Miss	Score	Expect	Rank	Peptide
☑	592	**846.42**	**845.42**	**845.43**	**-0.01**	**0**	**9**	**83**	**1**	**K.DWTPTVK.V**
☑	1521	**665.36**	**1328.71**	**1328.73**	**-0.02**	**0**	**93**	**2.5e-07**	**1**	**K.VNEGTVVAVAAATK.D**
☑	1813	**554.94**	**1661.80**	**1661.85**	**-0.05**	**0**	**(35)**	**0.14**	**1**	**K.VDDTVLLPEFGGSSVK.V**
☑	1814	**831.94**	**1661.86**	**1661.85**	**0.01**	**0**	**37**	**0.1**	**1**	**K.VDDTVLLPEFGGSSVK.V**

Proteins matching the same set of peptides:

gi|71410857 **Mass:** 20085 **Score:** 96 **Queries matched:** 4
10 kDa heat shock protein [Trypanosoma cruzi strain CL Brener]

---

53. gi|85822985 **Mass:** 28912 **Score:** 93 **Queries matched:** 3
elongation factor 1-alpha [Thraustochytriidae sp. P19]

☐ Check to include this hit in error tolerant search or archive report

Query	Observed	Mr(expt)	Mr(calc)	Delta	Miss	Score	Expect	Rank	Peptide
867	488.28	974.55	974.54	0.01	0	63	0.00038	1	R.LPLQDVYK.I
985	513.31	1024.61	1024.60	0.01	0	71	3.7e-05	1	K.IGGIGTVPVGR.V
2298	841.69	2522.05	2522.15	-0.10	0	26	1	2	K.SVEMHHESVPEAQPGDNVGFNVK.N + Oxidation (M)

---

54. gi|89329735 **Mass:** 50628 **Score:** 93 **Queries matched:** 3
translation elongation factor 1-alpha [Capsaspora owczarzaki]

☐ Check to include this hit in error tolerant search or archive report

Query	Observed	Mr(expt)	Mr(calc)	Delta	Miss	Score	Expect	Rank	Peptide
867	488.28	974.55	974.54	0.01	0	63	0.00038	1	R.LPLQDVYK.I
985	513.31	1024.61	1024.60	0.01	0	71	3.7e-05	1	K.IGGIGTVPVGR.V
2298	841.69	2522.05	2522.15	-0.10	0	26	1	2	K.SVEMHHESLPEANPGDNVGFNVK.N + Oxidation (M)

---

55. gi|156987872 **Mass:** 33243 **Score:** 93 **Queries matched:** 3
translation elongation factor 1-alpha [Phytophthora quercina]

☐ Check to include this hit in error tolerant search or archive report

Query	Observed	Mr(expt)	Mr(calc)	Delta	Miss	Score	Expect	Rank	Peptide
867	488.28	974.55	974.54	0.01	0	63	0.00038	1	R.LPLQDVYK.I
948	506.33	1010.64	1010.57	0.07	1	12	27	7	K.RPSDKPRR.L
985	513.31	1024.61	1024.60	0.01	0	71	3.7e-05	1	K.IGGIGTVPVGR.V

---

56. gi|50660750 **Mass:** 24233 **Score:** 93 **Queries matched:** 3

translation elongation factor 1 alpha [Phytophthora hibernalis]
☐ Check to include this hit in error tolerant search or archive report

	Query	Observed	Mr(expt)	Mr(calc)	Delta	Miss	Score	Expect	Rank	Peptide
	867	488.28	974.55	974.54	0.01	0	63	0.00038	1	R.LPLQDVYK.I
	985	513.31	1024.61	1024.60	0.01	0	71	3.7e-05	1	K.IGGIGTVPVGR.V
☑	2204	**765.97**	**2294.88**	**2295.02**	**-0.14**	**0**	**8**	**91**	**1**	**K.SVEMHHESVPEAKPGDNVGFN.- + Oxidation (M)**

---

57. gi\|59859762 **Mass:** 31104 **Score:** 93 **Queries matched:** 3
elongation factor 1 alpha [Phaeodactylum tricornutum]
☐ Check to include this hit in error tolerant search or archive report

Query	Observed	Mr(expt)	Mr(calc)	Delta	Miss	Score	Expect	Rank	Peptide
867	488.28	974.55	974.54	0.01	0	63	0.00038	1	R.LPLQDVYK.I
985	513.31	1024.61	1024.60	0.01	0	71	3.7e-05	1	K.IGGIGTVPVGR.V
1971	**954.43**	**1906.84**	**1906.96**	**-0.11**	**2**	**4**	**1.9e+02**	**5**	**K.STTTGHLIYKCGDIDKR.T**

---

58. gi\|156987628 **Mass:** 34948 **Score:** 93 **Queries matched:** 3
translation elongation factor 1-alpha [Phytophthora brassicae]
☐ Check to include this hit in error tolerant search or archive report

	Query	Observed	Mr(expt)	Mr(calc)	Delta	Miss	Score	Expect	Rank	Peptide
	867	488.28	974.55	974.54	0.01	0	63	0.00038	1	R.LPLQDVYK.I
	985	513.31	1024.61	1024.60	0.01	0	71	3.7e-05	1	K.IGGIGTVPVGR.V
☑	677	**889.75**	**2666.22**	**2666.30**	**-0.08**	**1**	**1**	**1.4e+03**	**1**	**K.SSNMPWYKGPYLLENLDTLNAPK.R + Oxidation (M)**

---

59. gi\|56156697 **Mass:** 44176 **Score:** 93 **Queries matched:** 3
elongation factor 1A [Trichia persimilis]
☐ Check to include this hit in error tolerant search or archive report

Query	Observed	Mr(expt)	Mr(calc)	Delta	Miss	Score	Expect	Rank	Peptide
867	488.28	974.55	974.54	0.01	0	63	0.00038	1	R.IPLQDVYK.I
985	513.31	1024.61	1024.60	0.01	0	71	3.7e-05	1	K.IGGIGTVPVGR.V

1904	600.28	1797.82	1797.93	-0.11	2	1	3.8e+02	4	-.HTTGHLIYKCGGIDKR.T

60. gi|1706590 **Mass:** 56612 **Score:** 93 **Queries matched:** 3
Elongation factor 1-alpha S (EF-1-alpha S) (Sporophyte-specific EF-1-alpha)

☐ Check to include this hit in error tolerant search or archive report

Query	Observed	Mr(expt)	Mr(calc)	Delta	Miss	Score	Expect	Rank	Peptide
867	488.28	974.55	974.54	0.01	0	63	0.00038	1	R.LPLQDVYK.I
985	513.31	1024.61	1024.60	0.01	0	71	3.7e-05	1	K.IGGIGTVPVGR.V
2028	**1007.97**	**2013.92**	**2014.08**	**-0.17**	**2**	**1**	**3.6e+02**	**4**	**K.KTVAVGVIQCVQPRNMAK.G + Carbamidomethyl (C); Oxidation (M)**

61. gi|50660760 **Mass:** 25387 **Score:** 93 **Queries matched:** 3
translation elongation factor 1 alpha [Phytophthora insolita]

☐ Check to include this hit in error tolerant search or archive report

Query	Observed	Mr(expt)	Mr(calc)	Delta	Miss	Score	Expect	Rank	Peptide
867	488.28	974.55	974.54	0.01	0	63	0.00038	1	R.LPLQDVYK.I
985	513.31	1024.61	1024.60	0.01	0	71	3.7e-05	1	K.IGGIGTVPVGR.V
1536	**1348.58**	**4042.71**	**4042.89**	**-0.18**	**2**	**0**	**1.1e+03**	**4**	**R.DFIKNMITGTSQADCAQLVVASGVGECEAGICKEGQTR.E + 2 Carbamidomethyl (C)**

62. gi|71425263 **Mass:** 10693 **Score:** 93 **Queries matched:** 3
10 kDa heat shock protein [Trypanosoma cruzi strain CL Brener]

☐ Check to include this hit in error tolerant search or archive report

	Query	Observed	Mr(expt)	Mr(calc)	Delta	Miss	Score	Expect	Rank	Peptide
	592	846.42	845.42	845.43	-0.01	0	9	83	1	K.DWTPTVK.V
	1521	665.36	1328.71	1328.73	-0.02	0	93	2.5e-07	1	K.VNEGTVVAVAAATK.D
☑	1812	**831.45**	**1660.88**	**1660.87**	**0.02**	**0**	**38**	**0.072**	**1**	**K.VNDTVLLPEFGGSSVK.V**

63. gi|1314208 **Mass:** 46927 **Score:** 91 **Queries matched:** 2
alpha-tubulin

☐ Check to include this hit in error tolerant search or archive report

Query	Observed	Mr(expt)	Mr(calc)	Delta	Miss	Score	Expect	Rank	Peptide
☑ 1854	858.48	1714.94	1714.91	0.02	0	87	9e-07	1	R.AVFLDLEPTVVDEIR.T
☑ 1855	572.66	1714.95	1714.91	0.04	0	(43)	0.021	1	R.AVFLDLEPTVVDEIR.T

Proteins matching the same set of peptides:

gi|3915082 **Mass:** 49696 **Score:** 91 **Queries matched:** 2
Tubulin alpha chain

gi|71397525 **Mass:** 49768 **Score:** 91 **Queries matched:** 2
alpha tubulin [Trypanosoma cruzi strain CL Brener]

gi|74229924 **Mass:** 49766 **Score:** 91 **Queries matched:** 2
alpha-tubulin [Trypanosoma danilewskyi]

gi|91983193 **Mass:** 38117 **Score:** 91 **Queries matched:** 2
alpha tubulin [Parabodo caudatus]

gi|91983196 **Mass:** 49777 **Score:** 91 **Queries matched:** 2
alpha tubulin [Trypanosoma cyclops]

gi|91983200 **Mass:** 49749 **Score:** 91 **Queries matched:** 2
alpha tubulin [Trypanosoma grayi]

---

64. gi|71425751 **Mass:** 16513 **Score:** 91 **Queries matched:** 3
centrin [Trypanosoma cruzi strain CL Brener]

☐ Check to include this hit in error tolerant search or archive report

Query	Observed	Mr(expt)	Mr(calc)	Delta	Miss	Score	Expect	Rank	Peptide
☑ 767	466.20	930.39	930.49	-0.10	0	29	0.75	1	K.GLGFGDLPR.D
☑ 2297	839.39	2515.14	2515.12	0.01	0	40	0.046	1	R.EAFNLFDADGSGAIDAEEMALAMK.G
☑ 2481	1161.85	3482.51	3482.52	-0.01	0	70	3.6e-05	1	K.LLGENPGDDVLQEMIAEADEDGDGEVSFDEFK.N

---

65. gi|71414147 **Mass:** 12226 **Score:** 88 **Queries matched:** 2
cytochrome c [Trypanosoma cruzi strain CL Brener]

☐ Check to include this hit in error tolerant search or archive report

Query	Observed	Mr(expt)	Mr(calc)	Delta	Miss	Score	Expect	Rank	Peptide
☑ 1465	641.79	1281.58	1281.60	-0.02	0	76	1.2e-05	1	R.HSGTVEGFAYSK.A

☑ 1525	667.91	1333.81	1333.72	0.09	1	43	0.029	1	R.ADLIAYLATLRD.-

---

66. gi|71408270 **Mass:** 17813 **Score:** 88 **Queries matched:** 1
calmodulin [Trypanosoma cruzi strain CL Brener]
☐ Check to include this hit in error tolerant search or archive report

Query	Observed	Mr(expt)	Mr(calc)	Delta	Miss	Score	Expect	Rank	Peptide
☑ 1694	758.99	1515.97	1515.88	0.09	0	88	9.1e-07	1	K.LSELITLLTSLGEK.M

Proteins matching the same set of peptides:
gi|71744728 **Mass:** 17831 **Score:** 88 **Queries matched:** 1
calmodulin [Trypanosoma brucei TREU927]

---

67. gi|71664019 **Mass:** 51642 **Score:** 88 **Queries matched:** 4
hypothetical protein [Trypanosoma cruzi strain CL Brener]
☐ Check to include this hit in error tolerant search or archive report

Query	Observed	Mr(expt)	Mr(calc)	Delta	Miss	Score	Expect	Rank	Peptide
☑ 951	507.28	1012.55	1012.59	-0.04	0	52	0.0029	1	K.ELELAVALR.Q
☑ 1034	524.76	1047.50	1047.49	0.01	0	49	0.007	1	R.YSDLFFEK.H
☑ 1735	523.29	1566.84	1566.76	0.08	0	24	1.7	1	R.NQALMHEVNELNR.R
☑ 1797	823.31	1644.61	1644.76	-0.15	0	57	0.0012	1	R.QNETDVTPQELESR.K

---

68. gi|71658999 **Mass:** 100785 **Score:** 86 **Queries matched:** 3
pyruvate phosphate dikinase [Trypanosoma cruzi strain CL Brener]
☐ Check to include this hit in error tolerant search or archive report

Query	Observed	Mr(expt)	Mr(calc)	Delta	Miss	Score	Expect	Rank	Peptide
☑ 1360	609.30	1216.59	1216.60	-0.02	0	57	0.0011	1	K.TAEETLAAAGQR.V
☑ 1502	654.91	1307.81	1307.79	0.02	0	62	0.00029	1	K.VIPEIMIPLVGK.K
2249	797.42	2389.23	2389.06	0.17	1	6	1.1e+02	5	R.SFGAEGVGLCRTEHMFFEGSR.I + Carbamidomethyl (C); Oxidation (M)

---

69. gi|71413591 **Mass:** 16740 **Score:** 85 **Queries matched:** 2
hypothetical protein [Trypanosoma cruzi strain CL Brener]
☐ Check to include this hit in error tolerant search or archive report

	Query	Observed	Mr(expt)	Mr(calc)	Delta	Miss	Score	Expect	Rank	Peptide
	44	430.50	858.98	859.51	-0.53	0	12	98	9	R.LSLSLATR.E
☑	1667	741.85	1481.69	1481.75	-0.05	0	85	1.3e-06	1	K.TETVSSTETVVTTK.N

---

70. gi|19171192 **Mass:** 33181 **Score:** 85 **Queries matched:** 1
phosphoenolpyruvate mutase [Trypanosoma cruzi]
☐ Check to include this hit in error tolerant search or archive report

	Query	Observed	Mr(expt)	Mr(calc)	Delta	Miss	Score	Expect	Rank	Peptide
☑	1859	862.93	1723.84	1723.84	0.01	0	85	1.4e-06	1	R.LPEYLEAEEMYLPK.K

Proteins matching the same set of peptides:
gi|71654808 **Mass:** 33165 **Score:** 85 **Queries matched:** 1
phosphoenolpyruvate mutase [Trypanosoma cruzi strain CL Brener]
gi|71651621 **Mass:** 33145 **Score:** 85 **Queries matched:** 1
phosphoenolpyruvate mutase [Trypanosoma cruzi strain CL Brener]

---

71. gi|71414193 **Mass:** 24694 **Score:** 85 **Queries matched:** 1
adenylate kinase [Trypanosoma cruzi strain CL Brener]
☐ Check to include this hit in error tolerant search or archive report

	Query	Observed	Mr(expt)	Mr(calc)	Delta	Miss	Score	Expect	Rank	Peptide
☑	1717	771.94	1541.87	1541.85	0.02	0	85	1.6e-06	1	K.VILFDAPDDVIVAR.T

Proteins matching the same set of peptides:
gi|71664239 **Mass:** 24423 **Score:** 85 **Queries matched:** 1
adenylate kinase [Trypanosoma cruzi strain CL Brener]

---

72. gi|28195111 **Mass:** 56703 **Score:** 84 **Queries matched:** 2
protein kinase A regulatory subunit [Trypanosoma cruzi]

☐ Check to include this hit in error tolerant search or archive report

	Query	Observed	Mr(expt)	Mr(calc)	Delta	Miss	Score	Expect	Rank	Peptide
☑	1304	592.35	1182.69	1182.72	-0.04	0	90	4.5e-07	1	R.GILAVLDLIEK.L
☑	2233	785.99	2354.94	2355.11	-0.17	0	24	1.9	1	R.SNPSPSQIEEIDLSHNYVGNR.G

Proteins matching the same set of peptides:
gi|71657902 **Mass:** 56717 **Score:** 84 **Queries matched:** 2
protein kinase A regulatory subunit [Trypanosoma cruzi strain CL Brener]

---

73. gi|71666956 **Mass:** 40800 **Score:** 84 **Queries matched:** 1
hypothetical protein [Trypanosoma cruzi strain CL Brener]
☐ Check to include this hit in error tolerant search or archive report

	Query	Observed	Mr(expt)	Mr(calc)	Delta	Miss	Score	Expect	Rank	Peptide
☑	1618	718.87	1435.73	1435.73	-0.00	0	84	1.9e-06	1	K.IISVADQYVESGR.K

---

74. gi|71651382 **Mass:** 12177 **Score:** 83 **Queries matched:** 1
thioredoxin [Trypanosoma cruzi strain CL Brener]
☐ Check to include this hit in error tolerant search or archive report

	Query	Observed	Mr(expt)	Mr(calc)	Delta	Miss	Score	Expect	Rank	Peptide
☑	1622	719.40	1436.78	1436.73	0.05	0	83	2.3e-06	1	M.PVVDVYSVEQFR.E

Proteins matching the same set of peptides:
gi|71653602 **Mass:** 12191 **Score:** 83 **Queries matched:** 1
thioredoxin [Trypanosoma cruzi strain CL Brener]

---

75. gi|71654654 **Mass:** 21232 **Score:** 83 **Queries matched:** 2
kinetoplast DNA-associated protein [Trypanosoma cruzi strain CL Brener]
☐ Check to include this hit in error tolerant search or archive report

Query	Observed	Mr(expt)	Mr(calc)	Delta	Miss	Score	Expect	Rank	Peptide
807	475.27	948.52	948.54	-0.01	2	15	17	3	K.TTSASKRAK.S

2023	670.36	2008.07	2008.05	0.02	0	83	2.3e-06	1	R.FGALSQLYDVSKPLDVEK.E

---

76. gi|71650885 **Mass:** 46722 **Score:** 82 **Queries matched:** 3
ATP-dependent DEAD/H RNA helicase [Trypanosoma cruzi strain CL Brener]
Check to include this hit in error tolerant search or archive report

Query	Observed	Mr(expt)	Mr(calc)	Delta	Miss	Score	Expect	Rank	Peptide
1134	547.79	1093.56	1093.55	0.01	0	52	0.0031	1	R.ELQMGIFEK.G
1337	602.40	1202.79	1202.69	0.10	0	64	0.00021	1	K.TASFVIPVLEK.V
2113	718.03	2151.07	2151.13	-0.06	0	34	0.22	1	K.GFERPSPVQEEAIPVALQGK.D

Proteins matching the same set of peptides:
gi|71747184 **Mass:** 46439 **Score:** 82 **Queries matched:** 3
ATP-dependent DEAD-box RNA helicase [Trypanosoma brucei TREU927]

---

77. gi|71414729 **Mass:** 20383 **Score:** 81 **Queries matched:** 1
ras-related protein rab-5 [Trypanosoma cruzi strain CL Brener]
Check to include this hit in error tolerant search or archive report

Query	Observed	Mr(expt)	Mr(calc)	Delta	Miss	Score	Expect	Rank	Peptide
1104	543.27	1084.52	1084.65	-0.13	0	81	3.7e-06	1	K.IVLLGESAVGK.S

Proteins matching the same set of peptides:
gi|71665955 **Mass:** 24024 **Score:** 81 **Queries matched:** 1
ras-related protein rab-5 [Trypanosoma cruzi strain CL Brener]

---

78. gi|167538218 **Score:** 81 **Queries matched:** 1
hypothetical protein [Monosiga brevicollis MX1]
Check to include this hit in error tolerant search or archive report

Query	Observed	Mr(expt)	Mr(calc)	Delta	Miss	Score	Expect	Rank	Peptide
1104	543.27	1084.52	1084.65	-0.13	0	81	3.7e-06	1	K.LVLLGESAVGK.S

---

79. gi|71403985 **Score:** 81 **Queries matched:** 1
t-complex protein 1, delta subunit [Trypanosoma cruzi strain CL Brener]
☐ Check to include this hit in error tolerant search or archive report

	Query	Observed	Mr(expt)	Mr(calc)	Delta	Miss	Score	Expect	Rank	Peptide
☑	1693	**758.91**	**1515.81**	**1515.81**	**-0.01**	**0**	**81**	**5.3e-06**	**1**	**R.GEVLISNDGATILSK.L**

Proteins matching the same set of peptides:
gi|71661493 **Score:** 81 **Queries matched:** 1

---

80. gi|71746618 **Mass:** 58325 **Score:** 81 **Queries matched:** 1
t-complex protein 1 subunit delta [Trypanosoma brucei TREU927]
☐ Check to include this hit in error tolerant search or archive report

Query	Observed	Mr(expt)	Mr(calc)	Delta	Miss	Score	Expect	Rank	Peptide
1693	758.91	1515.81	1515.81	-0.01	0	81	5.3e-06	1	R.GEVIISNDGATILSK.L

---

81. gi|71659870 **Mass:** 39391 **Score:** 80 **Queries matched:** 1
hypothetical protein [Trypanosoma cruzi strain CL Brener]
☐ Check to include this hit in error tolerant search or archive report

	Query	Observed	Mr(expt)	Mr(calc)	Delta	Miss	Score	Expect	Rank	Peptide
☑	394	**717.66**	**1433.32**	**1433.70**	**-0.39**	**0**	**80**	**1.7e-05**	**1**	**R.TETAPYIETPAGGK.S**

Proteins matching the same set of peptides:
gi|71659884 **Mass:** 39256 **Score:** 80 **Queries matched:** 1
hypothetical protein [Trypanosoma cruzi strain CL Brener]

---

82. gi|167539657 **Mass:** 13019 **Score:** 80 **Queries matched:** 1
hypothetical protein [Monosiga brevicollis MX1]
☐ Check to include this hit in error tolerant search or archive report

	Query	Observed	Mr(expt)	Mr(calc)	Delta	Miss	Score	Expect	Rank	Peptide
☑	1494	**651.82**	**1301.62**	**1301.64**	**-0.02**	**0**	**80**	**4.9e-06**	**1**	**R.LNIWDTAGQER.F**

83. gi|71662790 **Mass:** 107780 **Score:** 79 **Queries matched:** 1
trans-sialidase [Trypanosoma cruzi strain CL Brener]
☐ Check to include this hit in error tolerant search or archive report

	Query	Observed	Mr(expt)	Mr(calc)	Delta	Miss	Score	Expect	Rank	Peptide
☑	2008	**991.92**	**1981.83**	**1981.83**	**-0.01**	**0**	**79**	**6.8e-06**	**1**	**K.AIGSSAGEDGESSGAADTDSAK.V**

84. gi|3004649 **Mass:** 23911 **Score:** 77 **Queries matched:** 3
putative GTP-binding protein RAB11 [Trypanosoma cruzi]
☐ Check to include this hit in error tolerant search or archive report

	Query	Observed	Mr(expt)	Mr(calc)	Delta	Miss	Score	Expect	Rank	Peptide
	1026	**522.31**	**1042.60**	**1042.60**	**0.00**	**0**	**(10)**	**55**	**4**	**K.VVIIGDSGVGK.S**
☑	1027	**522.31**	**1042.61**	**1042.60**	**0.01**	**0**	**38**	**0.095**	**1**	**K.VVIIGDSGVGK.S**
	1494	651.82	1301.62	1301.64	-0.02	0	77	1.1e-05	2	K.VQIWDTAGQER.F

Proteins matching the same set of peptides:
gi|71410719 **Mass:** 23824 **Score:** 77 **Queries matched:** 3
small GTP-binding protein Rab11 [Trypanosoma cruzi strain CL Brener]
gi|71417072 **Mass:** 23793 **Score:** 77 **Queries matched:** 3
small GTP-binding protein Rab11 [Trypanosoma cruzi strain CL Brener]

85. gi|71659614 **Mass:** 23329 **Score:** 76 **Queries matched:** 1
hypothetical protein [Trypanosoma cruzi strain CL Brener]
☐ Check to include this hit in error tolerant search or archive report

	Query	Observed	Mr(expt)	Mr(calc)	Delta	Miss	Score	Expect	Rank	Peptide
☑	1609	**713.39**	**1424.77**	**1424.78**	**-0.01**	**0**	**76**	**1.2e-05**	**1**	**R.VVQPVLLENMQR.K**

Proteins matching the same set of peptides:
gi|71663777 **Mass:** 23275 **Score:** 76 **Queries matched:** 1
hypothetical protein [Trypanosoma cruzi strain CL Brener]

86. gi|84105387 **Score:** 74 **Queries matched:** 4
cytosolic heat shock protein 70 [Reclinomonas americana]
☐ Check to include this hit in error tolerant search or archive report

Query	Observed	Mr(expt)	Mr(calc)	Delta	Miss	Score	Expect	Rank	Peptide
1487	650.37	1298.72	1298.60	0.12	0	24	1.9	2	R.FEELCADLFR.K + Carbamidomethyl (C)
1808	830.46	1658.90	1658.89	0.01	0	74	1.8e-05	1	R.IINEPTAAAIAYGLDK.T
1828	564.89	1691.66	1691.90	-0.24	1	(0)	5e+02	8	K.KAVITVPAYFNDAQR.T
1829	846.88	1691.75	1691.90	-0.15	1	11	39	3	K.KAVITVPAYFNDAQR.T

87. gi|168830545 **Score:** 74 **Queries matched:** 3
cytosolic heat shock protein 70 [Andalucia godoyi]
☐ Check to include this hit in error tolerant search or archive report

Query	Observed	Mr(expt)	Mr(calc)	Delta	Miss	Score	Expect	Rank	Peptide
564	**824.87**	**823.86**	**823.43**	**0.43**	**0**	**4**	**6.5e+02**	**2**	**R.DNHLLGR.F**
1665	494.61	1480.81	1480.80	0.01	0	2	4.1e+02	6	K.SQIHDIVLVGGSTR.I
1808	830.46	1658.90	1658.89	0.01	0	74	1.8e-05	1	R.IINEPTAAAIAYGLDK.K

88. gi|468014 **Score:** 74 **Queries matched:** 2
cytoplasmic 70 kDa heat shock protein
☐ Check to include this hit in error tolerant search or archive report

Query	Observed	Mr(expt)	Mr(calc)	Delta	Miss	Score	Expect	Rank	Peptide
1665	494.61	1480.81	1480.76	0.05	0	2	4.1e+02	6	K.NDVHDIVLVGGSTR.I
1808	830.46	1658.90	1658.89	0.01	0	74	1.8e-05	1	R.IINEPTAAAIAYGLDK.S

Proteins matching the same set of peptides:
gi|159115494 **Score:** 74 **Queries matched:** 2

89. gi|88770694 **Score:** 74 **Queries matched:** 2
70 kDa heat shock protein [Rhodomonas salina]
☐ Check to include this hit in error tolerant search or archive report

Query	Observed	Mr(expt)	Mr(calc)	Delta	Miss	Score	Expect	Rank	Peptide
1808	830.46	1658.90	1658.89	0.01	0	74	1.8e-05	1	R.IINEPTAAAIAYGLDK.K
304	**649.41**	**1945.21**	**1944.97**	**0.23**	**1**	**0**	**1.5e+03**	**2**	**R.FQDPAVQEDIKHFPFK.V**

---

90. gi|194476867 **Score:** 74 **Queries matched:** 1
molecular chaperone DnaK [Paulinella chromatophora]
☐ Check to include this hit in error tolerant search or archive report

Query	Observed	Mr(expt)	Mr(calc)	Delta	Miss	Score	Expect	Rank	Peptide
1808	830.46	1658.90	1658.89	0.01	0	74	1.8e-05	1	R.IINEPTAAALAYGLDK.K

---

91. gi|409155 **Mass:** 23776 **Score:** 74 **Queries matched:** 1
ADG1
☐ Check to include this hit in error tolerant search or archive report

	Query	Observed	Mr(expt)	Mr(calc)	Delta	Miss	Score	Expect	Rank	Peptide
☑	1579	**693.34**	**1384.66**	**1384.74**	**-0.08**	**1**	**74**	**2e-05**	**1**	**R.RLELQNVEQTR.S**

Proteins matching the same set of peptides:
gi|71404794 **Mass:** 13762 **Score:** 74 **Queries matched:** 1
ADG1 [Trypanosoma cruzi strain CL Brener]
gi|71651101 **Mass:** 13750 **Score:** 74 **Queries matched:** 1
ADG1-like protein [Trypanosoma cruzi strain CL Brener]
gi|154332541 **Mass:** 12988 **Score:** 74 **Queries matched:** 1
hypothetical protein [Leishmania braziliensis MHOM/BR/75/M2904]
gi|157864691 **Mass:** 16887 **Score:** 74 **Queries matched:** 1
hypothetical protein [Leishmania major strain Friedlin]

---

92. gi|71409831 **Mass:** 11459 **Score:** 74 **Queries matched:** 1
basic transcription factor 3a [Trypanosoma cruzi strain CL Brener]
☐ Check to include this hit in error tolerant search or archive report

Query	Observed	Mr(expt)	Mr(calc)	Delta	Miss	Score	Expect	Rank	Peptide

	Query	Observed	Mr(expt)	Mr(calc)	Delta	Miss	Score	Expect	Rank	Peptide
☑	1076	**536.27**	**1070.53**	**1070.57**	**-0.05**	**0**	**74**	**2.3e-05**	**1**	**M.PNITQETLR.K**

---

93. gi|71663174 **Mass:** 45236 **Score:** 73 **Queries matched:** 1
succinyl-CoA ligase [GDP-forming] beta-chain [Trypanosoma cruzi]
☐ Check to include this hit in error tolerant search or archive report

	Query	Observed	Mr(expt)	Mr(calc)	Delta	Miss	Score	Expect	Rank	Peptide
☑	1256	**580.33**	**1158.65**	**1158.61**	**0.03**	**0**	**73**	**2.6e-05**	**1**	**R.TLEEVEAALGK.I**

---

94. gi|10119899 **Mass:** 100749 **Score:** 73 **Queries matched:** 3
pyruvate phosphate dikinase 1 [Trypanosoma cruzi]
☐ Check to include this hit in error tolerant search or archive report

	Query	Observed	Mr(expt)	Mr(calc)	Delta	Miss	Score	Expect	Rank	Peptide
	1360	609.30	1216.59	1216.60	-0.02	0	57	0.0011	1	K.TAEETLAAAGQR.V
☑	1480	**647.93**	**1293.85**	**1293.77**	**0.08**	**0**	**52**	**0.0031**	**1**	**K.VVPEIMIPLVGK.K**
	2249	797.42	2389.23	2389.06	0.17	1	6	1.1e+02	5	R.SFGAEGVGLCRTEHMFFEGSR.I + Carbamidomethyl (C); Oxidation (M)

Proteins matching the same set of peptides:
gi|71666490 **Mass:** 100767 **Score:** 73 **Queries matched:** 3
pyruvate phosphate dikinase [Trypanosoma cruzi strain CL Brener]

---

95. gi|71649977 **Mass:** 18984 **Score:** 73 **Queries matched:** 1
hypothetical protein [Trypanosoma cruzi strain CL Brener]
☐ Check to include this hit in error tolerant search or archive report

	Query	Observed	Mr(expt)	Mr(calc)	Delta	Miss	Score	Expect	Rank	Peptide
☑	1348	**605.78**	**1209.55**	**1209.62**	**-0.08**	**0**	**73**	**2.2e-05**	**1**	**K.FTENTISITGK.G**

Proteins matching the same set of peptides:
gi|71651746 **Mass:** 19515 **Score:** 73 **Queries matched:** 1
hypothetical protein [Trypanosoma cruzi strain CL Brener]

---

96. gi|71404111 **Mass:** 17506 **Score:** 72 **Queries matched:** 1
40S ribosomal protein S15 [Trypanosoma cruzi strain CL Brener]
☐ Check to include this hit in error tolerant search or archive report

	Query	Observed	Mr(expt)	Mr(calc)	Delta	Miss	Score	Expect	Rank	Peptide
☑	1905	901.92	1801.83	1801.93	-0.11	0	72	3e-05	1	R.GLEIDPLLALSEEEFK.T

Proteins matching the same set of peptides:
gi|154337453 **Mass:** 17447 **Score:** 72 **Queries matched:** 1
40S ribosomal protein S15 [Leishmania braziliensis MHOM/BR/75/M2904]
gi|157869287 **Mass:** 17424 **Score:** 72 **Queries matched:** 1
40S ribosomal protein S15 [Leishmania major strain Friedlin]

---

97. gi|10632 **Mass:** 10404 **Score:** 71 **Queries matched:** 1
TcP2a-RA [Trypanosoma cruzi]
☐ Check to include this hit in error tolerant search or archive report

	Query	Observed	Mr(expt)	Mr(calc)	Delta	Miss	Score	Expect	Rank	Peptide
☑	1280	586.22	1170.42	1170.49	-0.07	0	71	4.4e-05	1	K.DFDTVCTEGK.S + Carbamidomethyl (C)

Proteins matching the same set of peptides:
gi|10640 **Mass:** 10521 **Score:** 71 **Queries matched:** 1
ribosomal P-JL5 protein [Trypanosoma cruzi]
gi|162144 **Mass:** 10306 **Score:** 71 **Queries matched:** 1
acidic ribosomal protein
gi|71407483 **Mass:** 10565 **Score:** 71 **Queries matched:** 1
60S acidic ribosomal protein P2 beta (H6.4) [Trypanosoma cruzi strain CL Brener]
gi|71663723 **Mass:** 10505 **Score:** 71 **Queries matched:** 1
60S acidic ribosomal protein P2 [Trypanosoma cruzi strain CL Brener]

---

98. gi|118766644 **Mass:** 44464 **Score:** 71 **Queries matched:** 2
elongation factor-1 alpha [Oxymonadida environmental sample]
☐ Check to include this hit in error tolerant search or archive report

Query	Observed	Mr(expt)	Mr(calc)	Delta	Miss	Score	Expect	Rank	Peptide

985	513.31	1024.61	1024.60	0.01	0	71	3.7e-05	1	K.IGGIGTVPVGR.V
1472	646.32	1290.63	1290.70	-0.07	2	22	3	3	K.FREIQSKIDR.R

---

99. gi|56156659 Mass: 26484 Score: 71 Queries matched: 2
elongation factor 1A [Echinostelium minutum]
☐ Check to include this hit in error tolerant search or archive report

Query	Observed	Mr(expt)	Mr(calc)	Delta	Miss	Score	Expect	Rank	Peptide
985	513.31	1024.61	1024.60	0.01	0	71	3.7e-05	1	K.IGGIGTVPVGR.V
1658	490.97	1469.88	1469.80	0.08	0	3	2.3e+02	5	R.HRPPYPLQAHVR.G

---

100. gi|71663474 Mass: 68534 Score: 68 Queries matched: 2
dynein [Trypanosoma cruzi strain CL Brener]
☐ Check to include this hit in error tolerant search or archive report

Query	Observed	Mr(expt)	Mr(calc)	Delta	Miss	Score	Expect	Rank	Peptide
☑ 1224	572.32	1142.63	1142.59	0.04	0	35	0.17	1	R.LQEELEVQR.R
☑ 1613	715.88	1429.74	1429.73	0.00	0	68	7.1e-05	1	K.AAIGQMLENQSIR.D

---

101. gi|1781355 Score: 68 Queries matched: 1
histone H2A [Trypanosoma cruzi]
☐ Check to include this hit in error tolerant search or archive report

Query	Observed	Mr(expt)	Mr(calc)	Delta	Miss	Score	Expect	Rank	Peptide
☑ 1250	578.77	1155.52	1155.56	-0.04	0	68	6.7e-05	1	R.HDDDLGMLLK.D

Proteins matching the same set of peptides:

gi|2222802	Score: 68	Queries matched: 1
gi|18266856	Score: 68	Queries matched: 1
gi|71409700	Score: 68	Queries matched: 1
gi|71409702	Score: 68	Queries matched: 1
gi|71649895	Score: 68	Queries matched: 1
gi|71664770	Score: 68	Queries matched: 1

gi|71664802 **Score:** 68 **Queries matched:** 1

---

102. gi|13384081 **Mass:** 13942 **Score:** 68 **Queries matched:** 1
histone H2A [Leishmania infantum]
☐ Check to include this hit in error tolerant search or archive report

Query	Observed	Mr(expt)	Mr(calc)	Delta	Miss	Score	Expect	Rank	Peptide
1250	578.77	1155.52	1155.56	-0.04	0	68	6.7e-05	1	R.HDDDIGMLLK.N

Proteins matching the same set of peptides:
gi|16973355 **Mass:** 13841 **Score:** 68 **Queries matched:** 1
histone H2A [Leishmania infantum]
gi|16973359 **Mass:** 13872 **Score:** 68 **Queries matched:** 1
histone H2A [Leishmania infantum]

---

103. gi|71659778 **Mass:** 61371 **Score:** 68 **Queries matched:** 1
poly(A)-binding protein [Trypanosoma cruzi strain CL Brener]
☐ Check to include this hit in error tolerant search or archive report

	Query	Observed	Mr(expt)	Mr(calc)	Delta	Miss	Score	Expect	Rank	Peptide
☑	1276	**585.78**	**1169.55**	**1169.64**	**-0.09**	**0**	**68**	**6.4e-05**	**1**	**K.VQEALEVLNR.H**

Proteins matching the same set of peptides:
gi|159162751 **Mass:** 9085 **Score:** 68 **Queries matched:** 1
Chain A, Solution Structure Of C-Terminal Domain From Trypanosoma Cruzi Poly(A)-Binding Protein

---

104. gi|37727511 **Mass:** 29319 **Score:** 68 **Queries matched:** 2
adenylate kinase [Trypanosoma cruzi]
☐ Check to include this hit in error tolerant search or archive report

	Query	Observed	Mr(expt)	Mr(calc)	Delta	Miss	Score	Expect	Rank	Peptide
☑	999	**516.29**	**1030.57**	**1030.53**	**0.04**	**0**	**70**	**5.6e-05**	**1**	**R.AEVAAGTEVGK.M**
☑	1001	**516.31**	**1030.60**	**1030.53**	**0.07**	**0**	**(33)**	**0.28**	**1**	**R.AEVAAGTEVGK.M**

Proteins matching the same set of peptides:
gi|71416210 **Mass:** 29323 **Score:** 68 **Queries matched:** 2
adenylate kinase [Trypanosoma cruzi strain CL Brener]
gi|71667254 **Mass:** 29374 **Score:** 68 **Queries matched:** 2
adenylate kinase [Trypanosoma cruzi strain CL Brener]

---

105. gi|71664627 **Mass:** 50806 **Score:** 67 **Queries matched:** 3
hypothetical protein [Trypanosoma cruzi strain CL Brener]
☐ Check to include this hit in error tolerant search or archive report

	Query	Observed	Mr(expt)	Mr(calc)	Delta	Miss	Score	Expect	Rank	Peptide
☑	211	**595.97**	**1189.92**	**1189.46**	**0.46**	**0**	**3**	**6.3e+02**	**1**	**K.CHNSVCGEER.S + Carbamidomethyl (C)**
☑	1341	**603.26**	**1204.50**	**1204.62**	**-0.12**	**0**	**67**	**0.0001**	**1**	**R.VFGSISTNPQR.L**
☑	1455	**635.35**	**1268.69**	**1268.55**	**0.14**	**0**	**33**	**0.27**	**1**	**R.QPVTEDMMYR.V**

---

106. gi|53849793 **Mass:** 32571 **Score:** 66 **Queries matched:** 4
glycosomal glyceraldehyde phosphate dehydrogenase [Trypanosoma mega]
☐ Check to include this hit in error tolerant search or archive report

Query	Observed	Mr(expt)	Mr(calc)	Delta	Miss	Score	Expect	Rank	Peptide
540	406.22	810.43	810.41	0.02	0	17	7.7	5	K.LTGMSFR.V
1312	594.34	1186.66	1186.64	0.02	0	34	0.21	1	K.AVGMVIPSTQGK.L
1577	692.86	1383.71	1383.77	-0.06	0	59	0.0006	1	R.AAAVNIIPSTTGAAK.A
1647	487.27	1458.80	1458.80	-0.00	2	38	0.077	1	R.DTSIKEIDAALKR.A

Proteins matching the same set of peptides:
gi|53849831 **Mass:** 30859 **Score:** 66 **Queries matched:** 4
glycosomal glyceraldehyde phosphate dehydrogenase [Trypanosoma lewisi]
gi|53849833 **Mass:** 32472 **Score:** 66 **Queries matched:** 4
glycosomal glyceraldehyde phosphate dehydrogenase [Trypanosoma microti]
gi|53849849 **Mass:** 33565 **Score:** 66 **Queries matched:** 4
glycosomal glyceraldehyde phosphate dehydrogenase [Trypanosoma sp. Australian rabbit R5]
gi|53849851 **Mass:** 30626 **Score:** 66 **Queries matched:** 4
glycosomal glyceraldehyde phosphate dehydrogenase [Trypanosoma theileri]
gi|159157539 **Mass:** 35137 **Score:** 66 **Queries matched:** 4

glycosomal glyceraldehyde-3-phosphate dehydrogenase [Trypanosoma kuseli]
gi|159157541 **Mass:** 35192 **Score:** 66 **Queries matched:** 4
glycosomal glyceraldehyde-3-phosphate dehydrogenase [Trypanosoma grosi]

---

107. gi|158577406 **Mass:** 36564 **Score:** 66 **Queries matched:** 4
glycosomal glyceraldehyde-3-phosphate dehydrogenase [Crithidia oncopelti]
☐ Check to include this hit in error tolerant search or archive report

Query	Observed	Mr(expt)	Mr(calc)	Delta	Miss	Score	Expect	Rank	Peptide
540	406.22	810.43	810.41	0.02	0	17	7.7	5	K.LTGMSFR.V
1312	594.34	1186.66	1186.67	-0.02	1	34	0.21	1	K.AVGMVIPSTKGK.L
1577	692.86	1383.71	1383.77	-0.06	0	59	0.0006	1	R.AAAVNIIPSTTGAAK.A
1647	487.27	1458.80	1458.80	-0.00	2	38	0.077	1	R.DTSIKEIDAALKR.A

Proteins matching the same set of peptides:
gi|158577417 **Mass:** 36694 **Score:** 66 **Queries matched:** 4
glycosomal glyceraldehyde-3-phosphate dehydrogenase [Crithidia oncopelti]

---

108. gi|71660723 **Mass:** 116424 **Score:** 66 **Queries matched:** 2
hypothetical protein [Trypanosoma cruzi strain CL Brener]
☐ Check to include this hit in error tolerant search or archive report

	Query	Observed	Mr(expt)	Mr(calc)	Delta	Miss	Score	Expect	Rank	Peptide
☑	1971	954.43	1906.84	1906.96	-0.12	0	65	0.00015	1	R.IVATDLSEPLPAPADDAGR.A
☑	2257	**805.97**	**2414.88**	**2415.03**	**-0.15**	**0**	**25**	**1.6**	**1**	**R.AEEPCQHEIEQNYPDSEGVGK.F + Carbamidomethyl (C)**

Proteins matching the same set of peptides:
gi|71418777 **Mass:** 116402 **Score:** 66 **Queries matched:** 2
hypothetical protein [Trypanosoma cruzi strain CL Brener]

---

109. gi|71410849 **Mass:** 29589 **Score:** 66 **Queries matched:** 2
protein disulfide isomerase [Trypanosoma cruzi strain CL Brener]
☐ Check to include this hit in error tolerant search or archive report

Query	Observed	Mr(expt)	Mr(calc)	Delta	Miss	Score	Expect	Rank	Peptide

1396	619.78	1237.55	1237.56	-0.01	0	66	0.00012	1	R.NFDAVVMDEAK.D
1825	563.83	1688.47	1687.89	0.58	1	2	4.9e+02	2	K.KAAADLTGVEAVQYPR.I

---

110. gi|71405387 **Mass:** 17182 **Score:** 65 **Queries matched:** 3
hypothetical protein [Trypanosoma cruzi strain CL Brener]
☐ Check to include this hit in error tolerant search or archive report

	Query	Observed	Mr(expt)	Mr(calc)	Delta	Miss	Score	Expect	Rank	Peptide
☑	1299	591.76	1181.51	1181.57	-0.06	0	31	0.31	1	R.YHVDYTSVAK.Q
☑	1451	634.33	1266.64	1266.66	-0.01	0	64	0.00017	1	R.ASVADQEGHILK.E
☑	1452	423.22	1266.64	1266.66	-0.01	0	(31)	0.33	1	R.ASVADQEGHILK.E

---

111. gi|71424867 **Mass:** 75756 **Score:** 64 **Queries matched:** 1
ATP-dependent zinc metallopeptidase [Trypanosoma cruzi strain CL Brener]
☐ Check to include this hit in error tolerant search or archive report

	Query	Observed	Mr(expt)	Mr(calc)	Delta	Miss	Score	Expect	Rank	Peptide
☑	400	723.00	1443.98	1443.76	0.23	0	64	0.0005	1	K.TLQDSIDDVLIGR.K

Proteins matching the same set of peptides:
gi|71651983 **Mass:** 75662 **Score:** 64 **Queries matched:** 1
ATP-dependent zinc metallopeptidase [Trypanosoma cruzi strain CL Brener]

---

112. gi|71403861 **Mass:** 76587 **Score:** 64 **Queries matched:** 1
trans-sialidase [Trypanosoma cruzi strain CL Brener]
☐ Check to include this hit in error tolerant search or archive report

	Query	Observed	Mr(expt)	Mr(calc)	Delta	Miss	Score	Expect	Rank	Peptide
☑	1920	908.96	1815.91	1815.94	-0.03	0	64	0.0002	1	R.SVEELQWVDLFVPQK.T

Proteins matching the same set of peptides:
gi|71404338 **Mass:** 48533 **Score:** 64 **Queries matched:** 1
trans-sialidase [Trypanosoma cruzi strain CL Brener]

113. gi|71420685 **Mass:** 15423 **Score:** 63 **Queries matched:** 1
intraflagellar transport (IFT) protein [Trypanosoma cruzi strain CL Brener]
☐ Check to include this hit in error tolerant search or archive report

	Query	Observed	Mr(expt)	Mr(calc)	Delta	Miss	Score	Expect	Rank	Peptide
☑	1291	**588.26**	**1174.51**	**1174.55**	**-0.04**	**0**	**63**	**0.0003**	**1**	**R.NIVESEAEER.F**

Proteins matching the same set of peptides:
gi|71422341 **Mass:** 15447 **Score:** 63 **Queries matched:** 1
intraflagellar transport (IFT) protein [Trypanosoma cruzi strain CL Brener]
gi|72390311 **Mass:** 15410 **Score:** 63 **Queries matched:** 1
intraflagellar transport (IFT) protein [Trypanosoma brucei TREU927]
gi|154341743 **Mass:** 15433 **Score:** 63 **Queries matched:** 1
intraflagellar transport (IFT) protein [Leishmania braziliensis MHOM/BR/75/M2904]
gi|157872524 **Mass:** 15416 **Score:** 63 **Queries matched:** 1
intraflagellar transport (IFT) protein [Leishmania major strain Friedlin]

---

114. gi|123495829 **Score:** 63 **Queries matched:** 1
small GTP-binding protein [Trichomonas vaginalis G3]
☐ Check to include this hit in error tolerant search or archive report

Query	Observed	Mr(expt)	Mr(calc)	Delta	Miss	Score	Expect	Rank	Peptide
1104	543.27	1084.52	1084.65	-0.13	0	63	0.00025	3	K.ILVIGESAVGK.S

---

115. gi|23306650 **Score:** 63 **Queries matched:** 2
heat shock protein 70 [Carpediemonas membranifera]
☐ Check to include this hit in error tolerant search or archive report

Query	Observed	Mr(expt)	Mr(calc)	Delta	Miss	Score	Expect	Rank	Peptide
1379	614.82	1227.62	1227.62	0.00	0	63	0.00029	2	R.VEIIANDQGNR.T
1783	**542.31**	**1623.90**	**1623.82**	**0.08**	**0**	**11**	**38**	**5**	**R.TFQVEEISGMVLQK.M + Oxidation (M)**

---

116. gi|1322228 **Mass:** 30530 **Score:** 63 **Queries matched:** 2

elongation factor 1 alpha

☐ Check to include this hit in error tolerant search or archive report

	Query	Observed	Mr(expt)	Mr(calc)	Delta	Miss	Score	Expect	Rank	Peptide
☑	715	**911.09**	**910.08**	**910.43**	**-0.34**	**0**	**8**	**2.3e+02**	**1**	**R.TIEEYEK.K**
	867	488.28	974.55	974.54	0.01	0	63	0.00038	1	R.LPLQDVYK.I

---

117. gi|438596 **Score:** 63 **Queries matched:** 1

elongation factor 1-alpha

☐ Check to include this hit in error tolerant search or archive report

Query	Observed	Mr(expt)	Mr(calc)	Delta	Miss	Score	Expect	Rank	Peptide
867	488.28	974.55	974.54	0.01	0	63	0.00038	1	R.LPIQDVYK.I

Proteins matching the same set of peptides:

gi|585082 **Score:** 63 **Queries matched:** 1
gi|1322226 **Score:** 63 **Queries matched:** 1
gi|2147344 **Score:** 63 **Queries matched:** 1
gi|2522342 **Score:** 63 **Queries matched:** 1
gi|6017764 **Score:** 63 **Queries matched:** 1
gi|6017766 **Score:** 63 **Queries matched:** 1
gi|6017768 **Score:** 63 **Queries matched:** 1
gi|6017770 **Score:** 63 **Queries matched:** 1
gi|6017772 **Score:** 63 **Queries matched:** 1
gi|6017774 **Score:** 63 **Queries matched:** 1
gi|6017776 **Score:** 63 **Queries matched:** 1
gi|6017778 **Score:** 63 **Queries matched:** 1
gi|6017780 **Score:** 63 **Queries matched:** 1
gi|6017782 **Score:** 63 **Queries matched:** 1
gi|7417250 **Score:** 63 **Queries matched:** 1
gi|34596984 **Score:** 63 **Queries matched:** 1
gi|159108518 **Score:** 63 **Queries matched:** 1

---

118. gi|71404920 **Mass:** 25087 **Score:** 62 **Queries matched:** 4

hypothetical protein [Trypanosoma cruzi strain CL Brener]

☐ Check to include this hit in error tolerant search or archive report

Query	Observed	Mr(expt)	Mr(calc)	Delta	Miss	Score	Expect	Rank	Peptide
1299	591.76	1181.51	1181.57	-0.06	0	31	0.31	1	R.YHVDYTSVAK.Q
1451	634.33	1266.64	1266.66	-0.01	0	64	0.00017	1	R.ASVADQEGHILK.E
1452	423.22	1266.64	1266.66	-0.01	0	(31)	0.33	1	R.ASVADQEGHILK.E
☑ 1887	**592.09**	**1773.26**	**1772.90**	**0.36**	**0**	**20**	**6.2**	**1**	**R.VTFQFQVVGGGNHDIR.A**

---

119. gi|71420832 **Mass:** 87541 **Score:** 61 **Queries matched:** 1
NUP-1 protein [Trypanosoma cruzi strain CL Brener]
☐ Check to include this hit in error tolerant search or archive report

Query	Observed	Mr(expt)	Mr(calc)	Delta	Miss	Score	Expect	Rank	Peptide
☑ 1109	**543.84**	**1085.67**	**1085.61**	**0.07**	**0**	**61**	**0.00038**	**1**	**R.VVEALSAELR.E**

Proteins matching the same set of peptides:
gi|38202202 **Mass:** 300496 **Score:** 61 **Queries matched:** 1
Tcr300 [Trypanosoma cruzi]
gi|88659206 **Mass:** 348452 **Score:** 61 **Queries matched:** 1
NUP-1 [Trypanosoma cruzi]

---

120. gi|71402229 **Mass:** 32610 **Score:** 60 **Queries matched:** 1
ubiquitin-like protein [Trypanosoma cruzi strain CL Brener]
☐ Check to include this hit in error tolerant search or archive report

Query	Observed	Mr(expt)	Mr(calc)	Delta	Miss	Score	Expect	Rank	Peptide
☑ 1561	**688.34**	**1374.66**	**1374.76**	**-0.10**	**0**	**60**	**0.00049**	**1**	**R.VLYQSQLQQLR.E**

Proteins matching the same set of peptides:
gi|71408304 **Mass:** 32648 **Score:** 60 **Queries matched:** 1
ubiquitin-like protein [Trypanosoma cruzi strain CL Brener]

---

121. gi|71420441 **Mass:** 7307 **Score:** 59 **Queries matched:** 1
hypothetical protein [Trypanosoma cruzi strain CL Brener]
☐ Check to include this hit in error tolerant search or archive report

Query	Observed	Mr(expt)	Mr(calc)	Delta	Miss	Score	Expect	Rank	Peptide
☑ 1965	629.97	1886.90	1886.90	-0.00	0	59	0.00064	1	R.IYDDDGNLKPGETTPPR.N

Proteins matching the same set of peptides:

gi|71663656 **Mass:** 35448 **Score:** 59 **Queries matched:** 1
hypothetical protein [Trypanosoma cruzi strain CL Brener]

---

122. gi|71661916 **Mass:** 17759 **Score:** 59 **Queries matched:** 1
RNA-binding protein [Trypanosoma cruzi strain CL Brener]

☐ Check to include this hit in error tolerant search or archive report

Query	Observed	Mr(expt)	Mr(calc)	Delta	Miss	Score	Expect	Rank	Peptide
☑ 495	789.33	2364.97	2365.21	-0.25	0	59	0.0018	1	R.RPPGFAFLTITPSQAMEFVEK.F

---

123. gi|167375825 **Mass:** 24465 **Score:** 59 **Queries matched:** 1
hypothetical protein [Entamoeba dispar SAW760]

☐ Check to include this hit in error tolerant search or archive report

Query	Observed	Mr(expt)	Mr(calc)	Delta	Miss	Score	Expect	Rank	Peptide
☑ 1495	651.84	1301.67	1301.68	-0.01	0	59	0.00059	1	K.DVTIDISIAEAR.I

---

124. gi|71405064 **Mass:** 10861 **Score:** 59 **Queries matched:** 2
60S acidic ribosomal protein P2 [Trypanosoma cruzi strain CL Brener]

☐ Check to include this hit in error tolerant search or archive report

Query	Observed	Mr(expt)	Mr(calc)	Delta	Miss	Score	Expect	Rank	Peptide
☑ 1171	558.83	1115.65	1115.62	0.03	0	42	0.037	1	R.SVATLVAEGAAK.M
☑ 2355	902.43	2704.28	2704.36	-0.08	0	48	0.0055	1	K.MSAVAVSAAPAAGGAAAPAAAAGGAAAPAAADAK.K

---

125. gi|154341937 **Mass:** 39180 **Score:** 59 **Queries matched:** 5
glyceraldehyde 3-phosphate dehydrogenase,glycosomal [Leishmania braziliensis MHOM/BR/75/M2904]

☐ Check to include this hit in error tolerant search or archive report

Query	Observed	Mr(expt)	Mr(calc)	Delta	Miss	Score	Expect	Rank	Peptide
540	406.22	810.43	810.41	0.02	0	17	7.7	5	K.LTGMSFR.V
1312	594.34	1186.66	1186.67	-0.02	1	34	0.21	1	K.AVGMVIPSTKGK.L
1352	**606.84**	**1211.67**	**1211.72**	**-0.06**	**1**	**2**	**3.4e+02**	**4**	**K.KVVISAPASGGVK.T**
1577	692.86	1383.71	1383.77	-0.06	0	59	0.0006	1	R.AAAVNIIPSTTGAAK.A
1913	604.59	1810.74	1810.81	-0.06	0	13	21	2	K.IVSWYDNEWGYSHR.V

---

126. gi|71656526 **Mass:** 17060 **Score:** 58 **Queries matched:** 1
hypothetical protein [Trypanosoma cruzi strain CL Brener]
☐ Check to include this hit in error tolerant search or archive report

	Query	Observed	Mr(expt)	Mr(calc)	Delta	Miss	Score	Expect	Rank	Peptide
☑	1642	**727.84**	**1453.66**	**1453.70**	**-0.05**	**0**	**58**	**0.00073**	**1**	**K.DVNVQPEGDSLPGK.G**

Proteins matching the same set of peptides:
gi|71664372 **Mass:** 17118 **Score:** 58 **Queries matched:** 1
hypothetical protein [Trypanosoma cruzi strain CL Brener]

---

127. gi|9954108 **Mass:** 34491 **Score:** 57 **Queries matched:** 1
RNA binding protein RGGm [Trypanosoma cruzi]
☐ Check to include this hit in error tolerant search or archive report

	Query	Observed	Mr(expt)	Mr(calc)	Delta	Miss	Score	Expect	Rank	Peptide
☑	1605	**710.31**	**1418.60**	**1418.70**	**-0.10**	**0**	**57**	**0.0011**	**1**	**R.AVVEFVTPEDASR.A**

Proteins matching the same set of peptides:
gi|71410145 **Mass:** 34635 **Score:** 57 **Queries matched:** 1
RNA-binding protein RGGm [Trypanosoma cruzi strain CL Brener]
gi|71652462 **Mass:** 33389 **Score:** 57 **Queries matched:** 1
RNA-binding protein RGGm [Trypanosoma cruzi strain CL Brener]

---

128. gi|12083381 **Mass:** 64218 **Score:** 56 **Queries matched:** 3

antigen 38 [Trypanosoma cruzi]

☐ Check to include this hit in error tolerant search or archive report

	Query	Observed	Mr(expt)	Mr(calc)	Delta	Miss	Score	Expect	Rank	Peptide
☑	**1726**	**779.38**	**1556.74**	**1556.82**	**-0.08**	**0**	**44**	**0.019**	**1**	**R.ILLFPEGNQEEIR.R**
☑	**1853**	**572.00**	**1712.97**	**1712.92**	**0.05**	**1**	**25**	**1.3**	**1**	**R.ILLFPEGNQEEIRR.H**
☑	**2360**	**905.65**	**2713.92**	**2714.05**	**-0.14**	**1**	**39**	**0.08**	**1**	**R.YRDEEEMDDEGGLEEAQEGNDER.E**

Proteins matching the same set of peptides:

gi|71410919 **Mass:** 63977 **Score:** 56 **Queries matched:** 3

lectin [Trypanosoma cruzi strain CL Brener]

---

129. gi|4323557 **Mass:** 60528 **Score:** 56 **Queries matched:** 2

chaperonin 60.2 precursor [Leishmania donovani]

☐ Check to include this hit in error tolerant search or archive report

	Query	Observed	Mr(expt)	Mr(calc)	Delta	Miss	Score	Expect	Rank	Peptide
	588	422.26	842.51	842.52	-0.02	0	56	0.0015	1	K.LSGGVAVIK.V
	1775	809.33	1616.65	1616.84	-0.18	2	11	37	2	K.VGGATELEVSEKKDR.V

Proteins matching the same set of peptides:

gi|146104317 **Mass:** 60517 **Score:** 56 **Queries matched:** 2

chaperonin Hsp60, mitochondrial precursor [Leishmania infantum]

---

130. gi|1217626 **Mass:** 59740 **Score:** 56 **Queries matched:** 2

heat shock protein 60 [Euglena gracilis]

☐ Check to include this hit in error tolerant search or archive report

	Query	Observed	Mr(expt)	Mr(calc)	Delta	Miss	Score	Expect	Rank	Peptide
	588	422.26	842.51	842.52	-0.02	0	56	0.0015	1	K.LSGGVAVIK.V
☑	**1906**	**904.02**	**1806.04**	**1805.87**	**0.16**	**0**	**8**	**85**	**1**	**K.TLDTELEVVEGMSLDR.G**

Proteins matching the same set of peptides:

gi|2493645 **Mass:** 59669 **Score:** 56 **Queries matched:** 2

RecName: Full=Chaperonin CPN60, mitochondrial; AltName: Full=HSP 60; Flags: Precursor

---

131. gi|123438714 **Score:** 55 **Queries matched:** 1
small GTP-binding protein [Trichomonas vaginalis G3]
☐ Check to include this hit in error tolerant search or archive report

Query	Observed	Mr(expt)	Mr(calc)	Delta	Miss	Score	Expect	Rank	Peptide
1494	651.82	1301.62	1301.64	-0.02	0	55	0.0015	3	K.LQVWDTAGQER.F

Proteins matching the same set of peptides:
gi|123451581 **Score:** 55 **Queries matched:** 1

---

132. gi|167527390 **Score:** 55 **Queries matched:** 1
hypothetical protein [Monosiga brevicollis MX1]
☐ Check to include this hit in error tolerant search or archive report

Query	Observed	Mr(expt)	Mr(calc)	Delta	Miss	Score	Expect	Rank	Peptide
1494	651.82	1301.62	1301.64	-0.02	0	55	0.0015	3	K.IQVWDTAGQER.F

---

133. gi|71409962 **Mass:** 12839 **Score:** 54 **Queries matched:** 2
calpain-like cysteine peptidase [Trypanosoma cruzi strain CL Brener]
☐ Check to include this hit in error tolerant search or archive report

	Query	Observed	Mr(expt)	Mr(calc)	Delta	Miss	Score	Expect	Rank	Peptide
☑	1149	**1102.46**	**1101.46**	**1101.58**	**-0.13**	**0**	**14**	**49**	**1**	**K.LNFEANPVAK.-**
☑	1491	**650.80**	**1299.59**	**1299.62**	**-0.04**	**1**	**54**	**0.0024**	**1**	**K.KQWAFYNDTK.E**

Proteins matching the same set of peptides:
gi|71411006 **Mass:** 12798 **Score:** 54 **Queries matched:** 2
calpain-like cysteine peptidase [Trypanosoma cruzi strain CL Brener]

---

134. gi|71407010 **Mass:** 12231 **Score:** 54 **Queries matched:** 2
cytochrome c [Trypanosoma cruzi strain CL Brener]
☐ Check to include this hit in error tolerant search or archive report

Query	Observed	Mr(expt)	Mr(calc)	Delta	Miss	Score	Expect	Rank	Peptide
☑ 1225	**573.30**	**1144.59**	**1144.54**	**0.05**	**0**	**39**	**0.072**	**1**	**R.SGTVEGFAYSK.A**
1525	667.91	1333.81	1333.72	0.09	1	43	0.029	1	R.ADLIAYLATLRD.-

---

135. gi|10638 **Mass:** 10415 **Score:** 54 **Queries matched:** 1
ribosomal protein P-JL5 [Trypanosoma cruzi]
☐ Check to include this hit in error tolerant search or archive report

Query	Observed	Mr(expt)	Mr(calc)	Delta	Miss	Score	Expect	Rank	Peptide
☑ 1219	**571.25**	**1140.48**	**1140.48**	**0.00**	**0**	**54**	**0.0021**	**1**	**K.DFDTVCAEGK.S + Carbamidomethyl (C)**

Proteins matching the same set of peptides:
gi|436146 **Mass:** 10405 **Score:** 54 **Queries matched:** 1
TcP2beta [Trypanosoma cruzi]

---

136. gi|1729846 **Mass:** 49711 **Score:** 53 **Queries matched:** 1
Tubulin beta-3 chain (Beta-3-tubulin)
☐ Check to include this hit in error tolerant search or archive report

Query	Observed	Mr(expt)	Mr(calc)	Delta	Miss	Score	Expect	Rank	Peptide
1773	808.43	1614.85	1614.83	0.02	0	53	0.0024	1	R.AVLIDLEPGTMDAVR.A + Oxidation (M)

---

137. gi|71412308 **Mass:** 152202 **Score:** 53 **Queries matched:** 1
hypothetical protein [Trypanosoma cruzi strain CL Brener]
☐ Check to include this hit in error tolerant search or archive report

Query	Observed	Mr(expt)	Mr(calc)	Delta	Miss	Score	Expect	Rank	Peptide
☑ 1923	**607.33**	**1818.95**	**1818.95**	**0.01**	**0**	**53**	**0.0024**	**1**	**R.LVVETELHPADAELQR.R**

Proteins matching the same set of peptides:
gi|71649424 **Mass:** 151616 **Score:** 53 **Queries matched:** 1
hypothetical protein [Trypanosoma cruzi strain CL Brener]

138. gi|71398774 **Mass:** 29630 **Score:** 53 **Queries matched:** 1
hypothetical protein [Trypanosoma cruzi strain CL Brener]
☐ Check to include this hit in error tolerant search or archive report

	Query	Observed	Mr(expt)	Mr(calc)	Delta	Miss	Score	Expect	Rank	Peptide
☑	1300	591.82	1181.63	1181.57	0.05	0	53	0.0021	1	K.FSVDVEYAPR.C

Proteins matching the same set of peptides:
gi|71398776 **Mass:** 27357 **Score:** 53 **Queries matched:** 1
hypothetical protein [Trypanosoma cruzi strain CL Brener]
gi|71398971 **Mass:** 29609 **Score:** 53 **Queries matched:** 1
hypothetical protein [Trypanosoma cruzi strain CL Brener]
gi|71404900 **Mass:** 29621 **Score:** 53 **Queries matched:** 1
hypothetical protein [Trypanosoma cruzi strain CL Brener]
gi|71661320 **Mass:** 29622 **Score:** 53 **Queries matched:** 1
hypothetical protein [Trypanosoma cruzi strain CL Brener]
gi|71668366 **Mass:** 29617 **Score:** 53 **Queries matched:** 1
hypothetical protein [Trypanosoma cruzi strain CL Brener]

139. gi|71403995 **Mass:** 36507 **Score:** 52 **Queries matched:** 1
mucin-like glycoprotein [Trypanosoma cruzi strain CL Brener]
☐ Check to include this hit in error tolerant search or archive report

	Query	Observed	Mr(expt)	Mr(calc)	Delta	Miss	Score	Expect	Rank	Peptide
☑	1874	878.89	1755.77	1755.84	-0.07	0	52	0.0026	1	R.EATDGAQQLQSQPQAGK.A

Proteins matching the same set of peptides:
gi|71406134 **Mass:** 36450 **Score:** 52 **Queries matched:** 1
mucin-like glycoprotein [Trypanosoma cruzi strain CL Brener]
gi|71406333 **Mass:** 36521 **Score:** 52 **Queries matched:** 1
hypothetical protein [Trypanosoma cruzi strain CL Brener]

140. gi|163881518 **Score:** 52 **Queries matched:** 1
heat shock protein 70 [Trimastix pyriformis]

☐ Check to include this hit in error tolerant search or archive report

Query	Observed	Mr(expt)	Mr(calc)	Delta	Miss	Score	Expect	Rank	Peptide
1672	744.34	1486.67	1486.69	-0.03	0	52	0.0028	2	R.TTPSYVAFTESER.L

---

141. gi|71401492 **Mass:** 46811 **Score:** 52 **Queries matched:** 2
fatty acid desaturase [Trypanosoma cruzi strain CL Brener]
☐ Check to include this hit in error tolerant search or archive report

	Query	Observed	Mr(expt)	Mr(calc)	Delta	Miss	Score	Expect	Rank	Peptide
☑	1721	**776.84**	**1551.67**	**1551.80**	**-0.13**	**0**	**(28)**	**0.69**	**1**	**R.VDNLTVAPGPPDVMK.A**
☑	1722	**776.87**	**1551.73**	**1551.80**	**-0.07**	**0**	**55**	**0.0015**	**1**	**R.VDNLTVAPGPPDVMK.A**

---

142. gi|28779307 **Score:** 51 **Queries matched:** 2
beta-tubulin [Streblomastix strix]
☐ Check to include this hit in error tolerant search or archive report

Query	Observed	Mr(expt)	Mr(calc)	Delta	Miss	Score	Expect	Rank	Peptide
1630	723.83	1445.65	1445.68	-0.03	0	51	0.0039	1	K.EVDEQMLNVQNK.N
1773	808.43	1614.85	1614.83	0.02	0	38	0.079	3	R.AILVDLEPGTMDSVR.A

Proteins matching the same set of peptides:
gi|28779311 **Score:** 51 **Queries matched:** 2
gi|28779458 **Score:** 51 **Queries matched:** 2
gi|86451920 **Score:** 51 **Queries matched:** 2
gi|118766718 **Score:** 51 **Queries matched:** 2
gi|188766742 **Score:** 51 **Queries matched:** 2

---

143. gi|156988212 **Score:** 51 **Queries matched:** 2
beta-tubulin [Phytophthora polonica]
☐ Check to include this hit in error tolerant search or archive report

Query	Observed	Mr(expt)	Mr(calc)	Delta	Miss	Score	Expect	Rank	Peptide
1531	671.28	1340.55	1340.67	-0.13	0	25	1.6	2	R.IVVYYNEATGGR.Y

1630	723.83	1445.65	1445.68	-0.03	0	51	0.0039	1	K.EVDEQMLNVQNK.N

Proteins matching the same set of peptides:
gi|156988214 **Score:** 51 **Queries matched:** 2

---

144. gi|1729847 **Score:** 51 **Queries matched:** 2
Tubulin beta-4 chain (Beta-4-tubulin)
☐ Check to include this hit in error tolerant search or archive report

Query	Observed	Mr(expt)	Mr(calc)	Delta	Miss	Score	Expect	Rank	Peptide
1630	723.83	1445.65	1445.68	-0.03	0	51	0.0039	1	K.EVDEQMLNVQNK.N
2433	1031.78	3092.33	3092.40	-0.07	0	20	3.9	2	K.FWEVISDEHGIDPTGTYNGDSDLQLER.V

---

145. gi|55274319 **Score:** 51 **Queries matched:** 2
beta-tubulin [Phytophthora ramorum]
☐ Check to include this hit in error tolerant search or archive report

Query	Observed	Mr(expt)	Mr(calc)	Delta	Miss	Score	Expect	Rank	Peptide
1630	723.83	1445.65	1445.68	-0.03	0	51	0.0039	1	K.EVDEQMLNVQNK.N
1773	808.43	1614.85	1614.81	0.04	0	12	29	8	R.AILMDLPPGTMDSVR.A

---

146. gi|188766724 **Score:** 51 **Queries matched:** 2
beta-tubulin [Phytophthora ramorum]
☐ Check to include this hit in error tolerant search or archive report

Query	Observed	Mr(expt)	Mr(calc)	Delta	Miss	Score	Expect	Rank	Peptide
1296	**393.95**	**1178.83**	**1178.69**	**0.14**	**0**	**9**	**55**	**2**	**K.LAVNLIPFHR.L**
1630	723.83	1445.65	1445.68	-0.03	0	51	0.0039	1	K.EVDEQMLNVQNK.N

---

147. gi|11596164 **Score:** 51 **Queries matched:** 2
beta-tubulin [Acrasis rosea]
☐ Check to include this hit in error tolerant search or archive report

Query	Observed	Mr(expt)	Mr(calc)	Delta	Miss	Score	Expect	Rank	Peptide
1630	723.83	1445.65	1445.68	-0.03	0	51	0.0039	1	K.EVDEQMLNVQNK.N
1756	530.97	1589.89	1589.85	0.03	2	7	1e+02	6	R.HGRYLTASAVFRGR.M

---

148. gi|3790449 **Score:** 51 **Queries matched:** 2
beta-tubulin-1 [Chlorarachnion CCMP621]
☐ Check to include this hit in error tolerant search or archive report

Query	Observed	Mr(expt)	Mr(calc)	Delta	Miss	Score	Expect	Rank	Peptide
1630	723.83	1445.65	1445.68	-0.03	0	51	0.0039	1	K.EVDEQMLNVQNK.N
2433	1031.78	3092.33	3092.40	-0.07	0	4	1.5e+02	3	K.FWEVVSDEHGVDPTGTYQGESDLQLER.I

---

149. gi|157887509 **Score:** 51 **Queries matched:** 2
beta tubulin [Plasmodiophora brassicae]
☐ Check to include this hit in error tolerant search or archive report

Query	Observed	Mr(expt)	Mr(calc)	Delta	Miss	Score	Expect	Rank	Peptide
1549	453.58	1357.71	1357.64	0.07	0	4	2.2e+02	9	R.MMATFSVFPSPK.V + Oxidation (M)
1630	723.83	1445.65	1445.68	-0.03	0	51	0.0039	1	K.EVDEQMLNVQNK.N

---

150. gi|168830539 **Score:** 51 **Queries matched:** 2
beta-tubulin [Andalucia godoyi]
☐ Check to include this hit in error tolerant search or archive report

Query	Observed	Mr(expt)	Mr(calc)	Delta	Miss	Score	Expect	Rank	Peptide
1630	723.83	1445.65	1445.68	-0.03	0	51	0.0039	1	K.EVDEQMLNVQNK.N
1980	**641.35**	**1921.02**	**1920.85**	**0.17**	**0**	**3**	**2.1e+02**	**4**	**K.MAVTFCGNSTAIQEMFR.R + Oxidation (M)**

---

151. gi|55274341 **Mass:** 32085 **Score:** 51 **Queries matched:** 2
beta-tubulin [Phytophthora ramorum]
☐ Check to include this hit in error tolerant search or archive report

Query	Observed	Mr(expt)	Mr(calc)	Delta	Miss	Score	Expect	Rank	Peptide

Query	Observed	Mr(expt)	Mr(calc)	Delta	Miss	Score	Expect	Rank	Peptide
1630	723.83	1445.65	1445.68	-0.03	0	51	0.0039	1	K.EVDEQMLNVQNK.N
☑ 790	**942.29**	**2823.84**	**2823.34**	**0.51**	**0**	**3**	**8.9e+02**	**1**	**K.LTTPTYGDLNHLVCANMSGITTCLR.F + 2 Carbamidomethyl (C); Oxidation (M)**

152. gi|3790456 **Mass:** 43100 **Score:** 51 **Queries matched:** 1
beta-tubulin-5 [Chlorarachnion CCMP621]
☐ Check to include this hit in error tolerant search or archive report

Query	Observed	Mr(expt)	Mr(calc)	Delta	Miss	Score	Expect	Rank	Peptide
1630	723.83	1445.65	1445.68	-0.03	0	51	0.0039	1	K.DIDEQMLNVQNK.N

153. gi|71656799 **Mass:** 199163 **Score:** 51 **Queries matched:** 2
hypothetical protein [Trypanosoma cruzi strain CL Brener]
☐ Check to include this hit in error tolerant search or archive report

Query	Observed	Mr(expt)	Mr(calc)	Delta	Miss	Score	Expect	Rank	Peptide
1509	**658.35**	**1314.69**	**1314.75**	**-0.06**	**2**	**10**	**45**	**2**	**K.LAPMKLFDPKR.V**
☑ 1659	**736.91**	**1471.81**	**1471.72**	**0.09**	**0**	**51**	**0.0048**	**1**	**R.ASGPTAVDGTAPEATK.L**

Proteins matching the same set of peptides:
gi|71423437 **Mass:** 199490 **Score:** 51 **Queries matched:** 2
hypothetical protein [Trypanosoma cruzi strain CL Brener]

154. gi|71409309 **Mass:** 23271 **Score:** 50 **Queries matched:** 2
hypothetical protein [Trypanosoma cruzi strain CL Brener]
☐ Check to include this hit in error tolerant search or archive report

Query	Observed	Mr(expt)	Mr(calc)	Delta	Miss	Score	Expect	Rank	Peptide
☑ 1509	658.35	1314.69	1314.66	0.03	0	25	1.6	1	K.HVYPYDPPTVK.I
☑ 1740	**785.84**	**1569.66**	**1569.80**	**-0.14**	**1**	**50**	**0.005**	**1**	**K.KLPESEIQEQVDR.I**

Proteins matching the same set of peptides:
gi|71420399 **Mass:** 23299 **Score:** 50 **Queries matched:** 2
hypothetical protein [Trypanosoma cruzi strain CL Brener]

155. gi|123487079 **Score:** 50 **Queries matched:** 1
IBR domain containing protein [Trichomonas vaginalis G3]
☐ Check to include this hit in error tolerant search or archive report

	Query	Observed	Mr(expt)	Mr(calc)	Delta	Miss	Score	Expect	Rank	Peptide
	1386	616.84	1231.67	1231.62	0.05	2	50	0.0061	2	K.RCPNCKAAIEK.N

156. gi|71655671 **Mass:** 25094 **Score:** 49 **Queries matched:** 1
hypothetical protein [Trypanosoma cruzi strain CL Brener]
☐ Check to include this hit in error tolerant search or archive report

	Query	Observed	Mr(expt)	Mr(calc)	Delta	Miss	Score	Expect	Rank	Peptide
☑	1981	**641.37**	**1921.09**	**1921.05**	**0.04**	**0**	**49**	**0.0052**	**1**	**K.ISAMGAAIHNAVNIAEIVK.R**

Proteins matching the same set of peptides:
gi|71655673 **Mass:** 24439 **Score:** 49 **Queries matched:** 1
hypothetical protein [Trypanosoma cruzi strain CL Brener]

157. gi|71411394 **Mass:** 40782 **Score:** 48 **Queries matched:** 1
fructose-bisphosphate aldolase, glycosomal [Trypanosoma cruzi strain CL Brener]
☐ Check to include this hit in error tolerant search or archive report

	Query	Observed	Mr(expt)	Mr(calc)	Delta	Miss	Score	Expect	Rank	Peptide
☑	1634	725.33	1448.65	1448.76	-0.11	0	48	0.007	1	K.ASTGETFVQLLQR.K

Proteins matching the same set of peptides:
gi|71414541 **Mass:** 40808 **Score:** 48 **Queries matched:** 1
fructose-bisphosphate aldolase, glycosomal [Trypanosoma cruzi strain CL Brener]
gi|71414543 **Mass:** 40780 **Score:** 48 **Queries matched:** 1
fructose-bisphosphate aldolase, glycosomal [Trypanosoma cruzi strain CL Brener]

158. gi|154346376 **Score:** 48 **Queries matched:** 1
hypothetical protein [Leishmania braziliensis MHOM/BR/75/M2904]

Check to include this hit in error tolerant search or archive report

Query	Observed	Mr(expt)	Mr(calc)	Delta	Miss	Score	Expect	Rank	Peptide
1104	543.27	1084.52	1084.65	-0.13	0	48	0.0076	4	R.ILVLGEASVGK.T

---

159. gi|71410728 **Mass:** 13987 **Score:** 48 **Queries matched:** 1
nuclear transport factor 2 [Trypanosoma cruzi strain CL Brener]
Check to include this hit in error tolerant search or archive report

Query	Observed	Mr(expt)	Mr(calc)	Delta	Miss	Score	Expect	Rank	Peptide
☑ 1556	**685.92**	**1369.84**	**1369.70**	**0.13**	**0**	**48**	**0.0073**	**1**	**R.FANLGFNEAIFK.A**

Proteins matching the same set of peptides:
gi|71417055 **Mass:** 13946 **Score:** 48 **Queries matched:** 1
nuclear transport factor 2 [Trypanosoma cruzi strain CL Brener]

---

160. gi|71665043 **Mass:** 10372 **Score:** 48 **Queries matched:** 1
hypothetical protein [Trypanosoma cruzi strain CL Brener]
Check to include this hit in error tolerant search or archive report

Query	Observed	Mr(expt)	Mr(calc)	Delta	Miss	Score	Expect	Rank	Peptide
☑ 1475	**647.31**	**1292.61**	**1292.60**	**0.01**	**0**	**48**	**0.0085**	**1**	**R.LQENYDAGVER.E**

---

161. gi|71422058 **Mass:** 14910 **Score:** 48 **Queries matched:** 2
mitochondrial RNA binding protein [Trypanosoma cruzi strain CL Brener]
Check to include this hit in error tolerant search or archive report

Query	Observed	Mr(expt)	Mr(calc)	Delta	Miss	Score	Expect	Rank	Peptide
☑ 774	**468.24**	**934.46**	**934.47**	**-0.01**	**0**	**48**	**0.012**	**1**	**K.VISWMSGR.G**
☑ 1559	**686.32**	**1370.62**	**1370.64**	**-0.01**	**1**	**29**	**0.65**	**1**	**R.GFGFIEDDTDKK.Q**

---

162. gi|58414949 **Score:** 48 **Queries matched:** 5

polyubiquitin [Stauracon pallidus]

☐ Check to include this hit in error tolerant search or archive report

Query	Observed	Mr(expt)	Mr(calc)	Delta	Miss	Score	Expect	Rank	Peptide
459	383.22	764.43	764.43	0.01	0	25	1.4	4	-.MQIFVK.T
1070	534.32	1066.62	1066.61	0.00	0	48	0.0086	1	K.ESTLHLVLR.L
1701	508.62	1522.83	1522.77	0.06	1	23	2.7	1	K.IQDKEGIPPDQQR.L
1867	874.42	1746.83	1746.89	-0.06	0	21	3.8	5	K.TITLEVEASDSIENVK.A
2174	750.04	2247.10	2247.18	-0.09	1	5	1.5e+02	8	K.TLTGKTITLEVEASDSIENVK.A

---

163. gi|33358312 **Score:** 48 **Queries matched:** 2

ubiquitin-like protein Ublp94.4 [Acanthamoeba castellanii]

☐ Check to include this hit in error tolerant search or archive report

Query	Observed	Mr(expt)	Mr(calc)	Delta	Miss	Score	Expect	Rank	Peptide
1070	534.32	1066.62	1066.61	0.00	0	48	0.0086	1	K.ESTLHLVLR.L
1701	508.62	1522.83	1522.77	0.06	1	23	2.7	1	K.LQDKEGIPPDQQR.L

---

164. gi|154411942 **Mass:** 27817 **Score:** 48 **Queries matched:** 1

hypothetical protein [Trichomonas vaginalis G3]

☐ Check to include this hit in error tolerant search or archive report

Query	Observed	Mr(expt)	Mr(calc)	Delta	Miss	Score	Expect	Rank	Peptide
1070	534.32	1066.62	1066.61	0.00	0	48	0.0086	1	R.DTTIHLVLR.C

---

165. gi|71402841 **Mass:** 17709 **Score:** 47 **Queries matched:** 1

60S ribosomal protein L12 [Trypanosoma cruzi strain CL Brener]

☐ Check to include this hit in error tolerant search or archive report

Query	Observed	Mr(expt)	Mr(calc)	Delta	Miss	Score	Expect	Rank	Peptide
☑ 2253	**802.03**	**2403.07**	**2403.18**	**-0.11**	**0**	**47**	**0.0089**	**1**	**K.SVVMEVLGTALSVGCTIDGENPR.E + Carbamidomethyl (C)**

Proteins matching the same set of peptides:

gi|71413453 **Mass:** 17681 **Score:** 47 **Queries matched:** 1
60S ribosomal protein L12 [Trypanosoma cruzi strain CL Brener]
gi|71417151 **Mass:** 21668 **Score:** 47 **Queries matched:** 1
60S ribosomal protein L12 [Trypanosoma cruzi strain CL Brener]
gi|71650641 **Mass:** 17680 **Score:** 47 **Queries matched:** 1
60S ribosomal protein L12 [Trypanosoma cruzi strain CL Brener]

---

166. gi|133055 **Mass:** 10747 **Score:** 47 **Queries matched:** 1
60S acidic ribosomal protein P1
☐ Check to include this hit in error tolerant search or archive report

	Query	Observed	Mr(expt)	Mr(calc)	Delta	Miss	Score	Expect	Rank	Peptide
☑	2312	850.43	2548.26	2548.34	-0.08	0	47	0.0094	1	K.VSFGGVAPAAGGATAAPAAAAAAAAPAAAAAK.K

---

167. gi|71412664 **Mass:** 60479 **Score:** 46 **Queries matched:** 1
hypothetical protein [Trypanosoma cruzi strain CL Brener]
☐ Check to include this hit in error tolerant search or archive report

	Query	Observed	Mr(expt)	Mr(calc)	Delta	Miss	Score	Expect	Rank	Peptide
☑	1747	788.87	1575.73	1575.78	-0.04	0	46	0.013	1	R.SPGFNPAAVPYTPMK.T

---

168. gi|71411561 **Mass:** 14251 **Score:** 46 **Queries matched:** 1
hypothetical protein [Trypanosoma cruzi strain CL Brener]
☐ Check to include this hit in error tolerant search or archive report

	Query	Observed	Mr(expt)	Mr(calc)	Delta	Miss	Score	Expect	Rank	Peptide
☑	1039	526.31	1050.60	1050.57	0.03	0	46	0.013	1	R.LATFTELTR.Q

Proteins matching the same set of peptides:
gi|71667238 **Mass:** 14290 **Score:** 46 **Queries matched:** 1
hypothetical protein [Trypanosoma cruzi strain CL Brener]

---

169. gi|154420635 **Score:** 45 **Queries matched:** 1
DEAD/DEAH box helicase family protein [Trichomonas vaginalis G3]

☐ Check to include this hit in error tolerant search or archive report

Query	Observed	Mr(expt)	Mr(calc)	Delta	Miss	Score	Expect	Rank	Peptide
1920	908.96	1815.91	1815.92	-0.01	2	45	0.015	2	K.RMKAMLDIEEEVPQK.M

---

170. gi|71659535 **Mass:** 28380 **Score:** 45 **Queries matched:** 1
COP-coated vesicle membrane protein erv25 precursor [Trypanosoma cruzi strain CL Brener]
☐ Check to include this hit in error tolerant search or archive report

	Query	Observed	Mr(expt)	Mr(calc)	Delta	Miss	Score	Expect	Rank	Peptide
☑	1978	**640.61**	**1918.81**	**1918.84**	**-0.03**	**0**	**45**	**0.014**	**1**	**K.DTHPVCFVEEVDESTR.V + Carbamidomethyl (C)**

---

171. gi|11055687 **Mass:** 63589 **Score:** 45 **Queries matched:** 2
ATPase alpha subunit [Trypanosoma brucei brucei]
☐ Check to include this hit in error tolerant search or archive report

	Query	Observed	Mr(expt)	Mr(calc)	Delta	Miss	Score	Expect	Rank	Peptide
☑	1194	**564.82**	**1127.62**	**1127.59**	**0.03**	**0**	**45**	**0.017**	**1**	**K.VDTGAPNIVSR.S**
☑	253	**621.11**	**1860.31**	**1860.07**	**0.25**	**0**	**23**	**10**	**1**	**K.VMATGQLLHIPVGAGVLGK.V**

Proteins matching the same set of peptides:
gi|71401425 **Mass:** 25841 **Score:** 45 **Queries matched:** 2
ATP synthase, alpha chain, mitochondrial precursor [Trypanosoma cruzi strain CL Brener]
gi|72392136 **Mass:** 63462 **Score:** 45 **Queries matched:** 2
ATP synthase alpha chain, mitochondrial precursor [Trypanosoma brucei TREU927]

---

172. gi|33867787 **Mass:** 21361 **Score:** 44 **Queries matched:** 1
21 kDa cyclophilin [Trypanosoma cruzi]
☐ Check to include this hit in error tolerant search or archive report

	Query	Observed	Mr(expt)	Mr(calc)	Delta	Miss	Score	Expect	Rank	Peptide
☑	991	514.79	1027.58	1027.58	-0.01	0	44	0.016	1	K.HVVFGQITK.G

Proteins matching the same set of peptides:
gi|71406866 **Mass:** 21389 **Score:** 44 **Queries matched:** 1
21 kDa cyclophilin [Trypanosoma cruzi strain CL Brener]
gi|71412806 **Mass:** 22908 **Score:** 44 **Queries matched:** 1
cyclophilin [Trypanosoma cruzi strain CL Brener]

---

173. gi|71404616 **Mass:** 11926 **Score:** 44 **Queries matched:** 3
hypothetical protein [Trypanosoma cruzi strain CL Brener]
☐ Check to include this hit in error tolerant search or archive report

	Query	Observed	Mr(expt)	Mr(calc)	Delta	Miss	Score	Expect	Rank	Peptide
☑	2079	700.33	2097.97	2098.00	-0.02	0	32	0.28	1	K.GLEHYGLDPTDAEVANEIR.K
☑	431	743.02	2226.04	2226.09	-0.05	1	21	14	1	K.GLEHYGLDPTDAEVANEIRK.Y
☑	2206	766.35	2296.02	2296.13	-0.11	0	36	0.13	1	R.HLNYEELENNIGGLPITENK.L

Proteins matching the same set of peptides:
gi|71405087 **Mass:** 11954 **Score:** 44 **Queries matched:** 3
hypothetical protein [Trypanosoma cruzi strain CL Brener]

---

174. gi|123420403 **Score:** 44 **Queries matched:** 1
hypothetical protein [Trichomonas vaginalis G3]
☐ Check to include this hit in error tolerant search or archive report

Query	Observed	Mr(expt)	Mr(calc)	Delta	Miss	Score	Expect	Rank	Peptide
1492	650.86	1299.71	1299.71	-0.01	1	44	0.023	2	K.GQVSLKSIPESR.I

Proteins matching the same set of peptides:
gi|123498457 **Score:** 44 **Queries matched:** 1
gi|154411769 **Score:** 44 **Queries matched:** 1

---

175. gi|167516814 **Score:** 44 **Queries matched:** 2
hypothetical protein [Monosiga brevicollis MX1]
☐ Check to include this hit in error tolerant search or archive report

Query	Observed	Mr(expt)	Mr(calc)	Delta	Miss	Score	Expect	Rank	Peptide
1386	616.84	1231.67	1231.66	0.01	2	44	0.02	3	K.DRTSNAAKLTR.R
1682	**753.31**	**1504.61**	**1504.97**	**-0.36**	**1**	**2**	**2.9e+02**	**5**	**R.LIVNIPDLVLLKR.M**

---

176. gi\|27734387 **Score:** 44 **Queries matched:** 2
polyubiquitin [Euglypha rotunda]
☐ Check to include this hit in error tolerant search or archive report

Query	Observed	Mr(expt)	Mr(calc)	Delta	Miss	Score	Expect	Rank	Peptide
1701	508.62	1522.83	1522.77	0.06	1	23	2.7	1	K.IQDKEGIPPDQQR.L
1867	874.42	1746.83	1746.89	-0.06	0	44	0.02	2	K.TITLDVEASDTIENVK.Q

Proteins matching the same set of peptides:
gi\|27734383 **Score:** 44 **Queries matched:** 2

---

177. gi\|461992 **Mass:** 24450 **Score:** 44 **Queries matched:** 2
25 kDa elongation factor 1-beta (EF-1-beta)
☐ Check to include this hit in error tolerant search or archive report

	Query	Observed	Mr(expt)	Mr(calc)	Delta	Miss	Score	Expect	Rank	Peptide
☑	1695	**506.96**	**1517.86**	**1517.85**	**0.02**	**1**	**23**	**2.8**	**1**	**K.LFLGGTKPSKEDVK.L**
☑	2275	**825.69**	**2474.06**	**2474.27**	**-0.21**	**0**	**44**	**0.017**	**1**	**K.SSILFDVKPWDDTVDLQALANK.L**

---

178. gi\|71402893 **Mass:** 24437 **Score:** 44 **Queries matched:** 2
25 kDa translation elongation factor 1-beta [Trypanosoma cruzi strain CL Brener]
☐ Check to include this hit in error tolerant search or archive report

Query	Observed	Mr(expt)	Mr(calc)	Delta	Miss	Score	Expect	Rank	Peptide
1695	506.96	1517.86	1517.81	0.05	0	21	4.1	2	K.LFLGGTKPSQEDVK.L
2275	825.69	2474.06	2474.27	-0.21	0	44	0.017	1	K.SSILFDVKPWDDTVDLQALANK.L

---

179. gi\|225587 **Score:** 43 **Queries matched:** 3
tubulin alpha

Check to include this hit in error tolerant search or archive report

Query	Observed	Mr(expt)	Mr(calc)	Delta	Miss	Score	Expect	Rank	Peptide
1854	858.48	1714.94	1714.91	0.02	0	43	0.021	2	K.AVFLDLEPTVIDEVR.T
1855	572.66	1714.95	1714.91	0.04	0	(31)	0.33	2	K.AVFLDLEPTVIDEVR.T
**734**	**919.35**	**2755.01**	**2755.38**	**-0.37**	**2**	**3**	**7.9e+02**	**5**	**K.VQRAVCMLSNTTAIAEVFSRIDMK.F + Carbamidomethyl (C); Oxidation (M)**

---

180. gi|84105377 **Score:** 43 **Queries matched:** 2
alpha tubulin 2 [Rhynchopus sp. ATCC 50230]
Check to include this hit in error tolerant search or archive report

Query	Observed	Mr(expt)	Mr(calc)	Delta	Miss	Score	Expect	Rank	Peptide
1854	858.48	1714.94	1714.88	0.06	0	43	0.021	2	R.AVMLDLEPTVIDEVR.T + Oxidation (M)
1855	572.66	1714.95	1714.88	0.07	0	(31)	0.33	2	R.AVMLDLEPTVIDEVR.T + Oxidation (M)

Proteins matching the same set of peptides:
gi|84105383 **Score:** 43 **Queries matched:** 2

---

181. gi|116222245 **Score:** 43 **Queries matched:** 2
alpha tubulin [Thaumatomonas sp. TMT002]
Check to include this hit in error tolerant search or archive report

Query	Observed	Mr(expt)	Mr(calc)	Delta	Miss	Score	Expect	Rank	Peptide
1854	858.48	1714.94	1714.91	0.02	0	43	0.021	2	R.AVFIDLEPTVIDEVR.T
1855	572.66	1714.95	1714.91	0.04	0	(31)	0.33	2	R.AVFIDLEPTVIDEVR.T

Proteins matching the same set of peptides:
gi|116222249 **Score:** 43 **Queries matched:** 2
gi|116222289 **Score:** 43 **Queries matched:** 2
gi|156066414 **Score:** 43 **Queries matched:** 2

---

182. gi|146081753 **Score:** 43 **Queries matched:** 2
hypothetical protein [Leishmania infantum JPCM5]
Check to include this hit in error tolerant search or archive report

Query	Observed	Mr(expt)	Mr(calc)	Delta	Miss	Score	Expect	Rank	Peptide
1461	640.34	1278.66	1278.63	0.04	0	43	0.026	2	R.LMQLDVFEER.A
1462	640.38	1278.74	1278.63	0.11	0	(40)	0.058	2	R.LMQLDVFEER.A

Proteins matching the same set of peptides:

gi|154334391 **Score:** 43 **Queries matched:** 2

gi|157866707 **Score:** 43 **Queries matched:** 2

Peptide matches not assigned to protein hits: (no details means no match)

	Query	Observed	Mr(expt)	Mr(calc)	Delta	Miss	Score	Expect	Rank	Peptide
☑	2276	826.15	2475.42	2475.25	0.16	0	41	0.035	1	SSILFDVKPWDDTVDLQALADK
☑	121	543.00	1625.98	1625.70	0.28	0	40	0.13	1	KPGEYESVTDDCAR + Carbamidomethyl (C)
☑	141	557.39	1669.15	1668.91	0.24	0	40	0.13	1	FGQAPLTKPWGNVVR
☑	1760	798.89	1595.77	1595.79	-0.03	0	39	0.058	1	YVDMFEAGIIDPVK
☑	1112	544.36	1086.70	1086.60	0.09	0	39	0.061	1	LENTTQIIR
☑	1055	530.80	1059.58	1059.60	-0.02	1	39	0.069	1	DVIDIVKMK
☑	1320	596.82	1191.63	1191.56	0.07	0	39	0.065	1	CVGEVCGIDLK + Carbamidomethyl (C)
☑	1522	665.40	1328.79	1328.77	0.02	1	39	0.066	1	VKTLESLLAAER
☑	1498	652.85	1303.68	1303.67	0.01	0	39	0.079	1	MPWLAIPFSSR
☑	2165	746.38	2236.12	2236.03	0.09	2	38	0.078	1	KVNLDDDDRWLEAEFDEK
☑	2112	717.96	2150.86	2150.91	-0.05	0	37	0.1	1	LADHCLASAGNQNTCSSMSK + 2 Carbamidomethyl (C)
☑	1158	553.76	1105.51	1105.53	-0.03	0	37	0.096	1	NFMPQTVNR
☑	791	471.78	941.55	941.52	0.04	0	37	0.087	1	IEIPLDSR
☑	1580	463.28	1386.82	1386.82	-0.00	1	36	0.15	1	KVPIVPLEDLHK
☑	1553	682.89	1363.77	1363.73	0.04	0	35	0.15	1	VSIVATDIFTGNK
☑	1720	775.83	1549.65	1549.82	-0.18	0	35	0.16	1	WGIIEETVLCIFK
☑	706	454.22	906.42	906.49	-0.07	0	35	0.16	1	VTNTGLFR
☑	1000	516.30	1030.58	1030.55	0.03	0	35	0.19	1	VLMPLAESR + Oxidation (M)
☑	1038	526.28	1050.54	1050.57	-0.04	0	35	0.15	1	VVAFLYDPK
☑	135	552.71	1103.41	1103.60	-0.19	0	35	0.39	1	FAEALLCIPK
☑	673	443.83	885.66	885.51	0.14	1	35	0.18	1	VRAVASQR
☑	118	539.53	1077.05	1077.56	-0.52	0	35	0.56	1	FLGAYGTPPR
☑	1063	532.75	1063.49	1063.51	-0.02	0	34	0.23	1	VVAMGDGPFR + Oxidation (M)

☑	1799	550.96	1649.87	1649.88	-0.01	2	34	0.18	1	FLKGQGTWTKEDIK
☑	1727	520.27	1557.79	1557.85	-0.06	0	34	0.2	1	DIVVLRPEVYAER
☑	349	684.42	683.41	683.42	-0.01	0	33	0.17	1	EPVVLK
☑	1696	507.26	1518.75	1518.65	0.11	0	33	0.25	1	ACYNCGQPGHLSR + 2 Carbamidomethyl (C)
☑	2336	876.41	2626.20	2626.21	-0.01	1	32	0.23	1	MIEGVELSEDIFEELKEEACTR + Carbamidomethyl (C)
☑	637	435.28	868.54	868.49	0.06	1	32	0.25	1	RDAGVVPR
☑	1849	855.91	1709.81	1709.90	-0.09	0	32	0.3	1	LLDVVCSPENLNIPK + Carbamidomethyl (C)
☑	920	499.31	996.60	996.62	-0.02	0	31	0.3	1	LVLVVEPTK
☑	512	797.50	796.49	796.47	0.02	0	31	1.1	1	ILLDLNP
☑	2177	750.39	2248.16	2248.09	0.07	1	31	0.34	1	KGAPQTSSSGGSTTATTTAANAGPK
☑	1130	546.80	1091.58	1091.59	-0.01	0	31	0.45	1	TLESLPFTGK
☑	1979	961.21	1920.41	1920.90	-0.48	0	30	0.69	1	GLGFDVDTLEEELEAER
☑	675	444.31	886.60	886.53	0.08	0	30	0.59	1	ILLFPER
☑	2239	790.02	2367.04	2367.20	-0.16	1	30	0.44	1	KIQPSIPEDTTTGVGGLGNVDNR
☑	1431	629.84	1257.67	1257.66	0.01	0	30	0.55	1	SLCEGLVEAIPK
☑	1891	596.00	1784.97	1784.89	0.08	0	30	0.48	1	LQQESKPEQLDSVER
☑	1651	732.94	1463.86	1463.76	0.10	0	30	0.52	1	EAQTPVPAEQGLPK
☑	1155	553.30	1104.58	1104.62	-0.04	1	29	0.6	1	IIEKVFDNK
☑	459	383.22	764.43	764.39	0.04	1	29	0.57	1	MKDMLK
☑	1558	686.32	1370.62	1370.73	-0.11	0	29	0.65	1	NIDIEIDELVAK
☑	718	911.44	910.43	910.55	-0.12	0	29	0.59	1	DGLPVGLLK
☑	2403	977.43	2929.26	2929.30	-0.05	0	28	0.53	1	GDGNDDQAQFEEEIMHENEAILGALGK
☑	299	645.77	1289.54	1289.70	-0.16	1	28	3.3	1	IQFGGDEVVKAK
☑	1762	800.86	1599.70	1599.85	-0.16	0	28	0.72	1	ELIAFGTPLEATNPK
☑	1191	564.33	1126.65	1126.60	0.05	1	28	0.69	1	SIDRVPSEPK
☑	1885	886.95	1771.88	1771.78	0.10	0	28	0.68	1	GSINSNVMNNSFNSSGK + Oxidation (M)
☑	1631	723.93	1445.84	1445.75	0.09	1	28	0.83	1	ILIETVREACDK + Carbamidomethyl (C)
☑	2124	725.01	2172.01	2171.96	0.05	0	27	0.91	1	ATANSGTSQPQQPSQEGETPAS
☑	1367	611.37	1220.72	1220.59	0.14	0	27	1.1	1	ENHISFLSMK + Oxidation (M)
☑	2396	963.76	2888.26	2888.23	0.03	0	27	0.86	1	TEYQQAAAANSSSSSGNTDSSQGEQQQK
☑	869	489.31	976.60	976.60	0.00	1	27	1.1	1	LDKYLVVK
☑	1501	654.34	1306.67	1306.67	0.00	2	26	1.3	1	KIMEAGSWKNK + Oxidation (M)
☑	1581	695.36	1388.70	1388.79	-0.09	2	26	1.2	1	RSLLTSAPFNKR
☑	1730	521.29	1560.85	1560.75	0.10	0	26	1.3	1	LPPGTQESDEPHVR

☑	1123	545.72	1089.42	1089.60	-0.17	1	26	1.4	1	LSMQVKSAAR
☑	1197	565.81	1129.61	1129.60	0.02	0	25	1.6	1	LLQSVNDDVK
☑	1368	611.37	1220.72	1220.59	0.14	0	25	1.6	1	ENHISFLSMK + Oxidation (M)
☑	1079	536.79	1071.57	1071.64	-0.06	0	25	1.8	1	TPLICATLLK
☑	1472	646.32	1290.63	1290.69	-0.06	0	25	1.7	1	IATELMSLIER + Oxidation (M)
☑	641	436.51	871.01	870.53	0.48	1	25	3.3	1	VIEKNIR
☑	2140	1101.30	2200.58	2200.13	0.46	0	24	2.6	1	IENVAEVPLVVEDAVQGYEK
☑	1220	571.25	1140.48	1140.50	-0.02	0	24	1.9	1	GLSSSSSEEMK
☑	1469	643.84	1285.66	1285.67	-0.01	1	24	1.8	1	RLGPSWATWGR
☑	1649	730.90	1459.79	1459.74	0.06	1	24	1.8	1	YSVGEIYTKCIK + Carbamidomethyl (C)
☑	1567	691.30	1380.59	1380.72	-0.14	2	24	1.6	1	KNAEYKIDSVSK
☑	285	638.90	1275.78	1275.76	0.03	1	24	6.5	1	IKSIYDVAVIR
☑	1490	650.73	1299.45	1299.58	-0.13	1	24	2.4	1	KVEDMQTDYR + Oxidation (M)
☑	940	504.24	1006.47	1006.54	-0.07	0	24	1.9	1	HPPQIIMR + Oxidation (M)
☑	1181	561.34	1120.67	1120.67	0.00	1	24	2	1	YLLCKIIAK + Carbamidomethyl (C)
☑	1582	695.82	1389.62	1389.69	-0.08	0	24	2.4	1	CAETSLLEAQLR + Carbamidomethyl (C)
☑	856	487.25	972.48	972.60	-0.12	2	24	2.5	1	IELKTNKK
☑	747	462.76	923.50	923.46	0.04	1	24	1.7	1	WHPDKNK
☑	1913	604.59	1810.74	1810.81	-0.07	1	24	2	1	LVDGCAFSFHCQDKK + 2 Carbamidomethyl (C)
☑	2167	747.29	2238.85	2239.11	-0.26	0	24	2	1	IQPSIPEDTTTGVGGLGNVDNR
☑	2196	759.08	2274.21	2274.28	-0.07	0	24	1.9	1	ISQRPNVTGIIVVNNEGVPIR
☑	471	774.15	1546.28	1546.67	-0.39	0	24	9	1	LPEASSNTAEEDER
☑	1547	679.33	1356.65	1356.72	-0.08	1	23	2.4	1	INETNDSKLVPK
☑	2012	664.44	1990.31	1989.98	0.32	0	23	2.4	1	SLTDFMGQGVPEEIAQLR
☑	189	588.27	587.26	587.34	-0.08	0	23	7.2	1	AITGAR
☑	412	733.88	1465.75	1465.71	0.05	0	23	10	1	NASQDGPAPHVFAR
☑	1298	590.86	1179.71	1179.73	-0.03	1	23	2.4	1	NLILKNLPQK
☑	1078	536.29	1070.58	1070.61	-0.03	0	23	2.5	1	GVVLTVEQAR
☑	1780	811.86	1621.70	1621.80	-0.10	0	23	2.2	1	EVYEDMQAIIAVNK
☑	1763	800.91	1599.80	1599.80	-0.00	0	23	2.4	1	GADPVNEELTVLSEK
☑	347	682.28	2043.81	2043.95	-0.14	1	23	6.3	1	LLCSVAEGRSAAMAMMIDK + 3 Oxidation (M)
☑	1031	1046.09	1045.08	1045.50	-0.43	0	23	5.3	1	GIEEASVDAR
☑	1738	785.41	1568.80	1568.92	-0.12	1	23	2.5	1	SARLETLLLAVVER
☑	1718	772.37	1542.72	1542.83	-0.11	0	23	2.5	1	INEVLTQVLTEER

☑	1458	638.29	1274.57	1274.71	-0.14	1	23	2.7	1	DVALAYLRAGAR
☑	1752	793.42	1584.82	1584.89	-0.08	1	23	2.7	1	VGSTSPTRLLNLTAR
☑	142	557.79	1113.57	1113.60	-0.02	0	22	10	1	CRPLILDER
☑	1761	799.97	1597.92	1597.88	0.04	1	22	3.2	1	QQQEQQNTIKLIK
☑	1282	586.34	1170.66	1170.61	0.04	1	22	3.2	1	SSHRITSVER
☑	2314	851.40	2551.19	2551.34	-0.15	0	22	2.4	1	LLDAISEAGNVTHLVSTLEELEAK
☑	414	368.23	734.45	734.37	0.08	0	22	3.4	1	SSVWTR
☑	1886	886.96	1771.91	1772.02	-0.11	1	22	2.7	1	LNLMLASTIKGLLQNK + Oxidation (M)
☑	2300	843.76	2528.26	2528.24	0.01	0	22	2.9	1	LVDEIGSQLLQYSDGFYTLPDR
☑	1719	773.43	1544.84	1544.84	-0.01	0	22	3.2	1	IFTPLLNEIETQK
☑	540	406.22	810.43	810.50	-0.07	0	22	2.7	1	LTLSHLK
☑	2106	715.01	2142.00	2142.10	-0.10	0	21	3.3	1	HVTSLHHLDMLLNGTLEGR
☑	1464	641.34	1280.66	1280.55	0.11	0	21	3.4	1	MNVNNSECINK + Oxidation (M)
☑	1313	594.83	1187.64	1187.62	0.03	1	21	4	1	VKVLCPQCNK + Carbamidomethyl (C)
☑	386	712.33	711.32	711.31	0.01	0	21	9.5	1	LCAFAGM
☑	53	459.25	916.49	916.55	-0.05	1	21	9.1	1	VLVRSSTR
☑	323	660.77	659.76	659.39	0.37	0	21	9.7	1	SITIIN
☑	1251	578.87	1155.72	1155.66	0.05	0	21	3.9	1	SPTVSIAAAAIR
☑	1363	610.78	1219.54	1219.56	-0.02	1	21	4.4	1	RMVEDAEAAGR + Oxidation (M)
☑	1650	731.87	1461.72	1461.72	0.00	1	21	4.5	1	TIPANGKFSEDQR
☑	2317	852.61	2554.81	2555.09	-0.29	0	21	6	1	TCVSASHQEQQEEEQQQPQER + Carbamidomethyl (C)
☑	1405	621.26	1240.50	1240.64	-0.13	0	20	4.3	1	LDGYSLMIVSK + Oxidation (M)
☑	979	511.79	1021.57	1021.37	0.20	0	20	4.4	1	MAMMMTSR + 4 Oxidation (M)
☑	1783	542.31	1623.90	1623.80	0.10	1	20	4.5	1	RATTGEHHSYRPGR
☑	978	511.73	1021.45	1021.35	0.10	0	20	4.5	1	MAMMMTCR + 3 Oxidation (M)
☑	302	647.90	646.89	647.35	-0.46	0	20	12	1	TETAVK
☑	392	715.98	2144.93	2145.04	-0.11	1	20	21	1	QHAAHALEELKQDEELER
☑	348	683.78	2048.31	2048.04	0.27	1	20	15	1	GASELRECMELLLTTLGR + Carbamidomethyl (C)
☑	2420	1004.78	3011.33	3011.38	-0.05	0	20	4.3	1	YSDPALCTVDTSSEVVLNTYPDGAPQGR + Carbamidomethyl (C)
☑	1412	622.33	1242.65	1242.63	0.03	0	20	5.3	1	RPGDTSCVRPR
☑	1663	738.35	1474.69	1474.78	-0.10	1	20	5.1	1	LFSSNSFGFLKTK
☑	1683	754.33	1506.64	1506.81	-0.18	2	20	6.7	1	QGHSGRGSRLTPVR
☑	1054	530.33	1058.65	1058.47	0.19	0	20	6.5	1	YYDIDQSR
☑	1564	690.35	1378.69	1378.65	0.05	0	20	5.9	1	EAQLAAYSAENGR

☑	226	604.21	1809.60	1809.94	-0.34	0	19	19	1	DTQAVIVAAVIHAMTDR
☑	1508	658.31	1314.61	1314.63	-0.02	1	19	5.7	1	YIDCASSIKMK + Carbamidomethyl (C)
☑	634	434.68	867.35	867.38	-0.03	0	19	4.2	1	ASEMTCVK
☑	1528	446.92	1337.75	1337.63	0.12	0	19	5.4	1	DEGLAALQAYCK + Carbamidomethyl (C)
☑	129	549.67	1646.00	1645.88	0.12	1	19	15	1	TLLDEAEGVKTELTK
☑	1751	528.96	1583.86	1583.78	0.08	0	19	5.7	1	NAPTAANPPHGAVPDR
☑	1677	498.60	1492.78	1492.69	0.09	1	19	5.7	1	KSYVLTGFCCMNK
☑	1798	550.21	1647.62	1647.86	-0.24	2	19	6.2	1	VVERLSGETVEKMR + Oxidation (M)
☑	2304	844.92	2531.73	2531.27	0.46	0	19	9.2	1	DSFVSPSLVSAGGVIAAFAEGHTNAK
☑	1340	602.87	1203.72	1203.64	0.08	0	19	6.4	1	ISIEEIMELK
☑	530	403.26	804.51	804.46	0.05	1	19	7.2	1	GALRGGFK
☑	1602	708.92	1415.82	1415.71	0.11	1	19	6.2	1	DVSQKCPNDIGIK
☑	1438	631.82	1261.63	1261.64	-0.02	1	19	7	1	EVPSRSTLAMR + Oxidation (M)
☑	1429	627.80	1253.59	1253.61	-0.02	2	19	6.1	1	SSSRSTSRSGSR
☑	328	665.22	664.22	664.38	-0.16	0	19	5.4	1	SIVYAL
☑	1596	471.46	1411.35	1411.75	-0.40	0	19	8.1	1	NELLPEFQGLPR
☑	1037	525.24	1048.46	1048.48	-0.02	0	19	7.3	1	GSSGQHSHPR
☑	1427	627.40	1252.78	1252.73	0.06	1	19	7.3	1	RTPILQQIER
☑	1848	855.44	1708.87	1708.97	-0.10	2	19	6.2	1	SLLMFSRLYPKNLK
☑	1030	523.30	1044.60	1044.55	0.05	0	19	7.4	1	ISALEDEIR
☑	67	485.95	1454.84	1454.77	0.07	1	19	15	1	SPCQLAQPALSRK + Carbamidomethyl (C)
☑	314	327.69	653.37	653.36	0.01	1	18	4	1	KDVHR
☑	975	510.76	1019.51	1019.47	0.04	0	18	7.7	1	NCSLEDAIR
☑	1327	599.92	1197.83	1197.54	0.29	2	18	6.7	1	FACEEKCRR + Carbamidomethyl (C)
☑	781	469.76	937.50	937.47	0.03	0	18	6.8	1	LMIEGGYR
☑	222	602.29	1202.57	1202.62	-0.05	1	18	26	1	INQASGKMAQR
☑	1533	672.89	1343.76	1343.64	0.11	1	18	7.9	1	AAEAERAAAEAER
☑	1725	778.91	1555.80	1555.76	0.04	0	18	9.1	1	ASSEAFNFLQSTVR
☑	221	601.39	1801.15	1800.96	0.19	1	18	23	1	IEEISEFIKNNIQPK
☑	1374	612.84	1223.67	1223.69	-0.02	1	18	7.4	1	LTLGPLPKDDR
☑	923	499.76	997.52	997.63	-0.11	1	18	6.2	1	KGGGLIGGLVK
☑	1548	679.80	1357.58	1357.81	-0.23	1	18	8	1	KPKEFLSALLGR
☑	1554	683.32	1364.63	1364.70	-0.07	2	18	7.6	1	YARRAMAELER
☑	765	465.78	929.55	929.54	0.01	1	18	11	1	IITRTNGR

☑	69	489.83	488.82	488.23	0.59	0	18	21	1	GGEAR
☑	751	463.27	924.52	924.47	0.05	0	18	6.6	1	MVLTYGNK
☑	32	371.09	1110.25	1110.63	-0.38	0	18	15	1	IPLVLDGAEGK
☑	547	408.20	814.40	814.54	-0.14	2	18	8.8	1	AKIKSLR
☑	2240	790.13	2367.38	2367.18	0.20	1	18	7.4	1	NSDFETPLHVAVREGQIDNVK
☑	1077	536.29	1070.57	1070.50	0.07	0	18	9.1	1	MTAPMHEVR
☑	2330	866.39	2596.14	2596.34	-0.20	1	17	8.1	1	TAALFEARTPAVTYYCYLFVLK + Carbamidomethyl (C)
☑	1418	624.86	1247.70	1247.68	0.03	0	17	9.1	1	QLVYELLQDK
☑	1067	533.32	1064.62	1064.60	0.02	1	17	8.5	1	HSRGTVRPR
☑	934	502.73	1003.45	1003.52	-0.08	1	17	10	1	APSMSLRAR + Oxidation (M)
☑	1936	612.92	1835.75	1835.93	-0.18	0	17	9	1	ICENFSQIIEEGLTLK
☑	249	619.87	618.86	619.31	-0.45	0	17	20	1	MGGSLR
☑	1444	633.29	1264.57	1264.70	-0.12	2	17	10	1	GYELVKLRCGK
☑	1314	594.83	1187.65	1187.60	0.04	2	17	10	1	KALDDWERR
☑	690	899.42	898.41	898.42	-0.01	0	17	8.7	1	CAIYNSTK
☑	1468	643.84	1285.66	1285.67	-0.02	1	17	10	1	QAQSGGKNVEIR
☑	525	803.54	802.54	802.40	0.13	1	17	14	1	RNTEAGR
☑	1879	588.23	1761.67	1761.79	-0.12	0	17	9.4	1	MIHELNNQYIGDCR + Carbamidomethyl (C)
☑	361	692.72	2075.15	2075.15	0.00	0	17	34	1	MAALLVRPVASHAPLGMLGR + Oxidation (M)
☑	1237	576.28	1150.54	1150.45	0.09	0	17	10	1	YTEESYCEK
☑	231	606.98	1817.93	1818.07	-0.14	0	17	32	1	LIQPLNLDKPVIAAGTR
☑	204	593.20	1184.38	1184.68	-0.30	1	17	29	1	ILKYLSYSAK
☑	816	952.40	1902.78	1902.85	-0.07	1	17	30	1	SAYELGDVRGCSAYAER + Carbamidomethyl (C)
☑	46	438.59	875.17	875.43	-0.26	0	17	23	1	QVMNSAAR
☑	51	450.79	899.56	899.46	0.10	0	17	23	1	FLQVYCK
☑	2077	1048.52	2095.03	2095.04	-0.02	2	16	10	1	DAHTATGPVHRDITDFSKK
☑	1804	827.94	1653.87	1653.77	0.09	1	16	11	1	RHCQHMISEQLEK + Oxidation (M)
☑	44	430.50	858.98	859.47	-0.48	0	16	38	1	ISAEEALK
☑	2017	999.00	1996.00	1996.10	-0.10	1	16	15	1	GVAKALFDLDIQIEHSIK
☑	1321	597.49	1192.97	1192.63	0.34	0	16	14	1	ICLFSGEIVR + Carbamidomethyl (C)
☑	64	484.56	1450.64	1450.71	-0.07	0	16	26	1	TDCISLLAPYEAR
☑	286	639.35	1276.68	1276.66	0.02	0	16	31	1	HLEPVAEPMVR
☑	1440	421.73	1262.15	1262.60	-0.45	2	16	24	1	EKEREAAQMR + Oxidation (M)
☑	1753	793.77	1585.53	1585.69	-0.16	0	16	13	1	LQSFACGTDDMQVR + Oxidation (M)

☑	1532	672.85	1343.69	1343.78	-0.09	0	16	13	1	SQLVVQSFVIPK
☑	1938	612.95	1835.82	1835.98	-0.16	2	16	12	1	LAKHQGGARELIEQMR
☑	745	462.53	923.04	923.52	-0.48	0	16	18	1	LIHSEGLR
☑	380	706.35	705.34	705.32	0.02	0	16	12	1	TEDDVK
☑	969	509.82	1017.64	1017.60	0.03	1	16	15	1	IPALKWYK
☑	1674	744.84	1487.66	1487.79	-0.14	2	16	12	1	TKDGKTPLSVATDR
☑	1967	632.69	1895.04	1895.07	-0.03	2	16	11	1	HLSTAICQANLKLIDKK
☑	2180	1126.09	2250.17	2250.02	0.15	1	16	12	1	NEWMSSGQGLKVVDEDGEVR + Oxidation (M)
☑	325	663.59	662.58	662.25	0.33	0	16	17	1	DDEER
☑	331	668.81	1335.60	1335.71	-0.12	0	16	47	1	ETASPVVQQLHK
☑	1724	519.08	1554.23	1553.75	0.48	0	16	22	1	AVQMGIANSYESLR + Oxidation (M)
☑	70	489.94	977.86	977.51	0.35	1	16	33	1	RLSNMSVR + Oxidation (M)
☑	1818	835.46	1668.91	1668.81	0.10	1	16	13	1	SIDATSVATTMASRSR + Oxidation (M)
☑	1353	607.30	1212.58	1212.65	-0.07	1	16	13	1	ELGKGSEAPVAR
☑	815	476.43	950.85	951.40	-0.54	0	16	20	1	CGDAVDSTK + Carbamidomethyl (C)
☑	1029	1045.42	3133.25	3133.46	-0.20	2	16	38	1	QLDDMRAIHAAINSFSGVMTKMMMLMK + 4 Oxidation (M)
☑	1446	633.32	1264.62	1264.67	-0.05	0	16	15	1	NTIVTENFISK
☑	359	692.39	691.39	691.40	-0.01	0	16	40	1	GLFLSR
☑	1754	793.85	1585.69	1585.82	-0.13	1	16	15	1	IEIEEIKNDLENK
☑	1417	623.85	1245.68	1245.74	-0.06	2	16	14	1	KVKEEMIILK + Oxidation (M)
☑	1517	663.28	1324.54	1324.69	-0.15	2	15	15	1	SRYTKSLMPSR
☑	1908	604.03	1809.07	1808.89	0.17	0	15	13	1	ELQVQLDVGQNYEFK
☑	1562	689.36	1376.70	1376.73	-0.03	2	15	14	1	KARTTLSETNEK
☑	341	679.61	1357.20	1356.80	0.40	1	15	61	1	SPIKSNISISLAK
☑	1344	604.82	1207.62	1207.58	0.03	0	15	15	1	GDATLVQYADR
☑	895	494.88	987.75	987.56	0.19	0	15	17	1	VISLVNESK
☑	41	418.04	1251.10	1250.65	0.45	1	15	44	1	CTPNIKFFGPK
☑	1202	1133.01	1132.01	1131.59	0.41	0	15	56	1	TYHVTEALAK
☑	726	457.30	912.58	912.45	0.13	1	15	13	1	WKEEGHK
☑	2083	701.70	2102.09	2101.94	0.15	1	15	14	1	AHFDYDFSKVFTDYYGK
☑	185	583.32	1164.63	1164.69	-0.05	1	15	35	1	YTLLTTAVKR
☑	1816	557.26	1668.76	1668.91	-0.15	0	15	15	1	FGQAPLTKPWGNVVR
☑	1901	899.45	1796.88	1796.97	-0.09	0	15	14	1	EIAEFLILHGSDINVK
☑	390	714.98	713.98	713.41	0.57	0	15	28	1	AAAAAVVAA

☑	914	498.25	994.48	994.52	-0.04	0	15	14	1	LVLFSCEK + Carbamidomethyl (C)
☑	1140	549.83	1097.64	1097.64	-0.00	2	15	14	1	AAALRRDAVR
☑	1746	788.71	1575.41	1575.91	-0.50	1	15	23	1	LQSFVRAATTTLIR
☑	1934	612.41	1834.21	1833.91	0.30	1	15	17	1	CLHTPCGRELAAHWLK
☑	420	739.00	1475.98	1475.71	0.27	1	15	46	1	SSEVGHAEYVRSR
☑	1882	589.58	1765.72	1766.01	-0.29	1	15	15	1	EVVLSIKHVPQNLYK
☑	68	487.24	1458.70	1458.76	-0.06	2	15	39	1	RCAERLVDLAEK + Carbamidomethyl (C)
☑	1608	712.35	1422.68	1422.69	-0.01	1	15	17	1	KEANEFGFTGPAR
☑	1703	763.29	1524.56	1524.86	-0.30	1	15	17	1	QLVDLEAEIRALR
☑	1173	559.33	1116.65	1116.63	0.01	2	15	17	1	LTKLNREML
☑	90	516.30	515.30	515.30	0.00	0	15	4.2	1	AEALL
☑	374	699.91	1397.81	1397.67	0.14	1	14	61	1	EVELCMSFKQK + Carbamidomethyl (C)
☑	109	529.61	1585.80	1585.86	-0.06	2	14	50	1	TTLPIQLERSRCVA
☑	714	455.73	909.44	909.60	-0.16	1	14	14	1	VVKILNPK
☑	1511	660.29	1318.56	1318.63	-0.07	0	14	18	1	LDPMMENTLQK
☑	2016	665.38	1993.11	1992.97	0.14	2	14	16	1	RCEEGRASAFEELVELR
☑	1687	755.80	1509.59	1509.79	-0.20	0	14	18	1	VAGQEPNQLVQATR
☑	958	508.27	1014.54	1014.63	-0.09	2	14	22	1	RLLSAATRK
☑	1616	717.89	1433.78	1433.68	0.10	1	14	18	1	RLLECVNDMER + Carbamidomethyl (C)
☑	1467	643.26	1284.52	1284.60	-0.08	1	14	19	1	EISCVRCSFK + 2 Carbamidomethyl (C)
☑	702	905.82	1809.63	1809.83	-0.20	1	14	58	1	FPGDFGGPGMGPGGFGGRK + Oxidation (M)
☑	1748	527.29	1578.84	1578.88	-0.04	1	14	18	1	TNSVTAGPIPLPTRR
☑	593	423.72	845.42	845.39	0.03	0	14	25	1	FPTGCHK + Carbamidomethyl (C)
☑	1597	707.26	1412.51	1412.75	-0.24	1	14	17	1	NEKLEADLVPGTK
☑	577	832.52	2494.55	2494.21	0.34	2	14	65	1	MCSEAQRRGGLLCEDTTTLVVR + Carbamidomethyl (C)
☑	408	730.53	1459.04	1458.69	0.34	1	14	75	1	ESTYDCLRFIR + Carbamidomethyl (C)
☑	1587	465.25	1392.72	1392.69	0.03	1	14	18	1	KSEEEENNFVAVK
☑	1810	554.29	1659.84	1659.81	0.03	1	14	18	1	SVEDLYDKLHNEAK
☑	1449	633.81	1265.60	1265.64	-0.04	0	14	19	1	HHGWGVYVVGR
☑	1523	665.85	1329.68	1329.76	-0.08	2	14	22	1	TLSRCPVIASRK
☑	1733	783.84	1565.67	1565.93	-0.26	1	14	19	1	LPLATRTRPEIATK
☑	1729	780.92	1559.84	1559.74	0.10	1	14	20	1	MYDKNLINNTYR + Oxidation (M)
☑	1088	539.48	1076.94	1076.55	0.38	1	14	31	1	MSAVLADDKK
☑	892	494.79	987.56	987.52	0.05	0	14	23	1	QVLEACLGR

☑	1603	709.50	1416.98	1416.69	0.30	1	14	24	1	LMNPPKTMSQDR
☑	1450	634.01	1266.00	1265.69	0.32	0	14	25	1	EALTHGWALLR
☑	1940	919.47	1836.93	1836.87	0.06	1	14	19	1	CAVACKIMTPLCNAASK + 2 Carbamidomethyl (C)
☑	2249	797.42	2389.23	2389.13	0.10	1	14	16	1	CVVRVIDTNDSNAQLGGGETER + Carbamidomethyl (C)
☑	2158	742.24	2223.70	2223.91	-0.21	1	14	31	1	VTVEQVMCTDMYCREGCK + 2 Carbamidomethyl (C); Oxidation (M)
☑	883	985.48	2953.40	2953.58	-0.18	2	14	53	1	FEKNLIAEDNILFIPTIPGVDDPKQK
☑	2227	783.70	2348.07	2348.17	-0.11	1	14	17	1	ELVLNMNVDQITNEAKNLMK + 2 Oxidation (M)
☑	37	403.21	1206.61	1206.64	-0.03	0	14	46	1	CVVLAVAETFR
☑	701	453.25	904.49	904.46	0.03	0	14	26	1	ASDVSTAVR
☑	1214	570.21	1138.40	1138.52	-0.12	1	14	21	1	AKEMSESTEK
☑	1856	858.90	1715.79	1715.90	-0.11	2	14	21	1	QLQSAQRSLSEARSR
☑	2183	752.40	2254.19	2254.00	0.20	0	14	21	1	GGEQMLLTSDSSVDCDEILDK
☑	75	497.29	1488.86	1488.79	0.07	2	14	40	1	MYNILFRFNKK + Oxidation (M)
☑	1673	744.37	1486.73	1486.83	-0.09	2	14	21	1	RLIDNFLTPDKR
☑	2329	863.22	2586.62	2586.27	0.36	1	13	21	1	CSKITDIIINGNISEIGSSAFSSCK
☑	1425	418.54	1252.59	1252.53	0.06	0	13	24	1	MMNSFEYFGK
☑	631	433.78	865.55	865.47	0.08	0	13	23	1	AVVSFICK
☑	2182	751.77	2252.29	2252.10	0.19	2	13	22	1	ERFLACMEGINRASAATGEVK
☑	1390	617.87	1233.72	1233.63	0.09	0	13	21	1	EMSPILSLNSK + Oxidation (M)
☑	1264	389.20	1164.59	1164.60	-0.01	0	13	21	1	FNIIALCESR
☑	2261	809.75	2426.24	2426.24	0.00	0	13	21	1	CTLSNLQNLSLFESTISTLQSK
☑	1075	536.14	1070.27	1070.56	-0.29	0	13	25	1	VQAGFSGHIR
☑	2207	767.11	2298.31	2298.08	0.23	0	13	20	1	AQDELIEAPLAASQTDDVCPR + Carbamidomethyl (C)
☑	1476	647.43	1292.84	1292.75	0.10	0	13	25	1	ILVVDIHESIR
☑	1163	555.32	1108.62	1108.58	0.04	0	13	21	1	VAAYTQESLK
☑	25	338.57	1012.70	1012.57	0.13	0	13	50	1	ECPLLLLR + Carbamidomethyl (C)
☑	1572	691.97	1381.93	1381.79	0.14	2	13	21	1	QVKVHRLVMEK + Oxidation (M)
☑	1619	479.75	1436.22	1436.76	-0.54	2	13	43	1	LRDTSVFRNVCK
☑	1676	747.34	1492.66	1492.80	-0.14	0	13	23	1	IIADLQHQLESAR
☑	1847	854.38	1706.74	1706.84	-0.10	2	13	23	1	CQDKTSSDKPSLRSR
☑	1551	681.33	1360.65	1360.74	-0.09	1	13	24	1	DIIFSNGPKVSGK
☑	457	764.59	763.58	763.42	0.15	2	13	88	1	LFKDKN
☑	1591	703.39	1404.76	1404.71	0.05	0	13	28	1	LWGTLLSPSEMR + Oxidation (M)
☑	1941	919.47	1836.93	1836.88	0.05	1	13	24	1	RLQIMHDDYENFLK + Oxidation (M)

☑	2053	1025.94	2049.86	2049.98	-0.12	1	13	25	1	ACHHLSLLYDDRFFTR + Carbamidomethyl (C)
☑	101	521.23	1560.68	1560.80	-0.13	1	13	52	1	TTTLAADVARMQQR
☑	1645	486.72	1457.14	1456.76	0.38	2	13	38	1	SYKVAVTMKTDSK
☑	180	581.48	1160.94	1160.63	0.31	1	13	88	1	EKLLVSTEDK
☑	993	515.28	1028.54	1028.66	-0.12	0	13	27	1	LIPYLIAVK
☑	2146	1106.02	2210.03	2210.10	-0.07	0	13	23	1	YICDAGVLVEIQINDAYVR + Carbamidomethyl (C)
☑	49	447.58	893.15	893.47	-0.32	0	13	51	1	HSLGDLPR
☑	1380	614.84	1227.67	1227.64	0.04	0	13	28	1	NLLLHYNEGR
☑	700	905.12	904.11	904.43	-0.32	0	13	37	1	NITSAEDR
☑	1460	640.33	1278.65	1278.70	-0.05	1	13	28	1	VLINGHDRDLK
☑	1281	586.28	1170.55	1170.59	-0.04	0	13	29	1	IYPCYFPIR
☑	1198	565.82	1129.63	1129.59	0.04	0	13	31	1	LLEELEAGEK
☑	256	622.04	1242.07	1242.61	-0.55	0	13	96	1	TGHINLTMNSR
☑	1479	432.24	1293.71	1293.69	0.02	0	13	26	1	IPCRPLNEQPK
☑	498	790.86	2369.54	2370.04	-0.50	2	13	1.2e+02	1	ECKKCSETSGNIQGVPNCVSCK + Carbamidomethyl (C)
☑	1100	1084.49	2166.98	2167.08	-0.10	1	13	73	1	GMQELEEQLNTAHQLRVR + Oxidation (M)
☑	1538	674.88	1347.76	1347.70	0.05	0	13	28	1	GEYTIPTADILR
☑	1926	609.92	1826.74	1826.97	-0.23	2	13	29	1	GHRWSVLVGMTVASRR + Oxidation (M)
☑	1414	622.92	1243.83	1243.68	0.16	0	13	30	1	DVQDLSVSLIR
☑	198	592.78	1183.55	1183.69	-0.14	1	13	1.1e+02	1	ALDAEKVVLAR
☑	469	772.39	771.38	771.40	-0.02	0	13	84	1	ERPASGR
☑	724	457.21	912.40	912.48	-0.08	0	12	23	1	VWVEQPR
☑	1500	436.49	1306.43	1306.59	-0.16	1	12	30	1	RDEICCWVAR + Carbamidomethyl (C)
☑	1599	708.33	1414.65	1414.84	-0.19	1	12	27	1	VLDVVPKKPHQR
☑	567	826.98	825.97	826.37	-0.40	0	12	1e+02	1	QSHEGNR
☑	1488	433.93	1298.76	1298.68	0.08	0	12	28	1	LPSTCDLAARPR
☑	2084	702.79	2105.36	2105.00	0.36	2	12	29	1	HDCFGERVVDADGKLGPYK
☑	2441	1042.98	3125.92	3126.50	-0.58	2	12	29	1	CLEDGKQNVNICGLTEHPQSNVKSIMR + 2 Carbamidomethyl (C)
☑	1026	522.31	1042.60	1042.60	0.00	1	12	35	1	TPTLNKLEK
☑	599	849.90	848.90	848.44	0.46	1	12	64	1	CWVWKK
☑	158	566.83	1697.47	1697.91	-0.44	0	12	92	1	NINPFILLDFVHEK
☑	372	699.52	2095.53	2095.11	0.43	2	12	1.1e+02	1	EYEKTARLALESVIGFGGR
☑	2072	697.90	2090.68	2091.26	-0.58	2	12	34	1	RLLALNKTISFFSLISLR
☑	1755	795.32	1588.63	1588.78	-0.15	0	12	34	1	ATTIMLPASTDPNNK + Oxidation (M)

☑	842	967.88	1933.75	1933.93	-0.18	1	12	90	1	VKDLIPEDFINPSNCCK
☑	1432	629.89	1257.77	1257.70	0.07	2	12	32	1	RSVRGVCLSPK + Carbamidomethyl (C)
☑	1305	592.50	1182.99	1183.55	-0.57	1	12	39	1	FKNMGDESLK + Oxidation (M)
☑	1482	648.31	1294.60	1294.63	-0.03	0	12	28	1	MAFVGYLYTSK + Oxidation (M)
☑	1708	764.85	1527.69	1527.76	-0.07	0	12	32	1	SNSSLLPATQPGSNR
☑	300	647.17	646.17	646.24	-0.07	0	12	1.3e+02	1	NCAPDA + Carbamidomethyl (C)
☑	156	565.99	1694.96	1694.81	0.15	1	12	83	1	FRTGSNFHCGMLIR + Carbamidomethyl (C)
☑	2025	670.68	2009.03	2008.90	0.13	1	12	29	1	RSIDNEPEPCYQIMNK + Carbamidomethyl (C); Oxidation (M)
☑	72	491.23	980.44	980.57	-0.12	0	12	66	1	LSHVIDAVK
☑	552	818.71	1635.41	1634.91	0.50	0	12	1.1e+02	1	LLNSILDMYATILR
☑	6	263.01	786.02	785.46	0.56	1	12	64	1	EPKLTAK
☑	805	949.40	948.39	948.45	-0.06	0	12	33	1	SGISDTGANK
☑	423	740.67	2218.98	2219.20	-0.22	1	12	1.1e+02	1	EITAVSSWLSVLHRCLLHR
☑	1296	393.95	1178.83	1178.58	0.25	0	12	31	1	LAMIGMEVQR + 2 Oxidation (M)
☑	1407	621.36	1240.70	1240.64	0.05	0	12	31	1	DAPGSQALVVER
☑	79	502.61	501.60	501.32	0.29	0	12	5.5	1	SIIIG
☑	1684	754.84	1507.66	1507.83	-0.16	2	12	31	1	FIRGTVFLDERR
☑	1964	629.42	1885.23	1885.01	0.22	0	12	34	1	LITALGGSGTALGCSPAIAR + Carbamidomethyl (C)
☑	1731	781.96	1561.91	1561.74	0.17	0	11	34	1	GSPAQTTLAMEETAR
☑	2056	685.33	2052.97	2053.16	-0.20	1	11	35	1	IIANDLNALTNSISRGIIR
☑	1922	606.62	1816.83	1816.97	-0.14	1	11	37	1	VEQKMAVQLLVAWMR + Oxidation (M)
☑	932	502.26	1002.50	1002.37	0.13	0	11	50	1	CEFCMTR + 2 Carbamidomethyl (C)
☑	251	620.22	619.21	619.32	-0.11	0	11	36	1	VLYEP
☑	1669	495.70	1484.07	1483.77	0.30	1	11	37	1	FCINMMLKLLSR + Oxidation (M)
☑	371	699.41	698.41	698.33	0.08	0	11	89	1	VFSSIM + Oxidation (M)
☑	1148	1101.27	2200.53	2200.08	0.45	0	11	1.1e+02	1	TQPAAAMDVEFVQGVNLPGEK
☑	1685	755.35	1508.69	1508.85	-0.16	1	11	36	1	NKNLKPIHISSMK
☑	274	629.28	628.27	628.37	-0.09	0	11	1e+02	1	KPASAR
☑	2037	675.97	2024.89	2024.93	-0.04	1	11	31	1	FYSSNFGAQAFEGCEKLK
☑	368	697.89	1393.76	1393.73	0.03	1	11	1.2e+02	1	HSDDPVVSALAKR
☑	1815	833.86	1665.71	1665.81	-0.09	1	11	34	1	MLSARSTVLDDECVK
☑	801	947.96	946.95	946.51	0.44	1	11	1.5e+02	1	EATETLRK
☑	1195	1128.80	3383.37	3383.67	-0.29	2	11	1.3e+02	1	TAEEARVMGHFLLSAGVKATEFTTLSTESER + Oxidation (M)
☑	1952	929.41	1856.80	1856.92	-0.12	0	11	38	1	IISCVYLPSAEFCLSR + Carbamidomethyl (C)

☑	107	527.35	1579.03	1578.76	0.27	0	11	72	1	MDPNLSVIEEYAAK
☑	327	664.97	1327.93	1327.72	0.21	2	11	1.1e+02	1	VREGLGLDKGER
☑	1723	777.36	1552.71	1552.81	-0.10	2	11	37	1	VRLCRDENQVPPK
☑	1811	831.01	1660.00	1659.76	0.24	0	11	36	1	GCFLMELFALDNSK + Carbamidomethyl (C); Oxidation (M)
☑	1881	883.83	1765.64	1765.86	-0.22	1	11	36	1	AVSSDAMALLEEMQKK + Oxidation (M)
☑	1569	691.32	1380.62	1380.68	-0.06	0	11	34	1	NMIFVLVCEEK + Carbamidomethyl (C)
☑	2126	727.12	2178.33	2178.09	0.24	1	11	35	1	GPGTTTAATTETGASVACSLVKR
☑	293	641.36	640.35	640.29	0.06	0	11	19	1	ENHNK
☑	298	645.52	1289.03	1288.66	0.37	1	11	1.7e+02	1	DVKEGETVGSLR
☑	1161	554.13	1106.26	1106.49	-0.24	1	11	39	1	MYEHAERR + Oxidation (M)
☑	2273	823.42	2467.23	2467.18	0.06	2	11	37	1	GVDGNVNGDRRSTLTYMEENLK
☑	1595	704.90	1407.79	1407.86	-0.07	1	11	35	1	LALIASALKAGPQR
☑	1690	505.10	1512.28	1512.71	-0.43	2	11	70	1	KQMMANEINKMK + 3 Oxidation (M)
☑	2121	722.67	2164.99	2165.08	-0.09	0	11	36	1	VEDINGDMTTVTVDSSLLIK + Oxidation (M)
☑	125	544.97	1087.93	1087.56	0.37	0	11	1.3e+02	1	SNAVTDIVNR
☑	1537	674.87	1347.73	1347.63	0.10	0	11	41	1	GGGGATAAASTWWR
☑	1626	480.97	1439.89	1439.65	0.24	1	11	38	1	NECRGTLEEYAR
☑	437	750.40	2248.17	2248.15	0.01	2	11	1.2e+02	1	VSGLVKVGDDVFAVAEARCGEK
☑	988	514.29	1026.56	1026.64	-0.08	1	11	35	1	DVVGGIKVLK
☑	897	495.21	988.41	988.46	-0.05	1	11	45	1	ARAGDEDQK
☑	80	502.89	501.88	501.32	0.56	0	11	1.1e+02	1	LGLLS
☑	1665	494.61	1480.81	1480.68	0.13	1	11	53	1	MELSVSEDGQRSK + Oxidation (M)
☑	1888	592.54	1774.61	1774.76	-0.15	1	11	42	1	MESSISGSGESSGSSKSR + Oxidation (M)
☑	102	521.78	1562.33	1561.84	0.49	1	11	1.3e+02	1	MKTILNSIEELQK + Oxidation (M)
☑	247	618.84	1853.49	1853.83	-0.35	2	11	2.2e+02	1	QPDGYSRERASMNAEK + Oxidation (M)
☑	2027	672.31	2013.91	2013.86	0.05	1	11	39	1	ESRHDGNDLGNPCTPMAK + Carbamidomethyl (C); Oxidation (M)
☑	111	532.13	1062.25	1062.61	-0.36	1	11	1.3e+02	1	EISFLKQAK
☑	2364	910.82	2729.44	2729.34	0.10	0	11	36	1	VDLLPHGVLAASVAEDGTGHPGFADER
☑	24	334.12	999.35	999.57	-0.22	0	11	69	1	NLAKPATTGK
☑	39	414.21	826.41	826.50	-0.10	1	11	75	1	KATAALPR
☑	2181	751.07	2250.19	2250.02	0.17	0	10	40	1	GDLYELVEDGELHEQYWR
☑	1589	467.27	1398.78	1398.88	-0.10	1	10	42	1	LLLSVASKSQLIK
☑	83	508.79	1015.56	1015.53	0.03	0	10	1.3e+02	1	QPGSASGALTK
☑	337	677.17	2028.48	2027.95	0.53	1	10	1.8e+02	1	ANEEAKAAQGAEDLAGVNDR

☑	132	550.85	1099.69	1099.66	0.03	1	10	1.1e+02	1	ILKTGGAELAK
☑	1819	835.98	1669.94	1669.91	0.03	1	10	42	1	LGQLESRGALSALEAR
☑	1973	637.14	1908.39	1908.08	0.31	2	10	69	1	ERIQDLLDGKEVKPIR
☑	74	493.87	1478.58	1478.74	-0.16	1	10	93	1	DYLPLVKQMGDGK + Oxidation (M)
☑	1794	548.25	1641.72	1641.88	-0.16	2	10	44	1	WKAQGQVQEKLAEK
☑	355	690.23	1378.45	1378.69	-0.24	2	10	1.6e+02	1	MSRSSKSLQDPK + Oxidation (M)
☑	689	897.05	896.04	896.48	-0.44	1	10	64	1	DKFVNFK
☑	2266	817.75	2450.22	2450.36	-0.15	2	10	39	1	VVDDKGTVLNTVGPFAPPRLTVR
☑	451	760.48	759.47	759.47	0.00	1	10	64	1	AGKMILK
☑	1573	692.39	1382.78	1382.80	-0.02	1	10	45	1	LLTAQLQSPTRR
☑	2404	981.07	2940.18	2940.23	-0.05	1	10	39	1	RMQSSPYGSNMGYSQNMVMMQGGMPR + Oxidation (M)
☑	1406	621.28	1240.54	1240.64	-0.10	0	10	44	1	LDGYSLMIVSK + Oxidation (M)
☑	1861	576.63	1726.87	1726.84	0.03	0	10	42	1	NGNTPLHLAAESNSFR
☑	2343	881.39	2641.14	2641.23	-0.09	1	10	37	1	NEGHVQYAAMQPRVEVGEGDAAAAR + Oxidation (M)
☑	98	520.27	1557.78	1557.92	-0.14	1	10	89	1	VLLKMILIGDSGVGK + Oxidation (M)
☑	207	594.24	1186.46	1186.63	-0.18	1	10	1.2e+02	1	EANLREQLSK
☑	2041	677.36	2029.07	2029.01	0.06	2	10	44	1	LPPRYVSSSSYLSRDMR + Oxidation (M)
☑	2119	1080.61	2159.20	2159.12	0.08	1	10	40	1	GPATMASGVKHTTVFVGNLTR + Oxidation (M)
☑	466	770.83	1539.65	1539.84	-0.19	1	10	1.5e+02	1	SPQRGSVTEIPTLR
☑	785	470.68	939.35	939.45	-0.10	0	10	37	1	MTGLLMSR + 2 Oxidation (M)
☑	265	626.79	1877.34	1876.75	0.58	0	10	1.5e+02	1	MSETESLMCYDPESAK + Carbamidomethyl (C)
☑	448	758.86	1515.70	1515.83	-0.14	2	10	2.2e+02	1	VQLAWTRRIDMK
☑	234	610.39	1828.14	1827.90	0.24	1	10	1.2e+02	1	NEKGDVADITPENASLR
☑	1439	421.56	1261.66	1261.63	0.02	2	10	51	1	LDDLCKNKEK + Carbamidomethyl (C)
☑	1875	586.29	1755.86	1755.97	-0.11	1	10	44	1	SLLAGLRVSCAALETPR
☑	35	392.29	1173.85	1173.51	0.34	0	10	84	1	MMMVDTSSIK + 2 Oxidation (M)
☑	1174	559.36	1116.70	1116.58	0.12	0	10	50	1	TNNLQVVDSK
☑	1227	1147.95	1146.94	1146.57	0.38	1	10	1.4e+02	1	YLKTHNDEK
☑	1254	579.77	1157.53	1157.59	-0.07	0	10	51	1	VEEILDDAVR
☑	2063	692.72	2075.14	2074.90	0.24	0	10	44	1	TCLCSVCGGVMCLSSLQNK + 2 Carbamidomethyl (C); Oxidation (M)
☑	1935	612.87	1835.58	1835.98	-0.41	1	10	63	1	ALRMFPDALPHVGQLR + Oxidation (M)
☑	208	594.57	593.56	593.25	0.31	0	10	21	1	ECNTK
☑	270	628.29	627.28	627.40	-0.11	0	10	33	1	SLLPAK
☑	228	605.12	1812.33	1811.91	0.42	2	10	1.9e+02	1	MSFLYMKSVYRAVFA

☑	615	856.45	1710.89	1710.87	0.02	1	10	1.3e+02	1	EVAQIQVHMCERLR
☑	2169	747.74	2240.20	2240.28	-0.08	1	10	52	1	NGTVLVDKVPFAIQSVLDVVK
☑	1478	647.85	1293.69	1293.65	0.05	0	10	50	1	DAADFRPISFR
☑	938	503.79	1005.57	1005.59	-0.02	0	10	54	1	VLINGYSIK
☑	236	611.55	610.54	610.30	0.25	0	10	1.8e+02	1	ATEYK
☑	534	807.40	806.39	806.43	-0.04	2	10	75	1	KKGMSEK
☑	146	559.97	1117.93	1117.50	0.42	0	10	1.6e+02	1	NHTVFEDEK
☑	229	606.12	605.12	605.32	-0.20	0	10	45	1	ISGAMK
☑	2179	751.05	2250.12	2250.24	-0.12	1	10	49	1	TLFTIDQVIYSVNNKVQIR
☑	1868	874.47	1746.92	1746.81	0.11	1	10	54	1	RITANNAAAATDDAGGCR
☑	78	499.78	1496.32	1496.83	-0.51	1	10	1.3e+02	1	APQSLLQTKNGNVK
☑	1271	584.79	1167.57	1167.64	-0.07	1	10	61	1	FNKVSLSFAR
☑	1625	720.92	1439.83	1439.75	0.08	0	10	51	1	TYEELVPIVYSK
☑	1899	898.95	1795.88	1795.93	-0.04	0	10	51	1	TWVNPQAVPPGEFLNK
☑	546	814.88	813.88	814.43	-0.55	0	10	2.1e+02	1	AGLDVNAR
☑	645	874.35	873.34	873.52	-0.18	1	10	1.9e+02	1	SLRVIMR
☑	1796	548.74	1643.20	1643.77	-0.57	0	10	72	1	MMFINNCIIHHQK + Oxidation (M)
☑	2213	770.00	2306.97	2307.13	-0.16	1	10	46	1	VICPDYVCPFNHGVTAAAFRK
☑	73	491.83	1472.48	1472.92	-0.44	1	9	1.1e+02	1	LLLLQRVLHPGSK
☑	566	413.73	825.45	825.54	-0.10	0	9	36	1	ALIQIIR
☑	1686	755.62	1509.23	1508.80	0.43	1	9	99	1	VENTAFLRTIAMK + Oxidation (M)
☑	232	608.35	607.34	607.34	-0.00	0	9	49	1	AAPRPP
☑	1101	542.82	1083.63	1083.61	0.01	0	9	60	1	ACPVVLEVVR
☑	1378	614.65	1227.30	1227.74	-0.45	0	9	74	1	LVQSVIELLSK
☑	1838	567.17	1698.50	1698.85	-0.35	1	9	68	1	TALMYAAAASRFEAAR
☑	2408	983.79	2948.34	2948.24	0.10	2	9	42	1	HSAPCGMGGQKMPQFCETKCTFTEK + 3 Carbamidomethyl (C); 2 Oxidation (M)
☑	1571	461.23	1380.68	1380.69	-0.01	1	9	51	1	WGKDVGCAFLTGK
☑	1823	561.96	1682.86	1682.91	-0.06	0	9	50	1	ASPACNLIGLTNIQIR
☑	456	762.89	761.88	761.47	0.41	2	9	1.3e+02	1	VRGKFR
☑	1636	725.82	1449.62	1449.64	-0.02	0	9	56	1	IAFYYEHNCYK
☑	1426	627.34	1252.66	1252.75	-0.09	0	9	61	1	NLAVVLIALGDR
☑	861	975.03	974.02	973.57	0.45	1	9	2.6e+02	1	EVAKFKPR
☑	34	382.96	1145.85	1145.57	0.28	0	9	1.8e+02	1	MHSIPHEAPK
☑	1555	1369.71	2737.41	2737.25	0.17	1	9	1.3e+02	1	IPDFPDAMSGWNAVSSFGSYISFRS

☑	1716	514.00	1538.98	1538.73	0.25	2	9	56	1	IDSGMERMRSVAR + 2 Oxidation (M)
☑	2039	1014.35	2026.69	2026.13	0.55	1	9	72	1	GGLLRSATFANLHSVLTAAK
☑	2105	1070.53	2139.04	2139.05	-0.01	2	9	58	1	YLLDPVTCRIWEMGDKK + Carbamidomethyl (C); Oxidation (M)
☑	2223	780.78	2339.31	2339.14	0.17	1	9	54	1	YTLLEICSYQGAANCFKFLR
☑	186	583.83	1165.64	1165.55	0.10	1	9	1.8e+02	1	AASRFAGSEDR
☑	713	910.39	2728.16	2728.28	-0.12	1	9	1.5e+02	1	DAATKNESNCLLMASYYGHLDIVK + Carbamidomethyl (C); Oxidation (M)
☑	2102	710.88	2129.62	2129.11	0.51	2	9	88	1	YSELRRNGTLPWARPDAK
☑	704	906.81	905.80	905.46	0.34	0	9	70	1	VEALFGDR
☑	1094	1079.15	2156.28	2156.12	0.16	2	9	1.9e+02	1	SAEAVLREGLERIEATEQR
☑	1274	390.44	1168.31	1168.58	-0.27	1	9	59	1	CLEYEIKSK + Carbamidomethyl (C)
☑	126	545.01	1088.01	1087.56	0.44	0	9	2.3e+02	1	AGNVLHHANR
☑	620	860.41	859.41	859.48	-0.08	1	9	71	1	MAILLKDG
☑	824	477.94	953.87	953.55	0.32	1	9	60	1	GPKVMVPAR
☑	1375	613.32	1224.63	1224.69	-0.06	1	9	51	1	LTIFSLKMTR + Oxidation (M)
☑	1937	918.91	1835.80	1835.73	0.08	1	9	58	1	THSSSRSPDCCTGDGGR + 2 Carbamidomethyl (C)
☑	1832	847.37	1692.73	1692.87	-0.14	1	9	57	1	VRSDHNVALQAEQAR
☑	271	628.38	1254.74	1254.60	0.14	1	9	1.5e+02	1	AAAMAAYSEARK + Oxidation (M)
☑	1918	605.80	1814.37	1813.96	0.41	0	9	1e+02	1	LLQFINPNSDNITTPK
☑	199	592.89	1775.64	1775.98	-0.34	2	9	2.1e+02	1	YLISVGANKEAKAIDGK
☑	647	876.54	2626.60	2626.47	0.13	2	9	1.9e+02	1	YLAAIGPEPPNEVIGKAVAHVARVR
☑	275	629.91	628.91	628.38	0.53	0	9	2.8e+02	1	VIVGIE
☑	1601	472.93	1415.78	1415.75	0.03	2	9	60	1	GVDSHKDKVYIR
☑	1766	536.95	1607.83	1607.86	-0.03	0	9	59	1	VVVVGSGAVDHTALER
☑	973	510.28	1018.55	1018.54	0.01	0	9	87	1	GSPVTGLFNK
☑	549	816.45	1630.89	1630.90	-0.00	0	9	1.9e+02	1	LFELAPSAAFPVLEK
☑	356	690.35	1378.68	1378.74	-0.05	0	9	2e+02	1	VDIAQWPQLPGR
☑	1041	527.00	1051.98	1051.48	0.50	1	9	1.1e+02	1	NYMEDPKR
☑	38	412.33	1233.96	1233.63	0.33	1	9	1.4e+02	1	SMFHLTGASKR
☑	831	480.21	958.41	958.39	0.02	0	9	71	1	CDICYNTK
☑	147	562.36	1684.07	1683.94	0.13	2	9	1.6e+02	1	RISALSVAQRVAEER
☑	1931	611.96	1832.85	1833.00	-0.15	2	9	61	1	YLISLGANINEKGEKGK
☑	1453	634.82	1267.63	1267.66	-0.03	1	9	56	1	HTLLNDSKANR
☑	481	783.33	1564.64	1564.77	-0.13	1	9	1.7e+02	1	CIMSENIKLHSYK
☑	77	499.34	996.66	996.50	0.16	0	9	1.3e+02	1	MCPVVHVR + Carbamidomethyl (C)

☑	1980	641.35	1921.02	1921.01	0.01	0	9	58	1	LLITPHCGNLTENVIER
☑	2281	830.45	2488.33	2488.10	0.22	1	9	54	1	RVGSHAPHDENMTLAEMYMQR + Oxidation (M)
☑	190	588.33	587.32	587.35	-0.03	0	9	77	1	ITIEI
☑	230	606.69	605.69	605.23	0.46	0	9	71	1	DDGGDK
☑	515	798.96	797.95	797.45	0.50	1	9	97	1	RVEAAPR
☑	2246	793.93	2378.77	2378.20	0.56	2	9	89	1	AARGDDCTIFLVGNKSDLQSIR
☑	396	719.70	1437.38	1437.70	-0.32	0	9	2.2e+02	1	SHPTWVEGGAELR
☑	1262	1164.52	2327.03	2327.15	-0.12	1	9	1.9e+02	1	YRIYCTMECGMPVPLLPVVK + Oxidation (M)
☑	1668	742.64	1483.27	1483.72	-0.45	1	9	1.1e+02	1	HQQMRNGELIDK + Oxidation (M)
☑	1496	1303.17	3906.50	3905.93	0.57	2	9	2.3e+02	1	VIYRSEVCPRLFFFPCSIQGGAGDEAPVLLHGESR + Carbamidomethyl (C)
☑	1658	490.97	1469.88	1469.67	0.20	0	9	64	1	AGLTGIMMESMMAK
☑	1138	549.77	1097.53	1097.62	-0.09	0	9	61	1	NVLQVIEQR
☑	1153	552.74	1103.47	1103.60	-0.13	0	9	82	1	IAIPNSFNTK
☑	343	681.24	1360.46	1360.62	-0.15	0	9	2e+02	1	TMWAYISSQMK + Oxidation (M)
☑	574	415.67	829.32	829.43	-0.11	0	9	82	1	EVPATWK
☑	76	498.54	995.06	995.50	-0.44	1	9	1.7e+02	1	IGDKSFSSR
☑	1858	862.90	1723.78	1723.92	-0.13	0	9	63	1	DMELLLNADSHLLIK
☑	290	639.81	638.80	638.34	0.46	0	8	85	1	FLVNF
☑	571	829.43	1656.84	1656.89	-0.06	2	8	2.2e+02	1	EKEEGVVDEGVVIKK
☑	1827	845.84	1689.66	1689.88	-0.22	1	8	70	1	SGARMLASLISDVQSR
☑	550	817.47	816.47	816.40	0.07	0	8	91	1	VADEEVR
☑	1741	786.19	1570.37	1570.81	-0.43	1	8	1.1e+02	1	DDAVTAIAQVQRER
☑	22	303.18	604.34	604.30	0.04	0	8	1.5e+02	1	AEMVR
☑	2152	738.05	2211.12	2211.06	0.06	0	8	93	1	ILACSNSFEVLELSPSNCTK + Carbamidomethyl (C)
☑	1113	544.72	1087.43	1087.58	-0.15	0	8	88	1	LALSAQGLCR + Carbamidomethyl (C)
☑	1999	656.63	1966.88	1967.06	-0.18	1	8	71	1	LRVAVVGGGPAGACAAETLAK + Carbamidomethyl (C)
☑	1563	689.91	1377.80	1377.78	0.01	1	8	79	1	ITTPNPGAARPKR
☑	1990	976.96	1951.90	1952.14	-0.23	1	8	63	1	LTHSFIIPLSVFVRAPR
☑	799	947.23	2838.66	2838.30	0.36	2	8	2.7e+02	1	VRCCNTTPTCIFSFSAGLFRCGWR + 2 Carbamidomethyl (C)
☑	2325	861.46	2581.36	2581.39	-0.04	0	8	60	1	LPEQSSNAMLPLGPVLPPVVPSAPR + Oxidation (M)
☑	1172	1117.39	1116.38	1116.67	-0.28	1	8	2.3e+02	1	KLYPQSIIR
☑	1578	692.94	1383.87	1383.73	0.14	2	8	68	1	TCGPNKKIQPNK + Carbamidomethyl (C)
☑	1586	696.94	1391.87	1391.81	0.06	0	8	71	1	LDLLQLLPPSQR
☑	1771	538.93	1613.76	1613.79	-0.02	1	8	74	1	RGTSTLFDSMAWVK + Oxidation (M)

☑	1845	851.89	1701.76	1701.87	-0.11	2	8	73	1	LGRLEVPMREAMER + Oxidation (M)
☑	26	352.30	1053.86	1053.63	0.23	1	8	1.8e+02	1	RQLVPNTVK
☑	474	775.17	2322.48	2322.20	0.28	2	8	2.8e+02	1	QGEEANILRQGVGTHGLNSKSK
☑	1093	1079.14	2156.26	2156.02	0.24	2	8	2.3e+02	1	SSVNDETVTKSMKCQIASGR + Oxidation (M)
☑	303	648.83	1295.64	1295.66	-0.03	1	8	3.6e+02	1	FFNNRTDAALK
☑	1157	553.58	1105.15	1105.61	-0.46	0	8	1.3e+02	1	TILNFSGINK
☑	1826	563.93	1688.76	1688.86	-0.10	2	8	74	1	ERAGAAAGETGTLSRSR
☑	1705	763.86	1525.71	1525.73	-0.03	0	8	66	1	NWDMVDHALQGLK
☑	884	493.31	984.61	984.47	0.14	1	8	68	1	HTEARDTR
☑	1583	695.82	1389.63	1389.75	-0.12	0	8	87	1	VVLNENISFIDK
☑	364	694.26	2079.77	2079.87	-0.10	1	8	2.6e+02	1	MYSYFCFSSMQVQDKK + Carbamidomethyl (C); 2 Oxidation (M)
☑	885	985.95	984.95	984.50	0.44	0	8	2.5e+02	1	ELDAVPWR
☑	444	756.13	2265.38	2265.23	0.15	2	8	2.7e+02	1	MLLRSRLFLEAGMAAAAATLK + 2 Oxidation (M)
☑	1260	582.27	1162.52	1162.60	-0.08	1	8	73	1	NNIMVLCRK + Carbamidomethyl (C); Oxidation (M)
☑	669	443.21	884.42	884.47	-0.06	0	8	63	1	LQGDSLPR
☑	112	532.29	1062.57	1062.55	0.02	1	8	1.9e+02	1	EAQERVFGK
☑	1732	783.40	1564.78	1564.78	-0.00	2	8	80	1	NENAVRARFLQMT + Oxidation (M)
☑	239	612.85	1223.69	1223.59	0.10	1	8	2.3e+02	1	TGKGDYAQAGTR
☑	636	435.27	868.52	868.55	-0.04	1	8	58	1	LWPVVKK
☑	1682	753.31	1504.61	1504.81	-0.20	1	8	72	1	VVSPPASSQQPPRR
☑	137	554.67	1107.32	1107.58	-0.26	1	8	1.6e+02	1	RHPDVTEVR
☑	1310	594.07	1186.12	1186.67	-0.55	1	8	1.6e+02	1	IIHDQLHRR
☑	1442	632.49	1262.97	1262.69	0.28	2	8	91	1	IDPFARNRFK
☑	2127	1090.47	2178.93	2179.20	-0.27	0	8	78	1	FVVITVDNVSGQLLHTGVPGK
☑	183	582.65	1744.93	1744.89	0.04	0	8	1.8e+02	1	TEDPANFDALVIEIAK
☑	1323	597.65	1193.28	1192.72	0.56	0	8	87	1	VLYGGFVIVVK
☑	130	550.64	1099.27	1099.47	-0.21	0	8	1.8e+02	1	DLAEMNDHR
☑	2211	769.38	2305.12	2305.14	-0.03	2	8	68	1	LEQGKGVSEVTGSHTYSRQSR
☑	2136	731.92	2192.74	2193.15	-0.41	1	8	95	1	RTIHVLVVVPDGAGGSASDTSR
☑	587	843.50	842.50	842.52	-0.03	2	8	2.4e+02	1	IKKELNV
☑	2386	952.94	2855.80	2855.47	0.33	2	8	96	1	LADSVDATQSHSPPYISKFRPVERR
☑	1883	886.05	1770.10	1770.00	0.10	2	8	77	1	ELDTNKLKTQVLVNR
☑	117	538.34	1074.67	1074.61	0.06	2	8	1.8e+02	1	MISLGRARR + Oxidation (M)
☑	1543	676.42	1350.83	1350.70	0.13	1	8	76	1	VCSAFEKLWGVL

☑	1803	827.93	1653.85	1653.80	0.04	2	8	81	1	RSSFEHGYMQIKR + Oxidation (M)
☑	1193	1128.46	1127.45	1127.67	-0.21	2	8	2.3e+02	1	DGIKVNIKNK
☑	144	558.81	1673.41	1672.89	0.52	2	8	3.5e+02	1	GGDSGVSKIKWAAELR
☑	309	326.13	650.25	650.34	-0.09	1	8	70	1	CTTKAK
☑	662	880.91	1759.81	1759.90	-0.09	2	8	3.4e+02	1	LTNERAQRDLEMLR + Oxidation (M)
☑	1994	654.36	1960.04	1960.05	-0.01	1	8	77	1	RVTFPAQLELIGNEAFR
☑	381	707.07	1412.13	1411.74	0.39	1	8	2.8e+02	1	SFSNVAIFCLRR
☑	2137	732.64	2194.89	2195.02	-0.13	0	8	1.1e+02	1	QVCMCPLAAAIGFTSEAVDR + 2 Carbamidomethyl (C)
☑	845	968.76	967.75	967.55	0.20	0	8	77	1	SGPLPTAVAR
☑	1844	568.23	1701.67	1701.87	-0.20	2	8	86	1	HCLKCNTIKEICIK + Carbamidomethyl (C)
☑	465	770.62	769.61	769.38	0.23	1	8	3e+02	1	NRSEHK
☑	1361	609.80	1217.59	1217.62	-0.03	1	8	93	1	RDPNGNGFLTK
☑	1560	686.35	1370.69	1370.60	0.09	0	8	86	1	VEDLSVEMYDR + Oxidation (M)
☑	734	919.35	2755.01	2754.45	0.57	1	8	2.6e+02	1	LDTYNVRVNLHPEVTASFPVSIQR
☑	2226	783.64	2347.91	2348.27	-0.36	1	8	75	1	ISVTKENQYLTSSVIGLNVQR
☑	1366	611.27	1220.52	1220.72	-0.20	2	8	89	1	RGHSVLLRQR
☑	1758	796.86	1591.70	1591.90	-0.20	1	8	80	1	SQILKVVYIICEK + Carbamidomethyl (C)
☑	1526	669.39	1336.77	1336.79	-0.02	2	8	85	1	ELPVAAAIKRNR
☑	2046	1018.48	2034.95	2035.17	-0.21	0	8	90	1	LLVQHEFNLIIPMLLSR
☑	1822	559.13	1674.38	1673.86	0.52	1	8	1.5e+02	1	TLGVNREATQEEISK
☑	370	699.37	698.37	698.32	0.04	0	8	55	1	SYTSNK
☑	143	558.80	1115.59	1115.60	-0.02	1	7	3.8e+02	1	TRNGGVTAVNK
☑	1600	472.91	1415.71	1415.79	-0.08	0	7	84	1	LVTLTGAVLSEADK
☑	613	428.29	854.56	854.50	0.06	0	7	85	1	VAASAPALR
☑	195	589.52	588.51	588.31	0.20	0	7	1.1e+02	1	DIVDK
☑	537	808.41	807.40	807.45	-0.05	1	7	80	1	EIFSKGK
☑	2369	919.32	2754.93	2755.26	-0.33	0	7	1.1e+02	1	INNYYFLFCNESVNTSTFFSFAK
☑	1505	437.60	1309.78	1309.59	0.18	1	7	77	1	MSHEFFCPKK + Carbamidomethyl (C)
☑	463	768.60	2302.76	2303.25	-0.49	2	7	3e+02	1	MDPAHARLSQVQLVIAAAKER
☑	248	619.10	1236.19	1236.66	-0.47	2	7	3.6e+02	1	GRAGEVYRTTK
☑	174	577.01	576.00	576.27	-0.27	0	7	71	1	GGVGCK + Carbamidomethyl (C)
☑	1652	733.13	1464.24	1464.81	-0.57	2	7	2e+02	1	DRVKQTMSFILK
☑	1588	698.52	1395.02	1394.68	0.34	1	7	97	1	AMSKLVIDMEDK + Oxidation (M)
☑	2100	1064.68	2127.34	2126.91	0.43	1	7	94	1	VCFCATGGGKNDNNNNIDGSK

☑	304	649.41	1945.21	1945.01	0.20	2	7	2.9e+02	1	SHRNYIMEEDLLKAVK
☑	139	555.30	1662.89	1662.77	0.12	0	7	1.9e+02	1	VLESNQQMDQVMNK
☑	2344	881.47	2641.38	2641.16	0.22	0	7	74	1	SIPQCTLHNFPSEINHCCEWAR + Carbamidomethyl (C)
☑	1950	618.84	1853.48	1853.89	-0.40	0	7	1.6e+02	1	LWACSACHGIVHLSCVR
☑	284	638.32	637.31	637.29	0.02	0	7	65	1	AISSSSS
☑	840	484.24	966.47	966.48	-0.02	0	7	82	1	CLSNFLTI + Carbamidomethyl (C)
☑	2338	877.12	2628.33	2628.17	0.15	0	7	75	1	MALHYADEAMLFNSNEYLSHTR + Oxidation (M)
☑	1800	826.16	1650.30	1649.86	0.44	1	7	1.6e+02	1	YTAEEALIDTQIRK
☑	1675	747.33	1492.65	1492.64	0.01	1	7	94	1	ARNVYMYEMER + 2 Oxidation (M)
☑	1843	567.98	1700.92	1700.84	0.08	0	7	99	1	LAFMHAWLADHPHR
☑	2301	844.73	2531.17	2531.24	-0.07	0	7	90	1	SMMLDNLVQQYSSELVVASFVR + Oxidation (M)
☑	387	712.36	1422.70	1422.68	0.03	1	7	2.3e+02	1	GERQAGPPFHGDR
☑	2022	670.34	2007.99	2007.86	0.13	1	7	84	1	EDGKCDLYSGLGDTHEGR + Carbamidomethyl (C)
☑	629	433.72	865.43	865.40	0.03	1	7	1e+02	1	MQSDKNK + Oxidation (M)
☑	1568	691.32	1380.62	1380.72	-0.11	2	7	88	1	KNAEYKIDSVSK
☑	1852	857.47	1712.92	1712.84	0.08	0	7	90	1	TDTNCIDVNEHILVK
☑	499	791.07	2370.20	2370.20	0.00	1	7	3.4e+02	1	RLSNESGGVADVQTLACDAVLPR
☑	167	571.78	1712.31	1711.87	0.44	1	7	3.2e+02	1	DSALLLEDRLGEPER
☑	242	614.09	1226.16	1226.53	-0.38	1	7	3.3e+02	1	MGSSSGSGGSNRK + Oxidation (M)
☑	917	996.91	995.90	996.50	-0.59	1	7	1.2e+02	1	DYSTRLSR
☑	2157	742.13	2223.37	2223.14	0.23	1	7	94	1	NNPGSTPFISASRNGHLEVVK
☑	507	397.23	792.45	792.45	-0.00	0	7	1.1e+02	1	LTLGSFR
☑	591	846.04	845.03	845.46	-0.43	0	7	2.3e+02	1	PSSSSLLR
☑	149	563.34	1686.98	1686.80	0.18	1	7	2.6e+02	1	TKEIITYEEMSAEK + Oxidation (M)
☑	346	681.96	2042.85	2042.86	-0.01	1	7	3.1e+02	1	CGDSRPGSTCRSASCSLDK + 2 Carbamidomethyl (C)
☑	36	396.11	790.20	790.42	-0.22	0	7	2.3e+02	1	TVATGTNK
☑	1247	578.29	1154.56	1154.62	-0.06	0	7	1e+02	1	LPSPLEEGSVK
☑	1706	764.83	1527.64	1527.84	-0.19	2	7	1.1e+02	1	AGNAGAAGTAKELAKAK
☑	2272	823.12	2466.32	2466.14	0.18	1	7	85	1	RGVACYMLVLALLCSSCCMSVR + Carbamidomethyl (C); 2 Oxidation (M)
☑	822	477.71	953.40	953.53	-0.12	0	7	76	1	IISMSLFK + Oxidation (M)
☑	1394	1237.70	1236.70	1236.58	0.12	0	7	2.6e+02	1	EMNLTTQSWK
☑	419	738.64	2212.90	2213.10	-0.19	2	7	3.5e+02	1	DGCNLVRADNNGILKGGSPTR + Carbamidomethyl (C)
☑	658	440.27	878.53	878.54	-0.02	1	7	97	1	LRHNVIK
☑	1021	520.84	1039.67	1039.50	0.16	0	7	98	1	GGAIFSEMTK

☑	397	719.92	2156.73	2156.14	0.59	2	7	4.5e+02	1	HGKIDDYGPVVYVNKVPTR
☑	330	668.78	1335.54	1335.70	-0.16	1	7	3.3e+02	1	TFVETVKEDLR
☑	2145	737.04	2208.11	2208.12	-0.01	2	7	1.3e+02	1	AKAPAPGCPMLAAPSMKSLPDR
☑	846	969.01	968.00	967.48	0.52	0	7	1.7e+02	1	GGSSSAPHIR
☑	1691	757.38	1512.75	1512.83	-0.07	2	7	1e+02	1	EAVELRLEEARAK
☑	1530	670.87	1339.73	1339.77	-0.04	2	7	96	1	EALIDAVARARR
☑	1942	614.51	1840.52	1839.93	0.60	1	7	1.5e+02	1	EVETHMRTIAALQEGR
☑	1503	655.36	1308.70	1308.72	-0.02	0	7	1.1e+02	1	APILHDVFVNGK
☑	2216	772.10	2313.28	2313.24	0.04	2	7	92	1	GGMPIMYKGHMKLLETAIVPK
☑	736	921.89	1841.77	1841.98	-0.21	1	7	3.6e+02	1	TYGWKVIDLLHNLDR
☑	1916	604.91	1811.71	1812.00	-0.29	1	7	95	1	RPLLRTNQAESATLSR
☑	1963	629.02	1884.04	1884.00	0.04	2	7	1e+02	1	QRNPFAIGRSLNAEPSK
☑	519	802.09	1602.16	1601.78	0.39	0	7	4.1e+02	1	IAETDEEEVLIDAR
☑	1939	918.92	1835.83	1835.95	-0.13	0	7	1e+02	1	LTFVSLQEMLPLNGMK + Oxidation (M)
☑	458	765.23	1528.45	1528.91	-0.46	1	7	3.9e+02	1	ELSALSLSKIVEIK
☑	2201	762.80	2285.39	2285.21	0.18	0	7	1.1e+02	1	YPFSIITQYIPNGSLFNALK
☑	1590	702.96	1403.90	1403.71	0.19	2	7	1.1e+02	1	EGLKIEKSDEEK
☑	1846	568.91	1703.71	1703.85	-0.14	1	7	1.1e+02	1	MAEQSLKELDSGIQR
☑	2243	793.42	2377.24	2377.19	0.05	0	7	91	1	FSSEFTAMTVTVLSLFDDVLR
☑	735	461.29	920.57	920.45	0.11	0	6	1.1e+02	1	LFAPGNMR + Oxidation (M)
☑	1152	552.20	1102.39	1102.57	-0.18	0	6	1.2e+02	1	YPRPQTWR
☑	2024	670.58	2008.73	2009.05	-0.32	2	6	1.1e+02	1	NLVKKAIEMFTEISQDK + Oxidation (M)
☑	1546	678.56	1355.11	1355.71	-0.59	1	6	1.8e+02	1	EMVKALMPAPNR
☑	1552	682.87	1363.73	1363.67	0.05	0	6	1.2e+02	1	DDLFNTNASIVR
☑	260	623.76	1868.27	1867.92	0.35	2	6	3.9e+02	1	MRAARDVEGADNPIEPK
☑	261	625.16	624.16	624.33	-0.18	0	6	56	1	AHEIR
☑	1633	724.38	1446.75	1446.86	-0.11	1	6	1.3e+02	1	KVSLLAAASVYLGR
☑	1352	606.84	1211.67	1211.58	0.09	0	6	1.2e+02	1	DQNDQAPSLPK
☑	257	622.11	1242.21	1241.71	0.50	1	6	4.7e+02	1	RLIEVSDAAIR
☑	896	494.93	987.85	987.52	0.32	0	6	1.4e+02	1	GQEVIVSEK
☑	1831	564.99	1691.94	1691.74	0.20	1	6	1.2e+02	1	LCTSWATEEKECHR
☑	91	517.39	1549.16	1548.69	0.47	0	6	3.3e+02	1	NCLITTCMHPICK + Carbamidomethyl (C); Oxidation (M)
☑	110	529.86	528.85	529.29	-0.44	0	6	67	1	TGAGPK
☑	479	781.83	1561.65	1561.74	-0.08	2	6	4.2e+02	1	MRSGGGGGDGGGGRLQK + Oxidation (M)

☑	598	849.23	2544.67	2545.03	-0.36	1	6	4.6e+02	1	MSEYCYMLCALGVSARCCASDR + 2 Carbamidomethyl (C)
☑	93	519.13	1554.36	1554.87	-0.51	2	6	3.3e+02	1	GGNGEEALKIKAQIK
☑	122	544.03	1629.08	1628.81	0.27	2	6	4e+02	1	VSAPATPERCEKWR
☑	170	575.27	574.26	574.31	-0.05	0	6	1.4e+02	1	AYPPK
☑	445	756.90	2267.69	2267.19	0.49	2	6	4.5e+02	1	LRLSLSSRMSEIWSSATLSK + Oxidation (M)
☑	1466	642.73	1283.45	1283.65	-0.20	0	6	1.1e+02	1	GLAMPLSEPDVR
☑	2345	882.74	2645.19	2645.28	-0.09	1	6	94	1	WGEGMGAEFAAKGANVQLGPGLCVAR + Carbamidomethyl (C)
☑	1445	1265.59	2529.16	2529.38	-0.22	1	6	3e+02	1	VLLCTLLNEVSVLLNETKSTQR + Carbamidomethyl (C)
☑	2220	775.71	2324.11	2324.20	-0.08	1	6	1.1e+02	1	ENFLEVSGSVPMELLEKYLK
☑	937	503.77	1005.52	1005.46	0.06	0	6	1.2e+02	1	SDGTAVDDVK
☑	1144	1099.73	2197.45	2197.11	0.34	1	6	3.8e+02	1	EMYNSLGTPISAVATGIKAMK + Oxidation (M)
☑	2313	850.74	2549.20	2549.27	-0.07	1	6	95	1	LQIKEEHENNITNLQNEINEK
☑	338	677.97	2030.88	2030.98	-0.10	0	6	4.4e+02	1	VNALIDNLFTGVFETTYD
☑	1699	762.00	1521.99	1521.70	0.30	0	6	1.2e+02	1	GSSGCIQTIDIDEK + Carbamidomethyl (C)
☑	1454	635.08	1268.15	1268.67	-0.52	1	6	2.3e+02	1	QPNPFLARNGR
☑	2139	733.16	2196.47	2196.03	0.43	2	6	1.6e+02	1	MSCVEDQRVILCPCTICRK
☑	220	601.34	600.34	600.40	-0.06	0	6	1.2e+02	1	TVILR
☑	964	1016.80	3047.37	3047.44	-0.07	1	6	4.2e+02	1	YMSGLKEELNPFTYLNVYFYHFEK + Oxidation (M)
☑	1960	939.94	1877.86	1877.93	-0.07	1	6	1.1e+02	1	NAAHTNFISVSPPHCRK
☑	2480	1158.56	3472.66	3472.88	-0.21	2	6	78	1	YPSNTLLVCDDFAGKGLVSKPGSPLANIITKVR
☑	1307	1184.94	2367.87	2368.19	-0.32	2	6	3.4e+02	1	VKHILVCGHYGCGGAKAALGDSR + Carbamidomethyl (C)
☑	1966	631.54	1891.59	1891.08	0.51	2	6	1.5e+02	1	LSTLAVRRDLHELAAAR
☑	191	588.59	1762.76	1762.80	-0.04	0	6	4.9e+02	1	QFGFSNSELMGDFLR + Oxidation (M)
☑	1385	1232.64	2463.27	2463.22	0.04	1	6	3e+02	1	YRAACVAGPSPPASTLLDYSTPAR
☑	1473	646.87	1291.72	1291.58	0.14	0	6	1.4e+02	1	NVVCENSAATER
☑	2279	829.37	2485.09	2485.29	-0.21	2	6	1.1e+02	1	GEAVVMAEEVTEKPEVLPMRKK + Oxidation (M)
☑	305	649.88	648.87	649.31	-0.44	0	6	2.6e+02	1	TTMGPK + Oxidation (M)
☑	849	485.66	969.31	969.56	-0.26	1	6	1e+02	1	LPKQESLR
☑	876	981.25	1960.49	1960.01	0.48	1	6	4.3e+02	1	TFLTEICFDVAITFGKR
☑	1805	552.38	1654.12	1653.91	0.22	0	6	1.3e+02	1	VPFFTVPLMHPVVR + Oxidation (M)
☑	1383	1231.10	1230.09	1230.65	-0.56	2	6	4.6e+02	1	ENVRKVMEAR
☑	2442	1043.47	3127.39	3127.52	-0.12	1	6	90	1	VKNGPESIYSSENIISELSSQSNSASSVSK
☑	769	932.16	1862.31	1862.02	0.29	2	6	4.3e+02	1	GVWGNKGSVGIRATIYGK
☑	115	538.07	537.06	537.25	-0.19	0	6	4.4e+02	1	YGGNK

☑	1604	709.65	1417.30	1417.66	-0.36	1	6	2.3e+02	1	YYSSTNSTPRSR
☑	730	916.08	1830.14	1829.98	0.17	1	6	5.3e+02	1	MLCVTANLIRGWLQR + Carbamidomethyl (C)
☑	829	479.68	957.34	957.48	-0.14	0	6	1.4e+02	1	ELEPQTNK
☑	193	589.32	1176.63	1176.56	0.07	1	6	4.1e+02	1	EAGMKALNGDR + Oxidation (M)
☑	708	908.60	2722.78	2723.22	-0.43	1	6	4e+02	1	SGIVVDRCEDAWCPTCINELNMAK + Carbamidomethyl (C)
☑	442	752.96	1503.90	1503.77	0.13	1	6	4.5e+02	1	RILSTIGDNMEQK
☑	2381	942.05	2823.12	2823.16	-0.04	0	6	1.1e+02	1	DDALHSLGEFDGETMVDCVCHAMQK + Carbamidomethyl (C); Oxidation (M)
☑	254	621.28	1860.83	1860.91	-0.08	0	6	3.5e+02	1	QEESQMLLSIDVLNDK
☑	522	802.77	2405.29	2405.13	0.16	2	6	4.2e+02	1	NSAVDGNTKAPEAGATAGGPSMMKK + Oxidation (M)
☑	959	1015.85	3044.51	3044.63	-0.11	1	6	4.2e+02	1	QLSTVNEKDSPNLIQAIYTLILSIEDK
☑	1889	594.54	1780.60	1781.04	-0.44	2	6	1.2e+02	1	AVAKLKQLIDGGVDNIK
☑	263	626.48	1876.42	1876.94	-0.52	1	6	4e+02	1	AMEEINMKLNASLEIR + Oxidation (M)
☑	652	877.36	2629.05	2629.19	-0.14	0	6	3.8e+02	1	MFCEQVFDEAQPQTIALEFGNR + Carbamidomethyl (C)
☑	1175	1118.09	3351.25	3351.50	-0.24	0	6	5e+02	1	NESAEFTIVMSCTKPVGFSMNMQVNSSQGR + Carbamidomethyl (C); Oxidation (M)
☑	972	510.27	1018.53	1018.50	0.03	1	6	1.8e+02	1	KSPGDSSSVR
☑	1594	704.80	1407.59	1407.63	-0.04	0	6	1.2e+02	1	MGSSYKPGTYSSK + Oxidation (M)
☑	2292	837.34	2508.98	2508.96	0.02	2	6	1.4e+02	1	RCCCVLEMTEEEDMRGCSR + 4 Carbamidomethyl (C); 2 Oxidation (M)
☑	889	988.15	2961.43	2961.53	-0.11	2	6	4.5e+02	1	RADYTKLDADGLIKPGMPVMGGDILVGK + 2 Oxidation (M)
☑	1968	949.52	1897.02	1896.96	0.06	0	6	1.2e+02	1	SGDAPLPQWVDIFVPEK
☑	743	923.41	922.41	922.43	-0.02	0	6	1.2e+02	1	VAMALGCDK + Oxidation (M)
☑	2043	1017.46	2032.91	2033.06	-0.15	2	6	1.3e+02	1	ESLVVSERDVSAVCKSLR + Carbamidomethyl (C)
☑	2147	737.69	2210.06	2210.03	0.03	1	6	1.2e+02	1	EGCSGNVMKEILGQSELSWK + Oxidation (M)
☑	529	805.08	2412.22	2412.16	0.06	2	6	4.3e+02	1	ADGDIAAAATGTAGSGRSCASPPPRR
☑	649	876.66	1751.31	1751.89	-0.58	1	6	4.8e+02	1	KECINFVVACGFIPR + Carbamidomethyl (C)
☑	1655	490.25	1467.73	1467.80	-0.07	2	6	1.2e+02	1	YREMVRNLFIK
☑	1907	603.33	1806.97	1806.94	0.04	0	6	1.6e+02	1	SNWEPGTTHQVAIVLR
☑	1988	970.45	1938.89	1939.05	-0.16	2	6	1.2e+02	1	LRIEADQLEDERLAIR
☑	1384	616.60	1231.19	1231.73	-0.54	1	6	2.5e+02	1	IAKVVLEFGTR
☑	2164	745.69	2234.04	2233.99	0.05	0	6	1.2e+02	1	SDDALPHGSFVGDAPSSGSFER
☑	2250	798.39	2392.15	2392.06	0.09	1	6	1.1e+02	1	EEMCVRGNVNDNMADITGPVR + Carbamidomethyl (C); Oxidation (M)
☑	2367	914.84	2741.50	2741.56	-0.06	2	6	1e+02	1	QQFQPIIVARIASGALSQAFKDLIK
☑	2285	1247.54	2493.06	2493.14	-0.08	2	6	1.2e+02	1	DLAKAMCRLCNVFNCGEGLDHK + Carbamidomethyl (C)
☑	2324	858.89	2573.65	2574.22	-0.57	1	6	1.5e+02	1	ESGPFLLSPDMIQAACWPDDVKR
☑	2015	997.53	1993.05	1992.81	0.24	0	6	1.2e+02	1	NDMSMLFFDHVEMAMK + 3 Oxidation (M)

☑	960	1016.33	3045.96	3046.41	-0.45	0	6	5e+02	1	YNFMIGETSLHCAFLGGKPDIMNECLK + Oxidation (M)
☑	59	470.79	939.57	939.32	0.25	0	6	2.8e+02	1	DNDMQGSSA + Oxidation (M)
☑	63	480.74	959.46	959.51	-0.05	1	6	3.9e+02	1	GVVETSGRR
☑	333	671.84	670.84	670.36	0.47	0	6	5.9e+02	1	EANLPK
☑	1205	1135.25	1134.24	1134.58	-0.34	1	6	4.2e+02	1	ANFEELRTR
☑	712	909.91	2726.71	2726.23	0.49	1	6	5e+02	1	TYMPGEVMACPHMPGSARQLCFLR + 2 Oxidation (M)
☑	2026	1005.54	2009.08	2009.13	-0.06	1	5	1.3e+02	1	LYILLVASVALFAMRCAR
☑	888	988.03	2961.08	2961.42	-0.34	2	5	6e+02	1	AQMEAATRGLSFDDVDATAEAVPLGDRR
☑	595	847.98	2540.91	2540.37	0.55	2	5	6e+02	1	WKQVADLALEKGMLELAEGALQK
☑	2171	747.82	2240.43	2240.17	0.26	2	5	1.6e+02	1	MLSLTTIKPRDRDCYSITK
☑	1628	722.37	1442.73	1442.77	-0.03	2	5	1.4e+02	1	KEFNALYQFRK
☑	1779	541.27	1620.79	1620.75	0.04	0	5	1.3e+02	1	ALQPSNVFVGCNGCR + Carbamidomethyl (C)
☑	439	750.95	749.94	749.40	0.55	0	5	2.3e+02	1	FIDVEK
☑	2290	836.13	2505.36	2505.24	0.13	0	5	1.2e+02	1	SPIIFCGDIHGQLEDLFELFR + Carbamidomethyl (C)
☑	1105	1085.55	3253.63	3253.72	-0.09	2	5	3.7e+02	1	RHFEYVCPVTVEVVGALPGGCRVVSAVAALR
☑	427	741.78	740.77	740.39	0.39	0	5	1.1e+02	1	SFVFNK
☑	1824	844.85	1687.69	1687.84	-0.15	0	5	1.5e+02	1	VQELEAAAASSAEVASR
☑	2238	789.13	2364.37	2364.26	0.11	2	5	1.2e+02	1	LRPRIMPQNPDSRTVVWGAR + Oxidation (M)
☑	826	478.91	955.81	955.47	0.35	0	5	1.4e+02	1	EFLQEYK
☑	1470	644.81	1287.60	1287.70	-0.10	0	5	1.6e+02	1	DPQLILTFWR
☑	1825	563.83	1688.47	1687.92	0.55	1	5	2.1e+02	1	YHSLGTVGAVRLMLR + Oxidation (M)
☑	264	626.53	625.52	625.29	0.23	0	5	50	1	NGGNHK
☑	124	544.87	1087.73	1087.66	0.07	2	5	4.7e+02	1	ITVAKKAETK
☑	573	829.95	828.94	828.47	0.47	2	5	3.4e+02	1	FNHKRK
☑	2108	715.42	2143.25	2142.91	0.34	0	5	1.2e+02	1	CSHPDIALCPESFNDPER + 2 Carbamidomethyl (C)
☑	564	824.87	823.86	823.38	0.48	0	5	5.2e+02	1	NDSYLGR
☑	903	991.84	990.84	990.53	0.30	0	5	1.8e+02	1	LLTSDISSR
☑	878	983.85	1965.68	1965.99	-0.32	1	5	4.4e+02	1	TLAEAVANGEMTLRTAFR + Oxidation (M)
☑	1074	1070.73	3209.18	3208.62	0.56	0	5	5e+02	1	MGTGTGCLYACAPFTLIVSILLFMLSAMLR + Oxidation (M)
☑	435	746.74	745.73	745.39	0.35	0	5	2.2e+02	1	TYHIGR
☑	770	466.81	931.61	931.47	0.14	1	5	1.8e+02	1	RGGSPTTEK
☑	1540	675.30	1348.60	1348.64	-0.05	0	5	1.4e+02	1	NGPNVDGAMAYIK
☑	1749	528.25	1581.73	1581.82	-0.08	0	5	1.5e+02	1	STNNLQNFGISIFK
☑	241	613.77	612.76	613.32	-0.56	0	5	87	1	THTQK

1860	863.17	1724.32	1724.89	-0.56	0	5	2.6e+02	1	AQETVPTAAAAGLPACVR
1147	550.84	1099.66	1099.56	0.10	1	5	1.6e+02	1	RCFTLAFSR
2483	1164.18	3489.51	3489.46	0.05	1	5	1e+02	1	NSFTNANELTDEEEDEDYMWESRYLFSR
342	680.32	1358.63	1358.68	-0.05	0	5	4.3e+02	1	NGDQSALTALVDR
2374	922.20	2763.57	2763.51	0.06	1	5	1.2e+02	1	TLPTVVDFLEVEEIGVEHLLRDIK
925	500.36	998.70	998.57	0.13	1	5	1.3e+02	1	KISHVPYR
1481	648.10	1294.19	1293.71	0.48	1	5	2.6e+02	1	YVAMNSIKQIK
1850	856.16	1710.30	1710.88	-0.58	2	5	2.3e+02	1	LTEADVYARFRQSR
1788	544.85	1631.54	1631.91	-0.37	2	5	1.5e+02	1	EEIIKYLLNKQIE
1710	764.87	1527.72	1527.68	0.04	0	5	1.6e+02	1	YAGSSTGATTSSPADR
1329	600.33	1198.65	1198.72	-0.07	1	5	1.7e+02	1	FPLEVLRAVR
2328	862.40	2584.18	2584.35	-0.17	2	5	1.2e+02	1	FYFLSDEELLEILSQAKEVRR
1697	760.99	1519.96	1519.77	0.19	0	5	1.5e+02	1	EAAAAAMTTVTAAVSR
1989	973.43	1944.84	1945.02	-0.18	2	5	1.5e+02	1	KMGGTVDVARWPVVSATR + Oxidation (M)
1210	1138.34	2274.67	2275.25	-0.59	2	5	5.1e+02	1	VGDTTTLGILGDTKIRAIEFR
1664	738.57	1475.13	1475.70	-0.58	0	5	2.1e+02	1	VMSLANQTDVNER
524	402.21	802.41	802.49	-0.08	1	5	2e+02	1	LNVKTTK
409	366.18	730.34	730.41	-0.08	1	5	2.2e+02	1	FTHKAK
1570	461.23	1380.65	1380.71	-0.06	2	5	1.4e+02	1	TPRYGIEFRDK
1736	785.22	1568.42	1568.63	-0.21	0	5	2e+02	1	AEIEGMCDALCER + 2 Carbamidomethyl (C); Oxidation (M)
243	614.36	1226.70	1226.49	0.21	0	5	3.9e+02	1	EEMSAMVDQR + 2 Oxidation (M)
225	603.11	602.11	602.28	-0.18	0	5	2e+02	1	MTHSK
1610	713.96	1425.90	1425.59	0.31	0	5	1.4e+02	1	SDFAEDEGEGVSGK
262	625.23	1872.65	1872.95	-0.30	2	5	4e+02	1	AEEGIRAATDKDLDTIR
351	687.35	1372.69	1372.70	-0.01	1	5	5e+02	1	GLWEGGGGMVRVR
731	458.92	915.82	915.51	0.31	1	5	2.1e+02	1	VRADGIGTK
1709	764.86	1527.71	1527.81	-0.10	0	5	1.6e+02	1	LLQSAVCAAPTIER + Carbamidomethyl (C)
1095	1079.22	1078.21	1078.60	-0.39	2	5	4.5e+02	1	KDKEVLGYK
1638	726.67	1451.34	1451.75	-0.41	1	5	2.3e+02	1	VGARFTVSNTSSAR
622	861.77	1721.53	1721.92	-0.39	1	5	5.3e+02	1	VAQATQAAESPVAAPRR
646	875.72	1749.43	1749.90	-0.47	1	5	5.9e+02	1	DFECVIADLIQKQTK
1015	1037.19	3108.55	3108.40	0.15	2	5	4.7e+02	1	FVFHTYRTNGMTTAALSGSECMFKFCK + 2 Oxidation (M)
395	718.87	1435.73	1435.71	0.01	1	5	8e+02	1	SIDPSARVPGYMK + Oxidation (M)
2302	844.80	2531.39	2531.17	0.22	1	5	1.5e+02	1	KAEAATLSSQAMHSTHCSLAEQSK + Oxidation (M)

☑	901	991.02	2970.04	2969.60	0.43	2	5	7.5e+02	1	QEVYKIMEHIHVLLDIAQSVGYKIK + Oxidation (M)
☑	1253	1157.72	3470.13	3470.65	-0.52	2	5	4.9e+02	1	LNMICLEGLTEAKLKAMTWYNGTFGYMQR + Carbamidomethyl (C); 2 Oxidation (M)
☑	131	550.79	1099.56	1099.67	-0.11	2	5	5.3e+02	1	RQEVLSKIK
☑	1369	611.93	1221.85	1221.67	0.18	0	5	1.7e+02	1	HVVEIDELLR
☑	2044	679.18	2034.52	2034.06	0.46	0	5	2.9e+02	1	SQQTINLINDAFIESTLK
☑	1884	886.94	1771.86	1771.80	0.06	0	5	1.4e+02	1	ECLHIEGLHCTFGDAK
☑	363	693.14	692.13	692.31	-0.17	0	5	1.4e+02	1	FMHSR + Oxidation (M)
☑	2418	1002.68	3005.03	3005.45	-0.42	1	5	2.1e+02	1	MALLPAVDAENVGNDSDAQFQTSTTVRR
☑	173	576.87	575.87	576.30	-0.44	1	5	2.5e+02	1	APRAY
☑	353	688.74	2063.21	2063.17	0.04	1	5	5.6e+02	1	MFINELTTGLVKLLTNIK + Oxidation (M)
☑	651	877.23	1752.45	1752.95	-0.50	1	5	6.3e+02	1	QPFGPQGGGKLGPVSLSK
☑	614	856.39	2566.15	2566.45	-0.31	1	5	4.3e+02	1	WGYPLLLAAAVYGALHALTAIARR
☑	1566	1380.54	4138.58	4139.11	-0.52	2	5	4.5e+02	1	NIYSCSKIAAEALIGSIPVNTRQDFLDTICNDIPFLK + Carbamidomethyl (C)
☑	297	643.61	1285.21	1285.54	-0.34	0	5	6.4e+02	1	QDVMVCSAFDR + Oxidation (M)
☑	728	914.04	913.04	913.43	-0.39	0	5	5.6e+02	1	TTSHSPER
☑	539	810.03	1618.04	1617.95	0.09	0	5	5.2e+02	1	FLILLSSIIPISMR + Oxidation (M)
☑	1656	490.25	1467.74	1467.88	-0.13	1	5	1.6e+02	1	IVGSKSGLPQLLTR
☑	2116	720.08	2157.22	2157.03	0.19	1	5	1.5e+02	1	RAVLVGFYMEAQCEALER + Carbamidomethyl (C); Oxidation (M)
☑	21	299.22	894.65	894.48	0.17	1	5	2.4e+02	1	DFASKLSK
☑	2280	829.72	2486.15	2486.04	0.11	2	5	1.3e+02	1	GDQACAPAAQWRRECCGADHER + Carbamidomethyl (C)
☑	965	1016.98	3047.91	3047.61	0.30	1	5	7.7e+02	1	VVVFQQDPLAGPQTPDEATLIAKWTAPR
☑	2339	877.67	2629.98	2629.39	0.59	2	5	1.6e+02	1	NETCFLDISPRDLALKVLGAIER + Carbamidomethyl (C)
☑	217	600.86	599.85	599.38	0.48	0	5	3e+02	1	GVVAVR
☑	569	827.89	1653.76	1653.90	-0.13	2	4	6.9e+02	1	LKAEFGLLREHADR
☑	1768	806.14	1610.26	1609.80	0.46	0	4	2.8e+02	1	NAGHWQAALWTVEK
☑	2274	1236.58	2471.15	2471.29	-0.14	2	4	1.7e+02	1	LRDPQPVVLDMLNALPECHRR
☑	555	820.91	819.91	820.35	-0.44	1	4	3.9e+02	1	CEGNDKR
☑	1433	629.92	1257.82	1257.68	0.14	1	4	1.8e+02	1	LHDKGSLGLYR
☑	610	855.19	1708.36	1708.82	-0.46	2	4	6.2e+02	1	CTVRNCCVEATVARK + Carbamidomethyl (C)
☑	294	641.46	1921.36	1921.92	-0.56	0	4	5e+02	1	HPTISDVYLTCGDVQFK
☑	532	806.74	805.73	805.41	0.32	0	4	2e+02	1	NLMGVTR + Oxidation (M)
☑	1072	1069.37	3205.10	3205.43	-0.33	1	4	5.7e+02	1	MMTCRLLCALLVLALCCCPSVCMAESEDK + Carbamidomethyl (C)
☑	2149	737.91	2210.70	2211.06	-0.36	1	4	3.1e+02	1	GRTGLVCSCVLMGLGICSNAK + 2 Carbamidomethyl (C); Oxidation (M)
☑	1863	578.27	1731.80	1731.85	-0.05	2	4	1.6e+02	1	CNELMVRVRADEAAR

☑	1326	599.81	1197.61	1197.64	-0.02	0	4	1.7e+02	1	HTSAITALQEK
☑	154	565.08	1128.14	1128.69	-0.55	1	4	6.8e+02	1	LSVSAAALAKAK
☑	2269	819.97	2456.89	2457.01	-0.13	1	4	1.9e+02	1	VQCGECRCVYCEECSIAVHR + 3 Carbamidomethyl (C)
☑	1774	539.85	1616.54	1616.94	-0.40	2	4	1.8e+02	1	IFELKEELKDLLK
☑	2267	818.75	2453.22	2453.23	-0.01	2	4	1.4e+02	1	LAKFEFKHLCVLVDQQMTCK + Carbamidomethyl (C); Oxidation (M)
☑	320	658.70	1973.08	1972.88	0.21	1	4	5.8e+02	1	GERHSSMSHTGTSSGSAAAR
☑	1809	830.89	1659.77	1659.87	-0.10	1	4	1.7e+02	1	NPSKTIILFAYYCK
☑	2120	722.45	2164.34	2164.11	0.23	2	4	1.6e+02	1	RSAGRYMKPFLHAFSPGAR + Oxidation (M)
☑	2316	851.71	2552.11	2552.27	-0.15	2	4	1.5e+02	1	WNRVLSPQISKDPWTPEEDQK
☑	440	751.80	750.79	750.34	0.45	0	4	2.7e+02	1	MSCIPK + Carbamidomethyl (C); Oxidation (M)
☑	683	447.27	892.52	892.40	0.12	0	4	1.8e+02	1	MNSIEDGK
☑	1506	1314.15	3939.42	3939.02	0.40	1	4	6.5e+02	1	QDPVDSVPCGNTVGLVGLDQVLIKSGTLSDVEEAFPLK
☑	1411	1243.44	2484.87	2485.33	-0.46	1	4	5.8e+02	1	KEIELITQVIDGTFNEIDPALK
☑	695	901.92	2702.74	2703.32	-0.59	2	4	7.9e+02	1	ISIIPRGRAGGYTQQMQDEAMEPR
☑	579	835.98	834.97	834.39	0.58	0	4	7.6e+02	1	DVEGLMR + Oxidation (M)
☑	52	457.24	912.46	912.56	-0.11	0	4	3.3e+02	1	AIVELQLK
☑	1930	917.42	1832.82	1832.94	-0.12	1	4	1.8e+02	1	QATGRYLLIADCPAWR
☑	140	556.36	1110.70	1110.71	-0.01	2	4	4.2e+02	1	AVIPTKKQVK
☑	1263	583.26	1164.50	1164.70	-0.20	2	4	1.7e+02	1	GLKHKLENVK
☑	1372	612.58	1223.15	1222.61	0.54	0	4	3.3e+02	1	SSCLIHLHEK + Carbamidomethyl (C)
☑	86	513.62	1537.83	1537.74	0.08	0	4	3.6e+02	1	YASPETVTEAAMLR
☑	1258	1161.67	1160.66	1160.59	0.07	0	4	5.1e+02	1	NVETQAVCVAK
☑	1165	1111.23	3330.67	3330.63	0.03	2	4	6e+02	1	QKESQAYQVEVSYTHDAIAVAGAAASPRNGGR
☑	2271	821.74	2462.19	2462.12	0.07	0	4	2e+02	1	NWSAEFVECIAGELGLTHMPDK + Oxidation (M)
☑	134	552.64	551.63	551.30	0.33	0	4	13	1	VISSF
☑	2200	762.41	2284.20	2283.90	0.30	1	4	1.8e+02	1	CGEQCSPRVTVGGEEGCCEER + Carbamidomethyl (C)
☑	633	867.29	2598.85	2598.38	0.46	2	4	5.9e+02	1	GHTVSVPIDVRPGTTTMTVRRFR + Oxidation (M)
☑	1459	1278.79	1277.78	1277.77	0.01	2	4	5.5e+02	1	AIVARVSPPRGR
☑	1135	1095.55	2189.08	2189.14	-0.06	2	4	5.3e+02	1	VDIEYLENERARQVTLNK
☑	1714	513.60	1537.77	1537.56	0.22	0	4	1.9e+02	1	NYDDPEGMHDACR + Oxidation (M)
☑	1953	930.28	1858.56	1858.81	-0.25	1	4	2.5e+02	1	SMNTEMDQNKEYIEK
☑	362	692.95	691.94	691.37	0.57	1	4	6.9e+02	1	QAMSKK
☑	827	958.14	2871.40	2871.58	-0.19	2	4	6.5e+02	1	LLIGIPANSTQSVCRSAAPMLLYRLK + Carbamidomethyl (C)
☑	1557	686.20	1370.38	1370.61	-0.23	2	4	2.2e+02	1	RCEEFKNMNK + Carbamidomethyl (C); Oxidation (M)

☑	1245	1154.82	2307.63	2307.04	0.59	0	4	5.9e+02	1	SAETMESAETIYTSVISESTR + Oxidation (M)
☑	1772	539.11	1614.31	1614.87	-0.56	1	4	3.6e+02	1	LHVMTNLAFLERR + Oxidation (M)
☑	2402	976.71	2927.10	2927.53	-0.43	0	4	1.9e+02	1	TGTSSQAGGAVLPVVTGGVVPTSSGVEIICK + Carbamidomethyl (C)
☑	755	926.71	2777.11	2777.46	-0.36	2	4	6.6e+02	1	CTSPPPAAGKAAGVQGGLAISVEANTVRR
☑	1897	896.38	1790.74	1790.83	-0.10	1	4	2.3e+02	1	MQGSFISKCENYIQK + Oxidation (M)
☑	1297	1179.89	3536.65	3536.77	-0.12	2	4	5.6e+02	1	LNRQAKDWNSFLEAISYFIVSAQNFVYADK
☑	1661	737.29	1472.56	1472.71	-0.15	1	4	2.1e+02	1	LTREAIDSYYDK
☑	2230	784.70	2351.09	2351.27	-0.18	2	4	1.7e+02	1	MKIVVLTGGIACGKSTVADIFR + Carbamidomethyl (C); Oxidation (M)
☑	2410	987.18	2958.51	2958.33	0.18	2	4	1.5e+02	1	FAEMHEIFDDGRGGAGGGVDADRHSVEK
☑	793	943.46	1884.90	1884.90	-0.00	2	4	5.1e+02	1	MAGVPAPADEVRGSCRGR + Carbamidomethyl (C)
☑	477	780.17	2337.49	2337.09	0.40	1	4	7.1e+02	1	GMMSVLVCCPAVAVKGAEAEAR + 2 Carbamidomethyl (C); 2 Oxidation (M)
☑	788	940.93	1879.84	1879.86	-0.02	0	4	6.7e+02	1	VQMMNSQMNLTEAIDR
☑	898	495.28	988.55	988.49	0.06	2	4	2.1e+02	1	EAKNKDER
☑	282	637.85	636.84	637.39	-0.55	0	4	2.7e+02	1	AAHIVK
☑	2192	757.35	2269.02	2269.07	-0.06	0	4	1.9e+02	1	LCETYQTSINLLNNCINEK + Carbamidomethyl (C)
☑	2054	685.02	2052.05	2052.14	-0.09	2	4	1.8e+02	1	IKKVLQGSDCVISLHVEK + Carbamidomethyl (C)
☑	563	412.76	823.51	823.49	0.02	1	4	1.4e+02	1	DRIPVPK
☑	1767	537.60	1609.77	1609.78	-0.01	2	4	1.9e+02	1	TQFLEKEDSEEKK
☑	258	623.16	1866.45	1865.96	0.49	1	4	7.9e+02	1	QLAAAIGYDKATDMLASK
☑	319	657.89	656.88	657.38	-0.50	0	4	4.4e+02	1	GIAELR
☑	1457	1275.45	3823.33	3823.83	-0.51	1	4	6.1e+02	1	TGVTLDCFMQIVQDPKALVAYPGHMPLMGYNWR + Carbamidomethyl (C); Oxidation (M
☑	1536	1348.58	4042.71	4042.96	-0.25	2	4	4.9e+02	1	VVSTKQYPLVYLTSFSTTDCQGKQIFAVNFDDESR + Carbamidomethyl (C)
☑	2111	716.68	2147.02	2147.00	0.03	2	4	1.7e+02	1	RNACIAPWCEDPACEKTIK
☑	1126	1090.94	3269.80	3269.37	0.42	1	4	6.2e+02	1	GNTFLKDNACVNSDGCTGNTYADPVTMTCK + 2 Carbamidomethyl (C); Oxidation (M)
☑	1242	577.50	1152.98	1152.64	0.33	0	4	2.4e+02	1	HVACASLLLAR
☑	2160	1113.13	2224.25	2224.21	0.05	0	4	2.1e+02	1	HNLVRPVLELHCAITAPWR
☑	817	477.26	952.52	952.64	-0.13	1	4	1.8e+02	1	TIIIPRIK
☑	178	580.53	1159.05	1159.57	-0.52	0	4	6.9e+02	1	IWMENINPK + Oxidation (M)
☑	1898	897.06	1792.11	1792.02	0.09	1	4	2.4e+02	1	MFGRFLLAGAILQLAR + Oxidation (M)
☑	772	934.53	2800.57	2800.31	0.26	2	4	6.3e+02	1	DRAVTCMAWNCKENDILAVGYSAVR + Oxidation (M)
☑	1035	1048.67	3142.98	3142.52	0.46	1	4	6.6e+02	1	LVEYNNVSFHKMNIVMFAYAVEHICR + Oxidation (M)
☑	1402	620.33	1238.65	1238.56	0.08	0	4	1.9e+02	1	ELGEQNEHQR
☑	1688	755.85	1509.68	1509.72	-0.05	1	4	2.1e+02	1	KNGGNSELFNSLCK
☑	2265	815.39	2443.16	2443.28	-0.11	2	4	1.9e+02	1	FEQPRRPVNPVFTQSWAEKK

☑	136	554.26	1106.51	1106.61	-0.10	2	4	4e+02	1	GKDKLEVYR
☑	954	1014.11	2026.21	2025.98	0.24	0	4	6.7e+02	1	IFVCDTVSCDGVGVFRPR + Carbamidomethyl (C)
☑	1086	1077.03	1076.02	1076.52	-0.50	1	4	8.6e+02	1	SSSKGGDNGLR
☑	1252	579.25	1156.48	1156.57	-0.10	1	4	2.1e+02	1	HDFDALRQR
☑	113	533.80	1065.58	1065.47	0.12	0	4	6.7e+02	1	NQDVFGCAR + Carbamidomethyl (C)
☑	1244	1154.56	3460.64	3460.71	-0.07	2	4	5e+02	1	SSSVSSSHLAGGDCSSSVVLAARLMQRVAEIGSR + Carbamidomethyl (C)
☑	1983	643.23	1926.66	1926.89	-0.23	1	4	2.1e+02	1	STGFEVNASCGAICDLRK + Carbamidomethyl (C)
☑	1915	604.67	1810.99	1810.99	-0.00	1	4	1.9e+02	1	VLRQGESSDVPVGTIVR
☑	946	1011.17	2020.33	2020.77	-0.44	1	4	5.9e+02	1	NGVCTSCSDGCRSCESATK + 2 Carbamidomethyl (C)
☑	2231	785.86	2354.55	2353.97	0.58	0	4	2.5e+02	1	CLTCQEEGMLDEMAEHWTTK
☑	489	393.19	784.37	784.44	-0.07	0	4	1.8e+02	1	ATPALGQK
☑	741	923.11	922.10	922.52	-0.42	2	4	6.4e+02	1	REVRHAR
☑	1318	1191.10	3570.27	3569.72	0.55	2	4	8.4e+02	1	LDEAMAPAAAPATALFCANSACLEYVPYRTRAR + Carbamidomethyl (C)
☑	1008	1034.47	3100.40	3100.63	-0.23	2	4	6.1e+02	1	TRSPAWSGEKGFVIVSGEGGILTFDLLHK
☑	2094	1061.11	2120.20	2120.03	0.17	0	4	1.9e+02	1	WVSGQLDNSTQSALCVSLR + Carbamidomethyl (C)
☑	1484	1296.45	3886.34	3886.87	-0.53	1	4	6.3e+02	1	HCCFVRQGNGCVLLRPLEGNLTYINDEQEPISR + 2 Carbamidomethyl (C)
☑	2333	874.75	2621.24	2621.33	-0.09	2	4	1.8e+02	1	FACQKYAVEVEMLLKHGAVTEAK + Carbamidomethyl (C)
☑	2346	883.16	2646.45	2646.32	0.13	2	4	1.8e+02	1	ALPLDDDLVNDIEAGRVNPRDDPK
☑	2380	941.81	2822.41	2822.46	-0.06	1	4	1.7e+02	1	IGIGAQMVGIAQGAMDIVMPYLFQRK + Oxidation (M)
☑	1928	916.52	1831.02	1830.97	0.05	1	4	2e+02	1	SCGRITNAGAVALLSSVR + Carbamidomethyl (C)
☑	506	793.34	1584.67	1584.86	-0.19	1	4	6.5e+02	1	SFPKLQADEKPGIR
☑	443	755.42	754.41	754.50	-0.08	0	4	1.3e+02	1	VIQIIGI
☑	1328	1198.89	1197.88	1197.68	0.20	0	4	6.7e+02	1	ALLSLCTPVPK + Carbamidomethyl (C)
☑	2466	1108.69	3323.06	3323.65	-0.59	2	4	2.3e+02	1	DSDSTLRVTVDQAVATISMPSFRMKPPSQK + 2 Oxidation (M)
☑	551	818.58	1635.15	1634.87	0.28	0	4	8.3e+02	1	MASSIVCLIGAVSLQK + Oxidation (M)
☑	1025	1043.39	1042.39	1042.48	-0.10	0	4	6.9e+02	1	GMLDMFGIK + 2 Oxidation (M)
☑	1507	658.30	1314.59	1314.65	-0.07	1	3	2.3e+02	1	EENDNLAKINR
☑	857	974.10	2919.27	2919.36	-0.09	0	3	8.2e+02	1	CPYNPFTPPTYHPLPSPSTPPATMYK + Oxidation (M)
☑	268	627.40	1879.19	1879.00	0.19	0	3	5.8e+02	1	LNADLNHLMLTGDALLR
☑	1354	607.39	1212.77	1212.62	0.16	0	3	2.1e+02	1	HLTCLEELAK + Carbamidomethyl (C)
☑	2085	703.34	2107.00	2107.08	-0.09	1	3	2.1e+02	1	VEMRTGVLLNPNDLEHVR + Oxidation (M)
☑	503	792.83	1583.65	1583.70	-0.05	1	3	8.6e+02	1	SYARGTEEEQQMR
☑	572	829.45	2485.31	2485.23	0.08	2	3	7e+02	1	FAPEMCYTQCQIPIYIPKKR + Carbamidomethyl (C)
☑	1637	725.86	1449.70	1449.64	0.06	0	3	2.2e+02	1	SSGICLECNHLMK + Oxidation (M)

☑	1108	1086.38	2170.75	2171.08	-0.33	2	3	7.4e+02	1	MQQVRDEIAAEVAAAEERR
☑	980	1023.60	2045.18	2045.03	0.15	1	3	6e+02	1	LVSPHTVEDERHSGIVDR
☑	2242	793.09	2376.24	2376.16	0.08	2	3	1.8e+02	1	LNAAQERFSPDDLRMVQASGR + Oxidation (M)
☑	133	551.58	1101.15	1100.59	0.56	1	3	7.3e+02	1	IMDAIKADPK
☑	2327	862.14	2583.40	2583.30	0.09	2	3	1.8e+02	1	YGLRFSEDGKWLDAELDLLLCK
☑	1177	1119.52	1118.51	1118.55	-0.04	1	3	6.1e+02	1	EDGVRWTTR
☑	252	620.43	1858.26	1857.92	0.34	1	3	7e+02	1	AIMDDNLKSFIAFTEK + Oxidation (M)
☑	2159	1113.11	2224.21	2224.08	0.13	2	3	2.4e+02	1	ATLRSAACLNQACRSSSTATR + Carbamidomethyl (C)
☑	2319	854.97	2561.89	2562.26	-0.37	0	3	2.7e+02	1	QYLIEEPEINGTLGHTAAWQHR
☑	2397	964.05	2889.14	2889.32	-0.18	1	3	2.2e+02	1	MPNNDLLKSSISSGNNNDNNNINDASK
☑	2091	705.95	2114.83	2115.06	-0.22	0	3	2e+02	1	LTQELQFPVEITPPSCADK
☑	1230	1149.49	3445.46	3445.55	-0.09	2	3	6e+02	1	GNGCCGCCDAKPEYKLGRPLVSGKTTPCFNDGR
☑	1107	1086.27	3255.78	3255.58	0.20	1	3	7.7e+02	1	VDISSSSRLEEIGFGLFAGCTSLSYISDFR
☑	2199	1142.74	2283.47	2283.11	0.36	2	3	2.6e+02	1	MASQFTQSQIEAKMNEIRR + Oxidation (M)
☑	2459	1095.40	3283.18	3283.49	-0.31	0	3	2.6e+02	1	SGTYTGIFEYHPICMMLAFVMVMPDAVR + Carbamidomethyl (C); 3 Oxidation (M)
☑	1065	1065.50	2128.99	2129.03	-0.04	1	3	6.3e+02	1	AIVDNEYFVKELCTCLR + 2 Carbamidomethyl (C)
☑	315	654.92	1307.83	1307.55	0.28	0	3	6.7e+02	1	HWCYFDQPR + Carbamidomethyl (C)
☑	1200	566.45	1130.89	1130.58	0.31	2	3	2.7e+02	1	KNDASNRGAAK
☑	2407	983.74	2948.19	2948.68	-0.48	0	3	1.9e+02	1	LLAPLLGFHFIAIIVALWYPVGTPCPK
☑	1048	1056.38	3166.13	3166.58	-0.45	2	3	7.4e+02	1	ANARQQLLMATANDLTRGLSVADFCQFR + Carbamidomethyl (C)
☑	804	475.14	948.27	948.47	-0.20	0	3	2.5e+02	1	ADPGLSYAR
☑	2189	1133.91	2265.80	2266.21	-0.41	2	3	2.6e+02	1	DLETLIDQLCPIRGAATRGPK
☑	1951	618.99	1853.95	1853.88	0.07	1	3	2.1e+02	1	GLGNCSSFSVAAAASSRGGR
☑	306	649.88	1946.61	1946.94	-0.33	1	3	1.1e+03	1	EIMECVIAQGADINAKDK
☑	921	997.64	2989.90	2989.45	0.45	1	3	7e+02	1	MNRILSIDTVAHTITCEAGAVMEEVMR
☑	365	694.92	693.91	694.23	-0.32	0	3	3.8e+02	1	CCCPR + 2 Carbamidomethyl (C)
☑	1471	1289.11	2576.20	2576.25	-0.05	1	3	8.4e+02	1	LSSMLKLQNASSMSALMLLCTMR + 3 Oxidation (M)
☑	324	661.45	660.45	660.31	0.14	0	3	4e+02	1	DQAEAK
☑	821	954.29	953.28	953.49	-0.21	2	3	1.8e+02	1	GREHSRGR
☑	997	1031.16	2060.30	2060.01	0.29	2	3	8.4e+02	1	AAKLLRDDDGTDGSGLDSVR
☑	1702	763.03	1524.05	1523.74	0.31	1	3	2.7e+02	1	YCLASRLAEQNEK
☑	905	992.49	1982.96	1982.81	0.15	1	3	8e+02	1	ECPCTREAQIPSCCSSR + 2 Carbamidomethyl (C)
☑	385	712.21	2133.60	2133.09	0.51	2	3	7.6e+02	1	VCLMCGGLNGTVRRVRPMR + Oxidation (M)
☑	2258	806.09	2415.25	2415.14	0.11	2	3	2.1e+02	1	GLVISDTGSGFEEGGRCFRMAAR

☑	784	939.67	2816.00	2816.28	-0.28	1	3	8.7e+02	1	QSAESSPEALMADSESKPSSHRWQR + Oxidation (M)
☑	1345	1209.52	1208.51	1208.54	-0.03	1	3	6.2e+02	1	MPVNSRGCSSR + Oxidation (M)
☑	909	993.96	2978.86	2978.37	0.49	0	3	9.2e+02	1	NMGGSSSGVTAVCVDSTSEHIISGGADGLVR + Oxidation (M)
☑	366	696.15	2085.43	2084.92	0.51	0	3	9.8e+02	1	LDEFATGEQIMGTGSDVDGK + Oxidation (M)
☑	1900	599.77	1796.28	1796.83	-0.55	0	3	3.2e+02	1	SPSQHGGQMLSPSLCPR + Oxidation (M)
☑	1162	554.89	1107.78	1107.65	0.12	1	3	2.4e+02	1	HKSTGRPVVK
☑	2092	707.02	2118.05	2118.07	-0.02	1	3	2.2e+02	1	LKEINNQSSILQSESIGCR
☑	369	698.08	2091.22	2091.04	0.18	1	3	7.9e+02	1	SQANTGTEGEGKLFQLHFK
☑	417	737.57	2209.70	2209.90	-0.20	1	3	9e+02	1	QGLFCFAEFMESERDDDR + Oxidation (M)
☑	462	766.99	2297.95	2298.17	-0.22	1	3	8.3e+02	1	TDEQGHIPSLRHSPAPTNLTK
☑	2082	1052.02	2102.02	2102.06	-0.04	2	3	2.3e+02	1	EGVGDSRPNQLQRFRSEK
☑	2379	939.66	2815.97	2816.30	-0.33	2	3	3.1e+02	1	CSAGSVPGENGCKATEHKYAVPPSWK + 2 Carbamidomethyl (C)
☑	848	485.07	968.13	968.57	-0.44	1	3	2.7e+02	1	KNALLADPK
☑	2348	886.03	2655.07	2655.25	-0.18	2	3	1.9e+02	1	CVSIKDQASTGSVCVKSPTGGWCTK + 2 Carbamidomethyl (C)
☑	383	710.28	1418.55	1418.81	-0.26	1	3	7.4e+02	1	LDKNTSGILLFAK
☑	273	629.24	1256.46	1256.60	-0.14	0	3	7.3e+02	1	ASNNTSAAASPAVP
☑	2488	1189.33	3564.97	3564.46	0.51	2	3	1.7e+02	1	AECAREPCMASGMCTLLNPRISDCASWCCTR + 3 Carbamidomethyl (C); Oxidation (M
☑	245	617.99	1233.97	1233.65	0.32	1	3	9.7e+02	1	FGLQRLEESR
☑	266	626.90	1251.79	1251.65	0.14	2	3	6.6e+02	1	HSGSRNPVSRR
☑	2099	1062.65	2123.29	2123.06	0.23	1	3	2.4e+02	1	HGNPMPVRSISTLSFHDTK
☑	501	791.28	2370.83	2371.19	-0.36	0	3	8.8e+02	1	NVGQVIEEIGVVCEANDIITEK
☑	2366	914.63	2740.88	2740.41	0.47	1	3	3.3e+02	1	VYTNLLQLVSNYEMLLDSKLNDR
☑	2219	775.58	2323.72	2324.10	-0.38	1	3	3.8e+02	1	NNYKNDDVCFSFVPHLVTGR
☑	375	700.30	699.29	699.34	-0.05	0	3	2.2e+02	1	INPDEL
☑	992	1028.95	3083.82	3083.55	0.27	2	3	8.2e+02	1	ENEYSPQSFQWAIDKLQTTTEKTIVK
☑	1050	1057.00	3167.98	3167.63	0.35	2	3	9.9e+02	1	EIALVTQMMNIRGKDFNVSVINSSTAVAK + 2 Oxidation (M)
☑	1069	1067.62	3199.82	3199.36	0.46	1	3	6.8e+02	1	STARSSVCVCVCVCVCVCVCVCVCVR + 2 Carbamidomethyl (C)
☑	65	484.60	1450.78	1450.66	0.12	2	3	5e+02	1	MDKSSKNSNGNNR
☑	246	618.18	617.17	617.34	-0.17	0	3	3.8e+02	1	IVSGDK
☑	1042	1053.32	3156.95	3156.37	0.57	0	3	8.5e+02	1	GGNEIYCGECHLLGDPWICLVCGFVGCSR + Carbamidomethyl (C)
☑	584	839.54	2515.61	2515.23	0.39	2	3	8.2e+02	1	SELTKFAKAHANEVEAACWTPR + Carbamidomethyl (C)
☑	910	332.03	993.07	992.48	0.59	1	3	4.6e+02	1	MCEVLDRK
☑	2376	928.78	2783.32	2783.28	0.04	0	3	2.1e+02	1	FSNYYPISSEAYNIEISSLNEESK
☑	430	742.74	2225.20	2224.99	0.21	0	3	7.3e+02	1	YQPDMSAIFVSLMCCTSLK + Carbamidomethyl (C); 2 Oxidation (M)

☑	2068	1041.98	2081.95	2082.02	-0.07	0	3	2.7e+02	1	YDIIQVSLNSDMLSDIEK
☑	874	980.02	2937.03	2937.40	-0.36	1	3	1.1e+03	1	YIDICSEKGMISEAFYVQVICDGIK + 2 Carbamidomethyl (C)
☑	518	801.16	800.15	800.38	-0.22	0	3	3e+02	1	DQNTPAR
☑	1014	1037.05	3108.12	3107.62	0.50	1	3	1e+03	1	MAMIVPALMAPALMNAPAMPVAGSARVAVPK + 2 Oxidation (M)
☑	902	496.26	990.50	990.46	0.04	0	3	3e+02	1	DESVTVDAR
☑	1269	1167.47	2332.93	2333.02	-0.09	1	3	7.5e+02	1	QALPSELEDCSANETDNERR + Carbamidomethyl (C)
☑	2209	767.15	2298.42	2298.96	-0.54	0	3	2.5e+02	1	CFAAENGYECIFHATGCPPR + 2 Carbamidomethyl (C)
☑	798	946.37	1890.72	1890.95	-0.23	0	3	9.1e+02	1	AEAEYPDPVIPVAAAHNK
☑	1226	1146.41	3436.20	3436.58	-0.38	2	3	9.6e+02	1	NTFGGGGRGYGYGNGTKFYPSSGGGMSFISIGEAK + Oxidation (M)
☑	1207	1135.32	3402.93	3402.57	0.37	2	3	9.3e+02	1	ENQLVEEKQKENQNQTQEEIQNETSEEK
☑	1474	647.27	1292.53	1292.64	-0.11	2	3	2.9e+02	1	EMVNRVRSMR + Oxidation (M)
☑	2028	1007.97	2013.92	2014.10	-0.18	0	3	2.5e+02	1	TPLTGATFVADETVPALLAK
☑	2457	1088.48	3262.42	3262.62	-0.20	2	2	1.9e+02	1	RDFEEWCRPLFDAAVALRDEAIAATGGGVK
☑	432	744.41	743.40	743.36	0.04	2	2	3.6e+02	1	KRYYD
☑	1750	792.85	1583.68	1583.89	-0.21	1	2	2.7e+02	1	NVVVNGLILSEDGKK
☑	393	717.04	1432.07	1431.86	0.22	1	2	1e+03	1	ELLHNVTLRIPK
☑	1189	1126.48	3376.42	3375.84	0.57	0	2	7.4e+02	1	SAFIGQAAQAMLLWLSAVLSAFQIGAIVMLQK
☑	1996	655.24	1962.71	1963.09	-0.38	2	2	3.2e+02	1	FVETASRVFTVDIVPRK
☑	2494	1257.25	3768.74	3768.85	-0.11	0	2	1.6e+02	1	HLFYSAVLLLVVMVCCGSGAAGVAGEPAVSTFEWR + 2 Carbamidomethyl (C); Oxidatio
☑	182	582.51	1744.51	1744.04	0.47	2	2	8.8e+02	1	KVSAKVPIPNLNSIHK
☑	1156	1105.71	3314.12	3314.49	-0.37	1	2	9e+02	1	EGQLHGAEATTEPHAIGRSSTVESDQCYADR
☑	1545	678.10	1354.19	1354.72	-0.53	2	2	5.3e+02	1	FLEVHERNRR
☑	354	688.78	687.77	687.40	0.37	0	2	5.4e+02	1	IPSIMK
☑	554	820.02	1638.03	1637.88	0.15	2	2	1.1e+03	1	TSKVDSKNLSWFVK
☑	825	955.19	954.18	954.60	-0.42	1	2	2.4e+02	1	VIANRAALK
☑	2007	991.13	1980.25	1979.86	0.39	0	2	3.5e+02	1	GSYLNVTAATMEEMYER + Oxidation (M)
☑	494	788.91	2363.71	2363.19	0.51	1	2	1.4e+03	1	ASFMSFISAEVMLLRELLMK + 3 Oxidation (M)
☑	2078	700.09	2097.24	2096.91	0.33	1	2	2.5e+02	1	NSSQWIEDKYTYEFCK + Carbamidomethyl (C)
☑	344	681.54	1361.06	1360.76	0.30	1	2	1.1e+03	1	KYPDIIIESGVK
☑	676	445.20	888.38	888.45	-0.07	0	2	4.3e+02	1	VSQLCPSR
☑	313	653.84	652.83	652.34	0.49	0	2	3.3e+02	1	HVAGNR
☑	559	411.85	821.69	821.43	0.26	0	2	2.6e+02	1	LSAVTMGK + Oxidation (M)
☑	970	1018.65	3052.92	3053.43	-0.51	2	2	9.7e+02	1	DSKEISHFYIGGDGNSARSEEDVSVTVR
☑	401	723.78	722.77	722.29	0.48	0	2	9.3e+02	1	VDSDCK + Carbamidomethyl (C)

☑	1793	820.70	1639.39	1639.80	-0.41	1	2	4.7e+02	1	MPNDKDIVGQPGPTR + Oxidation (M)
☑	2306	1267.31	2532.60	2532.27	0.33	2	2	3e+02	1	MAPLTQRCCGVSASSAIRSSPLAAR
☑	422	740.33	2217.96	2218.02	-0.07	0	2	7.1e+02	1	YLSSSANALAMQGDLSGTAMSK + Oxidation (M)
☑	436	747.06	746.05	746.39	-0.34	0	2	4e+02	1	LGTSAGNK
☑	2299	843.48	2527.43	2527.24	0.19	0	2	2.4e+02	1	CGQSPLGWVFSHFVGAVHHHLGR
☑	1132	1093.24	2184.48	2184.02	0.46	2	2	8.6e+02	1	CEKKEMPNSVPPTPEVDER
☑	1275	1170.08	3507.22	3506.63	0.59	1	2	1e+03	1	TLPLEEFIHKCSNAFGYDNSEQEIYIGDFK
☑	1954	930.30	1858.59	1859.05	-0.45	2	2	3.3e+02	1	VIKLEQCRNIFLQQK
☑	2463	1099.67	3296.00	3295.68	0.32	1	2	2.7e+02	1	TFLITQGETVLKDVMGQFPYEAAEGVPSLR
☑	1179	1119.98	2237.96	2237.37	0.59	1	2	9.6e+02	1	LFLSMLPLILPIVVAVSRTR
☑	680	892.29	2673.85	2674.04	-0.20	2	2	1e+03	1	CPQYCKTCECANSCKVCSEGYK + 4 Carbamidomethyl (C)
☑	1231	1149.65	2297.28	2297.17	0.11	1	2	7.5e+02	1	DDAVLSAIGMEDGSVIHVVRSK
☑	2095	1061.17	2120.33	2120.04	0.29	1	2	2.7e+02	1	LLISKEAGMNDNQGGSALMR + Oxidation (M)
☑	1395	1238.34	3711.99	3711.78	0.21	2	2	8.4e+02	1	ICQQVGTGRIGFQACQQFKDGEGEVIVGMGDGSLK + Carbamidomethyl (C)
☑	2350	896.66	2686.95	2686.40	0.55	1	2	3e+02	1	MCLVEVSKSPKPQASCALPFLPGLR + Oxidation (M)
☑	1770	807.11	1612.20	1612.78	-0.58	1	2	4.2e+02	1	KVTAELGGGDSMFASK + Oxidation (M)
☑	2453	1072.97	3215.89	3215.53	0.36	1	2	2.4e+02	1	NVALCDMGGCVEDAAGKPRNGLLISGVPACR + 2 Carbamidomethyl (C); Oxidation (M
☑	1136	1096.03	3285.07	3285.56	-0.48	0	2	1.2e+03	1	IFNITSSPMVEGGSDCDGLSFPGNPYDLIIK
☑	1278	1171.26	2340.51	2339.95	0.56	2	2	9.2e+02	1	SGCCCARTSSNDNPQNSNRR + 3 Carbamidomethyl (C)
☑	841	967.70	2900.06	2900.55	-0.48	1	2	1e+03	1	GALEIVSPPTFYLGPRECTEVVLVVR + Carbamidomethyl (C)
☑	1208	1135.73	3404.16	3403.71	0.45	2	2	9.9e+02	1	SKFSGWRCAVPHPVTADELKPVDSSHDILR + Carbamidomethyl (C)
☑	660	880.20	2637.59	2638.10	-0.52	0	2	1.1e+03	1	VLMCTYSNPCCFGPGLCFETPDGK + Carbamidomethyl (C)
☑	2373	922.14	2763.40	2763.27	0.13	2	2	2.3e+02	1	KLDGYSCADVTNLCRDAVQMVFDK + Carbamidomethyl (C); Oxidation (M)
☑	1739	785.68	1569.34	1569.80	-0.46	2	2	4.9e+02	1	AEKAKEAGQPVGEEK
☑	301	647.39	1939.16	1938.89	0.27	1	2	9.5e+02	1	RLLDSSMGGSAMSEQQAR + Oxidation (M)
☑	2029	1008.26	2014.50	2013.99	0.51	2	2	4.9e+02	1	QNVGEVRNRQNITDDTR
☑	516	400.70	799.39	799.45	-0.06	1	2	2.8e+02	1	ARGPLMR
☑	1234	1150.07	3447.20	3446.86	0.34	2	2	1.2e+03	1	ELVNSIDNPLHVDSTHSLIKGKIQLFAVTMK
☑	517	800.90	2399.67	2400.06	-0.40	0	2	1.3e+03	1	QEMTELNGVEMPGASIEVYCK + Carbamidomethyl (C); Oxidation (M)
☑	45	432.80	1295.37	1295.62	-0.26	1	2	7e+02	1	RGFENSTSSVGR
☑	2485	1177.96	3530.85	3530.52	0.33	0	2	2e+02	1	VELNETSSESDEEDAFDEEEAPTQQYIDALK
☑	1998	983.63	1965.24	1965.06	0.18	0	2	3.2e+02	1	VAAPSTVLIDDLHLICGTK
☑	2241	792.99	2375.95	2376.20	-0.25	1	2	2.6e+02	1	EDSAASIISSLINKTTDDDIIR
☑	1424	1252.82	2503.63	2504.12	-0.48	0	2	9.3e+02	1	AETALFPGYVACCGDYTHCLVSK + Carbamidomethyl (C)

☑	2359	905.07	2712.18	2712.41	-0.22	2	2	2.3e+02	1	KSVCEPVQTGITAIDAMIPIGRGQR + Carbamidomethyl (C); Oxidation (M)
☑	553	819.33	818.33	818.38	-0.05	0	2	3.9e+02	1	EEDSALR
☑	956	1014.43	2026.85	2026.95	-0.10	1	2	8.6e+02	1	CVFATRNNVPDSLVMCMK
☑	310	651.69	1952.06	1952.00	0.06	0	2	9e+02	1	STAVAAVTGEPPVAGGIGADGR
☑	679	891.91	890.90	890.47	0.43	1	2	6.5e+02	1	MPPFSGKK
☑	725	913.49	2737.45	2737.31	0.14	0	2	7.8e+02	1	AAIKPVEEIDIEDEAEEIDVDPDAL
☑	1160	1107.21	3318.61	3318.61	-0.00	0	2	8.9e+02	1	QTGLMHTLMVPVCASGVHVNADGQPLMAVGGR + Carbamidomethyl (C); Oxidation (M)
☑	2234	786.48	2356.42	2356.22	0.20	2	2	2.7e+02	1	EEQTTKCIADPALSVAARLQR + Carbamidomethyl (C)
☑	2423	1007.00	3017.97	3018.47	-0.50	1	2	3.9e+02	1	GGLSHFYNRTAMLEEVPVNMPVSIADR + Oxidation (M)
☑	2383	949.72	2846.15	2846.40	-0.25	2	2	2.9e+02	1	TFNEGYEICDSYPRQMILPIKVSK + Oxidation (M)
☑	1062	1064.38	2126.75	2127.07	-0.32	0	2	1.1e+03	1	ATELQLVMEANSELLMHAK
☑	1090	1078.39	2154.77	2154.87	-0.11	2	2	1e+03	1	CPNHCKTCNSLSNCTKCDK + Carbamidomethyl (C)
☑	1820	837.47	1672.92	1672.80	0.12	1	2	3.3e+02	1	MNMAVQEYLTAKMK + Oxidation (M)
☑	2377	930.26	2787.75	2787.37	0.38	1	2	3.4e+02	1	VAPQALQNCLQDMPNLAEYLEGKAR + Oxidation (M)
☑	528	804.95	1607.88	1607.76	0.12	0	2	1.4e+03	1	IETYQHSIPYSDR
☑	1332	1203.15	3606.42	3605.82	0.60	2	2	1.2e+03	1	QVISEYIHCLELLPSNEYLVDAFKHSKFNI + Carbamidomethyl (C)
☑	2162	745.26	2232.76	2233.10	-0.33	0	2	3.5e+02	1	FVVGCLMLNGQALQPMHYR + Carbamidomethyl (C)
☑	2493	1247.41	3739.20	3738.94	0.25	1	2	2.4e+02	1	ALTQCAVALLRLFLQHTDVADDAACPLASIHLHR + Carbamidomethyl (C)
☑	1434	1259.14	3774.40	3775.00	-0.60	2	2	1.2e+03	1	KDGFTGFVKGIGTGLTGLVMHPVSGVIDATGSLITASR + Oxidation (M)
☑	2288	834.33	2499.97	2500.34	-0.37	1	2	2.9e+02	1	VCAAIGDMAVNAHLLLMIAKYVK + Carbamidomethyl (C)
☑	628	865.85	2594.54	2594.17	0.37	1	2	1.1e+03	1	AIMNNDLEMFITFTERDEFDK + Oxidation (M)
☑	648	876.61	875.60	875.39	0.22	0	2	1.1e+03	1	IDEEENK
☑	983	1024.67	2047.33	2047.07	0.26	0	2	1e+03	1	YPPNDELSVLSPVLVMFK
☑	1266	583.33	1164.64	1164.61	0.03	1	2	2.9e+02	1	FSNAMKAALGR
☑	1268	1167.42	2332.82	2333.17	-0.35	1	2	1e+03	1	FIDLIPDDGDMELYGPVLRR
☑	2428	1016.43	3046.27	3046.56	-0.29	2	2	2.4e+02	1	EVKLEMKDGSTIVLLSEYLSGCSAFISK
☑	1283	1172.14	1171.14	1170.62	0.51	0	2	1.2e+03	1	NEDIPITTLR
☑	1606	474.60	1420.79	1420.66	0.13	0	2	3.7e+02	1	ESCSVTQSPVTAR + Carbamidomethyl (C)
☑	2491	1213.94	3638.79	3638.94	-0.14	2	2	2e+02	1	SSSINPTPEVLISTATTEPQRSQVTATPSALTVKK
☑	470	773.21	2316.59	2316.24	0.36	2	2	1.4e+03	1	GIDVTGIKGTVSVLNAWCATRR
☑	2337	877.09	2628.26	2628.41	-0.15	2	2	2.6e+02	1	LHGLCRTLVSNLVQGVFQGFERR
☑	1235	1150.63	3448.87	3448.64	0.22	2	2	8.3e+02	1	MGSGDGVEDYINKYQRIPLTSIWMSQYPGK + Oxidation (M)
☑	1382	410.95	1229.82	1229.60	0.22	0	2	3.2e+02	1	WTSLDPEVQR
☑	2125	725.02	2172.04	2172.10	-0.06	2	2	2.9e+02	1	ENAELIQSKQDATQAAKEAK

☑	461	766.48	765.48	765.41	0.06	1	2	2.9e+02	1	SRNYVK
☑	332	671.61	670.60	670.40	0.20	0	2	2.3e+02	1	AATLPAK
☑	541	812.50	1622.99	1622.70	0.29	0	2	8.2e+02	1	VGTLTECGGGGIECDR + Carbamidomethyl (C)
☑	2198	761.43	2281.27	2281.23	0.04	2	2	3.5e+02	1	ANEVIKPIDDKALDGEKWLK
☑	60	471.25	1410.73	1410.78	-0.05	1	2	5.8e+02	1	EANNATPAKALAIK
☑	295	642.30	1923.89	1923.90	-0.00	0	2	7.9e+02	1	YQVENWAQCCLLLER + Carbamidomethyl (C)
☑	1061	532.68	1063.35	1063.55	-0.21	0	2	4.2e+02	1	EFGVVGDLTK
☑	2151	738.04	2211.11	2211.06	0.05	2	2	4.4e+02	1	TAPQEHEKKAVDDFPDGATR
☑	85	513.36	512.35	512.30	0.05	0	2	6.8e+02	1	NAVPI
☑	918	997.13	1992.25	1991.97	0.28	2	2	9.4e+02	1	RKSSGNSDLHTYAAESAAK
☑	952	1013.69	3038.05	3037.68	0.37	0	2	1.1e+03	1	FCQISMLYVVSCVIVPLPLIISVVAFK + Carbamidomethyl (C)
☑	2310	848.76	2543.26	2543.18	0.08	0	2	2.6e+02	1	EVDVGHQMVELQMPCVVSADLR + Carbamidomethyl (C); 2 Oxidation (M)
☑	2426	1013.87	3038.58	3038.19	0.39	0	2	2.7e+02	1	LEYGAQACCPLPNATCCGDVEGHCCPGDR + Carbamidomethyl (C)
☑	1301	1182.65	3544.92	3544.36	0.56	2	2	8.3e+02	1	CYACQTGCLSCTDSTHCQQCSDGFDKRGDK + 5 Carbamidomethyl (C)
☑	659	879.96	878.95	878.45	0.51	1	2	7e+02	1	GLRVMCK + Carbamidomethyl (C); Oxidation (M)
☑	2320	1282.28	2562.54	2562.51	0.03	2	2	3.2e+02	1	YNLLKRNPLALPLILDAMPLLR + Oxidation (M)
☑	187	587.14	1758.40	1758.93	-0.54	0	2	1.4e+03	1	LLEACAFSEIGPQLLR
☑	188	587.22	1172.42	1172.66	-0.24	0	2	1e+03	1	AIPFAVETGLR
☑	1890	892.46	1782.90	1782.95	-0.05	1	2	3e+02	1	QHEPYARAGILYPIR
☑	2086	1054.57	2107.12	2106.93	0.19	0	2	3.2e+02	1	MCANTSASVHVPTVCDLMR + Carbamidomethyl (C); Oxidation (M)
☑	2277	826.33	2475.98	2476.33	-0.35	2	2	2.7e+02	1	GLHCFRVFSIFKGVHFQSLVR
☑	377	705.64	704.63	704.30	0.34	1	2	4e+02	1	AEGEKDG
☑	764	930.54	2788.60	2788.36	0.25	0	2	1.1e+03	1	EEQVQAGAPAYLVQYPQDVIDAQEK
☑	1192	1127.74	3380.20	3379.63	0.58	2	2	1.1e+03	1	RLLCSYCCLPRGPEMSPLPLSPFELPCSAK + Carbamidomethyl (C); Oxidation (M)
☑	1365	1221.25	2440.48	2440.41	0.08	1	2	9.9e+02	1	ELLTQYPVTTRLSLTGPLIVAR
☑	2399	967.60	2899.77	2899.32	0.45	2	2	3.3e+02	1	RQDNDDENAMTLIAAAHGKMQAGDPAK + 2 Oxidation (M)
☑	2263	813.16	2436.47	2436.18	0.29	1	2	3e+02	1	DEDVLAMAPTSFDLLNLSARDK + Oxidation (M)
☑	656	878.77	1755.54	1755.96	-0.43	2	2	1e+03	1	IKELEEEREELLVK
☑	1033	1048.26	2094.51	2094.01	0.51	0	2	1.2e+03	1	QSFAFQPETENDAIALWK
☑	2352	899.50	2695.48	2695.41	0.06	2	2	2.8e+02	1	NILHIACEKGNLNLVKSLIDSGCNK
☑	1483	1295.94	3884.80	3885.04	-0.24	2	2	9.7e+02	1	VLLREIQYMGAPDLRHQPIILLFMACTIEDVSK + Carbamidomethyl (C); Oxidation (M
☑	1632	724.24	1446.47	1446.71	-0.24	0	2	4.1e+02	1	EGASAVTALCQEIR
☑	95	519.25	518.24	518.28	-0.04	1	2	1.4e+02	1	SRIGS
☑	544	813.95	812.94	812.37	0.57	0	2	5.5e+02	1	CAELYSK

☑	612	855.37	1708.72	1708.91	-0.19	1	2	9.5e+02	1	GCTILTFKEDLTLQK
☑	766	930.57	2788.69	2788.16	0.53	0	2	1.1e+03	1	AHHCHTNMNALSLDCLSCYEEQR + 2 Carbamidomethyl (C)
☑	1331	1201.93	2401.84	2402.19	-0.35	2	2	9.7e+02	1	ALFSHAVEINDKAKAIEDMER + Oxidation (M)
☑	1330	1200.23	3597.66	3597.91	-0.26	0	2	1.1e+03	1	SLLLPDHSPTCVSSPLPAIAAAAPATVTPTFTLPNK
☑	296	642.39	1282.77	1282.66	0.11	1	1	8.3e+02	1	TAIDKHADAVSR
☑	1010	1035.00	3101.98	3102.52	-0.54	0	1	1.4e+03	1	QLLGIDYFNAPDISSFDQNVIAEFTASK
☑	1182	1122.21	3363.61	3363.62	-0.00	1	1	1.1e+03	1	GLNTIPGKCLVYDNETGALNAIMDGFMVTFK + 2 Oxidation (M)
☑	1272	1168.66	3502.97	3502.82	0.15	0	1	9.5e+02	1	MLAVGLESSALPAIVVSAAILGAYWLGATSGMPDR + Oxidation (M)
☑	450	759.17	2274.48	2274.95	-0.47	2	1	1.5e+03	1	EYASERTEACKEPSSCGESR + Carbamidomethyl (C)
☑	418	738.15	1474.29	1474.82	-0.53	1	1	1.4e+03	1	APPLSFIKAIMSGK + Oxidation (M)
☑	890	988.24	1974.46	1975.01	-0.55	2	1	1.3e+03	1	GRHVGRSLLHHVDSFCR
☑	1646	730.21	1458.40	1458.75	-0.35	0	1	4.2e+02	1	TNSCVAVVEAGKPK + Carbamidomethyl (C)
☑	1975	637.78	1910.31	1910.11	0.20	2	1	4.2e+02	1	KFIGIKVHSGLLNQQTK
☑	761	927.77	2780.28	2780.35	-0.07	1	1	1.1e+03	1	DIPKMIEFAIIEYEINLNDEDEK
☑	947	1011.45	3031.34	3031.35	-0.02	1	1	8.5e+02	1	MCDQRGSLAVVEGICSNVHDDLQESNR + Carbamidomethyl (C)
☑	1641	727.51	1453.01	1452.73	0.28	0	1	3.4e+02	1	ADPHATTTGGVLGTR
☑	2361	906.34	2716.01	2716.55	-0.54	2	1	3.5e+02	1	LLRSLARHSAAVAATSPTGSLIAAVQR
☑	291	640.50	1918.48	1919.07	-0.59	2	1	1.2e+03	1	VGVVGGKGCGKSTFVNLLK + Carbamidomethyl (C)
☑	405	726.07	725.07	725.39	-0.32	0	1	2.2e+02	1	FYIQR
☑	2069	1042.77	2083.53	2083.20	0.33	2	1	5.5e+02	1	LLTLGFLRLNISTHSSRR
☑	908	993.84	992.84	992.47	0.37	0	1	3.5e+02	1	VHFYGQDK
☑	1068	1067.13	1066.12	1065.60	0.51	2	1	1.1e+03	1	QLDRHAKAK
☑	496	789.72	1577.42	1577.66	-0.24	1	1	1.2e+03	1	FDEKEMESQYEK + Oxidation (M)
☑	89	516.27	1030.52	1030.58	-0.06	1	1	8.7e+02	1	DNKAPVLFK
☑	955	1014.32	3039.95	3040.49	-0.54	2	1	1.3e+03	1	VWCVRWCPTALVLASCSGDTTVKFWGR
☑	1325	1196.28	2390.55	2390.18	0.37	1	1	9.7e+02	1	NGNTAGAEGRRPPHSQTAGQGTVK
☑	335	673.86	672.85	672.36	0.49	0	1	9.4e+02	1	DGAVVGR
☑	1141	1098.77	2195.52	2195.94	-0.43	2	1	1.3e+03	1	MAEEEEEARRMAEEEAVR + 2 Oxidation (M)
☑	1259	1162.18	2322.34	2321.96	0.38	0	1	1.2e+03	1	GECINTIIEYCEEQNGYGCK + Carbamidomethyl (C)
☑	276	629.98	1886.91	1886.87	0.04	1	1	1.5e+03	1	ARCDVDASHMVVQDLR + Carbamidomethyl (C); Oxidation (M)
☑	523	803.38	2407.10	2407.27	-0.16	2	1	1.3e+03	1	KQNEMIIGTFFTIQNVPARGK + Oxidation (M)
☑	710	909.23	1816.44	1816.00	0.44	1	1	1.3e+03	1	VGMAFVNASVQPLTRVK
☑	1143	1099.46	3295.37	3295.64	-0.27	2	1	9.7e+02	1	ELEVYTQPEVPPDLSRIKWIPNEGMPCR
☑	617	857.71	1713.40	1713.78	-0.37	1	1	1.2e+03	1	AREQHGTAASGDASSGGR

☑	1073	1069.83	2137.64	2137.99	-0.35	1	1	1.1e+03	1	HIMRYISAVSGDTEEMQR + Oxidation (M)
☑	1170	1116.52	3346.53	3346.71	-0.18	2	1	9.7e+02	1	ISMYLMDQRALVLSVFAAGNTAMVAYIPAKA + 2 Oxidation (M)
☑	2032	1009.68	2017.34	2016.96	0.38	2	1	4.4e+02	1	LWMAPDPADRSMASSRAR
☑	2215	1156.77	2311.53	2311.17	0.36	2	1	5e+02	1	HKNVFTNDNDRVSPLTEVAR
☑	545	814.09	813.09	813.45	-0.36	1	1	4.1e+02	1	PVSAERR
☑	1051	1058.11	1057.11	1056.56	0.55	0	1	1.2e+03	1	EEQAAQLLR
☑	2237	789.11	2364.32	2364.36	-0.04	0	1	3.1e+02	1	TLMWVLLLNFCIIVPHALIR
☑	894	988.73	2963.16	2962.56	0.60	1	1	1.3e+03	1	VPLHCPLVEVLVPMPRAYESVLSQMR
☑	1421	1251.48	1250.47	1250.68	-0.22	2	1	9.7e+02	1	KPDDRHSLKR
☑	1947	924.44	1846.87	1847.01	-0.14	2	1	3.8e+02	1	RLTIKHPSGAADQGLQR
☑	467	771.67	2312.00	2312.20	-0.20	2	1	1.3e+03	1	FVKNDLWHRVIVSGAGGGADSK
☑	1364	1221.10	2440.18	2440.26	-0.08	2	1	1.3e+03	1	MKIQAALAVTALAMADAASFRMK + 2 Oxidation (M)
☑	2070	696.21	2085.61	2085.99	-0.38	2	1	5.5e+02	1	AFMASNAPADAQRHGRQSR + Oxidation (M)
☑	2224	781.06	2340.16	2340.39	-0.23	2	1	3e+02	1	LSITLKPLHKYKINFPTSIK
☑	1241	577.32	1152.63	1152.60	0.03	0	1	3.1e+02	1	VWVEISAGHR
☑	913	498.20	994.39	994.47	-0.08	1	1	3.2e+02	1	CMREAITR + Oxidation (M)
☑	1489	433.99	1298.96	1298.58	0.38	1	1	3.8e+02	1	SMAAVGMSKDMR + Oxidation (M)
☑	1909	905.79	1809.57	1809.78	-0.21	0	1	4.3e+02	1	MSADADASPTDPFLNSR + Oxidation (M)
☑	194	589.50	588.49	588.30	0.19	0	1	4.7e+02	1	WAANK
☑	668	884.16	883.15	883.59	-0.44	0	1	1.1e+03	1	ILSAVLLR
☑	819	953.73	952.72	952.45	0.27	0	1	1.3e+03	1	ISDDYNVK
☑	2388	953.36	2857.04	2857.36	-0.32	1	1	3.8e+02	1	GLEYTASWSAGNVACDDVLEAAAAFAKK
☑	1324	1195.40	2388.80	2389.02	-0.22	2	1	1.1e+03	1	CSGRDCVWASGDYGSSLEERR + Carbamidomethyl (C)
☑	1914	906.48	1810.94	1810.80	0.13	2	1	3.5e+02	1	RSNCCPTCRYEIPR + 2 Carbamidomethyl (C)
☑	862	975.18	1948.34	1948.90	-0.55	0	1	1.3e+03	1	GFFFFDASSGAAAVQAGDGK
☑	981	1023.70	1022.69	1022.52	0.17	1	1	1.2e+03	1	TFKQWADK
☑	1024	1043.06	3126.15	3126.49	-0.34	1	1	1.4e+03	1	MNDHDAVVMSGTALKHVTCGTVDDVTVVR + Carbamidomethyl (C)
☑	851	971.35	1940.68	1940.95	-0.27	1	1	1.2e+03	1	IGEFITRIDFNTSDEGK
☑	105	525.58	1049.14	1049.59	-0.45	0	1	9.3e+02	1	LFTVAVACVK
☑	813	951.60	950.59	950.47	0.12	0	1	4.7e+02	1	MSLADISSK
☑	358	692.31	2073.90	2074.10	-0.20	0	1	1.1e+03	1	ALALVVLSCWACLGLDVSK + 2 Carbamidomethyl (C)
☑	2002	988.32	1974.63	1975.10	-0.46	2	1	4.5e+02	1	SPTEHVKRQQLTVPSIR
☑	866	975.53	974.52	974.56	-0.04	2	1	5.3e+02	1	RSATSVAKR
☑	1216	1140.36	1139.35	1139.52	-0.17	0	1	3.5e+02	1	CEELMSAITK + Oxidation (M)

☑	2004	660.20	1977.58	1977.08	0.50	1	1	5.3e+02	1	LPTDELKLEFLDYLIR
☑	1317	1190.65	2379.29	2379.04	0.25	1	1	1e+03	1	MDGASLQQWGELMKESHASCR + Oxidation (M)
☑	794	944.72	1887.43	1886.92	0.51	0	1	1.5e+03	1	YGDFSSVSLLLQTCAAR + Carbamidomethyl (C)
☑	2133	730.70	2189.06	2188.98	0.09	2	1	3.6e+02	1	KECGSVGGEPPRNSDNSANGSK
☑	2235	787.24	2358.69	2358.17	0.52	0	1	5.8e+02	1	ILSIAMNNHGIAATIGSDEMIR + 2 Oxidation (M)
☑	536	808.40	2422.19	2422.29	-0.10	1	1	1.1e+03	1	LLFFNYIQQSINNIPSSSKIP
☑	1343	1205.96	2409.91	2410.06	-0.15	1	1	1.1e+03	1	DCMVCWDAPAPHPAEADGRVVR + Oxidation (M)
☑	510	797.18	2388.51	2388.09	0.42	1	1	1.4e+03	1	AHGGPGCSSSFTALPRGEQDTGTR
☑	693	900.46	2698.36	2698.36	-0.01	2	1	1e+03	1	CPAIRVMATNKAHELLGGSGSASVTR + Carbamidomethyl (C); Oxidation (M)
☑	1660	737.08	1472.15	1472.67	-0.52	2	1	6.2e+02	1	RECHCGIGGGGKSR + Carbamidomethyl (C)
☑	644	873.36	872.35	872.51	-0.16	2	1	1.3e+03	1	AKGGKSTPK
☑	1270	1167.87	1166.87	1166.54	0.32	1	1	1.2e+03	1	SPMKTDVMAR + 2 Oxidation (M)
☑	1419	1249.21	3744.61	3744.50	0.11	2	1	1.4e+03	1	TCFDGRTNSFCTGYGSCALDSETQTYKCNCNK + 4 Carbamidomethyl (C)
☑	1447	1265.89	3794.64	3794.83	-0.20	1	1	1.2e+03	1	HAAAEEARATASGADGDTEETLNIVGLQWLSEEVVR
☑	2128	727.66	2179.96	2180.23	-0.27	1	1	3.6e+02	1	FLLEVVDAVAAAIEPGRVGVR
☑	1060	1063.06	3186.16	3185.75	0.41	1	1	1.6e+03	1	DPSLSLPKLPVALSTSPASLDTLPNISSPLR
☑	1257	1160.73	3479.16	3479.52	-0.37	2	1	1.2e+03	1	SCHTMGSAMGAVCGIPFMGENEVKGRTTGYMK + 2 Carbamidomethyl (C); Oxidation (
☑	1878	881.76	1761.51	1761.78	-0.27	1	1	4.9e+02	1	YDDGDYEGADFRVLK
☑	744	462.22	922.42	922.51	-0.09	0	1	3.5e+02	1	AEVPPPVSK
☑	2248	795.29	2382.85	2382.35	0.50	1	1	4.4e+02	1	KDVSDSLALQKPTLLNTATVLR
☑	531	806.11	2415.31	2414.97	0.34	0	1	1.3e+03	1	THCETSCMSESGCTVAMAAQVR + 2 Carbamidomethyl (C)
☑	2363	910.21	2727.61	2727.29	0.33	0	1	3.3e+02	1	LAGFTGFHLSIGMEGLCVTTESSNEK
☑	357	691.73	690.72	690.39	0.33	1	1	5.7e+02	1	DITSKK
☑	1728	780.28	1558.55	1558.82	-0.28	1	1	3.8e+02	1	LMQASQRIAELQR + Oxidation (M)
☑	2244	1189.89	2377.78	2378.20	-0.43	1	1	5.1e+02	1	TCSMSLEKVSTIVGIMLELHR + 2 Oxidation (M)
☑	2270	821.67	2461.98	2462.18	-0.20	1	1	4.3e+02	1	TPDHTKNALGALQPDAEEAGGSQR
☑	996	1030.73	3089.17	3088.66	0.51	1	1	1.4e+03	1	LLVVCGWTLILDIVETLCGMRGIPYVR + Carbamidomethyl (C)
☑	2188	756.05	2265.14	2265.13	0.01	1	1	4.3e+02	1	KSSVCVCTPSVVAVVTASVGCR + 2 Carbamidomethyl (C)
☑	1131	546.86	1091.71	1091.46	0.25	0	1	4.1e+02	1	CYGDFTTASK
☑	2464	1100.58	3298.72	3298.57	0.16	1	1	2.8e+02	1	AAIPALTSVWTCDNFCAHKGVECTEAGLHVR
☑	163	569.67	568.66	568.29	0.38	0	1	97	1	LEPNP
☑	2392	960.01	2877.01	2877.43	-0.42	2	1	5e+02	1	VDGDTLMRLTAHDLYYMGVPLRDAR
☑	2049	1019.03	2036.05	2036.03	0.02	0	1	3.5e+02	1	TLVDAAAVAAFIQEHHDTK
☑	1006	1032.89	3095.66	3095.63	0.03	2	1	1.3e+03	1	HRPAKCFMAVLLPLGDVAIFGTPMPARR + 2 Oxidation (M)

☑	859	974.20	2919.57	2919.28	0.29	0	1	1.4e+03	1	AAFQACCAWSVEAVLDGCMFDQLAAAR + Carbamidomethyl (C); Oxidation (M)
☑	2334	1313.39	2624.78	2624.39	0.39	1	1	5.2e+02	1	VICMIDLVIGYTAIQTMAIWARK + Oxidation (M)
☑	2191	757.04	2268.09	2268.27	-0.18	1	1	3.9e+02	1	VVLLFTQSDVTSSLYLSIRK
☑	403	363.46	724.91	725.30	-0.39	0	1	4.4e+02	1	GDSYER
☑	1381	615.72	1229.42	1229.70	-0.29	2	1	4e+02	1	IQRQLASMKR
☑	1519	663.93	1325.85	1325.75	0.10	0	1	3.7e+02	1	TVVPIHLGCFLK
☑	1992	979.92	1957.84	1958.12	-0.29	2	1	3.6e+02	1	RKPPAKIVWHATGIADAK
☑	2247	794.39	2380.16	2380.30	-0.14	1	1	3.4e+02	1	VVELDHHMHAISLREQLIIK
☑	2484	1172.86	3515.56	3515.71	-0.14	1	1	2.8e+02	1	CMIAGVLSCGLTHTAITPLDVTKCNMQINPGK + 3 Carbamidomethyl (C); Oxidation (
☑	1392	1235.58	2469.15	2468.93	0.22	0	1	1e+03	1	MVDDDFSDDYDDYDEEEKPK
☑	1518	1326.12	3975.34	3975.83	-0.49	2	1	1.3e+03	1	CLKNLKPVTWGTANCHEFEEAWMICRAYETYR + 2 Carbamidomethyl (C)
☑	484	784.21	2349.61	2349.11	0.50	0	1	1.4e+03	1	LHLYDCISTMADAVGGALAEAR + Carbamidomethyl (C); Oxidation (M)
☑	1309	1186.80	3557.38	3556.84	0.53	2	1	1.3e+03	1	ERRRPLDDPAITVFELQNHYTANELSVFVK
☑	1362	1220.49	3658.44	3658.92	-0.47	1	1	1.3e+03	1	ATIEAELPVLLGKVELLCNQLPSDLYYADDVVR
☑	928	1000.86	2999.55	2999.63	-0.09	1	1	1.3e+03	1	IPLVLLLWCASVLLCDVQSAAFVEKR + 2 Carbamidomethyl (C)
☑	2416	1000.91	2999.70	2999.41	0.29	1	1	4e+02	1	TTTRHEDEGSPASPSSLSTATAAVVAGEDR
☑	1388	1233.31	2464.61	2465.19	-0.58	2	1	1.4e+03	1	ITKPDKFCNCLERFFNEFSK
☑	1089	1078.13	3231.37	3231.54	-0.17	2	1	1.5e+03	1	VVQQVAEHGFNCFRCPISCDLIHKWMR + Oxidation (M)
☑	2332	873.95	2618.82	2619.22	-0.40	1	1	5.6e+02	1	ESDADEWGLLLVKGEVSGEEESNK
☑	2089	1057.83	2113.65	2113.10	0.54	2	1	5.5e+02	1	VCENIPIVLVGNKCDVKDR
☑	945	1010.66	3028.96	3029.46	-0.50	2	1	1.3e+03	1	RAQTAMNSIGLDMDPYEDLSPPDPKIR
☑	1196	1129.95	3386.83	3386.71	0.11	2	1	1.3e+03	1	DVVITYDYTACIHQNILHKEDGMVGVRLGK
☑	1204	1134.19	3399.56	3399.56	0.00	2	1	1.4e+03	1	CMGLYGERVGCLHAVTSSPQEAKCVNSQFGR + Carbamidomethyl (C); Oxidation (M)
☑	2391	959.67	2875.98	2876.45	-0.47	1	1	5.4e+02	1	EAFDAMVEQIVVSRIVAEEDEILQK + Oxidation (M)
☑	1238	1151.77	3452.29	3452.69	-0.40	1	1	1.4e+03	1	EPQGQLTLAKDEYGVVCTAHSLPGVLVHCMK + 2 Carbamidomethyl (C); Oxidation (M
☑	2071	1044.27	2086.52	2086.02	0.50	2	1	6.4e+02	1	DGDILKIGDFGYASMAKNR + Oxidation (M)
☑	998	1031.46	3091.36	3091.43	-0.07	2	1	1.2e+03	1	LEQCSGDWDDSSAQKQKGTQVVEPPFR + Carbamidomethyl (C)
☑	1084	1076.90	2151.79	2151.95	-0.16	2	1	1.2e+03	1	NYERGSRYSSDILAMCCK + Carbamidomethyl (C)
☑	2135	1097.14	2192.26	2191.93	0.33	2	1	3.6e+02	1	RMEGAVRDEMDMEEDVHK + Oxidation (M)
☑	763	928.82	2783.44	2783.51	-0.08	0	1	1.2e+03	1	GESLILSIAALLQQIVEVSETQGMVR
☑	810	950.99	2849.95	2849.38	0.57	1	1	1.9e+03	1	FSYRPLPMCVPKMTPSVSNTAHSSR + Carbamidomethyl (C)
☑	2372	921.58	2761.72	2762.28	-0.56	2	1	4.4e+02	1	GCFTADGEGSVKQGPGNAADQGKWNLR
☑	802	948.27	1894.52	1894.94	-0.42	0	1	1.5e+03	1	LEAPGTQSAVAPSGSEAPAR
☑	844	968.37	2902.08	2902.38	-0.30	1	1	1.3e+03	1	MFHRAFVSSSDLTGCTIALSSVCTQK + 2 Carbamidomethyl (C)

☑	1974	956.16	1910.31	1909.91	0.40	0	1	5e+02	1	SSIAVAASPLCCTSLCTAR + Carbamidomethyl (C)
☑	1040	1051.64	3151.90	3152.26	-0.36	1	1	1.2e+03	1	KEGNNCNSHTCTSCTTGYFLYMGGCYK + 3 Carbamidomethyl (C)
☑	1542	676.25	1350.49	1350.70	-0.22	0	1	3.9e+02	1	LTGLESVSPSAYK
☑	2208	767.14	2298.39	2298.09	0.30	2	1	3.8e+02	1	VWPDGNPNTYKVNHWRCR + Carbamidomethyl (C)
☑	933	1004.45	2006.88	2006.84	0.04	0	1	1.2e+03	1	GVSSSSSSSTAASSTSSAGCR + Carbamidomethyl (C)
☑	1190	1127.12	3378.33	3378.71	-0.38	2	1	1.5e+03	1	VLLQEEMDRLVQTLVSQAVGCRSYNVMPK + Carbamidomethyl (C); Oxidation (M)
☑	2308	847.93	2540.77	2541.30	-0.53	2	1	5.8e+02	1	LMQGRSGGIVEASAEVAHMMAKIR
☑	404	725.99	2174.95	2174.87	0.08	0	1	1.1e+03	1	GAAAVPEAVECGNGEDNDGGSGEA
☑	2477	1135.72	3404.14	3403.76	0.38	1	1	4.7e+02	1	DTNGSCGAAYRPIQEITAASPQFLKLLNQIK + Carbamidomethyl (C)
☑	521	802.46	2404.37	2404.20	0.17	2	1	1.4e+03	1	WLFMMCGPVQFRRLQLYK + Carbamidomethyl (C); 2 Oxidation (M)
☑	2471	1121.70	3362.08	3361.72	0.37	2	1	4.5e+02	1	RAPVSCGALVRTCNLSGSAPNIICLLGGPGSHK + 2 Carbamidomethyl (C)
☑	1002	1031.96	3092.85	3092.64	0.21	0	1	1.6e+03	1	VNPHERPNITEILATPIMLNEGLQHLK + Oxidation (M)
☑	2011	995.85	1989.69	1989.98	-0.29	0	1	4.5e+02	1	SLTDFMGQGVPEEIAQLR
☑	2218	774.53	2320.57	2321.13	-0.56	2	1	5.7e+02	1	EERGECDELVDIQVQYKLR
☑	54	462.71	1385.11	1384.62	0.49	1	1	1e+03	1	MMSTHSNKYVR + 2 Oxidation (M)
☑	957	1015.26	2028.50	2028.98	-0.47	0	1	1.6e+03	1	VLYSEVCMILSCAPLMK + 2 Carbamidomethyl (C); Oxidation (M)
☑	860	974.45	973.44	973.53	-0.08	0	1	5.1e+02	1	DMILTIPR + Oxidation (M)
☑	638	869.88	1737.75	1737.84	-0.10	1	1	1.3e+03	1	YIIGEQNGGFKANGDR
☑	407	729.96	2186.86	2187.10	-0.25	1	0	1.9e+03	1	IINVWAVTKDAAMTNYYSK
☑	1243	1154.55	3460.64	3460.83	-0.19	2	0	1.1e+03	1	NDGMYTVANGRMGQKPQLSIPRVPARPPVGVR
☑	1273	584.89	1167.77	1167.54	0.24	0	0	5.1e+02	1	QMVTNSGMLR + 2 Oxidation (M)
☑	2170	747.76	2240.27	2240.19	0.08	0	0	4.5e+02	1	MLNSELLSSFHATIATIPAPK
☑	1593	704.30	1406.59	1406.68	-0.09	1	0	4.4e+02	1	EQTMAERQASLK + Oxidation (M)
☑	1129	1092.37	3274.07	3274.65	-0.57	2	0	1.5e+03	1	VDASTAMVLAAAPKLTGTPRSTSTDGAAAAATER + Oxidation (M)
☑	1292	1177.24	3528.69	3528.65	0.03	2	0	1.3e+03	1	TPLMCLAASRGTDVGFAALTRAGAEFDAVDGCGR + 2 Carbamidomethyl (C); Oxidatior
☑	2303	844.86	2531.56	2531.38	0.18	2	0	4.7e+02	1	GPSLASLKSIARNTIDGMFAIIEK
☑	1211	1139.08	3414.22	3414.60	-0.38	2	0	1.7e+03	1	VEELDDSDNPIYHDKIESLQKTIEDVENQ
☑	717	911.40	2731.18	2731.52	-0.34	1	0	1.2e+03	1	VKALIMDLIHNIEVVDALIDAGIEK
☑	835	964.02	1926.03	1925.90	0.13	0	0	1.9e+03	1	LLFMTDCCLEAVAEAAAR
☑	322	659.25	658.24	658.24	0.00	0	0	6.2e+02	1	DCDHGI
☑	1166	1111.65	2221.28	2221.11	0.17	0	0	1.3e+03	1	TCLLMLNYVAGPLTLENDNK
☑	1355	1213.91	3638.71	3638.79	-0.08	2	0	1.3e+03	1	VLSEAVANRQDMLVTDARSTGVPAVVCSAGAHDSGR
☑	927	1000.78	1999.54	1999.01	0.53	1	0	1.6e+03	1	NPALERGSIQNCICVLR + 2 Carbamidomethyl (C)
☑	1203	1133.78	3398.31	3398.69	-0.38	1	0	1.6e+03	1	SIGTDKAGSSLFSGSIISDITNVTANSIQMQEK

2051	682.09	2043.24	2043.10	0.14	2	0	4.7e+02	1	TNMRLVLRDHLLSAYNK
609	854.53	2560.57	2560.24	0.32	2	0	1.3e+03	1	SNPSRILSRCEDIANNSNSTVAR + Carbamidomethyl (C)
1236	576.27	1150.52	1150.68	-0.16	1	0	4.4e+02	1	INTLIHNAKK
2098	1062.35	2122.68	2122.20	0.48	2	0	5.6e+02	1	VEGVGIRPTKKPGGASKSAQR
674	887.01	886.00	886.52	-0.51	1	0	1e+03	1	IGGCIRIR
1151	1102.77	3305.29	3305.68	-0.39	2	0	1.5e+03	1	MPSLNNSFVGGAAQQVDIPQSISTREIFKR + Oxidation (M)
1255	580.32	1158.62	1158.60	0.02	0	0	4.9e+02	1	ASTAAAKPSAER
2045	1018.46	2034.90	2035.10	-0.20	0	0	4.6e+02	1	AITDAPFILSAGEQPLPAPK
171	576.37	575.36	575.32	0.04	1	0	3.9e+02	1	IRGCK
1420	1250.59	2499.17	2499.24	-0.06	2	0	1e+03	1	ALTRECIANFGKSLFGGENIMR + Carbamidomethyl (C); Oxidation (M)
1643	728.27	1454.53	1454.87	-0.34	2	0	4.3e+02	1	EKGIALKDVIELK
1837	848.77	1695.53	1695.91	-0.38	1	0	4.8e+02	1	LFKIAFMSGGQAAIAR + Oxidation (M)
2148	737.77	2210.28	2210.16	0.12	2	0	4e+02	1	SYILQSRCLGSSNLLRSWK
949	1011.83	1010.82	1010.54	0.28	0	0	3.7e+02	1	AALAEGIVPGGG
1334	1203.57	3607.69	3607.73	-0.04	2	0	1.2e+03	1	WSEDGVITPSAKIPNPDAYEDQSNILKSEYNK
2221	777.90	2330.69	2330.17	0.52	1	0	6.7e+02	1	VTPNPGGVTFVSANKQGVGMENK
974	1019.83	2037.65	2037.84	-0.19	1	0	1.5e+03	1	MYSTAKCNESCTSSDLNK + Carbamidomethyl (C)
1715	770.38	1538.74	1538.82	-0.08	0	0	4.3e+02	1	LLSQAGDVWPNALR
148	562.67	1123.32	1123.61	-0.29	1	0	1.2e+03	1	IICIEFMKK
475	776.21	2325.61	2325.09	0.52	1	0	1.9e+03	1	LSCGPMMREVSTDNQFIPLR + 2 Oxidation (M)
1463	641.31	1280.60	1280.58	0.02	1	0	4.2e+02	1	MCCAADRVSVAR
1229	1149.08	3444.23	3444.75	-0.52	0	0	1.8e+03	1	DGVGFPPPPPPPPPPPSYVEALSPAPEYRPAPR
698	904.52	903.51	903.48	0.03	2	0	5.1e+02	1	ASKAKEDR
1342	603.36	1204.70	1204.60	0.09	1	0	4.6e+02	1	DDMIKDDLLK
244	614.36	613.35	613.34	0.01	0	0	1.1e+03	1	TPAPTK
426	741.75	740.74	740.33	0.41	0	0	3e+02	1	FNTTCR
1176	1119.40	2236.78	2237.21	-0.43	2	0	1.5e+03	1	EIKAQAPESMTIKIIAPPER + Oxidation (M)
1534	673.00	1344.00	1343.70	0.30	2	0	5.3e+02	1	AVEKNPMNAKAR + Oxidation (M)
2449	1057.48	3169.43	3169.52	-0.09	2	0	3.2e+02	1	AARTLSPKPSTVGMERMCMTEVVSDVAAK + Carbamidomethyl (C); 3 Oxidation (M)
667	884.05	2649.13	2649.08	0.05	0	0	1.4e+03	1	NHSCDWCGGLDNSEAASATEAASPTR
1128	1091.51	3271.50	3271.55	-0.05	1	0	1.3e+03	1	WEANHCHWMNAFTGCGRPLFALLKAWR + Carbamidomethyl (C)
1862	865.64	1729.26	1728.71	0.55	0	0	6.1e+02	1	MASQYIGNYDNYMK + 2 Oxidation (M)
562	824.51	823.50	823.35	0.16	0	0	3.3e+02	1	MYSMHR
640	870.53	869.52	869.41	0.11	0	0	3.5e+02	1	AAGPMLSAH + Oxidation (M)

☑	2438	1040.90	3119.67	3119.63	0.04	0	0	3.5e+02	1	TESGSTPLIHASINGHLDVVQYLISVGANK
☑	2445	1047.20	3138.58	3138.47	0.11	1	0	3.3e+02	1	IHCEVLPAGFAQDREEGAEPGEISVESGR + Carbamidomethyl (C)
☑	1199	1131.37	2260.73	2260.18	0.55	2	0	1.5e+03	1	FVDATETALAQENKKTKPNR
☑	1122	1090.29	3267.84	3267.57	0.27	2	0	1.6e+03	1	IDMEATVPCSATAMMLGGSSGLLARAERVNK + Carbamidomethyl (C); 2 Oxidation (M
☑	1142	1098.95	3293.83	3293.65	0.17	2	0	1.4e+03	1	RLLTDFEYYLIDALTDPQKCVESSLSFK
☑	1689	756.20	1510.38	1510.74	-0.37	1	0	6.5e+02	1	ATLDGVVTYEGRCK
☑	1869	583.53	1747.57	1747.83	-0.26	0	0	4.6e+02	1	YTFANCTLLTTVDMR
☑	642	872.53	871.52	871.45	0.07	0	0	5.4e+02	1	DERPTVR
☑	873	490.21	978.40	978.52	-0.12	1	0	4.8e+02	1	SRFSINQK
☑	2448	1055.88	3164.60	3164.48	0.12	0	0	3.1e+02	1	YFITFYDSYIQVLNEGHCYYIESLK + Carbamidomethyl (C)
☑	1864	868.10	1734.19	1733.92	0.27	2	0	4.8e+02	1	EVLHVHEKEPAYRK
☑	1836	848.33	1694.64	1694.87	-0.22	1	0	4.4e+02	1	GGLFSLEVRDASMVAK + Oxidation (M)
☑	1921	606.59	1816.75	1816.83	-0.08	1	0	4.9e+02	1	VEELTDHCCKLEVDK + Carbamidomethyl (C)
☑	1841	567.73	1700.17	1699.89	0.28	1	0	5e+02	1	RNSSGPHHPLTTQIR
☑	2035	675.59	2023.74	2023.99	-0.25	1	0	4.9e+02	1	SMKFQFPLLCEYFLSR + Oxidation (M)
☑	666	883.36	2647.04	2647.40	-0.36	2	0	1.2e+03	1	VLFRVPGAPQADWVDIYNRLYR
☑	576	831.59	2491.74	2492.23	-0.49	1	0	1.9e+03	1	LSGCLMNVNQADSRHQVGLPPEK
☑	1222	1142.96	3425.86	3425.83	0.03	1	0	1.4e+03	1	LYVVNEIVSTEESYARALQTVVDLYITPLK
☑	777	936.09	2805.25	2805.44	-0.19	2	0	1.7e+03	1	MNQLESLKKHTCVVIDTADFSLLK + Carbamidomethyl (C); Oxidation (M)
☑	2185	753.35	2257.02	2257.05	-0.03	1	0	4.1e+02	1	NREADINTALMQFSNNFTR + Oxidation (M)
☑	2411	990.12	2967.32	2967.57	-0.25	2	0	3.5e+02	1	GQRPAGLTHASHGSRVLELEEELRGLR
☑	1393	1237.30	2472.59	2472.16	0.43	0	0	1.3e+03	1	DFDLSILDELCEITFEINSEK
☑	1428	1254.55	3760.63	3760.78	-0.15	1	0	1.2e+03	1	AMMMTGRVLLLVCALCVLWCCGAAVVSSMPDVTGR + Carbamidomethyl (C); 3 Oxidatic
☑	2409	984.19	2949.56	2949.34	0.22	2	0	3.6e+02	1	MLSPVRFDGSQCVMRYGVCVCVCAR + 3 Carbamidomethyl (C)
☑	376	703.98	702.97	702.37	0.59	0	0	9.9e+02	1	AGLMAPK + Oxidation (M)
☑	2122	1083.84	2165.67	2165.84	-0.17	1	0	6.3e+02	1	TCGQCGRTGTMMVCSWCR + 3 Carbamidomethyl (C); Oxidation (M)
☑	586	842.28	2523.83	2524.12	-0.29	1	0	1.5e+03	1	LGMDECAKSVGMPCHGYVVLDER + Oxidation (M)
☑	2038	1014.24	2026.46	2026.80	-0.34	1	0	7.1e+02	1	AMCWSKDGTHVAMGCDDK + Carbamidomethyl (C); Oxidation (M)
☑	2293	837.50	2509.48	2510.08	-0.60	2	0	5.3e+02	1	MGGKTNLCELYRTYCAEEGCR + 2 Carbamidomethyl (C)
☑	2353	900.28	2697.82	2697.27	0.54	1	0	6.4e+02	1	MVDANVDNDISLAMISVNARFGDSK + Oxidation (M)
☑	1137	1096.98	3287.93	3287.59	0.34	2	0	1.4e+03	1	RPNQQQLHGGVGNNAGSANRNNNNANRYPR
☑	123	544.77	543.77	543.28	0.49	0	0	5.3e+02	1	SGAPGR
☑	1053	1059.51	2117.00	2116.94	0.07	0	0	1.4e+03	1	TGNQSANVFDISEEQMFGK + Oxidation (M)
☑	2384	949.78	2846.32	2846.41	-0.08	2	0	4.2e+02	1	GAEKGVPNACIEYANCLINGIAVEKDR

☑	2434	1034.25	3099.73	3099.58	0.15	1	0	3.6e+02	1	TQLIFVSRTSATMTAAVDLEGVALHGHMK + Oxidation (M)
☑	486	784.51	2350.50	2349.96	0.54	2	0	1.3e+03	1	CYACIAHCKICSNSNSCRSCK + Carbamidomethyl (C)
☑	1350	1211.83	3632.48	3632.88	-0.40	1	0	1.5e+03	1	MAHRPDDSKYALFFTCVLSHLPMVAVVAFLVR
☑	1218	1141.23	2280.46	2280.14	0.32	2	0	1.5e+03	1	RNDFQLSKIIGTQMDMVER
☑	1358	1217.10	3648.28	3648.63	-0.35	1	0	1.8e+03	1	STLVQFGNYTTTSQNTSDCGVHGDCKDLVYMHK
☑	2018	999.89	1997.77	1998.04	-0.27	2	0	4.2e+02	1	AKDTWGHSSISTGLNIRR
☑	1	*227.13*	*226.13*							
☑	2	*229.03*	*228.02*							
☑	3	*229.13*	*228.12*							
☑	4	*229.16*	*228.15*							
☑	5	*235.12*	*234.11*							
☑	7	*284.95*	*283.94*							
☑	8	*284.97*	*283.96*							
☑	9	*284.99*	*283.98*							
☑	10	*285.00*	*283.99*							
☑	11	*285.00*	*283.99*							
☑	12	*285.02*	*284.01*							
☑	13	*285.18*	*284.17*							
☑	14	*297.10*	*296.09*							
☑	15	*298.99*	*297.98*							
☑	16	*299.00*	*297.99*							
☑	17	*299.03*	*298.02*							
☑	18	*299.04*	*298.03*							
☑	19	*299.07*	*298.06*							
☑	20	*299.10*	*298.09*							
☑	23	*329.82*	*328.81*							
☑	27	*355.07*	*354.07*							
☑	28	*355.19*	*354.18*							
☑	29	*359.19*	*358.18*							
☑	30	*371.06*	*370.06*							
☑	31	*371.07*	*370.06*							
☑	33	*371.10*	*370.10*							
☑	40	*416.16*	*415.15*							
☑	42	*429.05*	*428.04*							

☑	43	429.45	428.44
☑	47	445.03	444.02
☑	48	445.06	444.06
☑	50	449.15	448.14
☑	55	464.00	462.99
☑	56	465.16	464.15
☑	57	465.33	464.32
☑	58	466.51	465.50
☑	61	479.82	478.81
☑	62	480.69	479.68
☑	66	484.63	483.62
☑	71	490.25	489.24
☑	81	504.21	503.20
☑	82	508.62	507.62
☑	84	509.74	508.73
☑	87	513.75	512.75
☑	88	514.71	513.70
☑	92	519.12	518.11
☑	94	519.14	518.14
☑	96	519.45	518.44
☑	97	520.17	519.16
☑	99	520.33	519.32
☑	100	521.07	520.06
☑	103	523.34	522.33
☑	104	525.37	524.37
☑	106	527.21	526.20
☑	108	528.59	527.58
☑	114	536.56	535.55
☑	116	538.09	537.08
☑	119	539.72	538.71
☑	120	540.76	539.75
☑	127	546.47	545.47
☑	128	549.53	548.53
☑	138	554.70	553.70

☑	145	559.70	558.69
☑	150	563.62	562.62
☑	151	564.71	563.70
☑	152	564.94	563.93
☑	153	565.05	564.05
☑	155	565.84	564.83
☑	157	566.62	565.61
☑	159	284.92	567.82
☑	160	284.94	567.87
☑	161	284.96	567.90
☑	162	568.99	567.99
☑	164	569.84	568.83
☑	165	570.33	569.32
☑	166	570.62	569.61
☑	168	571.87	570.86
☑	169	572.22	571.21
☑	172	576.66	575.65
☑	175	579.35	578.34
☑	176	579.69	578.68
☑	177	580.26	579.25
☑	179	580.65	579.64
☑	181	581.55	580.54
☑	184	583.05	582.04
☑	192	588.82	587.81
☑	196	591.01	590.00
☑	197	592.51	591.51
☑	200	593.05	592.05
☑	201	593.07	592.06
☑	202	593.09	592.08
☑	203	593.13	592.12
☑	205	593.34	592.33
☑	206	593.35	592.35
☑	210	595.90	594.89
☑	212	596.92	595.92

☑	213	298.99	595.96
☑	214	597.34	596.33
☑	215	597.79	596.78
☑	216	599.44	598.43
☑	218	600.94	599.93
☑	219	601.34	600.34
☑	223	602.52	601.51
☑	224	602.98	601.97
☑	233	610.05	609.04
☑	235	611.03	610.02
☑	237	612.18	611.17
☑	238	612.75	611.74
☑	240	613.31	612.31
☑	250	620.06	619.05
☑	259	623.33	622.32
☑	267	627.26	626.25
☑	269	627.70	626.69
☑	272	628.90	627.89
☑	278	634.04	633.03
☑	280	635.35	634.34
☑	283	638.01	637.00
☑	287	639.45	638.44
☑	288	639.47	638.46
☑	289	639.50	638.49
☑	292	640.53	639.53
☑	308	650.66	649.66
☑	311	651.76	650.76
☑	312	651.96	650.95
☑	316	655.04	654.03
☑	317	655.68	654.68
☑	318	657.71	656.70
☑	321	658.84	657.83
☑	326	663.82	662.81
☑	329	665.33	664.32

☑	334	673.46	672.46
☑	336	675.22	674.21
☑	339	678.25	677.24
☑	340	226.86	677.55
☑	345	341.42	680.83
☑	350	687.24	686.23
☑	352	688.58	687.57
☑	360	692.63	691.62
☑	367	697.88	696.87
☑	373	699.62	698.62
☑	378	705.97	704.97
☑	379	706.13	705.12
☑	382	708.59	707.58
☑	384	710.79	709.79
☑	388	713.80	712.80
☑	389	714.33	713.32
☑	391	715.07	714.06
☑	398	720.33	719.32
☑	399	721.91	720.90
☑	402	725.11	724.10
☑	406	727.96	726.95
☑	410	732.75	731.74
☑	411	733.79	732.78
☑	413	734.11	733.10
☑	415	736.51	735.50
☑	416	737.28	736.27
☑	421	739.40	738.39
☑	424	371.15	740.28
☑	425	371.17	740.33
☑	428	741.97	740.96
☑	429	742.52	741.51
☑	433	745.00	743.99
☑	434	746.29	745.29
☑	438	750.51	749.51

☑	441	752.73	751.72
☑	446	757.93	756.93
☑	447	758.54	757.53
☑	449	758.89	757.88
☑	452	760.62	759.61
☑	453	761.87	760.86
☑	454	762.19	761.18
☑	455	762.86	761.85
☑	460	766.16	765.15
☑	464	770.60	769.60
☑	468	771.72	770.71
☑	472	774.88	773.87
☑	473	775.08	774.07
☑	476	777.03	776.02
☑	478	780.21	779.20
☑	480	782.20	781.19
☑	482	783.33	782.32
☑	483	783.75	782.74
☑	485	784.40	783.39
☑	487	785.13	784.12
☑	488	785.37	784.36
☑	490	393.21	784.41
☑	491	786.05	785.04
☑	492	786.27	785.26
☑	493	788.71	787.70
☑	497	789.96	788.95
☑	500	791.24	790.23
☑	502	791.57	790.56
☑	504	792.98	791.97
☑	505	793.10	792.09
☑	508	794.42	793.41
☑	509	795.70	794.69
☑	511	797.35	796.34
☑	513	797.88	796.87

☑	514	798.86	797.85
☑	520	802.20	801.19
☑	526	804.58	803.57
☑	527	804.91	803.91
☑	533	807.08	806.07
☑	535	808.08	807.07
☑	538	808.77	807.76
☑	542	813.08	812.07
☑	543	813.86	812.86
☑	548	815.46	814.45
☑	556	821.10	820.09
☑	557	821.41	820.40
☑	558	411.63	821.24
☑	560	822.74	821.73
☑	561	823.50	822.49
☑	565	825.74	824.73
☑	568	827.69	826.68
☑	578	833.51	832.50
☑	580	836.51	835.50
☑	581	837.83	836.82
☑	582	838.09	837.08
☑	583	838.09	837.08
☑	585	839.89	838.88
☑	589	843.74	842.73
☑	590	844.31	843.30
☑	594	847.43	846.42
☑	596	848.78	847.78
☑	597	848.98	847.97
☑	600	850.22	849.21
☑	601	851.39	850.39
☑	602	851.72	850.71
☑	603	851.91	850.91
☑	604	284.91	851.71
☑	605	284.94	851.78

☑	606	853.16	852.16
☑	607	853.23	852.22
☑	608	854.23	853.22
☑	611	855.35	854.34
☑	616	429.15	856.28
☑	618	857.80	856.79
☑	619	859.33	858.32
☑	621	860.49	859.48
☑	623	863.02	862.01
☑	624	432.22	862.43
☑	625	863.81	862.80
☑	626	865.61	864.60
☑	627	865.78	864.77
☑	630	866.44	865.43
☑	632	866.71	865.70
☑	635	868.68	867.68
☑	639	870.47	869.46
☑	643	872.85	871.84
☑	650	876.68	875.67
☑	654	877.74	876.73
☑	657	878.94	877.93
☑	661	880.34	879.33
☑	663	880.96	879.95
☑	664	881.30	880.29
☑	665	883.26	882.26
☑	670	885.60	884.60
☑	671	886.37	885.36
☑	672	886.46	885.45
☑	678	890.33	889.32
☑	681	892.80	891.79
☑	682	893.40	892.39
☑	684	894.28	893.28
☑	685	895.20	894.19
☑	686	895.65	894.64

☑	687	895.70	894.69
☑	688	895.74	894.73
☑	691	899.81	898.80
☑	692	900.40	899.39
☑	694	900.55	899.54
☑	696	903.87	902.86
☑	697	452.54	903.06
☑	699	904.72	903.71
☑	703	906.78	905.78
☑	705	907.11	906.10
☑	707	908.55	907.55
☑	709	909.01	908.00
☑	711	909.73	908.72
☑	716	911.12	910.12
☑	719	912.18	911.17
☑	720	912.34	911.33
☑	721	912.41	911.40
☑	723	912.95	911.94
☑	727	913.65	912.65
☑	732	918.24	917.23
☑	733	919.30	918.29
☑	737	922.03	921.02
☑	738	922.60	921.59
☑	739	922.85	921.85
☑	742	462.10	922.19
☑	746	924.40	923.40
☑	748	924.93	923.93
☑	749	925.29	924.28
☑	750	463.19	924.37
☑	752	926.42	925.42
☑	753	926.44	925.44
☑	754	926.66	925.65
☑	756	926.79	925.79
☑	757	463.90	925.79

☑	758	927.18	2778.51	2778.53	-0.01	2	---	1	GLKALNAKVEIQHGLVSAITTGHEHR
☑	759	927.27	926.26						
☑	760	927.67	926.67						
☑	768	931.76	930.75						
☑	771	933.99	932.98						
☑	773	934.86	933.85						
☑	775	935.58	934.57						
☑	776	935.59	934.58						
☑	778	937.96	936.95						
☑	779	938.44	937.43						
☑	780	469.74	937.47						
☑	782	939.04	938.04						
☑	783	939.67	938.67						
☑	786	940.58	939.57						
☑	787	940.74	939.73						
☑	789	941.92	940.91						
☑	792	942.58	941.58						
☑	795	945.65	944.65						
☑	796	945.99	944.98						
☑	797	946.11	945.11						
☑	800	947.52	946.51						
☑	803	949.21	948.20						
☑	806	949.43	948.42						
☑	808	950.44	949.43						
☑	809	950.77	949.76						
☑	811	951.29	950.29						
☑	812	951.33	950.32						
☑	814	951.78	950.77						
☑	818	953.71	952.70						
☑	820	953.88	952.87						
☑	823	477.81	953.61	953.50	0.10	0	---	1	TIHTGVGNR
☑	828	958.24	957.23						
☑	830	958.81	957.80						
☑	832	959.96	958.95						

☑	833	961.25	960.25
☑	834	962.69	961.68
☑	836	964.55	963.55
☑	838	966.70	965.69
☑	839	967.45	966.44
☑	843	968.24	967.23
☑	847	969.12	968.11
☑	850	971.06	970.06
☑	853	971.65	970.64
☑	854	972.01	971.01
☑	855	972.45	971.44
☑	858	974.14	973.13
☑	863	488.20	974.38
☑	868	977.08	976.07
☑	870	978.38	977.37
☑	871	979.09	978.08
☑	872	979.28	978.27
☑	875	490.68	979.34
☑	877	981.49	980.48
☑	879	984.43	983.43
☑	880	984.57	983.56
☑	881	984.87	983.86
☑	882	985.00	983.99
☑	886	493.96	985.90
☑	887	987.21	986.20
☑	891	988.40	987.39
☑	893	988.66	987.65
☑	900	990.40	989.39
☑	904	992.47	991.46
☑	906	993.63	992.62
☑	907	993.64	992.63
☑	911	994.17	993.17
☑	912	995.09	994.09
☑	915	995.80	994.79

☑	916	996.09	995.09
☑	919	997.54	996.53
☑	922	998.09	997.08
☑	924	999.43	998.42
☑	926	1000.12	999.11
☑	929	1001.67	1000.66
☑	930	1002.71	1001.71
☑	931	1003.32	1002.31
☑	935	1004.47	1003.46
☑	939	503.80	1005.59
☑	941	1008.58	1007.57
☑	942	1008.64	1007.64
☑	943	1009.63	1008.62
☑	944	1010.24	1009.23
☑	950	1012.29	1011.28
☑	953	1013.78	1012.77
☑	966	1017.09	1016.09
☑	967	1017.11	1016.10
☑	968	509.23	1016.44
☑	971	1019.05	1018.04
☑	976	1021.45	1020.45
☑	977	1021.95	1020.94
☑	982	1024.15	1023.14
☑	984	1024.81	1023.81
☑	986	1026.19	1025.19
☑	987	1027.19	1026.18
☑	989	1027.96	1026.95
☑	990	514.79	1027.57
☑	995	1029.73	1028.73
☑	1003	1032.09	1031.08
☑	1004	1032.53	1031.52
☑	1005	1032.73	1031.72
☑	1009	1034.87	1033.87
☑	1011	1035.11	1034.10

☑	1016	1037.38	1036.38
☑	1018	1038.11	1037.10
☑	1019	1039.93	1038.92
☑	1020	1040.60	1039.59
☑	1022	1041.26	1040.25
☑	1023	1042.18	1041.17
☑	1028	1045.27	1044.26
☑	1032	1046.53	1045.53
☑	1036	1048.79	1047.78
☑	1043	527.26	1052.50
☑	1044	1054.03	1053.02
☑	1045	1054.09	1053.08
☑	1046	1054.50	1053.49
☑	1047	1056.03	1055.03
☑	1049	1056.66	1055.65
☑	1056	1061.25	1060.24
☑	1057	1061.83	1060.82
☑	1058	1062.36	1061.35
☑	1059	1062.83	1061.82
☑	1071	1068.57	1067.56
☑	1080	1072.64	1071.64
☑	1081	1074.54	1073.53
☑	1082	1075.70	1074.70
☑	1083	1076.00	1074.99
☑	1085	1076.92	1075.91
☑	1087	1077.23	1076.22
☑	1092	1078.72	1077.71
☑	1096	1081.48	1080.47
☑	1097	1082.83	1081.82
☑	1098	1083.09	1082.08
☑	1099	1084.15	1083.15
☑	1102	1084.83	1083.83
☑	1103	1085.05	1084.04
☑	1106	1085.74	1084.73

☑	1110	544.28	1086.54
☑	1111	1087.59	1086.58
☑	1117	1088.64	1087.63
☑	1119	1089.71	1088.70
☑	1120	1089.78	1088.77
☑	1121	1089.96	1088.95
☑	1127	1091.09	1090.08
☑	1133	1093.48	1092.47
☑	1139	1098.55	1097.54
☑	1145	1099.94	1098.93
☑	1146	1100.57	1099.56
☑	1150	1102.73	1101.73
☑	1154	552.74	1103.47
☑	1159	1107.14	1106.14
☑	1164	1109.71	1108.71
☑	1167	1113.14	1112.13
☑	1168	1113.95	1112.94
☑	1178	1119.73	1118.73
☑	1180	1120.59	1119.58
☑	1183	1122.68	1121.67
☑	1184	1124.00	1122.99
☑	1185	1124.60	1123.60
☑	1201	1133.00	1132.00
☑	1206	1135.31	1134.30
☑	1212	1139.15	1138.14
☑	1213	1139.25	1138.24
☑	1217	1140.56	1139.55
☑	1221	1142.78	1141.77
☑	1223	1143.44	1142.44
☑	1228	1148.80	1147.79
☑	1232	575.39	1148.77
☑	1233	1149.90	1148.89
☑	1239	1152.53	1151.53
☑	1240	1152.81	1151.81

☑	1246	578.22	1154.42
☑	1248	1155.65	1154.64
☑	1261	1164.30	1163.30
☑	1277	1171.17	1170.16
☑	1279	1171.40	1170.39
☑	1284	1172.59	1171.59
☑	1285	1173.70	1172.69
☑	1287	587.96	1173.91
☑	1288	1175.01	1174.00
☑	1289	1175.11	1174.10
☑	1290	1175.48	1174.48
☑	1293	1177.46	1176.45
☑	1294	1178.88	1177.87
☑	1295	1179.68	1178.68
☑	1302	1182.68	1181.67
☑	1303	1183.17	1182.16
☑	1306	1184.52	1183.51
☑	1308	593.14	1184.26
☑	1315	1188.78	1187.77
☑	1316	1189.15	1188.14
☑	1319	596.30	1190.58
☑	1322	1194.27	1193.26
☑	1333	1203.42	1202.41
☑	1346	1210.00	1209.00
☑	1347	1210.18	1209.17
☑	1349	1210.74	1209.73
☑	1351	1212.48	1211.48
☑	1357	1216.52	1215.52
☑	1359	1217.58	1216.57
☑	1370	611.98	1221.94
☑	1371	1223.73	1222.72
☑	1373	1224.59	1223.58
☑	1376	1226.39	1225.38
☑	1377	1227.17	1226.16

☑	1387	1233.27	1232.26
☑	1391	618.24	1234.47
☑	1398	619.88	1237.75
☑	1403	1240.18	1239.17
☑	1404	1241.23	1240.22
☑	1408	621.37	1240.73
☑	1409	1242.19	1241.18
☑	1413	622.34	1242.67
☑	1416	1245.68	1244.67
☑	1422	1251.79	1250.78
☑	1423	1251.80	1250.79
☑	1435	1261.07	1260.06
☑	1443	1265.32	1264.31
☑	1448	1266.03	1265.02
☑	1456	1273.70	1272.69
☑	1477	647.75	1293.48
☑	1486	1298.80	1297.79
☑	1493	650.94	1299.87
☑	1497	1304.12	1303.11
☑	1513	660.86	1319.70
☑	1516	1325.05	1324.04
☑	1520	664.04	1326.06
☑	1527	669.48	1336.95
☑	1535	673.36	1344.70
☑	1541	1350.22	1349.21
☑	1544	678.01	1354.01
☑	1565	690.39	1378.77
☑	1574	692.53	1383.05
☑	1585	696.93	1391.85
☑	1607	711.88	1421.74
☑	1617	718.74	1435.47
☑	1627	721.66	1441.30
☑	1648	730.90	1459.79
☑	1692	505.86	1514.56

☑	1698	761.84	1521.66
☑	1700	762.12	1522.23
☑	1711	765.32	1528.62
☑	1712	767.36	1532.70
☑	1713	769.05	1536.08
☑	1742	786.38	1570.75
☑	1759	797.90	1593.78
☑	1769	806.23	1610.45
☑	1777	810.24	1618.46
☑	1778	810.56	1619.11
☑	1784	813.45	1624.88
☑	1785	814.93	1627.85
☑	1786	815.25	1628.49
☑	1789	817.07	1632.13
☑	1791	819.46	1636.90
☑	1792	819.81	1637.60
☑	1806	828.43	1654.85
☑	1817	835.45	1668.89
☑	1821	837.95	1673.89
☑	1833	847.47	1692.93
☑	1834	848.14	1694.26
☑	1835	848.27	1694.53
☑	1839	850.64	1699.27
☑	1840	850.73	1699.45
☑	1851	857.33	1712.64
☑	1865	869.48	1736.94
☑	1866	872.40	1742.79
☑	1870	876.21	1750.41
☑	1871	876.92	1751.83
☑	1873	878.43	1754.85
☑	1877	881.46	1760.90
☑	1895	895.34	1788.67
☑	1910	905.84	1809.67
☑	1917	605.05	1812.13

☑	1919	908.49	1814.96
☑	1927	916.21	1830.40
☑	1929	916.79	1831.56
☑	1933	918.10	1834.19
☑	1948	925.62	1849.23
☑	1955	931.44	1860.87
☑	1956	931.47	1860.92
☑	1957	937.64	1873.27
☑	1958	937.91	1873.80
☑	1959	938.36	1874.71
☑	1961	940.46	1878.91
☑	1962	942.41	1882.81
☑	1969	952.12	1902.22
☑	1972	636.75	1907.24
☑	1976	959.50	1917.00
☑	1977	959.89	1917.77
☑	1982	962.05	1922.09
☑	1984	965.78	1929.55
☑	1985	967.03	1932.04
☑	1986	645.35	1933.03
☑	1987	968.09	1934.16
☑	1991	979.83	1957.64
☑	1993	980.52	1959.03
☑	2003	660.05	1977.13
☑	2005	990.62	1979.23
☑	2006	991.02	1980.03
☑	2009	992.43	1982.85
☑	2010	993.98	1985.95
☑	2013	996.75	1991.48
☑	2014	997.01	1992.01
☑	2019	1000.71	1999.40
☑	2020	1004.54	2007.07
☑	2021	1004.69	2007.37
☑	2030	673.28	2016.81

2031	1009.45	2016.88
2033	1010.01	2018.00
2034	1011.49	2020.97
2036	675.62	2023.85
2040	1014.67	2027.33
2042	1015.65	2029.28
2047	679.35	2035.01
2048	1018.70	2035.38
2050	1021.98	2041.95
2052	1023.73	2045.44
2055	1027.48	2052.96
2057	1027.59	2053.16
2058	1029.53	2057.05
2059	1032.66	2063.30
2060	1033.12	2064.22
2061	1037.04	2072.06
2062	1038.27	2074.53
2064	1039.13	2076.24
2065	1039.93	2077.85
2066	1040.26	2078.50
2067	1041.34	2080.66
2073	1046.50	2090.99
2074	1047.47	2092.93
2075	1047.74	2093.47
2076	1047.94	2093.88
2080	1050.04	2098.07
2081	1050.59	2099.16
2087	1055.23	2108.45
2088	1055.99	2109.96
2090	1058.24	2114.47
2097	1062.30	2122.58
2101	710.26	2127.77
2103	1066.50	2130.99
2104	1069.99	2137.96

☑	2107	1072.07	2142.13
☑	2109	1072.77	2143.53
☑	2110	1072.85	2143.68
☑	2114	1077.70	2153.40
☑	2118	1080.47	2158.93
☑	2123	1085.51	2169.01
☑	2129	728.66	2182.97
☑	2130	1092.89	2183.77
☑	2131	730.07	2187.18
☑	2132	1095.49	2188.96
☑	2134	731.42	2191.23
☑	2138	1098.62	2195.22
☑	2141	1103.91	2205.81
☑	2142	1104.20	2206.39
☑	2143	1104.67	2207.33
☑	2144	1104.93	2207.85
☑	2150	1106.45	2210.89
☑	2153	1108.75	2215.49
☑	2154	1108.79	2215.57
☑	2155	741.08	2220.22
☑	2156	1112.59	2223.17
☑	2161	1114.43	2226.85
☑	2163	1117.42	2232.83
☑	2172	1121.52	2241.02
☑	2173	1124.09	2246.16
☑	2176	1124.65	2247.28
☑	2178	1126.02	2250.02
☑	2184	1128.41	2254.81
☑	2186	753.83	2258.47
☑	2187	1132.55	2263.09
☑	2190	1134.11	2266.21
☑	2193	1136.09	2270.16
☑	2194	1136.68	2271.34
☑	2195	1137.52	2273.03

☑	2197	1140.55	2279.08
☑	2202	1147.82	2293.62
☑	2203	1148.18	2294.35
☑	2205	1148.85	2295.68
☑	2212	1153.85	2305.68
☑	2214	1154.71	2307.40
☑	2217	1158.35	2314.68
☑	2225	1172.92	2343.83
☑	2229	1176.20	2350.39
☑	2232	1178.34	2354.67
☑	2236	1181.28	2360.55
☑	2245	793.91	2378.72
☑	2254	1203.01	2404.01
☑	2255	1203.27	2404.52
☑	2260	809.73	2426.17
☑	2262	812.96	2435.85
☑	2264	814.60	2440.78
☑	2268	1227.83	2453.64
☑	2278	827.72	2480.14
☑	2286	1248.09	2494.16
☑	2287	833.51	2497.52
☑	2289	835.01	2502.02
☑	2296	838.02	2511.04
☑	2305	845.07	2532.18
☑	2307	845.74	2534.21
☑	2309	848.48	2542.43
☑	2311	849.06	2544.16
☑	2315	851.43	2551.26
☑	2318	854.11	2559.30
☑	2326	861.50	2581.49
☑	2331	867.52	2599.54
☑	2335	876.14	2625.39
☑	2340	880.00	2636.98
☑	2342	880.40	2638.19

☑	2347	883.48	2647.41
☑	2349	893.96	2678.86
☑	2351	898.11	2691.30
☑	2356	904.42	2710.25
☑	2357	904.48	2710.42
☑	2358	904.92	2711.74
☑	2362	1360.81	2719.60
☑	2365	912.70	2735.08
☑	2368	918.26	2751.76
☑	2370	919.53	2755.56
☑	2371	921.39	2761.14
☑	2375	924.36	2770.06
☑	2378	939.54	2815.59
☑	2382	945.99	2834.95
☑	2385	952.46	2854.35
☑	2387	953.10	2856.27
☑	2390	958.41	2872.22
☑	2393	960.18	2877.53
☑	2394	961.20	2880.57
☑	2395	962.54	2884.61
☑	2398	965.44	2893.30
☑	2400	967.86	2900.57
☑	2401	972.45	2914.32
☑	2405	981.97	2942.89
☑	2406	982.27	2943.79
☑	2412	992.07	2973.18
☑	2413	994.03	2979.08
☑	2414	994.13	2979.36
☑	2415	995.45	2983.32
☑	2417	1001.87	3002.58
☑	2419	1003.56	3007.66
☑	2421	1006.08	3015.21
☑	2422	1006.46	3016.34
☑	2424	1009.45	3025.33

☑	2425	1011.36	3031.05
☑	2427	1015.82	3044.43
☑	2429	1024.56	3070.67
☑	2430	1028.59	3082.75
☑	2431	1029.54	3085.60
☑	2432	1030.39	3088.15
☑	2435	1035.58	3103.71
☑	2436	1036.51	3106.51
☑	2437	1037.88	3110.61
☑	2439	1041.71	3122.10
☑	2440	1042.89	3125.65
☑	2443	1044.40	3130.18
☑	2444	1044.63	3130.88
☑	2446	1050.89	3149.65
☑	2447	1052.29	3153.84
☑	2450	1059.68	3176.03
☑	2451	1065.54	3193.60
☑	2452	1066.93	3197.76
☑	2454	1082.56	3244.65
☑	2455	1084.31	3249.91
☑	2456	1086.46	3256.35
☑	2458	1093.52	3277.55
☑	2460	1095.81	3284.41
☑	2461	1096.08	3285.23
☑	2462	1098.74	3293.20
☑	2465	1101.56	3301.64
☑	2467	1111.43	3331.27
☑	2468	1111.94	3332.80
☑	2469	1117.34	3348.99
☑	2470	1118.55	3352.62
☑	2472	1122.03	3363.08
☑	2473	1123.94	3368.81
☑	2474	1126.55	3376.63
☑	2475	1126.69	3377.06

☑	2476	*1127.45*	*3379.34*
☑	2478	*1140.42*	*3418.24*
☑	2482	*1163.00*	*3485.99*
☑	2486	*1180.51*	*3538.52*
☑	2487	*1184.95*	*3551.83*
☑	2489	*1206.96*	*3617.86*
☑	2490	*1208.48*	*3622.43*
☑	2492	*1222.46*	*3664.35*
☑	2495	*1260.04*	*3777.10*

Search Parameters

```
Type of search         : MS/MS Ion Search
Enzyme                 : Trypsin
Variable modifications : Carbamidomethyl (C),Oxidation (M)
Mass values            : Monoisotopic
Protein Mass           : Unrestricted
Peptide Mass Tolerance : ± 0.6 Da
Fragment Mass Tolerance: ± 0.3 Da
Max Missed Cleavages   : 2
Instrument type        : ESI-TRAP
Number of queries      : 2495
```

Mascot: http://www.matrixscience.com/
