# Supplementary material for: Macrophage-infectivity potentiator of Trypanosoma cruzi (TcMIP) is a new pro-type 1 immuno-stimulating protein for neonatal human cells and vaccines in mice
Source: Front Immunol. 2023 Mar 23;14:1138526. doi: 10.3389/fimmu.2023.1138526 (PMC10077492; doi:10.3389/fimmu.2023.1138526)
Supplement: Supplementary file 8 [file DataSheet_6.pdf]

Mascot Search Results

User : GM
Email : gabriel.mazzucchelli@ulg.ac.be
Search title : Submitted from 090116-Adj-OGE39-8923-otherEuk-sprot by Mascot Daemon on MASPEC39
MS data file : F:\DATA\Archives\ESQUIRE\2009 esquire\Adjuvac\090114\090116-ADJ-OGE39_8923.mgf
Database : Sprot 55.5 (389046 sequences; 139778124 residues)
Taxonomy : Other Eukaryota (2925 sequences)
Timestamp : 19 Jan 2009 at 08:16:35 GMT
Significant hits:

CH60_TRYCR	Q95046|CH60_TRYCR Chaperonin HSP60, mitochondrial precursor - Trypanosoma cruzi
TBB_TRYCR	P08562|TBB_TRYCR Tubulin beta chain - Trypanosoma cruzi
TBB_TRYBR	P04107|TBB_TRYBR Tubulin beta chain - Trypanosoma brucei rhodesiense
CH60_TRYBB	Q37683|CH60_TRYBB Chaperonin HSP60, mitochondrial precursor - Trypanosoma brucei brucei
G3PG_TRYCR	P22513|G3PG_TRYCR Glyceraldehyde-3-phosphate dehydrogenase, glycosomal - Trypanosoma cruzi
FCA1_TRYCR	P07749|FCA1_TRYCR Flagellar calcium-binding protein - Trypanosoma cruzi
MIP_TRYCR	Q09734|MIP_TRYCR Macrophage infectivity potentiator precursor - Trypanosoma cruzi
UBIQ_TRYCR	P08565|UBIQ_TRYCR Ubiquitin - Trypanosoma cruzi
TPIS_TRYCR	P52270|TPIS_TRYCR Triosephosphate isomerase, glycosomal - Trypanosoma cruzi
HSP70_BRELC	P16394|HSP70_BRELC Heat shock 70 kDa protein - Bremia lactucae (Lettuce downy mildew)
EF1A_TRYBB	P41166|EF1A_TRYBB Elongation factor 1-alpha - Trypanosoma brucei brucei
CALM_EUGGR	P11118|CALM_EUGGR Calmodulin - Euglena gracilis
EF1A_BLAHO	P54959|EF1A_BLAHO Elongation factor 1-alpha - Blastocystis hominis
HSP70_LEIMA	P14834|HSP70_LEIMA Heat shock 70 kDa protein - Leishmania major
HSP70_TRYCR	P05456|HSP70_TRYCR Heat shock 70 kDa protein - Trypanosoma cruzi
HSP70_LEIAM	Q07437|HSP70_LEIAM Heat shock 70 kDa protein - Leishmania amazonensis
EF1AC_PORPU	P50256|EF1AC_PORPU Elongation factor 1-alpha C - Porphyra purpurea
EF1AS_PORPU	P50257|EF1AS_PORPU Elongation factor 1-alpha S - Porphyra purpurea
FCA1_TRYRA	Q27052|FCA1_TRYRA Flagellar calcium-binding protein - Trypanosoma rangeli
TBA_TRYCR	Q27352|TBA_TRYCR Tubulin alpha chain - Trypanosoma cruzi
UBIQ_ACACA	P49634|UBIQ_ACACA Ubiquitin - Acanthamoeba castellanii (Amoeba)
CYC_CRION	P00077|CYC_CRION Cytochrome c - Crithidia oncopelti
G3PG_CRIFA	O96423|G3PG_CRIFA Glyceraldehyde-3-phosphate dehydrogenase, glycosomal - Crithidia fasciculata
HSP70_PYRSA	P37899|HSP70_PYRSA Heat shock 70 kDa protein - Pyrenomonas salina
RLA2_TRYCR	P23632|RLA2_TRYCR 60S acidic ribosomal protein P2-A - Trypanosoma cruzi
H2A_TRYCR	P35066|H2A_TRYCR Histone H2A - Trypanosoma cruzi
EF1A_ENTHI	P31018|EF1A_ENTHI Elongation factor 1-alpha - Entamoeba histolytica
EF1A_GIALA	Q08046|EF1A_GIALA Elongation factor 1-alpha - Giardia lamblia (Giardia intestinalis)
HSP71_TRYCR	P20583|HSP71_TRYCR Heat shock 70 kDa protein, mitochondrial precursor - Trypanosoma cruzi
CH60_EUGGR	Q39727|CH60_EUGGR Chaperonin CPN60, mitochondrial precursor - Euglena gracilis
CH60_PORYE	Q1XDD8|CH60_PORYE 60 kDa chaperonin - Porphyra yezoensis
TBAD_PHYPO	P50258|TBAD_PHYPO Tubulin alpha-1A chain - Physarum polycephalum (Slime mold)
G3PG_TRYBB	P22512|G3PG_TRYBB Glyceraldehyde-3-phosphate dehydrogenase, glycosomal - Trypanosoma brucei brucei
EF1B_TRYCR	P34827|EF1B_TRYCR 25 kDa elongation factor 1-beta - Trypanosoma cruzi

RLA1_TRYCR	P26643|RLA1_TRYCR 60S acidic ribosomal protein P1 - Trypanosoma cruzi
RLA3_TRYCR	P26795|RLA3_TRYCR 60S acidic ribosomal protein P2-B - Trypanosoma cruzi
RL10_EUGGR	Q39724|RL10_EUGGR 60S ribosomal protein L10 - Euglena gracilis
TBB_GIALA	P05304|TBB_GIALA Tubulin beta chain - Giardia lamblia (Giardia intestinalis)
RK32_THAPS	A0T0V4|RK32_THAPS Chloroplast 50S ribosomal protein L32 - Thalassiosira pseudonana (Marine diatom)
YCF55_PORYE	Q1XDT4|YCF55_PORYE Uncharacterized protein ycf55 - Porphyra yezoensis
RK16_EMIHU	Q4G358|RK16_EMIHU Chloroplast 50S ribosomal protein L16 - Emiliania huxleyi
NDUS1_DICCI	Q2LCP5|NDUS1_DICCI NADH-ubiquinone oxidoreductase 75 kDa subunit - Dictyostelium citrinum (Slime mo
RPOA_EUGGR	P48337|RPOA_EUGGR DNA-directed RNA polymerase subunit alpha - Euglena gracilis
MDR_LEITA	P21441|MDR_LEITA Multidrug resistance protein - Leishmania tarentolae (Sauroleishmania tarentolae)
HSP71_LEIMA	P12076|HSP71_LEIMA Heat shock 70-related protein 1, mitochondrial precursor - Leishmania major
RPB1A_TRYBB	P17546|RPB1A_TRYBB DNA-directed RNA polymerase II subunit RPB1-A - Trypanosoma brucei brucei
RPOC2_CYAPA	P48120|RPOC2_CYAPA DNA-directed RNA polymerase subunit beta'' - Cyanophora paradoxa
RPOC2_PHATR	A0T0D9|RPOC2_PHATR DNA-directed RNA polymerase subunit beta'' - Phaeodactylum tricornutum

Probability Based Mowse Score

Ions score is -10*Log(P), where P is the probability that the observed match is a random event.
Individual ions scores > 22 indicate identity or extensive homology (p<0.05).
Protein scores are derived from ions scores as a non-probabilistic basis for ranking protein hits.

Score Distribution

Peptide Summary Report

Format As | Peptide Summary | Help

Significance threshold p< 0.05 | Max. number of hits AUTO

Standard scoring ○ MudPIT scoring ◉ Ions score cut-off 0 | Show sub-sets ☐

Show pop-ups ◉ Suppress pop-ups ○ Sort unassigned [Decreasing Score] Require bold red ☐

[Select All] [Select None] [Search Selected] ☐ **Error tolerant** [Archive Report]

1. CH60_TRYCR **Mass:** 59374 **Score:** 1147 **Queries matched:** 28
Q95046|CH60_TRYCR Chaperonin HSP60, mitochondrial precursor - Trypanosoma cruzi
☐ Check to include this hit in error tolerant search or archive report

	Query	Observed	Mr(expt)	Mr(calc)	Delta	Miss	Score	Expect	Rank	Peptide
☑	588	422.26	842.51	842.52	-0.02	0	56	1.8e-05	1	K.LSGGVAVIK.V
☑	1013	518.74	1035.47	1035.50	-0.03	0	42	0.00054	1	R.ITDALCSTR.A + Carbamidomethyl (C)
☑	1169	557.79	1113.57	1113.64	-0.07	0	82	3.4e-08	1	R.AVSAVATTLGPK.G
☑	1336	602.32	1202.62	1202.58	0.04	0	105	2.7e-10	1	K.VGGGSEVEVNEK.K
☑	1389	617.34	1232.66	1232.72	-0.07	0	31	0.0051	1	R.LPAHTIVLNAGK.E
☑	1510	659.84	1317.67	1317.69	-0.02	0	88	1.4e-08	1	R.NVIIEQSYGAPK.I
☑	1512	660.81	1319.61	1319.62	-0.01	0	50	4.6e-05	1	R.CIATGTNPIDMK.R + Carbamidomethyl (C)
☑	1529	670.30	1338.59	1338.61	-0.01	0	85	1.6e-08	1	R.GLIDGETSDYNR.E
☑	1550	680.82	1359.63	1359.67	-0.04	0	58	1e-05	1	R.GYISPYFVTDAK.A
☑	1584	696.41	1390.81	1390.73	0.08	0	103	3.7e-10	1	K.AELEDAFVLVSAK.K
☑	1644	728.88	1455.75	1455.80	-0.05	0	99	8.5e-10	1	R.AVGVILQSVAEQSR.K
☑	1764	803.94	1605.86	1605.92	-0.06	0	53	2.5e-05	1	R.AAVQEGIVPGGGVALLR.A
☑	575	831.42	1660.84	1660.83	0.01	0	77	4e-07	1	K.ALDSLLGDSSLTADQR.T
☑	1828	564.89	1691.66	1691.81	-0.15	0	(29)	0.007	1	K.VLENNDVTVGYDAQR.D
☑	1829	846.88	1691.75	1691.81	-0.07	0	108	9.2e-11	1	K.VLENNDVTVGYDAQR.D
☑	1909	905.79	1809.57	1809.85	-0.28	0	(1)	4.9	1	K.TMTTELEVVEGMSIDR.G
☑	702	905.82	1809.63	1809.85	-0.22	0	(6)	4.5	1	K.TMTTELEVVEGMSIDR.G
☑	1911	905.88	1809.74	1809.85	-0.11	0	(35)	0.0018	1	K.TMTTELEVVEGMSIDR.G
☑	227	604.28	1809.83	1809.85	-0.02	0	(33)	0.0088	1	K.TMTTELEVVEGMSIDR.G
☑	1912	604.29	1809.86	1809.85	0.01	0	(45)	0.00016	1	K.TMTTELEVVEGMSIDR.G
☑	1925	913.89	1825.77	1825.84	-0.07	0	71	4e-07	1	K.TMTTELEVVEGMSIDR.G + Oxidation (M)
☑	1997	655.69	1964.05	1963.98	0.07	1	53	3e-05	1	K.AIEFKDPFENMGAQLVR.Q
☑	2294	837.71	2510.11	2510.25	-0.14	0	(63)	2.1e-06	1	K.TNDLAGDGTTTSAVLVASVFSESLR.C
☑	2295	1256.10	2510.19	2510.25	-0.06	0	115	1.3e-11	1	K.TNDLAGDGTTTSAVLVASVFSESLR.C
	2304	844.92	2531.73	2532.29	-0.56	2	1	6.4	2	K.DDTVLLNGGGESSMVKERVELLR.G + Oxidation (M)
☑	2321	1287.17	2572.33	2572.29	0.04	0	(67)	9.7e-07	1	R.LVGEEGSGLELDAENFDPAILGTVK.K

☑	2322	858.46	2572.35	2572.29	0.06	0	73	2.8e-07	1	R.LVGEEGSGLELDAENFDPAILGTVK.K
☑	2323	858.63	2572.87	2572.29	0.58	0	(1)	5.8	1	R.LVGEEGSGLELDAENFDPAILGTVK.K

---

2. TBB_TRYCR **Mass:** 49541 **Score:** 281 **Queries matched:** 5
P08562|TBB_TRYCR Tubulin beta chain - Trypanosoma cruzi
☐ Check to include this hit in error tolerant search or archive report

	Query	Observed	Mr(expt)	Mr(calc)	Delta	Miss	Score	Expect	Rank	Peptide
☑	1531	671.28	1340.55	1340.64	-0.09	0	87	1.2e-08	1	R.INVYFDEATGGR.Y
☑	1630	723.83	1445.65	1445.68	-0.03	0	51	4.2e-05	1	K.EVDEQMLNVQNK.N
☑	1773	808.43	1614.85	1614.83	0.02	0	53	2.2e-05	1	R.AVLIDLEPGTMDSVR.A
☑	1946	924.44	1846.86	1846.87	-0.01	0	87	1e-08	1	R.EIVCVQAGQCGNQIGSK.F + 2 Carbamidomethyl (C)
☑	2433	1031.78	3092.33	3092.40	-0.07	0	81	3.1e-08	1	K.FWEVISDEHGVDPTGTYQGDSDLQLER.I

---

3. TBB_TRYBR **Mass:** 49672 **Score:** 272 **Queries matched:** 5
P04107|TBB_TRYBR Tubulin beta chain - Trypanosoma brucei rhodesiense
☐ Check to include this hit in error tolerant search or archive report

	Query	Observed	Mr(expt)	Mr(calc)	Delta	Miss	Score	Expect	Rank	Peptide
☑	1007	517.26	1032.50	1032.45	0.06	0	46	0.00019	1	K.NMMQAADPR.H
	1531	671.28	1340.55	1340.64	-0.09	0	87	1.2e-08	1	R.INVYFDEATGGR.Y
	1630	723.83	1445.65	1445.68	-0.03	0	51	4.2e-05	1	K.EVDEQMLNVQNK.N
	1946	924.44	1846.86	1846.87	-0.01	0	87	1e-08	1	R.EIVCVQAGQCGNQIGSK.F + 2 Carbamidomethyl (C)
	2433	1031.78	3092.33	3092.40	-0.07	0	81	3.1e-08	1	K.FWEVISDEHGVDPTGTYQGDSDLQLER.I

---

4. CH60_TRYBB **Mass:** 59492 **Score:** 229 **Queries matched:** 5
Q37683|CH60_TRYBB Chaperonin HSP60, mitochondrial precursor - Trypanosoma brucei brucei
☐ Check to include this hit in error tolerant search or archive report

	Query	Observed	Mr(expt)	Mr(calc)	Delta	Miss	Score	Expect	Rank	Peptide
	1013	518.74	1035.47	1035.50	-0.03	0	42	0.00054	1	R.ITDALCSTR.A + Carbamidomethyl (C)
	1441	632.31	1262.61	1262.62	-0.01	0	0	6	2	K.SIATGTNPIDMK.R + Oxidation (M)
	1510	659.84	1317.67	1317.69	-0.02	0	88	1.4e-08	1	R.NVIIEQSYGAPK.I

1550	680.82	1359.63	1359.67	-0.04	0	58	1e-05	1	R.GYISPYFVTDAK.T
1584	696.41	1390.81	1390.73	0.08	0	103	3.7e-10	1	K.AELEDAFVLVSAK.K

---

5. G3PG_TRYCR **Mass:** 39036 **Score:** 222 **Queries matched:** 9

P22513|G3PG_TRYCR Glyceraldehyde-3-phosphate dehydrogenase, glycosomal - Trypanosoma cruzi

☐ Check to include this hit in error tolerant search or archive report

	Query	Observed	Mr(expt)	Mr(calc)	Delta	Miss	Score	Expect	Rank	Peptide
	540	**406.22**	**810.43**	**810.41**	**0.02**	**0**	**17**	**0.097**	**2**	**K.LTGMSFR.V**
☑	1312	**594.34**	**1186.66**	**1186.64**	**0.02**	**0**	**34**	**0.0028**	**1**	**K.AVGMVIPSTQGK.L**
☑	1577	**692.86**	**1383.71**	**1383.77**	**-0.06**	**0**	**59**	**7.3e-06**	**1**	**R.AAAVNIIPSTTGAAK.A**
☑	1647	**487.27**	**1458.80**	**1458.77**	**0.03**	**1**	**38**	**0.0011**	**1**	**R.DTSIQEIDAALKR.A**
☑	1892	**894.48**	**1786.94**	**1786.95**	**-0.00**	**0**	**(16)**	**0.13**	**1**	**R.VPTPDVSVVDLTFTAAR.D**
☑	1893	**894.49**	**1786.96**	**1786.95**	**0.01**	**0**	**102**	**3.2e-10**	**1**	**R.VPTPDVSVVDLTFTAAR.D**
☑	1894	**596.72**	**1787.14**	**1786.95**	**0.19**	**0**	**(52)**	**3.1e-05**	**1**	**R.VPTPDVSVVDLTFTAAR.D**
☑	1913	**604.59**	**1810.74**	**1810.81**	**-0.06**	**0**	**13**	**0.21**	**1**	**K.IVSWYDNEWGYSHR.V**
☑	2168	**1121.07**	**2240.12**	**2240.06**	**0.06**	**0**	**33**	**0.0026**	**1**	**K.GILGYTDEELVSADFINDNR.S**

---

6. FCA1_TRYCR **Mass:** 23721 **Score:** 187 **Queries matched:** 6

P07749|FCA1_TRYCR Flagellar calcium-binding protein - Trypanosoma cruzi

☐ Check to include this hit in error tolerant search or archive report

	Query	Observed	Mr(expt)	Mr(calc)	Delta	Miss	Score	Expect	Rank	Peptide
☑	530	**403.26**	**804.51**	**804.49**	**0.03**	**1**	**7**	**1.3**	**1**	**R.RIELFK.K**
☑	653	**439.24**	**876.47**	**876.43**	**0.03**	**0**	**43**	**0.00038**	**1**	**K.LDEFTPR.V**
☑	899	**495.30**	**988.58**	**988.52**	**0.06**	**0**	**31**	**0.0052**	**1**	**K.VEDPAALFK.E**
☑	1598	**707.77**	**1413.53**	**1413.59**	**-0.06**	**0**	**55**	**2.3e-05**	**1**	**K.LDADGDPDNVPESA.-**
☑	1662	**737.90**	**1473.78**	**1473.68**	**0.11**	**0**	**90**	**7.4e-09**	**1**	**K.GSEDFVEFLEFR.L**
☑	1924	**607.96**	**1820.84**	**1820.84**	**-0.00**	**0**	**47**	**0.00013**	**1**	**K.LCYDEVHSGCLEVLK.L + 2 Carbamidomethyl (C)**

---

7. MIP_TRYCR **Mass:** 22122 **Score:** 185 **Queries matched:** 6

Q09734|MIP_TRYCR Macrophage infectivity potentiator precursor - Trypanosoma cruzi

☐ Check to include this hit in error tolerant search or archive report

	Query	Observed	Mr(expt)	Mr(calc)	Delta	Miss	Score	Expect	Rank	Peptide
☑	961	508.77	1015.54	1015.58	-0.05	0	(37)	0.0014	1	K.LPSGLVFQR.I
☑	962	508.78	1015.54	1015.58	-0.04	0	51	5.6e-05	1	K.LPSGLVFQR.I
☑	963	508.87	1015.72	1015.58	0.14	0	(44)	0.00025	1	K.LPSGLVFQR.I
☑	1286	587.80	1173.60	1173.59	0.01	0	63	2.6e-06	1	R.TAEEVDEILR.K
☑	1339	602.86	1203.71	1203.61	0.10	0	68	1e-06	1	K.GWTEALQLMR.E
☑	1900	599.77	1796.28	1795.99	0.28	1	2	4.1	1	K.AAQPDAVKLPSGLVFQR.I

8. UBIQ_TRYCR   **Mass:** 8504   **Score:** 178   **Queries matched:** 5
P08565|UBIQ_TRYCR Ubiquitin - Trypanosoma cruzi
☐ Check to include this hit in error tolerant search or archive report

	Query	Observed	Mr(expt)	Mr(calc)	Delta	Miss	Score	Expect	Rank	Peptide
☑	459	383.22	764.43	764.43	0.01	0	25	0.017	1	-.MQIFVK.T
☑	1066	533.27	1064.53	1064.55	-0.02	0	59	7.7e-06	1	R.TLADYNIQK.E
☑	1070	534.32	1066.62	1066.61	0.00	0	48	0.00011	1	K.ESTLHLVLR.L
☑	1701	508.62	1522.83	1522.77	0.06	1	23	0.029	1	K.IQDKEGIPPDQQR.L
☑	1867	874.42	1746.83	1746.89	-0.06	0	100	5.7e-10	1	K.TIALEVESSDTIENVK.A

9. TPIS_TRYCR   **Mass:** 27312   **Score:** 167   **Queries matched:** 4
P52270|TPIS_TRYCR Triosephosphate isomerase, glycosomal - Trypanosoma cruzi
☐ Check to include this hit in error tolerant search or archive report

	Query	Observed	Mr(expt)	Mr(calc)	Delta	Miss	Score	Expect	Rank	Peptide
☑	1386	616.84	1231.67	1231.67	0.00	0	95	2e-09	1	K.FQIAAQNAITR.S
☑	1634	725.33	1448.65	1448.79	-0.14	0	31	0.0041	1	R.SGAFTGEVSLQILK.D
☑	1707	764.84	1527.66	1527.75	-0.09	0	72	4.1e-07	1	R.LYYGETNEIVAEK.V
☑	1857	573.63	1717.87	1717.91	-0.05	0	25	0.018	1	K.VATPQQAQEVHELLR.R

10. HSP70_BRELC   **Mass:** 74041   **Score:** 163   **Queries matched:** 5
P16394|HSP70_BRELC Heat shock 70 kDa protein - Bremia lactucae (Lettuce downy mildew)
☐ Check to include this hit in error tolerant search or archive report

	Query	Observed	Mr(expt)	Mr(calc)	Delta	Miss	Score	Expect	Rank	Peptide
	1379	614.82	1227.62	1227.62	0.00	0	63	3.5e-06	2	R.VEIIANDQGNR.T
☑	1380	614.84	1227.67	1227.62	0.05	0	(9)	0.85	1	R.VEIIANDQGNR.T
☑	1672	744.34	1486.67	1486.69	-0.03	0	68	1.3e-06	1	R.TTPSYVAFTDTER.L
☑	1808	830.46	1658.90	1658.89	0.01	0	74	2.2e-07	1	R.IINEPTAAAIAYGLDK.K
	2053	1025.94	2049.86	2050.05	-0.19	1	0	4.9	2	K.EVKNAVITVPAYFNDSQR.Q

---

11. EF1A_TRYBB **Mass:** 49003 **Score:** 146 **Queries matched:** 4
P41166|EF1A_TRYBB Elongation factor 1-alpha - Trypanosoma brucei brucei
☐ Check to include this hit in error tolerant search or archive report

	Query	Observed	Mr(expt)	Mr(calc)	Delta	Miss	Score	Expect	Rank	Peptide
☑	762	464.80	927.58	927.58	0.00	0	24	0.024	1	R.QTVAVGIIK.A
☑	985	513.31	1024.61	1024.60	0.01	0	71	4.3e-07	1	K.IGGIGTVPVGR.V
☑	2228	784.39	2350.15	2350.13	0.02	0	22	0.026	1	R.MVPQKPMCVEVFNDYAPLGR.F + Carbamidomethyl (C)
☑	2298	841.69	2522.05	2522.19	-0.14	0	83	1.9e-08	1	K.SIEMHHEQLAEATPGDNVGFNVK.N

---

12. CALM_EUGGR **Mass:** 16845 **Score:** 144 **Queries matched:** 5
P11118|CALM_EUGGR Calmodulin - Euglena gracilis
☐ Check to include this hit in error tolerant search or archive report

	Query	Observed	Mr(expt)	Mr(calc)	Delta	Miss	Score	Expect	Rank	Peptide
☑	991	514.79	1027.58	1027.51	0.06	0	3	2.9	1	R.HVMTNLGEK.L
☑	1539	675.27	1348.53	1348.62	-0.09	0	87	1.2e-08	1	K.LTDEEVDEMIR.E
☑	740	922.87	1843.73	1843.88	-0.16	1	62	1.1e-05	1	K.EAFSLFDKDGDGTITTK.E
☑	1944	615.63	1843.88	1843.88	-0.00	1	(32)	0.0035	1	K.EAFSLFDKDGDGTITTK.E
☑	1945	615.63	1843.88	1843.88	-0.00	1	(23)	0.025	1	K.EAFSLFDKDGDGTITTK.E

Proteins matching the same set of peptides:
CALM_TRYCR **Mass:** 16814 **Score:** 144 **Queries matched:** 5
P18061|CALM_TRYCR Calmodulin - Trypanosoma cruzi

---

13. EF1A_BLAHO **Mass:** 47792 **Score:** 120 **Queries matched:** 3
P54959|EF1A_BLAHO Elongation factor 1-alpha - Blastocystis hominis
☐ Check to include this hit in error tolerant search or archive report

	Query	Observed	Mr(expt)	Mr(calc)	Delta	Miss	Score	Expect	Rank	Peptide
	762	464.80	927.58	927.58	0.00	0	24	0.024	1	R.QTVAVGIIK.S
☑	867	**488.28**	**974.55**	**974.54**	**0.01**	**0**	**63**	**4.6e-06**	**1**	**R.LPLQDVYK.I**
	985	513.31	1024.61	1024.60	0.01	0	71	4.3e-07	1	K.IGGIGTVPVGR.V

---

14. HSP70_LEIMA **Mass:** 56500 **Score:** 120 **Queries matched:** 5
P14834|HSP70_LEIMA Heat shock 70 kDa protein - Leishmania major
☐ Check to include this hit in error tolerant search or archive report

	Query	Observed	Mr(expt)	Mr(calc)	Delta	Miss	Score	Expect	Rank	Peptide
	359	**692.39**	**691.39**	**691.39**	**-0.00**	**0**	**2**	**11**	**2**	**R.GIFEVK.A**
☑	1379	614.82	1227.62	1227.62	0.00	0	66	1.6e-06	1	R.LDIIANDQGNR.T
	1380	614.84	1227.67	1227.62	0.05	0	(9)	0.85	1	R.LDIIANDQGNR.T
☑	1665	**494.61**	**1480.81**	**1480.81**	**0.00**	**1**	**2**	**4.7**	**1**	**K.RSVHDVVLVGGSTR.I**
	1808	830.46	1658.90	1658.89	0.01	0	74	2.2e-07	1	R.IINEPTAAAIAYGLDK.G

---

15. HSP70_TRYCR **Mass:** 73762 **Score:** 120 **Queries matched:** 4
P05456|HSP70_TRYCR Heat shock 70 kDa protein - Trypanosoma cruzi
☐ Check to include this hit in error tolerant search or archive report

	Query	Observed	Mr(expt)	Mr(calc)	Delta	Miss	Score	Expect	Rank	Peptide
	1379	614.82	1227.62	1227.62	0.00	0	63	3.5e-06	2	R.VEIIANDQGNR.T
	1380	614.84	1227.67	1227.62	0.05	0	(9)	0.85	1	R.VEIIANDQGNR.T
☑	1487	**650.37**	**1298.72**	**1298.60**	**0.12**	**0**	**24**	**0.026**	**1**	**R.FEELCGELFR.G + Carbamidomethyl (C)**
	1808	830.46	1658.90	1658.89	0.01	0	74	2.2e-07	1	R.IINEPTAAAIAYGLDK.V

---

16. HSP70_LEIAM **Mass:** 71153 **Score:** 116 **Queries matched:** 4
Q07437|HSP70_LEIAM Heat shock 70 kDa protein - Leishmania amazonensis
☐ Check to include this hit in error tolerant search or archive report

Query	Observed	Mr(expt)	Mr(calc)	Delta	Miss	Score	Expect	Rank	Peptide
1379	614.82	1227.62	1227.62	0.00	0	63	3.5e-06	2	R.VEIIANDQGNR.T
1380	614.84	1227.67	1227.62	0.05	0	(9)	0.85	1	R.VEIIANDQGNR.T
1665	494.61	1480.81	1480.81	0.00	1	2	4.7	1	K.RSVHDVVLVGGSTR.I
1808	830.46	1658.90	1658.89	0.01	0	74	2.2e-07	1	R.IINEPTAAAIAYGLDK.G

---

17. EF1AC_PORPU **Mass:** 49142 **Score:** 113 **Queries matched:** 3
P50256|EF1AC_PORPU Elongation factor 1-alpha C - Porphyra purpurea
☐ Check to include this hit in error tolerant search or archive report

	Query	Observed	Mr(expt)	Mr(calc)	Delta	Miss	Score	Expect	Rank	Peptide
	867	488.28	974.55	974.54	0.01	0	63	4.6e-06	1	R.LPLQDVYK.I
	985	513.31	1024.61	1024.60	0.01	0	71	4.3e-07	1	K.IGGIGTVPVGR.V
☑	1338	**602.83**	**1203.65**	**1203.65**	**0.00**	**2**	**10**	**0.7**	**1**	**K.KLEDSPKMIK.S + Oxidation (M)**

---

18. EF1AS_PORPU **Mass:** 56612 **Score:** 113 **Queries matched:** 3
P50257|EF1AS_PORPU Elongation factor 1-alpha S - Porphyra purpurea
☐ Check to include this hit in error tolerant search or archive report

	Query	Observed	Mr(expt)	Mr(calc)	Delta	Miss	Score	Expect	Rank	Peptide
	867	488.28	974.55	974.54	0.01	0	63	4.6e-06	1	R.LPLQDVYK.I
	985	513.31	1024.61	1024.60	0.01	0	71	4.3e-07	1	K.IGGIGTVPVGR.V
☑	2028	**1007.97**	**2013.92**	**2014.08**	**-0.17**	**2**	**1**	**4**	**1**	**K.KTVAVGVIQCVQPRNMAK.G + Carbamidomethyl (C); Oxidation (M)**

---

19. FCA1_TRYRA **Mass:** 23105 **Score:** 112 **Queries matched:** 3
Q27052|FCA1_TRYRA Flagellar calcium-binding protein - Trypanosoma rangeli
☐ Check to include this hit in error tolerant search or archive report

Query	Observed	Mr(expt)	Mr(calc)	Delta	Miss	Score	Expect	Rank	Peptide
653	439.24	876.47	876.43	0.03	0	43	0.00038	1	K.LDEFTPR.V
1304	**592.35**	**1182.69**	**1182.51**	**0.17**	**1**	**7**	**1.2**	**5**	**-.MGACGSKGSAGNK.D + Oxidation (M)**
1662	737.90	1473.78	1473.68	0.11	0	90	7.4e-09	1	K.GSEDFVEFLEFR.L

20. TBA_TRYCR Mass: 49696 Score: 111 Queries matched: 3
Q27352|TBA_TRYCR Tubulin alpha chain - Trypanosoma cruzi
☐ Check to include this hit in error tolerant search or archive report

	Query	Observed	Mr(expt)	Mr(calc)	Delta	Miss	Score	Expect	Rank	Peptide
☑	158	566.83	1131.65	1131.56	0.09	0	11	1.2	1	K.EIVDLCLDR.I + Carbamidomethyl (C)
☑	1854	858.48	1714.94	1714.91	0.02	0	87	9.9e-09	1	R.AVFLDLEPTVVDEIR.T
☑	1855	572.66	1714.95	1714.91	0.04	0	(43)	0.00023	1	R.AVFLDLEPTVVDEIR.T

21. UBIQ_ACACA Mass: 8591 Score: 98 Queries matched: 5
P49634|UBIQ_ACACA Ubiquitin - Acanthamoeba castellanii (Amoeba)
☐ Check to include this hit in error tolerant search or archive report

Query	Observed	Mr(expt)	Mr(calc)	Delta	Miss	Score	Expect	Rank	Peptide
459	383.22	764.43	764.43	0.01	0	25	0.017	1	-.MQIFVK.T
1066	533.27	1064.53	1064.55	-0.02	0	59	7.7e-06	1	R.TLADYNIQK.E
1070	534.32	1066.62	1066.61	0.00	0	48	0.00011	1	K.ESTLHLVLR.L
1701	508.62	1522.83	1522.77	0.06	1	23	0.029	1	K.IQDKEGIPPDQQR.L
2043	1017.46	2032.91	2033.05	-0.15	1	2	3	3	K.TITLEVESSDTIENVKQK.I

22. CYC_CRION Mass: 12046 Score: 76 Queries matched: 2
P00077|CYC_CRION Cytochrome c - Crithidia oncopelti
☐ Check to include this hit in error tolerant search or archive report

	Query	Observed	Mr(expt)	Mr(calc)	Delta	Miss	Score	Expect	Rank	Peptide
	1437	631.78	1261.54	1261.69	-0.15	0	8	0.78	2	R.ADLIAYLENLK.-
☑	1465	641.79	1281.58	1281.60	-0.02	0	76	1.2e-07	1	R.HSGTVEGFAYSK.A

23. G3PG_CRIFA Mass: 39063 Score: 76 Queries matched: 3
O96423|G3PG_CRIFA Glyceraldehyde-3-phosphate dehydrogenase, glycosomal - Crithidia fasciculata
☐ Check to include this hit in error tolerant search or archive report

Query	Observed	Mr(expt)	Mr(calc)	Delta	Miss	Score	Expect	Rank	Peptide

540	406.22	810.43	810.41	0.02	0	17	0.097	2	K.LTGMSFR.V
1312	594.34	1186.66	1186.67	-0.02	1	34	0.0028	1	K.AVGMVIPSTKGK.L
1577	692.86	1383.71	1383.77	-0.06	0	59	7.3e-06	1	R.AAAVNIIPSTTGAAK.A

Proteins matching the same set of peptides:
G3PG_LEIME **Mass:** 39008 **Score:** 76 **Queries matched:** 3
Q27890|G3PG_LEIME Glyceraldehyde-3-phosphate dehydrogenase, glycosomal - Leishmania mexicana

---

24. HSP70_PYRSA **Mass:** 72035 **Score:** 74 **Queries matched:** 3
P37899|HSP70_PYRSA Heat shock 70 kDa protein - Pyrenomonas salina
☐ Check to include this hit in error tolerant search or archive report

Query	Observed	Mr(expt)	Mr(calc)	Delta	Miss	Score	Expect	Rank	Peptide
1663	**738.35**	**1474.69**	**1474.75**	**-0.07**	**2**	**0**	**6**	**2**	**K.IQEEDKKSIEEK.V**
1808	830.46	1658.90	1658.89	0.01	0	74	2.2e-07	1	R.IINEPTAAAIAYGLDK.K
2053	1025.94	2049.86	2050.05	-0.19	1	0	4.9	2	K.EVKNAVITVPAYFNDSQR.Q

---

25. RLA2_TRYCR **Mass:** 10505 **Score:** 71 **Queries matched:** 1
P23632|RLA2_TRYCR 60S acidic ribosomal protein P2-A - Trypanosoma cruzi
☐ Check to include this hit in error tolerant search or archive report

	Query	Observed	Mr(expt)	Mr(calc)	Delta	Miss	Score	Expect	Rank	Peptide
☑	1280	**586.22**	**1170.42**	**1170.49**	**-0.07**	**0**	**71**	**5.4e-07**	**1**	**K.DFDTVCTEGK.S + Carbamidomethyl (C)**

---

26. H2A_TRYCR **Mass:** 14357 **Score:** 68 **Queries matched:** 2
P35066|H2A_TRYCR Histone H2A - Trypanosoma cruzi
☐ Check to include this hit in error tolerant search or archive report

	Query	Observed	Mr(expt)	Mr(calc)	Delta	Miss	Score	Expect	Rank	Peptide
☑	1250	**578.77**	**1155.52**	**1155.56**	**-0.04**	**0**	**68**	**9.7e-07**	**1**	**R.HDDDLGMLLK.D**
☑	1744	**525.04**	**1572.10**	**1571.89**	**0.21**	**2**	**2**	**3.6**	**1**	**R.SGGRSAKAGLIFPVGR.V**

---

27. EF1A_ENTHI **Mass:** 47234 **Score:** 63 **Queries matched:** 3

P31018|EF1A_ENTHI Elongation factor 1-alpha - Entamoeba histolytica

☐ Check to include this hit in error tolerant search or archive report

	Query	Observed	Mr(expt)	Mr(calc)	Delta	Miss	Score	Expect	Rank	Peptide
	867	488.28	974.55	974.54	0.01	0	63	4.6e-06	1	R.LPLQDVYK.I
	1181	**561.34**	**1120.67**	**1120.59**	**0.08**	**0**	**6**	**1.3**	**2**	**K.YAWVLDNLK.A**
☑	1485	**649.30**	**1296.58**	**1296.61**	**-0.03**	**1**	**2**	**3.8**	**1**	**K.MDAIQYKQER.Y + Oxidation (M)**

---

28. EF1A_GIALA **Mass:** 43922 **Score:** 63 **Queries matched:** 1

Q08046|EF1A_GIALA Elongation factor 1-alpha - Giardia lamblia (Giardia intestinalis)

☐ Check to include this hit in error tolerant search or archive report

Query	Observed	Mr(expt)	Mr(calc)	Delta	Miss	Score	Expect	Rank	Peptide
867	488.28	974.55	974.54	0.01	0	63	4.6e-06	1	R.LPIQDVYK.I

---

29. HSP71_TRYCR **Mass:** 71102 **Score:** 56 **Queries matched:** 1

P20583|HSP71_TRYCR Heat shock 70 kDa protein, mitochondrial precursor - Trypanosoma cruzi

☐ Check to include this hit in error tolerant search or archive report

	Query	Observed	Mr(expt)	Mr(calc)	Delta	Miss	Score	Expect	Rank	Peptide
☑	1064	**532.77**	**1063.52**	**1063.53**	**-0.01**	**0**	**56**	**1.6e-05**	**1**	**R.VLENTEGFR.A**

---

30. CH60_EUGGR **Mass:** 59669 **Score:** 56 **Queries matched:** 2

Q39727|CH60_EUGGR Chaperonin CPN60, mitochondrial precursor - Euglena gracilis

☐ Check to include this hit in error tolerant search or archive report

	Query	Observed	Mr(expt)	Mr(calc)	Delta	Miss	Score	Expect	Rank	Peptide
	588	422.26	842.51	842.52	-0.02	0	56	1.8e-05	1	K.LSGGVAVIK.V
☑	1906	**904.02**	**1806.04**	**1805.87**	**0.16**	**0**	**8**	**0.63**	**1**	**K.TLDTELEVVEGMSLDR.G**

---

31. CH60_PORYE **Mass:** 56992 **Score:** 56 **Queries matched:** 2

Q1XDD8|CH60_PORYE 60 kDa chaperonin - Porphyra yezoensis

☐ Check to include this hit in error tolerant search or archive report

Query	Observed	Mr(expt)	Mr(calc)	Delta	Miss	Score	Expect	Rank	Peptide
588	422.26	842.51	842.52	-0.02	0	56	1.8e-05	1	K.LSGGVAVIK.V
**1123**	**545.72**	**1089.42**	**1089.60**	**-0.17**	**2**	**3**	**3.4**	**4**	**K.VKSRCEQIK.R**

---

32. TBAD_PHYPO **Mass:** 49821 **Score:** 56 **Queries matched:** 3
P50258|TBAD_PHYPO Tubulin alpha-1A chain - Physarum polycephalum (Slime mold)
☐ Check to include this hit in error tolerant search or archive report

Query	Observed	Mr(expt)	Mr(calc)	Delta	Miss	Score	Expect	Rank	Peptide
158	566.83	1131.65	1131.56	0.09	0	11	1.2	1	K.EIVDLCLDR.V + Carbamidomethyl (C)
1854	858.48	1714.94	1714.91	0.02	0	43	0.00023	2	R.AVFLDLEPTVIDEVR.T
1855	572.66	1714.95	1714.91	0.04	0	(31)	0.0036	2	R.AVFLDLEPTVIDEVR.T

---

33. G3PG_TRYBB **Mass:** 39013 **Score:** 54 **Queries matched:** 4
P22512|G3PG_TRYBB Glyceraldehyde-3-phosphate dehydrogenase, glycosomal - Trypanosoma brucei brucei
☐ Check to include this hit in error tolerant search or archive report

	Query	Observed	Mr(expt)	Mr(calc)	Delta	Miss	Score	Expect	Rank	Peptide
☑	540	406.22	810.43	810.41	0.02	0	18	0.079	1	K.LTGMAFR.V + Oxidation (M)
	1312	594.34	1186.66	1186.64	0.02	0	34	0.0028	1	K.AVGMVIPSTQGK.L
	1647	487.27	1458.80	1458.80	-0.00	2	38	0.0011	1	R.DTSIKEIDAALKR.A
	1913	604.59	1810.74	1810.81	-0.06	0	13	0.21	1	K.IVSWYDNEWGYSHR.V

---

34. EF1B_TRYCR **Mass:** 24450 **Score:** 49 **Queries matched:** 2
P34827|EF1B_TRYCR 25 kDa elongation factor 1-beta - Trypanosoma cruzi
☐ Check to include this hit in error tolerant search or archive report

	Query	Observed	Mr(expt)	Mr(calc)	Delta	Miss	Score	Expect	Rank	Peptide
☑	1695	**506.96**	**1517.86**	**1517.85**	**0.02**	**1**	**23**	**0.03**	**1**	**K.LFLGGTKPSKEDVK.L**
☑	2275	**825.69**	**2474.06**	**2474.27**	**-0.21**	**0**	**44**	**0.0002**	**1**	**K.SSILFDVKPWDDTVDLQALANK.L**

---

35. RLA1_TRYCR Mass: 10747 Score: 47 Queries matched: 1
P26643|RLA1_TRYCR 60S acidic ribosomal protein P1 - Trypanosoma cruzi
☐ Check to include this hit in error tolerant search or archive report

	Query	Observed	Mr(expt)	Mr(calc)	Delta	Miss	Score	Expect	Rank	Peptide
☑	2312	**850.43**	**2548.26**	**2548.34**	**-0.08**	**0**	**47**	**8.5e-05**	**1**	**K.VSFGGVAPAAGGATAAPAAAAAAAAPAAAAAK.K**

---

36. RLA3_TRYCR Mass: 10919 Score: 42 Queries matched: 1
P26795|RLA3_TRYCR 60S acidic ribosomal protein P2-B - Trypanosoma cruzi
☐ Check to include this hit in error tolerant search or archive report

	Query	Observed	Mr(expt)	Mr(calc)	Delta	Miss	Score	Expect	Rank	Peptide
☑	1171	**558.83**	**1115.65**	**1115.62**	**0.03**	**0**	**42**	**0.00045**	**1**	**R.SVATLVAEGAAK.M**

---

37. RL10_EUGGR Mass: 24626 Score: 39 Queries matched: 1
Q39724|RL10_EUGGR 60S ribosomal protein L10 - Euglena gracilis
☐ Check to include this hit in error tolerant search or archive report

	Query	Observed	Mr(expt)	Mr(calc)	Delta	Miss	Score	Expect	Rank	Peptide
☑	1112	**544.36**	**1086.70**	**1086.64**	**0.06**	**1**	**39**	**0.00084**	**1**	**R.VDGVSTKILR.N**

---

38. TBB_GIALA Score: 38 Queries matched: 1
P05304|TBB_GIALA Tubulin beta chain - Giardia lamblia (Giardia intestinalis)
☐ Check to include this hit in error tolerant search or archive report

Query	Observed	Mr(expt)	Mr(calc)	Delta	Miss	Score	Expect	Rank	Peptide
1773	808.43	1614.85	1614.83	0.02	0	38	0.00072	2	R.AILVDLEPGTMDSVR.A

---

39. RK32_THAPS Score: 38 Queries matched: 1
A0T0V4|RK32_THAPS Chloroplast 50S ribosomal protein L32 - Thalassiosira pseudonana (Marine diatom)
☐ Check to include this hit in error tolerant search or archive report

Query	Observed	Mr(expt)	Mr(calc)	Delta	Miss	Score	Expect	Rank	Peptide
1112	544.36	1086.70	1086.68	0.02	1	38	0.001	2	K.SLSLAKSILR.G

---

40. YCF55_PORYE **Score:** 28 **Queries matched:** 1

Q1XDT4|YCF55_PORYE Uncharacterized protein ycf55 - Porphyra yezoensis

☐ Check to include this hit in error tolerant search or archive report

Query	Observed	Mr(expt)	Mr(calc)	Delta	Miss	Score	Expect	Rank	Peptide
1379	614.82	1227.62	1227.70	-0.08	0	28	0.0096	3	K.NNLIWNTIIK.N

---

41. RK16_EMIHU **Mass:** 15623 **Score:** 27 **Queries matched:** 1

Q4G358|RK16_EMIHU Chloroplast 50S ribosomal protein L16 - Emiliania huxleyi

☐ Check to include this hit in error tolerant search or archive report

	Query	Observed	Mr(expt)	Mr(calc)	Delta	Miss	Score	Expect	Rank	Peptide
☑	1341	**603.26**	**1204.50**	**1204.62**	**-0.12**	**1**	**27**	**0.012**	**1**	**R.VFPDRSVTER.A**

---

42. NDUS1_DICCI **Mass:** 79554 **Score:** 26 **Queries matched:** 4

Q2LCP5|NDUS1_DICCI NADH-ubiquinone oxidoreductase 75 kDa subunit - Dictyostelium citrinum (Slime mo

☐ Check to include this hit in error tolerant search or archive report

	Query	Observed	Mr(expt)	Mr(calc)	Delta	Miss	Score	Expect	Rank	Peptide
☑	593	**423.72**	**845.42**	**845.36**	**0.06**	**0**	**4**	**3.1**	**1**	**R.CIHCTR.C + 2 Carbamidomethyl (C)**
☑	1501	**654.34**	**1306.67**	**1306.67**	**0.00**	**2**	**26**	**0.012**	**1**	**K.KIMEAGSWKNK.T + Oxidation (M)**
	1727	**520.27**	**1557.79**	**1557.73**	**0.06**	**0**	**4**	**2.6**	**2**	**K.GGNVLYSIDSNEYK.V**
☑	1859	**862.93**	**1723.84**	**1723.80**	**0.05**	**1**	**3**	**2.5**	**1**	**R.FKINEIECEVDEEK.E**

---

43. RPOA_EUGGR **Score:** 25 **Queries matched:** 1

P48337|RPOA_EUGGR DNA-directed RNA polymerase subunit alpha - Euglena gracilis

☐ Check to include this hit in error tolerant search or archive report

Query	Observed	Mr(expt)	Mr(calc)	Delta	Miss	Score	Expect	Rank	Peptide
1070	534.32	1066.62	1066.65	-0.04	1	25	0.018	2	K.YLKIYVLR.S

44. MDR_LEITA Mass: 172125 Score: 24 Queries matched: 2
P21441|MDR_LEITA Multidrug resistance protein - Leishmania tarentolae (Sauroleishmania tarentolae)
☐ Check to include this hit in error tolerant search or archive report

	Query	Observed	Mr(expt)	Mr(calc)	Delta	Miss	Score	Expect	Rank	Peptide
☑	435	**746.74**	**745.73**	**745.39**	**0.35**	**0**	**5**	**2.7**	**1**	**R.TYHIGR.K**
	1634	725.33	1448.65	1448.78	-0.13	2	24	0.024	2	R.QLMCMARALLKR.G + Oxidation (M)

45. HSP71_LEIMA Mass: 68288 Score: 23 Queries matched: 2
P12076|HSP71_LEIMA Heat shock 70-related protein 1, mitochondrial precursor - Leishmania major
☐ Check to include this hit in error tolerant search or archive report

	Query	Observed	Mr(expt)	Mr(calc)	Delta	Miss	Score	Expect	Rank	Peptide
☑	807	**475.27**	**948.52**	**948.53**	**-0.00**	**0**	**23**	**0.029**	**1**	**R.TTPSVVAFK.G**
☑	361	**692.72**	**1383.43**	**1383.64**	**-0.20**	**1**	**3**	**8.7**	**1**	**R.DSEQHAEADRVK.R**

46. RPB1A_TRYBB Mass: 196330 Score: 22 Queries matched: 4
P17546|RPB1A_TRYBB DNA-directed RNA polymerase II subunit RPB1-A - Trypanosoma brucei brucei
☐ Check to include this hit in error tolerant search or archive report

	Query	Observed	Mr(expt)	Mr(calc)	Delta	Miss	Score	Expect	Rank	Peptide
	1265	**583.31**	**1164.61**	**1164.67**	**-0.06**	**1**	**2**	**2.9**	**7**	**K.KLQHVTGLNR.L**
☑	1680	**750.92**	**1499.83**	**1499.85**	**-0.02**	**2**	**22**	**0.037**	**1**	**K.ALDMKDVKQAILR.V**
☑	1681	**500.98**	**1499.93**	**1499.85**	**0.08**	**2**	**(9)**	**0.78**	**1**	**K.ALDMKDVKQAILR.V**
☑	466	**770.83**	**2309.47**	**2310.05**	**-0.58**	**1**	**0**	**14**	**1**	**R.TLPHFMLDDYGETSRGMANR.G**

47. RPOC2_CYAPA Score: 22 Queries matched: 4
P48120|RPOC2_CYAPA DNA-directed RNA polymerase subunit beta'' - Cyanophora paradoxa
☐ Check to include this hit in error tolerant search or archive report

Query	Observed	Mr(expt)	Mr(calc)	Delta	Miss	Score	Expect	Rank	Peptide

856	**487.25**	**972.48**	**972.52**	**-0.05**	**0**	**9**	**0.57**	**2**	**K.IQNETQLK.H**
1039	**526.31**	**1050.60**	**1050.51**	**0.09**	**0**	**4**	**1.9**	**2**	**K.DLGFHYATK.A**
1169	557.79	1113.57	1113.68	-0.11	1	10	0.56	2	K.ENGKILISLK.D
1773	808.43	1614.85	1614.93	-0.08	2	22	0.029	3	K.IRNKHSGVVELVHK.K

---

48. RPOC2_PHATR **Mass:** 162927 **Score:** 22 **Queries matched:** 3
A0T0D9|RPOC2_PHATR DNA-directed RNA polymerase subunit beta'' - Phaeodactylum tricornutum
☐ Check to include this hit in error tolerant search or archive report

	Query	Observed	Mr(expt)	Mr(calc)	Delta	Miss	Score	Expect	Rank	Peptide
☑	220	**601.34**	**600.34**	**600.40**	**-0.06**	**0**	**6**	**1.8**	**1**	**K.TVLLR.T**
☑	570	**414.76**	**827.51**	**827.49**	**0.03**	**1**	**4**	**2.4**	**1**	**R.VSPRLEK.N**
☑	1052	**529.83**	**1057.65**	**1057.64**	**0.01**	**1**	**22**	**0.041**	**1**	**K.LIDLKISEK.F**

Peptide matches not assigned to protein hits: (no details means no match)

	Query	Observed	Mr(expt)	Mr(calc)	Delta	Miss	Score	Expect	Rank	Peptide
☑	774	**468.24**	**934.46**	**934.51**	**-0.05**	**1**	**21**	**0.062**	**1**	**VISKWMR + Oxidation (M)**
☑	1000	**516.30**	**1030.58**	**1030.61**	**-0.02**	**0**	**21**	**0.051**	**1**	**VIDLLAPYK**
☑	1567	**691.30**	**1380.59**	**1380.69**	**-0.11**	**1**	**20**	**0.07**	**1**	**ELESKGYEWLK**
☑	1109	**543.84**	**1085.67**	**1085.61**	**0.07**	**0**	**19**	**0.083**	**1**	**QAQALSEVLK**
☑	53	**459.25**	**916.49**	**916.50**	**-0.00**	**0**	**17**	**0.27**	**1**	**VLVYHMR**
☑	1437	631.78	1261.54	1261.64	-0.10	0	17	0.12	1	AMVVLSNTGAGAR + Oxidation (M)
☑	1304	592.35	1182.69	1182.68	0.01	0	16	0.15	1	ILNLLCVPGNK
☑	1397	**619.81**	**1237.61**	**1237.68**	**-0.06**	**0**	**16**	**0.15**	**1**	**LASISDIHINR**
☑	1780	**811.86**	**1621.70**	**1621.90**	**-0.19**	**0**	**16**	**0.13**	**1**	**YGLPLIFEIETISK**
☑	1091	**539.75**	**1077.48**	**1077.49**	**-0.01**	**0**	**16**	**0.16**	**1**	**VLCMGVGGGNV + Carbamidomethyl (C); Oxidation (M)**
☑	1265	583.31	1164.61	1164.67	-0.06	2	16	0.13	1	KIHEQNKIR
☑	1446	**633.32**	**1264.62**	**1264.67**	**-0.05**	**0**	**16**	**0.2**	**1**	**NTIVTENFISK**
☑	359	692.39	691.39	691.39	-0.00	0	16	0.52	1	VAFLDK
☑	1575	**462.20**	**1383.58**	**1383.72**	**-0.14**	**1**	**15**	**0.17**	**1**	**HGIEKVAEQVMK + Oxidation (M)**
☑	1472	**646.32**	**1290.63**	**1290.70**	**-0.07**	**1**	**15**	**0.18**	**1**	**EPKYIPIFER**
☑	1469	**643.84**	**1285.66**	**1285.66**	**-0.00**	**1**	**15**	**0.24**	**1**	**MIHIGNNTKSR + Oxidation (M)**
☑	1612	**714.39**	**1426.77**	**1426.73**	**0.04**	**0**	**15**	**0.21**	**1**	**LIIQIGCYSDFR**

☑	765	465.78	929.55	929.55	-0.00	1	15	0.24	1	ILDSKNLK
☑	1548	679.80	1357.58	1357.70	-0.12	0	14	0.2	1	CALIEAAQAANVK + Carbamidomethyl (C)
☑	1561	688.34	1374.66	1374.72	-0.06	0	14	0.24	1	ENMELGLTVTLR
☑	1748	527.29	1578.84	1578.88	-0.04	1	14	0.2	1	TNSVTAGPIPLPTRR
☑	1298	590.86	1179.71	1179.73	-0.03	2	14	0.2	1	VKIITPPERK
☑	1282	586.34	1170.66	1170.61	0.04	1	14	0.29	1	DTSLRVPNGGR
☑	701	453.25	904.49	904.46	0.03	0	14	0.36	1	ASDVSTAVR
☑	781	469.76	937.50	937.40	0.11	0	14	0.25	1	MPEGEYGR
☑	1155	553.30	1104.58	1104.55	0.03	0	13	0.32	1	LLEAEEVMR + Oxidation (M)
☑	1896	896.02	1790.02	1790.02	0.01	2	13	0.25	1	SFALTREASLRTLGLR
☑	1181	561.34	1120.67	1120.66	0.01	2	13	0.26	1	RNRHNIIAK
☑	1158	553.76	1105.51	1105.56	-0.05	0	13	0.3	1	NMLLYPSPR + Oxidation (M)
☑	1649	730.90	1459.79	1459.71	0.08	1	13	0.37	1	MEKEMMLYLLK + 2 Oxidation (M)
☑	1611	714.39	1426.76	1426.73	0.03	0	12	0.37	1	LIIQIGCYSDFR
☑	791	471.78	941.55	941.55	-0.00	0	12	0.4	1	ELIGILER
☑	1654	734.81	1467.61	1467.86	-0.25	2	12	0.39	1	ALSGGQKQRIAIAR
☑	1360	609.30	1216.59	1216.69	-0.10	1	12	0.44	1	GGKIGLFGGAGVGK
☑	1885	886.95	1771.88	1772.00	-0.12	1	12	0.35	1	RIIENTGISSSIIIEK
☑	975	510.76	1019.51	1019.59	-0.08	2	12	0.5	1	RMSTKLIR + Oxidation (M)
☑	1872	585.35	1753.02	1752.86	0.16	1	12	0.35	1	SFWGPPHGIEVERDK
☑	1756	530.97	1589.89	1589.85	0.04	1	11	0.44	1	KYLSVNSNYVLYK
☑	1410	621.91	1241.81	1241.70	0.12	0	11	0.41	1	GLNLGSALEGALK
☑	46	438.59	1312.75	1312.72	0.03	0	11	1	1	TTLHLVASCLQK
☑	856	487.25	972.48	972.58	-0.10	1	11	0.38	1	ELLPFAKR
☑	1629	482.01	1443.02	1442.85	0.17	0	11	0.54	1	VLSQLGSSLVTALR
☑	1191	564.33	1126.65	1126.62	0.03	0	11	0.48	1	TLPGVAVLDDK
☑	1476	647.43	1292.84	1292.71	0.13	0	11	0.49	1	NNPVLIGEPGVGK
☑	1017	519.30	1036.58	1036.63	-0.04	2	11	0.4	1	TLIRKNHR
☑	1123	545.72	1089.42	1089.62	-0.20	2	11	0.63	1	MAADLLKGKK + Oxidation (M)
☑	1663	738.35	1474.69	1474.72	-0.03	0	11	0.53	1	FIDCYLQFLEK + Carbamidomethyl (C)
☑	132	550.85	1099.69	1099.66	0.03	1	10	1.4	1	ILKTGGAELAK
☑	1320	596.82	1191.63	1191.53	0.10	0	10	0.46	1	CVNASTANAER + Carbamidomethyl (C)
☑	1088	539.48	1076.94	1076.42	0.52	0	10	0.82	1	MCMSPSEHR
☑	1940	919.47	1836.93	1836.91	0.02	2	10	0.6	1	GHSLADPDELRSRQEK

☑	1752	793.42	1584.82	1584.76	0.05	2	10	0.65	1	FEEPPSPREGERR
☑	1596	471.46	1411.35	1411.70	-0.35	1	10	0.76	1	NLELMREHQAR + Oxidation (M)
☑	1762	800.86	1599.70	1599.84	-0.14	0	10	0.55	1	IAGVSHEFSTINGIR
☑	1030	523.30	1044.60	1044.63	-0.03	1	10	0.83	1	SLSALAKSIR
☑	229	606.12	605.12	605.32	-0.20	0	10	0.6	1	ISGAMK
☑	994	515.29	1028.57	1028.61	-0.04	2	10	0.57	1	NLGNSIRKK
☑	101	521.23	1040.45	1040.57	-0.12	1	10	1.4	1	TPRGAAALER
☑	546	814.88	813.88	814.47	-0.59	1	10	2.8	1	DQIVKGR
☑	1782	542.29	1623.85	1623.95	-0.10	2	10	0.59	1	SKLVVISANCPPIRK
☑	1635	725.38	1448.75	1448.68	0.07	1	10	0.63	1	KFEEIDSAPEER
☑	566	413.73	825.45	825.54	-0.10	0	9	0.49	1	LLAQILR
☑	1039	526.31	1050.60	1050.44	0.16	0	9	0.52	1	GDINCSETR + Carbamidomethyl (C)
☑	1608	712.35	1422.68	1422.75	-0.08	1	9	0.81	1	KTVCQFTGEILK + Carbamidomethyl (C)
☑	1642	727.84	1453.66	1453.83	-0.17	2	9	0.63	1	DGKRVDTVIGAVPK
☑	1571	461.23	1380.68	1380.69	-0.01	1	9	0.76	1	WGKDVGCAFLTGK
☑	1163	555.32	1108.62	1108.54	0.08	0	9	0.6	1	AVAMSSTEGLK + Oxidation (M)
☑	209	594.79	1187.56	1187.61	-0.06	1	9	2.5	1	QLDTEGRIEK
☑	940	504.24	1006.47	1006.48	-0.01	0	9	0.82	1	ESNEIMLR + Oxidation (M)
☑	1538	674.88	1347.76	1347.71	0.05	1	9	0.77	1	SVEVCDGLIKSAK
☑	713	910.39	2728.16	2728.26	-0.10	0	9	1.9	1	LCEHIQGFVPEQLSEAMCIASHMR
☑	1450	634.01	1266.00	1265.61	0.39	0	9	0.83	1	TAVESACMILR + Carbamidomethyl (C); Oxidation (M)
☑	1012	518.28	1034.56	1034.56	-0.00	0	9	0.81	1	MDLFLQIR
☑	978	511.73	1021.45	1021.46	-0.01	0	9	0.53	1	METLPESSL + Oxidation (M)
☑	189	588.27	587.26	587.33	-0.07	0	9	2.7	1	AIAGEK
☑	592	846.42	845.42	845.51	-0.09	2	9	1.1	1	RLSSKQK
☑	979	511.79	1021.57	1021.46	0.10	0	9	0.53	1	METLPESSL + Oxidation (M)
☑	1679	750.34	1498.67	1498.70	-0.04	2	9	0.77	1	ECKTCSLTETKEK
☑	51	450.79	899.56	899.48	0.08	0	9	1.8	1	VANQQVNK
☑	999	516.29	1030.57	1030.54	0.02	1	9	0.86	1	LADAEASAKR
☑	293	641.36	640.35	640.29	0.06	0	9	0.42	1	MSYPK + Oxidation (M)
☑	1475	647.31	1292.61	1292.66	-0.05	1	9	0.8	1	LMKSAIGEGMTR
☑	1525	667.91	1333.81	1333.59	0.22	1	9	0.77	1	FVRTECMNYR + Oxidation (M)
☑	1719	773.43	1544.84	1544.78	0.06	0	9	0.9	1	HSCLIINFESNLR
☑	76	498.54	995.06	995.50	-0.44	1	9	2	1	IGDKSFSSR

☑	135	552.71	1103.41	1103.63	-0.21	0	9	2.2	1	MSLAIAEILK + Oxidation (M)
☑	198	592.78	1775.32	1774.95	0.37	2	9	3.5	1	LQVKELDLKTANMEK + Oxidation (M)
☑	1451	634.33	1266.64	1266.80	-0.16	0	8	0.91	1	VIILQTRPLSK
☑	1558	686.32	1370.62	1370.75	-0.13	1	8	0.99	1	KVDLHAYIWAR
☑	747	462.76	923.50	923.46	0.04	0	8	0.83	1	GESIYLDK
☑	1812	831.45	1660.88	1660.74	0.14	0	8	0.87	1	LIHFMIDEDDEER
☑	1264	389.20	1164.59	1164.62	-0.03	0	8	0.73	1	QFVPLAFTDK
☑	1219	571.25	1140.48	1140.59	-0.12	1	8	0.66	1	LASFYERAGK
☑	1417	623.85	1245.68	1245.76	-0.08	1	8	0.7	1	MKILKPITFR
☑	1936	612.92	1835.75	1835.97	-0.22	1	8	0.97	1	SFDLDVLKDSCALILK + Carbamidomethyl (C)
☑	718	911.44	910.43	910.52	-0.09	0	8	0.63	1	HNVSTIIK
☑	1631	723.93	1445.84	1445.80	0.04	0	8	0.9	1	LYPQEFVLKPGR
☑	1572	691.97	1381.93	1381.76	0.17	0	8	0.94	1	IIEIANINPETR
☑	1001	516.31	1030.60	1030.54	0.06	1	8	1.1	1	LADAEASAKR
☑	1511	660.29	1318.56	1318.71	-0.16	0	8	1	1	AIASSPSEGIFIK
☑	1299	591.76	1181.51	1181.72	-0.22	2	8	0.93	1	SGQKVRILGPK
☑	690	899.42	898.41	898.52	-0.11	1	8	1.1	1	EGKVTPIR
☑	1186	562.81	1123.61	1123.57	0.04	0	8	1	1	VCFGITDTIR
☑	2360	905.65	2713.92	2714.22	-0.31	1	8	1.3	1	GNVMLLENVRFYSEENGENAEER + Oxidation (M)
☑	394	717.66	2149.97	2150.11	-0.13	1	8	4.1	1	EIIENACSTAATIVALRTMK + Oxidation (M)
☑	70	489.94	977.86	977.46	0.40	0	8	2.3	1	VQNEFDAR
☑	1356	608.33	1214.64	1214.67	-0.03	1	7	1.1	1	GGQIVRAAGTSAK
☑	1079	536.79	1071.57	1071.63	-0.06	2	7	1.4	1	KSRYLLHR
☑	1363	610.78	1219.54	1219.67	-0.12	1	7	0.95	1	SGGNLIAYAKAR
☑	1592	703.85	1405.69	1405.65	0.04	1	7	1.1	1	DEIERMVNDASK
☑	195	589.52	588.51	588.31	0.20	0	7	1.6	1	DLVDK
☑	1134	547.79	1093.56	1093.58	-0.02	1	7	1	1	ELKVDFSTR
☑	1761	799.97	1597.92	1597.86	0.06	0	7	1.2	1	EMAINLFYPFIIK
☑	457	764.59	763.58	763.38	0.20	0	7	4.2	1	IMDEIK + Oxidation (M)
☑	1197	565.81	1129.61	1129.45	0.16	2	7	1.3	1	MNKEKCCCR + Oxidation (M)
☑	1524	667.89	1333.76	1333.75	0.01	2	7	1.1	1	SRVVYQIKGER
☑	1209	568.79	1135.58	1135.63	-0.06	2	7	1.2	1	RVLRGQDHR
☑	1599	708.33	1414.65	1414.76	-0.11	1	7	1.3	1	IALLHYTDGEKR
☑	1581	695.36	1388.70	1388.68	0.02	0	7	1.2	1	ENLLPNASTSESK

☑	1449	633.81	1265.60	1265.67	-0.08	1	7	1	1 LSKHEPIADTR
☑	44	430.50	858.98	858.48	0.50	1	7	4.8	1 EAIKEAAK
☑	1314	594.83	1187.65	1187.56	0.09	1	7	1.4	1 KLGEEPQECR
☑	349	684.42	683.41	683.33	0.08	0	7	0.69	1 GMGYLK + Oxidation (M)
☑	1125	545.84	1089.67	1089.56	0.11	1	7	1.6	1 AKVCGWIEGK
☑	1399	620.26	1238.50	1238.61	-0.10	2	7	1.2	1 AKTACPKSDYR
☑	414	368.23	734.45	734.43	0.02	1	7	1.5	1 KFVSVVG
☑	1517	663.28	1324.54	1324.60	-0.06	0	6	1.9	1 SSCSIGLSECGLL + Carbamidomethyl (C)
☑	951	507.28	1012.55	1012.61	-0.07	2	6	1.2	1 KERQLALR
☑	1337	602.40	1202.79	1202.61	0.18	0	6	1.9	1 IQNIVGSCDVR
☑	865	488.26	974.51	974.50	0.01	1	6	1.9	1 AQKESGEVK
☑	1335	602.31	1202.61	1202.60	0.02	1	6	2	1 KINESMEPQK
☑	118	539.53	1077.05	1077.48	-0.43	1	6	4.7	1 GKSPESECNK
☑	307	650.27	649.27	649.38	-0.11	1	6	1.8	1 KFIDK
☑	1800	826.16	1650.30	1650.80	-0.50	0	6	2.2	1 TCLGAAAGGNTVNEFVK
☑	1118	545.27	1088.54	1088.59	-0.06	1	6	1.5	1 QGAQTTRTVK
☑	1495	651.84	1301.67	1301.66	0.00	0	6	1.8	1 QSVNEPMITGVK
☑	599	849.90	848.90	848.40	0.50	1	6	3.2	1 MKTPCGR + Carbamidomethyl (C)
☑	1727	520.27	1557.79	1557.77	0.02	0	6	1.5	1 DITPLPYNGCRPR + Carbamidomethyl (C)
☑	323	660.77	659.76	659.39	0.37	0	6	4	1 SIDVVK
☑	253	621.11	1860.31	1860.89	-0.58	2	6	5.7	1 RTASTAEGPAAESKESGGR
☑	869	489.31	976.60	976.57	0.03	0	6	2	1 TILYQIAR
☑	1765	804.35	1606.68	1606.81	-0.14	1	6	1.3	1 ILKSNFDNEVCLR + Carbamidomethyl (C)
☑	263	626.48	1876.42	1876.94	-0.52	1	6	5	1 FSPELAAACEVWKEIK + Carbamidomethyl (C)
☑	675	444.31	886.60	886.52	0.08	0	6	2	1 ILAQSSLR
☑	274	629.28	1256.55	1256.77	-0.22	1	6	5.4	1 ILLINSKSLEK
☑	1220	571.25	1140.48	1140.57	-0.09	2	6	1.2	1 KDSTTDSKLF
☑	1938	612.95	1835.82	1835.97	-0.15	1	6	1.7	1 SFDLDVLKDSCALILK + Carbamidomethyl (C)
☑	1038	526.28	1050.54	1050.54	0.00	0	6	1.3	1 ISSMISELR + Oxidation (M)
☑	1499	653.82	1305.62	1305.65	-0.03	1	6	1.4	1 KHYDFQIAER
☑	2083	701.70	2102.09	2102.02	0.07	0	6	1.9	1 FGEMEVWALEGFGAAFTLK
☑	641	436.51	871.01	870.53	0.48	2	5	3.6	1 AAAKKASPK
☑	2001	656.97	1967.87	1967.96	-0.09	0	5	1.2	1 MSVQTSQQTVNLQTEFK
☑	1639	726.84	1451.67	1451.74	-0.07	2	5	1.4	1 YHVEPRRGEGPR

☑	69	489.83	488.82	488.30	0.53	0	5	4	1	GVSVK
☑	1490	650.73	1299.45	1299.71	-0.26	1	5	1.9	1	CNGVLEGIRIAR
☑	1532	672.85	1343.69	1343.74	-0.06	1	5	1.8	1	KIDLETIQAWK
☑	547	408.20	814.40	814.49	-0.10	1	5	3	1	AQLIDKK
☑	126	545.01	544.00	544.32	-0.32	0	5	6.4	1	ELVGK
☑	1618	718.87	1435.73	1435.58	0.15	1	5	1.6	1	NMMADCDPRNGR + Carbamidomethyl (C)
☑	1570	461.23	1380.65	1380.71	-0.06	2	5	2.1	1	TPRYGIEFRDK
☑	1594	704.80	1407.59	1407.78	-0.18	0	5	1.7	1	YSNPYVLLVVNK
☑	328	665.22	664.22	664.38	-0.16	0	5	2	1	FLTTAI
☑	1559	686.32	1370.62	1370.60	0.02	2	5	2.3	1	DKTAAGMMDCKK + Carbamidomethyl (C); Oxidation (M)
☑	1116	544.74	1087.47	1087.57	-0.10	0	5	1.8	1	TPVSLDMLGR
☑	1522	665.40	1328.79	1328.79	-0.00	2	5	1.9	1	IVSRRSNGLTVK
☑	1668	742.64	1483.27	1482.88	0.39	2	5	4.1	1	RIIEKNIELLDK
☑	1724	519.08	1554.23	1553.86	0.37	2	5	2.9	1	RNNLQWFRVPPK
☑	1528	446.92	1337.75	1337.58	0.17	0	5	1.7	1	ELISNSSDACDK + Carbamidomethyl (C)
☑	1547	679.33	1356.65	1356.65	0.00	1	5	2.3	1	WRFYTEQEAK
☑	1492	650.86	1299.71	1299.78	-0.07	2	5	2.2	1	ELLKVIKSQDK
☑	396	719.70	718.69	718.35	0.34	1	5	6.5	1	ERVCGR
☑	1453	634.82	1267.63	1267.63	0.00	1	5	2.5	1	EKMVQVMFEK
☑	1844	568.23	1701.67	1701.88	-0.21	1	5	1.9	1	LPRFQGTATNVNQEK
☑	271	628.38	1254.74	1254.72	0.03	1	5	5.5	1	TLPGVAILTDKQ
☑	1378	614.65	1227.30	1227.68	-0.38	1	5	3.2	1	ARLVSTPTMPR
☑	80	502.89	1003.76	1003.44	0.32	1	5	5.5	1	ENSDNRNR
☑	412	733.88	2198.63	2198.07	0.56	1	4	8.7	1	TDSLCTLNMVPGISVYGEKR + Oxidation (M)
☑	1853	572.00	1712.97	1712.79	0.18	1	4	1.4	1	LIEFEMIMDDNRR + 2 Oxidation (M)
☑	331	668.81	1335.60	1335.63	-0.04	1	4	5.8	1	IMGETDEQSAKK
☑	892	494.79	987.56	987.52	0.04	0	4	2.6	1	NILEGSEVK
☑	934	502.73	1003.45	1003.58	-0.13	0	4	2.4	1	YKPGTVALR
☑	1523	665.85	1329.68	1329.71	-0.03	1	4	2.2	1	VMDIRVLTPDR + Oxidation (M)
☑	1787	816.38	1630.75	1630.85	-0.10	0	4	2.2	1	DGVPVPTIITVYDDK
☑	180	581.48	1741.41	1741.92	-0.51	2	4	7.6	1	QKAYNAYIKCVSLGK + Carbamidomethyl (C)
☑	2301	844.73	2531.17	2531.19	-0.02	2	4	2.1	1	HPGIMVGMDQKDAYVGDEAQSKR
☑	1874	878.89	1755.77	1756.01	-0.24	2	4	1.9	1	KTQDSIAVLKQTTPVK
☑	637	435.28	868.54	868.54	0.01	0	4	1.7	1	QPVITALK

☑	1602	708.92	1415.82	1415.77	0.05	1	4	2	1	GISISEAAAVRSAGK
☑	917	996.91	995.90	995.50	0.40	1	4	2.7	1	NGSGKSTFAK
☑	221	601.39	1801.15	1800.90	0.25	2	4	7.3	1	EGNTVLSYPDKKDHAK
☑	1569	691.32	1380.62	1380.68	-0.06	0	4	2.6	1	TLTVEDTGIGMTK + Oxidation (M)
☑	282	637.85	636.84	636.35	0.49	0	4	3.5	1	DYIVK
☑	222	602.29	1202.57	1202.63	-0.06	2	4	9.3	1	ELKKAETNDR
☑	1396	619.78	1237.55	1237.53	0.02	0	4	2.3	1	NLDMSICEADK
☑	2105	1070.53	2139.04	2139.13	-0.08	1	4	1.9	1	VSSGIGFLDHMLTALAKHSR
☑	319	657.89	656.88	657.42	-0.54	0	4	5.8	1	GIITVR
☑	249	619.87	618.86	619.35	-0.49	0	4	5.8	1	LSSSVK
☑	419	738.64	2212.90	2213.18	-0.28	2	4	8.7	1	KTNESASRIFPVALNVDQPK
☑	1638	726.67	1451.34	1450.78	0.55	0	4	3.2	1	IFSGINAAFVDLGK
☑	136	554.26	1106.51	1106.61	-0.10	2	4	5.1	1	GKDKLEVYR
☑	993	515.28	1028.54	1028.59	-0.05	1	4	2.3	1	ILPTNKESK
☑	1458	638.29	1274.57	1274.68	-0.11	1	4	2.5	1	DGLIDTAVKTSR
☑	1452	423.22	1266.64	1266.67	-0.02	2	4	2.8	1	GISAGKSKNSYR
☑	41	418.04	834.07	834.53	-0.47	2	4	8.8	1	AKTFKLK
☑	1718	772.37	1542.72	1542.75	-0.03	1	4	2.3	1	MAPPASASKAGAAEER
☑	141	557.39	1669.15	1668.70	0.45	0	3	5.7	1	MCNLSVYYFECPK + Carbamidomethyl (C); Oxidation (M)
☑	1354	607.39	1212.77	1212.68	0.09	0	3	2.3	1	MIGPILEEIAK
☑	36	396.11	790.20	790.47	-0.27	0	3	6.7	1	AAYVLVR
☑	936	502.78	1003.55	1003.50	0.04	1	3	3	1	LSQTDRER
☑	1628	722.37	1442.73	1442.73	0.01	0	3	3	1	NQFISNVSHELR
☑	1468	643.84	1285.66	1285.74	-0.08	1	3	3.5	1	IEQTNKAITLR
☑	1521	665.36	1328.71	1328.67	0.04	1	3	2.8	1	DLVRLDNNADGK
☑	1568	691.32	1380.62	1380.69	-0.08	1	3	3.2	1	ELESKGYEWLK
☑	231	606.98	605.98	605.38	0.60	0	3	7.5	1	IITFI
☑	372	699.52	2095.53	2096.08	-0.55	0	3	8.1	1	MNVGQIFECILGISAFNLK
☑	1537	674.87	1347.73	1347.78	-0.04	1	3	3	1	ADLTKFIVVTNK
☑	2023	670.36	2008.07	2007.89	0.17	2	3	2.2	1	DDYGAPLCPCRHYENKK
☑	1063	532.75	1063.49	1063.64	-0.15	0	3	3.6	1	VVDLIAHVAK
☑	905	992.49	1982.96	1982.81	0.15	1	3	8.7	1	ECPCTREAQIPSCCSSR + 2 Carbamidomethyl (C)
☑	1923	607.33	1818.95	1819.01	-0.06	2	3	2.9	1	VLLESKKILYENENK
☑	93	519.13	1554.36	1554.85	-0.49	1	3	8.3	1	MSDILFQLGYIKK

☑	102	521.78	1562.33	1561.88	0.45	2	3	8.5	1	KLRLFTSGILGDDK
☑	673	443.83	885.66	885.54	0.12	1	3	3.4	1	IQAIRGTK
☑	190	588.33	587.32	587.36	-0.04	0	3	4.7	1	TIINK
☑	469	772.39	771.38	771.47	-0.09	1	3	8.4	1	IRISQR
☑	519	802.09	801.08	801.45	-0.37	0	3	12	1	ASGLLWR
☑	1886	886.96	1771.91	1772.00	-0.09	1	3	2.7	1	RIIENTGISSSIIIEK
☑	26	352.30	1053.86	1053.59	0.27	1	3	6.5	1	EDLIRVGPR
☑	67	485.95	1454.84	1454.76	0.08	2	3	6.6	1	IAAEKWGGGKIYY
☑	1436	631.27	1260.53	1260.63	-0.10	1	3	4	1	MDELVNWARK
☑	2043	1017.46	2032.91	2033.01	-0.11	2	3	2.6	1	IAPMEQRKLQSSMNVNR + 2 Oxidation (M)
☑	1172	1117.39	3349.15	3348.62	0.52	1	3	9	1	TGKDIIIAALMGAEEFGFGTVAMIATGCVMAR + Carbamidomethyl (C); 3 Oxidation (M
☑	1981	641.37	1921.09	1920.99	0.10	2	3	2.8	1	IGGVMSMGDRGTGKSTIVR
☑	83	508.79	1015.56	1015.39	0.17	0	3	8.7	1	DPFEEHEN
☑	1694	758.99	1515.97	1515.72	0.24	0	3	3.2	1	YTDNYIEPLNFK
☑	2355	902.43	2704.28	2704.24	0.04	2	3	2.5	1	GHYLNVTAGNCEEMYKRADYAVK + Carbamidomethyl (C); Oxidation (M)
☑	186	583.83	1748.47	1748.96	-0.49	1	3	8.7	1	DIAKAYGLSLLELESK
☑	346	681.96	1361.90	1361.69	0.21	0	3	9.9	1	AVMDAGVEASVVSK
☑	355	690.23	689.23	689.30	-0.07	0	3	11	1	QNDEGK
☑	1734	783.92	1565.82	1565.79	0.03	1	3	2.8	1	TRLNFTATCSIDPK
☑	1674	744.84	1487.66	1487.73	-0.07	0	3	3.5	1	LEEIEDMLINNR
☑	1720	775.83	1549.65	1549.87	-0.23	1	3	3.2	1	EVKLALTLNSAYTK
☑	1187	563.31	1124.60	1124.54	0.06	0	3	2.5	1	AVSMSSTEGLK + Oxidation (M)
☑	1533	672.89	1343.76	1343.70	0.05	1	3	3.4	1	SKEVSPNLSQQK
☑	1494	651.82	1301.62	1301.73	-0.11	2	3	4	1	VGSKELEASKVR
☑	2317	852.61	2554.81	2555.28	-0.47	2	3	4.3	1	EAGGITQKIGAYEVEIDYKDQTK
☑	1670	743.83	1485.65	1485.64	0.01	1	2	3.2	1	MHGHAEACMRVR + Carbamidomethyl (C); 2 Oxidation (M)
☑	655	878.55	2632.62	2632.38	0.24	2	2	8.7	1	YCAGLADKVNGTVPPRSGNFLGIVK + Carbamidomethyl (C)
☑	837	483.27	964.53	964.52	0.01	1	2	3.4	1	YLGEKVEK
☑	170	575.27	574.26	574.28	-0.02	0	2	4.6	1	GNTQR
☑	199	592.89	1183.76	1183.72	0.04	2	2	12	1	GTKIMRILPR
☑	2007	991.13	1980.25	1979.86	0.39	0	2	3.8	1	GSYLNVTAATMEEMYER + Oxidation (M)
☑	1779	541.27	1620.79	1620.74	0.04	1	2	3.3	1	SITFSSDGGCAQHRR
☑	347	682.28	2043.81	2044.10	-0.30	2	2	8.9	1	VSIYLKYKQDMTSLLSR
☑	348	683.78	2048.31	2048.07	0.24	0	2	9.1	1	AAELVGEYVAQQALNFGIR

1717	771.94	1541.87	1541.91	-0.04	2	2	3.1	1	AVETLKQKGLVSAAK
1676	747.34	1492.66	1492.84	-0.17	1	2	2.8	1	ILSMVKTVNMTIK + Oxidation (M)
1790	818.35	1634.69	1634.83	-0.14	1	2	2.9	1	MVSSLANELREGTTK
1753	793.77	1585.53	1585.79	-0.27	1	2	3	1	QIQEGLGKDAAEEAK
1949	927.15	1852.29	1852.02	0.27	0	2	4.5	1	AYFGSITVLIGLPTCIK + Carbamidomethyl (C)
75	497.29	1488.86	1488.71	0.14	1	2	7.3	1	DPLEDDLSNLCKK
1770	807.11	1612.20	1612.78	-0.58	1	2	4.3	1	KVTAELGGGDSMFASK + Oxidation (M)
1597	707.26	1412.51	1412.80	-0.29	1	2	3.1	1	SAGQAAKEVVALAAK
652	877.36	2629.05	2628.47	0.58	2	2	11	1	VSLSTRVAQWPGAQLGRLLPHVLM
1655	490.25	1467.73	1467.71	0.02	1	2	3.7	1	ENMLGKTVNFSGR + Oxidation (M)
2012	664.44	1990.31	1990.10	0.20	2	2	3.1	1	AVGKVIPSLNGKLTGMSFR + Oxidation (M)
1576	692.82	1383.62	1383.79	-0.17	2	2	3.5	1	MLLNLFKFSKK + Oxidation (M)
1210	1138.34	2274.67	2275.07	-0.41	2	2	11	1	RSDCFTKYNTCVLLQNQDK
2037	675.97	2024.89	2025.00	-0.11	0	2	2.9	1	VLASYGDIFVSDAFGTAHR
2359	905.07	2712.18	2712.41	-0.22	2	2	3.1	1	KSVCEPVQTGITAIDSMIPIGRGQR + Carbamidomethyl (C)
2367	914.84	2741.50	2741.37	0.13	2	2	3	1	GTNPEECNPQFVAELARVATIRNR + Carbamidomethyl (C)
1721	776.84	1551.67	1551.84	-0.18	0	2	3.9	1	GMVLAKPGSITPHTK + Oxidation (M)
822	477.71	953.40	953.59	-0.19	0	2	3.5	1	IKPTPGLTK
1405	621.26	1240.50	1240.73	-0.23	2	2	3.4	1	ICTKAIKSLHK
2337	877.09	2628.26	2628.41	-0.15	2	2	2.6	1	LHGLCRTLVSNLVQGVFQGFERR
1509	658.35	1314.69	1314.69	0.00	2	2	4.2	1	ILNEARNDKDK
1995	655.07	1962.19	1961.95	0.25	1	2	4.2	1	NMNLFSHITECEKLAR + Carbamidomethyl (C)
374	699.91	1397.81	1397.78	0.03	1	2	14	1	DIELVTKQDPIK
1650	731.87	1461.72	1461.71	0.01	1	2	3.3	1	MLCPPDVAFEKR + Carbamidomethyl (C)
129	549.67	1646.00	1645.92	0.09	1	2	11	1	KSSNIQNILNKPYK
1291	588.26	1174.51	1174.56	-0.04	0	2	4.4	1	LNGDFCIHTR
704	906.81	905.80	905.50	0.30	0	2	4.9	1	VVAFDSIR
1104	543.27	1084.52	1084.64	-0.11	2	2	4.5	1	QAGLVRKEGK
1619	479.75	1436.22	1435.73	0.49	1	2	7.2	1	DGIVQFETNGKTK
368	697.89	1393.76	1393.71	0.05	1	2	13	1	MNSLAQLGKYNR
1366	611.27	1220.52	1220.58	-0.06	1	2	3.5	1	GGMHWKSFSGK
1603	709.50	1416.98	1416.71	0.27	1	2	4.6	1	RGWVPANYVQDI
38	412.33	1233.96	1233.58	0.38	1	2	7.8	1	MSDRVEVNER
1115	544.73	1087.45	1087.51	-0.06	0	2	3.9	1	HLAPQYCEK

☑	1101	542.82	1083.63	1083.70	-0.08	1	2	4	1	SRLLLEILK
☑	2053	1025.94	2049.86	2050.03	-0.17	2	2	3.9	1	MKKQNLVSFPENLEAMR + Oxidation (M)
☑	1464	641.34	1280.66	1280.56	0.10	0	2	4.2	1	ELISNSSDACDK
☑	1783	542.31	1623.90	1623.87	0.03	2	2	3.8	1	MNLLKHCVVNGPRK + Oxidation (M)
☑	423	740.67	2218.98	2219.18	-0.20	2	1	11	1	AKSHIYGVHSLEEVREIPR
☑	849	485.66	969.31	969.54	-0.23	0	1	4.2	1	VRPNEVTR
☑	2204	765.97	2294.88	2295.08	-0.20	0	1	3	1	HYTPAVDMWSIGCIFAELAR + Oxidation (M)
☑	2304	844.92	2531.73	2532.30	-0.57	1	1	5.9	1	HVLVILTDMSSYADALREVSAAR + Oxidation (M)
☑	2459	1095.40	3283.18	3283.65	-0.48	1	1	4.6	1	FRCQMPVVEYEALPIWQRPPIVDDTPK + Carbamidomethyl (C)
☑	341	679.61	678.60	678.37	0.23	1	1	18	1	AFDKAK
☑	1114	544.73	1087.45	1087.57	-0.12	0	1	4	1	TPVSLDMLGR
☑	1726	779.38	1556.74	1556.91	-0.16	1	1	3.9	1	MTLQISAQVVKALR
☑	1461	640.34	1278.66	1278.69	-0.02	2	1	4.6	1	VRYKGIVCER + Carbamidomethyl (C)
☑	68	487.24	972.47	972.54	-0.07	2	1	9.2	1	EKGIENRK
☑	445	756.90	2267.69	2267.16	0.53	1	1	18	1	NAAIVWEEETLHKFLENPK
☑	1813	554.94	1661.80	1661.88	-0.08	2	1	4.1	1	KVPERPTVCNVRGAH
☑	230	606.69	605.69	605.30	0.38	0	1	4.8	1	DTATAK
☑	1793	820.70	1639.39	1639.70	-0.31	1	1	6.5	1	DCPSSQGGSRGGYGQK + Carbamidomethyl (C)
☑	314	327.69	653.37	653.31	0.06	0	1	2	1	NPMHR
☑	1954	930.30	1858.59	1859.01	-0.42	2	1	5.7	1	RVVVGKENTTIISDSNK
☑	2290	836.13	2505.36	2505.30	0.07	2	1	3.7	1	QNVQKLIDSLKLSIQEDDYEK
☑	1802	551.67	1652.00	1651.95	0.05	1	1	4.1	1	VLWLRNNIAIAVDR
☑	1267	583.74	1165.47	1165.54	-0.06	1	1	4.5	1	GEGYRDVNEK
☑	1849	855.91	1709.81	1709.97	-0.16	1	1	4.5	1	LSFINSVSYKSLKPK
☑	914	498.25	994.48	994.62	-0.14	0	1	3.7	1	LVLLGAPGAGK
☑	1418	624.86	1247.70	1247.61	0.09	1	1	5.3	1	SNSSQGKGSIQVG
☑	1775	809.33	1616.65	1616.81	-0.16	2	1	5.4	1	QMLYTRFFDPKR + Oxidation (M)
☑	395	718.87	2153.59	2153.26	0.33	1	1	23	1	TAVLAMGSGLGAALVVRTPLLK + Oxidation (M)
☑	824	477.94	953.87	954.46	-0.60	0	1	5	1	NNNTSIHR
☑	1249	578.39	1154.76	1154.71	0.05	2	1	4.8	1	LLERAAKLNK
☑	137	554.67	1660.99	1660.90	0.08	0	1	9.9	1	LIAYLTAGAPDINTTK
☑	285	638.90	1275.78	1275.69	0.10	2	1	15	1	KLEEMETKLR
☑	1276	585.78	1169.55	1169.62	-0.07	0	1	3.7	1	HEYSLLPGVR
☑	1582	695.82	1389.62	1389.69	-0.08	0	1	4.8	1	AGSLATEVMENIR

☑	275	629.91	628.91	628.31	0.60	0	1	21	1	MAVHR + Oxidation (M)
☑	1683	754.33	1506.64	1506.74	-0.10	1	1	5.5	1	FQLYKSGAYSDTK
☑	1755	795.32	1588.63	1588.79	-0.16	1	1	4.7	1	ITEQDICANLEKR + Carbamidomethyl (C)
☑	1760	798.89	1595.77	1595.89	-0.12	0	1	5.4	1	IVQVNNDENILVVK
☑	1194	564.82	1127.62	1127.56	0.06	0	1	4.7	1	DAEWVGQVPK
☑	1796	548.74	1643.20	1642.86	0.34	1	1	4.9	1	NAQQDAQNIVSSKIK
☑	1491	650.80	1299.59	1299.72	-0.14	1	1	5.3	1	EKQVPVLVCTK + Carbamidomethyl (C)
☑	1353	607.30	1212.58	1212.65	-0.07	0	1	4.2	1	FRPQEQRPR
☑	243	614.36	1840.05	1839.91	0.14	0	1	16	1	EGPIICEVQQLLGDNR + Carbamidomethyl (C)
☑	400	723.00	1443.98	1443.73	0.25	1	1	15	1	FISKYCWATIGR
☑	2302	844.80	2531.39	2531.19	0.20	2	1	4.6	1	FYSTESGLEMTTLKDYVTRMK + 2 Oxidation (M)
☑	1673	744.37	1486.73	1486.74	-0.00	2	1	7.3	1	QRGGRGNAGGQHHR
☑	258	623.16	1866.45	1867.03	-0.58	2	1	19	1	KAEVLINGPGSGRETAIR
☑	1441	632.31	1262.61	1262.71	-0.10	1	1	5.2	1	VVIAQKMIMSK + Oxidation (M)
☑	1684	754.84	1507.66	1507.74	-0.08	0	1	3.9	1	MTNIDVISDMLTR
☑	2257	805.97	2414.88	2415.20	-0.32	2	1	4.2	1	FISQSAWFSRSIDCQNLTGKK
☑	2152	738.05	2211.12	2211.10	0.02	0	1	5.5	1	FMPVYVTEPSVEECISILR
☑	193	589.32	588.31	588.32	-0.01	0	1	17	1	SDIVR
☑	1801	551.66	1651.95	1651.79	0.16	0	1	4.6	1	ILPFDEMYGPEVSR
☑	1124	545.76	1089.51	1089.53	-0.02	0	1	6.3	1	VMVICNNPK + Carbamidomethyl (C); Oxidation (M)
☑	549	816.45	1630.89	1630.76	0.14	1	1	16	1	LMCASTACKTMITK + 2 Carbamidomethyl (C); Oxidation (M)
☑	1732	783.40	1564.78	1564.86	-0.08	2	1	4.8	1	TKQVGKDLVFSTSR
☑	2180	1126.09	2250.17	2250.09	0.08	2	1	3	1	AASGGFEKGLDRENEPVLSMK + Oxidation (M)
☑	751	463.27	924.52	924.49	0.03	0	1	4.1	1	HLDSVLEI
☑	479	781.83	2342.48	2343.08	-0.60	1	1	15	1	TISLLGMKMDTSDFYTFADR + 2 Oxidation (M)
☑	2249	797.42	2389.23	2389.15	0.08	2	1	3.6	1	GPMTHGSKNHRAPGSIGMGTSPGR
☑	6	263.01	786.02	786.47	-0.45	0	1	12	1	MISPVLK
☑	226	604.21	1809.60	1810.04	-0.44	0	1	17	1	VAVMGAAGGIGQPLSLLLK + Oxidation (M)
☑	2343	881.39	2641.14	2641.43	-0.30	1	0	2.7	1	VENGKILTQQPLIYLTAEQENLK
☑	1658	490.97	1469.88	1469.78	0.10	0	0	4.9	1	ILLNTNSSDPQIR
☑	387	712.36	711.35	711.41	-0.06	0	0	14	1	MVHVVK
☑	1716	514.00	1538.98	1539.00	-0.03	1	0	4.2	1	IVVALLIGIFIGRR
☑	501	791.28	2370.83	2371.35	-0.52	2	0	18	1	CGSVAVRLVPAPRGTGIVAAPVPK + Carbamidomethyl (C)
☑	816	952.40	1902.78	1902.74	0.05	1	0	12	1	QMFDPKNMMADCDPR + Carbamidomethyl (C); 3 Oxidation (M)

☑	2329	863.22	2586.62	2586.24	0.38	1	0	5	1	DFRSSTSVVEAHLPEEVPEGMVR + Oxidation (M)
☑	1431	629.84	1257.67	1257.73	-0.06	1	0	4.5	1	VSGLSQIKIGEK
☑	1797	823.31	1644.61	1644.81	-0.19	1	0	5.4	1	EGMRNVAAGANPMAIK + Oxidation (M)
☑	1327	599.92	1197.83	1197.51	0.31	0	0	5	1	DSYVGDEAQSK
☑	1514	441.91	1322.70	1322.67	0.03	2	0	4.3	1	TMKNFWNRAR
☑	799	947.23	1892.44	1892.00	0.44	1	0	18	1	VIMSQITRAPGIYYHK + Oxidation (M)
☑	1689	756.20	1510.38	1510.74	-0.37	1	0	9	1	ATLDGVVTYEGRCK
☑	463	768.60	2302.76	2302.17	0.59	2	0	17	1	KLDNSKVALVYGQMNEPPGAR + Oxidation (M)
☑	298	645.52	1933.54	1934.07	-0.52	2	0	25	1	LTGSLTKSSSLVSIEKER
☑	467	771.67	2312.00	2312.09	-0.09	2	0	18	1	QQTLAQSMSDMDMERLLKR + 2 Oxidation (M)
☑	1383	1231.10	3690.28	3689.79	0.49	1	0	18	1	TFQDLEESVQFRTASNITDGQIFSINVTPTMGK + Oxidation (M)
☑	1640	727.34	1452.67	1452.85	-0.18	2	0	5.5	1	ISRRLGDLPGLTR
☑	1722	776.87	1551.73	1551.84	-0.11	0	0	5.7	1	GMVLAKPGSITPHTK + Oxidation (M)
☑	878	983.85	982.84	982.54	0.29	0	0	15	1	DHLSVATLK
☑	1311	594.32	1186.62	1186.66	-0.04	2	0	7	1	LAKGFRGSHSK
☑	376	703.98	702.97	702.37	0.59	0	0	14	1	AGLMAPK + Oxidation (M)
☑	1368	611.37	1220.72	1220.66	0.07	1	0	5	1	KYIADHSIFK
☑	1930	917.42	1832.82	1832.91	-0.10	2	0	5	1	RSGADATPETRAAFVER
☑	2111	716.68	2147.02	2147.00	0.02	1	0	4.4	1	YELYTVCRPSSDEKLCNK
☑	1230	1149.49	2296.97	2297.22	-0.25	2	0	14	1	QNLPNLSQFDVNARREGVLK
☑	1825	563.83	1688.47	1687.98	0.48	2	0	9.2	1	ISPHKVRQVLDQIR
☑	2121	722.67	2164.99	2165.03	-0.05	1	0	4.8	1	ASSKTLLFTSECVSESHPDK
☑	1	*227.13*	*226.13*							
☑	2	*229.03*	*228.02*							
☑	3	*229.13*	*228.12*							
☑	4	*229.16*	*228.15*							
☑	5	*235.12*	*234.11*							
☑	7	*284.95*	*283.94*							
☑	8	*284.97*	*283.96*							
☑	9	*284.99*	*283.98*							
☑	10	*285.00*	*283.99*							
☑	11	*285.00*	*283.99*							
☑	12	*285.02*	*284.01*							
☑	13	*285.18*	*284.17*							

☑	14	297.10	296.09
☑	15	298.99	297.98
☑	16	299.00	297.99
☑	17	299.03	298.02
☑	18	299.04	298.03
☑	19	299.07	298.06
☑	20	299.10	298.09
☑	21	299.22	298.22
☑	22	303.18	302.17
☑	23	329.82	328.81
☑	24	334.12	333.12
☑	25	338.57	337.57
☑	27	355.07	354.07
☑	28	355.19	354.18
☑	29	359.19	358.18
☑	30	371.06	370.06
☑	31	371.07	370.06
☑	32	371.09	370.08
☑	33	371.10	370.10
☑	34	382.96	381.95
☑	35	392.29	391.28
☑	37	403.21	402.20
☑	39	414.21	413.20
☑	40	416.16	415.15
☑	42	429.05	428.04
☑	43	429.45	428.44
☑	45	432.80	431.79
☑	47	445.03	444.02
☑	48	445.06	444.06
☑	49	447.58	446.57
☑	50	449.15	448.14
☑	52	457.24	456.23
☑	54	462.71	461.70
☑	55	464.00	462.99

☑	56	*465.16*	*464.15*
☑	57	*465.33*	*464.32*
☑	58	*466.51*	*465.50*
☑	59	*470.79*	*469.78*
☑	60	*471.25*	*470.24*
☑	61	*479.82*	*478.81*
☑	62	*480.69*	*479.68*
☑	63	*480.74*	*479.73*
☑	64	*484.56*	*483.55*
☑	65	*484.60*	*483.59*
☑	66	*484.63*	*483.62*
☑	71	*490.25*	*489.24*
☑	72	*491.23*	*490.22*
☑	73	*491.83*	*490.83*
☑	74	*493.87*	*492.86*
☑	77	*499.34*	*498.33*
☑	78	*499.78*	*498.77*
☑	79	*502.61*	*501.60*
☑	81	*504.21*	*503.20*
☑	82	*508.62*	*507.62*
☑	84	*509.74*	*508.73*
☑	85	*513.36*	*512.35*
☑	86	*513.62*	*512.61*
☑	87	*513.75*	*512.75*
☑	88	*514.71*	*513.70*
☑	89	*516.27*	*515.26*
☑	90	*516.30*	*515.30*
☑	91	*517.39*	*516.39*
☑	92	*519.12*	*518.11*
☑	94	*519.14*	*518.14*
☑	95	*519.25*	*518.24*
☑	96	*519.45*	*518.44*
☑	97	*520.17*	*519.16*
☑	98	*520.27*	*519.26*

☑	99	520.33	519.32
☑	100	521.07	520.06
☑	103	523.34	522.33
☑	104	525.37	524.37
☑	105	525.58	524.57
☑	106	527.21	526.20
☑	107	527.35	526.34
☑	108	528.59	527.58
☑	109	529.61	528.60
☑	110	529.86	528.85
☑	111	532.13	531.13
☑	112	532.29	531.28
☑	113	533.80	532.79
☑	114	536.56	535.55
☑	115	538.07	537.06
☑	116	538.09	537.08
☑	117	538.34	537.34
☑	119	539.72	538.71
☑	120	540.76	539.75
☑	121	543.00	541.99
☑	122	544.03	543.03
☑	123	544.77	543.77
☑	124	544.87	543.87
☑	125	544.97	543.96
☑	127	546.47	545.47
☑	128	549.53	548.53
☑	130	550.64	549.63
☑	131	550.79	549.78
☑	133	551.58	550.58
☑	134	552.64	551.63
☑	138	554.70	553.70
☑	139	555.30	554.30
☑	140	556.36	555.35
☑	142	557.79	556.79

☑	143	558.80	557.79
☑	144	558.81	557.80
☑	145	559.70	558.69
☑	146	559.97	558.96
☑	147	562.36	561.36
☑	148	562.67	561.66
☑	149	563.34	562.33
☑	150	563.62	562.62
☑	151	564.71	563.70
☑	152	564.94	563.93
☑	153	565.05	564.05
☑	154	565.08	564.07
☑	155	565.84	564.83
☑	156	565.99	564.99
☑	157	566.62	565.61
☑	159	284.92	567.82
☑	160	284.94	567.87
☑	161	284.96	567.90
☑	162	568.99	567.99
☑	163	569.67	568.66
☑	164	569.84	568.83
☑	165	570.33	569.32
☑	166	570.62	569.61
☑	167	571.78	570.77
☑	168	571.87	570.86
☑	169	572.22	571.21
☑	171	576.37	575.36
☑	172	576.66	575.65
☑	173	576.87	575.87
☑	174	577.01	576.00
☑	175	579.35	578.34
☑	176	579.69	578.68
☑	177	580.26	579.25
☑	178	580.53	579.52

☑	179	580.65	579.64
☑	181	581.55	580.54
☑	182	582.51	581.50
☑	183	582.65	581.64
☑	184	583.05	582.04
☑	185	583.32	582.32
☑	187	587.14	586.13
☑	188	587.22	586.21
☑	191	588.59	587.59
☑	192	588.82	587.81
☑	194	589.50	588.49
☑	196	591.01	590.00
☑	197	592.51	591.51
☑	200	593.05	592.05
☑	201	593.07	592.06
☑	202	593.09	592.08
☑	203	593.13	592.12
☑	204	593.20	592.19
☑	205	593.34	592.33
☑	206	593.35	592.35
☑	207	594.24	593.23
☑	208	594.57	593.56
☑	210	595.90	594.89
☑	211	595.97	594.96
☑	212	596.92	595.92
☑	213	298.99	595.96
☑	214	597.34	596.33
☑	215	597.79	596.78
☑	216	599.44	598.43
☑	217	600.86	599.85
☑	218	600.94	599.93
☑	219	601.34	600.34
☑	223	602.52	601.51
☑	224	602.98	601.97

☑	225	603.11	602.11
☑	228	605.12	604.11
☑	232	608.35	607.34
☑	233	610.05	609.04
☑	234	610.39	609.38
☑	235	611.03	610.02
☑	236	611.55	610.54
☑	237	612.18	611.17
☑	238	612.75	611.74
☑	239	612.85	611.84
☑	240	613.31	612.31
☑	241	613.77	612.76
☑	242	614.09	613.08
☑	244	614.36	613.35
☑	245	617.99	616.98
☑	246	618.18	617.17
☑	247	618.84	617.83
☑	248	619.10	618.09
☑	250	620.06	619.05
☑	251	620.22	619.21
☑	252	620.43	619.42
☑	254	621.28	620.28
☑	255	621.64	620.63
☑	256	622.04	621.03
☑	257	622.11	621.11
☑	259	623.33	622.32
☑	260	623.76	622.76
☑	261	625.16	624.16
☑	262	625.23	624.22
☑	264	626.53	625.52
☑	265	626.79	625.78
☑	266	626.90	625.90
☑	267	627.26	626.25
☑	268	627.40	626.40

☑	269	627.70	626.69
☑	270	628.29	627.28
☑	272	628.90	627.89
☑	273	629.24	628.23
☑	276	629.98	628.97
☑	277	631.20	630.19
☑	278	634.04	633.03
☑	279	634.31	633.30
☑	280	635.35	634.34
☑	281	637.33	636.32
☑	283	638.01	637.00
☑	284	638.32	637.31
☑	286	639.35	638.34
☑	287	639.45	638.44
☑	288	639.47	638.46
☑	289	639.50	638.49
☑	290	639.81	638.80
☑	291	640.50	639.49
☑	292	640.53	639.53
☑	294	641.46	640.45
☑	295	642.30	641.30
☑	296	642.39	641.39
☑	297	643.61	642.60
☑	299	645.77	644.77
☑	300	647.17	646.17
☑	301	647.39	646.39
☑	302	647.90	646.89
☑	303	648.83	647.82
☑	304	649.41	648.40
☑	305	649.88	648.87
☑	306	649.88	648.87
☑	308	650.66	649.66
☑	309	326.13	650.25
☑	310	651.69	650.69

☑	311	651.76	650.76
☑	312	651.96	650.95
☑	313	653.84	652.83
☑	315	654.92	653.91
☑	316	655.04	654.03
☑	317	655.68	654.68
☑	318	657.71	656.70
☑	320	658.70	657.69
☑	321	658.84	657.83
☑	322	659.25	658.24
☑	324	661.45	660.45
☑	325	663.59	662.58
☑	326	663.82	662.81
☑	327	664.97	663.96
☑	329	665.33	664.32
☑	330	668.78	667.77
☑	332	671.61	670.60
☑	333	671.84	670.84
☑	334	673.46	672.46
☑	335	673.86	672.85
☑	336	675.22	674.21
☑	337	677.17	676.16
☑	338	677.97	676.96
☑	339	678.25	677.24
☑	340	226.86	677.55
☑	342	680.32	679.31
☑	343	681.24	680.23
☑	344	681.54	680.53
☑	345	341.42	680.83
☑	350	687.24	686.23
☑	351	687.35	686.35
☑	352	688.58	687.57
☑	353	688.74	687.74
☑	354	688.78	687.77

☑	356	*690.35*	*689.34*
☑	357	*691.73*	*690.72*
☑	358	*692.31*	*691.30*
☑	360	*692.63*	*691.62*
☑	362	*692.95*	*691.94*
☑	363	*693.14*	*692.13*
☑	364	*694.26*	*693.26*
☑	365	*694.92*	*693.91*
☑	366	*696.15*	*695.14*
☑	367	*697.88*	*696.87*
☑	369	*698.08*	*697.07*
☑	370	*699.37*	*698.37*
☑	371	*699.41*	*698.41*
☑	373	*699.62*	*698.62*
☑	375	*700.30*	*699.29*
☑	377	*705.64*	*704.63*
☑	378	*705.97*	*704.97*
☑	379	*706.13*	*705.12*
☑	380	*706.35*	*705.34*
☑	381	*707.07*	*706.06*
☑	382	*708.59*	*707.58*
☑	383	*710.28*	*709.28*
☑	384	*710.79*	*709.79*
☑	385	*712.21*	*711.20*
☑	386	*712.33*	*711.32*
☑	388	*713.80*	*712.80*
☑	389	*714.33*	*713.32*
☑	390	*714.98*	*713.98*
☑	391	*715.07*	*714.06*
☑	392	*715.98*	*714.98*
☑	393	*717.04*	*716.04*
☑	397	*719.92*	*718.91*
☑	398	*720.33*	*719.32*
☑	399	*721.91*	*720.90*

☑	401	723.78	722.77
☑	402	725.11	724.10
☑	403	363.46	724.91
☑	404	725.99	724.98
☑	405	726.07	725.07
☑	406	727.96	726.95
☑	407	729.96	728.95
☑	408	730.53	729.52
☑	409	366.18	730.34
☑	410	732.75	731.74
☑	411	733.79	732.78
☑	413	734.11	733.10
☑	415	736.51	735.50
☑	416	737.28	736.27
☑	417	737.57	736.57
☑	418	738.15	737.15
☑	420	739.00	737.99
☑	421	739.40	738.39
☑	422	740.33	739.32
☑	424	371.15	740.28
☑	425	371.17	740.33
☑	426	741.75	740.74
☑	427	741.78	740.77
☑	428	741.97	740.96
☑	429	742.52	741.51
☑	430	742.74	741.73
☑	431	743.02	742.01
☑	432	744.41	743.40
☑	433	745.00	743.99
☑	434	746.29	745.29
☑	436	747.06	746.05
☑	437	750.40	749.39
☑	438	750.51	749.51
☑	439	750.95	749.94

☑	440	751.80	750.79
☑	441	752.73	751.72
☑	442	752.96	751.95
☑	443	755.42	754.41
☑	444	756.13	755.13
☑	446	757.93	756.93
☑	447	758.54	757.53
☑	448	758.86	757.85
☑	449	758.89	757.88
☑	450	759.17	758.16
☑	451	760.48	759.47
☑	452	760.62	759.61
☑	453	761.87	760.86
☑	454	762.19	761.18
☑	455	762.86	761.85
☑	456	762.89	761.88
☑	458	765.23	764.23
☑	460	766.16	765.15
☑	461	766.48	765.48
☑	462	766.99	765.98
☑	464	770.60	769.60
☑	465	770.62	769.61
☑	468	771.72	770.71
☑	470	773.21	772.20
☑	471	774.15	773.14
☑	472	774.88	773.87
☑	473	775.08	774.07
☑	474	775.17	774.16
☑	475	776.21	775.20
☑	476	777.03	776.02
☑	477	780.17	779.16
☑	478	780.21	779.20
☑	480	782.20	781.19
☑	481	783.33	782.32

☑	482	783.33	782.32
☑	483	783.75	782.74
☑	484	784.21	783.20
☑	485	784.40	783.39
☑	486	784.51	783.50
☑	487	785.13	784.12
☑	488	785.37	784.36
☑	489	393.19	784.37
☑	490	393.21	784.41
☑	491	786.05	785.04
☑	492	786.27	785.26
☑	493	788.71	787.70
☑	494	788.91	787.90
☑	495	789.33	788.32
☑	496	789.72	788.71
☑	497	789.96	788.95
☑	498	790.86	789.85
☑	499	791.07	790.07
☑	500	791.24	790.23
☑	502	791.57	790.56
☑	503	792.83	791.83
☑	504	792.98	791.97
☑	505	793.10	792.09
☑	506	793.34	792.33
☑	507	397.23	792.45
☑	508	794.42	793.41
☑	509	795.70	794.69
☑	510	797.18	796.17
☑	511	797.35	796.34
☑	512	797.50	796.49
☑	513	797.88	796.87
☑	514	798.86	797.85
☑	515	798.96	797.95
☑	516	400.70	799.39

☑	517	800.90	799.89
☑	518	801.16	800.15
☑	520	802.20	801.19
☑	521	802.46	801.46
☑	522	802.77	801.76
☑	523	803.38	802.37
☑	524	402.21	802.41
☑	525	803.54	802.54
☑	526	804.58	803.57
☑	527	804.91	803.91
☑	528	804.95	803.94
☑	529	805.08	804.07
☑	531	806.11	805.10
☑	532	806.74	805.73
☑	533	807.08	806.07
☑	534	807.40	806.39
☑	535	808.08	807.07
☑	536	808.40	807.40
☑	537	808.41	807.40
☑	538	808.77	807.76
☑	539	810.03	809.02
☑	541	812.50	811.50
☑	542	813.08	812.07
☑	543	813.86	812.86
☑	544	813.95	812.94
☑	545	814.09	813.09
☑	548	815.46	814.45
☑	550	817.47	816.47
☑	551	818.58	817.58
☑	552	818.71	817.70
☑	553	819.33	818.33
☑	554	820.02	819.02
☑	555	820.91	819.91
☑	556	821.10	820.09

☑	557	821.41	820.40
☑	558	411.63	821.24
☑	559	411.85	821.69
☑	560	822.74	821.73
☑	561	823.50	822.49
☑	562	824.51	823.50
☑	563	412.76	823.51
☑	564	824.87	823.86
☑	565	825.74	824.73
☑	567	826.98	825.97
☑	568	827.69	826.68
☑	569	827.89	826.88
☑	571	829.43	828.42
☑	572	829.45	828.44
☑	573	829.95	828.94
☑	574	415.67	829.32
☑	576	831.59	830.58
☑	577	832.52	831.52
☑	578	833.51	832.50
☑	579	835.98	834.97
☑	580	836.51	835.50
☑	581	837.83	836.82
☑	582	838.09	837.08
☑	583	838.09	837.08
☑	584	839.54	838.54
☑	585	839.89	838.88
☑	586	842.28	841.28
☑	587	843.50	842.50
☑	589	843.74	842.73
☑	590	844.31	843.30
☑	591	846.04	845.03
☑	594	847.43	846.42
☑	595	847.98	846.97
☑	596	848.78	847.78

☑	597	848.98	847.97
☑	598	849.23	848.22
☑	600	850.22	849.21
☑	601	851.39	850.39
☑	602	851.72	850.71
☑	603	851.91	850.91
☑	604	284.91	851.71
☑	605	284.94	851.78
☑	606	853.16	852.16
☑	607	853.23	852.22
☑	608	854.23	853.22
☑	609	854.53	853.52
☑	610	855.19	854.18
☑	611	855.35	854.34
☑	612	855.37	854.36
☑	613	428.29	854.56
☑	614	856.39	855.38
☑	615	856.45	855.44
☑	616	429.15	856.28
☑	617	857.71	856.70
☑	618	857.80	856.79
☑	619	859.33	858.32
☑	620	860.41	859.41
☑	621	860.49	859.48
☑	622	861.77	860.76
☑	623	863.02	862.01
☑	624	432.22	862.43
☑	625	863.81	862.80
☑	626	865.61	864.60
☑	627	865.78	864.77
☑	628	865.85	864.85
☑	629	433.72	865.43
☑	630	866.44	865.43
☑	631	433.78	865.55

☑	632	866.71	865.70
☑	633	867.29	866.28
☑	634	434.68	867.35
☑	635	868.68	867.68
☑	636	435.27	868.52
☑	638	869.88	868.87
☑	639	870.47	869.46
☑	640	870.53	869.52
☑	642	872.53	871.52
☑	643	872.85	871.84
☑	644	873.36	872.35
☑	645	874.35	873.34
☑	646	875.72	874.71
☑	647	876.54	875.53
☑	648	876.61	875.60
☑	649	876.66	875.65
☑	650	876.68	875.67
☑	651	877.23	876.23
☑	654	877.74	876.73
☑	656	878.77	877.77
☑	657	878.94	877.93
☑	658	440.27	878.53
☑	659	879.96	878.95
☑	660	880.20	879.20
☑	661	880.34	879.33
☑	662	880.91	879.90
☑	663	880.96	879.95
☑	664	881.30	880.29
☑	665	883.26	882.26
☑	666	883.36	882.35
☑	667	884.05	883.04
☑	668	884.16	883.15
☑	669	443.21	884.42
☑	670	885.60	884.60

☑	671	886.37	885.36
☑	672	886.46	885.45
☑	674	887.01	886.00
☑	676	445.20	888.38
☑	677	889.75	888.74
☑	678	890.33	889.32
☑	679	891.91	890.90
☑	680	892.29	891.28
☑	681	892.80	891.79
☑	682	893.40	892.39
☑	683	447.27	892.52
☑	684	894.28	893.28
☑	685	895.20	894.19
☑	686	895.65	894.64
☑	687	895.70	894.69
☑	688	895.74	894.73
☑	689	897.05	896.04
☑	691	899.81	898.80
☑	692	900.40	899.39
☑	693	900.46	899.45
☑	694	900.55	899.54
☑	695	901.92	900.91
☑	696	903.87	902.86
☑	697	452.54	903.06
☑	698	904.52	903.51
☑	699	904.72	903.71
☑	700	905.12	904.11
☑	703	906.78	905.78
☑	705	907.11	906.10
☑	706	454.22	906.42
☑	707	908.55	907.55
☑	708	908.60	907.59
☑	709	909.01	908.00
☑	710	909.23	908.22

☑	711	909.73	908.72
☑	712	909.91	908.90
☑	714	455.73	909.44
☑	715	911.09	910.08
☑	716	911.12	910.12
☑	717	911.40	910.39
☑	719	912.18	911.17
☑	720	912.34	911.33
☑	721	912.41	911.40
☑	722	456.74	911.46
☑	723	912.95	911.94
☑	724	457.21	912.40
☑	725	913.49	912.48
☑	726	457.30	912.58
☑	727	913.65	912.65
☑	728	914.04	913.04
☑	729	458.24	914.46
☑	730	916.08	915.07
☑	731	458.92	915.82
☑	732	918.24	917.23
☑	733	919.30	918.29
☑	734	919.35	918.34
☑	735	461.29	920.57
☑	736	921.89	920.89
☑	737	922.03	921.02
☑	738	922.60	921.59
☑	739	922.85	921.85
☑	741	923.11	922.10
☑	742	462.10	922.19
☑	743	923.41	922.41
☑	744	462.22	922.42
☑	745	462.53	923.04
☑	746	924.40	923.40
☑	748	924.93	923.93

☑	749	925.29	924.28
☑	750	463.19	924.37
☑	752	926.42	925.42
☑	753	926.44	925.44
☑	754	926.66	925.65
☑	755	926.71	925.70
☑	756	926.79	925.79
☑	757	463.90	925.79
☑	758	927.18	926.17
☑	759	927.27	926.26
☑	760	927.67	926.67
☑	761	927.77	926.76
☑	763	928.82	927.81
☑	764	930.54	929.53
☑	766	930.57	929.56
☑	767	466.20	930.39
☑	768	931.76	930.75
☑	769	932.16	931.15
☑	770	466.81	931.61
☑	771	933.99	932.98
☑	772	934.53	933.52
☑	773	934.86	933.85
☑	775	935.58	934.57
☑	776	935.59	934.58
☑	777	936.09	935.08
☑	778	937.96	936.95
☑	779	938.44	937.43
☑	780	469.74	937.47
☑	782	939.04	938.04
☑	783	939.67	938.67
☑	784	939.67	938.67
☑	785	470.68	939.35
☑	786	940.58	939.57
☑	787	940.74	939.73

☑	788	940.93	939.92
☑	789	941.92	940.91
☑	790	942.29	941.28
☑	792	942.58	941.58
☑	793	943.46	942.45
☑	794	944.72	943.71
☑	795	945.65	944.65
☑	796	945.99	944.98
☑	797	946.11	945.11
☑	798	946.37	945.36
☑	800	947.52	946.51
☑	801	947.96	946.95
☑	802	948.27	947.26
☑	803	949.21	948.20
☑	804	475.14	948.27
☑	805	949.40	948.39
☑	806	949.43	948.42
☑	808	950.44	949.43
☑	809	950.77	949.76
☑	810	950.99	949.98
☑	811	951.29	950.29
☑	812	951.33	950.32
☑	813	951.60	950.59
☑	814	951.78	950.77
☑	815	476.43	950.85
☑	817	477.26	952.52
☑	818	953.71	952.70
☑	819	953.73	952.72
☑	820	953.88	952.87
☑	821	954.29	953.28
☑	823	477.81	953.61
☑	825	955.19	954.18
☑	826	478.91	955.81
☑	827	958.14	957.13

☑	828	958.24	957.23
☑	829	479.68	957.34
☑	830	958.81	957.80
☑	831	480.21	958.41
☑	832	959.96	958.95
☑	833	961.25	960.25
☑	834	962.69	961.68
☑	835	964.02	963.02
☑	836	964.55	963.55
☑	838	966.70	965.69
☑	839	967.45	966.44
☑	840	484.24	966.47
☑	841	967.70	966.69
☑	842	967.88	966.87
☑	843	968.24	967.23
☑	844	968.37	967.36
☑	845	968.76	967.75
☑	846	969.01	968.00
☑	847	969.12	968.11
☑	848	485.07	968.13
☑	850	971.06	970.06
☑	851	971.35	970.34
☑	852	486.23	970.45
☑	853	971.65	970.64
☑	854	972.01	971.01
☑	855	972.45	971.44
☑	857	974.10	973.09
☑	858	974.14	973.13
☑	859	974.20	973.19
☑	860	974.45	973.44
☑	861	975.03	974.02
☑	862	975.18	974.17
☑	863	488.20	974.38
☑	864	488.24	974.46

☑	866	975.53	974.52
☑	868	977.08	976.07
☑	870	978.38	977.37
☑	871	979.09	978.08
☑	872	979.28	978.27
☑	873	490.21	978.40
☑	874	980.02	979.01
☑	875	490.68	979.34
☑	876	981.25	980.25
☑	877	981.49	980.48
☑	879	984.43	983.43
☑	880	984.57	983.56
☑	881	984.87	983.86
☑	882	985.00	983.99
☑	883	985.48	984.47
☑	884	493.31	984.61
☑	885	985.95	984.95
☑	886	493.96	985.90
☑	887	987.21	986.20
☑	888	988.03	987.03
☑	889	988.15	987.14
☑	890	988.24	987.23
☑	891	988.40	987.39
☑	893	988.66	987.65
☑	894	988.73	987.72
☑	895	494.88	987.75
☑	896	494.93	987.85
☑	897	495.21	988.41
☑	898	495.28	988.55
☑	900	990.40	989.39
☑	901	991.02	990.01
☑	902	496.26	990.50
☑	903	991.84	990.84
☑	904	992.47	991.46

☑	906	993.63	992.62
☑	907	993.64	992.63
☑	908	993.84	992.84
☑	909	993.96	992.95
☑	910	332.03	993.07
☑	911	994.17	993.17
☑	912	995.09	994.09
☑	913	498.20	994.39
☑	915	995.80	994.79
☑	916	996.09	995.09
☑	918	997.13	996.12
☑	919	997.54	996.53
☑	920	499.31	996.60
☑	921	997.64	996.63
☑	922	998.09	997.08
☑	923	499.76	997.52
☑	924	999.43	998.42
☑	925	500.36	998.70
☑	926	1000.12	999.11
☑	927	1000.78	999.77
☑	928	1000.86	999.85
☑	929	1001.67	1000.66
☑	930	1002.71	1001.71
☑	931	1003.32	1002.31
☑	932	502.26	1002.50
☑	933	1004.45	1003.44
☑	935	1004.47	1003.46
☑	937	503.77	1005.52
☑	938	503.79	1005.57
☑	939	503.80	1005.59
☑	941	1008.58	1007.57
☑	942	1008.64	1007.64
☑	943	1009.63	1008.62
☑	944	1010.24	1009.23

☑	945	1010.66	1009.65
☑	946	1011.17	1010.17
☑	947	1011.45	1010.45
☑	948	506.33	1010.64
☑	949	1011.83	1010.82
☑	950	1012.29	1011.28
☑	952	1013.69	1012.68
☑	953	1013.78	1012.77
☑	954	1014.11	1013.11
☑	955	1014.32	1013.32
☑	956	1014.43	1013.42
☑	957	1015.26	1014.25
☑	958	508.27	1014.54
☑	959	1015.85	1014.84
☑	960	1016.33	1015.32
☑	964	1016.80	1015.79
☑	965	1016.98	1015.97
☑	966	1017.09	1016.09
☑	967	1017.11	1016.10
☑	968	509.23	1016.44
☑	969	509.82	1017.64
☑	970	1018.65	1017.64
☑	971	1019.05	1018.04
☑	972	510.27	1018.53
☑	973	510.28	1018.55
☑	974	1019.83	1018.82
☑	976	1021.45	1020.45
☑	977	1021.95	1020.94
☑	980	1023.60	1022.59
☑	981	1023.70	1022.69
☑	982	1024.15	1023.14
☑	983	1024.67	1023.66
☑	984	1024.81	1023.81
☑	986	1026.19	1025.19

☑	987	1027.19	1026.18
☑	988	514.29	1026.56
☑	989	1027.96	1026.95
☑	990	514.79	1027.57
☑	992	1028.95	1027.94
☑	995	1029.73	1028.73
☑	996	1030.73	1029.72
☑	997	1031.16	1030.15
☑	998	1031.46	1030.45
☑	1002	1031.96	1030.95
☑	1003	1032.09	1031.08
☑	1004	1032.53	1031.52
☑	1005	1032.73	1031.72
☑	1006	1032.89	1031.89
☑	1008	1034.47	1033.47
☑	1009	1034.87	1033.87
☑	1010	1035.00	1033.99
☑	1011	1035.11	1034.10
☑	1014	1037.05	1036.04
☑	1015	1037.19	1036.18
☑	1016	1037.38	1036.38
☑	1018	1038.11	1037.10
☑	1019	1039.93	1038.92
☑	1020	1040.60	1039.59
☑	1021	520.84	1039.67
☑	1022	1041.26	1040.25
☑	1023	1042.18	1041.17
☑	1024	1043.06	1042.05
☑	1025	1043.39	1042.39
☑	1026	522.31	1042.60
☑	1027	522.31	1042.61
☑	1028	1045.27	1044.26
☑	1029	1045.42	1044.42
☑	1031	1046.09	1045.08

☑	1032	1046.53	1045.53
☑	1033	1048.26	1047.26
☑	1034	524.76	1047.50
☑	1035	1048.67	1047.66
☑	1036	1048.79	1047.78
☑	1037	525.24	1048.46
☑	1040	1051.64	1050.63
☑	1041	527.00	1051.98
☑	1042	1053.32	1052.32
☑	1043	527.26	1052.50
☑	1044	1054.03	1053.02
☑	1045	1054.09	1053.08
☑	1046	1054.50	1053.49
☑	1047	1056.03	1055.03
☑	1048	1056.38	1055.38
☑	1049	1056.66	1055.65
☑	1050	1057.00	1055.99
☑	1051	1058.11	1057.11
☑	1053	1059.51	1058.50
☑	1054	530.33	1058.65
☑	1055	530.80	1059.58
☑	1056	1061.25	1060.24
☑	1057	1061.83	1060.82
☑	1058	1062.36	1061.35
☑	1059	1062.83	1061.82
☑	1060	1063.06	1062.05
☑	1061	532.68	1063.35
☑	1062	1064.38	1063.37
☑	1065	1065.50	1064.49
☑	1067	533.32	1064.62
☑	1068	1067.13	1066.12
☑	1069	1067.62	1066.61
☑	1071	1068.57	1067.56
☑	1072	1069.37	1068.37

1073	1069.83	1068.82
1074	1070.73	1069.73
1075	536.14	1070.27
1076	536.27	1070.53
1077	536.29	1070.57
1078	536.29	1070.58
1080	1072.64	1071.64
1081	1074.54	1073.53
1082	1075.70	1074.70
1083	1076.00	1074.99
1084	1076.90	1075.89
1085	1076.92	1075.91
1086	1077.03	1076.02
1087	1077.23	1076.22
1089	1078.13	1077.12
1090	1078.39	1077.38
1092	1078.72	1077.71
1093	1079.14	1078.13
1094	1079.15	1078.14
1095	1079.22	1078.21
1096	1081.48	1080.47
1097	1082.83	1081.82
1098	1083.09	1082.08
1099	1084.15	1083.15
1100	1084.49	1083.49
1102	1084.83	1083.83
1103	1085.05	1084.04
1105	1085.55	1084.54
1106	1085.74	1084.73
1107	1086.27	1085.26
1108	1086.38	1085.37
1110	544.28	1086.54
1111	1087.59	1086.58
1113	544.72	1087.43

☑	1117	*1088.64*	*1087.63*
☑	1119	*1089.71*	*1088.70*
☑	1120	*1089.78*	*1088.77*
☑	1121	*1089.96*	*1088.95*
☑	1122	*1090.29*	*1089.28*
☑	1126	*1090.94*	*1089.93*
☑	1127	*1091.09*	*1090.08*
☑	1128	*1091.51*	*1090.50*
☑	1129	*1092.37*	*1091.36*
☑	1130	*546.80*	*1091.58*
☑	1131	*546.86*	*1091.71*
☑	1132	*1093.24*	*1092.24*
☑	1133	*1093.48*	*1092.47*
☑	1135	*1095.55*	*1094.54*
☑	1136	*1096.03*	*1095.02*
☑	1137	*1096.98*	*1095.98*
☑	1138	*549.77*	*1097.53*
☑	1139	*1098.55*	*1097.54*
☑	1140	*549.83*	*1097.64*
☑	1141	*1098.77*	*1097.76*
☑	1142	*1098.95*	*1097.94*
☑	1143	*1099.46*	*1098.46*
☑	1144	*1099.73*	*1098.73*
☑	1145	*1099.94*	*1098.93*
☑	1146	*1100.57*	*1099.56*
☑	1147	*550.84*	*1099.66*
☑	1148	*1101.27*	*1100.26*
☑	1149	*1102.46*	*1101.46*
☑	1150	*1102.73*	*1101.73*
☑	1151	*1102.77*	*1101.76*
☑	1152	*552.20*	*1102.39*
☑	1153	*552.74*	*1103.47*
☑	1154	*552.74*	*1103.47*
☑	1156	*1105.71*	*1104.71*

☑	1157	553.58	1105.15
☑	1159	1107.14	1106.14
☑	1160	1107.21	1106.20
☑	1161	554.13	1106.26
☑	1162	554.89	1107.78
☑	1164	1109.71	1108.71
☑	1165	1111.23	1110.22
☑	1166	1111.65	1110.64
☑	1167	1113.14	1112.13
☑	1168	1113.95	1112.94
☑	1170	1116.52	1115.51
☑	1173	559.33	1116.65
☑	1174	559.36	1116.70
☑	1175	1118.09	1117.08
☑	1176	1119.40	1118.39
☑	1177	1119.52	1118.51
☑	1178	1119.73	1118.73
☑	1179	1119.98	1118.98
☑	1180	1120.59	1119.58
☑	1182	1122.21	1121.20
☑	1183	1122.68	1121.67
☑	1184	1124.00	1122.99
☑	1185	1124.60	1123.60
☑	1188	563.31	1124.61
☑	1189	1126.48	1125.47
☑	1190	1127.12	1126.11
☑	1192	1127.74	1126.73
☑	1193	1128.46	1127.45
☑	1195	1128.80	1127.79
☑	1196	1129.95	1128.94
☑	1198	565.82	1129.63
☑	1199	1131.37	1130.36
☑	1200	566.45	1130.89
☑	1201	1133.00	1132.00

☑	1202	1133.01	1132.01
☑	1203	1133.78	1132.77
☑	1204	1134.19	1133.19
☑	1205	1135.25	1134.24
☑	1206	1135.31	1134.30
☑	1207	1135.32	1134.31
☑	1208	1135.73	1134.72
☑	1211	1139.08	1138.07
☑	1212	1139.15	1138.14
☑	1213	1139.25	1138.24
☑	1214	570.21	1138.40
☑	1215	570.31	1138.60
☑	1216	1140.36	1139.35
☑	1217	1140.56	1139.55
☑	1218	1141.23	1140.23
☑	1221	1142.78	1141.77
☑	1222	1142.96	1141.95
☑	1223	1143.44	1142.44
☑	1224	572.32	1142.63
☑	1225	573.30	1144.59
☑	1226	1146.41	1145.40
☑	1227	1147.95	1146.94
☑	1228	1148.80	1147.79
☑	1229	1149.08	1148.08
☑	1231	1149.65	1148.64
☑	1232	575.39	1148.77
☑	1233	1149.90	1148.89
☑	1234	1150.07	1149.07
☑	1235	1150.63	1149.62
☑	1236	576.27	1150.52
☑	1237	576.28	1150.54
☑	1238	1151.77	1150.76
☑	1239	1152.53	1151.53
☑	1240	1152.81	1151.81

☑	1241	577.32	1152.63
☑	1242	577.50	1152.98
☑	1243	1154.55	1153.55
☑	1244	1154.56	1153.55
☑	1245	1154.82	1153.82
☑	1246	578.22	1154.42
☑	1247	578.29	1154.56
☑	1248	1155.65	1154.64
☑	1251	578.87	1155.72
☑	1252	579.25	1156.48
☑	1253	1157.72	1156.71
☑	1254	579.77	1157.53
☑	1255	580.32	1158.62
☑	1256	580.33	1158.65
☑	1257	1160.73	1159.72
☑	1258	1161.67	1160.66
☑	1259	1162.18	1161.17
☑	1260	582.27	1162.52
☑	1261	1164.30	1163.30
☑	1262	1164.52	1163.52
☑	1263	583.26	1164.50
☑	1266	583.33	1164.64
☑	1268	1167.42	1166.41
☑	1269	1167.47	1166.46
☑	1270	1167.87	1166.87
☑	1271	584.79	1167.57
☑	1272	1168.66	1167.66
☑	1273	584.89	1167.77
☑	1274	390.44	1168.31
☑	1275	1170.08	1169.07
☑	1277	1171.17	1170.16
☑	1278	1171.26	1170.26
☑	1279	1171.40	1170.39
☑	1281	586.28	1170.55

☑	1283	*1172.14*	*1171.14*
☑	1284	*1172.59*	*1171.59*
☑	1285	*1173.70*	*1172.69*
☑	1287	*587.96*	*1173.91*
☑	1288	*1175.01*	*1174.00*
☑	1289	*1175.11*	*1174.10*
☑	1290	*1175.48*	*1174.48*
☑	1292	*1177.24*	*1176.23*
☑	1293	*1177.46*	*1176.45*
☑	1294	*1178.88*	*1177.87*
☑	1295	*1179.68*	*1178.68*
☑	1296	*393.95*	*1178.83*
☑	1297	*1179.89*	*1178.88*
☑	1300	*591.82*	*1181.63*
☑	1301	*1182.65*	*1181.64*
☑	1302	*1182.68*	*1181.67*
☑	1303	*1183.17*	*1182.16*
☑	1305	*592.50*	*1182.99*
☑	1306	*1184.52*	*1183.51*
☑	1307	*1184.94*	*1183.93*
☑	1308	*593.14*	*1184.26*
☑	1309	*1186.80*	*1185.79*
☑	1310	*594.07*	*1186.12*
☑	1313	*594.83*	*1187.64*
☑	1315	*1188.78*	*1187.77*
☑	1316	*1189.15*	*1188.14*
☑	1317	*1190.65*	*1189.64*
☑	1318	*1191.10*	*1190.09*
☑	1319	*596.30*	*1190.58*
☑	1321	*597.49*	*1192.97*
☑	1322	*1194.27*	*1193.26*
☑	1323	*597.65*	*1193.28*
☑	1324	*1195.40*	*1194.40*
☑	1325	*1196.28*	*1195.28*

☑	1326	599.81	1197.61
☑	1328	1198.89	1197.88
☑	1329	600.33	1198.65
☑	1330	1200.23	1199.22
☑	1331	1201.93	1200.92
☑	1332	1203.15	1202.14
☑	1333	1203.42	1202.41
☑	1334	1203.57	1202.56
☑	1340	602.87	1203.72
☑	1342	603.36	1204.70
☑	1343	1205.96	1204.96
☑	1344	604.82	1207.62
☑	1345	1209.52	1208.51
☑	1346	1210.00	1209.00
☑	1347	1210.18	1209.17
☑	1348	605.78	1209.55
☑	1349	1210.74	1209.73
☑	1350	1211.83	1210.83
☑	1351	1212.48	1211.48
☑	1352	606.84	1211.67
☑	1355	1213.91	1212.90
☑	1357	1216.52	1215.52
☑	1358	1217.10	1216.09
☑	1359	1217.58	1216.57
☑	1361	609.80	1217.59
☑	1362	1220.49	1219.48
☑	1364	1221.10	1220.09
☑	1365	1221.25	1220.24
☑	1367	611.37	1220.72
☑	1369	611.93	1221.85
☑	1370	611.98	1221.94
☑	1371	1223.73	1222.72
☑	1372	612.58	1223.15
☑	1373	1224.59	1223.58

☑	1374	612.84	1223.67
☑	1375	613.32	1224.63
☑	1376	1226.39	1225.38
☑	1377	1227.17	1226.16
☑	1381	615.72	1229.42
☑	1382	410.95	1229.82
☑	1384	616.60	1231.19
☑	1385	1232.64	1231.63
☑	1387	1233.27	1232.26
☑	1388	1233.31	1232.31
☑	1390	617.87	1233.72
☑	1391	618.24	1234.47
☑	1392	1235.58	1234.58
☑	1393	1237.30	1236.29
☑	1394	1237.70	1236.70
☑	1395	1238.34	1237.33
☑	1398	619.88	1237.75
☑	1400	620.26	1238.51
☑	1401	620.31	1238.61
☑	1402	620.33	1238.65
☑	1403	1240.18	1239.17
☑	1404	1241.23	1240.22
☑	1406	621.28	1240.54
☑	1407	621.36	1240.70
☑	1408	621.37	1240.73
☑	1409	1242.19	1241.18
☑	1411	1243.44	1242.44
☑	1412	622.33	1242.65
☑	1413	622.34	1242.67
☑	1414	622.92	1243.83
☑	1415	623.31	1244.61
☑	1416	1245.68	1244.67
☑	1419	1249.21	1248.20
☑	1420	1250.59	1249.59

☑	1421	1251.48	1250.47
☑	1422	1251.79	1250.78
☑	1423	1251.80	1250.79
☑	1424	1252.82	1251.82
☑	1425	418.54	1252.59
☑	1426	627.34	1252.66
☑	1427	627.40	1252.78
☑	1428	1254.55	1253.54
☑	1429	627.80	1253.59
☑	1430	629.37	1256.73
☑	1432	629.89	1257.77
☑	1433	629.92	1257.82
☑	1434	1259.14	1258.13
☑	1435	1261.07	1260.06
☑	1438	631.82	1261.63
☑	1439	421.56	1261.66
☑	1440	421.73	1262.15
☑	1442	632.49	1262.97
☑	1443	1265.32	1264.31
☑	1444	633.29	1264.57
☑	1445	1265.59	1264.58
☑	1447	1265.89	1264.88
☑	1448	1266.03	1265.02
☑	1454	635.08	1268.15
☑	1455	635.35	1268.69
☑	1456	1273.70	1272.69
☑	1457	1275.45	1274.44
☑	1459	1278.79	1277.78
☑	1460	640.33	1278.65
☑	1462	640.38	1278.74
☑	1463	641.31	1280.60
☑	1466	642.73	1283.45
☑	1467	643.26	1284.52
☑	1470	644.81	1287.60

☑	1471	1289.11	1288.10
☑	1473	646.87	1291.72
☑	1474	647.27	1292.53
☑	1477	647.75	1293.48
☑	1478	647.85	1293.69
☑	1479	432.24	1293.71
☑	1480	647.93	1293.85
☑	1481	648.10	1294.19
☑	1482	648.31	1294.60
☑	1483	1295.94	1294.93
☑	1484	1296.45	1295.45
☑	1486	1298.80	1297.79
☑	1488	433.93	1298.76
☑	1489	433.99	1298.96
☑	1493	650.94	1299.87
☑	1496	1303.17	1302.17
☑	1497	1304.12	1303.11
☑	1498	652.85	1303.68
☑	1500	436.49	1306.43
☑	1502	654.91	1307.81
☑	1503	655.36	1308.70
☑	1504	655.38	1308.75
☑	1505	437.60	1309.78
☑	1506	1314.15	1313.14
☑	1507	658.30	1314.59
☑	1508	658.31	1314.61
☑	1513	660.86	1319.70
☑	1515	662.38	1322.75
☑	1516	1325.05	1324.04
☑	1518	1326.12	1325.11
☑	1519	663.93	1325.85
☑	1520	664.04	1326.06
☑	1526	669.39	1336.77
☑	1527	669.48	1336.95

☑	1530	670.87	1339.73
☑	1534	673.00	1344.00
☑	1535	673.36	1344.70
☑	1536	1348.58	1347.57
☑	1540	675.30	1348.60
☑	1541	1350.22	1349.21
☑	1542	676.25	1350.49
☑	1543	676.42	1350.83
☑	1544	678.01	1354.01
☑	1545	678.10	1354.19
☑	1546	678.56	1355.11
☑	1549	453.58	1357.71
☑	1551	681.33	1360.65
☑	1552	682.87	1363.73
☑	1553	682.89	1363.77
☑	1554	683.32	1364.63
☑	1555	1369.71	1368.71
☑	1556	685.92	1369.84
☑	1557	686.20	1370.38
☑	1560	686.35	1370.69
☑	1562	689.36	1376.70
☑	1563	689.91	1377.80
☑	1564	690.35	1378.69
☑	1565	690.39	1378.77
☑	1566	1380.54	1379.53
☑	1573	692.39	1382.78
☑	1574	692.53	1383.05
☑	1578	692.94	1383.87
☑	1579	693.34	1384.66
☑	1580	463.28	1386.82
☑	1583	695.82	1389.63
☑	1585	696.93	1391.85
☑	1586	696.94	1391.87
☑	1587	465.25	1392.72

☑	1588	698.52	1395.02
☑	1589	467.27	1398.78
☑	1590	702.96	1403.90
☑	1591	703.39	1404.76
☑	1593	704.30	1406.59
☑	1595	704.90	1407.79
☑	1600	472.91	1415.71
☑	1601	472.93	1415.78
☑	1604	709.65	1417.30
☑	1605	710.31	1418.60
☑	1606	474.60	1420.79
☑	1607	711.88	1421.74
☑	1609	713.39	1424.77
☑	1610	713.96	1425.90
☑	1613	715.88	1429.74
☑	1614	717.85	1433.68
☑	1615	478.92	1433.74
☑	1616	717.89	1433.78
☑	1617	718.74	1435.47
☑	1620	719.32	1436.63
☑	1621	479.89	1436.64
☑	1622	719.40	1436.78
☑	1623	719.90	1437.78
☑	1624	720.38	1438.75
☑	1625	720.92	1439.83
☑	1626	480.97	1439.89
☑	1627	721.66	1441.30
☑	1632	724.24	1446.47
☑	1633	724.38	1446.75
☑	1636	725.82	1449.62
☑	1637	725.86	1449.70
☑	1641	727.51	1453.01
☑	1643	728.27	1454.53
☑	1645	486.72	1457.14

☑	1646	730.21	1458.40
☑	1648	730.90	1459.79
☑	1651	732.94	1463.86
☑	1652	733.13	1464.24
☑	1653	734.34	1466.67
☑	1656	490.25	1467.74
☑	1657	735.32	1468.62
☑	1659	736.91	1471.81
☑	1660	737.08	1472.15
☑	1661	737.29	1472.56
☑	1664	738.57	1475.13
☑	1666	741.79	1481.56
☑	1667	741.85	1481.69
☑	1669	495.70	1484.07
☑	1671	496.23	1485.67
☑	1675	747.33	1492.65
☑	1677	498.60	1492.78
☑	1678	500.31	1497.90
☑	1682	753.31	1504.61
☑	1685	755.35	1508.69
☑	1686	755.62	1509.23
☑	1687	755.80	1509.59
☑	1688	755.85	1509.68
☑	1690	505.10	1512.28
☑	1691	757.38	1512.75
☑	1692	505.86	1514.56
☑	1693	758.91	1515.81
☑	1696	507.26	1518.75
☑	1697	760.99	1519.96
☑	1698	761.84	1521.66
☑	1699	762.00	1521.99
☑	1700	762.12	1522.23
☑	1702	763.03	1524.05
☑	1703	763.29	1524.56

☑	1704	763.82	1525.63
☑	1705	763.86	1525.71
☑	1706	764.83	1527.64
☑	1708	764.85	1527.69
☑	1709	764.86	1527.71
☑	1710	764.87	1527.72
☑	1711	765.32	1528.62
☑	1712	767.36	1532.70
☑	1713	769.05	1536.08
☑	1714	513.60	1537.77
☑	1715	770.38	1538.74
☑	1723	777.36	1552.71
☑	1725	778.91	1555.80
☑	1728	780.28	1558.55
☑	1729	780.92	1559.84
☑	1730	521.29	1560.85
☑	1731	781.96	1561.91
☑	1733	783.84	1565.67
☑	1735	523.29	1566.84
☑	1736	785.22	1568.42
☑	1737	523.90	1568.69
☑	1738	785.41	1568.80
☑	1739	785.68	1569.34
☑	1740	785.84	1569.66
☑	1741	786.19	1570.37
☑	1742	786.38	1570.75
☑	1743	786.98	1571.94
☑	1745	787.37	1572.73
☑	1746	788.71	1575.41
☑	1747	788.87	1575.73
☑	1749	528.25	1581.73
☑	1750	792.85	1583.68
☑	1751	528.96	1583.86
☑	1754	793.85	1585.69

☑	1757	795.97	1589.93
☑	1758	796.86	1591.70
☑	1759	797.90	1593.78
☑	1763	800.91	1599.80
☑	1766	536.95	1607.83
☑	1767	537.60	1609.77
☑	1768	806.14	1610.26
☑	1769	806.23	1610.45
☑	1771	538.93	1613.76
☑	1772	539.11	1614.31
☑	1774	539.85	1616.54
☑	1776	539.91	1616.71
☑	1777	810.24	1618.46
☑	1778	810.56	1619.11
☑	1781	541.94	1622.81
☑	1784	813.45	1624.88
☑	1785	814.93	1627.85
☑	1786	815.25	1628.49
☑	1788	544.85	1631.54
☑	1789	817.07	1632.13
☑	1791	819.46	1636.90
☑	1792	819.81	1637.60
☑	1794	548.25	1641.72
☑	1795	822.35	1642.68
☑	1798	550.21	1647.62
☑	1799	550.96	1649.87
☑	1803	827.93	1653.85
☑	1804	827.94	1653.87
☑	1805	552.38	1654.12
☑	1806	828.43	1654.85
☑	1807	828.46	1654.90
☑	1809	830.89	1659.77
☑	1810	554.29	1659.84
☑	1811	831.01	1660.00

☑	1814	831.94	1661.86
☑	1815	833.86	1665.71
☑	1816	557.26	1668.76
☑	1817	835.45	1668.89
☑	1818	835.46	1668.91
☑	1819	835.98	1669.94
☑	1820	837.47	1672.92
☑	1821	837.95	1673.89
☑	1822	559.13	1674.38
☑	1823	561.96	1682.86
☑	1824	844.85	1687.69
☑	1826	563.93	1688.76
☑	1827	845.84	1689.66
☑	1830	846.93	1691.84
☑	1831	564.99	1691.94
☑	1832	847.37	1692.73
☑	1833	847.47	1692.93
☑	1834	848.14	1694.26
☑	1835	848.27	1694.53
☑	1836	848.33	1694.64
☑	1837	848.77	1695.53
☑	1838	567.17	1698.50
☑	1839	850.64	1699.27
☑	1840	850.73	1699.45
☑	1841	567.73	1700.17
☑	1842	851.34	1700.67
☑	1843	567.98	1700.92
☑	1845	851.89	1701.76
☑	1846	568.91	1703.71
☑	1847	854.38	1706.74
☑	1848	855.44	1708.87
☑	1850	856.16	1710.30
☑	1851	857.33	1712.64
☑	1852	857.47	1712.92

☑	1856	858.90	1715.79
☑	1858	862.90	1723.78
☑	1860	863.17	1724.32
☑	1861	576.63	1726.87
☑	1862	865.64	1729.26
☑	1863	578.27	1731.80
☑	1864	868.10	1734.19
☑	1865	869.48	1736.94
☑	1866	872.40	1742.79
☑	1868	874.47	1746.92
☑	1869	583.53	1747.57
☑	1870	876.21	1750.41
☑	1871	876.92	1751.83
☑	1873	878.43	1754.85
☑	1875	586.29	1755.86
☑	1876	881.38	1760.75
☑	1877	881.46	1760.90
☑	1878	881.76	1761.51
☑	1879	588.23	1761.67
☑	1880	588.66	1762.95
☑	1881	883.83	1765.64
☑	1882	589.58	1765.72
☑	1883	886.05	1770.10
☑	1884	886.94	1771.86
☑	1887	592.09	1773.26
☑	1888	592.54	1774.61
☑	1889	594.54	1780.60
☑	1890	892.46	1782.90
☑	1891	596.00	1784.97
☑	1895	895.34	1788.67
☑	1897	896.38	1790.74
☑	1898	897.06	1792.11
☑	1899	898.95	1795.88
☑	1901	899.45	1796.88

☑	1902	600.27	1797.79
☑	1903	899.91	1797.80
☑	1904	600.28	1797.82
☑	1905	901.92	1801.83
☑	1907	603.33	1806.97
☑	1908	604.03	1809.07
☑	1910	905.84	1809.67
☑	1914	906.48	1810.94
☑	1915	604.67	1810.99
☑	1916	604.91	1811.71
☑	1917	605.05	1812.13
☑	1918	605.80	1814.37
☑	1919	908.49	1814.96
☑	1920	908.96	1815.91
☑	1921	606.59	1816.75
☑	1922	606.62	1816.83
☑	1926	609.92	1826.74
☑	1927	916.21	1830.40
☑	1928	916.52	1831.02
☑	1929	916.79	1831.56
☑	1931	611.96	1832.85
☑	1932	917.90	1833.78
☑	1933	918.10	1834.19
☑	1934	612.41	1834.21
☑	1935	612.87	1835.58
☑	1937	918.91	1835.80
☑	1939	918.92	1835.83
☑	1941	919.47	1836.93
☑	1942	614.51	1840.52
☑	1943	614.63	1840.87
☑	1947	924.44	1846.87
☑	1948	925.62	1849.23
☑	1950	618.84	1853.48
☑	1951	618.99	1853.95

☑	1952	929.41	1856.80
☑	1953	930.28	1858.56
☑	1955	931.44	1860.87
☑	1956	931.47	1860.92
☑	1957	937.64	1873.27
☑	1958	937.91	1873.80
☑	1959	938.36	1874.71
☑	1960	939.94	1877.86
☑	1961	940.46	1878.91
☑	1962	942.41	1882.81
☑	1963	629.02	1884.04
☑	1964	629.42	1885.23
☑	1965	629.97	1886.90
☑	1966	631.54	1891.59
☑	1967	632.69	1895.04
☑	1968	949.52	1897.02
☑	1969	952.12	1902.22
☑	1970	953.42	1904.84
☑	1971	954.43	1906.84
☑	1972	636.75	1907.24
☑	1973	637.14	1908.39
☑	1974	956.16	1910.31
☑	1975	637.78	1910.31
☑	1976	959.50	1917.00
☑	1977	959.89	1917.77
☑	1978	640.61	1918.81
☑	1979	961.21	1920.41
☑	1980	641.35	1921.02
☑	1982	962.05	1922.09
☑	1983	643.23	1926.66
☑	1984	965.78	1929.55
☑	1985	967.03	1932.04
☑	1986	645.35	1933.03
☑	1987	968.09	1934.16

1988	970.45	1938.89
1989	973.43	1944.84
1990	976.96	1951.90
1991	979.83	1957.64
1992	979.92	1957.84
1993	980.52	1959.03
1994	654.36	1960.04
1996	655.24	1962.71
1998	983.63	1965.24
1999	656.63	1966.88
2000	984.93	1967.85
2002	988.32	1974.63
2003	660.05	1977.13
2004	660.20	1977.58
2005	990.62	1979.23
2006	991.02	1980.03
2008	991.92	1981.83
2009	992.43	1982.85
2010	993.98	1985.95
2011	995.85	1989.69
2013	996.75	1991.48
2014	997.01	1992.01
2015	997.53	1993.05
2016	665.38	1993.11
2017	999.00	1996.00
2018	999.89	1997.77
2019	1000.71	1999.40
2020	1004.54	2007.07
2021	1004.69	2007.37
2022	670.34	2007.99
2024	670.58	2008.73
2025	670.68	2009.03
2026	1005.54	2009.08
2027	672.31	2013.91

☑	2029	1008.26	2014.50
☑	2030	673.28	2016.81
☑	2031	1009.45	2016.88
☑	2032	1009.68	2017.34
☑	2033	1010.01	2018.00
☑	2034	1011.49	2020.97
☑	2035	675.59	2023.74
☑	2036	675.62	2023.85
☑	2038	1014.24	2026.46
☑	2039	1014.35	2026.69
☑	2040	1014.67	2027.33
☑	2041	677.36	2029.07
☑	2042	1015.65	2029.28
☑	2044	679.18	2034.52
☑	2045	1018.46	2034.90
☑	2046	1018.48	2034.95
☑	2047	679.35	2035.01
☑	2048	1018.70	2035.38
☑	2049	1019.03	2036.05
☑	2050	1021.98	2041.95
☑	2051	682.09	2043.24
☑	2052	1023.73	2045.44
☑	2054	685.02	2052.05
☑	2055	1027.48	2052.96
☑	2056	685.33	2052.97
☑	2057	1027.59	2053.16
☑	2058	1029.53	2057.05
☑	2059	1032.66	2063.30
☑	2060	1033.12	2064.22
☑	2061	1037.04	2072.06
☑	2062	1038.27	2074.53
☑	2063	692.72	2075.14
☑	2064	1039.13	2076.24
☑	2065	1039.93	2077.85

☑	2066	1040.26	2078.50
☑	2067	1041.34	2080.66
☑	2068	1041.98	2081.95
☑	2069	1042.77	2083.53
☑	2070	696.21	2085.61
☑	2071	1044.27	2086.52
☑	2072	697.90	2090.68
☑	2073	1046.50	2090.99
☑	2074	1047.47	2092.93
☑	2075	1047.74	2093.47
☑	2076	1047.94	2093.88
☑	2077	1048.52	2095.03
☑	2078	700.09	2097.24
☑	2079	700.33	2097.97
☑	2080	1050.04	2098.07
☑	2081	1050.59	2099.16
☑	2082	1052.02	2102.02
☑	2084	702.79	2105.36
☑	2085	703.34	2107.00
☑	2086	1054.57	2107.12
☑	2087	1055.23	2108.45
☑	2088	1055.99	2109.96
☑	2089	1057.83	2113.65
☑	2090	1058.24	2114.47
☑	2091	705.95	2114.83
☑	2092	707.02	2118.05
☑	2093	1061.07	2120.13
☑	2094	1061.11	2120.20
☑	2095	1061.17	2120.33
☑	2096	707.79	2120.35
☑	2097	1062.30	2122.58
☑	2098	1062.35	2122.68
☑	2099	1062.65	2123.29
☑	2100	1064.68	2127.34

☑	2101	710.26	2127.77
☑	2102	710.88	2129.62
☑	2103	1066.50	2130.99
☑	2104	1069.99	2137.96
☑	2106	715.01	2142.00
☑	2107	1072.07	2142.13
☑	2108	715.42	2143.25
☑	2109	1072.77	2143.53
☑	2110	1072.85	2143.68
☑	2112	717.96	2150.86
☑	2113	718.03	2151.07
☑	2114	1077.70	2153.40
☑	2115	1079.54	2157.06
☑	2116	720.08	2157.22
☑	2117	720.11	2157.30
☑	2118	1080.47	2158.93
☑	2119	1080.61	2159.20
☑	2120	722.45	2164.34
☑	2122	1083.84	2165.67
☑	2123	1085.51	2169.01
☑	2124	725.01	2172.01
☑	2125	725.02	2172.04
☑	2126	727.12	2178.33
☑	2127	1090.47	2178.93
☑	2128	727.66	2179.96
☑	2129	728.66	2182.97
☑	2130	1092.89	2183.77
☑	2131	730.07	2187.18
☑	2132	1095.49	2188.96
☑	2133	730.70	2189.06
☑	2134	731.42	2191.23
☑	2135	1097.14	2192.26
☑	2136	731.92	2192.74
☑	2137	732.64	2194.89

☑	2138	1098.62	2195.22
☑	2139	733.16	2196.47
☑	2140	1101.30	2200.58
☑	2141	1103.91	2205.81
☑	2142	1104.20	2206.39
☑	2143	1104.67	2207.33
☑	2144	1104.93	2207.85
☑	2145	737.04	2208.11
☑	2146	1106.02	2210.03
☑	2147	737.69	2210.06
☑	2148	737.77	2210.28
☑	2149	737.91	2210.70
☑	2150	1106.45	2210.89
☑	2151	738.04	2211.11
☑	2153	1108.75	2215.49
☑	2154	1108.79	2215.57
☑	2155	741.08	2220.22
☑	2156	1112.59	2223.17
☑	2157	742.13	2223.37
☑	2158	742.24	2223.70
☑	2159	1113.11	2224.21
☑	2160	1113.13	2224.25
☑	2161	1114.43	2226.85
☑	2162	745.26	2232.76
☑	2163	1117.42	2232.83
☑	2164	745.69	2234.04
☑	2165	746.38	2236.12
☑	2166	747.14	2238.39
☑	2167	747.29	2238.85
☑	2169	747.74	2240.20
☑	2170	747.76	2240.27
☑	2171	747.82	2240.43
☑	2172	1121.52	2241.02
☑	2173	1124.09	2246.16

☑	2174	750.04	2247.10
☑	2175	1124.56	2247.11
☑	2176	1124.65	2247.28
☑	2177	750.39	2248.16
☑	2178	1126.02	2250.02
☑	2179	751.05	2250.12
☑	2181	751.07	2250.19
☑	2182	751.77	2252.29
☑	2183	752.40	2254.19
☑	2184	1128.41	2254.81
☑	2185	753.35	2257.02
☑	2186	753.83	2258.47
☑	2187	1132.55	2263.09
☑	2188	756.05	2265.14
☑	2189	1133.91	2265.80
☑	2190	1134.11	2266.21
☑	2191	757.04	2268.09
☑	2192	757.35	2269.02
☑	2193	1136.09	2270.16
☑	2194	1136.68	2271.34
☑	2195	1137.52	2273.03
☑	2196	759.08	2274.21
☑	2197	1140.55	2279.08
☑	2198	761.43	2281.27
☑	2199	1142.74	2283.47
☑	2200	762.41	2284.20
☑	2201	762.80	2285.39
☑	2202	1147.82	2293.62
☑	2203	1148.18	2294.35
☑	2205	1148.85	2295.68
☑	2206	766.35	2296.02
☑	2207	767.11	2298.31
☑	2208	767.14	2298.39
☑	2209	767.15	2298.42

☑	2210	768.08	2301.22
☑	2211	769.38	2305.12
☑	2212	1153.85	2305.68
☑	2213	770.00	2306.97
☑	2214	1154.71	2307.40
☑	2215	1156.77	2311.53
☑	2216	772.10	2313.28
☑	2217	1158.35	2314.68
☑	2218	774.53	2320.57
☑	2219	775.58	2323.72
☑	2220	775.71	2324.11
☑	2221	777.90	2330.69
☑	2222	1169.51	2337.01
☑	2223	780.78	2339.31
☑	2224	781.06	2340.16
☑	2225	1172.92	2343.83
☑	2226	783.64	2347.91
☑	2227	783.70	2348.07
☑	2229	1176.20	2350.39
☑	2230	784.70	2351.09
☑	2231	785.86	2354.55
☑	2232	1178.34	2354.67
☑	2233	785.99	2354.94
☑	2234	786.48	2356.42
☑	2235	787.24	2358.69
☑	2236	1181.28	2360.55
☑	2237	789.11	2364.32
☑	2238	789.13	2364.37
☑	2239	790.02	2367.04
☑	2240	790.13	2367.38
☑	2241	792.99	2375.95
☑	2242	793.09	2376.24
☑	2243	793.42	2377.24
☑	2244	1189.89	2377.78

☑	2245	793.91	2378.72
☑	2246	793.93	2378.77
☑	2247	794.39	2380.16
☑	2248	795.29	2382.85
☑	2250	798.39	2392.15
☑	2251	1197.52	2393.03
☑	2252	798.73	2393.15
☑	2253	802.03	2403.07
☑	2254	1203.01	2404.01
☑	2255	1203.27	2404.52
☑	2256	805.09	2412.23
☑	2258	806.09	2415.25
☑	2259	1210.54	2419.06
☑	2260	809.73	2426.17
☑	2261	809.75	2426.24
☑	2262	812.96	2435.85
☑	2263	813.16	2436.47
☑	2264	814.60	2440.78
☑	2265	815.39	2443.16
☑	2266	817.75	2450.22
☑	2267	818.75	2453.22
☑	2268	1227.83	2453.64
☑	2269	819.97	2456.89
☑	2270	821.67	2461.98
☑	2271	821.74	2462.19
☑	2272	823.12	2466.32
☑	2273	823.42	2467.23
☑	2274	1236.58	2471.15
☑	2276	826.15	2475.42
☑	2277	826.33	2475.98
☑	2278	827.72	2480.14
☑	2279	829.37	2485.09
☑	2280	829.72	2486.15
☑	2281	830.45	2488.33

☑	2282	831.05	2490.12
☑	2283	1246.11	2490.20
☑	2284	831.27	2490.79
☑	2285	1247.54	2493.06
☑	2286	1248.09	2494.16
☑	2287	833.51	2497.52
☑	2288	834.33	2499.97
☑	2289	835.01	2502.02
☑	2291	836.41	2506.21
☑	2292	837.34	2508.98
☑	2293	837.50	2509.48
☑	2296	838.02	2511.04
☑	2297	839.39	2515.14
☑	2299	843.48	2527.43
☑	2300	843.76	2528.26
☑	2303	844.86	2531.56
☑	2305	845.07	2532.18
☑	2306	1267.31	2532.60
☑	2307	845.74	2534.21
☑	2308	847.93	2540.77
☑	2309	848.48	2542.43
☑	2310	848.76	2543.26
☑	2311	849.06	2544.16
☑	2313	850.74	2549.20
☑	2314	851.40	2551.19
☑	2315	851.43	2551.26
☑	2316	851.71	2552.11
☑	2318	854.11	2559.30
☑	2319	854.97	2561.89
☑	2320	1282.28	2562.54
☑	2324	858.89	2573.65
☑	2325	861.46	2581.36
☑	2326	861.50	2581.49
☑	2327	862.14	2583.40

☑	2328	862.40	2584.18
☑	2330	866.39	2596.14
☑	2331	867.52	2599.54
☑	2332	873.95	2618.82
☑	2333	874.75	2621.24
☑	2334	1313.39	2624.78
☑	2335	876.14	2625.39
☑	2336	876.41	2626.20
☑	2338	877.12	2628.33
☑	2339	877.67	2629.98
☑	2340	880.00	2636.98
☑	2341	880.13	2637.36
☑	2342	880.40	2638.19
☑	2344	881.47	2641.38
☑	2345	882.74	2645.19
☑	2346	883.16	2646.45
☑	2347	883.48	2647.41
☑	2348	886.03	2655.07
☑	2349	893.96	2678.86
☑	2350	896.66	2686.95
☑	2351	898.11	2691.30
☑	2352	899.50	2695.48
☑	2353	900.28	2697.82
☑	2354	901.16	2700.45
☑	2356	904.42	2710.25
☑	2357	904.48	2710.42
☑	2358	904.92	2711.74
☑	2361	906.34	2716.01
☑	2362	1360.81	2719.60
☑	2363	910.21	2727.61
☑	2364	910.82	2729.44
☑	2365	912.70	2735.08
☑	2366	914.63	2740.88
☑	2368	918.26	2751.76

☑	2369	919.32	2754.93
☑	2370	919.53	2755.56
☑	2371	921.39	2761.14
☑	2372	921.58	2761.72
☑	2373	922.14	2763.40
☑	2374	922.20	2763.57
☑	2375	924.36	2770.06
☑	2376	928.78	2783.32
☑	2377	930.26	2787.75
☑	2378	939.54	2815.59
☑	2379	939.66	2815.97
☑	2380	941.81	2822.41
☑	2381	942.05	2823.12
☑	2382	945.99	2834.95
☑	2383	949.72	2846.15
☑	2384	949.78	2846.32
☑	2385	952.46	2854.35
☑	2386	952.94	2855.80
☑	2387	953.10	2856.27
☑	2388	953.36	2857.04
☑	2389	957.84	2870.49
☑	2390	958.41	2872.22
☑	2391	959.67	2875.98
☑	2392	960.01	2877.01
☑	2393	960.18	2877.53
☑	2394	961.20	2880.57
☑	2395	962.54	2884.61
☑	2396	963.76	2888.26
☑	2397	964.05	2889.14
☑	2398	965.44	2893.30
☑	2399	967.60	2899.77
☑	2400	967.86	2900.57
☑	2401	972.45	2914.32
☑	2402	976.71	2927.10

☑	2403	977.43	2929.26
☑	2404	981.07	2940.18
☑	2405	981.97	2942.89
☑	2406	982.27	2943.79
☑	2407	983.74	2948.19
☑	2408	983.79	2948.34
☑	2409	984.19	2949.56
☑	2410	987.18	2958.51
☑	2411	990.12	2967.32
☑	2412	992.07	2973.18
☑	2413	994.03	2979.08
☑	2414	994.13	2979.36
☑	2415	995.45	2983.32
☑	2416	1000.91	2999.70
☑	2417	1001.87	3002.58
☑	2418	1002.68	3005.03
☑	2419	1003.56	3007.66
☑	2420	1004.78	3011.33
☑	2421	1006.08	3015.21
☑	2422	1006.46	3016.34
☑	2423	1007.00	3017.97
☑	2424	1009.45	3025.33
☑	2425	1011.36	3031.05
☑	2426	1013.87	3038.58
☑	2427	1015.82	3044.43
☑	2428	1016.43	3046.27
☑	2429	1024.56	3070.67
☑	2430	1028.59	3082.75
☑	2431	1029.54	3085.60
☑	2432	1030.39	3088.15
☑	2434	1034.25	3099.73
☑	2435	1035.58	3103.71
☑	2436	1036.51	3106.51
☑	2437	1037.88	3110.61

☑	2438	*1040.90*	*3119.67*
☑	2439	*1041.71*	*3122.10*
☑	2440	*1042.89*	*3125.65*
☑	2441	*1042.98*	*3125.92*
☑	2442	*1043.47*	*3127.39*
☑	2443	*1044.40*	*3130.18*
☑	2444	*1044.63*	*3130.88*
☑	2445	*1047.20*	*3138.58*
☑	2446	*1050.89*	*3149.65*
☑	2447	*1052.29*	*3153.84*
☑	2448	*1055.88*	*3164.60*
☑	2449	*1057.48*	*3169.43*
☑	2450	*1059.68*	*3176.03*
☑	2451	*1065.54*	*3193.60*
☑	2452	*1066.93*	*3197.76*
☑	2453	*1072.97*	*3215.89*
☑	2454	*1082.56*	*3244.65*
☑	2455	*1084.31*	*3249.91*
☑	2456	*1086.46*	*3256.35*
☑	2457	*1088.48*	*3262.42*
☑	2458	*1093.52*	*3277.55*
☑	2460	*1095.81*	*3284.41*
☑	2461	*1096.08*	*3285.23*
☑	2462	*1098.74*	*3293.20*
☑	2463	*1099.67*	*3296.00*
☑	2464	*1100.58*	*3298.72*
☑	2465	*1101.56*	*3301.64*
☑	2466	*1108.69*	*3323.06*
☑	2467	*1111.43*	*3331.27*
☑	2468	*1111.94*	*3332.80*
☑	2469	*1117.34*	*3348.99*
☑	2470	*1118.55*	*3352.62*
☑	2471	*1121.70*	*3362.08*
☑	2472	*1122.03*	*3363.08*

☑	2473	*1123.94*	*3368.81*
☑	2474	*1126.55*	*3376.63*
☑	2475	*1126.69*	*3377.06*
☑	2476	*1127.45*	*3379.34*
☑	2477	*1135.72*	*3404.14*
☑	2478	*1140.42*	*3418.24*
☑	2479	*1149.18*	*3444.53*
☑	2480	*1158.56*	*3472.66*
☑	2481	*1161.85*	*3482.51*
☑	2482	*1163.00*	*3485.99*
☑	2483	*1164.18*	*3489.51*
☑	2484	*1172.86*	*3515.56*
☑	2485	*1177.96*	*3530.85*
☑	2486	*1180.51*	*3538.52*
☑	2487	*1184.95*	*3551.83*
☑	2488	*1189.33*	*3564.97*
☑	2489	*1206.96*	*3617.86*
☑	2490	*1208.48*	*3622.43*
☑	2491	*1213.94*	*3638.79*
☑	2492	*1222.46*	*3664.35*
☑	2493	*1247.41*	*3739.20*
☑	2494	*1257.25*	*3768.74*
☑	2495	*1260.04*	*3777.10*

Search Parameters

```
Type of search         : MS/MS Ion Search
Enzyme                 : Trypsin
Variable modifications : Carbamidomethyl (C),Oxidation (M)
Mass values            : Monoisotopic
Protein Mass           : Unrestricted
Peptide Mass Tolerance : ± 0.6 Da
Fragment Mass Tolerance: ± 0.3 Da
Max Missed Cleavages   : 2
Instrument type        : ESI-TRAP
Number of queries      : 2495
```

Mascot: http://www.matrixscience.com/
