# Supplementary material for: Macrophage-infectivity potentiator of Trypanosoma cruzi (TcMIP) is a new pro-type 1 immuno-stimulating protein for neonatal human cells and vaccines in mice
Source: Front Immunol. 2023 Mar 23;14:1138526. doi: 10.3389/fimmu.2023.1138526 (PMC10077492; doi:10.3389/fimmu.2023.1138526)
Supplement: Supplementary file 9 [file DataSheet_7.pdf]

Mascot Search Results

User : GM
Email : gabriel.mazzucchelli@ulg.ac.be
Search title : Submitted from 090220-adj-oge42-9454-otherEuk-NCBi by Mascot Daemon on MASPEC39
MS data file : F:\DATA\Archives\ESQUIRE\2009 esquire\Adjuvac\090220\090220-Adj-OGE42_9454.mgf
Database : NCBInr 20090202 (6497659 sequences; 2228453757 residues)
Taxonomy : Other Eukaryota (159614 sequences)
Timestamp : 23 Feb 2009 at 08:44:23 GMT

Significant hits:

gi|71655864	I/6 autoantigen [Trypanosoma cruzi strain CL Brener]
gi|295363	heat shock protein 60
gi|50659756	heat shock protein 70 [Trypanosoma cruzi]
gi|71425779	I/6 autoantigen [Trypanosoma cruzi strain CL Brener]
gi|71400377	microtubule-associated protein [Trypanosoma cruzi strain CL Brener]
gi|3885846	beta tubulin [Trypanosoma cruzi]
gi|71651558	hypothetical protein [Trypanosoma cruzi strain CL Brener]
gi|162030	calmodulin A
gi|71413559	hypothetical protein [Trypanosoma cruzi strain CL Brener]
gi|71407337	hypothetical protein [Trypanosoma cruzi strain CL Brener]
gi|10673	unnamed protein product [Trypanosoma cruzi]
gi|119859	RecName: Full=Flagellar calcium-binding protein; Short=FCABP; AltName: Full=1F8 protein; AltName: F
gi|71667953	hypothetical protein [Trypanosoma cruzi strain CL Brener]
gi|71399455	surface protein TolT [Trypanosoma cruzi strain CL Brener]
gi|71401084	(H+)-ATPase G subunit [Trypanosoma cruzi strain CL Brener]
gi|71659663	hypothetical protein [Trypanosoma cruzi strain CL Brener]
gi|71664216	heat shock 70 kDa protein, mitochondrial precursor [Trypanosoma cruzi strain CL Brener]
gi|71651158	hypothetical protein [Trypanosoma cruzi strain CL Brener]
gi|71410853	10 kDa heat shock protein [Trypanosoma cruzi strain CL Brener]
gi|71402480	hypothetical protein [Trypanosoma cruzi strain CL Brener]
gi|71404821	trans-sialidase [Trypanosoma cruzi strain CL Brener]
gi|12083381	antigen 38 [Trypanosoma cruzi]
gi|71405064	60S acidic ribosomal protein P2 [Trypanosoma cruzi strain CL Brener]
gi|71423525	vesicle-associated membrane protein [Trypanosoma cruzi strain CL Brener]
gi|71413591	hypothetical protein [Trypanosoma cruzi strain CL Brener]
gi|71648964	hypothetical protein [Trypanosoma cruzi strain CL Brener]
gi|71659778	poly(A)-binding protein [Trypanosoma cruzi strain CL Brener]
gi|5726483	nucleoside diphosphate kinase [Trypanosoma brucei]
gi|463375	ubiquitin
gi|76781043	polyubiquitin [Massisteria marina]
gi|71656402	hypothetical protein [Trypanosoma cruzi strain CL Brener]
gi|71407515	heat shock 70 kDa protein, mitochondrial precursor [Trypanosoma cruzi strain CL Brener]
gi|55824400	heat shock protein 70 cytosolic isoform [Rhynchobodo ATCC50359]
gi|89329739	heat shock protein 70 [Capsaspora owczarzaki]

gi|71403916	elongation factor 1-alpha (EF-1-alpha) [Trypanosoma cruzi strain CL Brener]
gi|7327288	microtubule associated protein homolog [Trypanosoma cruzi]
gi|50660742	translation elongation factor 1 alpha [Phytophthora fragariae var. rubi]
gi|4139170	elongation translation factor 1 alpha [Cyanophora paradoxa]
gi|50660750	translation elongation factor 1 alpha [Phytophthora hibernalis]
gi|56156697	elongation factor 1A [Trichia persimilis]
gi|50660710	translation elongation factor 1 alpha [Phytophthora sojae]
gi|71401749	hypothetical protein [Trypanosoma cruzi strain CL Brener]
gi|71408516	hypothetical protein [Trypanosoma cruzi strain CL Brener]
gi|71405983	hypothetical protein [Trypanosoma cruzi strain CL Brener]
gi|1781355	histone H2A [Trypanosoma cruzi]
gi|13384081	histone H2A [Leishmania infantum]
gi|57903381	heat shock protein 70 [Spumella uniguttata]
gi|10119899	pyruvate phosphate dikinase 1 [Trypanosoma cruzi]
gi|71404564	protein kinase C substrate protein, heavy chain [Trypanosoma cruzi strain CL Brener]
gi|33694252	heat shock protein 70 [Rhynchopus sp. ATCC50230]
gi|123592	RecName: Full=Heat shock 70 kDa protein
gi|123603	RecName: Full=Heat shock 70 kDa protein
gi|55824402	heat shock protein 70 cytosolic isoform [Cryptobia salmositica]
gi|23306650	heat shock protein 70 [Carpediemonas membranifera]
gi|71652570	hypothetical protein [Trypanosoma cruzi strain CL Brener]
gi|71419111	nascent polypeptide associated complex subunit [Trypanosoma cruzi strain CL Brener]
gi|71665037	kinesin [Trypanosoma cruzi strain CL Brener]
gi|71422090	electron-transfer-flavoprotein, alpha polypeptide [Trypanosoma cruzi strain CL Brener]
gi|28779462	elongation factor-1 alpha [Streblomastix strix]
gi|71404826	hypothetical protein [Trypanosoma cruzi strain CL Brener]
gi|71403972	3,2-trans-enoyl-CoA isomerase [Trypanosoma cruzi strain CL Brener]
gi|71665810	hypothetical protein [Trypanosoma cruzi strain CL Brener]
gi|71399904	histone H4 [Trypanosoma cruzi strain CL Brener]
gi|167375825	hypothetical protein [Entamoeba dispar SAW760]
gi|71664019	hypothetical protein [Trypanosoma cruzi strain CL Brener]
gi|71655108	hypothetical protein [Trypanosoma cruzi strain CL Brener]
gi|71667211	hypothetical protein [Trypanosoma cruzi strain CL Brener]
gi|71660723	hypothetical protein [Trypanosoma cruzi strain CL Brener]
gi|71403618	hypothetical protein Tc00.1047053504411.10 [Trypanosoma cruzi strain CL Brener]
gi|58414949	polyubiquitin [Stauracon pallidus]
gi|33358312	ubiquitin-like protein Ublp94.4 [Acanthamoeba castellanii]
gi|154411942	hypothetical protein [Trichomonas vaginalis G3]
gi|9954108	RNA binding protein RGGm [Trypanosoma cruzi]
gi|71404616	hypothetical protein [Trypanosoma cruzi strain CL Brener]
gi|71660349	hypothetical protein [Trypanosoma cruzi strain CL Brener]
gi|71425751	centrin [Trypanosoma cruzi strain CL Brener]
gi|53829568	HSP70 [Ministeria vibrans]
gi|84105385	cytosolic heat shock protein 70 [Malawimonas jakobiformis]
gi|71664824	mucin TcMUCII [Trypanosoma cruzi strain CL Brener]

gi|71411561	hypothetical protein [Trypanosoma cruzi strain CL Brener]
gi|1322226	elongation factor 1 alpha
gi|71665461	enolase [Trypanosoma cruzi strain CL Brener]
gi|71412664	hypothetical protein [Trypanosoma cruzi strain CL Brener]
gi|71402512	eukaryotic translation initiation factor 2 subunit [Trypanosoma cruzi strain CL Brener]
gi|71649424	hypothetical protein [Trypanosoma cruzi strain CL Brener]
gi|71412308	hypothetical protein [Trypanosoma cruzi strain CL Brener]
gi|1170958	RecName: Full=Macrophage infectivity potentiator; AltName: Full=Peptidyl-prolyl cis-trans isomerase
gi|71398774	hypothetical protein [Trypanosoma cruzi strain CL Brener]
gi|71399257	hypothetical protein [Trypanosoma cruzi strain CL Brener]
gi|886414	TCJ2 [Trypanosoma cruzi]
gi|71666956	hypothetical protein [Trypanosoma cruzi strain CL Brener]
gi|71408304	ubiquitin-like protein [Trypanosoma cruzi strain CL Brener]
gi|167536654	hypothetical protein [Monosiga brevicollis MX1]
gi|154346020	cullin-like protein [Leishmania braziliensis MHOM/BR/75/M2904]
gi|120679	RecName: Full=Glyceraldehyde-3-phosphate dehydrogenase, glycosomal; Short=GAPDH
gi|71653479	cytochrome b5-like [Trypanosoma cruzi strain CL Brener]
gi|71659820	calmodulin [Trypanosoma cruzi strain CL Brener]
gi|133055	60S acidic ribosomal protein P1
gi|71418715	60S acidic ribosomal protein [Trypanosoma cruzi strain CL Brener]
gi|27734387	polyubiquitin [Euglypha rotunda]

Probability Based Mowse Score

Ions score is -10*Log(P), where P is the probability that the observed match is a random event.
Individual ions scores > 42 indicate identity or extensive homology (p<0.05).
Protein scores are derived from ions scores as a non-probabilistic basis for ranking protein hits.

Score Distribution

Peptide Summary Report

Format As | Peptide Summary | Help

Significance threshold p< 0.05 | Max. number of hits AUTO

Standard scoring ○ MudPIT scoring ◉ | Ions score cut-off 0 | Show sub-sets ☐

Show pop-ups ◉ Suppress pop-ups ○ | Sort unassigned Decreasing Score | Require bold red ☐

Select All | Select None | Search Selected | ☐ **Error tolerant** | Archive Report

1. gi|71655864 **Mass:** 23105 **Score:** 377 **Queries matched:** 9

I/6 autoantigen [Trypanosoma cruzi strain CL Brener]

☐ Check to include this hit in error tolerant search or archive report

	Query	Observed	Mr(expt)	Mr(calc)	Delta	Miss	Score	Expect	Rank	Peptide
☑	1515	623.26	1244.52	1244.60	-0.09	0	82	3.3e-06	1	M.PISPAEAFEER.H
☑	1545	636.22	1270.43	1270.53	-0.10	0	66	0.00012	1	R.LMEAMYGDEGR.V
☑	1554	639.76	1277.51	1277.61	-0.10	0	93	2.5e-07	1	K.LSSIEEATDSAR.Y
☑	1919	585.96	1754.85	1754.99	-0.14	0	(37)	0.087	1	K.VIPPSLALVAALESGYR.F
☑	1920	878.47	1754.92	1754.99	-0.07	0	88	7.6e-07	1	K.VIPPSLALVAALESGYR.F
☑	1924	882.38	1762.74	1762.93	-0.18	1	66	0.00012	1	K.KEEPTPPPPPPPPQQK.K
☑	1925	588.60	1762.77	1762.93	-0.15	1	(50)	0.005	1	K.KEEPTPPPPPPPPQQK.K
☑	2262	780.00	2336.98	2337.10	-0.12	0	(52)	0.0029	1	K.TGSSQDALSADEIDALFDVLDR.E
☑	2263	1169.50	2336.99	2337.10	-0.10	0	111	3.5e-09	1	K.TGSSQDALSADEIDALFDVLDR.E

---

2. gi|295363 **Mass:** 59349 **Score:** 374 **Queries matched:** 8

heat shock protein 60

☐ Check to include this hit in error tolerant search or archive report

	Query	Observed	Mr(expt)	Mr(calc)	Delta	Miss	Score	Expect	Rank	Peptide
☑	1264	557.77	1113.52	1113.64	-0.12	0	88	7.9e-07	1	R.AVSAVATTLGPK.G
☑	1444	602.26	1202.50	1202.58	-0.08	0	102	3.8e-08	1	K.VGGGSEVEVNEK.K
☑	1495	411.86	1232.56	1232.72	-0.16	0	25	1.8	1	R.LPAHTIVLNAGK.E
☑	1611	659.79	1317.56	1317.69	-0.13	0	87	1.1e-06	1	R.NVIIEQSYGAPK.I
☑	1629	670.24	1338.47	1338.61	-0.14	0	87	1e-06	1	R.GLIDGETSDYNR.E

Query	Observed	Mr(expt)	Mr(calc)	Delta	Miss	Score	Expect	Rank	Peptide
1818	798.27	1594.54	1594.78	-0.25	0	69	5.5e-05	1	R.YVNMFEAGIIDPAR.V
1830	806.33	1610.65	1610.78	-0.13	0	(61)	0.00037	1	R.YVNMFEAGIIDPAR.V + Oxidation (M)
1881	846.85	1691.68	1691.81	-0.13	0	92	3.6e-07	1	K.VLENNDVTVGYDAQR.D

Proteins matching the same set of peptides:

gi|3023478 **Mass:** 59374 **Score:** 374 **Queries matched:** 8
RecName: Full=Chaperonin HSP60, mitochondrial; Short=Protein Cpn60; AltName: Full=groEL protein; Al

gi|71665064 **Mass:** 59351 **Score:** 374 **Queries matched:** 8
chaperonin HSP60, mitochondrial precursor [Trypanosoma cruzi strain CL Brener]

gi|71665068 **Mass:** 59129 **Score:** 374 **Queries matched:** 8
chaperonin HSP60, mitochondrial precursor [Trypanosoma cruzi strain CL Brener]

---

3. gi|50659756 **Mass:** 71330 **Score:** 343 **Queries matched:** 14
heat shock protein 70 [Trypanosoma cruzi]

☐ Check to include this hit in error tolerant search or archive report

Query	Observed	Mr(expt)	Mr(calc)	Delta	Miss	Score	Expect	Rank	Peptide
1005	502.71	1003.40	1003.48	-0.08	0	50	0.0051	1	R.LSEEEIER.M
1453	605.68	1209.34	1209.48	-0.14	0	4	1.8e+02	3	R.EAAEFEDEDR.K
1505	619.79	1237.56	1237.63	-0.07	0	75	1.4e-05	1	R.NSLESVAYSLR.N
282	650.24	1298.46	1298.62	-0.16	0	43	0.079	1	K.FEELNMELFK.G
1679	468.53	1402.57	1402.73	-0.16	1	55	0.0019	1	K.LSADDKSAVEAAVK.E
1704	479.84	1436.51	1436.68	-0.18	0	(23)	2.3	1	K.GDVHIIPNDMGNR.I
1705	719.27	1436.53	1436.68	-0.16	0	37	0.086	1	K.GDVHIIPNDMGNR.I
1714	725.28	1448.56	1448.75	-0.20	0	68	8.4e-05	1	R.ITPSVVAFTETER.L
1715	725.34	1448.66	1448.75	-0.10	0	(67)	9.7e-05	1	R.ITPSVVAFTETER.L
1717	727.27	1452.52	1452.68	-0.16	0	(24)	2	1	K.GDVHIIPNDMGNR.I + Oxidation (M)
1746	742.37	1482.72	1482.84	-0.12	1	78	8.2e-06	1	R.DKLQSVTNPIIQK.V
2136	1054.37	2106.72	2106.88	-0.16	0	74	2.1e-05	1	K.VYQSGGGADGDERPEPMDDL.-
2137	703.25	2106.73	2106.88	-0.15	0	(29)	0.65	1	K.VYQSGGGADGDERPEPMDDL.-
2318	833.91	2498.70	2499.05	-0.35	1	82	4.6e-06	1	K.EAMQFLDDNPNADKEEYDEAR.D

Proteins matching the same set of peptides:

gi|71415505 **Mass:** 71272 **Score:** 343 **Queries matched:** 14

glucose-regulated protein 78 [Trypanosoma cruzi strain CL Brener]

---

4. gi|71425779 **Mass:** 23211 **Score:** 337 **Queries matched:** 9
I/6 autoantigen [Trypanosoma cruzi strain CL Brener]
☐ Check to include this hit in error tolerant search or archive report

	Query	Observed	Mr(expt)	Mr(calc)	Delta	Miss	Score	Expect	Rank	Peptide
	666	**427.16**	**852.31**	**852.45**	**-0.14**	**0**	**25**	**1.4**	**3**	**R.YPGFLTR.D**
	1515	623.26	1244.52	1244.60	-0.09	0	82	3.3e-06	1	M.PISPAEAFEER.H
	1545	636.22	1270.43	1270.53	-0.10	0	66	0.00012	1	R.LMEAMYGDEGR.V
	1554	639.76	1277.51	1277.61	-0.10	0	93	2.5e-07	1	K.LSSIEEATDSAR.Y
☑	1917	**585.26**	**1752.76**	**1752.90**	**-0.15**	**1**	**49**	**0.0062**	**1**	**K.KEEPTSPPPPPPPQQK.K**
	1919	585.96	1754.85	1754.99	-0.14	0	(37)	0.087	1	K.VIPPSLALVAALESGYR.F
	1920	878.47	1754.92	1754.99	-0.07	0	88	7.6e-07	1	K.VIPPSLALVAALESGYR.F
	2262	780.00	2336.98	2337.10	-0.12	0	(52)	0.0029	1	K.TGSSQDALSADEIDALFDVLDR.E
	2263	1169.50	2336.99	2337.10	-0.10	0	111	3.5e-09	1	K.TGSSQDALSADEIDALFDVLDR.E

---

5. gi|71400377 **Mass:** 85164 **Score:** 309 **Queries matched:** 13
microtubule-associated protein [Trypanosoma cruzi strain CL Brener]
☐ Check to include this hit in error tolerant search or archive report

	Query	Observed	Mr(expt)	Mr(calc)	Delta	Miss	Score	Expect	Rank	Peptide
☑	1716	**726.82**	**1451.62**	**1451.73**	**-0.11**	**0**	**79**	**6.1e-06**	**1**	**R.ALPLEEQEDVGPR.H**
☑	1718	**727.30**	**1452.59**	**1452.71**	**-0.12**	**0**	**77**	**9.7e-06**	**1**	**R.ALPLEEEEDVGPR.H**
☑	1724	**734.29**	**1466.57**	**1466.70**	**-0.13**	**0**	**67**	**0.00011**	**1**	**R.ALPQEEQEDVGPR.H**
☑	1725	**489.87**	**1466.57**	**1466.70**	**-0.13**	**0**	**(30)**	**0.52**	**1**	**R.ALPQEEQEDVGPR.H**
☑	1728	**734.79**	**1467.57**	**1467.68**	**-0.11**	**0**	**74**	**1.7e-05**	**1**	**R.ALPQEEEEDVGPR.H**
☑	1729	**734.80**	**1467.58**	**1467.68**	**-0.10**	**0**	**(73)**	**2.1e-05**	**1**	**R.ALPQEEEEDVGPR.H**
☑	40	**490.61**	**1468.79**	**1468.74**	**0.05**	**0**	**14**	**40**	**1**	**R.ALLLEEEEDVGPR.H**
☑	1825	**536.84**	**1607.50**	**1607.83**	**-0.33**	**1**	**19**	**6.9**	**1**	**K.RALPLEEQEDVGPR.H**
☑	1837	**812.33**	**1622.64**	**1622.80**	**-0.16**	**1**	**58**	**0.00065**	**1**	**K.RALPQEEQEDVGPR.H**
☑	1838	**542.23**	**1623.67**	**1623.79**	**-0.12**	**1**	**54**	**0.002**	**1**	**K.RALPQEEEEDVGPR.H**
☑	1943	**899.84**	**1797.66**	**1797.85**	**-0.20**	**0**	**34**	**0.2**	**1**	**R.STTQDAYRPVDPSAYK.R**
☑	1944	**600.23**	**1797.66**	**1797.85**	**-0.19**	**0**	**(27)**	**1**	**1**	**R.STTQDAYRPVDPSAYK.R**
☑	1945	**600.25**	**1797.74**	**1797.85**	**-0.11**	**0**	**(18)**	**7.6**	**1**	**R.STTQDAYRPVDPSAYK.R**

6. gi|3885846 **Mass:** 9102 **Score:** 231 **Queries matched:** 4
beta tubulin [Trypanosoma cruzi]
☐ Check to include this hit in error tolerant search or archive report

	Query	Observed	Mr(expt)	Mr(calc)	Delta	Miss	Score	Expect	Rank	Peptide
☑	1631	671.31	1340.60	1340.64	-0.03	0	74	2.1e-05	1	R.INVYFDEATGGR.Y
☑	1978	616.58	1846.73	1846.87	-0.14	0	(57)	0.00099	1	R.EIVCVQAGQCGNQIGSK.F + 2 Carbamidomethyl (C)
☑	1979	924.37	1846.73	1846.87	-0.14	0	119	6.2e-10	1	R.EIVCVQAGQCGNQIGSK.F + 2 Carbamidomethyl (C)
☑	2430	1031.76	3092.26	3092.40	-0.14	0	64	0.00016	1	K.FWEVISDEHGVDPTGTYQGDSDLQLER.I

Proteins matching the same set of peptides:

gi|91983178 **Mass:** 10597 **Score:** 231 **Queries matched:** 4
beta tubulin [Crithidia deanei]

gi|111115853 **Mass:** 49628 **Score:** 231 **Queries matched:** 4
beta tubulin [Trypanosoma evansi]

gi|115504281 **Mass:** 49672 **Score:** 231 **Queries matched:** 4
beta tubulin [Trypanosoma brucei]

gi|166203636 **Mass:** 24112 **Score:** 231 **Queries matched:** 4
beta tubulin [Trypanosoma evansi]

gi|169125731 **Mass:** 49655 **Score:** 231 **Queries matched:** 4
beta-tubulin [Trypanosoma evansi]

gi|1220547 **Mass:** 49410 **Score:** 231 **Queries matched:** 4
beta tubulin

gi|3915883 **Mass:** 49541 **Score:** 231 **Queries matched:** 4
Tubulin beta chain (Beta-tubulin)

gi|18568139 **Mass:** 49668 **Score:** 231 **Queries matched:** 4
beta tubulin 1.9 [Trypanosoma cruzi]

gi|71656281 **Mass:** 49668 **Score:** 231 **Queries matched:** 4
beta tubulin [Trypanosoma cruzi strain CL Brener]

gi|74229926 **Mass:** 49696 **Score:** 231 **Queries matched:** 4
beta-tubulin [Trypanosoma danilewskyi]

gi|91983201 **Mass:** 42807 **Score:** 231 **Queries matched:** 4
beta tubulin [Trypanosoma grayi]

7. gi|71651558 **Mass:** 42796 **Score:** 204 **Queries matched:** 9
hypothetical protein [Trypanosoma cruzi strain CL Brener]

☐ Check to include this hit in error tolerant search or archive report

	Query	Observed	Mr(expt)	Mr(calc)	Delta	Miss	Score	Expect	Rank	Peptide
☑	819	466.17	930.32	930.48	-0.16	0	45	0.019	1	K.VDLDNLSR.R
☑	1081	518.27	1034.53	1034.56	-0.03	0	37	0.1	1	R.FVLASIEEK.E
☑	1508	620.25	1238.49	1238.56	-0.07	0	68	7.9e-05	1	K.APEAAAEFDYR.E
☑	1583	649.25	1296.48	1296.60	-0.12	0	84	2e-06	1	K.VYASQEDMINK.N
☑	1664	461.87	1382.59	1382.77	-0.18	0	34	0.17	1	K.QDLQHVLIYVR.M
☑	1748	743.79	1485.57	1485.71	-0.14	0	52	0.003	1	R.SHVQQVWTSVCR.E + Carbamidomethyl (C)
☑	1791	776.25	1550.49	1550.73	-0.24	0	68	7.4e-05	1	R.QFGDVVLLEDECK.D + Carbamidomethyl (C)
☑	2271	785.64	2353.90	2354.07	-0.16	0	(24)	1.9	1	R.ELGEAAVQEFEEYSSASGAAPGR.R
☑	2272	1178.00	2353.99	2354.07	-0.08	0	57	0.00078	1	R.ELGEAAVQEFEEYSSASGAAPGR.R

Proteins matching the same set of peptides:
gi|71662736 **Mass:** 42788 **Score:** 204 **Queries matched:** 9
hypothetical protein [Trypanosoma cruzi strain CL Brener]

---

8. gi|162030 **Mass:** 16829 **Score:** 189 **Queries matched:** 7
calmodulin A

☐ Check to include this hit in error tolerant search or archive report

	Query	Observed	Mr(expt)	Mr(calc)	Delta	Miss	Score	Expect	Rank	Peptide
☑	1637	675.28	1348.55	1348.62	-0.07	0	74	2.1e-05	1	K.LTDEEVDEMIR.E
☑	1908	580.60	1738.76	1738.83	-0.06	1	22	3.1	1	R.VFDKDGNGFNSAAELR.H
	1909	870.39	1738.76	1738.83	-0.06	1	(8)	89	3	R.VFDKDGNGFNSAAELR.H
☑	1976	922.88	1843.74	1843.88	-0.15	1	52	0.0034	1	K.EAFSLFDKDGDGTITTK.E
☑	1977	615.59	1843.74	1843.88	-0.14	1	(47)	0.01	1	K.EAFSLFDKDGDGTITTK.E
☑	906	964.19	1926.37	1926.85	-0.47	0	78	2.5e-05	1	R.EADVDGDGQINYEEFVK.M
☑	277	647.95	1940.81	1940.88	-0.07	2	43	0.087	1	R.KMQDSDSEEEIKEAFR.V

---

9. gi|71413559 **Mass:** 44080 **Score:** 183 **Queries matched:** 7
hypothetical protein [Trypanosoma cruzi strain CL Brener]

☐ Check to include this hit in error tolerant search or archive report

	Query	Observed	Mr(expt)	Mr(calc)	Delta	Miss	Score	Expect	Rank	Peptide
☑	1285	563.24	1124.47	1124.63	-0.16	0	(19)	6.2	1	R.QFIPASLPPR.Q
☑	126	563.25	1124.49	1124.63	-0.14	0	38	0.21	1	R.QFIPASLPPR.Q
☑	1308	568.73	1135.44	1135.56	-0.12	0	55	0.0015	1	K.GVEDVEPVHR.K
☑	1418	594.27	1186.52	1186.63	-0.12	0	58	0.00082	1	K.ALTEEWILGR.K
☑	1701	717.82	1433.63	1433.71	-0.08	0	66	0.00012	1	R.FHEQTSVNLFGR.A
☑	1882	846.90	1691.78	1691.85	-0.07	0	90	5.7e-07	1	K.ANESLDVLNLGQYTR.E
☑	2018	637.26	1908.76	1908.97	-0.21	0	43	0.022	1	K.DLVEAPARPASANAAAGGSGK.K

---

10. gi|71407337 **Mass:** 16350 **Score:** 170 **Queries matched:** 7

hypothetical protein [Trypanosoma cruzi strain CL Brener]

☐ Check to include this hit in error tolerant search or archive report

	Query	Observed	Mr(expt)	Mr(calc)	Delta	Miss	Score	Expect	Rank	Peptide
☑	934	488.18	974.34	974.45	-0.12	0	39	0.082	1	K.NMYTGVYK.A
☑	68	519.21	1036.41	1036.54	-0.14	0	34	0.5	1	K.ITLSDFVDK.A
☑	1085	519.21	1036.41	1036.54	-0.13	0	(26)	1.2	1	K.ITLSDFVDK.A
☑	1209	545.73	1089.44	1089.54	-0.11	0	72	3.6e-05	1	R.NSGSLSGVVDR.R
☑	1380	583.29	1164.56	1164.64	-0.08	1	77	9.5e-06	1	R.KITLSDFVDK.A
☑	1762	750.33	1498.65	1498.73	-0.08	0	42	0.031	1	K.QFTSTDADLLFNK.V
☑	1965	917.88	1833.75	1833.84	-0.10	0	77	1e-05	1	K.SAEELIADISSCSPEAR.A + Carbamidomethyl (C)

---

11. gi|10673 **Mass:** 14649 **Score:** 169 **Queries matched:** 5

unnamed protein product [Trypanosoma cruzi]

☐ Check to include this hit in error tolerant search or archive report

	Query	Observed	Mr(expt)	Mr(calc)	Delta	Miss	Score	Expect	Rank	Peptide
	477	383.18	764.34	764.43	-0.08	0	23	2.3	4	-.MQIFVK.T
☑	1155	533.24	1064.47	1064.55	-0.08	0	69	6.1e-05	1	R.TLADYNIQK.E
☑	1164	534.28	1066.55	1066.61	-0.07	0	55	0.0016	1	K.ESTLHLVLR.L
☑	1779	508.55	1522.63	1522.77	-0.15	1	25	1.6	1	K.IQDKEGIPPDQQR.L

☑ 1914	874.39	1746.76	1746.89	-0.13	0	124	2.1e-10	1	K.TIALEVESSDTIENVK.A

Proteins matching the same set of peptides:

gi|10674 **Mass:** 8763 **Score:** 169 **Queries matched:** 5
unnamed protein product [Trypanosoma cruzi]

gi|136677 **Mass:** 8504 **Score:** 169 **Queries matched:** 5
RecName: Full=Ubiquitin

gi|162337 **Mass:** 40106 **Score:** 169 **Queries matched:** 5
ubiquitin precursor

---

12. gi|119859 **Mass:** 23721 **Score:** 162 **Queries matched:** 7
RecName: Full=Flagellar calcium-binding protein; Short=FCABP; AltName: Full=1F8 protein; AltName: F

☐ Check to include this hit in error tolerant search or archive report

Query	Observed	Mr(expt)	Mr(calc)	Delta	Miss	Score	Expect	Rank	Peptide
☑ 707	439.17	876.33	876.43	-0.11	0	50	0.0061	1	K.LDEFTPR.V
☑ 980	495.22	988.43	988.52	-0.09	0	65	0.00015	1	K.VEDPAALFK.E
☑ 1686	707.72	1413.42	1413.59	-0.17	0	52	0.0037	1	K.LDADGDPDNVPESA.-
1687	707.79	1413.57	1413.59	-0.02	0	(13)	23	2	K.LDADGDPDNVPESA.-
☑ 1736	737.82	1473.62	1473.68	-0.06	0	69	5.8e-05	1	K.GSEDFVEFLEFR.L
☑ 1957	911.35	1820.68	1820.84	-0.16	0	55	0.0017	1	K.LCYDEVHSGCLEVLK.L + 2 Carbamidomethyl (C)
☑ 1958	607.91	1820.70	1820.84	-0.15	0	(38)	0.078	1	K.LCYDEVHSGCLEVLK.L + 2 Carbamidomethyl (C)

Proteins matching the same set of peptides:

gi|1552212 **Mass:** 23727 **Score:** 162 **Queries matched:** 7
calcium-binding protein [Trypanosoma cruzi]

gi|71406002 **Mass:** 23827 **Score:** 162 **Queries matched:** 7
flagellar calcium-binding protein [Trypanosoma cruzi strain CL Brener]

gi|71406004 **Mass:** 23799 **Score:** 162 **Queries matched:** 7
flagellar calcium-binding protein [Trypanosoma cruzi strain CL Brener]

gi|71412217 **Mass:** 23707 **Score:** 162 **Queries matched:** 7
calcium-binding protein [Trypanosoma cruzi strain CL Brener]

gi|71656918 **Mass:** 23713 **Score:** 162 **Queries matched:** 7
flagellar calcium-binding protein [Trypanosoma cruzi strain CL Brener]

---

13. gi|71667953 **Mass:** 45869 **Score:** 153 **Queries matched:** 8
hypothetical protein [Trypanosoma cruzi strain CL Brener]
☐ Check to include this hit in error tolerant search or archive report

	Query	Observed	Mr(expt)	Mr(calc)	Delta	Miss	Score	Expect	Rank	Peptide
☑	782	456.67	911.32	911.41	-0.10	0	35	0.12	1	R.FEEFANR.A
☑	1139	529.77	1057.53	1057.60	-0.08	0	70	5.3e-05	1	K.LQVELLDTK.H
☑	1446	602.78	1203.55	1203.63	-0.09	0	39	0.061	1	R.IQSILSETGEK.E
☑	1509	620.29	1238.57	1238.54	0.03	0	46	0.01	1	K.ETQEYVDEAR.L
☑	1591	650.81	1299.61	1299.70	-0.09	0	87	1.4e-06	1	R.LEELQAELLSR.Q
☑	1665	462.16	1383.45	1383.64	-0.19	0	40	0.044	1	R.HHSDDEVAAYIK.E
☑	1666	692.75	1383.48	1383.64	-0.17	0	(30)	0.47	1	R.HHSDDEVAAYIK.E
☑	1696	714.33	1426.64	1426.73	-0.09	0	52	0.0032	1	K.ELPELQVLSEDR.A

---

14. gi|71399455 **Mass:** 24752 **Score:** 139 **Queries matched:** 2
surface protein TolT [Trypanosoma cruzi strain CL Brener]
☐ Check to include this hit in error tolerant search or archive report

	Query	Observed	Mr(expt)	Mr(calc)	Delta	Miss	Score	Expect	Rank	Peptide
☑	1421	594.80	1187.58	1187.64	-0.06	0	68	9e-05	1	R.AAEQTVLSLEK.A
☑	1763	750.88	1499.74	1499.83	-0.09	0	110	4.3e-09	1	R.TLAQDVAATASALLR.Q

Proteins matching the same set of peptides:
gi|71402301 **Mass:** 32319 **Score:** 139 **Queries matched:** 2
surface protein TolT [Trypanosoma cruzi strain CL Brener]

---

15. gi|71401084 **Mass:** 12749 **Score:** 136 **Queries matched:** 2
(H+)-ATPase G subunit [Trypanosoma cruzi strain CL Brener]
☐ Check to include this hit in error tolerant search or archive report

	Query	Observed	Mr(expt)	Mr(calc)	Delta	Miss	Score	Expect	Rank	Peptide
☑	1414	593.81	1185.60	1185.64	-0.03	0	67	9.7e-05	1	R.GTELQLQELR.T
☑	1875	843.38	1684.74	1684.84	-0.10	0	108	7.6e-09	1	R.AQQLSGADAENLELAR.G

Proteins matching the same set of peptides:
gi|71424114 **Mass:** 12763 **Score:** 136 **Queries matched:** 2
(H+)-ATPase G subunit [Trypanosoma cruzi strain CL Brener]

---

16. gi|71659663 **Mass:** 31532 **Score:** 127 **Queries matched:** 2
hypothetical protein [Trypanosoma cruzi strain CL Brener]
☐ Check to include this hit in error tolerant search or archive report

	Query	Observed	Mr(expt)	Mr(calc)	Delta	Miss	Score	Expect	Rank	Peptide
☑	588	**818.29**	**1634.56**	**1634.78**	**-0.22**	**0**	**124**	**6.3e-10**	**1**	**R.MVSENESAINDVVTK.L**
☑	2352	**880.04**	**2637.09**	**2637.29**	**-0.20**	**0**	**33**	**0.21**	**1**	**K.VPNDFVPSCAQVDHSLVQQELQK.R + Carbamidomethyl (C)**

---

17. gi|71664216 **Mass:** 18525 **Score:** 126 **Queries matched:** 2
heat shock 70 kDa protein, mitochondrial precursor [Trypanosoma cruzi strain CL Brener]
☐ Check to include this hit in error tolerant search or archive report

	Query	Observed	Mr(expt)	Mr(calc)	Delta	Miss	Score	Expect	Rank	Peptide
☑	2395	**963.67**	**2887.98**	**2888.23**	**-0.25**	**0**	**67**	**0.00012**	**1**	**R.TEYQQAAAANSSSSSGNTDSSQGEQQQK.G**
☑	2481	**1149.13**	**3444.38**	**3444.49**	**-0.11**	**1**	**79**	**4.9e-06**	**1**	**R.TEYQQAAAANSSSSSGNTDSSQGEQQQKGDQQK.Q**

---

18. gi|71651158 **Mass:** 16325 **Score:** 123 **Queries matched:** 7
hypothetical protein [Trypanosoma cruzi strain CL Brener]
☐ Check to include this hit in error tolerant search or archive report

	Query	Observed	Mr(expt)	Mr(calc)	Delta	Miss	Score	Expect	Rank	Peptide
	934	488.18	974.34	974.45	-0.12	0	39	0.082	1	K.NMYTGVYK.A
	68	519.21	1036.41	1036.54	-0.14	0	34	0.5	1	K.ITLSDFVDK.A
	1085	519.21	1036.41	1036.54	-0.13	0	(26)	1.2	1	K.ITLSDFVDK.A
	1209	545.73	1089.44	1089.54	-0.11	0	72	3.6e-05	1	R.NSGSLSGVVDR.R
	1380	583.29	1164.56	1164.64	-0.08	1	77	9.5e-06	1	R.KITLSDFVDK.A
	1762	750.33	1498.65	1498.73	-0.08	0	42	0.031	1	K.QFTSTDADLLFNK.V
☑	1987	**932.24**	**1862.46**	**1861.87**	**0.58**	**0**	**16**	**27**	**1**	**K.SVEELIADISSCSPEAR.A + Carbamidomethyl (C)**

---

19. gi|71410853 **Mass:** 10694 **Score:** 110 **Queries matched:** 5
10 kDa heat shock protein [Trypanosoma cruzi strain CL Brener]
☐ Check to include this hit in error tolerant search or archive report

	Query	Observed	Mr(expt)	Mr(calc)	Delta	Miss	Score	Expect	Rank	Peptide
	646	423.68	845.34	845.43	-0.09	0	13	36	2	K.DWTPTVK.V
	1406	591.31	1180.61	1180.68	-0.08	0	28	0.61	2	K.AGVLIPEQVAGK.V
☑	1620	443.88	1328.61	1328.73	-0.12	0	(52)	0.0031	1	K.VNEGTVVAVAAATK.D
☑	1621	665.32	1328.62	1328.73	-0.11	0	94	2.1e-07	1	K.VNEGTVVAVAAATK.D
☑	1860	831.87	1661.72	1661.85	-0.13	0	28	0.82	1	K.VDDTVLLPEFGGSSVK.V

Proteins matching the same set of peptides:
gi|71410857 **Mass:** 20085 **Score:** 110 **Queries matched:** 5
10 kDa heat shock protein [Trypanosoma cruzi strain CL Brener]

---

20. gi|71402480 **Mass:** 37891 **Score:** 106 **Queries matched:** 3
hypothetical protein [Trypanosoma cruzi strain CL Brener]
☐ Check to include this hit in error tolerant search or archive report

	Query	Observed	Mr(expt)	Mr(calc)	Delta	Miss	Score	Expect	Rank	Peptide
☑	1287	563.78	1125.55	1125.64	-0.09	0	38	0.081	1	R.SLIPDDVVIR.D
☑	1520	626.79	1251.56	1251.68	-0.12	0	105	1.4e-08	1	R.HAELVQLSDLK.E
	1573	646.32	1290.63	1290.69	-0.06	1	8	86	9	K.EVLRTFIEER.R

Proteins matching the same set of peptides:
gi|71418019 **Mass:** 37850 **Score:** 106 **Queries matched:** 3
hypothetical protein [Trypanosoma cruzi strain CL Brener]

---

21. gi|71404821 **Mass:** 88843 **Score:** 103 **Queries matched:** 1
trans-sialidase [Trypanosoma cruzi strain CL Brener]
☐ Check to include this hit in error tolerant search or archive report

	Query	Observed	Mr(expt)	Mr(calc)	Delta	Miss	Score	Expect	Rank	Peptide
☑	2045	984.85	1967.68	1967.86	-0.18	0	103	2.4e-08	1	K.ATGSSAGEDSESSGAAGTDLAK.G

Proteins matching the same set of peptides:
gi|71393748 **Mass:** 37829 **Score:** 103 **Queries matched:** 1
trans-sialidase [Trypanosoma cruzi strain CL Brener]

---

22. gi|12083381 **Mass:** 64218 **Score:** 102 **Queries matched:** 1
antigen 38 [Trypanosoma cruzi]
☐ Check to include this hit in error tolerant search or archive report

Query	Observed	Mr(expt)	Mr(calc)	Delta	Miss	Score	Expect	Rank	Peptide
☑ 2369	905.73	2714.17	2714.05	0.12	1	102	2.2e-08	1	R.YRDEEEMDDEGGLEEAQEGNDER.E

Proteins matching the same set of peptides:
gi|71410919 **Mass:** 63977 **Score:** 102 **Queries matched:** 1
lectin [Trypanosoma cruzi strain CL Brener]

---

23. gi|71405064 **Mass:** 10861 **Score:** 99 **Queries matched:** 2
60S acidic ribosomal protein P2 [Trypanosoma cruzi strain CL Brener]
☐ Check to include this hit in error tolerant search or archive report

Query	Observed	Mr(expt)	Mr(calc)	Delta	Miss	Score	Expect	Rank	Peptide
☑ 1269	558.74	1115.47	1115.62	-0.15	0	87	1.1e-06	1	R.SVATLVAEGAAK.M
☑ 2365	902.41	2704.21	2704.36	-0.15	0	43	0.018	1	K.MSAVAVSAAPAAGGAAAPAAAAGGAAAPAAADAK.K

---

24. gi|71423525 **Mass:** 24104 **Score:** 98 **Queries matched:** 1
vesicle-associated membrane protein [Trypanosoma cruzi strain CL Brener]
☐ Check to include this hit in error tolerant search or archive report

Query	Observed	Mr(expt)	Mr(calc)	Delta	Miss	Score	Expect	Rank	Peptide
☑ 2290	1197.55	2393.09	2393.09	-0.00	0	98	6.2e-08	1	K.ITALNDDINQVVDVMMDNMDK.V

Proteins matching the same set of peptides:
gi|72389462 **Mass:** 24386 **Score:** 98 **Queries matched:** 1

vesicle-associated membrane protein [Trypanosoma brucei TREU927]

---

25. gi|71413591 **Mass:** 16740 **Score:** 94 **Queries matched:** 2
hypothetical protein [Trypanosoma cruzi strain CL Brener]

☐ Check to include this hit in error tolerant search or archive report

	Query	Observed	Mr(expt)	Mr(calc)	Delta	Miss	Score	Expect	Rank	Peptide
☑	1745	741.80	1481.60	1481.75	-0.15	0	94	2e-07	1	K.TETVSSTETVVTTK.N
	98	544.22	1629.64	1629.79	-0.14	0	13	67	2	K.NVPTAANVPHDAGPDR.L

---

26. gi|71648964 **Mass:** 52877 **Score:** 92 **Queries matched:** 2
hypothetical protein [Trypanosoma cruzi strain CL Brener]

☐ Check to include this hit in error tolerant search or archive report

	Query	Observed	Mr(expt)	Mr(calc)	Delta	Miss	Score	Expect	Rank	Peptide
☑	1842	815.33	1628.64	1628.78	-0.14	0	92	3.2e-07	1	R.QLQEQSENLQQER.A
☑	1843	543.89	1628.64	1628.78	-0.13	0	(16)	13	1	R.QLQEQSENLQQER.A

Proteins matching the same set of peptides:
gi|71659037 **Mass:** 52427 **Score:** 92 **Queries matched:** 2
hypothetical protein [Trypanosoma cruzi strain CL Brener]

---

27. gi|71659778 **Mass:** 61371 **Score:** 91 **Queries matched:** 3
poly(A)-binding protein [Trypanosoma cruzi strain CL Brener]

☐ Check to include this hit in error tolerant search or archive report

	Query	Observed	Mr(expt)	Mr(calc)	Delta	Miss	Score	Expect	Rank	Peptide
☑	1389	585.79	1169.57	1169.64	-0.07	0	75	1.2e-05	1	K.VQEALEVLNR.H
☑	1799	522.60	1564.78	1564.87	-0.09	0	(32)	0.36	1	R.LYNHIVAINPAAAAK.V
☑	1800	783.40	1564.79	1564.87	-0.08	0	52	0.003	1	R.LYNHIVAINPAAAAK.V

Proteins matching the same set of peptides:
gi|159162751 **Mass:** 9085 **Score:** 91 **Queries matched:** 3

Chain A, Solution Structure Of C-Terminal Domain From Trypanosoma Cruzi Poly(A)-Binding Protein

---

28. gi|5726483 **Mass:** 16775 **Score:** 87 **Queries matched:** 1
nucleoside diphosphate kinase [Trypanosoma brucei]
☐ Check to include this hit in error tolerant search or archive report

	Query	Observed	Mr(expt)	Mr(calc)	Delta	Miss	Score	Expect	Rank	Peptide
☑	1884	**847.91**	**1693.81**	**1693.94**	**-0.13**	**0**	**87**	**7.9e-07**	**1**	**R.VLLGATNPADSLPGTIR.G**

Proteins matching the same set of peptides:
gi|71667532 **Mass:** 16868 **Score:** 87 **Queries matched:** 1
nucleoside diphosphate kinase [Trypanosoma cruzi strain CL Brener]
gi|74026208 **Mass:** 16847 **Score:** 87 **Queries matched:** 1
nucleoside diphosphate kinase [Trypanosoma brucei TREU927]

---

29. gi|463375 **Mass:** 8572 **Score:** 87 **Queries matched:** 4
ubiquitin
☐ Check to include this hit in error tolerant search or archive report

	Query	Observed	Mr(expt)	Mr(calc)	Delta	Miss	Score	Expect	Rank	Peptide
	477	383.18	764.34	764.43	-0.08	0	23	2.3	4	-.MQIFVK.T
	1164	534.28	1066.55	1066.61	-0.07	0	55	0.0016	1	K.ESTLHLVLR.L
	1779	508.55	1522.63	1522.77	-0.15	1	25	1.6	1	K.IQDKEGIPPDQQR.L
☑	1939	**894.35**	**1786.69**	**1786.92**	**-0.23**	**0**	**66**	**0.00014**	**1**	**R.TITLEVEPSDTIENVK.A**

Proteins matching the same set of peptides:
gi|7799051 **Mass:** 17236 **Score:** 87 **Queries matched:** 4
ubiquitin [Cyanidium caldarium]

---

30. gi|76781043 **Mass:** 7341 **Score:** 85 **Queries matched:** 3
polyubiquitin [Massisteria marina]
☐ Check to include this hit in error tolerant search or archive report

Query	Observed	Mr(expt)	Mr(calc)	Delta	Miss	Score	Expect	Rank	Peptide
1155	533.24	1064.47	1064.55	-0.08	0	69	6.1e-05	1	R.TLADYNIQK.E

	1164	534.28	1066.55	1066.61	-0.07	0	55	0.0016	1	K.ESTLHLVLR.L
☑	1915	**583.96**	**1748.85**	**1748.87**	**-0.02**	**0**	**12**	**33**	**1**	**K.TITLDVDSSDTIENVK.Q**

---

31. gi|71656402 **Mass:** 143742 **Score:** 83 **Queries matched:** 1
hypothetical protein [Trypanosoma cruzi strain CL Brener]
☐ Check to include this hit in error tolerant search or archive report

	Query	Observed	Mr(expt)	Mr(calc)	Delta	Miss	Score	Expect	Rank	Peptide
☑	1821	**801.33**	**1600.65**	**1600.77**	**-0.12**	**0**	**83**	**2.3e-06**	**1**	**R.GLQEVSEQAEDLQR.Q**

Proteins matching the same set of peptides:
gi|71409302 **Mass:** 132032 **Score:** 83 **Queries matched:** 1
hypothetical protein [Trypanosoma cruzi strain CL Brener]

---

32. gi|71407515 **Score:** 79 **Queries matched:** 2
heat shock 70 kDa protein, mitochondrial precursor [Trypanosoma cruzi strain CL Brener]
☐ Check to include this hit in error tolerant search or archive report

Query	Observed	Mr(expt)	Mr(calc)	Delta	Miss	Score	Expect	Rank	Peptide
1535	**630.80**	**1259.59**	**1259.65**	**-0.06**	**1**	**16**	**16**	**2**	**K.QCIKDAAVDLK.E + Carbamidomethyl (C)**
2481	1149.13	3444.38	3444.45	-0.07	0	79	4.9e-06	1	R.TEYQQAAAANSSSSSGNTDSSQGEQQQQGDQQK.Q

---

33. gi|55824400 **Mass:** 66906 **Score:** 78 **Queries matched:** 3
heat shock protein 70 cytosolic isoform [Rhynchobodo ATCC50359]
☐ Check to include this hit in error tolerant search or archive report

	Query	Observed	Mr(expt)	Mr(calc)	Delta	Miss	Score	Expect	Rank	Peptide
☑	1488	**614.76**	**1227.51**	**1227.62**	**-0.11**	**0**	**66**	**0.00014**	**1**	**R.VEIIANDQGNR.T**
	1744	**741.35**	**1480.69**	**1480.81**	**-0.13**	**1**	**18**	**10**	**4**	**K.RSVHDVVLVGGSTR.I**
☑	1751	**744.30**	**1486.59**	**1486.69**	**-0.10**	**0**	**52**	**0.0029**	**1**	**R.TTPSYVAFTDTER.L**

---

34. gi|89329739 **Mass:** 53354 **Score:** 78 **Queries matched:** 3
heat shock protein 70 [Capsaspora owczarzaki]

☐ Check to include this hit in error tolerant search or archive report

Query	Observed	Mr(expt)	Mr(calc)	Delta	Miss	Score	Expect	Rank	Peptide
1488	614.76	1227.51	1227.62	-0.11	0	66	0.00014	1	K.VEIIANDQGNR.T
282	650.24	1298.46	1298.60	-0.14	0	16	40	4	R.FEELCADLFR.G + Carbamidomethyl (C)
1751	744.30	1486.59	1486.69	-0.10	0	52	0.0029	1	R.TTPSYVAFTDTER.L

---

35. gi|71403916 **Mass:** 30716 **Score:** 76 **Queries matched:** 4
elongation factor 1-alpha (EF-1-alpha) [Trypanosoma cruzi strain CL Brener]

☐ Check to include this hit in error tolerant search or archive report

	Query	Observed	Mr(expt)	Mr(calc)	Delta	Miss	Score	Expect	Rank	Peptide
☑	935	**488.28**	**974.54**	**974.54**	**-0.00**	**0**	**52**	**0.0048**	**1**	**R.LPLQDVYK.I**
☑	1056	**513.25**	**1024.50**	**1024.60**	**-0.11**	**0**	**61**	**0.00037**	**1**	**K.IGGIGTVPVGR.V**
☑	2316	**831.02**	**2490.04**	**2490.27**	**-0.22**	**0**	**32**	**0.25**	**1**	**R.VETGTMKPGDVVTFAPANVTTEVK.S**
☑	2322	**836.35**	**2506.02**	**2506.26**	**-0.25**	**0**	**(27)**	**0.83**	**1**	**R.VETGTMKPGDVVTFAPANVTTEVK.S + Oxidation (M)**

Proteins matching the same set of peptides:

gi|704459 **Mass:** 43546 **Score:** 76 **Queries matched:** 4
elongation factor 1 alpha [Trypanosoma cruzi]

gi|1929445 **Mass:** 49018 **Score:** 76 **Queries matched:** 4
elongation factor 1-alpha [Trypanosoma cruzi]

gi|52424046 **Mass:** 49067 **Score:** 76 **Queries matched:** 4
elongation factor alpha G5 [Trypanosoma cruzi]

gi|61207234 **Mass:** 47727 **Score:** 76 **Queries matched:** 4
elongation factor 1-alpha [Trypanosoma cruzi]

gi|61207240 **Mass:** 47728 **Score:** 76 **Queries matched:** 4
elongation factor 1-alpha [Trypanosoma cruzi]

gi|61207250 **Mass:** 47741 **Score:** 76 **Queries matched:** 4
elongation factor 1-alpha [Trypanosoma cruzi]

gi|61207254 **Mass:** 47727 **Score:** 76 **Queries matched:** 4
elongation factor 1-alpha [Trypanosoma cruzi]

gi|61207256 **Mass:** 47843 **Score:** 76 **Queries matched:** 4
elongation factor 1-alpha [Trypanosoma cruzi]

gi|61207270 **Mass:** 48276 **Score:** 76 **Queries matched:** 4
elongation factor 1-alpha [Trypanosoma cruzi]
gi|61207272 **Mass:** 47767 **Score:** 76 **Queries matched:** 4
elongation factor 1-alpha [Trypanosoma cruzi]
gi|61207276 **Mass:** 47784 **Score:** 76 **Queries matched:** 4
elongation factor 1-alpha [Trypanosoma cruzi]
gi|61207288 **Mass:** 47711 **Score:** 76 **Queries matched:** 4
elongation factor 1-alpha [Trypanosoma cruzi]
gi|61207290 **Mass:** 47769 **Score:** 76 **Queries matched:** 4
elongation factor 1-alpha [Trypanosoma cruzi]
gi|61207296 **Mass:** 47667 **Score:** 76 **Queries matched:** 4
elongation factor 1-alpha [Trypanosoma cruzi]
gi|61207306 **Mass:** 47815 **Score:** 76 **Queries matched:** 4
elongation factor 1-alpha [Trypanosoma cruzi]
gi|61207316 **Mass:** 47797 **Score:** 76 **Queries matched:** 4
elongation factor 1-alpha [Trypanosoma cruzi]
gi|71403912 **Mass:** 48316 **Score:** 76 **Queries matched:** 4
elongation factor 1-alpha (EF-1-alpha) [Trypanosoma cruzi strain CL Brener]
gi|71403914 **Mass:** 49125 **Score:** 76 **Queries matched:** 4
elongation factor 1-alpha (EF-1-alpha) [Trypanosoma cruzi strain CL Brener]
gi|71408910 **Mass:** 49083 **Score:** 76 **Queries matched:** 4
elongation factor 1-alpha (EF-1-alpha) [Trypanosoma cruzi strain CL Brener]
gi|71408922 **Mass:** 42751 **Score:** 76 **Queries matched:** 4
elongation factor 1-alpha (EF-1-alpha) [Trypanosoma cruzi strain CL Brener]
gi|71664927 **Mass:** 49097 **Score:** 76 **Queries matched:** 4
elongation factor 1-alpha (EF-1-alpha) [Trypanosoma cruzi strain CL Brener]

---

36. gi|7327288 **Mass:** 4425 **Score:** 75 **Queries matched:** 4
microtubule associated protein homolog [Trypanosoma cruzi]
☐ Check to include this hit in error tolerant search or archive report

	Query	Observed	Mr(expt)	Mr(calc)	Delta	Miss	Score	Expect	Rank	Peptide
☑	1557	**642.75**	**1283.50**	**1283.56**	**-0.07**	**0**	**65**	**0.00014**	**1**	**M.PQEEEEDVGPR.H**
	1943	899.84	1797.66	1797.85	-0.20	0	34	0.2	1	R.STTQDAYRPVDPSAYK.R
	1944	600.23	1797.66	1797.85	-0.19	0	(27)	1	1	R.STTQDAYRPVDPSAYK.R
	1945	600.25	1797.74	1797.85	-0.11	0	(18)	7.6	1	R.STTQDAYRPVDPSAYK.R

37. gi|50660742 **Mass:** 33848 **Score:** 74 **Queries matched:** 3
translation elongation factor 1 alpha [Phytophthora fragariae var. rubi]
☐ Check to include this hit in error tolerant search or archive report

	Query	Observed	Mr(expt)	Mr(calc)	Delta	Miss	Score	Expect	Rank	Peptide
	935	488.28	974.54	974.54	-0.00	0	52	0.0048	1	R.LPLQDVYK.I
	1056	513.25	1024.50	1024.60	-0.11	0	61	0.00037	1	K.IGGIGTVPVGR.V
☑	632	**842.05**	**2523.13**	**2523.21**	**-0.08**	**0**	**21**	**11**	**1**	**K.SVEMHHESLLEAVPGDNVGFNVK.N + Oxidation (M)**

38. gi|4139170 **Mass:** 49079 **Score:** 74 **Queries matched:** 3
elongation translation factor 1 alpha [Cyanophora paradoxa]
☐ Check to include this hit in error tolerant search or archive report

Query	Observed	Mr(expt)	Mr(calc)	Delta	Miss	Score	Expect	Rank	Peptide
935	488.28	974.54	974.54	-0.00	0	52	0.0048	1	R.LPLQDVYK.I
1056	513.25	1024.50	1024.60	-0.11	0	61	0.00037	1	K.IGGIGTVPVGR.V
632	842.05	2523.13	2523.15	-0.02	0	19	20	2	K.SVEMHHESMPEALPGDNVGFNVK.N

39. gi|50660750 **Mass:** 24233 **Score:** 74 **Queries matched:** 3
translation elongation factor 1 alpha [Phytophthora hibernalis]
☐ Check to include this hit in error tolerant search or archive report

	Query	Observed	Mr(expt)	Mr(calc)	Delta	Miss	Score	Expect	Rank	Peptide
	935	488.28	974.54	974.54	-0.00	0	52	0.0048	1	R.LPLQDVYK.I
	1056	513.25	1024.50	1024.60	-0.11	0	61	0.00037	1	K.IGGIGTVPVGR.V
☑	2238	**765.92**	**2294.75**	**2295.02**	**-0.27**	**0**	**15**	**24**	**1**	**K.SVEMHHESVPEAKPGDNVGFN.- + Oxidation (M)**

40. gi|56156697 **Mass:** 44176 **Score:** 74 **Queries matched:** 4
elongation factor 1A [Trichia persimilis]
☐ Check to include this hit in error tolerant search or archive report

Query	Observed	Mr(expt)	Mr(calc)	Delta	Miss	Score	Expect	Rank	Peptide

Query	Observed	Mr(expt)	Mr(calc)	Delta	Miss	Score	Expect	Rank	Peptide
935	488.28	974.54	974.54	-0.00	0	52	0.0048	1	R.IPLQDVYK.I
1056	513.25	1024.50	1024.60	-0.11	0	61	0.00037	1	K.IGGIGTVPVGR.V
1944	600.23	1797.66	1797.93	-0.27	2	3	2.3e+02	5	-.HTTGHLIYKCGGIDKR.T
2316	831.02	2490.04	2490.30	-0.26	0	6	91	5	R.VETGVLKPGMNVTFSPAGLTTEVK.S + Oxidation (M)

---

41. gi|50660710 **Mass:** 33292 **Score:** 74 **Queries matched:** 3
translation elongation factor 1 alpha [Phytophthora sojae]
☐ Check to include this hit in error tolerant search or archive report

Query	Observed	Mr(expt)	Mr(calc)	Delta	Miss	Score	Expect	Rank	Peptide
935	488.28	974.54	974.54	-0.00	0	52	0.0048	1	R.LPLQDVYK.I
1056	513.25	1024.50	1024.60	-0.11	0	61	0.00037	1	K.IGGIGTVPVGR.V
2470	**1116.38**	**3346.12**	**3345.59**	**0.53**	**0**	**2**	**3.5e+02**	**4**	**R.DFICNMITGTSQADCAILVVASGVGEFEAGISK.E**

---

42. gi|71401749 **Mass:** 26271 **Score:** 70 **Queries matched:** 2
hypothetical protein [Trypanosoma cruzi strain CL Brener]
☐ Check to include this hit in error tolerant search or archive report

	Query	Observed	Mr(expt)	Mr(calc)	Delta	Miss	Score	Expect	Rank	Peptide
☑	2163	**717.91**	**2150.71**	**2150.91**	**-0.20**	**0**	**71**	**5.6e-05**	**1**	**R.LADHCLASAGNQNTCSSMSK.L + 2 Carbamidomethyl (C)**
☑	2442	**1057.16**	**3168.46**	**3168.51**	**-0.04**	**0**	**21**	**3**	**1**	**R.GLDVSAEPQSEVCSALAYVTESFFLQHGK.R + Carbamidomethyl (C)**

Proteins matching the same set of peptides:
gi|71415005 **Mass:** 26250 **Score:** 70 **Queries matched:** 2
hypothetical protein [Trypanosoma cruzi strain CL Brener]

---

43. gi|71408516 **Mass:** 8594 **Score:** 69 **Queries matched:** 1
hypothetical protein [Trypanosoma cruzi strain CL Brener]
☐ Check to include this hit in error tolerant search or archive report

	Query	Observed	Mr(expt)	Mr(calc)	Delta	Miss	Score	Expect	Rank	Peptide
☑	2215	**746.65**	**2236.93**	**2237.10**	**-0.16**	**0**	**69**	**5.8e-05**	**1**	**R.YSSPGYVAQKPDGAGGIETVNK.V**

---

44. gi|71405983 **Mass:** 37298 **Score:** 69 **Queries matched:** 1
hypothetical protein [Trypanosoma cruzi strain CL Brener]
☐ Check to include this hit in error tolerant search or archive report

	Query	Observed	Mr(expt)	Mr(calc)	Delta	Miss	Score	Expect	Rank	Peptide
☑	1850	**824.30**	**1646.58**	**1646.73**	**-0.15**	**0**	**69**	**7e-05**	**1**	**R.NMIDEDTVEEIDPK.E**

Proteins matching the same set of peptides:
gi|71408335 **Mass:** 37305 **Score:** 69 **Queries matched:** 1
hypothetical protein [Trypanosoma cruzi strain CL Brener]

---

45. gi|1781355 **Score:** 69 **Queries matched:** 1
histone H2A [Trypanosoma cruzi]
☐ Check to include this hit in error tolerant search or archive report

	Query	Observed	Mr(expt)	Mr(calc)	Delta	Miss	Score	Expect	Rank	Peptide
☑	1355	**578.77**	**1155.53**	**1155.56**	**-0.03**	**0**	**69**	**6.3e-05**	**1**	**R.HDDDLGMLLK.D**

Proteins matching the same set of peptides:
gi|2222802 **Score:** 69 **Queries matched:** 1
gi|18266856 **Score:** 69 **Queries matched:** 1
gi|71409700 **Score:** 69 **Queries matched:** 1
gi|71409702 **Score:** 69 **Queries matched:** 1
gi|71649895 **Score:** 69 **Queries matched:** 1
gi|71664770 **Score:** 69 **Queries matched:** 1
gi|71664802 **Score:** 69 **Queries matched:** 1

---

46. gi|13384081 **Mass:** 13942 **Score:** 69 **Queries matched:** 1
histone H2A [Leishmania infantum]
☐ Check to include this hit in error tolerant search or archive report

Query	Observed	Mr(expt)	Mr(calc)	Delta	Miss	Score	Expect	Rank	Peptide
1355	578.77	1155.53	1155.56	-0.03	0	69	6.3e-05	1	R.HDDDIGMLLK.N

Proteins matching the same set of peptides:
gi|16973355 **Mass:** 13841 **Score:** 69 **Queries matched:** 1
histone H2A [Leishmania infantum]
gi|16973359 **Mass:** 13872 **Score:** 69 **Queries matched:** 1
histone H2A [Leishmania infantum]

---

47. gi|57903381 **Mass:** 40318 **Score:** 68 **Queries matched:** 2
heat shock protein 70 [Spumella uniguttata]
☐ Check to include this hit in error tolerant search or archive report

	Query	Observed	Mr(expt)	Mr(calc)	Delta	Miss	Score	Expect	Rank	Peptide
	1488	614.76	1227.51	1227.62	-0.11	0	66	0.00014	1	R.VEIIANDQGNR.T
☑	1744	741.35	1480.69	1480.80	-0.11	0	42	0.038	1	K.SQVHEIVLVGGSTR.I

---

48. gi|10119899 **Mass:** 100749 **Score:** 68 **Queries matched:** 2
pyruvate phosphate dikinase 1 [Trypanosoma cruzi]
☐ Check to include this hit in error tolerant search or archive report

	Query	Observed	Mr(expt)	Mr(calc)	Delta	Miss	Score	Expect	Rank	Peptide
☑	1467	**609.27**	**1216.52**	**1216.60**	**-0.09**	**0**	**68**	**9.2e-05**	**1**	**K.TAEETLAAAGQR.V**
☑	580	**814.01**	**2439.00**	**2439.24**	**-0.25**	**0**	**9**	**2.2e+02**	**1**	**R.LLDPPLHEFVPHEDAAQAELAK.K**

Proteins matching the same set of peptides:
gi|71658999 **Mass:** 100785 **Score:** 68 **Queries matched:** 2
pyruvate phosphate dikinase [Trypanosoma cruzi strain CL Brener]
gi|71666490 **Mass:** 100767 **Score:** 68 **Queries matched:** 2
pyruvate phosphate dikinase [Trypanosoma cruzi strain CL Brener]

---

49. gi|71404564 **Mass:** 55484 **Score:** 66 **Queries matched:** 2
protein kinase C substrate protein, heavy chain [Trypanosoma cruzi strain CL Brener]
☐ Check to include this hit in error tolerant search or archive report

	Query	Observed	Mr(expt)	Mr(calc)	Delta	Miss	Score	Expect	Rank	Peptide
☑	1535	630.80	1259.59	1259.65	-0.06	0	63	0.00028	1	K.LSSSVEQIQNR.L

☑ 2387	940.97	2819.89	2820.14	-0.26	0	26	1.7	1	K.VAYDQECDHEPLQCSNVCENPEK.D + 3 Carbamidomethyl (C)

50. gi|33694252 **Score:** 66 **Queries matched:** 3
heat shock protein 70 [Rhynchopus sp. ATCC50230]
☐ Check to include this hit in error tolerant search or archive report

Query	Observed	Mr(expt)	Mr(calc)	Delta	Miss	Score	Expect	Rank	Peptide
1488	614.76	1227.51	1227.62	-0.11	0	66	0.00014	1	R.VEIIANDQGNR.T
1554	639.76	1277.51	1277.63	-0.12	0	12	32	10	K.DAGTISGMEILR.I + Oxidation (M)
282	650.24	1298.46	1298.60	-0.14	0	16	40	4	R.FEELCADLFR.S + Carbamidomethyl (C)

51. gi|123592 **Mass:** 56500 **Score:** 66 **Queries matched:** 2
RecName: Full=Heat shock 70 kDa protein
☐ Check to include this hit in error tolerant search or archive report

Query	Observed	Mr(expt)	Mr(calc)	Delta	Miss	Score	Expect	Rank	Peptide
1488	614.76	1227.51	1227.62	-0.11	0	66	0.00014	1	R.LDIIANDQGNR.T
1744	741.35	1480.69	1480.81	-0.13	1	18	10	4	K.RSVHDVVLVGGSTR.I

52. gi|123603 **Score:** 66 **Queries matched:** 2
RecName: Full=Heat shock 70 kDa protein
☐ Check to include this hit in error tolerant search or archive report

Query	Observed	Mr(expt)	Mr(calc)	Delta	Miss	Score	Expect	Rank	Peptide
1488	614.76	1227.51	1227.62	-0.11	0	66	0.00014	1	R.VEIIANDQGNR.T
282	650.24	1298.46	1298.60	-0.14	0	14	59	6	R.FEELCGELFR.G + Carbamidomethyl (C)

53. gi|55824402 **Score:** 66 **Queries matched:** 2
heat shock protein 70 cytosolic isoform [Cryptobia salmositica]
☐ Check to include this hit in error tolerant search or archive report

Query	Observed	Mr(expt)	Mr(calc)	Delta	Miss	Score	Expect	Rank	Peptide
1432	**598.26**	**1194.51**	**1194.57**	**-0.05**	**1**	**9**	**50**	**6**	**K.DPCGNHRALR.R + Carbamidomethyl (C)**

1488	614.76	1227.51	1227.62	-0.11	0	66	0.00014	1	R.VEIIANDQGNR.T

54. gi|23306650 **Score:** 66 **Queries matched:** 2
heat shock protein 70 [Carpediemonas membranifera]
☐ Check to include this hit in error tolerant search or archive report

Query	Observed	Mr(expt)	Mr(calc)	Delta	Miss	Score	Expect	Rank	Peptide
1488	614.76	1227.51	1227.62	-0.11	0	66	0.00014	1	R.VEIIANDQGNR.T
97	**542.33**	**1623.97**	**1623.82**	**0.15**	**0**	**5**	**2.8e+02**	**7**	**R.TFQVEEISGMVLQK.M + Oxidation (M)**

55. gi|71652570 **Mass:** 19019 **Score:** 66 **Queries matched:** 2
hypothetical protein [Trypanosoma cruzi strain CL Brener]
☐ Check to include this hit in error tolerant search or archive report

	Query	Observed	Mr(expt)	Mr(calc)	Delta	Miss	Score	Expect	Rank	Peptide
☑	1807	**788.30**	**1574.58**	**1574.68**	**-0.10**	**0**	**66**	**0.00014**	**1**	**R.NEGDLTNEAAAGEER.E**
☑	2410	**1004.42**	**3010.25**	**3010.50**	**-0.25**	**0**	**5**	**1.3e+02**	**1**	**K.SQHEQQPPPPPPPQQQQQGPAVTPVASR.A**

56. gi|71419111 **Mass:** 19554 **Score:** 61 **Queries matched:** 1
nascent polypeptide associated complex subunit [Trypanosoma cruzi strain CL Brener]
☐ Check to include this hit in error tolerant search or archive report

	Query	Observed	Mr(expt)	Mr(calc)	Delta	Miss	Score	Expect	Rank	Peptide
☑	2075	**1012.44**	**2022.87**	**2023.00**	**-0.13**	**0**	**61**	**0.00032**	**1**	**R.VEEVPTTAETPETPVPAEK.T**

Proteins matching the same set of peptides:
gi|71422974 **Mass:** 19564 **Score:** 61 **Queries matched:** 1
nascent polypeptide associated complex subunit [Trypanosoma cruzi strain CL Brener]

57. gi|71665037 **Mass:** 85375 **Score:** 61 **Queries matched:** 1
kinesin [Trypanosoma cruzi strain CL Brener]
☐ Check to include this hit in error tolerant search or archive report

Query	Observed	Mr(expt)	Mr(calc)	Delta	Miss	Score	Expect	Rank	Peptide
☑ 2105	690.23	2067.68	2067.95	-0.27	0	61	0.00048	1	R.TDVITQGDAADAHSEQPSAR.R

---

58. gi|71422090 **Mass:** 33476 **Score:** 61 **Queries matched:** 1
electron-transfer-flavoprotein, alpha polypeptide [Trypanosoma cruzi strain CL Brener]
☐ Check to include this hit in error tolerant search or archive report

Query	Observed	Mr(expt)	Mr(calc)	Delta	Miss	Score	Expect	Rank	Peptide
☑ 1316	570.27	1138.52	1138.66	-0.14	0	61	0.00037	1	K.ILNELAEPLK.A

Proteins matching the same set of peptides:
gi|71649252 **Mass:** 33464 **Score:** 61 **Queries matched:** 1
electron-transfer-flavoprotein, alpha polypeptide [Trypanosoma cruzi strain CL Brener]

---

59. gi|28779462 **Mass:** 44139 **Score:** 61 **Queries matched:** 2
elongation factor-1 alpha [Streblomastix strix]
☐ Check to include this hit in error tolerant search or archive report

Query	Observed	Mr(expt)	Mr(calc)	Delta	Miss	Score	Expect	Rank	Peptide
1056	513.25	1024.50	1024.60	-0.11	0	61	0.00037	1	K.IGGIGTVPVGR.V
☑ 502	777.12	2328.34	2328.15	0.19	2	2	1.2e+03	1	K.QMIVLVNKMDDKSVSFSEAR.Y + 2 Oxidation (M)

---

60. gi|71404826 **Mass:** 123383 **Score:** 60 **Queries matched:** 2
hypothetical protein [Trypanosoma cruzi strain CL Brener]
☐ Check to include this hit in error tolerant search or archive report

Query	Observed	Mr(expt)	Mr(calc)	Delta	Miss	Score	Expect	Rank	Peptide
☑ 265	640.35	1278.68	1278.63	0.05	0	60	0.0011	1	R.IMQLDIFDER.A
☑ 1694	713.34	1424.66	1424.70	-0.04	0	18	7.5	1	R.NAGAGDNIPGDVVAR.E

---

61. gi|71403972 **Mass:** 39482 **Score:** 59 **Queries matched:** 1
3,2-trans-enoyl-CoA isomerase [Trypanosoma cruzi strain CL Brener]

☐ Check to include this hit in error tolerant search or archive report

	Query	Observed	Mr(expt)	Mr(calc)	Delta	Miss	Score	Expect	Rank	Peptide
☑	1849	822.82	1643.62	1643.82	-0.20	0	59	0.00062	1	R.MLQLGETPTADEALR.I

Proteins matching the same set of peptides:

gi|71405843 **Mass:** 45053 **Score:** 59 **Queries matched:** 1
3,2-trans-enoyl-CoA isomerase, mitochondrial precursor [Trypanosoma cruzi strain CL Brener]

gi|72388432 **Mass:** 45305 **Score:** 59 **Queries matched:** 1
3,2-trans-enoyl-CoA isomerase, mitochondrial precursor [Trypanosoma brucei TREU927]

---

62. gi|71665810 **Mass:** 112109 **Score:** 58 **Queries matched:** 1
hypothetical protein [Trypanosoma cruzi strain CL Brener]

☐ Check to include this hit in error tolerant search or archive report

	Query	Observed	Mr(expt)	Mr(calc)	Delta	Miss	Score	Expect	Rank	Peptide
☑	1687	707.79	1413.57	1413.71	-0.14	0	58	0.00071	1	R.ELVSPTEDVQAAR.Q

---

63. gi|71399904 **Mass:** 11280 **Score:** 58 **Queries matched:** 2
histone H4 [Trypanosoma cruzi strain CL Brener]

☐ Check to include this hit in error tolerant search or archive report

	Query	Observed	Mr(expt)	Mr(calc)	Delta	Miss	Score	Expect	Rank	Peptide
☑	160	588.68	1175.35	1175.51	-0.16	0	33	0.81	1	R.DATAYTEYSR.K
☑	1528	629.31	1256.60	1256.71	-0.11	0	53	0.0024	1	K.TVTAVDVVNALR.K

Proteins matching the same set of peptides:

gi|71400209 **Mass:** 11163 **Score:** 58 **Queries matched:** 2
histone H4 [Trypanosoma cruzi strain CL Brener]

gi|71409084 **Mass:** 11177 **Score:** 58 **Queries matched:** 2
histone H4 [Trypanosoma cruzi strain CL Brener]

gi|71409100 **Mass:** 6890 **Score:** 58 **Queries matched:** 2
histone H4 [Trypanosoma cruzi strain CL Brener]

gi|72389584 **Mass:** 11135 **Score:** 58 **Queries matched:** 2

histone H4 [Trypanosoma brucei TREU927]

---

64. gi|167375825 **Mass:** 24465 **Score:** 58 **Queries matched:** 2
hypothetical protein [Entamoeba dispar SAW760]
☐ Check to include this hit in error tolerant search or archive report

	Query	Observed	Mr(expt)	Mr(calc)	Delta	Miss	Score	Expect	Rank	Peptide
	156	584.69	1167.36	1167.57	-0.21	0	9	1.4e+02	2	-.MTTEITDTIK.L + Oxidation (M)
☑	1596	651.84	1301.66	1301.68	-0.02	0	58	0.00077	1	K.DVTIDISIAEAR.I

---

65. gi|71664019 **Mass:** 51642 **Score:** 57 **Queries matched:** 1
hypothetical protein [Trypanosoma cruzi strain CL Brener]
☐ Check to include this hit in error tolerant search or archive report

	Query	Observed	Mr(expt)	Mr(calc)	Delta	Miss	Score	Expect	Rank	Peptide
☑	1548	636.83	1271.64	1271.69	-0.05	0	57	0.00089	1	R.LLIQEGIAEMR.D

---

66. gi|71655108 **Mass:** 53810 **Score:** 57 **Queries matched:** 1
hypothetical protein [Trypanosoma cruzi strain CL Brener]
☐ Check to include this hit in error tolerant search or archive report

	Query	Observed	Mr(expt)	Mr(calc)	Delta	Miss	Score	Expect	Rank	Peptide
☑	1708	481.26	1440.75	1440.74	0.01	0	57	0.00093	1	K.ISAGGNAENLRPSR.V

Proteins matching the same set of peptides:
gi|71663349 **Mass:** 53721 **Score:** 57 **Queries matched:** 1
hypothetical protein [Trypanosoma cruzi strain CL Brener]

---

67. gi|71667211 **Mass:** 30381 **Score:** 57 **Queries matched:** 4
hypothetical protein [Trypanosoma cruzi strain CL Brener]
☐ Check to include this hit in error tolerant search or archive report

Query	Observed	Mr(expt)	Mr(calc)	Delta	Miss	Score	Expect	Rank	Peptide

26	469.46	1405.37	1405.60	-0.24	1	(19)	18	1	K.EEFKDDFDFSK.S
1682	703.75	1405.50	1405.60	-0.11	1	55	0.0017	1	K.EEFKDDFDFSK.S
2293	805.02	2412.05	2412.24	-0.20	0	(20)	4.5	1	R.GQEAGLAQIPPADQLFDQIVFR.G
2294	1207.05	2412.09	2412.24	-0.15	0	29	0.54	1	R.GQEAGLAQIPPADQLFDQIVFR.G

---

68. gi|71660723 **Mass:** 116424 **Score:** 55 **Queries matched:** 2
hypothetical protein [Trypanosoma cruzi strain CL Brener]
Check to include this hit in error tolerant search or archive report

Query	Observed	Mr(expt)	Mr(calc)	Delta	Miss	Score	Expect	Rank	Peptide
2015	954.41	1906.80	1906.96	-0.16	0	57	0.00092	1	R.IVATDLSEPLPAPADDAGR.A
2296	805.93	2414.76	2415.03	-0.27	0	20	7.7	1	R.AEEPCQHEIEQNYPDSEGVGK.F + Carbamidomethyl (C)

Proteins matching the same set of peptides:
gi|71418777 **Mass:** 116402 **Score:** 55 **Queries matched:** 2
hypothetical protein [Trypanosoma cruzi strain CL Brener]

---

69. gi|71403618 **Mass:** 60494 **Score:** 55 **Queries matched:** 1
hypothetical protein Tc00.1047053504411.10 [Trypanosoma cruzi strain CL Brener]
Check to include this hit in error tolerant search or archive report

Query	Observed	Mr(expt)	Mr(calc)	Delta	Miss	Score	Expect	Rank	Peptide
1651	687.23	1372.44	1372.67	-0.23	0	55	0.0016	1	K.GPTASTGAGVEPAMK.A

Proteins matching the same set of peptides:
gi|71405377 **Mass:** 60491 **Score:** 55 **Queries matched:** 1
hypothetical protein [Trypanosoma cruzi strain CL Brener]

---

70. gi|58414949 **Score:** 55 **Queries matched:** 4
polyubiquitin [Stauracon pallidus]
Check to include this hit in error tolerant search or archive report

Query	Observed	Mr(expt)	Mr(calc)	Delta	Miss	Score	Expect	Rank	Peptide

477	383.18	764.34	764.43	-0.08	0	23	2.3	4	-.MQIFVK.T
1164	534.28	1066.55	1066.61	-0.07	0	55	0.0016	1	K.ESTLHLVLR.L
1779	508.55	1522.63	1522.77	-0.15	1	25	1.6	1	K.IQDKEGIPPDQQR.L
1914	874.39	1746.76	1746.89	-0.13	0	24	2.2	3	K.TITLEVEASDSIENVK.A

---

71. gi|33358312 **Score:** 55 **Queries matched:** 2
ubiquitin-like protein Ublp94.4 [Acanthamoeba castellanii]
☐ Check to include this hit in error tolerant search or archive report

Query	Observed	Mr(expt)	Mr(calc)	Delta	Miss	Score	Expect	Rank	Peptide
1164	534.28	1066.55	1066.61	-0.07	0	55	0.0016	1	K.ESTLHLVLR.L
1779	508.55	1522.63	1522.77	-0.15	1	25	1.6	1	K.LQDKEGIPPDQQR.L

---

72. gi|154411942 **Mass:** 27817 **Score:** 55 **Queries matched:** 1
hypothetical protein [Trichomonas vaginalis G3]
☐ Check to include this hit in error tolerant search or archive report

Query	Observed	Mr(expt)	Mr(calc)	Delta	Miss	Score	Expect	Rank	Peptide
1164	534.28	1066.55	1066.61	-0.07	0	55	0.0016	1	R.DTTIHLVLR.C

---

73. gi|9954108 **Mass:** 34491 **Score:** 55 **Queries matched:** 2
RNA binding protein RGGm [Trypanosoma cruzi]
☐ Check to include this hit in error tolerant search or archive report

	Query	Observed	Mr(expt)	Mr(calc)	Delta	Miss	Score	Expect	Rank	Peptide
☑	1690	**710.23**	**1418.45**	**1418.70**	**-0.26**	**0**	**(29)**	**0.69**	**1**	**R.AVVEFVTPEDASR.A**
☑	1691	**710.25**	**1418.48**	**1418.70**	**-0.22**	**0**	**53**	**0.0026**	**1**	**R.AVVEFVTPEDASR.A**

Proteins matching the same set of peptides:
gi|71410145 **Mass:** 34635 **Score:** 55 **Queries matched:** 2
RNA-binding protein RGGm [Trypanosoma cruzi strain CL Brener]
gi|71652462 **Mass:** 33389 **Score:** 55 **Queries matched:** 2
RNA-binding protein RGGm [Trypanosoma cruzi strain CL Brener]

74. gi|71404616 **Mass:** 11926 **Score:** 54 **Queries matched:** 4
hypothetical protein [Trypanosoma cruzi strain CL Brener]
☐ Check to include this hit in error tolerant search or archive report

	Query	Observed	Mr(expt)	Mr(calc)	Delta	Miss	Score	Expect	Rank	Peptide
☑	2124	700.29	2097.85	2098.00	-0.15	0	44	0.017	1	K.GLEHYGLDPTDAEVANEIR.K
☑	2125	1049.94	2097.86	2098.00	-0.14	0	(28)	0.7	1	K.GLEHYGLDPTDAEVANEIR.K
☑	2209	742.95	2225.83	2226.09	-0.26	1	15	15	1	K.GLEHYGLDPTDAEVANEIRK.Y
☑	2241	766.31	2295.92	2296.13	-0.21	0	34	0.2	1	R.HLNYEELENNIGGLPITENK.L

Proteins matching the same set of peptides:
gi|71405087 **Mass:** 11954 **Score:** 54 **Queries matched:** 4
hypothetical protein [Trypanosoma cruzi strain CL Brener]

75. gi|71660349 **Mass:** 50950 **Score:** 53 **Queries matched:** 1
hypothetical protein [Trypanosoma cruzi strain CL Brener]
☐ Check to include this hit in error tolerant search or archive report

	Query	Observed	Mr(expt)	Mr(calc)	Delta	Miss	Score	Expect	Rank	Peptide
☑	2333	845.90	2534.68	2535.02	-0.34	0	53	0.003	1	R.AHQDSCPQCSQEATAVCTSDGAR.S + 3 Carbamidomethyl (C)

76. gi|71425751 **Mass:** 16513 **Score:** 53 **Queries matched:** 1
centrin [Trypanosoma cruzi strain CL Brener]
☐ Check to include this hit in error tolerant search or archive report

	Query	Observed	Mr(expt)	Mr(calc)	Delta	Miss	Score	Expect	Rank	Peptide
☑	820	466.23	930.44	930.49	-0.05	0	53	0.0027	1	K.GLGFGDLPR.D

Proteins matching the same set of peptides:
gi|72391340 **Mass:** 16542 **Score:** 53 **Queries matched:** 1
centrin [Trypanosoma brucei TREU927]
gi|154337629 **Mass:** 16487 **Score:** 53 **Queries matched:** 1
Ca2+-binding EF-hand protein [Leishmania braziliensis MHOM/BR/75/M2904]

77. gi|53829568 **Mass:** 52571 **Score:** 52 **Queries matched:** 3
HSP70 [Ministeria vibrans]
☐ Check to include this hit in error tolerant search or archive report

Query	Observed	Mr(expt)	Mr(calc)	Delta	Miss	Score	Expect	Rank	Peptide
1549	**637.89**	**1273.76**	**1273.71**	**0.05**	**2**	**0**	**4.8e+02**	**7**	**K.KDLTQSARSLR.R**
282	650.24	1298.46	1298.60	-0.14	0	16	40	4	R.FEELCADLFR.G + Carbamidomethyl (C)
1751	744.30	1486.59	1486.69	-0.10	0	52	0.0029	1	R.TTPSYVAFTDTER.L

78. gi|84105385 **Mass:** 68015 **Score:** 52 **Queries matched:** 2
cytosolic heat shock protein 70 [Malawimonas jakobiformis]
☐ Check to include this hit in error tolerant search or archive report

Query	Observed	Mr(expt)	Mr(calc)	Delta	Miss	Score	Expect	Rank	Peptide
282	650.24	1298.46	1298.60	-0.14	0	14	59	6	R.FEELCIDQFR.K
1751	744.30	1486.59	1486.69	-0.10	0	52	0.0029	1	R.TTPSYVAFTDTER.L

79. gi|71664824 **Mass:** 31803 **Score:** 52 **Queries matched:** 2
mucin TcMUCII [Trypanosoma cruzi strain CL Brener]
☐ Check to include this hit in error tolerant search or archive report

	Query	Observed	Mr(expt)	Mr(calc)	Delta	Miss	Score	Expect	Rank	Peptide
☑	1867	**838.66**	**1675.31**	**1675.79**	**-0.48**	**0**	**52**	**0.0054**	**1**	**K.QGPPSAPQGQPVDENR.L**
	1934	**886.27**	**1770.53**	**1770.87**	**-0.35**	**0**	**2**	**4.1e+02**	**4**	**K.NAGLSEDASGLQGITNPK.N**

80. gi|71411561 **Mass:** 14251 **Score:** 52 **Queries matched:** 2
hypothetical protein [Trypanosoma cruzi strain CL Brener]
☐ Check to include this hit in error tolerant search or archive report

	Query	Observed	Mr(expt)	Mr(calc)	Delta	Miss	Score	Expect	Rank	Peptide
☑	77	**526.27**	**1050.53**	**1050.57**	**-0.04**	**0**	**52**	**0.0068**	**1**	**R.LATFTELTR.Q**
☑	64	**514.85**	**1541.51**	**1541.71**	**-0.20**	**0**	**13**	**53**	**1**	**R.DGQSITWHEVNEK.G**

Proteins matching the same set of peptides:
gi|71667238 **Mass:** 14290 **Score:** 52 **Queries matched:** 2
hypothetical protein [Trypanosoma cruzi strain CL Brener]

---

81. gi|1322226 **Mass:** 44368 **Score:** 52 **Queries matched:** 2
elongation factor 1 alpha
☐ Check to include this hit in error tolerant search or archive report

	Query	Observed	Mr(expt)	Mr(calc)	Delta	Miss	Score	Expect	Rank	Peptide
	935	488.28	974.54	974.54	-0.00	0	52	0.0048	1	R.LPIQDVYK.I
☑	1064	**1028.93**	**1027.92**	**1027.50**	**0.42**	**1**	**8**	**2.2e+02**	**1**	**K.TEMQKTFK.Q + Oxidation (M)**

---

82. gi|71665461 **Mass:** 46415 **Score:** 51 **Queries matched:** 3
enolase [Trypanosoma cruzi strain CL Brener]
☐ Check to include this hit in error tolerant search or archive report

	Query	Observed	Mr(expt)	Mr(calc)	Delta	Miss	Score	Expect	Rank	Peptide
☑	1602	655.25	1308.48	1308.74	-0.26	0	(25)	1.8	1	K.NVNDVLAPALVGK.D
☑	1604	655.39	1308.77	1308.74	0.03	0	46	0.011	1	K.NVNDVLAPALVGK.D
☑	1975	614.60	1840.76	1840.87	-0.11	0	35	0.16	1	R.SAVPSGASTGIHEACELR.D + Carbamidomethyl (C)

---

83. gi|71412664 **Mass:** 60479 **Score:** 50 **Queries matched:** 2
hypothetical protein [Trypanosoma cruzi strain CL Brener]
☐ Check to include this hit in error tolerant search or archive report

	Query	Observed	Mr(expt)	Mr(calc)	Delta	Miss	Score	Expect	Rank	Peptide
	1680	702.84	1403.67	1403.69	-0.03	1	15	14	2	K.TTYKEELYTTR.L
☑	1809	788.81	1575.61	1575.78	-0.16	0	50	0.0053	1	R.SPGFNPAAVPYTPMK.T

---

84. gi|71402512 **Mass:** 52546 **Score:** 48 **Queries matched:** 1
eukaryotic translation initiation factor 2 subunit [Trypanosoma cruzi strain CL Brener]
☐ Check to include this hit in error tolerant search or archive report

Query	Observed	Mr(expt)	Mr(calc)	Delta	Miss	Score	Expect	Rank	Peptide
☑ 1747	743.37	1484.73	1484.78	-0.06	0	48	0.0075	1	R.GLATQDINALNLDK.I

---

85. gi|71649424 **Mass:** 151616 **Score:** 48 **Queries matched:** 2
hypothetical protein [Trypanosoma cruzi strain CL Brener]
☐ Check to include this hit in error tolerant search or archive report

Query	Observed	Mr(expt)	Mr(calc)	Delta	Miss	Score	Expect	Rank	Peptide
☑ 1956	607.27	1818.79	1818.95	-0.15	0	48	0.0085	1	R.LVVETELHPADAELQR.R
☑ 1502	1236.42	2470.82	2470.23	0.59	1	4	5.6e+02	1	R.IEELISSQVEYKGVSTSVEAMR.L + Oxidation (M)

---

86. gi|71412308 **Mass:** 152202 **Score:** 48 **Queries matched:** 2
hypothetical protein [Trypanosoma cruzi strain CL Brener]
☐ Check to include this hit in error tolerant search or archive report

Query	Observed	Mr(expt)	Mr(calc)	Delta	Miss	Score	Expect	Rank	Peptide
1956	607.27	1818.79	1818.95	-0.15	0	48	0.0085	1	R.LVVETELHPADAELQR.R
1502	1236.42	2470.82	2470.26	0.56	2	2	9.9e+02	7	R.IEELISSKVEYKGVSTSVEAMR.L + Oxidation (M)

---

87. gi|1170958 **Mass:** 22122 **Score:** 47 **Queries matched:** 2
RecName: Full=Macrophage infectivity potentiator; AltName: Full=Peptidyl-prolyl cis-trans isomerase
☐ Check to include this hit in error tolerant search or archive report

Query	Observed	Mr(expt)	Mr(calc)	Delta	Miss	Score	Expect	Rank	Peptide
☑ 1037	508.75	1015.48	1015.58	-0.10	0	47	0.012	1	K.LPSGLVFQR.I
1750	496.27	1485.80	1485.83	-0.03	0	4	1.8e+02	7	R.GKPTTFRPNEVIK.G

Proteins matching the same set of peptides:
gi|21465757 **Mass:** 18822 **Score:** 47 **Queries matched:** 2
Chain A, Trypanosoma Cruzi Macrophage Infectivity Potentiator (Tcmip)
gi|71418561 **Mass:** 22140 **Score:** 47 **Queries matched:** 2
macrophage infectivity potentiator, precursor [Trypanosoma cruzi strain CL Brener]

88. gi|71398774 **Mass:** 29630 **Score:** 47 **Queries matched:** 1
hypothetical protein [Trypanosoma cruzi strain CL Brener]
☐ Check to include this hit in error tolerant search or archive report

	Query	Observed	Mr(expt)	Mr(calc)	Delta	Miss	Score	Expect	Rank	Peptide
☑	168	591.70	1181.38	1181.57	-0.19	0	47	0.032	1	K.FSVDVEYAPR.C

Proteins matching the same set of peptides:
gi|71398776 **Mass:** 27357 **Score:** 47 **Queries matched:** 1
hypothetical protein [Trypanosoma cruzi strain CL Brener]
gi|71398971 **Mass:** 29609 **Score:** 47 **Queries matched:** 1
hypothetical protein [Trypanosoma cruzi strain CL Brener]
gi|71404900 **Mass:** 29621 **Score:** 47 **Queries matched:** 1
hypothetical protein [Trypanosoma cruzi strain CL Brener]
gi|71661320 **Mass:** 29622 **Score:** 47 **Queries matched:** 1
hypothetical protein [Trypanosoma cruzi strain CL Brener]
gi|71668366 **Mass:** 29617 **Score:** 47 **Queries matched:** 1
hypothetical protein [Trypanosoma cruzi strain CL Brener]

---

89. gi|71399257 **Mass:** 15927 **Score:** 46 **Queries matched:** 2
hypothetical protein [Trypanosoma cruzi strain CL Brener]
☐ Check to include this hit in error tolerant search or archive report

	Query	Observed	Mr(expt)	Mr(calc)	Delta	Miss	Score	Expect	Rank	Peptide
☑	1951	905.35	1808.69	1808.85	-0.15	0	46	0.01	1	R.EQPPTQPAQAQEMAQR.V
	1960	913.43	1824.84	1824.84	-0.00	0	(7)	88	2	R.EQPPTQPAQAQEMAQR.V + Oxidation (M)

Proteins matching the same set of peptides:
gi|71405782 **Mass:** 15933 **Score:** 46 **Queries matched:** 2
hypothetical protein [Trypanosoma cruzi strain CL Brener]

---

90. gi|886414 **Mass:** 44190 **Score:** 46 **Queries matched:** 1
TCJ2 [Trypanosoma cruzi]
☐ Check to include this hit in error tolerant search or archive report

Query	Observed	Mr(expt)	Mr(calc)	Delta	Miss	Score	Expect	Rank	Peptide
2056	666.64	1996.90	1996.97	-0.07	1	46	0.012	1	K.FYDSLGVSPDASVDEIKR.A

Proteins matching the same set of peptides:
gi|71417326 **Mass:** 44176 **Score:** 46 **Queries matched:** 1
heat shock protein DnaJ [Trypanosoma cruzi strain CL Brener]

---

91. gi|71666956 **Mass:** 40800 **Score:** 45 **Queries matched:** 1
hypothetical protein [Trypanosoma cruzi strain CL Brener]
☐ Check to include this hit in error tolerant search or archive report

Query	Observed	Mr(expt)	Mr(calc)	Delta	Miss	Score	Expect	Rank	Peptide
1625	667.72	1333.42	1333.59	-0.17	0	45	0.016	1	K.QEINECVETGR.E + Carbamidomethyl (C)

---

92. gi|71408304 **Mass:** 32648 **Score:** 45 **Queries matched:** 2
ubiquitin-like protein [Trypanosoma cruzi strain CL Brener]
☐ Check to include this hit in error tolerant search or archive report

Query	Observed	Mr(expt)	Mr(calc)	Delta	Miss	Score	Expect	Rank	Peptide
1123	527.29	1052.57	1052.72	-0.15	2	15	13	6	R.IVLRGRVLK.D
1655	688.30	1374.58	1374.76	-0.18	0	45	0.018	1	R.VLYQSQLQQLR.E

---

93. gi|167536654 **Mass:** 104427 **Score:** 44 **Queries matched:** 2
hypothetical protein [Monosiga brevicollis MX1]
☐ Check to include this hit in error tolerant search or archive report

Query	Observed	Mr(expt)	Mr(calc)	Delta	Miss	Score	Expect	Rank	Peptide
1411	592.77	1183.53	1183.67	-0.14	1	44	0.019	1	R.NVELQRQGLK.A
1733	736.26	1470.51	1470.77	-0.26	1	8	78	10	K.KGDDDLLAVLQER.T

---

94. gi|154346020 **Mass:** 79350 **Score:** 44 **Queries matched:** 3
cullin-like protein [Leishmania braziliensis MHOM/BR/75/M2904]

☐ Check to include this hit in error tolerant search or archive report

	Query	Observed	Mr(expt)	Mr(calc)	Delta	Miss	Score	Expect	Rank	Peptide
	758	903.76	902.76	902.45	0.31	0	2	1.1e+03	3	K.SEGIINDR.S
☑	1279	1123.10	1122.09	1121.56	0.54	1	3	8.6e+02	1	K.EVSRNSFQR.I
☑	1790	775.80	1549.59	1549.82	-0.23	0	44	0.021	1	K.WGIIEETVLCIFK.K

---

95. gi|120679 **Mass:** 39036 **Score:** 43 **Queries matched:** 1
RecName: Full=Glyceraldehyde-3-phosphate dehydrogenase, glycosomal; Short=GAPDH

☐ Check to include this hit in error tolerant search or archive report

	Query	Observed	Mr(expt)	Mr(calc)	Delta	Miss	Score	Expect	Rank	Peptide
☑	1668	692.84	1383.66	1383.77	-0.11	0	43	0.023	1	R.AAAVNIIPSTTGAAK.A

Proteins matching the same set of peptides:

gi|1311019 **Mass:** 38618 **Score:** 43 **Queries matched:** 1
Chain A, Crystal Structure Of Glycosomal Glyceraldehyde-3-Phosphate Dehydrogenase From Leishmania M

gi|3023811 **Mass:** 39008 **Score:** 43 **Queries matched:** 1
RecName: Full=Glyceraldehyde-3-phosphate dehydrogenase, glycosomal; Short=GAPDH

gi|3318907 **Mass:** 38877 **Score:** 43 **Queries matched:** 1
Chain A, Glycosomal Glyceraldehyde-3-Phosphate Dehydrogenase In A Monoclinic Crystal Form

gi|4105573 **Mass:** 39029 **Score:** 43 **Queries matched:** 1
glycosomal glyceraldehyde-3-phosphate dehydrogenase [Leptomonas seymouri]

gi|4335878 **Mass:** 36910 **Score:** 43 **Queries matched:** 1
glycosomal glyceraldehyde-3-phosphate dehydrogenase [Leptomonas seymouri]

gi|4335880 **Mass:** 36843 **Score:** 43 **Queries matched:** 1
glycosomal glyceraldehyde-3-phosphate dehydrogenase [Crithidia fasciculata]

gi|4335882 **Mass:** 36978 **Score:** 43 **Queries matched:** 1
glycosomal glyceraldehyde-3-phosphate dehydrogenase [Crithidia luciliae]

gi|12802907 **Mass:** 39195 **Score:** 43 **Queries matched:** 1
glycosomal glyceraldehyde-3-phosphate dehydrogenase [Leptomonas sp. Nfm]

gi|12958498 **Mass:** 39090 **Score:** 43 **Queries matched:** 1
glycosomal glyceraldehyde-3-phosphate dehydrogenase [Leptomonas sp. Cfm]

gi|12964748 **Mass:** 39248 **Score:** 43 **Queries matched:** 1

glycosomal glyceraldehyde-3-phosphate dehydrogenase [Wallaceina brevicula]
gi|12964782 **Mass:** 39243 **Score:** 43 **Queries matched:** 1
glycosomal glyceraldehyde-3-phosphate dehydrogenase [Leptomonas peterhoffi]
gi|12964784 **Mass:** 39118 **Score:** 43 **Queries matched:** 1
glycosomal glyceraldehyde-3-phosphate dehydrogenase [Blastocrithidia gerricola]
gi|14150819 **Mass:** 39155 **Score:** 43 **Queries matched:** 1
glycosomal glyceraldehyde-3-phosphate dehydrogenase [Leptomonas sp. F2]
gi|26393926 **Mass:** 39063 **Score:** 43 **Queries matched:** 1
RecName: Full=Glyceraldehyde-3-phosphate dehydrogenase, glycosomal; Short=GAPDH
gi|53849793 **Mass:** 32571 **Score:** 43 **Queries matched:** 1
glycosomal glyceraldehyde phosphate dehydrogenase [Trypanosoma mega]
gi|53849799 **Mass:** 32277 **Score:** 43 **Queries matched:** 1
glycosomal glyceraldehyde phosphate dehydrogenase [Trypanosoma rotatorium]
gi|53849807 **Mass:** 31715 **Score:** 43 **Queries matched:** 1
glycosomal glyceraldehyde phosphate dehydrogenase [Trypanosoma sp. T. grayi-like-F4]
gi|53849819 **Mass:** 32327 **Score:** 43 **Queries matched:** 1
glycosomal glyceraldehyde phosphate dehydrogenase [Trypanosoma binneyi]
gi|53849823 **Mass:** 35106 **Score:** 43 **Queries matched:** 1
glycosomal glyceraldehyde phosphate dehydrogenase [Trypanosoma cruzi]
gi|53849825 **Mass:** 32281 **Score:** 43 **Queries matched:** 1
glycosomal glyceraldehyde phosphate dehydrogenase [Trypanosoma cruzi]
gi|53849827 **Mass:** 34570 **Score:** 43 **Queries matched:** 1
glycosomal glyceraldehyde phosphate dehydrogenase [Trypanosoma cruzi marinkellei]
gi|53849831 **Mass:** 30859 **Score:** 43 **Queries matched:** 1
glycosomal glyceraldehyde phosphate dehydrogenase [Trypanosoma lewisi]
gi|53849833 **Mass:** 32472 **Score:** 43 **Queries matched:** 1
glycosomal glyceraldehyde phosphate dehydrogenase [Trypanosoma microti]
gi|53849837 **Mass:** 32413 **Score:** 43 **Queries matched:** 1
glycosomal glyceraldehyde phosphate dehydrogenase [Trypanosoma pestanai]
gi|53849839 **Mass:** 30722 **Score:** 43 **Queries matched:** 1
glycosomal glyceraldehyde phosphate dehydrogenase [Trypanosoma sp. kangaroo H25]
gi|53849843 **Mass:** 32346 **Score:** 43 **Queries matched:** 1
glycosomal glyceraldehyde phosphate dehydrogenase [Trypanosoma sp. wallaby ABF]
gi|53849849 **Mass:** 33565 **Score:** 43 **Queries matched:** 1
glycosomal glyceraldehyde phosphate dehydrogenase [Trypanosoma sp. Australian rabbit R5]
gi|53849851 **Mass:** 30626 **Score:** 43 **Queries matched:** 1

glycosomal glyceraldehyde phosphate dehydrogenase [Trypanosoma theileri]
gi|71422448 **Mass:** 39008 **Score:** 43 **Queries matched:** 1
glyceraldehyde 3-phosphate dehydrogenase [Trypanosoma cruzi strain CL Brener]
gi|71650185 **Mass:** 38993 **Score:** 43 **Queries matched:** 1
glyceraldehyde 3-phosphate dehydrogenase [Trypanosoma cruzi strain CL Brener]
gi|72398621 **Mass:** 38929 **Score:** 43 **Queries matched:** 1
glycosomal glyceraldehyde-3-phosphate dehydrogenase [Herpetomonas megaseliae]
gi|72398623 **Mass:** 38974 **Score:** 43 **Queries matched:** 1
glycosomal glyceraldehyde-3-phosphate dehydrogenase [Herpetomonas muscarum]
gi|72398625 **Mass:** 39034 **Score:** 43 **Queries matched:** 1
glycosomal glyceraldehyde-3-phosphate dehydrogenase [Leishmania tarentolae]
gi|88707325 **Mass:** 39010 **Score:** 43 **Queries matched:** 1
glycosomal glyceraldehyde-3-phosphate dehydrogenase [Leptomonas costaricensis]
gi|90025012 **Mass:** 30568 **Score:** 43 **Queries matched:** 1
glycosomal glyceraldehyde-3-phosphate dehydrogenase [Sergeia podlipaevi]
gi|118133302 **Mass:** 39149 **Score:** 43 **Queries matched:** 1
glycosomal glyceraldehyde-3-phosphate dehydrogenase [Leptomonas acus]
gi|118133306 **Mass:** 39025 **Score:** 43 **Queries matched:** 1
glycosomal glyceraldehyde-3-phosphate dehydrogenase [Leptomonas bifurcata]
gi|154341937 **Mass:** 39180 **Score:** 43 **Queries matched:** 1
glyceraldehyde 3-phosphate dehydrogenase,glycosomal [Leishmania braziliensis MHOM/BR/75/M2904]
gi|156523912 **Mass:** 36418 **Score:** 43 **Queries matched:** 1
glycosomal glyceraldehyde-3-phosphate dehydrogenase [Leptomonas sp. tarcoles]
gi|156523918 **Mass:** 34851 **Score:** 43 **Queries matched:** 1
glycosomal glyceraldehyde-3-phosphate dehydrogenase [Crithidia deanei]
gi|156523920 **Mass:** 36561 **Score:** 43 **Queries matched:** 1
glycosomal glyceraldehyde-3-phosphate dehydrogenase [Leptomonas sp. P]
gi|158576045 **Mass:** 36487 **Score:** 43 **Queries matched:** 1
glycosomal glyceraldehyde-3-phosphate dehydrogenase [Trypanosomatidae sp. 119YS]
gi|158576143 **Mass:** 39171 **Score:** 43 **Queries matched:** 1
glycosomal glyceraldehyde-3-phosphate dehydrogenase [Trypanosomatidae sp. 127AL]
gi|158576178 **Mass:** 36319 **Score:** 43 **Queries matched:** 1
glycosomal glyceraldehyde-3-phosphate dehydrogenase [Trypanosomatidae sp. 128SI]
gi|158576201 **Mass:** 36720 **Score:** 43 **Queries matched:** 1
glycosomal glyceraldehyde-3-phosphate dehydrogenase [Wallaceina inconstans]
gi|158577406 **Mass:** 36564 **Score:** 43 **Queries matched:** 1

glycosomal glyceraldehyde-3-phosphate dehydrogenase [Crithidia oncopelti]
gi|158577417 **Mass:** 36694 **Score:** 43 **Queries matched:** 1
glycosomal glyceraldehyde-3-phosphate dehydrogenase [Crithidia oncopelti]
gi|158577432 **Mass:** 35387 **Score:** 43 **Queries matched:** 1
glycosomal glyceraldehyde-3-phosphate dehydrogenase [Blastocrithidia culicis]
gi|158577450 **Mass:** 36511 **Score:** 43 **Queries matched:** 1
glycosomal glyceraldehyde-3-phosphate dehydrogenase [Blastocrithidia culicis]
gi|158828856 **Mass:** 36334 **Score:** 43 **Queries matched:** 1
glycosomal glyceraldehyde-3-phosphate dehydrogenase [Blastocrithidia miridarum]
gi|158828858 **Mass:** 36539 **Score:** 43 **Queries matched:** 1
glycosomal glyceraldehyde-3-phosphate dehydrogenase [Blastocrithidia miridarum]
gi|158828862 **Mass:** 36662 **Score:** 43 **Queries matched:** 1
glycosomal glyceraldehyde-3-phosphate dehydrogenase [Leptomonas collosoma]
gi|159157539 **Mass:** 35137 **Score:** 43 **Queries matched:** 1
glycosomal glyceraldehyde-3-phosphate dehydrogenase [Trypanosoma kuseli]
gi|159157541 **Mass:** 35192 **Score:** 43 **Queries matched:** 1
glycosomal glyceraldehyde-3-phosphate dehydrogenase [Trypanosoma grosi]
gi|159157547 **Mass:** 34955 **Score:** 43 **Queries matched:** 1
glycosomal glyceraldehyde-3-phosphate dehydrogenase [Trypanosoma cruzi]
gi|169264614 **Mass:** 31557 **Score:** 43 **Queries matched:** 1
glycosomal glyceraldehyde-3-phosphate dehydrogenase [uncultured kinetoplastid]
gi|169264616 **Mass:** 29907 **Score:** 43 **Queries matched:** 1
glycosomal glyceraldehyde-3-phosphate dehydrogenase [uncultured kinetoplastid]

---

96. gi|71653479 **Mass:** 12654 **Score:** 43 **Queries matched:** 1
cytochrome b5-like [Trypanosoma cruzi strain CL Brener]
☐ Check to include this hit in error tolerant search or archive report

	Query	Observed	Mr(expt)	Mr(calc)	Delta	Miss	Score	Expect	Rank	Peptide
☑	1931	590.53	1768.58	1768.87	-0.29	0	43	0.028	1	K.FVDQHPGGVDTLTGAAGK.D

Proteins matching the same set of peptides:
gi|71664175 **Mass:** 10049 **Score:** 43 **Queries matched:** 1
cytochrome b5 [Trypanosoma cruzi strain CL Brener]

---

97. gi|71659820 **Mass:** 17641 **Score:** 43 **Queries matched:** 1

calmodulin [Trypanosoma cruzi strain CL Brener]

☐ Check to include this hit in error tolerant search or archive report

	Query	Observed	Mr(expt)	Mr(calc)	Delta	Miss	Score	Expect	Rank	Peptide
☑	1941	599.93	1796.78	1796.89	-0.12	1	43	0.024	1	R.VFDKDLDGTVSVAEFR.H

Proteins matching the same set of peptides:

gi|72549191 **Mass:** 15679 **Score:** 43 **Queries matched:** 1
EF hand containing protein [Leishmania major strain Friedlin]

gi|146101748 **Mass:** 15698 **Score:** 43 **Queries matched:** 1
EF hand containing protein; calmodulin-like protein [Leishmania infantum]

gi|154345097 **Mass:** 15583 **Score:** 43 **Queries matched:** 1
EF hand containing protein [Leishmania braziliensis MHOM/BR/75/M2904]

---

98. gi|133055 **Mass:** 10747 **Score:** 42 **Queries matched:** 1
60S acidic ribosomal protein P1

☐ Check to include this hit in error tolerant search or archive report

	Query	Observed	Mr(expt)	Mr(calc)	Delta	Miss	Score	Expect	Rank	Peptide
☑	2335	850.38	2548.12	2548.34	-0.22	0	42	0.026	1	K.VSFGGVAPAAGGATAAPAAAAAAAPAAAAAK.K

---

99. gi|71418715 **Score:** 42 **Queries matched:** 1
60S acidic ribosomal protein [Trypanosoma cruzi strain CL Brener]

☐ Check to include this hit in error tolerant search or archive report

Query	Observed	Mr(expt)	Mr(calc)	Delta	Miss	Score	Expect	Rank	Peptide
2335	850.38	2548.12	2548.34	-0.22	0	42	0.026	1	K.VSFGGVAPAAGGATAAPAAAAAAAPAAAAAAK.K

---

100. gi|27734387 **Score:** 42 **Queries matched:** 2
polyubiquitin [Euglypha rotunda]

☐ Check to include this hit in error tolerant search or archive report

Query	Observed	Mr(expt)	Mr(calc)	Delta	Miss	Score	Expect	Rank	Peptide

1779	508.55	1522.63	1522.77	-0.15	1	25	1.6	1	K.IQDKEGIPPDQQR.L
1914	874.39	1746.76	1746.89	-0.13	0	42	0.029	2	K.TITLDVEASDTIENVK.Q

Proteins matching the same set of peptides:
gi|27734383 **Score:** 42 **Queries matched:** 2

Peptide matches not assigned to protein hits: (no details means no match)

	Query	Observed	Mr(expt)	Mr(calc)	Delta	Miss	Score	Expect	Rank	Peptide
☑	1999	629.92	1886.73	1886.90	-0.17	0	41	0.04	1	IYDDDGNLKPGETTPPR
☑	1244	552.72	1103.43	1103.59	-0.16	0	41	0.044	1	ILDEIICASK
☑	2164	717.99	2150.95	2151.13	-0.18	0	40	0.054	1	GFERPSPVQEEAIPVALQGK
☑	1792	776.85	1551.69	1551.80	-0.11	0	40	0.047	1	VDNLTVAPGPPDVMK
☑	1642	682.87	1363.72	1363.73	-0.01	0	37	0.11	1	VSIVATDIFTGNK
☑	14	434.14	1299.40	1299.62	-0.23	1	36	0.26	1	KQWAFYNDTK
☑	930	487.22	972.43	972.55	-0.12	0	36	0.17	1	LEISALEAK
☑	1697	715.22	1428.43	1428.72	-0.29	0	35	0.14	1	EAVGSLSPQPTSTR
☑	1947	600.91	1799.72	1799.84	-0.12	1	35	0.13	1	IVQVPDDGADEKEEEK
☑	1070	516.26	1030.50	1030.55	-0.05	0	35	0.2	1	VLMPLAESR + Oxidation (M)
☑	858	471.74	941.47	941.52	-0.05	0	35	0.15	1	IEIVSPER
☑	1671	463.25	1386.72	1386.82	-0.11	1	35	0.18	1	KVPIVPLEDLHK
☑	1935	887.37	1772.73	1772.87	-0.14	1	35	0.18	1	KLLEEVGDGIDVDDEK
☑	573	406.17	810.32	810.39	-0.07	0	34	0.14	1	MAFALDK + Oxidation (M)
☑	1967	917.93	1833.84	1833.98	-0.14	2	34	0.18	1	NHTSKVAAGLDGANPVKR
☑	1090	519.72	1037.42	1037.53	-0.11	0	34	0.22	1	FNQEVFVR
☑	377	705.74	1409.46	1409.62	-0.16	0	33	0.74	1	EGDMLSLMETER
☑	2030	642.60	1924.78	1924.94	-0.16	1	33	0.28	1	LCNFAGDIYKEPEITR + Carbamidomethyl (C)
☑	1377	583.23	1164.45	1164.57	-0.12	0	32	0.25	1	LENELMATTK + Oxidation (M)
☑	1774	506.92	1517.74	1517.85	-0.10	1	31	0.39	1	LFLGGTKPSKEDVK
☑	666	427.16	852.31	852.45	-0.14	1	31	0.34	1	IMKFGNK + Oxidation (M)
☑	1249	554.25	1106.49	1106.47	0.02	0	30	0.42	1	GTEEEQQMR
☑	1406	591.31	1180.61	1180.66	-0.05	1	30	0.39	1	SQRLDQLPPK
☑	1577	647.28	1292.55	1292.59	-0.04	0	30	0.48	1	MEEEVLAMWR
☑	1402	590.25	1178.49	1178.57	-0.07	1	30	0.51	1	YEEQLDRAR
☑	634	421.71	841.40	841.47	-0.06	0	29	0.49	1	VAAVETPR

☑	1916	877.31	1752.61	1752.89	-0.28	1	28	0.7	1	EKLAYVAEDFQAELK
☑	1720	727.77	1453.53	1453.70	-0.18	0	28	0.83	1	DVNVQPEGDSLPGK
☑	873	476.20	950.38	950.48	-0.10	1	27	1.2	1	LYEENKR
☑	159	587.92	1760.73	1760.84	-0.11	0	27	4.3	1	EHTPGINFDQYEAIK
☑	1659	691.24	1380.47	1380.70	-0.23	0	26	1	1	GIQLYVDMLGEK + Oxidation (M)
☑	1511	621.20	1240.38	1240.64	-0.26	0	26	1.2	1	LDGYSLMIVSK + Oxidation (M)
☑	1684	705.34	1408.66	1408.78	-0.12	0	26	1.5	1	HLVALAPDGIFTR
☑	1731	490.55	1468.61	1468.80	-0.19	0	26	1.4	1	AFLDQKPEGVPLR
☑	477	383.18	764.34	764.39	-0.05	2	25	1.4	1	MKMDKI
☑	1475	611.34	1220.66	1220.59	0.08	0	25	1.6	1	ENHISFLSMK + Oxidation (M)
☑	1845	818.83	1635.65	1635.81	-0.16	1	25	1.5	1	FAKVDADNNTDIVSK
☑	1123	527.29	1052.57	1052.54	0.03	0	25	1.5	1	LVIMSATMR + 2 Oxidation (M)
☑	1735	736.81	1471.60	1471.72	-0.12	0	24	2	1	ASGPTAVDGTAPEATK
☑	456	379.16	756.31	756.45	-0.14	0	24	1.6	1	LDAVIAR
☑	1141	530.21	1058.40	1058.47	-0.06	0	24	2.5	1	YYDIDQSR
☑	1600	654.28	1306.54	1306.67	-0.13	2	24	2.2	1	KIMEAGSWKNK + Oxidation (M)
☑	1649	686.25	1370.49	1370.73	-0.24	0	24	2.1	1	NIDIEIDELVAK
☑	999	500.26	998.51	998.60	-0.09	1	24	1.9	1	VLFSLHRK
☑	1789	772.32	1542.63	1542.83	-0.19	0	23	2.3	1	INEVLTQVLTEER
☑	1319	571.22	1140.43	1140.57	-0.13	1	23	2.6	1	SYIESAEKSK
☑	610	831.08	2490.22	2490.32	-0.10	0	23	9.4	1	IEAVYLMSIIPHFISLNMLMR
☑	1210	545.73	1089.45	1089.60	-0.15	1	23	2.7	1	LSMQVKSAAR
☑	1732	491.16	1470.46	1470.67	-0.21	0	23	2.7	1	ECPTRPPGVMGDR + Carbamidomethyl (C)
☑	1401	588.77	1175.52	1175.62	-0.10	1	23	2.9	1	SMAPVRAMSVK
☑	61	514.17	1539.50	1539.89	-0.38	0	23	5.6	1	LVEVTLLADAVELR
☑	1639	679.28	1356.54	1356.72	-0.19	1	23	3	1	NLDDEIRILEK
☑	1589	650.73	1299.44	1299.58	-0.14	1	22	4.1	1	KVEDMQTDYR + Oxidation (M)
☑	1239	551.74	1101.46	1101.60	-0.15	0	22	3.6	1	NTEILQSLGK
☑	1067	515.28	1028.54	1028.65	-0.10	2	21	3.7	1	VLSKSRIAR
☑	1815	793.82	1585.63	1585.82	-0.19	1	21	3.9	1	IEIEEIKNDLENK
☑	185	601.24	1200.46	1200.68	-0.21	2	21	11	1	RELLRDMLR
☑	1795	521.20	1560.58	1560.75	-0.18	0	21	3.9	1	LPPGTQESDEPHVR
☑	1674	695.82	1389.63	1389.69	-0.07	0	21	4.8	1	CAETSLLEAQLR + Carbamidomethyl (C)
☑	117	555.58	1663.73	1663.86	-0.13	1	21	11	1	RRPSASVLSTSSSSSR

☑	1739	738.33	1474.65	1474.77	-0.12	2	20	4.3	1	MEEKKLQEAIEK
☑	559	403.13	804.24	804.47	-0.23	0	20	5.3	1	VIGIFEK
☑	1769	755.36	1508.71	1508.83	-0.12	2	20	4.7	1	LRFWSSALMRVK + Oxidation (M)
☑	161	589.39	1765.15	1764.96	0.18	1	20	17	1	HGGVHLTIRAEFVTTK
☑	1770	755.80	1509.60	1509.78	-0.18	1	20	5.2	1	NQLPNQRNWVNK
☑	167	591.17	590.17	590.27	-0.10	0	20	4.7	1	DASGNK
☑	1290	564.27	1126.52	1126.60	-0.08	1	20	4.9	1	SIDRVPSEPK
☑	1648	685.33	1368.65	1368.78	-0.13	2	19	5.1	1	RSNEKLISVAPR
☑	1622	665.84	1329.66	1329.74	-0.08	1	19	6.4	1	KVLIANNGMAATK
☑	1734	736.77	1471.53	1471.82	-0.29	2	19	6.2	1	LVSDANVVRSTRR
☑	1108	523.70	1045.38	1045.52	-0.13	1	19	6.3	1	DGEVDVTRR
☑	1972	613.23	1836.67	1836.92	-0.24	1	19	5.8	1	LSQAMRAGLDYADALSR
☑	1835	811.75	1621.48	1621.80	-0.32	0	19	6.5	1	EVYEDMQAIIAVNK
☑	1018	505.24	1008.46	1008.54	-0.08	0	19	6	1	LLHDAAWLA
☑	1531	629.83	1257.65	1257.70	-0.05	1	19	6.6	1	TVRDVVTALER
☑	1207	545.20	1088.38	1088.59	-0.20	0	19	7.1	1	TGLADWSLVK
☑	1672	695.33	1388.65	1388.79	-0.14	2	19	6.2	1	RSLLTSAPFNKR
☑	1781	765.25	1528.48	1528.79	-0.31	0	19	7.2	1	SMESLPQSVTVVPR
☑	172	594.25	1186.48	1186.63	-0.15	0	19	17	1	QQIQTLSGGQK
☑	1218	547.67	1093.34	1093.46	-0.12	0	19	6.6	1	CTLDEEEAK + Carbamidomethyl (C)
☑	1904	572.91	1715.70	1715.90	-0.21	2	18	7	1	QLQSAQRSLSEARSR
☑	1566	643.77	1285.54	1285.65	-0.12	0	18	7.3	1	LQEEQEILER
☑	1660	691.27	1380.52	1380.67	-0.15	1	18	6.5	1	ARSQLTGSMTEGK + Oxidation (M)
☑	2336	852.62	2554.84	2555.09	-0.25	0	18	9.3	1	TCVSASHQEQQEEEQQQPQER + Carbamidomethyl (C)
☑	1733	736.26	1470.51	1470.78	-0.28	1	18	7.3	1	RPVINMTQRGQR + Oxidation (M)
☑	137	571.18	1140.35	1140.48	-0.12	0	18	19	1	DFDTVCAEGK + Carbamidomethyl (C)
☑	1644	683.28	1364.54	1364.62	-0.08	0	18	7.1	1	DFLMLPCEGANR
☑	817	465.71	929.40	929.52	-0.11	0	18	10	1	ITVIDQNK
☑	1749	743.85	1485.68	1485.70	-0.02	0	18	7.6	1	AAYDGLSGLYDDVK
☑	1156	533.26	1064.50	1064.60	-0.09	1	18	7.5	1	HSRGTVRPR
☑	69	519.23	1036.44	1036.52	-0.08	1	18	20	1	GKDQYQSIV
☑	1929	589.60	1765.77	1766.01	-0.24	1	18	8	1	EVVLSIKHVPQNLYK
☑	1721	731.82	1461.62	1461.72	-0.11	1	17	9.2	1	TIPANGKFSEDQR
☑	432	738.35	737.34	737.36	-0.02	0	17	6.3	1	DFTEVK

☑	1522	627.32	1252.62	1252.73	-0.10	1	17	9.8	1	RTPILQQIER
☑	342	684.28	683.27	683.42	-0.15	0	17	6.6	1	LEVVPK
☑	1903	858.84	1715.67	1715.91	-0.24	2	17	9.3	1	AAALSWLRMTPAEKR + Oxidation (M)
☑	1852	824.78	1647.55	1647.92	-0.37	1	17	10	1	LRIENADLLHWLR
☑	954	490.71	979.40	979.52	-0.11	0	17	9.1	1	MLVESVFR
☑	1275	561.26	1120.50	1120.58	-0.08	0	17	9.6	1	EQEQYLLAK
☑	51	504.17	1509.47	1509.72	-0.24	0	17	21	1	MSSIGLSVLQGCDK + Carbamidomethyl (C); Oxidation (M)
☑	1970	612.90	1835.67	1835.98	-0.31	2	17	9.8	1	LAKHQGGARELIEQMR
☑	1083	518.71	1035.40	1035.49	-0.09	0	17	9.8	1	LNMTVADEK + Oxidation (M)
☑	581	814.27	813.27	813.48	-0.22	1	17	35	1	VVVDRAR
☑	516	788.36	2362.07	2362.09	-0.02	1	17	36	1	FLKSDFSGQPGFDYNDTTPAR
☑	1254	555.20	1108.38	1108.50	-0.12	1	16	9.7	1	DAADCSRMLK
☑	452	751.90	1501.79	1501.74	0.06	0	16	48	1	ATTSTAGPGEASSLPR
☑	605	414.17	826.33	826.39	-0.06	0	16	9.9	1	FAAYLNE
☑	235	624.73	1247.45	1247.65	-0.20	0	16	33	1	NVLFIGESGNAK
☑	343	685.29	684.28	684.38	-0.10	0	16	8.4	1	ELAQPK
☑	2160	715.98	2144.93	2145.04	-0.12	1	16	9.7	1	QHAAHALEELKQDEELER
☑	1104	523.24	1044.47	1044.55	-0.07	0	16	13	1	VTILNENDK
☑	97	542.33	1623.97	1623.80	0.17	1	16	22	1	RATTGEHHSYRPGR
☑	74	523.77	1045.53	1045.54	-0.01	1	16	36	1	EASKVDEIR
☑	1277	561.74	1121.47	1121.55	-0.08	0	16	12	1	ELQNQTYAR
☑	1752	744.77	1487.53	1487.74	-0.21	0	16	12	1	SASVGICSDPILNR + Carbamidomethyl (C)
☑	321	673.31	1344.61	1344.74	-0.13	2	16	42	1	GKKESQEAISIR
☑	1586	650.16	1298.31	1298.69	-0.38	0	16	17	1	SPVASGAAAAAALSR
☑	2219	1126.22	2250.43	2250.02	0.41	1	16	14	1	NEWMSSGQGLKVVDEDGEVR + Oxidation (M)
☑	1680	702.84	1403.67	1403.70	-0.03	1	16	13	1	KYDFVEETVFK
☑	284	650.77	649.76	650.34	-0.58	0	16	25	1	YLEAR
☑	1643	683.26	1364.51	1364.58	-0.07	0	16	13	1	EMFALMSYMDK
☑	2202	1105.98	2209.94	2210.14	-0.20	1	16	12	1	WAENAEGFVSLETLLTFKR
☑	699	873.80	872.79	872.50	0.30	0	15	47	1	LASLENVK
☑	1314	569.70	1137.38	1137.52	-0.14	1	15	13	1	EEGLEMKMR + Oxidation (M)
☑	111	550.18	1647.51	1647.91	-0.39	1	15	34	1	LKPNASLSPHSDVKR
☑	1767	754.29	1506.56	1506.81	-0.25	2	15	18	1	QGHSGRGSRLTPVR
☑	1027	506.21	1010.41	1010.38	0.03	0	15	11	1	AEAEMCER + Carbamidomethyl (C); Oxidation (M)

☑	1608	658.22	1314.42	1314.64	-0.22	0	15	15	1	AVEALAAEEDAAR
☑	1663	692.28	1382.56	1382.80	-0.24	2	15	14	1	CLTPKKVEIPAK + Carbamidomethyl (C)
☑	849	469.72	937.42	937.40	0.02	0	15	14	1	MLDEMAGR + Oxidation (M)
☑	83	533.91	1598.71	1598.94	-0.22	2	15	31	1	GISVTVLARDLEKAK
☑	514	786.70	2357.09	2357.10	-0.01	0	15	53	1	DNSGQPITNVSPEDGTEAQLSAK
☑	553	803.31	802.31	802.36	-0.06	0	15	56	1	CPLADER
☑	1623	666.24	1330.47	1330.70	-0.23	1	15	16	1	RLMLSEVAQER
☑	2235	761.98	2282.91	2283.17	-0.27	1	15	14	1	NYSKELPEINITHQEDILK
☑	336	677.89	2030.66	2031.07	-0.41	1	15	76	1	LIPREAMGMTLLVTDVEK + Oxidation (M)
☑	529	794.30	2379.88	2380.19	-0.31	1	15	49	1	NSLRMAHIGPQISEITNNQNK + Oxidation (M)
☑	37	488.75	975.49	975.52	-0.02	1	15	60	1	RDGVSVCIK
☑	1432	598.26	1194.51	1194.60	-0.09	0	15	15	1	IVAFENAFER
☑	138	571.23	570.22	570.34	-0.12	0	15	41	1	VVEPK
☑	2233	761.71	2282.12	2282.26	-0.14	1	14	15	1	SLLKAISITECYDFVLNLLK
☑	657	850.41	849.40	849.40	-0.00	0	14	53	1	QSAMIER + Oxidation (M)
☑	100	545.13	1632.36	1632.93	-0.57	1	14	69	1	FIQTIAAVASSRLTR
☑	883	319.17	954.47	954.59	-0.11	0	14	14	1	GEVVALVLR
☑	1617	663.27	1324.54	1324.68	-0.15	2	14	20	1	KSCVSLAAKSMK + Carbamidomethyl (C); Oxidation (M)
☑	565	807.79	1613.57	1613.91	-0.35	2	14	71	1	KITNNEVIEIKWK
☑	32	480.01	1437.01	1436.62	0.38	1	14	65	1	DRGNQTMNNCLR + Oxidation (M)
☑	257	636.99	635.98	636.26	-0.29	0	14	54	1	GSCAGSR
☑	1612	660.23	1318.44	1318.63	-0.18	0	14	21	1	LDPMMENTLQK
☑	110	549.78	1646.32	1645.81	0.51	1	14	73	1	AVGVCDDNTVAELRGK
☑	740	893.68	892.67	892.40	0.27	1	14	72	1	QNSSRQSS
☑	1757	747.31	1492.60	1492.67	-0.07	1	14	20	1	MFGSGMKTMTGSVK + 2 Oxidation (M)
☑	1966	612.29	1833.84	1833.97	-0.13	1	14	20	1	ISNILSQKSEGTTLSEK
☑	1561	643.22	1284.43	1284.60	-0.17	1	14	22	1	EISCVRCSFK + 2 Carbamidomethyl (C)
☑	1758	498.55	1492.63	1492.76	-0.13	1	14	21	1	TNSIAAETGMIKNK + Oxidation (M)
☑	261	638.63	1275.25	1275.65	-0.40	0	14	76	1	SGNTCTPLVLSK + Carbamidomethyl (C)
☑	1453	605.68	1209.34	1209.64	-0.31	1	13	20	1	VRARPNGNGNR
☑	1534	630.78	1259.54	1259.62	-0.08	1	13	27	1	RAPSASSSSSPAR
☑	2236	762.06	2283.16	2283.17	-0.01	1	13	21	1	NYSKELPEINITHQEDILK
☑	2016	636.94	1907.80	1907.86	-0.07	1	13	22	1	QEDGPRYCGVTTLNPCR
☑	310	663.39	662.39	662.34	0.04	0	13	31	1	LCATQK

☑	1481	612.85	1223.68	1223.58	0.10	1	13	24	1	CIVRMLECK + 2 Carbamidomethyl (C); Oxidation (M)
☑	1961	611.29	1830.84	1830.99	-0.15	2	13	23	1	KVAQQTKSGAVASASATAR
☑	98	544.22	1629.64	1629.83	-0.19	1	13	65	1	NVDPMIMQLRELR + Oxidation (M)
☑	1540	633.28	1264.54	1264.66	-0.12	1	13	28	1	LREMFGANSLK
☑	646	423.68	845.34	845.39	-0.04	0	13	35	1	FPTGCHK + Carbamidomethyl (C)
☑	1868	839.78	1677.55	1677.81	-0.26	0	13	27	1	AGAASTNQSAGVSAMLAR + Oxidation (M)
☑	424	731.87	2192.60	2192.00	0.60	2	13	1.4e+02	1	CIKCAIGCAHCLNEKECIK + 2 Carbamidomethyl (C)
☑	950	490.22	978.42	978.52	-0.10	0	13	28	1	FCLLALGDK
☑	163	589.89	1177.76	1177.72	0.04	1	13	1.1e+02	1	LLAGEPVPVRK
☑	301	659.86	1976.55	1976.97	-0.42	0	12	1.5e+02	1	DGILGAIHSATMMSTITAR + 2 Oxidation (M)
☑	31	479.64	1435.90	1435.77	0.13	1	12	61	1	SSVMSLQKTALQK + Oxidation (M)
☑	146	579.70	1157.38	1157.59	-0.21	0	12	74	1	VEEILDDAVR
☑	1021	1010.34	3028.00	3028.57	-0.58	2	12	96	1	CAVYGGVGLLNLYKQCSLVAFTGVQRR + 2 Carbamidomethyl (C)
☑	1339	576.18	1150.34	1150.45	-0.11	0	12	28	1	YTEESYCEK
☑	1692	474.19	1419.56	1419.76	-0.20	1	12	27	1	YHALVITEYRR
☑	120	557.49	1669.44	1668.91	0.53	1	12	91	1	IVDSTIRYFEVTVK
☑	1578	432.20	1293.59	1293.69	-0.10	0	12	28	1	IPCRPLNEQPK
☑	1889	568.49	1702.44	1702.01	0.43	2	12	45	1	LSKISIPSGIRSIGFK
☑	339	682.27	1362.52	1362.76	-0.24	2	12	74	1	QLFEAVKSKSAR
☑	1	284.93	851.75	851.38	0.37	0	12	41	1	TQSNTACK
☑	2158	714.77	2141.28	2141.05	0.23	0	12	29	1	NILLLASNNETYDEEYIK
☑	470	763.98	762.97	763.39	-0.41	0	12	1.1e+02	1	GGQDFLK
☑	305	661.25	1320.48	1320.59	-0.11	0	12	1.1e+02	1	EVSMAEEEELR
☑	2172	1083.49	2164.96	2165.06	-0.09	1	12	28	1	KQSSSYEPSCISFLIHPSR
☑	367	700.88	1399.74	1399.69	0.05	0	12	1.4e+02	1	QLGETPTADEALR
☑	1618	664.05	1326.09	1326.66	-0.57	1	12	45	1	RMTPMSAVAHAR
☑	2127	700.62	2098.83	2099.11	-0.28	1	12	32	1	VLEIWFNGAIPEKADEIR
☑	2073	674.77	2021.30	2021.09	0.21	2	12	32	1	GRQSAVSPTQPKPAASQRR
☑	781	456.61	911.20	911.41	-0.21	0	12	27	1	VDSGEFMK
☑	1796	521.21	1560.60	1560.81	-0.21	2	12	33	1	GVAMAMRSPIGGMKR
☑	1046	510.23	1018.45	1018.52	-0.07	0	12	46	1	AAGIEGGAFAR
☑	1806	787.30	1572.59	1572.80	-0.21	0	12	42	1	EALEALEASSEAVVR
☑	2054	665.31	1992.90	1992.92	-0.02	2	11	30	1	VEDERSSSTKVSGSPEGDK
☑	433	738.82	737.81	738.35	-0.54	0	11	42	1	CFSVQR

☑	1934	886.27	1770.53	1770.97	-0.45	2	11	43	1	VTAENLEKELKELQK
☑	204	610.25	609.24	609.37	-0.13	1	11	15	1	ARVHK
☑	1497	617.76	1233.51	1233.73	-0.22	2	11	34	1	KIFNVVGKAMK
☑	60	513.05	1536.12	1535.67	0.45	0	11	99	1	CISYCGPGGAGHMVK + Carbamidomethyl (C)
☑	81	532.48	1594.42	1593.85	0.57	1	11	1.2e+02	1	EAHLQARLEAIESK
☑	375	705.05	2112.14	2112.05	0.09	2	11	1.3e+02	1	YEAGGHCAILPRNRAEDIK
☑	1573	646.32	1290.63	1290.74	-0.11	2	11	39	1	GLFDLSISRKR
☑	1963	611.95	1832.81	1833.01	-0.20	2	11	35	1	MFSLGRVPLAKVLGCSR
☑	1840	813.35	1624.68	1624.94	-0.26	1	11	38	1	ALSIAVPEKTVEALGK
☑	87	537.08	1072.15	1071.67	0.49	2	11	1.3e+02	1	KEVTNKLIK
☑	233	624.29	623.28	623.27	0.01	0	11	25	1	FGGAVAC
☑	1836	541.61	1621.81	1621.85	-0.04	2	11	35	1	LSKVVKGELMESMR + Oxidation (M)
☑	281	649.86	1297.71	1297.72	-0.01	1	11	1.8e+02	1	IVNSKGMTALHK
☑	2218	751.01	2250.00	2250.02	-0.02	1	11	37	1	NEWMSSGQGLKVVDEDGEVR + Oxidation (M)
☑	1256	1110.11	1109.10	1108.59	0.52	1	11	1.3e+02	1	DSLAKAVYSR
☑	95	540.25	1078.48	1078.65	-0.17	1	11	74	1	ELPPGRLVAK
☑	65	515.17	1028.33	1028.61	-0.28	1	11	93	1	LNVSITRAR
☑	2423	1023.76	3068.26	3068.37	-0.11	1	11	31	1	GVLCLGCSCYFLVEEFCETLTRCPR + 4 Carbamidomethyl (C)
☑	323	673.92	1345.83	1345.67	0.16	1	11	2.1e+02	1	MGGKPSKQEEQK
☑	338	678.33	677.32	677.33	-0.01	0	11	60	1	AQSTSGK
☑	25	468.15	1401.44	1401.76	-0.32	2	11	1e+02	1	EKVKEIVELCR + Carbamidomethyl (C)
☑	34	483.06	1446.16	1446.72	-0.56	2	11	1.3e+02	1	EEDKDEVSGKLAK
☑	467	763.41	1524.81	1524.70	0.11	1	11	1.3e+02	1	LQVEECFDSGSRR
☑	156	584.69	1167.36	1167.51	-0.15	1	11	1e+02	1	CRTDVMGGSSR
☑	2055	998.96	1995.91	1996.10	-0.19	1	11	55	1	GVAKALFDLDIQIEHSIK
☑	366	700.45	2098.33	2098.08	0.25	2	11	1.2e+02	1	SGGAMPFARNFTIADRFLK
☑	847	936.98	1871.95	1871.89	0.06	0	11	1.8e+02	1	WTDMNAALGYVAFLER + Oxidation (M)
☑	195	604.39	1206.76	1206.62	0.14	1	10	1.5e+02	1	NGADKLEAYVK
☑	663	851.95	2552.82	2553.02	-0.20	1	10	2e+02	1	DCNTCTEKTCTSCSSPNAYINK + 3 Carbamidomethyl (C)
☑	952	980.00	979.00	978.43	0.56	0	10	1.8e+02	1	LDDMADATK
☑	719	881.30	880.29	880.44	-0.15	1	10	40	1	NSTFERK
☑	1205	1089.33	2176.64	2177.04	-0.40	1	10	1.6e+02	1	EGVLEWFVDMHLRDYPR + Oxidation (M)
☑	1510	620.53	1239.05	1238.71	0.34	1	10	67	1	TLLSPAAARSPR
☑	1740	738.47	1474.92	1474.69	0.23	0	10	46	1	SIELENIMCNPR + Carbamidomethyl (C)

☑	358	695.32	2082.94	2083.20	-0.27	2	10	1.3e+02	1	IIAPPESGRKAVLIQAHQR
☑	1675	464.41	1390.22	1390.74	-0.52	2	10	98	1	VNVGGTMRHHRK
☑	2101	687.29	2058.84	2058.86	-0.01	1	10	46	1	DHENMCVFMLSVCRMR + Carbamidomethyl (C); 2 Oxidation (M)
☑	320	672.44	2014.30	2014.04	0.26	1	10	1.5e+02	1	TEEGVFATMPRPASRIPR
☑	398	717.47	716.46	716.36	0.10	0	10	1.7e+02	1	NAHTFK
☑	1043	1018.22	1017.21	1017.56	-0.36	2	10	1.8e+02	1	DVMEIKRK
☑	286	650.92	1299.83	1299.71	0.11	2	10	2e+02	1	KRIEDAGEAIAK
☑	250	631.86	630.85	631.30	-0.45	0	10	2.9e+02	1	DLPCK + Carbamidomethyl (C)
☑	521	790.36	789.35	789.42	-0.07	0	10	62	1	SISVDAAK
☑	1759	498.55	1492.63	1492.87	-0.24	1	10	48	1	NDVGAVKALLPIQR
☑	96	542.26	1082.51	1082.66	-0.15	1	10	93	1	IVQFHLKAK
☑	1191	1081.10	1080.10	1080.45	-0.35	0	10	1.7e+02	1	QASACAGWCK + Carbamidomethyl (C)
☑	1719	727.32	1452.63	1452.71	-0.08	0	10	47	1	SIIDMSLDFAWR
☑	112	553.23	1104.44	1104.68	-0.24	2	10	1.1e+02	1	LARKSVYLR
☑	617	835.77	2504.30	2504.10	0.20	1	10	1.5e+02	1	SQLRVAPTGGSDSDTQGEEDNADR
☑	299	659.55	1975.64	1976.00	-0.36	2	10	2.4e+02	1	RDPHGAGGGTAGGGGARALSVR
☑	90	538.78	1613.33	1613.93	-0.60	2	10	1.6e+02	1	STKLIHGGIRYLEK
☑	2226	753.18	2256.51	2257.04	-0.53	1	10	77	1	EYHVGDACFAMASLYRGVPR + Oxidation (M)
☑	771	908.04	907.03	907.47	-0.44	1	10	1.9e+02	1	GHASQPRR
☑	2011	636.10	1905.28	1905.81	-0.53	0	10	55	1	TEQQMNDINLGNENDR + Oxidation (M)
☑	1713	724.30	1446.58	1446.81	-0.23	1	10	61	1	NSLLLHRSPASPR
☑	613	831.87	2492.60	2493.15	-0.55	2	10	2.4e+02	1	GGLDFLKDDENINSQPCMRWR
☑	1768	503.87	1508.57	1508.81	-0.24	0	10	52	1	LFDDGPRPPIVQR
☑	53	505.85	1514.54	1514.77	-0.23	2	10	1.1e+02	1	EERAIQREVEEK
☑	946	489.94	977.86	978.44	-0.58	0	10	70	1	IDNMQSQK + Oxidation (M)
☑	1440	1202.13	2402.25	2402.08	0.17	1	10	2.1e+02	1	NRIPVCWTCSACQGTTSIYDK + Carbamidomethyl (C)
☑	2017	637.24	1908.69	1908.82	-0.13	1	10	51	1	DAASDCGASAGAGNAGGHHRK
☑	2292	802.05	2403.14	2403.24	-0.10	2	10	52	1	VGHGGMTAGAVCENAKTFVVSLKK
☑	471	764.09	763.08	763.39	-0.31	0	10	1.7e+02	1	GGQDFLK
☑	220	618.98	1235.95	1235.57	0.38	1	10	1.7e+02	1	SACRSSAGSTPR + Carbamidomethyl (C)
☑	1120	1050.74	3149.20	3149.31	-0.11	1	9	1.9e+02	1	QSDGACQQCSSGYFLMGGGCYQVSKYPGR + Carbamidomethyl (C); Oxidation (M)
☑	2067	1007.41	2012.80	2013.06	-0.26	2	9	56	1	GAAAHRRNHAAELAAIQEK
☑	1756	747.02	1492.03	1491.80	0.23	2	9	54	1	LKHMVEDKIHSR
☑	2176	724.70	2171.09	2171.20	-0.11	2	9	52	1	SSVIMRKPSLQQGTRKPSR + Oxidation (M)

☑	1892	569.19	1704.54	1704.88	-0.34	2	9	62	1	KMAGRAMLFAGPPGTGK + Oxidation (M)
☑	530	794.48	1586.94	1586.82	0.12	1	9	1.7e+02	1	VLSGGTMGSPRAVSPR + Oxidation (M)
☑	1877	843.88	1685.74	1685.90	-0.16	0	9	53	1	IYGDNLPAELIEIAR
☑	91	539.17	1076.33	1076.57	-0.25	1	9	1.4e+02	1	TVQTRYGPR
☑	50	503.23	1506.68	1506.74	-0.06	1	9	1.5e+02	1	RPGFSSALRDLSSD
☑	1798	522.28	1563.83	1563.79	0.04	2	9	53	1	NKEYVPLSSDEKR
☑	2188	727.31	2178.92	2179.15	-0.23	2	9	58	1	NSVNKGSKASTVLAAGAHTPNR
☑	237	625.35	1248.69	1248.70	-0.01	0	9	1.4e+02	1	ADLIAYLETLK
☑	309	663.25	662.24	662.30	-0.06	0	9	77	1	TEGNSR
☑	461	758.85	757.85	758.41	-0.57	0	9	1.2e+02	1	LLPCGTR
☑	1400	1176.10	1175.09	1174.66	0.43	1	9	2.4e+02	1	RPPFDRIFK
☑	1980	925.36	1848.70	1848.82	-0.12	2	9	57	1	CMYIFAGKCGGRNSNR + Carbamidomethyl (C); Oxidation (M)
☑	1012	1007.27	1006.27	1006.44	-0.17	0	9	2.1e+02	1	EWSCVPMR
☑	1661	461.21	1380.60	1380.66	-0.06	1	9	52	1	GESNAEKTITCTK
☑	561	806.57	1611.12	1610.79	0.33	0	9	2.3e+02	1	LELVEGFYDNSGIR
☑	1148	1062.31	1061.30	1061.54	-0.24	0	9	67	1	GDMLLGEALK + Oxidation (M)
☑	2378	919.28	2754.83	2754.29	0.54	0	9	74	1	YILASAEAEADSQSAVECTLEELQK + Carbamidomethyl (C)
☑	1555	640.32	1278.62	1278.72	-0.10	0	9	62	1	VLIAASHLHYR
☑	815	929.35	2785.03	2785.44	-0.41	1	9	1.8e+02	1	SRGALVVLGGECEGKPVTSAWACILR + 2 Carbamidomethyl (C)
☑	43	494.22	986.42	986.51	-0.09	0	9	1.4e+02	1	LLDTGNAQR
☑	1040	1016.96	1015.95	1016.51	-0.56	2	9	2.5e+02	1	ERRGQESR
☑	1593	651.57	1301.12	1301.68	-0.56	0	9	1.3e+02	1	IVPLFVSCDPR + Carbamidomethyl (C)
☑	326	675.25	2022.72	2023.14	-0.42	1	9	1.9e+02	1	NNVIVVNGQKILCVQAVR + Carbamidomethyl (C)
☑	2039	650.94	1949.80	1949.87	-0.07	1	9	57	1	LTDGEVNDMNDSKNINR + Oxidation (M)
☑	1909	870.39	1738.76	1738.86	-0.10	1	9	63	1	TVSFLGLCNCGRSLR + 2 Carbamidomethyl (C)
☑	1743	740.83	1479.65	1479.54	0.11	0	9	69	1	EGNCNGDDDIDEK + Carbamidomethyl (C)
☑	132	567.02	1698.04	1697.79	0.24	0	9	2.1e+02	1	DLEMAYNLAVCLMR + Carbamidomethyl (C)
☑	316	669.74	1337.46	1337.66	-0.20	0	9	1.6e+02	1	NLFGSSAPDGVFK
☑	795	920.71	919.70	919.51	0.19	2	9	2.2e+02	1	MMLKRNK
☑	1712	722.33	1442.65	1442.92	-0.27	1	9	60	1	VLLGIIIGQKVYK
☑	176	596.89	595.88	596.28	-0.39	0	9	1.6e+02	1	GNHGGR
☑	1866	558.34	1671.99	1671.89	0.09	1	9	63	1	RYPIDASVDLPSIAR
☑	2089	683.29	2046.84	2047.01	-0.17	1	9	59	1	LECPDCHLGLGYLFVRR + Carbamidomethyl (C)
☑	1906	576.27	1725.78	1725.80	-0.02	0	9	59	1	ACATSVVVCDVGTSTTR + Carbamidomethyl (C)

☑	275	647.33	1292.65	1292.71	-0.06	0	9	2e+02	1	IIVVQPNPDGNK
☑	536	796.46	795.45	795.44	0.02	0	9	1.8e+02	1	QVSVHAR
☑	1865	836.88	1671.75	1671.90	-0.15	0	9	64	1	SAVIAVPMGSGSGASLIR
☑	131	566.36	1130.70	1130.59	0.10	0	9	1.4e+02	1	ARPGAAAYAGAR
☑	1460	607.47	1212.93	1212.65	0.28	0	9	66	1	AEAAGAPSATVLR
☑	191	602.58	1203.15	1203.62	-0.47	0	9	2.9e+02	1	SALPDGSLLTCK
☑	416	728.95	2183.83	2184.15	-0.32	2	9	2.4e+02	1	LQTIASKCLLHERAEMIR + Carbamidomethyl (C); Oxidation (M)
☑	2187	1090.46	2178.91	2178.94	-0.03	0	9	66	1	NSISSETSDAGGIQNNSHSMK + Oxidation (M)
☑	1420	594.78	1187.55	1187.69	-0.14	2	9	70	1	EIISKTKQNK
☑	1803	522.95	1565.83	1565.77	0.06	1	9	65	1	RVLSCVPLYCDGK + 2 Carbamidomethyl (C)
☑	296	656.51	655.50	655.29	0.21	0	9	2.3e+02	1	DDGPPR
☑	29	475.40	1423.17	1422.80	0.37	0	9	1.9e+02	1	SLIYHLHSAGVVK
☑	1645	683.38	1364.74	1364.66	0.08	0	9	63	1	CESSSLVESLVGR
☑	2298	807.69	2420.05	2420.19	-0.14	0	9	55	1	HHATACAFLSTPSLYFTLVGER
☑	1702	717.84	1433.67	1433.73	-0.06	0	9	64	1	LNKPNINEAFMK + Oxidation (M)
☑	777	909.99	908.98	909.56	-0.58	0	9	1.1e+02	1	VNAIPIGVK
☑	1405	591.25	1180.48	1180.63	-0.15	1	9	60	1	LGGPEVRSNPR
☑	1755	497.88	1490.62	1490.76	-0.14	2	9	89	1	ALSRACGRASALCR + Carbamidomethyl (C)
☑	178	597.75	1790.22	1789.77	0.45	0	9	1.9e+02	1	AANDASPTTGNSDNAAGEK
☑	1828	537.55	1609.63	1609.90	-0.27	1	9	64	1	TKQPAPPPMFIVIR + Oxidation (M)
☑	1876	562.67	1685.00	1684.95	0.05	1	9	67	1	EQSTELWLLKGILR
☑	2077	676.23	2025.65	2025.10	0.56	2	8	78	1	DITVKFFSEKPCKLSGVK
☑	1683	704.72	1407.42	1407.63	-0.21	0	8	65	1	CVGGMGFLDPPSR + Carbamidomethyl (C); Oxidation (M)
☑	355	692.43	2074.27	2073.98	0.29	0	8	2.1e+02	1	DLGADPLNPTYCNLPSTAGR
☑	207	613.64	1837.89	1837.84	0.05	0	8	1.7e+02	1	YVENWLTEIEHMMK + Oxidation (M)
☑	1500	618.60	1235.18	1234.63	0.54	1	8	1.3e+02	1	RAPYDLFEPK
☑	869	948.13	2841.36	2841.43	-0.07	2	8	2.2e+02	1	AANAGKGYGQIAAEMGYVNLCTKGLVAR + Oxidation (M)
☑	1971	918.88	1835.74	1835.73	0.01	1	8	69	1	THSSSRSPDCCTGDGGR + 2 Carbamidomethyl (C)
☑	295	655.09	1962.24	1962.83	-0.59	1	8	2.4e+02	1	TCWCCGNGGSKNFVFAR + 2 Carbamidomethyl (C)
☑	182	599.99	1197.96	1197.70	0.26	0	8	2e+02	1	EVTPNATIILK
☑	814	929.08	928.07	927.54	0.53	0	8	2.5e+02	1	ALLDQLQK
☑	194	604.31	1206.61	1206.82	-0.21	2	8	2.4e+02	1	RIPILKNLLK
☑	177	597.37	1192.72	1192.54	0.18	0	8	1.8e+02	1	LSDCVLMPCR + Carbamidomethyl (C)
☑	267	641.67	1922.00	1921.98	0.02	2	8	1.8e+02	1	FYEGTGIKFAPFSFRR

☑	730	889.32	888.32	888.45	-0.13	1	8	2.6e+02	1	RSMPAEAK
☑	1969	918.83	1835.64	1835.73	-0.09	1	8	74	1	THSSSRSPDCCTGDGGR + 2 Carbamidomethyl (C)
☑	405	720.18	719.17	719.35	-0.18	0	8	80	1	CEEVIK
☑	303	660.64	1319.27	1319.73	-0.46	0	8	3.1e+02	1	YITPSTDLVALK
☑	1862	833.83	1665.65	1665.86	-0.21	1	8	69	1	LLIPYEAKMQMTGR + Oxidation (M)
☑	335	677.87	1353.73	1353.69	0.04	1	8	3.7e+02	1	GGQTREHAMLVR
☑	1058	1025.72	2049.43	2049.25	0.18	2	8	2.6e+02	1	NLATLVRRAILQLQALTR
☑	2350	877.72	2630.15	2630.42	-0.27	2	8	59	1	TFYRFFLVREEQTVILATELR
☑	88	537.71	1610.12	1609.93	0.19	2	8	1.8e+02	1	KPIAKKYYTVPFR
☑	1376	583.09	1164.17	1163.58	0.59	0	8	1.4e+02	1	LVLYVDDDGR
☑	149	580.67	1739.00	1738.96	0.04	2	8	2e+02	1	SRLFSELFKLSIGDK
☑	2027	641.02	1920.03	1920.07	-0.04	2	8	69	1	GLISPKLINFKVFDDSK
☑	1928	883.81	1765.61	1765.86	-0.25	0	8	76	1	TFLGATTGVHTFNDGTK
☑	1441	601.78	1201.55	1201.71	-0.16	1	8	95	1	IKEQIMIISK
☑	1817	531.23	1590.66	1590.73	-0.07	1	8	82	1	AEIEQTKADNADMR
☑	135	569.99	1706.95	1706.91	0.04	1	8	1.9e+02	1	NEDVILKCLWTVFK
☑	198	607.35	1819.04	1818.87	0.17	1	8	2.2e+02	1	CRFSGNLDAPVHGHPR + Carbamidomethyl (C)
☑	1681	702.96	1403.91	1403.67	0.24	0	8	77	1	MGAGLSQQPTPSSK + Oxidation (M)
☑	38	488.85	1463.54	1463.78	-0.24	2	8	2e+02	1	KNGTVVSTGGASKMK
☑	125	562.89	561.88	561.29	0.59	0	8	90	1	AVMNK
☑	878	477.07	952.12	952.50	-0.38	1	8	96	1	NGHWVRGK
☑	1562	643.32	1284.62	1284.75	-0.14	1	8	83	1	RLLSLLNSNQK
☑	127	564.99	563.98	564.27	-0.29	0	8	2.4e+02	1	SLSCR
☑	315	669.21	1336.40	1336.68	-0.28	1	8	2.4e+02	1	RGAAFFFSAAHR
☑	1650	686.79	1371.56	1371.61	-0.05	0	8	83	1	WEEIANSGGQPAN
☑	1253	555.16	1108.31	1108.59	-0.28	0	8	73	1	LFDDAFIIR
☑	39	489.33	976.65	976.45	0.20	0	8	2.2e+02	1	NEDASSLNK
☑	340	683.01	1364.00	1363.75	0.25	0	8	2.1e+02	1	TLDVYPVVPPHK
☑	58	512.51	1023.00	1022.45	0.55	0	8	2.3e+02	1	GVQNMSEMK
☑	114	554.00	1105.98	1106.58	-0.60	0	8	2.3e+02	1	IINFDQMVK
☑	324	674.59	1347.17	1346.81	0.36	2	8	3.5e+02	1	MIRLIFKDIAK
☑	2287	795.27	2382.78	2383.18	-0.40	1	8	1.1e+02	1	TTEATDLTLSYVRLLGCSGGGGGR
☑	106	546.55	1091.09	1090.55	0.55	1	8	3e+02	1	SRNSSGQLSR
☑	1741	1476.64	4426.89	4426.31	0.58	2	8	1.9e+02	1	LHIYAVKEEIPNLIIDFDIPQNIEIQKYIQLNDEMSK

☑	735	892.06	1782.10	1781.88	0.22	1	8	3e+02	1	EGRHVTEIMNEALQR
☑	1232	1100.90	2199.79	2200.18	-0.38	2	8	2.3e+02	1	FQFLTLISSVQKEEEFKK
☑	1438	1201.10	2400.19	2400.36	-0.17	0	8	3.3e+02	1	YYIGLLIGLAFGTAGFLVPFIR
☑	453	753.30	1504.59	1504.73	-0.13	1	8	2.3e+02	1	ELIPEEELCRMK + Oxidation (M)
☑	79	527.43	1579.26	1578.85	0.40	1	8	2.3e+02	1	AFAPLTVARTQYNK
☑	1580	648.15	1294.29	1294.60	-0.31	1	8	1.1e+02	1	GQSEIKFDDEK
☑	2024	640.66	1918.95	1918.95	-0.00	0	8	75	1	IDPWSCGIILIEMTQK + Carbamidomethyl (C); Oxidation (M)
☑	2303	815.68	2444.02	2444.20	-0.17	2	8	70	1	YKDFIEHQIFRICNSMSAVK + Oxidation (M)
☑	914	969.51	1937.02	1936.88	0.14	1	7	2.3e+02	1	DEKGGVDGAWAVAQDYEK
☑	179	598.37	597.36	597.30	0.07	0	7	24	1	GATHGR
☑	201	608.57	1215.13	1214.69	0.45	2	7	3.6e+02	1	EIEELQKKAK
☑	919	486.21	970.40	970.57	-0.17	0	7	81	1	EVVLPSSLK
☑	616	834.15	2499.42	2499.16	0.26	2	7	3.1e+02	1	YSRVINSDSNGDADMGMRALATR
☑	2222	751.97	2252.90	2252.98	-0.08	2	7	86	1	SHHCKTCGVCVAFKDHHCK + 2 Carbamidomethyl (C)
☑	563	807.40	2419.17	2419.38	-0.21	2	7	3e+02	1	INDILPVNIFIHSADKIKELK
☑	1517	1247.08	3738.23	3737.89	0.34	2	7	3.2e+02	1	VNLNKLVDELLSQNESSDCALKTISSFLFYYK + Carbamidomethyl (C)
☑	171	593.78	592.77	592.25	0.52	0	7	3.6e+02	1	DVCEK
☑	552	802.73	1603.44	1603.73	-0.29	1	7	3.1e+02	1	VHHCSTSNKCIYR + Carbamidomethyl (C)
☑	245	629.99	1257.96	1257.53	0.43	0	7	3.8e+02	1	EECHVNDEVK + Carbamidomethyl (C)
☑	656	850.36	849.35	849.33	0.02	0	7	92	1	ECEGEQR
☑	2282	793.26	2376.75	2377.24	-0.49	2	7	1.2e+02	1	SRAPLFEKAHRPHVFTAEER
☑	1075	1033.07	1032.06	1032.53	-0.47	0	7	4.1e+02	1	SVLLCGADAK + Carbamidomethyl (C)
☑	1605	437.51	1309.52	1309.67	-0.15	2	7	79	1	APPMPRAPSRCK
☑	1930	590.28	1767.81	1767.85	-0.04	2	7	84	1	MRESEEYIEIGKER
☑	101	545.17	1632.49	1632.88	-0.39	0	7	2.8e+02	1	DSISADHLLLIHTAK
☑	696	873.06	872.05	872.44	-0.38	1	7	2.2e+02	1	RVENELN
☑	1960	913.43	1824.84	1825.02	-0.18	2	7	80	1	RLGPLSGLTQNGESIRK
☑	1689	472.93	1415.78	1415.65	0.13	1	7	89	1	TQHEQMERETK
☑	893	959.41	958.40	958.56	-0.15	2	7	2.6e+02	1	IKTERGQK
☑	1656	689.27	1376.53	1376.59	-0.05	0	7	93	1	SDCTPPELWACR
☑	1936	888.76	1775.51	1775.76	-0.25	1	7	1.4e+02	1	FLDNSSSDEDNYKSR
☑	2068	672.21	2013.61	2013.07	0.54	2	7	1.3e+02	1	DKLLKELEALQDVGETGR
☑	2171	1083.45	2164.89	2165.12	-0.23	1	7	88	1	DDITNGYKWLLLAYIPDR
☑	1113	1048.48	1047.48	1047.55	-0.07	0	7	2.7e+02	1	QAQLYVNGR

☑	1344	576.48	1150.95	1150.60	0.35	1	7	1.3e+02	1	YLAEAVRDSK
☑	1827	537.25	1608.72	1608.85	-0.13	0	7	84	1	VYNVVIINGSPYSGK
☑	226	621.43	1240.84	1240.71	0.13	1	7	2.8e+02	1	LVAPAASLSEKR
☑	328	676.52	1351.03	1350.85	0.18	0	7	4e+02	1	ALEVVIGLPTVIK
☑	73	523.28	1566.82	1566.75	0.07	1	7	2.1e+02	1	GGSGVHAAAGAAGDGEKR
☑	292	654.23	1306.44	1306.67	-0.23	1	7	2.6e+02	1	LMLEGFQRGEK
☑	2379	922.63	2764.88	2765.27	-0.38	2	7	1.3e+02	1	ERPSFGTKACRSTADEDDVSAEPAAR
☑	770	907.03	1812.05	1811.83	0.21	0	7	3.8e+02	1	FAEAVLNFCSPNCIER
☑	341	683.21	2046.60	2046.09	0.51	1	7	2.9e+02	1	LAIGLVEEMSADLGGFLRR
☑	359	697.33	2088.95	2088.92	0.03	1	7	2.3e+02	1	DVSSLTMADGRMYSDVGNR + Oxidation (M)
☑	241	626.86	1877.55	1877.91	-0.35	2	7	3e+02	1	SSIGGDGSDRLCWREIK
☑	1984	619.99	1856.95	1856.91	0.04	1	7	1e+02	1	ICEQVNSPDLRLDNGK + Carbamidomethyl (C)
☑	2476	1132.46	3394.36	3394.61	-0.25	1	7	80	1	FEWSCSSSILQCTPQLGHMSPGSTKQLTLR + Carbamidomethyl (C); Oxidation (M)
☑	1997	628.89	1883.66	1883.82	-0.16	1	7	1e+02	1	AEDDKESHSLYVAGNSY
☑	118	557.04	1668.10	1667.77	0.32	1	7	3.4e+02	1	SNTSAHNRSHQHHR
☑	369	701.28	700.27	700.34	-0.07	0	7	1.2e+02	1	IPDDNK
☑	992	996.59	2986.75	2986.44	0.31	2	7	2.6e+02	1	ERAQSFFFFFCAVWSLSQLSMRQR + Oxidation (M)
☑	1157	533.40	1064.79	1064.50	0.29	1	7	95	1	FRSMHLCR + Oxidation (M)
☑	123	559.97	1676.90	1676.95	-0.05	1	7	3.1e+02	1	TLREVILYNTNITK
☑	1703	719.13	1436.24	1435.77	0.47	1	7	1.9e+02	1	ASKDQAYAIISAAK
☑	457	757.46	1512.91	1512.73	0.18	1	7	2.8e+02	1	SQNSSFTPRAFGSK
☑	2220	751.38	2251.13	2251.00	0.13	2	7	87	1	MRRGEDGVSTPGVDAASGADGCK + Oxidation (M)
☑	113	553.25	552.24	552.36	-0.12	0	7	18	1	IPVPK
☑	494	772.67	1543.33	1543.73	-0.40	0	7	4e+02	1	GNVNDNMADITGPVK
☑	1525	628.49	1254.97	1254.61	0.36	2	7	1.1e+02	1	ETMLSSCKGRK + Oxidation (M)
☑	1213	1092.14	3273.40	3273.58	-0.19	2	7	3.6e+02	1	DGGVSRREGGIVEGCAAQAVPGLGACGHVGPQGR + Carbamidomethyl (C)
☑	979	495.20	988.38	988.55	-0.17	1	7	1.1e+02	1	ITAKAINCR
☑	52	505.33	1512.96	1512.74	0.21	1	7	2.3e+02	1	AEEPTEEQPLSKR
☑	1707	720.89	1439.76	1439.79	-0.03	1	7	99	1	KHLVLDMSLVNR + Oxidation (M)
☑	1549	637.89	1273.76	1273.70	0.06	2	7	1.1e+02	1	QQISSAEKQKK
☑	1879	564.64	1690.89	1690.85	0.04	0	7	99	1	NGGSLAQVEALTEQFK
☑	2014	636.37	1906.09	1905.92	0.17	0	7	1e+02	1	ATSAVSITAGATSCAQPASR + Carbamidomethyl (C)
☑	1898	571.61	1711.81	1712.02	-0.21	2	7	99	1	EKKQTQIIGLSIINK
☑	542	399.24	796.47	796.44	0.02	0	7	89	1	TVPEVPR

☑	48	498.23	1491.67	1491.78	-0.11	1	7	2e+02	1	MTVDIARLIEMGK + Oxidation (M)
☑	472	764.13	2289.38	2288.99	0.39	1	7	3.8e+02	1	VSNNTQGSCNYQNSGRGAGYR + Carbamidomethyl (C)
☑	507	781.02	2340.05	2340.28	-0.23	2	7	3.4e+02	1	NINNALDVLHSRNIIHRDVK
☑	2102	687.40	2059.17	2058.99	0.18	2	7	1.1e+02	1	MPRGAGAGCDQDVCRVVIGR
☑	1252	1109.21	3324.60	3324.55	0.04	0	7	3e+02	1	TCLMMTCVGSLVTCAAFYVQPLQCFSPLGGK + Carbamidomethyl (C)
☑	1780	764.79	1527.57	1527.81	-0.24	0	7	1.1e+02	1	APLCVVSGLQEVTR + Carbamidomethyl (C)
☑	428	734.43	2200.26	2200.30	-0.04	2	6	3.3e+02	1	IKLRPTPPLHPISARSYVR
☑	15	439.91	1316.70	1316.68	0.03	0	6	2.9e+02	1	EVPPFLAPEYR
☑	139	571.61	1141.20	1141.69	-0.50	1	6	2.9e+02	1	LVSQRTLGLR
☑	352	689.25	688.24	688.30	-0.05	0	6	1.7e+02	1	ANPECR
☑	751	901.90	900.89	900.48	0.41	1	6	2.1e+02	1	TQSGPAKGR
☑	1097	1040.28	3117.83	3117.45	0.38	1	6	3.9e+02	1	GCCVIHVTAPPGRNICWHPQLGGEATCK + 3 Carbamidomethyl (C)
☑	1492	1231.13	3690.37	3690.67	-0.29	2	6	4.3e+02	1	LQVCTDDSSDDGEEDDGDDLEKEISRLVPAAVAK + Carbamidomethyl (C)
☑	1321	1142.43	3424.26	3424.54	-0.28	0	6	3.4e+02	1	SVTLPGTSGDTCWHLCEQHVGGVEQTQGQEGK + Carbamidomethyl (C)
☑	1493	1231.56	3691.66	3691.73	-0.07	1	6	3e+02	1	DVPEPIPLCTYAICDAEYISQKTYICLTCNQK + Carbamidomethyl (C)
☑	1610	659.24	1316.46	1316.69	-0.23	1	6	1.7e+02	1	NAAVVDIGACKTR
☑	2169	722.32	2163.92	2164.16	-0.23	1	6	99	1	GCLGVLIFNVEPRHLVDAR + Carbamidomethyl (C)
☑	624	839.93	2516.77	2516.29	0.48	1	6	4.7e+02	1	VLSNGGNQNLGGNDFDKVISDIIK
☑	78	526.48	1576.41	1576.78	-0.38	0	6	3.3e+02	1	VVQEAVSGAYEAAQR
☑	327	338.40	674.79	675.38	-0.59	0	6	2.7e+02	1	ISSVIAS
☑	534	796.14	1590.26	1589.86	0.39	2	6	4.1e+02	1	QQPMFLVRLMRR + Oxidation (M)
☑	595	822.65	2464.94	2465.25	-0.31	1	6	4.2e+02	1	SHKVVASVSNFMAVLPFGDAAYR
☑	1894	854.29	1706.56	1706.84	-0.28	2	6	1.2e+02	1	CQDKTSSDKPSLRSR
☑	1607	1314.12	3939.35	3938.84	0.51	2	6	4e+02	1	VVEDGEEEEAASSQQHSTDVGRANDTASPPLPPHRAR
☑	2037	648.59	1942.74	1942.91	-0.17	0	6	1.3e+02	1	MEDQAPTTHNVDVPFVK + Oxidation (M)
☑	872	950.73	1899.45	1899.91	-0.47	1	6	4.1e+02	1	LDSIHAEWDARLAEMK + Oxidation (M)
☑	1524	627.95	1253.89	1253.81	0.09	2	6	1.1e+02	1	STGLPVAIKKLK
☑	150	580.69	1739.05	1738.97	0.07	0	6	3.2e+02	1	LAWVQQQLSAVEVLR
☑	175	595.90	1784.69	1784.90	-0.21	1	6	3e+02	1	KCAPGLTQLHFSSPSR + Carbamidomethyl (C)
☑	2324	837.31	2508.90	2509.30	-0.41	2	6	1.6e+02	1	EIEIKEENKENNIENLPLSVR
☑	693	871.93	870.93	870.50	0.43	0	6	2.3e+02	1	LLNLPACK
☑	1955	606.59	1816.75	1816.87	-0.12	1	6	1.3e+02	1	PANDCVLMDKIIEGDGK
☑	672	857.96	1713.90	1713.99	-0.09	2	6	5.4e+02	1	MKIVLRSIVGSEQVR
☑	435	739.54	738.53	738.31	0.22	0	6	4.1e+02	1	AGCEFR + Carbamidomethyl (C)

☑	1563	643.50	1284.98	1284.75	0.23	1	6	1.4e+02	1	SLVQVVAGTKQR
☑	170	593.76	1778.26	1777.90	0.36	0	6	4.6e+02	1	GPDLETQATAAAAALHNK
☑	1983	928.42	1854.82	1854.95	-0.13	0	6	1.2e+02	1	WTWPPPHHSTLVELR
☑	173	594.96	1187.91	1187.50	0.41	0	6	3e+02	1	YYDEIENDK
☑	478	765.82	764.81	765.31	-0.50	0	6	4.5e+02	1	ACNCDLK
☑	497	774.49	2320.45	2320.14	0.32	2	6	4.1e+02	1	IGENMIDLHNYLKYDGGRGR
☑	9	412.74	823.46	823.46	0.01	0	6	3e+02	1	LSPPASPR
☑	1162	1066.70	3197.09	3197.46	-0.38	2	6	3.9e+02	1	DRSIMAGDGSEAPEVASTNSGDPLIEHDRR + Oxidation (M)
☑	1811	528.09	1581.23	1580.92	0.31	2	6	2.2e+02	1	VDPRLKTLIENGVK
☑	302	660.31	1977.92	1978.01	-0.09	0	6	4.2e+02	1	QPSVIFIDEIDSLLSMR + Oxidation (M)
☑	337	677.97	2030.90	2031.10	-0.20	2	6	4.7e+02	1	TLLTMDPSKLLGNETVRK + Oxidation (M)
☑	1426	596.26	1190.50	1190.63	-0.13	1	6	1.3e+02	1	ADKGGFQDLLK
☑	884	956.51	955.51	955.56	-0.05	1	6	3.4e+02	1	DALGVARVR
☑	165	590.90	1769.69	1769.84	-0.15	0	6	4.7e+02	1	GFIMSDGTNSVNMLLR + Oxidation (M)
☑	278	648.38	647.38	647.34	0.03	1	6	4e+02	1	AKACQK
☑	3	327.62	979.83	979.59	0.23	2	6	2e+02	1	DRIPVPKR
☑	104	546.29	1090.56	1090.52	0.04	0	6	3.4e+02	1	LHAMTEAYR
☑	1465	608.23	1214.45	1214.50	-0.05	0	6	1.4e+02	1	EETTTADSSMK + Oxidation (M)
☑	2203	737.66	2209.97	2210.03	-0.06	1	6	1.1e+02	1	EGCSGNVMKEILGQSELSWK + Oxidation (M)
☑	374	704.96	1407.91	1407.72	0.19	1	6	5e+02	1	KMVNATSIVNGMK + Oxidation (M)
☑	2409	993.64	2977.90	2977.41	0.49	2	6	1.6e+02	1	FCPVTFAECGVLVEGAANYGCVFRRR + 2 Carbamidomethyl (C)
☑	363	699.94	1397.87	1397.72	0.15	0	6	4.2e+02	1	FSNTVFNDTIIK
☑	1991	939.15	1876.29	1875.89	0.40	1	6	1.7e+02	1	SLLSDRGVSCPGAWDGTR
☑	2132	1052.95	2103.89	2103.90	-0.01	2	6	1.1e+02	1	QRNGAEECQGRGGNEAEDK + Carbamidomethyl (C)
☑	365	700.32	2097.94	2098.07	-0.14	2	6	3.6e+02	1	YEGMRFADMRDLLLQLK
☑	793	919.75	2756.23	2756.46	-0.24	2	6	4.5e+02	1	DLEIKIIPDKANGTLSIQDSGIGMTK
☑	1820	798.91	1595.80	1595.85	-0.05	1	6	1.3e+02	1	NGEVEIIPNEINKK
☑	1883	847.27	1692.52	1692.88	-0.36	0	6	1.4e+02	1	FALAVVANGGPAATSYGK
☑	304	660.84	1979.50	1979.89	-0.39	1	6	8.4e+02	1	EQMMVGDTCPGTQRTGAAK
☑	570	810.76	809.75	809.38	0.38	0	6	1.5e+02	1	NPHGNSGK
☑	922	971.93	1941.84	1942.03	-0.19	1	6	4.3e+02	1	LAPHVVDCTRVIHQAAR + Carbamidomethyl (C)
☑	856	942.21	1882.41	1881.84	0.57	0	6	4.5e+02	1	DPATTCVECLPGYMLNR
☑	620	838.01	837.00	836.41	0.60	0	6	4.9e+02	1	TMETSIR
☑	1962	611.92	1832.74	1833.00	-0.26	2	6	1.3e+02	1	YLISLGANINEKGEKGK

☑	512	393.11	784.21	784.38	-0.17	0	6	1.1e+02	1	TAFTMSK
☑	56	508.92	1015.83	1015.57	0.26	1	6	3.3e+02	1	ETNNLLKGK
☑	2431	1038.96	3113.86	3113.61	0.25	2	6	1.2e+02	1	HHLRGCLQAVADRLALYGPCLPAVQEAR + Carbamidomethyl (C)
☑	985	991.40	1980.79	1980.92	-0.14	0	6	4e+02	1	THQSTAPTSAGPSNSAPSQR
☑	2247	770.38	2308.10	2308.23	-0.13	2	6	1.2e+02	1	KVSNEFNIQNIFTPFVKQR
☑	500	776.19	775.19	775.46	-0.27	1	6	1.7e+02	1	SSTVVKR
☑	1348	1153.15	3456.43	3456.50	-0.07	1	5	4.3e+02	1	MEFLNGMPVIDVGSPMLGMHSYRETMCYK + Carbamidomethyl (C); 4 Oxidation (M)
☑	1634	1345.89	4034.65	4034.69	-0.04	1	5	4.2e+02	1	GAISCLSCDSSCLSCDPTNGYCLTCNKGSYLNTQTNK + 3 Carbamidomethyl (C)
☑	1737	737.92	1473.82	1473.82	-0.00	2	5	1.4e+02	1	KLLIQNTEMKEK
☑	1399	588.26	1174.51	1174.62	-0.12	1	5	1.6e+02	1	MLNAQSSRLR
☑	1771	755.83	1509.64	1509.82	-0.18	1	5	1.4e+02	1	KEVPQNLLDDIAR
☑	532	795.49	1588.96	1588.92	0.04	2	5	4.2e+02	1	KKAEEVFNGLLLTK
☑	1294	1129.39	3385.16	3384.75	0.41	0	5	4.6e+02	1	GVAGPLPEGPAAPPEVYMPFRPFVGMPVPPGVR
☑	1403	590.37	1178.73	1178.66	0.07	2	5	1.3e+02	1	AKSLAQMFRK
☑	606	827.89	2480.66	2480.17	0.49	1	5	5.7e+02	1	MMNQYDAEISKQIAEGAEIPAR + Oxidation (M)
☑	218	617.85	1233.69	1233.63	0.06	1	5	7.5e+02	1	KLEEEQSMLK
☑	2026	960.84	1919.66	1919.89	-0.22	0	5	1.4e+02	1	AALIYQNMEAAHMNEAK + Oxidation (M)
☑	2280	792.97	2375.89	2376.16	-0.27	1	5	1.3e+02	1	SLSEISTSLSDKIHNMSGEVAR + Oxidation (M)
☑	1178	537.76	1073.50	1073.60	-0.10	1	5	1.7e+02	1	MTRQLQGLK
☑	143	575.38	574.37	574.33	0.04	1	5	1.7e+02	1	VKTLD
☑	334	677.80	1353.59	1353.65	-0.06	0	5	6.7e+02	1	LEGLLSAHEDDR
☑	1329	574.25	1146.48	1146.70	-0.22	2	5	1.5e+02	1	TTASLLAKSKK
☑	1347	576.94	1151.87	1151.60	0.27	1	5	1.5e+02	1	FACVTKEGLK + Carbamidomethyl (C)
☑	2096	684.95	2051.83	2051.88	-0.04	1	5	1.3e+02	1	DKLGVWNCNEDDNNGFR + Carbamidomethyl (C)
☑	253	634.10	1899.27	1898.97	0.30	2	5	5.8e+02	1	GDIEFKNVWFKYPTR
☑	1766	753.28	1504.54	1504.81	-0.27	1	5	1.4e+02	1	VVSPPASSQQPPRR
☑	1885	848.83	1695.64	1695.86	-0.22	0	5	1.4e+02	1	VLSSTNSLCGYGLGLR + Carbamidomethyl (C)
☑	1700	716.41	1430.80	1430.85	-0.05	1	5	1.5e+02	1	ELTKGLVGLLFNK
☑	1474	1221.31	1220.30	1220.70	-0.40	1	5	4.3e+02	1	LNEHQRLLAK
☑	921	971.93	1941.84	1941.99	-0.15	0	5	4.7e+02	1	FSEVIDLSSSDIVAGYIK
☑	2456	1087.15	3258.44	3258.62	-0.18	2	5	1e+02	1	MPFLQTIVSVSLDDQKRANLSAAYGMICR + 2 Oxidation (M)
☑	344	685.73	684.72	684.39	0.33	0	5	1.2e+02	1	ALPSAAR
☑	1518	1247.11	2492.21	2492.36	-0.15	0	5	5.6e+02	1	MIGEIIIGGIIGWIISYFLPCK + Carbamidomethyl (C)
☑	1530	629.51	1257.01	1256.70	0.31	2	5	1.8e+02	1	LKDVHQKYAR

☑	1546	1272.15	1271.14	1271.54	-0.39	1	5	5.2e+02	1	GGPKGGGGEGGEEGQ
☑	969	984.90	2951.69	2951.43	0.25	1	5	4.4e+02	1	DGASVAAVAAAAAAAELSMLLCCRCAATVAK + Carbamidomethyl (C); Oxidation (M)
☑	2364	900.42	2698.24	2698.43	-0.19	2	5	1.2e+02	1	MIIFFLFFFFNLSLSNKSNNKK
☑	329	676.61	2026.82	2026.89	-0.08	1	5	6.2e+02	1	VVERAAGMGAGGGEGYGQSCR + Oxidation (M)
☑	17	445.24	888.47	888.51	-0.04	2	5	4e+02	1	TKKAAAGSR
☑	2224	752.38	2254.12	2254.11	0.00	2	5	1.5e+02	1	SSKLSSSSGGGSVTGRSSVSSTPR
☑	2344	866.34	2595.99	2596.34	-0.35	1	5	1.6e+02	1	TAALFEARTPAVTYYCYLFVLK + Carbamidomethyl (C)
☑	1614	661.53	1321.05	1321.62	-0.57	1	5	2e+02	1	KGMETENEQLK + Oxidation (M)
☑	1014	1008.11	1007.10	1006.51	0.59	2	5	5.5e+02	1	AKGEEMAKK + Oxidation (M)
☑	2308	1235.81	2469.61	2470.15	-0.54	2	5	1.8e+02	1	MNFETKLNEQKQIMTDNNEK + Oxidation (M)
☑	1959	913.21	1824.41	1823.92	0.49	0	5	2.5e+02	1	GIAHGGVCLDNVLVSADGK
☑	47	497.17	1488.49	1488.69	-0.20	0	5	2.9e+02	1	EQLYGYLDMTTR
☑	224	620.83	1239.64	1239.60	0.04	1	5	6.9e+02	1	LIMKNAEMMK + 2 Oxidation (M)
☑	262	639.23	638.22	638.28	-0.05	0	5	1.2e+02	1	EMMTK
☑	1982	618.68	1853.02	1852.86	0.15	1	5	1.6e+02	1	EQMNKIISMNNSWMK
☑	411	725.05	1448.10	1447.79	0.31	2	5	4.2e+02	1	RQYTINLRQEK
☑	1257	1110.18	3327.52	3327.69	-0.17	1	5	4.4e+02	1	NQVDLTLIDLPGMTRVAVEGQSENIEETIK + Oxidation (M)
☑	2259	775.27	2322.78	2323.18	-0.40	1	5	1.8e+02	1	AGTPQYIPPYVIKGISDSFDR
☑	2310	825.78	2474.33	2474.18	0.15	0	5	1.3e+02	1	VAYFNEIGTSPVNSISFQQDMK
☑	1832	807.80	1613.58	1613.64	-0.06	1	5	1.6e+02	1	GCCDALAMERMER + 2 Carbamidomethyl (C); Oxidation (M)
☑	1345	1152.67	3454.98	3455.33	-0.36	1	5	4.3e+02	1	ATNGAASDADTSASSCSCSHCSCCCSSVGLRSTR + 3 Carbamidomethyl (C)
☑	653	849.20	1696.38	1696.89	-0.51	2	5	6.4e+02	1	GLAVNDSLSKKWDHK
☑	157	586.74	585.73	585.36	0.37	0	5	1.4e+02	1	ANIIR
☑	1407	591.77	1181.52	1181.57	-0.05	0	5	1.3e+02	1	ENLPVEYYR
☑	200	608.29	1821.85	1821.80	0.05	1	5	4.9e+02	1	AEGARGSTSNAMGNADGTR
☑	1921	586.32	1755.94	1755.73	0.21	0	5	1.5e+02	1	SSGDACDDTLVCVTEK + 2 Carbamidomethyl (C)
☑	1750	496.27	1485.80	1485.66	0.14	0	5	1.5e+02	1	DVWAHNLEDEMK
☑	1459	607.14	1212.28	1212.66	-0.38	1	5	2.2e+02	1	GSRSTPGPTVVR
☑	1585	1298.06	3891.16	3890.64	0.53	1	5	5.1e+02	1	CICTLEASCTANVNHDSECGGKGQACYGVLGSSTGER + 3 Carbamidomethyl (C)
☑	158	587.65	1759.93	1759.99	-0.06	1	5	5.1e+02	1	SVVIAAPECVKSLFLK + Carbamidomethyl (C)
☑	5	364.10	726.18	726.26	-0.08	0	5	2.7e+02	1	TCCMNR
☑	2383	928.67	2783.00	2783.10	-0.09	1	5	1.8e+02	1	NVNGTCDKCLFENCSECTSDVCTK + 3 Carbamidomethyl (C)
☑	1995	627.91	1880.71	1881.02	-0.31	2	5	1.5e+02	1	IANRKMYVLFEEQLK
☑	2393	960.69	2879.06	2878.48	0.58	1	5	1.8e+02	1	NRLPTAPLHPVTAAELLCELHPSEDR

☑	1386	1169.45	1168.44	1168.65	-0.20	0	5	4.6e+02	1	GNVVEINPVTK
☑	1450	1209.45	1208.45	1208.56	-0.12	1	5	4.8e+02	1	MCEAAIQKGSR + Oxidation (M)
☑	99	544.77	1631.28	1631.79	-0.51	1	5	6.9e+02	1	ELAETLEEQGKEEK
☑	821	931.58	930.57	930.51	0.06	1	5	1.9e+02	1	AVLRMEGR
☑	19	452.55	1354.63	1354.68	-0.05	0	5	4.6e+02	1	IGCISHHGYLAK + Carbamidomethyl (C)
☑	1846	546.30	1635.88	1635.80	0.09	1	5	1.6e+02	1	LVSCMEPGGMCLLKR
☑	584	815.40	814.39	814.39	-0.00	1	5	1.8e+02	1	KYLSSMS
☑	671	857.03	2568.08	2568.22	-0.14	1	5	5.9e+02	1	MECKGLPTMGILNTINPYCEIR + Carbamidomethyl (C); Oxidation (M)
☑	1730	490.50	1468.47	1468.80	-0.32	0	5	1.8e+02	1	IVLEGVPCELIER
☑	1753	497.19	1488.55	1488.82	-0.27	1	5	1.6e+02	1	ERGMPPPPRPVLK + Oxidation (M)
☑	1109	523.77	1045.53	1045.47	0.06	0	5	1.9e+02	1	HITCCDLK + 2 Carbamidomethyl (C)
☑	773	908.28	1814.56	1814.91	-0.35	2	5	6e+02	1	LEARMAEDPSQLSRGR
☑	1471	609.75	1217.48	1217.55	-0.08	0	5	1.9e+02	1	LGPNDMCEIAR
☑	2156	714.41	2140.21	2140.02	0.18	0	5	1.4e+02	1	MFCETLDIIGDIEIEPFR
☑	148	580.66	1159.31	1159.61	-0.30	0	5	4.6e+02	1	GCAIVAAMLSPK
☑	1245	1105.13	1104.12	1103.60	0.52	1	5	7.1e+02	1	KDISLEPFR
☑	1858	830.85	1659.68	1659.74	-0.06	1	5	1.6e+02	1	GYCQDVLSYLEAKD + Carbamidomethyl (C)
☑	491	771.79	1541.57	1541.07	0.51	2	5	5.3e+02	1	VIIIIIVKVYIKK
☑	1765	502.51	1504.51	1504.75	-0.24	1	5	1.7e+02	1	MDCQNRAAIALVK + Carbamidomethyl (C); Oxidation (M)
☑	89	537.96	1073.90	1073.46	0.44	0	5	4.1e+02	1	DCYISFNR + Carbamidomethyl (C)
☑	1533	1260.04	1259.03	1259.62	-0.59	1	5	5.1e+02	1	SGVDGNTVEARR
☑	92	539.28	1614.81	1614.90	-0.09	2	5	4e+02	1	VRRQVCSALSQLAK + Carbamidomethyl (C)
☑	1306	1135.08	1134.07	1134.53	-0.45	1	5	7e+02	1	NKDDQATTSR
☑	612	831.66	830.65	830.46	0.19	0	4	2.4e+02	1	IQSSIQR
☑	678	860.65	2578.93	2578.37	0.56	2	4	6.6e+02	1	LCMETPFSAVVAAAAYAKAIKAPAK + Carbamidomethyl (C)
☑	1775	760.44	1518.86	1518.86	-0.01	1	4	1.8e+02	1	ATKPAIPARAAEAPR
☑	243	627.21	626.20	626.28	-0.09	0	4	81	1	FMSSR
☑	1334	1150.14	1149.14	1148.64	0.49	1	4	5.8e+02	1	FKLTLSEPSK
☑	2343	863.63	2587.86	2588.20	-0.34	0	4	2.2e+02	1	AYDIPSPCSYMELNIQFLCVPL + Carbamidomethyl (C); Oxidation (M)
☑	2142	704.96	2111.87	2112.05	-0.18	2	4	1.8e+02	1	FVDLIDNKKDEIMEMIK + 2 Oxidation (M)
☑	423	731.61	2191.80	2192.09	-0.29	2	4	7.6e+02	1	ICRDFNPENRVQFGTAGLR
☑	1964	612.24	1833.69	1833.93	-0.25	1	4	1.7e+02	1	VMQSGEMKLDIIVNNK + Oxidation (M)
☑	247	631.10	1260.18	1259.63	0.54	1	4	8.8e+02	1	TFMSIARQYK + Oxidation (M)
☑	674	859.25	2574.74	2575.29	-0.55	1	4	7e+02	1	ADETVVEEKTEFDVIIQEVPSAK

☑	130	566.31	1130.61	1130.62	-0.01	0	4	3.9e+02	1	IIDTVSIENK
☑	684	433.67	865.33	865.47	-0.14	1	4	1.8e+02	1	DRITFSK
☑	1456	1211.15	3630.42	3630.79	-0.36	2	4	5.8e+02	1	GGVLCVDKEGVCSLLILEPPNFHQQRMIMMK + 2 Carbamidomethyl (C); 2 Oxidation
☑	300	659.69	658.68	658.38	0.30	0	4	2.5e+02	1	AATQLR
☑	1078	1034.81	2067.60	2067.86	-0.26	1	4	6.2e+02	1	HCGCAIRYCPCCGAELAGK + 2 Carbamidomethyl (C)
☑	1324	1143.61	3427.81	3427.72	0.09	1	4	4.9e+02	1	FNHGTAELSLVSPRPVLDSDEDGRAPLSHGVR
☑	2488	1172.74	3515.21	3515.70	-0.48	1	4	1.9e+02	1	DTGIIPCQEGVDGVPCYGNLGPEWELDVATIKK
☑	268	642.35	641.34	641.45	-0.10	1	4	4.3e+02	1	KGLVII
☑	1387	1169.96	2337.90	2338.17	-0.28	1	4	4.9e+02	1	VPGQYECSFSGLNTSKPAKLR + Carbamidomethyl (C)
☑	2112	1039.92	2077.83	2078.11	-0.28	2	4	1.6e+02	1	IKTDMLISIDVSLRSMEK
☑	778	910.15	1818.29	1818.04	0.25	2	4	5.3e+02	1	KITGMIDFPIKIAVTR + Oxidation (M)
☑	1208	1089.98	3266.92	3267.51	-0.58	1	4	6.3e+02	1	CLVGSWETTPAAGEARALFHECVEAYQSSR
☑	1654	687.74	1373.46	1373.54	-0.09	0	4	2.3e+02	1	MESMLSNMMPR + 3 Oxidation (M)
☑	1949	602.72	1805.15	1804.99	0.16	2	4	2e+02	1	ESIEKELSIFIQKNK
☑	1060	1027.55	3079.63	3079.42	0.21	1	4	4.7e+02	1	SLSQLHNPSPFSTLRCWEASGSGYDQR + Carbamidomethyl (C)
☑	1777	761.80	1521.58	1521.66	-0.07	0	4	1.8e+02	1	TATDGDDGGALWESK
☑	205	610.94	609.94	609.35	0.59	0	4	97	1	IDLHL
☑	1805	787.20	1572.39	1572.69	-0.30	1	4	3.3e+02	1	NYMSGDPSSKQTMK
☑	249	631.48	1891.42	1891.94	-0.53	0	4	8.5e+02	1	YVEFINIFDCLFELK
☑	346	687.09	1372.16	1372.70	-0.53	2	4	7.2e+02	1	APKKAGMAMPESR
☑	246	630.92	629.91	629.35	0.56	0	4	5.8e+02	1	AAEAIR
☑	1017	1008.92	1007.91	1008.51	-0.60	2	4	6.8e+02	1	KMCDLKAK + Carbamidomethyl (C); Oxidation (M)
☑	84	534.54	1600.60	1600.86	-0.26	0	4	5.3e+02	1	LNEVFLNILNQER
☑	1190	1079.40	1078.39	1078.54	-0.15	1	4	5.4e+02	1	SQQSALCKSK
☑	1353	1154.49	1153.48	1153.64	-0.16	2	4	4.6e+02	1	HMKKQGLNAK
☑	248	631.27	630.26	630.31	-0.05	0	4	3.4e+02	1	HFAEK
☑	695	872.86	871.85	871.41	0.44	1	4	6.4e+02	1	TGEMKYK + Oxidation (M)
☑	1455	1210.66	3628.96	3628.74	0.22	2	4	4.4e+02	1	MRFEALKHHLAALPTHVCDDNGASVGPPTAMNR + Carbamidomethyl (C); Oxidation (M
☑	2291	799.24	2394.69	2395.26	-0.56	2	4	3e+02	1	QFQAMYAEKKDLEAIITQLR
☑	72	521.35	1040.69	1040.60	0.09	2	4	3.9e+02	1	LKFNAHRR
☑	631	841.83	840.82	841.38	-0.56	0	4	1.9e+02	1	QYSDTTK
☑	2330	844.70	2531.07	2531.31	-0.24	2	4	1.8e+02	1	TTAAKTAEAVAEALRGTTVGEEEVK
☑	313	668.06	1334.10	1333.65	0.45	0	4	6.3e+02	1	WSGMFGTPQVPK
☑	174	595.64	1783.90	1783.83	0.07	0	4	5.2e+02	1	MEGTTSVANNSSTTSAVK

☑	413	725.26	2172.75	2173.00	-0.25	0	4	5e+02	1	AALHNSQFHVICTCSGCNIR
☑	1084	1036.88	3107.62	3107.76	-0.13	1	4	5.5e+02	1	ALRNVALLLGALIVQEPAPLLLDFFDMR
☑	2441	1053.63	3157.88	3157.58	0.30	2	4	1.7e+02	1	MGVPSVLFVCTGNICRSPVAEVICSKYTK + Carbamidomethyl (C)
☑	1437	1200.98	3599.91	3599.44	0.47	1	4	5.2e+02	1	YFDGSNCVSCGSNCSLCTVNGKCSLCDDSTYLK + 2 Carbamidomethyl (C)
☑	1685	705.80	1409.59	1409.75	-0.16	2	4	2e+02	1	GHKVYLKDASHR
☑	270	644.24	643.24	643.27	-0.04	0	4	2.3e+02	1	ACEHK + Carbamidomethyl (C)
☑	1794	781.25	1560.48	1560.85	-0.38	1	4	2e+02	1	LTCPTGLKNATTLTK
☑	637	843.43	842.42	842.53	-0.11	1	4	5.8e+02	1	AGRLISVK
☑	1300	1132.69	2263.38	2263.31	0.07	2	4	6.2e+02	1	LIAGHTNKFEQIKGEILKPK
☑	2315	830.40	2488.18	2488.22	-0.04	0	4	1.6e+02	1	ALDAFDLPMLAYHLVTAAGSGPCR
☑	2434	1043.42	3127.25	3127.41	-0.16	0	4	1.6e+02	1	HPEAFCEVQLPGCVHVAMASEACLPFR + 3 Carbamidomethyl (C); Oxidation (M)
☑	1948	600.97	1799.87	1799.88	-0.01	0	4	1.8e+02	1	ECIIEVDEHLLNVMK + Oxidation (M)
☑	2175	724.43	2170.26	2170.21	0.05	1	4	1.6e+02	1	MTEAIVGLLLMTNRRPAIR + Oxidation (M)
☑	692	871.72	1741.43	1741.91	-0.48	0	4	6.7e+02	1	VPFKPFFATTDFTPK
☑	2283	793.75	2378.22	2378.05	0.18	1	4	1.7e+02	1	GMCPDMIMNPHGFPSRMTVGK + Carbamidomethyl (C); Oxidation (M)
☑	994	997.58	2989.73	2989.50	0.23	1	4	5e+02	1	MSTMPVTLRTTTTVFLLCGICALDVSK + Carbamidomethyl (C); 2 Oxidation (M)
☑	2194	1097.40	2192.78	2193.02	-0.23	2	4	2.1e+02	1	LCIYMKCFDDNLVEKSEK + Oxidation (M)
☑	1711	721.88	1441.74	1441.81	-0.06	2	4	1.9e+02	1	LKHMVLDKISSR + Oxidation (M)
☑	1527	1257.01	3768.01	3767.57	0.44	1	4	5.6e+02	1	SKNGSVCVCVCVCVCVCVCVCVCVCVCVCVYK + Carbamidomethyl (C)
☑	1243	1104.07	2206.12	2206.07	0.05	1	4	8.3e+02	1	HGMQQGTHMETSLTLHRVK + Oxidation (M)
☑	1603	655.29	1308.56	1308.65	-0.09	1	4	2.1e+02	1	LMKSAIGEGMTR + Oxidation (M)
☑	444	745.71	2234.11	2234.19	-0.08	2	4	7.1e+02	1	APIQSFIDTMNKFIPVNRK + Oxidation (M)
☑	1676	696.38	1390.75	1390.73	0.03	2	4	2.1e+02	1	FARRQLGDLCR + Carbamidomethyl (C)
☑	1764	502.49	1504.44	1504.87	-0.43	1	4	2.1e+02	1	LTGRPRPGVSRGPR
☑	203	609.98	1217.94	1217.61	0.33	0	4	5.5e+02	1	EMQLTAELQR
☑	1151	532.22	1062.42	1062.56	-0.14	1	4	2.2e+02	1	RVAAASEGFR
☑	700	873.92	872.91	873.46	-0.54	0	4	5.6e+02	1	GSVVDVNGK
☑	400	718.37	1434.73	1434.72	0.01	0	4	6.7e+02	1	RPNAYAMQCLLR
☑	1280	1123.14	3366.40	3365.83	0.57	1	4	6.9e+02	1	SSIFSVKSVGTLVHLMLCAGTGLMFLGALLLR + 2 Oxidation (M)
☑	2204	738.98	2213.92	2214.06	-0.14	2	4	1.8e+02	1	TMRKNTVDSDFDVGGISAGTK + Oxidation (M)
☑	1130	1056.03	3165.06	3165.45	-0.39	1	4	7.5e+02	1	GCMGLTTGMCVGPPSNLDRNILCPPGQSYK + Carbamidomethyl (C)
☑	788	917.04	2748.11	2748.27	-0.16	1	4	8.6e+02	1	TGKYSSSGIFTCVGGEYDIHEISNK + Carbamidomethyl (C)
☑	2320	834.86	2501.56	2501.47	0.08	0	4	2.1e+02	1	QPILHPSNSGSIVPIKPAPVIIPK
☑	142	575.33	1148.65	1148.60	0.04	0	4	6.3e+02	1	VHSAIAGGAPNR

☑	252	633.77	1265.52	1265.69	-0.17	2	4	9e+02	1	RHRNIVMQGR
☑	2025	960.62	1919.22	1918.74	0.47	0	4	1.9e+02	1	SADAEMESMMGTVGCSDAK
☑	1121	1050.83	3149.47	3149.50	-0.03	1	4	6.6e+02	1	VGVMCGGMDQAISILGEKDHACVISFVPK + 2 Carbamidomethyl (C); 2 Oxidation (M
☑	1410	1182.85	3545.53	3544.95	0.57	1	4	6.3e+02	1	HSRSTVVLLVVGCVFISLIAFVCGVMVVLPCLR + Oxidation (M)
☑	109	548.69	547.68	547.33	0.35	1	4	1.5e+02	1	AKTTK
☑	1251	555.02	1108.02	1108.56	-0.55	1	4	3.6e+02	1	DSRSPPTPPR
☑	721	884.35	2650.02	2650.34	-0.32	2	4	5.4e+02	1	FQIVSLNEHFKNHRMMFLFGR
☑	2081	677.60	2029.77	2029.96	-0.18	1	4	2.2e+02	1	QQEAGEELCGPGKNCIIK + 2 Carbamidomethyl (C)
☑	591	820.77	2459.29	2459.23	0.06	2	4	6.9e+02	1	MHVCISHFPFRVTVLRCWR + Carbamidomethyl (C); Oxidation (M)
☑	386	709.89	2126.66	2126.86	-0.20	1	4	7.9e+02	1	TQCVRACQVDNCEQCNADK
☑	537	796.50	2386.46	2386.14	0.32	1	4	6e+02	1	ATDYLETLCAKADGVIETVCSK + Carbamidomethyl (C)
☑	833	933.71	2798.11	2798.28	-0.17	0	4	7.7e+02	1	ELYDLVDLDHSGGIDYNEMVNMLK + Oxidation (M)
☑	1415	1186.92	2371.82	2372.23	-0.42	2	4	6.1e+02	1	RLGSSTFRSSLDLWHLPSTGR
☑	280	649.27	648.26	648.30	-0.04	0	4	6.6e+02	1	GATCAAR
☑	1262	1114.01	3339.01	3338.63	0.38	2	4	7.5e+02	1	HLQTDPERGAVLLTADRDSFLDIGDPNMSR
☑	217	617.26	616.25	616.32	-0.07	0	4	2.9e+02	1	DVDIR
☑	202	608.69	1823.06	1822.83	0.23	1	4	7e+02	1	AAEASTSSRGADDFVDPK
☑	417	729.19	2184.54	2185.09	-0.55	2	4	8.4e+02	1	AYEFTQTGVGPERVRFSNK
☑	881	477.58	953.14	953.58	-0.44	2	4	1.9e+02	1	QAPKRVGAK
☑	1552	638.72	1275.42	1275.70	-0.28	2	4	2.9e+02	1	RKDETVIMIR + Oxidation (M)
☑	1001	1001.58	3001.72	3001.38	0.35	2	4	6.1e+02	1	ETEVEEEETPKGDEEDADKPKIEEVK
☑	1173	1072.26	2142.50	2142.04	0.46	1	4	7.2e+02	1	LCPQQSATLLKCHMTEPAR + Oxidation (M)
☑	1231	1100.42	3298.24	3298.53	-0.29	2	4	6.3e+02	1	CPDHENFRQLALIAYSDYGGCQVRVMGEK
☑	1519	1247.61	1246.60	1246.64	-0.04	0	4	2.6e+02	1	MALLATDIPMR + Oxidation (M)
☑	1673	695.35	1388.68	1388.73	-0.06	0	4	2.1e+02	1	GLIEALHCLHAR + Carbamidomethyl (C)
☑	2258	775.23	2322.67	2322.15	0.52	0	4	3.3e+02	1	AILTISQDQQEITDCAFSLAR
☑	519	789.40	2365.18	2365.12	0.06	2	4	6.5e+02	1	SNSARAQSNDPMATDVRNYLR
☑	1169	1071.03	3210.06	3209.60	0.46	1	4	8.7e+02	1	QLPTQRQTLMFSATQTTDVQMLGQMSLR
☑	2392	950.86	2849.57	2849.43	0.14	1	4	1.6e+02	1	AHPTGAVIACGGGIVEREANCAVLQATK + 2 Carbamidomethyl (C)
☑	291	653.86	1958.55	1958.94	-0.39	2	4	8.3e+02	1	MSIGATSQRRGEDPWPR + Oxidation (M)
☑	1880	564.85	1691.52	1691.81	-0.29	0	4	2.7e+02	1	VHGSVALDMCFIATGR + Oxidation (M)
☑	1395	1174.63	2347.24	2346.99	0.26	1	3	5.5e+02	1	GYGNMMINFSYMLSSRECR + Carbamidomethyl (C); 2 Oxidation (M)
☑	850	938.60	937.59	937.45	0.14	0	3	2.1e+02	1	NIHNGNNR
☑	926	972.36	2914.05	2914.35	-0.30	1	3	7e+02	1	ENSSNAMSGCCSVYVANIPPSVDSVKLK + Oxidation (M)

☑	1993	939.71	1877.41	1877.74	-0.33	2	3	3.7e+02	1	STRICTKCDTSCETCNG + Carbamidomethyl (C)
☑	356	693.18	692.17	692.28	-0.11	0	3	1.9e+02	1	SCSPDK + Carbamidomethyl (C)
☑	929	973.41	1944.80	1945.13	-0.33	2	3	6.8e+02	1	SIPAGRTILLKNFNVFR
☑	2114	694.56	2080.66	2080.95	-0.29	1	3	2.8e+02	1	EEKNTDTSDTTKPNYPNK
☑	1893	853.35	1704.68	1704.80	-0.12	1	3	2.1e+02	1	KDNTPVEIMNDANTK + Oxidation (M)
☑	732	890.13	2667.38	2667.35	0.04	1	3	8.4e+02	1	NASQILLGDASANSPIEEEEGLVRR
☑	1698	715.94	1429.87	1429.64	0.23	1	3	2.2e+02	1	TCESVVMKSMSR + Carbamidomethyl (C); Oxidation (M)
☑	255	634.40	633.39	633.33	0.06	1	3	2.6e+02	1	ELTSKG
☑	414	726.14	2175.40	2175.04	0.36	2	3	7.3e+02	1	GLAGARVSRCAAGGDIQDDTSR
☑	1260	1112.56	1111.55	1111.65	-0.09	1	3	5.7e+02	1	VRAALENLAR
☑	1981	618.13	1851.38	1850.88	0.49	0	3	3.8e+02	1	NFESEQMILNQVDLR + Oxidation (M)
☑	1594	1302.47	2602.92	2603.11	-0.19	0	3	6.9e+02	1	EEEPVTLTKPQSSNNEPQEAGGCC + Carbamidomethyl (C)
☑	166	590.99	1179.97	1179.67	0.30	1	3	7e+02	1	NVISQHNKIK
☑	957	981.82	980.82	980.54	0.28	0	3	2.2e+02	1	QILTCLYK
☑	625	840.16	2517.45	2517.28	0.17	0	3	7.5e+02	1	MQNLNHLIIFVESETSWLCLK
☑	649	846.90	2537.68	2537.29	0.38	2	3	1.1e+03	1	GIAIPTMASALDSRYISSLRDER + Oxidation (M)
☑	2321	835.43	2503.26	2503.36	-0.10	1	3	2.2e+02	1	VLQTMSTIDPLFSLEKVNEVLK
☑	283	650.70	1299.39	1299.59	-0.20	1	3	8.3e+02	1	RGPASIGSCHCR + Carbamidomethyl (C)
☑	2304	820.68	2459.02	2459.29	-0.26	2	3	1.9e+02	1	KDRVAFCAAVLGSYGAHAVQAIR + Carbamidomethyl (C)
☑	1332	1149.09	3444.24	3444.65	-0.41	1	3	9.3e+02	1	ELITCWSFSVRPFQWCFSEPGRTNTAGIR + Carbamidomethyl (C)
☑	1624	1332.64	3994.89	3994.93	-0.05	2	3	5.3e+02	1	KYTSINDFFKDLQLIHDNSSLYNGPTHMLTHMAK + Oxidation (M)
☑	1439	601.36	1200.71	1200.57	0.15	1	3	2.4e+02	1	FTAFSDEEKK
☑	1888	568.48	1702.40	1702.94	-0.54	1	3	4e+02	1	EHGEAIIYLVRYLK
☑	1670	1385.60	2769.19	2769.48	-0.29	2	3	5.3e+02	1	QRQQYSRPIIRAHLFSSIGDIER
☑	312	666.24	1330.47	1330.76	-0.29	1	3	7.5e+02	1	LVKDIFVAAAER
☑	1292	1128.71	1127.70	1127.67	0.03	2	3	7.3e+02	1	ALRAIWSRR
☑	932	974.84	1947.66	1947.12	0.54	2	3	7.1e+02	1	GRPLPPLPGTAAVFKRDR
☑	1486	1228.47	2454.92	2455.43	-0.51	2	3	7.1e+02	1	NAKTGVPQHLIRSATAILPLVEK
☑	910	966.95	965.94	965.53	0.41	0	3	4e+02	1	VFAFQAGVK
☑	412	725.20	2172.57	2173.02	-0.45	1	3	7.8e+02	1	RVCRPPTAETPGGGGSGSSSEK + Carbamidomethyl (C)
☑	1570	645.77	1289.53	1289.74	-0.21	2	3	2.6e+02	1	LKMAAAKATEIK + Oxidation (M)
☑	1448	1206.47	2410.94	2411.11	-0.18	1	3	6.7e+02	1	AMAYYCAAADVHTVWAVGNDRK
☑	2092	683.39	2047.16	2046.96	0.20	1	3	2.2e+02	1	GHEICSSSLVTSAEEAKSR + Carbamidomethyl (C)
☑	119	557.18	556.17	556.30	-0.12	0	3	79	1	GGPQAK

☑	385	709.60	2125.79	2126.05	-0.26	2	3	8.5e+02	1	ANEEQVRQEAQREAAAQAK
☑	1487	1228.47	3682.39	3682.76	-0.36	1	3	7.1e+02	1	WHHVMEVPGGEGTGMEVHEGKPPQPWTYKAVGR
☑	2157	714.74	2141.20	2141.09	0.11	0	3	2.3e+02	1	FQLVIAVNENMCLEVIYK + Oxidation (M)
☑	1571	1290.65	2579.28	2579.24	0.04	1	3	5.9e+02	1	ALMMRPSRPLLSGDMHSSDRFK + 3 Oxidation (M)
☑	397	715.65	2143.94	2144.11	-0.17	2	3	9e+02	1	IQEEAINELKRCEEVITK
☑	1034	1015.15	2028.28	2028.16	0.12	2	3	8.2e+02	1	AVIDTLAAAVRRGFSVQVR
☑	1133	528.78	1055.55	1055.67	-0.12	0	3	2.4e+02	1	VLGSVISLLR
☑	197	606.04	1210.06	1209.59	0.48	0	3	7.8e+02	1	TTASTQTHHAR
☑	1761	749.90	1497.80	1497.63	0.17	0	3	2.3e+02	1	QSCMMQDLENVK + Carbamidomethyl (C); Oxidation (M)
☑	524	792.83	791.82	792.42	-0.59	1	3	4.3e+02	1	KITEMR + Oxidation (M)
☑	2470	1116.38	3346.12	3346.65	-0.54	2	3	2.7e+02	1	HRQGNEMSPTANGLVSMPPIFVRAPEEPAAK + Oxidation (M)
☑	1458	1212.79	1211.78	1211.64	0.14	2	3	7.9e+02	1	INLKDCGKHK + Carbamidomethyl (C)
☑	758	903.76	902.76	902.48	0.28	0	3	8.5e+02	1	TSALIGADR
☑	807	463.76	925.50	925.56	-0.06	0	3	2e+02	1	VLTDLIPR
☑	892	480.14	958.28	958.54	-0.27	0	3	2.7e+02	1	TILASDALR
☑	750	901.33	900.32	900.50	-0.18	1	3	7.9e+02	1	INNKEVGK
☑	2178	1086.89	2171.77	2172.12	-0.35	1	3	2.6e+02	1	QEEFHDKTIIFIIGGPGSGK
☑	1787	514.00	1538.97	1538.91	0.06	2	3	2.3e+02	1	LASVMVPLRRLER
☑	1371	582.24	1162.46	1162.55	-0.09	0	3	2.4e+02	1	ANIYGFHDAR
☑	503	777.29	2328.84	2329.29	-0.45	2	3	7.7e+02	1	LFWDVVESLTRAQQRQLLK
☑	783	913.15	2736.44	2737.00	-0.56	1	3	7.8e+02	1	NNTGLCMGCDPNCEESCKSPSMCNK + Carbamidomethyl (C); Oxidation (M)
☑	2174	723.73	2168.16	2168.06	0.10	1	3	2.3e+02	1	LATAPGCVLGHLSDAGCARSR + 2 Carbamidomethyl (C)
☑	2375	914.24	2739.68	2739.34	0.35	2	3	2.4e+02	1	MHPGGYYFNPLKFGKDEADLAELK
☑	2110	691.99	2072.94	2073.04	-0.10	1	3	2.2e+02	1	KPGMTRDDLFHTNASIVR + Oxidation (M)
☑	2339	858.51	2572.51	2573.03	-0.52	0	3	2e+02	1	DDTTTTETTGETETGYSESLGDTY
☑	418	729.41	2185.21	2185.03	0.18	0	3	7.3e+02	1	TMAEQYGVPVILHTDHCAK + Carbamidomethyl (C); Oxidation (M)
☑	805	925.93	2774.77	2775.27	-0.50	2	3	8.7e+02	1	AFCPNTDDELEALLSYQRCFRER
☑	206	612.71	1223.40	1223.60	-0.20	1	3	6.3e+02	1	TCPADLYKTR + Carbamidomethyl (C)
☑	2050	991.82	1981.63	1982.11	-0.48	2	3	3.2e+02	1	KPPRTEMIASVRAELLR + Oxidation (M)
☑	2232	761.50	2281.47	2281.11	0.36	2	3	3.2e+02	1	YQLMRAVGSNEMATPWQRK + Oxidation (M)
☑	944	978.27	977.27	977.52	-0.25	0	3	2.7e+02	1	FVQEAVGTK
☑	466	761.90	760.90	760.38	0.51	1	3	6.5e+02	1	KAGGSADR
☑	1434	1197.52	3589.54	3589.65	-0.10	1	3	6.2e+02	1	TMRGGAGTHLTFPIFSTLYGASCGEEQGPLSQSSS + Oxidation (M)
☑	196	604.73	1811.16	1810.97	0.20	1	3	9.9e+02	1	LSFACSALLPSVSFNKK

☑	792	919.41	918.40	918.52	-0.12	1	3	2.9e+02	1	VPGKLYDK
☑	681	861.52	2581.53	2581.07	0.46	2	3	8.1e+02	1	DKYFFMFGNCYCPECLKCTR + 2 Carbamidomethyl (C)
☑	1059	1026.19	2050.36	2049.99	0.36	0	3	7.2e+02	1	QTLWPAHCVQDTPGADLAK
☑	1291	1127.66	1126.65	1126.60	0.05	0	3	6.7e+02	1	SGSVTPTHTLK
☑	2483	1159.40	3475.18	3474.63	0.54	1	3	2.9e+02	1	RSGQYLDFLVDTAAASCLSYLENLHDWMTR
☑	1662	692.20	1382.38	1381.78	0.60	0	3	2.8e+02	1	LSTTPTPQLPSLK
☑	1096	1039.90	1038.90	1038.57	0.33	1	3	7e+02	1	NKTVTVYSK
☑	1657	689.62	1377.23	1377.80	-0.56	0	3	4.9e+02	1	AESVLELIVLHR
☑	963	983.52	2947.55	2947.37	0.18	1	3	7e+02	1	HAMWLGFASGGCEQVLPADGGLRVACMR + Oxidation (M)
☑	2455	1082.67	3245.00	3245.53	-0.53	0	3	2.6e+02	1	DEEMSETIFDIVSFYLVATLDIEFFEK + Oxidation (M)
☑	1425	1190.29	1189.28	1189.61	-0.33	2	3	8e+02	1	MQEKELKER
☑	180	599.32	1794.94	1794.94	-0.00	2	3	6.2e+02	1	IRLHRNLEGPFQCR + Carbamidomethyl (C)
☑	806	926.46	1850.91	1850.92	-0.01	1	3	7.1e+02	1	LCVADRQATGSLVTTYP + Carbamidomethyl (C)
☑	1217	1094.30	3279.87	3279.70	0.17	1	3	8.6e+02	1	GCLSLSHFINHLTQQGIDGRALTGTVCILR + Carbamidomethyl (C)
☑	2274	788.76	2363.27	2363.06	0.20	1	3	2.2e+02	1	ALQHDSMHVSYFSACNRNAR + Carbamidomethyl (C)
☑	705	876.89	875.88	875.40	0.48	0	3	1e+03	1	LGEGESER
☑	2486	1164.23	3489.66	3489.57	0.09	2	3	1.7e+02	1	GILGMHSDGEDEDDDHGRRPPASGPSKGMAINR + Oxidation (M)
☑	880	954.02	2859.04	2859.37	-0.34	1	3	9.2e+02	1	VYGAARYEWGWCIRPHNVWFGHR
☑	2120	697.42	2089.23	2089.11	0.12	2	3	2.3e+02	1	EAETEKETLMLQLTGLRK
☑	2167	722.05	2163.12	2163.10	0.02	0	3	2.5e+02	1	SQPAQQMPHVPQPQLPQPR
☑	1307	1135.90	3404.67	3404.75	-0.08	0	3	7.7e+02	1	NYCLVVLMNNFHYAAALLPDIIESVVIADK + Carbamidomethyl (C)
☑	2033	645.03	1932.06	1931.94	0.12	1	3	2.3e+02	1	VDKGIEIVDDEESLPQF
☑	232	623.34	1244.67	1244.64	0.04	1	3	7.9e+02	1	NNWSRGGLVSR
☑	256	634.96	1267.91	1267.62	0.30	0	3	8.3e+02	1	LDAIDAHSAEAR
☑	1182	1076.24	3225.69	3225.55	0.15	1	3	8e+02	1	SVLHSTRTTDSVCVTTSSETLVEDLDAFR + Carbamidomethyl (C)
☑	627	840.38	839.37	839.45	-0.08	0	3	6.7e+02	1	AGIHIDSK
☑	2170	722.49	2164.46	2164.03	0.43	2	3	3.3e+02	1	FGTYWAQRCPTRFMVTK + Carbamidomethyl (C); Oxidation (M)
☑	2058	667.71	2000.11	2000.00	0.11	2	3	2.9e+02	1	TQNYPQSKTLFKSLCDK
☑	2144	1058.56	2115.10	2115.01	0.09	1	3	2.3e+02	1	GVKNVVNGAAADTAAGDAQACR + Carbamidomethyl (C)
☑	36	486.18	1455.52	1455.81	-0.29	1	3	5.1e+02	1	YYALNTFPKVLK
☑	45	495.34	1483.00	1482.62	0.38	1	3	5.9e+02	1	ERCATVGDDGTMR + Carbamidomethyl (C); Oxidation (M)
☑	720	884.33	883.32	883.51	-0.19	1	3	7.1e+02	1	ARTRPQR
☑	1560	643.18	1284.34	1284.58	-0.24	1	3	3.1e+02	1	EMDQNMKFVK + Oxidation (M)
☑	28	474.52	947.03	947.42	-0.39	0	3	8.8e+02	1	SNWPADMK

☑	2134	702.53	2104.57	2105.06	-0.49	2	3	4e+02	1	KQMQLENQNLQEMSKIK + Oxidation (M)
☑	548	800.25	2397.74	2398.33	-0.59	1	3	9.1e+02	1	ETGALKVIVISTGSELNVAVEAAK
☑	557	804.84	2411.49	2411.22	0.27	2	3	1e+03	1	TKVQTNRSEYGNLSITQGLMR + Oxidation (M)
☑	1053	1024.06	1023.05	1023.51	-0.46	2	3	9.4e+02	1	GSSSRGKMAK + Oxidation (M)
☑	1222	1096.55	3286.62	3286.71	-0.09	2	3	6.2e+02	1	RIISVGCLLDQGCSVTKMTATEIVILSHDK + Carbamidomethyl (C)
☑	1831	807.43	1612.85	1612.71	0.14	2	3	3e+02	1	QASEMEEEKMKNK + 2 Oxidation (M)
☑	989	331.93	992.77	992.43	0.34	1	3	2.4e+02	1	ADQARSVGSC
☑	1615	1325.06	2648.10	2648.34	-0.24	1	3	7.3e+02	1	VAAAVAAGFMVIACIGETLQERESGR
☑	429	735.30	2202.87	2203.02	-0.15	1	3	8.2e+02	1	VTKVEVESVSSDEDFDFGSK
☑	1447	1204.90	3611.69	3611.82	-0.13	2	3	8.7e+02	1	QEPIPTSESELMAVFEALQVEFLTRYEATRK
☑	1504	1237.65	3709.93	3709.87	0.06	2	3	6.5e+02	1	QIKEANASYVEKPRNEWIYQYPCQAVIVASR + Carbamidomethyl (C)
☑	1760	748.76	1495.50	1495.75	-0.26	0	3	3e+02	1	ASHVQWSATVPSAR
☑	727	886.32	2655.94	2656.19	-0.24	2	3	8.2e+02	1	QMENHESDTCATHGDSGVKLPEKK + Oxidation (M)
☑	1248	1106.47	1105.46	1105.51	-0.05	0	3	8.4e+02	1	CVYISSSYK + Carbamidomethyl (C)
☑	1238	551.70	1101.39	1101.55	-0.16	0	3	2.9e+02	1	TADHMVTLAK + Oxidation (M)
☑	756	903.17	2706.49	2706.21	0.28	0	3	1e+03	1	ETLAAVNECNVIWMDGVMFMHHK + 2 Oxidation (M)
☑	1470	1218.27	2434.52	2434.30	0.22	2	3	8.2e+02	1	ILDNRLKCDDVPSPQVIISPR + Carbamidomethyl (C)
☑	931	973.58	2917.72	2917.56	0.16	0	3	8.5e+02	1	ADVLVSSLLLVYFGTEHMVLDTLVQR
☑	939	977.37	976.36	976.51	-0.15	1	3	8.2e+02	1	GCGGVRLTSK
☑	227	621.64	1861.89	1861.89	0.00	1	3	8.4e+02	1	QDVLCVNGVMDELKDK + Carbamidomethyl (C)
☑	1100	1043.35	2084.68	2085.05	-0.37	1	3	9.9e+02	1	AVSPDFLQSSPQIKGPGDSR
☑	1349	1153.89	2305.77	2305.93	-0.16	1	3	7.6e+02	1	ACYHCGQPGHFSRECPNMR + 2 Carbamidomethyl (C)
☑	2445	1062.61	3184.82	3184.43	0.39	1	3	2.1e+02	1	LAWWESFCLGKVYSMMAECNGILSAMR + Carbamidomethyl (C); 2 Oxidation (M)
☑	981	495.66	989.30	989.54	-0.24	2	2	3.4e+02	1	KRAAEMLR + Oxidation (M)
☑	2497	1256.61	3766.81	3766.66	0.15	2	2	1.6e+02	1	DNRSWEAIYQESADHATTSFLNDPKEAEEEER
☑	1194	1083.08	3246.23	3246.51	-0.28	0	2	9.5e+02	1	YIADANMVFITAGMGGGTGTGAAPVVAEVCMEK + Oxidation (M)
☑	1479	1223.36	3667.05	3666.73	0.33	1	2	8.4e+02	1	FKMAILTMVSYYDVAFTFDAEFLCDLLNQCR
☑	1372	582.35	1162.69	1162.60	0.10	1	2	2.7e+02	1	CNACLSLVRK + Carbamidomethyl (C)
☑	1469	1217.66	3649.97	3649.71	0.26	1	2	7e+02	1	AKEAPASASMHTTVISSPNTTSHPGAPSSSLPCSER + Carbamidomethyl (C)
☑	1033	1014.83	3041.48	3041.58	-0.11	1	2	8.8e+02	1	QIYIKLQQAMEALAIHKPDNPCGFLK + Carbamidomethyl (C); Oxidation (M)
☑	1103	1045.10	3132.27	3132.48	-0.20	0	2	1e+03	1	VSFGAGGICWPQLGGVSGMLHCTGVVMAPR + 2 Carbamidomethyl (C); 2 Oxidation (
☑	1250	1108.99	3323.95	3323.39	0.56	1	2	9.1e+02	1	CVCVCVCVCVCVCVCVCVLDEQRR + 5 Carbamidomethyl (C)
☑	1429	398.81	1193.41	1193.70	-0.30	1	2	2.8e+02	1	KVFVVHGGVPR
☑	1214	1092.43	3274.27	3274.58	-0.31	2	2	8.1e+02	1	VYVFLQNCIHPKFNLSSGKQEDFDMATK + Oxidation (M)

☑	1286	1126.04	3375.10	3375.29	-0.19	0	2	1e+03	1	SASLDNGPDSLSSDSCTCSCTTSTSDFNGSCR + 3 Carbamidomethyl (C)
☑	1542	1267.38	3799.10	3799.63	-0.53	0	2	8.3e+02	1	MAGLPSVSVVPCDGCAAALACFDALTASFFECACSCR + 2 Carbamidomethyl (C)
☑	1857	827.79	1653.57	1653.85	-0.29	1	2	2.9e+02	1	ECFIANGKLGYIAAK + Carbamidomethyl (C)
☑	675	860.08	1718.15	1717.73	0.42	0	2	1e+03	1	MSSQSPEGMESTFLR + 2 Oxidation (M)
☑	717	881.02	2640.05	2640.14	-0.10	0	2	9.8e+02	1	CMALEVAEMCVVDDTDTVPVENK + 2 Carbamidomethyl (C); Oxidation (M)
☑	1462	1215.20	3642.58	3642.65	-0.07	0	2	9.3e+02	1	LTMCSEHQVAFCVAEALGSDLKPVMLDWAMCR + Carbamidomethyl (C); 2 Oxidation (
☑	481	767.08	1532.14	1531.82	0.31	1	2	8.6e+02	1	NNNISSKSLSDILK
☑	2189	728.52	2182.54	2182.12	0.41	0	2	4.4e+02	1	LNVSFVVTGASWKPLYDMR
☑	554	803.45	802.44	802.39	0.06	1	2	1e+03	1	RTSHMR + Oxidation (M)
☑	839	935.40	934.39	934.51	-0.11	1	2	3.9e+02	1	LRSFMPGK
☑	1126	1055.18	1054.17	1054.51	-0.34	0	2	7.8e+02	1	MPPPPASGSAK + Oxidation (M)
☑	1575	646.58	1291.15	1290.63	0.51	1	2	6e+02	1	DGVTALMRAADR + Oxidation (M)
☑	27	471.24	1410.69	1410.62	0.06	2	2	5.1e+02	1	MCGKDSGVEERK + Carbamidomethyl (C); Oxidation (M)
☑	1063	1028.75	3083.22	3083.51	-0.30	2	2	9.6e+02	1	KARPSRPASDATMTMGDLIHYNPRNPR + 2 Oxidation (M)
☑	841	935.49	934.48	934.51	-0.02	0	2	8.8e+02	1	QLAFMLGR
☑	1337	1150.20	1149.19	1149.61	-0.42	1	2	4e+02	1	TDVAVKAQYR
☑	1413	1184.92	2367.83	2368.04	-0.22	1	2	8.4e+02	1	CFHGAVMIDDDRMMIVGGNAGK + 2 Oxidation (M)
☑	1848	821.28	1640.55	1639.97	0.58	1	2	3.2e+02	1	SMLIQREILALIPK + Oxidation (M)
☑	258	637.92	636.92	636.32	0.59	0	2	3e+02	1	FTDVR
☑	918	970.62	2908.82	2908.43	0.39	2	2	8.3e+02	1	NAMIAAAGGDSGAQNNYGVMLLNGKGVRK + 2 Oxidation (M)
☑	988	993.50	992.49	992.60	-0.11	2	2	2.6e+02	1	AYAALKKTK
☑	1160	533.76	1065.50	1065.63	-0.13	2	2	2.6e+02	1	HDKQVAIKK
☑	1374	1164.76	2327.51	2327.07	0.44	1	2	1e+03	1	ESSSDSSDEFRPLSPCLTSKR
☑	1381	1166.46	2330.90	2331.17	-0.27	2	2	7.5e+02	1	GVDFKECSSLFAPIMLTMSKK
☑	140	572.11	1713.31	1712.87	0.43	2	2	1e+03	1	TIGKYGAKIDYEEAR
☑	354	691.82	690.81	690.39	0.42	1	2	6.6e+02	1	DITSKK
☑	1988	622.64	1864.91	1864.96	-0.05	0	2	2.6e+02	1	FAMLVNEVENVTATALK + Oxidation (M)
☑	2182	725.91	2174.72	2175.06	-0.34	1	2	3.2e+02	1	MREIICLQIGQCGNQVGEK + Carbamidomethyl (C)
☑	541	797.08	2388.22	2388.24	-0.03	1	2	8.4e+02	1	GWTAMMYAAQNGHLSVVKLLAK
☑	942	977.83	2930.46	2930.45	0.00	1	2	8.7e+02	1	YFIENGVHNTLISMNIPKEIEYYK + Oxidation (M)
☑	1137	1058.20	3171.59	3171.32	0.27	1	2	9.2e+02	1	ARAGVTCHACAEGYGLSGGSCSPCTAAGCAR + 5 Carbamidomethyl (C)
☑	2261	1168.37	2334.73	2335.05	-0.32	2	2	3.9e+02	1	SSGARAVGSGPVCGAGARDGHSEHG + Carbamidomethyl (C)
☑	162	589.85	588.84	588.31	0.53	0	2	6.8e+02	1	DVIDK
☑	2090	683.30	2046.87	2047.12	-0.25	0	2	2.7e+02	1	LTYLLCFPLDGGPSLVALR

☑	2192	1095.85	2189.68	2189.12	0.56	1	2	4.1e+02	1	KLAHDPLPLWCLWQDAQR
☑	307	662.40	1984.17	1984.03	0.14	0	2	9.5e+02	1	QQTDIIHQLTVDLDFAK
☑	1320	1142.23	2282.45	2281.94	0.51	1	2	8.4e+02	1	FGEEYEREGGAMGSMSGLMGR + 2 Oxidation (M)
☑	2370	906.29	2715.84	2716.31	-0.47	0	2	3.9e+02	1	LLCDQLHAYGYVALPYYTDLEEK
☑	1176	1073.88	2145.74	2146.13	-0.39	2	2	8.5e+02	1	SRNLIKMTDEINTILEEK
☑	2342	863.51	2587.51	2587.26	0.25	2	2	2.4e+02	1	WCVTTRQRGSAPSGAPSTDGEAVVR
☑	2452	1077.05	3228.13	3227.57	0.56	2	2	3.3e+02	1	ASAPSAPLTPIPSLRCRSCCSMPAPSSSLAR + 2 Carbamidomethyl (C)
☑	370	701.32	1400.62	1400.71	-0.09	1	2	9.5e+02	1	LGFMCRVFISR + Carbamidomethyl (C); Oxidation (M)
☑	800	923.64	2767.90	2768.31	-0.42	0	2	1e+03	1	SSRPGSSLAAHSEPDLSSSANASPLMGR
☑	1442	1202.66	3604.96	3604.44	0.51	0	2	8.4e+02	1	GGTSPDTVSYSYDMTVGENGPGEWGDSEEFSTCK + Oxidation (M)
☑	2301	810.75	2429.22	2429.19	0.03	0	2	2.6e+02	1	LGHVADTTNGGSSISGIPHEEPVR
☑	1142	530.29	1058.57	1058.51	0.06	0	2	3.6e+02	1	GCHVGNFLR + Carbamidomethyl (C)
☑	1168	1070.23	3207.68	3207.55	0.13	0	2	9.4e+02	1	DVSNYINIGVDEVPGNTYYANQLASHINK
☑	1485	1228.43	3682.26	3681.72	0.54	2	2	9.9e+02	1	VQDAEDNDMVSGFSGMMTSINNPEARSNLLGRVK
☑	66	517.22	516.21	516.27	-0.05	0	2	1.5e+02	1	DVGAR
☑	2366	903.36	2707.06	2707.30	-0.23	1	2	2.5e+02	1	GTLLSLYVCRQGNCGIDHVQDFR + 2 Carbamidomethyl (C)
☑	593	821.83	2462.46	2462.05	0.41	2	2	1.1e+03	1	VAHRFYRQHCTACGDGMYMK + Carbamidomethyl (C); 2 Oxidation (M)
☑	876	952.59	2854.74	2854.51	0.23	2	2	8.7e+02	1	HSIYAARGTVDLVLVVPEGRISMESR
☑	1513	1242.75	3725.22	3725.73	-0.52	2	2	8.1e+02	1	DRRIESDLYPVTLGGYSLLEFCCYHGAVDCFK + Carbamidomethyl (C)
☑	1144	1059.84	2117.66	2117.07	0.59	1	2	9.8e+02	1	TWPLELIDFPVNNTMRR + Oxidation (M)
☑	1532	1259.23	3774.67	3774.81	-0.14	0	2	9.8e+02	1	NVFSGCTSLTYVNIPSTVLSLPDHLFENCCFLK + 2 Carbamidomethyl (C)
☑	1619	1329.48	3985.42	3984.96	0.46	2	2	8.7e+02	1	DVWIDLNLSKDHAREHPLENAICELVANALDASEK + Carbamidomethyl (C)
☑	2208	742.53	2224.57	2224.30	0.27	1	2	5.1e+02	1	LVVSKHPLLNLFIINVDYK
☑	585	817.27	816.26	816.42	-0.16	0	2	3.9e+02	1	VDDIDIK
☑	1295	1131.73	1130.72	1130.54	0.19	0	2	9.7e+02	1	EDEFVLHSR
☑	2242	766.40	2296.17	2296.16	0.01	0	2	2.8e+02	1	EVLFEMNHVLEDMAIHLLK + Oxidation (M)
☑	381	707.74	2120.20	2120.20	-0.00	2	2	9.4e+02	1	EAYRVLKVGGVLNVLEFSK
☑	2020	638.59	1912.75	1912.98	-0.24	1	2	2.7e+02	1	AAGDQCTPLRVALDLWK + Carbamidomethyl (C)
☑	1031	507.21	1012.41	1012.48	-0.07	1	2	2.6e+02	1	GSSHKNNNR
☑	571	810.86	2429.55	2429.22	0.33	1	2	1.1e+03	1	LSENDEIQNIIKIMGLDPMEK
☑	1303	1133.99	3398.95	3399.49	-0.55	2	2	1e+03	1	GAAALQSQMFRCTHSVQSRAMGCQMTMHAR + Carbamidomethyl (C); 3 Oxidation (M
☑	2475	1129.37	3385.09	3385.61	-0.51	1	2	3.2e+02	1	CIGGGILACGTTHTAVCPLDVVKCNMQVSPER + 2 Carbamidomethyl (C)
☑	1024	1010.68	3029.02	3029.37	-0.35	1	2	9.9e+02	1	GRGSFVSGYDDAPYFEPMNISSPFFSR
☑	2472	1124.53	3370.57	3370.53	0.04	2	2	2.2e+02	1	ALYMEYSRSNGAFVGRVTNGGEHMLCFSSR + 2 Oxidation (M)

☑	576	812.94	811.94	812.40	-0.46	1	2	1.1e+03	1	HSSGNRR
☑	1899	856.92	1711.82	1711.94	-0.12	1	2	2.9e+02	1	EIEENLKIQIENIK
☑	1228	1099.90	3296.68	3296.65	0.03	2	2	8.6e+02	1	HIAVDMNAPFAVHAGSCVWRLLSSSVAMRR + Oxidation (M)
☑	728	886.85	2657.51	2657.46	0.05	2	2	9.2e+02	1	GTLEFALTPALLDRWLELAKLCK + Carbamidomethyl (C)
☑	1335	1150.15	3447.43	3447.71	-0.28	2	2	1e+03	1	AEDKGDHYVLNGNKFWITNGPDADVLVVYAK
☑	2285	1191.64	2381.26	2381.21	0.05	2	2	2.6e+02	1	TCRSLIFKRPNVCVCVCVWR
☑	864	946.25	1890.49	1891.09	-0.60	2	2	1.2e+03	1	KSNQPAKALDVAIPAQLK
☑	958	982.51	2944.52	2944.53	-0.01	1	2	8.4e+02	1	DLLHVGMSLEIQELIDLYKMNVIDK + Oxidation (M)
☑	464	760.61	2278.82	2278.90	-0.08	1	2	1.3e+03	1	FMCFCCANHTMTSYFKEK + Carbamidomethyl (C); 2 Oxidation (M)
☑	1153	1064.87	3191.60	3191.52	0.08	0	2	1.1e+03	1	STVCVHSFLLTLAAGESFFLLPYCCGSR + 3 Carbamidomethyl (C)
☑	1118	1050.07	3147.18	3147.74	-0.56	2	2	1.2e+03	1	RMRDEIPLSVPMLALLIPLAQSVAEELK + Oxidation (M)
☑	923	972.08	2913.22	2913.52	-0.30	2	2	1e+03	1	TIYCIGIGDPCKNLAARYQISFIIK + 2 Carbamidomethyl (C)
☑	1230	550.61	1099.20	1099.49	-0.29	0	2	4.6e+02	1	DPTTNFYSR
☑	492	772.18	2313.52	2314.12	-0.59	0	2	1.3e+03	1	CLDFTQELGSIVSFDLVSER + Carbamidomethyl (C)
☑	1288	1126.83	2251.65	2251.11	0.53	2	2	1e+03	1	HRMPFPLAAFSTTPTCFRR + Oxidation (M)
☑	1361	1158.89	3473.65	3473.56	0.09	1	2	9.4e+02	1	AWDSSMNLMPAFPTWNVMGMMNNPWYRVK
☑	2243	766.43	2296.27	2296.21	0.06	2	2	2.9e+02	1	GHLEIVKNLISLGADKEAMDK + Oxidation (M)
☑	2489	1183.67	3547.99	3547.73	0.27	1	2	2.2e+02	1	SLFESCLLLPEAQGMQHLLCASQRTSVSWEK + Carbamidomethyl (C)
☑	1026	1011.17	3030.49	3030.29	0.20	1	2	8.7e+02	1	VADEEIDSVEDGDITTEGSMTGMNGRMR + Oxidation (M)
☑	350	688.74	2063.19	2063.08	0.11	2	2	1.1e+03	1	CIKLLIRTQEGAQDAYGK + Carbamidomethyl (C)
☑	1416	1187.42	1186.41	1186.60	-0.19	1	2	1.1e+03	1	GFMDHVRGLR
☑	547	400.63	799.24	799.45	-0.21	1	2	2.9e+02	1	ARGPLMR
☑	1088	1037.83	2073.64	2073.95	-0.30	0	2	9.3e+02	1	STGSNNGANNSTPAGAPSPFAR
☑	105	546.39	545.39	545.30	0.09	1	2	2e+02	1	KCPAK
☑	1235	1101.77	2201.53	2202.10	-0.58	2	2	1.1e+03	1	SALKGKHQPAVGSCDSVCFLR
☑	1352	1154.42	1153.41	1153.43	-0.02	0	2	9.4e+02	1	GSCSPCIDNCR
☑	2279	792.62	2374.85	2375.21	-0.35	0	2	3.4e+02	1	IEAKPQSYIPSQTQSQQVSTR
☑	635	842.48	2524.43	2524.11	0.32	1	2	8e+02	1	YIGPGGVCHFCGAKGHTEAECFK + 2 Carbamidomethyl (C)
☑	915	969.72	2906.15	2905.56	0.59	2	2	1.1e+03	1	IPIRPRDITNGAIVAACNTVNGEERVK
☑	943	977.89	2930.65	2930.51	0.14	2	2	1e+03	1	LASQEALQEKAAMRQALEVTTTQLER + Oxidation (M)
☑	2385	938.09	2811.24	2811.24	-0.01	1	2	2.5e+02	1	GRVIEENMMLPVWTDGCSIESCNGR + Oxidation (M)
☑	912	485.02	968.02	967.53	0.49	0	2	4.9e+02	1	VVKPSVPQD
☑	2251	1157.22	2312.43	2312.17	0.26	2	2	3.5e+02	1	CAFQRTAPALYDEAMKGSLLK
☑	1038	1016.87	3047.59	3047.43	0.17	1	2	1e+03	1	YSKECQLDEWMLRPNSFALNPYTSR

☑	861	944.24	1886.47	1886.02	0.45	1	2	1.3e+03	1	EYVSKLIQFENYIIK
☑	1092	1039.33	3114.97	3114.54	0.42	2	2	1.1e+03	1	MKGSASKGNTIAFGDYALQATEPVWLTSR + Oxidation (M)
☑	589	818.82	2453.43	2453.18	0.25	1	2	1.1e+03	1	MQWIKSVYNDIVSTELDDGPK + Oxidation (M)
☑	2269	784.29	2349.86	2349.27	0.59	1	2	3.1e+02	1	GRSQVIVMAATNRPNAIDPALR
☑	364	700.14	2097.39	2097.06	0.33	1	2	1.2e+03	1	NYGLAHGIGRSGDILSDQPK
☑	799	923.06	2766.16	2766.23	-0.07	1	2	1.1e+03	1	HVSWLKENNACTFGELGCQCATGK + 3 Carbamidomethyl (C)
☑	1304	1134.21	3399.60	3399.84	-0.23	2	2	1e+03	1	AVSMVVPDYSLIAEIILFSEGFTTAKVLSRK + Oxidation (M)
☑	920	971.90	2912.67	2912.58	0.08	1	2	9.3e+02	1	YGFAGVVLRGIDYPYFLSQPLLPSIK
☑	1330	1147.93	3440.77	3440.68	0.08	2	2	9.3e+02	1	DNVSVTIVQAIEVFSNSRRMICTPTVDSMR + Carbamidomethyl (C); Oxidation (M)
☑	240	626.04	1875.11	1874.89	0.21	0	2	9.1e+02	1	EGSEGSVVEVSVQLISCGP
☑	445	745.78	2234.32	2234.15	0.17	0	2	1.2e+03	1	ETTMISLQVTATQIEEVVSR
☑	1595	1302.66	2603.30	2603.24	0.06	2	2	8.1e+02	1	LHAADLQLMQRRMGDNTDTVMR + 2 Oxidation (M)
☑	2012	636.27	1905.78	1905.75	0.03	1	2	3.2e+02	1	CSPCKIDSCFECTSNR + 2 Carbamidomethyl (C)
☑	501	776.25	2325.73	2325.19	0.53	2	2	1.2e+03	1	MSISLFIGRAASSTPIGASCKR + Carbamidomethyl (C); Oxidation (M)
☑	687	868.77	1735.52	1735.89	-0.37	2	2	9.3e+02	1	SAKTATPLSERSFADR
☑	1490	1229.84	2457.67	2458.09	-0.42	0	2	1e+03	1	LFDLNDDVMDISASMGMAMPTGK
☑	2216	1123.84	2245.67	2245.16	0.51	1	2	5.1e+02	1	GSPGVSALDPSDVRAAVAQGHVR
☑	556	804.63	2410.87	2411.32	-0.45	2	2	1.3e+03	1	LGIPMPKGVLLYGAPGCGKSAVAR + Carbamidomethyl (C)
☑	443	745.26	2232.76	2233.02	-0.26	0	2	1.1e+03	1	FSQGLQAPPESLIMQATADDD
☑	2458	1091.31	3270.89	3270.44	0.46	1	2	2.6e+02	1	ARGYDMYGGQSHLCSTTLCGHGSGNSAGVGVR + Carbamidomethyl (C); Oxidation (M
☑	645	423.58	845.15	845.39	-0.24	0	2	4.5e+02	1	EGGAVEER
☑	144	576.71	575.70	575.27	0.43	0	2	4.2e+02	1	ATNDR
☑	846	936.71	935.71	935.41	0.30	1	2	3.2e+02	1	DRCAQMGR
☑	57	511.20	510.20	510.17	0.02	0	2	11	1	CSSNT
☑	82	533.02	532.01	532.25	-0.23	0	2	1.2e+03	1	AQLSD
☑	623	838.65	837.64	837.37	0.28	0	2	3.7e+02	1	GGVTSMDR + Oxidation (M)
☑	669	854.58	2560.71	2560.38	0.33	2	2	1.1e+03	1	NIAVQLFKFLEHYRPETKAEK
☑	1023	505.70	1009.40	1009.59	-0.20	1	2	2.9e+02	1	HVEKILGSK
☑	904	482.21	962.42	962.43	-0.02	0	2	3.4e+02	1	DPQMVMSR
☑	2095	1026.90	2051.79	2051.98	-0.19	1	2	3.1e+02	1	VQTFQDGVSRESLDENTK
☑	2397	968.21	2901.60	2901.49	0.11	1	2	2.7e+02	1	RLGGNNGTDVIPLMVVPLMLDLMDFR + Oxidation (M)
☑	891	958.55	2872.62	2872.22	0.39	1	2	9.6e+02	1	GPVCCLSVCPGNDEKYLVSGGSDGSCR + 3 Carbamidomethyl (C)
☑	1212	1091.66	1090.65	1090.55	0.10	0	2	9.9e+02	1	DDILEYPVK
☑	190	602.51	1203.00	1202.53	0.47	0	2	1.5e+03	1	HQTLHSCCFK

☑	239	625.86	1874.56	1873.97	0.60	2	2	9.9e+02	1	QQRMLQDDILEVKSR + Oxidation (M)
☑	1016	1008.91	3023.70	3023.38	0.32	2	2	1.2e+03	1	KVVMSAPSKDDTPMFVMGVNHEEYHK + 3 Oxidation (M)
☑	183	600.58	1798.72	1798.98	-0.26	2	2	1.2e+03	1	RWQDLSNNVAILSKR
☑	1065	1029.12	2056.22	2055.99	0.23	1	2	1.1e+03	1	QESDIFFCIAGKQGEGVTK
☑	1009	503.84	1005.67	1005.54	0.13	0	2	3.5e+02	1	ASAHIHGSVK
☑	1015	1008.58	2015.15	2015.02	0.12	0	2	1.1e+03	1	QLNVEDVELSPNGDLFVK
☑	1424	1190.24	3567.69	3567.61	0.08	1	2	1e+03	1	MMVEDTHMTMTGFSDVTSLNYSQRNLLAVGR + 4 Oxidation (M)
☑	601	825.18	1648.35	1647.76	0.59	0	2	1.2e+03	1	EWYESGAVNGVPANR
☑	867	947.71	1893.40	1893.90	-0.50	0	2	1.2e+03	1	DDVVAFFDTEYLAVYK
☑	1007	1005.71	3014.10	3014.48	-0.38	2	2	1.2e+03	1	HNQYYFSSYKSTSLLTEYTVSKVNR
☑	2447	1065.92	3194.75	3194.51	0.23	0	2	2.4e+02	1	NLMQEFSNILCLFEALSIMGFDTWGVK + Carbamidomethyl (C); 2 Oxidation (M)
☑	1055	1024.33	2046.64	2046.06	0.59	1	2	1.1e+03	1	TPEYFGFLRSVFQISQK
☑	1404	1180.73	3539.16	3539.61	-0.45	2	2	1e+03	1	YTNIDIYEAVCSAAGEGRLVEKEDEHSDDVR + Carbamidomethyl (C)
☑	1451	1209.69	2417.36	2417.39	-0.03	2	2	8.5e+02	1	GSDLLLQSLLVRVLTDIRHNR
☑	347	687.69	2060.05	2060.13	-0.08	2	2	1.2e+03	1	FRNGMVARVVVDLAAATVR + Oxidation (M)
☑	2193	731.80	2192.38	2192.12	0.26	1	2	3.4e+02	1	EELGGSIASFPTGVVNFQRGK
☑	184	600.77	1799.28	1798.88	0.39	1	2	1.1e+03	1	YEGIDVRDVSTLNYR
☑	1050	1022.30	2042.59	2043.09	-0.50	2	2	1.3e+03	1	RLTESLQNVNDRIWTAK
☑	393	714.44	713.44	713.42	0.02	1	2	3.1e+02	1	KGVTGPR
☑	1093	1039.35	3115.03	3114.54	0.49	1	2	1.2e+03	1	FGIMCVGTTPFESSSVPYVRVNLYVHR + Carbamidomethyl (C)
☑	2325	1256.76	2511.50	2512.10	-0.59	2	2	3.3e+02	1	MKYLEMGGGAAADALRDGDADDDR
☑	1652	1374.05	4119.12	4119.11	0.02	2	2	9.1e+02	1	ILLEDCTRITTLAALASIHTISEVNVCGCSGVKSFLSTP + Carbamidomethyl (C)
☑	67	518.50	1034.99	1034.56	0.42	0	2	1.1e+03	1	TIPVVTDYK
☑	368	700.89	699.88	700.36	-0.48	0	2	7e+02	1	FYIMK
☑	1409	1182.77	2363.53	2363.17	0.36	2	2	1e+03	1	REAHADGGNALLASMYPAFKTK + Oxidation (M)
☑	1635	673.67	1345.33	1345.71	-0.38	1	2	5.6e+02	1	YVALFKFCIDK
☑	2313	829.39	2485.15	2485.24	-0.09	1	2	3e+02	1	DVLSTKGMQSTVETVPWIHWR + Oxidation (M)
☑	2388	945.97	2834.87	2835.40	-0.52	1	2	4.8e+02	1	IQSGGGFGYLRAATATPDLAPSSAGPSMR
☑	1396	1175.14	3522.41	3522.75	-0.34	2	2	1.3e+03	1	WHAIKNTFVTTLEVRSSAQEFAMIVDWMGR
☑	604	826.75	2477.21	2477.24	-0.03	1	2	9.7e+02	1	SSSGVRCQTTASSAVHFNLVTLR + Carbamidomethyl (C)
☑	1556	1281.12	3840.34	3840.84	-0.49	2	2	1.2e+03	1	ALLVFGEQKGEKPGGTAMESHADAEMFTAMVRNFR + Oxidation (M)
☑	1106	1045.68	3134.01	3133.49	0.51	1	1	1.1e+03	1	FAMVMTGSETLLEQSSHWKEVLYMLR + 3 Oxidation (M)
☑	1445	1203.58	1202.58	1202.59	-0.01	1	1	9.3e+02	1	SAKDAENAELR
☑	2460	1095.75	3284.22	3284.69	-0.47	2	1	3.4e+02	1	MRLSTLALVQLDDREVVNDVMPLEPIMR + 2 Oxidation (M)

☑	737	892.61	2674.80	2674.29	0.51	2	1	1.2e+03	1	QLSCVYGDKGVPESLVMRMGGYGTK
☑	1197	1084.62	3250.85	3250.33	0.53	2	1	9.7e+02	1	LMETLCQCGYRTPQGSNKEGDGTEQADSC + 2 Carbamidomethyl (C); Oxidation (M)
☑	1869	840.17	1678.32	1678.80	-0.48	0	1	6.1e+02	1	YSSFAEDQFVAFIR
☑	2288	1193.41	2384.80	2385.09	-0.29	0	1	4.3e+02	1	ESYNNIPNVAIMTAANCSTTSK + Carbamidomethyl (C)
☑	2429	1031.05	3090.13	3089.59	0.55	2	1	3.8e+02	1	MVVQALNGSILKLICCKGECIGVPSTAR + 3 Carbamidomethyl (C); Oxidation (M)
☑	419	730.76	1459.52	1459.74	-0.23	0	1	1.2e+03	1	NDAFVANVVQEVR
☑	422	731.39	2191.16	2191.10	0.06	0	1	1.2e+03	1	GDPGALPPAASSLSVFQHTSPR
☑	2468	1108.06	3321.14	3321.72	-0.58	1	1	3.9e+02	1	MSIVTTGMGTGSTEIIINEILALKCIDIETR
☑	564	807.78	2420.32	2420.24	0.07	2	1	1.2e+03	1	DLIAELRECLTQRTLEMTASK
☑	1149	1062.35	3184.04	3183.49	0.55	2	1	1.2e+03	1	HACNMKTDSSGTAVLVESAGSGAFGTMVQRR + Oxidation (M)
☑	1499	1235.73	3704.16	3704.00	0.16	2	1	9.3e+02	1	TAGTIIGGVLGTKAGNPAMGAQAGNAVGGIAEGILNKIGGK
☑	2177	724.90	2171.67	2171.24	0.43	2	1	5e+02	1	DLRLPLGINSLKEAIHASPK
☑	515	787.87	2360.60	2360.27	0.33	2	1	1.6e+03	1	FSRNGRALDSTVLLACLNVVR + Carbamidomethyl (C)
☑	2097	1027.92	2053.84	2053.95	-0.12	2	1	3.2e+02	1	GMGSAAASRGSADCTQKEALK + Oxidation (M)
☑	2234	761.74	2282.19	2282.35	-0.15	0	1	2.9e+02	1	MDIITLICSLVILGAIAVLVR + Carbamidomethyl (C)
☑	272	646.47	1290.92	1290.65	0.27	0	1	1.4e+03	1	DPAALGITEYNK
☑	2113	1040.99	2079.96	2079.94	0.02	2	1	4.7e+02	1	GGMGMDVGAGASAEGVGSDRKR + Oxidation (M)
☑	890	958.52	2872.53	2872.38	0.15	2	1	1e+03	1	YVLTPSDKEYAPKYSYSSDELYVR
☑	896	960.30	2877.87	2877.44	0.43	2	1	1.4e+03	1	DSVPRVMGAMGVGLGYICMPPAKLVER + 2 Oxidation (M)
☑	59	512.79	511.78	512.23	-0.45	0	1	24	1	DGGHK
☑	933	975.17	974.17	974.41	-0.24	0	1	1.3e+03	1	QNNNDENK
☑	2100	686.54	2056.60	2056.99	-0.39	2	1	5.5e+02	1	ASAARAAMMKDSSSATGTVTK + Oxidation (M)
☑	2331	845.46	2533.36	2533.12	0.23	2	1	2.8e+02	1	GTDHRGSLVADTSPECSSDSSGRR + Carbamidomethyl (C)
☑	1653	1374.09	4119.24	4119.32	-0.08	1	1	9.8e+02	1	LVLVCPTPLSSLTAMTNAVVRRPLVDGYQLPDIVVLLK + Oxidation (M)
☑	1710	481.56	1441.66	1441.67	-0.01	2	1	3.4e+02	1	RNEEMHQKNEK
☑	1797	782.81	1563.61	1563.84	-0.23	2	1	3.3e+02	1	AIYSEVQKRSLDR
☑	322	673.83	672.82	672.36	0.46	1	1	9.4e+02	1	TRDGPK
☑	2231	760.69	2279.05	2279.19	-0.14	1	1	3.3e+02	1	GLSVQERVVVCICLAGFPFSR
☑	2255	1159.40	2316.78	2317.19	-0.41	2	1	4e+02	1	GKLMIDTDDEITRLENIGIR + Oxidation (M)
☑	199	608.18	1821.52	1821.92	-0.39	2	1	1.3e+03	1	TAENFRALCTGEKGLGR
☑	1333	1149.56	3445.65	3445.55	0.10	1	1	9e+02	1	DCCGCILTDKILQQYDFNGFTFTVCIDWK + Carbamidomethyl (C)
☑	1398	1175.40	2348.79	2348.20	0.59	2	1	1.2e+03	1	DLTKTAVISGGMIPKVNCCIR + 2 Carbamidomethyl (C); Oxidation (M)
☑	2329	842.45	2524.32	2524.43	-0.12	2	1	3e+02	1	LKEGIKLSLSMALAAYIQTYALK
☑	1030	1013.39	2024.77	2024.97	-0.20	2	1	1e+03	1	RSRADSTASAATSAAASAMSR

☑	1068	1030.29	2058.56	2058.05	0.51	2	1	1.3e+03	1	DIMALHDKILEMDNKIK + 2 Oxidation (M)
☑	1464	1215.45	1214.44	1214.66	-0.22	0	1	1.1e+03	1	TLAGELLQAGSR
☑	2071	672.42	2014.23	2013.99	0.24	0	1	3.4e+02	1	YIFENSTWTHVVYSLR
☑	2491	1203.34	3606.99	3606.74	0.25	2	1	2.3e+02	1	KEAEGCDCLQGFQICHSLGGGTGAGMGTLLIAKIR + Carbamidomethyl (C); Oxidation
☑	2451	1076.35	3226.04	3225.56	0.48	0	1	4e+02	1	AAAAPGASLGSNGQLEAKPQSVNSSSAANSAQER
☑	1810	788.99	1575.96	1575.83	0.13	2	1	3.7e+02	1	VISCLKDERVEASK
☑	63	514.81	1541.40	1541.75	-0.35	0	1	9.4e+02	1	QPSTDPFAAPQQQK
☑	1273	1119.96	3356.85	3356.44	0.41	0	1	1.1e+03	1	MSLYIFCSSAGATPACTSSSCSFEITSMLSSR + 2 Oxidation (M)
☑	2109	691.64	2071.91	2071.93	-0.03	1	1	3.5e+02	1	DAICKLLMAMATMCQQR + 2 Carbamidomethyl (C); 2 Oxidation (M)
☑	2438	1047.60	3139.78	3139.55	0.24	1	1	2.7e+02	1	SDLAVNKFNLELETESLEQMMVIFPDK
☑	311	664.20	1989.59	1989.01	0.58	2	1	1.3e+03	1	MMRRSLFQGVYTLCLR + Oxidation (M)
☑	679	860.83	2579.46	2579.25	0.21	2	1	1.2e+03	1	DSRVVLGAGCAETAMANAIEKAAMK + Carbamidomethyl (C); Oxidation (M)
☑	2477	1135.86	3404.56	3404.64	-0.09	2	1	2.6e+02	1	NLNAGQQCISPDFVLCHEAVLDKFLSECRR
☑	451	751.78	2252.32	2252.08	0.25	0	1	1.3e+03	1	LSAELLMESDTTGGSRPMSVR + Oxidation (M)
☑	1124	1053.81	3158.41	3158.41	-0.00	1	1	1.1e+03	1	DTAPGQSMCTQASEGKCTEAAPGYFLNPLR + Oxidation (M)
☑	1229	1100.17	3297.48	3297.63	-0.15	1	1	1.2e+03	1	VMVTAGLDQEVKMWNIEQLQCIGTIGFMK + Oxidation (M)
☑	499	774.93	2321.76	2322.32	-0.55	2	1	1.7e+03	1	IGRLIAWTNKSITVLAAPENR
☑	602	825.86	824.85	825.36	-0.51	0	1	4.3e+02	1	SNHDEPK
☑	298	658.65	1972.92	1973.16	-0.24	0	1	1.4e+03	1	IAYIHLLIGTHAAIAIQR
☑	476	765.34	2292.99	2293.16	-0.17	2	1	1.1e+03	1	TLRSQQQMHLNSTSKISYR + Oxidation (M)
☑	1066	1029.25	2056.49	2056.02	0.47	2	1	1.3e+03	1	MHSCVLSGHRIGRVEYR + Carbamidomethyl (C)
☑	809	927.08	2778.21	2778.62	-0.41	2	1	1.2e+03	1	MVLVRYCLFLRIWALLLLSLTSR
☑	2469	1115.80	3344.36	3344.75	-0.39	1	1	2.8e+02	1	GDSYIGVEIIDVSAPILNTSAKLIEAAQAQMK
☑	941	977.64	2929.91	2930.46	-0.55	1	1	1.2e+03	1	VSQNSDFSIKPFSLQKMFTESQINR
☑	1313	1138.33	2274.64	2275.23	-0.59	2	1	1.2e+03	1	MWGLSAKLVAFQILTNPKCR
☑	2070	1008.01	2014.01	2013.97	0.04	1	1	3.5e+02	1	VLGQMINEGVRQHAMGDK + 2 Oxidation (M)
☑	838	935.17	1868.33	1868.06	0.27	1	1	1.4e+03	1	MTQTIPNKPIILSKER
☑	868	947.80	2840.39	2840.45	-0.06	1	1	1.1e+03	1	LNLPQANPGLHMSFTGSPGTGKTTVATK + Oxidation (M)
☑	1636	1347.62	4039.84	4039.94	-0.10	1	1	8.8e+02	1	LKTDLDAETEAHCTSLIDCLLETAYSLMVQDAAVVSR + Oxidation (M)
☑	746	895.95	894.94	895.51	-0.57	2	1	1.5e+03	1	YSVKKSGK
☑	2398	973.24	2916.68	2917.26	-0.58	0	1	3.2e+02	1	SINIPSSITSFGNYCFYGCGCEEELK + Carbamidomethyl (C)
☑	485	769.14	2304.40	2304.13	0.26	1	1	1.3e+03	1	YVATLPRHAHHYNEFFFR
☑	1341	1151.88	2301.75	2302.18	-0.43	2	1	1.2e+03	1	EPVTSAPFQPRDPTATPHRAK
☑	1641	681.07	1360.12	1360.70	-0.59	2	1	6.4e+02	1	VCVLKKDGEGEK + Carbamidomethyl (C)

☑	2007	950.22	1898.42	1898.98	-0.57	0	1	6.1e+02	1	AELNPDLVDEGVSTSILK
☑	1328	1146.55	3436.62	3436.52	0.10	1	1	1.1e+03	1	YALCARPSSFEQSPNRDTGMYIGMPQSDSR + Carbamidomethyl (C); Oxidation (M)
☑	1466	1217.15	3648.42	3648.91	-0.49	1	1	1.4e+03	1	VMNYFKQIVMGVSTIHQHCIIHGDLKPGNILK + Oxidation (M)
☑	1638	675.88	1349.74	1349.68	0.07	0	1	3.6e+02	1	VHIMSVTPDSHK
☑	2373	909.86	2726.55	2726.41	0.14	1	1	3.4e+02	1	LIIAKAPASHSTISNATAGIGMEMQAK + Oxidation (M)
☑	2049	661.36	1981.06	1981.03	0.03	0	1	3.6e+02	1	ANVLSPLPPLETGAAAAMSR + Oxidation (M)
☑	391	712.79	711.78	711.37	0.42	0	1	2.9e+02	1	THGLER
☑	899	961.98	2882.93	2882.42	0.51	0	1	1.7e+03	1	ILCSAVLNNSMVISADAGSLLALEFMQ + Carbamidomethyl (C); Oxidation (M)
☑	1393	1172.54	2343.07	2343.15	-0.08	2	1	9.9e+02	1	EKRSALLLLMYHAMFEGMR + 3 Oxidation (M)
☑	1870	840.22	1678.42	1678.87	-0.45	1	1	6e+02	1	QFESLDNIRVLMAK + Oxidation (M)
☑	748	899.89	898.88	898.47	0.42	0	1	4.8e+02	1	MVHVGGGVK + Oxidation (M)
☑	24	462.42	1384.25	1383.65	0.59	1	1	1e+03	1	EPISFDMVMKR + 2 Oxidation (M)
☑	587	817.62	2449.82	2450.11	-0.29	1	1	1.5e+03	1	MFCVSCMAGTKQQEPLFHHTR
☑	1950	904.89	1807.77	1807.78	-0.00	0	1	3.6e+02	1	NDDVILTSDTCSDEPK + Carbamidomethyl (C)
☑	1305	1134.89	3401.66	3401.67	-0.01	2	1	1.1e+03	1	MPSSTKSSADQASSEVQEVLINGKLYDVSGFK
☑	449	747.68	746.68	746.42	0.26	1	1	4.9e+02	1	EETIKK
☑	1297	1131.98	2261.94	2262.17	-0.23	2	1	1.2e+03	1	ALKKCPSSTLLWMEFLSHR + Oxidation (M)
☑	726	886.02	2655.04	2655.03	0.00	0	1	1.3e+03	1	EECDGGINCDETCHCLPGYIQDK + 2 Carbamidomethyl (C)
☑	1192	1081.23	2160.45	2160.93	-0.49	0	1	1.1e+03	1	MLSSTNGVCDIASACACKPMK + 2 Oxidation (M)
☑	2146	1063.16	2124.31	2124.01	0.30	1	1	3.5e+02	1	TCVCVCVCVCVQGGQKLIR + 2 Carbamidomethyl (C)
☑	314	668.76	2003.27	2002.90	0.37	2	1	1.2e+03	1	DGSLSARRQAMGWMYDK + 2 Oxidation (M)
☑	810	927.59	2779.75	2779.48	0.26	2	1	1.2e+03	1	IDTLPQARNIAADMDLPRGALYPLR
☑	1174	1072.40	2142.79	2143.15	-0.36	0	1	1.2e+03	1	SSGYQIMVTLLIMIFCVIL
☑	1491	615.79	1229.56	1229.60	-0.03	1	1	3.7e+02	1	LSCFSRSAFR + Carbamidomethyl (C)
☑	345	686.97	685.96	686.40	-0.43	0	1	7.4e+02	1	LLAAGDK
☑	533	796.11	2385.30	2385.18	0.12	0	1	1.2e+03	1	CVYISPPPSHSFAAGEAVAVDLR
☑	1073	1032.51	2063.01	2063.02	-0.01	2	1	1.1e+03	1	ECISLVSSMKYNSFATRK
☑	1227	1099.82	3296.44	3296.50	-0.07	1	1	1.2e+03	1	NMALVSYDNEKNPYYQCIDLSMTNPITK + 2 Oxidation (M)
☑	426	732.34	1462.66	1462.68	-0.02	0	1	1.3e+03	1	AGWGPGGCAFASLNR
☑	840	935.46	2803.35	2803.44	-0.09	2	1	1.2e+03	1	TQVLVNRSSGGEKCPFQTAVLAGTQR + Carbamidomethyl (C)
☑	1391	1171.86	3512.56	3512.72	-0.16	1	1	1.3e+03	1	GNLFAFGGHLIEIFQHFFDMAFTYGFRFIA
☑	2076	675.36	2023.05	2022.90	0.15	1	1	3.4e+02	1	MSCTFGGNTTAVQEMFKR + Oxidation (M)
☑	668	854.08	2559.22	2559.40	-0.18	1	1	1.1e+03	1	MNFVNFVTGSSKLPIGGLANLRPK
☑	1054	1024.20	2046.38	2046.18	0.20	1	1	1.1e+03	1	IYALVASIHPLRIYVYR

☑	1326	1143.96	3428.85	3428.55	0.30	2	1	1.2e+03	1	QLRSYSTFYYNFIKNIPCPSGYDCSDYK + Carbamidomethyl (C)
☑	2371	906.34	2716.01	2716.33	-0.32	1	1	3.9e+02	1	WHQIAFMSIDAEEVERVVGDTLR + Oxidation (M)
☑	712	879.78	1757.54	1757.83	-0.29	2	1	1.2e+03	1	QGGGNNDEKVNQEKNK
☑	1039	1016.89	3047.65	3047.54	0.10	2	1	1.2e+03	1	GCESLKNVILPNLTIANDNAFKGCESLK + Carbamidomethyl (C)
☑	1529	1257.72	3770.14	3770.11	0.04	2	1	9.7e+02	1	DFLCGLLVSITPQTPRSLQEAVIDAVGLIYLKLK + Carbamidomethyl (C)
☑	2300	809.36	2425.07	2425.22	-0.15	1	1	3.2e+02	1	MLDVANRPMPKPEMFAAHRAK + Oxidation (M)
☑	1003	502.19	1002.37	1002.47	-0.09	1	1	5.5e+02	1	SYFESKDK
☑	1954	907.35	1812.69	1812.87	-0.19	1	1	3.6e+02	1	VADYLSAKMCNLDVAK + Carbamidomethyl (C); Oxidation (M)
☑	2367	904.55	2710.63	2710.32	0.30	2	1	3.8e+02	1	WVHLACTDKKIVENTQFMFESK + Carbamidomethyl (C)
☑	2372	909.19	2724.54	2724.38	0.16	2	1	2.9e+02	1	AVEEALHCLACRACVERWLLAVR + 2 Carbamidomethyl (C)
☑	2437	1047.46	3139.35	3139.33	0.02	0	1	2.8e+02	1	CGSCVCPHYVYMPNEPEEIGEGWLTR + 3 Carbamidomethyl (C)
☑	738	893.50	892.50	892.55	-0.05	2	1	1e+03	1	RLVKYSK
☑	1296	566.42	1130.83	1130.59	0.24	1	1	4.2e+02	1	MTEGPVRGLR + Oxidation (M)
☑	1579	1295.15	2588.30	2588.46	-0.16	2	1	1.4e+03	1	LKALLSIFGRETPVELEFAQISK
☑	440	742.55	2224.63	2225.18	-0.54	2	1	1.5e+03	1	KEMVDFLISLGVDFNIKNK + Oxidation (M)
☑	982	990.40	2968.18	2968.26	-0.09	1	1	1.2e+03	1	CDGFVPGCAVCTPSPDASGAPVCASCKAGK + 3 Carbamidomethyl (C)
☑	1117	1050.06	1049.05	1049.59	-0.54	1	1	7.6e+02	1	IFPMSLKAK + Oxidation (M)
☑	1494	1232.32	3693.95	3693.87	0.08	2	1	1.3e+03	1	RDWPLSLDVLSTTSWPKLSSCLPAGETQPIPDK + Carbamidomethyl (C)
☑	208	613.92	1838.75	1839.03	-0.28	0	1	1.1e+03	1	DILQIQLTGYGIHLQK
☑	383	708.64	2122.89	2122.91	-0.02	1	1	1.4e+03	1	WMSDGGCGDPSVVEWKEGK + Carbamidomethyl (C)
☑	765	905.91	2714.71	2714.32	0.39	2	1	1.6e+03	1	LPASREEHELIGCECYRPRMIR + Carbamidomethyl (C)
☑	973	987.39	2959.15	2958.66	0.50	0	1	1.2e+03	1	LVPATAIAVAAMALPAAATTTTAAPVPVNLR + Oxidation (M)
☑	1412	592.94	1183.86	1183.67	0.19	0	1	4e+02	1	QSAVFVAPLPR
☑	1521	1253.35	3757.01	3756.85	0.16	2	1	1.2e+03	1	AALEESRQHPVTTTPCAVVEAHSAEVADKDAGAAIR + Carbamidomethyl (C)
☑	1255	1109.57	3325.69	3325.46	0.23	2	1	9.7e+02	1	DNVLCVMQVTSIECVDGDDWDDRQTKER + Carbamidomethyl (C)
☑	1544	1269.43	3805.26	3805.01	0.24	1	1	1.3e+03	1	MGTSSFILLLFCLLDHFCTSSLHNAAKVLLLELR
☑	1223	1097.14	1096.14	1095.58	0.56	0	1	1.1e+03	1	IASGEIPIPEA
☑	1587	1299.51	3895.52	3895.16	0.36	2	1	1.1e+03	1	ELNEFIHRMATDKIIICFYILIMIGIIIFATLK
☑	1592	1301.36	3901.05	3901.14	-0.09	2	1	1.3e+03	1	FLTVLNCLINNKQFLNLSDVEVLKILNESELIR + Carbamidomethyl (C)
☑	2286	1192.39	2382.78	2382.19	0.59	2	1	5.5e+02	1	FSAPNYVAVKNDGTGVMEEVKK
☑	2341	862.80	2585.38	2584.96	0.41	0	1	3.2e+02	1	QWNYGMDEDDGENSGGGNDAPDAR + Oxidation (M)
☑	325	675.17	674.16	674.36	-0.20	1	1	5e+02	1	AKAEEK
☑	448	747.27	746.26	746.39	-0.13	0	1	5.1e+02	1	GQGTSGLK
☑	479	766.70	2297.09	2297.06	0.03	0	1	1.3e+03	1	LCIVVDTSVDEATEICQTACK + Carbamidomethyl (C)

☑	1559	1285.21	3852.61	3852.81	-0.20	2	1	1.3e+03	1	NILMSDNMIPQFTDIGNASKVDNSNELQDIDKNK + 2 Oxidation (M)
☑	1276	1122.01	2242.00	2242.16	-0.17	2	1	1.4e+03	1	SPRYYTHFVMKALIEMLK + Oxidation (M)
☑	1150	1062.48	3184.41	3184.83	-0.42	0	1	1.1e+03	1	VLVFLGAIGALAGMTVLSALMGLVVPSVLSVR + 2 Oxidation (M)
☑	1225	1098.34	3292.01	3291.44	0.57	2	1	1.5e+03	1	DCMDHIGFFAKCLGCSENDAHNYFKTIR + Carbamidomethyl (C)
☑	603	826.41	1650.80	1650.81	-0.01	2	1	9.4e+02	1	VKGKVEASCVAEMER + Oxidation (M)
☑	647	846.46	2536.35	2536.26	0.09	1	1	1.2e+03	1	STIGGDRTDLMFELQQDQIVVR + Oxidation (M)
☑	1048	1021.41	3061.21	3061.61	-0.40	2	1	1.2e+03	1	SFGALMKADTLDLASASVAGLLNKTSLDPR
☑	1062	1028.33	3081.97	3081.45	0.52	2	1	1.4e+03	1	ITMPMMVITMGGRFFDCKDAELTVTMK + Oxidation (M)
☑	1423	1189.53	1188.52	1188.69	-0.17	0	1	1e+03	1	GDLNYLILLR
☑	317	669.86	2006.55	2006.98	-0.42	2	1	1.4e+03	1	DGYLADTPTTVGSGGERRR
☑	1199	1086.14	3255.40	3255.66	-0.26	2	1	1.3e+03	1	KQTTLSLNSEINTSPNLFFSSQNSTELKK
☑	2084	1020.92	2039.83	2039.94	-0.11	2	1	3.7e+02	1	CYKQMNQDTEVPLDRK + Carbamidomethyl (C); Oxidation (M)
☑	1188	1078.94	3233.79	3233.46	0.33	2	1	1.2e+03	1	KHSETTSLERLSESSGGVGHTNSSTECGGSR + Carbamidomethyl (C)
☑	1461	1215.05	3642.12	3642.68	-0.56	2	1	1.3e+03	1	MGSSDDEWQNPPVFPGGRRPGAGGSGENAMLAARR + Oxidation (M)
☑	2254	772.35	2314.03	2314.12	-0.09	2	1	3.7e+02	1	ERFLYCMEGVNRAAAASGEIK
☑	2435	1043.70	3128.07	3127.74	0.33	0	1	5.1e+02	1	AVVAQSTSPVASALGAQASAGGLAVVAALLPQPR
☑	274	646.97	645.96	646.33	-0.37	0	1	1.7e+03	1	EASAAAK
☑	348	687.96	2060.86	2061.10	-0.23	1	1	1.8e+03	1	IMQFFSTVNSLIKTSFAK
☑	648	846.72	1691.43	1690.90	0.53	1	1	1.6e+03	1	VMLITAPVYAKDNEK
☑	1265	1114.62	3340.85	3340.93	-0.08	1	1	1e+03	1	LAARHVPQGSILLRPESVSSALSILLLTPFR
☑	1989	625.96	1874.87	1874.94	-0.07	2	1	3.6e+02	1	GSGSGAVRTVSPQKCGAASR
☑	2151	710.43	2128.27	2128.06	0.21	1	1	4.2e+02	1	IEYYEQSYSKHSLVVQR
☑	577	813.14	2436.40	2436.16	0.24	2	1	1.3e+03	1	RCCDPHLAADQAPAVIERMAR + 2 Carbamidomethyl (C)
☑	757	903.59	2707.73	2708.32	-0.59	2	1	1.4e+03	1	NVLQEIVQTEENLCKNMKLMDK + Carbamidomethyl (C); 2 Oxidation (M)
☑	2309	824.23	2469.66	2469.20	0.46	1	1	5.3e+02	1	LRDGGLTPTADTYAALIACCTSTR
☑	378	706.11	705.10	705.41	-0.30	0	1	4.1e+02	1	EFVLAK
☑	975	987.47	2959.38	2959.63	-0.25	0	1	1.2e+03	1	MAEAFSIISILLLLAATALGVAAHFCLK + Carbamidomethyl (C); Oxidation (M)
☑	990	994.85	2981.52	2981.38	0.14	2	1	1.2e+03	1	KEYNKYVLDILCDTGCGYQGDELTGK + Carbamidomethyl (C)
☑	2083	679.27	2034.80	2035.13	-0.33	2	1	4.3e+02	1	STISKANLSHRVTPLQQR
☑	2257	1162.08	2322.15	2322.23	-0.08	2	1	3.4e+02	1	TQLAVDAVQGQREELGAQPKGK
☑	124	562.71	561.70	562.26	-0.56	1	1	4.4e+02	1	VDDKS
☑	1025	1010.70	2019.39	2019.03	0.36	0	1	1.3e+03	1	QLSVEELVACLTVTTSNGR
☑	1336	1150.16	3447.46	3447.70	-0.25	2	1	1.3e+03	1	CGVAATTATAALVRCCGRAAVISPPLVTACGMQR + 2 Carbamidomethyl (C); Oxidatior
☑	1362	1160.70	3479.08	3478.71	0.37	0	1	1.2e+03	1	FAPPPADRPPTATATAASAAILGVNTSTGGHCCISR

☑	409	724.93	2171.78	2172.02	-0.24	1	1	1.2e+03	1	GYVCRGVGLSYQDFCVPPR + Carbamidomethyl (C)
☑	619	837.08	836.07	836.41	-0.33	1	1	4.1e+02	1	DCKSLSK + Carbamidomethyl (C)
☑	1094	1039.59	3115.74	3115.53	0.20	2	1	1e+03	1	NDTLEILTNCLKMSSDSQKLFAEVGGFR
☑	1507	1239.39	2476.76	2476.21	0.55	2	1	1.3e+03	1	IGEAQRDLCLRLGCMEPQLMK + Carbamidomethyl (C); Oxidation (M)
☑	1709	721.62	1441.23	1441.70	-0.47	0	1	7.3e+02	1	NVVVGGTAEPDQEK
☑	629	840.50	1679.00	1678.76	0.23	1	1	1.1e+03	1	HSGFEKMEIDVESR + Oxidation (M)
☑	2087	682.33	2043.96	2043.98	-0.03	1	1	3.5e+02	1	SCDLNNPTVGAMTAAAAPKR + Carbamidomethyl (C)
☑	1369	1163.02	3486.04	3485.54	0.50	2	1	1.4e+03	1	NMQTDSPSHSPMAQSSTNTAAFANSHCSHRKR
☑	1727	489.92	1466.74	1466.78	-0.04	1	1	4.8e+02	1	RDGIDDGLPLIQR
☑	2104	1034.79	2067.57	2067.11	0.46	2	1	7e+02	1	TGTGLHEGLEWAVKNIKSK
☑	827	932.83	931.82	931.45	0.38	1	1	1.3e+03	1	EADGARASR
☑	1114	1048.71	3143.10	3143.26	-0.16	0	1	1.6e+03	1	QGQGDIDSDVSDDEAPIIGDEDDDTYMGR + Oxidation (M)
☑	1283	1123.72	1122.71	1122.57	0.14	1	1	1.2e+03	1	RVTDTFEQK
☑	2399	974.30	2919.89	2920.25	-0.36	1	1	5e+02	1	DFGCGESNETMCYTGPQYAAFLGFKK + Carbamidomethyl (C)
☑	2482	1157.36	3469.07	3468.74	0.33	2	1	3.8e+02	1	LLRDPCDRVAILAMMPAAVHLSDNDLCELFK
☑	889	958.09	957.09	957.59	-0.50	0	1	8e+02	1	LAHVLLHR
☑	976	987.66	1973.31	1972.88	0.43	2	1	1.5e+03	1	DLSDYSRHKYSSDEGSK
☑	2400	981.06	2940.16	2940.45	-0.29	0	1	3.7e+02	1	GNFWPSTAFDSPDLAYMLGTLGLALQR
☑	1052	1023.64	3067.91	3067.53	0.38	1	1	1.2e+03	1	ARCDVVVDVALGGPNDEPLSADLATAIMAK + Carbamidomethyl (C)
☑	1091	1039.29	3114.84	3114.47	0.37	1	1	1.4e+03	1	TLALTYSKNHAFMGNNKPPCSSYAWEK + Carbamidomethyl (C)
☑	1152	1064.49	3190.44	3190.66	-0.22	1	1	1.2e+03	1	EGQLPLLQTPSTPQFGAADLRLPSEVAEAR
☑	263	639.67	1915.99	1915.92	0.06	2	1	1.1e+03	1	KTGVDEMVWYGRANFK + Oxidation (M)
☑	970	984.96	2951.85	2951.33	0.52	2	1	1.5e+03	1	TMERVHLCVHEMRWTCAGETYLDR + Oxidation (M)
☑	2239	765.95	2294.83	2295.02	-0.19	1	1	4.8e+02	1	FLEGARSMDQHWQTASMER + Oxidation (M)
☑	2346	873.39	2617.15	2617.44	-0.30	1	1	3.4e+02	1	VIPHGSDVLILGNSDKIVTSLDLGR
☑	436	739.58	2215.72	2216.12	-0.41	0	1	1.5e+03	1	QTIAISPTLPQEYADALEEK
☑	907	964.31	2889.91	2890.31	-0.40	0	1	1.6e+03	1	AAMENGNMESFTVEFAQMLLGFLHR + 3 Oxidation (M)
☑	1129	1055.90	3164.67	3164.48	0.19	2	1	1.1e+03	1	FVMIKYHRNPAESESHLADAADMDFVR + Oxidation (M)
☑	834	934.49	2800.45	2800.37	0.08	2	1	1.3e+03	1	ELYPGSGSGTSGTPTASAETIPSRGRHK
☑	1419	1188.11	3561.30	3561.59	-0.29	1	1	1.7e+03	1	DEDAAQDEPMPSTTPTTASPSTHAPATSQPSHKR + Oxidation (M)
☑	1452	1209.87	2417.73	2417.32	0.41	2	1	1.3e+03	1	INISLEMMILCASKLKLGVER + Carbamidomethyl (C)
☑	1626	1336.95	2671.89	2672.45	-0.56	2	1	1.2e+03	1	GRISVLEKMVLPEEILMSGGHHIK
☑	2473	1128.36	3382.05	3382.49	-0.44	1	1	4.5e+02	1	FNSLSSEHSDDLKITSMTEVENQTYMFDV + Oxidation (M)
☑	755	902.98	1803.94	1803.89	0.05	1	1	2e+03	1	IPSSSAARCESSVQVQR

☑	1553	638.85	1275.68	1275.76	-0.07	1	1	5.6e+02	1	IGKLAFTNTALK
☑	1576	1292.66	3874.96	3874.87	0.08	2	1	1.1e+03	1	FRPRTAEMLCTLINIAGKFFDSYCDQTHTNIR + 2 Carbamidomethyl (C)
☑	251	633.74	1265.46	1265.60	-0.14	1	1	1.6e+03	1	MSRVAAASCQAR + Oxidation (M)
☑	1601	1308.86	2615.71	2615.36	0.35	2	1	1.2e+03	1	FKDLLQGEQEQALNDITQERLK
☑	1647	1369.57	4105.69	4105.14	0.55	1	1	1e+03	1	ETPGVTDLHAAEGAFVPVIKLAMDGVELDLLFASLPVDR
☑	396	358.29	714.56	714.37	0.19	0	1	5.4e+02	1	LPDTNR
☑	895	959.75	1917.48	1916.96	0.52	0	1	1.5e+03	1	SAFGDVSAPDAAKPAAAPFK
☑	1080	1035.11	3102.31	3102.57	-0.25	1	1	1.4e+03	1	TTIHIMDEEIIQKLSSGCVVIDCESVIK
☑	2005	949.18	1896.34	1896.06	0.28	2	1	6e+02	1	VTRSIATDVLYSFLRR
☑	2454	1081.99	3242.95	3243.54	-0.59	1	1	3.9e+02	1	GDTCTASDVWSLGCVLYALATCRLVAEDVR + Carbamidomethyl (C)
☑	662	851.79	2552.36	2552.11	0.25	1	1	1.3e+03	1	DYEDNQLRFAMFCAAAMAATEK + Carbamidomethyl (C)
☑	1200	1087.33	3258.98	3259.38	-0.40	0	1	1.5e+03	1	HQSDYMNYTVMAMSFQSSQPEVIDEYK + 2 Oxidation (M)
☑	2135	1053.39	2104.77	2105.05	-0.28	1	1	4e+02	1	VTGIDIEMHQEVKTYSQK
☑	2328	842.36	2524.07	2524.36	-0.30	2	1	3.7e+02	1	VMLLAGDVGGTKSLLGLYLMEKGK + 2 Oxidation (M)
☑	1204	1088.95	3263.83	3263.45	0.38	0	1	1.4e+03	1	QFLEMTVSDPQGSSTPMNAFYVFDDANPR
☑	1677	1392.47	2782.93	2782.40	0.52	2	1	1.3e+03	1	AQPHQQTSAAALRQRCDAYLTQLR + Carbamidomethyl (C)
☑	960	982.75	1963.48	1963.01	0.47	1	1	1.6e+03	1	CVLVDSEPKVVTAVYER + Carbamidomethyl (C)
☑	1430	1194.74	3581.20	3581.66	-0.46	2	1	1.3e+03	1	YEGNGEVCITACSGAYLFTWSLEAAKRAEETGR
☑	626	840.23	2517.67	2517.28	0.40	1	0	1.5e+03	1	YFVSATSTPSPVQPIRNTHPYR
☑	1002	1003.18	3006.51	3006.49	0.02	1	0	1.6e+03	1	CFVKSAVFDIPSLCEYFLSLGANINEK
☑	1219	1095.07	3282.19	3281.60	0.59	2	0	1.7e+03	1	IKLNEDNNTIDSVEEIKTNMTTLNMLEK + 2 Oxidation (M)
☑	1896	856.45	1710.88	1710.95	-0.08	2	0	4.2e+02	1	NGKHDFIGKVQVTLR
☑	2396	965.56	2893.66	2893.47	0.19	2	0	3.7e+02	1	MAVEQRSESLAVVSATTTPTLKSDTQK + Oxidation (M)
☑	2426	1026.74	3077.20	3077.53	-0.33	1	0	3.7e+02	1	LTARDGNANAGAPPAVASSGNGCVALLDVSGPR
☑	843	936.30	2805.87	2806.32	-0.45	0	0	1.5e+03	1	SLEQCVTEAYNCTLRPCHSAVIQK + 2 Carbamidomethyl (C)
☑	1035	1015.98	3044.93	3045.51	-0.58	1	0	1.9e+03	1	LPIEENISSMKNIVMATNHVFETLCR + Carbamidomethyl (C)
☑	384	709.54	2125.59	2126.13	-0.55	2	0	1.6e+03	1	NLEEKIVKLNNENNSLQK
☑	597	412.20	822.39	822.43	-0.04	0	0	5e+02	1	ALHETPR
☑	1514	1243.83	2485.65	2486.23	-0.58	1	0	1.4e+03	1	SSPSVQTLSVPSSTQEMPSNPAKK
☑	2173	1084.55	2167.09	2167.28	-0.18	2	0	4.2e+02	1	GTVADQVPKAITLLMITKLR
☑	2465	1106.67	3316.99	3317.39	-0.40	2	0	4e+02	1	ILSCRCICRMGLYNSSGTCTPCTDSCAVCK + 2 Carbamidomethyl (C); Oxidation (M)
☑	599	824.49	823.49	823.43	0.06	0	0	3.2e+02	1	HDAAVVGR
☑	1098	1041.20	3120.59	3120.68	-0.09	1	0	1.5e+03	1	MKMSILIGVGHMTIGIWISLINHIHYK + Oxidation (M)
☑	1289	1126.96	3377.85	3377.56	0.29	1	0	1.3e+03	1	AQENEQAPLSCGVSSPVCLSGTLSSMSSPRSPR + Oxidation (M)

☑	1079	1035.03	2068.05	2067.84	0.21	0	0	1.8e+03	1	CHASHALFAANCDTMMTR + Carbamidomethyl (C); 2 Oxidation (M)
☑	630	840.52	2518.55	2519.11	-0.56	2	0	1.2e+03	1	MNNGGPYNGYPLMRSGCGTSKQR + 2 Oxidation (M)
☑	1278	1122.70	3365.08	3364.55	0.53	0	0	1.3e+03	1	LTCHSDTINDVVFPEGLSALFATCCGPDIR + 3 Carbamidomethyl (C)
☑	2185	1089.24	2176.46	2176.14	0.32	2	0	5.5e+02	1	LTYVLEITPEKQNSDNGKK
☑	1435	1198.35	2394.68	2394.95	-0.27	2	0	1.5e+03	1	CASCIQNCQTCGDTSTCKKCK + 3 Carbamidomethyl (C)
☑	1526	1256.18	3765.53	3765.95	-0.42	2	0	1.5e+03	1	EKEQPELPTELLDKQIEQGLLWGVFSNGEIQAK
☑	1895	855.05	1708.09	1707.81	0.28	0	0	4.3e+02	1	CCICLDGVLVGQTATR + Carbamidomethyl (C)
☑	1953	906.51	1811.00	1810.89	0.11	1	0	4.1e+02	1	GAGPLPLPCAREETESK + Carbamidomethyl (C)
☑	1443	1203.39	3607.15	3606.74	0.41	1	0	1.5e+03	1	SLGVNQFSVFYMDNCAVISRTHVMYALASDLR
☑	1489	1228.96	3683.86	3683.80	0.07	2	0	1.2e+03	1	RRGVPFSSGTSSPVQYELFNVEQLSNPDEAFAR
☑	1599	1306.96	3917.85	3917.94	-0.09	2	0	1.3e+03	1	WYMPHAYYAIKVSKDMVVVVIDTVLLHNCHER + Carbamidomethyl (C); 2 Oxidation (
☑	1597	1304.09	3909.24	3909.76	-0.53	1	0	1.4e+03	1	SFAGSHGMTGMTASQLNVADYSAIVANAAKMGGDSAVMK + 4 Oxidation (M)
☑	1293	1129.39	2256.76	2256.97	-0.21	0	0	1.5e+03	1	CNEPADEMSGVAAPVTSGGHSGK + Carbamidomethyl (C)
☑	1368	1162.27	3483.79	3483.78	0.01	0	0	1.3e+03	1	SLLPQGNVVGDLGTLANNQFDFDFLVANAGLHK
☑	1938	891.18	1780.36	1780.92	-0.57	1	0	7.3e+02	1	YLLMNGLKPTEKACK + Carbamidomethyl (C); Oxidation (M)
☑	1942	899.74	1797.46	1796.97	0.49	2	0	7.2e+02	1	RRVIYSILMNQNFK + Oxidation (M)
☑	41	492.80	491.80	492.24	-0.44	0	0	41	1	CVGSK
☑	392	713.27	712.27	712.39	-0.12	1	0	1.3e+03	1	EPVRAAA
☑	972	986.10	2955.29	2955.68	-0.39	2	0	1.5e+03	1	TVSAVTGPLVILDNVKFPKFSEIVNLR
☑	964	983.70	1965.39	1965.97	-0.59	0	0	1.6e+03	1	TMALDAGANAVFTEELVSK
☑	1006	1005.55	2009.08	2008.93	0.15	1	0	1.3e+03	1	EMCRMLDLDPNILTADK + 2 Oxidation (M)
☑	1022	1010.37	3028.07	3028.50	-0.43	1	0	1.4e+03	1	DLSIWDVKQHAWSPVSGTFEVAVGASSR
☑	1101	1043.97	3128.89	3128.45	0.44	2	0	1.7e+03	1	MSSVTAGISEAASACRAKMQAAGVNEACIR + 2 Carbamidomethyl (C); 2 Oxidation (
☑	1382	1167.75	2333.49	2333.14	0.35	1	0	1.4e+03	1	GSTPSSMAGITASVAAAAADASRQR
☑	1783	766.17	1530.32	1530.81	-0.49	0	0	7.9e+02	1	VALLDTGLCAGITER
☑	677	860.19	1718.36	1718.83	-0.47	1	0	1.7e+03	1	ATLDAKQSLEDQMNR
☑	644	846.04	2535.10	2535.21	-0.11	2	0	1.9e+03	1	EMFVEEIARQTGMKIHVDENK + 2 Oxidation (M)
☑	2347	1311.13	2620.24	2620.27	-0.03	2	0	3.8e+02	1	LQEMQYMKSLGNSIIKYMGEGGK + Oxidation (M)
☑	2422	1023.10	3066.27	3066.45	-0.18	1	0	3.4e+02	1	LSEESKINCQSLSNSLSFITTNVYDMK + Oxidation (M)
☑	639	844.72	843.71	843.41	0.30	0	0	5.8e+02	1	NPPSAGSSK
☑	716	880.84	2639.50	2639.45	0.05	0	0	1.5e+03	1	LLTSLEIEQCPQLSSLTIPSNLLK
☑	1179	1074.92	2147.83	2147.88	-0.05	0	0	1.4e+03	1	MSSFCAMSMSLDHLSPGMK + Carbamidomethyl (C); 2 Oxidation (M)
☑	1598	1305.86	2609.70	2609.14	0.56	2	0	1.3e+03	1	NGGSSMTLCTEGMELSPDSHRRR + Carbamidomethyl (C); 2 Oxidation (M)
☑	1166	1069.05	3204.13	3203.66	0.48	1	0	1.6e+03	1	YKPYMEKLLHDIYENAEQLPIDQLLK

☑	1782	765.92	1529.82	1529.81	0.01	2	0	4.7e+02	1	RTKDTFIQWHAK
☑	2009	635.12	1902.33	1902.01	0.32	2	0	6.3e+02	1	TPKVVEAMELSADLQKK + Oxidation (M)
☑	2053	997.20	1992.39	1991.95	0.43	1	0	5.7e+02	1	MIDKLGAQMSSFITYCK + Carbamidomethyl (C)
☑	2419	1012.92	3035.74	3036.34	-0.60	2	0	4e+02	1	SLNQMQQTRAKDAAHSDNGETCTQGSSR + Oxidation (M)
☑	2462	1098.51	3292.52	3292.55	-0.04	2	0	3.3e+02	1	HEARCISVDSCLNSKSSTWHSSVCVFVVR + Carbamidomethyl (C)
☑	722	884.46	883.45	883.39	0.06	1	0	1.1e+03	1	MGCGSSKSK
☑	1863	834.31	1666.61	1666.80	-0.20	2	0	4.2e+02	1	SSKSQKSSEIENESK
☑	1327	1145.14	2288.27	2288.03	0.24	1	0	1.7e+03	1	TEVVTDSSNCVTGKNPEMSFK + Oxidation (M)
☑	2093	1025.65	2049.29	2048.97	0.32	0	0	5.1e+02	1	AAGYSLWNNAVGTSPAEWR
☑	2498	1267.30	3798.89	3798.84	0.05	1	0	2.5e+02	1	MVADSLSQILSQVIDDVSFSQTHTASDDFRVYPK
☑	216	616.84	1847.51	1847.79	-0.28	1	0	2.7e+03	1	FEDAMASMDKNITESK + 2 Oxidation (M)
☑	546	800.19	799.18	799.35	-0.17	0	0	4.3e+02	1	DYLSMR + Oxidation (M)
☑	1541	1266.06	3795.17	3794.72	0.45	1	0	1.5e+03	1	DLPDHVTRNLNTWEHYYNSTTPETMEIPYDK + Oxidation (M)
☑	971	985.14	984.14	984.51	-0.38	0	0	1.5e+03	1	AFVIDHQR
☑	1036	1016.02	3045.04	3044.58	0.46	2	0	2.1e+03	1	LAAKTSLMLGFGSDGIALFRFEWLTER + Oxidation (M)
☑	1350	1153.89	3458.66	3458.72	-0.06	2	0	1.3e+03	1	NYHIFYEMLAGLSPTEKQQLGGLKTAQDYK + Oxidation (M)
☑	2307	822.90	2465.69	2465.19	0.51	0	0	6.6e+02	1	FDNLHICTDPPFNPHIMPLTK + Oxidation (M)
☑	640	845.69	844.69	844.46	0.23	0	0	1.8e+03	1	ALGMQLGR
☑	659	850.71	2549.12	2549.31	-0.19	1	0	1.6e+03	1	VSTEDNISTKVSTEDNISLGSIIK
☑	1457	1212.58	2423.14	2423.19	-0.05	1	0	1.3e+03	1	LPHYHLTGYQQSHLSEEEKK
☑	651	848.14	2541.41	2541.19	0.22	2	0	1.7e+03	1	MGARYDAPLWGSVDRATMGWAAK + 2 Oxidation (M)
☑	1132	1056.21	3165.60	3165.59	0.01	2	0	1.5e+03	1	LLKMVGSATFSNVFSCLDLKDNVTYCLK + Carbamidomethyl (C)
☑	2305	821.93	2462.77	2462.24	0.52	2	0	7.3e+02	1	KDAKVTCYVVSWLEEHLTWR
☑	1427	1193.39	2384.78	2385.17	-0.39	0	0	1.5e+03	1	TPCRPDFPPLTIINEDPEFK + Carbamidomethyl (C)
☑	1501	1236.40	3706.18	3705.71	0.47	2	0	1.5e+03	1	HVGPAMGTKLPMQSPGTQCIADYVDSEESGIDRK + Carbamidomethyl (C); 2 Oxidatior
☑	1483	1228.02	2454.02	2454.28	-0.26	2	0	1.3e+03	1	AILDDHLARCLFQDETRLAVR
☑	1309	1136.52	2271.03	2271.17	-0.15	1	0	1.2e+03	1	LWEEVLNLPNELRCYGVPK
☑	1359	1157.54	3469.58	3469.57	0.01	0	0	1.2e+03	1	CFHSPLTGADPGAVMSAAAGSAMGGTLVSEGYQTR + Carbamidomethyl (C); Oxidation
☑	1472	1219.17	3654.49	3654.95	-0.46	2	0	1.7e+03	1	IFYAYPVTDALSTVCSLLVALKTFINFYRQAK
☑	1496	1234.07	3699.18	3699.72	-0.54	1	0	1.6e+03	1	MLRSSPFSSGSDDEGLAPAGGPSAPETAAAAAAAGAGNGR
☑	555	803.49	2407.44	2407.14	0.30	2	0	1.7e+03	1	TLHWCDYQYAEFRRAVHR + Carbamidomethyl (C)
☑	1195	1084.03	3249.06	3248.52	0.54	2	0	1.7e+03	1	DMKVFIENEGSMRAVEDTYESLQVDLSK + Oxidation (M)
☑	1379	1165.51	2329.00	2329.04	-0.04	1	0	1.2e+03	1	YWNQATSENGCKWGTVEATK + Carbamidomethyl (C)
☑	2297	1209.02	2416.02	2416.29	-0.27	0	0	4.2e+02	1	SLLQCPIMPEGCSLALALIIFK + Carbamidomethyl (C)

☑	947	978.89	1955.78	1955.96	-0.19	0	0	1.5e+03	1	VEFYSILLANENGSSWK
☑	1478	1222.84	2443.67	2443.34	0.33	0	0	1.5e+03	1	LQTAGLSPLLFIMLDVGNEVSVK
☑	729	889.28	2664.83	2665.15	-0.31	1	0	1.9e+03	1	CIQCETNPYCILFDGSCNKCIK + 3 Carbamidomethyl (C)
☑	1927	883.56	1765.10	1764.83	0.27	1	0	4.6e+02	1	GDSRSFSSSEGPNTPIK
☑	454	753.84	1505.67	1505.71	-0.05	2	0	1.7e+03	1	AKRGQMLDDAEEK + Oxidation (M)
☑	1887	568.22	1701.64	1701.73	-0.09	0	0	4.9e+02	1	EVEECFNAYESNIR
☑	1538	1262.23	2522.45	2522.23	0.22	0	0	1.5e+03	1	ESSSGPSGQFEFITGTNIPGQGVVK
☑	1258	1111.10	2220.18	2220.12	0.06	2	0	1.8e+03	1	GHGASVHAVAGKRGPSSGSMVAGK + Oxidation (M)
☑	837	934.93	2801.77	2802.29	-0.52	2	0	2.1e+03	1	MPSKEFSVPSRAACPVSCSSFVNSR + 2 Carbamidomethyl (C); Oxidation (M)
☑	1111	1046.80	2091.59	2092.09	-0.50	1	0	1.8e+03	1	LIKSESVASCKPAEGEFLK + Carbamidomethyl (C)
☑	1512	621.22	1240.42	1240.63	-0.21	0	0	4.6e+02	1	AELSVASPAGPDK
☑	2425	1026.28	3075.83	3075.71	0.12	2	0	4e+02	1	ASMLISEAKKLISAVTNMKPDSILFQIK
☑	526	793.31	2376.90	2377.09	-0.20	2	0	1.5e+03	1	STAFSGRALCFSEEMDPFARR
☑	1366	1162.13	2322.24	2322.15	0.09	1	0	1.8e+03	1	CVGETGPYSLRIVSACELVGR + 2 Carbamidomethyl (C)
☑	615	832.62	2494.84	2495.03	-0.20	1	0	2e+03	1	VGEKMFCFQCQETNNNTGCTK + 2 Carbamidomethyl (C)
☑	956	981.78	2942.31	2942.32	-0.02	2	0	1.6e+03	1	MLEGRYYSCADLTNLVRDAAMMTMR + 2 Oxidation (M)
☑	1473	1220.54	2439.06	2439.22	-0.16	1	0	1.4e+03	1	MTAPGQLITCKINSSVVSAMTSK + Carbamidomethyl (C); Oxidation (M)
☑	2403	985.38	2953.13	2952.64	0.49	2	0	4.3e+02	1	GDLSIESIVLILAMKLAYPTKVFMIR + 2 Oxidation (M)
☑	462	758.97	2273.87	2274.19	-0.32	0	0	2.2e+03	1	LIPCSSAVSSFLVPSIDDGVLR
☑	569	810.52	2428.54	2428.25	0.28	2	0	1.5e+03	1	VNFNWSIFDIIRETKYLDR
☑	1397	1175.32	3522.94	3522.98	-0.05	1	0	1.6e+03	1	YIEFILDHLASVPAARIAIAQTTIIAGSTNILK
☑	2197	1099.90	2197.78	2198.17	-0.39	2	0	4.7e+02	1	EKPYGTITNKFDLFERIK
☑	1383	1168.05	3501.12	3500.75	0.37	1	0	1.8e+03	1	YTRLLMDVVEGYGLVGYVPLDVQNQEMMLR
☑	1480	612.24	1222.46	1222.66	-0.20	0	0	4.5e+02	1	LLTSIDFTSAR
☑	1041	1017.53	3049.58	3049.57	0.01	1	0	1.4e+03	1	IMIQILSNVFPFKDFWGHVCIVWNK + Oxidation (M)
☑	1044	1018.84	3053.49	3053.44	0.05	2	0	1.5e+03	1	DIAEISSQCQDVTANMPRYQETKDLAK
☑	2484	1162.89	3485.65	3485.72	-0.08	1	0	3.5e+02	1	HLDQEAEFVQKCKPAVALLFIDCPDEELTK + Carbamidomethyl (C)
☑	2	*288.21*	*287.20*							
☑	4	*359.08*	*358.08*							
☑	6	*371.00*	*369.99*							
☑	7	*371.08*	*370.07*							
☑	8	*205.97*	*409.92*							
☑	10	*415.80*	*414.79*							
☑	11	*423.58*	*422.58*							

☑	12	215.02	428.02
☑	13	432.70	431.69
☑	16	440.72	439.71
☑	18	223.99	445.97
☑	20	455.17	454.16
☑	21	455.79	454.78
☑	22	459.44	458.43
☑	23	460.40	459.39
☑	30	477.26	476.25
☑	33	482.19	481.18
☑	35	485.26	484.25
☑	42	493.14	492.13
☑	44	494.86	493.85
☑	46	496.18	495.17
☑	49	499.44	498.43
☑	54	506.27	505.26
☑	55	507.35	506.34
☑	62	514.50	513.49
☑	70	520.49	519.48
☑	71	520.83	519.82
☑	75	524.62	523.61
☑	76	525.37	524.36
☑	80	529.48	528.48
☑	85	535.25	534.24
☑	86	536.20	535.19
☑	93	540.00	539.00
☑	94	540.21	539.20
☑	102	546.04	545.03
☑	103	546.11	545.10
☑	107	547.91	546.90
☑	108	548.51	547.50
☑	115	554.33	553.32
☑	116	555.00	554.00
☑	121	557.95	556.94

☑	122	559.74	558.73
☑	128	565.26	564.25
☑	129	565.32	564.31
☑	133	567.77	566.76
☑	134	568.04	567.04
☑	136	570.40	569.40
☑	141	574.67	573.66
☑	145	576.95	575.94
☑	147	579.83	578.82
☑	151	581.15	580.15
☑	152	581.28	580.27
☑	153	582.03	581.03
☑	154	582.60	581.60
☑	155	583.22	582.21
☑	164	590.33	589.32
☑	169	593.50	592.49
☑	181	599.36	598.35
☑	186	601.68	600.68
☑	187	601.93	600.92
☑	188	602.26	601.25
☑	189	602.35	601.34
☑	192	603.67	602.67
☑	193	603.93	602.93
☑	209	615.24	614.24
☑	210	615.47	614.47
☑	211	615.67	614.66
☑	212	616.11	615.10
☑	213	616.23	615.22
☑	214	616.50	615.49
☑	215	616.67	615.66
☑	219	618.66	617.66
☑	221	619.30	618.29
☑	222	619.91	618.90
☑	223	620.26	619.26

☑	225	620.97	619.96
☑	228	622.31	621.30
☑	229	622.33	621.32
☑	230	622.42	621.41
☑	231	622.92	621.91
☑	234	624.66	623.65
☑	236	624.81	623.80
☑	238	625.80	624.79
☑	242	627.01	626.01
☑	244	629.61	628.61
☑	254	634.33	633.33
☑	259	638.22	637.21
☑	260	638.41	637.41
☑	264	640.29	639.28
☑	266	640.35	639.35
☑	269	643.00	641.99
☑	271	644.66	643.66
☑	273	646.74	645.73
☑	276	647.90	646.89
☑	279	648.96	647.95
☑	285	650.77	649.76
☑	287	326.10	650.18
☑	288	651.64	650.63
☑	289	652.17	651.16
☑	290	653.86	652.85
☑	293	654.69	653.68
☑	294	654.99	653.98
☑	297	658.30	657.29
☑	306	662.11	661.10
☑	308	663.06	662.05
☑	318	671.19	670.18
☑	319	672.27	671.26
☑	330	677.14	676.13
☑	331	677.29	676.28

☑	332	677.31	676.30
☑	333	677.40	676.39
☑	349	688.44	687.43
☑	351	689.03	688.03
☑	353	689.36	688.35
☑	357	694.37	693.36
☑	360	698.27	697.27
☑	361	699.27	698.27
☑	362	699.30	698.29
☑	371	702.08	701.07
☑	372	702.84	701.83
☑	373	703.98	702.97
☑	376	705.09	704.08
☑	379	706.21	705.21
☑	380	706.59	705.59
☑	382	707.96	706.95
☑	387	710.10	709.09
☑	388	710.90	709.89
☑	389	711.64	710.64
☑	390	712.66	711.65
☑	394	714.76	713.75
☑	395	715.31	714.30
☑	399	718.19	717.18
☑	401	719.01	718.00
☑	402	719.60	718.60
☑	403	719.73	718.72
☑	404	719.81	718.80
☑	406	720.21	719.21
☑	407	722.45	721.44
☑	408	723.76	722.75
☑	410	725.03	724.02
☑	415	726.32	725.31
☑	420	731.09	730.09
☑	421	731.12	730.12

☑	425	732.30	731.29
☑	427	733.26	732.26
☑	430	735.37	734.36
☑	431	737.39	736.39
☑	434	739.04	738.03
☑	437	739.61	738.60
☑	438	740.60	739.59
☑	439	741.23	740.23
☑	441	743.70	742.69
☑	442	743.95	742.95
☑	446	746.31	745.30
☑	447	746.38	745.38
☑	450	751.71	750.70
☑	455	755.94	754.93
☑	458	757.61	756.61
☑	459	757.81	756.81
☑	460	757.92	756.91
☑	463	760.50	759.49
☑	465	761.36	760.36
☑	468	763.83	762.82
☑	469	763.86	762.85
☑	473	764.33	763.32
☑	474	764.68	763.67
☑	475	765.04	764.04
☑	480	766.77	765.76
☑	482	767.14	766.14
☑	483	767.91	766.90
☑	484	768.85	767.84
☑	486	769.30	768.29
☑	487	769.88	768.87
☑	488	770.88	769.87
☑	489	771.10	770.09
☑	490	771.29	770.29
☑	493	772.39	771.39

☑	495	773.28	772.27
☑	496	774.33	773.32
☑	498	774.52	773.51
☑	504	779.23	778.23
☑	505	779.51	778.50
☑	506	780.40	779.40
☑	508	781.09	780.08
☑	509	784.19	783.18
☑	510	784.94	783.93
☑	511	785.10	784.09
☑	513	785.69	784.69
☑	517	788.74	787.73
☑	518	789.29	788.29
☑	520	789.84	788.83
☑	522	791.65	790.64
☑	523	792.47	791.47
☑	525	793.09	792.08
☑	527	793.90	792.89
☑	528	793.94	792.93
☑	531	794.85	793.85
☑	535	796.39	795.39
☑	538	796.52	795.52
☑	539	398.86	795.71
☑	540	796.89	795.88
☑	543	798.76	797.76
☑	544	799.15	798.14
☑	545	799.85	798.84
☑	549	800.44	799.43
☑	550	801.70	800.69
☑	551	802.66	801.66
☑	558	805.22	804.21
☑	560	805.82	804.81
☑	562	807.35	806.34
☑	566	808.38	807.38

☑	567	808.91	807.91
☑	568	809.02	808.01
☑	572	811.18	810.18
☑	574	812.42	811.42
☑	575	812.57	811.56
☑	578	813.39	812.39
☑	579	813.49	812.48
☑	582	814.76	813.75
☑	583	814.97	813.96
☑	586	817.42	816.41
☑	590	820.57	819.56
☑	592	821.60	820.59
☑	594	822.01	821.00
☑	596	412.02	822.03
☑	598	824.33	823.32
☑	600	824.67	823.66
☑	607	828.48	827.47
☑	608	829.08	828.07
☑	609	830.26	829.25
☑	611	831.18	830.17
☑	614	832.25	831.24
☑	618	836.75	835.74
☑	621	838.15	837.14
☑	622	838.37	837.36
☑	628	840.47	839.46
☑	633	842.26	841.25
☑	636	842.50	841.49
☑	638	844.04	843.03
☑	641	845.81	844.80
☑	642	845.88	844.87
☑	643	845.93	844.92
☑	650	848.09	847.08
☑	652	848.71	847.70
☑	654	849.36	848.35

☑	655	849.45	848.44
☑	658	850.70	849.69
☑	660	851.40	850.40
☑	661	851.74	850.73
☑	664	851.97	850.96
☑	665	852.02	851.01
☑	667	427.24	852.46
☑	670	855.06	854.06
☑	673	858.37	857.36
☑	676	860.14	859.13
☑	680	860.92	859.91
☑	682	863.16	862.16
☑	683	864.12	863.11
☑	685	867.90	866.89
☑	686	434.87	867.72
☑	688	868.90	867.89
☑	689	869.28	868.27
☑	690	869.95	868.95
☑	691	870.01	869.00
☑	694	872.60	871.59
☑	697	873.18	872.17
☑	698	873.76	872.75
☑	701	875.17	874.16
☑	702	438.19	874.36
☑	703	876.26	875.26
☑	704	876.71	875.70
☑	706	877.30	876.29
☑	708	877.87	876.86
☑	709	878.60	877.59
☑	710	879.07	878.06
☑	711	879.71	878.70
☑	713	879.96	878.95
☑	714	880.46	879.45
☑	715	880.82	879.81

☑	718	881.21	880.20
☑	723	885.04	884.03
☑	724	885.17	884.16
☑	725	885.33	884.33
☑	731	890.08	889.07
☑	733	445.90	889.78
☑	734	891.75	890.74
☑	736	892.60	891.59
☑	739	893.66	892.65
☑	741	894.15	893.15
☑	742	894.26	893.25
☑	743	894.52	893.51
☑	744	895.71	894.70
☑	745	895.73	894.73
☑	747	896.13	895.13
☑	749	900.78	899.78
☑	752	902.40	901.39
☑	753	902.43	901.42
☑	754	902.50	901.49
☑	759	903.95	902.94
☑	760	904.17	903.16
☑	761	452.71	903.40
☑	762	904.51	903.51
☑	763	904.86	903.85
☑	764	453.28	904.54
☑	766	453.46	904.91
☑	767	906.03	905.03
☑	768	906.65	905.64
☑	769	906.91	905.90
☑	772	908.14	907.14
☑	774	909.21	908.20
☑	775	909.56	908.56
☑	776	909.96	908.95
☑	779	910.84	909.83

☑	780	911.88	910.87
☑	784	913.90	912.89
☑	785	915.06	914.06
☑	786	915.69	914.68
☑	787	916.64	915.63
☑	789	917.45	916.44
☑	790	917.88	916.87
☑	791	919.02	918.01
☑	794	920.11	919.10
☑	796	921.00	920.00
☑	797	921.19	920.18
☑	798	922.71	921.71
☑	801	923.67	922.66
☑	802	924.30	923.29
☑	803	925.15	924.14
☑	804	925.70	924.69
☑	808	926.90	925.89
☑	811	927.97	926.96
☑	812	928.50	927.50
☑	813	928.67	927.66
☑	816	929.99	928.99
☑	818	930.64	929.64
☑	822	931.83	930.82
☑	823	931.92	930.91
☑	824	932.27	931.26
☑	825	932.35	931.34
☑	826	932.76	931.75
☑	828	933.11	932.10
☑	829	933.22	932.21
☑	830	933.24	932.23
☑	831	933.36	932.35
☑	832	933.58	932.57
☑	835	934.54	933.53
☑	836	934.85	933.84

☑	842	936.22	935.22
☑	844	936.56	935.55
☑	845	936.61	935.60
☑	848	937.83	936.83
☑	851	939.46	938.45
☑	852	940.27	939.26
☑	853	940.86	939.85
☑	854	941.72	940.71
☑	855	942.06	941.06
☑	857	942.30	941.29
☑	859	942.60	941.59
☑	860	944.04	943.04
☑	862	945.71	944.70
☑	863	945.89	944.89
☑	865	946.67	945.67
☑	866	946.95	945.94
☑	870	949.39	948.38
☑	871	950.04	949.03
☑	874	951.99	950.98
☑	875	952.42	951.41
☑	877	952.91	951.90
☑	879	953.54	952.54
☑	882	954.70	953.69
☑	885	956.73	955.72
☑	886	956.76	955.75
☑	887	957.71	956.70
☑	888	957.74	956.73
☑	894	959.58	958.57
☑	897	961.08	960.07
☑	898	961.89	960.89
☑	900	962.23	961.22
☑	901	962.82	961.81
☑	902	962.88	961.88
☑	903	963.41	962.40

☑	905	963.55	962.54
☑	908	966.19	965.18
☑	909	966.74	965.74
☑	911	967.04	966.04
☑	913	969.25	968.25
☑	916	970.08	969.07
☑	917	970.35	969.34
☑	924	972.24	971.23
☑	925	972.31	971.30
☑	927	973.06	972.05
☑	928	973.39	972.38
☑	936	976.01	975.00
☑	937	976.31	975.31
☑	938	977.36	976.35
☑	940	489.23	976.45
☑	945	978.43	977.42
☑	948	978.92	977.91
☑	949	979.40	978.39
☑	951	979.74	978.73
☑	953	980.32	979.31
☑	955	980.88	979.87
☑	959	982.54	981.53
☑	961	982.81	981.81
☑	962	982.87	981.86
☑	965	984.29	983.28
☑	966	984.29	983.28
☑	967	984.34	983.33
☑	968	984.70	983.69
☑	974	987.44	986.43
☑	977	988.56	987.55
☑	978	988.76	987.75
☑	983	990.75	989.74
☑	984	991.29	990.28
☑	986	991.57	990.56

☑	987	*993.42*	*992.41*
☑	991	*995.16*	*994.15*
☑	993	*997.49*	*996.48*
☑	995	*998.47*	*997.47*
☑	996	*499.86*	*997.70*
☑	997	*998.77*	*997.76*
☑	998	*998.87*	*997.86*
☑	1000	*1001.42*	*1000.42*
☑	1004	*1004.19*	*1003.19*
☑	1008	*1005.77*	*1004.77*
☑	1010	*1007.08*	*1006.07*
☑	1011	*1007.16*	*1006.15*
☑	1013	*1007.37*	*1006.36*
☑	1019	*1010.07*	*1009.06*
☑	1020	*1010.18*	*1009.17*
☑	1028	*1012.11*	*1011.10*
☑	1029	*1012.80*	*1011.79*
☑	1032	*1014.16*	*1013.15*
☑	1042	*1018.14*	*1017.13*
☑	1045	*1018.91*	*1017.90*
☑	1047	*1020.53*	*1019.52*
☑	1049	*1021.83*	*1020.82*
☑	1051	*1023.21*	*1022.20*
☑	1057	*1025.64*	*1024.63*
☑	1061	*1028.24*	*1027.24*
☑	1069	*1030.91*	*1029.90*
☑	1071	*1031.84*	*1030.83*
☑	1072	*1032.14*	*1031.14*
☑	1074	*1032.52*	*1031.52*
☑	1076	*1033.81*	*1032.80*
☑	1077	*1033.89*	*1032.88*
☑	1082	*1035.64*	*1034.64*
☑	1086	*1037.59*	*1036.58*
☑	1087	*1037.70*	*1036.69*

☑	1089	1038.18	1037.17
☑	1095	520.30	1038.59
☑	1099	1041.49	1040.48
☑	1102	1044.06	1043.05
☑	1105	1045.55	1044.54
☑	1107	1045.71	1044.70
☑	1110	1046.73	1045.72
☑	1112	1047.76	1046.76
☑	1115	1049.56	1048.55
☑	1116	1049.64	1048.64
☑	1119	1050.10	1049.09
☑	1122	1051.70	1050.70
☑	1125	1054.31	1053.30
☑	1127	1055.27	1054.26
☑	1128	1055.71	1054.70
☑	1131	1056.11	1055.10
☑	1134	1057.88	1056.87
☑	1135	1057.88	1056.87
☑	1136	1058.10	1057.10
☑	1138	1058.23	1057.23
☑	1140	1058.97	1057.97
☑	1143	1059.69	1058.68
☑	1145	1060.13	1059.12
☑	1146	1060.61	1059.60
☑	1147	1062.06	1061.05
☑	1154	1064.89	1063.89
☑	1158	1066.20	1065.20
☑	1159	1066.27	1065.27
☑	1161	1066.61	1065.60
☑	1163	1067.45	1066.45
☑	1165	1068.38	1067.37
☑	1167	1069.34	1068.33
☑	1170	1071.42	1070.41
☑	1171	1071.56	1070.55

☑	1172	1072.23	1071.22
☑	1175	1072.71	1071.70
☑	1177	1073.96	1072.95
☑	1180	1075.05	1074.04
☑	1181	1075.15	1074.14
☑	1183	1076.68	1075.68
☑	1184	1076.78	1075.77
☑	1185	1076.79	1075.78
☑	1186	1078.81	1077.80
☑	1187	1078.83	1077.82
☑	1189	1079.23	1078.22
☑	1193	1081.86	1080.86
☑	1196	1084.29	1083.28
☑	1198	1084.81	1083.80
☑	1201	1087.44	1086.44
☑	1202	1087.91	1086.90
☑	1203	363.47	1087.39
☑	1206	1089.37	1088.36
☑	1211	1090.95	1089.94
☑	1215	1093.26	1092.25
☑	1216	1093.72	1092.72
☑	1220	1095.57	1094.56
☑	1221	1096.10	1095.09
☑	1224	1097.79	1096.78
☑	1226	1098.73	1097.72
☑	1233	1101.05	1100.04
☑	1234	1101.33	1100.32
☑	1236	1101.78	1100.77
☑	1237	1102.15	1101.14
☑	1240	1102.61	1101.60
☑	1241	1102.98	1101.97
☑	1242	1103.14	1102.14
☑	1246	1105.31	1104.30
☑	1247	1105.97	1104.97

☑	1259	1112.25	1111.25
☑	1261	1113.60	1112.59
☑	1263	1114.13	1113.12
☑	1266	1114.78	1113.77
☑	1267	1114.94	1113.94
☑	1268	1115.17	1114.17
☑	1270	1118.35	1117.34
☑	1271	1118.93	1117.93
☑	1272	1119.55	1118.54
☑	1274	1121.06	1120.05
☑	1281	1123.24	1122.23
☑	1282	1123.62	1122.61
☑	1284	1124.52	1123.51
☑	1298	1132.26	1131.25
☑	1299	1132.52	1131.51
☑	1301	1133.93	1132.92
☑	1302	1133.93	1132.92
☑	1310	1136.82	1135.81
☑	1311	1137.78	1136.77
☑	1312	1138.18	1137.17
☑	1315	1138.85	1137.84
☑	1317	1140.37	1139.36
☑	1318	1140.73	1139.73
☑	1322	1142.78	1141.77
☑	1323	1143.14	1142.14
☑	1325	1143.82	1142.81
☑	1331	1148.55	1147.54
☑	1338	1150.43	1149.43
☑	1340	576.33	1150.65
☑	1342	576.45	1150.88
☑	1343	1151.91	1150.90
☑	1346	1152.77	1151.76
☑	1351	1154.30	1153.29
☑	1354	1154.57	1153.56

☑	1356	1156.93	1155.92
☑	1357	1157.06	1156.05
☑	1358	1157.47	1156.46
☑	1360	1158.55	1157.55
☑	1363	1161.36	1160.35
☑	1364	1161.56	1160.56
☑	1365	581.55	1161.09
☑	1367	1162.27	1161.26
☑	1370	1163.18	1162.17
☑	1373	1163.89	1162.88
☑	1375	1165.09	1164.08
☑	1378	1165.47	1164.46
☑	1384	1168.05	1167.04
☑	1385	1169.35	1168.34
☑	1388	1170.56	1169.56
☑	1390	1170.64	1169.64
☑	1392	1172.41	1171.40
☑	1394	1174.49	1173.49
☑	1408	1182.63	1181.62
☑	1417	1187.47	1186.46
☑	1422	1189.47	1188.46
☑	1428	1194.23	1193.23
☑	1431	1195.15	1194.14
☑	1433	1195.94	1194.94
☑	1436	1200.53	1199.53
☑	1449	1207.87	1206.86
☑	1454	1210.58	1209.57
☑	1463	1215.43	1214.42
☑	1468	1217.65	1216.64
☑	1476	1221.77	1220.76
☑	1477	611.58	1221.14
☑	1482	1226.34	1225.33
☑	1484	1228.04	1227.03
☑	1498	1234.93	1233.92

☑	1503	1236.93	1235.92
☑	1506	1239.14	1238.13
☑	1516	1246.78	1245.77
☑	1523	1253.89	1252.88
☑	1536	1260.62	1259.61
☑	1537	1260.95	1259.94
☑	1539	632.86	1263.70
☑	1543	1267.75	1266.74
☑	1547	636.62	1271.22
☑	1550	1275.92	1274.91
☑	1551	638.66	1275.31
☑	1558	1284.60	1283.59
☑	1564	1286.35	1285.34
☑	1565	1286.52	1285.51
☑	1567	1287.34	1286.33
☑	1568	644.35	1286.68
☑	1569	645.40	1288.78
☑	1572	1290.96	1289.95
☑	1574	646.33	1290.65
☑	1581	1295.30	1294.30
☑	1582	1295.46	1294.45
☑	1584	1297.83	1296.82
☑	1588	650.27	1298.53
☑	1590	1300.54	1299.53
☑	1606	1313.11	1312.10
☑	1609	658.70	1315.38
☑	1613	661.06	1320.11
☑	1616	1325.44	1324.44
☑	1627	1337.72	1336.71
☑	1628	1338.20	1337.19
☑	1630	1341.15	1340.14
☑	1632	671.91	1341.81
☑	1633	1343.00	1341.99
☑	1640	679.76	1357.51

☑	1646	684.26	1366.51
☑	1658	1379.38	1378.37
☑	1667	692.78	1383.54
☑	1669	692.84	1383.66
☑	1678	699.85	1397.68
☑	1688	708.82	1415.63
☑	1693	710.85	1419.68
☑	1695	713.89	1425.76
☑	1699	716.02	1430.03
☑	1706	719.59	1437.16
☑	1722	733.12	1464.22
☑	1723	733.76	1465.51
☑	1726	734.32	1466.63
☑	1738	737.94	1473.86
☑	1742	740.34	1478.66
☑	1754	745.86	1489.71
☑	1772	756.29	1510.57
☑	1773	756.97	1511.93
☑	1776	761.71	1521.40
☑	1778	762.22	1522.43
☑	1784	769.74	1537.47
☑	1785	770.36	1538.71
☑	1786	770.40	1538.79
☑	1788	514.62	1540.84
☑	1793	778.68	1555.34
☑	1801	783.82	1565.63
☑	1802	783.89	1565.76
☑	1804	786.47	1570.93
☑	1808	788.66	1575.32
☑	1812	792.48	1582.95
☑	1813	792.90	1583.78
☑	1814	793.54	1585.06
☑	1816	529.90	1586.68
☑	1819	798.72	1595.43

☑	1822	802.44	1602.86
☑	1823	802.61	1603.20
☑	1824	804.73	1607.45
☑	1826	804.83	1607.64
☑	1829	806.16	1610.30
☑	1833	808.90	1615.79
☑	1834	811.22	1620.42
☑	1839	813.29	1624.56
☑	1841	814.49	1626.96
☑	1844	818.36	1634.71
☑	1847	819.30	1636.58
☑	1851	824.31	1646.60
☑	1853	825.37	1648.72
☑	1854	825.89	1649.77
☑	1855	826.47	1650.92
☑	1856	827.07	1652.13
☑	1859	830.98	1659.95
☑	1861	556.20	1665.59
☑	1864	835.42	1668.82
☑	1871	842.03	1682.04
☑	1872	842.35	1682.68
☑	1873	842.43	1682.84
☑	1874	842.62	1683.22
☑	1878	846.30	1690.59
☑	1886	566.50	1696.49
☑	1890	853.02	1704.04
☑	1891	853.12	1704.22
☑	1897	856.88	1711.74
☑	1900	857.03	1712.05
☑	1901	857.54	1713.07
☑	1902	858.65	1715.28
☑	1905	862.24	1722.46
☑	1907	867.51	1733.00
☑	1910	871.67	1741.33

☑	1911	871.85	1741.68
☑	1912	872.36	1742.70
☑	1913	873.80	1745.59
☑	1918	877.61	1753.21
☑	1922	880.37	1758.73
☑	1923	881.55	1761.09
☑	1926	882.71	1763.40
☑	1932	885.90	1769.79
☑	1933	885.94	1769.87
☑	1937	890.48	1778.95
☑	1940	897.83	1793.65
☑	1946	900.53	1799.04
☑	1952	906.20	1810.39
☑	1968	918.04	1834.07
☑	1973	613.62	1837.84
☑	1974	920.44	1838.86
☑	1985	620.81	1859.41
☑	1986	931.14	1860.27
☑	1990	626.40	1876.17
☑	1992	939.21	1876.41
☑	1994	940.25	1878.48
☑	1996	941.60	1881.19
☑	1998	943.62	1885.22
☑	2000	944.79	1887.56
☑	2001	944.94	1887.87
☑	2002	945.05	1888.09
☑	2003	945.46	1888.91
☑	2004	948.11	1894.20
☑	2006	949.18	1896.35
☑	2008	951.96	1901.91
☑	2010	952.95	1903.89
☑	2013	953.99	1905.96
☑	2019	956.83	1911.64
☑	2021	958.50	1914.99

☑	2022	960.13	1918.24
☑	2023	960.18	1918.35
☑	2028	961.85	1921.68
☑	2029	962.60	1923.18
☑	2031	963.83	1925.65
☑	2032	964.32	1926.63
☑	2034	967.70	1933.39
☑	2035	968.44	1934.87
☑	2036	970.58	1939.15
☑	2038	973.52	1945.03
☑	2040	976.34	1950.67
☑	2041	979.23	1956.45
☑	2042	654.26	1959.77
☑	2043	981.02	1960.03
☑	2044	984.34	1966.66
☑	2046	987.97	1973.92
☑	2047	659.24	1974.68
☑	2048	990.28	1978.54
☑	2051	995.46	1988.91
☑	2052	996.41	1990.81
☑	2057	1000.84	1999.67
☑	2059	1001.74	2001.46
☑	2060	1003.03	2004.05
☑	2061	1003.22	2004.42
☑	2062	1003.24	2004.46
☑	2063	1004.63	2007.25
☑	2064	1005.65	2009.28
☑	2065	1006.89	2011.76
☑	2066	1007.39	2012.76
☑	2069	1007.97	2013.93
☑	2072	1008.46	2014.90
☑	2074	675.25	2022.73
☑	2078	1014.28	2026.55
☑	2079	1015.58	2029.15

☑	2080	1015.66	2029.31
☑	2082	1016.09	2030.16
☑	2085	1021.18	2040.34
☑	2086	1022.32	2042.62
☑	2088	1023.89	2045.77
☑	2091	1024.58	2047.14
☑	2094	1026.17	2050.32
☑	2098	1028.56	2055.11
☑	2099	1029.02	2056.03
☑	2103	689.54	2065.60
☑	2106	1036.42	2070.82
☑	2107	1036.51	2071.01
☑	2108	1036.90	2071.79
☑	2111	1038.96	2075.90
☑	2115	1042.69	2083.36
☑	2116	1042.71	2083.40
☑	2117	1043.15	2084.30
☑	2118	697.35	2089.03
☑	2119	1045.57	2089.13
☑	2121	1047.11	2092.20
☑	2122	1047.84	2093.67
☑	2123	1049.63	2097.26
☑	2126	1050.11	2098.20
☑	2128	1050.54	2099.06
☑	2129	1050.88	2099.75
☑	2130	1052.31	2102.61
☑	2131	1052.34	2102.66
☑	2133	1053.15	2104.29
☑	2138	1054.40	2106.79
☑	2139	1054.85	2107.68
☑	2140	1056.83	2111.64
☑	2141	1056.92	2111.83
☑	2143	1057.65	2113.29
☑	2145	1059.17	2116.34

☑	2147	709.14	2124.40
☑	2148	1063.64	2125.26
☑	2149	1064.00	2125.99
☑	2150	1064.63	2127.24
☑	2152	1065.43	2128.86
☑	2153	710.70	2129.07
☑	2154	1069.40	2136.79
☑	2155	1069.88	2137.74
☑	2159	1072.96	2143.90
☑	2161	1075.69	2149.36
☑	2162	1075.83	2149.64
☑	2165	1077.98	2153.94
☑	2166	1078.99	2155.97
☑	2168	1082.59	2163.16
☑	2179	1087.19	2172.37
☑	2180	725.31	2172.90
☑	2181	725.90	2174.68
☑	2183	1089.13	2176.25
☑	2184	1089.14	2176.28
☑	2186	1089.26	2176.50
☑	2190	1093.53	2185.04
☑	2191	729.43	2185.26
☑	2195	1097.89	2193.77
☑	2196	1098.33	2194.64
☑	2198	1103.03	2204.04
☑	2199	1103.13	2204.24
☑	2200	1105.52	2209.02
☑	2201	1105.83	2209.65
☑	2205	1108.33	2214.64
☑	2206	1109.71	2217.40
☑	2207	1111.05	2220.09
☑	2210	1115.22	2228.43
☑	2211	1115.51	2229.00
☑	2212	1116.93	2231.85

☑	2213	1117.54	2233.07
☑	2214	1117.58	2233.14
☑	2217	1126.00	2249.99
☑	2221	1127.28	2252.55
☑	2223	752.17	2253.49
☑	2225	1128.67	2255.32
☑	2227	1129.28	2256.55
☑	2228	755.16	2262.45
☑	2229	1135.87	2269.73
☑	2230	1137.86	2273.70
☑	2237	1144.82	2287.62
☑	2240	1148.96	2295.91
☑	2244	1151.12	2300.23
☑	2245	768.72	2303.15
☑	2246	1154.25	2306.48
☑	2248	770.70	2309.09
☑	2249	1155.61	2309.21
☑	2250	1156.97	2311.92
☑	2252	1157.44	2312.87
☑	2253	772.17	2313.50
☑	2256	1160.61	2319.21
☑	2260	1165.93	2329.84
☑	2264	1171.13	2340.24
☑	2265	1172.79	2343.56
☑	2266	782.98	2345.91
☑	2267	1174.36	2346.71
☑	2268	783.25	2346.73
☑	2270	1177.84	2353.67
☑	2273	786.49	2356.45
☑	2275	789.11	2364.30
☑	2276	1185.62	2369.23
☑	2277	1185.64	2369.26
☑	2278	1188.06	2374.11
☑	2281	793.00	2375.99

☑	2284	1190.40	2378.79
☑	2289	797.43	2389.27
☑	2295	1207.37	2412.73
☑	2299	807.88	2420.62
☑	2302	813.32	2436.94
☑	2306	1232.67	2463.34
☑	2311	1241.75	2481.48
☑	2312	1243.23	2484.45
☑	2314	830.08	2487.22
☑	2317	832.87	2495.59
☑	2319	834.82	2501.42
☑	2323	836.73	2507.15
☑	2326	840.12	2517.34
☑	2327	841.98	2522.93
☑	2332	1267.84	2533.67
☑	2334	848.79	2543.35
☑	2337	853.09	2556.26
☑	2338	854.36	2560.07
☑	2340	862.49	2584.43
☑	2345	868.92	2603.73
☑	2348	875.52	2623.54
☑	2349	876.09	2625.26
☑	2351	879.59	2635.74
☑	2353	882.05	2643.13
☑	2354	1322.73	2643.46
☑	2355	882.31	2643.90
☑	2356	883.53	2647.58
☑	2357	886.45	2656.32
☑	2358	887.99	2660.93
☑	2359	893.48	2677.41
☑	2360	894.53	2680.57
☑	2361	897.19	2688.54
☑	2362	897.75	2690.22
☑	2363	899.99	2696.95

☑	2368	904.77	2711.29
☑	2374	913.69	2738.04
☑	2376	915.54	2743.61
☑	2377	916.75	2747.23
☑	2380	924.00	2768.97
☑	2381	924.54	2770.61
☑	2382	925.01	2772.02
☑	2384	936.53	2806.57
☑	2386	939.35	2815.01
☑	2389	947.28	2838.81
☑	2390	948.60	2842.76
☑	2391	950.25	2847.72
☑	2394	960.77	2879.29
☑	2401	984.54	2950.60
☑	2402	984.91	2951.70
☑	2404	987.64	2959.91
☑	2405	988.24	2961.69
☑	2406	990.40	2968.19
☑	2407	991.13	2970.37
☑	2408	991.78	2972.31
☑	2411	1005.02	3012.03
☑	2412	1005.40	3013.19
☑	2413	1008.35	3022.02
☑	2414	1008.38	3022.12
☑	2415	1010.57	3028.68
☑	2416	1011.86	3032.55
☑	2417	1012.45	3034.31
☑	2418	1012.67	3035.00
☑	2420	1015.59	3043.75
☑	2421	1016.85	3047.54
☑	2424	1025.18	3072.53
☑	2427	1029.55	3085.63
☑	2428	1030.55	3088.62
☑	2432	1040.96	3119.86

☑	2433	1043.14	3126.39
☑	2436	1046.79	3137.36
☑	2439	1048.94	3143.79
☑	2440	1052.27	3153.79
☑	2443	1061.11	3180.31
☑	2444	1061.84	3182.50
☑	2446	1063.77	3188.29
☑	2448	1065.96	3194.87
☑	2449	1071.21	3210.62
☑	2450	1072.35	3214.04
☑	2453	1078.21	3231.61
☑	2457	1090.06	3267.16
☑	2459	1091.58	3271.72
☑	2461	1096.38	3286.11
☑	2463	1099.17	3294.49
☑	2464	1105.07	3312.19
☑	2466	1107.50	3319.49
☑	2467	1107.62	3319.85
☑	2471	1117.10	3348.29
☑	2474	1128.42	3382.25
☑	2478	1139.93	3416.76
☑	2479	1145.89	3434.65
☑	2480	1147.17	3438.48
☑	2485	1163.27	3486.79
☑	2487	1168.26	3501.75
☑	2490	1193.76	3578.26
☑	2492	1209.51	3625.49
☑	2493	1211.92	3632.75
☑	2494	1219.28	3654.81
☑	2495	1221.25	3660.73
☑	2496	1237.57	3709.68
☑	2499	1282.47	3844.39

Search Parameters

```
Type of search         : MS/MS Ion Search
Enzyme                 : Trypsin
Variable modifications : Carbamidomethyl (C),Oxidation (M)
Mass values            : Monoisotopic
Protein Mass           : Unrestricted
Peptide Mass Tolerance : ± 0.6 Da
Fragment Mass Tolerance: ± 0.3 Da
Max Missed Cleavages   : 2
Instrument type        : ESI-TRAP
Number of queries      : 2499
```

Mascot: http://www.matrixscience.com/
