# Supplementary material for: Macrophage-infectivity potentiator of Trypanosoma cruzi (TcMIP) is a new pro-type 1 immuno-stimulating protein for neonatal human cells and vaccines in mice
Source: Front Immunol. 2023 Mar 23;14:1138526. doi: 10.3389/fimmu.2023.1138526 (PMC10077492; doi:10.3389/fimmu.2023.1138526)
Supplement: Supplementary file 10 [file DataSheet_8.pdf]

Mascot Search Results

User : GM
Email : gabriel.mazzucchelli@ulg.ac.be
Search title : Submitted from 090220-adj-oge42-9454-otherEuk-sprot by Mascot Daemon on MASPEC39
MS data file : F:\DATA\Archives\ESQUIRE\2009 esquire\Adjuvac\090220\090220-Adj-OGE42_9454.mgf
Database : Sprot 56.7 (408099 sequences; 147085246 residues)
Taxonomy : Other Eukaryota (3013 sequences)
Timestamp : 23 Feb 2009 at 08:19:41 GMT
Significant hits:

CH60_TRYCR	Chaperonin HSP60, mitochondrial OS=Trypanosoma cruzi GN=HSP60 PE=2 SV=1
TBB_TRYBR	Tubulin beta chain OS=Trypanosoma brucei rhodesiense PE=3 SV=1
FCA1_TRYCR	Flagellar calcium-binding protein OS=Trypanosoma cruzi GN=FCABP PE=1 SV=1
UBIQ_TRYCR	Ubiquitin OS=Trypanosoma cruzi PE=3 SV=1
CALM_TRYBB	Calmodulin OS=Trypanosoma brucei brucei PE=3 SV=2
HSP70_BRELC	Heat shock 70 kDa protein OS=Bremia lactucae GN=HSP70 PE=3 SV=1
EF1AC_PORPU	Elongation factor 1-alpha C OS=Porphyra purpurea GN=TEF-C PE=2 SV=1
RLA3_TRYCR	60S acidic ribosomal protein P2-B OS=Trypanosoma cruzi PE=3 SV=1
H2A_TRYCR	Histone H2A OS=Trypanosoma cruzi PE=3 SV=2
HSP70_LEIAM	Heat shock 70 kDa protein OS=Leishmania amazonensis GN=HSP70 PE=2 SV=1
HSP70_LEIMA	Heat shock 70 kDa protein (Fragment) OS=Leishmania major GN=HSP70 PE=3 SV=1
HSP70_TRYCR	Heat shock 70 kDa protein OS=Trypanosoma cruzi GN=HSP70 PE=3 SV=1
HSP70_ACHKL	Heat shock 70 kDa protein OS=Achlya klebsiana GN=HSP70 PE=2 SV=1
EF1A_TRYBB	Elongation factor 1-alpha OS=Trypanosoma brucei brucei GN=TEF1 PE=2 SV=2
EF1A_ENTHI	Elongation factor 1-alpha OS=Entamoeba histolytica PE=2 SV=1
EF1A_GIALA	Elongation factor 1-alpha (Fragment) OS=Giardia lamblia GN=TEF1 PE=2 SV=1
MIP_TRYCR	Macrophage infectivity potentiator OS=Trypanosoma cruzi GN=MIP PE=1 SV=1
G3PG_CRIFA	Glyceraldehyde-3-phosphate dehydrogenase, glycosomal OS=Crithidia fasciculata GN=GAPDG PE=3 SV=3
RLA1_TRYCR	60S acidic ribosomal protein P1 OS=Trypanosoma cruzi PE=3 SV=1
EF1B_TRYCR	25 kDa elongation factor 1-beta OS=Trypanosoma cruzi PE=2 SV=1
YCF55_PORYE	Uncharacterized protein ycf55 OS=Porphyra yezoensis GN=ycf55 PE=3 SV=1
AGI6_TRYBB	I/6 autoantigen OS=Trypanosoma brucei brucei PE=2 SV=1
RPOA_EUGGR	DNA-directed RNA polymerase subunit alpha OS=Euglena gracilis GN=rpoA PE=3 SV=2
RK12_CYACA	50S ribosomal protein L12, chloroplastic OS=Cyanidium caldarium GN=rpl12 PE=3 SV=1
RK12_ODOSI	50S ribosomal protein L12, chloroplastic OS=Odontella sinensis GN=rpl12 PE=3 SV=1
NDUS1_DICCI	NADH-ubiquinone oxidoreductase 75 kDa subunit OS=Dictyostelium citrinum GN=nad11 PE=3 SV=2

Probability Based Mowse Score

Ions score is -10*Log(P), where P is the probability that the observed match is a random event.
Individual ions scores > 23 indicate identity or extensive homology (p<0.05).
Protein scores are derived from ions scores as a non-probabilistic basis for ranking protein hits.

Score Distribution

Peptide Summary Report

Format As | Peptide Summary | Help

Significance threshold p< 0.05 | Max. number of hits AUTO

Standard scoring ○ MudPIT scoring ◉ | Ions score cut-off 0 | Show sub-sets ☐

Show pop-ups ◉ Suppress pop-ups ○ | Sort unassigned Decreasing Score | Require bold red ☐

Select All | Select None | Search Selected | ☐ **Error tolerant** | Archive Report

1. CH60_TRYCR **Mass:** 59374 **Score:** 474 **Queries matched:** 8
Chaperonin HSP60, mitochondrial OS=Trypanosoma cruzi GN=HSP60 PE=2 SV=1
☐ Check to include this hit in error tolerant search or archive report

	Query	Observed	Mr(expt)	Mr(calc)	Delta	Miss	Score	Expect	Rank	Peptide
☑	1264	557.77	1113.52	1113.64	-0.12	0	88	9.7e-09	1	R.AVSAVATTLGPK.G
☑	1444	602.26	1202.50	1202.58	-0.08	0	102	5.7e-10	1	K.VGGGSEVEVNEK.K
☑	1495	411.86	1232.56	1232.72	-0.16	0	25	0.022	1	R.LPAHTIVLNAGK.E
☑	1611	659.79	1317.56	1317.69	-0.13	0	87	1.6e-08	1	R.NVIIEQSYGAPK.I
☑	1629	670.24	1338.47	1338.61	-0.14	0	87	1.2e-08	1	R.GLIDGETSDYNR.E
☑	1818	798.27	1594.54	1594.78	-0.25	0	69	6.5e-07	1	R.YVNMFEAGIIDPAR.V
☑	1830	806.33	1610.65	1610.78	-0.13	0	(61)	3.6e-06	1	R.YVNMFEAGIIDPAR.V + Oxidation (M)
☑	1881	846.85	1691.68	1691.81	-0.13	0	92	3.5e-09	1	K.VLENNDVTVGYDAQR.D

2. TBB_TRYBR Mass: 49672 Score: 257 Queries matched: 4
Tubulin beta chain OS=Trypanosoma brucei rhodesiense PE=3 SV=1
☐ Check to include this hit in error tolerant search or archive report

	Query	Observed	Mr(expt)	Mr(calc)	Delta	Miss	Score	Expect	Rank	Peptide
☑	1631	671.31	1340.60	1340.64	-0.03	0	74	2.5e-07	1	R.INVYFDEATGGR.Y
☑	1978	616.58	1846.73	1846.87	-0.14	0	(57)	9.9e-06	1	R.EIVCVQAGQCGNQIGSK.F + 2 Carbamidomethyl (C)
☑	1979	924.37	1846.73	1846.87	-0.14	0	119	6.3e-12	1	R.EIVCVQAGQCGNQIGSK.F + 2 Carbamidomethyl (C)
☑	2430	1031.76	3092.26	3092.40	-0.14	0	64	1.7e-06	1	K.FWEVISDEHGVDPTGTYQGDSDLQLER.I

Proteins matching the same set of peptides:
TBB_TRYCR Mass: 49541 Score: 257 Queries matched: 4
Tubulin beta chain OS=Trypanosoma cruzi PE=3 SV=2

3. FCA1_TRYCR Mass: 23721 Score: 226 Queries matched: 7
Flagellar calcium-binding protein OS=Trypanosoma cruzi GN=FCABP PE=1 SV=1
☐ Check to include this hit in error tolerant search or archive report

	Query	Observed	Mr(expt)	Mr(calc)	Delta	Miss	Score	Expect	Rank	Peptide
☑	707	439.17	876.33	876.43	-0.11	0	50	8.3e-05	1	K.LDEFTPR.V
☑	980	495.22	988.43	988.52	-0.09	0	65	2e-06	1	K.VEDPAALFK.E
☑	1686	707.72	1413.42	1413.59	-0.17	0	52	6.1e-05	1	K.LDADGDPDNVPESA.-
☑	1687	707.79	1413.57	1413.59	-0.02	0	(13)	0.38	1	K.LDADGDPDNVPESA.-
☑	1736	737.82	1473.62	1473.68	-0.06	0	69	8.6e-07	1	K.GSEDFVEFLEFR.L
☑	1957	911.35	1820.68	1820.84	-0.16	0	55	2.3e-05	1	K.LCYDEVHSGCLEVLK.L + 2 Carbamidomethyl (C)
☑	1958	607.91	1820.70	1820.84	-0.15	0	(38)	0.001	1	K.LCYDEVHSGCLEVLK.L + 2 Carbamidomethyl (C)

4. UBIQ_TRYCR Mass: 8504 Score: 215 Queries matched: 5
Ubiquitin OS=Trypanosoma cruzi PE=3 SV=1
☐ Check to include this hit in error tolerant search or archive report

Query	Observed	Mr(expt)	Mr(calc)	Delta	Miss	Score	Expect	Rank	Peptide

Query	Observed	Mr(expt)	Mr(calc)	Delta	Miss	Score	Expect	Rank	Peptide
☑ 477	383.18	764.34	764.43	-0.08	0	23	0.028	1	-.MQIFVK.T
☑ 1155	533.24	1064.47	1064.55	-0.08	0	69	7.8e-07	1	R.TLADYNIQK.E
☑ 1164	534.28	1066.55	1066.61	-0.07	0	55	2.1e-05	1	K.ESTLHLVLR.L
☑ 1779	508.55	1522.63	1522.77	-0.15	1	25	0.018	1	K.IQDKEGIPPDQQR.L
☑ 1914	874.39	1746.76	1746.89	-0.13	0	124	2.6e-12	1	K.TIALEVESSDTIENVK.A

---

5. CALM_TRYBB **Mass:** 16828 **Score:** 204 **Queries matched:** 5
Calmodulin OS=Trypanosoma brucei brucei PE=3 SV=2
☐ Check to include this hit in error tolerant search or archive report

Query	Observed	Mr(expt)	Mr(calc)	Delta	Miss	Score	Expect	Rank	Peptide
☑ 1637	675.28	1348.55	1348.62	-0.07	0	74	2.8e-07	1	K.LTDEEVDEMIR.E
☑ 1976	922.88	1843.74	1843.88	-0.15	1	52	3.6e-05	1	K.EAFSLFDKDGDGTITTK.E
☑ 1977	615.59	1843.74	1843.88	-0.14	1	(47)	0.00011	1	K.EAFSLFDKDGDGTITTK.E
☑ 906	964.19	1926.37	1926.85	-0.47	0	78	2.7e-07	1	R.EADVDGDGQINYEEFVK.M
☑ 277	647.95	1940.81	1940.88	-0.07	2	43	0.0011	1	R.KMQDSDSEEEIKEAFR.V

Proteins matching the same set of peptides:
CALM_TRYBG **Mass:** 16828 **Score:** 204 **Queries matched:** 5
Calmodulin OS=Trypanosoma brucei gambiense PE=3 SV=2
CALM_TRYCR **Mass:** 16814 **Score:** 204 **Queries matched:** 5
Calmodulin OS=Trypanosoma cruzi GN=CALA2 PE=3 SV=2

---

6. HSP70_BRELC **Mass:** 74041 **Score:** 96 **Queries matched:** 3
Heat shock 70 kDa protein OS=Bremia lactucae GN=HSP70 PE=3 SV=1
☐ Check to include this hit in error tolerant search or archive report

Query	Observed	Mr(expt)	Mr(calc)	Delta	Miss	Score	Expect	Rank	Peptide
☑ 1488	614.76	1227.51	1227.62	-0.11	0	66	1.8e-06	1	R.VEIIANDQGNR.T
☑ 1751	744.30	1486.59	1486.69	-0.10	0	52	5.4e-05	1	R.TTPSYVAFTDTER.L
☑ 114	554.00	1658.98	1658.89	0.09	0	5	5.4	1	R.IINEPTAAAIAYGLDK.K

---

7. EF1AC_PORPU **Mass:** 49142 **Score:** 92 **Queries matched:** 3

Elongation factor 1-alpha C OS=Porphyra purpurea GN=TEF-C PE=2 SV=1

☐ Check to include this hit in error tolerant search or archive report

	Query	Observed	Mr(expt)	Mr(calc)	Delta	Miss	Score	Expect	Rank	Peptide
☑	935	488.28	974.54	974.54	-0.00	0	52	6e-05	1	R.LPLQDVYK.I
☑	1056	513.25	1024.50	1024.60	-0.11	0	61	4.2e-06	1	K.IGGIGTVPVGR.V
	1446	602.78	1203.55	1203.65	-0.11	2	0	6.4	3	K.KLEDSPKMIK.S + Oxidation (M)

---

8. RLA3_TRYCR **Mass:** 10919 **Score:** 87 **Queries matched:** 1

60S acidic ribosomal protein P2-B OS=Trypanosoma cruzi PE=3 SV=1

☐ Check to include this hit in error tolerant search or archive report

	Query	Observed	Mr(expt)	Mr(calc)	Delta	Miss	Score	Expect	Rank	Peptide
☑	1269	558.74	1115.47	1115.62	-0.15	0	87	1.4e-08	1	R.SVATLVAEGAAK.M

---

9. H2A_TRYCR **Mass:** 14357 **Score:** 69 **Queries matched:** 1

Histone H2A OS=Trypanosoma cruzi PE=3 SV=2

☐ Check to include this hit in error tolerant search or archive report

	Query	Observed	Mr(expt)	Mr(calc)	Delta	Miss	Score	Expect	Rank	Peptide
☑	1355	578.77	1155.53	1155.56	-0.03	0	69	9.1e-07	1	R.HDDDLGMLLK.D

---

10. HSP70_LEIAM **Mass:** 71153 **Score:** 66 **Queries matched:** 3

Heat shock 70 kDa protein OS=Leishmania amazonensis GN=HSP70 PE=2 SV=1

☐ Check to include this hit in error tolerant search or archive report

	Query	Observed	Mr(expt)	Mr(calc)	Delta	Miss	Score	Expect	Rank	Peptide
	1488	614.76	1227.51	1227.62	-0.11	0	66	1.8e-06	1	R.VEIIANDQGNR.T
☑	1744	741.35	1480.69	1480.81	-0.13	1	18	0.14	1	K.RSVHDVVLVGGSTR.I
	114	554.00	1658.98	1658.89	0.09	0	5	5.4	1	R.IINEPTAAAIAYGLDK.G

---

11. HSP70_LEIMA **Mass:** 56500 **Score:** 66 **Queries matched:** 3

Heat shock 70 kDa protein (Fragment) OS=Leishmania major GN=HSP70 PE=3 SV=1

☐ Check to include this hit in error tolerant search or archive report

Query	Observed	Mr(expt)	Mr(calc)	Delta	Miss	Score	Expect	Rank	Peptide
1488	614.76	1227.51	1227.62	-0.11	0	66	1.8e-06	1	R.LDIIANDQGNR.T
1744	741.35	1480.69	1480.81	-0.13	1	18	0.14	1	K.RSVHDVVLVGGSTR.I
114	554.00	1658.98	1658.89	0.09	0	5	5.4	1	R.IINEPTAAAIAYGLDK.G

---

12. HSP70_TRYCR **Mass:** 73762 **Score:** 66 **Queries matched:** 3

Heat shock 70 kDa protein OS=Trypanosoma cruzi GN=HSP70 PE=3 SV=1

☐ Check to include this hit in error tolerant search or archive report

	Query	Observed	Mr(expt)	Mr(calc)	Delta	Miss	Score	Expect	Rank	Peptide
	1488	614.76	1227.51	1227.62	-0.11	0	66	1.8e-06	1	R.VEIIANDQGNR.T
☑	282	**650.24**	**1298.46**	**1298.60**	**-0.14**	**0**	**14**	**0.78**	**1**	**R.FEELCGELFR.G + Carbamidomethyl (C)**
	114	554.00	1658.98	1658.89	0.09	0	5	5.4	1	R.IINEPTAAAIAYGLDK.V

---

13. HSP70_ACHKL **Mass:** 71222 **Score:** 66 **Queries matched:** 2

Heat shock 70 kDa protein OS=Achlya klebsiana GN=HSP70 PE=2 SV=1

☐ Check to include this hit in error tolerant search or archive report

	Query	Observed	Mr(expt)	Mr(calc)	Delta	Miss	Score	Expect	Rank	Peptide
	1488	614.76	1227.51	1227.62	-0.11	0	66	1.8e-06	1	R.VEIIANDQGNR.T
☑	163	**589.89**	**1766.64**	**1766.90**	**-0.26**	**1**	**1**	**23**	**1**	**R.IEAKNGLENYAYNLR.N**

---

14. EF1A_TRYBB **Mass:** 49003 **Score:** 61 **Queries matched:** 3

Elongation factor 1-alpha OS=Trypanosoma brucei brucei GN=TEF1 PE=2 SV=2

☐ Check to include this hit in error tolerant search or archive report

Query	Observed	Mr(expt)	Mr(calc)	Delta	Miss	Score	Expect	Rank	Peptide
1056	513.25	1024.50	1024.60	-0.11	0	61	4.2e-06	1	K.IGGIGTVPVGR.V
1733	**736.26**	**1470.51**	**1470.64**	**-0.13**	**1**	**2**	**4.9**	**5**	**K.MDDKTVNYGQER.Y + Oxidation (M)**
1749	**743.85**	**1485.68**	**1485.61**	**0.07**	**1**	**4**	**2.3**	**2**	**K.QMVVCCNKMDDK.T + Carbamidomethyl (C); Oxidation (M)**

15. EF1A_ENTHI **Score:** 52 **Queries matched:** 2
Elongation factor 1-alpha OS=Entamoeba histolytica PE=2 SV=1
☐ Check to include this hit in error tolerant search or archive report

Query	Observed	Mr(expt)	Mr(calc)	Delta	Miss	Score	Expect	Rank	Peptide
935	488.28	974.54	974.54	-0.00	0	52	6e-05	1	R.LPLQDVYK.I
1583	**649.25**	**1296.48**	**1296.61**	**-0.14**	**1**	**2**	**4.2**	**2**	**K.MDAIQYKQER.Y + Oxidation (M)**

16. EF1A_GIALA **Mass:** 43922 **Score:** 52 **Queries matched:** 2
Elongation factor 1-alpha (Fragment) OS=Giardia lamblia GN=TEF1 PE=2 SV=1
☐ Check to include this hit in error tolerant search or archive report

Query	Observed	Mr(expt)	Mr(calc)	Delta	Miss	Score	Expect	Rank	Peptide
935	488.28	974.54	974.54	-0.00	0	52	6e-05	1	R.LPIQDVYK.I
1733	736.26	1470.51	1470.68	-0.17	2	2	4.9	5	K.MDDGQVKYSKER.Y + Oxidation (M)

17. MIP_TRYCR **Mass:** 22122 **Score:** 47 **Queries matched:** 2
Macrophage infectivity potentiator OS=Trypanosoma cruzi GN=MIP PE=1 SV=1
☐ Check to include this hit in error tolerant search or archive report

	Query	Observed	Mr(expt)	Mr(calc)	Delta	Miss	Score	Expect	Rank	Peptide
☑	1037	**508.75**	**1015.48**	**1015.58**	**-0.10**	**0**	**47**	**0.00013**	**1**	**K.LPSGLVFQR.I**
☑	1750	**496.27**	**1485.80**	**1485.83**	**-0.03**	**0**	**4**	**2.2**	**1**	**R.GKPTTFRPNEVIK.G**

18. G3PG_CRIFA **Mass:** 39063 **Score:** 43 **Queries matched:** 1
Glyceraldehyde-3-phosphate dehydrogenase, glycosomal OS=Crithidia fasciculata GN=GAPDG PE=3 SV=3
☐ Check to include this hit in error tolerant search or archive report

	Query	Observed	Mr(expt)	Mr(calc)	Delta	Miss	Score	Expect	Rank	Peptide
☑	1668	**692.84**	**1383.66**	**1383.77**	**-0.11**	**0**	**43**	**0.00029**	**1**	**R.AAAVNIIPSTTGAAK.A**

Proteins matching the same set of peptides:

G3PG_LEIME **Mass:** 39008 **Score:** 43 **Queries matched:** 1
Glyceraldehyde-3-phosphate dehydrogenase, glycosomal OS=Leishmania mexicana GN=GAPG PE=1 SV=3

G3PG_TRYCR **Mass:** 39036 **Score:** 43 **Queries matched:** 1
Glyceraldehyde-3-phosphate dehydrogenase, glycosomal OS=Trypanosoma cruzi PE=1 SV=1

---

19. RLA1_TRYCR **Mass:** 10747 **Score:** 42 **Queries matched:** 1
60S acidic ribosomal protein P1 OS=Trypanosoma cruzi PE=3 SV=1

☐ Check to include this hit in error tolerant search or archive report

	Query	Observed	Mr(expt)	Mr(calc)	Delta	Miss	Score	Expect	Rank	Peptide
☑	2335	**850.38**	**2548.12**	**2548.34**	**-0.22**	**0**	**42**	**0.00024**	**1**	**K.VSFGGVAPAAGGATAAPAAAAAAAAPAAAAAK.K**

---

20. EF1B_TRYCR **Mass:** 24450 **Score:** 31 **Queries matched:** 1
25 kDa elongation factor 1-beta OS=Trypanosoma cruzi PE=2 SV=1

☐ Check to include this hit in error tolerant search or archive report

	Query	Observed	Mr(expt)	Mr(calc)	Delta	Miss	Score	Expect	Rank	Peptide
☑	1774	**506.92**	**1517.74**	**1517.85**	**-0.10**	**1**	**31**	**0.0042**	**1**	**K.LFLGGTKPSKEDVK.L**

---

21. YCF55_PORYE **Mass:** 37791 **Score:** 28 **Queries matched:** 2
Uncharacterized protein ycf55 OS=Porphyra yezoensis GN=ycf55 PE=3 SV=1

☐ Check to include this hit in error tolerant search or archive report

	Query	Observed	Mr(expt)	Mr(calc)	Delta	Miss	Score	Expect	Rank	Peptide
☑	610	**831.08**	**830.07**	**830.49**	**-0.41**	**0**	**7**	**4**	**1**	**R.TSLEILR.R**
	1488	614.76	1227.51	1227.70	-0.19	0	28	0.01	3	K.NNLIWNTIIK.N

---

22. AGI6_TRYBB **Mass:** 27747 **Score:** 26 **Queries matched:** 2
I/6 autoantigen OS=Trypanosoma brucei brucei PE=2 SV=1

☐ Check to include this hit in error tolerant search or archive report

Query	Observed	Mr(expt)	Mr(calc)	Delta	Miss	Score	Expect	Rank	Peptide

☑	1623	666.24	1330.47	1330.67	-0.20	1	5	2.3	1	M.LCPPDVAFEKR.H + Carbamidomethyl (C)
☑	1684	705.34	1408.66	1408.78	-0.12	0	26	0.014	1	K.HLVALAPDGIFTR.A

---

23. RPOA_EUGGR **Score:** 25 **Queries matched:** 1
DNA-directed RNA polymerase subunit alpha OS=Euglena gracilis GN=rpoA PE=3 SV=2
☐ Check to include this hit in error tolerant search or archive report

Query	Observed	Mr(expt)	Mr(calc)	Delta	Miss	Score	Expect	Rank	Peptide
1164	534.28	1066.55	1066.65	-0.11	1	25	0.02	2	K.YLKIYVLR.S

---

24. RK12_CYACA **Score:** 24 **Queries matched:** 1
50S ribosomal protein L12, chloroplastic OS=Cyanidium caldarium GN=rpl12 PE=3 SV=1
☐ Check to include this hit in error tolerant search or archive report

Query	Observed	Mr(expt)	Mr(calc)	Delta	Miss	Score	Expect	Rank	Peptide
1269	558.74	1115.47	1115.65	-0.19	1	24	0.024	2	R.SITGLGLKEAK.E

Proteins matching the same set of peptides:
RK12_GRATL **Score:** 24 **Queries matched:** 1

---

25. RK12_ODOSI **Score:** 24 **Queries matched:** 1
50S ribosomal protein L12, chloroplastic OS=Odontella sinensis GN=rpl12 PE=3 SV=1
☐ Check to include this hit in error tolerant search or archive report

Query	Observed	Mr(expt)	Mr(calc)	Delta	Miss	Score	Expect	Rank	Peptide
1269	558.74	1115.47	1115.65	-0.19	1	24	0.024	2	R.SLTGLGLKEAK.E

Proteins matching the same set of peptides:
RK12_PHATR **Score:** 24 **Queries matched:** 1
RK12_PORPU **Score:** 24 **Queries matched:** 1

---

26. NDUS1_DICCI **Mass:** 79554 **Score:** 24 **Queries matched:** 1
NADH-ubiquinone oxidoreductase 75 kDa subunit OS=Dictyostelium citrinum GN=nad11 PE=3 SV=2

☐ Check to include this hit in error tolerant search or archive report

	Query	Observed	Mr(expt)	Mr(calc)	Delta	Miss	Score	Expect	Rank	Peptide
☑	1600	654.28	1306.54	1306.67	-0.13	2	24	0.022	1	K.KIMEAGSWKNK.T + Oxidation (M)

Peptide matches not assigned to protein hits: (no details means no match)

	Query	Observed	Mr(expt)	Mr(calc)	Delta	Miss	Score	Expect	Rank	Peptide
☑	1763	750.88	1499.74	1499.85	-0.11	2	23	0.036	1	ALDMKDVKQAILR
☑	1070	516.26	1030.50	1030.61	-0.11	0	21	0.054	1	VIDLLAPYK
☑	858	471.74	941.47	941.53	-0.06	0	21	0.053	1	QNIVNNIK
☑	1411	592.77	1183.53	1183.66	-0.13	0	17	0.13	1	NVTAGANPISIK
☑	1067	515.28	1028.54	1028.59	-0.05	0	17	0.12	1	VLEVEALTR
☑	1535	630.80	1259.59	1259.65	-0.06	1	16	0.18	1	QCIKDAAVDLK + Carbamidomethyl (C)
☑	1380	583.29	1164.56	1164.67	-0.11	2	16	0.14	1	KIHEQNKIR
☑	666	427.16	852.31	852.42	-0.11	0	14	0.17	1	LFNMSNK
☑	1729	734.80	1467.58	1467.86	-0.28	2	14	0.27	1	ALSGGQKQRIAIAR
☑	1275	561.26	1120.50	1120.66	-0.16	2	13	0.28	1	RNRHNIIAK
☑	2369	905.73	2714.17	2714.22	-0.06	1	13	0.26	1	GNVMLLENVRFYSEENGENAEER + Oxidation (M)
☑	573	406.17	810.32	810.41	-0.08	0	13	0.26	1	FCVSISR
☑	1659	691.24	1380.47	1380.69	-0.23	1	13	0.38	1	ELESKGYEWLK
☑	1739	738.33	1474.65	1474.72	-0.07	0	12	0.36	1	FIDCYLQFLEK + Carbamidomethyl (C)
☑	40	490.61	489.60	489.23	0.37	0	12	0.74	1	GSGNR
☑	817	465.71	929.40	929.55	-0.15	1	12	0.45	1	TLIEKAQK
☑	1540	633.28	1264.54	1264.70	-0.16	1	12	0.45	1	TNSKVLDFTIK
☑	68	519.21	1036.41	1036.63	-0.22	2	12	0.75	1	TLIRKNHR
☑	1731	490.55	1468.61	1468.79	-0.17	1	12	0.41	1	MADILFRLGYVR + Oxidation (M)
☑	930	487.22	972.43	972.58	-0.14	1	12	0.33	1	ELLPFAKR
☑	51	504.17	1509.47	1509.95	-0.48	2	11	1.2	1	LIAPRNIKLGIFR
☑	1665	462.16	1383.45	1383.72	-0.27	1	11	0.47	1	HGIEKVAEQVMK + Oxidation (M)
☑	1807	788.30	1574.58	1574.86	-0.28	2	11	0.5	1	MREQTVSAKILQR + Oxidation (M)
☑	1497	617.76	1233.51	1233.67	-0.16	2	11	0.46	1	KICKSCGLIR + 2 Carbamidomethyl (C)
☑	1467	609.27	1216.52	1216.70	-0.19	1	11	0.55	1	LASETAKTLVGK
☑	1290	564.27	1126.52	1126.62	-0.10	0	11	0.53	1	TLPGVAVLDDK

☑	1660	691.27	1380.52	1380.69	-0.16	1	11	0.58	1	WGKDVGCAFLTGK
☑	1708	481.26	1440.75	1440.80	-0.05	1	11	0.55	1	IQSNKIVDQLQR
☑	1821	801.33	1600.65	1600.88	-0.23	1	10	0.63	1	MLQIELQKWIQR + Oxidation (M)
☑	1005	502.71	1003.40	1003.50	-0.11	1	10	0.68	1	LSQTDRER
☑	32	480.01	1437.01	1436.69	0.31	1	10	1.9	1	RIQSMGFTNNNR
☑	1319	571.22	1140.43	1140.56	-0.13	2	10	0.43	1	YDKRTDCIK
☑	1655	688.30	1374.58	1374.72	-0.14	0	10	0.71	1	ENMELGLTVTLR
☑	1701	717.82	1433.63	1433.63	-0.01	2	9	0.63	1	KRMCMSPSEHR + Carbamidomethyl (C); Oxidation (M)
☑	1875	843.38	1684.74	1684.91	-0.18	1	9	0.66	1	VWEIKLENGEISIR
☑	237	625.35	1248.69	1248.70	-0.01	0	9	1.8	1	ADLIAYLETLK
☑	1661	461.21	1380.60	1380.69	-0.09	1	9	0.82	1	WGKDVGCAFLTGK
☑	1081	518.27	1034.53	1034.56	-0.03	0	9	0.79	1	MDLFLQIR
☑	1139	529.77	1057.53	1057.64	-0.11	1	9	0.82	1	LIDLKISEK
☑	934	488.18	974.34	974.53	-0.19	0	9	1.1	1	MTPIMGVVK
☑	1835	811.75	1621.48	1621.90	-0.42	0	9	0.91	1	YGLPLIFEIETISK
☑	1210	545.73	1089.45	1089.62	-0.17	2	8	1.1	1	MAADLLKGKK + Oxidation (M)
☑	1972	613.23	1836.67	1836.85	-0.18	1	8	0.96	1	IMAMREMILSDDEGAR
☑	1432	598.26	1194.51	1194.64	-0.13	1	8	0.7	1	VLCHGQARVR + Carbamidomethyl (C)
☑	1970	612.90	1835.67	1835.97	-0.30	1	8	1	1	SFDLDVLKDSCALILK + Carbamidomethyl (C)
☑	74	523.77	1045.53	1045.47	0.06	0	8	2.6	1	EAEQNAAADK
☑	77	526.27	1050.53	1050.55	-0.03	1	8	1.4	1	CKIDQLFK + Carbamidomethyl (C)
☑	1666	692.75	1383.48	1383.79	-0.32	2	8	0.95	1	MLLNLFKFSKK + Oxidation (M)
☑	321	673.31	1344.61	1344.75	-0.14	0	8	3.5	1	DIITQVSTLDIK
☑	125	562.89	561.88	561.31	0.57	0	8	1.1	1	AVTGSK
☑	1586	650.16	1298.31	1298.73	-0.42	2	7	1.6	1	EAAVLRRELDK
☑	156	584.69	1167.36	1167.61	-0.25	1	7	2.5	1	IKSFVMSGQR + Oxidation (M)
☑	456	379.16	756.31	756.47	-0.16	0	7	1.1	1	IEGVVIK
☑	34	483.06	964.10	964.43	-0.32	0	7	3.6	1	SPCGTGTSAK + Carbamidomethyl (C)
☑	1651	687.23	1372.44	1372.61	-0.18	1	7	1.3	1	MASREDCVYTAK
☑	1308	568.73	1135.44	1135.63	-0.19	2	7	1.2	1	RVLRGQDHR
☑	1406	591.31	1180.61	1180.75	-0.15	1	7	1.1	1	AVALVVPSLKGK
☑	123	559.97	1676.90	1676.87	0.03	0	7	4.2	1	TLEALSLDSCINITK + Carbamidomethyl (C)
☑	137	571.18	1140.35	1140.59	-0.24	1	6	3.2	1	LASFYERAGK
☑	1389	585.79	1169.57	1169.75	-0.18	2	6	1.1	1	VKTIVDVLRK

☑	820	466.23	930.44	930.45	-0.01	0	6	1.8	1	GLGTENNAR
☑	1749	743.85	1485.68	1485.84	-0.16	2	6	1.3	1	HLLKFMKDDVLK
☑	81	532.48	1594.42	1594.69	-0.28	0	6	5.1	1	GWCDEYFFIPYR
☑	2303	815.68	2444.02	2444.06	-0.03	2	6	1.1	1	CRSRMMNHCVTAVGYGSNSNGK + Carbamidomethyl (C); Oxidation (M)
☑	521	790.36	789.35	789.42	-0.07	0	6	1.8	1	VVSTDAAK
☑	170	593.76	1778.26	1777.90	0.36	0	6	5.6	1	GPDLETQATAAAAALHNK
☑	561	806.57	805.56	805.40	0.16	0	6	5	1	MIEEIR + Oxidation (M)
☑	1939	894.35	1786.69	1786.84	-0.15	1	6	1.4	1	EAQEKATQANQNDAAAK
☑	1481	612.85	1223.68	1223.63	0.05	0	6	1.6	1	VYEGQVLFNR
☑	2336	852.62	2554.84	2555.28	-0.43	2	6	1.9	1	EAGGITQKIGAYEVEIDYKDQTK
☑	1800	783.40	1564.79	1564.91	-0.12	2	6	1.5	1	VGKILGPRGLMPSPK + Oxidation (M)
☑	1928	883.81	1765.61	1765.91	-0.30	1	6	1.6	1	MALQSLPCRHGSPTIR
☑	461	758.85	757.85	757.36	0.49	0	6	3.2	1	QPEAEGK
☑	1249	554.25	1106.49	1106.64	-0.15	2	6	1.5	1	RKIYCVLR + Carbamidomethyl (C)
☑	1917	585.26	1752.76	1752.86	-0.10	1	5	1.5	1	SFWGPPHGIEVERDK
☑	1583	649.25	1296.48	1296.63	-0.15	2	5	1.8	1	MNKDFCIKNK + Carbamidomethyl (C)
☑	1682	703.75	1405.50	1405.65	-0.16	1	5	1.9	1	DEIERMVNDASK
☑	1947	600.91	1799.72	1799.98	-0.25	0	5	1.6	1	IVEQAVSNVTPFVEIR
☑	1696	714.33	1426.64	1426.73	-0.08	0	5	1.8	1	LIIQIGCYSDFR
☑	39	489.33	488.32	488.30	0.03	0	5	5.1	1	GVSVK
☑	1789	772.32	1542.63	1542.75	-0.11	1	5	1.6	1	MAPPASASKAGAAEER
☑	320	672.44	671.43	671.41	0.03	1	5	5.7	1	RDILR
☑	1421	594.80	1187.58	1187.57	0.01	1	5	2.1	1	QMVTKFGMSK + 2 Oxidation (M)
☑	73	523.28	1044.54	1044.55	-0.00	0	5	4.3	1	IMVICPNAK + Carbamidomethyl (C)
☑	1716	726.82	1451.62	1451.74	-0.12	2	5	1.5	1	YHVEPRRGEGPR
☑	1714	725.28	1448.56	1448.68	-0.12	1	5	1.8	1	KFEEIDSAPEER
☑	1596	651.84	1301.66	1301.66	-0.01	0	5	2.4	1	QSVNEPMITGVK
☑	366	700.45	1398.88	1398.62	0.26	1	5	5.6	1	CATVKMSENGTSR + Oxidation (M)
☑	1577	647.28	1292.55	1292.64	-0.09	0	5	1.9	1	FSQALASDPTTR
☑	101	545.17	544.16	544.32	-0.16	0	5	6.4	1	ELVGK
☑	1622	665.84	1329.66	1329.71	-0.04	1	5	2	1	VMDIRVLTPDR + Oxidation (M)
☑	1649	686.25	1370.49	1370.75	-0.25	1	5	2.4	1	KVDLHAYIWAR
☑	821	931.58	930.57	930.49	0.08	0	5	2.6	1	GGDPLIFGR
☑	1402	590.25	1178.49	1178.64	-0.15	1	5	1.6	1	NTSYVAIKQR

☑	674	859.25	2574.74	2575.29	-0.55	1	4	8.7	1	ADETVVEEKTEFDVIIQEVPSAK
☑	1728	734.79	1467.57	1467.86	-0.29	2	4	2.2	1	ALSGGQKQRIAIAR
☑	1639	679.28	1356.54	1356.76	-0.22	0	4	2.6	1	SAVLQNSPSTIIK
☑	2488	1172.74	3515.21	3515.70	-0.48	1	4	2.2	1	DTGIIPCQEGVDGVPCYGNLGPEWELDVATIKK
☑	126	563.25	562.25	562.30	-0.05	0	4	4.8	1	SSIEK
☑	1508	620.25	1238.49	1238.61	-0.12	2	4	2	1	AKTACPKSDYR
☑	1377	583.23	1164.45	1164.61	-0.16	0	4	1.9	1	GLAPEVPTPER
☑	1815	793.82	1585.63	1585.89	-0.26	2	4	1.8	1	IIKVSEIMHGKTSK + Oxidation (M)
☑	2220	751.38	2251.13	2251.07	0.06	1	4	1.9	1	MTGPTMQAVPCGINGFGRIGR + Carbamidomethyl (C); 2 Oxidation (M)
☑	1405	591.25	1180.48	1180.54	-0.06	1	4	2	1	SIAPCKQCMK + Carbamidomethyl (C); Oxidation (M)
☑	165	590.90	1769.69	1769.88	-0.19	0	4	9.6	1	AEQIGLEESEPTLAAGR
☑	14	434.14	1299.40	1299.56	-0.17	0	4	5.1	1	HMMNMTHNIR + Oxidation (M)
☑	207	613.64	1837.89	1837.93	-0.03	0	4	6.1	1	MLQGPHELMPVMQAIK + Oxidation (M)
☑	1603	655.29	1308.56	1308.65	-0.09	1	4	2.5	1	LMKSAIGEGMTR + Oxidation (M)
☑	1591	650.81	1299.61	1299.71	-0.10	2	4	2.7	1	LEKIEDELRR
☑	1277	561.74	1121.47	1121.64	-0.17	1	4	2.5	1	RTINQVVHR
☑	1648	685.33	1368.65	1368.80	-0.15	1	4	2.5	1	SIKLSIAEGLNPK
☑	534	796.14	1590.26	1590.79	-0.53	1	4	8.1	1	YAASRGVPCVADGGLR
☑	849	469.72	937.42	937.40	0.03	0	4	2.5	1	MPEGEYGR
☑	1209	545.73	1089.44	1089.63	-0.19	1	4	3.2	1	SGNFLGIVKR
☑	109	548.69	547.68	547.33	0.35	1	4	2.3	1	AKTTK
☑	1207	545.20	1088.38	1088.57	-0.19	0	4	2.8	1	TGSLNIDLEK
☑	1446	602.78	1203.55	1203.66	-0.12	1	4	2.9	1	KLIQMLESAR + Oxidation (M)
☑	1104	523.24	1044.47	1044.61	-0.14	1	3	3.5	1	RIDMILLR + Oxidation (M)
☑	1720	727.77	1453.53	1453.77	-0.24	2	3	2.6	1	KALDPKNICNPGK + Carbamidomethyl (C)
☑	1254	555.20	1108.38	1108.53	-0.14	0	3	2.4	1	NNSNIYQTR
☑	1852	824.78	1647.55	1647.85	-0.29	1	3	2.3	1	VWLLNTGYAGGRADR
☑	2073	674.77	2021.30	2021.04	0.26	2	3	1.9	1	ALDEGYEVSCLVRNLRK + Carbamidomethyl (C)
☑	565	807.79	1613.57	1613.94	-0.37	1	3	9.2	1	ITDITGVSLRGEIIK
☑	1885	848.83	1695.64	1695.95	-0.31	1	3	2.5	1	AVVVIGDENGKVGVGVGK
☑	1904	572.91	1715.70	1715.92	-0.22	2	3	3.1	1	ALNKPKAMASRDALCK
☑	255	634.40	633.39	633.33	0.06	0	3	3.3	1	EVATSK
☑	1746	742.37	1482.72	1482.82	-0.10	2	3	3.1	1	AAGLNKGSGEPNIKK
☑	1748	743.79	1485.57	1485.64	-0.07	1	3	2.7	1	MHGHAEACMRVR + Carbamidomethyl (C); 2 Oxidation (M)

☑	95	540.25	1617.72	1617.85	-0.14	2	3	5.5	1	PIGYPLVKAMDKDR + Oxidation (M)
☑	176	596.89	1787.65	1788.05	-0.40	0	3	7.2	1	VSTIQQILPVLEHIAK
☑	1733	736.26	1470.51	1470.76	-0.25	1	3	4.3	1	CDAIQIRLASPER
☑	2077	676.23	2025.65	2025.92	-0.27	2	3	3.6	1	KAASEWDDAYCEEVVRR
☑	2105	690.23	2067.68	2068.28	-0.60	1	3	3.8	1	DSLLNRLLLSLGILLFIR
☑	1766	753.28	1504.54	1504.81	-0.27	1	3	2.9	1	TIAITREGVIMACK
☑	100	545.13	1088.24	1088.55	-0.30	0	3	12	1	GDTANNSIAVK
☑	1966	612.29	1833.84	1833.90	-0.06	1	3	2.6	1	ISLNYYDMMKLSVNK + Oxidation (M)
☑	1920	878.47	1754.92	1754.97	-0.05	1	3	2.9	1	IKIASPQQVLSWTER
☑	1612	660.23	1318.44	1318.58	-0.14	0	3	3.4	1	CCALVVCAGAAPAG + 2 Carbamidomethyl (C)
☑	627	840.38	839.37	839.44	-0.07	0	3	6.5	1	IHQICAR
☑	548	800.25	2397.74	2398.33	-0.59	1	3	12	1	ETGALKVIVISTGSELNVAVEAAK
☑	1573	646.32	1290.63	1290.73	-0.10	1	3	3.1	1	VLFVGTKNQASK
☑	1083	518.71	1035.40	1035.54	-0.14	2	3	4.4	1	KSMIEREK + Oxidation (M)
☑	588	818.29	1634.56	1634.83	-0.27	1	3	10	1	MVSSLANELREGTTK
☑	1085	519.21	1036.41	1036.53	-0.12	1	3	2.6	1	VDLRDTYR
☑	1012	1007.27	1006.27	1006.49	-0.22	1	2	12	1	DRMSAATGAK
☑	1554	639.76	1277.51	1277.72	-0.21	0	2	3.1	1	ISALQHQVQVR
☑	481	767.08	1532.14	1531.82	0.31	1	2	9.9	1	NNNISSKSLSDILK
☑	530	794.48	1586.94	1586.87	0.07	2	2	9.1	1	KTYTYQNTLISKK
☑	1752	744.77	1487.53	1487.73	-0.20	0	2	3.8	1	LEEIEDMLINNR
☑	2293	805.02	2412.05	2412.11	-0.07	1	2	2.7	1	TLEAGAMVLADRGICCVDEFDK + Carbamidomethyl (C)
☑	185	601.24	600.23	600.40	-0.17	0	2	11	1	TVLLR
☑	1842	815.33	1628.64	1628.89	-0.25	2	2	3.3	1	LSCAGRNNRGLITVR
☑	162	589.85	588.84	588.32	0.52	0	2	10	1	VDISR
☑	1791	776.25	1550.49	1549.93	0.56	2	2	3.4	1	RLPDLVVIVDQKR
☑	1287	563.78	1125.55	1125.68	-0.13	0	2	4	1	EIAALLLIDR
☑	1589	650.73	1299.44	1299.71	-0.27	1	2	4	1	CNGVLEGIRIAR
☑	1534	630.78	1259.54	1259.66	-0.12	0	2	4.2	1	DSNLSIEELIK
☑	284	650.77	649.76	650.34	-0.58	0	2	7.4	1	YELAR
☑	1679	468.53	1402.57	1402.80	-0.23	2	2	3	1	ALKSMGITLKDAR
☑	1769	755.36	1508.71	1508.86	-0.15	2	2	2.8	1	EIFIKNKDYIVK
☑	37	488.75	1463.24	1462.80	0.44	1	2	13	1	QSLTLEQEFKLK
☑	1401	588.77	1175.52	1175.63	-0.12	1	2	3.9	1	AMKINESIVR + Oxidation (M)

☑	1838	542.23	1623.67	1623.95	-0.28	2	2	3.4	1	SKLVVISANCPPIRK
☑	580	814.01	813.00	812.50	0.50	1	2	14	1	IALNARR
☑	1361	1158.89	3473.65	3473.56	0.09	1	2	12	1	AWDSSMNLMPAFPTWNVMGMMNNPWYRVK
☑	1674	695.82	1389.63	1389.69	-0.07	0	2	3.9	1	AGSLATEVMENIR
☑	1790	775.80	1549.59	1549.87	-0.28	1	2	3.8	1	EVKLALTLNSAYTK
☑	2194	1097.40	2192.78	2193.18	-0.40	2	2	4.1	1	MFNQKIIEQASGKLTGSLTK
☑	1555	640.32	1278.62	1278.61	0.01	0	2	4.2	1	MEGSGSIAMRPK + Oxidation (M)
☑	1092	1039.33	3114.97	3114.54	0.42	2	2	13	1	MKGSASKGNTIAFGDYALQATEPVWLTSR + Oxidation (M)
☑	66	517.22	516.21	516.30	-0.09	0	2	2.1	1	LTGAR
☑	1663	692.28	1382.56	1382.80	-0.25	2	2	3.9	1	VHDGIIFQAKKK
☑	1251	555.02	1108.02	1107.70	0.32	0	2	6.9	1	AILAPEIIIR
☑	1316	570.27	1138.52	1138.68	-0.16	1	2	4.1	1	INLASPQRIK
☑	1418	594.27	1186.52	1186.53	-0.01	0	2	5.1	1	DNSIVDFFCK
☑	1961	611.29	1830.84	1830.98	-0.14	2	2	3.6	1	DAMVNLYLKIRQDPR
☑	1721	731.82	1461.62	1461.87	-0.26	2	1	3.7	1	QLKQGLFMLLKK + Oxidation (M)
☑	1796	521.21	1560.60	1560.82	-0.22	1	1	3.9	1	CVQSLGLRISCIR + 2 Carbamidomethyl (C)
☑	1566	643.77	1285.54	1285.67	-0.14	0	1	5.4	1	QLGSNSLLNNAR
☑	584	815.40	814.39	814.47	-0.08	1	1	7.2	1	RAAQIEK
☑	1398	1175.40	2348.79	2348.20	0.59	2	1	14	1	DLTKTAVISGGMIPKVNCCIR + 2 Carbamidomethyl (C); Oxidation (M)
☑	1621	665.32	1328.62	1328.59	0.03	0	1	4.4	1	MTFTDLNTENK + Oxidation (M)
☑	61	514.17	1539.50	1539.79	-0.29	1	1	9	1	INNRDVPDILEDK
☑	1007	1005.71	3014.10	3014.67	-0.56	2	1	16	1	DQIKLKQQIADAQLLLSTITIDYDLR
☑	2203	737.66	2209.97	2210.16	-0.19	2	1	3.4	1	RRPGESVGGARPSDSAARTVGK
☑	819	466.17	930.32	930.50	-0.19	0	1	5.6	1	DVITIENK
☑	553	803.31	2406.92	2407.26	-0.34	0	1	13	1	IEETTQASINTQQLSVITYLR
☑	1414	593.81	1185.60	1185.65	-0.05	1	1	4.7	1	DPKACTIILR + Carbamidomethyl (C)
☑	1792	776.85	1551.69	1551.80	-0.11	1	1	4.6	1	KDIEGTITCQFIK + Carbamidomethyl (C)
☑	1762	750.33	1498.65	1498.70	-0.05	2	1	4.6	1	ECKTCSLTETKEK
☑	868	947.80	2840.39	2840.45	-0.06	1	1	14	1	LNLPQANPGLHMSFTGSPGTGKTTVATK + Oxidation (M)
☑	149	580.67	1739.00	1738.91	0.09	2	1	11	1	QDLPDLVYKTEYKK
☑	452	751.90	2252.69	2252.31	0.38	2	1	18	1	KLCPRVPLTVTIPYILNQK + Carbamidomethyl (C)
☑	1441	601.78	1201.55	1201.74	-0.19	2	1	5.2	1	KISTLSLGQKK
☑	1757	747.31	1492.60	1492.79	-0.19	0	1	3.6	1	VQINASNIVSFSSK
☑	1344	576.48	1150.95	1150.56	0.38	0	1	5	1	NGAVQVDAYSK

☑	25	468.15	1401.44	1401.65	-0.21	0	1	11	1	EWPQWLDICR + Carbamidomethyl (C)
☑	2020	638.59	1912.75	1913.00	-0.26	1	1	4	1	WLETDKPGDRSLAQGLK
☑	873	476.20	950.38	950.48	-0.10	1	1	5.6	1	EFISDRGK
☑	2333	845.90	2534.68	2535.15	-0.47	2	1	4.4	1	HVFCTVCVVERWRCPSCQR + 4 Carbamidomethyl (C)
☑	1592	1301.36	3901.05	3901.14	-0.09	2	1	13	1	FLTVLNCLINNKQFLNLSDVEVLKILNESELIR + Carbamidomethyl (C)
☑	305	661.25	1980.72	1980.98	-0.27	0	1	16	1	LMTFGVPGITVTNVMGCGK + Carbamidomethyl (C)
☑	946	489.94	977.86	977.46	0.40	0	1	6.4	1	VQNEFDAR
☑	2024	640.66	1918.95	1919.03	-0.08	2	1	3.7	1	KLLNQMELLLSTCKNR + Oxidation (M)
☑	1795	521.20	1560.58	1560.77	-0.19	2	1	4.5	1	DFRGISYKTFDGR
☑	378	706.11	705.10	705.43	-0.33	1	1	4.4	1	LYVRR
☑	646	423.68	845.34	845.47	-0.13	0	1	6.7	1	VALEACIK
☑	1214	1092.43	3274.27	3274.52	-0.25	1	1	14	1	CPEVLFQPSFIGMESSGIHDCTFKTIMK + 2 Carbamidomethyl (C); Oxidation (M)
☑	1711	721.88	1441.74	1441.68	0.06	1	1	5.4	1	YCWATIGRICEK
☑	1244	552.72	1103.43	1103.56	-0.13	0	1	6.2	1	VELQNSASTR
☑	624	839.93	2516.77	2517.33	-0.57	2	1	20	1	CSLHLKSFDLDVLKDSCALILK + Carbamidomethyl (C)
☑	118	557.04	1668.10	1667.76	0.33	0	1	17	1	EQVFEMPTGGAAIMR + 2 Oxidation (M)
☑	1956	607.27	1818.79	1818.89	-0.10	1	1	5	1	LVFGHTFSDHMLKCK + Carbamidomethyl (C)
☑	130	566.31	1695.91	1695.83	0.09	1	1	10	1	MKSAVMAVACAAAPGFR + Oxidation (M)
☑	2254	772.35	2314.03	2314.12	-0.09	2	1	3.9	1	ERFLYCMEGVNRAAAATGEVK
☑	250	631.86	1261.71	1261.73	-0.02	0	1	30	1	IIVQAIIDYSK
☑	1768	503.87	1508.57	1508.77	-0.19	1	1	4	1	LFGGLVGESESKMR
☑	17	445.24	888.47	888.49	-0.02	0	1	12	1	FRPLSNR
☑	617	835.77	1669.53	1669.93	-0.40	2	1	16	1	SRKPLMEAAREAIAK
☑	2343	863.63	2587.86	2588.20	-0.34	1	1	5.8	1	NDGMSIRFDDNAAVIINQDNNPR
☑	1866	558.34	1671.99	1671.86	0.12	0	1	5.5	1	ILAQAAMEGQIDQLR + Oxidation (M)
☑	2101	687.29	2058.84	2059.02	-0.18	1	1	3.8	1	NFSFFNFYTESFRILK
☑	1732	491.16	1470.46	1470.76	-0.30	1	1	8.1	1	CDAIQIRLASPER
☑	2233	761.71	2282.12	2282.22	-0.10	2	0	3.3	1	YKGETKIFAPEEISSMVLLK
☑	926	972.36	2914.05	2914.31	-0.26	1	0	16	1	CICINCNHIAECSTYHLIESRHK + 4 Carbamidomethyl (C)
☑	1704	479.84	1436.51	1436.77	-0.26	2	0	5.8	1	MTNKTTKIQATGK + Oxidation (M)
☑	2124	700.29	2097.85	2098.23	-0.38	2	0	3.9	1	KNQFANKLIVIDLLSVQR
☑	1620	443.88	1328.61	1328.70	-0.09	1	0	5.4	1	RCQTANIASLPR
☑	634	421.71	841.40	841.43	-0.03	0	0	4.6	1	TENLTHK
☑	1522	627.32	1252.62	1252.79	-0.16	2	0	5.5	1	ILGPVTKELRK

☑	182	599.99	1197.96	1197.73	0.22	1	0	16	1	LKEEVNLIIK
☑	1006	1005.55	2009.08	2008.93	0.15	1	0	16	1	EMCRMLDLDPNILTADK + 2 Oxidation (M)
☑	311	664.20	663.20	663.30	-0.10	0	0	22	1	MGEVGR + Oxidation (M)
☑	1644	683.28	1364.54	1364.58	-0.03	1	0	4.5	1	DSSMSAYMMSKK
☑	516	788.36	2362.07	2362.15	-0.08	2	0	16	1	RETGNSIRFDDNAAVIINDDK
☑	1141	530.21	1058.40	1058.54	-0.13	0	0	7.8	1	NVEGTGGQVAK
☑	1908	580.60	1738.76	1739.02	-0.26	2	0	6.3	1	IDLDGKIALIEAAKAAK
☑	1798	522.28	1563.83	1563.80	0.03	1	0	4.3	1	MQKELTALAPSTMK + Oxidation (M)
☑	1169	1071.03	3210.06	3209.94	0.13	1	0	18	1	MIEFSLLTKITLSFTILFLILSIISIIK
☑	1333	1149.56	3445.65	3445.74	-0.10	2	0	13	1	AEFSKEVGSIICMIDLVIGYTAIQSMAKWAR + Oxidation (M)
☑	1691	710.25	1418.48	1418.84	-0.36	1	0	5.4	1	ATVVLPHGTGKAIR
☑	1675	464.41	1390.22	1390.70	-0.48	1	0	12	1	GRSLSAAQEAVMR + Oxidation (M)
☑	954	490.71	979.40	979.53	-0.13	1	0	4.8	1	VLRGQDHR
☑	958	982.51	1963.02	1962.95	0.07	0	0	15	1	VNGVQIECLESMVINSR + Carbamidomethyl (C); Oxidation (M)
☑	1381	1166.46	2330.90	2330.97	-0.07	0	0	15	1	GMNEDSVHSHCGLWDWDSLK + Oxidation (M)
☑	883	319.17	954.47	954.60	-0.12	1	0	5.3	1	RALLDVLR
☑	751	901.90	900.89	900.51	0.38	2	0	9.1	1	KSTGGKAPR
☑	839	935.40	934.39	934.53	-0.14	1	0	8.1	1	RITGLTMK + Oxidation (M)
☑	2189	728.52	2182.54	2182.03	0.51	2	0	7.5	1	CHTIMNCTKTCPKHLNPGK + Carbamidomethyl (C)
☑	1625	667.72	1333.42	1333.72	-0.30	0	0	5.9	1	ELNSYNLQIIK
☑	1645	683.38	1364.74	1364.64	0.10	0	0	4.8	1	IACLECGSSQLNK
☑	1672	695.33	1388.65	1388.68	-0.03	0	0	5.7	1	ENLLPNASTSESK
☑	78	526.48	1576.41	1576.88	-0.47	0	0	17	1	VQSLILNSVQSVYK
☑	1528	629.31	1256.60	1256.70	-0.09	1	0	7.1	1	SLPGVTVLTNKE
☑	1	*284.93*	*283.92*							
☑	2	*288.21*	*287.20*							
☑	3	*327.62*	*326.61*							
☑	4	*359.08*	*358.08*							
☑	5	*364.10*	*363.09*							
☑	6	*371.00*	*369.99*							
☑	7	*371.08*	*370.07*							
☑	8	*205.97*	*409.92*							
☑	9	*412.74*	*411.73*							
☑	10	*415.80*	*414.79*							

☑	11	423.58	422.58
☑	12	215.02	428.02
☑	13	432.70	431.69
☑	15	439.91	438.90
☑	16	440.72	439.71
☑	18	223.99	445.97
☑	19	452.55	451.54
☑	20	455.17	454.16
☑	21	455.79	454.78
☑	22	459.44	458.43
☑	23	460.40	459.39
☑	24	462.42	461.42
☑	26	469.46	468.46
☑	27	471.24	470.23
☑	28	474.52	473.51
☑	29	475.40	474.39
☑	30	477.26	476.25
☑	31	479.64	478.63
☑	33	482.19	481.18
☑	35	485.26	484.25
☑	36	486.18	485.17
☑	38	488.85	487.85
☑	41	492.80	491.80
☑	42	493.14	492.13
☑	43	494.22	493.21
☑	44	494.86	493.85
☑	45	495.34	494.33
☑	46	496.18	495.17
☑	47	497.17	496.16
☑	48	498.23	497.22
☑	49	499.44	498.43
☑	50	503.23	502.23
☑	52	505.33	504.32
☑	53	505.85	504.85

☑	54	506.27	505.26
☑	55	507.35	506.34
☑	56	508.92	507.91
☑	57	511.20	510.20
☑	58	512.51	511.50
☑	59	512.79	511.78
☑	60	513.05	512.04
☑	62	514.50	513.49
☑	63	514.81	513.80
☑	64	514.85	513.84
☑	65	515.17	514.16
☑	67	518.50	517.49
☑	69	519.23	518.22
☑	70	520.49	519.48
☑	71	520.83	519.82
☑	72	521.35	520.35
☑	75	524.62	523.61
☑	76	525.37	524.36
☑	79	527.43	526.42
☑	80	529.48	528.48
☑	82	533.02	532.01
☑	83	533.91	532.90
☑	84	534.54	533.53
☑	85	535.25	534.24
☑	86	536.20	535.19
☑	87	537.08	536.08
☑	88	537.71	536.71
☑	89	537.96	536.95
☑	90	538.78	537.78
☑	91	539.17	538.16
☑	92	539.28	538.27
☑	93	540.00	539.00
☑	94	540.21	539.20
☑	96	542.26	541.26

☑	97	542.33	541.32
☑	98	544.22	543.21
☑	99	544.77	543.76
☑	102	546.04	545.03
☑	103	546.11	545.10
☑	104	546.29	545.28
☑	105	546.39	545.39
☑	106	546.55	545.55
☑	107	547.91	546.90
☑	108	548.51	547.50
☑	110	549.78	548.77
☑	111	550.18	549.17
☑	112	553.23	552.22
☑	113	553.25	552.24
☑	115	554.33	553.32
☑	116	555.00	554.00
☑	117	555.58	554.58
☑	119	557.18	556.17
☑	120	557.49	556.48
☑	121	557.95	556.94
☑	122	559.74	558.73
☑	124	562.71	561.70
☑	127	564.99	563.98
☑	128	565.26	564.25
☑	129	565.32	564.31
☑	131	566.36	565.35
☑	132	567.02	566.01
☑	133	567.77	566.76
☑	134	568.04	567.04
☑	135	569.99	568.98
☑	136	570.40	569.40
☑	138	571.23	570.22
☑	139	571.61	570.60
☑	140	572.11	571.10

☑	141	574.67	573.66
☑	142	575.33	574.32
☑	143	575.38	574.37
☑	144	576.71	575.70
☑	145	576.95	575.94
☑	146	579.70	578.69
☑	147	579.83	578.82
☑	148	580.66	579.65
☑	150	580.69	579.68
☑	151	581.15	580.15
☑	152	581.28	580.27
☑	153	582.03	581.03
☑	154	582.60	581.60
☑	155	583.22	582.21
☑	157	586.74	585.73
☑	158	587.65	586.64
☑	159	587.92	586.91
☑	160	588.68	587.67
☑	161	589.39	588.38
☑	164	590.33	589.32
☑	166	590.99	589.98
☑	167	591.17	590.17
☑	168	591.70	590.69
☑	169	593.50	592.49
☑	171	593.78	592.77
☑	172	594.25	593.24
☑	173	594.96	593.95
☑	174	595.64	594.63
☑	175	595.90	594.90
☑	177	597.37	596.36
☑	178	597.75	596.74
☑	179	598.37	597.36
☑	180	599.32	598.31
☑	181	599.36	598.35

☑	183	600.58	599.57
☑	184	600.77	599.76
☑	186	601.68	600.68
☑	187	601.93	600.92
☑	188	602.26	601.25
☑	189	602.35	601.34
☑	190	602.51	601.50
☑	191	602.58	601.58
☑	192	603.67	602.67
☑	193	603.93	602.93
☑	194	604.31	603.30
☑	195	604.39	603.38
☑	196	604.73	603.72
☑	197	606.04	605.03
☑	198	607.35	606.35
☑	199	608.18	607.17
☑	200	608.29	607.28
☑	201	608.57	607.57
☑	202	608.69	607.69
☑	203	609.98	608.97
☑	204	610.25	609.24
☑	205	610.94	609.94
☑	206	612.71	611.70
☑	208	613.92	612.92
☑	209	615.24	614.24
☑	210	615.47	614.47
☑	211	615.67	614.66
☑	212	616.11	615.10
☑	213	616.23	615.22
☑	214	616.50	615.49
☑	215	616.67	615.66
☑	216	616.84	615.84
☑	217	617.26	616.25
☑	218	617.85	616.84

☑	219	618.66	617.66
☑	220	618.98	617.98
☑	221	619.30	618.29
☑	222	619.91	618.90
☑	223	620.26	619.26
☑	224	620.83	619.82
☑	225	620.97	619.96
☑	226	621.43	620.42
☑	227	621.64	620.63
☑	228	622.31	621.30
☑	229	622.33	621.32
☑	230	622.42	621.41
☑	231	622.92	621.91
☑	232	623.34	622.34
☑	233	624.29	623.28
☑	234	624.66	623.65
☑	235	624.73	623.73
☑	236	624.81	623.80
☑	238	625.80	624.79
☑	239	625.86	624.85
☑	240	626.04	625.04
☑	241	626.86	625.85
☑	242	627.01	626.01
☑	243	627.21	626.20
☑	244	629.61	628.61
☑	245	629.99	628.98
☑	246	630.92	629.91
☑	247	631.10	630.09
☑	248	631.27	630.26
☑	249	631.48	630.47
☑	251	633.74	632.73
☑	252	633.77	632.76
☑	253	634.10	633.09
☑	254	634.33	633.33

☑	256	634.96	633.96
☑	257	636.99	635.98
☑	258	637.92	636.92
☑	259	638.22	637.21
☑	260	638.41	637.41
☑	261	638.63	637.62
☑	262	639.23	638.22
☑	263	639.67	638.66
☑	264	640.29	639.28
☑	265	640.35	639.34
☑	266	640.35	639.35
☑	267	641.67	640.67
☑	268	642.35	641.34
☑	269	643.00	641.99
☑	270	644.24	643.24
☑	271	644.66	643.66
☑	272	646.47	645.46
☑	273	646.74	645.73
☑	274	646.97	645.96
☑	275	647.33	646.33
☑	276	647.90	646.89
☑	278	648.38	647.38
☑	279	648.96	647.95
☑	280	649.27	648.26
☑	281	649.86	648.85
☑	283	650.70	649.69
☑	285	650.77	649.76
☑	286	650.92	649.91
☑	287	326.10	650.18
☑	288	651.64	650.63
☑	289	652.17	651.16
☑	290	653.86	652.85
☑	291	653.86	652.85
☑	292	654.23	653.22

☑	293	654.69	653.68
☑	294	654.99	653.98
☑	295	655.09	654.08
☑	296	656.51	655.50
☑	297	658.30	657.29
☑	298	658.65	657.64
☑	299	659.55	658.55
☑	300	659.69	658.68
☑	301	659.86	658.85
☑	302	660.31	659.31
☑	303	660.64	659.64
☑	304	660.84	659.83
☑	306	662.11	661.10
☑	307	662.40	661.39
☑	308	663.06	662.05
☑	309	663.25	662.24
☑	310	663.39	662.39
☑	312	666.24	665.23
☑	313	668.06	667.05
☑	314	668.76	667.76
☑	315	669.21	668.20
☑	316	669.74	668.73
☑	317	669.86	668.85
☑	318	671.19	670.18
☑	319	672.27	671.26
☑	322	673.83	672.82
☑	323	673.92	672.91
☑	324	674.59	673.58
☑	325	675.17	674.16
☑	326	675.25	674.24
☑	327	338.40	674.79
☑	328	676.52	675.52
☑	329	676.61	675.61
☑	330	677.14	676.13

☑	331	677.29	676.28
☑	332	677.31	676.30
☑	333	677.40	676.39
☑	334	677.80	676.80
☑	335	677.87	676.87
☑	336	677.89	676.89
☑	337	677.97	676.97
☑	338	678.33	677.32
☑	339	682.27	681.26
☑	340	683.01	682.00
☑	341	683.21	682.20
☑	342	684.28	683.27
☑	343	685.29	684.28
☑	344	685.73	684.72
☑	345	686.97	685.96
☑	346	687.09	686.08
☑	347	687.69	686.68
☑	348	687.96	686.95
☑	349	688.44	687.43
☑	350	688.74	687.73
☑	351	689.03	688.03
☑	352	689.25	688.24
☑	353	689.36	688.35
☑	354	691.82	690.81
☑	355	692.43	691.42
☑	356	693.18	692.17
☑	357	694.37	693.36
☑	358	695.32	694.31
☑	359	697.33	696.32
☑	360	698.27	697.27
☑	361	699.27	698.27
☑	362	699.30	698.29
☑	363	699.94	698.93
☑	364	700.14	699.13

☑	365	700.32	699.31
☑	367	700.88	699.87
☑	368	700.89	699.88
☑	369	701.28	700.27
☑	370	701.32	700.31
☑	371	702.08	701.07
☑	372	702.84	701.83
☑	373	703.98	702.97
☑	374	704.96	703.96
☑	375	705.05	704.05
☑	376	705.09	704.08
☑	377	705.74	704.73
☑	379	706.21	705.21
☑	380	706.59	705.59
☑	381	707.74	706.73
☑	382	707.96	706.95
☑	383	708.64	707.63
☑	384	709.54	708.53
☑	385	709.60	708.60
☑	386	709.89	708.89
☑	387	710.10	709.09
☑	388	710.90	709.89
☑	389	711.64	710.64
☑	390	712.66	711.65
☑	391	712.79	711.78
☑	392	713.27	712.27
☑	393	714.44	713.44
☑	394	714.76	713.75
☑	395	715.31	714.30
☑	396	358.29	714.56
☑	397	715.65	714.65
☑	398	717.47	716.46
☑	399	718.19	717.18
☑	400	718.37	717.36

☑	401	719.01	718.00
☑	402	719.60	718.60
☑	403	719.73	718.72
☑	404	719.81	718.80
☑	405	720.18	719.17
☑	406	720.21	719.21
☑	407	722.45	721.44
☑	408	723.76	722.75
☑	409	724.93	723.93
☑	410	725.03	724.02
☑	411	725.05	724.05
☑	412	725.20	724.19
☑	413	725.26	724.25
☑	414	726.14	725.13
☑	415	726.32	725.31
☑	416	728.95	727.94
☑	417	729.19	728.18
☑	418	729.41	728.40
☑	419	730.76	729.76
☑	420	731.09	730.09
☑	421	731.12	730.12
☑	422	731.39	730.39
☑	423	731.61	730.60
☑	424	731.87	730.87
☑	425	732.30	731.29
☑	426	732.34	731.33
☑	427	733.26	732.26
☑	428	734.43	733.42
☑	429	735.30	734.29
☑	430	735.37	734.36
☑	431	737.39	736.39
☑	432	738.35	737.34
☑	433	738.82	737.81
☑	434	739.04	738.03

☑	435	739.54	738.53
☑	436	739.58	738.57
☑	437	739.61	738.60
☑	438	740.60	739.59
☑	439	741.23	740.23
☑	440	742.55	741.54
☑	441	743.70	742.69
☑	442	743.95	742.95
☑	443	745.26	744.25
☑	444	745.71	744.70
☑	445	745.78	744.77
☑	446	746.31	745.30
☑	447	746.38	745.38
☑	448	747.27	746.26
☑	449	747.68	746.68
☑	450	751.71	750.70
☑	451	751.78	750.77
☑	453	753.30	752.30
☑	454	753.84	752.83
☑	455	755.94	754.93
☑	457	757.46	756.46
☑	458	757.61	756.61
☑	459	757.81	756.81
☑	460	757.92	756.91
☑	462	758.97	757.96
☑	463	760.50	759.49
☑	464	760.61	759.61
☑	465	761.36	760.36
☑	466	761.90	760.90
☑	467	763.41	762.40
☑	468	763.83	762.82
☑	469	763.86	762.85
☑	470	763.98	762.97
☑	471	764.09	763.08

☑	472	764.13	763.13
☑	473	764.33	763.32
☑	474	764.68	763.67
☑	475	765.04	764.04
☑	476	765.34	764.33
☑	478	765.82	764.81
☑	479	766.70	765.70
☑	480	766.77	765.76
☑	482	767.14	766.14
☑	483	767.91	766.90
☑	484	768.85	767.84
☑	485	769.14	768.13
☑	486	769.30	768.29
☑	487	769.88	768.87
☑	488	770.88	769.87
☑	489	771.10	770.09
☑	490	771.29	770.29
☑	491	771.79	770.79
☑	492	772.18	771.17
☑	493	772.39	771.39
☑	494	772.67	771.67
☑	495	773.28	772.27
☑	496	774.33	773.32
☑	497	774.49	773.48
☑	498	774.52	773.51
☑	499	774.93	773.92
☑	500	776.19	775.19
☑	501	776.25	775.24
☑	502	777.12	776.11
☑	503	777.29	776.28
☑	504	779.23	778.23
☑	505	779.51	778.50
☑	506	780.40	779.40
☑	507	781.02	780.02

☑	508	*781.09*	*780.08*
☑	509	*784.19*	*783.18*
☑	510	*784.94*	*783.93*
☑	511	*785.10*	*784.09*
☑	512	*393.11*	*784.21*
☑	513	*785.69*	*784.69*
☑	514	*786.70*	*785.70*
☑	515	*787.87*	*786.87*
☑	517	*788.74*	*787.73*
☑	518	*789.29*	*788.29*
☑	519	*789.40*	*788.39*
☑	520	*789.84*	*788.83*
☑	522	*791.65*	*790.64*
☑	523	*792.47*	*791.47*
☑	524	*792.83*	*791.82*
☑	525	*793.09*	*792.08*
☑	526	*793.31*	*792.30*
☑	527	*793.90*	*792.89*
☑	528	*793.94*	*792.93*
☑	529	*794.30*	*793.29*
☑	531	*794.85*	*793.85*
☑	532	*795.49*	*794.48*
☑	533	*796.11*	*795.10*
☑	535	*796.39*	*795.39*
☑	536	*796.46*	*795.45*
☑	537	*796.50*	*795.49*
☑	538	*796.52*	*795.52*
☑	539	*398.86*	*795.71*
☑	540	*796.89*	*795.88*
☑	541	*797.08*	*796.07*
☑	542	*399.24*	*796.47*
☑	543	*798.76*	*797.76*
☑	544	*799.15*	*798.14*
☑	545	*799.85*	*798.84*

☑	546	800.19	799.18
☑	547	400.63	799.24
☑	549	800.44	799.43
☑	550	801.70	800.69
☑	551	802.66	801.66
☑	552	802.73	801.72
☑	554	803.45	802.44
☑	555	803.49	802.48
☑	556	804.63	803.62
☑	557	804.84	803.83
☑	558	805.22	804.21
☑	559	403.13	804.24
☑	560	805.82	804.81
☑	562	807.35	806.34
☑	563	807.40	806.39
☑	564	807.78	806.77
☑	566	808.38	807.38
☑	567	808.91	807.91
☑	568	809.02	808.01
☑	569	810.52	809.51
☑	570	810.76	809.75
☑	571	810.86	809.85
☑	572	811.18	810.18
☑	574	812.42	811.42
☑	575	812.57	811.56
☑	576	812.94	811.94
☑	577	813.14	812.13
☑	578	813.39	812.39
☑	579	813.49	812.48
☑	581	814.27	813.27
☑	582	814.76	813.75
☑	583	814.97	813.96
☑	585	817.27	816.26
☑	586	817.42	816.41

☑	587	817.62	816.61
☑	589	818.82	817.81
☑	590	820.57	819.56
☑	591	820.77	819.76
☑	592	821.60	820.59
☑	593	821.83	820.82
☑	594	822.01	821.00
☑	595	822.65	821.65
☑	596	412.02	822.03
☑	597	412.20	822.39
☑	598	824.33	823.32
☑	599	824.49	823.49
☑	600	824.67	823.66
☑	601	825.18	824.17
☑	602	825.86	824.85
☑	603	826.41	825.40
☑	604	826.75	825.74
☑	605	414.17	826.33
☑	606	827.89	826.89
☑	607	828.48	827.47
☑	608	829.08	828.07
☑	609	830.26	829.25
☑	611	831.18	830.17
☑	612	831.66	830.65
☑	613	831.87	830.87
☑	614	832.25	831.24
☑	615	832.62	831.61
☑	616	834.15	833.14
☑	618	836.75	835.74
☑	619	837.08	836.07
☑	620	838.01	837.00
☑	621	838.15	837.14
☑	622	838.37	837.36
☑	623	838.65	837.64

☑	625	840.16	839.15
☑	626	840.23	839.22
☑	628	840.47	839.46
☑	629	840.50	839.50
☑	630	840.52	839.52
☑	631	841.83	840.82
☑	632	842.05	841.04
☑	633	842.26	841.25
☑	635	842.48	841.48
☑	636	842.50	841.49
☑	637	843.43	842.42
☑	638	844.04	843.03
☑	639	844.72	843.71
☑	640	845.69	844.69
☑	641	845.81	844.80
☑	642	845.88	844.87
☑	643	845.93	844.92
☑	644	846.04	845.03
☑	645	423.58	845.15
☑	647	846.46	845.45
☑	648	846.72	845.71
☑	649	846.90	845.89
☑	650	848.09	847.08
☑	651	848.14	847.14
☑	652	848.71	847.70
☑	653	849.20	848.19
☑	654	849.36	848.35
☑	655	849.45	848.44
☑	656	850.36	849.35
☑	657	850.41	849.40
☑	658	850.70	849.69
☑	659	850.71	849.71
☑	660	851.40	850.40
☑	661	851.74	850.73

☑	662	851.79	850.79
☑	663	851.95	850.94
☑	664	851.97	850.96
☑	665	852.02	851.01
☑	667	427.24	852.46
☑	668	854.08	853.07
☑	669	854.58	853.57
☑	670	855.06	854.06
☑	671	857.03	856.03
☑	672	857.96	856.95
☑	673	858.37	857.36
☑	675	860.08	859.07
☑	676	860.14	859.13
☑	677	860.19	859.18
☑	678	860.65	859.64
☑	679	860.83	859.82
☑	680	860.92	859.91
☑	681	861.52	860.51
☑	682	863.16	862.16
☑	683	864.12	863.11
☑	684	433.67	865.33
☑	685	867.90	866.89
☑	686	434.87	867.72
☑	687	868.77	867.76
☑	688	868.90	867.89
☑	689	869.28	868.27
☑	690	869.95	868.95
☑	691	870.01	869.00
☑	692	871.72	870.71
☑	693	871.93	870.93
☑	694	872.60	871.59
☑	695	872.86	871.85
☑	696	873.06	872.05
☑	697	873.18	872.17

☑	698	873.76	872.75
☑	699	873.80	872.79
☑	700	873.92	872.91
☑	701	875.17	874.16
☑	702	438.19	874.36
☑	703	876.26	875.26
☑	704	876.71	875.70
☑	705	876.89	875.88
☑	706	877.30	876.29
☑	708	877.87	876.86
☑	709	878.60	877.59
☑	710	879.07	878.06
☑	711	879.71	878.70
☑	712	879.78	878.77
☑	713	879.96	878.95
☑	714	880.46	879.45
☑	715	880.82	879.81
☑	716	880.84	879.83
☑	717	881.02	880.02
☑	718	881.21	880.20
☑	719	881.30	880.29
☑	720	884.33	883.32
☑	721	884.35	883.34
☑	722	884.46	883.45
☑	723	885.04	884.03
☑	724	885.17	884.16
☑	725	885.33	884.33
☑	726	886.02	885.01
☑	727	886.32	885.31
☑	728	886.85	885.84
☑	729	889.28	888.28
☑	730	889.32	888.32
☑	731	890.08	889.07
☑	732	890.13	889.13

☑	733	445.90	889.78
☑	734	891.75	890.74
☑	735	892.06	891.05
☑	736	892.60	891.59
☑	737	892.61	891.60
☑	738	893.50	892.50
☑	739	893.66	892.65
☑	740	893.68	892.67
☑	741	894.15	893.15
☑	742	894.26	893.25
☑	743	894.52	893.51
☑	744	895.71	894.70
☑	745	895.73	894.73
☑	746	895.95	894.94
☑	747	896.13	895.13
☑	748	899.89	898.88
☑	749	900.78	899.78
☑	750	901.33	900.32
☑	752	902.40	901.39
☑	753	902.43	901.42
☑	754	902.50	901.49
☑	755	902.98	901.97
☑	756	903.17	902.16
☑	757	903.59	902.58
☑	758	903.76	902.76
☑	759	903.95	902.94
☑	760	904.17	903.16
☑	761	452.71	903.40
☑	762	904.51	903.51
☑	763	904.86	903.85
☑	764	453.28	904.54
☑	765	905.91	904.90
☑	766	453.46	904.91
☑	767	906.03	905.03

768	906.65	905.64
769	906.91	905.90
770	907.03	906.02
771	908.04	907.03
772	908.14	907.14
773	908.28	907.28
774	909.21	908.20
775	909.56	908.56
776	909.96	908.95
777	909.99	908.98
778	910.15	909.14
779	910.84	909.83
780	911.88	910.87
781	456.61	911.20
782	456.67	911.32
783	913.15	912.15
784	913.90	912.89
785	915.06	914.06
786	915.69	914.68
787	916.64	915.63
788	917.04	916.04
789	917.45	916.44
790	917.88	916.87
791	919.02	918.01
792	919.41	918.40
793	919.75	918.74
794	920.11	919.10
795	920.71	919.70
796	921.00	920.00
797	921.19	920.18
798	922.71	921.71
799	923.06	922.05
800	923.64	922.63
801	923.67	922.66

☑	802	924.30	923.29
☑	803	925.15	924.14
☑	804	925.70	924.69
☑	805	925.93	924.92
☑	806	926.46	925.46
☑	807	463.76	925.50
☑	808	926.90	925.89
☑	809	927.08	926.07
☑	810	927.59	926.58
☑	811	927.97	926.96
☑	812	928.50	927.50
☑	813	928.67	927.66
☑	814	929.08	928.07
☑	815	929.35	928.34
☑	816	929.99	928.99
☑	818	930.64	929.64
☑	822	931.83	930.82
☑	823	931.92	930.91
☑	824	932.27	931.26
☑	825	932.35	931.34
☑	826	932.76	931.75
☑	827	932.83	931.82
☑	828	933.11	932.10
☑	829	933.22	932.21
☑	830	933.24	932.23
☑	831	933.36	932.35
☑	832	933.58	932.57
☑	833	933.71	932.70
☑	834	934.49	933.48
☑	835	934.54	933.53
☑	836	934.85	933.84
☑	837	934.93	933.92
☑	838	935.17	934.16
☑	840	935.46	934.45

☑	841	935.49	934.48
☑	842	936.22	935.22
☑	843	936.30	935.29
☑	844	936.56	935.55
☑	845	936.61	935.60
☑	846	936.71	935.71
☑	847	936.98	935.97
☑	848	937.83	936.83
☑	850	938.60	937.59
☑	851	939.46	938.45
☑	852	940.27	939.26
☑	853	940.86	939.85
☑	854	941.72	940.71
☑	855	942.06	941.06
☑	856	942.21	941.20
☑	857	942.30	941.29
☑	859	942.60	941.59
☑	860	944.04	943.04
☑	861	944.24	943.24
☑	862	945.71	944.70
☑	863	945.89	944.89
☑	864	946.25	945.24
☑	865	946.67	945.67
☑	866	946.95	945.94
☑	867	947.71	946.70
☑	869	948.13	947.12
☑	870	949.39	948.38
☑	871	950.04	949.03
☑	872	950.73	949.72
☑	874	951.99	950.98
☑	875	952.42	951.41
☑	876	952.59	951.58
☑	877	952.91	951.90
☑	878	477.07	952.12

879	953.54	952.54
880	954.02	953.01
881	477.58	953.14
882	954.70	953.69
884	956.51	955.51
885	956.73	955.72
886	956.76	955.75
887	957.71	956.70
888	957.74	956.73
889	958.09	957.09
890	958.52	957.51
891	958.55	957.54
892	480.14	958.28
893	959.41	958.40
894	959.58	958.57
895	959.75	958.74
896	960.30	959.29
897	961.08	960.07
898	961.89	960.89
899	961.98	960.98
900	962.23	961.22
901	962.82	961.81
902	962.88	961.88
903	963.41	962.40
904	482.21	962.42
905	963.55	962.54
907	964.31	963.30
908	966.19	965.18
909	966.74	965.74
910	966.95	965.94
911	967.04	966.04
912	485.02	968.02
913	969.25	968.25
914	969.51	968.51

☑	915	969.72	968.72
☑	916	970.08	969.07
☑	917	970.35	969.34
☑	918	970.62	969.61
☑	919	486.21	970.40
☑	920	971.90	970.89
☑	921	971.93	970.92
☑	922	971.93	970.92
☑	923	972.08	971.07
☑	924	972.24	971.23
☑	925	972.31	971.30
☑	927	973.06	972.05
☑	928	973.39	972.38
☑	929	973.41	972.40
☑	931	973.58	972.57
☑	932	974.84	973.83
☑	933	975.17	974.17
☑	936	976.01	975.00
☑	937	976.31	975.31
☑	938	977.36	976.35
☑	939	977.37	976.36
☑	940	489.23	976.45
☑	941	977.64	976.64
☑	942	977.83	976.82
☑	943	977.89	976.88
☑	944	978.27	977.27
☑	945	978.43	977.42
☑	947	978.89	977.89
☑	948	978.92	977.91
☑	949	979.40	978.39
☑	950	490.22	978.42
☑	951	979.74	978.73
☑	952	980.00	979.00
☑	953	980.32	979.31

☑	955	980.88	979.87
☑	956	981.78	980.77
☑	957	981.82	980.82
☑	959	982.54	981.53
☑	960	982.75	981.74
☑	961	982.81	981.81
☑	962	982.87	981.86
☑	963	983.52	982.52
☑	964	983.70	982.69
☑	965	984.29	983.28
☑	966	984.29	983.28
☑	967	984.34	983.33
☑	968	984.70	983.69
☑	969	984.90	983.90
☑	970	984.96	983.95
☑	971	985.14	984.14
☑	972	986.10	985.10
☑	973	987.39	986.38
☑	974	987.44	986.43
☑	975	987.47	986.46
☑	976	987.66	986.65
☑	977	988.56	987.55
☑	978	988.76	987.75
☑	979	495.20	988.38
☑	981	495.66	989.30
☑	982	990.40	989.39
☑	983	990.75	989.74
☑	984	991.29	990.28
☑	985	991.40	990.39
☑	986	991.57	990.56
☑	987	993.42	992.41
☑	988	993.50	992.49
☑	989	331.93	992.77
☑	990	994.85	993.84

☑	991	995.16	994.15
☑	992	996.59	995.58
☑	993	997.49	996.48
☑	994	997.58	996.58
☑	995	998.47	997.47
☑	996	499.86	997.70
☑	997	998.77	997.76
☑	998	998.87	997.86
☑	999	500.26	998.51
☑	1000	1001.42	1000.42
☑	1001	1001.58	1000.57
☑	1002	1003.18	1002.17
☑	1003	502.19	1002.37
☑	1004	1004.19	1003.19
☑	1008	1005.77	1004.77
☑	1009	503.84	1005.67
☑	1010	1007.08	1006.07
☑	1011	1007.16	1006.15
☑	1013	1007.37	1006.36
☑	1014	1008.11	1007.10
☑	1015	1008.58	1007.57
☑	1016	1008.91	1007.90
☑	1017	1008.92	1007.91
☑	1018	505.24	1008.46
☑	1019	1010.07	1009.06
☑	1020	1010.18	1009.17
☑	1021	1010.34	1009.33
☑	1022	1010.37	1009.36
☑	1023	505.70	1009.40
☑	1024	1010.68	1009.67
☑	1025	1010.70	1009.70
☑	1026	1011.17	1010.16
☑	1027	506.21	1010.41
☑	1028	1012.11	1011.10

☑	1029	1012.80	1011.79
☑	1030	1013.39	1012.38
☑	1031	507.21	1012.41
☑	1032	1014.16	1013.15
☑	1033	1014.83	1013.83
☑	1034	1015.15	1014.14
☑	1035	1015.98	1014.98
☑	1036	1016.02	1015.01
☑	1038	1016.87	1015.86
☑	1039	1016.89	1015.88
☑	1040	1016.96	1015.95
☑	1041	1017.53	1016.53
☑	1042	1018.14	1017.13
☑	1043	1018.22	1017.21
☑	1044	1018.84	1017.83
☑	1045	1018.91	1017.90
☑	1046	510.23	1018.45
☑	1047	1020.53	1019.52
☑	1048	1021.41	1020.40
☑	1049	1021.83	1020.82
☑	1050	1022.30	1021.29
☑	1051	1023.21	1022.20
☑	1052	1023.64	1022.64
☑	1053	1024.06	1023.05
☑	1054	1024.20	1023.19
☑	1055	1024.33	1023.32
☑	1057	1025.64	1024.63
☑	1058	1025.72	1024.72
☑	1059	1026.19	1025.18
☑	1060	1027.55	1026.54
☑	1061	1028.24	1027.24
☑	1062	1028.33	1027.32
☑	1063	1028.75	1027.74
☑	1064	1028.93	1027.92

☑	1065	1029.12	1028.11
☑	1066	1029.25	1028.25
☑	1068	1030.29	1029.28
☑	1069	1030.91	1029.90
☑	1071	1031.84	1030.83
☑	1072	1032.14	1031.14
☑	1073	1032.51	1031.50
☑	1074	1032.52	1031.52
☑	1075	1033.07	1032.06
☑	1076	1033.81	1032.80
☑	1077	1033.89	1032.88
☑	1078	1034.81	1033.80
☑	1079	1035.03	1034.03
☑	1080	1035.11	1034.10
☑	1082	1035.64	1034.64
☑	1084	1036.88	1035.87
☑	1086	1037.59	1036.58
☑	1087	1037.70	1036.69
☑	1088	1037.83	1036.82
☑	1089	1038.18	1037.17
☑	1090	519.72	1037.42
☑	1091	1039.29	1038.28
☑	1093	1039.35	1038.34
☑	1094	1039.59	1038.58
☑	1095	520.30	1038.59
☑	1096	1039.90	1038.90
☑	1097	1040.28	1039.28
☑	1098	1041.20	1040.20
☑	1099	1041.49	1040.48
☑	1100	1043.35	1042.34
☑	1101	1043.97	1042.96
☑	1102	1044.06	1043.05
☑	1103	1045.10	1044.09
☑	1105	1045.55	1044.54

☑	1106	1045.68	1044.67
☑	1107	1045.71	1044.70
☑	1108	523.70	1045.38
☑	1109	523.77	1045.53
☑	1110	1046.73	1045.72
☑	1111	1046.80	1045.79
☑	1112	1047.76	1046.76
☑	1113	1048.48	1047.48
☑	1114	1048.71	1047.70
☑	1115	1049.56	1048.55
☑	1116	1049.64	1048.64
☑	1117	1050.06	1049.05
☑	1118	1050.07	1049.06
☑	1119	1050.10	1049.09
☑	1120	1050.74	1049.73
☑	1121	1050.83	1049.82
☑	1122	1051.70	1050.70
☑	1123	527.29	1052.57
☑	1124	1053.81	1052.80
☑	1125	1054.31	1053.30
☑	1126	1055.18	1054.17
☑	1127	1055.27	1054.26
☑	1128	1055.71	1054.70
☑	1129	1055.90	1054.89
☑	1130	1056.03	1055.02
☑	1131	1056.11	1055.10
☑	1132	1056.21	1055.20
☑	1133	528.78	1055.55
☑	1134	1057.88	1056.87
☑	1135	1057.88	1056.87
☑	1136	1058.10	1057.10
☑	1137	1058.20	1057.20
☑	1138	1058.23	1057.23
☑	1140	1058.97	1057.97

☑	1142	530.29	1058.57
☑	1143	1059.69	1058.68
☑	1144	1059.84	1058.83
☑	1145	1060.13	1059.12
☑	1146	1060.61	1059.60
☑	1147	1062.06	1061.05
☑	1148	1062.31	1061.30
☑	1149	1062.35	1061.35
☑	1150	1062.48	1061.47
☑	1151	532.22	1062.42
☑	1152	1064.49	1063.48
☑	1153	1064.87	1063.87
☑	1154	1064.89	1063.89
☑	1156	533.26	1064.50
☑	1157	533.40	1064.79
☑	1158	1066.20	1065.20
☑	1159	1066.27	1065.27
☑	1160	533.76	1065.50
☑	1161	1066.61	1065.60
☑	1162	1066.70	1065.70
☑	1163	1067.45	1066.45
☑	1165	1068.38	1067.37
☑	1166	1069.05	1068.04
☑	1167	1069.34	1068.33
☑	1168	1070.23	1069.23
☑	1170	1071.42	1070.41
☑	1171	1071.56	1070.55
☑	1172	1072.23	1071.22
☑	1173	1072.26	1071.25
☑	1174	1072.40	1071.39
☑	1175	1072.71	1071.70
☑	1176	1073.88	1072.87
☑	1177	1073.96	1072.95
☑	1178	537.76	1073.50

☑	1179	1074.92	1073.91
☑	1180	1075.05	1074.04
☑	1181	1075.15	1074.14
☑	1182	1076.24	1075.23
☑	1183	1076.68	1075.68
☑	1184	1076.78	1075.77
☑	1185	1076.79	1075.78
☑	1186	1078.81	1077.80
☑	1187	1078.83	1077.82
☑	1188	1078.94	1077.93
☑	1189	1079.23	1078.22
☑	1190	1079.40	1078.39
☑	1191	1081.10	1080.10
☑	1192	1081.23	1080.22
☑	1193	1081.86	1080.86
☑	1194	1083.08	1082.08
☑	1195	1084.03	1083.02
☑	1196	1084.29	1083.28
☑	1197	1084.62	1083.62
☑	1198	1084.81	1083.80
☑	1199	1086.14	1085.13
☑	1200	1087.33	1086.33
☑	1201	1087.44	1086.44
☑	1202	1087.91	1086.90
☑	1203	363.47	1087.39
☑	1204	1088.95	1087.94
☑	1205	1089.33	1088.32
☑	1206	1089.37	1088.36
☑	1208	1089.98	1088.97
☑	1211	1090.95	1089.94
☑	1212	1091.66	1090.65
☑	1213	1092.14	1091.13
☑	1215	1093.26	1092.25
☑	1216	1093.72	1092.72

☑	1217	1094.30	1093.29
☑	1218	547.67	1093.34
☑	1219	1095.07	1094.06
☑	1220	1095.57	1094.56
☑	1221	1096.10	1095.09
☑	1222	1096.55	1095.54
☑	1223	1097.14	1096.14
☑	1224	1097.79	1096.78
☑	1225	1098.34	1097.34
☑	1226	1098.73	1097.72
☑	1227	1099.82	1098.81
☑	1228	1099.90	1098.89
☑	1229	1100.17	1099.16
☑	1230	550.61	1099.20
☑	1231	1100.42	1099.41
☑	1232	1100.90	1099.90
☑	1233	1101.05	1100.04
☑	1234	1101.33	1100.32
☑	1235	1101.77	1100.76
☑	1236	1101.78	1100.77
☑	1237	1102.15	1101.14
☑	1238	551.70	1101.39
☑	1239	551.74	1101.46
☑	1240	1102.61	1101.60
☑	1241	1102.98	1101.97
☑	1242	1103.14	1102.14
☑	1243	1104.07	1103.06
☑	1245	1105.13	1104.12
☑	1246	1105.31	1104.30
☑	1247	1105.97	1104.97
☑	1248	1106.47	1105.46
☑	1250	1108.99	1107.98
☑	1252	1109.21	1108.20
☑	1253	555.16	1108.31

☑	1255	1109.57	1108.56
☑	1256	1110.11	1109.10
☑	1257	1110.18	1109.17
☑	1258	1111.10	1110.09
☑	1259	1112.25	1111.25
☑	1260	1112.56	1111.55
☑	1261	1113.60	1112.59
☑	1262	1114.01	1113.00
☑	1263	1114.13	1113.12
☑	1265	1114.62	1113.62
☑	1266	1114.78	1113.77
☑	1267	1114.94	1113.94
☑	1268	1115.17	1114.17
☑	1270	1118.35	1117.34
☑	1271	1118.93	1117.93
☑	1272	1119.55	1118.54
☑	1273	1119.96	1118.95
☑	1274	1121.06	1120.05
☑	1276	1122.01	1121.00
☑	1278	1122.70	1121.69
☑	1279	1123.10	1122.09
☑	1280	1123.14	1122.13
☑	1281	1123.24	1122.23
☑	1282	1123.62	1122.61
☑	1283	1123.72	1122.71
☑	1284	1124.52	1123.51
☑	1285	563.24	1124.47
☑	1286	1126.04	1125.03
☑	1288	1126.83	1125.82
☑	1289	1126.96	1125.95
☑	1291	1127.66	1126.65
☑	1292	1128.71	1127.70
☑	1293	1129.39	1128.38
☑	1294	1129.39	1128.39

☑	1295	1131.73	1130.72
☑	1296	566.42	1130.83
☑	1297	1131.98	1130.97
☑	1298	1132.26	1131.25
☑	1299	1132.52	1131.51
☑	1300	1132.69	1131.69
☑	1301	1133.93	1132.92
☑	1302	1133.93	1132.92
☑	1303	1133.99	1132.98
☑	1304	1134.21	1133.20
☑	1305	1134.89	1133.89
☑	1306	1135.08	1134.07
☑	1307	1135.90	1134.89
☑	1309	1136.52	1135.51
☑	1310	1136.82	1135.81
☑	1311	1137.78	1136.77
☑	1312	1138.18	1137.17
☑	1313	1138.33	1137.32
☑	1314	569.70	1137.38
☑	1315	1138.85	1137.84
☑	1317	1140.37	1139.36
☑	1318	1140.73	1139.73
☑	1320	1142.23	1141.23
☑	1321	1142.43	1141.42
☑	1322	1142.78	1141.77
☑	1323	1143.14	1142.14
☑	1324	1143.61	1142.60
☑	1325	1143.82	1142.81
☑	1326	1143.96	1142.95
☑	1327	1145.14	1144.13
☑	1328	1146.55	1145.54
☑	1329	574.25	1146.48
☑	1330	1147.93	1146.92
☑	1331	1148.55	1147.54

1332	1149.09	1148.08
1334	1150.14	1149.14
1335	1150.15	1149.14
1336	1150.16	1149.15
1337	1150.20	1149.19
1338	1150.43	1149.43
1339	576.18	1150.34
1340	576.33	1150.65
1341	1151.88	1150.87
1342	576.45	1150.88
1343	1151.91	1150.90
1345	1152.67	1151.66
1346	1152.77	1151.76
1347	576.94	1151.87
1348	1153.15	1152.14
1349	1153.89	1152.89
1350	1153.89	1152.89
1351	1154.30	1153.29
1352	1154.42	1153.41
1353	1154.49	1153.48
1354	1154.57	1153.56
1356	1156.93	1155.92
1357	1157.06	1156.05
1358	1157.47	1156.46
1359	1157.54	1156.53
1360	1158.55	1157.55
1362	1160.70	1159.69
1363	1161.36	1160.35
1364	1161.56	1160.56
1365	581.55	1161.09
1366	1162.13	1161.12
1367	1162.27	1161.26
1368	1162.27	1161.26
1369	1163.02	1162.01

☑	1370	1163.18	1162.17
☑	1371	582.24	1162.46
☑	1372	582.35	1162.69
☑	1373	1163.89	1162.88
☑	1374	1164.76	1163.76
☑	1375	1165.09	1164.08
☑	1376	583.09	1164.17
☑	1378	1165.47	1164.46
☑	1379	1165.51	1164.50
☑	1382	1167.75	1166.74
☑	1383	1168.05	1167.04
☑	1384	1168.05	1167.04
☑	1385	1169.35	1168.34
☑	1386	1169.45	1168.44
☑	1387	1169.96	1168.95
☑	1388	1170.56	1169.56
☑	1390	1170.64	1169.64
☑	1391	1171.86	1170.85
☑	1392	1172.41	1171.40
☑	1393	1172.54	1171.53
☑	1394	1174.49	1173.49
☑	1395	1174.63	1173.62
☑	1396	1175.14	1174.14
☑	1397	1175.32	1174.31
☑	1399	588.26	1174.51
☑	1400	1176.10	1175.09
☑	1403	590.37	1178.73
☑	1404	1180.73	1179.72
☑	1407	591.77	1181.52
☑	1408	1182.63	1181.62
☑	1409	1182.77	1181.76
☑	1410	1182.85	1181.84
☑	1412	592.94	1183.86
☑	1413	1184.92	1183.91

☑	1415	1186.92	1185.91
☑	1416	1187.42	1186.41
☑	1417	1187.47	1186.46
☑	1419	1188.11	1187.10
☑	1420	594.78	1187.55
☑	1422	1189.47	1188.46
☑	1423	1189.53	1188.52
☑	1424	1190.24	1189.23
☑	1425	1190.29	1189.28
☑	1426	596.26	1190.50
☑	1427	1193.39	1192.39
☑	1428	1194.23	1193.23
☑	1429	398.81	1193.41
☑	1430	1194.74	1193.73
☑	1431	1195.15	1194.14
☑	1433	1195.94	1194.94
☑	1434	1197.52	1196.51
☑	1435	1198.35	1197.34
☑	1436	1200.53	1199.53
☑	1437	1200.98	1199.97
☑	1438	1201.10	1200.10
☑	1439	601.36	1200.71
☑	1440	1202.13	1201.12
☑	1442	1202.66	1201.65
☑	1443	1203.39	1202.38
☑	1445	1203.58	1202.58
☑	1447	1204.90	1203.90
☑	1448	1206.47	1205.47
☑	1449	1207.87	1206.86
☑	1450	1209.45	1208.45
☑	1451	1209.69	1208.68
☑	1452	1209.87	1208.87
☑	1453	605.68	1209.34
☑	1454	1210.58	1209.57

☑	1455	1210.66	1209.65
☑	1456	1211.15	1210.14
☑	1457	1212.58	1211.57
☑	1458	1212.79	1211.78
☑	1459	607.14	1212.28
☑	1460	607.47	1212.93
☑	1461	1215.05	1214.04
☑	1462	1215.20	1214.19
☑	1463	1215.43	1214.42
☑	1464	1215.45	1214.44
☑	1465	608.23	1214.45
☑	1466	1217.15	1216.14
☑	1468	1217.65	1216.64
☑	1469	1217.66	1216.66
☑	1470	1218.27	1217.26
☑	1471	609.75	1217.48
☑	1472	1219.17	1218.16
☑	1473	1220.54	1219.53
☑	1474	1221.31	1220.30
☑	1475	611.34	1220.66
☑	1476	1221.77	1220.76
☑	1477	611.58	1221.14
☑	1478	1222.84	1221.83
☑	1479	1223.36	1222.35
☑	1480	612.24	1222.46
☑	1482	1226.34	1225.33
☑	1483	1228.02	1227.01
☑	1484	1228.04	1227.03
☑	1485	1228.43	1227.42
☑	1486	1228.47	1227.46
☑	1487	1228.47	1227.46
☑	1489	1228.96	1227.95
☑	1490	1229.84	1228.84
☑	1491	615.79	1229.56

☑	1492	1231.13	1230.12
☑	1493	1231.56	1230.55
☑	1494	1232.32	1231.32
☑	1496	1234.07	1233.06
☑	1498	1234.93	1233.92
☑	1499	1235.73	1234.72
☑	1500	618.60	1235.18
☑	1501	1236.40	1235.39
☑	1502	1236.42	1235.41
☑	1503	1236.93	1235.92
☑	1504	1237.65	1236.64
☑	1505	619.79	1237.56
☑	1506	1239.14	1238.13
☑	1507	1239.39	1238.38
☑	1509	620.29	1238.57
☑	1510	620.53	1239.05
☑	1511	621.20	1240.38
☑	1512	621.22	1240.42
☑	1513	1242.75	1241.74
☑	1514	1243.83	1242.83
☑	1515	623.26	1244.52
☑	1516	1246.78	1245.77
☑	1517	1247.08	1246.08
☑	1518	1247.11	1246.10
☑	1519	1247.61	1246.60
☑	1520	626.79	1251.56
☑	1521	1253.35	1252.34
☑	1523	1253.89	1252.88
☑	1524	627.95	1253.89
☑	1525	628.49	1254.97
☑	1526	1256.18	1255.18
☑	1527	1257.01	1256.00
☑	1529	1257.72	1256.71
☑	1530	629.51	1257.01

1531	*629.83*	*1257.65*
1532	*1259.23*	*1258.22*
1533	*1260.04*	*1259.03*
1536	*1260.62*	*1259.61*
1537	*1260.95*	*1259.94*
1538	*1262.23*	*1261.22*
1539	*632.86*	*1263.70*
1541	*1266.06*	*1265.06*
1542	*1267.38*	*1266.37*
1543	*1267.75*	*1266.74*
1544	*1269.43*	*1268.42*
1545	*636.22*	*1270.43*
1546	*1272.15*	*1271.14*
1547	*636.62*	*1271.22*
1548	*636.83*	*1271.64*
1549	*637.89*	*1273.76*
1550	*1275.92*	*1274.91*
1551	*638.66*	*1275.31*
1552	*638.72*	*1275.42*
1553	*638.85*	*1275.68*
1556	*1281.12*	*1280.11*
1557	*642.75*	*1283.50*
1558	*1284.60*	*1283.59*
1559	*1285.21*	*1284.20*
1560	*643.18*	*1284.34*
1561	*643.22*	*1284.43*
1562	*643.32*	*1284.62*
1563	*643.50*	*1284.98*
1564	*1286.35*	*1285.34*
1565	*1286.52*	*1285.51*
1567	*1287.34*	*1286.33*
1568	*644.35*	*1286.68*
1569	*645.40*	*1288.78*
1570	*645.77*	*1289.53*

☑	1571	1290.65	1289.64
☑	1572	1290.96	1289.95
☑	1574	646.33	1290.65
☑	1575	646.58	1291.15
☑	1576	1292.66	1291.65
☑	1578	432.20	1293.59
☑	1579	1295.15	1294.15
☑	1580	648.15	1294.29
☑	1581	1295.30	1294.30
☑	1582	1295.46	1294.45
☑	1584	1297.83	1296.82
☑	1585	1298.06	1297.05
☑	1587	1299.51	1298.51
☑	1588	650.27	1298.53
☑	1590	1300.54	1299.53
☑	1593	651.57	1301.12
☑	1594	1302.47	1301.46
☑	1595	1302.66	1301.65
☑	1597	1304.09	1303.08
☑	1598	1305.86	1304.85
☑	1599	1306.96	1305.95
☑	1601	1308.86	1307.85
☑	1602	655.25	1308.48
☑	1604	655.39	1308.77
☑	1605	437.51	1309.52
☑	1606	1313.11	1312.10
☑	1607	1314.12	1313.12
☑	1608	658.22	1314.42
☑	1609	658.70	1315.38
☑	1610	659.24	1316.46
☑	1613	661.06	1320.11
☑	1614	661.53	1321.05
☑	1615	1325.06	1324.05
☑	1616	1325.44	1324.44

☑	1617	663.27	1324.54
☑	1618	664.05	1326.09
☑	1619	1329.48	1328.47
☑	1624	1332.64	1331.63
☑	1626	1336.95	1335.95
☑	1627	1337.72	1336.71
☑	1628	1338.20	1337.19
☑	1630	1341.15	1340.14
☑	1632	671.91	1341.81
☑	1633	1343.00	1341.99
☑	1634	1345.89	1344.88
☑	1635	673.67	1345.33
☑	1636	1347.62	1346.61
☑	1638	675.88	1349.74
☑	1640	679.76	1357.51
☑	1641	681.07	1360.12
☑	1642	682.87	1363.72
☑	1643	683.26	1364.51
☑	1646	684.26	1366.51
☑	1647	1369.57	1368.56
☑	1650	686.79	1371.56
☑	1652	1374.05	1373.04
☑	1653	1374.09	1373.08
☑	1654	687.74	1373.46
☑	1656	689.27	1376.53
☑	1657	689.62	1377.23
☑	1658	1379.38	1378.37
☑	1662	692.20	1382.38
☑	1664	461.87	1382.59
☑	1667	692.78	1383.54
☑	1669	692.84	1383.66
☑	1670	1385.60	1384.60
☑	1671	463.25	1386.72
☑	1673	695.35	1388.68

1676	696.38	1390.75
1677	1392.47	1391.46
1678	699.85	1397.68
1680	702.84	1403.67
1681	702.96	1403.91
1683	704.72	1407.42
1685	705.80	1409.59
1688	708.82	1415.63
1689	472.93	1415.78
1690	710.23	1418.45
1692	474.19	1419.56
1693	710.85	1419.68
1694	713.34	1424.66
1695	713.89	1425.76
1697	715.22	1428.43
1698	715.94	1429.87
1699	716.02	1430.03
1700	716.41	1430.80
1702	717.84	1433.67
1703	719.13	1436.24
1705	719.27	1436.53
1706	719.59	1437.16
1707	720.89	1439.76
1709	721.62	1441.23
1710	481.56	1441.66
1712	722.33	1442.65
1713	724.30	1446.58
1715	725.34	1448.66
1717	727.27	1452.52
1718	727.30	1452.59
1719	727.32	1452.63
1722	733.12	1464.22
1723	733.76	1465.51
1724	734.29	1466.57

☑	1725	489.87	1466.57
☑	1726	734.32	1466.63
☑	1727	489.92	1466.74
☑	1730	490.50	1468.47
☑	1734	736.77	1471.53
☑	1735	736.81	1471.60
☑	1737	737.92	1473.82
☑	1738	737.94	1473.86
☑	1740	738.47	1474.92
☑	1741	1476.64	1475.63
☑	1742	740.34	1478.66
☑	1743	740.83	1479.65
☑	1745	741.80	1481.60
☑	1747	743.37	1484.73
☑	1753	497.19	1488.55
☑	1754	745.86	1489.71
☑	1755	497.88	1490.62
☑	1756	747.02	1492.03
☑	1758	498.55	1492.63
☑	1759	498.55	1492.63
☑	1760	748.76	1495.50
☑	1761	749.90	1497.80
☑	1764	502.49	1504.44
☑	1765	502.51	1504.51
☑	1767	754.29	1506.56
☑	1770	755.80	1509.60
☑	1771	755.83	1509.64
☑	1772	756.29	1510.57
☑	1773	756.97	1511.93
☑	1775	760.44	1518.86
☑	1776	761.71	1521.40
☑	1777	761.80	1521.58
☑	1778	762.22	1522.43
☑	1780	764.79	1527.57

☑	1781	765.25	1528.48
☑	1782	765.92	1529.82
☑	1783	766.17	1530.32
☑	1784	769.74	1537.47
☑	1785	770.36	1538.71
☑	1786	770.40	1538.79
☑	1787	514.00	1538.97
☑	1788	514.62	1540.84
☑	1793	778.68	1555.34
☑	1794	781.25	1560.48
☑	1797	782.81	1563.61
☑	1799	522.60	1564.78
☑	1801	783.82	1565.63
☑	1802	783.89	1565.76
☑	1803	522.95	1565.83
☑	1804	786.47	1570.93
☑	1805	787.20	1572.39
☑	1806	787.30	1572.59
☑	1808	788.66	1575.32
☑	1809	788.81	1575.61
☑	1810	788.99	1575.96
☑	1811	528.09	1581.23
☑	1812	792.48	1582.95
☑	1813	792.90	1583.78
☑	1814	793.54	1585.06
☑	1816	529.90	1586.68
☑	1817	531.23	1590.66
☑	1819	798.72	1595.43
☑	1820	798.91	1595.80
☑	1822	802.44	1602.86
☑	1823	802.61	1603.20
☑	1824	804.73	1607.45
☑	1825	536.84	1607.50
☑	1826	804.83	1607.64

☑	1827	537.25	1608.72
☑	1828	537.55	1609.63
☑	1829	806.16	1610.30
☑	1831	807.43	1612.85
☑	1832	807.80	1613.58
☑	1833	808.90	1615.79
☑	1834	811.22	1620.42
☑	1836	541.61	1621.81
☑	1837	812.33	1622.64
☑	1839	813.29	1624.56
☑	1840	813.35	1624.68
☑	1841	814.49	1626.96
☑	1843	543.89	1628.64
☑	1844	818.36	1634.71
☑	1845	818.83	1635.65
☑	1846	546.30	1635.88
☑	1847	819.30	1636.58
☑	1848	821.28	1640.55
☑	1849	822.82	1643.62
☑	1850	824.30	1646.58
☑	1851	824.31	1646.60
☑	1853	825.37	1648.72
☑	1854	825.89	1649.77
☑	1855	826.47	1650.92
☑	1856	827.07	1652.13
☑	1857	827.79	1653.57
☑	1858	830.85	1659.68
☑	1859	830.98	1659.95
☑	1860	831.87	1661.72
☑	1861	556.20	1665.59
☑	1862	833.83	1665.65
☑	1863	834.31	1666.61
☑	1864	835.42	1668.82
☑	1865	836.88	1671.75

☑	1867	838.66	1675.31
☑	1868	839.78	1677.55
☑	1869	840.17	1678.32
☑	1870	840.22	1678.42
☑	1871	842.03	1682.04
☑	1872	842.35	1682.68
☑	1873	842.43	1682.84
☑	1874	842.62	1683.22
☑	1876	562.67	1685.00
☑	1877	843.88	1685.74
☑	1878	846.30	1690.59
☑	1879	564.64	1690.89
☑	1880	564.85	1691.52
☑	1882	846.90	1691.78
☑	1883	847.27	1692.52
☑	1884	847.91	1693.81
☑	1886	566.50	1696.49
☑	1887	568.22	1701.64
☑	1888	568.48	1702.40
☑	1889	568.49	1702.44
☑	1890	853.02	1704.04
☑	1891	853.12	1704.22
☑	1892	569.19	1704.54
☑	1893	853.35	1704.68
☑	1894	854.29	1706.56
☑	1895	855.05	1708.09
☑	1896	856.45	1710.88
☑	1897	856.88	1711.74
☑	1898	571.61	1711.81
☑	1899	856.92	1711.82
☑	1900	857.03	1712.05
☑	1901	857.54	1713.07
☑	1902	858.65	1715.28
☑	1903	858.84	1715.67

☑	1905	862.24	1722.46
☑	1906	576.27	1725.78
☑	1907	867.51	1733.00
☑	1909	870.39	1738.76
☑	1910	871.67	1741.33
☑	1911	871.85	1741.68
☑	1912	872.36	1742.70
☑	1913	873.80	1745.59
☑	1915	583.96	1748.85
☑	1916	877.31	1752.61
☑	1918	877.61	1753.21
☑	1919	585.96	1754.85
☑	1921	586.32	1755.94
☑	1922	880.37	1758.73
☑	1923	881.55	1761.09
☑	1924	882.38	1762.74
☑	1925	588.60	1762.77
☑	1926	882.71	1763.40
☑	1927	883.56	1765.10
☑	1929	589.60	1765.77
☑	1930	590.28	1767.81
☑	1931	590.53	1768.58
☑	1932	885.90	1769.79
☑	1933	885.94	1769.87
☑	1934	886.27	1770.53
☑	1935	887.37	1772.73
☑	1936	888.76	1775.51
☑	1937	890.48	1778.95
☑	1938	891.18	1780.36
☑	1940	897.83	1793.65
☑	1941	599.93	1796.78
☑	1942	899.74	1797.46
☑	1943	899.84	1797.66
☑	1944	600.23	1797.66

☑	1945	600.25	1797.74
☑	1946	900.53	1799.04
☑	1948	600.97	1799.87
☑	1949	602.72	1805.15
☑	1950	904.89	1807.77
☑	1951	905.35	1808.69
☑	1952	906.20	1810.39
☑	1953	906.51	1811.00
☑	1954	907.35	1812.69
☑	1955	606.59	1816.75
☑	1959	913.21	1824.41
☑	1960	913.43	1824.84
☑	1962	611.92	1832.74
☑	1963	611.95	1832.81
☑	1964	612.24	1833.69
☑	1965	917.88	1833.75
☑	1967	917.93	1833.84
☑	1968	918.04	1834.07
☑	1969	918.83	1835.64
☑	1971	918.88	1835.74
☑	1973	613.62	1837.84
☑	1974	920.44	1838.86
☑	1975	614.60	1840.76
☑	1980	925.36	1848.70
☑	1981	618.13	1851.38
☑	1982	618.68	1853.02
☑	1983	928.42	1854.82
☑	1984	619.99	1856.95
☑	1985	620.81	1859.41
☑	1986	931.14	1860.27
☑	1987	932.24	1862.46
☑	1988	622.64	1864.91
☑	1989	625.96	1874.87
☑	1990	626.40	1876.17

☑	1991	939.15	1876.29
☑	1992	939.21	1876.41
☑	1993	939.71	1877.41
☑	1994	940.25	1878.48
☑	1995	627.91	1880.71
☑	1996	941.60	1881.19
☑	1997	628.89	1883.66
☑	1998	943.62	1885.22
☑	1999	629.92	1886.73
☑	2000	944.79	1887.56
☑	2001	944.94	1887.87
☑	2002	945.05	1888.09
☑	2003	945.46	1888.91
☑	2004	948.11	1894.20
☑	2005	949.18	1896.34
☑	2006	949.18	1896.35
☑	2007	950.22	1898.42
☑	2008	951.96	1901.91
☑	2009	635.12	1902.33
☑	2010	952.95	1903.89
☑	2011	636.10	1905.28
☑	2012	636.27	1905.78
☑	2013	953.99	1905.96
☑	2014	636.37	1906.09
☑	2015	954.41	1906.80
☑	2016	636.94	1907.80
☑	2017	637.24	1908.69
☑	2018	637.26	1908.76
☑	2019	956.83	1911.64
☑	2021	958.50	1914.99
☑	2022	960.13	1918.24
☑	2023	960.18	1918.35
☑	2025	960.62	1919.22
☑	2026	960.84	1919.66

☑	2027	641.02	1920.03
☑	2028	961.85	1921.68
☑	2029	962.60	1923.18
☑	2030	642.60	1924.78
☑	2031	963.83	1925.65
☑	2032	964.32	1926.63
☑	2033	645.03	1932.06
☑	2034	967.70	1933.39
☑	2035	968.44	1934.87
☑	2036	970.58	1939.15
☑	2037	648.59	1942.74
☑	2038	973.52	1945.03
☑	2039	650.94	1949.80
☑	2040	976.34	1950.67
☑	2041	979.23	1956.45
☑	2042	654.26	1959.77
☑	2043	981.02	1960.03
☑	2044	984.34	1966.66
☑	2045	984.85	1967.68
☑	2046	987.97	1973.92
☑	2047	659.24	1974.68
☑	2048	990.28	1978.54
☑	2049	661.36	1981.06
☑	2050	991.82	1981.63
☑	2051	995.46	1988.91
☑	2052	996.41	1990.81
☑	2053	997.20	1992.39
☑	2054	665.31	1992.90
☑	2055	998.96	1995.91
☑	2056	666.64	1996.90
☑	2057	1000.84	1999.67
☑	2058	667.71	2000.11
☑	2059	1001.74	2001.46
☑	2060	1003.03	2004.05

☑	2061	1003.22	2004.42
☑	2062	1003.24	2004.46
☑	2063	1004.63	2007.25
☑	2064	1005.65	2009.28
☑	2065	1006.89	2011.76
☑	2066	1007.39	2012.76
☑	2067	1007.41	2012.80
☑	2068	672.21	2013.61
☑	2069	1007.97	2013.93
☑	2070	1008.01	2014.01
☑	2071	672.42	2014.23
☑	2072	1008.46	2014.90
☑	2074	675.25	2022.73
☑	2075	1012.44	2022.87
☑	2076	675.36	2023.05
☑	2078	1014.28	2026.55
☑	2079	1015.58	2029.15
☑	2080	1015.66	2029.31
☑	2081	677.60	2029.77
☑	2082	1016.09	2030.16
☑	2083	679.27	2034.80
☑	2084	1020.92	2039.83
☑	2085	1021.18	2040.34
☑	2086	1022.32	2042.62
☑	2087	682.33	2043.96
☑	2088	1023.89	2045.77
☑	2089	683.29	2046.84
☑	2090	683.30	2046.87
☑	2091	1024.58	2047.14
☑	2092	683.39	2047.16
☑	2093	1025.65	2049.29
☑	2094	1026.17	2050.32
☑	2095	1026.90	2051.79
☑	2096	684.95	2051.83

☑	2097	1027.92	2053.84
☑	2098	1028.56	2055.11
☑	2099	1029.02	2056.03
☑	2100	686.54	2056.60
☑	2102	687.40	2059.17
☑	2103	689.54	2065.60
☑	2104	1034.79	2067.57
☑	2106	1036.42	2070.82
☑	2107	1036.51	2071.01
☑	2108	1036.90	2071.79
☑	2109	691.64	2071.91
☑	2110	691.99	2072.94
☑	2111	1038.96	2075.90
☑	2112	1039.92	2077.83
☑	2113	1040.99	2079.96
☑	2114	694.56	2080.66
☑	2115	1042.69	2083.36
☑	2116	1042.71	2083.40
☑	2117	1043.15	2084.30
☑	2118	697.35	2089.03
☑	2119	1045.57	2089.13
☑	2120	697.42	2089.23
☑	2121	1047.11	2092.20
☑	2122	1047.84	2093.67
☑	2123	1049.63	2097.26
☑	2125	1049.94	2097.86
☑	2126	1050.11	2098.20
☑	2127	700.62	2098.83
☑	2128	1050.54	2099.06
☑	2129	1050.88	2099.75
☑	2130	1052.31	2102.61
☑	2131	1052.34	2102.66
☑	2132	1052.95	2103.89
☑	2133	1053.15	2104.29

☑	2134	702.53	2104.57
☑	2135	1053.39	2104.77
☑	2136	1054.37	2106.72
☑	2137	703.25	2106.73
☑	2138	1054.40	2106.79
☑	2139	1054.85	2107.68
☑	2140	1056.83	2111.64
☑	2141	1056.92	2111.83
☑	2142	704.96	2111.87
☑	2143	1057.65	2113.29
☑	2144	1058.56	2115.10
☑	2145	1059.17	2116.34
☑	2146	1063.16	2124.31
☑	2147	709.14	2124.40
☑	2148	1063.64	2125.26
☑	2149	1064.00	2125.99
☑	2150	1064.63	2127.24
☑	2151	710.43	2128.27
☑	2152	1065.43	2128.86
☑	2153	710.70	2129.07
☑	2154	1069.40	2136.79
☑	2155	1069.88	2137.74
☑	2156	714.41	2140.21
☑	2157	714.74	2141.20
☑	2158	714.77	2141.28
☑	2159	1072.96	2143.90
☑	2160	715.98	2144.93
☑	2161	1075.69	2149.36
☑	2162	1075.83	2149.64
☑	2163	717.91	2150.71
☑	2164	717.99	2150.95
☑	2165	1077.98	2153.94
☑	2166	1078.99	2155.97
☑	2167	722.05	2163.12

☑	2168	1082.59	2163.16
☑	2169	722.32	2163.92
☑	2170	722.49	2164.46
☑	2171	1083.45	2164.89
☑	2172	1083.49	2164.96
☑	2173	1084.55	2167.09
☑	2174	723.73	2168.16
☑	2175	724.43	2170.26
☑	2176	724.70	2171.09
☑	2177	724.90	2171.67
☑	2178	1086.89	2171.77
☑	2179	1087.19	2172.37
☑	2180	725.31	2172.90
☑	2181	725.90	2174.68
☑	2182	725.91	2174.72
☑	2183	1089.13	2176.25
☑	2184	1089.14	2176.28
☑	2185	1089.24	2176.46
☑	2186	1089.26	2176.50
☑	2187	1090.46	2178.91
☑	2188	727.31	2178.92
☑	2190	1093.53	2185.04
☑	2191	729.43	2185.26
☑	2192	1095.85	2189.68
☑	2193	731.80	2192.38
☑	2195	1097.89	2193.77
☑	2196	1098.33	2194.64
☑	2197	1099.90	2197.78
☑	2198	1103.03	2204.04
☑	2199	1103.13	2204.24
☑	2200	1105.52	2209.02
☑	2201	1105.83	2209.65
☑	2202	1105.98	2209.94
☑	2204	738.98	2213.92

☑	2205	1108.33	2214.64
☑	2206	1109.71	2217.40
☑	2207	1111.05	2220.09
☑	2208	742.53	2224.57
☑	2209	742.95	2225.83
☑	2210	1115.22	2228.43
☑	2211	1115.51	2229.00
☑	2212	1116.93	2231.85
☑	2213	1117.54	2233.07
☑	2214	1117.58	2233.14
☑	2215	746.65	2236.93
☑	2216	1123.84	2245.67
☑	2217	1126.00	2249.99
☑	2218	751.01	2250.00
☑	2219	1126.22	2250.43
☑	2221	1127.28	2252.55
☑	2222	751.97	2252.90
☑	2223	752.17	2253.49
☑	2224	752.38	2254.12
☑	2225	1128.67	2255.32
☑	2226	753.18	2256.51
☑	2227	1129.28	2256.55
☑	2228	755.16	2262.45
☑	2229	1135.87	2269.73
☑	2230	1137.86	2273.70
☑	2231	760.69	2279.05
☑	2232	761.50	2281.47
☑	2234	761.74	2282.19
☑	2235	761.98	2282.91
☑	2236	762.06	2283.16
☑	2237	1144.82	2287.62
☑	2238	765.92	2294.75
☑	2239	765.95	2294.83
☑	2240	1148.96	2295.91

☑	2241	766.31	2295.92
☑	2242	766.40	2296.17
☑	2243	766.43	2296.27
☑	2244	1151.12	2300.23
☑	2245	768.72	2303.15
☑	2246	1154.25	2306.48
☑	2247	770.38	2308.10
☑	2248	770.70	2309.09
☑	2249	1155.61	2309.21
☑	2250	1156.97	2311.92
☑	2251	1157.22	2312.43
☑	2252	1157.44	2312.87
☑	2253	772.17	2313.50
☑	2255	1159.40	2316.78
☑	2256	1160.61	2319.21
☑	2257	1162.08	2322.15
☑	2258	775.23	2322.67
☑	2259	775.27	2322.78
☑	2260	1165.93	2329.84
☑	2261	1168.37	2334.73
☑	2262	780.00	2336.98
☑	2263	1169.50	2336.99
☑	2264	1171.13	2340.24
☑	2265	1172.79	2343.56
☑	2266	782.98	2345.91
☑	2267	1174.36	2346.71
☑	2268	783.25	2346.73
☑	2269	784.29	2349.86
☑	2270	1177.84	2353.67
☑	2271	785.64	2353.90
☑	2272	1178.00	2353.99
☑	2273	786.49	2356.45
☑	2274	788.76	2363.27
☑	2275	789.11	2364.30

☑	2276	1185.62	2369.23
☑	2277	1185.64	2369.26
☑	2278	1188.06	2374.11
☑	2279	792.62	2374.85
☑	2280	792.97	2375.89
☑	2281	793.00	2375.99
☑	2282	793.26	2376.75
☑	2283	793.75	2378.22
☑	2284	1190.40	2378.79
☑	2285	1191.64	2381.26
☑	2286	1192.39	2382.78
☑	2287	795.27	2382.78
☑	2288	1193.41	2384.80
☑	2289	797.43	2389.27
☑	2290	1197.55	2393.09
☑	2291	799.24	2394.69
☑	2292	802.05	2403.14
☑	2294	1207.05	2412.09
☑	2295	1207.37	2412.73
☑	2296	805.93	2414.76
☑	2297	1209.02	2416.02
☑	2298	807.69	2420.05
☑	2299	807.88	2420.62
☑	2300	809.36	2425.07
☑	2301	810.75	2429.22
☑	2302	813.32	2436.94
☑	2304	820.68	2459.02
☑	2305	821.93	2462.77
☑	2306	1232.67	2463.34
☑	2307	822.90	2465.69
☑	2308	1235.81	2469.61
☑	2309	824.23	2469.66
☑	2310	825.78	2474.33
☑	2311	1241.75	2481.48

☑	2312	1243.23	2484.45
☑	2313	829.39	2485.15
☑	2314	830.08	2487.22
☑	2315	830.40	2488.18
☑	2316	831.02	2490.04
☑	2317	832.87	2495.59
☑	2318	833.91	2498.70
☑	2319	834.82	2501.42
☑	2320	834.86	2501.56
☑	2321	835.43	2503.26
☑	2322	836.35	2506.02
☑	2323	836.73	2507.15
☑	2324	837.31	2508.90
☑	2325	1256.76	2511.50
☑	2326	840.12	2517.34
☑	2327	841.98	2522.93
☑	2328	842.36	2524.07
☑	2329	842.45	2524.32
☑	2330	844.70	2531.07
☑	2331	845.46	2533.36
☑	2332	1267.84	2533.67
☑	2334	848.79	2543.35
☑	2337	853.09	2556.26
☑	2338	854.36	2560.07
☑	2339	858.51	2572.51
☑	2340	862.49	2584.43
☑	2341	862.80	2585.38
☑	2342	863.51	2587.51
☑	2344	866.34	2595.99
☑	2345	868.92	2603.73
☑	2346	873.39	2617.15
☑	2347	1311.13	2620.24
☑	2348	875.52	2623.54
☑	2349	876.09	2625.26

☑	2350	877.72	2630.15
☑	2351	879.59	2635.74
☑	2352	880.04	2637.09
☑	2353	882.05	2643.13
☑	2354	1322.73	2643.46
☑	2355	882.31	2643.90
☑	2356	883.53	2647.58
☑	2357	886.45	2656.32
☑	2358	887.99	2660.93
☑	2359	893.48	2677.41
☑	2360	894.53	2680.57
☑	2361	897.19	2688.54
☑	2362	897.75	2690.22
☑	2363	899.99	2696.95
☑	2364	900.42	2698.24
☑	2365	902.41	2704.21
☑	2366	903.36	2707.06
☑	2367	904.55	2710.63
☑	2368	904.77	2711.29
☑	2370	906.29	2715.84
☑	2371	906.34	2716.01
☑	2372	909.19	2724.54
☑	2373	909.86	2726.55
☑	2374	913.69	2738.04
☑	2375	914.24	2739.68
☑	2376	915.54	2743.61
☑	2377	916.75	2747.23
☑	2378	919.28	2754.83
☑	2379	922.63	2764.88
☑	2380	924.00	2768.97
☑	2381	924.54	2770.61
☑	2382	925.01	2772.02
☑	2383	928.67	2783.00
☑	2384	936.53	2806.57

☑	2385	938.09	2811.24
☑	2386	939.35	2815.01
☑	2387	940.97	2819.89
☑	2388	945.97	2834.87
☑	2389	947.28	2838.81
☑	2390	948.60	2842.76
☑	2391	950.25	2847.72
☑	2392	950.86	2849.57
☑	2393	960.69	2879.06
☑	2394	960.77	2879.29
☑	2395	963.67	2887.98
☑	2396	965.56	2893.66
☑	2397	968.21	2901.60
☑	2398	973.24	2916.68
☑	2399	974.30	2919.89
☑	2400	981.06	2940.16
☑	2401	984.54	2950.60
☑	2402	984.91	2951.70
☑	2403	985.38	2953.13
☑	2404	987.64	2959.91
☑	2405	988.24	2961.69
☑	2406	990.40	2968.19
☑	2407	991.13	2970.37
☑	2408	991.78	2972.31
☑	2409	993.64	2977.90
☑	2410	1004.42	3010.25
☑	2411	1005.02	3012.03
☑	2412	1005.40	3013.19
☑	2413	1008.35	3022.02
☑	2414	1008.38	3022.12
☑	2415	1010.57	3028.68
☑	2416	1011.86	3032.55
☑	2417	1012.45	3034.31
☑	2418	1012.67	3035.00

☑	2419	1012.92	3035.74
☑	2420	1015.59	3043.75
☑	2421	1016.85	3047.54
☑	2422	1023.10	3066.27
☑	2423	1023.76	3068.26
☑	2424	1025.18	3072.53
☑	2425	1026.28	3075.83
☑	2426	1026.74	3077.20
☑	2427	1029.55	3085.63
☑	2428	1030.55	3088.62
☑	2429	1031.05	3090.13
☑	2431	1038.96	3113.86
☑	2432	1040.96	3119.86
☑	2433	1043.14	3126.39
☑	2434	1043.42	3127.25
☑	2435	1043.70	3128.07
☑	2436	1046.79	3137.36
☑	2437	1047.46	3139.35
☑	2438	1047.60	3139.78
☑	2439	1048.94	3143.79
☑	2440	1052.27	3153.79
☑	2441	1053.63	3157.88
☑	2442	1057.16	3168.46
☑	2443	1061.11	3180.31
☑	2444	1061.84	3182.50
☑	2445	1062.61	3184.82
☑	2446	1063.77	3188.29
☑	2447	1065.92	3194.75
☑	2448	1065.96	3194.87
☑	2449	1071.21	3210.62
☑	2450	1072.35	3214.04
☑	2451	1076.35	3226.04
☑	2452	1077.05	3228.13
☑	2453	1078.21	3231.61

☑	2454	1081.99	3242.95
☑	2455	1082.67	3245.00
☑	2456	1087.15	3258.44
☑	2457	1090.06	3267.16
☑	2458	1091.31	3270.89
☑	2459	1091.58	3271.72
☑	2460	1095.75	3284.22
☑	2461	1096.38	3286.11
☑	2462	1098.51	3292.52
☑	2463	1099.17	3294.49
☑	2464	1105.07	3312.19
☑	2465	1106.67	3316.99
☑	2466	1107.50	3319.49
☑	2467	1107.62	3319.85
☑	2468	1108.06	3321.14
☑	2469	1115.80	3344.36
☑	2470	1116.38	3346.12
☑	2471	1117.10	3348.29
☑	2472	1124.53	3370.57
☑	2473	1128.36	3382.05
☑	2474	1128.42	3382.25
☑	2475	1129.37	3385.09
☑	2476	1132.46	3394.36
☑	2477	1135.86	3404.56
☑	2478	1139.93	3416.76
☑	2479	1145.89	3434.65
☑	2480	1147.17	3438.48
☑	2481	1149.13	3444.38
☑	2482	1157.36	3469.07
☑	2483	1159.40	3475.18
☑	2484	1162.89	3485.65
☑	2485	1163.27	3486.79
☑	2486	1164.23	3489.66
☑	2487	1168.26	3501.75

☑	2489	*1183.67*	*3547.99*
☑	2490	*1193.76*	*3578.26*
☑	2491	*1203.34*	*3606.99*
☑	2492	*1209.51*	*3625.49*
☑	2493	*1211.92*	*3632.75*
☑	2494	*1219.28*	*3654.81*
☑	2495	*1221.25*	*3660.73*
☑	2496	*1237.57*	*3709.68*
☑	2497	*1256.61*	*3766.81*
☑	2498	*1267.30*	*3798.89*
☑	2499	*1282.47*	*3844.39*

Search Parameters

```
Type of search         : MS/MS Ion Search
Enzyme                 : Trypsin
Variable modifications : Carbamidomethyl (C),Oxidation (M)
Mass values            : Monoisotopic
Protein Mass           : Unrestricted
Peptide Mass Tolerance : ± 0.6 Da
Fragment Mass Tolerance: ± 0.3 Da
Max Missed Cleavages   : 2
Instrument type        : ESI-TRAP
Number of queries      : 2499
```

Mascot: http://www.matrixscience.com/
