# Supplementary material for: Macrophage-infectivity potentiator of Trypanosoma cruzi (TcMIP) is a new pro-type 1 immuno-stimulating protein for neonatal human cells and vaccines in mice
Source: Front Immunol. 2023 Mar 23;14:1138526. doi: 10.3389/fimmu.2023.1138526 (PMC10077492; doi:10.3389/fimmu.2023.1138526)
Supplement: Supplementary file 11 [file DataSheet_9.pdf]

Detailed Protein Report

Project Info

**Name:**	Adjuvac	**Date:**	July 7, 2009

Sample Info & Protocols

**Name:**	OGE 54-56 -supedex	**Date:**	January 18, 2010

Search Result Info

Search Result	Location	Search Engine	Database	Ident. Compounds
Demo-090617-other_eucaryot-Sprot-trp_PE_2010-01-18 15:31:12	/Adjuvac/OGE 54-56 -supedex/100115-OGE54-56-f14-500mM_GE4_01_12289.d	Mascot, 2.2.04	Sprot, Sprot_57.7.fasta	1/4434
Demo-090617-other_eucaryot-Sprot-trp_PE_2010-01-18 15:31:12	/Adjuvac/OGE 54-56 -supedex/100115-OGE54-56-f14-75mM_GE2_01_12287.d	Mascot, 2.2.04	Sprot, Sprot_57.7.fasta	6/3719
Demo-090617-other_eucaryot-Sprot-trp_PE_2010-01-18 15:31:12	/Adjuvac/OGE 54-56 -supedex/100115-OGE54-56-f14-150mM_GE3_01_12288.d	Mascot, 2.2.04	Sprot, Sprot_57.7.fasta	3/3855
Demo-090617-other_eucaryot-Sprot-trp_PE_2010-01-18 15:31:12	/Adjuvac/OGE 54-56 -supedex/100115-OGE54-56-f14-45mM_GE1_01_12286.d	Mascot, 2.2.04	Sprot, Sprot_57.7.fasta	0/4007
Compil F14_2010-02-08 15:29:43	Adjuvac/OGE 54-56 -supedex	by ProteinExtractor		6/16015

Protein 1: Macrophage infectivity potentiator OS=Trypanosoma cruzi GN=MIP PE=1 SV=1

**Accession:**	MIP_TRYCR	**Score:**	115.41
**Database:**	Sprot	**MW [kDa]:**	22.10
**Seq. Coverage [%]:**	8.70 %	**pI:**	7.70
		**No. of Peptides:**	2

10	20	30	40	50	60	70	80	90	100	110	120
MHRENYFSKI	AFCLLGVLFL	SCITSVQTVS	GDAASHEERM	NNYRKRVGRL	FMEQKAAQPD	AVKLPSGLVF	QRIARGSGKR	APAIDDKCEV	HYTGRLRDGT	VFDSSRERGK	PTTFRPNEVI
130	140	150	160	170	180	190	200				
KGWTEALQLM	REGDRWRLFI	PYDLAYGVTG	GGMIPPYSP	LEFDVELISI	KDGGKGRTAE	EVDEILRKAE	EDREDM				

Cmpd.	No. of Cmpds.	m/z meas.	Δ m/z [ppm]	z	Rt [min]	Score	P	Range	Sequence	Modification
2456	3	599.6962	40.19	3	94.7	59.1	1	56-72	K.AAQPDAVKLPSGLVFQR.I	
1653	2	508.8262	55.41	2	77.1	56.3	0	64-72	K.LPSGLVFQR.I	

Protein 2: Kinetoplastid membrane protein 11 OS=Trypanosoma cruzi GN=KMP-11 PE=2 SV=2

**Accession:**	KM11_TRYCR	**Score:**	55.23
**Database:**	Sprot	**MW [kDa]:**	11.00
**Seq. Coverage [%]:**	17.40 %	**pI:**	5.95
		**No. of Peptides:**	1

Detailed Protein Report

10	20	30	40	50	60	70	80	90	100
MATTLEEFSA	KLDRLDAEFA	KKMEEQNKKF	FADKPDESTL	SPEMKEHYEK	FEKMIQEHTD	KFNKKMHEHS	EHFKAKFAEL	LEQQKNAQFP	GK

Cmpd.	No. of Cmpds.	m/z meas.	Δ m/z [ppm]	z	Rt [min]	Score	P	Range	Sequence	Modification
1494	2	616.6582	-2.69	3	76.5	55.2	1	77-92	K.FAELLEQQKNAQFPGK.-	

Protein 3: Heat shock 70 kDa protein (Fragment) OS=Leishmania major GN=HSP70 PE=3 SV=1

**Accession:**	HSP70_LEIMA	**Score:**	50.01
**Database:**	Sprot	**MW [kDa]:**	56.50
**Seq. Coverage [%]:**	2.90 %	**pI:**	6.49
		**No. of Peptides:**	1

10	20	30	40	50	60	70	80	90	100	110	120
MTFDGAIGID	LGTTYSCVGV	WQNERLDIIA	NDQGNRTTPS	YVAFTDSERL	IGDAAKNQVA	MNPHNTVFDA	KRLIGRKFND	SVVQSDMKHW	PFKVTTKGDD	KPVISVQYRG	EEKTFTPEEI
**130**	**140**	**150**	**160**	**170**	**180**	**190**	**200**	**210**	**220**	**230**	**240**
SSMVLLKMKE	TAEAYLGKQV	KKAVVTVPAY	FNDSQRQATK	DAGTIAGLEV	VRIINEPTAA	AIAYGLDKGD	DGKERNVLIF	DLGGGTFDVT	LLTIDRGIFE	VKATNGDTHL	GGEDFDNRLV
**250**	**260**	**270**	**280**	**290**	**300**	**310**	**320**	**330**	**340**	**350**	**360**
TFFTEEFKRK	NKGKNLASSH	RALRRLRTAC	ERAKRTLSSA	TQATIEIDAL	FENIDFQATI	TRARFEELCG	DLFRSTIQPV	ERVLQDAKMD	KRSVHDVVLV	GGSTRIPKVQ	SLVSDFFGGK
**370**	**380**	**390**	**400**	**410**	**420**	**430**	**440**	**450**	**460**	**470**	**480**
ELNKSINPDE	AVAYGAAVQA	FILTGGKSKQ	TEGLLLLDVT	PLTLGIETAG	GVMTALIKRN	TTIPTKKSQI	FSTYADNQPG	VHIQVFEGER	AMTKDCHLLG	TFDLSGIPPA	PRGVPQIEVT
**490**	**500**	**510**	**520**								
FDLDANGILN	VSAEEKGTGK	RNQITITNDK	GRLSKD								

Cmpd.	No. of Cmpds.	m/z meas.	Δ m/z [ppm]	z	Rt [min]	Score	P	Range	Sequence	Modification
1449	1	565.6342	1.18	3	74.6	50.0	1	142-156	K.KAVVTVPAYFNDSQR.Q	

Protein 4: Heat shock 70 kDa protein, mitochondrial OS=Trypanosoma cruzi GN=MTP70 PE=3 SV=1

**Accession:**	HSP71_TRYCR	**Score:**	45.06
**Database:**	Sprot	**MW [kDa]:**	71.10
**Seq. Coverage [%]:**	4.30 %	**pI:**	8.61
		**No. of Peptides:**	1

Detailed Protein Report

10	20	30	40	50	60	70	80	90	100	110	120
MFARRLRGAG	SLAAASLARW	QSSKVTGDVI	GIDLGTTYSC	VAVMEGDKPR	VLENTEGFRA	TPSVVAFKGQ	EKLVGLAAKR	QAVTNPQSTF	FAVKRLIGRR	FEDSNIQHDI	KNVPYKIGRS
**130**	**140**	**150**	**160**	**170**	**180**	**190**	**200**	**210**	**220**	**230**	**240**
SNGDAWVQDA	NGKQYSPSQV	GAFVLEKMKE	TAENFLGRKV	SNAVVTCPAY	FNGPQRQATK	DAGTIAGLNV	IRVVNGPTAA	ALAYGLDKTK	DSMIAVYDLG	GGTFDISVLE	IAGGVFEVKA
**250**	**260**	**270**	**280**	**290**	**300**	**310**	**320**	**330**	**340**	**350**	**360**
TNGDTHLGGE	DFDLCLSDYI	LTEFKKSTGI	DLSNERMALQ	RIREAAEKAK	CELSTTMETE	VNLPFITANQ	DGAQHVQMTV	SRSKFESLAE	KLVQRSLGPC	KQCIKDAAVD	LKEISEVVLV
**370**	**380**	**390**	**400**	**410**	**420**	**430**	**440**	**450**	**460**	**470**	**480**
GGMTRMPKVI	EAVKQFFGRD	PFRGVNPDEA	VALGGATLGG	VLRRDVKGLV	LLDVTPLSLG	VETLGGVFTR	MIPKNTTIPT	KKSQTFFSTA	AFNQTQVGIK	VFQGEREMAA	DNQMMGQFDL
**490**	**500**	**510**	**520**	**530**	**540**	**550**	**560**	**570**	**580**	**590**	**600**
VGIPPAPRGV	PQIEVTFDIE	PNGICHVTAK	DKATGKTQNI	TITASGGLSK	EQIERMIRDS	ESHAESDRLK	RELVEVRNNA	ETQANTAERQ	LTEWKYVSDA	EKENVRTLLR	ACRKSMENPN
**610**	**620**	**630**	**640**	**650**	**660**						
VTKDELSAAT	DKLQKAVMEC	GRTEYQQAAA	GNSSSSSGNT	DSSQGEQQQQ	GDQQKQ						

Cmpd.	No. of Cmpds.	m/z meas.	Δ m/z [ppm]	z	Rt [min]	Score	P	Range	Sequence	Modification
2148	1	994.8026	-9.90	3	104.3	45.1	1	120-147	R.SSNGDAWVQDANGKQYSPSQVGA FVLEK.M	

**Protein 5:**	Histone H2B OS=Trypanosoma cruzi PE=3 SV=1		
**Accession:**	H2B_TRYCR	**Score:**	36.05
**Database:**	Sprot	**MW [kDa]:**	12.40
**Seq. Coverage [%]:**	8.00 %	**pI:**	12.00
		**No. of Peptides:**	1

10	20	30	40	50	60	70	80	90	100	110	120
MATPKSSSAN	RKKGGKKSHR	KPKRTWNVYI	NRSLKSINNH	MSMSGRTMKI	VNSFVNDLFE	RIACEAATVV	RVNKKRTLGA	RELQTAVRLV	LPADLAKHAM	AEGTKAVSHA	SS

Cmpd.	No. of Cmpds.	m/z meas.	Δ m/z [ppm]	z	Rt [min]	Score	P	Range	Sequence	Modification
1508	1	470.3472	106.08	2	77.0	36.0	0	89-97	R.LVLPADLAK.H	

Report for "100115-OGE54-56-f14_GA2_01_12285"
Chromatogram File:
D:\DATA\adjuvac\100115\100115-OGE54-56-f14_GA2_01_12285.d\100115-OGE54-56-f14_GA2_01_12285.unt

Created on: 1/16/2010 at: 05:21:38 pm on Windows system: MALDI03 by user: Administrator with HyStar Version 3.2.44.0
Operator: AD, Laboratory:
Total Chromatogram Runtime: 10.02 min

LC Method
General Information:
Author: Mazzu
LC Parameters (acquisition starting conditions)
Acquisition Parameters:
Runtime: 10.02 min
Slice Width: 2000.0 points/s
Additional Pump and Oven Parameters:
pump settings

Nr.	RunTime	Flow Rate	MinPress[bar]	MaxPress[bar]	Solvent A	Solvent B	Solvent C	Solvent D
1	10.00	0.00030	0	500	0.1% FA/CH3CN (98:2)	0.1% FA/CH3CN (20:80)		

LC Column:
LC Timetable

Time	Function	Value
0.00	Flow Rate	0.00030
0.00	Solvent Mix	100.0 0.0 0.0 0.0

Detector Settings
Detector1 : Dionex System
Offset: 0.00, InvertSignal: 0, Slicewidth: 0.00[points/sec]
Autozero: no, Spec Start: 0.00, Spec End: 0.00, Start Delay: 0
Data Channel: 1, DoReference: no, ReferenceStart: 254.00, ReferenceWidth: 10.00
DoSaveSpectra: no, SaveInterval: 1, SaveType: 1
Data Acquisition - Signals

SIGNAL No.	Source	Parameters	Autozero	Autodetection Parameters
1	Dionex System	Wavelength: 214		Slope[mAU/s]: 20.00 , Peak Par.: 5<br>Threshold: 0 , Smoothing: 2
2	HCT/esquire series		Intervals: BPC,MS	Smoothing Width: 20 , Min Points: 20<br>Threshold: 0 , Smoothing: 2
3	HCT/esquire series		Intervals: TIC,All	Smoothing Width: 20 , Min Points: 20<br>Threshold: 0 , Smoothing: 2
4	HCT/esquire series		Intervals: BPC,All	Smoothing Width: 20 , Min Points: 20<br>Threshold: 0 , Smoothing: 2
5	HCT/esquire series		Intervals: TIC,All MS/MS	Smoothing Width: 20 , Min Points: 20<br>Threshold: 0 , Smoothing: 2

Methods
Method: D:\METHODS\giga-methods\giga-2d-inject-10min-complete.m
HyStar_LC: Andrei-080930-2D-inject-10min-LC
HyStar_Autosampler: Standard
EsquireControl: 080922-GIGA-MS.m

Report for "100115-OGE54-56-f14-45mM_GE1_01_12286"

Chromatogram File:
D:\DATA\adjuvac\100115\100115-OGE54-56-f14-45mM_GE1_01_12286.d\100115-OGE54-56-f14-45mM_GE1_01_12286.unt

Created on: 1/16/2010 at: 08:35:32 pm on Windows system: MALDI03 by user: Administrator
with HyStar Version 3.2.44.0
Operator: AD, Laboratory:
Total Chromatogram Runtime: 190.02 min
LC Method
General Information:
Author: Mazzu
LC Parameters (acquisition starting conditions)
Acquisition Parameters:
Runtime: 190.02 min
Slice Width: 2000.0 points/s
Additional Pump and Oven Parameters:
pump settings

Nr.	RunTime	Flow Rate	MinPress[bar]	MaxPress[bar]	Solvent A	Solvent B	Solvent C	Solvent D
1	190.00	0.00030	0	500	0.1% FA/CH3CN (98:2)	0.1% FA/CH3CN (20:80)		

LC Column:
LC Timetable

Time	Function	Value
0.00	Flow Rate	0.00030
0.00	Solvent Mix	100.0 0.0 0.0 0.0

Detector Settings
Detector1 : Dionex System
Offset: 0.00, InvertSignal: 0, Slicewidth: 0.00[points/sec]
Autozero: no, Spec Start: 0.00, Spec End: 0.00, Start Delay: 0
Data Channel: 1, DoReference: no, ReferenceStart: 254.00, ReferenceWidth: 10.00
DoSaveSpectra: no, SaveInterval: 1, SaveType: 1
Data Acquisition - Signals

SIGNAL No.	Source	Parameters	Autozero	Autodetection Parameters
1	Dionex System	Wavelength: 214		Slope[mAU/s]: 20.00 , Peak Par.: 5<br>Threshold: 0 , Smoothing: 2
2	HCT/esquire series		Intervals: BPC,MS	Smoothing Width: 20 , Min Points: 20<br>Threshold: 0 , Smoothing: 2
3	HCT/esquire series		Intervals: TIC,All	Smoothing Width: 20 , Min Points: 20<br>Threshold: 0 , Smoothing: 2
4	HCT/esquire series		Intervals: BPC,All	Smoothing Width: 20 , Min Points: 20<br>Threshold: 0 , Smoothing: 2
5	HCT/esquire series		Intervals: TIC,All MS/MS	Smoothing Width: 20 , Min Points: 20<br>Threshold: 0 , Smoothing: 2

Methods
Method: D:\METHODS\giga-methods\giga-2d-190min-complete.m
HyStar_LC: GIGA-080930-2D-190min-LC
HyStar_Autosampler: Standard
EsquireControl: 080922-GIGA-MS.m

Report for "100115-OGE54-56-f14-75mM_GE2_01_12287"
Chromatogram File:
D:\DATA\adjuvac\100115\100115-OGE54-56-f14-75mM_GE2_01_12287.d\100115-OGE54-56-f14
75mM_GE2_01_12287.unt
Created on: 1/16/2010 at: 11:49:22 pm on Windows system: MALDI03 by user: Administrator
with HyStar Version 3.2.44.0
Operator: AD, Laboratory:
Total Chromatogram Runtime: 190.02 min
LC Method
General Information:
Author: Mazzu
LC Parameters (acquisition starting conditions)
Acquisition Parameters:
Runtime: 190.02 min
Slice Width: 2000.0 points/s
Additional Pump and Oven Parameters:
pump settings

Nr.	RunTime	Flow Rate	MinPress[bar]	MaxPress[bar]	Solvent A	Solvent B	Solvent C	Solvent D
1	190.00	0.00030	0	500	0.1% FA/CH3CN (98:2)	0.1% FA/CH3CN (20:80)		

LC Column:
LC Timetable

Time	Function	Value
0.00	Flow Rate	0.00030
0.00	Solvent Mix	100.0 0.0 0.0 0.0

Detector Settings
Detector1 : Dionex System
Offset: 0.00, InvertSignal: 0, Slicewidth: 0.00[points/sec]
Autozero: no, Spec Start: 0.00, Spec End: 0.00, Start Delay: 0
Data Channel: 1, DoReference: no, ReferenceStart: 254.00, ReferenceWidth: 10.00
DoSaveSpectra: no, SaveInterval: 1, SaveType: 1
Data Acquisition - Signals

SIGNAL No.	Source	Parameters	Autozero	Autodetection Parameters
1	Dionex System	Wavelength: 214	Slope[mAU/s]: 20.00 , Peak Par.: 5	Threshold: 0 , Smoothing: 2
2	HCT/esquire series		Intervals: BPC,MS	Smoothing Width: 20 , Min Points: 20<br>Threshold: 0 , Smoothing: 2
3	HCT/esquire series		Intervals: TIC,All	Smoothing Width: 20 , Min Points: 20<br>Threshold: 0 , Smoothing: 2
4	HCT/esquire series		Intervals: BPC,All	Smoothing Width: 20 , Min Points: 20<br>Threshold: 0 , Smoothing: 2
5	HCT/esquire series		Intervals: TIC,All MS/MS	Smoothing Width: 20 , Min Points: 20<br>Threshold: 0 , Smoothing: 2

Methods
Method: D:\METHODS\giga-methods\giga-2d-190min-complete.m
HyStar_LC: GIGA-080930-2D-190min-LC
HyStar_Autosampler: Standard
EsquireControl: 080922-GIGA-MS.m

Report for "100115-OGE54-56-f14-150mM_GE3_01_12288"
Chromatogram File:
D:\DATA\adjuvac\100115\100115-OGE54-56-f14-150mM_GE3_01_12288.d\100115-OGE54-56-f14-150mM_GE3_01_12288.unt
Created on: 1/17/2010 at: 03:02:48 am on Windows system: MALDI03 by user: Administrator
with HyStar Version 3.2.44.0
Operator: AD, Laboratory:
Total Chromatogram Runtime: 190.02 min
LC Method
General Information:
Author: Mazzu
LC Parameters (acquisition starting conditions)
Acquisition Parameters:
Runtime: 190.02 min
Slice Width: 2000.0 points/s
Additional Pump and Oven Parameters:
pump settings

Nr.	RunTime	Flow Rate	MinPress[bar]	MaxPress[bar]	Solvent A	Solvent B	Solvent C	Solvent D
1	190.00	0.00030	0	500	0.1% FA/CH3CN (98:2)	0.1% FA/CH3CN (20:80)		

LC Column:
LC Timetable

Time	Function	Value
0.00	Flow Rate	0.00030
0.00	Solvent Mix	100.0 0.0 0.0 0.0

Detector Settings
Detector1 : Dionex System
Offset: 0.00, InvertSignal: 0, Slicewidth: 0.00[points/sec]
Autozero: no, Spec Start: 0.00, Spec End: 0.00, Start Delay: 0
Data Channel: 1, DoReference: no, ReferenceStart: 254.00, ReferenceWidth: 10.00
DoSaveSpectra: no, SaveInterval: 1, SaveType: 1
Data Acquisition - Signals

SIGNAL No.	Source	Parameters	Autozero	Autodetection Parameters
1	Dionex System	Wavelength: 214		Slope[mAU/s]: 20.00 , Peak Par.: 5<br>Threshold: 0 , Smoothing: 2
2	HCT/esquire series		Intervals: BPC,MS	Smoothing Width: 20 , Min Points: 20<br>Threshold: 0 , Smoothing: 2
3	HCT/esquire series		Intervals: TIC,All	Smoothing Width: 20 , Min Points: 20<br>Threshold: 0 , Smoothing: 2
4	HCT/esquire series		Intervals: BPC,All	Smoothing Width: 20 , Min Points: 20<br>Threshold: 0 , Smoothing: 2
5	HCT/esquire series		Intervals: TIC,All MS/MS	Smoothing Width: 20 , Min Points: 20<br>Threshold: 0 , Smoothing: 2

Methods
Method: D:\METHODS\giga-methods\giga-2d-190min-complete.m
HyStar_LC: GIGA-080930-2D-190min-LC
HyStar_Autosampler: Standard
EsquireControl: 080922-GIGA-MS.m

Report for "100115-OGE54-56-f14-500mM_GE4_01_12289"
Chromatogram File:
D:\DATA\adjuvac\100115\100115-OGE54-56-f14-500mM_GE4_01_12289.d\100115-OGE54-56-f14-500mM_GE4_01_12289.unt
Created on: 1/17/2010 at: 06:16:38 am on Windows system: MALDI03 by user: Administrator
with HyStar Version 3.2.44.0
Operator: AD, Laboratory:
Total Chromatogram Runtime: 190.02 min
LC Method
General Information:
Author: Mazzu
LC Parameters (acquisition starting conditions)
Acquisition Parameters:
Runtime: 190.02 min
Slice Width: 2000.0 points/s
Additional Pump and Oven Parameters:
pump settings

Nr.	RunTime	Flow Rate	MinPress[bar]	MaxPress[bar]	Solvent A	Solvent B	Solvent C	Solvent D
1	190.00	0.00030	0	500	0.1% FA/CH3CN (98:2)	0.1% FA/CH3CN (20:80)		

LC Column:
LC Timetable

Time	Function	Value
0.00	Flow Rate	0.00030
0.00	Solvent Mix	100.0 0.0 0.0 0.0

Detector Settings
Detector1 : Dionex System
Offset: 0.00, InvertSignal: 0, Slicewidth: 0.00[points/sec]
Autozero: no, Spec Start: 0.00, Spec End: 0.00, Start Delay: 0
Data Channel: 1, DoReference: no, ReferenceStart: 254.00, ReferenceWidth: 10.00
DoSaveSpectra: no, SaveInterval: 1, SaveType: 1
Data Acquisition - Signals

SIGNAL No.	Source	Parameters	Autozero	Autodetection Parameters
1	Dionex System	Wavelength: 214		Slope[mAU/s]: 20.00 , Peak Par.: 5<br>Threshold: 0 , Smoothing: 2
2	HCT/esquire series		Intervals: BPC,MS	Smoothing Width: 20 , Min Points: 20<br>Threshold: 0 , Smoothing: 2
3	HCT/esquire series		Intervals: TIC,All	Smoothing Width: 20 , Min Points: 20<br>Threshold: 0 , Smoothing: 2
4	HCT/esquire series		Intervals: BPC,All	Smoothing Width: 20 , Min Points: 20<br>Threshold: 0 , Smoothing: 2
5	HCT/esquire series		Intervals: TIC,All MS/MS	Smoothing Width: 20 , Min Points: 20<br>Threshold: 0 , Smoothing: 2

Methods
Method: D:\METHODS\giga-methods\giga-2d-190min-complete.m
HyStar_LC: GIGA-080930-2D-190min-LC
HyStar_Autosampler: Standard
EsquireControl: 080922-GIGA-MS.m
