# Supplementary material for: Macrophage-infectivity potentiator of Trypanosoma cruzi (TcMIP) is a new pro-type 1 immuno-stimulating protein for neonatal human cells and vaccines in mice
Source: Front Immunol. 2023 Mar 23;14:1138526. doi: 10.3389/fimmu.2023.1138526 (PMC10077492; doi:10.3389/fimmu.2023.1138526)
Supplement: Supplementary file 12 [file DataSheet_10.pdf]

Mascot Search Results

User : Mike
Email :
Search title : other eucaryota
MS data file : P:\Adjuvac\090924\090925-adj-epimastig-diluT_11547.mgf
Database : Sprot 57.7 (497293 sequences; 175274722 residues)
Taxonomy : Other Eukaryota (3113 sequences)
Timestamp : 28 Sep 2009 at 09:04:43 GMT
Enzyme : Trypsin
Fixed modifications : Carbamidomethyl (C)
Variable modifications : Oxidation (M)
Mass values : Monoisotopic
Protein Mass : Unrestricted
Peptide Mass Tolerance : ± 0.6 Da
Fragment Mass Tolerance: ± 0.3 Da
Max Missed Cleavages : 1
Instrument type : ESI-TRAP
Number of queries : 2434
Protein hits :

CH60_TRYCR	Chaperonin HSP60, mitochondrial OS=Trypanosoma cruzi GN=HSP60 PE=2 SV=1
FCA1_TRYCR	Flagellar calcium-binding protein OS=Trypanosoma cruzi GN=FCABP PE=1 SV=1
CALM_TRYBB	Calmodulin OS=Trypanosoma brucei brucei PE=3 SV=2
UBIQ_TRYCR	Ubiquitin OS=Trypanosoma cruzi PE=3 SV=1
TBB_TRYBR	Tubulin beta chain OS=Trypanosoma brucei rhodesiense PE=3 SV=1
CH60_TRYBB	Chaperonin HSP60, mitochondrial OS=Trypanosoma brucei brucei GN=HSP60 PE=2 SV=2
CYSP_TRYCR	Cruzipain OS=Trypanosoma cruzi PE=1 SV=1
HSP70_TRYCR	Heat shock 70 kDa protein OS=Trypanosoma cruzi GN=HSP70 PE=3 SV=1
RLA1_TRYCR	60S acidic ribosomal protein P1 OS=Trypanosoma cruzi PE=3 SV=1
KM11B_LEIIN	Kinetoplastid membrane protein 11B OS=Leishmania infantum GN=KMP-11B PE=1 SV=1
CYSP_TRYBB	Cysteine proteinase OS=Trypanosoma brucei brucei PE=1 SV=1
KARG_TRYCR	Arginine kinase OS=Trypanosoma cruzi PE=1 SV=1
G3PG_TRYCR	Glyceraldehyde-3-phosphate dehydrogenase, glycosomal OS=Trypanosoma cruzi PE=1 SV=1
ATTY_TRYCR	Tyrosine aminotransferase OS=Trypanosoma cruzi PE=1 SV=2
TBA_EUGGR	Tubulin alpha chain OS=Euglena gracilis GN=TUBA PE=2 SV=1
G3PG_CRIFA	Glyceraldehyde-3-phosphate dehydrogenase, glycosomal OS=Crithidia fasciculata GN=GAPDG PE=3 SV=3
CHLN_CYAPA	Light-independent protochlorophyllide reductase subunit N OS=Cyanophora paradoxa GN=chlN PE=3 SV=1
YCF39_PORPU	Uncharacterized protein ycf39 OS=Porphyra purpurea GN=ycf39 PE=3 SV=1
EFTS_GALSU	Elongation factor Ts, chloroplastic OS=Galdieria sulphuraria GN=tsf PE=3 SV=1
DNAB_PORPU	Probable replicative DNA helicase OS=Porphyra purpurea GN=dnaB PE=3 SV=1

Select Summary Report

Format As | Select Summary (protein hits) | Help

Significance threshold p< 0.05 | Max. number of hits AUTO

Standard scoring ○ MudPIT scoring ● | Ions score or expect cut-off 0 | Show sub-sets 0

Show pop-ups ● Suppress pop-ups ○ | Sort unassigned Decreasing Score | Require bold red ☐

1. CH60_TRYCR **Mass:** 59602 **Score:** 445 **Queries matched:** 7 **emPAI:** 0.40

Chaperonin HSP60, mitochondrial OS=Trypanosoma cruzi GN=HSP60 PE=2 SV=1

Query	Observed	Mr(expt)	Mr(calc)	Delta	Miss	Score	Expect	Rank	Peptide
1642	602.28	1202.56	1202.58	-0.02	0	99	7.6e-10	1	K.VGGGSEVEVNEK.K
1768	670.28	1338.55	1338.61	-0.05	0	74	1.6e-07	1	R.GLIDGETSDYNR.E
1985	798.36	1594.70	1594.78	-0.08	0	97	6.2e-10	1	R.YVNMFEAGIIDPAR.V
1997	811.34	1620.67	1620.77	-0.10	0	47	7e-05	1	K.DDTVLLNGGGESSMVK.E
2016	831.43	1660.85	1660.83	0.02	0	83	1.6e-08	1	K.ALDSLLGDSSLTADQR.T
2036	846.88	1691.74	1691.81	-0.07	0	96	6.6e-10	1	K.VLENNDVTVGYDAQR.D 2037

---

2. FCA1_TRYCR **Mass:** 23949 **Score:** 383 **Queries matched:** 11 **emPAI:** 1.52

Flagellar calcium-binding protein OS=Trypanosoma cruzi GN=FCABP PE=1 SV=1

Query	Observed	Mr(expt)	Mr(calc)	Delta	Miss	Score	Expect	Rank	Peptide
1064	403.24	804.46	804.49	-0.03	1	17	0.12	1	R.RIELFK.K
1229	439.16	876.31	876.43	-0.12	0	50	7e-05	1	K.LDEFTPR.V
1423	495.26	988.52	988.52	-0.01	0	54	2.3e-05	1	K.VEDPAALFK.E
1839	707.79	1413.57	1413.59	-0.02	0	58	7.9e-06	1	K.LDADGDPDNVPESA.- 1838
1889	737.86	1473.70	1473.68	0.03	0	81	3.6e-08	1	K.GSEDFVEFLEFR.L
1290	911.39	1820.77	1820.84	-0.08	0	83	3.6e-08	1	K.LCYDEVHSGCLEVLK.L 2118 2119
2166	979.45	1956.88	1956.92	-0.04	0	90	3e-09	1	K.NGTGSVTFDEFAAWASAVK.L 2165

---

3. CALM_TRYBB **Mass:** 16828 **Score:** 238 **Queries matched:** 11 **emPAI:** 1.64

Calmodulin OS=Trypanosoma brucei brucei PE=3 SV=2

Query	Observed	Mr(expt)	Mr(calc)	Delta	Miss	Score	Expect	Rank	Peptide

134	269.06	804.15	804.42	-0.27	0	1	10	2	K.ELGTVMR.S
1361	478.76	955.51	955.47	0.05	0	46	0.00013	1	K.EAFSLFDK.D
1690	625.26	1248.51	1248.61	-0.10	0	59	5.3e-06	1	K.DGNGFISAAELR.H
756	675.54	1349.06	1348.62	0.44	0	(4)	6.4	1	K.LTDEEVDEMIR.E
1797	683.33	1364.64	1364.61	0.03	0	53	1.5e-05	1	K.LTDEEVDEMIR.E 1795
2061	580.29	1737.84	1737.87	-0.03	1	51	1.9e-05	1	R.VFDKDGNGFISAAELR.H
2111	605.26	1812.77	1812.78	-0.01	1	12	0.18	1	K.MQDSDSEEEIKEAFR.V
2128	615.63	1843.87	1843.88	-0.02	1	13	0.16	1	K.EAFSLFDKDGDGTITTK.E
2156	964.40	1926.80	1926.85	-0.05	0	98	6.1e-10	1	R.EADVDGDGQINYEEFVK.M 2157

Proteins matching the same set of peptides:

CALM_TRYBG **Mass:** 16828 **Score:** 238 **Queries matched:** 11
Calmodulin OS=Trypanosoma brucei gambiense PE=3 SV=2

CALM_TRYCR **Mass:** 16814 **Score:** 238 **Queries matched:** 11
Calmodulin OS=Trypanosoma cruzi GN=CALA2 PE=3 SV=2

---

4. UBIQ_TRYCR **Mass:** 8504 **Score:** 232 **Queries matched:** 7 **emPAI:** 3.54
Ubiquitin OS=Trypanosoma cruzi PE=3 SV=1

Query	Observed	Mr(expt)	Mr(calc)	Delta	Miss	Score	Expect	Rank	Peptide
971	383.21	764.41	764.43	-0.01	0	32	0.003	1	-.MQIFVK.T
1502	533.22	1064.43	1064.55	-0.12	0	19	0.052	1	R.TLADYNIQK.E
1507	534.30	1066.59	1066.61	-0.02	0	48	7.9e-05	1	K.ESTLHLVLR.L 1506
2069	874.42	1746.82	1746.89	-0.07	0	111	1.9e-11	1	K.TIALEVESSDTIENVK.A 2068 2070

---

5. TBB_TRYBR **Mass:** 50413 **Score:** 144 **Queries matched:** 3 **emPAI:** 0.18
Tubulin beta chain OS=Trypanosoma brucei rhodesiense PE=3 SV=1

Query	Observed	Mr(expt)	Mr(calc)	Delta	Miss	Score	Expect	Rank	Peptide
1770	671.25	1340.48	1340.64	-0.16	0	72	2.4e-07	1	R.INVYFDEATGGR.Y
1866	723.83	1445.65	1445.68	-0.04	0	34	0.0014	1	K.EVDEQMLNVQNK.N
2431	1031.78	3092.31	3092.40	-0.09	0	72	9e-08	1	K.FWEVISDEHGVDPTGTYQGDSDLQLER.I

Proteins matching the same set of peptides:

TBB_TRYCR **Mass:** 50225 **Score:** 144 **Queries matched:** 3
Tubulin beta chain OS=Trypanosoma cruzi PE=3 SV=2

---

6. CH60_TRYBB **Mass:** 59777 **Score:** 97 **Queries matched:** 2 **emPAI:** 0.05

Chaperonin HSP60, mitochondrial OS=Trypanosoma brucei brucei GN=HSP60 PE=2 SV=2

Query	Observed	Mr(expt)	Mr(calc)	Delta	Miss	Score	Expect	Rank	Peptide
1255	890.31	889.30	889.46	-0.16	0	2	6.8	2	K.LIGEAMEK.V
1985	798.36	1594.70	1594.78	-0.08	0	97	6.2e-10	1	R.YVNMFEAGIIDPAR.V

---

7. CYSP_TRYCR **Mass:** 50774 **Score:** 77 **Queries matched:** 2 **emPAI:** 0.06

Cruzipain OS=Trypanosoma cruzi PE=1 SV=1

Query	Observed	Mr(expt)	Mr(calc)	Delta	Miss	Score	Expect	Rank	Peptide
890	732.29	1462.57	1462.61	-0.04	0	77	2.5e-07	1	R.GSVEFFCGSSSSGR.L
358	514.78	1541.31	1541.71	-0.40	0	20	0.22	1	R.YHNGAAHFAAAQER.A

---

8. HSP70_TRYCR **Mass:** 74047 **Score:** 76 **Queries matched:** 4 **emPAI:** 0.12

Heat shock 70 kDa protein OS=Trypanosoma cruzi GN=HSP70 PE=3 SV=1

Query	Observed	Mr(expt)	Mr(calc)	Delta	Miss	Score	Expect	Rank	Peptide
1443	505.25	1008.49	1008.51	-0.03	0	24	0.018	1	K.EIAESYLGK.Q
1740	650.32	1298.62	1298.60	0.02	0	22	0.03	1	R.FEELCGELFR.G
1828	467.94	1400.81	1400.74	0.07	0	7	0.76	1	K.GDDKPVIQVQFR.G
1964	784.36	1566.70	1566.76	-0.06	0	67	5.8e-07	1	K.TFNPEEVSSMVLSK.M

---

9. RLA1_TRYCR **Mass:** 10804 **Score:** 71 **Queries matched:** 2 **emPAI:** 0.28

60S acidic ribosomal protein P1 OS=Trypanosoma cruzi PE=3 SV=1

Query	Observed	Mr(expt)	Mr(calc)	Delta	Miss	Score	Expect	Rank	Peptide
1550	558.78	1115.54	1115.58	-0.04	0	1	3	1	K.NVDINDVLSK.V
2149	952.77	1903.52	1903.73	-0.21	1	71	3.2e-07	1	K.KEEEEEDDDMGFGLFD.-

---

10. KM11B_LEIIN **Mass:** 11169 **Score:** 67 **Queries matched:** 1 **emPAI:** 0.27

Kinetoplastid membrane protein 11B OS=Leishmania infantum GN=KMP-11B PE=1 SV=1

Query	Observed	Mr(expt)	Mr(calc)	Delta	Miss	Score	Expect	Rank	Peptide
1540	553.32	1104.62	1104.58	0.03	0	67	1e-06	1	K.FAELLEQQK.A

Proteins matching the same set of peptides:

KM11C_LEIIN **Mass:** 11255 **Score:** 67 **Queries matched:** 1

Kinetoplastid membrane protein 11C OS=Leishmania infantum GN=KMP-11C PE=1 SV=1

KM11_LEIDO **Mass:** 11254 **Score:** 67 **Queries matched:** 1
Kinetoplastid membrane protein 11 OS=Leishmania donovani GN=KMP-11 PE=1 SV=1

KM11_LEITR **Mass:** 11227 **Score:** 67 **Queries matched:** 1
Kinetoplastid membrane protein 11 OS=Leishmania tropica GN=KMP-11 PE=3 SV=1

KM11_TRYBB **Mass:** 11069 **Score:** 67 **Queries matched:** 1
Kinetoplastid membrane protein 11 OS=Trypanosoma brucei brucei GN=KMP-11/1 PE=3 SV=1

KM11_TRYBR **Mass:** 11069 **Score:** 67 **Queries matched:** 1
Kinetoplastid membrane protein 11 OS=Trypanosoma brucei rhodesiense GN=KMP-11 PE=1 SV=1

KM11_TRYCR **Mass:** 11004 **Score:** 67 **Queries matched:** 1
Kinetoplastid membrane protein 11 OS=Trypanosoma cruzi GN=KMP-11 PE=2 SV=2

---

11. CYSP_TRYBB **Mass:** 49332 **Score:** 56 **Queries matched:** 2 **emPAI:** 0.06
Cysteine proteinase OS=Trypanosoma brucei brucei PE=1 SV=1

Query	Observed	Mr(expt)	Mr(calc)	Delta	Miss	Score	Expect	Rank	Peptide
1245	443.23	884.44	884.45	-0.01	0	56	1.1e-05	1	R.APAAVDWR.E
1379	483.25	964.48	964.46	0.01	1	0	4.6	2	K.DAKEEAFR.F

---

12. KARG_TRYCR **Mass:** 40457 **Score:** 51 **Queries matched:** 2 **emPAI:** 0.07
Arginine kinase OS=Trypanosoma cruzi PE=1 SV=1

Query	Observed	Mr(expt)	Mr(calc)	Delta	Miss	Score	Expect	Rank	Peptide
1724	644.82	1287.63	1287.67	-0.04	0	51	3.1e-05	1	K.EMQDGILELIK.A 1726

---

13. G3PG_TRYCR **Mass:** 39264 **Score:** 43 **Queries matched:** 3 **emPAI:** 0.15
Glyceraldehyde-3-phosphate dehydrogenase, glycosomal OS=Trypanosoma cruzi PE=1 SV=1

Query	Observed	Mr(expt)	Mr(calc)	Delta	Miss	Score	Expect	Rank	Peptide
1098	412.75	823.49	823.43	0.06	0	3	1.7	1	K.AAAEGHLR.G
1815	692.82	1383.63	1383.77	-0.15	0	35	0.0012	1	R.AAAVNIIPSTTGAAK.A
927	747.71	2240.11	2240.06	0.05	0	25	0.051	1	K.GILGYTDEELVSADFINDNR.S

---

14. ATTY_TRYCR **Mass:** 46708 **Score:** 42 **Queries matched:** 3 **emPAI:** 0.06
Tyrosine aminotransferase OS=Trypanosoma cruzi PE=1 SV=2

Query	Observed	Mr(expt)	Mr(calc)	Delta	Miss	Score	Expect	Rank	Peptide
1350	472.77	943.52	943.43	0.09	0	2	3.3	1	R.GAMYLMSR.I
1448	507.70	1013.39	1013.47	-0.08	0	42	0.00027	1	K.TDVEFFEK.L

1496	529.80	1057.58	1057.61	-0.03	0	1	3.3	4	K.NLLTSAAQIK.K

---

15. TBA_EUGGR **Mass:** 50488 **Score:** 35 **Queries matched:** 2 **emPAI:** 0.06
Tubulin alpha chain OS=Euglena gracilis GN=TUBA PE=2 SV=1

Query	Observed	Mr(expt)	Mr(calc)	Delta	Miss	Score	Expect	Rank	Peptide
1252	444.73	887.45	887.46	-0.01	0	35	0.0017	1	R.EDLAALEK.D
2052	573.64	1717.89	1717.87	0.02	0	15	0.1	1	R.NLDIERPTYTNLNR.L

Proteins matching the same set of peptides:
TBA_TRYBR **Mass:** 50383 **Score:** 35 **Queries matched:** 2
Tubulin alpha chain OS=Trypanosoma brucei rhodesiense PE=3 SV=1
TBA2_PELFA **Mass:** 50747 **Score:** 35 **Queries matched:** 2
Tubulin alpha-2 chain OS=Pelvetia fastigiata GN=TUBA2 PE=2 SV=1

---

16. G3PG_CRIFA **Mass:** 39291 **Score:** 35 **Queries matched:** 3 **emPAI:** 0.07
Glyceraldehyde-3-phosphate dehydrogenase, glycosomal OS=Crithidia fasciculata GN=GAPDG PE=3 SV=3

Query	Observed	Mr(expt)	Mr(calc)	Delta	Miss	Score	Expect	Rank	Peptide
377	521.00	519.99	520.29	-0.30	1	1	5.3	1	R.SSSKL.-
787	688.15	1374.29	1374.74	-0.46	1	7	3.1	2	-.MAPIKVGINGFGR.I
1815	692.82	1383.63	1383.77	-0.15	0	35	0.0012	1	R.AAAVNIIPSTTGAAK.A

---

17. CHLN_CYAPA **Mass:** 53100 **Score:** 31 **Queries matched:** 1 **emPAI:** 0.05
Light-independent protochlorophyllide reductase subunit N OS=Cyanophora paradoxa GN=chlN PE=3 SV=1

Query	Observed	Mr(expt)	Mr(calc)	Delta	Miss	Score	Expect	Rank	Peptide
1364	479.76	957.52	957.55	-0.03	0	31	0.004	1	R.DILELVTR.S

---

18. YCF39_PORPU **Mass:** 35824 **Score:** 23 **Queries matched:** 1 **emPAI:** 0.08
Uncharacterized protein ycf39 OS=Porphyra purpurea GN=ycf39 PE=3 SV=1

Query	Observed	Mr(expt)	Mr(calc)	Delta	Miss	Score	Expect	Rank	Peptide
1172	427.24	852.47	852.54	-0.07	0	23	0.018	1	R.ILPLVGNK.A

---

19. EFTS_GALSU **Score:** 23 **Queries matched:** 1
Elongation factor Ts, chloroplastic OS=Galdieria sulphuraria GN=tsf PE=3 SV=1

Query	Observed	Mr(expt)	Mr(calc)	Delta	Miss	Score	Expect	Rank	Peptide
1540	553.32	1104.62	1104.58	0.03	0	23	0.027	2	-.MSEISAQLVK.E

---

20. DNAB_PORPU **Mass:** 65842 **Score:** 22 **Queries matched:** 1 **emPAI:** 0.04

Probable replicative DNA helicase OS=Porphyra purpurea GN=dnaB PE=3 SV=1

Query	Observed	Mr(expt)	Mr(calc)	Delta	Miss	Score	Expect	Rank	Peptide
1615	**588.31**	**1174.61**	**1174.61**	**0.00**	**0**	**22**	**0.026**	**1**	**M.LTQESEDLLK.Q**

Mascot: http://www.matrixscience.com/
